# Extensive Association of Common Disease Variants with Regulatory Sequence

**DOI:** 10.1371/journal.pone.0165893

**Published:** 2016-11-22

**Authors:** Michal Mokry, Magdalena Harakalova, Folkert W. Asselbergs, Paul I. W. de Bakker, Edward E. S. Nieuwenhuis

**Affiliations:** 1 Division of Pediatrics, Wilhelmina Children's Hospital, University Medical Center Utrecht, Utrecht, 3584 EA, The Netherlands; 2 Department of Cardiology, Division Heart and Lungs, University Medical Centre Utrecht, Utrecht, 3508 GA, The Netherlands; 3 Durrer Center for Cardiogenetic Research, ICIN-Netherlands Heart Institute, Utrecht, 3501 DG, The Netherlands; 4 Institute of Cardiovascular Science, faculty of Population Health Sciences, University College London, London, WC1E 6BT, United Kingdom; 5 Department of Genetics, Center for Molecular Medicine, University Medical Center Utrecht, Utrecht, 3508 GA, The Netherlands; 6 Department of Epidemiology, Julius Center for Health Sciences and Primary Care, University Medical Center Utrecht, Utrecht, 3508 GA, The Netherlands; Huazhong Normal University, CHINA

## Abstract

Overlap between non-coding DNA regulatory sequences and common variant associations can help to identify specific cell and tissue types that are relevant for particular diseases. In a systematic manner, we analyzed variants from 94 genome-wide association studies (reporting at least 12 loci at p<5x10^-8^) by projecting them onto 466 epigenetic datasets (characterizing DNase I hypersensitive sites; DHSs) derived from various adult and fetal tissue samples and cell lines including many biological replicates. We were able to confirm many expected associations, such as the involvement of specific immune cell types in immune-related diseases and tissue types in diseases that affect specific organs, for example, inflammatory bowel disease and coronary artery disease. Other notable associations include adrenal glands in coronary artery disease, the immune system in Alzheimer’s disease, and the kidney for bone marrow density. The association signals for some GWAS (for example, myopia or age at menarche) did not show a clear pattern with any of the cell or tissue types studied. In general, the identified variants from GWAS tend to be located outside coding regions. Altogether, we have performed an extensive characterization of GWAS signals in relation to cell and tissue-specific DHSs, demonstrating a key role for regulatory mechanisms in common diseases and complex traits.

## Introduction

In the last decade, genome-wide association studies (GWAS) identified a plethora of single nucleotide polymorphisms (SNPs) robustly associated to various quantitative traits and complex diseases [1]. Interestingly, the vast majority of these SNPs are located outside of coding regions and do not affect the primary sequence of protein coding genes [1]. Due to linkage disequilibrium in the human genome, however, these SNPs should be considered as markers for nearby functional variants, including those that affect protein sequence as well as those that influence gene expression [2, 3]. Indeed, further fine-mapping of association peaks from GWAS is required for a complete understanding of genotype-phenotype correlations, especially for broad associated regions harboring multiple genes.

Each cell type in the human body has a unique set of regulatory regions and the cumulative span of those regions comprises as much as 80% of the non-coding DNA [4]. The plasticity and variability of active regulatory regions can be exploited for the identification of cell or tissue types that play a key role in the etiology of a given disease [3]. An excess overlap between variants associated with a human disease or a phenotypic trait and regulatory DNA sequence elements can thus point to “critical” cell type(s) that may play a causal role in the disease. This has been recently demonstrated for a limited number of diseases using histone H3 lysine 4 trimethylation (H3K4me3) chromatin immunoprecipitation (ChIP) and DNase I hypersensitive sites (DHSs) as readouts for cell-specific regulatory regions[3, 5–7]. Even though several studies have demonstrated the potential value of this approach, an extensive annotation of larger number of cell types or tissues against the catalog of genome-wide association results is still lacking.

Here we followed up on this idea in a systematic way. We tested all *bona fide* variants associated with diseases and traits from 94 different studies for excess overlap with DHSs assayed in 124 different cell types by the ENCODE consortium[4] and in 342 various adult end fetal tissue samples assayed for DHSs by the NIH Roadmap Epigenomics Project[8]. We report many known and novel associations between tissue and cell types and diseases.

## Results

### Datasets

To systematically annotate different GWAS loci with known regulatory elements we accessed the results of 2101 different GWAS from the NHGRI GWAS Catalog [9]. To increase the statistical power and the specificity of our analysis, we selected only GWAS with at least 12 independent loci each achieving genome-wide significance at a p-value < 5 x 10^−8^; altogether, we included the results from 94 GWAS for our study (S1 Table). Next, we downloaded 466 DHS datasets produced by the ENCODE consortium[10] or by the NIH Roadmap Epigenomics Project[8] (S2 Table). On average, the data comprised ~87,000 DHSs per tissue or cell type (S1A Fig) spanning on average 25 million bases (S1B Fig) per sample, i.e. slightly less than 1% of the human genome.

We have focused on these particular datasets, since DHSs are considered to be one of the best discriminative features [6] between cell types. Even though chromatin mark H3K4me3 was shown to be slightly better in predicting “critical” cell types[6], we used DHSs since these were available for a larger number of different cell types and tissues, and importantly, included data for biological replicates (S1C and S1D Fig).

### Strategy to associate regulatory elements with SNPs

**Fig 1:**
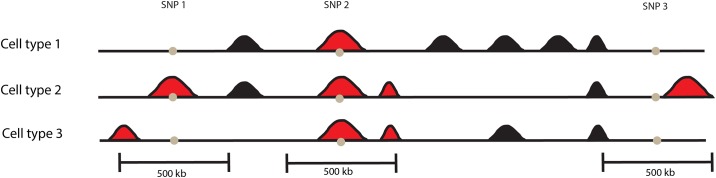
Schematic description of the association model used to quantify the overlap between regulatory elements (black and red DHS peaks) and disease-associated variants (SNP1, 2 and 3) in various cell types (cell type 1, 2 and 3). The model estimates the regulatory activity by counting the number of DHS peaks (red) located within the region of a 500kb with the associated tag SNP in the middle.

In order to estimate the enrichment of overlap between genomic elements, we opted for a conservative model that is not sensitive to linkage disequilibrium amongst nearby SNPs. For each independent SNP association peak from the GWAS, we counted the number of DHSs within a 500 kb wide region centered on the lead SNP (Fig 1). The model gives similar results with different region sizes (100, 250, 500 and 1000kb) (S2 Fig), while the 500kb wide region provides the best compromise between sensitivity and specificity, especially for GWAS with smaller number of associated loci. In addition a substantial part of distal chromatin interaction of promoters with regulatory elements is within this distance[11]. We estimated the level of enrichment and the statistical significance from a null model where appropriately matched SNP sets are also tested for overlap with the DHSs. The matched SNPs are selected in such a way to have a similar distribution with respect to transcriptional start sites, minor allele frequency [12] and similar number of genes in vicinity as the associated SNPs from the GWAS.

**Fig 2:**
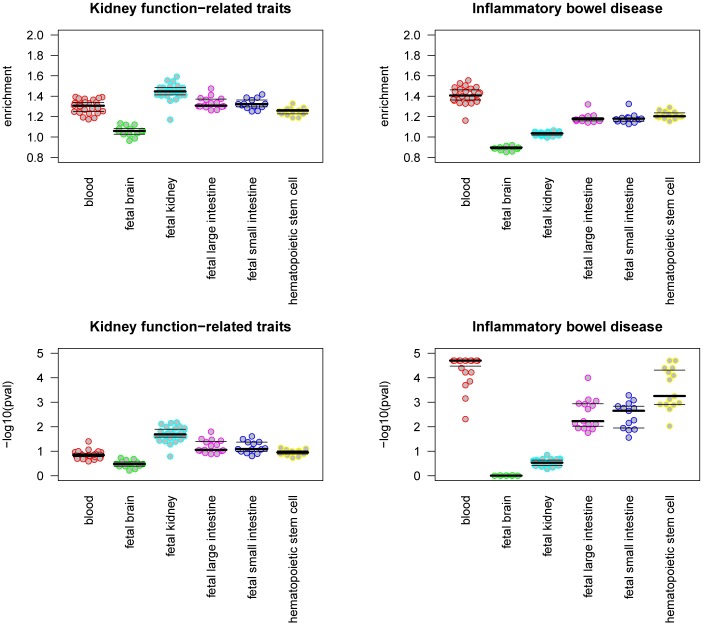
Reproducibility of the quantification model. Parallel dot-plots depicting the enrichment over the null distribution and the significance (p-value) between the regulatory elements active in selected tissues and cells (defined by the DHS) with variants associated to renal function and IBD. Each point represents the separate replicate of DHS assay in the given tissue.

To demonstrate the reliability and reproducibility of our model we selected two different GWASs that identified SNPs associated with renal function-related traits and inflammatory bowel disease (IBD). Based on pathogenesis of these particular traits [13, 14] we expected to find associations with specific cells/tissues (intestines, kidney and various immune cells). Each tissue/cell type is represented by multiple DHS datasets derived from different individuals, allowing us to explore the reproducibility of the model. We identified a significant enrichment of DHS sites nearby the associated variants within the expected cell types compared to the null model (Fig 2). The DHS from kidney exhibited the greatest enrichment in the vicinity of variants associated with renal function[15] (Average Fold Enrichment (AFE) = 1.45 +/- 0.08, P = 0.026), while the DHS sites identified in leukocytes and intestinal tissue were the most enriched sites for variants associated with IBD[16] (AFE = 1.34 +/- 0.11, P = 2x10^-5^ and AFE = 1.19 +/- 0.04, P = 0.0061; respectively). Importantly, we observed low variability between different replicates of the same tissue or cell type.

### Systematic association

**Fig 3:**
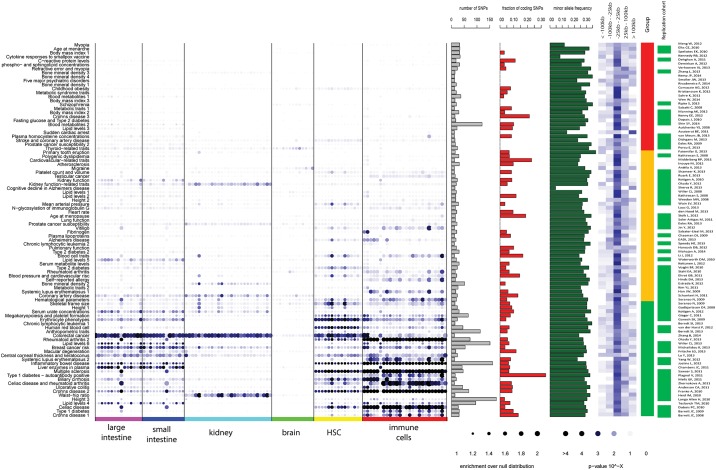
Systematic association of common variants identified by 94 different GWAS with open chromatin (DHS) in selected tissues and cells. The individual GWAS are sorted based on the minimal association p-value with 466 DHS (only selected DHS samples are shown). Size of the dot represents the enrichment over the null distribution and color of the dot represents the significance of the enrichment—with black being the most significant. The bar graphs depict the number of associated SNPs (grey), the fraction of coding SNPs (red) and minor allele frequency (green). Heatmap depicts the distribution of SNPs with respect to transcriptional start sites (intensity of blue depicts the fraction of SNPs within the distance bin). Green/orange/white bar shows the presence of the replication cohort (green—present, white—no replication cohort as specified in the in NHGRI GWAS Catalog) separately for each GWAS. Group bar depicts the annotation of GWAS to one of three “GWAS groups”. Green—GWAS with the best association p-value < = 0.0001, orange < = 0.01 and red—GWAS with the best association p-value < 0.01.

Having demonstrated the potential value of the approach, we systematically compared the associations of the loci from 94 GWAS with 342 DHS from the NIH Roadmap Epigenomics Consortium (Fig 3, S3 Fig and S3 Table) and 124 DHS from ENCODE consortium (S4 Fig and S4 Table). This resulted in a total of 7,878 statistically significant associations between GWAS variants and DHS datasets (P < 0.05).

**Fig 4:**
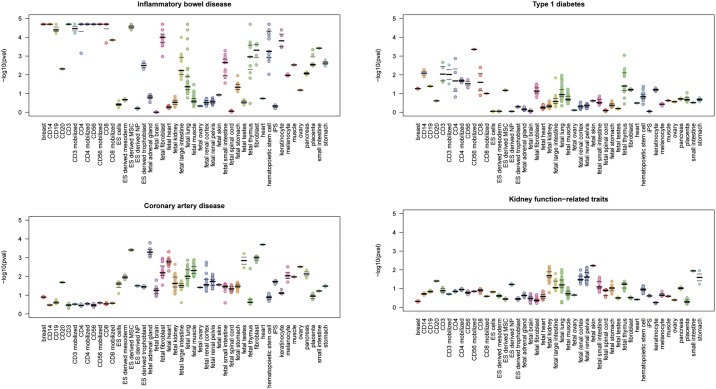
Tissues and cell types associated with common variants identified by selected GWASs. Each point represents the separate replicate of DHS assay in the given tissue.

We were able to confirm a large number of the expected and known associations. For example, the association of DHS active in immune cells and variants linked to numerous immune-related[17–20] diseases like inflammatory bowel disease (IBD) (AFE = 1.34 +/- 0.11, P = 2x10^-5^), celiac disease (AFE = 1.64 +/- 0.29, P = 0.00027), type 1 diabetes (AFE = 1.29 +/- 0.15, P = 0.046) and systemic lupus erythematosus (AFE = 1.53 +/- 0.30, P = 0.040) (Fig 4 and S2 and S3 Figs) were the most prominent. Also, numerous organ-specific traits like renal function-related traits, IBD or coronary heart disease showed overlaps between the associated genetic variants and DHS that are known to be “open” in the affected organ (kidney (AFE = 1.45 +/- 0.08, P = 0.026), intestine (AFE = 1.19 +/- 0.04, P = 0.0061) and heart (AFE = 1.55 +/- 0.05, P = 0.0016), respectively; see Fig 4, S2 and S3 Figs).

### Comparison of different GWAS groups

On the one hand, our results illustrate that numerous genetic variants associated with various traits overlap with DHSs active in relevant cell types (Fig 3), in some cases concordant with expectation. On the other hand, such DHS-cell type associations cannot be confirmed or detected for all GWAS studied here (Fig 3).

To explore this phenomenon in more detail, we have divided the GWAS into three similarly sized groups (green, orange and red group) based on the enrichment p-value of the most significant DHS dataset. Green group consist of GWASs with the most significant p-value of the DHS enrichment while the red group consist of the studies with the least significant p-value. The orange group consists of GWASs with the intermediate p-value.

**Fig 5:**
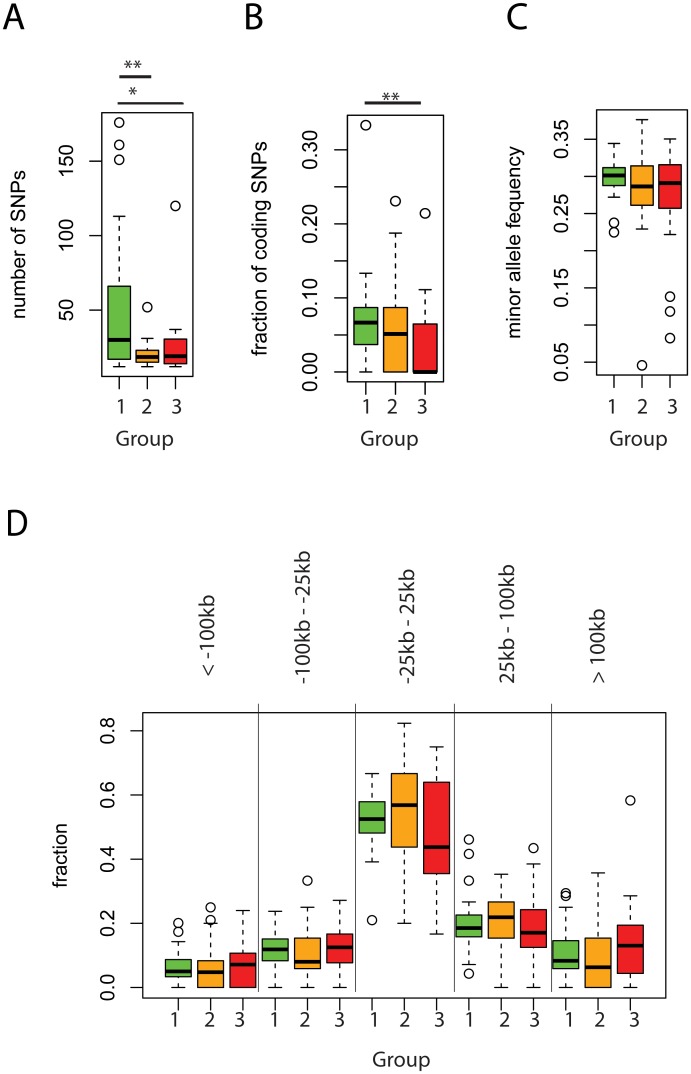
Comparison of selected GWAS parameter between three GWAS groups. A) The number of associated SNPs B) the fraction of coding SNPs C) minor allele frequency and D) the distribution of SNPs with respect to transcriptional start sites (no line—non significant, * p-value < 0.05, ** p-value < 0.01, using Wilcoxon signed-rank test), green—GWAS with the best association p-value < = 0.0001, orange < = 0.01 and red—GWAS with the best association p-value < 0.01.

A negative result in some GWAS could potentially be explained by having too few *bona fide* associations per study (trait), reducing the power to detect significant associations with tissue or cell types. This is consistent with the GWAS in the red group having a lower number of reported associations (Figs 3 and 5A) compared to the green group. Secondly, we explored if GWASs in different groups are more or less enriched in coding variants as a possible explanation for having poor association with regulatory regions. Interestingly, the percentage of tag SNPs with coding potential (missense, nonsense, frame shift and splice site variants) was higher in those GWASs that showed also more significant associations with non-coding regulatory regions (Fig 5B). The identified variants in all GWAS groups show similar average minor allele frequencies (Fig 5C) and are equally likely to be located close to the annotated transcriptional start sites (+/- 25kb) (Fig 5D). Finally, only 48% of the GWAS in the red group had replication data, compared to 71% and 79% of the studies in the orange and green group, respectively, based on information from the NHGRI GWAS Catalog [9] (Fig 3).

### Notable associations

Although some associations implicating certain cell or tissue types were unsurprising given known biology, we also observed several notable associations. For example, even though coronary artery disease due to atherosclerosis is a progressive inflammatory disease characterized by the accumulation of lipids, fibrous materials, and mineral in the arteries [21], loci identified by GWAS for coronary artery disease show a strong association with regulatory regions that are active in adrenal glands (AFE = 1.57 +/- 0.04, P = 0.00051) (Fig 4, S3 Fig and S3 Table). Interestingly, increased levels of androgens secreted by adrenal glands [22], Cushing’s syndrome [23], sympatho-adrenal medullary activation [24], and neuroendocrine stress axis [25] are all strongly associated with adrenal glandular dysfunction and increased risk for coronary heart disease. This provides some support for the adrenal glands as a potentially causal tissue type in coronary artery disease, among other mechanisms—possibly through the impairment of the action of the adrenal gland-secreted hormone adrenalin (epinephrine) resulting in decreased coronary perfusion [26]. However, additional functional studies are warranted [27].

Next, our analysis suggests the possible involvement of immune system (AFE = 1.30 +/- 0.13 P = 0.088) in Alzheimer’s disease pathogenesis [28]. Links between Alzheimer’s disease and local or systemic immunologic dysfunction have previously been described [29–31]. The immune mechanisms and microglia are shown to be involved in the clearance of amyloid beta peptide [32]. Also, the evidence from the retrospective studies shows that the levels of inflammatory proteins in plasma are increased even before the clinical onset of dementia [33]. Additionally, the inhibition of IL-12/IL-23 signaling—normally activated in response to a variety of pathogens, was found to reduce Alzheimer's disease-like pathology and cognitive decline [34]. These and our findings support a potential role of the immune system in Alzheimer’s disease.

Similarly, we found that the variants influencing bone mineral density were associated with open regulatory regions in the kidney (AFE = 1.22 +/- 0.05, P = 0.033) (S2 Fig and S3 Table). Interestingly, bone density regulation have been associated with renal function [35, 36]. Metabolic bone disease is a common complication of chronic kidney disease (CKD) due to control mechanisms for calcium and phosphorus homeostasis altered in CKD patients [37]. Again, these findings illustrate that plausible connections can be inferred from the integration of GWAS and epigenomic data, implicating potentially causal cell and tissue types for specific diseases and traits.

## Discussion

Here we provide an extensive characterization of the relation between open chromatin regions from hundreds of cell and tissue samples and *bona fide* SNP associations from GWAS for various human diseases and traits. Our results support the general idea that the etiology of complex disease can originate from specific cell or tissue types. Indeed, our analyses identify known and novel associations between complex diseases and specific cell and tissue types, which can serve as a resource for further research.

In many instances, GWAS variants show significant overlap with DHS sites from several different cell and tissue types. While this may reinforce the complex nature of the traits studied, it does not rule out the scenario that genetic variants can alter the function of ubiquitously active regulatory elements in a cell type specific manner under certain conditions [38], e.g. after cell activation. Future studies that incorporate gene expression profiles [39] might provide more specific insight into the critical cell types.

While many GWAS showed overlap of identified variants and open chromatin regions in relevant cell types, numerous GWAS showed weaker associations without any strong signal implicating a specific cell or tissue type. Perhaps for these traits the underlying genetic mechanisms might be different, or the relevant cell or tissue type was not present among the 466 DHS datasets evaluated. For some of these traits, we cannot exclude the possibility of multiple cell types and tissues being involved, which would cause the association signal to be diluted and to fall below the detection limit of our model.

In conclusion, we present an extensive association analysis between GWAS association signals and specific cell and tissue DHS sites, providing us with novel insights into underlying mechanisms of common diseases and a useful resource for further analyses.

## Materials and Methods

### GWAS datasets

GWAS datasets were accessed from the NHGRI GWAS Catalog (http://www.genome.gov/gwastudies/) [9] on at 2nd^th^ February 2015. We included GWAS with at least 12 reported loci at genome-wide significance (p<5 x 10^−8^) (S1 Table).

### DHS datasets

DHS datasets derived from 124 cell lines [3] (S2 Table) were accessed at the http://ftp.ebi.ac.uk/pub/databases/ensembl/encode/integration_data_jan2011/byDataType/openchrom/jan2011/fdrPeaks/ on 31^st^ July 2013.

DHS sequencing reads from 342 tissue samples produced by NIH Roadmap Epigenomics Project [8] were accessed via the 9^th^ release of Human Epigenome Atlas. Cisgenome v.2 [40] software package (-e 50, -maxgap 200, -minlen 200) was used for peak-calling from the Roadmap Epigenomics datasets against the common input sample. All called peaks have false discovery rate (FDR) < 0.05.

### Association of DHS and GWAS loci

A susceptibility locus was defined as a 500-kb wide region centered on the reported SNP from a GWAS. Neighboring SNPs reported by the GWAS were considered as a single locus. DHSs were considered as overlapping with a susceptibility locus when at least one base overlapped with the locus [12]. To calculate the enrichment of DHSs across susceptibility loci and to estimate the p-value, we compared the observed number of DHSs overlapping the susceptibility loci with the number of overlapping DHSs with 50,000 matched sets of random “null” loci. These “null” loci were sampled from SNPs present on the Human Omni1S genotyping chip (Illumina) with a similar minor allele frequency (+/- 5%) as the SNPs from the GWAS included in this study (using frequencies from ftp://ftp.ensembl.org/pub/release-72/variation/gvf/homo_sapiens/1000GENOMES-phase_1_EUR.gvf.gz). Next, we controlled for the non-random physical distribution of the susceptibility loci with respect to annotated genes by sampling only variants with similarly located closest annotated transcription start site as the SNPs from the GWAS (using annotation files downloaded from the Cisgenome website [40]). To this end, we defined distinct location bins [−200 k bp, −100 k bp, −25 k bp, −10 k bp, −5 k bp, 0 bp, 5 k bp, 10 k bp, 25 k bp, 100 k bp, 200 k bp] relative to the closest transcription start site (TSS). In addition, we have excluded those control SNPs that did not have similar number of annotated TSS within 500kb from SNP location (0 to 9 or 10 to 24 or more than 24 TSS) as the SNP from the GWAS.

### Computational analysis of GWAS loci

The GWAS were assigned into 3 groups based on the best association p-value. Green—GWAS with the best association p-value < = 0.0001, orange between >0.0001 and < = 0.01 and red—GWAS with the best association p-value > 0.01.

The information about minor allele frequency (based on Utah residents with Northern and Western European ancestry from the CEPH collection “CEU dataset”) for GWAS SNPs was accessed from the HapMap website [41]: http://hapmap.ncbi.nlm.nih.gov/downloads/ld_data/latest/ on 8th of August 2013.

The information about the potential impact of the SNP on protein product was accessed from Ensembl Variation 75 database (Homo sapiens Short Variation, GrCh37.p13) [42] via BioMart [43]. After manual inspection, all SNPs tagged as ‘missense_variant’, ‘splice_region_variant’, ‘frameshift_variant’, ‘stop_gained’, ‘stop_lost’, ‘feature_elongation’ or ‘splice_donor_variant’ for at least one annotated coding transcript were considered as SNPs with potential impact on protein sequence.

The analysis was performed using custom Perl and R scripts together with the utilities from Cisgenome[40].

## Supporting Information

S1 Fig Distribution of A) number of peaks and B) total base pairs covered in ENCODE and Roadmap Epigenomics DHS samples and correlation heatmaps between different DHS samples from ENCODE (C) and Roadmap Epigenomics (D). (TIF)

S2 Fig Reproducibility of the quantification model with different locus size (100, 250, 500 and 1000 kilobases). Parallel dot-plots depicting the enrichment over the null distribution between the regulatory elements active in selected tissues and cells (defined by the DHS) with variants associated to renal function and IBD. Each point represents the separate replicate of DHS assay in the given tissue.
(TIF)

S3 Fig Systematic association of common variants identified by 86 different GWAS with open chromatin in 342 tissue samples. The GWAS are sorted based on the minimal association p-value with 466 DHS tracks. Size of the dot represents the enrichment over the null distribution and color of the dot represents the significance of the enrichment—with black being the most significant.
(TIF)

S4 Fig Systematic association of common variants identified by 86 different GWAS with open chromatin regions in 124 cell lines. The GWAS are sorted based on the minimal association p-value with 466 DHS tracks. Size of the dot represents the enrichment over the null distribution and color of the dot represents the significance of the enrichment—with black being the most significant.
(TIF)

S1 Table List of GWAS. (XLS)

S2 Table List of DHS datasets. (XLS)

S3 Table Results of Roadmap data. (PDF)

S4 Table Results of ENCODE data. (PDF)

## Abbreviations

DHS: DNase I hypersensitive site
GWAS: genome-wide association study
SNP: single nucleotide polymorphism
H3K4me3: H3 lysine 4 trimethylation
ChIP: chromatin immunoprecipitation
