# Supplementary figures and images for: Extensive Association of Common Disease Variants with Regulatory Sequence
Source: PLoS One. 2016 Nov 22;11(11):e0165893. doi: 10.1371/journal.pone.0165893 (PMC5119736; doi:10.1371/journal.pone.0165893)

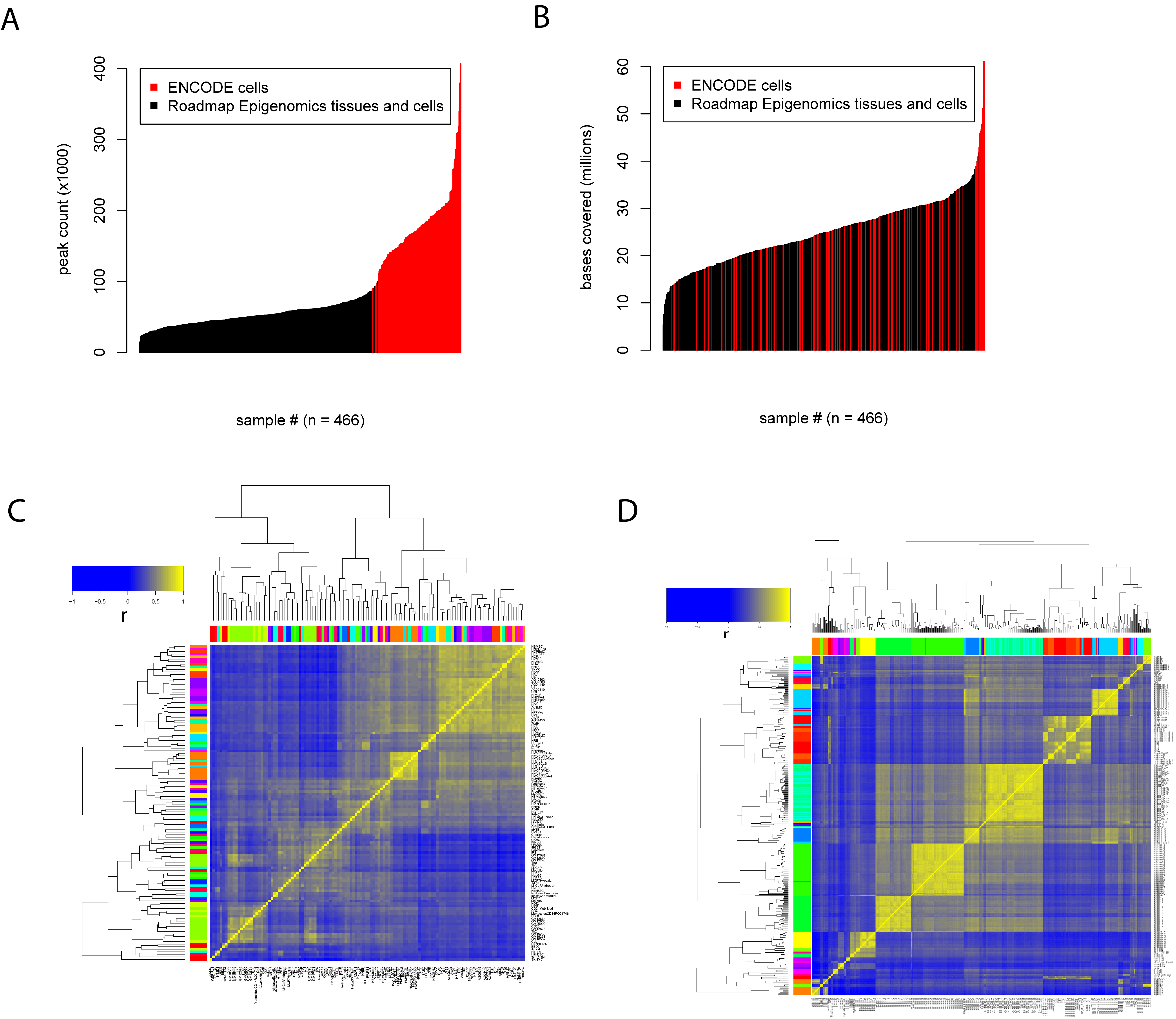

Supplement: S1 Fig — (TIF) [file pone.0165893.s001.tif]

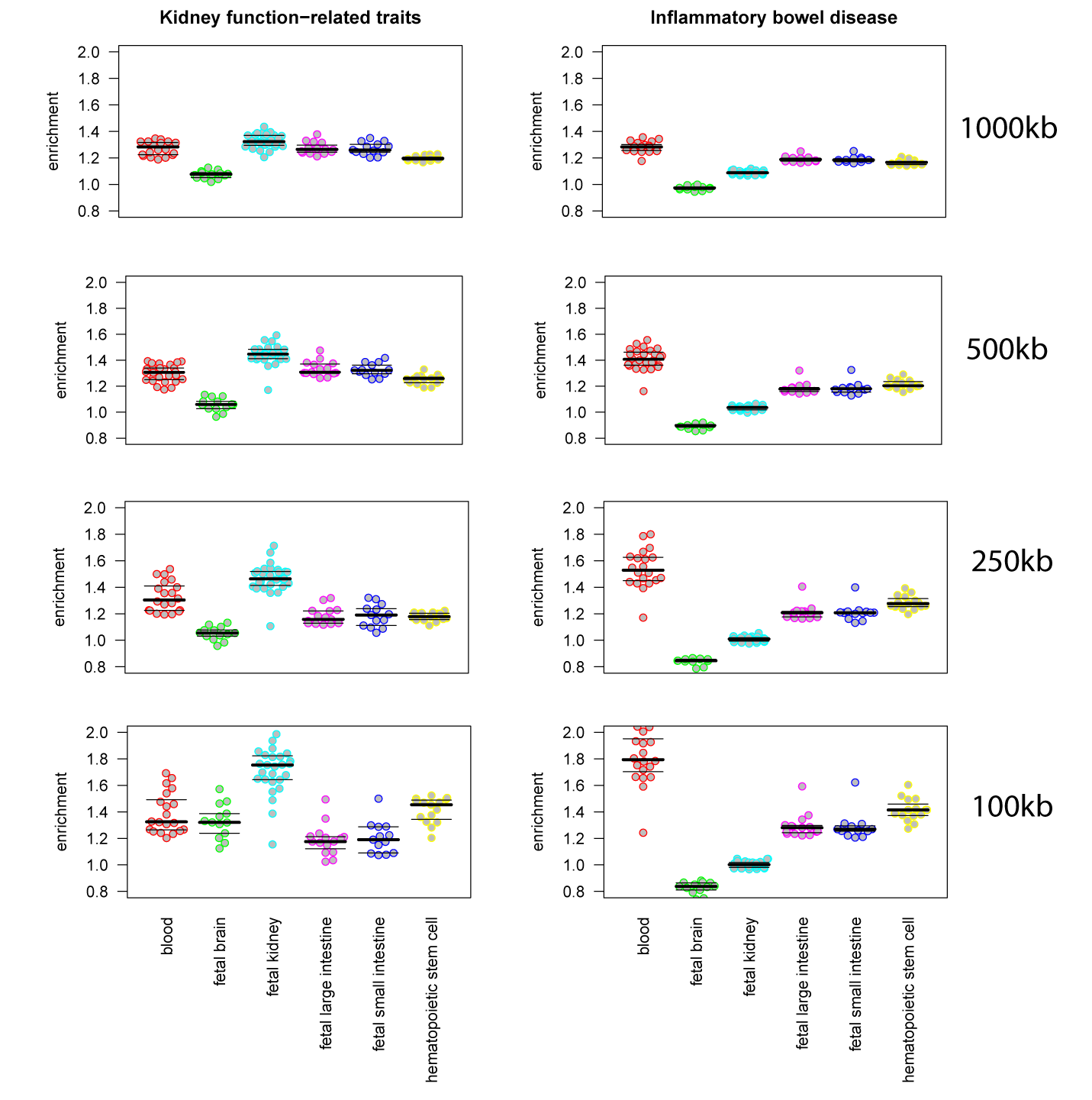

Supplement: S2 Fig — Parallel dot-plots depicting the enrichment over the null distribution between the regulatory elements active in selected tissues and cells (defined by the DHS) with variants associated to renal function and IBD. Each point represents the separate replicate of DHS assay in the given tissue. (TIF) [file pone.0165893.s002.tif]

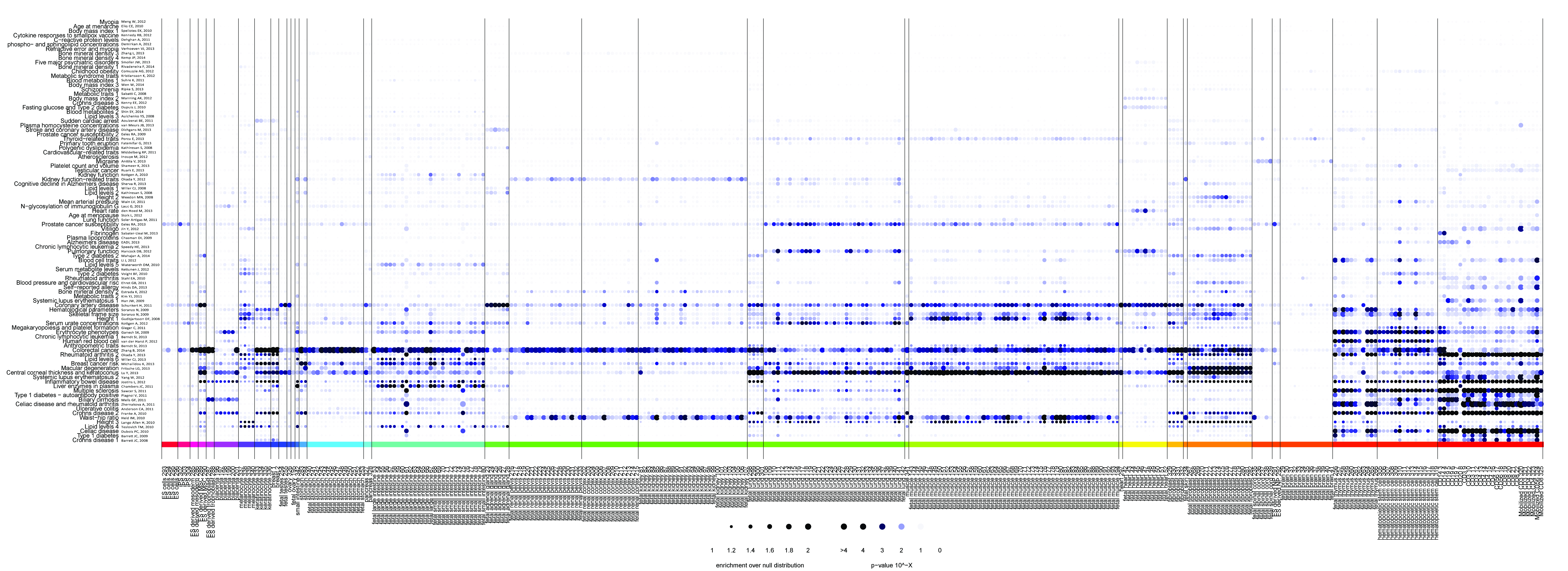

Supplement: S3 Fig — The GWAS are sorted based on the minimal association p-value with 466 DHS tracks. Size of the dot represents the enrichment over the null distribution and color of the dot represents the significance of the enrichment—with black being the most significant. (TIF) [file pone.0165893.s003.tif]

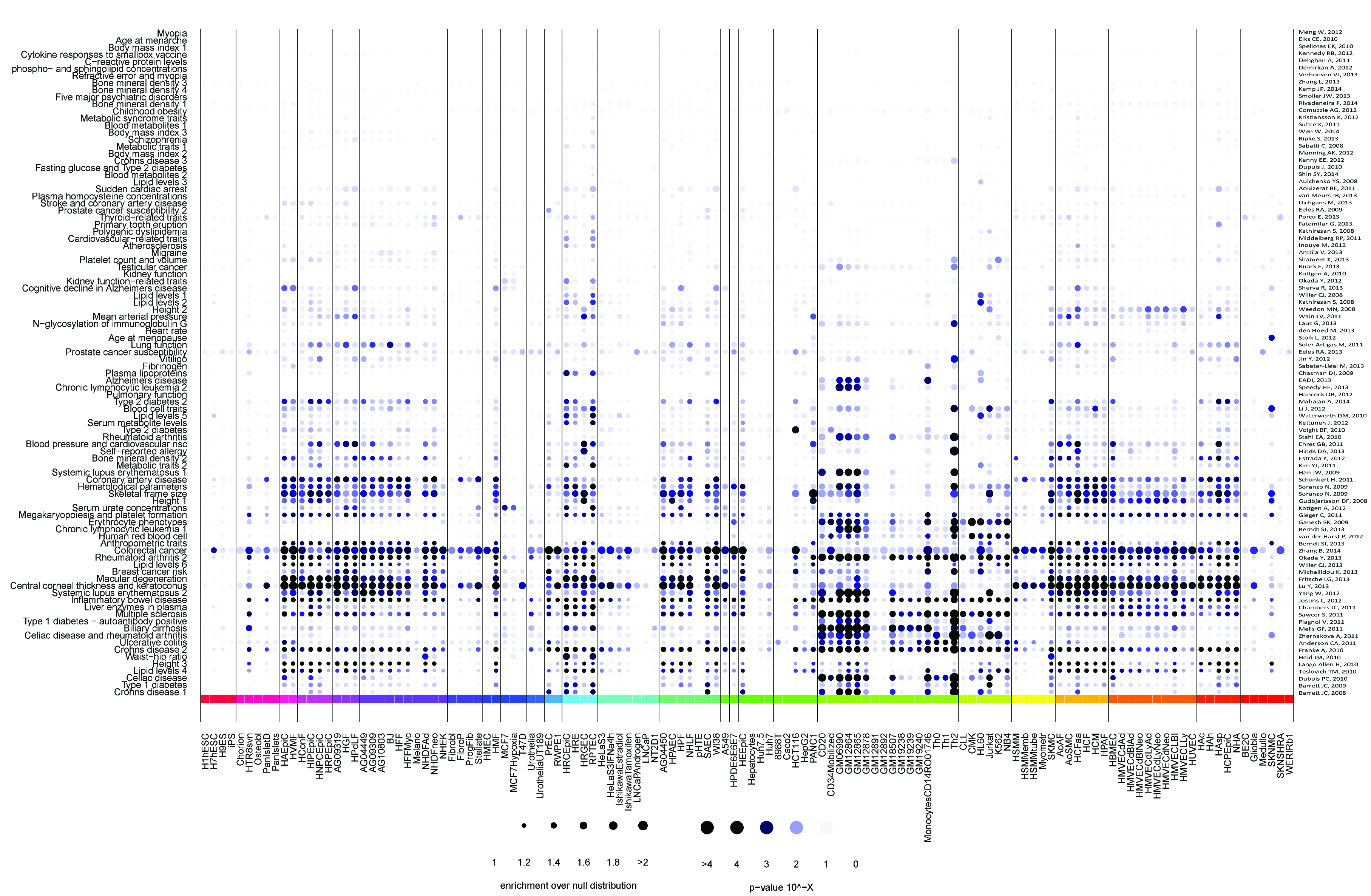

Supplement: S4 Fig — The GWAS are sorted based on the minimal association p-value with 466 DHS tracks. Size of the dot represents the enrichment over the null distribution and color of the dot represents the significance of the enrichment—with black being the most significant. (TIF) [file pone.0165893.s004.tif]
