# Supplementary material for: Extensive Association of Common Disease Variants with Regulatory Sequence
Source: PLoS One. 2016 Nov 22;11(11):e0165893. doi: 10.1371/journal.pone.0165893 (PMC5119736; doi:10.1371/journal.pone.0165893)
Supplement: S3 Table — (PDF) [file pone.0165893.s007.pdf]

Crohns disease 1

DHS sample	fold enrichment	p value
CD56 17	1.5648103	0.00086
Mobilized CD56 324	1.6104012	0.00094
CD4 14	1.5832611	0.00126
CD8 20	1.5748185	0.00172
CD3 11	1.5701763	0.00178
CD14 4	1.5363275	0.00184
breast 1	1.3011093	0.00650
CD14 3	1.4793563	0.00688
CD3 10	1.4747916	0.00774
Mobilized CD3 320	1.5276614	0.00782
CD4 13	1.5040878	0.00860
CD19 5	1.4753630	0.00860
CD3 9	1.4929586	0.00888
hematopoietic stem cell 310	1.3038237	0.01184
fetal thymus 256	1.4187968	0.01190
Mobilized CD4 323	1.4548440	0.01472
breast 2	1.2644578	0.01484
Mobilized CD4 322	1.4757677	0.01514
CD19 6	1.4353888	0.01862
keratinocyte 333	1.2218448	0.02060
keratinocyte 335	1.2268037	0.02098
CD8 21	1.4330630	0.02142
keratinocyte 334	1.2208590	0.02248
CD3 12	1.4143579	0.02622
CD8 22	1.4053857	0.02682
keratinocyte 336	1.2125786	0.02774
hematopoietic stem cell 314	1.2478902	0.03968
hematopoietic stem cell 317	1.2348235	0.04074
CD19 7	1.3619060	0.04090
fetal thymus 264	1.3068111	0.04166
CD4 16	1.3704713	0.04332
CD56 18	1.3504491	0.04596
Mobilized CD3 321	1.3482120	0.05720
fetal thymus 258	1.2684663	0.06430
CD8 19	1.3345976	0.06590
fetal thymus 265	1.2644088	0.06920
hematopoietic stem cell 312	1.2104249	0.07354
fetal thymus 266	1.2576014	0.07544
hematopoietic stem cell 309	1.1977514	0.09618
fetal large intestine 61	1.2371736	0.10912
placenta 200	1.1529796	0.12176
fetal thymus 260	1.2063374	0.12280
hematopoietic stem cell 313	1.1628621	0.12350
Mobilized CD8 325	1.2349732	0.12390
fetal fibroblast 268	1.1314625	0.13942
hematopoietic stem cell 308	1.1555243	0.13948
fetal fibroblast 282	1.1289065	0.13990
fetal fibroblast 281	1.1313167	0.14098
fetal thymus 259	1.1837690	0.15992
fetal fibroblast 273	1.1183094	0.16410
fetal fibroblast 275	1.1144635	0.16858
hematopoietic stem cell 318	1.1446499	0.17868
fetal fibroblast 272	1.1101943	0.18278
fetal fibroblast 276	1.1043075	0.18748
fetal fibroblast 279	1.1083226	0.18988
fetal fibroblast 274	1.1064299	0.19216
fetal lung 300	1.1129920	0.19690
hematopoietic stem cell 315	1.1252432	0.20210
fetal fibroblast 271	1.0953064	0.21344
fetal thymus 257	1.1428809	0.21618
muscle 341	1.1303607	0.21660
fibroblast 331	1.0967031	0.22420
fetal lung 301	1.1013999	0.22436
fetal fibroblast 277	1.0907094	0.23342
fetal thymus 261	1.1280263	0.23500
fetal fibroblast 280	1.0864095	0.23616
fetal fibroblast 278	1.0835323	0.25122
hematopoietic stem cell 305	1.0978490	0.25294
fetal small intestine 75	1.1282934	0.27378
CD4 15	1.1320158	0.27388
hematopoietic stem cell 316	1.0871817	0.27968
fetal fibroblast 267	1.0696839	0.28456
placenta 198	1.0761399	0.28544
placenta 199	1.0757317	0.28636
hematopoietic stem cell 306	1.0762124	0.29464
fetal large intestine 58	1.0753783	0.29688
ES derived MSC 289	1.0715744	0.29742
fibroblast 332	1.0659678	0.30002
ES derived MSC 290	1.0669660	0.30988
fetal lung 298	1.0672714	0.31060
small intestine 342	1.0738968	0.31142
placenta 197	1.0701976	0.31298
fetal large intestine 64	1.0669660	0.32100
hematopoietic stem cell 307	1.0621071	0.32748
fetal small intestine 70	1.0533105	0.34482
placenta 201	1.0539145	0.35298
fetal fibroblast 269	1.0435652	0.35354
hematopoietic stem cell 311	1.0497678	0.35618
fetal lung 111	1.0419672	0.35820
fetal small intestine 68	1.0462512	0.36938
fetal lung 299	1.0394897	0.38128
ES derived trophoblast 287	1.0341669	0.40048
fetal large intestine 55	1.0311677	0.40640
fetal large intestine 57	1.0285071	0.41276
fetal large intestine 60	1.0298897	0.41286
fetal small intestine 78	1.0214346	0.43236
fetal small intestine 74	1.0197615	0.44042
fetal fibroblast 270	1.0122094	0.45058
placenta 196	1.0143814	0.45082
CD20 8	1.0123291	0.45962
fetal large intestine 67	1.0081077	0.46694
fibroblast 330	1.0057055	0.47386
fetal small intestine 77	1.0040057	0.47708
melanocyte 337	1.0046687	0.47930
melanocyte 338	1.0048768	0.47970
ES derived trophoblast 288	1.0037727	0.48222
stomach 283	0.9984997	0.49052
fetal thymus 262	0.9919664	0.49092
hematopoietic stem cell 319	0.9912018	0.50846
fibroblast 329	0.9948139	0.50962
stomach 284	0.9915071	0.51114
fetal small intestine 80	0.9919391	0.51330
melanocyte 339	0.9928319	0.52134
fetal large intestine 54	0.9858871	0.52472
fetal small intestine 79	0.9736102	0.54840
fetal lung 128	0.9826928	0.55654
melanocyte 340	0.9801475	0.55978
fetal large intestine 63	0.9698994	0.56030
fetal lung 134	0.9744745	0.56544
fetal small intestine 72	0.9668223	0.57182
fetal large intestine 56	0.9671291	0.57260
heart 297	0.9591870	0.59346
fetal heart 47	0.9560463	0.59408
fetal small intestine 69	0.9455844	0.61594
fetal large intestine 65	0.9407219	0.62896
fetal small intestine 76	0.9360140	0.63926
fetal small intestine 73	0.9360397	0.64658
fetal large intestine 62	0.9305258	0.65070
fetal lung 141	0.9475142	0.66498
fetal lung 133	0.9437290	0.67372
fetal large intestine 59	0.9195921	0.68134
fetal small intestine 71	0.9145132	0.69602
fetal stomach 244	0.9140150	0.69660
fetal stomach 243	0.9173528	0.70176
ovary 326	0.9005126	0.70478
fetal large intestine 66	0.9041985	0.71306
fetal muscle 172	0.9266361	0.71506
fetal lung 116	0.9304456	0.71996
fetal lung 129	0.9239212	0.72154
fetal lung 117	0.9263210	0.72206
pancreas 328	0.9031689	0.72354
fetal lung 121	0.9276332	0.72394
fetal lung 125	0.9178656	0.72590
fetal lung 139	0.9131035	0.73170
pancreas 327	0.8935244	0.73268
fetal large intestine 53	0.8837529	0.74042
ES derived mesoderm 285	0.9186532	0.74746
fetal lung 136	0.9118358	0.74972
fetal lung 114	0.9210033	0.75116
fetal lung 126	0.9182952	0.75266
fetal lung 108	0.9004720	0.75662
fetal lung 120	0.9078062	0.75968
fetal lung 131	0.9159366	0.76384
fetal lung 122	0.8989975	0.76410
fetal heart 45	0.8934116	0.76608
fetal lung 137	0.9065761	0.76846
fetal lung 135	0.9022408	0.77150
fetal stomach 252	0.8749523	0.77202
fetal lung 127	0.8961431	0.77812
fetal stomach 245	0.8704363	0.77864
fetal adrenal gland 28	0.9045491	0.77922
fetal heart 42	0.8833713	0.78696
fetal heart 44	0.8622558	0.78722
fetal stomach 240	0.8579484	0.78734
fetal lung 118	0.9063214	0.79186
fetal stomach 247	0.8373428	0.79700
fetal heart 49	0.8793598	0.79764
fetal lung 110	0.8864470	0.80102
fetal kidney 85	0.8897331	0.81026
fetal lung 140	0.8917805	0.81052
fetal lung 124	0.8865153	0.81172
fetal lung 142	0.8920588	0.81330
fetal lung 132	0.8847981	0.81350
fetal lung 109	0.8922677	0.82306
fetal stomach 251	0.8525088	0.82392
fetal lung 130	0.8819134	0.82466
fetal heart 46	0.8628125	0.82494
ES derived mesoderm 286	0.8953376	0.82510
fetal lung 123	0.8760354	0.82698
fetal kidney 83	0.8390799	0.83302
fetal stomach 242	0.8121621	0.83380
fetal muscle 191	0.8598377	0.83548
fetal lung 113	0.8798899	0.83702
fetal stomach 241	0.8332677	0.83710
fetal stomach 246	0.8292723	0.84150
fetal adrenal gland 24	0.8460180	0.84228
fetal lung 138	0.8660352	0.84774
fetal lung 115	0.8740753	0.84938
fetal muscle 149	0.8476112	0.85006
fetal stomach 253	0.8229656	0.85132
fetal heart 51	0.8534635	0.85610
fetal lung 119	0.8752223	0.85644
fetal stomach 250	0.8199010	0.85710
fetal heart 48	0.8498224	0.85790
fetal heart 43	0.8552486	0.86012
fetal kidney 100	0.8391699	0.86726
fetal adrenal gland 25	0.8367103	0.86854
fetal adrenal gland 27	0.8373640	0.87902
fetal heart 52	0.8480106	0.88062
fetal muscle 180	0.8311639	0.88320
fetal stomach 248	0.8068318	0.88326
fetal heart 50	0.8308482	0.88396
fetal kidney 91	0.8421989	0.88474
fetal renal cortex 213	0.8383947	0.88784
fetal testes 255	0.8388379	0.88836
fetal muscle 148	0.8253190	0.89002
fetal stomach 249	0.7945017	0.89036
fetal kidney 81	0.8366456	0.89234
fetal renal pelvis 233	0.8191029	0.89532
fetal muscle 157	0.8381348	0.89564
fetal muscle 194	0.8435530	0.89694
fetal muscle 152	0.8307196	0.89910
fetal muscle 153	0.8119778	0.89962
fetal kidney 96	0.8210547	0.90282
fetal kidney 107	0.8102665	0.90314
fetal skin 234	0.8236734	0.90338
fetal renal pelvis 227	0.8140033	0.90374
fetal kidney 87	0.8169336	0.90388
fetal renal cortex 207	0.8159657	0.90432
fetal adrenal gland 26	0.8232790	0.90460
fetal renal pelvis 232	0.8015073	0.90486
fetal renal pelvis 226	0.8162645	0.90494
fetal kidney 102	0.7982906	0.90540
fetal muscle 187	0.8255117	0.90560
fetal thymus 263	0.8133983	0.90604
fetal renal cortex 211	0.7943586	0.90800
fetal renal pelvis 217	0.8010120	0.90858
fetal muscle 192	0.8206326	0.90890
fetal renal pelvis 216	0.7991671	0.91048
fetal renal pelvis 230	0.7910467	0.91050
fetal muscle 179	0.8171460	0.91060
fetal muscle 146	0.8157113	0.91096
fetal muscle 167	0.8202058	0.91334
fetal renal pelvis 218	0.7618702	0.91372
fetal kidney 106	0.8078851	0.91506
fetal muscle 166	0.8147775	0.91568
fetal renal cortex 215	0.8125664	0.91702
fetal muscle 165	0.8222087	0.91720
fetal muscle 155	0.7941736	0.91778
fetal renal pelvis 231	0.8124870	0.91902
fetal kidney 97	0.7975602	0.91948
fetal muscle 173	0.8048246	0.91972
fetal muscle 175	0.8116439	0.92036
fetal renal pelvis 228	0.7849789	0.92142
fetal muscle 190	0.7905550	0.92194
fetal muscle 158	0.8104472	0.92220
fetal kidney 84	0.7899012	0.92220
fetal kidney 94	0.7829632	0.92278
fetal muscle 188	0.8077409	0.92284
fetal kidney 99	0.7983164	0.92330
fetal kidney 92	0.8051073	0.92500
fetal testes 254	0.7950582	0.92522
fetal muscle 174	0.7987208	0.92552
fetal kidney 101	0.7969354	0.92570
fetal kidney 89	0.7892869	0.92726
fetal muscle 168	0.7958135	0.92846
fetal muscle 143	0.8143535	0.92858
fetal kidney 90	0.7839647	0.92924
fetal muscle 177	0.7842893	0.92966
fetal renal cortex 203	0.7827030	0.93216
fetal muscle 151	0.7797779	0.93230
fetal muscle 189	0.7992679	0.93276
fetal renal pelvis 219	0.7414029	0.93300
fetal muscle 186	0.7974299	0.93326
fetal muscle 154	0.7834468	0.93362
fetal muscle 159	0.7831931	0.93402
fetal renal cortex 214	0.7914269	0.93432
fetal renal cortex 205	0.7828388	0.93478
fetal muscle 184	0.8056870	0.93494
fetal kidney 104	0.7785681	0.93568
fetal renal cortex 210	0.7733823	0.93570
fetal muscle 183	0.8004652	0.93604
fetal renal pelvis 229	0.7863037	0.93752
fetal muscle 181	0.7919467	0.93794
fetal muscle 164	0.7870987	0.93830
fetal kidney 93	0.7634954	0.93968
fetal kidney 88	0.7861473	0.93984
ES cells 294	0.7620237	0.93988
fetal adrenal gland 23	0.8023503	0.93992
fetal renal cortex 206	0.7847264	0.94098
fetal ovary 195	0.7398447	0.94244
fetal muscle 144	0.7718854	0.94286
fetal kidney 95	0.7743552	0.94320
fetal muscle 160	0.7804129	0.94348
fetal renal pelvis 221	0.7817140	0.94370
fetal muscle 162	0.7911079	0.94396
fetal muscle 182	0.7871307	0.94404
fetal kidney 82	0.7493244	0.94406
fetal muscle 163	0.7768118	0.94416
fetal renal pelvis 222	0.7657307	0.94440
fetal muscle 156	0.7842118	0.94446
fetal muscle 145	0.7552518	0.94566
fetal renal cortex 209	0.7667346	0.94688
ES cells 295	0.7547395	0.94716
fetal lung 112	0.7985400	0.94826
fetal renal pelvis 223	0.7533437	0.94928
fetal muscle 147	0.7734433	0.94958
fetal renal pelvis 220	0.7629916	0.95036
fetal muscle 169	0.7763193	0.95044
iPS 302	0.7793007	0.95086
fetal renal cortex 204	0.7744767	0.95200
fetal renal pelvis 225	0.7569854	0.95252
fetal kidney 103	0.7664917	0.95372
fetal muscle 171	0.7814540	0.95422
fetal muscle 161	0.7633393	0.95580
fetal renal cortex 202	0.7662660	0.95608
fetal kidney 98	0.7539386	0.95630
fetal kidney 86	0.7136997	0.95684
fetal muscle 176	0.7830972	0.95748
fetal renal cortex 208	0.7483552	0.95784
fetal muscle 170	0.7773714	0.95848
fetal renal cortex 212	0.7661907	0.95872
ES cells 296	0.7360293	0.95928
fetal muscle 193	0.7523720	0.96080
fetal kidney 105	0.7558541	0.96376
iPS 303	0.7672269	0.96452
fetal muscle 185	0.7417542	0.96662
fetal muscle 150	0.7477838	0.96668
fetal renal pelvis 224	0.7537581	0.96750
fetal muscle 178	0.7473717	0.96856
iPS 304	0.7636987	0.96906
ES derived NP 291	0.7138237	0.97206
fetal spinal cord 237	0.7444199	0.98004
ES cells 293	0.7049183	0.98118
ES derived NP 292	0.6740421	0.98712
fetal spinal cord 236	0.6970857	0.99044
fetal spinal cord 235	0.7047877	0.99172
fetal brain 29	0.7743219	0.99318
fetal brain 37	0.7409569	0.99332
fetal spinal cord 238	0.6889217	0.99394
fetal brain 30	0.7715688	0.99522
fetal brain 35	0.7452638	0.99672
fetal brain 41	0.7013145	0.99722
fetal brain 36	0.7017309	0.99788
fetal spinal cord 239	0.6504220	0.99806
fetal brain 31	0.7343055	0.99836
fetal brain 33	0.6622666	0.99836
fetal brain 39	0.7092181	0.99840
fetal brain 40	0.7075816	0.99846
fetal brain 34	0.7103866	0.99882
fetal brain 38	0.6659531	0.99890
fetal brain 32	0.6767715	0.99930

Type 1 diabetes

DHS sample	fold enrichment	p value
Mobilized CD56 324	1.5975215	0.00044
fetal thymus 256	1.5862433	0.00090
CD4 14	1.5168100	0.00136
CD3 11	1.4947399	0.00218
Mobilized CD3 320	1.5705904	0.00300
CD8 20	1.4692920	0.00402
CD3 9	1.5045014	0.00422
fetal thymus 265	1.4570024	0.00478
CD14 4	1.4084976	0.00524
fetal thymus 258	1.4260768	0.00642
CD4 13	1.4765051	0.00754
fetal thymus 266	1.4096555	0.00978
CD8 21	1.4552172	0.01056
fetal lung 111	1.2745683	0.01062
fetal thymus 264	1.3850116	0.01204
CD14 3	1.3786645	0.01272
fetal lung 141	1.2542170	0.01418
fetal large intestine 61	1.3999981	0.01464
fetal lung 298	1.2789907	0.01594
Mobilized CD4 322	1.4223123	0.01640
CD56 17	1.3357003	0.01860
CD3 10	1.3612877	0.02016
fetal lung 299	1.2644402	0.02034
CD3 12	1.3898296	0.02412
Mobilized CD4 323	1.3727884	0.02624
fetal lung 301	1.2402264	0.02764
fetal lung 300	1.2336845	0.02814
Mobilized CD3 321	1.3871140	0.03102
fetal fibroblast 281	1.2114498	0.03222
fetal fibroblast 282	1.2000502	0.03528
fetal lung 133	1.2090385	0.03804
CD19 6	1.3334726	0.03926
fetal thymus 260	1.3049680	0.03988
CD19 7	1.3338731	0.04102
fetal fibroblast 272	1.1972723	0.04168
hematopoietic stem cell 310	1.2052091	0.04268
CD19 5	1.3077886	0.04290
fetal lung 128	1.1828371	0.04654
fetal thymus 261	1.2896075	0.04706
CD56 18	1.3130996	0.04888
fetal lung 114	1.1853627	0.04908
fetal fibroblast 274	1.1917485	0.04942
fetal fibroblast 268	1.1859851	0.04990
keratinocyte 335	1.1775039	0.05022
fetal fibroblast 273	1.1848985	0.05130
fetal lung 117	1.1948201	0.05180
breast 1	1.1853037	0.05326
fibroblast 330	1.1941025	0.05500
breast 2	1.1836731	0.05692
fibroblast 329	1.1858023	0.05804
fetal muscle 149	1.2225051	0.05916
keratinocyte 334	1.1674892	0.05924
hematopoietic stem cell 314	1.1972137	0.05928
CD8 22	1.3004426	0.06000
CD4 16	1.3132586	0.06010
fetal thymus 259	1.2742247	0.06126
fetal fibroblast 277	1.1795185	0.06304
ES derived MSC 289	1.2007644	0.06460
keratinocyte 336	1.1616432	0.06550
fibroblast 332	1.1738438	0.06658
fibroblast 331	1.1720771	0.06902
ES derived MSC 290	1.1944505	0.07070
fetal muscle 176	1.1875707	0.07162
fetal fibroblast 275	1.1597095	0.07348
fetal lung 126	1.1649647	0.07458
fetal fibroblast 271	1.1631627	0.07516
keratinocyte 333	1.1492968	0.07804
fetal thymus 257	1.2519589	0.08016
fetal lung 134	1.1764861	0.08148
fetal lung 140	1.1655182	0.08360
fetal lung 121	1.1565686	0.08534
fetal lung 131	1.1484028	0.08632
fetal fibroblast 276	1.1528148	0.08724
fetal lung 118	1.1500216	0.08736
fetal fibroblast 267	1.1581523	0.08964
placenta 200	1.1696892	0.08982
fetal lung 116	1.1480535	0.09168
fetal muscle 148	1.1823135	0.09234
fetal fibroblast 278	1.1525827	0.09352
hematopoietic stem cell 312	1.1713263	0.09360
fetal lung 119	1.1494564	0.09564
fetal fibroblast 269	1.1463479	0.09632
fetal muscle 181	1.1723619	0.09796
hematopoietic stem cell 308	1.1676901	0.09852
fetal lung 110	1.1610288	0.09948
Mobilized CD8 325	1.2431917	0.09970
fetal muscle 180	1.1730336	0.10640
fetal fibroblast 279	1.1426524	0.10684
fetal muscle 153	1.1725173	0.11054
fetal fibroblast 280	1.1371738	0.11378
fetal lung 108	1.1568824	0.11410
fetal lung 137	1.1409336	0.11740
fetal muscle 172	1.1383366	0.11806
fetal muscle 151	1.1677781	0.11840
hematopoietic stem cell 309	1.1576867	0.11868
fetal lung 115	1.1375374	0.11944
fetal lung 113	1.1312869	0.12316
CD8 19	1.2313683	0.12362
fetal muscle 167	1.1485593	0.12364
fetal large intestine 57	1.1563477	0.12416
hematopoietic stem cell 305	1.1573327	0.12708
fetal lung 142	1.1288375	0.12804
fetal muscle 143	1.1351429	0.13134
fetal muscle 152	1.1424679	0.13326
fetal lung 129	1.1315444	0.13510
fetal muscle 156	1.1439463	0.13522
fetal lung 136	1.1360693	0.13582
fetal small intestine 75	1.2557128	0.13612
fetal muscle 166	1.1412296	0.13846
fetal large intestine 67	1.1590182	0.13928
hematopoietic stem cell 313	1.1372980	0.14110
fetal muscle 169	1.1423209	0.14148
fetal lung 109	1.1157960	0.14288
fetal lung 120	1.1278393	0.14382
fetal muscle 154	1.1507417	0.14482
fetal muscle 194	1.1247807	0.14576
fetal lung 130	1.1246164	0.14738
hematopoietic stem cell 315	1.1434899	0.14880
fetal muscle 168	1.1393142	0.14926
fetal lung 125	1.1293916	0.14946
fetal muscle 187	1.1274501	0.15268
CD4 15	1.2214418	0.15302
placenta 199	1.1356051	0.15622
fetal muscle 157	1.1230606	0.15654
fetal fibroblast 270	1.1166801	0.15758
fetal muscle 164	1.1337363	0.15784
hematopoietic stem cell 317	1.1160152	0.16192
fetal muscle 179	1.1277236	0.16332
hematopoietic stem cell 306	1.1291425	0.16378
fetal muscle 192	1.1247203	0.16530
stomach 283	1.1509852	0.16808
fetal lung 138	1.1179226	0.16808
fetal large intestine 64	1.1359224	0.16850
fetal lung 124	1.1146882	0.17152
pancreas 327	1.1481238	0.17158
fetal thymus 262	1.1838275	0.17292
fetal muscle 183	1.1176831	0.17524
fetal small intestine 78	1.1204030	0.18030
hematopoietic stem cell 307	1.1222167	0.18044
hematopoietic stem cell 318	1.1293817	0.18092
fetal lung 139	1.1144955	0.18328
fetal small intestine 80	1.1294946	0.18346
fetal lung 135	1.1069232	0.18920
fetal muscle 155	1.1224057	0.18940
fetal large intestine 58	1.1143259	0.19398
fetal muscle 191	1.1137974	0.19430
placenta 201	1.1235622	0.19680
fetal muscle 190	1.1168011	0.20092
fetal small intestine 77	1.1218346	0.20354
fetal muscle 188	1.1041187	0.20500
fetal muscle 175	1.1055917	0.20578
fetal muscle 159	1.1107862	0.21008
fetal muscle 160	1.1052592	0.21342
fetal muscle 174	1.1047094	0.21364
fetal large intestine 55	1.1072675	0.21572
fetal small intestine 70	1.0983637	0.21850
fetal muscle 146	1.0976166	0.22062
placenta 198	1.0976202	0.22070
fetal lung 127	1.0933394	0.22082
pancreas 328	1.1149469	0.22098
fetal muscle 165	1.0938166	0.22168
fetal muscle 189	1.0976080	0.22346
hematopoietic stem cell 311	1.0987669	0.22420
fetal muscle 182	1.0934341	0.22884
fetal muscle 186	1.0915574	0.23288
muscle 341	1.1066717	0.23762
placenta 197	1.0994734	0.23826
hematopoietic stem cell 316	1.0987343	0.23984
fetal muscle 184	1.0849996	0.24006
fetal muscle 163	1.0913273	0.24386
fetal muscle 177	1.0951147	0.24422
CD20 8	1.0943152	0.24472
fetal skin 234	1.0939610	0.24538
fetal stomach 243	1.0958326	0.24568
fetal kidney 106	1.0912506	0.24828
fetal muscle 178	1.0874738	0.25070
fetal large intestine 56	1.0938143	0.25074
fetal muscle 147	1.0871947	0.25238
fetal lung 122	1.0835781	0.25278
fetal kidney 85	1.0778964	0.25548
fetal lung 132	1.0779113	0.25850
fetal muscle 193	1.0832918	0.26300
fetal large intestine 66	1.0905926	0.26422
fetal muscle 158	1.0767973	0.26510
fetal lung 123	1.0749950	0.26580
stomach 284	1.0930599	0.26742
fetal muscle 150	1.0831824	0.26874
fetal muscle 171	1.0747034	0.26938
ovary 326	1.0970211	0.27472
fetal small intestine 76	1.0869164	0.27720
fetal large intestine 60	1.0821490	0.28064
placenta 196	1.0788295	0.28210
fetal renal cortex 207	1.0766532	0.28470
fetal kidney 100	1.0760747	0.28510
fetal kidney 91	1.0688727	0.28986
fetal muscle 173	1.0704666	0.29246
fetal muscle 145	1.0742862	0.29772
fetal small intestine 79	1.0742451	0.30198
fetal stomach 252	1.0726193	0.30674
small intestine 342	1.0698871	0.30736
fetal large intestine 65	1.0689535	0.30942
fetal kidney 89	1.0658904	0.31038
fetal small intestine 68	1.0635409	0.31374
heart 297	1.0632727	0.32086
fetal small intestine 74	1.0621892	0.32574
fetal stomach 245	1.0633719	0.32962
melanocyte 340	1.0500504	0.33206
fetal muscle 161	1.0543404	0.33250
fetal large intestine 53	1.0634629	0.33474
fetal muscle 144	1.0549643	0.33538
fetal small intestine 72	1.0561883	0.33644
fetal stomach 251	1.0586311	0.33762
fetal large intestine 59	1.0568402	0.33968
fetal muscle 170	1.0497476	0.34030
fetal renal pelvis 220	1.0546427	0.34140
fetal muscle 185	1.0531926	0.34278
fetal renal pelvis 226	1.0504087	0.34632
fetal stomach 253	1.0551420	0.35030
fetal muscle 162	1.0451311	0.35122
fetal small intestine 69	1.0531200	0.35288
fetal large intestine 54	1.0498213	0.35514
fetal renal cortex 214	1.0465758	0.35758
fetal thymus 263	1.0465697	0.35762
fetal kidney 87	1.0439351	0.36320
fetal renal cortex 213	1.0425101	0.36366
fetal renal pelvis 217	1.0449409	0.36500
fetal stomach 244	1.0464806	0.36610
melanocyte 339	1.0357379	0.36780
melanocyte 338	1.0345786	0.36936
hematopoietic stem cell 319	1.0427013	0.37088
fetal stomach 248	1.0434757	0.37852
fetal renal pelvis 231	1.0355770	0.38262
fetal heart 42	1.0344182	0.38760
fetal renal pelvis 233	1.0320253	0.39564
fetal small intestine 73	1.0336231	0.39698
fetal kidney 82	1.0315139	0.40166
fetal renal pelvis 228	1.0308561	0.40474
fetal small intestine 71	1.0304958	0.40768
fetal renal pelvis 227	1.0255774	0.41202
fetal heart 52	1.0229778	0.41650
fetal kidney 81	1.0224870	0.42004
fetal kidney 88	1.0216584	0.42134
fetal kidney 92	1.0200439	0.42666
fetal renal cortex 211	1.0193958	0.43058
fetal large intestine 62	1.0215468	0.43184
fetal renal pelvis 221	1.0169170	0.43654
fetal renal pelvis 232	1.0176618	0.43672
fetal lung 112	1.0137657	0.44482
fetal heart 48	1.0141430	0.44664
fetal renal cortex 205	1.0137096	0.44860
fetal renal cortex 203	1.0118705	0.45270
fetal kidney 104	1.0119274	0.45342
melanocyte 337	1.0112067	0.45520
fetal renal pelvis 216	1.0093661	0.45898
fetal stomach 250	1.0094299	0.46014
fetal kidney 84	1.0086596	0.46112
fetal stomach 246	1.0081793	0.46184
fetal kidney 96	1.0040941	0.47556
fetal kidney 107	1.0031811	0.47608
fetal kidney 95	1.0004807	0.48392
fetal renal cortex 206	1.0003305	0.48636
ES derived trophoblast 287	1.0022832	0.48748
fetal renal pelvis 229	0.9979321	0.49228
fetal kidney 103	0.9944494	0.49788
fetal kidney 83	0.9941430	0.49870
fetal stomach 249	0.9911197	0.50134
fetal kidney 99	0.9904805	0.51300
fetal large intestine 63	0.9906008	0.51318
fetal adrenal gland 28	0.9929932	0.51634
fetal kidney 98	0.9870921	0.51994
fetal stomach 241	0.9852967	0.52068
fetal heart 46	0.9881444	0.52210
fetal renal pelvis 219	0.9839629	0.52310
fetal renal cortex 204	0.9891830	0.52402
fetal kidney 86	0.9785812	0.53166
ES derived trophoblast 288	0.9861895	0.53676
fetal stomach 240	0.9776511	0.53760
fetal stomach 247	0.9761027	0.53796
fetal kidney 102	0.9787341	0.54196
fetal kidney 105	0.9791967	0.54962
fetal renal cortex 202	0.9778834	0.55434
fetal kidney 94	0.9740685	0.55456
fetal stomach 242	0.9654144	0.55578
fetal heart 51	0.9761995	0.56252
fetal heart 50	0.9758771	0.56488
fetal kidney 97	0.9717577	0.56720
fetal renal pelvis 224	0.9727258	0.57120
fetal renal cortex 215	0.9711819	0.57428
fetal renal pelvis 225	0.9672574	0.57662
fetal kidney 101	0.9650282	0.59028
fetal heart 43	0.9695094	0.59190
fetal heart 47	0.9597284	0.59970
fetal renal pelvis 222	0.9579512	0.60040
fetal testes 255	0.9635390	0.60214
fetal renal pelvis 230	0.9549514	0.60496
fetal kidney 90	0.9568272	0.60810
fetal renal cortex 208	0.9554865	0.60922
fetal renal cortex 209	0.9551708	0.61408
fetal adrenal gland 26	0.9566721	0.61728
fetal kidney 93	0.9485000	0.62514
fetal renal pelvis 218	0.9389472	0.62576
fetal renal pelvis 223	0.9454981	0.63012
fetal heart 45	0.9491804	0.63984
fetal renal cortex 212	0.9465912	0.64514
fetal heart 49	0.9438132	0.65402
fetal testes 254	0.9327321	0.68332
fetal heart 44	0.9214477	0.68384
fetal renal cortex 210	0.9239247	0.69032
fetal adrenal gland 25	0.9144275	0.72306
fetal adrenal gland 27	0.9191156	0.72528
fetal adrenal gland 23	0.9178933	0.73816
fetal spinal cord 236	0.9119838	0.74622
fetal brain 41	0.9258832	0.74634
fetal brain 34	0.9297555	0.75114
fetal brain 35	0.9292780	0.76500
fetal ovary 195	0.8713776	0.77072
fetal spinal cord 237	0.9008717	0.79040
fetal brain 29	0.9234329	0.79262
fetal spinal cord 238	0.8980724	0.79272
fetal adrenal gland 24	0.8740527	0.79920
fetal brain 30	0.9168275	0.82236
ES derived mesoderm 285	0.8921037	0.83462
ES cells 294	0.8446986	0.84238
fetal brain 37	0.8861612	0.85470
ES cells 296	0.8309723	0.86380
fetal spinal cord 235	0.8665100	0.86636
fetal brain 39	0.8783436	0.87686
iPS 303	0.8521312	0.87960
ES derived NP 292	0.8324154	0.87970
fetal brain 31	0.8899240	0.88422
fetal brain 36	0.8669348	0.89226
fetal brain 40	0.8683173	0.89794
iPS 302	0.8353669	0.89856
fetal spinal cord 239	0.8423015	0.90424
ES derived NP 291	0.8053283	0.90740
ES cells 295	0.7995451	0.91022
iPS 304	0.8165930	0.93604
fetal brain 38	0.8316707	0.93624
ES cells 293	0.7791218	0.94186
fetal brain 33	0.8187867	0.94698
ES derived mesoderm 286	0.8311740	0.94856
fetal brain 32	0.8147995	0.96538

Celiac disease

DHS sample	fold enrichment	p value
Mobilized CD3 320	2.1736948	0.00002
Mobilized CD56 324	2.0792699	0.00002
CD8 20	2.0260561	0.00002
CD56 17	1.9567636	0.00002
CD4 14	1.9339112	0.00002
CD4 13	1.9360781	0.00004
CD3 11	1.8964768	0.00004
CD3 9	1.8868046	0.00004
CD14 4	1.7461884	0.00004
Mobilized CD4 322	1.8408645	0.00006
CD8 21	1.8804548	0.00008
CD8 22	1.8145010	0.00012
CD19 6	1.8022756	0.00012
CD3 10	1.7938559	0.00012
CD19 7	1.8658073	0.00014
CD3 12	1.8211748	0.00018
CD19 5	1.7459950	0.00018
hematopoietic stem cell 310	1.4943662	0.00020
CD4 16	1.7673076	0.00024
CD14 3	1.7421068	0.00024
Mobilized CD4 323	1.7294849	0.00034
fetal thymus 264	1.6366502	0.00044
fetal thymus 256	1.6721172	0.00050
fetal thymus 265	1.6200184	0.00080
hematopoietic stem cell 314	1.4374725	0.00080
CD56 18	1.7396770	0.00088
Mobilized CD3 321	1.7353802	0.00092
fetal thymus 266	1.5606758	0.00128
fetal large intestine 61	1.5883805	0.00144
fetal thymus 258	1.5565775	0.00176
hematopoietic stem cell 309	1.4454829	0.00214
hematopoietic stem cell 313	1.4018262	0.00238
hematopoietic stem cell 312	1.4170404	0.00270
hematopoietic stem cell 317	1.3523410	0.00342
CD8 19	1.6437246	0.00346
fetal thymus 261	1.5327694	0.00422
fetal thymus 260	1.4887727	0.00442
fetal small intestine 75	1.6677739	0.00498
fetal thymus 259	1.5005477	0.00690
Mobilized CD8 325	1.5023906	0.00828
hematopoietic stem cell 315	1.3725755	0.00862
fetal thymus 257	1.4387196	0.01176
hematopoietic stem cell 308	1.3114774	0.01264
hematopoietic stem cell 305	1.3444798	0.01296
CD4 15	1.5629099	0.01344
hematopoietic stem cell 318	1.3413956	0.01540
hematopoietic stem cell 306	1.3194788	0.01682
hematopoietic stem cell 307	1.3122765	0.01720
hematopoietic stem cell 311	1.3081401	0.01790
ES derived MSC 290	1.3097526	0.02188
fetal fibroblast 269	1.2524156	0.02372
fetal thymus 262	1.4312358	0.02872
ES derived MSC 289	1.2660707	0.04398
fetal fibroblast 270	1.2148446	0.05156
hematopoietic stem cell 316	1.2467453	0.05614
fetal fibroblast 273	1.1864554	0.06864
fetal lung 301	1.2083357	0.06984
fetal lung 300	1.1987019	0.07488
fetal fibroblast 279	1.1865979	0.07492
hematopoietic stem cell 319	1.2237762	0.07750
fetal fibroblast 277	1.1878599	0.08022
fetal fibroblast 281	1.1755791	0.08236
fetal fibroblast 282	1.1686175	0.08326
fetal fibroblast 280	1.1724344	0.08396
fetal fibroblast 275	1.1551905	0.10088
fetal lung 298	1.1705467	0.11970
fetal fibroblast 272	1.1455748	0.12116
breast 2	1.1372878	0.12700
fetal fibroblast 268	1.1414261	0.12752
fetal fibroblast 276	1.1376843	0.12930
keratinocyte 336	1.1241540	0.13874
keratinocyte 334	1.1232278	0.13984
fibroblast 331	1.1388217	0.14022
fetal fibroblast 274	1.1357757	0.14054
breast 1	1.1295700	0.14186
fetal fibroblast 267	1.1361895	0.15018
muscle 341	1.1740654	0.15344
fetal fibroblast 271	1.1175491	0.17010
fetal lung 299	1.1324337	0.17084
fetal fibroblast 278	1.1158776	0.18084
fibroblast 332	1.1138094	0.18246
fibroblast 330	1.1162118	0.18340
fibroblast 329	1.1144859	0.18516
keratinocyte 335	1.0994046	0.18936
placenta 200	1.1150044	0.19498
fetal lung 131	1.0979483	0.20778
keratinocyte 333	1.0819560	0.22824
fetal lung 128	1.0730997	0.26598
ES derived trophoblast 288	1.0693093	0.29952
stomach 284	1.0800939	0.30730
fetal lung 133	1.0604676	0.31236
melanocyte 339	1.0523288	0.31708
fetal lung 113	1.0581230	0.31712
fetal lung 116	1.0555911	0.31742
fetal lung 110	1.0621744	0.31762
fetal heart 47	1.0717555	0.32346
placenta 198	1.0560336	0.33688
ES derived trophoblast 287	1.0575580	0.34650
fetal lung 129	1.0474369	0.35262
melanocyte 340	1.0383465	0.36876
fetal lung 134	1.0404147	0.37562
heart 297	1.0438815	0.37748
fetal lung 111	1.0377092	0.37924
fetal lung 121	1.0348994	0.38250
fetal lung 115	1.0346728	0.38538
melanocyte 337	1.0287639	0.38994
fetal heart 42	1.0349268	0.39260
fetal lung 141	1.0318012	0.39578
fetal heart 49	1.0303332	0.40636
fetal lung 122	1.0277292	0.40778
placenta 197	1.0282888	0.41530
fetal lung 139	1.0238960	0.41842
fetal lung 142	1.0215903	0.42022
fetal lung 125	1.0227020	0.42236
fetal lung 126	1.0204904	0.42426
fetal lung 118	1.0198010	0.42630
fetal lung 137	1.0192865	0.42984
fetal lung 119	1.0176914	0.43880
fetal lung 138	1.0166869	0.43990
fetal lung 108	1.0164625	0.44002
placenta 196	1.0157736	0.44562
placenta 201	1.0161407	0.44850
fetal heart 51	1.0091128	0.45824
fetal heart 48	1.0091316	0.45828
fetal heart 43	1.0087035	0.45952
stomach 283	1.0102116	0.45962
placenta 199	1.0057703	0.47282
fetal lung 127	1.0039563	0.47864
fetal lung 140	1.0040479	0.47946
melanocyte 338	1.0013761	0.48988
fetal lung 136	0.9988474	0.49526
fetal lung 114	0.9965586	0.50148
fetal heart 52	0.9909304	0.51278
fetal lung 120	0.9911069	0.51610
fetal lung 109	0.9899379	0.52370
fetal heart 46	0.9763651	0.54058
fetal stomach 243	0.9777593	0.54372
fetal muscle 194	0.9832629	0.54462
fetal lung 130	0.9809538	0.54644
fetal muscle 172	0.9811300	0.54998
fetal renal pelvis 218	0.9665961	0.55030
fetal lung 135	0.9774090	0.55464
fetal heart 44	0.9640359	0.55476
fetal heart 50	0.9689751	0.56660
fetal heart 45	0.9654835	0.57354
fetal lung 123	0.9713971	0.57430
fetal lung 124	0.9708493	0.57516
fetal kidney 90	0.9613559	0.58324
fetal stomach 245	0.9533669	0.59362
fetal lung 117	0.9673346	0.59384
fetal stomach 244	0.9588490	0.59438
fetal large intestine 57	0.9586315	0.60784
fetal renal cortex 203	0.9421769	0.63648
fetal lung 132	0.9505262	0.63780
fetal kidney 93	0.9366699	0.63888
fetal stomach 247	0.9230709	0.63938
fetal muscle 177	0.9376630	0.64588
fetal stomach 253	0.9293419	0.65064
fetal large intestine 55	0.9416291	0.65142
fetal small intestine 77	0.9328808	0.65842
fetal stomach 250	0.9228284	0.66496
ovary 326	0.9113132	0.66854
fetal adrenal gland 28	0.9386099	0.67748
fetal renal pelvis 216	0.9239882	0.67756
fetal small intestine 70	0.9361739	0.67942
fetal large intestine 64	0.9286336	0.68038
fetal kidney 82	0.9165173	0.68114
fetal renal cortex 204	0.9301566	0.68128
fetal muscle 190	0.9205385	0.68406
fetal stomach 249	0.9107076	0.68852
fetal small intestine 76	0.9166485	0.68964
small intestine 342	0.9190414	0.69430
fetal kidney 107	0.9163670	0.70560
fetal small intestine 78	0.9231234	0.70690
fetal large intestine 65	0.9100188	0.70706
fetal stomach 241	0.9013437	0.71024
fetal renal cortex 208	0.9083386	0.71278
fetal stomach 252	0.9024725	0.72374
fetal small intestine 79	0.9000847	0.72610
fetal muscle 191	0.9083430	0.72656
fetal large intestine 56	0.9056129	0.72736
fetal renal pelvis 225	0.9014264	0.72928
fetal stomach 248	0.8961251	0.73516
fetal muscle 174	0.9053973	0.73546
fetal lung 112	0.9127233	0.73982
fetal renal pelvis 226	0.9038835	0.74054
fetal muscle 188	0.9063940	0.74222
fetal stomach 240	0.8838741	0.74412
fetal large intestine 58	0.9041019	0.75024
fetal muscle 146	0.9008595	0.75064
fetal skin 234	0.9004459	0.75274
fetal stomach 246	0.8804012	0.75392
fetal renal cortex 211	0.8893364	0.75450
fetal muscle 152	0.9013509	0.75500
fetal kidney 104	0.8941150	0.75542
fetal renal cortex 209	0.8901777	0.75698
fetal muscle 145	0.8829055	0.76296
fetal muscle 162	0.8980317	0.76522
fetal renal pelvis 228	0.8845986	0.76540
fetal renal pelvis 219	0.8669843	0.76674
fetal renal pelvis 222	0.8820682	0.76950
fetal kidney 106	0.8913198	0.77478
fetal kidney 100	0.8891015	0.77638
fetal kidney 86	0.8613324	0.77822
fetal renal pelvis 220	0.8838639	0.78124
fetal renal cortex 212	0.8866948	0.78362
fetal small intestine 69	0.8736490	0.78416
fetal large intestine 53	0.8678528	0.78446
fetal kidney 98	0.8792678	0.78630
fetal adrenal gland 23	0.8921112	0.78654
fetal muscle 183	0.8897720	0.78692
fetal kidney 101	0.8834549	0.78870
fetal muscle 186	0.8848993	0.79092
fetal muscle 156	0.8848400	0.79198
fetal renal pelvis 217	0.8739700	0.79284
fetal muscle 189	0.8813564	0.79554
fetal large intestine 60	0.8727353	0.79906
fetal kidney 97	0.8770557	0.79948
fetal stomach 251	0.8682991	0.79948
fetal muscle 143	0.8851884	0.80012
fetal renal cortex 214	0.8791064	0.80012
fetal adrenal gland 26	0.8812525	0.80216
fetal renal pelvis 227	0.8752921	0.80420
fetal small intestine 71	0.8667787	0.80562
fetal renal cortex 205	0.8721242	0.80658
fetal adrenal gland 25	0.8657229	0.80884
fetal small intestine 74	0.8700476	0.81072
fetal renal cortex 215	0.8750685	0.81132
fetal kidney 88	0.8716651	0.81228
fetal renal pelvis 232	0.8635452	0.81384
fetal thymus 263	0.8705384	0.81532
fetal renal pelvis 223	0.8577388	0.81582
fetal muscle 144	0.8629066	0.81698
fetal muscle 180	0.8634793	0.81902
fetal renal cortex 207	0.8702881	0.82064
fetal kidney 81	0.8707453	0.82254
fetal muscle 164	0.8643504	0.82282
fetal muscle 157	0.8742141	0.82332
fetal muscle 148	0.8642491	0.82390
ES derived mesoderm 285	0.8868136	0.82548
fetal kidney 89	0.8601385	0.82550
fetal renal pelvis 230	0.8503436	0.82658
fetal muscle 192	0.8678498	0.83046
fetal kidney 87	0.8647929	0.83092
fetal renal pelvis 224	0.8654154	0.83160
fetal muscle 163	0.8604070	0.83324
fetal muscle 149	0.8536106	0.83326
fetal muscle 147	0.8579886	0.83390
fetal renal cortex 206	0.8621157	0.83404
fetal kidney 83	0.8419813	0.83422
pancreas 328	0.8419595	0.83850
fetal kidney 96	0.8594147	0.83940
fetal testes 254	0.8546473	0.84138
pancreas 327	0.8331951	0.84502
fetal adrenal gland 24	0.8379687	0.84638
fetal muscle 161	0.8516598	0.84808
fetal stomach 242	0.8106810	0.84862
fetal kidney 102	0.8376942	0.84998
fetal kidney 105	0.8508314	0.85012
fetal muscle 168	0.8479161	0.85246
fetal muscle 187	0.8554039	0.85252
fetal muscle 167	0.8544067	0.85284
fetal kidney 95	0.8479061	0.85302
fetal muscle 193	0.8473286	0.85388
fetal large intestine 62	0.8323205	0.85640
fetal large intestine 66	0.8344033	0.85722
fetal muscle 184	0.8563921	0.85752
fetal muscle 176	0.8499523	0.85780
fetal kidney 91	0.8593428	0.85910
fetal muscle 158	0.8534253	0.85978
fetal kidney 84	0.8390823	0.86016
ES derived mesoderm 286	0.8802872	0.86092
fetal muscle 181	0.8419291	0.86228
fetal kidney 85	0.8590917	0.86238
fetal renal pelvis 221	0.8474651	0.86340
fetal kidney 94	0.8324657	0.86384
fetal adrenal gland 27	0.8435105	0.86438
CD20 8	0.8443762	0.86650
fetal renal cortex 210	0.8288565	0.86966
fetal large intestine 54	0.8287450	0.87250
fetal small intestine 72	0.8298503	0.87374
fetal large intestine 59	0.8231350	0.87386
fetal muscle 160	0.8347977	0.87448
ES derived NP 291	0.8174095	0.87504
fetal small intestine 68	0.8372241	0.87664
fetal muscle 166	0.8367953	0.87848
fetal muscle 182	0.8387502	0.87904
fetal kidney 99	0.8335007	0.87960
fetal kidney 103	0.8306691	0.88426
fetal kidney 92	0.8372944	0.88442
fetal muscle 165	0.8376888	0.88712
fetal muscle 171	0.8377620	0.88764
fetal small intestine 80	0.8224140	0.89022
fetal testes 255	0.8357138	0.89096
fetal muscle 150	0.8184630	0.89276
fetal muscle 155	0.8139914	0.89294
fetal muscle 173	0.8271075	0.89332
fetal large intestine 67	0.8129642	0.89392
fetal muscle 154	0.8149127	0.89396
fetal muscle 169	0.8235112	0.89598
fetal renal pelvis 233	0.8167831	0.89648
fetal muscle 159	0.8179013	0.89660
fetal muscle 175	0.8265199	0.89706
ES cells 293	0.8150739	0.90004
fetal spinal cord 237	0.8293575	0.90838
fetal small intestine 73	0.8018287	0.91020
fetal muscle 179	0.8098226	0.91040
fetal ovary 195	0.7777812	0.91044
fetal muscle 185	0.8050329	0.91050
fetal renal pelvis 229	0.8096317	0.91078
fetal muscle 153	0.8076382	0.91090
fetal renal cortex 202	0.8123260	0.91238
fetal muscle 178	0.8033617	0.91270
fetal large intestine 63	0.7931822	0.91284
fetal muscle 151	0.7885562	0.91770
fetal brain 34	0.8461101	0.91942
fetal renal pelvis 231	0.8128643	0.91978
ES derived NP 292	0.7782646	0.92766
fetal brain 29	0.8537570	0.92988
fetal renal cortex 213	0.8057757	0.93108
fetal muscle 170	0.7938086	0.94240
ES cells 296	0.7598417	0.94456
fetal brain 35	0.8334258	0.94458
fetal brain 30	0.8421009	0.94766
fetal spinal cord 236	0.7885239	0.94802
fetal brain 39	0.8149114	0.95212
fetal brain 41	0.8052847	0.95688
ES cells 294	0.7423214	0.96348
iPS 302	0.7698852	0.96404
ES cells 295	0.7304168	0.96548
fetal spinal cord 239	0.7564396	0.97374
fetal brain 32	0.7749491	0.97414
fetal brain 38	0.7726304	0.97524
fetal brain 37	0.7748535	0.97804
fetal spinal cord 238	0.7403456	0.98190
fetal brain 31	0.7958503	0.98360
fetal brain 33	0.7371448	0.98550
iPS 303	0.7363196	0.98556
fetal brain 36	0.7520850	0.98660
fetal spinal cord 235	0.7231403	0.98732
fetal brain 40	0.7381858	0.99456
iPS 304	0.6680130	0.99828

Lipid levels 4

DHS sample	fold enrichment	p value
melanocyte 338	1.2074684	0.00036
fetal adrenal gland 28	1.2285551	0.00062
melanocyte 339	1.1977714	0.00112
small intestine 342	1.2648624	0.00114
CD14 4	1.2764582	0.00122
melanocyte 337	1.1882585	0.00122
fetal large intestine 64	1.2464496	0.00150
melanocyte 340	1.2051047	0.00156
fetal small intestine 78	1.2286794	0.00162
fetal large intestine 55	1.2371249	0.00164
fetal small intestine 80	1.2437197	0.00168
fetal large intestine 57	1.2301819	0.00186
keratinocyte 334	1.1706486	0.00206
fetal large intestine 58	1.2251148	0.00218
fetal large intestine 67	1.2494622	0.00234
fetal small intestine 70	1.2067763	0.00250
CD20 8	1.2292118	0.00256
hematopoietic stem cell 310	1.1880301	0.00300
keratinocyte 336	1.1633269	0.00302
fetal large intestine 60	1.2384725	0.00336
CD14 3	1.2643469	0.00344
fetal small intestine 74	1.2264850	0.00350
Mobilized CD56 324	1.2506466	0.00432
fetal large intestine 56	1.2146319	0.00530
fibroblast 331	1.1705877	0.00574
keratinocyte 333	1.1481779	0.00584
CD8 21	1.2745468	0.00654
CD4 13	1.2662963	0.00654
Mobilized CD3 320	1.2745546	0.00682
fetal adrenal gland 25	1.2065876	0.00686
ES derived MSC 289	1.1896355	0.00734
fibroblast 330	1.1652589	0.00842
muscle 341	1.2212898	0.00848
fibroblast 329	1.1639955	0.00852
fibroblast 332	1.1580146	0.00886
CD56 18	1.2533240	0.00898
fetal adrenal gland 27	1.1873324	0.00900
fetal thymus 256	1.2186721	0.00944
placenta 200	1.1666611	0.01020
ES derived MSC 290	1.1815165	0.01026
fetal thymus 258	1.2227223	0.01046
fetal adrenal gland 24	1.2050215	0.01064
stomach 284	1.2051897	0.01092
fetal small intestine 68	1.1820615	0.01146
keratinocyte 335	1.1367104	0.01214
Mobilized CD4 323	1.2392876	0.01282
breast 2	1.1446534	0.01292
CD3 9	1.2304201	0.01316
fetal fibroblast 273	1.1504713	0.01364
fetal fibroblast 269	1.1437075	0.01440
fetal small intestine 77	1.1898573	0.01514
fetal adrenal gland 23	1.1620689	0.01526
fetal small intestine 73	1.1871347	0.01542
fetal fibroblast 279	1.1451066	0.01618
fetal small intestine 72	1.1800086	0.01640
CD56 17	1.1944622	0.01652
fetal lung 298	1.1567024	0.01686
fetal fibroblast 274	1.1431503	0.01694
Mobilized CD4 322	1.2307076	0.01772
fetal thymus 264	1.1977541	0.01794
fetal adrenal gland 26	1.1679579	0.01796
fetal thymus 265	1.1988608	0.01892
fetal large intestine 54	1.1810739	0.01928
fetal small intestine 69	1.1890240	0.01970
fetal fibroblast 277	1.1410411	0.02052
pancreas 328	1.1775009	0.02080
breast 1	1.1313354	0.02080
CD4 14	1.1903151	0.02154
CD3 11	1.1873603	0.02358
fetal fibroblast 276	1.1313196	0.02358
fetal large intestine 62	1.1797167	0.02370
fetal fibroblast 281	1.1323069	0.02410
fetal small intestine 79	1.1799946	0.02458
hematopoietic stem cell 314	1.1443057	0.02458
ovary 326	1.1978474	0.02480
fetal fibroblast 280	1.1301960	0.02572
fetal fibroblast 282	1.1254627	0.02622
placenta 198	1.1442105	0.02626
fetal thymus 260	1.1837141	0.02908
CD3 10	1.1827728	0.02908
fetal lung 299	1.1379799	0.02928
fetal lung 300	1.1347835	0.02950
fetal fibroblast 278	1.1299316	0.03056
ES derived mesoderm 286	1.1164566	0.03064
fetal fibroblast 268	1.1238286	0.03116
fetal fibroblast 275	1.1203482	0.03200
fetal lung 301	1.1317690	0.03220
fetal small intestine 76	1.1645711	0.03222
heart 297	1.1559424	0.03252
fetal thymus 266	1.1743011	0.03278
Mobilized CD3 321	1.2071478	0.03356
fetal large intestine 63	1.1599770	0.03374
CD19 7	1.1958847	0.03386
CD8 20	1.1742608	0.03388
fetal fibroblast 270	1.1245483	0.03444
fetal small intestine 71	1.1549653	0.03470
fetal lung 111	1.1241437	0.03526
placenta 201	1.1543648	0.03542
CD8 19	1.2061103	0.03596
Mobilized CD8 325	1.1963511	0.03642
stomach 283	1.1612141	0.03660
fetal thymus 259	1.1805271	0.03680
fetal fibroblast 272	1.1155757	0.03880
hematopoietic stem cell 312	1.1314601	0.03964
ES derived mesoderm 285	1.1172840	0.04060
fetal large intestine 65	1.1493680	0.04166
fetal fibroblast 267	1.1193449	0.04216
placenta 199	1.1336366	0.04260
pancreas 327	1.1557284	0.04266
fetal muscle 176	1.1222775	0.04436
fetal large intestine 66	1.1498931	0.04514
fetal heart 45	1.1324562	0.04540
fetal large intestine 59	1.1485615	0.04576
fetal fibroblast 271	1.1112180	0.04708
fetal testes 255	1.1289993	0.04720
placenta 197	1.1388572	0.04728
hematopoietic stem cell 309	1.1287600	0.04810
CD19 5	1.1641499	0.04898
fetal skin 234	1.1334282	0.04974
CD3 12	1.1788419	0.05070
CD19 6	1.1696353	0.05072
fetal lung 133	1.1091260	0.05186
fetal heart 52	1.1149504	0.05352
fetal large intestine 61	1.1614368	0.05440
fetal lung 117	1.1090745	0.05502
fetal lung 128	1.0970862	0.05756
fetal lung 140	1.1090232	0.05760
fetal muscle 180	1.1237042	0.05932
fetal heart 49	1.1178052	0.06370
fetal heart 47	1.1280246	0.06410
fetal heart 48	1.1159448	0.06504
hematopoietic stem cell 318	1.1237747	0.06738
hematopoietic stem cell 315	1.1181684	0.06746
fetal large intestine 53	1.1408026	0.06764
fetal muscle 169	1.1140841	0.06786
hematopoietic stem cell 305	1.1179502	0.06838
fetal heart 51	1.1117058	0.06944
fetal stomach 244	1.1301690	0.06992
fetal muscle 167	1.1079565	0.07016
fetal heart 46	1.1165608	0.07088
fetal testes 254	1.1203393	0.07106
hematopoietic stem cell 316	1.1184337	0.07668
CD4 15	1.1815350	0.07672
fetal muscle 149	1.1137068	0.07704
fetal lung 134	1.1015096	0.07796
fetal muscle 168	1.1093617	0.07914
fetal lung 116	1.0890618	0.08076
fetal muscle 151	1.1125376	0.08292
fetal thymus 257	1.1398263	0.08390
fetal muscle 184	1.0972947	0.08410
fetal muscle 166	1.1025848	0.08436
fetal muscle 157	1.0953520	0.08506
fetal muscle 153	1.1123547	0.08524
fetal thymus 261	1.1310077	0.08636
fetal muscle 181	1.1025116	0.08706
fetal lung 118	1.0873205	0.08780
iPS 304	1.0993788	0.08812
fetal muscle 143	1.0937554	0.08856
fetal muscle 165	1.0966082	0.08922
fetal heart 43	1.0984374	0.08936
ES derived trophoblast 288	1.0960317	0.09000
fetal lung 129	1.0938091	0.09028
fetal thymus 263	1.1049582	0.09064
placenta 196	1.1090610	0.09270
fetal muscle 175	1.0981318	0.09398
fetal muscle 182	1.0981843	0.09416
hematopoietic stem cell 308	1.0963507	0.09442
fetal muscle 179	1.0960590	0.09934
fetal lung 141	1.0846366	0.10116
CD8 22	1.1340218	0.10158
hematopoietic stem cell 306	1.0969344	0.10366
fetal muscle 164	1.0958797	0.10534
fetal lung 121	1.0810155	0.10656
CD4 16	1.1347386	0.10688
fetal muscle 172	1.0831523	0.10736
hematopoietic stem cell 317	1.0835680	0.10760
fetal muscle 152	1.0881400	0.11004
fetal lung 130	1.0817288	0.11768
fetal lung 125	1.0855739	0.11802
hematopoietic stem cell 307	1.0885826	0.12482
fetal muscle 148	1.0885596	0.12488
fetal heart 42	1.0887735	0.12522
fetal muscle 150	1.0884105	0.12544
ES cells 294	1.1025120	0.12720
fetal muscle 171	1.0821232	0.12894
fetal muscle 187	1.0814070	0.12902
fetal stomach 243	1.0928890	0.12912
fetal muscle 178	1.0888078	0.12940
fetal lung 119	1.0730767	0.13190
hematopoietic stem cell 311	1.0827646	0.13672
fetal lung 132	1.0770428	0.13734
fetal muscle 155	1.0869388	0.13838
fetal lung 114	1.0685373	0.14002
fetal muscle 154	1.0869128	0.14074
fetal muscle 193	1.0814046	0.14596
iPS 303	1.0778241	0.14940
fetal lung 120	1.0719040	0.14940
fetal muscle 159	1.0828213	0.15112
fetal renal pelvis 220	1.0857233	0.15300
fetal heart 50	1.0779039	0.15566
fetal renal cortex 213	1.0782511	0.15666
fetal muscle 191	1.0759191	0.15996
fetal muscle 173	1.0754845	0.16226
fetal muscle 170	1.0696646	0.16698
fetal kidney 81	1.0697450	0.16748
fetal lung 138	1.0676693	0.16876
fetal muscle 160	1.0731645	0.16968
ES derived trophoblast 287	1.0743613	0.17012
fetal muscle 174	1.0723073	0.17260
fetal muscle 183	1.0657094	0.17590
ES cells 296	1.0831456	0.17628
fetal muscle 194	1.0618890	0.18052
fetal lung 115	1.0599785	0.18060
fetal muscle 189	1.0672341	0.18292
fetal kidney 106	1.0719521	0.18622
fetal lung 124	1.0626500	0.18638
fetal muscle 147	1.0676902	0.18770
fetal kidney 91	1.0671843	0.18880
fetal muscle 158	1.0635019	0.19020
fetal stomach 240	1.0807885	0.19394
fetal stomach 250	1.0794909	0.19428
fetal lung 126	1.0544794	0.19640
fetal muscle 188	1.0616533	0.19706
fetal stomach 248	1.0750321	0.19924
fetal lung 110	1.0601368	0.19964
fetal kidney 85	1.0606456	0.20104
hematopoietic stem cell 313	1.0598369	0.20198
fetal kidney 95	1.0673197	0.20554
fetal stomach 253	1.0751405	0.20584
fetal renal cortex 207	1.0648474	0.20686
fetal lung 137	1.0545900	0.20754
iPS 302	1.0604969	0.21098
fetal renal pelvis 231	1.0602195	0.21148
fetal kidney 92	1.0624619	0.21156
fetal thymus 262	1.0865541	0.21864
fetal stomach 246	1.0721304	0.21870
fetal heart 44	1.0684242	0.22124
fetal small intestine 75	1.0948974	0.22318
fetal stomach 242	1.0781565	0.22358
fetal muscle 186	1.0545965	0.22576
fetal renal cortex 214	1.0578455	0.23220
fetal lung 127	1.0508657	0.23408
fetal stomach 252	1.0620010	0.23614
fetal kidney 87	1.0543420	0.24374
fetal lung 113	1.0446303	0.24388
fetal stomach 251	1.0590398	0.24402
fetal muscle 146	1.0501147	0.24444
fetal muscle 185	1.0540164	0.24460
fetal muscle 192	1.0502808	0.24500
fetal lung 123	1.0483945	0.24544
fetal stomach 241	1.0626680	0.24582
fetal renal pelvis 217	1.0556715	0.24954
fetal lung 122	1.0487385	0.24982
fetal lung 136	1.0460799	0.25184
fetal lung 108	1.0482040	0.25210
fetal kidney 89	1.0538820	0.25316
fetal kidney 99	1.0525649	0.25552
fetal muscle 156	1.0471718	0.26026
fetal muscle 163	1.0471756	0.26666
fetal renal pelvis 226	1.0480653	0.26986
fetal renal pelvis 228	1.0518467	0.27158
hematopoietic stem cell 319	1.0487086	0.27300
fetal kidney 88	1.0455811	0.27366
fetal lung 135	1.0416990	0.27428
fetal lung 109	1.0368142	0.27626
fetal lung 131	1.0366845	0.27766
fetal renal pelvis 232	1.0482603	0.28044
fetal kidney 105	1.0448546	0.28112
fetal lung 139	1.0416123	0.28246
fetal kidney 100	1.0451865	0.28610
fetal renal pelvis 227	1.0446408	0.28616
fetal muscle 190	1.0437582	0.28666
fetal muscle 145	1.0451693	0.28866
fetal muscle 161	1.0417194	0.28962
fetal kidney 103	1.0437581	0.29572
fetal stomach 247	1.0515950	0.29892
fetal renal cortex 202	1.0390349	0.30688
fetal renal pelvis 233	1.0400624	0.30692
fetal lung 142	1.0318752	0.30766
fetal muscle 162	1.0357688	0.30792
fetal kidney 83	1.0440574	0.30826
fetal renal cortex 204	1.0342653	0.31806
fetal brain 35	1.0261378	0.31886
fetal muscle 144	1.0356137	0.32190
fetal renal pelvis 221	1.0338610	0.32434
fetal stomach 245	1.0377883	0.32608
fetal kidney 84	1.0363914	0.32650
fetal kidney 82	1.0380021	0.32710
fetal stomach 249	1.0398344	0.32862
fetal renal pelvis 225	1.0348455	0.32984
fetal spinal cord 235	1.0308900	0.33330
fetal renal pelvis 219	1.0404281	0.33520
fetal renal pelvis 229	1.0318800	0.33628
fetal renal cortex 205	1.0327127	0.33638
fetal brain 34	1.0233651	0.34256
fetal spinal cord 237	1.0276985	0.34316
fetal brain 41	1.0239232	0.34668
fetal kidney 96	1.0289590	0.34784
fetal kidney 104	1.0299813	0.35058
fetal renal cortex 215	1.0279308	0.35242
fetal muscle 177	1.0280323	0.35522
fetal renal cortex 212	1.0280138	0.35542
ES cells 295	1.0311668	0.35644
fetal renal cortex 206	1.0260302	0.36448
fetal spinal cord 236	1.0249426	0.36874
fetal brain 40	1.0197799	0.37100
fetal kidney 94	1.0250945	0.37542
fetal brain 37	1.0184679	0.38000
fetal kidney 101	1.0224457	0.38196
ES derived NP 291	1.0254196	0.38202
fetal brain 31	1.0156662	0.38244
fetal renal cortex 211	1.0239328	0.38480
fetal renal pelvis 216	1.0216473	0.39022
fetal brain 39	1.0147915	0.39732
fetal kidney 102	1.0194537	0.40240
fetal renal pelvis 224	1.0169892	0.40316
fetal renal cortex 210	1.0186071	0.40360
fetal lung 112	1.0159022	0.40384
ES cells 293	1.0165169	0.41300
fetal kidney 97	1.0150396	0.41606
fetal brain 30	1.0101587	0.41944
fetal renal pelvis 223	1.0147849	0.42516
fetal brain 29	1.0093109	0.42668
fetal renal cortex 208	1.0120491	0.43438
fetal renal pelvis 230	1.0118194	0.43700
fetal kidney 98	1.0110135	0.43796
fetal ovary 195	1.0112860	0.44238
fetal brain 38	1.0082315	0.44572
ES derived NP 292	1.0097815	0.44890
fetal kidney 93	1.0065661	0.45986
fetal kidney 107	1.0023132	0.47928
fetal kidney 86	1.0015197	0.48324
fetal brain 36	0.9995179	0.49788
fetal kidney 90	0.9981029	0.50324
fetal renal pelvis 222	0.9956470	0.51210
fetal renal pelvis 218	0.9913093	0.52278
fetal renal cortex 203	0.9874413	0.55224
fetal spinal cord 239	0.9866683	0.56486
fetal renal cortex 209	0.9842930	0.56756
fetal brain 33	0.9778534	0.62656
fetal brain 32	0.9737135	0.66066
fetal spinal cord 238	0.9681171	0.66346

Height 3

DHS sample	fold enrichment	p value
fetal muscle 176	1.2294423	0.00002
fetal fibroblast 271	1.2184435	0.00002
fetal fibroblast 275	1.2049310	0.00002
fetal fibroblast 276	1.2067301	0.00004
fetal muscle 181	1.2423353	0.00006
fetal lung 111	1.2054863	0.00006
fetal fibroblast 272	1.1998804	0.00006
fetal lung 141	1.1881131	0.00006
fetal muscle 167	1.2144648	0.00008
fetal muscle 180	1.2267895	0.00010
fetal fibroblast 281	1.1929634	0.00010
fetal fibroblast 277	1.1880134	0.00014
fetal fibroblast 279	1.1764889	0.00014
fetal fibroblast 282	1.1758718	0.00014
fetal fibroblast 270	1.1812579	0.00020
fetal lung 119	1.1763594	0.00020
fetal fibroblast 273	1.1722880	0.00020
fetal fibroblast 268	1.1721245	0.00022
fetal muscle 152	1.2058159	0.00024
fetal fibroblast 267	1.1840262	0.00024
melanocyte 338	1.1575135	0.00024
fetal muscle 143	1.1913082	0.00026
fetal fibroblast 269	1.1757358	0.00030
fetal fibroblast 278	1.1746816	0.00030
fetal muscle 179	1.1952003	0.00032
fetal muscle 154	1.2109567	0.00034
fetal fibroblast 280	1.1689497	0.00038
fetal muscle 149	1.2104740	0.00040
melanocyte 339	1.1570894	0.00040
fetal lung 114	1.1624781	0.00042
fetal muscle 153	1.2078545	0.00046
fetal muscle 169	1.1964493	0.00048
fetal fibroblast 274	1.1694463	0.00048
fetal muscle 168	1.1906375	0.00050
fetal muscle 151	1.2101697	0.00054
fetal lung 117	1.1712654	0.00058
fibroblast 329	1.1675812	0.00062
fetal muscle 160	1.1979217	0.00066
melanocyte 340	1.1679226	0.00066
fibroblast 330	1.1623234	0.00096
fetal muscle 172	1.1618778	0.00096
fetal lung 133	1.1585503	0.00096
fetal muscle 166	1.1850834	0.00098
fetal muscle 150	1.1915082	0.00104
fetal muscle 164	1.1816398	0.00106
fetal lung 120	1.1655853	0.00106
fetal lung 299	1.1753083	0.00108
fetal lung 140	1.1611579	0.00110
fetal muscle 182	1.1762691	0.00112
fetal muscle 178	1.1857493	0.00118
fetal lung 118	1.1518451	0.00118
fetal lung 298	1.1712143	0.00122
fibroblast 332	1.1504850	0.00136
fetal muscle 165	1.1657514	0.00140
melanocyte 337	1.1301385	0.00142
fetal lung 121	1.1534352	0.00144
fetal muscle 148	1.1768493	0.00154
fetal muscle 187	1.1649098	0.00166
fetal lung 128	1.1412643	0.00178
fetal muscle 175	1.1712015	0.00180
fetal lung 301	1.1612556	0.00202
fetal lung 129	1.1555292	0.00202
fibroblast 331	1.1448443	0.00222
fetal muscle 157	1.1526029	0.00224
fetal thymus 264	1.2055700	0.00234
fetal lung 116	1.1372893	0.00240
fetal lung 300	1.1481721	0.00298
fetal muscle 183	1.1547386	0.00312
fetal muscle 158	1.1525398	0.00322
fetal thymus 258	1.2032447	0.00324
fetal muscle 155	1.1710121	0.00328
fetal muscle 184	1.1472416	0.00336
fetal thymus 256	1.1920022	0.00360
fetal muscle 156	1.1521742	0.00402
fetal muscle 194	1.1376710	0.00424
fetal muscle 159	1.1647462	0.00450
fetal lung 131	1.1269648	0.00450
fetal muscle 193	1.1559972	0.00502
fetal muscle 189	1.1512319	0.00502
fetal lung 132	1.1404437	0.00534
fetal lung 136	1.1385996	0.00614
fetal lung 113	1.1268526	0.00636
fetal muscle 171	1.1395344	0.00668
fetal thymus 263	1.1486309	0.00684
fetal lung 115	1.1255130	0.00748
fetal thymus 265	1.1755468	0.00814
fetal muscle 186	1.1373442	0.00824
fetal muscle 185	1.1475997	0.00870
fetal thymus 260	1.1739740	0.00888
CD56 18	1.1981302	0.00944
CD19 6	1.1931309	0.00966
fetal muscle 147	1.1390062	0.00966
fetal lung 134	1.1288251	0.01068
ES derived MSC 290	1.1323514	0.01170
fetal lung 130	1.1225961	0.01190
CD8 21	1.1921452	0.01202
fetal muscle 173	1.1303813	0.01278
fetal lung 138	1.1232628	0.01302
fetal muscle 188	1.1261760	0.01326
fetal lung 110	1.1247031	0.01334
fetal muscle 192	1.1247740	0.01342
ES derived MSC 289	1.1288838	0.01382
CD19 7	1.1842416	0.01394
fetal muscle 161	1.1316911	0.01434
fetal lung 109	1.1038703	0.01480
fetal heart 47	1.1406834	0.01650
fetal lung 139	1.1199219	0.01822
fetal lung 126	1.1039651	0.01828
fetal muscle 170	1.1153752	0.01846
fetal lung 142	1.1043258	0.01848
CD19 5	1.1621558	0.01910
fetal lung 108	1.1211229	0.01930
fetal lung 135	1.1137663	0.01990
fetal thymus 259	1.1613474	0.02010
fetal muscle 146	1.1162907	0.02068
fetal lung 112	1.1100231	0.02076
fetal lung 125	1.1133069	0.02140
fetal large intestine 61	1.1584686	0.02142
fetal lung 123	1.1099338	0.02178
fetal thymus 261	1.1522791	0.02228
fetal kidney 81	1.1113715	0.02238
fetal muscle 174	1.1177374	0.02296
fetal thymus 266	1.1445867	0.02360
CD3 10	1.1503072	0.02454
hematopoietic stem cell 310	1.1028449	0.02462
fetal lung 137	1.1030103	0.02620
CD3 9	1.1543403	0.02696
fetal muscle 190	1.1208683	0.02804
CD8 19	1.1723795	0.02868
fetal muscle 163	1.1136796	0.02902
fetal lung 124	1.1032759	0.02910
breast 2	1.0906279	0.02976
fetal muscle 177	1.1179458	0.02998
breast 1	1.0909006	0.03028
fetal heart 49	1.1146458	0.03060
fetal thymus 257	1.1484756	0.03084
fetal muscle 191	1.1102889	0.03154
fetal testes 255	1.1064010	0.03302
fetal small intestine 75	1.1835519	0.03350
keratinocyte 334	1.0809259	0.03380
keratinocyte 336	1.0806767	0.03458
Mobilized CD8 325	1.1539987	0.03472
keratinocyte 335	1.0812220	0.03512
hematopoietic stem cell 314	1.0997255	0.03746
keratinocyte 333	1.0773788	0.03780
Mobilized CD4 322	1.1488472	0.03890
hematopoietic stem cell 309	1.1030483	0.04104
CD3 11	1.1281217	0.04204
fetal heart 45	1.1044770	0.04212
fetal muscle 162	1.0967622	0.04218
CD3 12	1.1461012	0.04258
Mobilized CD56 324	1.1244746	0.04414
hematopoietic stem cell 308	1.0986046	0.04438
Mobilized CD3 321	1.1464131	0.04564
CD4 13	1.1404241	0.04588
hematopoietic stem cell 315	1.1027986	0.04602
Mobilized CD4 323	1.1382383	0.04734
fetal heart 46	1.1037952	0.04790
fetal heart 52	1.0916480	0.04988
fetal kidney 85	1.0905643	0.05008
fetal thymus 262	1.1476100	0.05150
muscle 341	1.1116025	0.05394
CD4 15	1.1577619	0.05580
fetal muscle 144	1.0967833	0.05724
hematopoietic stem cell 305	1.0944424	0.05796
CD56 17	1.1064328	0.06386
Mobilized CD3 320	1.1300990	0.06458
hematopoietic stem cell 318	1.0961777	0.06492
fetal lung 127	1.0836683	0.06564
fetal muscle 145	1.0975348	0.06616
ES derived NP 291	1.1041328	0.06720
fetal adrenal gland 28	1.0788558	0.06756
CD8 22	1.1201949	0.07238
fetal stomach 248	1.1015307	0.07250
fetal adrenal gland 27	1.0868315	0.07256
hematopoietic stem cell 316	1.0916219	0.07534
fetal heart 42	1.0861631	0.07562
CD8 20	1.1067718	0.07586
hematopoietic stem cell 312	1.0802726	0.07802
ES derived trophoblast 288	1.0780801	0.07900
hematopoietic stem cell 311	1.0826902	0.08028
fetal kidney 91	1.0809844	0.08028
ovary 326	1.1050303	0.08394
fetal adrenal gland 26	1.0826924	0.08534
fetal heart 48	1.0771182	0.09602
heart 297	1.0842836	0.09696
fetal heart 43	1.0717166	0.10020
fetal stomach 244	1.0849855	0.10172
fetal heart 44	1.0886147	0.10446
pancreas 328	1.0835351	0.10652
fetal adrenal gland 24	1.0821150	0.10852
ES cells 294	1.0835675	0.10940
fetal lung 122	1.0702860	0.11094
fetal stomach 253	1.0877790	0.11130
fetal stomach 243	1.0768084	0.11220
fetal renal pelvis 220	1.0758892	0.11620
fetal heart 51	1.0697602	0.11670
hematopoietic stem cell 313	1.0651785	0.12324
hematopoietic stem cell 306	1.0672038	0.12384
hematopoietic stem cell 307	1.0675100	0.12750
hematopoietic stem cell 317	1.0579562	0.13232
CD4 16	1.0932513	0.13466
fetal kidney 106	1.0662512	0.13650
ES derived mesoderm 286	1.0506508	0.13818
fetal adrenal gland 25	1.0692801	0.13940
ES derived trophoblast 287	1.0635061	0.14448
fetal large intestine 67	1.0680434	0.14750
placenta 201	1.0668303	0.15208
fetal stomach 249	1.0742154	0.15418
fetal stomach 250	1.0726742	0.15464
iPS 303	1.0573349	0.15622
CD4 14	1.0723084	0.15830
fetal small intestine 80	1.0595616	0.16538
CD14 3	1.0713007	0.16638
fetal renal cortex 207	1.0579216	0.16826
fetal stomach 251	1.0633395	0.17004
fetal stomach 246	1.0668630	0.17550
fetal kidney 103	1.0573094	0.18030
CD14 4	1.0624848	0.18310
pancreas 327	1.0635004	0.18396
fetal testes 254	1.0548036	0.18472
fetal skin 234	1.0528911	0.18544
fetal heart 50	1.0532213	0.18552
placenta 197	1.0561407	0.18654
fetal stomach 242	1.0697230	0.19322
placenta 199	1.0503707	0.19584
ES cells 296	1.0578003	0.19704
fetal kidney 87	1.0482803	0.20894
fetal kidney 100	1.0490600	0.20950
iPS 302	1.0448789	0.21528
fetal kidney 83	1.0543772	0.21564
stomach 283	1.0532048	0.21662
iPS 304	1.0426756	0.21696
placenta 196	1.0488646	0.21698
fetal large intestine 53	1.0546947	0.21940
ES cells 295	1.0518127	0.21942
fetal renal cortex 214	1.0456720	0.22246
fetal adrenal gland 23	1.0420326	0.22462
ES derived NP 292	1.0493424	0.22540
fetal ovary 195	1.0541147	0.22888
placenta 198	1.0413512	0.23028
placenta 200	1.0406130	0.23124
hematopoietic stem cell 319	1.0460092	0.23282
fetal stomach 245	1.0489942	0.23604
fetal renal pelvis 231	1.0409516	0.23612
fetal kidney 89	1.0443491	0.23624
fetal kidney 84	1.0438425	0.24230
fetal renal cortex 213	1.0389633	0.24418
fetal stomach 252	1.0442996	0.25152
fetal small intestine 69	1.0441252	0.25482
fetal stomach 241	1.0465424	0.25580
fetal large intestine 64	1.0396279	0.25990
fetal small intestine 78	1.0351893	0.26646
ES derived mesoderm 285	1.0297516	0.27076
fetal kidney 92	1.0347936	0.27156
fetal large intestine 54	1.0387101	0.27448
fetal kidney 99	1.0358887	0.27602
fetal renal pelvis 228	1.0381174	0.27722
fetal renal pelvis 226	1.0347992	0.27926
fetal large intestine 57	1.0346230	0.27998
fetal renal pelvis 227	1.0348937	0.28090
fetal renal pelvis 219	1.0414279	0.28558
fetal stomach 247	1.0438791	0.28596
ES cells 293	1.0346219	0.29082
fetal large intestine 66	1.0352761	0.29332
fetal renal pelvis 221	1.0310662	0.29488
fetal large intestine 55	1.0317372	0.29808
fetal small intestine 74	1.0319004	0.30022
stomach 284	1.0340717	0.30040
fetal large intestine 60	1.0314590	0.30732
fetal renal pelvis 217	1.0311304	0.30904
fetal renal cortex 205	1.0288744	0.31624
fetal small intestine 73	1.0286210	0.32454
CD20 8	1.0265674	0.32750
fetal large intestine 58	1.0254868	0.32842
fetal small intestine 71	1.0277132	0.33028
fetal small intestine 68	1.0252679	0.33286
fetal small intestine 79	1.0282936	0.33318
fetal renal pelvis 233	1.0253457	0.33680
fetal renal pelvis 232	1.0255134	0.34086
fetal renal cortex 206	1.0234490	0.34144
fetal large intestine 62	1.0272720	0.34170
fetal renal cortex 215	1.0231829	0.34280
fetal stomach 240	1.0287543	0.34292
fetal renal cortex 202	1.0230506	0.34398
fetal kidney 95	1.0234061	0.34798
fetal large intestine 59	1.0255086	0.34822
fetal kidney 94	1.0219850	0.35934
fetal kidney 105	1.0199473	0.36356
fetal kidney 98	1.0191326	0.37340
fetal small intestine 72	1.0190891	0.37924
fetal kidney 104	1.0177196	0.38302
small intestine 342	1.0182105	0.38338
fetal small intestine 76	1.0184058	0.38490
fetal kidney 88	1.0155678	0.39058
fetal renal pelvis 224	1.0147115	0.39274
fetal renal pelvis 229	1.0145515	0.39912
fetal large intestine 56	1.0151932	0.40160
fetal renal cortex 203	1.0135998	0.40470
fetal small intestine 77	1.0147536	0.40494
fetal renal pelvis 230	1.0147690	0.40584
fetal large intestine 63	1.0142705	0.40926
fetal kidney 82	1.0140040	0.41106
fetal large intestine 65	1.0125190	0.42078
fetal renal cortex 211	1.0106640	0.42848
fetal renal cortex 212	1.0076737	0.44308
fetal spinal cord 236	1.0071661	0.44342
fetal small intestine 70	1.0069363	0.44848
fetal kidney 96	1.0048261	0.46054
fetal kidney 102	1.0043121	0.46654
fetal kidney 97	1.0042313	0.46776
fetal kidney 107	1.0014295	0.48252
fetal renal cortex 208	1.0004755	0.49000
fetal renal cortex 204	0.9991964	0.50322
fetal renal pelvis 225	0.9977567	0.50656
fetal renal pelvis 216	0.9976776	0.50758
fetal kidney 86	0.9968780	0.50914
fetal renal pelvis 223	0.9975446	0.51158
fetal renal pelvis 222	0.9968042	0.51352
fetal renal pelvis 218	0.9947442	0.51860
fetal spinal cord 235	0.9921795	0.55160
fetal renal cortex 209	0.9909110	0.55202
fetal kidney 90	0.9904545	0.55416
fetal kidney 93	0.9895725	0.55754
fetal renal cortex 210	0.9883509	0.56522
fetal kidney 101	0.9871734	0.57866
fetal spinal cord 239	0.9738749	0.68118
fetal spinal cord 237	0.9721908	0.69552
fetal spinal cord 238	0.9645996	0.73728
fetal brain 37	0.9427901	0.89420
fetal brain 41	0.9339941	0.91972
fetal brain 36	0.9296531	0.93534
fetal brain 33	0.9124167	0.96076
fetal brain 39	0.9128030	0.97436
fetal brain 38	0.9041930	0.97732
fetal brain 40	0.9094824	0.98016
fetal brain 32	0.9030188	0.98366
fetal brain 34	0.9052521	0.98742
fetal brain 35	0.8971072	0.99392
fetal brain 31	0.8938872	0.99704
fetal brain 30	0.8940839	0.99760
fetal brain 29	0.8876523	0.99824

Waist−hip ratio

DHS sample	fold enrichment	p value
fetal muscle 176	1.9522641	0.00002
fetal kidney 85	1.9337447	0.00008
fetal muscle 181	1.8979515	0.00014
fetal muscle 167	1.8671887	0.00016
fetal muscle 179	1.8867635	0.00026
fetal muscle 180	1.9100895	0.00028
fetal lung 141	1.7108375	0.00036
fetal renal cortex 207	1.9427132	0.00042
fetal renal pelvis 231	1.8588656	0.00054
fetal kidney 106	1.9070687	0.00056
fetal muscle 187	1.7853517	0.00066
fetal kidney 92	1.8866560	0.00072
fetal renal pelvis 226	1.8325196	0.00102
fetal renal cortex 213	1.7864719	0.00102
fetal lung 111	1.6590087	0.00118
fetal kidney 95	1.8522285	0.00120
fetal muscle 143	1.6766860	0.00120
fetal muscle 161	1.8054878	0.00124
fetal lung 114	1.6112186	0.00126
fetal muscle 152	1.7285801	0.00128
fetal kidney 91	1.7686524	0.00142
fetal muscle 166	1.7274400	0.00152
fetal muscle 151	1.7951933	0.00174
fetal renal pelvis 227	1.8418479	0.00180
fetal muscle 182	1.7550804	0.00186
fetal renal cortex 202	1.7673969	0.00204
fetal lung 128	1.5602710	0.00212
fetal spinal cord 235	1.6971178	0.00216
fetal muscle 186	1.7094622	0.00246
fetal kidney 103	1.8236341	0.00250
fetal lung 119	1.5774690	0.00252
fetal skin 234	1.7152805	0.00254
fetal renal pelvis 221	1.7613391	0.00268
fetal muscle 188	1.7102902	0.00280
fetal muscle 178	1.7262084	0.00286
fetal lung 142	1.5953922	0.00288
fetal renal cortex 215	1.7165554	0.00290
fetal kidney 105	1.7221197	0.00322
fetal muscle 184	1.6461913	0.00332
fetal kidney 99	1.7323193	0.00342
fetal muscle 157	1.6169309	0.00352
fetal muscle 192	1.6596612	0.00360
fetal muscle 162	1.6630143	0.00370
fetal muscle 185	1.7429797	0.00374
fetal renal cortex 214	1.7449997	0.00388
fetal muscle 164	1.6975973	0.00390
fetal lung 140	1.5956631	0.00390
fetal lung 133	1.5761808	0.00400
fetal muscle 183	1.6449946	0.00432
fetal renal pelvis 220	1.7537864	0.00448
fetal renal pelvis 233	1.7125711	0.00456
fetal kidney 96	1.6741119	0.00460
fetal muscle 170	1.6396317	0.00460
fetal lung 113	1.5722575	0.00460
fetal kidney 84	1.7475578	0.00508
fetal muscle 165	1.5967253	0.00518
fetal lung 116	1.5154014	0.00522
fetal renal pelvis 229	1.6986327	0.00544
fetal lung 121	1.5336791	0.00566
fetal lung 117	1.5676467	0.00568
fetal renal pelvis 224	1.6479974	0.00578
fetal lung 109	1.4960446	0.00602
fetal renal cortex 212	1.6525713	0.00646
fetal muscle 189	1.6348770	0.00652
fetal muscle 168	1.6706920	0.00676
fetal lung 131	1.4957929	0.00700
fetal muscle 148	1.6357139	0.00708
fetal kidney 101	1.6460416	0.00740
fetal lung 129	1.5460014	0.00748
fetal muscle 175	1.6133953	0.00750
fetal muscle 147	1.6243897	0.00770
fetal lung 118	1.4915404	0.00794
fetal lung 138	1.5683524	0.00812
fetal muscle 194	1.5294447	0.00850
fetal kidney 100	1.6567207	0.00856
fetal muscle 169	1.5845567	0.00874
fetal fibroblast 276	1.4475088	0.00876
fetal muscle 163	1.6269255	0.00878
fetal kidney 89	1.6624512	0.00882
fetal kidney 97	1.6434536	0.00964
fetal kidney 94	1.6874022	0.00980
fetal muscle 149	1.6104022	0.01014
fetal kidney 102	1.6800888	0.01088
fetal muscle 172	1.4926724	0.01150
fetal renal pelvis 217	1.6577247	0.01154
fetal kidney 87	1.5978264	0.01166
fetal lung 124	1.5275381	0.01188
fetal muscle 190	1.6265141	0.01214
fetal muscle 155	1.6169041	0.01244
fetal muscle 177	1.6280984	0.01246
fetal kidney 98	1.6340090	0.01262
fetal renal cortex 204	1.5543846	0.01296
fetal lung 135	1.5227917	0.01334
fetal kidney 107	1.6095321	0.01354
fetal fibroblast 275	1.4160060	0.01382
fetal muscle 146	1.5345367	0.01390
fetal renal cortex 205	1.6005414	0.01412
fetal muscle 160	1.5569468	0.01462
fetal lung 136	1.5067151	0.01498
fetal muscle 150	1.5659209	0.01518
fetal lung 132	1.5029580	0.01524
fetal muscle 153	1.5911213	0.01566
fetal renal pelvis 222	1.6099544	0.01582
fetal kidney 104	1.5959949	0.01612
fetal kidney 88	1.5467098	0.01642
fetal spinal cord 238	1.4848522	0.01660
fetal lung 110	1.5010984	0.01700
fetal lung 115	1.4645808	0.01706
fetal renal cortex 208	1.5852832	0.01712
fetal kidney 90	1.5805651	0.01712
fetal renal cortex 203	1.5713263	0.01730
fetal renal cortex 206	1.5426166	0.01746
fetal renal pelvis 230	1.6412986	0.01764
fetal renal pelvis 228	1.6153246	0.01808
fetal fibroblast 272	1.4081533	0.01812
fetal kidney 81	1.5000227	0.01818
fetal lung 130	1.4836466	0.01856
fetal renal cortex 209	1.5656050	0.01932
fetal lung 125	1.4906156	0.01950
fetal muscle 171	1.4949434	0.01996
fetal muscle 158	1.4961354	0.02020
fetal lung 139	1.4988353	0.02050
fetal muscle 174	1.5326660	0.02082
fetal lung 123	1.4852219	0.02156
fetal kidney 83	1.6375460	0.02238
fetal muscle 159	1.5417330	0.02296
fetal lung 134	1.4601818	0.02326
fetal renal pelvis 223	1.5755063	0.02384
fetal fibroblast 271	1.3842106	0.02384
fetal lung 120	1.4451311	0.02396
fetal muscle 191	1.5053088	0.02418
fetal thymus 263	1.5313218	0.02422
fetal fibroblast 268	1.3829958	0.02436
fetal lung 126	1.4103631	0.02442
fetal renal pelvis 225	1.5524895	0.02480
fetal lung 112	1.4618466	0.02500
fetal muscle 156	1.4733557	0.02624
fetal renal pelvis 232	1.5593223	0.02676
fetal renal pelvis 216	1.5145564	0.02774
fetal renal cortex 211	1.5511540	0.02782
fetal muscle 154	1.5050865	0.02802
fetal renal cortex 210	1.5464793	0.02814
fetal muscle 173	1.4870779	0.02924
fetal fibroblast 282	1.3456230	0.03132
fetal kidney 93	1.5122017	0.03192
fetal lung 137	1.4056262	0.03242
fetal lung 122	1.4499605	0.03390
fetal muscle 144	1.4801844	0.03516
fetal fibroblast 273	1.3455540	0.03634
fetal muscle 193	1.4548284	0.03782
fetal kidney 82	1.5311582	0.03786
fetal fibroblast 274	1.3417588	0.03812
fetal adrenal gland 27	1.4665648	0.03888
fetal fibroblast 277	1.3529663	0.03956
fetal fibroblast 278	1.3417526	0.04592
fetal spinal cord 236	1.3954828	0.04718
fetal spinal cord 239	1.3509178	0.05238
melanocyte 338	1.2900415	0.05270
fetal heart 47	1.4254076	0.05290
fetal fibroblast 281	1.3119786	0.05484
fetal fibroblast 267	1.3213871	0.05574
fetal lung 127	1.3737178	0.05776
fetal muscle 145	1.4329358	0.05878
fetal adrenal gland 28	1.3580872	0.05976
fetal stomach 248	1.4742755	0.06046
fetal kidney 86	1.4862359	0.06194
fetal small intestine 80	1.3843918	0.06332
ES derived trophoblast 287	1.3555433	0.06352
fetal testes 255	1.3703790	0.06530
fetal renal pelvis 218	1.4988937	0.06736
melanocyte 337	1.2466338	0.07916
fetal large intestine 67	1.3650352	0.08514
fetal lung 108	1.3268052	0.08754
fetal stomach 251	1.3828051	0.08808
fetal large intestine 57	1.3280048	0.08864
melanocyte 339	1.2482717	0.08902
fetal stomach 250	1.4141801	0.08918
fetal stomach 246	1.3939426	0.09736
fetal adrenal gland 25	1.3549130	0.09776
fetal brain 32	1.2327726	0.10010
fetal large intestine 55	1.3114222	0.10492
fetal adrenal gland 23	1.2990830	0.10710
fetal small intestine 78	1.2948799	0.10716
fetal renal pelvis 219	1.3914522	0.10820
fetal heart 51	1.2830575	0.10978
fetal fibroblast 280	1.2366772	0.11086
fetal large intestine 64	1.3091395	0.11182
muscle 341	1.3460620	0.11292
ovary 326	1.3742268	0.11542
fetal heart 52	1.2586536	0.11620
pancreas 328	1.3204450	0.12290
ES derived trophoblast 288	1.2434546	0.12370
fetal stomach 243	1.3044822	0.12456
fetal stomach 252	1.3278519	0.12478
fetal large intestine 58	1.2662440	0.12838
fetal small intestine 68	1.2686199	0.13470
fetal fibroblast 279	1.2118285	0.13876
fetal stomach 249	1.3250271	0.13894
fetal adrenal gland 26	1.2674670	0.14150
fetal heart 48	1.2440595	0.14370
fetal brain 36	1.1972895	0.14370
fibroblast 331	1.2194958	0.14434
fetal heart 46	1.2539269	0.14500
fetal testes 254	1.2667803	0.14836
fetal spinal cord 237	1.2201721	0.15032
fibroblast 332	1.2143545	0.15058
fetal large intestine 63	1.2765924	0.15254
ES derived MSC 290	1.2311015	0.15444
fetal heart 45	1.2335948	0.15456
fetal stomach 241	1.3002500	0.15524
heart 297	1.2695576	0.15570
fetal stomach 240	1.3063985	0.15678
fetal heart 49	1.2320535	0.15706
fibroblast 329	1.2015218	0.16790
stomach 284	1.2519349	0.17594
fetal large intestine 59	1.2543382	0.17616
melanocyte 340	1.1800490	0.17688
fetal small intestine 70	1.2034522	0.18098
fetal large intestine 60	1.2299091	0.18518
fetal heart 50	1.2100768	0.18712
fetal large intestine 54	1.2268049	0.18742
fibroblast 330	1.1828371	0.19188
fetal adrenal gland 24	1.2421084	0.19254
placenta 196	1.2175529	0.19518
fetal large intestine 66	1.2264148	0.19706
fetal stomach 245	1.2361611	0.19768
ES derived MSC 289	1.1884856	0.20112
pancreas 327	1.2269647	0.20480
fetal small intestine 79	1.2208721	0.20546
fetal stomach 244	1.2208859	0.20586
keratinocyte 335	1.1464182	0.20688
fetal small intestine 72	1.2034441	0.21186
fetal large intestine 56	1.1988092	0.21264
stomach 283	1.2146706	0.21458
fetal brain 29	1.1132801	0.21586
CD14 3	1.2408332	0.21786
fetal large intestine 65	1.1964043	0.22166
fetal small intestine 74	1.1844199	0.22734
fetal heart 43	1.1578957	0.22750
fetal small intestine 77	1.1884256	0.22890
fetal small intestine 71	1.1840113	0.23408
placenta 198	1.1619857	0.23594
fetal brain 33	1.1334264	0.23876
fetal brain 31	1.0989657	0.24084
CD20 8	1.1641458	0.24348
fetal heart 42	1.1528089	0.24656
fetal stomach 247	1.2041671	0.24674
keratinocyte 333	1.1176152	0.24712
fetal small intestine 73	1.1714602	0.24768
hematopoietic stem cell 319	1.1775868	0.24904
keratinocyte 336	1.1155324	0.25696
fetal brain 30	1.0893056	0.25696
placenta 199	1.1498343	0.25712
fetal small intestine 76	1.1676931	0.25856
fetal ovary 195	1.1858073	0.25902
breast 1	1.1203331	0.26114
fetal stomach 253	1.1697612	0.26428
fetal fibroblast 270	1.1163158	0.26560
ES derived NP 292	1.1589798	0.26750
placenta 200	1.1302199	0.27204
hematopoietic stem cell 314	1.1330813	0.28118
breast 2	1.1054975	0.28686
fetal fibroblast 269	1.1033648	0.28856
fetal heart 44	1.1410764	0.28950
small intestine 342	1.1289891	0.30002
fetal large intestine 62	1.1305955	0.30150
fetal brain 34	1.0781014	0.30724
fetal large intestine 53	1.1297634	0.30746
fetal brain 35	1.0745104	0.30764
CD14 4	1.1315486	0.31560
placenta 197	1.1069844	0.32330
fetal small intestine 69	1.1116064	0.32642
fetal brain 41	1.0733476	0.32888
fetal brain 38	1.0731251	0.33356
fetal brain 39	1.0639687	0.33952
keratinocyte 334	1.0685202	0.34652
hematopoietic stem cell 310	1.0656199	0.36946
hematopoietic stem cell 308	1.0668880	0.37688
ES derived NP 291	1.0671077	0.38320
fetal brain 40	1.0426537	0.38700
hematopoietic stem cell 309	1.0607319	0.38718
ES cells 295	1.0470610	0.40408
hematopoietic stem cell 312	1.0465138	0.40906
ES derived mesoderm 285	1.0377927	0.41072
hematopoietic stem cell 317	1.0420413	0.41128
hematopoietic stem cell 305	1.0432651	0.41198
placenta 201	1.0419585	0.41354
fetal stomach 242	1.0323525	0.42640
hematopoietic stem cell 316	1.0320878	0.43108
hematopoietic stem cell 306	1.0315412	0.43160
CD3 10	1.0345982	0.43326
ES cells 296	1.0194454	0.44036
hematopoietic stem cell 311	1.0206090	0.44448
hematopoietic stem cell 313	1.0202314	0.44950
fetal thymus 265	1.0170040	0.45632
hematopoietic stem cell 307	1.0072835	0.46624
fetal brain 37	1.0059123	0.47056
CD19 6	0.9979134	0.47220
CD19 5	1.0014199	0.47372
CD19 7	0.9920770	0.47834
hematopoietic stem cell 315	0.9991382	0.48048
fetal thymus 256	1.0015719	0.48356
ES cells 293	0.9879628	0.48750
fetal lung 298	0.9859103	0.51748
hematopoietic stem cell 318	0.9561173	0.54082
fetal thymus 260	0.9486523	0.55048
fetal thymus 264	0.9478980	0.55454
fetal thymus 261	0.9384816	0.55962
ES derived mesoderm 286	0.9626959	0.56484
fetal thymus 258	0.9214098	0.58866
iPS 303	0.9303614	0.58928
fetal lung 299	0.9443121	0.59054
fetal thymus 266	0.9179825	0.59186
fetal thymus 257	0.9100803	0.59332
iPS 302	0.9289517	0.59546
fetal lung 300	0.9427653	0.60036
fetal lung 301	0.9355514	0.60908
CD56 17	0.9022489	0.61196
fetal thymus 262	0.8534145	0.63672
fetal small intestine 75	0.7805798	0.66720
iPS 304	0.8898739	0.67008
CD8 20	0.8416762	0.67318
fetal thymus 259	0.8455985	0.68022
Mobilized CD4 322	0.8098064	0.68692
CD3 12	0.8035018	0.68812
fetal large intestine 61	0.8178576	0.69804
CD4 15	0.7669185	0.69942
ES cells 294	0.8288703	0.70852
Mobilized CD3 321	0.7749954	0.71098
CD4 16	0.7730311	0.71152
Mobilized CD8 325	0.7814015	0.71424
CD3 11	0.8070960	0.71940
Mobilized CD56 324	0.8100594	0.72042
CD8 19	0.7501475	0.73052
CD4 14	0.7968666	0.73130
CD4 13	0.7395457	0.76312
CD8 22	0.7343121	0.76346
CD8 21	0.7397893	0.76408
Mobilized CD4 323	0.7336880	0.77254
CD56 18	0.7196163	0.79122
CD3 9	0.7069243	0.80806
Mobilized CD3 320	0.6124791	0.88278

Crohns disease 2

DHS sample	fold enrichment	p value
Mobilized CD56 324	1.7547082	0.00002
CD3 9	1.6948387	0.00002
CD4 13	1.6673497	0.00002
CD14 3	1.6577298	0.00002
CD8 20	1.6477306	0.00002
CD4 14	1.6466969	0.00002
Mobilized CD3 320	1.6391918	0.00002
Mobilized CD4 322	1.6374763	0.00002
CD14 4	1.6264785	0.00002
CD8 21	1.6244821	0.00002
CD3 11	1.6242722	0.00002
CD19 6	1.6236127	0.00002
CD56 17	1.6100993	0.00002
CD4 16	1.6059698	0.00002
CD3 10	1.6032473	0.00002
CD19 5	1.5876955	0.00002
fetal thymus 256	1.5267921	0.00002
hematopoietic stem cell 310	1.3483720	0.00002
Mobilized CD4 323	1.6236942	0.00004
CD3 12	1.5972325	0.00004
CD56 18	1.5947486	0.00004
CD8 22	1.5637758	0.00004
CD19 7	1.5532234	0.00004
fetal large intestine 61	1.5295530	0.00004
Mobilized CD8 325	1.5186829	0.00004
fetal thymus 264	1.4671685	0.00004
hematopoietic stem cell 314	1.3597137	0.00006
CD8 19	1.5863272	0.00008
fetal thymus 265	1.4648990	0.00008
fetal thymus 266	1.4613508	0.00008
Mobilized CD3 321	1.5900046	0.00012
fetal thymus 258	1.4600820	0.00012
breast 1	1.2999475	0.00012
breast 2	1.2843698	0.00024
hematopoietic stem cell 317	1.3168101	0.00028
fetal thymus 260	1.4265011	0.00032
hematopoietic stem cell 313	1.3132255	0.00032
fetal lung 301	1.3159203	0.00036
CD4 15	1.5507523	0.00046
fetal lung 300	1.2903191	0.00052
hematopoietic stem cell 309	1.3290551	0.00054
hematopoietic stem cell 312	1.3167681	0.00058
muscle 341	1.3816909	0.00062
keratinocyte 335	1.2417272	0.00064
stomach 284	1.3669624	0.00070
hematopoietic stem cell 308	1.3040441	0.00074
keratinocyte 336	1.2371331	0.00082
fetal small intestine 75	1.5164644	0.00086
fetal thymus 259	1.3987264	0.00090
fibroblast 331	1.2680847	0.00106
fetal fibroblast 273	1.2465959	0.00132
fetal fibroblast 281	1.2434105	0.00132
keratinocyte 334	1.2184345	0.00138
fetal fibroblast 282	1.2407090	0.00152
fetal fibroblast 272	1.2408594	0.00164
placenta 200	1.2648481	0.00172
keratinocyte 333	1.2154918	0.00172
fetal fibroblast 268	1.2419577	0.00176
fetal thymus 257	1.3874905	0.00178
fetal fibroblast 277	1.2514276	0.00186
stomach 283	1.3372819	0.00202
fetal fibroblast 276	1.2299639	0.00206
hematopoietic stem cell 307	1.2871567	0.00208
hematopoietic stem cell 318	1.2972798	0.00210
fetal fibroblast 274	1.2392389	0.00212
fetal fibroblast 279	1.2388540	0.00224
hematopoietic stem cell 311	1.2753929	0.00230
fetal thymus 261	1.3479658	0.00232
fibroblast 332	1.2398512	0.00236
fetal fibroblast 271	1.2333326	0.00236
hematopoietic stem cell 305	1.2853496	0.00238
fetal fibroblast 278	1.2378937	0.00248
ES derived MSC 289	1.2763580	0.00252
hematopoietic stem cell 316	1.2960457	0.00270
ES derived MSC 290	1.2779313	0.00270
hematopoietic stem cell 306	1.2767457	0.00270
placenta 197	1.2904882	0.00286
placenta 201	1.2954547	0.00296
fetal lung 299	1.2608892	0.00302
fibroblast 330	1.2379595	0.00326
fetal fibroblast 275	1.2187201	0.00348
fetal lung 298	1.2537922	0.00370
placenta 199	1.2649516	0.00386
fetal fibroblast 280	1.2168605	0.00394
placenta 198	1.2521098	0.00400
fetal fibroblast 267	1.2292453	0.00410
hematopoietic stem cell 315	1.2717296	0.00412
fetal thymus 262	1.3876909	0.00422
fibroblast 329	1.2242663	0.00428
placenta 196	1.2736530	0.00440
fetal large intestine 58	1.2533872	0.00454
fetal lung 134	1.2340165	0.00552
small intestine 342	1.2690818	0.00570
fetal large intestine 57	1.2452464	0.00700
fetal large intestine 64	1.2535697	0.00718
fetal large intestine 60	1.2613057	0.00720
pancreas 328	1.2650568	0.00868
fetal fibroblast 269	1.1943894	0.00876
fetal small intestine 80	1.2439552	0.00888
fetal small intestine 69	1.2661033	0.00910
pancreas 327	1.2700684	0.00986
fetal lung 141	1.1918502	0.01056
fetal large intestine 54	1.2454624	0.01076
fetal large intestine 55	1.2302842	0.01080
fetal large intestine 66	1.2469274	0.01244
fetal lung 128	1.1747348	0.01328
fetal small intestine 77	1.2400142	0.01344
fetal lung 111	1.1857376	0.01400
fetal small intestine 79	1.2459197	0.01408
fetal small intestine 74	1.2278533	0.01440
fetal small intestine 76	1.2436199	0.01442
fetal large intestine 56	1.2303119	0.01446
fetal lung 125	1.1990336	0.01476
hematopoietic stem cell 319	1.2296781	0.01490
fetal large intestine 59	1.2381118	0.01502
fetal large intestine 62	1.2448315	0.01506
fetal fibroblast 270	1.1821698	0.01512
fetal stomach 252	1.2446653	0.01534
fetal lung 117	1.1834004	0.01534
fetal lung 110	1.1948958	0.01586
fetal stomach 245	1.2436190	0.01596
fetal large intestine 65	1.2336223	0.01608
fetal small intestine 68	1.2151809	0.01686
fetal lung 133	1.1787270	0.01714
fetal lung 129	1.1832543	0.01774
fetal small intestine 78	1.1994678	0.01776
fetal stomach 242	1.2789411	0.01808
heart 297	1.2236103	0.01840
fetal stomach 244	1.2311296	0.01872
fetal large intestine 67	1.2206690	0.01882
fetal lung 122	1.1921567	0.01966
ovary 326	1.2555833	0.01968
fetal stomach 247	1.2673264	0.02112
fetal lung 139	1.1871729	0.02144
fetal lung 130	1.1756418	0.02158
fetal large intestine 63	1.2213301	0.02162
fetal kidney 83	1.2309473	0.02172
fetal lung 108	1.1887529	0.02224
fetal small intestine 71	1.2137992	0.02230
fetal lung 136	1.1771801	0.02250
fetal stomach 243	1.2076883	0.02262
fetal small intestine 70	1.1870636	0.02316
fetal stomach 240	1.2331328	0.02390
fetal stomach 241	1.2320194	0.02436
fetal lung 138	1.1737104	0.02500
fetal large intestine 53	1.2294547	0.02512
fetal lung 126	1.1577100	0.02576
fetal stomach 251	1.2118437	0.02640
fetal small intestine 73	1.2083923	0.02644
fetal stomach 253	1.2260407	0.02658
fetal lung 131	1.1497510	0.02750
fetal small intestine 72	1.2008880	0.02774
fetal lung 120	1.1661369	0.02802
fetal muscle 172	1.1602405	0.02814
fetal lung 135	1.1656146	0.03144
CD20 8	1.1874538	0.03148
fetal lung 123	1.1648664	0.03166
fetal lung 115	1.1536278	0.03206
fetal stomach 249	1.2165233	0.03234
fetal lung 127	1.1665194	0.03294
fetal stomach 246	1.2146893	0.03310
fetal lung 132	1.1589316	0.03476
fetal stomach 250	1.2084387	0.03660
fetal renal pelvis 220	1.1835796	0.03676
fetal lung 116	1.1398982	0.03796
fetal lung 137	1.1509621	0.03834
fetal kidney 89	1.1760195	0.04028
ES derived trophoblast 288	1.1541310	0.04078
fetal kidney 106	1.1726325	0.04106
fetal kidney 87	1.1709750	0.04228
fetal lung 142	1.1389193	0.04396
fetal renal pelvis 226	1.1676369	0.04494
fetal muscle 190	1.1726197	0.04528
fetal lung 114	1.1311733	0.04712
fetal muscle 191	1.1595216	0.04910
fetal renal pelvis 231	1.1572892	0.04912
fetal renal pelvis 227	1.1673447	0.04934
fetal lung 124	1.1430437	0.04938
fetal lung 140	1.1377722	0.05002
fetal lung 121	1.1318576	0.05048
ES derived trophoblast 287	1.1591546	0.05136
fetal renal pelvis 218	1.1991851	0.05158
fetal renal pelvis 232	1.1722255	0.05176
fetal lung 113	1.1320206	0.05384
fetal adrenal gland 26	1.1544317	0.05454
fetal kidney 91	1.1527446	0.05484
fetal renal cortex 211	1.1699441	0.05490
fetal adrenal gland 25	1.1622566	0.05508
fetal renal cortex 207	1.1600802	0.05536
fetal kidney 82	1.1767493	0.05552
fetal kidney 100	1.1571586	0.05626
fetal renal pelvis 217	1.1635555	0.05736
fetal renal pelvis 233	1.1593028	0.05742
fetal renal cortex 214	1.1524144	0.05870
fetal muscle 153	1.1590616	0.05896
fetal stomach 248	1.1746410	0.05898
fetal kidney 84	1.1603322	0.06114
fetal adrenal gland 24	1.1664057	0.06130
fetal muscle 189	1.1424718	0.06254
fetal lung 118	1.1211776	0.06260
fetal adrenal gland 28	1.1303283	0.06334
fetal renal pelvis 228	1.1621026	0.06534
fetal muscle 188	1.1381136	0.06572
fetal kidney 107	1.1509719	0.06596
fetal renal pelvis 230	1.1629664	0.06770
melanocyte 337	1.1078526	0.06784
fetal muscle 177	1.1487177	0.07006
fetal kidney 102	1.1555330	0.07048
fetal muscle 159	1.1459966	0.07104
melanocyte 338	1.1085684	0.07128
fetal muscle 192	1.1333259	0.07130
fetal muscle 194	1.1225504	0.07166
fetal muscle 149	1.1430391	0.07232
fetal heart 42	1.1421860	0.07242
fetal skin 234	1.1399703	0.07386
fetal kidney 85	1.1285677	0.07436
fetal kidney 88	1.1370507	0.07456
fetal muscle 145	1.1502051	0.07468
fetal renal pelvis 219	1.1693893	0.07658
melanocyte 340	1.1192027	0.07660
fetal lung 109	1.1098437	0.07660
melanocyte 339	1.1114323	0.07682
fetal renal cortex 215	1.1320770	0.07878
fetal renal cortex 213	1.1323938	0.07940
fetal muscle 186	1.1275130	0.08056
fetal muscle 180	1.1385903	0.08110
fetal heart 51	1.1332903	0.08150
fetal kidney 86	1.1633616	0.08176
fetal renal pelvis 216	1.1420726	0.08192
fetal renal pelvis 221	1.1319088	0.08284
fetal muscle 148	1.1323292	0.08296
fetal kidney 99	1.1365379	0.08334
fetal adrenal gland 23	1.1231034	0.08416
fetal renal pelvis 229	1.1335686	0.08538
fetal renal cortex 206	1.1299854	0.08548
fetal heart 44	1.1560775	0.08646
fetal lung 119	1.1091125	0.08914
fetal kidney 98	1.1351158	0.08926
fetal muscle 183	1.1194797	0.09088
fetal muscle 179	1.1237335	0.09098
fetal heart 47	1.1415534	0.09166
fetal renal cortex 205	1.1297849	0.09224
fetal heart 46	1.1325052	0.09254
fetal muscle 187	1.1185001	0.09260
fetal muscle 152	1.1174118	0.09340
ES derived mesoderm 286	1.0989386	0.09414
fetal kidney 96	1.1246574	0.09432
fetal lung 112	1.1130984	0.09438
fetal adrenal gland 27	1.1257401	0.09610
fetal kidney 104	1.1298750	0.09664
fetal renal pelvis 222	1.1315766	0.09784
fetal muscle 163	1.1227629	0.09838
fetal renal cortex 208	1.1311864	0.09896
fetal muscle 161	1.1207009	0.09908
fetal muscle 147	1.1212138	0.10142
fetal heart 45	1.1254597	0.10190
fetal kidney 97	1.1260279	0.10262
fetal muscle 174	1.1213077	0.10282
fetal renal pelvis 225	1.1281046	0.10366
fetal ovary 195	1.1445759	0.10678
fetal renal cortex 203	1.1221358	0.10752
fetal muscle 144	1.1209342	0.10848
fetal heart 50	1.1193712	0.10864
fetal muscle 155	1.1234695	0.10878
ES cells 295	1.1355366	0.11036
fetal renal cortex 209	1.1210275	0.11260
fetal muscle 193	1.1172408	0.11270
fetal kidney 101	1.1166521	0.11278
fetal muscle 146	1.1098308	0.11426
fetal kidney 90	1.1204161	0.11456
fetal heart 48	1.1156885	0.11474
fetal muscle 156	1.1099890	0.11478
fetal kidney 94	1.1245651	0.11524
fetal renal cortex 204	1.1089283	0.11592
fetal renal cortex 202	1.1143061	0.11636
fetal muscle 167	1.1065828	0.11662
fetal heart 43	1.1083955	0.11748
fetal heart 52	1.1065482	0.11908
ES cells 296	1.1319615	0.11918
fetal muscle 150	1.1137360	0.12030
fetal muscle 154	1.1167504	0.12116
fetal renal pelvis 223	1.1204867	0.12214
ES cells 294	1.1275637	0.12360
fetal muscle 175	1.1039284	0.12512
fetal renal cortex 210	1.1168989	0.12548
fetal muscle 171	1.1018521	0.12580
fetal kidney 95	1.1133259	0.12626
fetal muscle 157	1.0983936	0.12702
fetal muscle 166	1.1028275	0.12734
fetal muscle 160	1.1096128	0.12926
fetal muscle 173	1.1065294	0.12980
fetal muscle 151	1.1115491	0.13208
ES derived mesoderm 285	1.0906128	0.13246
fetal muscle 181	1.1026500	0.13370
fetal kidney 93	1.1139935	0.13390
fetal kidney 105	1.1032045	0.13518
fetal renal cortex 212	1.1023979	0.13558
fetal muscle 184	1.0934150	0.13836
iPS 303	1.0998550	0.13944
fetal kidney 81	1.0956717	0.13998
fetal testes 254	1.1035964	0.14440
fetal thymus 263	1.1010287	0.14630
fetal muscle 158	1.0927834	0.14682
fetal kidney 103	1.1041036	0.14810
fetal muscle 168	1.0998197	0.14816
fetal muscle 185	1.1002532	0.14934
fetal muscle 182	1.0936867	0.14956
fetal muscle 164	1.0960024	0.15014
fetal muscle 165	1.0888611	0.15378
fetal kidney 92	1.0953420	0.15390
fetal testes 255	1.0922050	0.15632
fetal heart 49	1.0983799	0.15728
fetal renal pelvis 224	1.0885831	0.16816
ES cells 293	1.0972415	0.17074
ES derived NP 291	1.1008820	0.17604
fetal muscle 162	1.0762324	0.19412
fetal muscle 170	1.0742457	0.19976
iPS 302	1.0754967	0.20538
fetal muscle 178	1.0744815	0.21514
fetal muscle 169	1.0717258	0.21518
fetal muscle 143	1.0640717	0.22546
ES derived NP 292	1.0615708	0.27508
iPS 304	1.0492703	0.28858
fetal spinal cord 236	1.0419963	0.31886
fetal spinal cord 237	1.0261842	0.37670
fetal spinal cord 235	1.0229580	0.38632
fetal spinal cord 238	1.0217702	0.39652
fetal muscle 176	1.0202731	0.40140
fetal spinal cord 239	0.9699309	0.62728
fetal brain 41	0.9726234	0.63784
fetal brain 38	0.9660052	0.66410
fetal brain 29	0.9721917	0.66680
fetal brain 39	0.9577210	0.71822
fetal brain 40	0.9498627	0.75602
fetal brain 36	0.9441612	0.76508
fetal brain 35	0.9438630	0.79998
fetal brain 34	0.9383800	0.80988
fetal brain 32	0.9284309	0.82896
fetal brain 37	0.9281416	0.83466
fetal brain 33	0.9164736	0.84606
fetal brain 31	0.9309757	0.85882
fetal brain 30	0.9230136	0.89712

Ulcerative colitis

DHS sample	fold enrichment	p value
CD14 4	1.4690047	0.00030
CD14 3	1.4249963	0.00198
CD56 17	1.3847545	0.00228
CD8 20	1.3333190	0.00996
CD3 10	1.3362529	0.01142
hematopoietic stem cell 317	1.2299132	0.01174
CD4 14	1.3107040	0.01294
Mobilized CD56 324	1.3108843	0.01540
CD19 5	1.3200261	0.01552
hematopoietic stem cell 312	1.2399614	0.01580
CD19 7	1.3303781	0.01930
CD19 6	1.3182528	0.02142
hematopoietic stem cell 313	1.2158585	0.02422
fetal lung 110	1.2135410	0.02516
fetal large intestine 64	1.2328910	0.02592
fetal large intestine 65	1.2481029	0.02658
CD3 11	1.2685876	0.02834
fetal large intestine 62	1.2506220	0.02990
fetal stomach 247	1.2888959	0.03036
fetal small intestine 76	1.2453566	0.03044
fetal lung 138	1.1964097	0.03124
hematopoietic stem cell 310	1.1902861	0.03164
fetal lung 122	1.2078081	0.03314
CD8 21	1.2926600	0.03374
fetal lung 131	1.1723998	0.03396
breast 1	1.1673508	0.03446
fetal small intestine 80	1.2165223	0.03454
keratinocyte 335	1.1574094	0.03472
ES derived MSC 290	1.2014889	0.03480
fetal lung 135	1.1949019	0.03508
fetal lung 129	1.1859309	0.03664
fetal large intestine 66	1.2335393	0.03844
ES derived MSC 289	1.1978280	0.03888
fetal small intestine 79	1.2310693	0.03928
fetal large intestine 59	1.2271564	0.03970
fetal small intestine 68	1.2081024	0.04072
keratinocyte 336	1.1502763	0.04076
fetal lung 125	1.1900748	0.04110
hematopoietic stem cell 311	1.1967244	0.04278
fetal large intestine 57	1.1980129	0.04302
hematopoietic stem cell 306	1.1976839	0.04328
fetal lung 300	1.1751266	0.04342
fetal small intestine 74	1.2096469	0.04442
small intestine 342	1.2073680	0.04552
fetal stomach 245	1.2286748	0.04574
fetal lung 134	1.1833239	0.04600
fetal large intestine 56	1.2080106	0.04620
breast 2	1.1549035	0.04712
fetal lung 127	1.1822116	0.04772
fetal lung 113	1.1605525	0.04828
fetal stomach 251	1.2131415	0.04890
keratinocyte 334	1.1410954	0.04922
fetal small intestine 77	1.2091014	0.04924
CD8 22	1.2599674	0.04948
fetal lung 142	1.1596960	0.04984
CD4 16	1.2715262	0.04990
hematopoietic stem cell 307	1.1894680	0.05036
fetal lung 123	1.1731849	0.05086
fetal large intestine 53	1.2336502	0.05130
fetal lung 139	1.1807478	0.05132
CD3 9	1.2487083	0.05144
fetal large intestine 58	1.1860481	0.05184
fetal large intestine 60	1.2006421	0.05332
hematopoietic stem cell 309	1.1834971	0.05416
fetal large intestine 55	1.1890712	0.05482
fetal large intestine 63	1.2067840	0.05514
fetal fibroblast 273	1.1522974	0.05576
fetal small intestine 72	1.1965134	0.05580
fetal lung 128	1.1439679	0.05876
fetal small intestine 70	1.1668693	0.05946
fetal stomach 248	1.2077145	0.06090
fetal lung 301	1.1613771	0.06126
Mobilized CD4 323	1.2390355	0.06206
fetal stomach 250	1.2137456	0.06212
CD3 12	1.2416633	0.06278
hematopoietic stem cell 314	1.1627146	0.06350
ES derived trophoblast 287	1.1796055	0.06376
fetal lung 136	1.1600409	0.06424
fetal stomach 249	1.2152019	0.06436
hematopoietic stem cell 319	1.1862707	0.06558
fetal lung 108	1.1677952	0.06606
fetal large intestine 67	1.1907779	0.06776
fetal small intestine 78	1.1637663	0.07034
CD4 13	1.2284935	0.07118
fetal lung 109	1.1335910	0.07158
fetal small intestine 73	1.1829535	0.07174
fetal fibroblast 279	1.1414576	0.07206
hematopoietic stem cell 316	1.1786765	0.07440
fetal small intestine 75	1.2720153	0.07556
muscle 341	1.1863415	0.07604
fetal lung 137	1.1452193	0.07636
hematopoietic stem cell 305	1.1674085	0.07688
fibroblast 331	1.1382154	0.07732
fetal lung 115	1.1408658	0.07848
fetal lung 124	1.1456179	0.07934
fetal stomach 240	1.1999978	0.07956
fetal lung 126	1.1349101	0.07980
fetal stomach 241	1.1935573	0.08154
hematopoietic stem cell 315	1.1644732	0.08198
stomach 284	1.1803105	0.08222
stomach 283	1.1817157	0.08304
fetal fibroblast 278	1.1366381	0.08354
fetal small intestine 71	1.1756060	0.08362
fetal renal cortex 203	1.1670159	0.08446
fetal lung 298	1.1440259	0.08722
fetal fibroblast 274	1.1321366	0.08866
Mobilized CD4 322	1.2102970	0.08960
CD56 18	1.2044540	0.09078
fetal large intestine 54	1.1666606	0.09178
CD20 8	1.1561053	0.09178
fetal fibroblast 269	1.1248176	0.09300
fetal small intestine 69	1.1733019	0.09344
keratinocyte 333	1.1077915	0.09398
fetal kidney 90	1.1618049	0.09422
Mobilized CD3 320	1.2152762	0.09426
ES derived trophoblast 288	1.1388770	0.09506
fetal renal pelvis 218	1.1950202	0.09518
Mobilized CD3 321	1.2134479	0.09540
fetal fibroblast 282	1.1202960	0.09600
fetal stomach 252	1.1688492	0.09616
fetal renal cortex 208	1.1590877	0.09832
fetal lung 111	1.1274272	0.09932
fetal lung 114	1.1205951	0.10092
fetal lung 130	1.1295826	0.10172
fibroblast 332	1.1219017	0.10190
fetal fibroblast 268	1.1189629	0.10226
hematopoietic stem cell 318	1.1531705	0.10492
fetal muscle 194	1.1251098	0.10644
fetal kidney 93	1.1546989	0.10968
fetal stomach 246	1.1664768	0.10982
fibroblast 330	1.1221729	0.11054
pancreas 327	1.1577418	0.11262
fetal kidney 101	1.1402024	0.11414
fetal muscle 161	1.1341513	0.11432
fetal fibroblast 280	1.1143919	0.11526
CD4 15	1.2150457	0.11604
fetal fibroblast 277	1.1186375	0.11668
fetal fibroblast 281	1.1127279	0.11690
fetal renal pelvis 216	1.1456584	0.11774
fetal fibroblast 267	1.1162067	0.12048
heart 297	1.1424868	0.12100
fetal stomach 253	1.1598172	0.12122
fetal renal pelvis 222	1.1438574	0.12178
fetal lung 121	1.1121937	0.12242
fibroblast 329	1.1132554	0.12460
fetal fibroblast 276	1.1038062	0.12874
fetal muscle 172	1.1100798	0.12886
hematopoietic stem cell 308	1.1214938	0.13044
fetal lung 141	1.1081927	0.13104
fetal renal cortex 211	1.1440253	0.13174
fetal large intestine 61	1.1606750	0.13242
fetal renal pelvis 219	1.1609244	0.13260
fetal lung 120	1.1137811	0.13442
fetal kidney 86	1.1548145	0.13610
fetal fibroblast 275	1.1021850	0.13610
fetal lung 132	1.1124315	0.13616
fetal lung 116	1.1010737	0.13664
fetal stomach 243	1.1317549	0.13928
fetal kidney 107	1.1285949	0.14062
fetal stomach 242	1.1642296	0.14102
CD8 19	1.1771960	0.14106
Mobilized CD8 325	1.1664844	0.14142
fetal muscle 177	1.1248659	0.14354
fetal thymus 257	1.1499385	0.14590
fetal renal cortex 204	1.1160800	0.14622
fetal muscle 190	1.1254252	0.14668
fetal kidney 97	1.1252230	0.14688
fetal muscle 145	1.1278281	0.14728
fetal renal pelvis 226	1.1215025	0.14788
placenta 201	1.1252653	0.14988
fetal kidney 94	1.1312138	0.15078
fetal lung 133	1.0985985	0.15218
fetal muscle 186	1.1073427	0.15774
fetal kidney 84	1.1228489	0.15784
fetal renal pelvis 225	1.1232649	0.15832
fetal renal cortex 209	1.1169166	0.16426
fetal kidney 88	1.1114541	0.16428
fetal kidney 98	1.1185485	0.16440
fetal muscle 163	1.1072729	0.16908
fetal kidney 96	1.1091471	0.17066
fetal renal cortex 206	1.1084597	0.17150
fetal muscle 144	1.1091315	0.17160
pancreas 328	1.1155747	0.17240
fetal fibroblast 271	1.0872713	0.17496
fetal kidney 89	1.1122886	0.17622
fetal kidney 104	1.1103852	0.17668
fetal renal pelvis 230	1.1183308	0.17692
fetal kidney 82	1.1213241	0.17764
fetal lung 119	1.0882475	0.17986
fetal kidney 105	1.1046464	0.18054
fetal fibroblast 270	1.0890327	0.18142
ovary 326	1.1242813	0.18602
placenta 200	1.0914556	0.18626
fetal renal pelvis 217	1.1082235	0.18826
fetal kidney 102	1.1112346	0.18934
fetal muscle 174	1.0973881	0.19004
fetal lung 117	1.0863978	0.19128
fetal stomach 244	1.1088622	0.19430
fetal muscle 148	1.0950094	0.19634
fetal muscle 162	1.0896331	0.19646
fetal muscle 168	1.0945200	0.19678
fetal renal cortex 210	1.1034810	0.19842
fetal thymus 261	1.1126173	0.20088
fetal lung 299	1.0868855	0.20266
fetal lung 112	1.0830373	0.20606
fetal muscle 192	1.0848042	0.21112
fetal renal pelvis 223	1.0979310	0.21298
fetal muscle 188	1.0833542	0.21378
fetal renal pelvis 231	1.0877612	0.21448
fetal renal pelvis 227	1.0934893	0.21510
fetal fibroblast 272	1.0731342	0.21684
fetal renal pelvis 232	1.0963957	0.21942
fetal renal pelvis 224	1.0870124	0.21984
placenta 196	1.0879939	0.22004
fetal kidney 91	1.0838897	0.22374
fetal muscle 189	1.0827624	0.22428
fetal kidney 95	1.0894196	0.22564
fetal muscle 155	1.0847644	0.22622
fetal renal cortex 215	1.0821842	0.22844
fetal thymus 262	1.1169993	0.22906
fetal muscle 193	1.0825449	0.22914
fetal kidney 83	1.0966634	0.23058
fetal muscle 191	1.0813760	0.23148
fetal renal cortex 212	1.0818417	0.23206
fetal renal pelvis 229	1.0839059	0.23248
fetal renal pelvis 221	1.0787382	0.24276
fetal kidney 106	1.0789064	0.24370
fetal thymus 266	1.0899794	0.24442
fetal renal cortex 213	1.0734364	0.24824
fetal skin 234	1.0766479	0.24840
fetal renal pelvis 233	1.0796996	0.24842
fetal muscle 187	1.0678471	0.25230
fetal renal cortex 214	1.0761180	0.25272
fetal muscle 147	1.0711836	0.25768
fetal thymus 264	1.0793460	0.26296
placenta 198	1.0653493	0.26338
fetal renal pelvis 228	1.0777620	0.26480
fetal lung 140	1.0591419	0.26770
fetal kidney 100	1.0709408	0.26778
placenta 199	1.0655586	0.26870
fetal muscle 164	1.0658925	0.26904
fetal kidney 99	1.0694632	0.27194
fetal kidney 87	1.0678274	0.27490
fetal renal cortex 205	1.0662589	0.27862
fetal muscle 184	1.0587330	0.27902
placenta 197	1.0648799	0.28070
fetal heart 52	1.0590465	0.28204
fetal renal cortex 202	1.0633120	0.28332
fetal muscle 183	1.0591911	0.28500
fetal thymus 259	1.0729914	0.29148
fetal muscle 146	1.0564073	0.29224
fetal heart 44	1.0670810	0.29752
fetal muscle 156	1.0556901	0.29794
fetal lung 118	1.0477295	0.30096
fetal thymus 260	1.0657806	0.30408
fetal heart 50	1.0552346	0.30570
fetal kidney 92	1.0537222	0.30772
fetal kidney 81	1.0504339	0.30804
fetal muscle 180	1.0499372	0.32306
fetal muscle 157	1.0437674	0.32520
fetal muscle 159	1.0502493	0.32586
fetal muscle 149	1.0490279	0.32712
fetal heart 46	1.0487631	0.32864
fetal thymus 256	1.0544143	0.32978
fetal heart 48	1.0462465	0.33092
fetal heart 51	1.0454155	0.33146
fetal muscle 153	1.0486675	0.33148
fetal thymus 265	1.0536261	0.33586
fetal muscle 171	1.0418993	0.33940
fetal muscle 160	1.0431115	0.34480
fetal heart 42	1.0419251	0.34834
fetal muscle 173	1.0395139	0.35386
fetal ovary 195	1.0464875	0.35868
fetal muscle 158	1.0353276	0.36148
fetal kidney 103	1.0355932	0.37054
fetal adrenal gland 24	1.0358789	0.37614
fetal adrenal gland 27	1.0337351	0.37684
fetal muscle 166	1.0306109	0.37866
fetal renal pelvis 220	1.0318675	0.38284
fetal heart 49	1.0281600	0.39048
fetal muscle 170	1.0269956	0.39060
fetal thymus 258	1.0329666	0.39260
fetal adrenal gland 25	1.0289953	0.39726
fetal muscle 165	1.0233363	0.40536
fetal heart 43	1.0207497	0.41310
fetal muscle 175	1.0209756	0.41360
fetal muscle 179	1.0202730	0.41990
fetal heart 47	1.0211713	0.42000
fetal muscle 185	1.0186640	0.42728
fetal muscle 152	1.0165790	0.42896
fetal thymus 263	1.0171078	0.43078
fetal testes 254	1.0171591	0.43210
ES cells 293	1.0120090	0.45066
ES cells 294	1.0083772	0.45948
fetal kidney 85	1.0061588	0.46146
ES derived NP 291	1.0083270	0.46432
fetal adrenal gland 28	1.0064815	0.46722
ES cells 295	1.0069949	0.46728
fetal muscle 151	1.0048360	0.47440
ES derived mesoderm 285	1.0049798	0.47478
fetal muscle 167	0.9997113	0.49138
fetal muscle 182	0.9966235	0.50494
fetal adrenal gland 23	0.9959670	0.50732
iPS 302	0.9926524	0.51728
fetal heart 45	0.9918013	0.51862
fetal muscle 178	0.9896295	0.52808
fetal muscle 154	0.9881049	0.53164
fetal spinal cord 237	0.9882451	0.53486
fetal muscle 150	0.9876011	0.53662
fetal adrenal gland 26	0.9867002	0.53870
ES cells 296	0.9824911	0.53970
fetal muscle 169	0.9859824	0.54276
fetal renal cortex 207	0.9821220	0.54884
fetal muscle 143	0.9860929	0.54886
ES derived mesoderm 286	0.9730494	0.61134
fetal spinal cord 238	0.9654172	0.61598
ES derived NP 292	0.9567246	0.62544
iPS 303	0.9599746	0.63554
fetal spinal cord 236	0.9583589	0.63846
fetal testes 255	0.9597670	0.64568
fetal muscle 181	0.9456093	0.68612
fetal muscle 176	0.9464773	0.69650
fetal brain 41	0.9478217	0.70494
iPS 304	0.9410227	0.70600
melanocyte 339	0.9463006	0.71828
melanocyte 340	0.9397330	0.72830
melanocyte 338	0.9285318	0.79166
fetal brain 39	0.9258208	0.79606
fetal spinal cord 239	0.9041361	0.81372
fetal spinal cord 235	0.8998263	0.82278
melanocyte 337	0.9198694	0.82626
fetal brain 38	0.9034237	0.83622
fetal brain 34	0.9145241	0.83696
fetal brain 35	0.9159674	0.84268
fetal brain 29	0.9061859	0.88794
fetal brain 40	0.8916835	0.88976
fetal brain 33	0.8733517	0.90034
fetal brain 36	0.8751378	0.90624
fetal brain 30	0.8835350	0.94468
fetal brain 37	0.8532776	0.94704
fetal brain 32	0.8494746	0.95038
fetal brain 31	0.8446051	0.97814

Celiac disease and rheumatoid arthritis

DHS sample	fold enrichment	p value
Mobilized CD3 320	2.8342268	0.00002
CD3 9	2.3991860	0.00004
CD3 11	2.3263162	0.00006
CD4 13	2.3384314	0.00010
CD8 21	2.2845091	0.00012
CD8 20	2.1185586	0.00024
fetal thymus 258	2.0877961	0.00028
CD56 17	2.0790874	0.00036
CD4 14	2.0677791	0.00040
CD8 19	2.2558716	0.00054
fetal thymus 266	2.0659466	0.00054
fetal thymus 264	2.0215778	0.00060
fetal thymus 265	2.0500481	0.00062
fetal thymus 260	2.0230165	0.00064
CD3 12	2.1253263	0.00080
CD56 18	2.1381445	0.00090
Mobilized CD4 322	2.0752672	0.00102
Mobilized CD3 321	2.0613282	0.00122
fetal large intestine 61	1.9641735	0.00126
Mobilized CD4 323	2.0375860	0.00140
CD8 22	2.0226641	0.00144
fetal thymus 256	2.1236087	0.00160
hematopoietic stem cell 310	1.6110884	0.00218
CD3 10	1.8890903	0.00226
fetal thymus 259	1.9368485	0.00278
fetal thymus 257	1.9060262	0.00302
fetal thymus 261	1.9281540	0.00374
hematopoietic stem cell 314	1.5665945	0.00424
Mobilized CD8 325	1.8797119	0.00480
CD19 5	1.8100695	0.00552
CD4 15	1.9515518	0.00692
Mobilized CD56 324	1.7318054	0.00836
fetal small intestine 75	1.9568546	0.01006
hematopoietic stem cell 309	1.5324824	0.01284
hematopoietic stem cell 315	1.5502458	0.01304
CD4 16	1.7492949	0.01388
CD19 6	1.7229954	0.01394
hematopoietic stem cell 308	1.5006411	0.01412
hematopoietic stem cell 305	1.5032101	0.01708
hematopoietic stem cell 318	1.5086819	0.01940
CD19 7	1.6700793	0.01950
fetal thymus 262	1.7022042	0.02610
hematopoietic stem cell 312	1.4287955	0.02792
hematopoietic stem cell 307	1.4380126	0.02886
hematopoietic stem cell 316	1.4779257	0.03038
hematopoietic stem cell 317	1.3812675	0.03078
hematopoietic stem cell 306	1.4096946	0.03846
hematopoietic stem cell 311	1.3865588	0.04482
ES derived mesoderm 285	1.3168034	0.05524
hematopoietic stem cell 313	1.3268709	0.06498
CD14 4	1.4321982	0.07056
fetal large intestine 64	1.3553470	0.07306
fetal small intestine 69	1.3814955	0.07340
fetal large intestine 55	1.3404632	0.07534
fetal small intestine 78	1.3150933	0.08708
fetal small intestine 80	1.3231210	0.09000
fetal small intestine 68	1.3186111	0.09074
fetal small intestine 77	1.3436103	0.09328
fetal large intestine 57	1.3079354	0.09604
placenta 200	1.2788269	0.09854
fetal large intestine 67	1.3274763	0.10186
fetal small intestine 74	1.2902693	0.11498
ES derived MSC 289	1.2881860	0.11572
breast 1	1.2297213	0.11644
fetal large intestine 60	1.2936728	0.12048
CD14 3	1.3514555	0.12062
fetal heart 47	1.3324212	0.12210
fetal adrenal gland 28	1.2434601	0.12340
fetal small intestine 70	1.2574285	0.12348
hematopoietic stem cell 319	1.2829563	0.12444
ES derived MSC 290	1.2630274	0.12960
small intestine 342	1.2866970	0.12976
CD20 8	1.2671328	0.13016
fetal large intestine 56	1.2720217	0.13402
fetal fibroblast 272	1.2081666	0.13568
fetal large intestine 58	1.2518519	0.14106
fetal fibroblast 279	1.2074575	0.14214
fetal fibroblast 280	1.2022728	0.14292
fetal small intestine 79	1.2731233	0.14634
fetal fibroblast 281	1.2027334	0.14640
fetal fibroblast 273	1.1997181	0.14662
placenta 198	1.2300150	0.14672
fetal large intestine 54	1.2536112	0.15198
fetal small intestine 72	1.2522489	0.15510
pancreas 328	1.2595210	0.15598
fetal large intestine 66	1.2555155	0.15870
fetal lung 299	1.2116257	0.15942
fetal small intestine 71	1.2379895	0.16638
fetal adrenal gland 25	1.2380837	0.16830
fetal fibroblast 271	1.1783971	0.17320
breast 2	1.1811533	0.17380
fetal fibroblast 282	1.1718745	0.17708
fetal fibroblast 268	1.1721318	0.17990
fetal fibroblast 269	1.1761250	0.18128
fetal fibroblast 267	1.1774414	0.18520
fetal stomach 243	1.2027499	0.18958
fetal fibroblast 270	1.1734481	0.19148
stomach 283	1.2232264	0.19288
fetal muscle 159	1.1888017	0.19664
fetal muscle 151	1.2003418	0.20282
fetal heart 45	1.2019742	0.20316
placenta 199	1.1846349	0.20380
fetal small intestine 73	1.1936125	0.20536
stomach 284	1.2097826	0.21314
pancreas 327	1.2052523	0.21314
fetal small intestine 76	1.2002034	0.21456
fetal large intestine 53	1.1994923	0.21700
melanocyte 337	1.1342641	0.21728
fetal lung 298	1.1608557	0.22156
ES derived trophoblast 288	1.1641794	0.22438
fetal adrenal gland 26	1.1674824	0.22644
fetal heart 49	1.1803007	0.22654
fetal fibroblast 276	1.1369718	0.22672
fetal lung 133	1.1385929	0.22980
fetal heart 42	1.1736144	0.23182
fetal large intestine 65	1.1777220	0.23554
fetal lung 128	1.1287846	0.23610
fetal lung 301	1.1480910	0.23732
melanocyte 340	1.1325585	0.23738
fetal fibroblast 277	1.1344800	0.23952
fetal muscle 154	1.1569087	0.24398
fetal large intestine 62	1.1701715	0.24838
fetal stomach 244	1.1593244	0.25170
placenta 196	1.1551344	0.25290
ES derived mesoderm 286	1.1147308	0.25340
fetal large intestine 63	1.1617061	0.25456
placenta 197	1.1524023	0.25484
keratinocyte 336	1.1180548	0.25616
fetal muscle 169	1.1395220	0.25696
fetal testes 255	1.1340876	0.25832
fetal fibroblast 274	1.1209938	0.25918
fetal muscle 149	1.1421270	0.26142
fetal lung 300	1.1292656	0.26216
muscle 341	1.1605261	0.26402
ovary 326	1.1733173	0.26404
keratinocyte 335	1.1121940	0.26444
fetal muscle 172	1.1198443	0.26598
fetal muscle 160	1.1340796	0.26648
melanocyte 338	1.1067999	0.26674
fetal stomach 248	1.1467329	0.26932
fetal adrenal gland 27	1.1332481	0.27118
fetal muscle 148	1.1289625	0.27308
fetal lung 116	1.1075038	0.27542
fetal large intestine 59	1.1417262	0.27680
fetal stomach 253	1.1437136	0.27700
fetal stomach 249	1.1419020	0.27848
fetal lung 140	1.1049730	0.28416
heart 297	1.1392976	0.28480
placenta 201	1.1352054	0.28550
fetal adrenal gland 23	1.1165604	0.28602
fetal fibroblast 278	1.1075388	0.28656
fetal muscle 187	1.1116327	0.28844
fetal muscle 153	1.1193059	0.29114
fetal fibroblast 275	1.0963329	0.29452
fetal muscle 166	1.1094962	0.29528
fetal heart 46	1.1177346	0.30320
fetal adrenal gland 24	1.1237735	0.30386
fetal renal pelvis 219	1.1227272	0.30756
fibroblast 332	1.0928734	0.31062
fibroblast 331	1.0936946	0.31118
melanocyte 339	1.0862820	0.31320
keratinocyte 334	1.0845415	0.31418
fetal muscle 143	1.0924231	0.31534
fetal muscle 168	1.0953818	0.32410
fetal stomach 245	1.1002632	0.32786
fetal muscle 185	1.0883321	0.33656
fetal muscle 191	1.0867895	0.33778
fetal thymus 263	1.0832449	0.33888
fetal stomach 242	1.1049179	0.33948
fetal muscle 164	1.0838766	0.34184
fetal testes 254	1.0868694	0.34222
ES derived NP 291	1.0918064	0.34274
ES cells 294	1.0893583	0.34452
fetal muscle 176	1.0732348	0.34838
keratinocyte 333	1.0651702	0.34872
fetal muscle 158	1.0724948	0.35258
fetal heart 51	1.0715322	0.35890
fetal heart 48	1.0709080	0.36180
fetal muscle 165	1.0674141	0.36194
fetal stomach 250	1.0774961	0.36396
fetal stomach 251	1.0727897	0.36590
fetal heart 43	1.0635739	0.36722
fetal muscle 157	1.0630920	0.36734
fetal muscle 194	1.0601291	0.36808
fetal stomach 246	1.0699188	0.37056
fetal muscle 182	1.0609118	0.37308
fetal lung 127	1.0601489	0.37532
fetal renal pelvis 220	1.0638451	0.37662
fetal heart 44	1.0682157	0.37902
fetal muscle 152	1.0589008	0.37924
fetal muscle 193	1.0601135	0.37926
fetal muscle 156	1.0564523	0.38010
fetal lung 115	1.0539820	0.38214
fetal muscle 155	1.0575250	0.38364
fetal kidney 81	1.0535106	0.38432
fetal stomach 240	1.0634266	0.38494
fibroblast 330	1.0547606	0.38538
fetal muscle 174	1.0540584	0.38910
fetal stomach 252	1.0560785	0.39028
fetal lung 108	1.0522526	0.39040
fetal renal pelvis 227	1.0523502	0.39280
fetal kidney 89	1.0525173	0.39334
fetal muscle 162	1.0484951	0.39450
fetal muscle 181	1.0496664	0.39532
fetal heart 52	1.0455013	0.39648
fetal muscle 179	1.0502011	0.39660
fetal lung 121	1.0441638	0.39754
fetal skin 234	1.0503156	0.40054
fetal brain 37	1.0367314	0.40116
fetal stomach 241	1.0493960	0.40144
fetal lung 118	1.0410084	0.40160
fetal muscle 146	1.0433925	0.40334
fetal lung 132	1.0429278	0.40366
ES derived trophoblast 287	1.0527489	0.40522
fetal muscle 183	1.0393817	0.41016
fetal renal cortex 207	1.0430608	0.41138
fetal lung 126	1.0356392	0.41526
fetal lung 141	1.0353341	0.41548
fetal heart 50	1.0337237	0.41818
fetal lung 120	1.0347036	0.41832
fetal kidney 91	1.0367181	0.41918
fetal lung 134	1.0333169	0.42318
fetal muscle 147	1.0303482	0.42770
fetal muscle 180	1.0321216	0.42946
fetal kidney 103	1.0318774	0.42966
fibroblast 329	1.0296092	0.43176
fetal muscle 150	1.0269591	0.43674
fetal muscle 167	1.0249417	0.43740
fetal muscle 177	1.0239137	0.44208
fetal lung 109	1.0205080	0.44394
fetal muscle 188	1.0191174	0.44600
fetal muscle 175	1.0173358	0.45398
fetal muscle 145	1.0155536	0.45444
fetal stomach 247	1.0110897	0.45632
fetal lung 111	1.0148090	0.45930
fetal muscle 189	1.0129660	0.46040
fetal lung 131	1.0137673	0.46460
fetal lung 112	1.0088563	0.46794
fetal muscle 171	1.0074910	0.47266
fetal lung 137	1.0069826	0.47296
fetal lung 119	1.0065141	0.47398
fetal lung 113	1.0071542	0.47420
fetal muscle 186	1.0037269	0.47456
fetal muscle 173	1.0027372	0.47704
fetal muscle 192	0.9989978	0.48424
fetal muscle 184	0.9995705	0.48576
fetal lung 117	1.0005408	0.48868
fetal lung 142	1.0007160	0.48916
fetal muscle 178	0.9966112	0.49012
fetal kidney 87	0.9962502	0.49300
fetal lung 114	0.9973458	0.49372
fetal renal cortex 213	0.9943837	0.49866
fetal muscle 190	0.9895616	0.49994
fetal renal cortex 214	0.9925904	0.50102
fetal renal cortex 205	0.9905887	0.50168
fetal muscle 170	0.9921405	0.50506
ES cells 296	0.9848774	0.50604
fetal kidney 84	0.9842822	0.50984
fetal kidney 82	0.9786502	0.51184
fetal kidney 93	0.9778246	0.51414
fetal renal cortex 206	0.9834143	0.51428
fetal renal pelvis 228	0.9768079	0.52158
fetal lung 124	0.9821384	0.52220
fetal muscle 144	0.9735009	0.52874
fetal kidney 83	0.9693490	0.52984
fetal renal pelvis 232	0.9691165	0.53258
fetal muscle 163	0.9691920	0.53528
ES derived NP 292	0.9619775	0.53880
fetal lung 129	0.9724411	0.54146
fetal renal pelvis 216	0.9641906	0.54218
fetal kidney 95	0.9666892	0.54352
fetal renal pelvis 231	0.9703913	0.54388
fetal lung 136	0.9727825	0.54474
ES cells 295	0.9568854	0.54884
fetal spinal cord 236	0.9642348	0.54944
iPS 303	0.9612427	0.55896
fetal lung 125	0.9588287	0.56390
fetal renal cortex 204	0.9545989	0.56646
fetal kidney 100	0.9530260	0.56914
fetal renal pelvis 226	0.9517590	0.56952
fetal renal pelvis 233	0.9524330	0.57036
fetal lung 130	0.9603099	0.57132
fetal lung 123	0.9577959	0.57362
fetal lung 138	0.9544010	0.57900
fetal renal cortex 211	0.9405465	0.58154
fetal renal pelvis 217	0.9423883	0.58170
fetal renal cortex 209	0.9401068	0.58250
fetal renal pelvis 229	0.9454578	0.58264
fetal renal cortex 208	0.9412592	0.58364
fetal kidney 88	0.9433117	0.58468
fetal kidney 86	0.9257800	0.58782
fetal ovary 195	0.9201255	0.58790
fetal kidney 107	0.9407831	0.58866
fetal lung 122	0.9402100	0.59570
fetal kidney 98	0.9336477	0.59772
fetal kidney 101	0.9362424	0.60166
fetal kidney 99	0.9337068	0.60278
fetal kidney 106	0.9372396	0.60356
ES cells 293	0.9262954	0.60676
fetal kidney 94	0.9257808	0.60870
fetal renal pelvis 225	0.9241792	0.60974
fetal kidney 102	0.9229828	0.61222
fetal lung 110	0.9337353	0.61478
fetal kidney 92	0.9323685	0.61512
fetal kidney 97	0.9218334	0.62110
fetal kidney 104	0.9200196	0.62478
fetal kidney 90	0.9136103	0.62998
fetal lung 135	0.9276886	0.63066
fetal lung 139	0.9240218	0.63676
iPS 302	0.9155167	0.63898
fetal renal cortex 202	0.9139486	0.64026
fetal renal pelvis 221	0.9154128	0.64292
fetal renal pelvis 224	0.9129997	0.64544
fetal renal pelvis 223	0.8979029	0.64564
fetal renal pelvis 222	0.9004192	0.64752
fetal kidney 96	0.9103750	0.64830
fetal renal cortex 210	0.8991843	0.64970
fetal renal pelvis 230	0.8937283	0.65508
fetal renal cortex 212	0.9050630	0.65818
fetal brain 40	0.9287407	0.65906
fetal renal cortex 203	0.8955824	0.66408
fetal muscle 161	0.8981802	0.66642
fetal kidney 105	0.8874838	0.68464
fetal renal pelvis 218	0.8460611	0.69672
iPS 304	0.8928020	0.69706
fetal brain 38	0.8968481	0.70188
fetal spinal cord 235	0.8838891	0.70602
fetal kidney 85	0.8940933	0.71028
fetal renal cortex 215	0.8755982	0.71246
fetal brain 34	0.8983340	0.72636
fetal spinal cord 237	0.8661401	0.73826
fetal spinal cord 238	0.8609528	0.74450
fetal brain 35	0.8953280	0.74588
fetal spinal cord 239	0.8391247	0.77916
fetal brain 32	0.8491867	0.79386
fetal brain 39	0.8507733	0.80846
fetal brain 41	0.8439745	0.80998
fetal brain 33	0.8241638	0.81704
fetal brain 36	0.8327834	0.83100
fetal brain 29	0.8534409	0.84282
fetal brain 30	0.8520707	0.84498
fetal brain 31	0.8061675	0.91036

Biliary cirrhosis

DHS sample	fold enrichment	p value
CD3 10	1.9499211	0.00014
hematopoietic stem cell 310	1.6085664	0.00026
CD19 5	1.8969972	0.00040
CD14 4	1.7535392	0.00048
CD19 6	1.8722418	0.00050
CD19 7	1.9180022	0.00078
CD14 3	1.7178333	0.00104
ES derived trophoblast 288	1.5691978	0.00194
fetal lung 111	1.4490290	0.00254
hematopoietic stem cell 314	1.5046381	0.00256
ES derived trophoblast 287	1.5904113	0.00276
hematopoietic stem cell 312	1.5019112	0.00288
CD4 14	1.6268019	0.00306
placenta 199	1.4882214	0.00448
Mobilized CD56 324	1.6026690	0.00452
hematopoietic stem cell 317	1.4285856	0.00462
fetal lung 141	1.3874191	0.00566
CD56 17	1.5452291	0.00624
hematopoietic stem cell 309	1.4602290	0.00688
placenta 200	1.4207928	0.00728
placenta 201	1.4993895	0.00748
placenta 197	1.4716992	0.00836
placenta 198	1.4216295	0.00926
hematopoietic stem cell 315	1.4587296	0.00936
hematopoietic stem cell 318	1.4726701	0.00974
hematopoietic stem cell 308	1.4239125	0.00984
Mobilized CD3 320	1.6503801	0.01012
fetal large intestine 57	1.4057684	0.01118
placenta 196	1.4596167	0.01120
fetal small intestine 80	1.4269175	0.01184
CD4 13	1.6033076	0.01188
hematopoietic stem cell 306	1.4221717	0.01266
fetal lung 128	1.3345377	0.01290
CD8 20	1.5145980	0.01336
fetal large intestine 60	1.4326685	0.01386
ES derived mesoderm 285	1.3584160	0.01424
fetal large intestine 58	1.3885143	0.01448
hematopoietic stem cell 311	1.3940436	0.01510
hematopoietic stem cell 313	1.3861687	0.01584
hematopoietic stem cell 305	1.4124119	0.01656
fetal large intestine 67	1.4222442	0.01666
ES derived mesoderm 286	1.3215376	0.01690
hematopoietic stem cell 316	1.4172240	0.01800
Mobilized CD4 323	1.5308759	0.01832
fetal lung 133	1.3242728	0.01888
small intestine 342	1.4144184	0.01934
fetal lung 108	1.3710530	0.01934
CD8 21	1.5441132	0.01940
fetal large intestine 64	1.3934783	0.01942
fetal small intestine 70	1.3594510	0.01996
fetal lung 129	1.3305421	0.02068
CD56 18	1.5305393	0.02084
fetal large intestine 56	1.3987309	0.02086
iPS 302	1.3719663	0.02094
CD3 9	1.5092759	0.02102
fetal small intestine 77	1.4031429	0.02158
fetal lung 113	1.3194268	0.02168
fetal lung 134	1.3429388	0.02194
fetal fibroblast 272	1.3296079	0.02252
fetal small intestine 68	1.3737203	0.02284
hematopoietic stem cell 307	1.3680552	0.02318
fetal large intestine 65	1.3955902	0.02470
fetal fibroblast 268	1.3120815	0.02560
CD3 11	1.4458704	0.02608
hematopoietic stem cell 319	1.3899352	0.02664
fetal fibroblast 271	1.3162661	0.02680
Mobilized CD8 325	1.5005141	0.02722
fetal thymus 266	1.4413835	0.02732
fetal small intestine 78	1.3426678	0.02736
fetal lung 109	1.2847391	0.02740
fetal large intestine 55	1.3515243	0.02778
fetal lung 137	1.3046515	0.02786
fetal large intestine 66	1.3975349	0.02846
fetal lung 127	1.3247060	0.02880
fetal lung 126	1.2886488	0.02898
fetal lung 140	1.3024889	0.02962
fetal lung 110	1.3160136	0.03080
muscle 341	1.3954475	0.03132
fetal fibroblast 277	1.3079184	0.03190
fetal lung 115	1.2904136	0.03204
fetal lung 122	1.3252371	0.03222
breast 1	1.2728946	0.03284
fetal lung 138	1.3079948	0.03318
fetal fibroblast 273	1.2996448	0.03320
fetal small intestine 74	1.3503911	0.03346
fetal thymus 260	1.4261633	0.03444
fetal fibroblast 274	1.2938395	0.03522
breast 2	1.2708394	0.03632
fetal thymus 258	1.4105508	0.03646
ES derived MSC 290	1.3341125	0.03684
fetal lung 136	1.2947387	0.03708
fetal small intestine 76	1.3704853	0.03712
fetal fibroblast 278	1.2971492	0.03734
fetal lung 114	1.2653034	0.03752
fetal kidney 85	1.3240606	0.03786
fetal thymus 256	1.4140587	0.03788
fetal small intestine 73	1.3530001	0.03796
CD20 8	1.3263779	0.03802
fetal fibroblast 267	1.2952912	0.03828
fetal lung 142	1.2739728	0.03882
fetal thymus 265	1.4096755	0.03910
CD4 16	1.4691853	0.03926
fetal small intestine 79	1.3608131	0.03962
keratinocyte 333	1.2357119	0.04066
fetal small intestine 69	1.3573824	0.04160
fetal thymus 257	1.4213023	0.04224
fetal lung 132	1.2819508	0.04256
fetal lung 139	1.2957740	0.04302
fetal fibroblast 281	1.2752967	0.04356
iPS 303	1.3014476	0.04376
fetal large intestine 53	1.3718942	0.04442
fetal small intestine 71	1.3316691	0.04452
stomach 283	1.3546091	0.04480
fetal fibroblast 282	1.2684776	0.04532
CD8 19	1.4590413	0.04540
fetal large intestine 62	1.3514206	0.04612
keratinocyte 334	1.2369243	0.04634
fetal muscle 191	1.3108532	0.04774
fetal lung 116	1.2480376	0.04792
fetal lung 131	1.2447892	0.04830
fetal lung 121	1.2540960	0.04948
fetal kidney 106	1.3277497	0.05022
ES derived MSC 289	1.3032721	0.05134
fetal large intestine 61	1.3792706	0.05326
fetal lung 124	1.2613071	0.05414
iPS 304	1.2754047	0.05480
fetal muscle 194	1.2593183	0.05504
keratinocyte 335	1.2220440	0.05596
fetal renal cortex 213	1.2946675	0.05620
fetal renal pelvis 231	1.2956347	0.05678
pancreas 328	1.3187583	0.05688
Mobilized CD4 322	1.4068893	0.05730
fetal renal pelvis 216	1.3138100	0.05772
fetal lung 125	1.2649677	0.05804
CD3 12	1.3982215	0.05882
fetal large intestine 59	1.3135710	0.05914
fetal muscle 193	1.2985320	0.05938
ES cells 293	1.3117142	0.05984
fetal large intestine 54	1.3077970	0.06004
fetal thymus 264	1.3482764	0.06010
fetal lung 118	1.2310189	0.06020
fetal lung 117	1.2425832	0.06046
fetal thymus 261	1.3614593	0.06172
fetal lung 300	1.2606725	0.06174
fetal muscle 188	1.2717068	0.06178
fetal small intestine 72	1.2939346	0.06184
fetal renal pelvis 226	1.2949075	0.06216
fetal fibroblast 275	1.2422723	0.06216
fetal muscle 184	1.2638234	0.06224
fetal lung 298	1.2730242	0.06226
fetal muscle 181	1.2782419	0.06286
fetal renal pelvis 224	1.2802554	0.06444
fetal fibroblast 276	1.2381566	0.06498
fetal fibroblast 279	1.2513160	0.06514
fetal muscle 159	1.2950762	0.06524
fetal fibroblast 280	1.2447237	0.06524
fetal thymus 262	1.4080459	0.06576
CD8 22	1.3789047	0.06594
fetal lung 120	1.2436206	0.06604
fetal kidney 82	1.3199616	0.06634
fetal renal pelvis 217	1.3012810	0.06648
fetal lung 135	1.2491902	0.06698
fetal stomach 245	1.3113232	0.06764
fetal renal cortex 204	1.2715553	0.06796
fetal renal pelvis 220	1.2930309	0.06866
Mobilized CD3 321	1.3883454	0.07072
fetal renal cortex 211	1.2996391	0.07210
fetal muscle 148	1.2727106	0.07284
fetal kidney 107	1.2760829	0.07322
fetal kidney 104	1.2854718	0.07330
stomach 284	1.2973436	0.07394
fetal lung 119	1.2205433	0.07514
fetal muscle 190	1.2804774	0.07596
fetal muscle 146	1.2550526	0.07600
fetal renal cortex 205	1.2754348	0.07622
ES cells 294	1.3020344	0.07660
fetal muscle 158	1.2501029	0.07670
ES cells 296	1.3046789	0.07690
fetal muscle 171	1.2505728	0.07708
fetal renal pelvis 233	1.2813712	0.07716
fetal large intestine 63	1.2814559	0.07724
fetal kidney 93	1.2855261	0.07758
fetal kidney 90	1.2761205	0.07808
fibroblast 329	1.2294817	0.07808
fibroblast 330	1.2325770	0.07944
fetal muscle 189	1.2557964	0.07950
fetal renal cortex 207	1.2778446	0.07954
fetal muscle 151	1.2708914	0.07998
fetal lung 112	1.2369237	0.08026
fetal muscle 145	1.2839274	0.08106
fetal lung 301	1.2391075	0.08108
keratinocyte 336	1.1918280	0.08112
fetal muscle 174	1.2569845	0.08276
fetal muscle 173	1.2598858	0.08282
fetal muscle 160	1.2571745	0.08324
fetal renal cortex 202	1.2585264	0.08350
fetal muscle 168	1.2660849	0.08446
fetal lung 130	1.2171497	0.08558
fetal renal cortex 208	1.2664121	0.08600
fetal muscle 179	1.2480921	0.08614
fetal fibroblast 269	1.2153924	0.08660
fetal muscle 172	1.2236055	0.08662
fetal kidney 81	1.2349579	0.08712
fetal renal cortex 209	1.2643651	0.08728
fetal renal pelvis 230	1.2800665	0.08958
fibroblast 332	1.2136421	0.08982
fetal muscle 187	1.2314865	0.09088
fetal muscle 163	1.2485047	0.09134
fetal renal pelvis 229	1.2511272	0.09138
fetal kidney 99	1.2556241	0.09144
fetal renal cortex 206	1.2435554	0.09322
fetal kidney 96	1.2391778	0.09434
fetal kidney 98	1.2555283	0.09444
fetal muscle 147	1.2371034	0.09446
fetal kidney 101	1.2496660	0.09498
fetal renal cortex 215	1.2374923	0.09562
fetal kidney 88	1.2378811	0.09604
fetal kidney 91	1.2368464	0.09622
fetal kidney 87	1.2432786	0.09638
fetal muscle 150	1.2466883	0.09648
fetal muscle 166	1.2297917	0.09962
fetal muscle 155	1.2463698	0.09980
fetal renal pelvis 227	1.2438023	0.10088
fetal kidney 89	1.2526358	0.10100
fetal thymus 259	1.3058337	0.10108
fetal skin 234	1.2409738	0.10146
fetal lung 123	1.2100148	0.10154
fetal kidney 105	1.2327214	0.10186
fetal muscle 162	1.2211257	0.10290
fetal kidney 95	1.2418614	0.10294
fetal kidney 84	1.2532654	0.10306
fetal kidney 97	1.2374159	0.10462
fetal stomach 251	1.2535082	0.10536
fetal renal pelvis 225	1.2450860	0.10630
fetal renal cortex 214	1.2357106	0.10734
fetal muscle 180	1.2325807	0.10856
fetal muscle 161	1.2253795	0.10930
fetal renal pelvis 232	1.2427663	0.10952
ES cells 295	1.2526836	0.10980
fetal muscle 192	1.2169937	0.10990
fetal muscle 164	1.2225516	0.11012
fetal kidney 100	1.2334320	0.11088
fibroblast 331	1.1957295	0.11236
fetal heart 47	1.2454208	0.11268
fetal lung 299	1.2100053	0.11302
fetal muscle 149	1.2288344	0.11330
fetal muscle 154	1.2333592	0.11418
fetal kidney 83	1.2563465	0.11480
fetal muscle 169	1.2191135	0.11522
fetal muscle 157	1.1995248	0.11528
fetal stomach 250	1.2576276	0.11538
fetal heart 50	1.2137353	0.11798
fetal renal pelvis 219	1.2661775	0.11908
fetal stomach 244	1.2423645	0.11976
fetal renal pelvis 223	1.2367936	0.11992
fetal renal pelvis 228	1.2374247	0.12016
fetal muscle 183	1.1999283	0.12110
fetal renal cortex 212	1.2110013	0.12144
fetal heart 49	1.2120078	0.12222
pancreas 327	1.2382449	0.12232
fetal ovary 195	1.2607266	0.12410
fetal heart 46	1.2121995	0.12488
fetal renal cortex 203	1.2161354	0.12604
fetal renal pelvis 218	1.2553893	0.12784
fetal muscle 182	1.2026813	0.12784
fetal kidney 102	1.2284937	0.12800
fetal muscle 165	1.1933266	0.12828
fetal small intestine 75	1.3343671	0.12898
fetal renal pelvis 222	1.2201687	0.12910
fetal heart 45	1.2047874	0.13006
fetal stomach 243	1.2129333	0.13068
fetal kidney 92	1.2072678	0.13120
fetal muscle 156	1.1958740	0.13128
fetal fibroblast 270	1.1800663	0.13142
fetal muscle 152	1.1962271	0.13168
ES derived NP 292	1.2211244	0.13170
fetal adrenal gland 26	1.1968179	0.13396
fetal muscle 175	1.1962864	0.13422
fetal kidney 94	1.2163365	0.13612
fetal stomach 248	1.2287482	0.13640
fetal muscle 176	1.1849446	0.13716
fetal muscle 177	1.2093120	0.13736
fetal heart 52	1.1766230	0.14274
fetal muscle 178	1.1971761	0.14304
fetal stomach 241	1.2250239	0.14422
fetal muscle 186	1.1853905	0.14514
fetal muscle 144	1.1981006	0.14638
heart 297	1.2056152	0.14858
fetal stomach 249	1.2178799	0.15272
fetal muscle 185	1.1934730	0.15348
fetal stomach 253	1.2177653	0.15424
CD4 15	1.2884491	0.15504
fetal stomach 240	1.2162557	0.15550
ES derived NP 291	1.2119372	0.15670
fetal adrenal gland 23	1.1707076	0.15694
fetal heart 48	1.1784098	0.16030
fetal kidney 86	1.2161741	0.16290
fetal thymus 263	1.1747167	0.16984
fetal stomach 246	1.1983681	0.17072
fetal renal pelvis 221	1.1693175	0.17208
fetal muscle 167	1.1557901	0.18086
fetal heart 51	1.1578227	0.18360
fetal renal cortex 210	1.1684721	0.18756
fetal muscle 153	1.1648184	0.19064
fetal adrenal gland 24	1.1691813	0.19340
fetal heart 43	1.1446052	0.19466
fetal muscle 170	1.1439625	0.19492
fetal stomach 252	1.1684425	0.19864
fetal heart 42	1.1462449	0.20224
fetal adrenal gland 27	1.1469322	0.20644
fetal testes 254	1.1475316	0.21014
fetal kidney 103	1.1463816	0.21284
fetal muscle 143	1.1315165	0.21328
fetal testes 255	1.1360925	0.21440
fetal spinal cord 236	1.1388614	0.21498
fetal brain 35	1.1035197	0.21856
fetal stomach 242	1.1749349	0.22198
melanocyte 337	1.1060279	0.22484
fetal stomach 247	1.1588547	0.23682
ovary 326	1.1314341	0.26606
fetal adrenal gland 28	1.0949332	0.27048
fetal spinal cord 235	1.0897114	0.28342
fetal brain 40	1.0781185	0.28738
fetal spinal cord 239	1.0841374	0.29946
fetal brain 32	1.0714771	0.30440
fetal adrenal gland 25	1.0894776	0.30678
fetal heart 44	1.0964415	0.30734
melanocyte 338	1.0700515	0.30882
fetal spinal cord 238	1.0765499	0.31668
fetal brain 34	1.0609105	0.32762
melanocyte 339	1.0500769	0.36118
fetal spinal cord 237	1.0523252	0.36268
fetal brain 39	1.0467223	0.36634
fetal brain 41	1.0358939	0.39306
fetal brain 31	1.0262804	0.40980
fetal brain 36	1.0243486	0.42700
fetal brain 29	1.0190386	0.43132
melanocyte 340	1.0162309	0.45226
fetal brain 38	1.0136697	0.45392
fetal brain 33	1.0011275	0.48736
fetal brain 37	0.9963711	0.50276
fetal brain 30	0.9921309	0.51712

Type 1 diabetes − autoantibody positive

DHS sample	fold enrichment	p value
Mobilized CD56 324	2.0609147	0.00008
CD3 11	1.7019581	0.00262
CD56 17	1.6375612	0.00268
CD8 20	1.7254195	0.00274
CD4 14	1.6909273	0.00316
CD56 18	1.7543267	0.00402
CD3 9	1.7874558	0.00416
fetal thymus 256	1.6155164	0.00504
CD3 10	1.6177801	0.00778
CD8 21	1.7190026	0.00812
fetal thymus 265	1.5816095	0.00938
Mobilized CD3 320	1.6750538	0.01006
CD19 5	1.6100310	0.01134
CD4 13	1.6551303	0.01354
fetal thymus 258	1.5506174	0.01394
fetal thymus 266	1.5383130	0.01582
fetal large intestine 61	1.6080740	0.01604
Mobilized CD4 322	1.6613460	0.01664
CD19 6	1.6091789	0.01670
CD19 7	1.6196973	0.01762
fetal thymus 264	1.4913955	0.02008
CD3 12	1.6076642	0.02356
Mobilized CD4 323	1.5552223	0.02452
CD14 4	1.4452939	0.02574
hematopoietic stem cell 314	1.3590585	0.02696
Mobilized CD3 321	1.5986119	0.02974
hematopoietic stem cell 310	1.3307831	0.03012
CD14 3	1.4468752	0.03264
hematopoietic stem cell 308	1.3562263	0.03334
CD8 19	1.5701221	0.03426
fetal thymus 260	1.4379142	0.03762
CD8 22	1.4951269	0.04234
hematopoietic stem cell 312	1.3237670	0.04292
hematopoietic stem cell 313	1.3279625	0.04466
hematopoietic stem cell 309	1.3301735	0.05146
hematopoietic stem cell 305	1.3397734	0.05176
fetal thymus 261	1.3841033	0.05828
hematopoietic stem cell 318	1.3327678	0.05834
CD4 16	1.4699722	0.05946
hematopoietic stem cell 315	1.3158344	0.05984
fetal thymus 257	1.4063511	0.06290
hematopoietic stem cell 316	1.3337927	0.06430
fetal thymus 259	1.3857000	0.06608
Mobilized CD8 325	1.4108867	0.07402
hematopoietic stem cell 306	1.2898110	0.07494
hematopoietic stem cell 317	1.2475641	0.08004
hematopoietic stem cell 307	1.2795238	0.08006
hematopoietic stem cell 311	1.2727768	0.08442
CD4 15	1.4202417	0.10532
fetal thymus 262	1.3135615	0.13394
fetal muscle 155	1.2030219	0.15346
fetal small intestine 75	1.3370732	0.15408
fetal lung 111	1.1713071	0.15666
fetal lung 117	1.1644422	0.16374
fetal muscle 149	1.1944253	0.16508
fetal lung 140	1.1531736	0.17976
fetal lung 299	1.1667916	0.18000
hematopoietic stem cell 319	1.1868431	0.18536
fetal lung 298	1.1619047	0.19034
fetal lung 141	1.1351455	0.19860
fetal muscle 180	1.1604309	0.20244
fetal muscle 168	1.1558270	0.20994
fetal lung 110	1.1399768	0.21320
fetal muscle 154	1.1522715	0.22066
fetal lung 135	1.1295192	0.22128
fetal muscle 191	1.1433911	0.22192
fetal muscle 159	1.1483849	0.22620
fetal lung 136	1.1271409	0.23028
placenta 201	1.1474517	0.23302
muscle 341	1.1527619	0.23668
fetal lung 133	1.1150720	0.23716
fetal lung 134	1.1245494	0.23766
fetal lung 121	1.1115431	0.23772
fetal muscle 145	1.1449586	0.24028
fetal lung 126	1.1112560	0.24064
fetal lung 108	1.1223557	0.24864
fetal muscle 148	1.1299550	0.24904
fetal lung 124	1.1125941	0.25280
fibroblast 330	1.1129722	0.25286
fetal muscle 169	1.1233007	0.25398
fetal lung 114	1.1034007	0.25442
keratinocyte 335	1.0971809	0.25672
fetal muscle 193	1.1245682	0.25712
fetal lung 119	1.1027030	0.25882
fetal renal cortex 207	1.1259368	0.26000
fetal lung 116	1.0959870	0.26268
fetal lung 115	1.1028417	0.26290
fibroblast 329	1.1077771	0.26298
fetal muscle 152	1.1108736	0.26412
fetal lung 118	1.0966751	0.26526
fetal muscle 192	1.1097985	0.26746
fetal lung 131	1.0890101	0.27368
fetal lung 120	1.1002251	0.27452
fetal lung 300	1.1034846	0.27470
fetal lung 128	1.0880242	0.27678
fetal muscle 164	1.1088446	0.27914
fetal muscle 156	1.1043632	0.27984
fetal lung 139	1.1007386	0.27990
fetal muscle 177	1.1087805	0.28302
fetal muscle 160	1.1062020	0.28426
keratinocyte 336	1.0812142	0.28660
fetal muscle 153	1.1084827	0.28834
fetal lung 137	1.0880425	0.28998
fetal thymus 263	1.1019645	0.29260
fetal lung 132	1.0877627	0.29882
fetal muscle 174	1.0957150	0.29888
placenta 199	1.0935363	0.30096
fetal lung 301	1.0922711	0.30120
fetal muscle 194	1.0838467	0.30236
fetal muscle 151	1.0971445	0.30356
stomach 283	1.1063246	0.30382
fetal muscle 150	1.0955780	0.30414
fetal fibroblast 272	1.0797903	0.30542
fetal lung 113	1.0778989	0.30706
fetal muscle 172	1.0810027	0.30870
fetal muscle 143	1.0817361	0.31256
fetal muscle 182	1.0854251	0.31314
heart 297	1.0918368	0.31822
fetal skin 234	1.0883930	0.31992
fetal muscle 173	1.0821480	0.32050
fetal lung 138	1.0759841	0.32064
fetal lung 122	1.0783721	0.32118
fetal muscle 166	1.0781706	0.32388
fetal muscle 158	1.0769979	0.32472
pancreas 327	1.0903632	0.32546
fetal muscle 167	1.0778727	0.32662
fetal muscle 178	1.0796508	0.32822
fetal renal pelvis 219	1.0977659	0.32902
fetal fibroblast 273	1.0661758	0.33644
fibroblast 331	1.0666132	0.33704
fetal muscle 176	1.0709840	0.33732
fetal muscle 188	1.0713929	0.33776
keratinocyte 334	1.0596345	0.33942
fetal lung 127	1.0662674	0.34092
pancreas 328	1.0778350	0.34542
fetal muscle 185	1.0726045	0.34586
ES derived MSC 289	1.0682438	0.34870
fetal lung 125	1.0624920	0.34938
placenta 196	1.0696958	0.35122
fetal fibroblast 281	1.0581341	0.35188
fetal renal pelvis 220	1.0692741	0.35396
fibroblast 332	1.0581108	0.35790
breast 1	1.0536333	0.36100
fetal fibroblast 274	1.0550578	0.36296
stomach 284	1.0679640	0.36304
fetal muscle 189	1.0597626	0.36482
placenta 197	1.0608623	0.36602
fetal kidney 85	1.0521381	0.36676
fetal lung 129	1.0529650	0.36760
fetal fibroblast 282	1.0512488	0.37008
fetal muscle 187	1.0544096	0.37298
placenta 200	1.0493195	0.37760
fetal kidney 83	1.0578975	0.37884
fetal lung 142	1.0450054	0.37902
keratinocyte 333	1.0417476	0.38034
fetal muscle 181	1.0516698	0.38080
fetal fibroblast 267	1.0490366	0.38184
fetal muscle 165	1.0495713	0.38244
fetal muscle 183	1.0477383	0.38358
ovary 326	1.0587071	0.38546
fetal kidney 100	1.0495859	0.38566
fetal muscle 163	1.0496800	0.38590
fetal lung 109	1.0410350	0.38598
fetal muscle 175	1.0484857	0.38684
ES derived MSC 290	1.0477378	0.38798
fetal muscle 186	1.0459677	0.38854
fetal lung 130	1.0433297	0.38898
fetal muscle 157	1.0442283	0.39238
fetal small intestine 69	1.0471136	0.39286
fetal ovary 195	1.0459429	0.39352
fetal lung 123	1.0421717	0.39420
fetal kidney 89	1.0449211	0.39546
fetal muscle 147	1.0426164	0.39976
fetal kidney 106	1.0381568	0.40682
fetal fibroblast 271	1.0358175	0.40854
fetal stomach 251	1.0358817	0.41374
fetal kidney 102	1.0317504	0.41700
fetal fibroblast 280	1.0301596	0.42114
melanocyte 340	1.0285103	0.42114
fetal small intestine 77	1.0325809	0.42178
fetal muscle 171	1.0295989	0.42264
fetal kidney 91	1.0269389	0.42324
fetal fibroblast 277	1.0300524	0.42332
fetal small intestine 78	1.0298052	0.42372
fetal kidney 87	1.0287669	0.42398
breast 2	1.0266803	0.42460
fetal muscle 179	1.0294378	0.42600
fetal muscle 190	1.0284617	0.42636
fetal large intestine 64	1.0301006	0.42732
fetal heart 42	1.0296050	0.42734
fetal fibroblast 268	1.0257687	0.42974
fetal large intestine 53	1.0251469	0.43104
melanocyte 339	1.0232940	0.43178
fetal renal cortex 214	1.0241598	0.43284
fetal stomach 252	1.0221477	0.43322
fetal renal pelvis 228	1.0227445	0.43626
fetal kidney 88	1.0203848	0.43976
fetal kidney 84	1.0189199	0.44204
fetal kidney 82	1.0169695	0.44328
fetal renal pelvis 230	1.0167154	0.44444
fetal kidney 86	1.0163200	0.44490
fetal large intestine 66	1.0169533	0.44590
melanocyte 338	1.0185143	0.44592
fetal stomach 244	1.0208805	0.44598
fetal stomach 243	1.0192740	0.44696
fetal fibroblast 278	1.0181239	0.44810
fetal large intestine 65	1.0159703	0.45062
fetal heart 52	1.0154750	0.45286
fetal stomach 245	1.0131723	0.45348
fetal muscle 184	1.0157717	0.45538
fetal small intestine 76	1.0145084	0.45538
fetal renal pelvis 227	1.0112647	0.45654
fetal renal cortex 213	1.0123802	0.45706
fetal adrenal gland 26	1.0117657	0.45758
fetal muscle 144	1.0116926	0.45848
fetal large intestine 60	1.0118851	0.45898
fetal renal pelvis 221	1.0108217	0.46012
fetal renal pelvis 233	1.0097843	0.46032
CD20 8	1.0144286	0.46072
fetal fibroblast 279	1.0123021	0.46072
fetal renal pelvis 218	1.0033334	0.46148
fetal renal cortex 203	1.0097537	0.46156
fetal muscle 170	1.0120756	0.46162
fetal renal pelvis 226	1.0092092	0.46284
fetal kidney 104	1.0053145	0.46618
fetal large intestine 54	1.0058377	0.46728
fetal stomach 242	0.9966209	0.47046
fetal stomach 248	1.0044521	0.47088
fetal stomach 253	1.0022265	0.47260
fetal muscle 146	1.0067679	0.47360
fetal renal pelvis 217	1.0021205	0.47400
fetal fibroblast 276	1.0070429	0.47448
fetal kidney 103	0.9980785	0.47554
fetal small intestine 79	1.0020467	0.47754
fetal stomach 250	0.9944068	0.48352
small intestine 342	1.0000319	0.48500
fetal adrenal gland 25	0.9999531	0.48626
fetal fibroblast 269	1.0005548	0.48822
fetal large intestine 57	0.9988896	0.49228
fetal renal pelvis 216	0.9917776	0.49264
placenta 198	0.9983307	0.49382
fetal renal cortex 211	0.9876780	0.49476
fetal kidney 92	0.9915724	0.49584
melanocyte 337	1.0002542	0.49652
fetal kidney 94	0.9872284	0.49658
fetal renal cortex 209	0.9900778	0.49686
fetal adrenal gland 23	0.9928043	0.49954
fetal fibroblast 275	0.9967026	0.50082
fetal renal pelvis 232	0.9851624	0.50276
fetal small intestine 80	0.9913385	0.50324
fetal large intestine 67	0.9914524	0.50478
fetal kidney 99	0.9826818	0.50920
fetal renal cortex 206	0.9852761	0.50992
fetal kidney 81	0.9894016	0.51198
fetal large intestine 56	0.9845394	0.51306
fetal small intestine 70	0.9897655	0.51432
fetal large intestine 62	0.9786813	0.51778
fetal kidney 107	0.9793525	0.51908
ES cells 296	0.9767317	0.52060
fetal renal cortex 208	0.9764621	0.52110
fetal large intestine 59	0.9758434	0.52658
fetal renal pelvis 225	0.9701010	0.52826
fetal renal cortex 205	0.9764150	0.52862
fetal heart 50	0.9799743	0.52880
fetal testes 255	0.9778128	0.53296
fetal renal cortex 204	0.9763532	0.53314
fetal muscle 161	0.9759614	0.53496
ES derived NP 291	0.9668237	0.53700
fetal renal pelvis 231	0.9701754	0.54052
fetal renal cortex 202	0.9684632	0.54218
fetal small intestine 68	0.9720202	0.54230
fetal adrenal gland 27	0.9706933	0.54278
fetal fibroblast 270	0.9752297	0.54320
fetal large intestine 55	0.9716289	0.54330
fetal stomach 249	0.9566187	0.54354
fetal kidney 95	0.9639782	0.54376
fetal large intestine 58	0.9746817	0.54384
ES derived NP 292	0.9669058	0.54470
fetal muscle 162	0.9740156	0.54516
fetal kidney 98	0.9630241	0.54712
fetal stomach 240	0.9524403	0.55176
fetal stomach 247	0.9467428	0.55232
fetal stomach 241	0.9482377	0.56120
fetal adrenal gland 28	0.9674695	0.56316
ES derived trophoblast 288	0.9646595	0.56462
fetal small intestine 73	0.9565340	0.56566
fetal kidney 93	0.9528198	0.56664
fetal renal pelvis 224	0.9574386	0.56740
fetal kidney 90	0.9501209	0.57196
fetal testes 254	0.9552368	0.57240
fetal stomach 246	0.9426393	0.57304
fetal heart 46	0.9542160	0.57792
ES cells 294	0.9453143	0.57804
fetal adrenal gland 24	0.9457559	0.57890
fetal renal pelvis 229	0.9476746	0.58322
fetal renal pelvis 223	0.9370872	0.59084
fetal renal cortex 210	0.9378786	0.59360
fetal small intestine 72	0.9435280	0.59784
fetal heart 47	0.9362929	0.60138
fetal heart 48	0.9437377	0.60258
fetal lung 112	0.9500541	0.60364
fetal kidney 97	0.9321229	0.60614
fetal renal pelvis 222	0.9276966	0.61072
fetal kidney 96	0.9307706	0.61812
fetal renal cortex 215	0.9299761	0.62106
fetal small intestine 74	0.9301037	0.62230
fetal kidney 105	0.9225548	0.63488
fetal kidney 101	0.9173217	0.64364
fetal renal cortex 212	0.9175410	0.64394
fetal heart 44	0.9075777	0.64782
fetal heart 43	0.9248077	0.64878
fetal heart 51	0.9199229	0.65306
fetal small intestine 71	0.9043414	0.66278
fetal heart 45	0.9088341	0.67918
fetal brain 37	0.9266621	0.68030
fetal large intestine 63	0.8881604	0.68896
ES cells 295	0.8835875	0.68916
fetal brain 34	0.9255130	0.69784
iPS 302	0.8933896	0.70666
fetal heart 49	0.8908156	0.70862
ES cells 293	0.8743626	0.71524
fetal brain 30	0.9229370	0.72788
fetal brain 35	0.9153253	0.73432
ES derived mesoderm 285	0.8945091	0.73922
iPS 304	0.8812681	0.74424
ES derived trophoblast 287	0.8704575	0.74450
fetal brain 29	0.9134323	0.74454
iPS 303	0.8723353	0.75354
fetal spinal cord 237	0.8779992	0.75760
fetal brain 40	0.8748793	0.80388
fetal brain 39	0.8703776	0.81398
fetal brain 41	0.8483939	0.83630
ES derived mesoderm 286	0.8465473	0.84232
fetal brain 31	0.8722809	0.84382
fetal brain 36	0.8355716	0.86452
fetal spinal cord 236	0.7971319	0.86952
fetal spinal cord 238	0.8034703	0.87092
fetal brain 32	0.8110441	0.89718
fetal spinal cord 235	0.7782361	0.91120
fetal spinal cord 239	0.7613559	0.92240
fetal brain 38	0.7702520	0.93256
fetal brain 33	0.7497883	0.94456

Multiple sclerosis

DHS sample	fold enrichment	p value
Mobilized CD56 324	2.0422009	0.00002
Mobilized CD3 320	1.9179164	0.00002
CD4 13	1.8597620	0.00002
CD3 10	1.8065641	0.00002
CD4 14	1.7938580	0.00002
CD19 5	1.7834192	0.00002
CD8 20	1.7773380	0.00002
CD3 9	1.7763886	0.00002
CD14 3	1.7683638	0.00002
CD19 6	1.7622821	0.00002
Mobilized CD4 323	1.7472527	0.00002
CD8 21	1.7452903	0.00002
CD14 4	1.7416202	0.00002
CD3 11	1.7382809	0.00002
CD56 17	1.7047331	0.00002
fetal thymus 256	1.6635034	0.00002
hematopoietic stem cell 310	1.5020850	0.00002
CD19 7	1.7723023	0.00004
CD56 18	1.6728612	0.00004
fetal thymus 258	1.6106133	0.00004
hematopoietic stem cell 309	1.4849781	0.00004
hematopoietic stem cell 314	1.4825687	0.00004
hematopoietic stem cell 312	1.4541420	0.00004
Mobilized CD4 322	1.7848928	0.00006
fetal thymus 266	1.5535891	0.00006
CD3 12	1.7506074	0.00008
Mobilized CD3 321	1.7466548	0.00008
CD8 22	1.6862860	0.00008
fetal thymus 265	1.5613598	0.00010
hematopoietic stem cell 315	1.4467339	0.00010
fetal large intestine 61	1.6431602	0.00012
hematopoietic stem cell 308	1.4357001	0.00012
hematopoietic stem cell 317	1.3772678	0.00018
fetal thymus 260	1.5424601	0.00020
CD4 16	1.6660444	0.00024
fetal thymus 264	1.5041398	0.00028
hematopoietic stem cell 313	1.4034093	0.00030
hematopoietic stem cell 318	1.4317498	0.00052
Mobilized CD8 325	1.5727603	0.00056
CD8 19	1.6263429	0.00060
hematopoietic stem cell 306	1.3695014	0.00066
fetal thymus 259	1.5149528	0.00074
hematopoietic stem cell 305	1.3764047	0.00108
hematopoietic stem cell 311	1.3595957	0.00114
hematopoietic stem cell 307	1.3540486	0.00142
hematopoietic stem cell 316	1.3818309	0.00158
fetal thymus 261	1.4362793	0.00198
fetal lung 141	1.2845172	0.00202
fetal thymus 257	1.4600118	0.00210
CD4 15	1.5866754	0.00254
fetal lung 128	1.2538070	0.00326
fetal small intestine 75	1.5860283	0.00384
fetal lung 140	1.2667274	0.00424
fetal thymus 262	1.4907551	0.00450
fetal lung 116	1.2434666	0.00488
fetal lung 126	1.2517015	0.00506
fetal lung 111	1.2666085	0.00540
fetal lung 110	1.2796956	0.00566
fetal lung 120	1.2623924	0.00616
hematopoietic stem cell 319	1.3221783	0.00626
keratinocyte 335	1.2216924	0.00630
fetal lung 117	1.2461020	0.00634
keratinocyte 336	1.2148418	0.00740
fetal lung 119	1.2394357	0.00764
fetal lung 115	1.2357272	0.00936
fetal lung 131	1.2204086	0.00952
fetal lung 129	1.2452111	0.00982
stomach 283	1.3238748	0.00992
fetal lung 121	1.2263003	0.01022
ES derived MSC 290	1.2606258	0.01080
fetal lung 133	1.2252486	0.01118
fetal lung 134	1.2442530	0.01210
fetal lung 108	1.2521934	0.01226
fetal lung 137	1.2293235	0.01234
fetal lung 118	1.2062470	0.01374
fetal lung 114	1.2056243	0.01434
ES derived MSC 289	1.2552171	0.01440
fetal lung 127	1.2272277	0.01524
keratinocyte 333	1.1889140	0.01630
fetal lung 113	1.2094600	0.01634
fetal lung 138	1.2235758	0.01664
fetal lung 136	1.2234813	0.01718
fetal lung 125	1.2278904	0.01720
fetal lung 135	1.2138242	0.02146
fetal large intestine 55	1.2387615	0.02166
fetal adrenal gland 26	1.2355257	0.02402
fetal fibroblast 272	1.1886556	0.02438
fetal lung 124	1.2027102	0.02598
breast 1	1.1838692	0.02624
fetal lung 142	1.1851575	0.02686
fetal stomach 245	1.2568850	0.02754
fetal lung 122	1.2110106	0.02806
fetal lung 139	1.2069100	0.02928
fetal large intestine 57	1.2163742	0.02932
fetal fibroblast 277	1.1885851	0.02986
fetal fibroblast 273	1.1799152	0.03056
fetal stomach 244	1.2490231	0.03060
fetal muscle 194	1.1886203	0.03110
fetal fibroblast 279	1.1797636	0.03210
fetal lung 132	1.1904771	0.03224
stomach 284	1.2360221	0.03470
fetal fibroblast 274	1.1759794	0.03510
fetal large intestine 64	1.2163676	0.03534
breast 2	1.1725972	0.03542
fetal stomach 243	1.2200093	0.03628
fetal fibroblast 282	1.1643281	0.03632
fetal small intestine 78	1.1971610	0.03668
keratinocyte 334	1.1584504	0.03738
placenta 200	1.1847053	0.03740
fetal large intestine 58	1.1986512	0.03784
small intestine 342	1.2161171	0.03816
ovary 326	1.2591627	0.03900
fetal lung 300	1.1833191	0.03932
fetal small intestine 77	1.2224432	0.03946
fetal fibroblast 269	1.1673540	0.03972
fetal small intestine 70	1.1878862	0.04036
fetal small intestine 71	1.2128463	0.04086
fetal large intestine 60	1.2151377	0.04170
fetal lung 130	1.1756331	0.04288
fetal large intestine 67	1.2145455	0.04292
fetal small intestine 68	1.1987118	0.04396
fetal fibroblast 280	1.1647869	0.04416
placenta 201	1.2115479	0.04428
fetal lung 123	1.1771466	0.04438
fibroblast 330	1.1657811	0.04652
fetal small intestine 80	1.1983898	0.04722
fetal large intestine 53	1.2300289	0.05042
fetal lung 298	1.1783592	0.05056
fibroblast 332	1.1559038	0.05086
fetal fibroblast 275	1.1504187	0.05090
fetal large intestine 56	1.1981192	0.05166
fibroblast 331	1.1590603	0.05196
fetal fibroblast 268	1.1511268	0.05230
fetal small intestine 74	1.1918986	0.05418
melanocyte 337	1.1392872	0.05644
fetal muscle 149	1.1854627	0.05750
fetal lung 301	1.1661327	0.05878
fetal small intestine 76	1.1997219	0.05952
fetal large intestine 66	1.1988872	0.05992
fetal lung 109	1.1404815	0.06092
fetal fibroblast 276	1.1422927	0.06126
fetal fibroblast 281	1.1456739	0.06178
CD20 8	1.1760655	0.06206
fetal fibroblast 270	1.1517232	0.06252
fetal lung 299	1.1641852	0.06272
fetal fibroblast 271	1.1459715	0.06344
fetal muscle 156	1.1679004	0.06348
fetal stomach 251	1.1920557	0.06432
fetal skin 234	1.1760660	0.06494
melanocyte 339	1.1411730	0.06550
fetal adrenal gland 23	1.1618612	0.06698
fetal small intestine 72	1.1797685	0.06752
fetal large intestine 65	1.1802593	0.07180
fetal muscle 146	1.1602367	0.07272
melanocyte 338	1.1289984	0.07300
fetal muscle 169	1.1648017	0.07344
fetal muscle 167	1.1567876	0.07370
fetal muscle 159	1.1694152	0.07440
fibroblast 329	1.1391222	0.07698
fetal large intestine 62	1.1837739	0.07736
fetal muscle 177	1.1705970	0.07776
fetal small intestine 79	1.1846875	0.07828
fetal small intestine 73	1.1717836	0.07888
placenta 198	1.1512416	0.07888
heart 297	1.1798160	0.07910
fetal stomach 241	1.1915731	0.07958
fetal stomach 249	1.1944789	0.07978
fetal fibroblast 278	1.1371987	0.08020
fetal muscle 151	1.1683837	0.08030
fetal stomach 250	1.1898193	0.08116
fetal muscle 152	1.1492327	0.08174
fetal renal cortex 207	1.1587401	0.08516
fetal adrenal gland 25	1.1675377	0.08534
fetal stomach 253	1.1853364	0.08548
fetal adrenal gland 27	1.1589397	0.08582
fetal muscle 148	1.1572428	0.08594
fetal stomach 247	1.2050673	0.08654
fetal kidney 106	1.1539910	0.08670
fetal muscle 164	1.1536090	0.08688
fetal muscle 174	1.1519855	0.08698
fetal small intestine 69	1.1733527	0.08946
pancreas 327	1.1799789	0.08974
fetal stomach 252	1.1698933	0.09104
fetal large intestine 59	1.1697952	0.09264
fetal large intestine 54	1.1617553	0.09310
fetal muscle 154	1.1564718	0.09314
fetal muscle 192	1.1410403	0.09380
fetal testes 254	1.1520182	0.09404
fetal muscle 153	1.1548422	0.09532
fetal muscle 160	1.1509058	0.09604
fetal kidney 100	1.1504741	0.09674
fetal muscle 155	1.1547054	0.09702
fetal muscle 191	1.1451752	0.09762
fetal adrenal gland 28	1.1315130	0.09808
fetal kidney 107	1.1477862	0.09816
fetal muscle 166	1.1411299	0.09898
fetal renal cortex 203	1.1484472	0.09922
fetal renal pelvis 228	1.1598808	0.09966
fetal renal pelvis 232	1.1569089	0.10034
fetal renal pelvis 220	1.1503945	0.10276
fetal stomach 248	1.1620560	0.10314
fetal fibroblast 267	1.1241467	0.10408
melanocyte 340	1.1217911	0.10528
placenta 199	1.1371108	0.10734
fetal muscle 168	1.1400386	0.10780
fetal kidney 84	1.1493918	0.10870
fetal stomach 246	1.1620113	0.10990
fetal large intestine 63	1.1543191	0.11066
fetal muscle 193	1.1389570	0.11078
fetal muscle 157	1.1271886	0.11124
fetal renal pelvis 217	1.1455522	0.11260
fetal renal pelvis 231	1.1294227	0.11344
fetal renal cortex 206	1.1347989	0.11388
ES derived trophoblast 288	1.1254254	0.11416
fetal muscle 175	1.1298631	0.11474
fetal renal cortex 214	1.1364637	0.11534
fetal muscle 145	1.1455622	0.11702
fetal renal cortex 213	1.1285771	0.11748
fetal stomach 240	1.1625554	0.11758
fetal heart 48	1.1338783	0.11814
fetal kidney 102	1.1437275	0.11842
fetal muscle 171	1.1243888	0.11970
fetal muscle 179	1.1298391	0.11990
fetal muscle 158	1.1241891	0.11994
fetal kidney 104	1.1373027	0.12022
pancreas 328	1.1493089	0.12184
fetal kidney 91	1.1244784	0.12318
fetal heart 46	1.1344415	0.12472
fetal muscle 165	1.1202004	0.12542
fetal heart 42	1.1300604	0.12592
fetal renal pelvis 223	1.1382240	0.12634
fetal muscle 189	1.1241486	0.12714
ES derived mesoderm 286	1.0993129	0.12834
fetal renal cortex 211	1.1389702	0.12858
fetal muscle 185	1.1312534	0.12870
fetal muscle 183	1.1185141	0.12898
fetal muscle 180	1.1312603	0.12914
fetal renal pelvis 219	1.1597445	0.12968
fetal muscle 178	1.1289413	0.12992
fetal muscle 176	1.1204158	0.12994
fetal renal pelvis 230	1.1382159	0.13176
fetal muscle 190	1.1319433	0.13216
fetal kidney 87	1.1261126	0.13280
fetal muscle 150	1.1306293	0.13306
fetal muscle 143	1.1136691	0.13330
fetal heart 52	1.1161271	0.13368
fetal lung 112	1.1102124	0.13536
ES derived trophoblast 287	1.1222510	0.13664
placenta 197	1.1272385	0.13736
fetal renal pelvis 218	1.1529982	0.13878
fetal renal pelvis 233	1.1251665	0.13956
fetal muscle 188	1.1166008	0.13994
fetal muscle 163	1.1208409	0.14008
fetal adrenal gland 24	1.1373505	0.14068
fetal kidney 94	1.1298346	0.14102
fetal muscle 181	1.1177110	0.14114
ES derived mesoderm 285	1.1014242	0.14272
fetal renal pelvis 216	1.1243771	0.14406
fetal renal pelvis 226	1.1177732	0.14470
fetal muscle 147	1.1179157	0.14508
fetal kidney 98	1.1218521	0.14538
placenta 196	1.1226556	0.14726
fetal heart 51	1.1158493	0.14764
fetal renal pelvis 225	1.1237719	0.14790
fetal muscle 182	1.1104292	0.15248
fetal muscle 187	1.1073632	0.15354
fetal muscle 144	1.1182384	0.15424
fetal muscle 162	1.1063933	0.15718
muscle 341	1.1331646	0.15746
fetal kidney 85	1.1012772	0.15956
fetal renal pelvis 224	1.1070258	0.16060
fetal renal cortex 204	1.1040854	0.16148
fetal muscle 184	1.1009204	0.16372
fetal kidney 93	1.1170545	0.16552
fetal kidney 82	1.1201928	0.16616
fetal renal pelvis 222	1.1140837	0.16626
fetal kidney 105	1.1039890	0.16638
fetal renal pelvis 227	1.1077070	0.16742
fetal renal pelvis 229	1.1055731	0.16972
fetal kidney 88	1.1045009	0.17076
fetal renal cortex 215	1.1017737	0.17134
fetal muscle 161	1.1037924	0.17162
fetal kidney 89	1.1073723	0.17264
fetal renal cortex 205	1.1054261	0.17358
fetal kidney 96	1.1020182	0.17500
fetal kidney 99	1.1030761	0.17752
fetal muscle 172	1.0892887	0.17780
ES derived NP 291	1.1196204	0.17984
fetal kidney 103	1.1033800	0.18320
fetal kidney 95	1.1029298	0.18356
fetal renal cortex 209	1.1030581	0.18530
fetal kidney 81	1.0933991	0.18584
fetal heart 43	1.0926802	0.19008
fetal kidney 83	1.1109356	0.19260
fetal renal cortex 202	1.0939870	0.19344
fetal heart 50	1.0964212	0.19382
fetal kidney 90	1.0972923	0.19788
fetal stomach 242	1.1262120	0.19850
fetal renal cortex 208	1.0974461	0.19964
ES cells 296	1.1085396	0.20116
fetal muscle 186	1.0888293	0.20348
fetal testes 255	1.0867767	0.20422
fetal muscle 173	1.0905369	0.20470
fetal kidney 92	1.0875934	0.20580
fetal kidney 86	1.1093457	0.20664
ES cells 294	1.1042037	0.20984
fetal renal pelvis 221	1.0869176	0.20996
fetal kidney 97	1.0896373	0.21094
fetal heart 49	1.0865458	0.21586
fetal renal cortex 210	1.0921198	0.21746
fetal renal cortex 212	1.0813730	0.22280
fetal heart 44	1.0993462	0.22406
fetal ovary 195	1.1008689	0.22520
fetal heart 45	1.0814069	0.23178
iPS 303	1.0724924	0.24016
iPS 302	1.0715428	0.24632
fetal kidney 101	1.0709543	0.25554
fetal heart 47	1.0753738	0.26406
ES cells 293	1.0688511	0.27716
fetal thymus 263	1.0629933	0.28320
ES derived NP 292	1.0661182	0.28798
ES cells 295	1.0638332	0.29902
iPS 304	1.0501201	0.30436
fetal spinal cord 236	1.0538578	0.30522
fetal muscle 170	1.0494288	0.31194
fetal spinal cord 235	1.0425033	0.33690
fetal spinal cord 237	1.0306965	0.37390
fetal brain 35	1.0247106	0.37580
fetal brain 34	1.0244994	0.38034
fetal spinal cord 239	1.0232571	0.40376
fetal spinal cord 238	1.0045695	0.47172
fetal brain 41	1.0022597	0.48338
fetal brain 39	0.9970142	0.50826
fetal brain 32	0.9855260	0.55914
fetal brain 40	0.9847964	0.56082
fetal brain 37	0.9824539	0.56712
fetal brain 36	0.9806540	0.57844
fetal brain 38	0.9751267	0.59290
fetal brain 33	0.9746219	0.59788
fetal brain 31	0.9667832	0.66588
fetal brain 29	0.9625362	0.68438
fetal brain 30	0.9575685	0.71334

Liver enzymes in plasma

DHS sample	fold enrichment	p value
small intestine 342	1.5038592	0.00010
fetal small intestine 80	1.4801750	0.00010
fetal small intestine 74	1.4713041	0.00012
fetal small intestine 78	1.4264071	0.00016
fetal large intestine 64	1.4379783	0.00022
fetal large intestine 57	1.4168852	0.00022
fetal large intestine 67	1.4497933	0.00028
fetal large intestine 60	1.4656672	0.00030
fetal large intestine 58	1.4145690	0.00032
fetal small intestine 70	1.3778587	0.00042
fetal small intestine 68	1.4023714	0.00052
fetal large intestine 55	1.4027663	0.00062
CD20 8	1.4012786	0.00080
fetal large intestine 56	1.3975529	0.00140
fetal small intestine 73	1.3978549	0.00180
fetal large intestine 62	1.3983784	0.00218
fetal large intestine 54	1.3776635	0.00266
breast 2	1.2628234	0.00344
fetal small intestine 72	1.3492359	0.00364
fetal small intestine 79	1.3754539	0.00398
fetal small intestine 77	1.3508041	0.00404
fetal large intestine 63	1.3441417	0.00504
fetal small intestine 69	1.3645581	0.00516
breast 1	1.2371584	0.00686
fetal small intestine 76	1.3392994	0.00728
fetal large intestine 65	1.3197077	0.00774
fetal adrenal gland 28	1.2465162	0.00846
fetal large intestine 59	1.3174821	0.00928
stomach 284	1.3174237	0.01134
fetal large intestine 66	1.3106357	0.01148
fetal small intestine 71	1.2979380	0.01186
fetal adrenal gland 27	1.2606132	0.01376
keratinocyte 334	1.1901339	0.01456
fetal adrenal gland 25	1.2693672	0.01548
fetal heart 45	1.2593216	0.01680
fetal lung 300	1.2219786	0.01748
keratinocyte 333	1.1810260	0.01848
fetal adrenal gland 23	1.2200879	0.02056
fetal skin 234	1.2431610	0.02060
fetal heart 47	1.2705211	0.02084
fetal adrenal gland 26	1.2404885	0.02112
fetal adrenal gland 24	1.2654741	0.02270
pancreas 328	1.2693500	0.02304
ES derived mesoderm 285	1.1978803	0.02306
stomach 283	1.2663197	0.02536
fetal lung 299	1.2174669	0.02548
fetal lung 301	1.2068226	0.02604
keratinocyte 336	1.1679347	0.02758
fetal large intestine 53	1.2691373	0.02792
fetal heart 46	1.2311328	0.03120
fibroblast 331	1.1836284	0.03190
pancreas 327	1.2596296	0.03224
muscle 341	1.2552710	0.03224
fetal heart 52	1.2024719	0.03336
fetal heart 43	1.2106787	0.03338
fibroblast 332	1.1795850	0.03402
fetal heart 48	1.2159766	0.03408
fetal heart 42	1.2129717	0.03572
fetal heart 51	1.2095996	0.03722
iPS 304	1.1983568	0.03766
fetal heart 50	1.1961871	0.04974
fetal heart 49	1.1922969	0.05288
fetal lung 298	1.1761899	0.05476
ES derived MSC 290	1.1800158	0.05492
fibroblast 330	1.1615755	0.05630
fetal testes 254	1.1899894	0.05826
fibroblast 329	1.1576094	0.05942
fetal brain 32	1.1417004	0.06188
keratinocyte 335	1.1346630	0.06212
ES derived mesoderm 286	1.1382104	0.06222
iPS 303	1.1738791	0.06390
placenta 200	1.1562723	0.06592
fetal fibroblast 269	1.1437633	0.06794
ES cells 294	1.2056302	0.06826
fetal lung 134	1.1595911	0.06872
ovary 326	1.2177792	0.07182
placenta 198	1.1542645	0.07630
fetal fibroblast 270	1.1398312	0.07690
fetal fibroblast 278	1.1401640	0.07724
fetal renal pelvis 228	1.1844303	0.07810
placenta 197	1.1703298	0.07840
fetal renal pelvis 216	1.1739437	0.07892
fetal stomach 242	1.2260165	0.07904
ES derived MSC 289	1.1588109	0.07968
fetal lung 109	1.1275703	0.08068
fetal stomach 244	1.1798817	0.08126
fetal stomach 252	1.1844603	0.08172
fetal renal cortex 207	1.1645042	0.08196
fetal heart 44	1.1934361	0.08322
fetal stomach 240	1.1936303	0.08514
fetal lung 137	1.1380273	0.08536
fetal kidney 87	1.1586178	0.08540
fetal fibroblast 280	1.1318159	0.08572
fetal fibroblast 281	1.1287677	0.08800
fetal fibroblast 274	1.1305322	0.08820
fetal kidney 103	1.1620208	0.09008
fetal lung 125	1.1431327	0.09104
fetal fibroblast 282	1.1221903	0.09322
melanocyte 337	1.1150682	0.09448
fetal kidney 100	1.1539389	0.09472
fetal fibroblast 268	1.1227795	0.09622
fetal lung 111	1.1301730	0.09642
fetal lung 127	1.1368979	0.09648
fetal renal cortex 211	1.1658024	0.09740
Mobilized CD8 325	1.2065730	0.09744
melanocyte 340	1.1278432	0.09782
fetal stomach 250	1.1795791	0.09886
fetal renal pelvis 233	1.1531125	0.09970
fetal renal pelvis 231	1.1451039	0.10160
melanocyte 338	1.1138598	0.10232
ES derived trophoblast 288	1.1323907	0.10260
fetal renal pelvis 227	1.1482067	0.10468
fetal stomach 245	1.1647939	0.10694
fetal renal pelvis 220	1.1500806	0.10756
fetal lung 128	1.1110968	0.10818
fetal kidney 83	1.1642052	0.11028
fetal lung 130	1.1228478	0.11262
ES cells 295	1.1612654	0.11338
heart 297	1.1547953	0.11370
fetal stomach 249	1.1663521	0.11610
fetal kidney 82	1.1548613	0.11788
CD8 21	1.1876758	0.11798
fetal fibroblast 271	1.1118670	0.11844
fetal fibroblast 279	1.1136112	0.11982
fetal renal pelvis 217	1.1438741	0.12092
fetal kidney 95	1.1363173	0.12098
fetal fibroblast 273	1.1084727	0.12424
fetal stomach 241	1.1576527	0.12440
fetal fibroblast 272	1.1099363	0.12448
fetal kidney 106	1.1333895	0.12740
fetal lung 117	1.1102283	0.12852
fetal lung 122	1.1239756	0.12858
fetal lung 139	1.1216365	0.12888
CD3 9	1.1685713	0.12940
fetal stomach 247	1.1694045	0.12962
Mobilized CD3 320	1.1808766	0.12990
fetal lung 131	1.1037870	0.13130
fetal lung 110	1.1184568	0.13240
fetal lung 116	1.1039070	0.13258
fetal ovary 195	1.1586798	0.13302
Mobilized CD4 322	1.1748938	0.13324
fetal renal pelvis 219	1.1599086	0.13330
fetal testes 255	1.1227661	0.13382
fetal stomach 246	1.1526472	0.13472
fetal fibroblast 277	1.1064901	0.13726
fetal lung 126	1.1031864	0.13860
fetal fibroblast 275	1.1001431	0.13948
fetal lung 138	1.1108537	0.13988
fetal brain 40	1.0957077	0.14090
fetal fibroblast 276	1.1009737	0.14112
iPS 302	1.1200355	0.14206
placenta 196	1.1263903	0.14226
fetal large intestine 61	1.1553089	0.14296
fetal stomach 248	1.1402392	0.14368
ES cells 296	1.1416573	0.14434
fetal spinal cord 238	1.1095343	0.14564
Mobilized CD4 323	1.1586488	0.14620
fetal renal cortex 213	1.1140124	0.14656
fetal kidney 99	1.1204065	0.14766
fetal lung 142	1.0996926	0.14802
placenta 199	1.1159524	0.14836
fetal stomach 253	1.1412562	0.14964
fetal kidney 89	1.1219545	0.15034
fetal stomach 251	1.1319693	0.15074
fetal kidney 84	1.1258029	0.15114
fetal kidney 88	1.1158955	0.15126
fetal lung 108	1.1112977	0.15278
fetal stomach 243	1.1221112	0.15320
fetal lung 113	1.0966826	0.15408
fetal lung 118	1.0940702	0.15478
fetal lung 129	1.1026303	0.15508
fetal kidney 85	1.1009309	0.15610
fetal spinal cord 239	1.1034466	0.15810
melanocyte 339	1.0894386	0.16358
fetal muscle 189	1.1069815	0.16364
fetal renal cortex 214	1.1111494	0.16452
fetal kidney 91	1.1030835	0.16500
fetal brain 38	1.0912381	0.16658
Mobilized CD3 321	1.1563737	0.16708
fetal renal cortex 205	1.1111406	0.16732
fetal lung 124	1.0969519	0.16904
CD4 13	1.1442285	0.17120
fetal renal pelvis 229	1.1078034	0.17204
ES cells 293	1.1150599	0.17380
fetal renal cortex 212	1.1030927	0.17470
fetal lung 114	1.0863489	0.17648
fetal renal pelvis 223	1.1111854	0.18232
fetal muscle 174	1.1008641	0.18364
fetal lung 123	1.0929365	0.18446
fetal brain 37	1.0819824	0.18594
fetal lung 121	1.0842067	0.18660
fetal fibroblast 267	1.0879663	0.18666
fetal brain 41	1.0795466	0.18910
CD3 11	1.1169370	0.19024
fetal renal pelvis 225	1.1043338	0.19204
fetal kidney 101	1.0980462	0.19264
fetal lung 120	1.0860649	0.19390
fetal spinal cord 236	1.0917338	0.19704
CD3 12	1.1304003	0.19786
fetal lung 141	1.0802929	0.19820
fetal renal pelvis 226	1.0949720	0.19840
CD4 15	1.1532819	0.19854
fetal renal cortex 206	1.0925413	0.19926
CD4 16	1.1306379	0.20002
fetal lung 133	1.0795070	0.20156
fetal lung 136	1.0853109	0.20210
fetal muscle 180	1.0946932	0.20508
fetal renal cortex 203	1.0941607	0.20750
fetal kidney 102	1.0992378	0.21068
fetal renal pelvis 232	1.0980547	0.21146
fetal kidney 105	1.0896924	0.21178
fetal kidney 107	1.0915798	0.21408
fetal kidney 97	1.0901935	0.21466
fetal muscle 177	1.0917606	0.21800
fetal renal cortex 210	1.0912107	0.22154
fetal renal pelvis 224	1.0837435	0.22286
fetal muscle 186	1.0818805	0.22306
fetal thymus 258	1.1003689	0.22346
fetal kidney 86	1.1035336	0.22452
fetal lung 115	1.0727664	0.22556
fetal brain 33	1.0716253	0.22726
fetal lung 132	1.0757980	0.22746
fetal kidney 104	1.0871235	0.22818
fetal renal cortex 215	1.0809958	0.22824
fetal spinal cord 235	1.0760078	0.22868
fetal lung 135	1.0764351	0.22934
fetal renal cortex 204	1.0776572	0.22948
Mobilized CD56 324	1.0992319	0.23344
CD8 20	1.0966170	0.23634
CD19 7	1.1069410	0.23690
fetal renal pelvis 218	1.1009433	0.23810
fetal kidney 93	1.0858872	0.23970
fetal lung 112	1.0704193	0.24072
fetal kidney 90	1.0810125	0.24132
fetal thymus 266	1.0907380	0.24244
fetal kidney 96	1.0750950	0.24398
CD4 14	1.0898180	0.24800
CD19 6	1.0979658	0.24906
CD8 19	1.1074899	0.24964
fetal thymus 256	1.0828949	0.25026
fetal muscle 173	1.0741058	0.25144
CD8 22	1.0983701	0.25156
fetal kidney 92	1.0703425	0.25488
fetal thymus 263	1.0724937	0.25598
fetal kidney 81	1.0672944	0.25628
fetal spinal cord 237	1.0650811	0.25640
CD3 10	1.0884648	0.25706
fetal muscle 175	1.0682801	0.25954
fetal renal pelvis 221	1.0693972	0.26034
fetal muscle 185	1.0728921	0.26050
fetal muscle 172	1.0611752	0.26172
CD56 18	1.0913237	0.26416
CD19 5	1.0860956	0.26482
fetal muscle 184	1.0628427	0.26594
fetal renal pelvis 222	1.0715745	0.26980
fetal renal cortex 208	1.0706507	0.27000
fetal muscle 171	1.0641936	0.27058
fetal muscle 152	1.0631975	0.27132
fetal thymus 261	1.0806676	0.27362
fetal thymus 260	1.0778068	0.27636
fetal brain 36	1.0535249	0.27702
fetal muscle 170	1.0613357	0.27784
fetal kidney 94	1.0697044	0.27786
fetal kidney 98	1.0668675	0.27950
hematopoietic stem cell 307	1.0627649	0.27996
fetal muscle 147	1.0601734	0.28896
fetal muscle 194	1.0541568	0.29002
fetal thymus 257	1.0757405	0.29140
fetal brain 31	1.0413463	0.29334
fetal muscle 188	1.0552957	0.29596
fetal muscle 187	1.0546563	0.29604
fetal muscle 155	1.0599770	0.29730
fetal lung 140	1.0499812	0.29960
fetal muscle 176	1.0533269	0.30222
fetal brain 39	1.0440820	0.30284
hematopoietic stem cell 309	1.0559708	0.30410
fetal renal pelvis 230	1.0604511	0.30796
placenta 201	1.0572687	0.30830
fetal muscle 159	1.0566566	0.30866
fetal muscle 179	1.0529467	0.30872
ES derived NP 292	1.0608544	0.30962
fetal muscle 158	1.0508804	0.31202
fetal lung 119	1.0456221	0.31274
fetal muscle 183	1.0496508	0.31398
fetal muscle 182	1.0504511	0.31558
fetal muscle 190	1.0543466	0.31646
fetal thymus 262	1.0724472	0.31740
fetal thymus 265	1.0607501	0.31846
fetal renal cortex 202	1.0483900	0.32514
fetal muscle 191	1.0472259	0.32922
fetal muscle 144	1.0484761	0.33106
fetal small intestine 75	1.0676978	0.33348
hematopoietic stem cell 316	1.0498978	0.33352
fetal muscle 166	1.0442561	0.33478
fetal muscle 143	1.0412211	0.33672
fetal renal cortex 209	1.0458443	0.33918
ES derived NP 291	1.0515054	0.34108
fetal muscle 164	1.0432637	0.34138
fetal muscle 192	1.0416452	0.34154
fetal muscle 156	1.0426141	0.34188
fetal muscle 148	1.0447174	0.34204
fetal muscle 161	1.0433130	0.34212
fetal muscle 163	1.0431247	0.34426
fetal muscle 149	1.0440442	0.34456
fetal muscle 169	1.0419908	0.34674
fetal thymus 259	1.0500396	0.34958
hematopoietic stem cell 319	1.0437247	0.35018
fetal muscle 165	1.0379136	0.35132
fetal brain 34	1.0302241	0.35418
fetal thymus 264	1.0455832	0.35486
fetal muscle 145	1.0416580	0.35842
fetal brain 35	1.0277011	0.36152
hematopoietic stem cell 315	1.0374644	0.36502
hematopoietic stem cell 306	1.0364783	0.36546
hematopoietic stem cell 311	1.0346722	0.36888
CD56 17	1.0379053	0.37560
fetal muscle 146	1.0317553	0.37818
fetal muscle 162	1.0256257	0.39554
fetal muscle 181	1.0265710	0.39584
fetal muscle 157	1.0254293	0.39590
fetal muscle 150	1.0261544	0.39986
fetal muscle 178	1.0262622	0.40172
fetal muscle 160	1.0245310	0.40616
CD14 4	1.0279899	0.40706
CD14 3	1.0293999	0.40768
fetal brain 29	1.0165454	0.41232
fetal muscle 193	1.0222185	0.41274
hematopoietic stem cell 305	1.0228845	0.41338
ES derived trophoblast 287	1.0238501	0.41408
fetal muscle 153	1.0222945	0.41746
fetal brain 30	1.0119495	0.43278
fetal muscle 151	1.0143438	0.43988
fetal muscle 154	1.0091382	0.45838
fetal muscle 167	1.0092762	0.45842
hematopoietic stem cell 308	1.0044474	0.47414
hematopoietic stem cell 318	1.0025387	0.48112
hematopoietic stem cell 313	1.0007810	0.48634
fetal muscle 168	1.0008791	0.48742
hematopoietic stem cell 312	1.0014864	0.48766
hematopoietic stem cell 310	1.0010243	0.49066
hematopoietic stem cell 317	0.9937647	0.51878
hematopoietic stem cell 314	0.9905291	0.53000

Inflammatory bowel disease

DHS sample	fold enrichment	p value
CD14 4	1.5561914	0.00002
CD14 3	1.5272620	0.00002
Mobilized CD3 320	1.5060797	0.00002
Mobilized CD56 324	1.4907069	0.00002
CD4 14	1.4865679	0.00002
CD4 13	1.4738694	0.00002
CD3 9	1.4677803	0.00002
CD8 20	1.4473906	0.00002
Mobilized CD4 322	1.4456814	0.00002
CD56 17	1.4456040	0.00002
CD3 11	1.4344832	0.00002
Mobilized CD4 323	1.4258441	0.00002
CD8 21	1.4139130	0.00002
CD4 16	1.4000530	0.00002
CD3 10	1.3999679	0.00002
CD56 18	1.3828322	0.00002
CD3 12	1.3812604	0.00002
CD19 5	1.3703267	0.00002
CD8 22	1.3576630	0.00002
fetal thymus 256	1.3335847	0.00002
hematopoietic stem cell 310	1.2913038	0.00002
hematopoietic stem cell 314	1.2666245	0.00002
ES derived MSC 290	1.2507962	0.00002
fetal lung 301	1.2347463	0.00002
breast 1	1.2238130	0.00002
breast 2	1.2192751	0.00002
fetal fibroblast 273	1.2136967	0.00002
fetal fibroblast 282	1.1929344	0.00002
CD19 6	1.3608950	0.00004
hematopoietic stem cell 312	1.2501500	0.00004
hematopoietic stem cell 309	1.2448054	0.00004
ES derived MSC 289	1.2434534	0.00004
fetal lung 300	1.2291385	0.00004
fetal fibroblast 281	1.2122675	0.00004
fetal fibroblast 277	1.2049573	0.00004
keratinocyte 335	1.1870131	0.00004
Mobilized CD3 321	1.3678685	0.00006
CD19 7	1.3477849	0.00006
hematopoietic stem cell 317	1.2277389	0.00006
hematopoietic stem cell 308	1.2135874	0.00008
fetal fibroblast 271	1.2023024	0.00008
fetal fibroblast 275	1.1901386	0.00008
keratinocyte 336	1.1867305	0.00008
fetal large intestine 61	1.3208234	0.00010
fetal fibroblast 272	1.1972455	0.00010
fetal fibroblast 276	1.1969928	0.00010
fetal fibroblast 268	1.1942460	0.00010
fetal lung 298	1.2194503	0.00012
hematopoietic stem cell 313	1.2177489	0.00012
fetal fibroblast 269	1.1825191	0.00012
Mobilized CD8 325	1.3305759	0.00014
fetal fibroblast 279	1.1946150	0.00016
fetal thymus 258	1.2719605	0.00018
fetal fibroblast 274	1.1982101	0.00018
CD8 19	1.3340096	0.00020
fetal fibroblast 278	1.1916547	0.00020
fibroblast 331	1.1893186	0.00022
fetal thymus 264	1.2669875	0.00026
fetal thymus 265	1.2635986	0.00026
fetal fibroblast 280	1.1798642	0.00026
fibroblast 332	1.1752344	0.00026
fetal fibroblast 267	1.1848648	0.00028
keratinocyte 334	1.1693120	0.00030
fetal thymus 266	1.2614455	0.00034
keratinocyte 333	1.1650267	0.00034
small intestine 342	1.2213720	0.00038
fetal lung 299	1.1979310	0.00042
placenta 200	1.1832433	0.00046
fetal small intestine 74	1.2107809	0.00052
hematopoietic stem cell 306	1.2038875	0.00056
CD4 15	1.3278925	0.00070
hematopoietic stem cell 315	1.1996093	0.00076
fetal large intestine 57	1.1900085	0.00078
fetal small intestine 75	1.3254658	0.00082
placenta 201	1.2075484	0.00084
fetal large intestine 67	1.2119304	0.00088
fibroblast 330	1.1708252	0.00092
hematopoietic stem cell 318	1.2004831	0.00106
fetal fibroblast 270	1.1623094	0.00106
fetal thymus 260	1.2377338	0.00110
fetal large intestine 58	1.1911767	0.00112
fetal small intestine 78	1.1807095	0.00112
fetal large intestine 60	1.2040175	0.00120
hematopoietic stem cell 316	1.2022318	0.00122
hematopoietic stem cell 305	1.1942705	0.00122
hematopoietic stem cell 311	1.1825240	0.00128
fetal large intestine 55	1.1905114	0.00136
fetal small intestine 70	1.1733942	0.00146
stomach 284	1.2061064	0.00162
fetal small intestine 80	1.1877150	0.00172
fetal small intestine 68	1.1877572	0.00174
fetal large intestine 64	1.1881798	0.00190
hematopoietic stem cell 307	1.1774517	0.00190
ES derived trophoblast 288	1.1599801	0.00210
fetal small intestine 77	1.1936606	0.00224
placenta 199	1.1703840	0.00252
fibroblast 329	1.1466182	0.00294
muscle 341	1.2035227	0.00298
placenta 197	1.1754748	0.00316
placenta 196	1.1773145	0.00320
fetal thymus 257	1.2261527	0.00328
fetal thymus 259	1.2205698	0.00336
placenta 198	1.1578671	0.00338
stomach 283	1.1994160	0.00342
fetal lung 111	1.1410211	0.00454
ES derived trophoblast 287	1.1650182	0.00486
CD20 8	1.1622856	0.00486
fetal lung 128	1.1245253	0.00530
fetal small intestine 69	1.1824779	0.00538
fetal large intestine 54	1.1642590	0.00594
fetal small intestine 79	1.1739261	0.00700
pancreas 328	1.1689636	0.00702
fetal large intestine 56	1.1589725	0.00710
fetal thymus 261	1.1899267	0.00770
fetal large intestine 62	1.1716740	0.00778
fetal large intestine 53	1.1782224	0.00802
melanocyte 338	1.1104498	0.00902
hematopoietic stem cell 319	1.1543901	0.00940
pancreas 327	1.1665292	0.01030
melanocyte 337	1.1057882	0.01060
fetal lung 140	1.1208413	0.01104
fetal stomach 244	1.1575458	0.01110
melanocyte 340	1.1194117	0.01124
fetal large intestine 66	1.1578308	0.01128
fetal small intestine 73	1.1524322	0.01132
melanocyte 339	1.1118314	0.01150
fetal large intestine 59	1.1586422	0.01156
fetal lung 134	1.1286010	0.01160
fetal lung 133	1.1182901	0.01204
fetal large intestine 65	1.1497041	0.01248
fetal lung 129	1.1219982	0.01268
fetal lung 141	1.1152649	0.01276
fetal small intestine 76	1.1550716	0.01280
fetal small intestine 72	1.1426630	0.01370
fetal thymus 262	1.2016370	0.01506
fetal large intestine 63	1.1427227	0.01754
fetal lung 117	1.1071037	0.02176
fetal lung 126	1.1012018	0.02264
fetal lung 116	1.0958194	0.02614
fetal small intestine 71	1.1281991	0.02746
fetal stomach 251	1.1288843	0.02788
fetal stomach 247	1.1522321	0.02812
fetal lung 110	1.1063253	0.02890
fetal muscle 172	1.0958735	0.03312
fetal stomach 252	1.1276106	0.03316
fetal lung 125	1.1032792	0.03392
fetal stomach 253	1.1312739	0.03414
fetal lung 138	1.0991477	0.03488
fetal stomach 245	1.1259485	0.03576
fetal lung 131	1.0878153	0.03588
fetal lung 121	1.0919800	0.03736
fetal lung 108	1.1001435	0.04350
fetal lung 120	1.0922939	0.04524
fetal stomach 249	1.1236451	0.04602
fetal stomach 246	1.1221225	0.04608
fetal lung 127	1.0929689	0.04832
fetal stomach 248	1.1173614	0.04848
fetal lung 122	1.0944469	0.05110
fetal lung 114	1.0801983	0.05302
fetal lung 115	1.0841313	0.05362
fetal stomach 243	1.1020710	0.05638
fetal lung 137	1.0835647	0.05758
fetal lung 109	1.0753837	0.05848
fetal lung 136	1.0853882	0.06044
fetal lung 130	1.0833880	0.06168
fetal stomach 242	1.1248384	0.06292
fetal stomach 250	1.1107638	0.06304
fetal lung 118	1.0766356	0.06324
ovary 326	1.1134802	0.06586
fetal stomach 240	1.1073843	0.07264
fetal muscle 149	1.0900748	0.07274
fetal stomach 241	1.1032818	0.07820
fetal lung 113	1.0721357	0.07918
fetal lung 123	1.0777230	0.07932
fetal lung 132	1.0767860	0.08264
fetal lung 139	1.0784178	0.08284
fetal muscle 148	1.0817367	0.08400
fetal lung 135	1.0752892	0.08568
fetal muscle 153	1.0829929	0.09122
fetal lung 119	1.0655450	0.09770
fetal lung 142	1.0648992	0.09826
fetal lung 124	1.0690886	0.10004
fetal adrenal gland 26	1.0739674	0.11304
fetal adrenal gland 25	1.0769421	0.11602
fetal skin 234	1.0723551	0.11814
fetal muscle 194	1.0614837	0.11922
fetal muscle 164	1.0650771	0.13146
fetal muscle 180	1.0662591	0.13634
fetal kidney 106	1.0658618	0.13972
fetal adrenal gland 28	1.0547976	0.15432
fetal muscle 152	1.0558451	0.15740
fetal adrenal gland 24	1.0680750	0.15774
fetal renal pelvis 220	1.0603938	0.16900
fetal lung 112	1.0523275	0.16982
fetal muscle 150	1.0562965	0.17032
fetal muscle 155	1.0586344	0.17052
fetal muscle 151	1.0580662	0.17330
fetal muscle 168	1.0553880	0.17662
fetal muscle 143	1.0492345	0.18008
fetal renal cortex 211	1.0597319	0.18060
heart 297	1.0583440	0.18404
fetal muscle 159	1.0542998	0.18654
fetal muscle 156	1.0498773	0.18678
fetal adrenal gland 27	1.0534667	0.18946
ES derived mesoderm 286	1.0420108	0.18964
fetal muscle 154	1.0535566	0.19172
fetal kidney 89	1.0528075	0.20000
fetal renal cortex 214	1.0502825	0.20106
fetal kidney 83	1.0570196	0.20336
fetal renal pelvis 227	1.0499564	0.20462
fetal kidney 84	1.0527979	0.20536
fetal testes 255	1.0475183	0.20578
fetal muscle 177	1.0500036	0.20654
fetal kidney 100	1.0492669	0.20882
fetal muscle 160	1.0476902	0.21218
fetal muscle 190	1.0491142	0.21244
fetal renal pelvis 231	1.0449886	0.21830
fetal renal cortex 213	1.0450255	0.21918
fetal muscle 166	1.0430659	0.22436
fetal kidney 87	1.0455656	0.22512
fetal kidney 91	1.0434063	0.22524
fetal muscle 191	1.0443421	0.22534
fetal muscle 169	1.0433656	0.22592
fetal renal pelvis 228	1.0485573	0.22868
fetal renal cortex 207	1.0456517	0.22972
fetal muscle 157	1.0385475	0.23514
fetal muscle 181	1.0415404	0.23520
fetal kidney 95	1.0437722	0.23800
ES derived mesoderm 285	1.0361547	0.24010
fetal renal pelvis 219	1.0513532	0.24118
fetal muscle 147	1.0409161	0.24130
fetal renal pelvis 233	1.0435151	0.24186
ES cells 294	1.0483889	0.24214
fetal renal pelvis 218	1.0511415	0.24356
fetal renal cortex 205	1.0419231	0.24600
fetal kidney 104	1.0421687	0.24738
fetal muscle 167	1.0373322	0.24870
fetal kidney 85	1.0372399	0.24908
fetal kidney 82	1.0443931	0.25146
fetal renal pelvis 226	1.0391757	0.25504
fetal kidney 88	1.0377032	0.25964
fetal muscle 145	1.0402884	0.26194
fetal muscle 188	1.0349307	0.26374
fetal muscle 163	1.0361346	0.26802
fetal muscle 189	1.0350461	0.26814
fetal kidney 107	1.0366200	0.27420
fetal renal pelvis 232	1.0375795	0.27496
fetal muscle 176	1.0323161	0.27764
fetal renal pelvis 221	1.0341419	0.27942
fetal renal pelvis 217	1.0359684	0.28208
fetal muscle 182	1.0319957	0.28226
fetal renal pelvis 216	1.0357724	0.28262
fetal muscle 174	1.0329825	0.28470
fetal muscle 161	1.0323879	0.28702
fetal muscle 187	1.0301971	0.28966
fetal muscle 183	1.0303578	0.29032
fetal adrenal gland 23	1.0306899	0.29384
fetal kidney 102	1.0355285	0.29394
fetal muscle 146	1.0298794	0.29394
fetal muscle 192	1.0299210	0.29404
fetal muscle 179	1.0302883	0.29770
fetal renal cortex 215	1.0304317	0.29818
fetal renal cortex 206	1.0304174	0.30134
fetal muscle 193	1.0300366	0.30392
fetal muscle 186	1.0279063	0.30592
fetal muscle 162	1.0275595	0.30718
fetal kidney 86	1.0353586	0.30790
fetal kidney 98	1.0306076	0.30864
fetal renal cortex 203	1.0301090	0.30968
fetal muscle 185	1.0288915	0.31250
fetal muscle 158	1.0262039	0.31496
fetal renal pelvis 230	1.0295895	0.32406
fetal kidney 99	1.0268030	0.32730
fetal renal pelvis 225	1.0268947	0.32860
fetal muscle 175	1.0247494	0.32996
fetal renal cortex 209	1.0261665	0.33590
fetal kidney 96	1.0244294	0.33876
fetal thymus 263	1.0238946	0.33972
fetal muscle 165	1.0212812	0.34458
fetal renal pelvis 229	1.0231758	0.34986
fetal renal pelvis 222	1.0232776	0.35308
fetal renal cortex 204	1.0210473	0.35308
fetal muscle 171	1.0205676	0.35394
fetal kidney 97	1.0221663	0.35500
fetal muscle 178	1.0209817	0.35792
fetal muscle 144	1.0209112	0.36164
fetal renal pelvis 223	1.0202266	0.37268
fetal kidney 103	1.0191920	0.37436
fetal renal cortex 208	1.0199176	0.37472
fetal muscle 184	1.0166820	0.37480
fetal muscle 173	1.0166873	0.38266
fetal kidney 93	1.0184819	0.38296
ES cells 296	1.0197585	0.38394
fetal kidney 92	1.0167319	0.38408
fetal kidney 90	1.0174265	0.38506
fetal kidney 101	1.0159872	0.39134
ES cells 295	1.0172652	0.39508
fetal renal cortex 210	1.0160277	0.39618
fetal testes 254	1.0137305	0.40912
iPS 303	1.0127066	0.40980
fetal renal pelvis 224	1.0117663	0.41550
fetal kidney 94	1.0105076	0.42988
fetal heart 42	1.0091064	0.43474
fetal heart 46	1.0080833	0.44294
fetal kidney 105	1.0062291	0.45390
fetal renal cortex 202	1.0057472	0.45688
fetal ovary 195	1.0057087	0.46166
fetal renal cortex 212	1.0045923	0.46248
iPS 302	1.0039362	0.46690
fetal heart 51	1.0002851	0.49328
fetal muscle 170	0.9992686	0.50060
fetal heart 50	0.9980130	0.50932
fetal heart 52	0.9971010	0.51872
fetal heart 48	0.9956403	0.52246
fetal kidney 81	0.9957319	0.52930
ES derived NP 291	0.9918170	0.54138
fetal heart 43	0.9903555	0.56294
ES cells 293	0.9882393	0.56676
fetal heart 45	0.9865180	0.58254
fetal heart 47	0.9820840	0.60400
fetal heart 44	0.9782702	0.61644
iPS 304	0.9814753	0.62264
fetal heart 49	0.9803869	0.62418
ES derived NP 292	0.9626155	0.70530
fetal spinal cord 236	0.9544087	0.78236
fetal spinal cord 235	0.9350742	0.88120
fetal spinal cord 237	0.9353856	0.88264
fetal spinal cord 238	0.9309365	0.89532
fetal brain 41	0.9210168	0.94846
fetal spinal cord 239	0.9076933	0.95440
fetal brain 36	0.9142851	0.96212
fetal brain 38	0.9003545	0.97736
fetal brain 40	0.9025527	0.98372
fetal brain 35	0.9061765	0.98642
fetal brain 34	0.8978713	0.98932
fetal brain 29	0.9026538	0.99314
fetal brain 39	0.8880471	0.99388
fetal brain 37	0.8717765	0.99748
fetal brain 30	0.8893843	0.99750
fetal brain 31	0.8843196	0.99816
fetal brain 33	0.8536822	0.99848
fetal brain 32	0.8595251	0.99872

Systemic lupus erythematosus 2

DHS sample	fold enrichment	p value
Mobilized CD56 324	2.1006897	0.00010
Mobilized CD3 320	2.2078373	0.00048
CD4 13	2.0470306	0.00116
CD19 5	1.9572379	0.00146
CD3 10	1.8847996	0.00170
CD4 14	1.7899317	0.00264
CD19 6	1.8839883	0.00412
fetal fibroblast 273	1.4875760	0.00432
CD19 7	1.8637093	0.00516
CD3 11	1.7273950	0.00630
CD3 9	1.7756602	0.00648
melanocyte 338	1.3899072	0.00778
CD8 20	1.6854830	0.00890
CD14 4	1.6104424	0.00932
fetal fibroblast 274	1.4362140	0.01020
fetal fibroblast 272	1.4398670	0.01054
fetal fibroblast 269	1.4360781	0.01066
fetal fibroblast 268	1.4314007	0.01080
fibroblast 331	1.4220468	0.01156
CD8 21	1.7534976	0.01180
fibroblast 332	1.4244968	0.01218
CD56 17	1.6004955	0.01274
fetal thymus 256	1.6109417	0.01322
fetal fibroblast 271	1.4125433	0.01356
fetal thymus 264	1.5965762	0.01538
Mobilized CD4 322	1.7100625	0.01558
fetal fibroblast 276	1.3853433	0.01598
Mobilized CD4 323	1.6610405	0.01644
fetal fibroblast 280	1.3955669	0.01684
keratinocyte 335	1.3582464	0.01684
hematopoietic stem cell 310	1.3915862	0.01716
fetal fibroblast 279	1.3917625	0.01778
fetal fibroblast 267	1.4246059	0.01810
keratinocyte 336	1.3564267	0.01846
fetal lung 300	1.4248658	0.01856
fetal fibroblast 282	1.3762529	0.01902
fetal fibroblast 275	1.3607179	0.02162
fetal fibroblast 277	1.3890866	0.02248
CD3 12	1.6421249	0.02270
fetal fibroblast 281	1.3789050	0.02330
fetal fibroblast 278	1.3805855	0.02372
keratinocyte 333	1.3228301	0.02506
fetal thymus 258	1.5508435	0.02584
CD56 18	1.6176918	0.02910
fetal lung 298	1.4069068	0.02914
keratinocyte 334	1.3228834	0.02924
fetal lung 301	1.3854938	0.02994
melanocyte 337	1.2882593	0.03140
fetal thymus 265	1.5131223	0.03224
ES derived MSC 290	1.3995042	0.03374
breast 2	1.3315971	0.03462
fetal small intestine 68	1.4058198	0.03470
fetal fibroblast 270	1.3505159	0.03542
Mobilized CD3 321	1.5912462	0.03644
breast 1	1.3198221	0.03994
ES derived MSC 289	1.3796683	0.04198
fetal thymus 260	1.4871176	0.04242
CD4 16	1.5359256	0.04300
CD8 19	1.5811372	0.04616
fibroblast 329	1.3086868	0.04658
hematopoietic stem cell 314	1.3271923	0.04680
fetal heart 47	1.4083712	0.04886
fetal thymus 266	1.4380304	0.05348
fetal large intestine 61	1.4448859	0.05478
CD8 22	1.4761197	0.05734
fetal large intestine 67	1.3664279	0.05986
CD14 3	1.4144739	0.06062
placenta 200	1.3032756	0.06130
fetal large intestine 58	1.3347814	0.06152
melanocyte 339	1.2509191	0.06210
fibroblast 330	1.2806608	0.06578
fetal heart 45	1.3311383	0.06958
CD20 8	1.3175821	0.07412
fetal small intestine 78	1.3010105	0.07576
fetal thymus 259	1.4183922	0.07582
placenta 198	1.2801121	0.08452
fetal large intestine 55	1.2970357	0.08638
CD4 15	1.4798415	0.08708
fetal lung 299	1.2774035	0.08766
fetal large intestine 64	1.3039814	0.09002
fetal large intestine 54	1.3084838	0.09222
fetal small intestine 74	1.2962081	0.09280
hematopoietic stem cell 317	1.2459227	0.09292
fetal thymus 261	1.3529298	0.09984
fetal small intestine 70	1.2559389	0.10174
fetal testes 255	1.2532293	0.10446
fetal heart 46	1.2770286	0.10660
hematopoietic stem cell 312	1.2554241	0.10806
fetal large intestine 60	1.2773283	0.10908
ES derived mesoderm 285	1.2156090	0.11252
fetal heart 43	1.2455500	0.11414
fetal small intestine 80	1.2638177	0.11658
melanocyte 340	1.1931632	0.12060
hematopoietic stem cell 313	1.2234855	0.12526
fetal small intestine 75	1.3753594	0.12960
Mobilized CD8 325	1.3341797	0.13040
fetal large intestine 57	1.2356139	0.13474
fetal heart 49	1.2354031	0.13508
hematopoietic stem cell 309	1.2211446	0.14280
fetal thymus 257	1.2995639	0.14300
placenta 199	1.2171456	0.14304
fetal large intestine 56	1.2352622	0.14588
placenta 197	1.2252063	0.15028
fetal muscle 172	1.1808092	0.16304
fetal lung 141	1.1681881	0.16628
fetal lung 111	1.1784752	0.16794
fetal large intestine 62	1.2241684	0.17330
fetal small intestine 73	1.2042115	0.17862
fetal small intestine 72	1.2021755	0.17862
hematopoietic stem cell 306	1.1846999	0.18138
hematopoietic stem cell 305	1.1877145	0.18258
fetal lung 121	1.1561372	0.18264
fetal lung 128	1.1495523	0.18300
hematopoietic stem cell 318	1.1944462	0.18500
small intestine 342	1.1907625	0.18970
fetal heart 48	1.1809991	0.19130
stomach 284	1.2056666	0.19344
fetal heart 42	1.1759179	0.19732
hematopoietic stem cell 308	1.1629489	0.19904
fetal lung 126	1.1384224	0.21378
fetal stomach 244	1.1716133	0.22034
fetal heart 51	1.1582710	0.22112
fetal muscle 176	1.1518252	0.22334
fetal muscle 151	1.1639195	0.22860
hematopoietic stem cell 315	1.1552869	0.22884
fetal lung 116	1.1247824	0.22916
fetal heart 52	1.1403037	0.22924
fetal lung 140	1.1242645	0.23918
placenta 201	1.1544765	0.23950
hematopoietic stem cell 316	1.1441722	0.24686
hematopoietic stem cell 307	1.1352196	0.24722
fetal heart 50	1.1417063	0.24742
fetal muscle 152	1.1319465	0.24814
fetal lung 133	1.1155038	0.25016
hematopoietic stem cell 311	1.1298884	0.25124
heart 297	1.1465501	0.25182
fetal large intestine 66	1.1493252	0.25310
fetal large intestine 63	1.1480546	0.25334
fetal heart 44	1.1536002	0.25486
fetal small intestine 79	1.1477110	0.25880
fetal lung 118	1.1054797	0.25914
fetal stomach 240	1.1475220	0.26620
fetal thymus 262	1.1683920	0.26848
fetal muscle 143	1.1040682	0.28290
ovary 326	1.1413068	0.28464
fetal stomach 252	1.1191832	0.28908
fetal muscle 194	1.0939853	0.29424
fetal small intestine 76	1.1194337	0.29776
fetal stomach 246	1.1170588	0.30108
fetal muscle 168	1.1016002	0.30220
fetal muscle 167	1.0982937	0.30324
fetal lung 131	1.0833864	0.30414
fetal muscle 160	1.0979241	0.30750
ES derived trophoblast 288	1.0924534	0.31034
hematopoietic stem cell 319	1.0955671	0.31860
ES derived mesoderm 286	1.0719064	0.31896
fetal large intestine 65	1.1014612	0.31908
fetal stomach 248	1.0995690	0.31992
muscle 341	1.1051897	0.32060
fetal muscle 164	1.0895617	0.32180
fetal muscle 181	1.0886684	0.32190
fetal large intestine 59	1.0962651	0.32850
fetal small intestine 77	1.0868684	0.33606
fetal lung 117	1.0689677	0.33966
fetal lung 119	1.0682247	0.34048
fetal lung 129	1.0700431	0.34162
placenta 196	1.0813173	0.34310
fetal lung 115	1.0671019	0.34494
fetal lung 108	1.0717607	0.34568
fetal muscle 150	1.0691015	0.35472
fetal muscle 148	1.0655668	0.36096
fetal muscle 179	1.0641987	0.36154
fetal lung 120	1.0567277	0.36602
fetal muscle 149	1.0644087	0.36664
fetal muscle 193	1.0600611	0.36832
fetal lung 137	1.0532089	0.37170
fetal testes 254	1.0608509	0.37278
fetal muscle 156	1.0498318	0.38640
fetal stomach 241	1.0539713	0.38882
fetal muscle 169	1.0463439	0.39206
fetal lung 142	1.0410934	0.39210
fetal muscle 154	1.0478656	0.39472
fetal muscle 153	1.0477530	0.39542
fetal lung 110	1.0415949	0.39658
fetal muscle 157	1.0426280	0.39670
fetal lung 114	1.0392912	0.39828
fetal stomach 249	1.0472983	0.40270
fetal lung 127	1.0407405	0.40294
fetal muscle 166	1.0389852	0.40408
fetal muscle 155	1.0415509	0.40476
fetal adrenal gland 28	1.0382879	0.40644
fetal muscle 174	1.0376339	0.41002
fetal muscle 162	1.0350124	0.41454
fetal lung 138	1.0324483	0.41474
fetal muscle 182	1.0335898	0.41620
fetal lung 130	1.0310628	0.41940
fetal muscle 175	1.0299293	0.42416
fetal lung 123	1.0270149	0.42516
fetal muscle 159	1.0276206	0.42886
ES derived trophoblast 287	1.0337849	0.42938
fetal lung 132	1.0262668	0.42998
fetal lung 134	1.0248975	0.43172
fetal stomach 245	1.0275524	0.43246
fetal lung 125	1.0240844	0.43264
pancreas 328	1.0293898	0.43328
fetal stomach 253	1.0255583	0.43410
fetal stomach 251	1.0221850	0.43502
fetal muscle 180	1.0244213	0.43812
fetal small intestine 71	1.0234297	0.43844
fetal muscle 165	1.0190320	0.44548
fetal muscle 191	1.0171460	0.44680
fetal muscle 192	1.0156121	0.44872
fetal muscle 187	1.0152974	0.44878
fetal stomach 243	1.0165982	0.45150
fetal muscle 147	1.0132325	0.45566
fetal muscle 188	1.0101725	0.45882
fetal muscle 163	1.0085991	0.45908
fetal kidney 91	1.0105908	0.46580
fetal renal cortex 207	1.0015996	0.47770
fetal skin 234	1.0027212	0.48256
stomach 283	0.9972523	0.48692
fetal muscle 183	0.9919820	0.49528
fetal adrenal gland 27	0.9948619	0.49622
fetal muscle 178	0.9892178	0.49690
fetal adrenal gland 26	0.9943271	0.49754
fetal stomach 250	0.9836611	0.49826
fetal muscle 161	0.9895970	0.49856
fetal muscle 177	0.9864911	0.49928
fetal ovary 195	0.9801349	0.50140
fetal kidney 92	0.9900900	0.50142
fetal lung 109	0.9909194	0.50664
fetal muscle 173	0.9846517	0.50924
fetal muscle 146	0.9873545	0.50950
fetal lung 124	0.9863403	0.51006
fetal lung 136	0.9856956	0.51268
ES derived NP 292	0.9774175	0.51504
fetal muscle 144	0.9770589	0.51586
fetal lung 113	0.9843553	0.51694
fetal adrenal gland 24	0.9782811	0.52006
fetal muscle 171	0.9802758	0.52374
fetal small intestine 69	0.9731348	0.52486
fetal kidney 85	0.9793362	0.52942
fetal muscle 145	0.9684536	0.53080
fetal lung 122	0.9726988	0.53364
fetal stomach 242	0.9562258	0.53454
fetal muscle 184	0.9745186	0.53460
fetal muscle 189	0.9740797	0.53496
fetal kidney 103	0.9700645	0.53698
fetal kidney 81	0.9726875	0.53890
fetal lung 139	0.9705931	0.54108
fetal lung 112	0.9736774	0.54278
fetal lung 135	0.9670033	0.55106
fetal kidney 106	0.9602079	0.55352
fetal stomach 247	0.9437916	0.55362
fetal kidney 95	0.9583320	0.55892
fetal renal cortex 213	0.9615489	0.56246
fetal muscle 158	0.9611510	0.56364
fetal thymus 263	0.9524792	0.57402
fetal renal cortex 215	0.9429399	0.59032
fetal muscle 186	0.9442872	0.59312
fetal large intestine 53	0.9275576	0.60040
fetal renal pelvis 220	0.9314749	0.60718
fetal renal pelvis 226	0.9264842	0.61788
fetal kidney 96	0.9249366	0.62210
ES derived NP 291	0.9148670	0.62578
iPS 302	0.9273254	0.62822
iPS 304	0.9248541	0.64220
fetal adrenal gland 23	0.9200115	0.64626
fetal renal pelvis 221	0.9095373	0.65126
fetal adrenal gland 25	0.9024937	0.65730
fetal kidney 99	0.9019993	0.66770
fetal renal pelvis 227	0.9004084	0.67026
fetal kidney 104	0.8920753	0.67048
fetal muscle 170	0.9056931	0.67416
fetal kidney 107	0.8859964	0.68558
fetal renal pelvis 231	0.8971644	0.68718
fetal renal pelvis 217	0.8827838	0.69056
fetal kidney 84	0.8804639	0.69106
fetal kidney 83	0.8670909	0.69282
pancreas 327	0.8660155	0.69820
fetal muscle 185	0.8817376	0.69958
fetal muscle 190	0.8738603	0.70198
iPS 303	0.8897294	0.70594
fetal spinal cord 235	0.8917077	0.70770
fetal renal pelvis 230	0.8611002	0.70888
fetal kidney 105	0.8742145	0.71222
fetal renal pelvis 219	0.8377949	0.71736
ES cells 293	0.8617814	0.72290
fetal kidney 98	0.8608274	0.72338
fetal kidney 94	0.8554180	0.72426
fetal renal cortex 206	0.8651340	0.72566
fetal kidney 88	0.8648363	0.72628
fetal renal cortex 214	0.8678154	0.72704
fetal renal pelvis 216	0.8547582	0.73080
fetal renal cortex 209	0.8525670	0.73346
fetal renal cortex 212	0.8641167	0.73382
ES cells 295	0.8448832	0.73744
fetal renal pelvis 232	0.8495065	0.73776
fetal renal pelvis 228	0.8473938	0.73940
fetal kidney 100	0.8582902	0.74282
fetal renal cortex 205	0.8512077	0.74580
fetal renal cortex 203	0.8447974	0.74706
fetal renal cortex 204	0.8592569	0.74808
fetal kidney 102	0.8361962	0.75010
fetal kidney 87	0.8547265	0.75178
fetal kidney 101	0.8455401	0.75494
fetal renal pelvis 224	0.8503789	0.76046
fetal renal pelvis 223	0.8234803	0.76974
fetal renal cortex 208	0.8247159	0.77070
fetal kidney 89	0.8360483	0.77112
fetal renal pelvis 229	0.8333076	0.77714
fetal renal pelvis 218	0.7868200	0.78122
fetal renal cortex 202	0.8332134	0.78224
fetal kidney 97	0.8255588	0.78540
fetal renal pelvis 225	0.8161705	0.78542
fetal renal cortex 211	0.8130056	0.78968
ES cells 296	0.8074167	0.79072
fetal renal pelvis 233	0.8182200	0.79342
fetal kidney 82	0.7917241	0.80524
fetal renal pelvis 222	0.8008809	0.80660
fetal kidney 90	0.8008142	0.81026
fetal renal cortex 210	0.7924173	0.81552
fetal kidney 93	0.7836485	0.82140
fetal kidney 86	0.7455789	0.83936
fetal spinal cord 236	0.7923664	0.86348
fetal brain 33	0.7936968	0.87260
fetal brain 32	0.8087138	0.87480
fetal spinal cord 238	0.7850804	0.87734
ES cells 294	0.7394996	0.88268
fetal spinal cord 237	0.7793234	0.88682
fetal spinal cord 239	0.7713061	0.89582
fetal brain 36	0.7346767	0.94662
fetal brain 35	0.7434802	0.95788
fetal brain 38	0.6996175	0.96494
fetal brain 40	0.7208896	0.96592
fetal brain 37	0.7048523	0.96882
fetal brain 41	0.6845453	0.97538
fetal brain 39	0.6943274	0.97556
fetal brain 29	0.7031829	0.98624
fetal brain 34	0.6710599	0.98780
fetal brain 31	0.6770124	0.99266
fetal brain 30	0.6369137	0.99690

Central corneal thickness and keratoconus

DHS sample	fold enrichment	p value
fetal muscle 151	2.0430254	0.00002
fetal muscle 150	1.9370653	0.00002
fetal muscle 166	1.9231909	0.00002
fetal lung 299	1.9224130	0.00002
fetal muscle 160	1.9204241	0.00002
fetal lung 300	1.9041442	0.00002
fetal muscle 148	1.8970224	0.00002
fetal muscle 167	1.8658727	0.00002
fetal muscle 179	1.8584295	0.00002
fibroblast 329	1.7506342	0.00002
fetal muscle 170	1.7478921	0.00002
fetal muscle 190	1.9717205	0.00004
fetal lung 298	1.9256401	0.00004
fetal muscle 181	1.8978052	0.00004
fetal muscle 180	1.8509353	0.00004
fetal muscle 161	1.8411289	0.00004
fetal muscle 152	1.8031820	0.00004
fetal muscle 183	1.7959940	0.00004
fibroblast 332	1.7477204	0.00004
fetal fibroblast 277	1.6904363	0.00004
fetal muscle 177	1.8399682	0.00006
fetal muscle 164	1.8084856	0.00006
fetal muscle 176	1.7572962	0.00006
fetal fibroblast 271	1.7177006	0.00006
fetal fibroblast 276	1.6404697	0.00006
fetal lung 301	1.8933471	0.00008
fetal muscle 149	1.8868574	0.00008
fetal muscle 154	1.8587721	0.00008
fetal muscle 169	1.8566057	0.00008
fetal muscle 186	1.8131499	0.00008
fetal muscle 156	1.7942545	0.00008
fetal muscle 188	1.7643985	0.00008
fibroblast 330	1.7317206	0.00008
fetal muscle 153	1.8528545	0.00010
fibroblast 331	1.7379052	0.00010
fetal muscle 157	1.7107440	0.00010
fetal muscle 184	1.6768603	0.00010
fetal fibroblast 274	1.6404657	0.00010
fetal muscle 178	1.7864319	0.00012
fetal muscle 175	1.7752833	0.00012
fetal muscle 171	1.7510687	0.00012
fetal fibroblast 272	1.6528804	0.00012
fetal fibroblast 279	1.6361651	0.00012
fetal muscle 147	1.8386353	0.00014
fetal muscle 192	1.7298654	0.00014
fetal muscle 146	1.7236543	0.00014
fetal muscle 143	1.6652989	0.00014
fetal muscle 155	1.8175777	0.00016
fetal muscle 182	1.7041765	0.00016
fetal muscle 172	1.6481863	0.00016
fetal muscle 163	1.8268135	0.00018
fetal muscle 185	1.7919696	0.00018
fetal lung 117	1.6526338	0.00018
fetal lung 118	1.6115445	0.00018
fetal fibroblast 275	1.5996973	0.00018
fetal muscle 189	1.7236268	0.00020
muscle 341	2.0027135	0.00022
fetal muscle 144	1.7736405	0.00022
fetal muscle 158	1.7244728	0.00022
fetal lung 133	1.6741930	0.00022
fetal fibroblast 269	1.5889731	0.00022
ES derived MSC 290	1.7418924	0.00026
fetal lung 119	1.5923209	0.00026
fetal lung 111	1.6695535	0.00028
fetal muscle 162	1.6686651	0.00028
fetal fibroblast 268	1.5843380	0.00028
fetal lung 132	1.6682549	0.00030
fetal lung 120	1.6788951	0.00032
fetal muscle 165	1.6461457	0.00032
fetal fibroblast 278	1.6166061	0.00032
fetal lung 141	1.5976989	0.00034
fetal muscle 173	1.7353943	0.00036
fetal lung 140	1.6136846	0.00036
fetal fibroblast 273	1.5741684	0.00038
fetal lung 136	1.6867833	0.00040
fetal lung 130	1.6725511	0.00040
fetal lung 129	1.6369044	0.00040
fetal lung 128	1.5844129	0.00040
fetal muscle 145	1.8166697	0.00044
fetal lung 115	1.6117105	0.00044
fetal lung 121	1.5853297	0.00046
fetal muscle 159	1.7417323	0.00048
fetal lung 124	1.6718195	0.00048
fetal muscle 187	1.6601966	0.00048
fetal lung 126	1.5760748	0.00050
fetal fibroblast 270	1.5720299	0.00050
fetal fibroblast 280	1.5781764	0.00056
fetal fibroblast 282	1.5442116	0.00056
heart 297	1.7963500	0.00060
fetal muscle 191	1.6787057	0.00060
fetal muscle 174	1.6773970	0.00062
fetal muscle 193	1.6959522	0.00064
fetal lung 138	1.6109505	0.00068
ES derived MSC 289	1.7071164	0.00070
fetal lung 116	1.5464851	0.00072
keratinocyte 335	1.5030562	0.00074
fetal muscle 194	1.6208892	0.00078
fetal fibroblast 267	1.5983593	0.00078
breast 2	1.5392562	0.00078
fetal fibroblast 281	1.5550774	0.00084
fetal lung 139	1.6296979	0.00092
fetal lung 110	1.6086578	0.00094
breast 1	1.5125666	0.00112
fetal lung 125	1.6054703	0.00114
keratinocyte 336	1.4670011	0.00132
fetal muscle 168	1.6471017	0.00136
placenta 201	1.7248569	0.00140
fetal lung 135	1.5957083	0.00152
fetal testes 255	1.5659453	0.00170
fetal lung 131	1.4745702	0.00176
fetal lung 134	1.5715708	0.00182
fetal lung 123	1.5646475	0.00182
fetal kidney 81	1.5502016	0.00182
fetal lung 113	1.5111682	0.00182
fetal thymus 263	1.6258922	0.00184
fetal lung 137	1.5172057	0.00184
keratinocyte 333	1.4258226	0.00228
Mobilized CD8 325	1.8742194	0.00236
fetal lung 122	1.5887665	0.00238
keratinocyte 334	1.4165103	0.00262
fetal heart 52	1.5353477	0.00264
fetal lung 114	1.4644059	0.00276
fetal testes 254	1.6167816	0.00280
pancreas 327	1.7044422	0.00302
fetal heart 50	1.5590306	0.00414
fetal heart 51	1.5428952	0.00438
fetal heart 48	1.5422103	0.00438
fetal lung 142	1.4469748	0.00456
ovary 326	1.7416957	0.00466
CD19 5	1.7590465	0.00474
fetal heart 45	1.5412928	0.00480
fetal heart 43	1.5077316	0.00480
fetal stomach 251	1.6069960	0.00488
placenta 196	1.5853667	0.00498
fetal stomach 246	1.6732988	0.00518
placenta 197	1.5957330	0.00534
fetal lung 127	1.4921052	0.00546
fetal stomach 249	1.6580252	0.00564
fetal large intestine 62	1.6213468	0.00584
fetal large intestine 64	1.5375943	0.00602
CD19 6	1.7589668	0.00610
hematopoietic stem cell 317	1.4515749	0.00624
placenta 198	1.5308136	0.00626
fetal small intestine 79	1.6102716	0.00638
hematopoietic stem cell 313	1.4858496	0.00642
placenta 200	1.5028830	0.00660
placenta 199	1.5220605	0.00662
fetal stomach 242	1.7377326	0.00676
fetal large intestine 66	1.6006870	0.00706
fetal lung 108	1.4882356	0.00718
fetal heart 46	1.5341621	0.00726
fetal large intestine 54	1.5513158	0.00746
fetal small intestine 73	1.5593504	0.00754
hematopoietic stem cell 309	1.5039740	0.00760
fetal small intestine 74	1.5386359	0.00766
hematopoietic stem cell 316	1.5272045	0.00782
fetal lung 112	1.4515716	0.00798
Mobilized CD56 324	1.6708469	0.00822
fetal stomach 241	1.6072619	0.00862
hematopoietic stem cell 318	1.5218746	0.00866
CD3 10	1.6562368	0.00886
fetal heart 49	1.4977125	0.00894
CD19 7	1.7220405	0.00910
stomach 283	1.5907636	0.00920
fetal lung 109	1.3879403	0.00920
fetal stomach 245	1.5659694	0.00940
fetal large intestine 59	1.5432598	0.00946
fetal heart 42	1.4961026	0.00948
hematopoietic stem cell 311	1.4666075	0.00950
fetal small intestine 76	1.5618325	0.00980
fetal large intestine 55	1.4863721	0.01016
fetal renal pelvis 216	1.5262052	0.01026
hematopoietic stem cell 315	1.5013603	0.01060
fetal small intestine 72	1.5131679	0.01064
fetal heart 47	1.5269137	0.01084
fetal small intestine 69	1.5522202	0.01100
hematopoietic stem cell 312	1.4620598	0.01124
pancreas 328	1.5383318	0.01134
fetal stomach 250	1.5815989	0.01150
Mobilized CD4 323	1.6804184	0.01158
hematopoietic stem cell 306	1.4576673	0.01158
fetal large intestine 65	1.5270964	0.01180
fetal large intestine 60	1.4955649	0.01216
CD56 17	1.5828210	0.01222
fetal small intestine 70	1.4319765	0.01234
CD14 4	1.5859260	0.01280
fetal stomach 253	1.5647605	0.01314
fetal stomach 252	1.5162220	0.01352
fetal large intestine 63	1.5214277	0.01418
fetal small intestine 68	1.4605568	0.01458
CD4 16	1.6676922	0.01488
small intestine 342	1.4777543	0.01512
fetal stomach 243	1.4735329	0.01514
ES derived trophoblast 288	1.4028439	0.01540
hematopoietic stem cell 307	1.4337668	0.01614
Mobilized CD3 321	1.6800342	0.01648
fetal ovary 195	1.5595535	0.01670
fetal small intestine 78	1.4242110	0.01726
fetal large intestine 57	1.4366758	0.01756
CD4 15	1.7578934	0.01818
fetal large intestine 56	1.4639641	0.01820
fetal large intestine 67	1.4633203	0.01822
fetal stomach 247	1.5966539	0.01836
CD8 19	1.7089484	0.01932
stomach 284	1.4885039	0.01952
CD20 8	1.4206445	0.01952
fetal heart 44	1.5307025	0.02008
fetal stomach 240	1.5213790	0.02044
fetal stomach 248	1.4929468	0.02066
fetal large intestine 53	1.5206842	0.02068
hematopoietic stem cell 319	1.4451592	0.02094
CD4 13	1.6256245	0.02114
fetal adrenal gland 27	1.4180169	0.02134
fetal small intestine 75	1.6933559	0.02238
CD8 21	1.6220492	0.02328
fetal renal cortex 211	1.4285052	0.02516
hematopoietic stem cell 310	1.3569114	0.02554
hematopoietic stem cell 314	1.3734280	0.02626
CD56 18	1.6133880	0.02672
fetal small intestine 77	1.4363127	0.02686
CD14 3	1.5281349	0.02698
fetal kidney 84	1.4221197	0.02718
fetal adrenal gland 26	1.3783785	0.02726
CD3 12	1.5876581	0.02766
fetal large intestine 58	1.3872301	0.02804
fetal renal cortex 214	1.3938203	0.02840
fetal adrenal gland 24	1.4355395	0.02858
fetal adrenal gland 28	1.3444850	0.02932
fetal kidney 88	1.3762280	0.02942
fetal renal pelvis 231	1.3710178	0.02992
fetal kidney 83	1.4520996	0.03000
fetal small intestine 80	1.3940981	0.03024
fetal renal pelvis 228	1.4222846	0.03046
fetal renal pelvis 218	1.4829558	0.03064
fetal renal pelvis 230	1.4242605	0.03070
fetal kidney 87	1.3743260	0.03116
hematopoietic stem cell 308	1.3552203	0.03126
fetal small intestine 71	1.4055297	0.03130
CD4 14	1.4952544	0.03150
hematopoietic stem cell 305	1.3801168	0.03184
CD8 22	1.5449534	0.03238
Mobilized CD4 322	1.5623230	0.03332
fetal renal pelvis 226	1.3767528	0.03344
fetal renal pelvis 232	1.4049288	0.03354
fetal renal pelvis 219	1.4833512	0.03396
fetal renal cortex 210	1.3970117	0.03404
fetal kidney 86	1.4650747	0.03414
fetal kidney 99	1.3762536	0.03430
fetal renal cortex 202	1.3547301	0.03434
CD8 20	1.4991314	0.03550
fetal thymus 262	1.5881893	0.03580
fetal kidney 89	1.3813060	0.03602
fetal large intestine 61	1.4773231	0.03676
fetal kidney 97	1.3572378	0.03678
fetal kidney 102	1.3910766	0.03682
fetal kidney 94	1.3860761	0.03878
fetal kidney 101	1.3535218	0.03990
fetal renal cortex 203	1.3592699	0.04086
fetal stomach 244	1.3933107	0.04134
fetal kidney 100	1.3461672	0.04174
fetal renal cortex 208	1.3630449	0.04280
fetal kidney 105	1.3345281	0.04344
ES derived trophoblast 287	1.3433208	0.04572
fetal renal pelvis 233	1.3435293	0.04684
fetal kidney 93	1.3606930	0.04720
fetal renal pelvis 229	1.3391437	0.04808
fetal kidney 107	1.3337727	0.04860
fetal renal pelvis 222	1.3471754	0.04928
fetal kidney 96	1.3265988	0.04944
CD3 9	1.4656411	0.04978
fetal kidney 104	1.3406662	0.04988
fetal renal cortex 204	1.3088458	0.05056
fetal renal pelvis 217	1.3488905	0.05070
fetal renal pelvis 223	1.3566163	0.05086
fetal renal pelvis 225	1.3397149	0.05158
fetal renal pelvis 224	1.3110791	0.05162
fetal kidney 82	1.3658139	0.05176
fetal adrenal gland 25	1.3450105	0.05242
fetal renal cortex 206	1.3109506	0.05326
fetal kidney 90	1.3282947	0.05330
Mobilized CD3 320	1.5008297	0.05370
fetal kidney 95	1.3323996	0.05412
fetal renal pelvis 221	1.3134350	0.05412
fetal renal pelvis 227	1.3264408	0.05490
fetal renal cortex 209	1.3244610	0.05702
melanocyte 338	1.2271891	0.05708
fetal renal cortex 215	1.2999771	0.05890
CD3 11	1.4188413	0.05966
fetal renal cortex 212	1.2945377	0.06076
fetal kidney 92	1.2982445	0.06472
fetal adrenal gland 23	1.2730034	0.06592
fetal renal cortex 205	1.2936784	0.07026
fetal kidney 98	1.2915892	0.07484
fetal kidney 103	1.2881085	0.07726
fetal thymus 257	1.3840265	0.07738
fetal renal pelvis 220	1.2776281	0.08510
fetal renal cortex 213	1.2458544	0.09152
fetal kidney 106	1.2534466	0.09340
melanocyte 337	1.1796577	0.10054
fetal renal cortex 207	1.2412225	0.10896
fetal kidney 91	1.2195682	0.11178
melanocyte 339	1.1617043	0.13024
fetal thymus 261	1.2802212	0.13922
fetal skin 234	1.2034638	0.13976
fetal kidney 85	1.1829953	0.14060
ES derived NP 292	1.2144592	0.14494
fetal spinal cord 235	1.1776151	0.14988
fetal thymus 265	1.2512791	0.15732
fetal thymus 266	1.2413413	0.16494
fetal spinal cord 236	1.1676765	0.16890
fetal thymus 258	1.2293631	0.17306
ES derived NP 291	1.1938399	0.17556
fetal thymus 259	1.2212198	0.18980
fetal thymus 256	1.2054608	0.19590
fetal thymus 260	1.2016683	0.20466
fetal thymus 264	1.1824188	0.22368
melanocyte 340	1.1069334	0.23608
ES cells 293	1.0964578	0.28856
ES cells 295	1.0980952	0.29220
fetal spinal cord 237	1.0820051	0.29698
ES derived mesoderm 286	1.0662257	0.30586
iPS 304	1.0710911	0.31516
iPS 302	1.0713143	0.32446
ES cells 294	1.0692843	0.33750
ES derived mesoderm 285	1.0568501	0.33812
fetal spinal cord 238	1.0620973	0.33862
ES cells 296	1.0687585	0.34436
fetal spinal cord 239	1.0548609	0.35872
fetal brain 33	1.0041602	0.47522
iPS 303	1.0019019	0.47640
fetal brain 31	0.9828593	0.55038
fetal brain 41	0.9522413	0.62720
fetal brain 39	0.9437161	0.65764
fetal brain 34	0.9407042	0.66748
fetal brain 32	0.9332816	0.66820
fetal brain 37	0.9264186	0.70588
fetal brain 30	0.9337793	0.71742
fetal brain 40	0.9246276	0.71990
fetal brain 38	0.9029527	0.74786
fetal brain 36	0.8996608	0.75198
fetal brain 29	0.9109648	0.78088
fetal brain 35	0.8908872	0.80734

Macular degeneration

DHS sample	fold enrichment	p value
fetal muscle 176	1.7007913	0.00030
fetal fibroblast 282	1.5582931	0.00030
fetal fibroblast 274	1.5689137	0.00032
fetal fibroblast 278	1.5737790	0.00038
fetal fibroblast 277	1.5832393	0.00040
fetal fibroblast 272	1.5719382	0.00040
fetal fibroblast 281	1.5765631	0.00042
fetal fibroblast 268	1.5729272	0.00046
fetal fibroblast 279	1.5523450	0.00054
fetal fibroblast 275	1.5417107	0.00054
fetal fibroblast 273	1.5606246	0.00060
fetal fibroblast 270	1.5501007	0.00066
fetal fibroblast 280	1.5390032	0.00066
fetal fibroblast 276	1.5348563	0.00084
fetal fibroblast 271	1.5280514	0.00100
fetal fibroblast 269	1.4920240	0.00130
stomach 284	1.6843011	0.00254
fetal muscle 143	1.5522126	0.00260
fetal muscle 151	1.6597842	0.00308
fetal lung 300	1.4910149	0.00314
fetal lung 128	1.4419797	0.00320
fetal muscle 181	1.5866977	0.00326
fetal fibroblast 267	1.4889211	0.00344
fetal lung 111	1.4777635	0.00362
fetal muscle 172	1.4798760	0.00366
fetal muscle 157	1.5002821	0.00460
fetal muscle 167	1.5112725	0.00500
fetal muscle 180	1.5760737	0.00506
fetal muscle 179	1.5508948	0.00526
melanocyte 338	1.3885315	0.00536
ES derived MSC 290	1.5169793	0.00540
ES derived MSC 289	1.5106589	0.00574
fetal lung 298	1.4653371	0.00602
fetal muscle 165	1.4969224	0.00606
fetal large intestine 67	1.5577705	0.00622
breast 2	1.4074543	0.00672
fetal muscle 166	1.5087637	0.00746
fetal muscle 169	1.5054805	0.00816
melanocyte 337	1.3570100	0.00820
fetal muscle 164	1.5309778	0.00830
fetal lung 134	1.4616946	0.00842
fetal muscle 149	1.5539423	0.00844
fetal lung 299	1.4472876	0.00850
fetal muscle 168	1.5301380	0.00868
fetal small intestine 70	1.4567557	0.00902
fetal muscle 148	1.5071925	0.00920
fetal muscle 153	1.5434064	0.00994
fetal muscle 184	1.4447332	0.01036
fetal muscle 187	1.4522638	0.01066
fetal lung 141	1.3992972	0.01074
fetal lung 301	1.4229324	0.01082
fetal lung 114	1.3651209	0.01182
fetal muscle 152	1.4478024	0.01248
pancreas 328	1.5449939	0.01294
keratinocyte 336	1.3369419	0.01302
fetal lung 129	1.4068600	0.01412
fetal lung 119	1.3773694	0.01430
melanocyte 339	1.3441510	0.01438
fetal muscle 182	1.4445239	0.01456
keratinocyte 334	1.3257617	0.01462
keratinocyte 333	1.3201336	0.01464
fetal muscle 178	1.4676362	0.01542
fetal stomach 243	1.4778922	0.01578
fibroblast 331	1.3504144	0.01634
fetal muscle 150	1.4633027	0.01646
fetal muscle 183	1.4213023	0.01646
fetal muscle 161	1.4565857	0.01660
fetal kidney 81	1.4080345	0.01670
fetal skin 234	1.4560525	0.01674
CD20 8	1.4405251	0.01686
breast 1	1.3509895	0.01722
ES derived trophoblast 287	1.4419603	0.01732
fetal large intestine 58	1.4188478	0.01740
fetal muscle 154	1.4751176	0.01790
fetal small intestine 68	1.4412204	0.01844
fetal lung 121	1.3549013	0.01850
fetal muscle 189	1.4293054	0.01852
fetal small intestine 80	1.4319620	0.01868
fetal stomach 244	1.5043446	0.01892
fetal large intestine 55	1.4311344	0.02000
fibroblast 332	1.3364860	0.02004
fetal muscle 155	1.4843951	0.02018
fetal muscle 188	1.4148931	0.02018
fetal muscle 162	1.3996538	0.02028
fetal large intestine 57	1.4098787	0.02050
fetal muscle 174	1.4274737	0.02116
pancreas 327	1.4980129	0.02150
keratinocyte 335	1.3063090	0.02172
fetal muscle 192	1.4101114	0.02254
fetal muscle 175	1.4121197	0.02278
stomach 283	1.4838087	0.02286
fetal large intestine 64	1.4219915	0.02286
fetal muscle 186	1.4007813	0.02294
fetal muscle 159	1.4468871	0.02348
fetal small intestine 78	1.3915738	0.02378
fetal muscle 173	1.4214186	0.02380
fetal thymus 263	1.4259550	0.02386
fetal muscle 146	1.3911505	0.02434
melanocyte 340	1.3234274	0.02506
fetal muscle 163	1.4271477	0.02574
fetal lung 140	1.3471428	0.02602
fetal muscle 147	1.4060775	0.02640
fetal lung 109	1.3121501	0.02642
fetal muscle 185	1.4212582	0.02658
fetal lung 137	1.3449309	0.02698
ES derived trophoblast 288	1.3630230	0.02720
fetal muscle 191	1.3980761	0.02752
fetal lung 126	1.3227152	0.02856
fetal muscle 160	1.4053239	0.03066
fetal lung 116	1.3002796	0.03086
fetal lung 133	1.3225773	0.03208
fetal muscle 156	1.3741543	0.03244
fibroblast 329	1.3079816	0.03356
fetal small intestine 77	1.4174985	0.03400
fetal muscle 171	1.3618158	0.03434
fetal lung 131	1.2981207	0.03438
fibroblast 330	1.3101998	0.03464
fetal large intestine 56	1.3881936	0.03596
fetal adrenal gland 25	1.3787324	0.03616
heart 297	1.3929174	0.03626
fetal large intestine 60	1.4028373	0.03650
fetal lung 120	1.3267480	0.03712
fetal muscle 158	1.3444631	0.03898
fetal muscle 193	1.3808883	0.03910
fetal lung 125	1.3376749	0.03938
fetal stomach 248	1.4349317	0.03948
muscle 341	1.4023549	0.03952
fetal large intestine 63	1.4070241	0.03982
fetal small intestine 74	1.3772346	0.04026
fetal lung 113	1.2976869	0.04040
ovary 326	1.4422178	0.04280
fetal lung 118	1.2850783	0.04302
fetal lung 117	1.3009246	0.04354
fetal stomach 251	1.4029720	0.04488
fetal large intestine 62	1.3982979	0.04538
fetal small intestine 76	1.3919729	0.04548
fetal muscle 194	1.3060307	0.04586
fetal lung 142	1.2901420	0.04668
fetal small intestine 73	1.3714564	0.04774
fetal muscle 177	1.3667349	0.04842
fetal muscle 170	1.3178181	0.04888
fetal lung 110	1.3119176	0.05018
fetal thymus 256	1.3627437	0.05098
fetal large intestine 59	1.3803047	0.05106
fetal large intestine 66	1.3720744	0.05336
fetal lung 132	1.2962063	0.05348
fetal muscle 144	1.3431331	0.05422
fetal large intestine 53	1.4094543	0.05484
ES derived mesoderm 285	1.2778413	0.05484
fetal small intestine 72	1.3441583	0.05780
fetal muscle 190	1.3503608	0.05810
fetal large intestine 54	1.3444229	0.06214
CD14 3	1.3715324	0.06274
fetal lung 136	1.2810572	0.06304
fetal adrenal gland 24	1.3388102	0.06440
fetal small intestine 71	1.3392449	0.06524
fetal small intestine 79	1.3479850	0.06570
fetal lung 108	1.2942254	0.06606
fetal stomach 252	1.3576128	0.06718
fetal lung 115	1.2556579	0.06848
fetal renal pelvis 226	1.3238470	0.06860
fetal adrenal gland 28	1.2585931	0.06922
fetal muscle 145	1.3376704	0.06968
fetal kidney 101	1.3154211	0.07122
CD56 18	1.3852911	0.07136
fetal large intestine 65	1.3214902	0.07204
placenta 199	1.2936969	0.07266
fetal adrenal gland 23	1.2601966	0.07790
CD14 4	1.3204734	0.07800
placenta 200	1.2646916	0.07808
fetal heart 47	1.3049123	0.07812
fetal stomach 253	1.3449671	0.07986
fetal thymus 265	1.3220472	0.08006
fetal stomach 245	1.3273693	0.08040
fetal kidney 85	1.2901946	0.08154
fetal lung 127	1.2600464	0.08160
fetal stomach 246	1.3447120	0.08234
fetal lung 139	1.2663470	0.08246
fetal renal pelvis 231	1.2941839	0.08318
fetal lung 138	1.2557708	0.08354
small intestine 342	1.2931676	0.08426
fetal lung 122	1.2654824	0.08428
fetal renal cortex 213	1.2887530	0.08462
fetal kidney 106	1.3035545	0.08574
fetal lung 130	1.2437300	0.08616
fetal adrenal gland 27	1.2678257	0.08726
fetal stomach 250	1.3353580	0.08832
fetal kidney 91	1.2850392	0.09054
fetal heart 52	1.2448705	0.09092
fetal renal pelvis 220	1.3034521	0.09174
fetal testes 255	1.2546533	0.09224
fetal thymus 258	1.3014955	0.09312
fetal small intestine 69	1.3061267	0.09444
placenta 197	1.2814453	0.09534
fetal stomach 241	1.3155369	0.09862
fetal renal cortex 207	1.2835618	0.09912
placenta 196	1.2762865	0.10020
fetal renal pelvis 216	1.2779751	0.10150
fetal kidney 89	1.2834961	0.10374
fetal kidney 99	1.2794830	0.10540
CD56 17	1.2742221	0.10680
fetal renal cortex 205	1.2629892	0.10834
fetal renal cortex 204	1.2384715	0.10992
placenta 201	1.2645341	0.11260
fetal renal cortex 212	1.2447227	0.11356
fetal stomach 240	1.3035019	0.11398
fetal renal pelvis 217	1.2738893	0.11408
fetal stomach 247	1.3227031	0.11464
fetal heart 43	1.2210123	0.11464
fetal heart 51	1.2254330	0.11474
fetal kidney 87	1.2507937	0.11748
Mobilized CD56 324	1.2771476	0.11974
fetal renal cortex 206	1.2392018	0.12250
fetal renal pelvis 233	1.2538812	0.12352
fetal renal cortex 215	1.2351374	0.12498
fetal heart 48	1.2190232	0.12536
fetal heart 50	1.2238893	0.12554
fetal lung 112	1.2057544	0.12732
fetal heart 46	1.2267198	0.12790
fetal thymus 260	1.2597410	0.12832
fetal heart 49	1.2167802	0.12884
fetal renal pelvis 228	1.2628548	0.12902
fetal kidney 100	1.2434882	0.13346
fetal lung 135	1.2002382	0.13574
CD3 11	1.2548890	0.13658
fetal thymus 266	1.2436716	0.13850
fetal kidney 88	1.2239706	0.13860
fetal heart 45	1.2112019	0.13902
fetal brain 37	1.1631829	0.13992
fetal renal pelvis 229	1.2260479	0.14228
fetal renal cortex 211	1.2432471	0.14288
fetal lung 124	1.1927709	0.14306
placenta 198	1.1971949	0.14410
fetal heart 42	1.2005537	0.14506
fetal brain 30	1.1274942	0.14566
fetal lung 123	1.1896627	0.14732
fetal kidney 95	1.2274525	0.14872
CD19 5	1.2515862	0.14978
fetal kidney 107	1.2174374	0.15160
fetal kidney 105	1.2073849	0.15312
fetal kidney 92	1.2102613	0.15330
fetal renal pelvis 227	1.2195920	0.15494
fetal brain 35	1.1330507	0.15560
fetal kidney 84	1.2236563	0.15668
fetal renal pelvis 221	1.2053797	0.15790
CD4 13	1.2569931	0.15838
fetal adrenal gland 26	1.1904558	0.16510
CD8 20	1.2200627	0.16880
Mobilized CD4 323	1.2425635	0.16898
fetal kidney 96	1.1945768	0.17026
fetal brain 34	1.1311350	0.17078
fetal renal pelvis 232	1.2092443	0.17238
fetal renal pelvis 224	1.1843959	0.17362
fetal renal cortex 208	1.1980594	0.17614
fetal kidney 104	1.1952040	0.17820
CD3 10	1.2176672	0.17862
CD8 19	1.2496393	0.17888
fetal renal pelvis 225	1.1960480	0.18090
fetal brain 36	1.1396702	0.18228
fetal renal pelvis 223	1.1960120	0.18792
CD19 6	1.2237833	0.18870
fetal kidney 93	1.1866155	0.19080
Mobilized CD8 325	1.2267146	0.19132
fetal kidney 82	1.1979407	0.19266
fetal stomach 242	1.2247994	0.19536
fetal stomach 249	1.2039741	0.19578
hematopoietic stem cell 312	1.1601294	0.19666
fetal renal cortex 203	1.1719475	0.20090
fetal kidney 90	1.1715230	0.20296
fetal renal pelvis 219	1.2013987	0.20600
CD4 14	1.1849070	0.20642
CD19 7	1.2060210	0.20820
fetal thymus 264	1.1736614	0.20964
hematopoietic stem cell 314	1.1456193	0.21304
fetal renal cortex 214	1.1617612	0.21388
ES derived mesoderm 286	1.1220961	0.21488
fetal brain 40	1.1144045	0.21522
fetal kidney 98	1.1642423	0.21668
CD3 12	1.1971773	0.21746
fetal renal pelvis 218	1.1884008	0.21892
fetal renal pelvis 222	1.1631700	0.21994
CD8 22	1.1901933	0.22020
fetal renal pelvis 230	1.1728970	0.22146
hematopoietic stem cell 315	1.1484671	0.22292
fetal heart 44	1.1666296	0.22734
fetal kidney 83	1.1736035	0.22790
fetal kidney 97	1.1507719	0.22938
hematopoietic stem cell 310	1.1247206	0.23084
fetal thymus 257	1.1728885	0.23096
fetal kidney 94	1.1576250	0.23142
CD4 16	1.1867067	0.23166
CD8 21	1.1785724	0.23612
Mobilized CD3 321	1.1764191	0.24248
fetal renal cortex 202	1.1377032	0.24258
fetal kidney 86	1.1661554	0.24292
CD3 9	1.1653174	0.24482
fetal spinal cord 235	1.1196201	0.24738
fetal brain 39	1.0960324	0.24876
fetal thymus 259	1.1543702	0.24966
hematopoietic stem cell 309	1.1261209	0.25266
fetal spinal cord 237	1.1142931	0.25288
fetal renal cortex 210	1.1386284	0.25412
fetal testes 254	1.1282802	0.25746
fetal kidney 102	1.1386253	0.26052
fetal thymus 262	1.1629596	0.26186
fetal brain 29	1.0782957	0.26314
ES derived NP 291	1.1357237	0.26370
fetal brain 38	1.0967689	0.26510
hematopoietic stem cell 308	1.1132193	0.26538
fetal renal cortex 209	1.1235969	0.26836
iPS 303	1.1077740	0.27432
fetal thymus 261	1.1319406	0.27500
Mobilized CD4 322	1.1464410	0.27586
fetal kidney 103	1.1195088	0.27684
fetal large intestine 61	1.1338772	0.27762
CD4 15	1.1544099	0.28226
hematopoietic stem cell 305	1.1036319	0.28952
iPS 302	1.0962276	0.29726
fetal spinal cord 236	1.0916941	0.29992
ES cells 296	1.1071454	0.30182
hematopoietic stem cell 318	1.0984528	0.30614
fetal spinal cord 238	1.0859788	0.30688
fetal brain 31	1.0619609	0.30788
hematopoietic stem cell 316	1.0944575	0.31246
fetal ovary 195	1.1024058	0.31832
fetal brain 41	1.0665439	0.32438
iPS 304	1.0748636	0.33024
ES derived NP 292	1.0800697	0.33960
fetal small intestine 75	1.0916966	0.34318
hematopoietic stem cell 313	1.0666664	0.34694
hematopoietic stem cell 306	1.0618330	0.36328
Mobilized CD3 320	1.0806798	0.36700
ES cells 293	1.0535767	0.38112
ES cells 294	1.0523035	0.38740
hematopoietic stem cell 311	1.0413565	0.40100
fetal spinal cord 239	1.0298086	0.41784
hematopoietic stem cell 319	1.0254390	0.43310
fetal brain 32	1.0179419	0.44116
hematopoietic stem cell 307	1.0156054	0.45158
fetal brain 33	0.9963300	0.49798
hematopoietic stem cell 317	0.9926870	0.50618
ES cells 295	0.9793796	0.51836

Breast cancer risk

DHS sample	fold enrichment	p value
keratinocyte 334	1.3376997	0.00018
keratinocyte 335	1.3419331	0.00030
fetal small intestine 68	1.4573338	0.00050
keratinocyte 336	1.3187696	0.00052
stomach 283	1.5436032	0.00058
stomach 284	1.5057020	0.00058
breast 2	1.3293447	0.00070
keratinocyte 333	1.3066667	0.00070
fetal large intestine 67	1.4840321	0.00074
breast 1	1.3346045	0.00094
CD14 3	1.5631372	0.00128
fetal small intestine 70	1.3949009	0.00136
Mobilized CD3 320	1.6259359	0.00156
fetal large intestine 57	1.4109141	0.00176
fetal small intestine 80	1.4296138	0.00180
CD14 4	1.5012711	0.00194
fetal lung 114	1.3022237	0.00196
fetal stomach 248	1.4697932	0.00200
fetal large intestine 55	1.4102099	0.00202
fetal lung 109	1.2957719	0.00226
fetal lung 128	1.2902987	0.00246
fetal small intestine 77	1.4264768	0.00248
fetal lung 111	1.3179361	0.00248
fetal large intestine 58	1.3846105	0.00250
fetal large intestine 60	1.4203670	0.00260
fetal muscle 148	1.3574726	0.00276
fetal large intestine 64	1.3945787	0.00292
fetal lung 131	1.2807129	0.00298
fetal muscle 152	1.3308700	0.00324
fetal lung 108	1.3453067	0.00330
fetal lung 141	1.2922407	0.00334
fetal small intestine 78	1.3630245	0.00342
fetal small intestine 74	1.3979447	0.00354
fetal kidney 85	1.3190746	0.00358
fetal large intestine 56	1.4023205	0.00384
fetal muscle 176	1.3164487	0.00404
fetal lung 125	1.3166176	0.00416
fetal muscle 156	1.3231577	0.00422
CD20 8	1.3765822	0.00426
fetal lung 133	1.2816409	0.00438
fetal muscle 169	1.3320905	0.00440
fetal small intestine 73	1.4007921	0.00464
CD4 14	1.4539359	0.00466
muscle 341	1.4353306	0.00482
fetal lung 116	1.2656654	0.00488
fetal muscle 168	1.3386173	0.00490
fetal small intestine 76	1.4215870	0.00506
fetal muscle 155	1.3521071	0.00508
fetal muscle 190	1.3508633	0.00510
melanocyte 339	1.2542126	0.00520
fetal lung 142	1.2765942	0.00524
fetal large intestine 63	1.4131388	0.00530
fetal lung 137	1.2895263	0.00546
fetal muscle 183	1.3032513	0.00560
fetal stomach 240	1.4341319	0.00564
fetal muscle 151	1.3496662	0.00564
fetal muscle 164	1.3258568	0.00564
fetal muscle 146	1.3214239	0.00564
fibroblast 330	1.2915320	0.00570
fetal muscle 184	1.2941071	0.00594
fetal muscle 143	1.2891487	0.00596
fetal muscle 147	1.3164966	0.00598
fetal lung 110	1.3011946	0.00598
CD4 13	1.5063561	0.00602
fetal small intestine 72	1.3805914	0.00616
fetal lung 138	1.2990725	0.00616
Mobilized CD56 324	1.4438083	0.00624
fetal muscle 188	1.3070106	0.00626
fetal muscle 157	1.2943693	0.00628
fetal lung 119	1.2647932	0.00628
fetal muscle 149	1.3262209	0.00634
fetal muscle 178	1.3216187	0.00634
fetal muscle 181	1.3255132	0.00642
fetal muscle 167	1.3027980	0.00644
fetal lung 113	1.2683399	0.00646
fetal muscle 161	1.3190629	0.00662
fibroblast 329	1.2791722	0.00680
fetal muscle 171	1.2908074	0.00682
fetal large intestine 62	1.4014162	0.00688
fetal large intestine 54	1.3865614	0.00696
fetal muscle 177	1.3300730	0.00706
fetal stomach 244	1.3679340	0.00726
fetal large intestine 66	1.3873312	0.00738
fetal fibroblast 277	1.2622576	0.00744
melanocyte 340	1.2566340	0.00746
fetal lung 129	1.2791532	0.00756
fetal muscle 179	1.3057150	0.00760
fetal renal cortex 207	1.3247037	0.00798
fetal muscle 189	1.3000984	0.00800
fetal lung 139	1.2920886	0.00802
fetal lung 117	1.2637842	0.00802
fetal muscle 150	1.3232183	0.00816
fetal lung 122	1.3033528	0.00816
fetal muscle 192	1.2938623	0.00822
placenta 201	1.3663848	0.00826
placenta 197	1.3477599	0.00828
fetal small intestine 79	1.3870122	0.00834
CD4 16	1.4790968	0.00836
fetal lung 140	1.2673561	0.00838
fetal muscle 172	1.2644849	0.00844
fetal lung 127	1.2884483	0.00846
hematopoietic stem cell 309	1.3324629	0.00850
Mobilized CD4 322	1.4839304	0.00856
fetal muscle 160	1.3089047	0.00860
CD56 17	1.4076463	0.00864
fetal stomach 246	1.3878468	0.00872
fetal muscle 153	1.3190586	0.00904
fetal kidney 92	1.3029068	0.00914
fetal lung 135	1.2769475	0.00926
fetal muscle 186	1.2909400	0.00936
fetal large intestine 59	1.3708821	0.00940
fetal muscle 166	1.2872339	0.00940
CD8 20	1.4322619	0.00950
fetal stomach 252	1.3595075	0.00950
ES derived NP 291	1.3603733	0.00964
fetal muscle 144	1.3134240	0.00982
fetal large intestine 61	1.4118635	0.00986
fetal muscle 187	1.2764884	0.00994
fetal muscle 165	1.2696978	0.00996
fetal lung 126	1.2503128	0.01000
fetal lung 134	1.2815758	0.01016
hematopoietic stem cell 310	1.2806265	0.01016
fetal muscle 158	1.2842206	0.01020
fetal kidney 89	1.3144452	0.01028
hematopoietic stem cell 317	1.2774096	0.01072
fetal muscle 182	1.2853254	0.01090
fetal stomach 251	1.3467878	0.01092
fetal muscle 163	1.2956411	0.01092
fetal fibroblast 278	1.2490820	0.01116
fetal stomach 253	1.3787832	0.01118
fetal lung 121	1.2405192	0.01170
fetal lung 136	1.2605781	0.01178
melanocyte 337	1.2136865	0.01184
fetal muscle 174	1.2901186	0.01196
CD3 11	1.4061938	0.01200
Mobilized CD4 323	1.4429717	0.01206
fetal kidney 106	1.2906442	0.01208
fetal lung 123	1.2627079	0.01208
fetal lung 115	1.2453646	0.01214
fetal muscle 180	1.2969047	0.01226
ES derived trophoblast 288	1.2685332	0.01250
fetal muscle 162	1.2670814	0.01252
CD3 9	1.4239018	0.01258
fetal stomach 247	1.4312048	0.01264
fetal muscle 145	1.3181290	0.01266
fetal muscle 194	1.2484241	0.01270
fetal skin 234	1.2928155	0.01282
CD3 10	1.4041480	0.01314
small intestine 342	1.3250418	0.01346
CD8 22	1.4286788	0.01350
fetal muscle 173	1.2797710	0.01352
fetal large intestine 65	1.3436582	0.01354
fetal renal cortex 215	1.2797702	0.01356
hematopoietic stem cell 312	1.2975306	0.01372
fetal renal pelvis 231	1.2732885	0.01390
CD19 6	1.4228971	0.01396
fetal muscle 185	1.2915112	0.01402
fetal lung 120	1.2488590	0.01404
fetal lung 124	1.2488261	0.01426
fetal muscle 191	1.2854690	0.01428
fetal lung 118	1.2242904	0.01432
CD3 12	1.4408637	0.01434
placenta 200	1.2714964	0.01434
fetal lung 132	1.2500666	0.01448
fetal muscle 175	1.2650626	0.01494
fetal kidney 91	1.2672018	0.01508
fibroblast 332	1.2417992	0.01508
fetal renal cortex 213	1.2751805	0.01518
fetal kidney 101	1.2856024	0.01552
placenta 199	1.2808041	0.01554
fetal fibroblast 282	1.2207698	0.01558
fetal muscle 193	1.2803160	0.01574
fetal fibroblast 276	1.2175681	0.01592
fetal muscle 170	1.2513543	0.01618
fetal fibroblast 274	1.2272528	0.01628
fetal small intestine 69	1.3452952	0.01634
fetal kidney 98	1.2911819	0.01634
placenta 196	1.3030996	0.01644
fetal thymus 260	1.3817938	0.01654
fetal muscle 159	1.2846495	0.01656
fetal renal pelvis 229	1.2836653	0.01658
fetal kidney 99	1.2810107	0.01698
fetal small intestine 71	1.3180263	0.01722
fetal kidney 88	1.2694244	0.01752
fetal renal pelvis 220	1.2870812	0.01818
fetal fibroblast 281	1.2215435	0.01818
melanocyte 338	1.1999202	0.01818
fetal fibroblast 272	1.2194081	0.01826
fetal stomach 250	1.3390529	0.01832
fetal renal pelvis 226	1.2748563	0.01836
hematopoietic stem cell 318	1.3071329	0.01892
fetal fibroblast 275	1.2099562	0.01916
fetal small intestine 75	1.4567913	0.01924
fetal thymus 263	1.2658473	0.01926
fetal fibroblast 273	1.2155200	0.01940
hematopoietic stem cell 315	1.2973684	0.01942
fetal renal pelvis 233	1.2822326	0.01944
fetal lung 130	1.2354826	0.01954
fetal stomach 241	1.3412957	0.01982
fetal kidney 81	1.2480204	0.01982
fetal kidney 103	1.2759756	0.01986
fetal fibroblast 280	1.2198926	0.02004
fetal fibroblast 279	1.2232736	0.02014
fetal kidney 96	1.2611024	0.02060
fetal stomach 245	1.3215292	0.02068
ES derived MSC 290	1.2621448	0.02074
fetal fibroblast 268	1.2096540	0.02094
fetal stomach 249	1.3294280	0.02126
fetal kidney 94	1.2856092	0.02134
fetal muscle 154	1.2773377	0.02134
hematopoietic stem cell 316	1.2948283	0.02216
fetal stomach 243	1.2845485	0.02228
fetal fibroblast 267	1.2161729	0.02230
fetal renal pelvis 224	1.2508135	0.02234
fetal renal pelvis 227	1.2634553	0.02284
fetal kidney 95	1.2676630	0.02318
fibroblast 331	1.2239762	0.02318
Mobilized CD8 325	1.3878889	0.02344
hematopoietic stem cell 305	1.2718127	0.02384
hematopoietic stem cell 314	1.2558884	0.02384
Mobilized CD3 321	1.4022732	0.02410
fetal renal cortex 202	1.2510737	0.02494
fetal fibroblast 271	1.2063402	0.02524
fetal stomach 242	1.3686335	0.02534
fetal large intestine 53	1.3226541	0.02534
fetal thymus 256	1.3364742	0.02566
fetal renal cortex 204	1.2432676	0.02594
ES derived NP 292	1.2894794	0.02638
CD8 21	1.3857769	0.02642
ES derived trophoblast 287	1.2524607	0.02652
fetal renal cortex 203	1.2603711	0.02654
hematopoietic stem cell 313	1.2532900	0.02658
pancreas 327	1.3164372	0.02672
ES derived MSC 289	1.2468296	0.02792
hematopoietic stem cell 308	1.2472556	0.02822
fetal kidney 84	1.2544049	0.02868
CD19 5	1.3518740	0.02876
fetal renal cortex 214	1.2449513	0.02996
fetal renal cortex 212	1.2349116	0.03020
hematopoietic stem cell 306	1.2530623	0.03110
fetal renal pelvis 228	1.2676077	0.03134
fetal kidney 86	1.2981241	0.03158
fetal kidney 107	1.2487077	0.03190
fetal renal cortex 211	1.2651174	0.03198
fetal lung 112	1.2106751	0.03254
placenta 198	1.2332860	0.03286
CD8 19	1.3902094	0.03336
fetal kidney 83	1.2800290	0.03358
fetal kidney 87	1.2341236	0.03382
fetal kidney 104	1.2469772	0.03422
fetal renal pelvis 221	1.2294975	0.03504
fetal renal cortex 209	1.2436405	0.03518
fetal thymus 258	1.3132705	0.03560
ES derived mesoderm 285	1.1834166	0.03590
fetal renal pelvis 217	1.2482352	0.03600
fetal thymus 266	1.3137704	0.03614
fetal renal pelvis 216	1.2466732	0.03716
hematopoietic stem cell 319	1.2657685	0.03824
fetal kidney 102	1.2523255	0.03868
fetal renal pelvis 222	1.2419932	0.03966
iPS 302	1.2129126	0.04038
fetal renal pelvis 223	1.2485288	0.04140
fetal thymus 265	1.2997442	0.04202
ovary 326	1.3041332	0.04296
fetal kidney 100	1.2200747	0.04326
fetal renal cortex 208	1.2368286	0.04328
fetal thymus 261	1.3065150	0.04362
fetal renal cortex 205	1.2287002	0.04368
fetal testes 255	1.2145894	0.04520
fetal fibroblast 269	1.1771322	0.04522
fetal kidney 90	1.2311419	0.04612
fetal renal pelvis 230	1.2445322	0.04640
hematopoietic stem cell 311	1.2217471	0.04684
fetal kidney 97	1.2150643	0.04792
fetal renal pelvis 219	1.2766606	0.04794
fetal renal cortex 206	1.2111569	0.04804
CD4 15	1.3680465	0.04806
hematopoietic stem cell 307	1.2210420	0.04808
fetal fibroblast 270	1.1808233	0.04832
fetal lung 301	1.1997678	0.04834
fetal kidney 82	1.2465702	0.04858
CD56 18	1.3160219	0.05070
fetal lung 299	1.1973237	0.05104
fetal thymus 262	1.3363806	0.05110
fetal renal pelvis 232	1.2309229	0.05116
fetal kidney 105	1.2033579	0.05136
fetal kidney 93	1.2281822	0.05340
fetal renal pelvis 225	1.2174287	0.05562
fetal adrenal gland 27	1.2080582	0.05646
CD19 7	1.3060077	0.05666
fetal thymus 257	1.2814828	0.05770
fetal adrenal gland 25	1.2210798	0.05846
pancreas 328	1.2372056	0.05862
fetal thymus 264	1.2586750	0.06098
iPS 303	1.1774663	0.06672
fetal renal pelvis 218	1.2489980	0.06788
fetal adrenal gland 26	1.1912317	0.06850
iPS 304	1.1663253	0.06874
fetal adrenal gland 24	1.2182839	0.07342
fetal lung 300	1.1697814	0.07410
heart 297	1.2077668	0.07826
fetal ovary 195	1.2298260	0.07830
fetal testes 254	1.1909841	0.07872
fetal lung 298	1.1727690	0.07888
fetal renal cortex 210	1.1959675	0.08014
ES cells 293	1.1733970	0.09358
fetal adrenal gland 28	1.1428523	0.11006
fetal thymus 259	1.2031524	0.12328
ES cells 294	1.1458095	0.14408
fetal heart 42	1.1295376	0.15392
ES cells 295	1.1325358	0.16612
fetal adrenal gland 23	1.1142522	0.16856
ES cells 296	1.1277042	0.17424
fetal heart 43	1.1077115	0.18526
fetal heart 48	1.1125037	0.18896
fetal heart 52	1.0987589	0.19744
fetal heart 50	1.1084111	0.19864
ES derived mesoderm 286	1.0760950	0.20696
fetal heart 46	1.0947833	0.23464
fetal heart 51	1.0791130	0.25880
fetal spinal cord 235	1.0694391	0.26274
fetal heart 44	1.0878927	0.27476
fetal brain 37	1.0469102	0.29746
fetal spinal cord 237	1.0513800	0.31094
fetal heart 47	1.0591602	0.32164
fetal spinal cord 236	1.0479411	0.33382
fetal spinal cord 239	1.0389732	0.35612
fetal heart 45	1.0382147	0.37222
fetal heart 49	1.0352483	0.38080
fetal brain 36	1.0140012	0.43300
fetal spinal cord 238	1.0166887	0.43344
fetal brain 38	1.0058627	0.46876
fetal brain 41	0.9977314	0.49986
fetal brain 33	0.9795457	0.57192
fetal brain 32	0.9742744	0.60150
fetal brain 39	0.9695659	0.62728
fetal brain 40	0.9640216	0.65782
fetal brain 29	0.9679563	0.65884
fetal brain 35	0.9515008	0.71834
fetal brain 30	0.9563052	0.71946
fetal brain 34	0.9311584	0.78864
fetal brain 31	0.9268963	0.83082

Lipid levels 6

DHS sample	fold enrichment	p value
fetal large intestine 55	1.2035249	0.00030
fetal large intestine 57	1.1963381	0.00056
fetal small intestine 80	1.2091365	0.00058
fetal large intestine 67	1.2232220	0.00060
fetal large intestine 64	1.2055539	0.00064
fetal large intestine 58	1.1934505	0.00082
fetal small intestine 78	1.1833226	0.00082
fetal small intestine 74	1.2092366	0.00084
small intestine 342	1.2053804	0.00094
CD20 8	1.1889193	0.00102
fetal small intestine 70	1.1793459	0.00108
fetal large intestine 60	1.2020303	0.00120
melanocyte 338	1.1384844	0.00198
CD14 4	1.2025840	0.00210
fetal small intestine 68	1.1764629	0.00212
fetal large intestine 56	1.1780954	0.00256
CD14 3	1.2096656	0.00286
fibroblast 332	1.1433766	0.00326
fibroblast 331	1.1420487	0.00348
fibroblast 329	1.1425273	0.00428
melanocyte 340	1.1382261	0.00442
melanocyte 337	1.1211780	0.00478
Mobilized CD56 324	1.1949887	0.00506
fetal small intestine 72	1.1662431	0.00544
fibroblast 330	1.1393158	0.00598
fetal large intestine 54	1.1668039	0.00620
melanocyte 339	1.1243471	0.00638
fetal small intestine 73	1.1637924	0.00698
fetal fibroblast 269	1.1252041	0.00732
pancreas 328	1.1675161	0.00766
fetal muscle 176	1.1363583	0.00784
CD8 21	1.2080363	0.00822
fetal fibroblast 279	1.1287100	0.00822
keratinocyte 334	1.1112162	0.00870
fetal adrenal gland 28	1.1284070	0.00932
fetal fibroblast 276	1.1189575	0.00984
fetal lung 141	1.1195609	0.01026
keratinocyte 336	1.1066765	0.01066
fetal large intestine 63	1.1533015	0.01096
fetal lung 133	1.1197646	0.01122
fetal fibroblast 268	1.1161569	0.01122
fetal lung 117	1.1212029	0.01124
CD4 13	1.1918398	0.01138
fetal testes 255	1.1349673	0.01152
hematopoietic stem cell 310	1.1168830	0.01258
fetal heart 47	1.1495462	0.01290
fetal small intestine 79	1.1524789	0.01316
fetal small intestine 77	1.1483725	0.01348
ES derived MSC 289	1.1336067	0.01352
heart 297	1.1457754	0.01372
fetal lung 299	1.1276381	0.01392
fetal fibroblast 280	1.1150809	0.01396
fetal muscle 153	1.1385264	0.01444
fetal fibroblast 273	1.1132163	0.01448
fetal lung 140	1.1156220	0.01458
fetal small intestine 69	1.1547971	0.01520
muscle 341	1.1561135	0.01526
fetal large intestine 62	1.1502469	0.01564
fetal thymus 258	1.1572195	0.01596
fetal lung 298	1.1263327	0.01606
ovary 326	1.1678267	0.01696
fetal fibroblast 275	1.1077745	0.01696
fetal muscle 180	1.1300652	0.01730
fetal fibroblast 272	1.1077212	0.01750
ES derived MSC 290	1.1272943	0.01798
fetal muscle 149	1.1316889	0.01808
Mobilized CD4 323	1.1769409	0.01836
breast 2	1.1049686	0.01850
fetal lung 118	1.1028759	0.01856
fetal fibroblast 270	1.1116318	0.01898
fetal fibroblast 277	1.1104301	0.01912
fetal muscle 169	1.1229748	0.01928
fetal muscle 167	1.1171644	0.01944
Mobilized CD3 320	1.1801455	0.01998
fetal muscle 143	1.1126790	0.02008
Mobilized CD4 322	1.1737686	0.02126
fetal muscle 181	1.1195640	0.02138
fetal lung 111	1.1083859	0.02168
fetal fibroblast 271	1.1048537	0.02176
fetal adrenal gland 27	1.1240264	0.02180
CD3 10	1.1544520	0.02182
fetal fibroblast 281	1.1030799	0.02214
fetal small intestine 71	1.1323921	0.02236
fetal lung 300	1.1121717	0.02242
keratinocyte 333	1.0911637	0.02266
CD4 14	1.1481251	0.02268
fetal muscle 166	1.1158808	0.02282
CD3 9	1.1619129	0.02356
fetal adrenal gland 24	1.1394455	0.02376
fetal fibroblast 274	1.1036781	0.02488
fetal muscle 152	1.1132092	0.02516
CD56 18	1.1644225	0.02548
fetal fibroblast 278	1.1043825	0.02550
fetal thymus 256	1.1357656	0.02572
fetal small intestine 76	1.1323563	0.02618
fetal muscle 164	1.1157131	0.02624
fetal large intestine 65	1.1280912	0.02636
keratinocyte 335	1.0902201	0.02658
fetal muscle 151	1.1237542	0.02732
fetal fibroblast 282	1.0949260	0.02804
placenta 200	1.1055292	0.02806
fetal thymus 264	1.1355859	0.02834
fetal adrenal gland 25	1.1253113	0.02882
breast 1	1.0952918	0.02898
pancreas 327	1.1365187	0.02906
fetal large intestine 66	1.1290570	0.02978
fetal muscle 159	1.1173068	0.03060
fetal large intestine 59	1.1265942	0.03062
CD56 17	1.1281866	0.03246
fetal fibroblast 267	1.0988036	0.03252
stomach 284	1.1274048	0.03310
fetal lung 301	1.1055705	0.03362
CD19 7	1.1551647	0.03370
fetal lung 116	1.0908174	0.03418
fetal heart 45	1.1102176	0.03672
fetal muscle 148	1.1071851	0.03824
hematopoietic stem cell 314	1.1003150	0.03840
fetal muscle 178	1.1069031	0.03924
fetal muscle 168	1.1060917	0.03962
fetal muscle 157	1.0956624	0.04000
fetal lung 114	1.0872311	0.04020
stomach 283	1.1198762	0.04280
fetal lung 128	1.0823729	0.04312
placenta 201	1.1131016	0.04342
CD19 5	1.1322435	0.04456
fetal muscle 160	1.1025543	0.04490
fetal lung 134	1.0949555	0.04560
fetal muscle 154	1.1075111	0.04598
fetal muscle 150	1.1034837	0.04706
fetal muscle 172	1.0866283	0.04860
fetal lung 119	1.0857944	0.04888
CD3 11	1.1212325	0.04936
fetal large intestine 53	1.1213886	0.04990
fetal skin 234	1.1010643	0.04998
fetal lung 129	1.0888001	0.05146
fetal muscle 187	1.0909286	0.05202
fetal muscle 175	1.0937794	0.05368
fetal lung 132	1.0889663	0.05400
fetal thymus 265	1.1161267	0.05436
fetal adrenal gland 23	1.0916651	0.05462
fetal stomach 244	1.1086507	0.05566
fetal muscle 165	1.0872124	0.05582
fetal heart 49	1.0971968	0.05590
fetal muscle 182	1.0914699	0.05632
fetal lung 120	1.0863823	0.05638
fetal kidney 85	1.0889360	0.06082
fetal lung 121	1.0778027	0.06292
fetal adrenal gland 26	1.0941161	0.06330
placenta 198	1.0872907	0.06338
fetal muscle 179	1.0902350	0.06406
fetal lung 125	1.0850070	0.06478
CD19 6	1.1246862	0.06536
placenta 199	1.0895277	0.06554
fetal heart 46	1.0939132	0.06572
fetal thymus 260	1.1110081	0.06580
fetal thymus 259	1.1157976	0.06630
fetal muscle 155	1.0947294	0.06662
fetal renal cortex 213	1.0902343	0.06702
hematopoietic stem cell 312	1.0849553	0.06768
CD3 12	1.1262133	0.06772
fetal stomach 248	1.1033765	0.06936
fetal testes 254	1.0929856	0.06962
Mobilized CD3 321	1.1300686	0.07012
fetal lung 130	1.0792657	0.07194
hematopoietic stem cell 315	1.0886504	0.07386
fetal heart 51	1.0870142	0.07470
fetal thymus 266	1.1036645	0.07482
fetal lung 124	1.0779273	0.07634
CD8 19	1.1281254	0.07722
fetal heart 52	1.0799995	0.07780
fetal muscle 184	1.0772050	0.07896
ES derived mesoderm 286	1.0671584	0.07962
CD4 15	1.1384395	0.08160
placenta 197	1.0899644	0.08166
CD8 20	1.1030246	0.08296
fetal kidney 91	1.0823274	0.08306
fetal thymus 263	1.0837556	0.08354
fetal stomach 243	1.0887468	0.08366
fetal muscle 183	1.0766932	0.08550
fetal large intestine 61	1.1073757	0.08592
fetal muscle 193	1.0825632	0.08628
fetal muscle 171	1.0771738	0.08648
fetal heart 43	1.0775117	0.08722
fetal muscle 147	1.0795427	0.09180
fetal lung 115	1.0681195	0.09390
fetal muscle 156	1.0763308	0.09500
hematopoietic stem cell 309	1.0773472	0.09732
fetal muscle 194	1.0679905	0.09886
fetal lung 138	1.0698154	0.10312
fetal muscle 174	1.0754352	0.10444
fetal stomach 250	1.0900584	0.10578
hematopoietic stem cell 318	1.0793110	0.10716
fetal muscle 186	1.0708208	0.10864
Mobilized CD8 325	1.1038402	0.10914
fetal kidney 106	1.0764919	0.10944
fetal heart 48	1.0743895	0.10968
fetal muscle 191	1.0730983	0.11024
fetal stomach 240	1.0894320	0.11086
fetal muscle 192	1.0692279	0.11244
placenta 196	1.0772426	0.11260
fetal lung 110	1.0678436	0.11416
fetal lung 136	1.0662518	0.11590
fetal muscle 158	1.0663199	0.11698
fetal lung 126	1.0601918	0.11780
fetal thymus 257	1.0913810	0.11966
hematopoietic stem cell 308	1.0662376	0.12220
fetal stomach 251	1.0763701	0.12478
fetal renal cortex 207	1.0734550	0.12554
fetal muscle 189	1.0666724	0.12598
fetal thymus 261	1.0836045	0.12728
hematopoietic stem cell 305	1.0699369	0.12766
fetal muscle 173	1.0671817	0.12984
CD4 16	1.0960362	0.13270
fetal stomach 242	1.0900694	0.13376
fetal lung 123	1.0614004	0.13436
ES derived mesoderm 285	1.0564881	0.13756
fetal muscle 146	1.0615145	0.13836
hematopoietic stem cell 317	1.0551955	0.14306
fetal kidney 92	1.0639749	0.14526
fetal lung 137	1.0557907	0.14588
fetal heart 42	1.0632404	0.14690
fetal muscle 188	1.0594040	0.14972
fetal kidney 87	1.0636830	0.15038
fetal stomach 246	1.0732732	0.15132
fetal kidney 95	1.0648071	0.15318
ES cells 296	1.0708907	0.15332
fetal muscle 161	1.0611631	0.15384
fetal renal pelvis 231	1.0610652	0.15440
fetal renal pelvis 220	1.0655679	0.15488
hematopoietic stem cell 306	1.0591441	0.15502
fetal muscle 185	1.0621662	0.15592
fetal lung 135	1.0559122	0.15602
ES cells 294	1.0698560	0.15642
fetal stomach 241	1.0708572	0.15928
fetal lung 108	1.0577297	0.16188
fetal heart 44	1.0694282	0.16300
fetal stomach 252	1.0665798	0.16306
iPS 304	1.0541338	0.16488
fetal renal cortex 214	1.0599127	0.16544
hematopoietic stem cell 316	1.0609056	0.17010
fetal muscle 163	1.0571211	0.17080
fetal heart 50	1.0560874	0.17658
fetal muscle 170	1.0512428	0.17780
fetal renal pelvis 226	1.0555041	0.18090
fetal lung 131	1.0439212	0.18312
fetal muscle 145	1.0576113	0.18766
CD8 22	1.0714583	0.18808
fetal stomach 253	1.0626883	0.18852
hematopoietic stem cell 311	1.0514589	0.18946
fetal lung 113	1.0443671	0.19052
fetal lung 127	1.0485488	0.19104
fetal muscle 190	1.0542747	0.19216
fetal renal pelvis 228	1.0581791	0.19286
fetal kidney 103	1.0551221	0.19468
fetal lung 139	1.0477072	0.19906
fetal renal pelvis 217	1.0541206	0.19970
fetal stomach 249	1.0601618	0.19990
fetal muscle 144	1.0512381	0.20172
fetal kidney 99	1.0516309	0.20210
fetal renal pelvis 219	1.0619882	0.20608
hematopoietic stem cell 307	1.0478118	0.20868
fetal renal pelvis 227	1.0495532	0.21472
fetal muscle 177	1.0490228	0.21504
fetal kidney 83	1.0544476	0.21638
fetal kidney 84	1.0506624	0.21698
fetal stomach 247	1.0600901	0.21888
fetal renal cortex 205	1.0471570	0.22284
fetal renal pelvis 233	1.0457095	0.23242
fetal stomach 245	1.0495036	0.23294
fetal kidney 105	1.0430938	0.23352
fetal lung 122	1.0413391	0.23602
hematopoietic stem cell 313	1.0399466	0.23640
fetal kidney 100	1.0440903	0.24018
fetal renal pelvis 232	1.0453340	0.24254
fetal kidney 89	1.0438833	0.24352
fetal muscle 162	1.0382635	0.24428
fetal renal cortex 202	1.0418789	0.24442
iPS 302	1.0387594	0.25162
fetal lung 142	1.0333766	0.25394
ES derived trophoblast 288	1.0358889	0.25860
fetal renal cortex 211	1.0417021	0.26010
fetal renal pelvis 229	1.0380932	0.26302
iPS 303	1.0356409	0.26322
fetal lung 112	1.0318600	0.27614
fetal small intestine 75	1.0581148	0.27646
fetal lung 109	1.0274548	0.27858
hematopoietic stem cell 319	1.0368907	0.27946
fetal renal pelvis 230	1.0370410	0.28522
fetal renal pelvis 221	1.0320953	0.29166
fetal kidney 94	1.0355370	0.29430
fetal kidney 104	1.0325317	0.30006
fetal kidney 81	1.0275624	0.30478
fetal kidney 96	1.0293322	0.30604
fetal thymus 262	1.0425961	0.30802
fetal ovary 195	1.0351710	0.31528
fetal kidney 88	1.0267187	0.32312
ES cells 295	1.0309431	0.32320
fetal kidney 102	1.0279623	0.32982
fetal renal cortex 215	1.0250411	0.33076
fetal kidney 82	1.0282692	0.33424
ES derived NP 291	1.0253548	0.35268
fetal kidney 97	1.0182983	0.37696
fetal renal pelvis 216	1.0188193	0.37774
ES derived NP 292	1.0189148	0.38132
fetal renal pelvis 225	1.0182635	0.38340
fetal renal pelvis 218	1.0201803	0.38544
fetal renal cortex 212	1.0163848	0.38662
fetal kidney 101	1.0160668	0.38896
fetal renal cortex 206	1.0159723	0.39176
fetal kidney 98	1.0158481	0.39422
fetal kidney 107	1.0151759	0.39672
ES cells 293	1.0152493	0.39916
fetal renal cortex 210	1.0154604	0.40226
fetal kidney 86	1.0176134	0.40244
fetal renal pelvis 224	1.0133643	0.40414
fetal renal pelvis 223	1.0130163	0.41566
fetal spinal cord 235	1.0102012	0.42080
ES derived trophoblast 287	1.0106846	0.42754
fetal renal cortex 204	1.0081444	0.43842
fetal renal cortex 208	1.0086027	0.43902
fetal spinal cord 237	1.0052973	0.45618
fetal kidney 93	1.0065869	0.45652
fetal brain 41	1.0040269	0.46150
fetal brain 37	1.0041988	0.46206
fetal renal pelvis 222	1.0046335	0.46358
fetal brain 39	0.9984123	0.51046
fetal brain 35	0.9982077	0.51388
fetal spinal cord 236	0.9956074	0.52604
fetal brain 36	0.9952965	0.53288
fetal brain 34	0.9954050	0.53856
fetal kidney 90	0.9928078	0.53894
fetal renal cortex 203	0.9900816	0.55528
fetal renal cortex 209	0.9869832	0.57678
fetal brain 38	0.9881184	0.58866
fetal brain 40	0.9885925	0.59176
fetal brain 29	0.9880537	0.61118
fetal brain 31	0.9870175	0.61976
fetal spinal cord 239	0.9638325	0.73598
fetal brain 33	0.9654809	0.74448
fetal brain 30	0.9722285	0.75272
fetal spinal cord 238	0.9598742	0.76480
fetal brain 32	0.9619944	0.78170

Rheumatoid arthritis 2

DHS sample	fold enrichment	p value
Mobilized CD56 324	1.9499175	0.00002
Mobilized CD3 320	1.8651812	0.00002
CD4 13	1.8214025	0.00002
CD3 9	1.7981427	0.00002
CD19 5	1.7410844	0.00002
Mobilized CD4 322	1.7396810	0.00002
CD3 11	1.7346737	0.00002
CD4 14	1.7263454	0.00002
CD3 10	1.7227768	0.00002
CD8 21	1.7212847	0.00002
CD19 6	1.7144400	0.00002
CD8 20	1.7130636	0.00002
CD3 12	1.7112107	0.00002
Mobilized CD4 323	1.6802584	0.00002
CD19 7	1.6796600	0.00002
CD56 17	1.6784712	0.00002
CD56 18	1.6521745	0.00002
fetal thymus 256	1.5791507	0.00002
CD14 4	1.5536408	0.00002
fetal large intestine 61	1.5522593	0.00004
fetal thymus 264	1.4810196	0.00004
CD8 19	1.6452781	0.00006
Mobilized CD3 321	1.6134134	0.00006
CD8 22	1.5664561	0.00006
fetal thymus 258	1.5166338	0.00008
hematopoietic stem cell 310	1.3585014	0.00008
CD14 3	1.5125097	0.00010
breast 1	1.3016799	0.00016
fetal thymus 265	1.4591943	0.00018
fetal thymus 260	1.4404039	0.00020
CD4 16	1.5338520	0.00022
fetal thymus 266	1.4382294	0.00028
breast 2	1.2799629	0.00038
Mobilized CD8 325	1.5047377	0.00040
hematopoietic stem cell 314	1.3076561	0.00050
fetal fibroblast 273	1.2731729	0.00062
fetal fibroblast 279	1.2655509	0.00062
fetal fibroblast 268	1.2577812	0.00064
fetal fibroblast 280	1.2648152	0.00076
fetal fibroblast 272	1.2624538	0.00080
melanocyte 338	1.2359427	0.00080
CD4 15	1.5571931	0.00090
fetal fibroblast 282	1.2472467	0.00090
melanocyte 337	1.2242691	0.00104
fetal fibroblast 269	1.2454674	0.00112
keratinocyte 336	1.2312431	0.00126
fetal fibroblast 274	1.2516117	0.00146
hematopoietic stem cell 312	1.2903446	0.00148
fetal fibroblast 276	1.2336223	0.00152
keratinocyte 335	1.2250011	0.00152
fetal fibroblast 271	1.2420004	0.00164
fetal fibroblast 281	1.2427935	0.00170
hematopoietic stem cell 317	1.2522807	0.00204
fibroblast 332	1.2416544	0.00206
hematopoietic stem cell 308	1.2787441	0.00220
hematopoietic stem cell 309	1.2941069	0.00228
fetal fibroblast 277	1.2432040	0.00258
fetal fibroblast 275	1.2224581	0.00286
fetal fibroblast 270	1.2261286	0.00290
fetal fibroblast 267	1.2354437	0.00292
keratinocyte 333	1.2055599	0.00296
fetal thymus 261	1.3547455	0.00304
keratinocyte 334	1.2073824	0.00310
fibroblast 331	1.2327818	0.00334
fetal thymus 259	1.3553679	0.00364
hematopoietic stem cell 313	1.2519309	0.00420
melanocyte 339	1.2024163	0.00422
hematopoietic stem cell 306	1.2717164	0.00452
fetal small intestine 75	1.4503671	0.00568
fetal fibroblast 278	1.2107957	0.00616
hematopoietic stem cell 316	1.2629502	0.00716
hematopoietic stem cell 305	1.2498271	0.00742
ES derived MSC 290	1.2347225	0.00754
fetal lung 299	1.2292342	0.00764
placenta 200	1.2129124	0.00882
ES derived MSC 289	1.2327347	0.00920
fetal thymus 257	1.3108627	0.00992
hematopoietic stem cell 318	1.2516964	0.01008
melanocyte 340	1.1851360	0.01116
hematopoietic stem cell 315	1.2395774	0.01128
fetal lung 300	1.1979636	0.01342
fibroblast 330	1.1942977	0.01388
hematopoietic stem cell 307	1.2182120	0.01508
fetal lung 301	1.1972036	0.01564
placenta 198	1.2021750	0.01596
fetal small intestine 78	1.2062095	0.01774
hematopoietic stem cell 311	1.2093976	0.01802
fetal small intestine 70	1.1953084	0.01898
fetal lung 298	1.1963348	0.01968
fetal muscle 181	1.1908093	0.02168
CD20 8	1.2050322	0.02212
fetal large intestine 64	1.2091300	0.02240
stomach 284	1.2277706	0.02316
fetal muscle 169	1.1860699	0.02316
fetal large intestine 57	1.1935646	0.02396
fetal muscle 180	1.1945485	0.02412
fetal small intestine 68	1.1977367	0.02484
fibroblast 329	1.1631180	0.02620
pancreas 328	1.2175291	0.02626
small intestine 342	1.2046333	0.02752
fetal large intestine 67	1.2075201	0.02780
fetal small intestine 74	1.2008164	0.02784
fetal large intestine 58	1.1843026	0.02962
placenta 201	1.1993905	0.03180
fetal lung 128	1.1413096	0.03184
fetal muscle 154	1.1849845	0.03366
ES derived mesoderm 285	1.1465435	0.03658
fetal muscle 176	1.1568942	0.03706
fetal thymus 262	1.2650718	0.03962
fetal small intestine 80	1.1773781	0.04062
fetal large intestine 54	1.1887022	0.04092
fetal muscle 172	1.1450503	0.04100
fetal small intestine 73	1.1869834	0.04122
hematopoietic stem cell 319	1.1864189	0.04196
fetal muscle 153	1.1690637	0.04520
fetal large intestine 55	1.1616698	0.05224
fetal large intestine 60	1.1738889	0.05356
fetal muscle 143	1.1338485	0.05628
fetal testes 255	1.1486499	0.05682
fetal stomach 244	1.1742777	0.05714
fetal lung 121	1.1257335	0.05790
placenta 197	1.1637294	0.05854
fetal muscle 151	1.1568112	0.05914
fetal muscle 149	1.1510663	0.06116
fetal lung 111	1.1266907	0.06196
fetal lung 133	1.1231749	0.06234
ES derived mesoderm 286	1.1146511	0.06272
fetal muscle 167	1.1346854	0.06506
stomach 283	1.1741316	0.06546
fetal small intestine 72	1.1571328	0.06618
fetal muscle 148	1.1428795	0.06662
ES derived trophoblast 288	1.1330312	0.06706
placenta 199	1.1438317	0.06830
fetal muscle 155	1.1460695	0.06960
fetal muscle 152	1.1307893	0.07084
fetal muscle 175	1.1310506	0.07314
fetal large intestine 56	1.1501620	0.07548
fetal lung 141	1.1111032	0.07828
fetal muscle 166	1.1262313	0.07980
fetal muscle 164	1.1301167	0.08214
fetal muscle 168	1.1326858	0.08308
fetal muscle 159	1.1334788	0.08634
fetal muscle 193	1.1305930	0.08730
fetal lung 126	1.1061138	0.08774
fetal small intestine 79	1.1514551	0.08786
fetal muscle 191	1.1261288	0.08950
fetal lung 116	1.1017271	0.09114
fetal large intestine 66	1.1454392	0.09142
fetal lung 140	1.1082185	0.09268
fetal small intestine 76	1.1463001	0.09352
fetal lung 117	1.1074423	0.09380
placenta 196	1.1336379	0.09440
fetal large intestine 63	1.1418309	0.09748
fetal muscle 187	1.1126334	0.09896
fetal muscle 150	1.1230791	0.09904
fetal muscle 160	1.1215268	0.10044
fetal large intestine 62	1.1411947	0.10288
fetal muscle 194	1.1020870	0.10424
pancreas 327	1.1432085	0.10484
fetal muscle 182	1.1128357	0.10536
fetal lung 134	1.1099539	0.10560
fetal stomach 243	1.1280495	0.10638
fetal testes 254	1.1251816	0.10696
fetal lung 137	1.0994822	0.11284
fetal muscle 165	1.1039891	0.11512
fetal muscle 147	1.1105558	0.11586
fetal muscle 179	1.1077491	0.11818
fetal muscle 188	1.1037545	0.11838
fetal muscle 189	1.1064042	0.11936
fetal small intestine 69	1.1312047	0.12064
fetal muscle 158	1.1020058	0.12250
fetal muscle 174	1.1080556	0.12258
fetal muscle 183	1.1011811	0.12278
fetal muscle 157	1.0979819	0.12410
fetal muscle 184	1.0978721	0.12428
fetal lung 108	1.1051112	0.12600
fetal muscle 185	1.1088324	0.12714
ES derived NP 291	1.1283154	0.12904
fetal muscle 192	1.1000764	0.12926
fetal small intestine 77	1.1189975	0.12928
fetal muscle 162	1.0983015	0.13084
fetal muscle 177	1.1086472	0.13344
fetal muscle 146	1.0988910	0.13480
ES derived NP 292	1.1171202	0.13546
fetal kidney 91	1.0980340	0.13762
fetal muscle 186	1.0961309	0.13808
fetal lung 127	1.0945727	0.13828
fetal lung 119	1.0860542	0.13992
muscle 341	1.1194161	0.14164
fetal lung 142	1.0838190	0.14228
fetal lung 120	1.0889817	0.14408
fetal muscle 156	1.0945619	0.14484
fetal renal cortex 207	1.1010934	0.14734
fetal stomach 246	1.1193704	0.14988
fetal lung 129	1.0865270	0.15142
fetal large intestine 65	1.1096393	0.15164
fetal lung 131	1.0770494	0.15308
fetal stomach 248	1.1130964	0.15342
fetal muscle 178	1.0935585	0.15612
fetal heart 45	1.0969870	0.15618
ES derived trophoblast 287	1.0962543	0.15958
fetal lung 114	1.0765934	0.15996
fetal muscle 173	1.0914131	0.16130
fetal muscle 163	1.0907798	0.16346
fetal lung 109	1.0726201	0.16430
fetal stomach 253	1.1113023	0.16682
fetal lung 118	1.0733727	0.16696
fetal lung 115	1.0770579	0.16912
fetal lung 132	1.0796683	0.17014
fetal large intestine 59	1.1020790	0.17234
fetal large intestine 53	1.1076611	0.17358
fetal stomach 252	1.1003852	0.17498
fetal lung 110	1.0800547	0.17774
fetal stomach 240	1.1075399	0.17892
fetal muscle 171	1.0803945	0.17954
fetal stomach 251	1.0974169	0.17998
fetal muscle 145	1.0906259	0.18402
fetal kidney 92	1.0818987	0.18564
fetal stomach 245	1.0977274	0.18760
fetal kidney 106	1.0820962	0.18898
fetal adrenal gland 26	1.0842984	0.19060
ES cells 295	1.0955614	0.19174
heart 297	1.0886857	0.19426
fetal thymus 263	1.0801587	0.19818
fetal kidney 85	1.0726399	0.19884
fetal skin 234	1.0811053	0.19950
fetal muscle 190	1.0802545	0.20132
fetal lung 125	1.0711411	0.20248
fetal heart 47	1.0871395	0.20440
fetal ovary 195	1.0964134	0.20512
fetal renal cortex 213	1.0736089	0.20614
fetal lung 130	1.0673325	0.20666
fetal muscle 144	1.0773436	0.20812
fetal lung 136	1.0676744	0.20982
ovary 326	1.0945597	0.21458
fetal kidney 83	1.0860499	0.21542
fetal stomach 241	1.0866437	0.22058
fetal small intestine 71	1.0791179	0.22084
ES cells 296	1.0826137	0.22452
fetal lung 138	1.0637479	0.22542
fetal stomach 250	1.0818734	0.23610
fetal renal pelvis 217	1.0712674	0.23638
fetal lung 113	1.0552403	0.23678
fetal lung 122	1.0619852	0.24006
fetal stomach 242	1.0879120	0.24374
fetal renal pelvis 220	1.0670739	0.24702
iPS 302	1.0626203	0.24724
fetal lung 124	1.0563622	0.24740
fetal stomach 249	1.0772517	0.24768
fetal muscle 161	1.0610166	0.24910
fetal muscle 170	1.0559113	0.25542
fetal lung 123	1.0548351	0.25722
fetal kidney 89	1.0621923	0.25866
fetal kidney 99	1.0603613	0.26382
fetal kidney 100	1.0588424	0.26564
fetal heart 42	1.0584184	0.26712
fetal kidney 94	1.0627382	0.26786
fetal renal pelvis 219	1.0707748	0.27114
fetal renal pelvis 227	1.0560196	0.27544
fetal renal pelvis 226	1.0550845	0.27700
fetal renal cortex 214	1.0540167	0.27908
fetal kidney 96	1.0524184	0.27974
fetal lung 112	1.0487078	0.28000
fetal kidney 81	1.0475906	0.28542
fetal renal pelvis 233	1.0519353	0.29022
fetal adrenal gland 27	1.0516135	0.29228
fetal heart 49	1.0507914	0.29318
fetal kidney 87	1.0497998	0.29512
fetal kidney 95	1.0499589	0.29750
fetal heart 44	1.0572803	0.30184
fetal brain 34	1.0351844	0.30376
fetal renal pelvis 231	1.0456137	0.30408
fetal renal pelvis 221	1.0462243	0.30510
fetal kidney 101	1.0461357	0.30704
ES cells 293	1.0500476	0.30752
fetal renal cortex 202	1.0459971	0.30774
fetal kidney 103	1.0464471	0.31038
fetal heart 51	1.0444653	0.31152
fetal adrenal gland 28	1.0403482	0.31488
fetal renal pelvis 224	1.0416932	0.31668
fetal lung 139	1.0403906	0.31732
fetal heart 48	1.0430766	0.32096
fetal renal cortex 205	1.0428014	0.32118
fetal kidney 84	1.0441822	0.32208
ES cells 294	1.0479908	0.32298
iPS 303	1.0402524	0.32360
fetal renal pelvis 229	1.0416248	0.32384
fetal heart 52	1.0379056	0.32638
fetal heart 46	1.0418968	0.32880
fetal renal pelvis 230	1.0440955	0.33048
fetal kidney 107	1.0400659	0.33056
fetal renal cortex 206	1.0384371	0.33398
fetal kidney 102	1.0414526	0.33596
fetal adrenal gland 25	1.0420475	0.33636
fetal heart 43	1.0360228	0.33756
fetal renal cortex 204	1.0358938	0.34090
fetal renal cortex 211	1.0404987	0.34094
fetal renal cortex 215	1.0363025	0.34118
fetal lung 135	1.0326858	0.34596
iPS 304	1.0310996	0.35432
fetal stomach 247	1.0430979	0.35662
fetal renal pelvis 228	1.0352933	0.35854
fetal renal cortex 209	1.0317905	0.36528
fetal kidney 93	1.0318658	0.36828
fetal kidney 104	1.0286284	0.37804
fetal heart 50	1.0277926	0.37870
fetal renal pelvis 222	1.0272608	0.38564
fetal renal pelvis 232	1.0269766	0.38680
fetal kidney 90	1.0257757	0.38768
fetal renal cortex 212	1.0235919	0.39276
fetal renal pelvis 216	1.0226952	0.39950
fetal renal cortex 203	1.0193227	0.41180
fetal kidney 97	1.0190976	0.41246
fetal kidney 86	1.0216046	0.41444
fetal renal pelvis 223	1.0200312	0.41544
fetal renal pelvis 225	1.0186805	0.41766
fetal kidney 82	1.0173592	0.42386
fetal kidney 98	1.0157187	0.42806
fetal kidney 105	1.0152689	0.42870
fetal spinal cord 236	1.0148243	0.42920
fetal renal cortex 208	1.0150133	0.43062
fetal adrenal gland 23	1.0109398	0.44698
fetal adrenal gland 24	1.0095793	0.45780
fetal brain 35	1.0052974	0.45856
fetal spinal cord 237	1.0069082	0.46194
fetal kidney 88	1.0068463	0.46200
fetal renal pelvis 218	0.9992292	0.48912
fetal renal cortex 210	0.9954101	0.50940
fetal brain 40	0.9837174	0.58662
fetal brain 41	0.9784441	0.60678
fetal brain 29	0.9825982	0.60880
fetal spinal cord 238	0.9702070	0.62804
fetal brain 39	0.9752506	0.62898
fetal brain 37	0.9727362	0.63688
fetal spinal cord 239	0.9655920	0.64628
fetal spinal cord 235	0.9629102	0.66034
fetal brain 36	0.9650248	0.67366
fetal brain 31	0.9646393	0.71164
fetal brain 33	0.9381733	0.77298
fetal brain 30	0.9525773	0.78830
fetal brain 38	0.9353628	0.79688
fetal brain 32	0.9264752	0.83596

Colorectal cancer

DHS sample	fold enrichment	p value
ES derived MSC 289	2.118276	0.00002
breast 2	2.094644	0.00002
keratinocyte 333	1.899733	0.00002
ES derived trophoblast 287	2.405699	0.00004
breast 1	2.108479	0.00004
ES derived MSC 290	2.172241	0.00006
fibroblast 331	1.945071	0.00006
keratinocyte 334	1.846186	0.00006
ES derived trophoblast 288	2.070589	0.00010
keratinocyte 335	1.770626	0.00012
keratinocyte 336	1.754801	0.00014
ES derived mesoderm 285	1.820430	0.00016
fibroblast 332	1.872564	0.00018
fetal large intestine 53	2.317026	0.00020
fetal fibroblast 278	1.830991	0.00020
stomach 283	2.301456	0.00026
fibroblast 329	1.839142	0.00026
fetal stomach 248	2.207017	0.00028
fibroblast 330	1.857801	0.00032
fetal stomach 251	2.154057	0.00034
fetal large intestine 66	2.146394	0.00038
placenta 201	2.085404	0.00040
fetal stomach 243	2.069432	0.00042
CD14 4	2.238807	0.00044
hematopoietic stem cell 310	1.841513	0.00044
ES derived mesoderm 286	1.697057	0.00046
fetal large intestine 67	2.103399	0.00048
fetal skin 234	1.957831	0.00052
fetal stomach 246	2.153053	0.00054
fetal lung 301	1.886416	0.00054
CD14 3	2.285423	0.00064
fetal small intestine 76	2.119297	0.00064
fetal fibroblast 268	1.729648	0.00064
fetal stomach 245	2.143482	0.00072
hematopoietic stem cell 309	1.912872	0.00072
fetal lung 298	1.846032	0.00072
fetal stomach 250	2.143374	0.00076
fetal stomach 244	2.046466	0.00080
fetal stomach 252	2.010166	0.00082
fetal fibroblast 267	1.719367	0.00082
fetal fibroblast 275	1.649174	0.00088
fetal fibroblast 279	1.735678	0.00090
fetal fibroblast 277	1.725964	0.00090
fetal fibroblast 282	1.678261	0.00090
fetal muscle 164	1.963363	0.00092
fetal fibroblast 269	1.724316	0.00092
fetal stomach 249	2.127048	0.00094
fetal lung 299	1.868382	0.00094
fetal muscle 172	1.762709	0.00094
fetal kidney 91	1.857602	0.00098
hematopoietic stem cell 317	1.773323	0.00098
fetal fibroblast 280	1.711433	0.00098
fetal renal cortex 205	1.949223	0.00104
fetal large intestine 62	2.084049	0.00108
fetal muscle 191	1.916307	0.00108
fetal fibroblast 274	1.700089	0.00110
fetal large intestine 59	2.002631	0.00114
fetal fibroblast 273	1.675356	0.00114
fetal muscle 152	1.837237	0.00116
fetal large intestine 56	1.980002	0.00120
fetal small intestine 68	1.932205	0.00120
fetal small intestine 77	1.973943	0.00124
fetal renal cortex 214	1.911126	0.00124
fetal muscle 189	1.888089	0.00128
fetal lung 300	1.791690	0.00128
fetal renal pelvis 220	1.953506	0.00130
fetal renal cortex 207	1.928681	0.00132
fetal large intestine 60	1.950284	0.00134
hematopoietic stem cell 315	1.912688	0.00134
fetal renal cortex 213	1.818002	0.00134
fetal lung 109	1.690735	0.00134
fetal renal pelvis 231	1.883967	0.00136
fetal heart 48	1.879421	0.00136
fetal kidney 85	1.775829	0.00138
fetal heart 51	1.883562	0.00142
hematopoietic stem cell 312	1.822416	0.00142
fetal large intestine 58	1.861285	0.00144
fetal renal cortex 204	1.838239	0.00144
fetal muscle 180	1.910830	0.00146
fetal kidney 89	1.971365	0.00148
fetal muscle 154	1.957660	0.00148
fetal large intestine 65	2.013589	0.00150
fetal large intestine 63	1.978045	0.00152
fetal muscle 177	1.927491	0.00152
fetal muscle 174	1.896798	0.00152
fetal small intestine 70	1.811107	0.00152
fetal fibroblast 281	1.677425	0.00154
fetal kidney 97	1.922493	0.00156
fetal lung 110	1.816360	0.00158
fetal stomach 247	2.209611	0.00162
fetal kidney 88	1.894864	0.00162
fetal muscle 173	1.891510	0.00162
fetal stomach 253	2.051179	0.00164
fetal lung 138	1.780232	0.00164
fetal kidney 103	1.945399	0.00172
fetal muscle 148	1.860619	0.00172
fetal heart 44	2.116931	0.00174
fetal renal pelvis 228	1.959533	0.00174
fetal kidney 82	2.057525	0.00176
fetal muscle 190	1.889519	0.00176
fetal muscle 163	1.866319	0.00178
fetal renal pelvis 229	1.897656	0.00180
fetal kidney 81	1.794371	0.00182
fetal muscle 187	1.794071	0.00182
fetal large intestine 55	1.873672	0.00184
fetal renal pelvis 222	1.944081	0.00186
fetal large intestine 64	1.911962	0.00186
fetal small intestine 80	1.870395	0.00194
fetal renal pelvis 224	1.858006	0.00196
fetal heart 43	1.776929	0.00198
fetal stomach 241	2.000963	0.00200
fetal small intestine 71	1.932903	0.00200
fetal muscle 171	1.813586	0.00200
fetal small intestine 79	1.969361	0.00204
fetal muscle 183	1.783803	0.00204
fetal kidney 83	2.024030	0.00210
fetal muscle 156	1.833027	0.00214
hematopoietic stem cell 306	1.835808	0.00216
fetal kidney 95	1.871106	0.00220
fetal small intestine 69	1.954076	0.00222
fetal heart 46	1.866151	0.00222
fetal renal pelvis 223	1.990789	0.00228
fetal muscle 147	1.836441	0.00230
fetal heart 50	1.833084	0.00230
fetal renal pelvis 221	1.847770	0.00232
fetal muscle 162	1.802473	0.00234
fetal renal pelvis 226	1.859763	0.00236
fetal small intestine 72	1.886157	0.00238
fetal kidney 92	1.824904	0.00238
stomach 284	1.991604	0.00240
fetal kidney 87	1.833369	0.00240
fetal renal cortex 215	1.834172	0.00244
fetal renal pelvis 227	1.888098	0.00250
fetal heart 42	1.829795	0.00252
fetal muscle 145	1.929611	0.00256
fetal muscle 160	1.844861	0.00256
fetal muscle 146	1.825020	0.00262
fetal lung 134	1.759126	0.00262
fetal renal cortex 209	1.881953	0.00270
fetal kidney 106	1.865573	0.00270
fetal kidney 96	1.830481	0.00282
fetal small intestine 73	1.896522	0.00284
fetal muscle 169	1.813198	0.00286
fetal renal pelvis 233	1.882798	0.00288
hematopoietic stem cell 316	1.847863	0.00290
fetal muscle 159	1.845752	0.00290
fetal kidney 100	1.838189	0.00294
muscle 341	2.015735	0.00296
fetal kidney 104	1.877684	0.00296
fetal muscle 149	1.853987	0.00306
small intestine 342	1.847593	0.00306
fetal thymus 263	1.827792	0.00308
fetal muscle 188	1.752950	0.00310
fetal muscle 192	1.755516	0.00312
fetal muscle 178	1.797465	0.00316
fetal small intestine 74	1.827846	0.00318
fetal renal cortex 206	1.799309	0.00320
fetal stomach 240	1.982142	0.00322
fetal kidney 93	1.927453	0.00322
fetal lung 127	1.736539	0.00328
fetal large intestine 54	1.858750	0.00330
hematopoietic stem cell 314	1.717558	0.00334
fetal muscle 150	1.830032	0.00340
fetal kidney 90	1.876876	0.00344
fetal adrenal gland 23	1.741011	0.00346
fetal lung 122	1.756796	0.00348
fetal kidney 101	1.827431	0.00350
fetal muscle 181	1.781602	0.00354
fetal muscle 175	1.769494	0.00360
fetal muscle 193	1.809562	0.00366
fetal muscle 161	1.780294	0.00370
fetal renal pelvis 225	1.898868	0.00376
fetal heart 52	1.702555	0.00382
fetal lung 131	1.597077	0.00386
fetal renal cortex 203	1.822471	0.00388
fetal muscle 185	1.837817	0.00390
fetal muscle 170	1.732671	0.00392
fetal renal pelvis 232	1.871139	0.00396
fetal large intestine 57	1.754127	0.00396
fetal muscle 167	1.731781	0.00396
hematopoietic stem cell 307	1.790449	0.00398
hematopoietic stem cell 311	1.763175	0.00404
fetal kidney 102	1.913325	0.00406
hematopoietic stem cell 313	1.709878	0.00406
fetal fibroblast 270	1.629381	0.00408
fetal muscle 168	1.813621	0.00410
fetal renal pelvis 219	2.060084	0.00418
fetal muscle 186	1.732132	0.00418
hematopoietic stem cell 305	1.716746	0.00418
fetal fibroblast 276	1.555488	0.00420
fetal renal cortex 211	1.852829	0.00424
fetal muscle 184	1.686726	0.00430
fetal lung 139	1.726302	0.00434
fetal lung 137	1.661430	0.00438
fetal lung 113	1.626148	0.00440
fetal kidney 107	1.807417	0.00442
fetal muscle 182	1.730288	0.00446
fetal kidney 99	1.789496	0.00452
fetal small intestine 78	1.748855	0.00456
fetal renal pelvis 216	1.823314	0.00464
fetal renal cortex 212	1.765535	0.00470
fetal lung 136	1.664259	0.00472
fetal muscle 179	1.730036	0.00482
iPS 302	1.642933	0.00484
heart 297	1.883420	0.00488
fetal muscle 153	1.803637	0.00502
fetal muscle 176	1.683265	0.00508
CD20 8	1.760123	0.00518
fetal lung 108	1.726879	0.00540
fetal renal cortex 208	1.832921	0.00544
fetal lung 142	1.605512	0.00546
pancreas 327	1.931101	0.00550
fetal kidney 84	1.843220	0.00556
fetal kidney 98	1.812099	0.00564
fetal adrenal gland 25	1.807017	0.00564
fetal muscle 155	1.781783	0.00580
hematopoietic stem cell 319	1.772390	0.00588
fetal muscle 165	1.690696	0.00592
fetal adrenal gland 26	1.708580	0.00604
fetal kidney 94	1.838697	0.00610
fetal renal pelvis 230	1.868984	0.00618
fetal lung 114	1.559915	0.00630
fetal muscle 151	1.804580	0.00654
fetal lung 126	1.565946	0.00690
fetal muscle 144	1.749884	0.00692
fetal kidney 105	1.724185	0.00694
fetal muscle 166	1.684113	0.00702
fetal lung 125	1.651932	0.00702
fetal lung 135	1.644609	0.00702
fetal renal cortex 202	1.727184	0.00704
fetal stomach 242	1.944634	0.00706
fetal muscle 157	1.632480	0.00714
fetal renal pelvis 217	1.764060	0.00762
fetal lung 115	1.571780	0.00774
fetal lung 129	1.600931	0.00778
hematopoietic stem cell 318	1.729131	0.00806
fetal muscle 158	1.646209	0.00816
fetal fibroblast 272	1.536851	0.00830
fetal thymus 257	1.953826	0.00836
fetal lung 130	1.593011	0.00854
fetal renal cortex 210	1.800077	0.00856
fetal spinal cord 237	1.595839	0.00880
fetal lung 120	1.602553	0.00886
fetal thymus 260	1.927228	0.00902
fetal muscle 143	1.621958	0.00902
fetal renal pelvis 218	1.939406	0.00906
fetal thymus 266	1.866624	0.00908
fetal kidney 86	1.870430	0.00910
fetal lung 128	1.511951	0.00914
fetal testes 254	1.684399	0.00916
iPS 303	1.561927	0.00916
fetal lung 124	1.588270	0.00966
ES derived NP 291	1.726395	0.00988
ES cells 294	1.689947	0.01012
fetal muscle 194	1.549327	0.01016
fetal lung 112	1.588884	0.01024
melanocyte 340	1.484384	0.01084
fetal heart 49	1.677949	0.01096
CD56 17	1.876738	0.01104
fetal lung 118	1.497305	0.01126
Mobilized CD8 325	2.036050	0.01150
CD4 14	1.881168	0.01150
hematopoietic stem cell 308	1.589870	0.01156
fetal lung 132	1.562271	0.01180
fetal adrenal gland 28	1.577081	0.01186
melanocyte 339	1.441643	0.01198
fetal ovary 195	1.748865	0.01252
fetal fibroblast 271	1.483903	0.01400
fetal heart 45	1.641824	0.01410
fetal lung 116	1.474734	0.01506
fetal thymus 265	1.814667	0.01516
fetal thymus 258	1.822408	0.01546
fetal thymus 256	1.801872	0.01590
fetal spinal cord 235	1.527968	0.01596
fetal lung 119	1.493111	0.01626
fetal large intestine 61	1.821634	0.01682
CD56 18	1.972597	0.01694
fetal thymus 261	1.839695	0.01726
ovary 326	1.770580	0.01844
fetal testes 255	1.520511	0.01860
fetal lung 133	1.478690	0.01898
fetal lung 121	1.468545	0.02086
fetal lung 111	1.477349	0.02192
iPS 304	1.439759	0.02228
fetal spinal cord 238	1.494312	0.02232
fetal lung 141	1.452104	0.02300
CD4 16	1.824559	0.02340
fetal thymus 259	1.760943	0.02428
fetal spinal cord 236	1.509096	0.02440
placenta 196	1.563189	0.02536
fetal lung 123	1.483670	0.02556
CD3 11	1.754223	0.02610
fetal thymus 264	1.685921	0.02634
CD8 20	1.767557	0.02692
fetal adrenal gland 27	1.544277	0.02800
fetal thymus 262	1.860950	0.02846
fetal lung 117	1.451718	0.02880
ES cells 293	1.502041	0.03222
Mobilized CD4 323	1.760937	0.03272
pancreas 328	1.580636	0.03298
fetal heart 47	1.569633	0.03348
CD19 6	1.712854	0.03652
fetal lung 140	1.417626	0.03766
fetal spinal cord 239	1.417514	0.03770
placenta 200	1.445927	0.03774
fetal adrenal gland 24	1.573328	0.03850
fetal small intestine 75	1.759890	0.04108
CD4 13	1.738651	0.04320
CD8 21	1.740649	0.04608
CD3 10	1.620747	0.04878
fetal brain 40	1.309851	0.05042
CD19 5	1.608960	0.05430
melanocyte 337	1.298011	0.05480
CD8 19	1.710874	0.05814
ES derived NP 292	1.444615	0.06036
placenta 197	1.443818	0.06050
fetal brain 35	1.281011	0.06056
CD8 22	1.597721	0.06738
Mobilized CD3 321	1.626723	0.06740
CD3 9	1.593802	0.07016
fetal brain 34	1.277386	0.07106
ES cells 296	1.420474	0.07148
fetal brain 39	1.273406	0.07158
placenta 199	1.381368	0.07172
fetal brain 38	1.308127	0.07254
CD3 12	1.573676	0.07818
fetal brain 37	1.271655	0.08064
fetal brain 33	1.310473	0.08130
CD19 7	1.534366	0.08432
fetal brain 41	1.265886	0.08708
fetal brain 32	1.265986	0.09442
Mobilized CD4 322	1.505871	0.09740
ES cells 295	1.336518	0.10964
fetal brain 29	1.192957	0.11204
fetal brain 36	1.215891	0.13796
Mobilized CD56 324	1.386077	0.14158
melanocyte 338	1.203984	0.14272
placenta 198	1.244951	0.15658
fetal brain 30	1.156880	0.16102
CD4 15	1.397333	0.16388
fetal brain 31	1.118911	0.22682
Mobilized CD3 320	1.132375	0.33900

Anthropometric traits

DHS sample	fold enrichment	p value
fetal fibroblast 275	1.1847980	0.00292
fetal muscle 180	1.2252825	0.00328
fetal fibroblast 273	1.1854440	0.00346
fetal fibroblast 276	1.1792539	0.00380
fetal fibroblast 271	1.1806206	0.00386
fetal muscle 181	1.2116945	0.00406
fetal muscle 167	1.1960101	0.00490
fetal muscle 176	1.1897764	0.00502
fetal fibroblast 272	1.1729490	0.00522
melanocyte 338	1.1581752	0.00552
fetal thymus 258	1.2643433	0.00558
fetal fibroblast 269	1.1755675	0.00572
fetal muscle 168	1.2037379	0.00600
fetal thymus 256	1.2495881	0.00638
fetal lung 298	1.1954017	0.00648
fetal fibroblast 279	1.1709033	0.00704
fetal muscle 164	1.1924877	0.00744
hematopoietic stem cell 310	1.1747085	0.00838
fetal fibroblast 274	1.1621053	0.00858
fetal muscle 143	1.1700958	0.00916
fetal fibroblast 270	1.1633676	0.00930
fetal heart 47	1.2204104	0.00934
fetal lung 301	1.1802442	0.00934
fetal fibroblast 278	1.1633729	0.00968
fetal lung 299	1.1830681	0.00978
fetal fibroblast 280	1.1589936	0.00978
fetal muscle 149	1.1933981	0.00982
fetal muscle 160	1.1873985	0.01000
fetal fibroblast 268	1.1554149	0.01012
fetal muscle 153	1.1934515	0.01018
fetal muscle 151	1.1953812	0.01084
fetal thymus 264	1.2313540	0.01158
fetal fibroblast 281	1.1527407	0.01172
hematopoietic stem cell 314	1.1783682	0.01176
fetal fibroblast 277	1.1621558	0.01202
fetal muscle 175	1.1729333	0.01204
fetal thymus 265	1.2351272	0.01222
fetal muscle 154	1.1877905	0.01254
fetal muscle 172	1.1536611	0.01296
fetal fibroblast 282	1.1453242	0.01310
fetal lung 300	1.1653975	0.01380
fetal muscle 152	1.1645340	0.01384
fetal thymus 260	1.2295097	0.01486
fibroblast 329	1.1545948	0.01516
fetal thymus 266	1.2226255	0.01524
fetal muscle 187	1.1583877	0.01636
ES derived MSC 289	1.1725931	0.01696
melanocyte 337	1.1288107	0.01696
fetal muscle 179	1.1625694	0.01704
fetal muscle 182	1.1615129	0.01722
fetal lung 140	1.1456634	0.01748
ES derived MSC 290	1.1681022	0.01820
melanocyte 339	1.1323719	0.01894
fetal muscle 157	1.1487243	0.01912
fetal fibroblast 267	1.1470459	0.01922
fetal lung 118	1.1329614	0.02040
hematopoietic stem cell 309	1.1682852	0.02096
fibroblast 330	1.1475552	0.02098
fetal lung 111	1.1439781	0.02106
fetal lung 141	1.1371157	0.02122
fetal muscle 169	1.1591401	0.02174
fetal muscle 178	1.1614438	0.02196
fetal muscle 148	1.1540703	0.02510
fetal muscle 150	1.1575900	0.02568
fetal muscle 165	1.1400904	0.02576
fetal muscle 166	1.1493818	0.02586
fetal lung 128	1.1240338	0.02596
fetal lung 117	1.1329635	0.02688
fetal muscle 159	1.1553290	0.02836
fetal muscle 155	1.1568343	0.02856
Mobilized CD3 320	1.2349690	0.02952
fetal lung 133	1.1275347	0.03006
fetal lung 121	1.1268846	0.03072
fetal lung 120	1.1343477	0.03092
fetal heart 45	1.1562741	0.03134
fetal muscle 171	1.1396783	0.03156
fibroblast 332	1.1299921	0.03224
fetal thymus 259	1.2023051	0.03248
hematopoietic stem cell 318	1.1639226	0.03424
hematopoietic stem cell 305	1.1508420	0.03622
fetal heart 49	1.1510157	0.03700
melanocyte 340	1.1197190	0.03786
fetal muscle 156	1.1334288	0.03900
fetal muscle 158	1.1286315	0.04036
fetal lung 119	1.1153993	0.04054
fetal muscle 192	1.1296141	0.04102
fibroblast 331	1.1223019	0.04288
fetal lung 116	1.1119407	0.04292
fetal heart 46	1.1482431	0.04306
fetal thymus 261	1.1800693	0.04314
fetal muscle 193	1.1373070	0.04318
fetal muscle 189	1.1273046	0.04722
Mobilized CD56 324	1.1730799	0.04848
CD19 6	1.1925172	0.04912
fetal lung 114	1.1069271	0.04952
hematopoietic stem cell 317	1.1175551	0.05034
keratinocyte 334	1.0978959	0.05148
fetal testes 255	1.1236934	0.05290
CD19 5	1.1760118	0.05470
fetal muscle 186	1.1195953	0.05532
fetal lung 129	1.1124712	0.05638
keratinocyte 336	1.0952523	0.05732
fetal heart 42	1.1295949	0.05764
fetal muscle 185	1.1256614	0.05962
fetal thymus 263	1.1241042	0.06082
CD4 13	1.1822358	0.06128
fetal lung 115	1.1054848	0.06132
fetal muscle 184	1.1092746	0.06200
fetal heart 52	1.1155694	0.06238
fetal muscle 194	1.1054022	0.06278
fetal muscle 146	1.1133880	0.06282
CD3 10	1.1660478	0.06284
fetal muscle 183	1.1112523	0.06396
fetal lung 125	1.1125988	0.06418
fetal lung 134	1.1125914	0.06478
fetal lung 132	1.1083557	0.06500
fetal thymus 257	1.1654096	0.06580
fetal lung 108	1.1141270	0.06726
keratinocyte 335	1.0912920	0.06788
fetal muscle 161	1.1147219	0.06900
fetal lung 136	1.1058593	0.07092
CD14 4	1.1441190	0.07140
CD3 9	1.1680022	0.07212
fetal muscle 147	1.1110209	0.07464
hematopoietic stem cell 315	1.1216010	0.07532
fetal lung 126	1.0946250	0.07688
hematopoietic stem cell 308	1.1115092	0.07834
CD3 11	1.1462713	0.08098
fetal muscle 174	1.1066928	0.08298
Mobilized CD4 323	1.1610853	0.08530
hematopoietic stem cell 312	1.1073340	0.08662
fetal heart 44	1.1363740	0.08712
hematopoietic stem cell 316	1.1195042	0.08730
fetal muscle 173	1.1035819	0.08858
fetal lung 110	1.0982017	0.09060
keratinocyte 333	1.0790674	0.09100
CD19 7	1.1561500	0.09162
fetal muscle 177	1.1040740	0.09844
CD8 21	1.1534846	0.10100
hematopoietic stem cell 319	1.1121245	0.10120
CD4 14	1.1296727	0.10316
fetal heart 51	1.1014002	0.10360
breast 2	1.0817346	0.10570
fetal heart 48	1.1011949	0.10592
fetal lung 123	1.0900397	0.10618
CD14 3	1.1279705	0.10908
fetal lung 135	1.0882272	0.11136
CD56 17	1.1180155	0.11178
fetal large intestine 61	1.1331025	0.11304
fetal lung 124	1.0862036	0.11318
Mobilized CD8 325	1.1409020	0.11452
fetal lung 113	1.0789561	0.11476
fetal lung 130	1.0843904	0.11584
fetal lung 138	1.0856229	0.11648
hematopoietic stem cell 313	1.0911516	0.11790
hematopoietic stem cell 311	1.0923286	0.12352
hematopoietic stem cell 307	1.0930311	0.12388
CD8 19	1.1456681	0.12390
fetal muscle 170	1.0831122	0.12646
CD56 18	1.1337196	0.12730
fetal adrenal gland 26	1.0916669	0.12760
CD8 20	1.1163919	0.13090
fetal muscle 191	1.0861367	0.13126
fetal lung 131	1.0701291	0.13282
hematopoietic stem cell 306	1.0892078	0.13404
fetal lung 139	1.0818888	0.13446
fetal muscle 190	1.0895107	0.13594
fetal testes 254	1.0896686	0.13936
fetal adrenal gland 27	1.0868972	0.14144
fetal heart 43	1.0807108	0.14146
breast 1	1.0702334	0.14200
fetal lung 142	1.0694056	0.14428
fetal muscle 188	1.0772002	0.14664
fetal heart 50	1.0853575	0.14798
fetal lung 112	1.0730826	0.14942
fetal muscle 163	1.0804716	0.15018
Mobilized CD4 322	1.1218500	0.15052
fetal kidney 81	1.0724253	0.15236
fetal thymus 262	1.1263756	0.15242
fetal muscle 162	1.0737810	0.15506
fetal lung 137	1.0689551	0.15628
fetal muscle 144	1.0802489	0.15704
fetal muscle 145	1.0821730	0.16284
fetal kidney 85	1.0700742	0.16486
muscle 341	1.0907153	0.16662
fetal adrenal gland 28	1.0683006	0.16910
fetal lung 127	1.0687519	0.17142
fetal stomach 244	1.0831586	0.17402
fetal lung 109	1.0572359	0.18058
Mobilized CD3 321	1.1096492	0.18194
CD3 12	1.1032104	0.19094
fetal stomach 250	1.0820378	0.19254
fetal stomach 249	1.0831490	0.19274
fetal large intestine 53	1.0796031	0.20010
fetal adrenal gland 25	1.0723760	0.20326
heart 297	1.0705248	0.21546
fetal lung 122	1.0585408	0.21610
iPS 304	1.0549020	0.22370
fetal stomach 248	1.0680145	0.22586
CD8 22	1.0842322	0.22750
fetal kidney 91	1.0561917	0.22754
ES derived mesoderm 286	1.0439317	0.23282
fetal large intestine 67	1.0621478	0.23362
iPS 303	1.0495230	0.25384
ES derived mesoderm 285	1.0429077	0.25572
ovary 326	1.0645942	0.25638
fetal small intestine 75	1.0843833	0.25782
ES derived NP 291	1.0586565	0.25878
fetal stomach 253	1.0585991	0.26100
fetal adrenal gland 23	1.0469908	0.26144
CD4 15	1.0837612	0.26342
fetal stomach 246	1.0575397	0.26610
fetal kidney 106	1.0459168	0.27710
fetal small intestine 78	1.0441521	0.28130
CD4 16	1.0644346	0.28462
fetal kidney 83	1.0511496	0.28522
fetal stomach 241	1.0509135	0.29308
fetal kidney 103	1.0430672	0.29724
fetal kidney 100	1.0414011	0.29982
fetal stomach 251	1.0438064	0.30364
fetal renal pelvis 220	1.0414487	0.30542
fetal adrenal gland 24	1.0455434	0.30556
ES cells 294	1.0424714	0.31260
fetal large intestine 57	1.0374705	0.31570
small intestine 342	1.0399070	0.31738
iPS 302	1.0344803	0.32070
fetal small intestine 69	1.0410391	0.32296
fetal large intestine 55	1.0368850	0.32296
CD20 8	1.0358742	0.32584
fetal renal cortex 214	1.0354424	0.32594
fetal small intestine 74	1.0372514	0.32638
fetal stomach 242	1.0434474	0.33624
fetal stomach 243	1.0323205	0.34292
fetal stomach 247	1.0366018	0.35508
fetal stomach 245	1.0314443	0.35714
fetal renal pelvis 228	1.0297550	0.36002
fetal kidney 95	1.0274744	0.36066
fetal kidney 89	1.0267905	0.36456
fetal stomach 252	1.0275401	0.36866
fetal large intestine 64	1.0271921	0.36882
fetal kidney 92	1.0249535	0.36934
fetal small intestine 77	1.0267578	0.37230
fetal renal pelvis 221	1.0237905	0.37234
stomach 283	1.0294090	0.37274
fetal renal pelvis 227	1.0237429	0.37674
ES cells 296	1.0245823	0.38584
fetal small intestine 68	1.0221947	0.38840
fetal renal cortex 207	1.0209375	0.39074
fetal small intestine 72	1.0215044	0.39252
fetal large intestine 56	1.0210791	0.39434
fetal kidney 84	1.0199292	0.39772
ES cells 293	1.0197425	0.40138
fetal kidney 98	1.0185301	0.40422
placenta 201	1.0190708	0.40670
fetal kidney 99	1.0154492	0.41462
fetal renal pelvis 232	1.0164392	0.41490
fetal kidney 88	1.0150942	0.41550
fetal kidney 96	1.0154139	0.41618
fetal large intestine 54	1.0150986	0.42288
fetal small intestine 73	1.0150403	0.42412
fetal large intestine 66	1.0152715	0.42612
fetal ovary 195	1.0145642	0.42854
fetal kidney 94	1.0124200	0.43116
fetal renal cortex 215	1.0117819	0.43152
fetal large intestine 58	1.0125717	0.43238
fetal renal pelvis 217	1.0120127	0.43396
fetal large intestine 60	1.0125357	0.43406
fetal small intestine 71	1.0124519	0.43480
fetal renal pelvis 219	1.0126514	0.43830
fetal stomach 240	1.0115097	0.44258
fetal small intestine 80	1.0099144	0.44596
fetal renal pelvis 229	1.0080250	0.45194
fetal renal cortex 205	1.0080115	0.45398
ES cells 295	1.0078595	0.45750
fetal renal pelvis 226	1.0038751	0.47256
fetal skin 234	1.0047780	0.47398
fetal renal pelvis 230	1.0028772	0.47654
fetal large intestine 59	1.0000995	0.48874
fetal renal cortex 206	1.0002882	0.48970
fetal renal cortex 213	1.0005134	0.48992
ES derived NP 292	0.9996584	0.49036
fetal kidney 102	0.9995121	0.49044
fetal large intestine 63	0.9997273	0.49144
fetal kidney 105	1.0001503	0.49176
fetal renal pelvis 231	0.9995242	0.49398
fetal kidney 82	0.9989278	0.49628
fetal renal pelvis 222	0.9987830	0.49654
fetal renal cortex 202	0.9980329	0.50064
fetal small intestine 79	0.9973438	0.50126
fetal small intestine 70	0.9988620	0.50246
pancreas 328	0.9955361	0.51318
fetal small intestine 76	0.9947612	0.51452
fetal kidney 104	0.9948554	0.51814
fetal large intestine 65	0.9946651	0.51830
fetal kidney 107	0.9936197	0.52434
fetal renal pelvis 233	0.9915940	0.53420
pancreas 327	0.9898840	0.53494
fetal renal cortex 210	0.9904434	0.53640
stomach 284	0.9894296	0.53794
fetal large intestine 62	0.9868316	0.54756
fetal kidney 87	0.9888120	0.54796
fetal renal cortex 212	0.9858730	0.56380
fetal kidney 97	0.9846653	0.56816
fetal renal cortex 204	0.9841765	0.57618
fetal renal pelvis 224	0.9839754	0.57704
fetal kidney 86	0.9718543	0.60322
fetal renal pelvis 223	0.9752296	0.60516
placenta 197	0.9751504	0.60644
fetal renal cortex 211	0.9728204	0.61410
fetal renal pelvis 216	0.9736575	0.61612
fetal renal cortex 203	0.9744592	0.61708
fetal renal cortex 209	0.9740255	0.61808
fetal kidney 101	0.9739313	0.61962
fetal renal pelvis 218	0.9658284	0.62092
fetal spinal cord 236	0.9742819	0.62422
fetal renal pelvis 225	0.9704256	0.62844
fetal kidney 93	0.9665483	0.64190
fetal renal cortex 208	0.9657713	0.65100
fetal kidney 90	0.9659160	0.65298
placenta 200	0.9641233	0.68066
placenta 198	0.9631478	0.68078
fetal spinal cord 235	0.9606218	0.70262
ES derived trophoblast 288	0.9614110	0.70334
placenta 199	0.9466105	0.74600
placenta 196	0.9330936	0.78376
fetal spinal cord 239	0.9364835	0.80828
fetal spinal cord 237	0.9260883	0.85428
fetal spinal cord 238	0.9241280	0.85562
fetal brain 36	0.9301236	0.87076
fetal brain 41	0.9255582	0.89362
ES derived trophoblast 287	0.8985542	0.90694
fetal brain 33	0.9035590	0.92494
fetal brain 39	0.9186409	0.92578
fetal brain 32	0.9088945	0.93110
fetal brain 31	0.9229486	0.93754
fetal brain 38	0.8999522	0.94462
fetal brain 37	0.9052628	0.94682
fetal brain 34	0.9076788	0.95254
fetal brain 35	0.9120691	0.95514
fetal brain 29	0.9134843	0.96314
fetal brain 40	0.8953832	0.97010
fetal brain 30	0.8970030	0.98610

Human red blood cell

DHS sample	fold enrichment	p value
hematopoietic stem cell 310	1.4152043	0.00006
fetal thymus 256	1.5377825	0.00008
fetal thymus 258	1.5536428	0.00014
fetal thymus 264	1.4924850	0.00020
fetal thymus 260	1.5124373	0.00022
hematopoietic stem cell 317	1.3416713	0.00026
hematopoietic stem cell 314	1.3783424	0.00028
fetal thymus 265	1.5012108	0.00044
hematopoietic stem cell 309	1.3850883	0.00052
fetal thymus 266	1.4462182	0.00064
fetal thymus 259	1.4935531	0.00074
hematopoietic stem cell 313	1.3426385	0.00078
hematopoietic stem cell 312	1.3486801	0.00084
hematopoietic stem cell 315	1.3662752	0.00122
fetal thymus 261	1.4365210	0.00164
hematopoietic stem cell 308	1.3198641	0.00214
hematopoietic stem cell 316	1.3606893	0.00226
fetal thymus 257	1.4404332	0.00230
hematopoietic stem cell 307	1.3149568	0.00276
hematopoietic stem cell 306	1.3232478	0.00280
hematopoietic stem cell 305	1.3290412	0.00286
hematopoietic stem cell 318	1.3400613	0.00290
Mobilized CD56 324	1.3700374	0.00316
hematopoietic stem cell 311	1.3001759	0.00406
fetal thymus 262	1.4674043	0.00428
CD56 17	1.3150387	0.00810
CD14 4	1.3078269	0.00914
CD14 3	1.3243945	0.00996
CD56 18	1.3519364	0.01122
placenta 199	1.2488380	0.01316
CD8 21	1.3551490	0.01472
fetal large intestine 61	1.3279604	0.01482
Mobilized CD4 322	1.3505297	0.01488
Mobilized CD4 323	1.3411099	0.01502
placenta 200	1.2180036	0.01720
placenta 198	1.2236471	0.01752
CD19 7	1.3277600	0.02058
CD8 19	1.3467226	0.02114
CD4 14	1.2777750	0.02128
fetal large intestine 67	1.2443576	0.02254
CD19 5	1.2919983	0.02312
hematopoietic stem cell 319	1.2427903	0.02360
Mobilized CD8 325	1.3170656	0.02372
CD8 20	1.2762889	0.02390
CD3 10	1.2743070	0.02778
CD3 9	1.2790526	0.03094
CD3 12	1.2960732	0.03136
CD3 11	1.2536288	0.03258
CD19 6	1.2803398	0.03320
Mobilized CD3 321	1.2974228	0.03554
CD8 22	1.2819086	0.03690
CD4 13	1.2687754	0.03948
small intestine 342	1.2056953	0.04142
CD4 15	1.3298622	0.04154
placenta 197	1.2041456	0.04166
fetal small intestine 75	1.3269319	0.04352
CD4 16	1.2778088	0.04426
fetal small intestine 77	1.1996370	0.04804
fetal small intestine 72	1.1910906	0.04902
placenta 196	1.1932560	0.04950
placenta 201	1.2016106	0.04986
fetal small intestine 79	1.1970728	0.05472
fetal small intestine 69	1.1964644	0.05990
fetal large intestine 62	1.1928916	0.06166
fetal small intestine 73	1.1705516	0.07436
Mobilized CD3 320	1.2222546	0.07670
fetal small intestine 80	1.1618762	0.07754
fetal small intestine 74	1.1616545	0.07802
fetal small intestine 71	1.1695776	0.07934
fetal large intestine 63	1.1698977	0.08084
fetal large intestine 64	1.1556271	0.08088
fetal large intestine 59	1.1702705	0.08170
fetal small intestine 76	1.1726452	0.08176
fetal large intestine 60	1.1624721	0.08246
fetal small intestine 78	1.1443500	0.08420
fetal large intestine 54	1.1621447	0.08502
fetal small intestine 68	1.1498225	0.08516
fetal large intestine 58	1.1373505	0.09658
fetal large intestine 65	1.1495959	0.10172
CD20 8	1.1377520	0.10218
fetal large intestine 66	1.1538716	0.10414
stomach 283	1.1579446	0.10426
fetal large intestine 55	1.1310450	0.11622
fetal adrenal gland 26	1.1319916	0.11820
ES derived MSC 289	1.1212672	0.12420
fibroblast 332	1.1028128	0.12836
ES derived MSC 290	1.1179983	0.12868
fetal large intestine 56	1.1282546	0.12916
fibroblast 331	1.1016918	0.13030
fetal small intestine 70	1.1128853	0.13086
fibroblast 329	1.1043959	0.13120
heart 297	1.1323755	0.13536
muscle 341	1.1400571	0.13906
fetal heart 51	1.1097957	0.15254
fibroblast 330	1.0924576	0.16326
fetal large intestine 53	1.1285584	0.16332
fetal adrenal gland 27	1.1059113	0.16790
ovary 326	1.1299889	0.17274
fetal heart 46	1.1067433	0.17346
fetal large intestine 57	1.0976612	0.17540
pancreas 327	1.1138561	0.18124
pancreas 328	1.1064215	0.18830
ES cells 294	1.1150443	0.18840
fetal heart 52	1.0890656	0.18850
fetal heart 42	1.0963023	0.18862
ES cells 296	1.1144016	0.19012
fetal heart 49	1.0951636	0.19360
ES derived NP 291	1.1096746	0.19986
ES derived NP 292	1.1039613	0.20052
fetal adrenal gland 24	1.1012991	0.20114
fetal heart 43	1.0843699	0.20222
fetal kidney 89	1.0903980	0.21206
ES cells 295	1.1011626	0.21240
fetal heart 48	1.0843690	0.21500
fetal heart 50	1.0834405	0.21878
fetal kidney 106	1.0818790	0.22596
fetal adrenal gland 25	1.0852618	0.22658
fetal heart 44	1.0903203	0.23904
fetal stomach 242	1.1018105	0.24190
fetal stomach 252	1.0828927	0.24296
fetal lung 138	1.0671685	0.24578
fetal spinal cord 237	1.0688890	0.24694
fetal lung 128	1.0581687	0.24802
fetal lung 141	1.0618669	0.25068
fetal heart 45	1.0723336	0.25106
fetal ovary 195	1.0860361	0.25448
fetal stomach 250	1.0823314	0.25762
fetal kidney 102	1.0745670	0.26288
fetal stomach 244	1.0736845	0.26350
fetal lung 129	1.0608237	0.26540
fetal stomach 253	1.0770817	0.26712
fetal lung 132	1.0597887	0.26960
fetal kidney 86	1.0793063	0.27014
fetal lung 121	1.0556769	0.27150
fetal stomach 240	1.0777182	0.27160
fetal stomach 248	1.0734690	0.27310
fetal adrenal gland 28	1.0567748	0.27472
fetal renal pelvis 217	1.0672993	0.27564
ES cells 293	1.0699552	0.27570
fetal lung 122	1.0600624	0.27864
fetal renal pelvis 230	1.0700193	0.27896
fetal stomach 247	1.0790077	0.27944
fetal renal cortex 211	1.0665777	0.28076
fetal renal pelvis 220	1.0641102	0.28276
fetal lung 123	1.0559594	0.28334
fetal lung 124	1.0545239	0.28544
fetal fibroblast 280	1.0496385	0.28716
fetal stomach 241	1.0692563	0.28774
fetal lung 134	1.0550942	0.28882
fetal lung 301	1.0532154	0.29060
fetal lung 300	1.0516070	0.29240
fetal lung 117	1.0509611	0.29246
stomach 284	1.0644881	0.29260
fetal kidney 95	1.0580800	0.29580
fetal lung 140	1.0505074	0.29638
fetal kidney 90	1.0573965	0.29876
fetal renal pelvis 218	1.0700192	0.29892
fetal stomach 251	1.0619415	0.30070
fetal spinal cord 236	1.0549907	0.30072
breast 1	1.0451857	0.30324
fetal renal pelvis 219	1.0672732	0.30350
fetal lung 116	1.0448156	0.30500
fetal kidney 103	1.0549649	0.30554
fetal kidney 104	1.0548948	0.30726
melanocyte 337	1.0395872	0.30738
fetal lung 139	1.0498202	0.30996
iPS 302	1.0508313	0.31062
fetal lung 108	1.0500346	0.31192
fetal stomach 246	1.0585214	0.31310
fetal renal pelvis 227	1.0513485	0.31434
fetal renal cortex 202	1.0508875	0.31434
fetal renal pelvis 228	1.0551234	0.31550
iPS 304	1.0471222	0.31776
fetal heart 47	1.0533444	0.32154
fetal renal cortex 205	1.0487400	0.32282
fetal lung 299	1.0454597	0.32316
fetal stomach 249	1.0553632	0.32382
fetal fibroblast 273	1.0398856	0.32460
fetal lung 118	1.0391731	0.32542
fetal kidney 98	1.0485276	0.32708
fetal lung 120	1.0421734	0.32750
fetal kidney 93	1.0508899	0.32756
fetal renal pelvis 232	1.0497566	0.32816
fetal renal cortex 204	1.0448565	0.32926
fetal kidney 97	1.0463552	0.33236
fetal kidney 94	1.0481851	0.33288
fetal lung 110	1.0418962	0.33500
fetal brain 37	1.0373940	0.33622
fetal kidney 82	1.0480427	0.33714
fetal renal pelvis 233	1.0448949	0.33812
breast 2	1.0354304	0.33998
melanocyte 338	1.0334514	0.34080
fetal lung 115	1.0368942	0.34220
fetal testes 254	1.0427658	0.34352
fetal renal pelvis 216	1.0423695	0.34460
fetal kidney 107	1.0422309	0.34532
fetal fibroblast 278	1.0361123	0.34670
fetal lung 126	1.0352580	0.34734
fetal lung 298	1.0386652	0.34748
fetal lung 135	1.0379820	0.34750
fetal kidney 91	1.0386112	0.34856
fetal kidney 83	1.0463155	0.34880
fetal fibroblast 279	1.0338352	0.34966
fetal kidney 99	1.0402093	0.35078
iPS 303	1.0376237	0.35316
fetal lung 133	1.0335426	0.35372
fetal stomach 245	1.0433507	0.35392
fetal renal pelvis 221	1.0375820	0.35466
fetal fibroblast 269	1.0316879	0.35702
fetal renal cortex 203	1.0387544	0.35708
fetal kidney 100	1.0374418	0.35762
fetal brain 38	1.0324438	0.35914
fetal renal pelvis 223	1.0403575	0.35948
fetal kidney 87	1.0369065	0.36078
fetal renal pelvis 231	1.0355649	0.36170
fetal renal cortex 213	1.0355364	0.36212
ES derived trophoblast 288	1.0343734	0.36234
fetal stomach 243	1.0373970	0.36262
fetal adrenal gland 23	1.0330766	0.36610
fetal lung 119	1.0308407	0.36782
fetal renal pelvis 225	1.0352780	0.37030
fetal kidney 84	1.0351548	0.37168
fetal lung 109	1.0274958	0.37174
fetal lung 113	1.0289263	0.37344
fetal fibroblast 274	1.0277108	0.37542
melanocyte 340	1.0282008	0.37668
fetal renal pelvis 222	1.0328123	0.38064
fetal brain 41	1.0251758	0.38144
fetal lung 114	1.0255713	0.38630
fetal lung 127	1.0263148	0.38688
fetal brain 36	1.0249829	0.38754
fetal lung 125	1.0257918	0.38930
fetal lung 131	1.0225996	0.39654
fetal lung 137	1.0231030	0.39704
fetal renal cortex 207	1.0244499	0.40284
fetal fibroblast 267	1.0213111	0.40500
fetal renal pelvis 224	1.0233120	0.40666
fetal renal pelvis 226	1.0227558	0.40868
fetal kidney 105	1.0213004	0.41052
fetal lung 130	1.0203528	0.41132
fetal fibroblast 272	1.0185022	0.41502
fetal fibroblast 270	1.0183670	0.41512
fetal kidney 85	1.0186979	0.41836
fetal kidney 96	1.0190089	0.41914
fetal renal cortex 212	1.0198170	0.41968
fetal kidney 101	1.0190189	0.42078
fetal kidney 88	1.0175779	0.42258
fetal renal cortex 209	1.0188652	0.42420
fetal fibroblast 282	1.0157893	0.42438
fetal kidney 92	1.0180112	0.42536
fetal renal cortex 206	1.0162734	0.42706
fetal lung 112	1.0162438	0.42898
fetal fibroblast 277	1.0144797	0.43438
fetal brain 33	1.0142428	0.43508
fetal fibroblast 275	1.0127183	0.43822
fetal muscle 171	1.0131403	0.43904
fetal renal cortex 208	1.0136752	0.43956
fetal lung 136	1.0126784	0.43994
fetal muscle 154	1.0134043	0.44554
fetal muscle 194	1.0114020	0.44586
fetal fibroblast 268	1.0106999	0.44742
ES derived mesoderm 286	1.0093738	0.44996
fetal muscle 160	1.0108123	0.45230
fetal renal cortex 215	1.0089712	0.45814
fetal testes 255	1.0060996	0.46694
fetal renal cortex 214	1.0055355	0.46876
fetal muscle 145	1.0041178	0.47172
fetal muscle 191	1.0045675	0.47350
fetal muscle 150	1.0036238	0.47586
fetal renal cortex 210	1.0028181	0.47880
fetal fibroblast 276	1.0034554	0.47894
fetal muscle 173	1.0026219	0.48076
keratinocyte 336	1.0021792	0.48662
fetal renal pelvis 229	0.9999280	0.48990
fetal muscle 190	0.9991929	0.49006
fetal muscle 153	0.9999111	0.49040
ES derived mesoderm 285	0.9995744	0.49108
keratinocyte 333	1.0005784	0.49364
fetal spinal cord 239	0.9983171	0.49816
fetal muscle 169	0.9978026	0.50098
fetal fibroblast 271	0.9979854	0.50260
keratinocyte 335	0.9988250	0.50318
fetal spinal cord 235	0.9970436	0.50322
fetal muscle 175	0.9964778	0.50406
fetal thymus 263	0.9954832	0.50458
fetal muscle 159	0.9948532	0.50548
fetal muscle 177	0.9929800	0.51052
fetal muscle 144	0.9932384	0.51248
fetal lung 111	0.9955341	0.51350
fetal muscle 172	0.9939771	0.51898
melanocyte 339	0.9948781	0.51964
fetal muscle 168	0.9916448	0.52206
fetal muscle 163	0.9887218	0.53100
fetal lung 142	0.9907862	0.53396
fetal muscle 166	0.9882773	0.53438
fetal muscle 182	0.9885535	0.53482
fetal muscle 148	0.9874657	0.53486
fetal brain 32	0.9895580	0.53710
fetal fibroblast 281	0.9907112	0.53726
fetal brain 34	0.9900974	0.54124
fetal muscle 147	0.9860898	0.54124
fetal muscle 149	0.9836386	0.54838
fetal muscle 180	0.9833008	0.55234
fetal muscle 161	0.9801245	0.56094
fetal brain 40	0.9846095	0.56316
fetal skin 234	0.9797293	0.56564
fetal muscle 155	0.9763103	0.57050
fetal kidney 81	0.9786135	0.57744
fetal muscle 178	0.9742050	0.57920
fetal brain 39	0.9805618	0.58106
fetal muscle 189	0.9752043	0.58450
fetal muscle 188	0.9743504	0.58794
fetal muscle 174	0.9732920	0.58898
fetal muscle 179	0.9728506	0.59382
fetal muscle 193	0.9701289	0.59614
fetal muscle 187	0.9707331	0.60710
keratinocyte 334	0.9739078	0.62012
fetal muscle 151	0.9622191	0.62020
fetal muscle 156	0.9630953	0.62438
fetal muscle 183	0.9645382	0.62772
ES derived trophoblast 287	0.9602030	0.63050
fetal spinal cord 238	0.9617933	0.63490
fetal muscle 152	0.9620780	0.63496
fetal muscle 186	0.9615554	0.63536
fetal muscle 170	0.9612207	0.63944
fetal muscle 184	0.9629947	0.64078
fetal muscle 167	0.9601711	0.64276
fetal muscle 181	0.9576956	0.64620
fetal muscle 164	0.9563292	0.64968
fetal muscle 185	0.9520759	0.66000
fetal brain 31	0.9675113	0.66248
fetal muscle 192	0.9517569	0.67274
fetal muscle 158	0.9508426	0.67922
fetal muscle 165	0.9507313	0.67982
fetal muscle 146	0.9494665	0.68004
fetal muscle 157	0.9517477	0.68090
fetal brain 29	0.9645581	0.68152
fetal brain 35	0.9611726	0.68546
fetal muscle 143	0.9465014	0.69922
fetal muscle 162	0.9386366	0.71848
fetal muscle 176	0.9332720	0.73782
fetal brain 30	0.9435082	0.78080

Chronic lymphocytic leukemia 1

DHS sample	fold enrichment	p value
Mobilized CD56 324	1.5605461	0.02434
CD3 11	1.4879888	0.03894
CD8 20	1.4941089	0.04134
fetal thymus 256	1.3952230	0.06486
CD3 10	1.3920891	0.08502
CD4 14	1.3664211	0.08536
Mobilized CD3 320	1.4246890	0.09256
CD19 5	1.3784365	0.09514
fetal thymus 265	1.3408986	0.10738
Mobilized CD4 322	1.3726064	0.11266
CD3 9	1.3424557	0.12038
CD19 6	1.3348542	0.13400
CD8 21	1.3223119	0.14164
CD4 13	1.3184586	0.14164
fetal thymus 266	1.2794765	0.14642
fetal thymus 264	1.2759934	0.14696
CD56 17	1.2521653	0.14964
CD14 4	1.2473905	0.15514
CD3 12	1.2977193	0.16210
fetal thymus 258	1.2512554	0.16828
CD56 18	1.2697688	0.17782
fetal thymus 260	1.2471731	0.18058
CD8 22	1.2611395	0.18200
Mobilized CD3 321	1.2785086	0.18268
fetal large intestine 61	1.2237884	0.20618
CD8 19	1.2439950	0.21312
CD19 7	1.2005659	0.23498
Mobilized CD4 323	1.1901463	0.24542
CD14 3	1.1715906	0.24768
fetal thymus 259	1.1732843	0.25854
Mobilized CD8 325	1.1799009	0.25872
fibroblast 331	1.1112199	0.27036
fibroblast 332	1.1049609	0.28058
fetal small intestine 75	1.1681791	0.28626
CD4 16	1.1384732	0.29764
hematopoietic stem cell 310	1.0790929	0.32122
hematopoietic stem cell 317	1.0781706	0.32346
fetal thymus 261	1.1119009	0.32398
small intestine 342	1.0908423	0.33000
keratinocyte 336	1.0570379	0.35610
fetal small intestine 70	1.0673094	0.35722
fetal lung 128	1.0519358	0.37028
hematopoietic stem cell 313	1.0570779	0.37134
fetal large intestine 58	1.0563002	0.37698
placenta 198	1.0559367	0.37818
fibroblast 330	1.0520917	0.38466
hematopoietic stem cell 308	1.0496021	0.38900
ES derived MSC 289	1.0502461	0.38908
hematopoietic stem cell 314	1.0496236	0.39048
fetal fibroblast 275	1.0433364	0.39438
fetal adrenal gland 28	1.0423682	0.39922
melanocyte 338	1.0388543	0.40144
fetal lung 141	1.0352817	0.40516
keratinocyte 335	1.0363858	0.40732
fetal small intestine 78	1.0370441	0.41222
fetal fibroblast 276	1.0335216	0.41356
CD4 15	1.0316639	0.41392
hematopoietic stem cell 318	1.0311908	0.42618
fetal thymus 257	1.0273169	0.43260
placenta 200	1.0227621	0.43898
hematopoietic stem cell 312	1.0225697	0.44040
fibroblast 329	1.0219591	0.44300
ES derived MSC 290	1.0182302	0.44962
fetal adrenal gland 23	1.0150358	0.45082
fetal lung 115	1.0087351	0.46690
fetal fibroblast 272	1.0078727	0.47148
fetal fibroblast 280	1.0088495	0.47238
fetal large intestine 57	1.0045549	0.47262
fetal large intestine 64	1.0028131	0.47662
hematopoietic stem cell 309	1.0007869	0.48412
fetal lung 116	1.0020499	0.48554
fetal adrenal gland 25	0.9930237	0.48866
fetal large intestine 67	0.9929585	0.49188
fetal fibroblast 271	0.9985037	0.49448
fetal adrenal gland 26	0.9922356	0.49530
fetal adrenal gland 24	0.9850595	0.49708
fetal adrenal gland 27	0.9892050	0.49798
fetal small intestine 73	0.9854046	0.49928
hematopoietic stem cell 306	0.9880699	0.50248
fetal fibroblast 278	0.9930785	0.50562
fetal lung 133	0.9895265	0.50650
fetal thymus 262	0.9606889	0.50652
fetal large intestine 54	0.9782085	0.51146
hematopoietic stem cell 315	0.9846835	0.51272
fetal lung 114	0.9866969	0.51374
hematopoietic stem cell 307	0.9794308	0.51666
fetal fibroblast 282	0.9873142	0.51952
fetal large intestine 63	0.9688875	0.52682
fetal small intestine 69	0.9623888	0.53148
breast 2	0.9777322	0.53228
breast 1	0.9783389	0.53510
fetal lung 109	0.9754185	0.54336
fetal fibroblast 279	0.9750357	0.54706
fetal small intestine 68	0.9633844	0.54928
fetal lung 121	0.9711746	0.55010
fetal lung 111	0.9673903	0.55212
fetal lung 301	0.9673841	0.55512
fetal large intestine 65	0.9532008	0.55564
melanocyte 337	0.9736610	0.55790
fetal lung 120	0.9625531	0.55832
fetal fibroblast 268	0.9667437	0.56338
fetal fibroblast 273	0.9672905	0.56476
fetal fibroblast 274	0.9648173	0.56714
fetal lung 137	0.9607860	0.56848
hematopoietic stem cell 316	0.9464471	0.57178
hematopoietic stem cell 311	0.9493209	0.57434
placenta 199	0.9468621	0.57654
fetal small intestine 80	0.9482094	0.57662
fetal small intestine 76	0.9356423	0.58046
fetal fibroblast 277	0.9567170	0.58346
fetal lung 127	0.9462999	0.58732
keratinocyte 334	0.9585327	0.58742
fetal lung 135	0.9448988	0.58934
placenta 197	0.9332309	0.59052
fetal lung 126	0.9523661	0.59132
fetal muscle 194	0.9469808	0.59382
fetal large intestine 66	0.9233061	0.59842
fetal small intestine 77	0.9265541	0.59938
fetal muscle 154	0.9349457	0.60096
fetal lung 300	0.9448809	0.60216
hematopoietic stem cell 305	0.9342855	0.60392
hematopoietic stem cell 319	0.9250180	0.60632
fetal fibroblast 269	0.9494923	0.60706
fetal small intestine 72	0.9264523	0.60776
fetal fibroblast 281	0.9468715	0.60846
fetal lung 138	0.9365130	0.61018
fetal lung 113	0.9412716	0.61042
fetal large intestine 60	0.9213037	0.61472
placenta 201	0.9137631	0.61484
CD20 8	0.9290445	0.61596
fetal small intestine 71	0.9163428	0.61686
fetal small intestine 74	0.9195182	0.62048
fetal lung 131	0.9412134	0.62066
fetal fibroblast 270	0.9397769	0.62168
melanocyte 339	0.9421821	0.62294
heart 297	0.9182852	0.62324
fetal large intestine 55	0.9190115	0.62606
fetal renal cortex 212	0.9193143	0.62644
fetal lung 134	0.9248233	0.63170
fetal lung 136	0.9251925	0.63252
fetal small intestine 79	0.9018988	0.63306
fetal renal cortex 209	0.9098677	0.63516
fetal lung 132	0.9250628	0.63576
keratinocyte 333	0.9407732	0.63670
fetal lung 124	0.9237920	0.63714
fetal large intestine 62	0.8992166	0.63716
fetal lung 117	0.9264259	0.64248
fetal large intestine 59	0.8950016	0.64284
stomach 284	0.8960001	0.64412
fetal lung 299	0.9212045	0.64484
fetal lung 123	0.9150430	0.64826
stomach 283	0.8880186	0.64886
fetal muscle 179	0.9165355	0.65018
fetal renal pelvis 225	0.8975326	0.65134
fetal lung 110	0.9071772	0.65770
fetal renal pelvis 223	0.8890898	0.65972
fetal renal cortex 210	0.8864455	0.66376
fetal stomach 252	0.8801448	0.66596
fetal muscle 170	0.9070207	0.66976
fetal muscle 149	0.8959229	0.67246
fetal large intestine 56	0.8881781	0.67320
fetal lung 122	0.8958661	0.67478
fetal muscle 168	0.8971121	0.67670
fetal muscle 191	0.8931122	0.67724
melanocyte 340	0.9116095	0.67750
fetal lung 108	0.8953378	0.67906
fetal kidney 98	0.8819618	0.68104
fetal renal cortex 205	0.8859813	0.68144
fetal muscle 188	0.8951342	0.68234
fetal lung 119	0.9092929	0.68294
fetal testes 254	0.8856500	0.68430
fetal lung 298	0.8981812	0.68552
fetal muscle 172	0.9037359	0.68774
fetal stomach 251	0.8666305	0.68890
fetal kidney 101	0.8834333	0.69176
fetal muscle 156	0.8922787	0.69390
placenta 196	0.8700057	0.69392
fetal muscle 145	0.8701010	0.69406
fetal renal cortex 208	0.8726350	0.69434
fetal lung 125	0.8896286	0.69604
fetal renal cortex 203	0.8717161	0.70076
fetal stomach 245	0.8522498	0.70202
fetal stomach 244	0.8614905	0.70230
fetal renal pelvis 218	0.8331641	0.70388
fetal fibroblast 267	0.8969425	0.70674
fetal muscle 193	0.8764360	0.70688
fetal renal cortex 202	0.8739370	0.70694
fetal stomach 243	0.8680306	0.70762
fetal kidney 90	0.8659689	0.70868
fetal muscle 171	0.8831374	0.70966
fetal kidney 107	0.8648747	0.71334
fetal lung 130	0.8866484	0.71462
fetal lung 118	0.8983326	0.71472
fetal stomach 249	0.8316588	0.71488
fetal lung 142	0.8927123	0.71512
fetal muscle 166	0.8853493	0.71520
muscle 341	0.8501331	0.71676
fetal stomach 247	0.8164753	0.71678
fetal kidney 105	0.8689242	0.71956
fetal muscle 192	0.8773349	0.71966
fetal muscle 146	0.8761286	0.72118
fetal renal cortex 211	0.8481475	0.72156
fetal renal pelvis 230	0.8459519	0.72158
fetal kidney 102	0.8493112	0.72338
fetal muscle 177	0.8561789	0.72442
fetal kidney 89	0.8562117	0.72502
fetal kidney 86	0.8224316	0.72722
fetal muscle 155	0.8614139	0.72930
fetal kidney 93	0.8460822	0.72944
fetal renal cortex 204	0.8651355	0.73310
fetal muscle 151	0.8612881	0.73464
fetal muscle 182	0.8685691	0.73710
fetal muscle 162	0.8678406	0.73744
fetal muscle 175	0.8644072	0.73924
fetal stomach 246	0.8168556	0.73974
fetal lung 129	0.8701532	0.74092
fetal large intestine 53	0.8187295	0.74102
fetal muscle 159	0.8533696	0.74200
fetal kidney 81	0.8638211	0.74214
fetal muscle 153	0.8543878	0.74322
fetal lung 139	0.8564030	0.74394
fetal muscle 167	0.8690548	0.74454
fetal kidney 104	0.8425822	0.74714
fetal kidney 106	0.8456133	0.74742
fetal renal pelvis 216	0.8386742	0.75004
fetal kidney 103	0.8328316	0.75200
fetal ovary 195	0.8040534	0.75258
fetal muscle 174	0.8474152	0.75402
fetal kidney 97	0.8380847	0.75546
fetal kidney 96	0.8466282	0.75614
fetal stomach 242	0.7770692	0.75700
fetal muscle 190	0.8335423	0.75738
fetal muscle 186	0.8532230	0.75750
fetal stomach 250	0.8042912	0.75870
fetal lung 140	0.8649440	0.75880
fetal heart 48	0.8502142	0.76198
fetal muscle 157	0.8656444	0.76206
fetal thymus 263	0.8429498	0.76300
fetal muscle 143	0.8666127	0.76462
fetal stomach 241	0.7971085	0.76490
ES derived NP 292	0.8259912	0.76562
fetal muscle 187	0.8549862	0.76682
fetal heart 47	0.8309342	0.76890
fetal muscle 144	0.8317052	0.76980
fetal kidney 88	0.8361503	0.77042
fetal renal pelvis 219	0.7861269	0.77044
fetal renal cortex 206	0.8347296	0.77134
pancreas 328	0.8213737	0.77202
fetal heart 46	0.8380795	0.77286
fetal kidney 94	0.8145057	0.77344
fetal stomach 253	0.7964354	0.77420
fetal skin 234	0.8430651	0.77484
fetal muscle 150	0.8382143	0.77496
fetal stomach 248	0.8076064	0.77518
fetal testes 255	0.8470392	0.77600
fetal renal pelvis 217	0.8198216	0.77602
fetal muscle 189	0.8375468	0.77636
fetal renal pelvis 229	0.8278939	0.77832
fetal heart 44	0.8030870	0.77924
ovary 326	0.7888448	0.77952
fetal heart 43	0.8508508	0.77958
fetal muscle 180	0.8406171	0.78026
fetal heart 50	0.8327182	0.78120
fetal muscle 181	0.8426532	0.78254
fetal muscle 163	0.8292859	0.78390
fetal renal pelvis 227	0.8195548	0.78442
fetal renal pelvis 222	0.8123256	0.78494
fetal heart 52	0.8462634	0.78628
fetal muscle 164	0.8338033	0.78872
fetal muscle 158	0.8409903	0.78944
fetal muscle 161	0.8256229	0.79138
fetal muscle 173	0.8251172	0.79214
fetal stomach 240	0.7721660	0.79388
fetal kidney 95	0.8102681	0.79416
fetal renal pelvis 233	0.8143576	0.79474
fetal kidney 92	0.8187141	0.79524
fetal renal pelvis 221	0.8171024	0.79566
fetal muscle 178	0.8295639	0.79642
fetal renal cortex 207	0.8100865	0.80116
fetal heart 42	0.8225148	0.80174
fetal heart 49	0.8212323	0.80386
fetal kidney 100	0.8086815	0.80432
fetal kidney 99	0.8024165	0.80832
fetal muscle 185	0.8123636	0.80838
fetal kidney 83	0.7782482	0.80962
fetal muscle 169	0.8228478	0.80966
fetal kidney 91	0.8186482	0.81160
fetal renal cortex 215	0.8138501	0.81210
fetal kidney 85	0.8253608	0.81392
fetal muscle 152	0.8281342	0.81440
fetal muscle 183	0.8214830	0.81686
pancreas 327	0.7714197	0.81690
fetal renal pelvis 224	0.8080680	0.81994
fetal renal pelvis 232	0.7854284	0.81996
fetal kidney 82	0.7772317	0.82112
fetal renal pelvis 228	0.7787925	0.82224
fetal muscle 165	0.8235314	0.82248
fetal renal pelvis 231	0.8073531	0.82340
fetal spinal cord 238	0.8172314	0.82480
fetal muscle 184	0.8211263	0.82582
fetal renal pelvis 226	0.7955478	0.82694
fetal kidney 84	0.7807944	0.82998
fetal muscle 147	0.8004722	0.83038
fetal renal cortex 214	0.7871759	0.83048
fetal spinal cord 237	0.8175000	0.83066
fetal lung 112	0.8171914	0.83426
fetal muscle 148	0.7967812	0.83722
ES cells 296	0.7637835	0.83792
fetal renal cortex 213	0.7946531	0.83984
ES cells 293	0.7705196	0.84024
fetal muscle 160	0.7937336	0.84518
fetal spinal cord 239	0.8013229	0.84578
fetal muscle 176	0.8119306	0.84688
ES derived NP 291	0.7515743	0.85322
ES derived mesoderm 285	0.8162070	0.86090
ES derived trophoblast 287	0.7719101	0.86450
fetal heart 45	0.7753604	0.86474
fetal spinal cord 236	0.7776257	0.86558
fetal brain 36	0.8082571	0.87454
fetal kidney 87	0.7566839	0.87636
fetal heart 51	0.7679718	0.88006
fetal spinal cord 235	0.7661552	0.88722
ES cells 295	0.7113338	0.89628
fetal brain 37	0.7961751	0.89718
ES derived mesoderm 286	0.8018713	0.89858
fetal renal pelvis 220	0.7183516	0.90692
ES cells 294	0.6999286	0.91026
fetal brain 31	0.8079208	0.91626
fetal brain 40	0.7780169	0.92188
iPS 302	0.7266909	0.92242
fetal brain 34	0.7736306	0.93246
iPS 304	0.7293987	0.93376
ES derived trophoblast 288	0.7164537	0.93678
fetal brain 38	0.7408616	0.94122
fetal brain 41	0.7412794	0.94698
fetal brain 33	0.7249930	0.94802
fetal brain 39	0.7358827	0.95850
iPS 303	0.6821708	0.95894
fetal brain 32	0.7229226	0.96190
fetal brain 29	0.7475365	0.97036
fetal brain 35	0.7266057	0.97360
fetal brain 30	0.7317833	0.98076

Erythrocyte phenotypes

DHS sample	fold enrichment	p value
hematopoietic stem cell 310	1.6249377	0.00008
fetal thymus 258	1.7781446	0.00012
hematopoietic stem cell 314	1.6033699	0.00024
fetal thymus 256	1.7356614	0.00034
hematopoietic stem cell 317	1.5154938	0.00034
Mobilized CD56 324	1.7181859	0.00062
fetal thymus 265	1.7274447	0.00068
hematopoietic stem cell 309	1.5343573	0.00092
hematopoietic stem cell 312	1.5114140	0.00102
fetal thymus 264	1.6482872	0.00120
hematopoietic stem cell 308	1.5026180	0.00144
hematopoietic stem cell 315	1.5412254	0.00148
hematopoietic stem cell 305	1.5358093	0.00148
hematopoietic stem cell 313	1.4919684	0.00148
fetal thymus 259	1.7008943	0.00154
hematopoietic stem cell 307	1.5151652	0.00154
fetal thymus 260	1.6565111	0.00166
hematopoietic stem cell 318	1.5415290	0.00180
fetal thymus 266	1.6272420	0.00196
fetal large intestine 67	1.5374673	0.00208
placenta 198	1.4739045	0.00246
hematopoietic stem cell 306	1.4904216	0.00248
CD14 3	1.6167175	0.00270
placenta 200	1.4472069	0.00270
fetal thymus 261	1.6174691	0.00284
CD14 4	1.5650664	0.00302
fetal thymus 257	1.6372534	0.00304
hematopoietic stem cell 316	1.5221776	0.00336
hematopoietic stem cell 311	1.4598133	0.00372
placenta 199	1.4639748	0.00444
fetal small intestine 80	1.4567591	0.00474
Mobilized CD4 323	1.6222727	0.00480
small intestine 342	1.4732574	0.00500
CD19 5	1.5692566	0.00642
CD56 17	1.4872050	0.00688
CD8 21	1.5985028	0.00734
fetal large intestine 61	1.5664545	0.00762
Mobilized CD4 322	1.6003976	0.00774
CD56 18	1.5658139	0.00852
fetal thymus 262	1.6477645	0.00866
fetal small intestine 78	1.3831917	0.00874
fetal small intestine 77	1.4403735	0.00880
CD3 9	1.5543955	0.00918
fetal small intestine 68	1.4027832	0.00930
CD4 14	1.4929468	0.00938
fetal small intestine 74	1.4266780	0.00948
fetal large intestine 55	1.4024418	0.00980
CD3 10	1.5147261	0.01006
placenta 197	1.4391542	0.01102
CD4 13	1.5631758	0.01104
CD3 11	1.4842779	0.01108
CD19 7	1.5715106	0.01114
fetal small intestine 72	1.4191557	0.01116
fetal large intestine 64	1.4036587	0.01116
Mobilized CD8 325	1.5481536	0.01240
fetal large intestine 58	1.3769454	0.01256
CD20 8	1.3795575	0.01342
fetal large intestine 60	1.4061826	0.01364
CD3 12	1.5410124	0.01440
fetal large intestine 54	1.4089887	0.01440
fetal large intestine 63	1.4113058	0.01502
fetal small intestine 69	1.4246996	0.01534
CD8 20	1.4618053	0.01596
Mobilized CD3 320	1.5349993	0.01606
hematopoietic stem cell 319	1.3944082	0.01614
fetal large intestine 56	1.3769414	0.01802
fetal small intestine 73	1.3870556	0.01832
CD8 19	1.5516745	0.01894
fetal small intestine 79	1.3975502	0.01954
fetal large intestine 57	1.3379947	0.02014
CD19 6	1.4862792	0.02028
stomach 283	1.4127393	0.02082
fetal small intestine 70	1.3172141	0.02106
fetal large intestine 62	1.3952836	0.02136
CD4 15	1.5844800	0.02242
fetal small intestine 76	1.3779002	0.02302
placenta 196	1.3643388	0.02356
Mobilized CD3 321	1.4973604	0.02530
fetal large intestine 65	1.3572874	0.02552
stomach 284	1.3759428	0.02632
placenta 201	1.3575451	0.02838
CD8 22	1.4538468	0.02952
fetal small intestine 71	1.3457321	0.03008
CD4 16	1.4587547	0.03264
fetal small intestine 75	1.5306429	0.03520
fetal large intestine 59	1.3456150	0.03722
fetal large intestine 66	1.3251102	0.04160
fetal adrenal gland 26	1.2842535	0.04524
fetal large intestine 53	1.3356171	0.05020
pancreas 328	1.3044008	0.05124
pancreas 327	1.2962095	0.06276
muscle 341	1.2991539	0.06374
heart 297	1.2706129	0.06376
fetal adrenal gland 27	1.2527544	0.06396
ovary 326	1.3277808	0.06436
fetal adrenal gland 28	1.2145370	0.07164
ES derived MSC 289	1.2359271	0.07562
fetal lung 134	1.2203953	0.08444
melanocyte 338	1.1809589	0.08466
fetal adrenal gland 25	1.2368947	0.08642
fetal lung 129	1.2073025	0.08658
melanocyte 337	1.1717687	0.09164
ES derived trophoblast 288	1.1932302	0.09294
fetal stomach 252	1.2495486	0.09360
fetal adrenal gland 24	1.2426006	0.09452
fetal stomach 248	1.2468034	0.09538
fetal stomach 244	1.2439333	0.09662
fetal stomach 242	1.2934594	0.09754
melanocyte 340	1.1855710	0.09992
fetal lung 121	1.1775607	0.10350
fetal lung 133	1.1776560	0.10838
fetal lung 128	1.1636281	0.10896
fetal heart 44	1.2295715	0.10904
fetal ovary 195	1.2480894	0.11076
fetal lung 117	1.1805340	0.11118
fetal heart 46	1.1974597	0.11238
ES derived MSC 290	1.1962805	0.11290
fetal stomach 247	1.2620645	0.11434
fetal lung 141	1.1695528	0.11436
fetal lung 108	1.1914002	0.11670
melanocyte 339	1.1629152	0.11712
fetal lung 109	1.1570132	0.11898
fetal lung 120	1.1751718	0.11998
fetal lung 139	1.1879576	0.12146
fibroblast 332	1.1652626	0.12328
fetal lung 124	1.1729827	0.12490
fetal adrenal gland 23	1.1707423	0.12646
fetal brain 36	1.1431887	0.13010
fetal stomach 241	1.2221447	0.13064
fibroblast 330	1.1635901	0.13170
fetal renal cortex 207	1.1851382	0.13272
fibroblast 329	1.1604254	0.13384
fetal lung 138	1.1669455	0.13498
fetal stomach 251	1.1950179	0.13638
fetal lung 130	1.1627759	0.13672
fetal stomach 243	1.1905678	0.13754
fetal heart 47	1.1869408	0.13916
fetal kidney 106	1.1789300	0.13964
fibroblast 331	1.1549992	0.14022
keratinocyte 335	1.1310465	0.14112
fetal stomach 240	1.2115412	0.14446
fetal lung 127	1.1612362	0.14826
fetal heart 45	1.1643587	0.14954
fetal fibroblast 278	1.1431658	0.15142
fetal stomach 250	1.1991400	0.15354
fetal renal pelvis 220	1.1764155	0.15360
fetal lung 132	1.1521840	0.15506
fetal heart 51	1.1522909	0.16010
fetal lung 137	1.1421669	0.16018
fetal stomach 246	1.1901860	0.16020
keratinocyte 333	1.1175795	0.16072
ES derived NP 291	1.1817270	0.16356
fetal lung 140	1.1398039	0.16520
ES cells 294	1.1813264	0.16562
fetal lung 123	1.1467717	0.16598
fetal lung 110	1.1490137	0.16714
fetal spinal cord 236	1.1485662	0.16868
keratinocyte 336	1.1162888	0.16966
fetal lung 299	1.1466667	0.17068
fetal heart 50	1.1445857	0.17344
fetal testes 255	1.1425565	0.17542
fetal heart 43	1.1363632	0.17560
fetal heart 52	1.1311451	0.17896
ES cells 295	1.1689639	0.18092
fetal stomach 245	1.1651518	0.18272
fetal muscle 149	1.1495203	0.18322
fetal heart 48	1.1372277	0.18552
fetal heart 49	1.1403501	0.18570
ES derived NP 292	1.1594667	0.18574
fetal lung 131	1.1183910	0.18758
fetal spinal cord 237	1.1265284	0.18898
fetal stomach 253	1.1629159	0.19300
fetal fibroblast 267	1.1214916	0.19552
fetal lung 125	1.1296586	0.19734
fetal lung 301	1.1266281	0.19836
fetal kidney 100	1.1365023	0.20272
fetal spinal cord 235	1.1209298	0.20332
fetal fibroblast 273	1.1108491	0.20370
fetal lung 122	1.1274441	0.20532
fetal fibroblast 280	1.1098582	0.20806
fetal heart 42	1.1249797	0.20840
fetal fibroblast 274	1.1093249	0.21178
fetal stomach 249	1.1521214	0.21226
fetal lung 142	1.1078268	0.21452
fetal lung 111	1.1121522	0.21716
fetal muscle 153	1.1325045	0.21720
fetal lung 112	1.1130469	0.21940
fetal kidney 91	1.1195223	0.21990
fetal renal cortex 213	1.1193351	0.22044
fetal renal pelvis 219	1.1505208	0.22150
fetal lung 116	1.1020564	0.22180
fetal renal pelvis 228	1.1335226	0.22274
fetal lung 126	1.1025101	0.22446
fetal kidney 84	1.1238792	0.22614
fetal lung 114	1.1013002	0.22674
fetal brain 41	1.0954335	0.22708
breast 2	1.0978633	0.22788
fetal fibroblast 279	1.1028451	0.22824
keratinocyte 334	1.0872318	0.22962
fetal lung 115	1.1027783	0.23028
fetal lung 298	1.1128023	0.23058
fetal renal cortex 202	1.1151196	0.23130
fetal renal pelvis 227	1.1148203	0.23256
fetal kidney 89	1.1189439	0.23290
fetal lung 136	1.1084203	0.23330
fetal muscle 160	1.1156569	0.23338
breast 1	1.0941044	0.23350
fetal fibroblast 269	1.0975093	0.23428
ES cells 296	1.1295418	0.23440
ES derived trophoblast 287	1.1153301	0.23598
fetal kidney 95	1.1160503	0.23684
fetal brain 38	1.0934472	0.23760
fetal renal cortex 211	1.1214486	0.23920
fetal kidney 92	1.1077161	0.23984
fetal fibroblast 277	1.0986488	0.24054
fetal renal pelvis 231	1.1065319	0.24262
fetal testes 254	1.1116963	0.24470
fetal kidney 83	1.1236616	0.24734
fetal fibroblast 270	1.0930037	0.24762
fetal lung 135	1.1017711	0.24788
fetal fibroblast 272	1.0912841	0.24868
fetal lung 118	1.0887532	0.25166
fetal fibroblast 268	1.0867644	0.25374
fetal fibroblast 281	1.0875834	0.25506
fetal renal pelvis 217	1.1099180	0.25528
fetal renal pelvis 232	1.1090376	0.25620
fetal skin 234	1.1031469	0.25754
fetal renal pelvis 230	1.1112659	0.25898
fetal kidney 85	1.0889881	0.26484
fetal spinal cord 239	1.0894650	0.26666
ES derived mesoderm 285	1.0820330	0.26706
fetal fibroblast 282	1.0805829	0.26722
fetal renal pelvis 226	1.0955831	0.26768
fetal muscle 154	1.1021211	0.26834
ES derived mesoderm 286	1.0760471	0.26980
ES cells 293	1.1037641	0.27020
fetal muscle 172	1.0833645	0.27046
fetal lung 113	1.0830022	0.27076
fetal kidney 103	1.0962118	0.27186
fetal lung 300	1.0850732	0.27588
fetal muscle 190	1.0939435	0.27866
fetal kidney 99	1.0914248	0.28066
fetal fibroblast 276	1.0763697	0.28166
fetal brain 37	1.0719022	0.28168
fetal muscle 194	1.0785119	0.28402
fetal renal pelvis 224	1.0847794	0.28404
fetal renal cortex 204	1.0828723	0.28654
fetal kidney 86	1.1024275	0.28752
fetal renal cortex 206	1.0864347	0.28884
fetal brain 40	1.0650535	0.29000
fetal renal cortex 205	1.0839346	0.29100
fetal renal pelvis 221	1.0824211	0.29234
fetal kidney 87	1.0841775	0.29340
fetal kidney 94	1.0873609	0.29462
fetal fibroblast 271	1.0717665	0.29504
fetal lung 119	1.0730476	0.29636
fetal muscle 169	1.0817407	0.29694
fetal kidney 97	1.0834134	0.29714
iPS 304	1.0772579	0.29810
fetal kidney 102	1.0880279	0.29864
fetal kidney 104	1.0818588	0.29980
fetal spinal cord 238	1.0734151	0.30230
fetal fibroblast 275	1.0660575	0.30432
fetal renal cortex 214	1.0784154	0.30454
fetal muscle 159	1.0778807	0.31080
fetal kidney 107	1.0737982	0.31436
fetal renal pelvis 216	1.0757191	0.31488
fetal muscle 151	1.0739627	0.32000
fetal muscle 152	1.0666500	0.32246
fetal muscle 189	1.0674985	0.32440
fetal muscle 180	1.0702517	0.32448
fetal muscle 179	1.0674301	0.32498
fetal renal pelvis 218	1.0832333	0.32516
fetal renal cortex 212	1.0660839	0.32648
fetal kidney 98	1.0697907	0.32820
fetal renal cortex 203	1.0679842	0.32870
fetal muscle 171	1.0633692	0.33040
fetal brain 33	1.0561007	0.33084
fetal renal pelvis 233	1.0673816	0.33204
iPS 302	1.0632492	0.33340
fetal muscle 145	1.0686760	0.33524
fetal kidney 90	1.0646323	0.33724
fetal muscle 148	1.0623595	0.34214
fetal brain 34	1.0460393	0.34300
fetal kidney 93	1.0625561	0.34392
fetal kidney 82	1.0644298	0.34624
fetal kidney 88	1.0557336	0.34962
fetal renal cortex 215	1.0542671	0.35036
fetal thymus 263	1.0566300	0.35292
fetal muscle 147	1.0545995	0.35508
iPS 303	1.0524435	0.35608
fetal kidney 105	1.0522016	0.36118
fetal kidney 101	1.0514222	0.36238
fetal muscle 143	1.0485530	0.36256
fetal muscle 178	1.0522379	0.36280
fetal muscle 166	1.0487778	0.36792
fetal muscle 185	1.0503679	0.36916
fetal muscle 191	1.0480251	0.37182
fetal muscle 161	1.0466972	0.37402
fetal renal cortex 208	1.0480613	0.37428
fetal muscle 155	1.0482154	0.37522
fetal renal pelvis 229	1.0457621	0.37540
fetal muscle 173	1.0454409	0.37616
fetal muscle 150	1.0470761	0.37650
fetal brain 39	1.0358498	0.37686
fetal renal pelvis 222	1.0468938	0.37804
fetal muscle 168	1.0444718	0.38368
fetal muscle 181	1.0416818	0.38406
fetal muscle 144	1.0420007	0.38426
fetal muscle 183	1.0400697	0.38576
fetal muscle 177	1.0419159	0.38630
fetal renal cortex 210	1.0424983	0.38904
fetal muscle 163	1.0367410	0.39516
fetal muscle 174	1.0363414	0.39778
fetal renal pelvis 225	1.0367346	0.39838
fetal kidney 96	1.0329724	0.40516
fetal kidney 81	1.0301673	0.40836
fetal muscle 164	1.0331001	0.40920
fetal muscle 175	1.0287442	0.41764
fetal muscle 157	1.0233080	0.42720
fetal brain 32	1.0193757	0.42902
fetal renal pelvis 223	1.0237530	0.43068
fetal muscle 167	1.0186329	0.43846
fetal muscle 182	1.0198962	0.43936
fetal muscle 188	1.0183868	0.44014
fetal muscle 193	1.0172049	0.44372
fetal muscle 156	1.0176495	0.44402
fetal brain 35	1.0129683	0.44588
fetal renal cortex 209	1.0135763	0.45498
fetal muscle 186	1.0130511	0.45754
fetal muscle 165	1.0127484	0.45792
fetal muscle 146	1.0053339	0.47574
fetal muscle 184	1.0043987	0.47826
fetal brain 29	1.0026944	0.48330
fetal muscle 170	1.0016269	0.48444
fetal muscle 162	1.0024060	0.48468
fetal muscle 187	1.0015919	0.48628
fetal muscle 158	0.9991817	0.48980
fetal muscle 192	0.9811833	0.53650
fetal brain 31	0.9877761	0.53734
fetal muscle 176	0.9676231	0.57504
fetal brain 30	0.9472534	0.68824

Megakaryopoiesis and platelet formation

DHS sample	fold enrichment	p value
hematopoietic stem cell 310	1.2669312	0.00056
hematopoietic stem cell 314	1.2637536	0.00116
CD14 3	1.3352318	0.00180
hematopoietic stem cell 308	1.2486621	0.00294
CD14 4	1.2817214	0.00398
fetal heart 47	1.2731670	0.00444
hematopoietic stem cell 309	1.2402711	0.00450
hematopoietic stem cell 312	1.2313934	0.00512
melanocyte 338	1.1851013	0.00550
CD3 11	1.2771116	0.00570
Mobilized CD56 324	1.2863560	0.00626
fibroblast 330	1.1962574	0.00764
hematopoietic stem cell 307	1.2260713	0.00804
fibroblast 332	1.1860745	0.00810
fibroblast 331	1.1874924	0.00850
hematopoietic stem cell 311	1.2141361	0.00980
hematopoietic stem cell 317	1.1865339	0.00980
CD4 14	1.2562121	0.00988
CD8 20	1.2579774	0.01060
Mobilized CD4 323	1.2906636	0.01078
CD3 9	1.2776028	0.01082
hematopoietic stem cell 315	1.2223571	0.01088
hematopoietic stem cell 305	1.2184977	0.01088
fetal heart 45	1.2192140	0.01102
CD8 21	1.2881271	0.01164
small intestine 342	1.2252293	0.01208
melanocyte 337	1.1552696	0.01226
hematopoietic stem cell 306	1.2068094	0.01282
fetal lung 298	1.1960975	0.01314
CD3 10	1.2525096	0.01346
hematopoietic stem cell 316	1.2206965	0.01372
fetal heart 49	1.2077103	0.01376
fetal fibroblast 270	1.1738888	0.01392
fetal heart 46	1.2137634	0.01458
fetal heart 48	1.2027681	0.01504
keratinocyte 335	1.1552806	0.01516
fibroblast 329	1.1740601	0.01554
fetal fibroblast 278	1.1706298	0.01562
CD56 18	1.2707075	0.01574
Mobilized CD4 322	1.2743724	0.01590
fetal fibroblast 279	1.1674283	0.01592
fetal fibroblast 269	1.1640749	0.01612
fetal small intestine 78	1.1910720	0.01638
hematopoietic stem cell 318	1.2086233	0.01670
fetal fibroblast 272	1.1591278	0.01776
CD4 13	1.2592370	0.01810
fetal lung 301	1.1781528	0.01862
CD8 19	1.2821749	0.01910
hematopoietic stem cell 313	1.1792367	0.01962
hematopoietic stem cell 319	1.2067474	0.01996
fetal fibroblast 275	1.1525416	0.02016
fetal fibroblast 280	1.1567450	0.02038
fetal fibroblast 271	1.1553123	0.02122
CD19 5	1.2341838	0.02140
fetal large intestine 54	1.2044266	0.02156
CD19 6	1.2470968	0.02174
fetal lung 300	1.1677561	0.02234
fetal adrenal gland 27	1.1877340	0.02326
fetal fibroblast 281	1.1525567	0.02338
fetal fibroblast 282	1.1455490	0.02408
keratinocyte 334	1.1432027	0.02500
CD3 12	1.2504927	0.02538
keratinocyte 333	1.1394593	0.02538
fetal large intestine 60	1.1953489	0.02626
melanocyte 339	1.1461354	0.02630
CD56 17	1.2019048	0.02674
fetal heart 44	1.2126586	0.02710
keratinocyte 336	1.1361323	0.02732
fetal heart 42	1.1779981	0.02770
fetal large intestine 58	1.1726842	0.02832
ES derived MSC 289	1.1719373	0.02920
fetal lung 299	1.1639762	0.02954
fetal fibroblast 268	1.1406419	0.03024
fetal heart 43	1.1623613	0.03030
ES derived MSC 290	1.1669384	0.03042
fetal large intestine 61	1.2192006	0.03210
Mobilized CD8 325	1.2333270	0.03264
fetal lung 128	1.1343078	0.03410
fetal fibroblast 274	1.1394801	0.03466
CD20 8	1.1716715	0.03494
fetal adrenal gland 23	1.1590317	0.03526
fetal testes 255	1.1657697	0.03576
CD19 7	1.2234127	0.03660
fetal heart 52	1.1523727	0.03736
fetal large intestine 64	1.1700513	0.03740
fetal heart 51	1.1618572	0.03760
fetal fibroblast 277	1.1409761	0.03792
fetal large intestine 57	1.1617786	0.03862
fetal small intestine 70	1.1507386	0.03964
fetal small intestine 69	1.1878327	0.04008
fetal large intestine 55	1.1637479	0.04060
heart 297	1.1762559	0.04088
fetal muscle 172	1.1369134	0.04162
fetal heart 50	1.1610457	0.04218
fetal fibroblast 276	1.1285767	0.04230
fetal thymus 264	1.1894356	0.04312
Mobilized CD3 321	1.2258257	0.04342
fetal adrenal gland 28	1.1422738	0.04358
fetal fibroblast 273	1.1285999	0.04424
ES derived trophoblast 288	1.1435655	0.04452
fetal thymus 258	1.1921990	0.04516
melanocyte 340	1.1334169	0.04538
fetal thymus 266	1.1891291	0.04568
fetal small intestine 74	1.1595783	0.04936
fetal large intestine 65	1.1646893	0.04946
fetal kidney 81	1.1414605	0.04960
fetal thymus 265	1.1879030	0.05010
fetal adrenal gland 26	1.1533401	0.05010
fetal muscle 168	1.1524826	0.05164
CD8 22	1.2027669	0.05178
fetal small intestine 73	1.1648337	0.05208
fetal small intestine 68	1.1483688	0.05494
fetal large intestine 63	1.1622293	0.05528
fetal muscle 173	1.1482689	0.05532
fetal small intestine 79	1.1671371	0.05614
fetal muscle 193	1.1482900	0.05712
fetal thymus 259	1.1896399	0.05800
placenta 201	1.1561619	0.05862
fetal lung 111	1.1260657	0.05950
fetal fibroblast 267	1.1219088	0.06066
fetal kidney 106	1.1493558	0.06090
fetal kidney 85	1.1392283	0.06092
fetal lung 141	1.1206433	0.06104
fetal renal cortex 209	1.1498670	0.06166
fetal testes 254	1.1458868	0.06224
placenta 200	1.1277983	0.06232
ovary 326	1.1779240	0.06338
fetal thymus 263	1.1432970	0.06462
CD4 15	1.2214902	0.06538
fetal renal cortex 204	1.1323821	0.06764
fetal small intestine 76	1.1554372	0.06776
fetal muscle 190	1.1451859	0.06822
fetal small intestine 80	1.1408720	0.06872
fetal thymus 256	1.1603257	0.06876
fetal kidney 97	1.1423060	0.06890
muscle 341	1.1565693	0.06980
fetal kidney 83	1.1614539	0.07000
fetal lung 126	1.1144041	0.07024
fetal small intestine 71	1.1462967	0.07120
fetal kidney 105	1.1354851	0.07154
placenta 196	1.1410756	0.07214
fetal muscle 189	1.1314205	0.07214
fetal large intestine 67	1.1484947	0.07268
fetal kidney 88	1.1337609	0.07290
fetal adrenal gland 24	1.1504698	0.07420
placenta 198	1.1234397	0.07452
fetal muscle 149	1.1386100	0.07456
fetal renal cortex 208	1.1415827	0.07520
fetal muscle 151	1.1373020	0.07590
fetal thymus 261	1.1643655	0.07610
fetal muscle 148	1.1322991	0.07620
fetal kidney 90	1.1378883	0.07708
fetal large intestine 56	1.1374850	0.07708
fetal large intestine 66	1.1457106	0.07932
fetal small intestine 72	1.1385125	0.07932
Mobilized CD3 320	1.1767915	0.07958
fetal lung 133	1.1102562	0.08146
fetal thymus 260	1.1570688	0.08198
fetal stomach 244	1.1445809	0.08200
breast 1	1.1052804	0.08234
fetal adrenal gland 25	1.1343335	0.08300
fetal small intestine 77	1.1379120	0.08378
fetal renal pelvis 223	1.1403817	0.08414
fetal renal pelvis 216	1.1363796	0.08436
fetal lung 116	1.1034288	0.08446
fetal muscle 192	1.1205582	0.08448
fetal renal cortex 210	1.1355001	0.08646
stomach 283	1.1447236	0.08648
fetal large intestine 59	1.1387207	0.08752
fetal muscle 163	1.1262696	0.08804
fetal kidney 91	1.1278376	0.08860
fetal large intestine 62	1.1416684	0.08868
fetal muscle 147	1.1253748	0.08882
fetal lung 117	1.1101003	0.08900
fetal renal cortex 207	1.1314981	0.08928
breast 2	1.1024736	0.08972
fetal muscle 177	1.1301995	0.08988
fetal lung 138	1.1142697	0.09004
fetal muscle 180	1.1263874	0.09038
fetal muscle 179	1.1190109	0.09052
fetal kidney 99	1.1293828	0.09170
fetal renal cortex 203	1.1263045	0.09264
fetal muscle 169	1.1192081	0.09274
fetal kidney 107	1.1260314	0.09320
stomach 284	1.1375510	0.09402
fetal lung 120	1.1097071	0.09428
fetal renal pelvis 230	1.1370134	0.09498
fetal muscle 145	1.1314686	0.09502
fetal kidney 92	1.1240380	0.09504
fetal renal pelvis 220	1.1330603	0.09638
fetal muscle 170	1.1116304	0.09662
fetal muscle 181	1.1159885	0.09686
fetal lung 115	1.1041140	0.09782
fetal renal pelvis 226	1.1220692	0.09840
fetal kidney 102	1.1315250	0.09896
placenta 199	1.1159658	0.09948
fetal muscle 161	1.1194613	0.09952
CD4 16	1.1617461	0.10062
iPS 303	1.1137494	0.10154
fetal muscle 174	1.1168955	0.10196
fetal muscle 191	1.1175584	0.10216
fetal kidney 89	1.1251385	0.10256
placenta 197	1.1213568	0.10312
fetal renal pelvis 228	1.1319183	0.10352
fetal lung 110	1.1080856	0.10368
fetal kidney 82	1.1325407	0.10374
fetal lung 127	1.1082527	0.10380
fetal thymus 257	1.1504157	0.10472
fetal muscle 164	1.1131009	0.10476
fetal muscle 175	1.1125554	0.10532
fetal kidney 101	1.1171662	0.10550
fetal kidney 94	1.1278111	0.10552
fetal renal pelvis 225	1.1221677	0.10590
fetal renal cortex 212	1.1148334	0.10640
fetal muscle 153	1.1207697	0.10650
fetal lung 135	1.1048830	0.10692
fetal kidney 96	1.1132554	0.10706
fetal renal pelvis 231	1.1135588	0.10914
fetal lung 140	1.1000888	0.10964
fetal lung 118	1.0938728	0.10968
iPS 302	1.1105333	0.10998
fetal muscle 165	1.1033994	0.11002
fetal renal cortex 206	1.1141263	0.11016
ES derived mesoderm 286	1.0895826	0.11028
fetal lung 125	1.1053660	0.11202
fetal muscle 188	1.1068760	0.11210
fetal ovary 195	1.1403930	0.11214
fetal renal pelvis 224	1.1087280	0.11444
fetal renal cortex 205	1.1144859	0.11464
fetal muscle 187	1.1032730	0.11482
fetal renal cortex 202	1.1111542	0.11610
fetal kidney 84	1.1195113	0.11624
fetal lung 124	1.0988699	0.11698
fetal kidney 93	1.1178001	0.11752
fetal muscle 182	1.1032319	0.11934
fetal renal pelvis 233	1.1148297	0.11936
fetal lung 108	1.1045271	0.11972
fetal lung 132	1.0985433	0.12148
fetal kidney 104	1.1120411	0.12180
fetal renal pelvis 222	1.1138464	0.12254
fetal lung 134	1.0990114	0.12418
fetal stomach 245	1.1195858	0.12492
fetal lung 121	1.0892902	0.12674
fetal kidney 87	1.1090594	0.12684
ES derived trophoblast 287	1.1044593	0.12742
fetal muscle 159	1.1085426	0.12754
fetal muscle 171	1.0977890	0.12876
fetal renal pelvis 229	1.1053223	0.12968
fetal stomach 248	1.1192737	0.13082
fetal renal pelvis 218	1.1303884	0.13094
fetal muscle 144	1.1056800	0.13286
fetal lung 122	1.0968039	0.13570
fetal stomach 251	1.1112878	0.13666
fetal lung 130	1.0898216	0.13834
fetal lung 114	1.0831559	0.13844
fetal muscle 143	1.0896708	0.13902
fetal kidney 98	1.1048308	0.13928
fetal large intestine 53	1.1197228	0.14034
fetal stomach 252	1.1105372	0.14088
fetal renal cortex 215	1.0975679	0.14130
fetal renal pelvis 221	1.0990633	0.14212
fetal lung 123	1.0898371	0.14244
fetal renal pelvis 217	1.1038492	0.14452
fetal lung 112	1.0879545	0.14476
fetal lung 113	1.0813642	0.14574
fetal muscle 157	1.0877538	0.14610
fetal muscle 185	1.0976311	0.14722
fetal stomach 246	1.1134958	0.14734
fetal lung 129	1.0867242	0.14744
fetal lung 109	1.0763199	0.14754
ES cells 296	1.1092936	0.14776
fetal stomach 241	1.1135661	0.14814
iPS 304	1.0893793	0.14978
fetal kidney 103	1.1011405	0.15210
fetal stomach 240	1.1132990	0.15290
fetal muscle 155	1.0968666	0.15416
fetal lung 136	1.0850356	0.15428
fetal muscle 156	1.0890709	0.15614
fetal renal cortex 211	1.1011657	0.15860
ES cells 295	1.1042799	0.15866
fetal stomach 250	1.1092222	0.15870
fetal muscle 186	1.0866689	0.15936
fetal renal pelvis 227	1.0950667	0.15978
fetal muscle 162	1.0849255	0.15978
fetal lung 119	1.0772365	0.16048
fetal muscle 183	1.0838645	0.16388
fetal muscle 160	1.0902385	0.16416
fetal lung 142	1.0744421	0.16832
fetal muscle 184	1.0796601	0.16850
fetal muscle 178	1.0880455	0.16878
fetal lung 131	1.0706610	0.16900
fetal kidney 95	1.0918510	0.17096
ES cells 294	1.0977184	0.17304
fetal muscle 150	1.0860954	0.17338
fetal small intestine 75	1.1374518	0.17434
fetal muscle 167	1.0792029	0.17518
fetal muscle 158	1.0798140	0.17590
fetal renal pelvis 219	1.1048924	0.17982
pancreas 328	1.0926994	0.18082
fetal kidney 86	1.0991075	0.18580
fetal stomach 242	1.1093197	0.18674
fetal renal cortex 214	1.0827987	0.18684
fetal muscle 154	1.0842903	0.18906
ES derived NP 292	1.0884862	0.18928
fetal stomach 243	1.0845552	0.18950
fetal lung 137	1.0701504	0.19234
fetal kidney 100	1.0820062	0.19416
fetal renal pelvis 232	1.0827578	0.19972
fetal stomach 249	1.0900372	0.20138
fetal muscle 146	1.0709609	0.20336
fetal muscle 166	1.0722194	0.20498
pancreas 327	1.0860788	0.20544
fetal skin 234	1.0759951	0.21152
fetal stomach 253	1.0857079	0.21160
ES cells 293	1.0782793	0.21166
fetal renal cortex 213	1.0702256	0.21258
fetal muscle 152	1.0661923	0.21650
fetal stomach 247	1.0898326	0.21666
fetal lung 139	1.0661026	0.21958
ES derived NP 291	1.0800623	0.21974
fetal muscle 194	1.0583409	0.23044
ES derived mesoderm 285	1.0558828	0.23406
fetal muscle 176	1.0590821	0.23988
fetal thymus 262	1.0827470	0.26218
fetal spinal cord 236	1.0519305	0.27346
fetal spinal cord 238	1.0447492	0.29428
fetal brain 37	1.0384701	0.29980
fetal spinal cord 239	1.0370279	0.32438
fetal spinal cord 237	1.0329617	0.34120
fetal spinal cord 235	1.0263653	0.37038
fetal brain 38	1.0073447	0.45406
fetal brain 40	1.0060856	0.46054
fetal brain 41	1.0040154	0.47182
fetal brain 31	1.0025825	0.47748
fetal brain 39	0.9919054	0.53674
fetal brain 29	0.9920198	0.54412
fetal brain 35	0.9844357	0.58596
fetal brain 36	0.9744892	0.62648
fetal brain 30	0.9778294	0.63454
fetal brain 34	0.9701067	0.66186
fetal brain 33	0.9586751	0.69504
fetal brain 32	0.9327081	0.81674

Serum urate concentrations

DHS sample	fold enrichment	p value
fetal lung 140	1.428439	0.00048
fetal lung 141	1.378683	0.00052
fetal lung 117	1.411886	0.00062
fetal lung 118	1.376573	0.00074
fetal lung 114	1.361619	0.00096
fetal lung 132	1.412728	0.00102
fibroblast 329	1.369166	0.00170
fetal lung 119	1.354387	0.00190
fetal lung 133	1.354214	0.00200
fetal lung 125	1.377588	0.00236
fetal lung 120	1.367358	0.00238
fibroblast 330	1.356280	0.00240
fetal lung 121	1.333948	0.00264
fetal lung 111	1.335502	0.00278
fetal lung 129	1.350992	0.00316
fetal large intestine 67	1.442810	0.00330
fetal lung 128	1.301037	0.00360
fetal lung 123	1.348088	0.00410
fetal large intestine 58	1.366452	0.00428
fetal lung 116	1.301610	0.00466
fetal large intestine 55	1.380588	0.00488
fetal lung 134	1.351275	0.00544
fetal lung 139	1.345389	0.00564
fetal small intestine 80	1.387736	0.00570
fetal renal pelvis 220	1.406343	0.00582
fetal small intestine 74	1.381716	0.00636
fetal adrenal gland 28	1.315760	0.00654
fetal skin 234	1.368307	0.00686
fetal kidney 85	1.326982	0.00694
iPS 304	1.337086	0.00696
fetal large intestine 60	1.390434	0.00706
fetal lung 112	1.316086	0.00706
fetal large intestine 56	1.382726	0.00714
fetal lung 138	1.318404	0.00718
muscle 341	1.431170	0.00722
fetal lung 131	1.282176	0.00722
fetal lung 135	1.321843	0.00728
fetal lung 126	1.289837	0.00734
fetal small intestine 78	1.334626	0.00756
fetal small intestine 77	1.385523	0.00766
fetal kidney 106	1.367141	0.00780
fetal kidney 91	1.352299	0.00920
fetal lung 110	1.314935	0.00944
fetal muscle 194	1.291593	0.00954
fetal adrenal gland 23	1.315672	0.00988
fetal adrenal gland 25	1.361086	0.00992
fetal lung 130	1.299695	0.01018
fetal large intestine 57	1.325674	0.01034
fetal lung 113	1.280126	0.01038
fetal lung 115	1.284841	0.01042
fetal lung 301	1.298650	0.01048
fetal adrenal gland 26	1.343331	0.01056
iPS 303	1.313378	0.01058
heart 297	1.361798	0.01098
fetal renal cortex 207	1.348819	0.01112
small intestine 342	1.352530	0.01130
fetal lung 300	1.295417	0.01142
fetal small intestine 70	1.299931	0.01154
fetal stomach 248	1.392183	0.01166
CD20 8	1.329924	0.01170
ES derived mesoderm 285	1.272664	0.01174
fetal lung 124	1.298359	0.01180
fetal lung 108	1.318943	0.01184
fetal large intestine 64	1.341230	0.01200
fetal lung 137	1.280810	0.01270
fetal testes 255	1.308678	0.01316
fetal adrenal gland 27	1.323643	0.01322
fetal lung 136	1.291546	0.01328
fetal small intestine 68	1.322500	0.01332
fibroblast 331	1.271966	0.01348
fetal small intestine 72	1.326777	0.01400
fetal lung 127	1.295161	0.01428
fetal kidney 103	1.343670	0.01474
fibroblast 332	1.262828	0.01500
fetal stomach 250	1.388400	0.01518
melanocyte 338	1.241694	0.01586
fetal lung 142	1.255013	0.01626
fetal large intestine 54	1.335951	0.01628
fetal adrenal gland 24	1.349105	0.01648
fetal renal pelvis 233	1.320557	0.01658
fetal small intestine 69	1.354263	0.01674
fetal lung 298	1.291792	0.01690
fetal large intestine 66	1.344335	0.01710
fetal lung 122	1.297581	0.01738
fetal renal cortex 213	1.295000	0.01758
pancreas 328	1.348728	0.01762
fetal stomach 246	1.362581	0.01848
fetal large intestine 59	1.330151	0.01926
fetal large intestine 65	1.326832	0.01930
fetal muscle 151	1.314134	0.01930
ES derived mesoderm 286	1.233216	0.01948
melanocyte 340	1.256546	0.01962
ES derived NP 292	1.321371	0.01966
pancreas 327	1.347742	0.02008
fetal kidney 87	1.296799	0.02020
fetal small intestine 71	1.320632	0.02100
fetal renal cortex 214	1.297516	0.02114
fetal kidney 99	1.293889	0.02150
melanocyte 337	1.216328	0.02154
ES derived NP 291	1.343674	0.02164
ES cells 296	1.334263	0.02194
fetal small intestine 73	1.314562	0.02196
fetal kidney 92	1.287097	0.02256
fetal lung 109	1.226529	0.02280
fetal muscle 159	1.298865	0.02304
fetal renal pelvis 226	1.283452	0.02316
fetal kidney 95	1.298588	0.02330
ES derived MSC 289	1.281055	0.02368
fetal fibroblast 269	1.232030	0.02382
fetal heart 44	1.329302	0.02400
fetal stomach 243	1.297721	0.02408
fetal small intestine 76	1.317712	0.02442
fetal large intestine 63	1.309994	0.02462
fetal large intestine 62	1.325621	0.02474
ES cells 294	1.322374	0.02498
fetal kidney 84	1.301155	0.02506
fetal lung 299	1.256135	0.02506
fetal muscle 180	1.282866	0.02518
fetal renal cortex 202	1.280744	0.02528
fetal small intestine 79	1.313459	0.02566
ES cells 295	1.314962	0.02606
fetal kidney 100	1.291923	0.02612
fetal stomach 253	1.337559	0.02676
fetal stomach 252	1.320626	0.02688
fetal renal pelvis 231	1.269575	0.02706
fetal stomach 241	1.338741	0.02716
fetal testes 254	1.280610	0.02720
fetal muscle 181	1.258852	0.02746
fetal renal pelvis 228	1.309157	0.02790
fetal renal pelvis 217	1.296869	0.02816
fetal stomach 240	1.340480	0.02838
ES cells 293	1.286335	0.02852
fetal thymus 258	1.327311	0.02878
fetal renal pelvis 232	1.295192	0.02914
fetal renal cortex 205	1.276822	0.02914
fetal fibroblast 280	1.220077	0.03092
fetal stomach 251	1.303377	0.03106
fetal stomach 247	1.360674	0.03116
fetal muscle 186	1.253239	0.03146
ES derived MSC 290	1.259465	0.03170
fetal muscle 149	1.277828	0.03178
fetal renal pelvis 230	1.303430	0.03188
fetal stomach 244	1.298248	0.03220
fetal kidney 89	1.281126	0.03280
fetal heart 46	1.269959	0.03286
fetal fibroblast 270	1.220009	0.03298
fetal muscle 148	1.257821	0.03396
fetal renal cortex 211	1.293076	0.03428
fetal kidney 83	1.305777	0.03430
fetal muscle 169	1.250252	0.03438
fetal muscle 161	1.261150	0.03462
fetal muscle 155	1.273435	0.03500
stomach 284	1.304732	0.03522
fetal muscle 156	1.246536	0.03558
fetal muscle 171	1.238015	0.03690
fetal muscle 174	1.251094	0.03714
fetal muscle 164	1.248727	0.03770
fetal fibroblast 279	1.212902	0.03792
fetal muscle 182	1.239642	0.03830
iPS 302	1.241979	0.03866
fetal renal pelvis 219	1.315897	0.03962
fetal kidney 102	1.280586	0.04034
fetal fibroblast 272	1.202783	0.04038
fetal fibroblast 271	1.204392	0.04078
fetal renal pelvis 227	1.257144	0.04124
fetal fibroblast 268	1.198778	0.04132
fetal muscle 167	1.230459	0.04148
fetal muscle 153	1.258036	0.04166
fetal muscle 172	1.206507	0.04188
fetal stomach 242	1.345614	0.04208
fetal kidney 94	1.270953	0.04216
fetal thymus 263	1.246256	0.04226
fetal heart 48	1.239624	0.04244
fetal spinal cord 236	1.238131	0.04256
fetal fibroblast 276	1.199017	0.04276
fetal muscle 166	1.231496	0.04326
fetal fibroblast 274	1.204252	0.04332
fetal heart 47	1.267550	0.04376
fetal muscle 165	1.219400	0.04390
hematopoietic stem cell 310	1.208888	0.04404
fetal muscle 154	1.255482	0.04428
fetal muscle 158	1.221908	0.04468
fetal muscle 176	1.216957	0.04502
fetal ovary 195	1.302316	0.04510
fetal renal cortex 212	1.239497	0.04528
fetal kidney 101	1.243919	0.04560
fetal heart 45	1.238962	0.04576
fetal muscle 178	1.239017	0.04596
fetal muscle 179	1.226893	0.04610
fetal muscle 187	1.218722	0.04612
fetal fibroblast 278	1.204496	0.04636
fetal heart 51	1.230404	0.04642
fetal renal pelvis 221	1.235446	0.04646
fetal large intestine 53	1.293813	0.04720
fetal fibroblast 277	1.207314	0.04754
fetal muscle 143	1.211559	0.04776
fetal heart 42	1.231149	0.04902
hematopoietic stem cell 308	1.220286	0.04918
fetal fibroblast 273	1.190160	0.05042
fetal muscle 168	1.237217	0.05052
fetal muscle 189	1.223654	0.05064
fetal spinal cord 235	1.211367	0.05098
fetal fibroblast 267	1.194653	0.05146
fetal muscle 160	1.228502	0.05156
fetal thymus 256	1.264442	0.05168
fetal muscle 150	1.234330	0.05296
fetal fibroblast 281	1.184556	0.05340
fetal renal cortex 215	1.224590	0.05436
fetal muscle 184	1.201243	0.05456
ovary 326	1.287677	0.05550
fetal muscle 188	1.210919	0.05628
fetal kidney 104	1.241023	0.05644
fetal heart 52	1.203073	0.05674
stomach 283	1.262818	0.05684
fetal fibroblast 282	1.176617	0.05686
fetal muscle 147	1.222536	0.05724
fetal kidney 88	1.219063	0.05842
fetal muscle 157	1.194581	0.05922
fetal muscle 191	1.222620	0.05946
fetal muscle 185	1.224929	0.05998
fetal kidney 82	1.250294	0.06094
melanocyte 339	1.175302	0.06112
fetal renal pelvis 216	1.234830	0.06206
fetal renal cortex 206	1.218113	0.06206
fetal muscle 146	1.207302	0.06216
fetal renal pelvis 229	1.219994	0.06294
fetal kidney 97	1.221503	0.06454
fetal stomach 249	1.263211	0.06460
fetal heart 49	1.210871	0.06488
fetal muscle 163	1.215203	0.06520
fetal thymus 259	1.262843	0.06746
fetal renal pelvis 224	1.205486	0.06850
fetal thymus 257	1.268927	0.06860
fetal muscle 175	1.199103	0.06902
fetal muscle 173	1.205295	0.07052
fetal kidney 105	1.205888	0.07064
fetal thymus 260	1.249272	0.07076
fetal muscle 190	1.217122	0.07112
fetal fibroblast 275	1.166936	0.07176
fetal muscle 192	1.196008	0.07220
fetal muscle 183	1.190043	0.07238
fetal kidney 98	1.217664	0.07306
fetal muscle 170	1.187207	0.07326
hematopoietic stem cell 315	1.208353	0.07350
fetal heart 50	1.199679	0.07420
fetal muscle 152	1.189194	0.07436
fetal renal cortex 203	1.212180	0.07582
fetal muscle 177	1.211475	0.07646
fetal spinal cord 239	1.182489	0.07802
hematopoietic stem cell 316	1.210825	0.07838
fetal stomach 245	1.225834	0.07850
hematopoietic stem cell 306	1.193352	0.07936
keratinocyte 333	1.148304	0.08022
fetal thymus 265	1.232588	0.08110
hematopoietic stem cell 309	1.189964	0.08304
fetal kidney 86	1.239144	0.08378
fetal kidney 107	1.196996	0.08380
fetal kidney 96	1.188690	0.08686
hematopoietic stem cell 318	1.199337	0.08710
fetal thymus 261	1.231144	0.08734
fetal renal pelvis 223	1.208477	0.08756
fetal thymus 264	1.217527	0.08776
hematopoietic stem cell 311	1.181793	0.08834
fetal renal cortex 204	1.176757	0.09102
keratinocyte 334	1.144097	0.09170
placenta 199	1.181598	0.09290
fetal muscle 144	1.192470	0.09332
fetal spinal cord 237	1.169137	0.09430
fetal spinal cord 238	1.170368	0.09510
hematopoietic stem cell 313	1.167516	0.09736
hematopoietic stem cell 312	1.172329	0.09778
hematopoietic stem cell 307	1.176728	0.09856
fetal renal cortex 208	1.192966	0.09934
CD56 18	1.244188	0.09998
CD19 7	1.246821	0.10064
fetal renal pelvis 225	1.189954	0.10128
fetal large intestine 61	1.232805	0.10182
fetal renal cortex 209	1.182736	0.10396
CD19 6	1.239950	0.10430
CD14 3	1.214589	0.10506
hematopoietic stem cell 317	1.145566	0.10656
fetal renal pelvis 222	1.185372	0.10740
fetal kidney 93	1.189274	0.10884
fetal thymus 266	1.200300	0.11054
hematopoietic stem cell 305	1.170044	0.11172
fetal brain 36	1.136456	0.11332
CD14 4	1.191126	0.11342
fetal muscle 193	1.168859	0.11416
fetal heart 43	1.155321	0.11472
Mobilized CD56 324	1.204454	0.11768
CD8 21	1.233275	0.11774
fetal muscle 145	1.179758	0.11786
breast 2	1.139111	0.11796
keratinocyte 336	1.125529	0.11934
ES derived trophoblast 288	1.151265	0.11946
fetal thymus 262	1.237109	0.12036
fetal renal pelvis 218	1.204729	0.12166
Mobilized CD4 323	1.223092	0.12176
placenta 197	1.170368	0.12286
hematopoietic stem cell 314	1.147703	0.12350
CD4 15	1.264665	0.12538
fetal muscle 162	1.148505	0.12548
hematopoietic stem cell 319	1.169648	0.12686
Mobilized CD4 322	1.226300	0.12730
keratinocyte 335	1.119497	0.13328
fetal kidney 90	1.158093	0.13560
fetal kidney 81	1.138232	0.13908
placenta 196	1.155880	0.14064
CD3 12	1.208397	0.14270
Mobilized CD8 325	1.205293	0.14552
fetal renal cortex 210	1.153640	0.15098
Mobilized CD3 321	1.204624	0.15292
CD3 9	1.184003	0.15760
CD19 5	1.172817	0.16172
breast 1	1.109532	0.16830
CD8 19	1.191232	0.17116
placenta 198	1.122769	0.17126
CD4 13	1.176080	0.17354
placenta 200	1.117111	0.17488
ES derived trophoblast 287	1.132011	0.17552
fetal brain 38	1.108308	0.17710
placenta 201	1.133022	0.18024
CD3 10	1.149573	0.18754
CD4 14	1.143304	0.19000
fetal brain 41	1.098555	0.19068
fetal small intestine 75	1.186977	0.19636
fetal brain 37	1.087136	0.21802
CD3 11	1.125121	0.22220
CD4 16	1.140519	0.22584
Mobilized CD3 320	1.127506	0.24722
CD8 22	1.105462	0.27318
fetal brain 40	1.060866	0.27938
CD56 17	1.087484	0.28200
fetal brain 33	1.061233	0.29876
fetal brain 39	1.051725	0.31186
fetal brain 32	1.040390	0.35400
CD8 20	1.048573	0.37100
fetal brain 35	1.029819	0.37554
fetal brain 34	1.016558	0.43040
fetal brain 29	1.011011	0.44666
fetal brain 31	0.991240	0.53148
fetal brain 30	0.966491	0.63830

Height 1

DHS sample	fold enrichment	p value
fetal muscle 176	1.6457016	0.00022
fetal muscle 152	1.6178571	0.00022
fetal muscle 181	1.6250759	0.00048
fetal muscle 151	1.6732770	0.00058
fetal muscle 180	1.6384969	0.00064
fetal muscle 166	1.5866419	0.00068
fetal muscle 167	1.5712036	0.00084
fetal muscle 143	1.5350181	0.00100
fetal lung 111	1.5056585	0.00108
fetal muscle 179	1.5554289	0.00110
melanocyte 338	1.4200208	0.00130
fetal muscle 149	1.5839616	0.00134
fetal muscle 148	1.5625864	0.00134
fetal muscle 169	1.5408309	0.00160
fetal muscle 157	1.4907560	0.00170
fetal muscle 187	1.4843389	0.00176
fetal muscle 154	1.5624937	0.00224
fetal muscle 182	1.5012308	0.00232
fetal muscle 164	1.5286842	0.00248
fetal muscle 183	1.4720343	0.00248
fetal muscle 160	1.5315996	0.00276
melanocyte 337	1.3577117	0.00290
fetal muscle 165	1.4539397	0.00296
fetal muscle 155	1.5327950	0.00304
fetal muscle 171	1.4507566	0.00336
fetal muscle 175	1.4818967	0.00344
fetal muscle 153	1.5386899	0.00354
fetal fibroblast 274	1.3804128	0.00382
fetal muscle 150	1.5171190	0.00396
fetal fibroblast 273	1.3715090	0.00424
fetal muscle 156	1.4539812	0.00462
melanocyte 339	1.3588308	0.00480
fetal muscle 189	1.4392019	0.00502
fetal muscle 168	1.4787250	0.00520
fetal lung 140	1.4023921	0.00526
fetal kidney 85	1.4781869	0.00554
fetal fibroblast 271	1.3547994	0.00590
keratinocyte 336	1.3223736	0.00596
fetal fibroblast 272	1.3550356	0.00610
fetal muscle 147	1.4466662	0.00622
fetal muscle 178	1.4675927	0.00650
fetal lung 141	1.3819094	0.00652
fetal muscle 158	1.4128235	0.00672
fetal lung 128	1.3407455	0.00730
fetal fibroblast 281	1.3423923	0.00776
fetal muscle 192	1.4146085	0.00782
melanocyte 340	1.3560314	0.00786
fetal muscle 184	1.3855157	0.00794
fetal muscle 159	1.4475796	0.00806
hematopoietic stem cell 310	1.3657242	0.00810
fetal muscle 172	1.3612751	0.00842
keratinocyte 334	1.3160609	0.00846
fetal lung 117	1.3673946	0.00856
hematopoietic stem cell 314	1.3774002	0.00886
fetal fibroblast 276	1.3247943	0.00896
fetal muscle 186	1.4011527	0.00992
fetal lung 119	1.3499625	0.01176
fetal fibroblast 277	1.3261248	0.01182
fetal muscle 161	1.4015286	0.01186
fetal fibroblast 278	1.3295756	0.01218
fetal stomach 244	1.4353519	0.01228
fetal fibroblast 269	1.3155992	0.01260
fetal lung 133	1.3319986	0.01292
keratinocyte 335	1.2876307	0.01312
fetal fibroblast 275	1.3051647	0.01326
fetal fibroblast 267	1.3213658	0.01334
fetal muscle 190	1.4046786	0.01374
fetal lung 120	1.3493451	0.01378
fetal muscle 146	1.3769949	0.01412
fetal fibroblast 280	1.3130054	0.01416
fetal lung 118	1.3189492	0.01448
fetal muscle 163	1.3838315	0.01588
keratinocyte 333	1.2806868	0.01592
fetal fibroblast 270	1.3138104	0.01628
fetal muscle 185	1.3919219	0.01676
fetal muscle 188	1.3438516	0.01752
fetal skin 234	1.3792725	0.01838
fetal muscle 170	1.3451664	0.01856
fetal muscle 144	1.3748287	0.01892
fetal kidney 91	1.3923322	0.01994
fetal fibroblast 282	1.2800216	0.02000
fetal testes 255	1.3385895	0.02120
fetal lung 298	1.3204345	0.02122
fetal fibroblast 279	1.2933693	0.02132
fetal lung 114	1.3004239	0.02236
hematopoietic stem cell 309	1.3358392	0.02406
fetal fibroblast 268	1.2708550	0.02478
fetal lung 121	1.2852068	0.02544
fetal lung 115	1.2857411	0.02618
fetal kidney 92	1.3457271	0.02656
ES derived mesoderm 285	1.2871136	0.02732
fetal lung 129	1.3000412	0.02822
fetal muscle 194	1.2851586	0.03032
fetal lung 132	1.2926008	0.03078
fetal thymus 258	1.4058025	0.03176
fetal lung 299	1.2890601	0.03224
fetal muscle 174	1.3114014	0.03320
Mobilized CD56 324	1.3832359	0.03430
fetal stomach 248	1.3671079	0.03448
fetal muscle 173	1.3137869	0.03458
fetal kidney 106	1.3258602	0.03526
fetal large intestine 67	1.3472387	0.03536
fetal lung 301	1.2813806	0.03548
fetal lung 131	1.2537059	0.03592
fetal muscle 162	1.2924148	0.03606
fetal muscle 193	1.3159493	0.03636
fetal lung 116	1.2599336	0.03640
breast 1	1.2459840	0.03760
fetal muscle 177	1.3166680	0.03862
fetal lung 300	1.2710954	0.03872
fetal lung 134	1.2833702	0.03910
fetal lung 108	1.2892137	0.03920
fetal stomach 249	1.3637088	0.03970
ES derived MSC 289	1.2823554	0.04082
fetal lung 110	1.2768303	0.04124
fetal lung 113	1.2545270	0.04196
hematopoietic stem cell 308	1.2722101	0.04232
fetal lung 142	1.2458821	0.04246
fetal muscle 145	1.3230465	0.04340
fetal lung 125	1.2741621	0.04418
breast 2	1.2417405	0.04418
fetal renal cortex 213	1.2954704	0.04778
fetal small intestine 78	1.2691302	0.04814
fetal large intestine 61	1.3682535	0.04858
fetal kidney 103	1.3180613	0.04906
ES derived MSC 290	1.2658085	0.04940
fetal lung 112	1.2565922	0.04972
fetal lung 126	1.2371439	0.05020
fetal muscle 191	1.2801699	0.05046
fetal lung 130	1.2493457	0.05062
hematopoietic stem cell 305	1.2763573	0.05130
fetal renal pelvis 220	1.3071165	0.05238
fetal thymus 256	1.3337572	0.05452
fibroblast 332	1.2245717	0.05604
fetal stomach 253	1.3206293	0.05726
Mobilized CD3 320	1.3932243	0.05814
fetal lung 109	1.2164480	0.05826
fetal stomach 246	1.3127523	0.05896
ES derived mesoderm 286	1.2094414	0.05916
fetal renal cortex 207	1.2878192	0.05950
fetal lung 127	1.2448734	0.05976
fetal small intestine 80	1.2628241	0.06244
fetal heart 47	1.3006500	0.06264
fetal thymus 263	1.2636194	0.06348
fetal kidney 95	1.2737126	0.06396
fetal thymus 264	1.3125973	0.06738
fetal stomach 243	1.2667094	0.06744
fetal large intestine 53	1.3110791	0.06860
fetal stomach 251	1.2754307	0.06936
fetal kidney 99	1.2672475	0.07006
fetal small intestine 68	1.2516748	0.07042
fetal lung 123	1.2261582	0.07060
fetal kidney 83	1.2940701	0.07078
ovary 326	1.3214604	0.07154
CD20 8	1.2511750	0.07300
fetal kidney 100	1.2529505	0.07618
fetal small intestine 74	1.2526142	0.07704
fetal stomach 250	1.2857302	0.07712
CD8 21	1.3327197	0.07884
fetal renal pelvis 226	1.2444848	0.07950
fetal kidney 89	1.2571663	0.07976
fetal kidney 84	1.2599938	0.08012
fetal renal pelvis 227	1.2503834	0.08064
CD3 9	1.3074384	0.08102
fetal renal pelvis 231	1.2324184	0.08248
fetal large intestine 57	1.2282538	0.08270
placenta 200	1.2041824	0.08398
fibroblast 330	1.1977299	0.08400
fibroblast 329	1.1978858	0.08474
fetal thymus 265	1.2862107	0.08542
fetal large intestine 58	1.2218580	0.08640
fetal lung 138	1.2104782	0.08640
fetal stomach 252	1.2529372	0.08940
fetal kidney 87	1.2281644	0.09018
fetal lung 124	1.2034120	0.09018
fetal lung 136	1.2091724	0.09026
fibroblast 331	1.1864481	0.09058
fetal thymus 266	1.2789298	0.09118
fetal lung 139	1.2094415	0.09164
CD4 13	1.3038409	0.09172
fetal renal pelvis 217	1.2403551	0.09188
CD56 18	1.3051680	0.09200
fetal renal pelvis 221	1.2225164	0.09320
fetal lung 135	1.2033204	0.09574
fetal renal cortex 215	1.2212825	0.09582
hematopoietic stem cell 312	1.2059081	0.09636
hematopoietic stem cell 318	1.2234724	0.09862
fetal small intestine 70	1.1997703	0.10092
fetal renal cortex 214	1.2175480	0.10218
Mobilized CD4 322	1.2888544	0.10232
fetal lung 137	1.1886307	0.10348
CD4 15	1.3300149	0.10638
hematopoietic stem cell 311	1.1987812	0.10804
fetal testes 254	1.2058995	0.11008
fetal thymus 260	1.2522856	0.11060
fetal large intestine 54	1.2167094	0.11072
fetal small intestine 75	1.3189311	0.11172
fetal thymus 257	1.2643199	0.11582
hematopoietic stem cell 316	1.2063469	0.11594
fetal small intestine 77	1.2144679	0.11654
Mobilized CD8 325	1.2695544	0.11828
fetal kidney 94	1.2177137	0.11860
fetal large intestine 64	1.2000391	0.11916
fetal small intestine 72	1.2076747	0.11974
fetal kidney 88	1.1924483	0.12030
fetal lung 122	1.1881241	0.12090
fetal large intestine 56	1.2010833	0.12170
CD19 6	1.2573612	0.12352
CD19 7	1.2581198	0.12488
Mobilized CD3 321	1.2641212	0.12764
CD8 20	1.2319628	0.12818
hematopoietic stem cell 307	1.1801526	0.13106
Mobilized CD4 323	1.2504486	0.13172
hematopoietic stem cell 317	1.1589868	0.13172
CD8 19	1.2745303	0.13288
fetal large intestine 60	1.1956471	0.13300
fetal renal pelvis 228	1.2076994	0.13334
fetal renal cortex 202	1.1818652	0.13882
hematopoietic stem cell 315	1.1803278	0.13882
fetal stomach 241	1.2107363	0.13954
fetal kidney 81	1.1665614	0.13988
fetal large intestine 63	1.1979658	0.14008
fetal renal pelvis 233	1.1854598	0.14036
fetal stomach 247	1.2295800	0.14070
fetal renal pelvis 232	1.1937869	0.14258
fetal renal pelvis 230	1.1986065	0.14338
placenta 199	1.1640418	0.14482
fetal small intestine 69	1.1937178	0.14620
fetal thymus 259	1.2277793	0.14736
fetal heart 45	1.1822719	0.14768
placenta 201	1.1827342	0.14876
CD56 17	1.1984831	0.14922
hematopoietic stem cell 306	1.1647492	0.14940
CD19 5	1.2180140	0.14950
placenta 197	1.1726858	0.15002
CD3 12	1.2334499	0.15100
fetal heart 46	1.1842346	0.15290
fetal kidney 98	1.1773422	0.15292
CD3 10	1.2119258	0.15336
fetal small intestine 73	1.1791314	0.15368
fetal brain 37	1.1330837	0.15422
fetal stomach 242	1.2168742	0.15634
fetal renal pelvis 222	1.1782602	0.15674
fetal kidney 96	1.1654158	0.15758
fetal renal pelvis 229	1.1680138	0.15846
CD3 11	1.1998339	0.15894
fetal renal cortex 206	1.1636685	0.15906
fetal heart 52	1.1559198	0.15988
fetal renal pelvis 224	1.1600390	0.16124
fetal stomach 245	1.1817732	0.16218
pancreas 328	1.1804266	0.16228
fetal renal cortex 203	1.1682798	0.16310
fetal kidney 105	1.1621598	0.16570
fetal renal cortex 205	1.1629522	0.16672
fetal large intestine 55	1.1576564	0.16690
fetal small intestine 71	1.1681248	0.16764
ES derived trophoblast 288	1.1483295	0.16818
fetal brain 36	1.1285907	0.16864
small intestine 342	1.1685439	0.17098
fetal kidney 102	1.1726076	0.17172
ES derived trophoblast 287	1.1615095	0.17432
fetal kidney 104	1.1604005	0.17714
hematopoietic stem cell 319	1.1561896	0.17718
placenta 198	1.1392598	0.17764
hematopoietic stem cell 313	1.1380525	0.17940
fetal heart 49	1.1549947	0.18114
fetal thymus 261	1.1836659	0.18480
fetal renal cortex 210	1.1570499	0.18588
CD4 14	1.1723070	0.18810
fetal renal cortex 212	1.1393165	0.18936
fetal adrenal gland 28	1.1275922	0.19086
fetal large intestine 66	1.1527467	0.19686
fetal stomach 240	1.1627280	0.19832
fetal renal cortex 209	1.1443245	0.19838
fetal renal cortex 211	1.1484107	0.20158
fetal renal pelvis 219	1.1689035	0.20236
fetal spinal cord 235	1.1234859	0.20292
fetal kidney 97	1.1372899	0.20492
fetal large intestine 62	1.1492585	0.20558
fetal adrenal gland 26	1.1285012	0.21050
fetal renal pelvis 223	1.1412736	0.21426
fetal heart 42	1.1318678	0.21622
CD8 22	1.1667276	0.21694
placenta 196	1.1296343	0.21892
fetal kidney 82	1.1389581	0.22294
fetal kidney 107	1.1243897	0.22366
fetal thymus 262	1.1741132	0.22432
heart 297	1.1354121	0.22600
fetal adrenal gland 27	1.1199551	0.22854
stomach 283	1.1375148	0.23016
fetal adrenal gland 25	1.1266023	0.23172
fetal spinal cord 237	1.1048250	0.23630
fetal brain 40	1.0876320	0.23744
fetal renal pelvis 216	1.1205500	0.23782
fetal renal cortex 208	1.1180715	0.24222
fetal heart 48	1.1143108	0.24526
fetal small intestine 79	1.1213243	0.24620
fetal renal cortex 204	1.1050225	0.24824
fetal small intestine 76	1.1179990	0.25322
fetal brain 34	1.0789791	0.25352
fetal kidney 101	1.1042154	0.25674
fetal spinal cord 236	1.1000740	0.25736
fetal large intestine 59	1.1143873	0.25912
CD14 4	1.1229850	0.25918
fetal renal pelvis 225	1.1060584	0.26298
fetal heart 51	1.1007644	0.26500
CD14 3	1.1180480	0.27662
fetal large intestine 65	1.0987388	0.27934
CD4 16	1.1187818	0.28198
fetal heart 50	1.0922316	0.28440
fetal brain 41	1.0674451	0.29172
fetal kidney 90	1.0862389	0.29862
pancreas 327	1.0905589	0.30672
fetal renal pelvis 218	1.0954785	0.30722
fetal heart 43	1.0766747	0.30740
fetal adrenal gland 24	1.0868804	0.30850
fetal kidney 86	1.0900716	0.31042
fetal adrenal gland 23	1.0671285	0.32160
fetal brain 39	1.0542684	0.32232
ES cells 296	1.0747528	0.32716
fetal brain 31	1.0416903	0.34692
fetal ovary 195	1.0642621	0.35102
fetal brain 38	1.0468967	0.35980
stomach 284	1.0565456	0.36690
fetal heart 44	1.0560631	0.37018
fetal kidney 93	1.0528822	0.37030
ES derived NP 291	1.0493060	0.37856
ES cells 294	1.0468494	0.38048
fetal spinal cord 239	1.0369403	0.39030
fetal brain 32	1.0337436	0.39182
iPS 303	1.0331591	0.39920
iPS 302	1.0324944	0.40252
iPS 304	1.0273283	0.41286
fetal brain 35	1.0210512	0.42168
ES cells 293	1.0245542	0.42570
fetal brain 30	1.0166027	0.43088
muscle 341	1.0006377	0.48222
fetal brain 29	1.0026556	0.48412
fetal spinal cord 238	0.9948559	0.49708
ES cells 295	0.9644985	0.55712
ES derived NP 292	0.9627016	0.56310
fetal brain 33	0.9554560	0.60776

Skeletal frame size

DHS sample	fold enrichment	p value
hematopoietic stem cell 310	1.7956225	0.00062
hematopoietic stem cell 314	1.8263844	0.00064
fetal muscle 181	1.7411370	0.00214
fetal fibroblast 273	1.6045468	0.00262
hematopoietic stem cell 309	1.7531938	0.00372
fetal muscle 176	1.6537788	0.00384
fetal fibroblast 275	1.5552272	0.00424
fetal fibroblast 277	1.6062210	0.00426
melanocyte 339	1.5447031	0.00430
fetal fibroblast 276	1.5575093	0.00472
fetal thymus 258	1.9130355	0.00478
fetal thymus 265	1.8715094	0.00478
fetal lung 111	1.5862181	0.00512
melanocyte 338	1.4915718	0.00514
fetal fibroblast 274	1.5648044	0.00518
fetal muscle 180	1.6705222	0.00588
fetal muscle 187	1.5948418	0.00618
fetal muscle 160	1.6310560	0.00644
fetal muscle 166	1.6097562	0.00652
fetal thymus 256	1.8379084	0.00660
fetal muscle 167	1.5864053	0.00662
melanocyte 337	1.4645393	0.00712
hematopoietic stem cell 308	1.6485468	0.00758
fetal fibroblast 271	1.5189544	0.00760
fetal muscle 152	1.5753521	0.00774
Mobilized CD56 324	1.8298192	0.00778
fetal kidney 85	1.5882172	0.00786
fetal fibroblast 268	1.5055140	0.00790
fetal muscle 143	1.5643857	0.00792
fetal muscle 151	1.6637586	0.00800
fetal fibroblast 278	1.5387144	0.00800
fibroblast 329	1.5462378	0.00806
fetal muscle 149	1.6273080	0.00808
fetal muscle 154	1.6496509	0.00832
fetal muscle 179	1.5950119	0.00896
fetal muscle 157	1.5385590	0.00930
fetal muscle 153	1.6364430	0.00944
fetal muscle 175	1.5814555	0.00970
hematopoietic stem cell 317	1.5355415	0.00990
fetal muscle 148	1.5897359	0.01012
fetal fibroblast 281	1.4978171	0.01022
fetal muscle 169	1.5822450	0.01044
fetal lung 141	1.4821356	0.01068
fetal lung 128	1.4437388	0.01122
hematopoietic stem cell 312	1.5934934	0.01170
fetal fibroblast 269	1.4892424	0.01192
fibroblast 332	1.5023042	0.01202
fetal thymus 264	1.7277547	0.01274
fetal fibroblast 272	1.4712871	0.01276
fibroblast 330	1.5088061	0.01310
fibroblast 331	1.4986832	0.01314
fetal muscle 182	1.5409308	0.01346
fetal fibroblast 267	1.4960169	0.01370
fetal muscle 183	1.5199521	0.01374
hematopoietic stem cell 305	1.6117868	0.01406
fetal lung 114	1.4459684	0.01408
fetal thymus 266	1.7169017	0.01436
fetal heart 49	1.5712819	0.01494
fetal lung 299	1.5444913	0.01536
fetal muscle 178	1.5431349	0.01538
CD8 21	1.8598073	0.01540
melanocyte 340	1.4551833	0.01540
CD56 18	1.8483141	0.01548
fetal heart 47	1.6167457	0.01552
fetal muscle 150	1.5498289	0.01586
fetal muscle 184	1.4863174	0.01586
fetal fibroblast 282	1.4427348	0.01616
keratinocyte 334	1.3924868	0.01636
fetal thymus 260	1.7210451	0.01646
breast 2	1.4431384	0.01720
fetal muscle 165	1.4965501	0.01746
hematopoietic stem cell 318	1.6051302	0.01754
fetal lung 300	1.5054703	0.01774
fetal muscle 172	1.4708617	0.01782
fetal lung 298	1.5299801	0.01790
fetal muscle 171	1.4976459	0.01792
fetal lung 301	1.5184734	0.01796
fetal fibroblast 280	1.4597334	0.01806
keratinocyte 336	1.3937299	0.01810
fetal fibroblast 270	1.4615852	0.01832
fetal muscle 159	1.5557728	0.01848
CD3 10	1.7437752	0.01864
fetal muscle 158	1.4847481	0.01864
fetal muscle 168	1.5383922	0.01866
Mobilized CD4 323	1.8021104	0.01898
fetal heart 45	1.5366205	0.01898
fetal lung 121	1.4291126	0.01902
Mobilized CD3 320	1.8384302	0.01912
fetal lung 118	1.4196998	0.01938
fetal thymus 257	1.7141371	0.01966
fetal fibroblast 279	1.4545760	0.02000
fetal thymus 259	1.7198980	0.02032
hematopoietic stem cell 315	1.5641151	0.02084
fetal lung 120	1.4644985	0.02092
fetal large intestine 67	1.5926332	0.02110
fetal large intestine 61	1.7125910	0.02280
hematopoietic stem cell 316	1.5957841	0.02290
hematopoietic stem cell 311	1.5207438	0.02290
CD8 19	1.8509892	0.02312
fetal lung 140	1.4312047	0.02332
fetal muscle 164	1.4954836	0.02338
fetal muscle 156	1.4723798	0.02382
fetal muscle 147	1.4876764	0.02386
fetal lung 117	1.4318321	0.02402
fetal kidney 91	1.5073901	0.02522
fetal muscle 185	1.4983686	0.02558
fetal muscle 189	1.4741232	0.02594
keratinocyte 333	1.3548228	0.02596
fetal skin 234	1.5126321	0.02634
fetal muscle 186	1.4590794	0.02702
CD56 17	1.6007117	0.02726
CD19 5	1.6787656	0.02744
keratinocyte 335	1.3592366	0.02862
hematopoietic stem cell 306	1.5032001	0.02886
CD4 13	1.7214445	0.02892
fetal lung 116	1.3856730	0.02916
CD19 7	1.7455104	0.02956
fetal lung 133	1.3989083	0.03006
fetal testes 255	1.4522372	0.03054
ES derived mesoderm 286	1.3659419	0.03084
fetal muscle 146	1.4411044	0.03138
hematopoietic stem cell 313	1.4575790	0.03156
fetal lung 119	1.3855034	0.03182
fetal muscle 170	1.4238440	0.03202
CD3 9	1.6918109	0.03214
hematopoietic stem cell 307	1.4862649	0.03244
fetal muscle 161	1.4418014	0.03244
fetal lung 132	1.4183711	0.03320
breast 1	1.3693300	0.03452
fetal thymus 263	1.4570097	0.03454
CD3 11	1.6197223	0.03494
fetal lung 110	1.4156409	0.03506
fetal heart 46	1.4774576	0.03550
CD19 6	1.6726530	0.03572
CD20 8	1.4754877	0.03744
fetal lung 129	1.3935066	0.03764
fetal muscle 155	1.4644383	0.03782
Mobilized CD8 325	1.6508625	0.04044
fetal brain 34	1.3055465	0.04052
fetal lung 134	1.3964550	0.04092
ES derived MSC 289	1.4430842	0.04094
Mobilized CD4 322	1.6846393	0.04158
CD8 20	1.5928994	0.04198
fetal lung 138	1.3840785	0.04290
fetal muscle 188	1.3975272	0.04318
fetal small intestine 80	1.4542945	0.04420
hematopoietic stem cell 319	1.4736387	0.04524
CD3 12	1.6497408	0.04622
fetal muscle 144	1.4192798	0.04718
fetal lung 115	1.3545516	0.04752
ES derived mesoderm 285	1.3462278	0.04788
fetal lung 130	1.3658716	0.04800
fetal thymus 261	1.5334942	0.04812
fetal muscle 192	1.3871517	0.04852
fetal lung 126	1.3368296	0.04962
fetal heart 52	1.3743598	0.04974
fetal heart 42	1.4036917	0.04984
fetal kidney 106	1.4242690	0.05042
CD14 4	1.4819827	0.05094
fetal kidney 92	1.4027501	0.05140
fetal adrenal gland 28	1.3701338	0.05220
fetal lung 112	1.3609334	0.05334
ES derived MSC 290	1.4003446	0.05382
fetal large intestine 57	1.4108989	0.05546
CD4 14	1.5306584	0.05568
fetal muscle 190	1.4084474	0.05588
fetal renal pelvis 220	1.4319110	0.05644
fetal lung 125	1.3659547	0.05654
fetal brain 31	1.2458737	0.05748
fetal heart 48	1.3852514	0.05834
fetal heart 51	1.3809098	0.05870
fetal lung 108	1.3649129	0.06088
fetal stomach 244	1.4478398	0.06154
fetal small intestine 78	1.3814594	0.06356
fetal muscle 194	1.3274311	0.06374
fetal adrenal gland 27	1.4102046	0.06434
fetal kidney 95	1.3857852	0.06434
fetal muscle 174	1.3676417	0.06460
fetal small intestine 68	1.3957116	0.06506
fetal muscle 163	1.3653667	0.06512
fetal renal pelvis 217	1.4047833	0.06728
fetal lung 127	1.3375125	0.06764
fetal renal pelvis 231	1.3526015	0.06910
fetal kidney 99	1.3692598	0.06974
CD4 15	1.6620665	0.06978
fetal muscle 177	1.3767579	0.07024
fetal renal pelvis 226	1.3620515	0.07052
fetal lung 113	1.2956583	0.07248
Mobilized CD3 321	1.5678351	0.07294
fetal brain 37	1.2702756	0.07318
fetal kidney 89	1.3765565	0.07320
fetal renal pelvis 229	1.3557991	0.07322
fetal brain 36	1.2756024	0.07364
fetal large intestine 58	1.3631947	0.07398
fetal kidney 81	1.3161137	0.07432
fetal renal pelvis 227	1.3648942	0.07482
fetal lung 135	1.3186966	0.07492
fetal muscle 145	1.3869259	0.07502
fetal muscle 162	1.3204409	0.07656
fetal lung 131	1.2726637	0.07660
fetal kidney 94	1.3774477	0.07696
fetal lung 123	1.3165128	0.07700
fetal lung 137	1.2998892	0.07858
fetal muscle 191	1.3405397	0.07906
fetal heart 44	1.4215180	0.07966
CD8 22	1.5193755	0.07972
fetal heart 43	1.3205946	0.08050
fetal small intestine 74	1.3796356	0.08062
fetal muscle 173	1.3345009	0.08112
fetal renal cortex 215	1.3286811	0.08156
fetal kidney 103	1.3603345	0.08166
fetal brain 35	1.2188572	0.08300
fetal small intestine 70	1.3273889	0.08306
fetal kidney 83	1.4059621	0.08378
fetal lung 124	1.3006563	0.08394
fetal renal cortex 213	1.3321808	0.08560
fetal muscle 193	1.3308820	0.08690
fetal kidney 100	1.3401441	0.08832
fetal renal cortex 207	1.3412519	0.08834
fetal testes 254	1.3491831	0.08848
fetal renal pelvis 221	1.3207781	0.08856
fetal kidney 105	1.3172718	0.08870
fetal large intestine 64	1.3553123	0.08884
fetal renal cortex 202	1.3195805	0.09120
fetal large intestine 63	1.3775877	0.09162
fetal lung 122	1.3072047	0.09258
fetal lung 142	1.2572893	0.09362
fetal adrenal gland 26	1.3398080	0.09484
fetal thymus 262	1.4720603	0.09514
fetal heart 50	1.3240302	0.09532
small intestine 342	1.3531722	0.09806
fetal adrenal gland 25	1.3573383	0.09870
fetal adrenal gland 23	1.3038236	0.09902
CD14 3	1.4060148	0.09962
fetal stomach 248	1.3667432	0.10002
fetal renal pelvis 228	1.3516132	0.10090
fetal spinal cord 236	1.2977522	0.10250
fetal lung 136	1.2798568	0.10314
fetal kidney 87	1.3112101	0.10394
fetal small intestine 71	1.3432209	0.10500
fetal small intestine 77	1.3416941	0.10574
fetal renal pelvis 230	1.3354894	0.10748
fetal small intestine 72	1.3309897	0.10782
fetal stomach 246	1.3703936	0.10904
fetal lung 139	1.2788852	0.11060
fetal stomach 253	1.3627226	0.11062
fetal kidney 96	1.2834374	0.11068
fetal lung 109	1.2267657	0.11092
fetal kidney 98	1.3065903	0.11100
fetal stomach 243	1.3140512	0.11166
fetal kidney 84	1.3175723	0.11218
fetal stomach 250	1.3546296	0.11332
fetal small intestine 73	1.3270295	0.11440
fetal brain 38	1.2361836	0.11444
fetal large intestine 54	1.3283305	0.11528
fetal small intestine 75	1.4976383	0.11580
fetal stomach 249	1.3482769	0.11804
fetal renal cortex 209	1.2891300	0.11874
fetal kidney 102	1.3045139	0.11910
fetal renal cortex 212	1.2656165	0.12008
fetal large intestine 55	1.2997072	0.12130
fetal brain 41	1.2127054	0.12196
fetal renal cortex 205	1.2760946	0.12760
fetal brain 39	1.1959459	0.12826
fetal renal pelvis 232	1.2955987	0.12866
fetal spinal cord 235	1.2447018	0.12948
fetal large intestine 53	1.3321467	0.13240
fetal renal cortex 203	1.2686576	0.13644
fetal spinal cord 237	1.2359883	0.13738
iPS 304	1.2448552	0.13766
fetal kidney 88	1.2531775	0.13790
fetal stomach 252	1.2993884	0.13792
iPS 303	1.2523423	0.13822
fetal renal cortex 206	1.2552153	0.13846
iPS 302	1.2527202	0.13872
fetal brain 40	1.1907399	0.13962
fetal renal cortex 214	1.2546875	0.14314
fetal kidney 101	1.2501940	0.14468
fetal kidney 97	1.2504315	0.14558
fetal renal cortex 211	1.2742475	0.14630
fetal small intestine 69	1.2987620	0.14682
heart 297	1.2811579	0.14846
fetal renal pelvis 223	1.2677660	0.14888
fetal adrenal gland 24	1.2960810	0.14944
fetal large intestine 62	1.2901691	0.15052
fetal renal pelvis 233	1.2521639	0.15096
ovary 326	1.3323153	0.15178
fetal renal pelvis 222	1.2579511	0.15196
fetal kidney 90	1.2448609	0.15544
fetal kidney 104	1.2474899	0.15624
fetal renal cortex 204	1.2266567	0.15664
fetal renal pelvis 224	1.2255363	0.15856
fetal renal pelvis 216	1.2442026	0.15966
fetal stomach 251	1.2615326	0.15990
fetal large intestine 66	1.2660065	0.16356
fetal renal cortex 210	1.2436193	0.16496
fetal small intestine 79	1.2654599	0.16712
fetal renal pelvis 225	1.2351972	0.17014
CD4 16	1.3337732	0.17040
fetal stomach 245	1.2548933	0.17302
fetal stomach 241	1.2610380	0.17320
fetal ovary 195	1.2763272	0.17384
fetal stomach 242	1.3040989	0.17596
fetal kidney 107	1.2203793	0.17638
stomach 283	1.2591321	0.17674
fetal renal cortex 208	1.2289202	0.17686
fetal renal pelvis 219	1.2732172	0.17818
fetal large intestine 56	1.2333887	0.17870
fetal small intestine 76	1.2497230	0.18062
fetal kidney 82	1.2326620	0.18654
fetal stomach 247	1.2718174	0.18788
fetal kidney 93	1.2225520	0.18814
fetal spinal cord 239	1.1773162	0.20166
ES derived trophoblast 288	1.1744563	0.21210
fetal kidney 86	1.2168559	0.21438
fetal brain 30	1.1114064	0.21472
fetal large intestine 59	1.2057641	0.21952
fetal large intestine 65	1.1989702	0.22306
ES cells 296	1.1953115	0.23130
muscle 341	1.1863102	0.24422
fetal large intestine 60	1.1732907	0.24434
fetal stomach 240	1.1852318	0.24876
placenta 201	1.1664673	0.25072
fetal brain 33	1.1269383	0.25638
fetal brain 29	1.0887293	0.26296
fetal brain 32	1.1105070	0.26688
fetal renal pelvis 218	1.1592335	0.27554
ES cells 294	1.1437240	0.28298
stomach 284	1.1455390	0.28606
placenta 200	1.1176628	0.28858
placenta 198	1.1075468	0.30858
fetal spinal cord 238	1.0994046	0.31044
placenta 197	1.1131607	0.31518
ES derived trophoblast 287	1.1086755	0.31854
placenta 199	1.0988408	0.32586
ES cells 293	1.1012605	0.32666
ES cells 295	1.0903592	0.34840
ES derived NP 292	1.0708505	0.37154
pancreas 328	1.0660044	0.38282
placenta 196	1.0473613	0.40774
pancreas 327	1.0389635	0.41820
ES derived NP 291	0.9770103	0.50722

Hematological parameters

DHS sample	fold enrichment	p value
fetal thymus 256	1.687220	0.00046
fetal thymus 258	1.748673	0.00054
fetal thymus 264	1.660414	0.00118
fetal thymus 265	1.654052	0.00126
CD8 21	1.741085	0.00128
Mobilized CD56 324	1.666190	0.00164
fetal thymus 266	1.640704	0.00180
hematopoietic stem cell 310	1.418170	0.00226
fetal thymus 260	1.617170	0.00332
fetal thymus 259	1.630230	0.00424
keratinocyte 333	1.324684	0.00454
keratinocyte 336	1.325308	0.00474
hematopoietic stem cell 314	1.405730	0.00498
keratinocyte 335	1.331907	0.00502
CD56 18	1.632902	0.00514
fetal large intestine 61	1.587428	0.00528
breast 2	1.355365	0.00552
Mobilized CD4 323	1.598014	0.00580
CD3 11	1.540696	0.00610
fetal fibroblast 273	1.356708	0.00666
Mobilized CD4 322	1.625495	0.00670
CD3 12	1.628692	0.00700
CD3 9	1.589404	0.00728
keratinocyte 334	1.307836	0.00776
fetal kidney 85	1.370600	0.00790
fetal adrenal gland 28	1.381586	0.00818
CD8 19	1.641324	0.00870
Mobilized CD3 320	1.607359	0.00924
fetal fibroblast 268	1.336573	0.00964
breast 1	1.317972	0.01060
melanocyte 337	1.296435	0.01090
fetal kidney 91	1.386793	0.01110
fetal fibroblast 275	1.321419	0.01132
melanocyte 338	1.306987	0.01162
fetal thymus 261	1.500723	0.01212
CD4 13	1.542170	0.01220
Mobilized CD8 325	1.562110	0.01290
fetal thymus 257	1.530463	0.01294
CD3 10	1.502420	0.01306
hematopoietic stem cell 308	1.357779	0.01324
fetal fibroblast 274	1.319143	0.01414
fetal adrenal gland 27	1.395062	0.01416
CD4 14	1.458826	0.01454
CD19 5	1.501184	0.01460
fetal fibroblast 282	1.310451	0.01466
fibroblast 331	1.313964	0.01470
fetal lung 298	1.355965	0.01498
fetal fibroblast 281	1.316618	0.01584
fetal fibroblast 272	1.304984	0.01648
CD56 17	1.410196	0.01686
Mobilized CD3 321	1.558976	0.01694
fetal fibroblast 278	1.312268	0.01732
CD8 20	1.447097	0.01740
fetal fibroblast 271	1.300032	0.01854
hematopoietic stem cell 309	1.345859	0.01862
fetal fibroblast 276	1.292494	0.01886
CD19 6	1.502803	0.02026
CD19 7	1.489958	0.02308
fetal lung 299	1.325406	0.02342
fetal fibroblast 269	1.277885	0.02368
hematopoietic stem cell 312	1.316345	0.02504
fetal fibroblast 277	1.292140	0.02616
melanocyte 340	1.296921	0.02672
fetal lung 133	1.288047	0.02678
fibroblast 332	1.273728	0.02772
CD4 15	1.554153	0.02858
fetal large intestine 67	1.350122	0.02870
fetal adrenal gland 26	1.343840	0.02872
melanocyte 339	1.266841	0.02874
fetal lung 140	1.281898	0.02916
hematopoietic stem cell 318	1.345557	0.03060
fetal small intestine 68	1.321172	0.03088
fetal lung 301	1.291957	0.03198
fibroblast 329	1.269098	0.03228
hematopoietic stem cell 315	1.319103	0.03270
hematopoietic stem cell 305	1.323611	0.03338
fetal lung 300	1.282568	0.03468
placenta 200	1.270294	0.03512
fetal lung 117	1.271266	0.03540
placenta 198	1.281426	0.03594
fetal lung 128	1.245350	0.03702
CD8 22	1.429266	0.03784
hematopoietic stem cell 317	1.260494	0.03806
fetal large intestine 64	1.305643	0.03898
fetal small intestine 78	1.281545	0.04012
fetal fibroblast 267	1.264591	0.04020
fetal fibroblast 279	1.250604	0.04050
fetal lung 141	1.247680	0.04064
fetal testes 255	1.293374	0.04102
hematopoietic stem cell 316	1.321061	0.04106
placenta 199	1.279446	0.04110
fetal large intestine 55	1.293701	0.04150
fetal renal pelvis 220	1.304082	0.04198
fetal adrenal gland 25	1.327738	0.04232
fetal lung 116	1.239795	0.04290
ovary 326	1.372408	0.04296
fetal fibroblast 280	1.236758	0.04440
hematopoietic stem cell 306	1.282380	0.04480
fetal muscle 172	1.248439	0.04480
fetal kidney 106	1.289560	0.04496
fetal fibroblast 270	1.243129	0.04654
fetal renal cortex 213	1.273685	0.04656
fetal lung 111	1.250779	0.04668
fetal heart 47	1.314916	0.04962
fetal heart 45	1.283651	0.04966
CD20 8	1.276214	0.05068
hematopoietic stem cell 307	1.275111	0.05110
fetal kidney 103	1.281743	0.05186
fetal muscle 180	1.286830	0.05250
fetal stomach 244	1.307576	0.05306
fibroblast 330	1.241523	0.05340
fetal large intestine 57	1.264785	0.05412
hematopoietic stem cell 311	1.265201	0.05428
fetal lung 120	1.244039	0.05552
fetal large intestine 60	1.286581	0.05644
fetal small intestine 75	1.466310	0.05778
fetal large intestine 58	1.252648	0.06008
placenta 197	1.266204	0.06134
fetal kidney 95	1.260794	0.06186
fetal renal pelvis 231	1.253069	0.06228
fetal adrenal gland 24	1.306188	0.06266
fetal kidney 87	1.260078	0.06268
fetal brain 40	1.206081	0.06402
fetal thymus 262	1.394112	0.06442
fetal small intestine 77	1.281714	0.06468
fetal brain 37	1.207548	0.06596
fetal lung 118	1.211963	0.06662
fetal renal cortex 207	1.254280	0.06740
ES derived MSC 289	1.250979	0.06766
ES derived MSC 290	1.245349	0.06956
hematopoietic stem cell 313	1.230522	0.07128
fetal brain 31	1.169628	0.07166
small intestine 342	1.257873	0.07300
fetal small intestine 74	1.256240	0.07340
fetal lung 115	1.212644	0.07416
CD4 16	1.353021	0.07446
fetal renal pelvis 227	1.242382	0.07548
fetal lung 121	1.206016	0.07670
fetal kidney 92	1.234933	0.07774
fetal lung 114	1.197560	0.07788
fetal large intestine 54	1.251098	0.08024
fetal kidney 89	1.241930	0.08046
fetal small intestine 70	1.217241	0.08094
fetal kidney 100	1.239946	0.08128
fetal small intestine 73	1.252336	0.08330
fetal lung 119	1.204093	0.08342
fetal adrenal gland 23	1.220565	0.08714
fetal small intestine 80	1.235915	0.08796
ES derived trophoblast 288	1.204195	0.08812
fetal kidney 83	1.260485	0.08828
fetal stomach 248	1.253469	0.09074
fetal lung 134	1.210612	0.09100
fetal renal pelvis 221	1.216778	0.09122
fetal lung 132	1.203189	0.09198
fetal kidney 99	1.223013	0.09200
ES derived trophoblast 287	1.222913	0.09390
fetal heart 49	1.223147	0.09486
fetal brain 30	1.144187	0.09520
fetal renal pelvis 228	1.240333	0.09616
fetal small intestine 76	1.240593	0.09818
fetal large intestine 63	1.234511	0.10008
fetal renal cortex 214	1.214186	0.10046
ES derived mesoderm 286	1.171058	0.10046
stomach 283	1.242633	0.10340
fetal renal pelvis 229	1.207945	0.10474
CD14 3	1.251717	0.10484
fetal renal pelvis 232	1.222903	0.10510
fetal lung 124	1.193617	0.10564
fetal kidney 84	1.216886	0.10690
placenta 196	1.211767	0.10844
fetal large intestine 66	1.229633	0.11020
fetal muscle 181	1.199520	0.11066
fetal brain 36	1.168423	0.11116
fetal large intestine 59	1.227926	0.11182
fetal heart 44	1.237310	0.11226
fetal small intestine 69	1.228528	0.11252
fetal muscle 149	1.207556	0.11256
fetal small intestine 72	1.209977	0.11278
fetal renal pelvis 226	1.198802	0.11294
fetal muscle 160	1.203620	0.11314
fetal lung 126	1.171144	0.11402
fetal lung 131	1.165631	0.11404
fetal stomach 243	1.209831	0.11686
fetal large intestine 53	1.235413	0.11696
fetal brain 34	1.147399	0.11760
fetal renal cortex 205	1.200295	0.11882
placenta 201	1.211311	0.11962
fetal brain 29	1.134728	0.12130
fetal muscle 154	1.204632	0.12146
fetal heart 42	1.196777	0.12170
fetal small intestine 79	1.214430	0.12324
fetal lung 130	1.172420	0.12568
iPS 303	1.186723	0.12612
fetal lung 129	1.171588	0.13034
fetal heart 48	1.185084	0.13066
iPS 302	1.187995	0.13076
fetal large intestine 56	1.191981	0.13182
fetal heart 46	1.191416	0.13282
fetal lung 125	1.174177	0.13476
fetal large intestine 62	1.209576	0.13512
fetal skin 234	1.185169	0.13636
ES derived mesoderm 285	1.156015	0.13680
fetal muscle 153	1.188896	0.13758
fetal brain 39	1.141434	0.13818
fetal lung 135	1.165691	0.14004
fetal lung 123	1.166163	0.14030
fetal lung 112	1.163821	0.14070
fetal lung 142	1.151846	0.14214
fetal testes 254	1.185389	0.14258
fetal lung 136	1.165592	0.14264
fetal muscle 168	1.176546	0.14634
fetal muscle 194	1.155979	0.14640
fetal muscle 176	1.164945	0.14764
fetal large intestine 65	1.189034	0.14766
fetal stomach 253	1.205452	0.14816
fetal muscle 151	1.181385	0.14830
fetal stomach 251	1.191646	0.14878
fetal muscle 159	1.179125	0.15052
fetal lung 113	1.145241	0.15146
fetal stomach 252	1.191094	0.15256
fetal renal pelvis 233	1.173977	0.15372
fetal renal pelvis 217	1.177010	0.15396
CD14 4	1.189871	0.15454
fetal muscle 182	1.159656	0.15554
fetal brain 41	1.133891	0.15690
iPS 304	1.159748	0.15730
ES derived NP 291	1.200690	0.15786
fetal kidney 105	1.157481	0.15808
hematopoietic stem cell 319	1.173864	0.15848
fetal brain 35	1.118060	0.15916
fetal muscle 167	1.156877	0.15948
fetal lung 110	1.156746	0.15952
fetal muscle 148	1.163810	0.15968
fetal thymus 263	1.163887	0.16124
fetal ovary 195	1.204476	0.16210
fetal brain 38	1.137251	0.16270
fetal muscle 187	1.153073	0.16292
fetal muscle 178	1.161250	0.16348
fetal renal cortex 215	1.154287	0.16402
heart 297	1.174625	0.16504
fetal muscle 155	1.169816	0.16506
fetal kidney 98	1.164766	0.16608
fetal lung 108	1.157649	0.16624
pancreas 328	1.177271	0.16674
fetal stomach 250	1.184257	0.16966
fetal muscle 193	1.156403	0.17368
fetal kidney 94	1.163922	0.17374
fetal renal cortex 202	1.147755	0.17822
fetal small intestine 71	1.159672	0.17946
fetal muscle 164	1.149843	0.17990
fetal heart 52	1.138762	0.18046
fetal muscle 169	1.145377	0.18254
fetal kidney 102	1.156935	0.18320
fetal renal pelvis 230	1.159705	0.18416
fetal heart 51	1.143979	0.18724
fetal stomach 246	1.171634	0.18736
fetal lung 138	1.134704	0.18762
fetal muscle 179	1.140432	0.18798
ES cells 295	1.170825	0.19032
fetal muscle 185	1.145904	0.19174
fetal renal pelvis 224	1.134378	0.19476
pancreas 327	1.160010	0.19674
fetal stomach 240	1.164301	0.19762
fetal muscle 166	1.131812	0.19776
fetal muscle 143	1.126560	0.19862
fetal lung 137	1.121966	0.20196
fetal renal cortex 211	1.143488	0.20596
fetal muscle 157	1.123291	0.20622
fetal heart 43	1.124657	0.20724
fetal muscle 152	1.126875	0.20814
fetal muscle 150	1.134795	0.20858
ES cells 294	1.151704	0.21226
fetal kidney 101	1.127836	0.21300
fetal muscle 165	1.116133	0.21840
fetal muscle 171	1.120913	0.21886
ES cells 296	1.149547	0.21918
fetal kidney 88	1.120843	0.21924
fetal muscle 163	1.126554	0.22052
fetal kidney 107	1.124280	0.22152
fetal renal pelvis 216	1.129810	0.22196
fetal kidney 82	1.136592	0.22258
fetal muscle 173	1.123231	0.22266
fetal stomach 242	1.165270	0.22354
fetal kidney 96	1.119684	0.22354
fetal lung 139	1.118389	0.22414
fetal renal pelvis 223	1.130029	0.22728
fetal muscle 192	1.114731	0.22866
fetal spinal cord 236	1.114108	0.23100
muscle 341	1.137356	0.23166
ES derived NP 292	1.134906	0.23246
stomach 284	1.130228	0.23346
fetal kidney 97	1.117454	0.23494
fetal spinal cord 235	1.102179	0.23992
fetal kidney 104	1.116745	0.24326
fetal renal cortex 209	1.112464	0.24606
fetal lung 127	1.103574	0.24624
fetal muscle 170	1.103993	0.24668
fetal stomach 249	1.128164	0.24990
fetal renal cortex 210	1.113405	0.25214
fetal renal pelvis 225	1.109721	0.25358
fetal lung 122	1.101869	0.25624
fetal renal cortex 212	1.101020	0.25672
fetal spinal cord 238	1.094828	0.25902
fetal renal cortex 206	1.102173	0.25926
fetal renal pelvis 222	1.106653	0.25960
fetal stomach 247	1.131561	0.26002
fetal muscle 174	1.101818	0.26110
fetal stomach 241	1.120252	0.26140
fetal lung 109	1.082496	0.26254
fetal brain 33	1.086888	0.26330
fetal brain 32	1.080339	0.26408
fetal renal cortex 204	1.092230	0.27052
fetal heart 50	1.098263	0.27110
fetal renal cortex 208	1.098415	0.27282
fetal stomach 245	1.104896	0.27564
fetal muscle 156	1.087856	0.28358
fetal kidney 90	1.090347	0.28856
fetal renal pelvis 219	1.101818	0.29794
fetal muscle 161	1.081924	0.29800
ES cells 293	1.089868	0.30128
fetal spinal cord 239	1.073356	0.30132
fetal muscle 184	1.074733	0.30200
fetal muscle 175	1.078047	0.30418
fetal muscle 158	1.070443	0.31872
fetal spinal cord 237	1.065284	0.32194
fetal muscle 189	1.071167	0.32224
fetal muscle 186	1.070090	0.32244
fetal muscle 147	1.069246	0.32550
fetal renal cortex 203	1.069467	0.32976
fetal muscle 183	1.062003	0.33534
fetal kidney 93	1.068147	0.33772
fetal muscle 190	1.061608	0.34858
fetal kidney 86	1.063169	0.35850
fetal renal pelvis 218	1.061848	0.36422
fetal muscle 188	1.046178	0.37504
fetal muscle 145	1.049950	0.37814
fetal muscle 191	1.040701	0.39254
fetal muscle 146	1.024989	0.42808
fetal muscle 177	1.023078	0.43388
fetal muscle 162	1.021871	0.43422
fetal kidney 81	1.017028	0.44606
fetal muscle 144	1.015054	0.44870

Coronary artery disease

DHS sample	fold enrichment	p value
fetal adrenal gland 28	1.5674848	0.00016
heart 297	1.7079830	0.00020
fetal adrenal gland 27	1.5707139	0.00034
ES derived MSC 289	1.5732989	0.00036
ES derived MSC 290	1.5517348	0.00042
fetal heart 52	1.5484439	0.00048
fetal adrenal gland 25	1.6165782	0.00050
fetal adrenal gland 26	1.5689471	0.00052
fetal testes 255	1.5140866	0.00060
fetal adrenal gland 24	1.6194420	0.00076
fibroblast 332	1.4584456	0.00082
fibroblast 331	1.4449862	0.00084
fetal adrenal gland 23	1.4998126	0.00090
fetal heart 43	1.5151904	0.00104
fetal fibroblast 269	1.4463374	0.00108
fibroblast 329	1.4386744	0.00122
fetal heart 48	1.5222287	0.00130
fetal muscle 172	1.4622299	0.00130
fetal fibroblast 270	1.4538083	0.00130
fetal lung 300	1.4539623	0.00136
fetal heart 51	1.5232479	0.00138
fetal muscle 178	1.5351546	0.00146
fetal heart 42	1.5244964	0.00146
fetal muscle 146	1.5027513	0.00146
fetal fibroblast 280	1.4292281	0.00148
fetal fibroblast 279	1.4377034	0.00156
fetal heart 47	1.5847193	0.00168
fetal heart 50	1.5067989	0.00178
fetal renal cortex 213	1.4580906	0.00178
fibroblast 330	1.4240237	0.00178
fetal muscle 179	1.5083880	0.00182
fetal muscle 168	1.4985076	0.00182
fetal muscle 167	1.4765455	0.00184
fetal muscle 143	1.4594278	0.00198
fetal heart 49	1.5296454	0.00212
fetal muscle 165	1.4526758	0.00214
fetal heart 46	1.5355032	0.00220
fetal kidney 92	1.4731869	0.00226
fetal muscle 192	1.4643181	0.00230
fetal lung 301	1.4329936	0.00236
fetal muscle 166	1.4588607	0.00238
fetal lung 128	1.3814118	0.00246
fetal renal cortex 207	1.4823114	0.00252
fetal lung 298	1.4434191	0.00254
fetal muscle 169	1.4774969	0.00278
fetal heart 45	1.5157418	0.00288
fetal muscle 149	1.5008946	0.00288
fetal muscle 154	1.5095722	0.00292
fetal muscle 164	1.4721900	0.00298
ovary 326	1.6072702	0.00306
melanocyte 338	1.3354985	0.00306
fetal muscle 181	1.4750138	0.00308
fetal muscle 182	1.4340250	0.00308
fetal lung 133	1.3850217	0.00316
fetal muscle 150	1.4764432	0.00328
fetal fibroblast 278	1.3951850	0.00330
fetal testes 254	1.4645530	0.00342
fetal lung 111	1.3985190	0.00348
fetal muscle 147	1.4589762	0.00354
fetal lung 129	1.3975789	0.00358
fetal thymus 263	1.4473294	0.00378
fetal lung 137	1.3949067	0.00378
fetal lung 299	1.4252041	0.00382
fetal muscle 174	1.4472236	0.00414
fetal muscle 189	1.4357641	0.00424
fetal muscle 175	1.4342563	0.00430
fetal muscle 171	1.4241754	0.00430
fetal lung 132	1.3955759	0.00438
fetal muscle 184	1.3983619	0.00444
fetal muscle 159	1.4489035	0.00450
fetal lung 119	1.3628454	0.00450
pancreas 327	1.5286495	0.00454
fetal muscle 148	1.4556879	0.00460
fetal muscle 151	1.4820467	0.00474
fetal fibroblast 272	1.3629164	0.00492
fetal muscle 183	1.4078178	0.00494
fetal lung 121	1.3501855	0.00500
fetal muscle 193	1.4427716	0.00504
fetal lung 140	1.3660447	0.00504
fetal muscle 157	1.3964390	0.00506
fetal heart 44	1.5595697	0.00508
fetal muscle 161	1.4393763	0.00516
fetal muscle 191	1.4489163	0.00530
fetal lung 141	1.3640888	0.00532
fetal fibroblast 275	1.3525387	0.00564
fetal lung 118	1.3441515	0.00574
fetal muscle 187	1.4015099	0.00582
fetal muscle 162	1.4049479	0.00608
fetal muscle 186	1.3980931	0.00628
fetal muscle 180	1.4395028	0.00630
fetal fibroblast 282	1.3416619	0.00634
fetal fibroblast 268	1.3479680	0.00644
fetal muscle 156	1.4017413	0.00650
fetal muscle 160	1.4243521	0.00664
fetal muscle 155	1.4476493	0.00668
fetal lung 125	1.3845613	0.00674
fetal renal cortex 214	1.4205273	0.00678
fetal fibroblast 276	1.3344561	0.00684
fetal kidney 81	1.3846663	0.00700
fetal muscle 190	1.4446227	0.00706
fetal muscle 158	1.3866008	0.00714
fetal muscle 188	1.3924765	0.00718
fetal muscle 194	1.3590792	0.00720
fetal kidney 106	1.4094971	0.00732
fetal muscle 153	1.4369478	0.00736
melanocyte 339	1.3123297	0.00754
fetal fibroblast 267	1.3603094	0.00760
fetal renal pelvis 228	1.4555438	0.00772
fetal kidney 85	1.3604559	0.00794
fetal lung 110	1.3797107	0.00806
ES derived mesoderm 286	1.3165455	0.00822
fetal kidney 87	1.4078681	0.00824
fetal kidney 95	1.4097520	0.00830
fetal muscle 185	1.4148522	0.00842
fetal lung 131	1.3206267	0.00850
fetal fibroblast 273	1.3356699	0.00858
fetal lung 120	1.3509255	0.00862
fetal muscle 144	1.4175364	0.00908
fetal muscle 177	1.4217167	0.00912
fetal muscle 152	1.3861062	0.00936
fetal muscle 176	1.3824648	0.00944
fetal fibroblast 277	1.3480032	0.00946
fetal muscle 145	1.4469811	0.00966
fetal lung 114	1.3176647	0.00980
fetal muscle 163	1.4001323	0.00986
fetal lung 138	1.3535522	0.00990
fetal kidney 100	1.4104575	0.01002
fetal muscle 170	1.3704819	0.01012
fetal renal pelvis 220	1.4010108	0.01018
fetal muscle 173	1.3891953	0.01028
fetal lung 134	1.3626998	0.01034
muscle 341	1.4475984	0.01056
fetal lung 108	1.3765835	0.01078
melanocyte 337	1.2723384	0.01114
fetal renal pelvis 226	1.3898170	0.01128
fetal fibroblast 271	1.3213326	0.01128
fetal lung 126	1.3148423	0.01132
fetal fibroblast 274	1.3292692	0.01146
fetal lung 142	1.3187436	0.01152
fetal lung 123	1.3434497	0.01174
fetal kidney 103	1.3967827	0.01182
fetal kidney 91	1.3508207	0.01182
pancreas 328	1.4333617	0.01194
fetal lung 127	1.3526601	0.01198
fetal lung 115	1.3203648	0.01238
fetal lung 117	1.3125857	0.01272
fetal lung 139	1.3557492	0.01292
fetal renal pelvis 232	1.4022409	0.01358
fetal lung 116	1.2975731	0.01386
fetal renal pelvis 231	1.3617472	0.01396
fetal renal cortex 202	1.3708218	0.01414
fetal renal pelvis 227	1.3766783	0.01436
ES derived mesoderm 285	1.3030405	0.01468
fetal kidney 84	1.3947909	0.01476
fetal renal cortex 206	1.3674862	0.01476
fetal kidney 89	1.3853061	0.01486
fetal renal pelvis 221	1.3574629	0.01510
fetal lung 109	1.2861102	0.01516
fetal brain 36	1.3074808	0.01572
fetal renal pelvis 217	1.3945216	0.01574
fetal lung 130	1.3121889	0.01606
fetal renal pelvis 216	1.4011078	0.01624
fetal lung 113	1.3065542	0.01632
fetal lung 135	1.3271616	0.01652
iPS 304	1.3329716	0.01660
fetal lung 124	1.3176887	0.01662
fetal large intestine 63	1.3923019	0.01692
fetal lung 136	1.3172463	0.01720
fetal large intestine 56	1.3481306	0.01734
fetal large intestine 66	1.3847489	0.01814
iPS 302	1.3343060	0.01886
ES cells 295	1.3954444	0.01896
fetal kidney 99	1.3541951	0.01952
melanocyte 340	1.2842768	0.01980
fetal renal cortex 205	1.3522948	0.02018
fetal stomach 251	1.3789781	0.02034
CD20 8	1.3276377	0.02072
fetal renal pelvis 230	1.3858487	0.02148
ES cells 294	1.3888956	0.02160
fetal kidney 83	1.3935251	0.02174
fetal stomach 248	1.3865334	0.02190
fetal large intestine 54	1.3472420	0.02294
fetal renal pelvis 224	1.3302156	0.02304
fetal renal pelvis 233	1.3440625	0.02342
fetal kidney 94	1.3664947	0.02360
fetal kidney 88	1.3341452	0.02378
fetal lung 112	1.2938822	0.02426
fetal stomach 243	1.3437478	0.02468
fetal large intestine 62	1.3692386	0.02498
fetal small intestine 71	1.3457504	0.02498
fetal stomach 253	1.3930000	0.02528
iPS 303	1.3031673	0.02554
fetal large intestine 53	1.3812151	0.02598
fetal stomach 252	1.3666083	0.02634
fetal renal pelvis 229	1.3287709	0.02640
fetal small intestine 72	1.3300124	0.02666
fetal kidney 96	1.3275644	0.02666
fetal renal cortex 212	1.3183498	0.02668
fetal large intestine 64	1.3185990	0.02772
fetal skin 234	1.3169397	0.02782
fetal small intestine 78	1.2914293	0.02790
fetal spinal cord 237	1.2987713	0.02806
fetal renal cortex 211	1.3518076	0.02850
fetal fibroblast 281	1.2657354	0.02910
fetal renal cortex 204	1.3095859	0.02920
fetal small intestine 79	1.3500658	0.02922
fetal kidney 98	1.3349845	0.02950
ES derived NP 291	1.3708331	0.02958
stomach 284	1.3582112	0.02960
ES cells 296	1.3700315	0.02982
fetal kidney 105	1.3152432	0.02982
fetal lung 122	1.3027636	0.02984
fetal renal pelvis 219	1.3997335	0.03046
fetal stomach 246	1.3729438	0.03068
fetal renal cortex 203	1.3310315	0.03084
fetal large intestine 59	1.3389015	0.03096
fetal renal pelvis 222	1.3388341	0.03102
fetal stomach 242	1.4265828	0.03132
fetal renal pelvis 223	1.3501926	0.03136
ES derived trophoblast 288	1.2854678	0.03172
fetal kidney 82	1.3633612	0.03180
fetal small intestine 69	1.3450985	0.03216
fetal renal cortex 215	1.3084284	0.03226
fetal large intestine 55	1.2959203	0.03272
fetal small intestine 73	1.3201009	0.03286
ES derived NP 292	1.3428489	0.03384
fetal large intestine 65	1.3233594	0.03410
fetal large intestine 67	1.3050243	0.03442
fetal small intestine 80	1.2903794	0.03462
fetal stomach 250	1.3537453	0.03522
fetal kidney 97	1.3123428	0.03566
fetal spinal cord 239	1.2806394	0.03574
fetal small intestine 76	1.3360473	0.03584
fetal brain 41	1.2467419	0.03588
stomach 283	1.3596381	0.03622
fetal brain 40	1.2297314	0.03726
fetal kidney 104	1.3159310	0.03792
fetal ovary 195	1.3556463	0.03850
fetal stomach 249	1.3544162	0.03882
fetal stomach 244	1.3177852	0.03888
fetal small intestine 70	1.2615743	0.03956
fetal renal cortex 210	1.3182844	0.04006
fetal stomach 245	1.3340495	0.04072
ES derived trophoblast 287	1.2945654	0.04072
fetal small intestine 74	1.2904094	0.04090
fetal renal pelvis 225	1.3122636	0.04320
fetal small intestine 77	1.2878085	0.04360
fetal large intestine 57	1.2617556	0.04526
fetal renal pelvis 218	1.3654804	0.04554
fetal stomach 240	1.3432411	0.04558
fetal brain 35	1.1982475	0.04608
fetal spinal cord 236	1.2714976	0.04630
keratinocyte 336	1.2014768	0.04902
fetal large intestine 58	1.2546126	0.04982
fetal brain 34	1.2000181	0.05044
fetal kidney 101	1.2757988	0.05086
fetal large intestine 60	1.2792329	0.05108
fetal kidney 102	1.2979128	0.05160
fetal kidney 90	1.2904490	0.05336
fetal renal cortex 209	1.2800379	0.05434
fetal brain 39	1.2080930	0.05606
fetal brain 33	1.2358369	0.05678
fetal kidney 93	1.2891081	0.05780
fetal spinal cord 238	1.2374265	0.05872
fetal brain 38	1.2218089	0.05880
fetal stomach 241	1.2995880	0.05920
small intestine 342	1.2664616	0.05972
fetal renal cortex 208	1.2759768	0.05984
fetal small intestine 68	1.2434963	0.06230
fetal stomach 247	1.3257386	0.06686
fetal spinal cord 235	1.2212626	0.07194
placenta 201	1.2487538	0.07738
fetal brain 29	1.1561535	0.07792
keratinocyte 333	1.1712803	0.07828
fetal kidney 107	1.2403650	0.07864
keratinocyte 335	1.1739579	0.07970
hematopoietic stem cell 314	1.2032707	0.08044
ES cells 293	1.2445234	0.08172
fetal brain 37	1.1839772	0.08266
keratinocyte 334	1.1681824	0.08332
fetal kidney 86	1.2784140	0.08538
fetal brain 32	1.1856948	0.08634
placenta 196	1.2177237	0.09516
hematopoietic stem cell 318	1.2150478	0.09944
hematopoietic stem cell 315	1.2037386	0.10366
fetal brain 31	1.1391210	0.10544
hematopoietic stem cell 305	1.1990630	0.10658
placenta 200	1.1754713	0.11322
fetal brain 30	1.1291240	0.11366
placenta 199	1.1872078	0.11446
breast 2	1.1555007	0.11732
hematopoietic stem cell 308	1.1753528	0.12240
hematopoietic stem cell 310	1.1551608	0.12498
hematopoietic stem cell 316	1.1882237	0.12858
hematopoietic stem cell 312	1.1655075	0.13076
breast 1	1.1428562	0.13632
hematopoietic stem cell 307	1.1640453	0.14324
hematopoietic stem cell 309	1.1632836	0.14544
hematopoietic stem cell 319	1.1795530	0.14580
fetal thymus 260	1.1963439	0.16126
fetal thymus 258	1.1912837	0.16164
placenta 198	1.1430118	0.16840
placenta 197	1.1532431	0.17116
fetal thymus 264	1.1809437	0.17120
CD19 7	1.1992932	0.17308
hematopoietic stem cell 311	1.1408574	0.17638
hematopoietic stem cell 313	1.1327463	0.18124
hematopoietic stem cell 306	1.1331733	0.18834
fetal thymus 256	1.1581849	0.20068
hematopoietic stem cell 317	1.1085940	0.20632
fetal thymus 261	1.1392022	0.23972
fetal thymus 259	1.1401589	0.24078
fetal thymus 265	1.1289504	0.24776
CD8 21	1.1402629	0.25052
fetal large intestine 61	1.1277230	0.25146
fetal thymus 266	1.1262340	0.25248
fetal small intestine 75	1.1471003	0.25484
CD19 6	1.1314971	0.25648
Mobilized CD56 324	1.1229165	0.25738
fetal thymus 257	1.1281828	0.26048
Mobilized CD8 325	1.1240487	0.26802
CD3 12	1.1260105	0.26972
CD56 18	1.1229356	0.27582
CD19 5	1.1092496	0.28300
Mobilized CD4 322	1.1143656	0.28444
CD8 22	1.1095271	0.28686
fetal thymus 262	1.1148093	0.29406
Mobilized CD4 323	1.1085669	0.29444
Mobilized CD3 320	1.1153603	0.29492
CD4 15	1.1198014	0.29646
CD8 19	1.1124886	0.29660
Mobilized CD3 321	1.1017620	0.31084
CD8 20	1.0866654	0.31540
CD3 10	1.0793786	0.33258
CD14 4	1.0755412	0.33414
CD3 9	1.0780000	0.33772
CD14 3	1.0751755	0.33852
CD4 13	1.0757968	0.34714
CD4 14	1.0555380	0.37106
CD4 16	1.0494818	0.38918
CD56 17	1.0237398	0.43422
CD3 11	0.9980205	0.49226

Systemic lupus erythematosus 1

DHS sample	fold enrichment	p value
Mobilized CD3 320	1.8426036	0.00168
CD3 10	1.7072049	0.00236
CD19 5	1.6942106	0.00338
Mobilized CD56 324	1.6059644	0.00708
CD19 6	1.6513178	0.00844
CD56 17	1.5243649	0.01026
CD3 11	1.5409267	0.01088
CD8 20	1.5246699	0.01298
CD4 13	1.5986930	0.01368
CD19 7	1.6001759	0.01510
CD56 18	1.5629354	0.01828
CD4 14	1.4703587	0.02048
CD3 9	1.5198968	0.02136
CD8 21	1.5076448	0.03062
CD3 12	1.4635858	0.04316
CD14 4	1.3720751	0.04528
Mobilized CD4 322	1.4431791	0.04860
CD8 22	1.4273752	0.05290
Mobilized CD4 323	1.4196757	0.05326
fetal thymus 256	1.3306789	0.05732
CD14 3	1.3323644	0.07552
CD8 19	1.3927551	0.08082
fetal thymus 264	1.2934727	0.08588
Mobilized CD3 321	1.3617940	0.09038
CD4 16	1.3507199	0.09342
fetal thymus 258	1.2553744	0.12326
hematopoietic stem cell 310	1.1713428	0.13412
fetal thymus 265	1.2258211	0.14626
fetal fibroblast 273	1.1464000	0.16800
fetal thymus 266	1.2015011	0.16842
ES derived MSC 289	1.1643024	0.17724
ES derived MSC 290	1.1642050	0.17796
fetal large intestine 61	1.2070850	0.18312
Mobilized CD8 325	1.2047857	0.19802
hematopoietic stem cell 317	1.1294442	0.20220
fetal thymus 260	1.1725752	0.20406
CD4 15	1.2233540	0.20532
fetal fibroblast 268	1.1173800	0.21824
fetal thymus 259	1.1583737	0.23578
fetal thymus 261	1.1529506	0.23982
fetal fibroblast 267	1.1122869	0.24304
fetal small intestine 75	1.1693983	0.25170
hematopoietic stem cell 312	1.1121711	0.25250
fetal fibroblast 272	1.0977562	0.25714
hematopoietic stem cell 314	1.1027297	0.26386
fetal fibroblast 274	1.0878824	0.27868
ES derived mesoderm 285	1.0915022	0.28300
fibroblast 331	1.0838401	0.28920
hematopoietic stem cell 313	1.0887889	0.29184
fetal fibroblast 276	1.0786584	0.29364
fetal fibroblast 275	1.0746932	0.30236
fetal fibroblast 282	1.0713535	0.30980
fetal fibroblast 278	1.0753216	0.31030
fetal fibroblast 281	1.0727620	0.31298
fetal thymus 257	1.0996465	0.31708
fetal fibroblast 277	1.0733348	0.31724
fetal fibroblast 279	1.0687444	0.32394
fibroblast 332	1.0673705	0.32506
fetal lung 301	1.0672767	0.33592
hematopoietic stem cell 318	1.0721385	0.33610
fetal fibroblast 271	1.0599120	0.34314
fetal fibroblast 280	1.0488499	0.36600
hematopoietic stem cell 306	1.0537998	0.36770
fetal lung 300	1.0451426	0.38134
hematopoietic stem cell 305	1.0447875	0.38892
fetal heart 47	1.0443061	0.39082
fetal thymus 262	1.0474477	0.40026
keratinocyte 336	1.0291195	0.41304
hematopoietic stem cell 316	1.0327528	0.41492
fetal fibroblast 269	1.0281388	0.41742
keratinocyte 335	1.0261102	0.42214
fetal heart 45	1.0277887	0.42702
keratinocyte 334	1.0220848	0.42860
hematopoietic stem cell 309	1.0225326	0.44010
ES derived mesoderm 286	1.0184420	0.44106
fetal fibroblast 270	1.0186291	0.44264
keratinocyte 333	1.0170438	0.44364
hematopoietic stem cell 308	1.0146971	0.45142
hematopoietic stem cell 311	1.0137399	0.45352
fetal lung 298	1.0156928	0.45464
breast 1	1.0119733	0.46140
hematopoietic stem cell 315	1.0105591	0.46394
hematopoietic stem cell 307	1.0066704	0.47078
fetal heart 46	0.9998423	0.48430
fetal heart 49	0.9982064	0.49070
fetal testes 255	0.9925236	0.49826
fibroblast 329	0.9899896	0.51534
fetal heart 43	0.9864201	0.51878
fetal heart 48	0.9832691	0.52206
fetal heart 42	0.9835461	0.52538
breast 2	0.9857601	0.52752
heart 297	0.9769561	0.53202
fetal lung 299	0.9731521	0.55270
fibroblast 330	0.9745324	0.55386
fetal heart 51	0.9675192	0.56040
ovary 326	0.9499501	0.56232
melanocyte 337	0.9722559	0.57374
hematopoietic stem cell 319	0.9509663	0.58606
fetal heart 52	0.9573813	0.58670
fetal heart 50	0.9501192	0.59490
fetal small intestine 70	0.9476518	0.61358
muscle 341	0.9267026	0.61704
fetal heart 44	0.9227564	0.62740
melanocyte 338	0.9457407	0.64204
fetal large intestine 54	0.9174598	0.64742
fetal large intestine 58	0.9277925	0.64792
fetal small intestine 68	0.9230972	0.65118
fetal large intestine 64	0.9202716	0.65334
small intestine 342	0.9152155	0.66596
fetal testes 254	0.9002384	0.68218
fetal small intestine 78	0.9111183	0.68574
melanocyte 340	0.9226421	0.68800
fetal muscle 176	0.9091108	0.68878
pancreas 328	0.8886420	0.69658
stomach 284	0.8887429	0.69786
fetal muscle 172	0.9118932	0.70036
fetal lung 111	0.9096003	0.71052
fetal small intestine 80	0.8921812	0.71282
melanocyte 339	0.9173731	0.71288
fetal small intestine 74	0.8877410	0.71332
fetal large intestine 62	0.8772204	0.71622
placenta 200	0.9030713	0.72404
fetal muscle 143	0.8949456	0.72746
fetal small intestine 72	0.8792455	0.72778
fetal large intestine 60	0.8752323	0.73306
fetal small intestine 73	0.8700091	0.73574
fetal small intestine 79	0.8630509	0.74040
fetal stomach 248	0.8449255	0.75650
CD20 8	0.8708985	0.75766
fetal large intestine 55	0.8638492	0.76470
iPS 302	0.8631565	0.76604
fetal stomach 240	0.8276055	0.76944
fetal large intestine 56	0.8510205	0.77818
ES derived NP 291	0.8346438	0.77930
fetal lung 126	0.8766715	0.78682
fetal lung 128	0.8823586	0.78892
placenta 197	0.8482423	0.78952
placenta 198	0.8636112	0.78968
fetal large intestine 65	0.8376497	0.79014
fetal ovary 195	0.8100992	0.79114
fetal muscle 152	0.8564392	0.79276
fetal lung 118	0.8740822	0.79494
fetal large intestine 57	0.8490453	0.79512
fetal lung 116	0.8766031	0.79552
fetal large intestine 63	0.8336325	0.79846
fetal skin 234	0.8402451	0.79936
fetal large intestine 67	0.8290861	0.80494
ES derived NP 292	0.8202048	0.80526
fetal muscle 180	0.8411260	0.80658
fetal muscle 165	0.8545620	0.80664
fetal small intestine 76	0.8189780	0.81186
fetal large intestine 66	0.8153865	0.81574
pancreas 327	0.8093176	0.81690
iPS 304	0.8391096	0.82160
ES cells 296	0.8038336	0.82180
ES derived trophoblast 287	0.8281309	0.82286
iPS 303	0.8339609	0.82322
fetal muscle 191	0.8319718	0.82362
ES derived trophoblast 288	0.8410476	0.82520
fetal stomach 242	0.7679290	0.82670
ES cells 295	0.8003741	0.82916
fetal lung 108	0.8309067	0.83384
fetal stomach 249	0.7832345	0.83462
ES cells 293	0.8033544	0.83520
fetal muscle 173	0.8232989	0.83772
fetal stomach 241	0.7840579	0.83796
fetal stomach 246	0.7835484	0.83916
fetal lung 121	0.8468872	0.83978
fetal muscle 194	0.8376968	0.84104
fetal muscle 174	0.8193630	0.84200
placenta 199	0.8263102	0.84264
fetal muscle 168	0.8184714	0.84316
fetal stomach 253	0.7807900	0.84330
fetal muscle 193	0.8180850	0.84372
placenta 201	0.8039278	0.84438
fetal stomach 244	0.7926047	0.84572
fetal lung 131	0.8454242	0.85550
fetal stomach 250	0.7702889	0.85590
fetal muscle 164	0.8083172	0.85672
fetal muscle 179	0.8134685	0.85720
fetal kidney 81	0.8224945	0.85900
fetal muscle 148	0.8046718	0.85956
stomach 283	0.7790809	0.86058
fetal lung 138	0.8189907	0.86224
fetal muscle 188	0.8132022	0.86312
fetal muscle 166	0.8112340	0.86516
fetal muscle 159	0.7946774	0.86534
fetal renal pelvis 219	0.7531242	0.86608
fetal lung 115	0.8262204	0.86898
fetal muscle 167	0.8087670	0.87056
fetal small intestine 69	0.7703509	0.87134
fetal muscle 147	0.7971759	0.87174
fetal muscle 162	0.8069807	0.87310
fetal lung 136	0.8109488	0.87318
fetal lung 141	0.8273173	0.87394
fetal muscle 169	0.7963248	0.87510
fetal muscle 163	0.7942611	0.87528
ES cells 294	0.7577430	0.87546
fetal muscle 192	0.8046255	0.87626
fetal lung 125	0.8049797	0.87630
fetal lung 123	0.8085175	0.87658
fetal kidney 96	0.7923686	0.87668
fetal lung 127	0.8075080	0.87714
fetal muscle 160	0.7910190	0.87806
fetal kidney 95	0.7789633	0.88028
fetal stomach 245	0.7615094	0.88092
fetal lung 133	0.8207902	0.88122
fetal lung 132	0.8068236	0.88134
fetal stomach 252	0.7602345	0.88148
fetal muscle 149	0.7832774	0.88356
fetal stomach 247	0.7225222	0.88470
fetal renal cortex 207	0.7815272	0.88476
fetal renal cortex 215	0.7903232	0.88524
fetal muscle 161	0.7890794	0.88530
fetal muscle 157	0.8053696	0.88548
fetal large intestine 59	0.7626637	0.88642
fetal muscle 158	0.7975551	0.88696
fetal muscle 182	0.7950130	0.88900
fetal muscle 187	0.7992302	0.88958
fetal lung 137	0.8070568	0.89006
fetal kidney 91	0.7838469	0.89008
fetal adrenal gland 24	0.7526119	0.89142
fetal muscle 155	0.7752781	0.89206
fetal muscle 175	0.7853921	0.89238
fetal muscle 186	0.7917075	0.89272
fetal muscle 151	0.7729136	0.89416
fetal kidney 102	0.7560863	0.89462
fetal lung 124	0.7962207	0.89472
fetal stomach 251	0.7511362	0.89476
fetal muscle 146	0.7851168	0.89510
fetal renal pelvis 221	0.7786919	0.89528
fetal lung 119	0.8108930	0.89698
fetal muscle 177	0.7682682	0.89772
fetal lung 120	0.7945247	0.89986
fetal lung 129	0.7979813	0.90142
fetal adrenal gland 26	0.7672323	0.90154
fetal muscle 185	0.7677820	0.90220
fetal kidney 90	0.7610115	0.90276
fetal muscle 153	0.7623825	0.90378
fetal renal pelvis 217	0.7504261	0.90436
fetal renal cortex 204	0.7747805	0.90470
fetal muscle 171	0.7833097	0.90486
fetal kidney 107	0.7654062	0.90500
fetal kidney 94	0.7461382	0.90526
fetal renal cortex 213	0.7732735	0.90602
fetal muscle 156	0.7736664	0.90770
fetal small intestine 77	0.7467824	0.90794
fetal muscle 154	0.7543816	0.90884
fetal thymus 263	0.7621596	0.90930
fetal small intestine 71	0.7462787	0.90946
fetal renal cortex 205	0.7550797	0.90956
fetal kidney 104	0.7534645	0.90966
fetal adrenal gland 27	0.7614640	0.90990
fetal renal cortex 208	0.7528879	0.91060
placenta 196	0.7587612	0.91062
fetal lung 110	0.7783684	0.91150
fetal kidney 84	0.7473428	0.91188
fetal muscle 145	0.7432882	0.91320
fetal muscle 181	0.7622225	0.91394
fetal muscle 183	0.7742412	0.91428
fetal renal pelvis 230	0.7310964	0.91440
fetal kidney 82	0.7279658	0.91476
fetal renal pelvis 227	0.7491472	0.91482
fetal lung 142	0.7965827	0.91492
fetal lung 113	0.7916607	0.91542
fetal muscle 144	0.7561180	0.91570
fetal muscle 189	0.7654933	0.91708
fetal lung 140	0.7858498	0.91766
fetal kidney 83	0.7148851	0.91772
fetal lung 117	0.7856201	0.91790
fetal kidney 92	0.7611704	0.91832
fetal kidney 103	0.7431095	0.91960
fetal renal cortex 209	0.7422645	0.91964
fetal renal pelvis 226	0.7492208	0.92150
fetal muscle 150	0.7503468	0.92238
fetal kidney 93	0.7320655	0.92276
fetal kidney 98	0.7389828	0.92456
fetal renal pelvis 218	0.6876180	0.92626
fetal renal cortex 206	0.7473497	0.92642
fetal renal cortex 211	0.7207836	0.92922
fetal muscle 184	0.7660709	0.92938
fetal lung 134	0.7599102	0.92944
fetal lung 109	0.7924310	0.92998
fetal renal pelvis 228	0.7129004	0.93014
fetal stomach 243	0.7238794	0.93106
fetal renal pelvis 222	0.7213236	0.93130
fetal lung 130	0.7650024	0.93174
fetal lung 122	0.7479057	0.93380
fetal renal pelvis 216	0.7276477	0.93380
fetal kidney 106	0.7339247	0.93396
fetal kidney 99	0.7312674	0.93462
fetal muscle 178	0.7347159	0.93508
fetal renal cortex 212	0.7438020	0.93520
fetal kidney 87	0.7304486	0.93558
fetal adrenal gland 28	0.7601009	0.93666
fetal lung 139	0.7491758	0.93772
fetal renal pelvis 220	0.7169485	0.93774
fetal kidney 101	0.7298335	0.93810
fetal lung 135	0.7518958	0.93854
fetal lung 114	0.7772136	0.93902
fetal large intestine 53	0.6806680	0.93954
fetal kidney 89	0.7151188	0.93974
fetal adrenal gland 23	0.7370565	0.94018
fetal renal pelvis 224	0.7352200	0.94130
fetal kidney 105	0.7257822	0.94166
fetal kidney 88	0.7273148	0.94182
fetal renal cortex 210	0.7095105	0.94232
fetal renal cortex 214	0.7232338	0.94302
fetal kidney 85	0.7533913	0.94412
fetal kidney 86	0.6694807	0.94432
fetal renal cortex 203	0.7116309	0.94520
fetal renal pelvis 225	0.7034042	0.94972
fetal renal pelvis 223	0.6930608	0.95012
fetal lung 112	0.7438352	0.95046
fetal kidney 97	0.7042843	0.95170
fetal adrenal gland 25	0.6949563	0.95178
fetal renal pelvis 229	0.7077226	0.95258
fetal kidney 100	0.7019297	0.95362
fetal renal pelvis 231	0.7185775	0.95470
fetal renal pelvis 233	0.7000871	0.95472
fetal renal pelvis 232	0.6879434	0.95532
fetal renal cortex 202	0.7039595	0.95568
fetal muscle 170	0.7193781	0.96090
fetal muscle 190	0.6926045	0.96214
fetal spinal cord 238	0.7225730	0.96220
fetal spinal cord 239	0.7195101	0.96306
fetal spinal cord 235	0.7110749	0.96996
fetal spinal cord 236	0.6861364	0.97440
fetal brain 41	0.7233549	0.97814
fetal brain 40	0.7139514	0.98564
fetal spinal cord 237	0.6679515	0.98564
fetal brain 36	0.6895936	0.98816
fetal brain 33	0.6702230	0.98882
fetal brain 35	0.7164808	0.99006
fetal brain 34	0.6992804	0.99040
fetal brain 39	0.6903543	0.99146
fetal brain 37	0.6809550	0.99176
fetal brain 29	0.7031300	0.99508
fetal brain 38	0.6426669	0.99586
fetal brain 32	0.6507241	0.99644
fetal brain 30	0.6753612	0.99786
fetal brain 31	0.6549625	0.99886

Metabolic traits 2

DHS sample	fold enrichment	p value
fetal thymus 258	1.4359353	0.01042
fetal thymus 256	1.4018970	0.01138
small intestine 342	1.3552041	0.01472
CD14 4	1.3994056	0.01480
fetal adrenal gland 28	1.2576853	0.02798
fetal thymus 265	1.3489443	0.02944
fetal fibroblast 269	1.2326860	0.03138
fetal thymus 260	1.3447896	0.03244
fetal large intestine 64	1.2932294	0.03404
fetal fibroblast 282	1.2263196	0.03604
fibroblast 331	1.2352907	0.03848
muscle 341	1.3232161	0.03862
fibroblast 332	1.2321852	0.03922
fetal lung 300	1.2415657	0.03960
fetal fibroblast 274	1.2229339	0.04410
fetal lung 301	1.2384241	0.04804
fetal lung 298	1.2435286	0.04808
fibroblast 329	1.2248458	0.04862
keratinocyte 333	1.1851867	0.04878
fetal lung 299	1.2381926	0.05006
fetal fibroblast 280	1.2060377	0.05310
fetal small intestine 70	1.2286402	0.05408
fetal fibroblast 273	1.2056333	0.05418
iPS 304	1.2315900	0.05446
fetal thymus 264	1.2852499	0.05544
fetal fibroblast 279	1.2049681	0.05548
fetal thymus 266	1.2873326	0.05604
fetal fibroblast 281	1.2035821	0.05832
fetal large intestine 57	1.2355064	0.05906
CD14 3	1.2939174	0.05924
keratinocyte 336	1.1762895	0.06034
ES derived mesoderm 286	1.1798583	0.06528
fetal small intestine 80	1.2371403	0.06748
keratinocyte 334	1.1708550	0.06800
fibroblast 330	1.2053358	0.06802
fetal small intestine 74	1.2409528	0.06808
fetal thymus 259	1.2874043	0.06838
fetal large intestine 55	1.2354158	0.06840
keratinocyte 335	1.1697542	0.06972
fetal small intestine 78	1.2161365	0.07152
fetal thymus 261	1.2677710	0.07596
fetal large intestine 60	1.2335712	0.07754
fetal large intestine 67	1.2303451	0.07948
fetal fibroblast 270	1.1811393	0.08100
ES derived MSC 290	1.2062864	0.08264
CD20 8	1.2143427	0.08340
hematopoietic stem cell 309	1.2074533	0.08464
ES cells 294	1.2299795	0.09036
hematopoietic stem cell 310	1.1771980	0.09170
ES derived mesoderm 285	1.1699662	0.09218
stomach 284	1.2306759	0.09298
ES derived MSC 289	1.1976096	0.09326
fetal small intestine 68	1.2043719	0.09358
fetal fibroblast 271	1.1676213	0.09564
fetal large intestine 56	1.2085704	0.09750
fetal fibroblast 268	1.1613387	0.10176
fetal adrenal gland 23	1.1775870	0.10368
fetal fibroblast 277	1.1633321	0.10656
fetal fibroblast 267	1.1664464	0.10736
breast 1	1.1567448	0.10770
fetal fibroblast 272	1.1585766	0.10796
fetal skin 234	1.1941572	0.10916
fetal fibroblast 278	1.1623434	0.10930
fetal adrenal gland 25	1.1905097	0.11084
fetal small intestine 73	1.2004004	0.11172
fetal large intestine 63	1.2018137	0.11204
hematopoietic stem cell 314	1.1691735	0.11448
fetal small intestine 69	1.2035384	0.11778
fetal large intestine 58	1.1762418	0.11878
fetal fibroblast 275	1.1439325	0.12094
iPS 303	1.1657340	0.12582
breast 2	1.1426349	0.12588
placenta 200	1.1585052	0.12600
ES cells 296	1.1964503	0.12780
fetal small intestine 79	1.1929196	0.12836
hematopoietic stem cell 312	1.1645457	0.12958
fetal large intestine 54	1.1812995	0.13092
fetal small intestine 72	1.1767223	0.13218
fetal thymus 262	1.2347925	0.13816
ES derived trophoblast 288	1.1508678	0.13972
fetal large intestine 62	1.1864127	0.14040
fetal fibroblast 276	1.1290254	0.14960
pancreas 328	1.1710994	0.15180
iPS 302	1.1503489	0.15230
fetal small intestine 71	1.1657521	0.15272
hematopoietic stem cell 306	1.1543637	0.15302
fetal thymus 257	1.1907797	0.15692
fetal small intestine 76	1.1676519	0.15976
hematopoietic stem cell 315	1.1537660	0.16142
fetal large intestine 65	1.1587226	0.16448
melanocyte 338	1.1085691	0.17470
fetal lung 119	1.1215002	0.17638
CD3 10	1.1669053	0.17654
hematopoietic stem cell 305	1.1374406	0.18128
fetal adrenal gland 27	1.1293068	0.18874
fetal kidney 91	1.1307988	0.19020
fetal renal pelvis 227	1.1372960	0.19060
fetal large intestine 59	1.1446191	0.19104
pancreas 327	1.1500026	0.19300
fetal lung 121	1.1098125	0.19616
fetal adrenal gland 24	1.1391534	0.19648
fetal adrenal gland 26	1.1257954	0.19732
melanocyte 337	1.0954841	0.19934
fetal small intestine 77	1.1335157	0.20062
CD19 5	1.1525087	0.20156
fetal lung 118	1.1032641	0.20280
placenta 198	1.1138841	0.20440
hematopoietic stem cell 318	1.1299889	0.20496
Mobilized CD56 324	1.1452322	0.20564
CD56 17	1.1338921	0.21018
hematopoietic stem cell 308	1.1132345	0.21192
hematopoietic stem cell 311	1.1159370	0.21430
fetal kidney 81	1.1066893	0.21596
fetal renal pelvis 228	1.1281336	0.21658
fetal muscle 172	1.1003063	0.21882
ES cells 295	1.1275648	0.22188
hematopoietic stem cell 317	1.0988902	0.22398
fetal renal cortex 213	1.1104512	0.22580
fetal lung 128	1.0907839	0.22590
fetal renal pelvis 220	1.1179385	0.22852
fetal lung 140	1.0987464	0.22886
hematopoietic stem cell 319	1.1145780	0.23028
fetal lung 133	1.0954011	0.23048
hematopoietic stem cell 307	1.1082615	0.23088
hematopoietic stem cell 313	1.1040724	0.23224
CD4 13	1.1388403	0.23472
fetal lung 132	1.0979573	0.23892
fetal large intestine 53	1.1219376	0.23980
hematopoietic stem cell 316	1.1083725	0.24402
fetal heart 43	1.0933682	0.24420
fetal kidney 95	1.1053709	0.24580
fetal lung 134	1.0921267	0.25046
fetal kidney 103	1.1016935	0.25252
fetal lung 125	1.0917230	0.25322
fetal large intestine 66	1.1087205	0.25328
fetal kidney 99	1.1000352	0.25334
placenta 197	1.1008183	0.25442
ES cells 293	1.1018360	0.25642
fetal lung 111	1.0845442	0.25642
CD19 6	1.1208197	0.25714
melanocyte 340	1.0841627	0.25850
fetal renal pelvis 217	1.0985641	0.26260
CD19 7	1.1183070	0.26384
fetal renal pelvis 231	1.0913069	0.26442
fetal lung 141	1.0784628	0.26446
fetal kidney 100	1.0946273	0.26594
CD8 21	1.1195448	0.26620
fetal lung 110	1.0845119	0.26664
stomach 283	1.1045141	0.26758
CD56 18	1.1175740	0.27056
fetal muscle 185	1.0903134	0.27296
heart 297	1.0937425	0.27490
fetal muscle 192	1.0804995	0.27706
CD4 16	1.1129303	0.27720
fetal lung 116	1.0712622	0.27862
fetal thymus 263	1.0833743	0.28410
fetal lung 120	1.0749756	0.28418
fetal stomach 244	1.0921800	0.28450
fetal muscle 157	1.0745844	0.28600
CD4 14	1.0942610	0.28840
fetal renal cortex 205	1.0831891	0.28860
fetal muscle 189	1.0780378	0.28880
Mobilized CD4 323	1.1029459	0.29012
Mobilized CD3 320	1.1050852	0.29156
fetal kidney 106	1.0811958	0.29208
fetal renal pelvis 232	1.0868996	0.29216
fetal muscle 175	1.0769783	0.29276
Mobilized CD8 325	1.1020276	0.29300
fetal heart 52	1.0730803	0.29402
placenta 201	1.0825449	0.29404
fetal renal pelvis 221	1.0754263	0.29668
fetal lung 138	1.0708831	0.29792
fetal lung 129	1.0695990	0.29922
fetal muscle 191	1.0753346	0.30112
fetal lung 131	1.0611745	0.30254
fetal lung 117	1.0671817	0.30288
ES derived trophoblast 287	1.0755961	0.30404
fetal lung 136	1.0687322	0.30424
fetal kidney 82	1.0809570	0.30430
fetal renal cortex 215	1.0720952	0.30560
fetal renal pelvis 233	1.0755950	0.30670
fetal lung 113	1.0609974	0.30938
fetal heart 45	1.0697717	0.30966
melanocyte 339	1.0579710	0.31098
fetal kidney 84	1.0742242	0.31196
fetal kidney 92	1.0687333	0.31230
fetal muscle 182	1.0654837	0.31684
CD3 9	1.0843752	0.31764
fetal heart 42	1.0669917	0.31822
fetal lung 109	1.0543496	0.31866
fetal kidney 105	1.0661488	0.32052
fetal muscle 167	1.0631132	0.32188
fetal renal cortex 207	1.0675225	0.32274
fetal kidney 87	1.0660114	0.32300
fetal renal pelvis 224	1.0625558	0.32336
fetal kidney 83	1.0730394	0.32576
fetal renal cortex 212	1.0630330	0.32764
fetal renal cortex 202	1.0637087	0.32864
fetal kidney 85	1.0553640	0.32908
fetal kidney 89	1.0646196	0.32956
fetal lung 112	1.0563323	0.33102
ES derived NP 292	1.0662780	0.33280
fetal renal pelvis 229	1.0626247	0.33328
fetal lung 123	1.0547932	0.33508
fetal lung 114	1.0513083	0.33532
fetal renal pelvis 219	1.0711393	0.33730
fetal kidney 102	1.0632257	0.33762
fetal brain 36	1.0489093	0.34090
Mobilized CD4 322	1.0733260	0.34240
fetal brain 39	1.0455331	0.34284
placenta 196	1.0582320	0.34326
fetal muscle 168	1.0560929	0.34446
fetal muscle 176	1.0541579	0.34450
fetal muscle 152	1.0538382	0.34668
fetal renal cortex 214	1.0556496	0.34680
fetal muscle 151	1.0579752	0.34780
fetal muscle 159	1.0549906	0.34970
fetal muscle 166	1.0526939	0.35176
fetal renal pelvis 223	1.0565003	0.35306
fetal muscle 148	1.0528433	0.35392
fetal brain 40	1.0420819	0.35434
fetal muscle 146	1.0495633	0.35438
CD8 22	1.0647017	0.35478
fetal lung 108	1.0506702	0.35510
fetal renal cortex 211	1.0555915	0.35584
fetal kidney 98	1.0525662	0.35678
CD8 19	1.0690846	0.35712
fetal heart 50	1.0498660	0.35716
ES derived NP 291	1.0570646	0.36010
CD8 20	1.0576586	0.36100
fetal renal cortex 206	1.0489981	0.36110
fetal lung 137	1.0444293	0.36210
ovary 326	1.0591420	0.36274
fetal kidney 96	1.0492911	0.36324
fetal muscle 194	1.0413094	0.36772
fetal muscle 171	1.0438906	0.36802
fetal heart 46	1.0469776	0.36838
fetal kidney 88	1.0444838	0.36962
fetal renal pelvis 218	1.0538798	0.37022
fetal renal pelvis 226	1.0460147	0.37162
fetal brain 41	1.0379651	0.37286
fetal heart 48	1.0432306	0.37374
fetal renal pelvis 225	1.0446792	0.37750
fetal lung 124	1.0371767	0.38382
fetal heart 51	1.0387648	0.38384
fetal muscle 186	1.0385162	0.38398
fetal renal pelvis 230	1.0417564	0.38508
fetal lung 130	1.0363545	0.38546
fetal lung 115	1.0348565	0.38738
fetal muscle 145	1.0404362	0.38760
fetal heart 49	1.0374887	0.38866
fetal muscle 188	1.0352205	0.38874
fetal muscle 165	1.0358393	0.39052
fetal muscle 155	1.0389448	0.39082
fetal kidney 107	1.0369819	0.39156
fetal muscle 144	1.0365098	0.39358
fetal kidney 101	1.0372511	0.39368
fetal renal cortex 204	1.0361927	0.39412
fetal lung 126	1.0312949	0.39540
fetal stomach 241	1.0386106	0.39666
fetal muscle 170	1.0340881	0.39684
fetal muscle 169	1.0348769	0.39758
fetal muscle 187	1.0330058	0.39774
CD3 11	1.0412388	0.39796
fetal large intestine 61	1.0409318	0.39854
fetal lung 139	1.0323379	0.39874
CD3 12	1.0434494	0.39894
fetal renal cortex 208	1.0341668	0.40048
fetal muscle 174	1.0340625	0.40098
fetal lung 135	1.0310051	0.40116
fetal kidney 86	1.0374514	0.40276
Mobilized CD3 321	1.0400442	0.40406
fetal muscle 190	1.0331237	0.40474
fetal brain 31	1.0228602	0.40490
fetal muscle 179	1.0308729	0.40688
fetal kidney 90	1.0309698	0.40882
fetal muscle 153	1.0308590	0.40912
fetal kidney 97	1.0303769	0.40980
fetal lung 127	1.0290101	0.40982
fetal muscle 173	1.0286657	0.41072
fetal brain 38	1.0256233	0.41094
fetal brain 34	1.0237235	0.41134
fetal muscle 156	1.0274113	0.41346
fetal stomach 252	1.0297936	0.41398
fetal muscle 162	1.0259557	0.41640
fetal stomach 242	1.0317051	0.41812
placenta 199	1.0254364	0.41866
fetal muscle 143	1.0237027	0.41960
fetal kidney 104	1.0259560	0.41996
CD4 15	1.0290831	0.42352
fetal spinal cord 237	1.0219701	0.42692
fetal muscle 184	1.0207496	0.43066
fetal stomach 251	1.0220622	0.43084
fetal testes 254	1.0205467	0.43406
fetal renal pelvis 216	1.0200268	0.43444
fetal kidney 93	1.0201534	0.43498
fetal stomach 240	1.0211562	0.43600
fetal muscle 147	1.0191033	0.43642
fetal lung 122	1.0183866	0.43770
fetal stomach 243	1.0192510	0.43844
fetal renal pelvis 222	1.0184665	0.43864
fetal muscle 181	1.0162314	0.44206
fetal kidney 94	1.0159354	0.44394
fetal heart 47	1.0162721	0.44460
fetal renal cortex 209	1.0160624	0.44476
fetal stomach 250	1.0166699	0.44662
fetal muscle 149	1.0151135	0.44844
fetal brain 32	1.0126446	0.44894
fetal muscle 158	1.0125535	0.45272
fetal lung 142	1.0119935	0.45390
fetal muscle 180	1.0129063	0.45520
fetal muscle 177	1.0120871	0.45668
fetal brain 30	1.0090888	0.45788
fetal small intestine 75	1.0063656	0.45954
fetal muscle 193	1.0099553	0.46048
fetal muscle 150	1.0103732	0.46060
fetal muscle 183	1.0095083	0.46438
fetal stomach 253	1.0070177	0.46646
fetal renal cortex 210	1.0072296	0.46702
fetal brain 29	1.0077270	0.46712
fetal renal cortex 203	1.0069043	0.47006
fetal spinal cord 236	1.0045087	0.47328
fetal heart 44	1.0044990	0.47334
fetal brain 35	1.0054741	0.47402
fetal stomach 246	1.0030651	0.47698
fetal stomach 247	1.0000787	0.48028
fetal muscle 178	1.0040166	0.48108
fetal muscle 164	1.0032491	0.48184
fetal muscle 163	1.0010208	0.48242
fetal stomach 248	1.0008979	0.48404
fetal muscle 154	0.9983042	0.49006
fetal muscle 160	0.9952925	0.50138
fetal ovary 195	0.9824890	0.51910
fetal muscle 161	0.9881961	0.52394
fetal stomach 249	0.9806356	0.52544
fetal stomach 245	0.9785727	0.53710
fetal spinal cord 235	0.9728278	0.56008
fetal testes 255	0.9720750	0.56724
fetal spinal cord 238	0.9708009	0.56962
fetal brain 37	0.9725438	0.58114
fetal spinal cord 239	0.9591898	0.60502
fetal brain 33	0.9523256	0.63462

Bone mineral density 2

DHS sample	fold enrichment	p value
fetal kidney 85	1.3007753	0.00288
fetal fibroblast 276	1.2609519	0.00324
fetal kidney 92	1.3173400	0.00402
fetal fibroblast 275	1.2397099	0.00612
fetal muscle 181	1.2898167	0.00640
fetal renal cortex 207	1.2897406	0.00854
fetal kidney 91	1.2677824	0.00886
fetal renal cortex 202	1.2758849	0.00916
fetal fibroblast 272	1.2326262	0.00938
ES derived MSC 290	1.2684085	0.01094
fetal fibroblast 282	1.2188295	0.01114
melanocyte 337	1.1982904	0.01258
fetal fibroblast 271	1.2204561	0.01294
fetal renal pelvis 221	1.2568786	0.01308
fetal fibroblast 278	1.2230297	0.01422
fetal fibroblast 268	1.2124321	0.01450
ES derived MSC 289	1.2580280	0.01516
fetal renal cortex 213	1.2427148	0.01534
fetal fibroblast 281	1.2092953	0.01584
fetal kidney 95	1.2534695	0.01710
fetal kidney 103	1.2584220	0.01850
fetal fibroblast 273	1.2016958	0.01932
fetal renal pelvis 230	1.2707238	0.01948
fetal kidney 96	1.2384416	0.01996
melanocyte 338	1.1850867	0.02064
fetal fibroblast 277	1.2076416	0.02080
fetal renal pelvis 226	1.2384683	0.02128
fetal kidney 89	1.2487118	0.02168
fetal fibroblast 274	1.1946488	0.02362
fetal muscle 179	1.2205474	0.02394
fetal renal cortex 214	1.2306372	0.02430
fetal kidney 100	1.2348697	0.02434
fetal kidney 84	1.2452337	0.02448
fetal kidney 99	1.2356972	0.02496
fetal kidney 83	1.2706682	0.02498
fetal muscle 175	1.2159705	0.02542
fetal muscle 151	1.2379080	0.02728
fetal renal pelvis 220	1.2369762	0.02734
fetal muscle 143	1.2003186	0.02736
fetal fibroblast 267	1.1951370	0.02770
fetal muscle 166	1.2131649	0.02798
fetal kidney 106	1.2229684	0.02906
fetal spinal cord 235	1.2016481	0.03062
fetal renal cortex 215	1.2100808	0.03096
fetal kidney 101	1.2155369	0.03098
fetal renal pelvis 233	1.2232833	0.03114
fetal muscle 182	1.2048293	0.03208
fetal renal cortex 205	1.2189512	0.03232
fetal renal pelvis 227	1.2230252	0.03236
fetal kidney 98	1.2239601	0.03272
fetal kidney 94	1.2325939	0.03296
fetal renal cortex 212	1.2052435	0.03338
fetal renal cortex 206	1.2072080	0.03440
fetal muscle 169	1.2077225	0.03464
fetal lung 301	1.1982071	0.03600
fetal fibroblast 269	1.1688517	0.03738
fetal muscle 160	1.2094980	0.03762
fetal muscle 183	1.1904134	0.03796
fetal muscle 149	1.2108263	0.03960
keratinocyte 336	1.1559549	0.04036
fetal muscle 185	1.2047211	0.04130
fetal kidney 97	1.2071257	0.04138
fetal muscle 187	1.1845052	0.04162
fetal kidney 105	1.1973506	0.04230
fetal muscle 171	1.1861729	0.04250
fibroblast 331	1.1744032	0.04258
fetal muscle 172	1.1705110	0.04262
fetal renal pelvis 224	1.1919394	0.04310
fetal renal pelvis 217	1.2150309	0.04406
fetal renal pelvis 232	1.2130763	0.04420
fetal renal pelvis 231	1.1894242	0.04430
fetal fibroblast 280	1.1622620	0.04570
fetal muscle 189	1.1880088	0.04580
fetal muscle 178	1.1945042	0.04708
fetal muscle 165	1.1772571	0.04724
fetal muscle 150	1.1964870	0.04746
fetal renal cortex 209	1.2004477	0.04812
fibroblast 330	1.1720511	0.04830
fetal renal pelvis 229	1.1924009	0.04922
fibroblast 332	1.1680838	0.04924
fetal kidney 87	1.1903996	0.04928
fetal renal pelvis 228	1.2114357	0.04940
fetal muscle 161	1.1868353	0.05032
fetal muscle 155	1.1959950	0.05060
fetal muscle 190	1.1945099	0.05192
fetal fibroblast 279	1.1603422	0.05200
fetal muscle 167	1.1777681	0.05208
fetal muscle 152	1.1734628	0.05456
fetal fibroblast 270	1.1577304	0.05508
fetal kidney 102	1.2023460	0.05550
fetal renal cortex 203	1.1898199	0.05588
fetal kidney 88	1.1773479	0.05746
fetal skin 234	1.1813586	0.05760
fetal kidney 107	1.1830522	0.05896
melanocyte 339	1.1460397	0.05956
keratinocyte 335	1.1414325	0.06020
fibroblast 329	1.1580298	0.06032
fetal renal pelvis 223	1.1940928	0.06100
fetal renal cortex 211	1.1948038	0.06104
fetal muscle 159	1.1840881	0.06152
fetal kidney 104	1.1867112	0.06190
fetal muscle 148	1.1755844	0.06342
fetal renal pelvis 222	1.1864023	0.06408
fetal muscle 170	1.1601062	0.06462
fetal muscle 176	1.1609077	0.06528
keratinocyte 334	1.1339902	0.06566
fetal lung 298	1.1681643	0.06606
fetal muscle 153	1.1809516	0.06746
fetal testes 255	1.1641943	0.06772
fetal muscle 186	1.1606709	0.06780
fetal renal cortex 204	1.1614379	0.06926
keratinocyte 333	1.1303140	0.07196
breast 1	1.1396960	0.07230
fetal muscle 147	1.1653400	0.07304
fetal small intestine 80	1.1752295	0.07314
fetal muscle 158	1.1550535	0.07374
fetal renal cortex 208	1.1747924	0.07426
fetal muscle 154	1.1736748	0.07786
melanocyte 340	1.1369642	0.08036
fetal muscle 163	1.1602595	0.08056
fetal stomach 244	1.1836134	0.08182
fetal muscle 180	1.1642781	0.08208
fetal renal cortex 210	1.1713415	0.08352
fetal kidney 93	1.1708354	0.08376
fetal muscle 192	1.1463354	0.08438
fetal lung 300	1.1463441	0.08544
fetal kidney 90	1.1625561	0.08570
fetal stomach 250	1.1868234	0.08778
fetal lung 116	1.1260972	0.08862
fetal renal pelvis 225	1.1631392	0.09036
fetal muscle 184	1.1376060	0.09168
fetal muscle 157	1.1329018	0.09598
fetal renal pelvis 216	1.1548967	0.09706
fetal muscle 193	1.1464984	0.09752
fetal muscle 164	1.1462789	0.09790
fetal muscle 156	1.1410525	0.09852
breast 2	1.1236639	0.09930
fetal large intestine 57	1.1467387	0.10044
fetal stomach 251	1.1645610	0.10072
fetal adrenal gland 28	1.1343331	0.10142
iPS 304	1.1345103	0.10220
fetal lung 299	1.1387185	0.10298
fetal large intestine 60	1.1581242	0.10328
fetal muscle 177	1.1453279	0.10726
fetal muscle 174	1.1397464	0.10736
fetal adrenal gland 27	1.1422551	0.10956
fetal small intestine 74	1.1518744	0.11030
fetal muscle 162	1.1289419	0.11124
fetal adrenal gland 24	1.1622547	0.11238
fetal thymus 263	1.1377863	0.11298
fetal renal pelvis 219	1.1764360	0.11348
fetal small intestine 69	1.1586735	0.11466
fetal stomach 246	1.1618097	0.11572
fetal lung 118	1.1096296	0.11596
fetal stomach 248	1.1574274	0.11810
fetal kidney 82	1.1524249	0.11836
fetal stomach 240	1.1640175	0.12158
fetal testes 254	1.1345904	0.12286
fetal muscle 144	1.1338513	0.12302
fetal spinal cord 236	1.1256204	0.12416
fetal kidney 86	1.1602728	0.12500
fetal muscle 146	1.1244585	0.12552
fetal lung 114	1.1038472	0.12936
fetal stomach 252	1.1446625	0.12956
fetal adrenal gland 26	1.1291967	0.12964
fetal muscle 168	1.1261078	0.13238
fetal stomach 243	1.1317194	0.13576
fetal small intestine 68	1.1289376	0.13622
ES cells 295	1.1426520	0.13644
fetal stomach 249	1.1482358	0.13978
fetal large intestine 64	1.1287584	0.14180
fetal muscle 173	1.1165423	0.14484
fetal muscle 191	1.1177450	0.14682
fetal large intestine 67	1.1303771	0.14778
fetal large intestine 55	1.1207130	0.15210
fetal small intestine 71	1.1268700	0.15320
fetal small intestine 78	1.1151264	0.15330
fetal lung 119	1.0937646	0.15456
ES derived trophoblast 288	1.1027940	0.15672
fetal small intestine 77	1.1244423	0.16002
fetal lung 126	1.0920613	0.16192
fetal stomach 253	1.1348640	0.16204
fetal stomach 241	1.1326906	0.16402
fetal large intestine 58	1.1108624	0.16718
fetal muscle 188	1.1007331	0.16732
fetal large intestine 66	1.1238899	0.16768
fetal muscle 145	1.1154576	0.16886
fetal adrenal gland 25	1.1178894	0.17170
fetal small intestine 73	1.1167827	0.17334
iPS 303	1.1009476	0.17364
fetal lung 140	1.0894348	0.17538
fetal small intestine 70	1.1009077	0.17572
fetal lung 136	1.0938755	0.17620
fetal muscle 194	1.0899927	0.17702
fetal large intestine 63	1.1174032	0.17744
fetal lung 123	1.0915933	0.18476
fetal lung 124	1.0890251	0.18722
fetal large intestine 59	1.1127954	0.18812
fetal renal pelvis 218	1.1217606	0.18828
fetal large intestine 65	1.1092591	0.19114
fetal large intestine 62	1.1122444	0.19214
fetal spinal cord 238	1.0874356	0.19370
fetal spinal cord 239	1.0865752	0.19374
fetal lung 131	1.0773056	0.19498
ES derived NP 292	1.1063255	0.19592
ES cells 294	1.1103503	0.19602
fetal stomach 245	1.1079084	0.19792
CD20 8	1.0981999	0.20014
fetal small intestine 72	1.1030072	0.20064
ES cells 293	1.0985907	0.20284
fetal lung 141	1.0765513	0.20594
fetal lung 113	1.0758384	0.20958
small intestine 342	1.0989997	0.21050
fetal lung 115	1.0769130	0.21086
fetal large intestine 53	1.1063279	0.21142
fetal adrenal gland 23	1.0847999	0.21224
fetal stomach 247	1.1174350	0.21378
fetal large intestine 56	1.0951599	0.21514
fetal lung 132	1.0751176	0.22310
fetal lung 129	1.0742077	0.22790
fetal lung 133	1.0689780	0.22982
fetal lung 109	1.0659653	0.23066
fetal large intestine 54	1.0897752	0.23256
fetal ovary 195	1.0970752	0.23418
iPS 302	1.0778318	0.23578
fetal lung 121	1.0667387	0.23612
ES cells 296	1.0931187	0.23622
ES derived mesoderm 286	1.0617560	0.23812
fetal lung 120	1.0687680	0.24086
fetal lung 117	1.0673906	0.24176
ES derived NP 291	1.0880069	0.24476
fetal lung 139	1.0714746	0.24690
fetal lung 128	1.0617062	0.24696
fetal small intestine 79	1.0851837	0.25054
ES derived trophoblast 287	1.0748457	0.25098
fetal heart 47	1.0820611	0.25696
fetal stomach 242	1.0953482	0.25734
fetal lung 135	1.0649134	0.25838
ES derived mesoderm 285	1.0602055	0.25930
fetal small intestine 76	1.0819122	0.26026
ovary 326	1.0857336	0.26776
fetal kidney 81	1.0615700	0.26964
pancreas 328	1.0747280	0.27534
placenta 199	1.0635654	0.28530
hematopoietic stem cell 319	1.0689628	0.28662
fetal lung 134	1.0563482	0.28788
fetal heart 45	1.0630098	0.28836
fetal lung 137	1.0513516	0.29580
fetal lung 130	1.0516494	0.29860
fetal lung 142	1.0486137	0.29886
fetal lung 108	1.0555377	0.29974
fetal lung 138	1.0477493	0.31316
fetal lung 111	1.0440109	0.31730
placenta 198	1.0514192	0.31758
fetal heart 52	1.0442648	0.32830
fetal lung 110	1.0435009	0.33120
fetal lung 112	1.0409122	0.33712
fetal spinal cord 237	1.0388841	0.34384
fetal brain 39	1.0324624	0.34582
fetal heart 49	1.0439050	0.34716
fetal heart 42	1.0428941	0.34736
stomach 283	1.0487838	0.34742
fetal heart 51	1.0379630	0.35836
hematopoietic stem cell 307	1.0389864	0.36092
fetal lung 125	1.0344416	0.36194
fetal heart 43	1.0358323	0.36220
fetal heart 48	1.0370222	0.36246
fetal small intestine 75	1.0543228	0.36348
Mobilized CD56 324	1.0402785	0.38040
heart 297	1.0305643	0.39504
placenta 197	1.0299017	0.39604
hematopoietic stem cell 308	1.0241556	0.40810
fetal lung 127	1.0210370	0.41114
placenta 200	1.0219018	0.41346
placenta 196	1.0226024	0.41530
fetal heart 50	1.0222228	0.41560
fetal brain 30	1.0136400	0.41728
fetal brain 37	1.0151079	0.42282
muscle 341	1.0196065	0.43334
CD4 13	1.0187505	0.43992
fetal lung 122	1.0138631	0.44002
CD56 18	1.0130447	0.45514
fetal heart 46	1.0091302	0.45812
fetal brain 35	1.0056075	0.46484
Mobilized CD4 322	1.0072425	0.46710
hematopoietic stem cell 305	1.0061904	0.46792
CD8 22	1.0047114	0.47278
CD4 15	1.0014990	0.47692
Mobilized CD3 321	1.0026180	0.47764
stomach 284	1.0039609	0.47802
fetal brain 29	1.0014731	0.48624
CD3 12	0.9967336	0.49114
fetal large intestine 61	0.9968624	0.49558
pancreas 327	0.9963052	0.50136
fetal brain 33	0.9967327	0.50802
CD19 5	0.9918046	0.50920
hematopoietic stem cell 311	0.9931344	0.51248
placenta 201	0.9916523	0.51624
CD8 21	0.9869015	0.51938
hematopoietic stem cell 314	0.9917894	0.52216
hematopoietic stem cell 313	0.9852988	0.54356
fetal brain 41	0.9866923	0.55326
CD4 16	0.9692318	0.55416
Mobilized CD8 325	0.9712874	0.55526
CD3 9	0.9732933	0.55578
CD19 6	0.9707963	0.55744
CD8 19	0.9682246	0.55864
fetal thymus 257	0.9716174	0.56020
CD3 11	0.9734245	0.56060
hematopoietic stem cell 317	0.9823419	0.56094
hematopoietic stem cell 306	0.9781049	0.56578
fetal brain 32	0.9822202	0.57634
fetal heart 44	0.9690433	0.57932
CD19 7	0.9617336	0.57994
hematopoietic stem cell 316	0.9715695	0.58078
fetal thymus 265	0.9590820	0.60470
Mobilized CD4 323	0.9504132	0.60954
CD3 10	0.9531803	0.61240
fetal thymus 259	0.9505623	0.61816
fetal brain 40	0.9739627	0.61992
CD56 17	0.9536455	0.62182
fetal thymus 258	0.9512024	0.62570
CD4 14	0.9508589	0.62760
hematopoietic stem cell 309	0.9596997	0.62810
hematopoietic stem cell 310	0.9632742	0.63520
fetal thymus 262	0.9332403	0.63998
fetal brain 34	0.9704544	0.64072
CD8 20	0.9427898	0.64610
fetal thymus 261	0.9385000	0.65504
hematopoietic stem cell 315	0.9481084	0.65816
fetal thymus 264	0.9395531	0.66150
hematopoietic stem cell 318	0.9457411	0.66152
fetal brain 31	0.9628858	0.69172
fetal thymus 266	0.9266557	0.69252
fetal thymus 256	0.9263522	0.69398
hematopoietic stem cell 312	0.9377411	0.70466
Mobilized CD3 320	0.9056459	0.70926
fetal thymus 260	0.9119720	0.72456
fetal brain 38	0.9444541	0.72986
fetal brain 36	0.9377095	0.75148
CD14 4	0.9014510	0.76196
CD14 3	0.8819144	0.78746

Self−reported allergy

DHS sample	fold enrichment	p value
Mobilized CD56 324	1.9838083	0.00186
Mobilized CD3 320	1.9435541	0.00618
CD3 11	1.7783409	0.00916
CD4 14	1.7372159	0.01038
CD8 20	1.7258467	0.01236
CD56 17	1.5953778	0.01924
CD3 10	1.6730962	0.02066
CD3 9	1.6991372	0.02384
CD19 5	1.6364107	0.02652
ES derived MSC 289	1.4174591	0.03724
Mobilized CD4 322	1.6589047	0.03824
Mobilized CD4 323	1.6247746	0.03938
ES derived MSC 290	1.3904349	0.04204
CD19 6	1.5895825	0.04394
CD19 7	1.6093056	0.04428
CD8 21	1.5977298	0.04648
CD3 12	1.6047261	0.04818
CD4 13	1.5813789	0.04984
CD14 4	1.4560151	0.05282
CD56 18	1.5436594	0.05434
CD8 22	1.5122967	0.06918
Mobilized CD3 321	1.5429963	0.07092
CD8 19	1.5360326	0.07850
CD14 3	1.4167548	0.08022
fetal lung 301	1.2941999	0.08748
fetal fibroblast 269	1.2463456	0.09052
fetal large intestine 61	1.4188060	0.09424
fetal lung 300	1.2708894	0.09666
hematopoietic stem cell 310	1.2636679	0.10162
CD4 16	1.4417079	0.10406
fetal fibroblast 273	1.2252568	0.10522
fetal thymus 256	1.3413355	0.11032
Mobilized CD8 325	1.4111307	0.11298
fibroblast 331	1.2441750	0.11472
fetal lung 298	1.2504819	0.12364
fetal thymus 264	1.3185193	0.12682
hematopoietic stem cell 314	1.2507410	0.13060
keratinocyte 334	1.1839438	0.14082
fetal fibroblast 270	1.1993330	0.14140
fetal thymus 258	1.3024723	0.14246
fetal fibroblast 279	1.1964179	0.14352
fetal fibroblast 277	1.1935841	0.15230
fetal fibroblast 274	1.1769336	0.16066
fibroblast 332	1.1924929	0.16496
breast 2	1.1760589	0.17042
fibroblast 330	1.1914594	0.17046
breast 1	1.1730254	0.17484
fetal fibroblast 280	1.1689382	0.17880
keratinocyte 336	1.1512800	0.18608
hematopoietic stem cell 317	1.1720888	0.19420
fetal lung 299	1.1803420	0.19940
fibroblast 329	1.1661825	0.20066
keratinocyte 335	1.1454292	0.20074
keratinocyte 333	1.1367578	0.20298
hematopoietic stem cell 312	1.1803802	0.20726
fetal thymus 260	1.2177134	0.21116
hematopoietic stem cell 308	1.1744496	0.21656
fetal thymus 265	1.2063244	0.21792
fetal thymus 266	1.2064985	0.21822
CD4 15	1.2752443	0.21876
fetal fibroblast 275	1.1312359	0.22212
stomach 284	1.1678034	0.23568
fetal fibroblast 276	1.1219374	0.24280
fetal small intestine 75	1.2076633	0.25842
small intestine 342	1.1440037	0.26096
stomach 283	1.1537171	0.26406
hematopoietic stem cell 309	1.1324207	0.27132
fetal fibroblast 282	1.1027268	0.27214
fetal fibroblast 278	1.1076083	0.28000
fetal fibroblast 271	1.0982917	0.28346
hematopoietic stem cell 318	1.1275153	0.29032
fetal fibroblast 272	1.0911165	0.29792
fetal thymus 259	1.1377630	0.29922
fetal stomach 244	1.1236155	0.30054
fetal thymus 257	1.1399555	0.30154
fetal small intestine 80	1.1088074	0.30402
hematopoietic stem cell 306	1.1062391	0.31072
muscle 341	1.1137723	0.31506
fetal fibroblast 267	1.0837523	0.31934
fetal fibroblast 281	1.0774329	0.32592
hematopoietic stem cell 316	1.0947356	0.33198
pancreas 327	1.0975744	0.33898
fetal lung 140	1.0705690	0.34598
fetal thymus 261	1.0921448	0.35220
hematopoietic stem cell 305	1.0776688	0.35586
fetal lung 111	1.0649792	0.35842
pancreas 328	1.0794229	0.35982
hematopoietic stem cell 313	1.0680609	0.36262
fetal fibroblast 268	1.0567803	0.36936
hematopoietic stem cell 311	1.0657370	0.36956
fetal small intestine 70	1.0623417	0.36996
fetal large intestine 67	1.0680175	0.37406
fetal small intestine 68	1.0648381	0.37412
hematopoietic stem cell 315	1.0564947	0.38976
fetal large intestine 57	1.0512844	0.39220
fetal stomach 252	1.0450191	0.40548
fetal stomach 251	1.0436282	0.40698
fetal stomach 246	1.0426673	0.40982
heart 297	1.0404631	0.41426
fetal large intestine 66	1.0381808	0.41618
ovary 326	1.0388158	0.41894
fetal large intestine 55	1.0342975	0.42044
fetal stomach 253	1.0343036	0.42076
fetal small intestine 77	1.0318302	0.42982
fetal lung 128	1.0275542	0.43208
fetal muscle 172	1.0248905	0.43438
CD20 8	1.0281959	0.43766
hematopoietic stem cell 307	1.0252081	0.43902
hematopoietic stem cell 319	1.0190076	0.44574
fetal stomach 243	1.0179180	0.44964
fetal large intestine 60	1.0170976	0.45258
fetal stomach 248	1.0147253	0.45456
fetal small intestine 73	1.0145051	0.45666
fetal stomach 242	1.0025922	0.45738
fetal lung 133	1.0143688	0.45826
fetal small intestine 79	1.0124744	0.45946
fetal heart 48	1.0111328	0.46294
fetal large intestine 62	1.0073275	0.46604
fetal large intestine 53	1.0009406	0.46638
fetal muscle 179	1.0081868	0.47032
fetal large intestine 56	1.0046571	0.47498
fetal heart 42	1.0033373	0.47730
fetal small intestine 69	0.9965255	0.47794
fetal small intestine 74	1.0008356	0.48070
fetal large intestine 64	0.9996619	0.48346
fetal lung 141	1.0017741	0.48450
fetal muscle 154	0.9961077	0.49110
fetal stomach 249	0.9803300	0.49356
fetal heart 46	0.9910307	0.49776
fetal heart 43	0.9947842	0.49782
melanocyte 337	0.9971553	0.50198
fetal stomach 241	0.9797043	0.50386
fetal small intestine 78	0.9892716	0.50486
fetal muscle 183	0.9893106	0.50624
fetal muscle 151	0.9854641	0.50842
fetal lung 121	0.9899654	0.51044
fetal heart 47	0.9826685	0.51050
fetal heart 52	0.9880146	0.51078
fetal lung 110	0.9847042	0.51286
fetal heart 51	0.9845898	0.51384
fetal lung 125	0.9846147	0.51390
melanocyte 338	0.9893218	0.51974
fetal stomach 247	0.9621874	0.51996
fetal heart 49	0.9781072	0.52102
fetal lung 108	0.9735637	0.53030
fetal large intestine 59	0.9651955	0.53034
fetal stomach 250	0.9600707	0.53124
fetal muscle 152	0.9768203	0.53214
fetal lung 132	0.9762798	0.53312
fetal muscle 174	0.9698619	0.53498
fetal thymus 262	0.9332831	0.54286
fetal muscle 150	0.9671358	0.54292
fetal large intestine 65	0.9615528	0.54342
fetal small intestine 71	0.9572912	0.54594
fetal muscle 148	0.9617320	0.54982
fetal stomach 245	0.9493054	0.55404
fetal large intestine 54	0.9527905	0.55478
placenta 201	0.9536752	0.55542
fetal lung 130	0.9648578	0.55624
fetal lung 129	0.9660503	0.55650
fetal muscle 153	0.9545598	0.55840
fetal kidney 91	0.9600316	0.56006
fetal muscle 189	0.9591255	0.56010
fetal lung 116	0.9684391	0.56024
fetal lung 126	0.9656955	0.56174
fetal muscle 155	0.9530328	0.56216
fetal heart 45	0.9573977	0.56250
fetal muscle 181	0.9597293	0.56436
fetal small intestine 76	0.9466477	0.56456
fetal lung 118	0.9667814	0.56494
fetal stomach 240	0.9353389	0.56678
fetal lung 134	0.9572104	0.57038
fetal lung 135	0.9566141	0.57046
fetal heart 50	0.9512800	0.57570
fetal muscle 180	0.9503149	0.57628
fetal muscle 149	0.9474729	0.57666
fetal large intestine 63	0.9352124	0.58174
fetal muscle 194	0.9525044	0.58336
fetal lung 114	0.9567165	0.58422
fetal lung 115	0.9517294	0.58688
fetal lung 120	0.9492757	0.58864
fetal muscle 146	0.9452823	0.58864
fetal small intestine 72	0.9362954	0.59042
fetal large intestine 58	0.9420884	0.59360
fetal muscle 167	0.9457653	0.59644
fetal lung 131	0.9496664	0.60076
fetal muscle 164	0.9369079	0.60332
fetal lung 127	0.9344765	0.60830
fetal lung 122	0.9311241	0.61120
fetal lung 113	0.9399086	0.61500
fetal heart 44	0.9115071	0.61906
fetal muscle 157	0.9306203	0.62586
fetal lung 123	0.9271575	0.62780
fetal renal pelvis 230	0.9016869	0.63108
fetal muscle 188	0.9226252	0.63206
fetal muscle 147	0.9183005	0.63296
fetal muscle 156	0.9200845	0.63426
fetal muscle 163	0.9160291	0.63532
melanocyte 340	0.9348363	0.63542
fetal lung 124	0.9219449	0.63560
fetal muscle 178	0.9183536	0.63794
fetal lung 119	0.9294816	0.63992
fetal muscle 190	0.9054621	0.64038
fetal renal cortex 203	0.9029977	0.64712
fetal muscle 175	0.9141481	0.64774
fetal muscle 161	0.9100238	0.64854
fetal muscle 160	0.9086171	0.64982
fetal lung 112	0.9134623	0.65190
fetal muscle 145	0.8950904	0.65206
fetal lung 138	0.9142469	0.65406
fetal lung 139	0.9114073	0.65456
fetal muscle 176	0.9186658	0.65512
fetal lung 117	0.9152770	0.65572
fetal muscle 159	0.8995393	0.65592
fetal lung 136	0.9118217	0.65804
fetal muscle 166	0.9072270	0.66550
fetal muscle 186	0.9035280	0.66672
fetal renal pelvis 222	0.8863109	0.66768
fetal muscle 177	0.8912127	0.66800
fetal lung 142	0.9133062	0.67192
fetal muscle 168	0.8961720	0.67444
fetal kidney 92	0.8965757	0.67498
fetal muscle 187	0.9018957	0.67810
fetal renal pelvis 226	0.8893999	0.68316
fetal kidney 94	0.8715350	0.68502
fetal muscle 144	0.8814412	0.68734
fetal muscle 182	0.8941290	0.68800
fetal muscle 158	0.8958266	0.68888
fetal adrenal gland 26	0.8745757	0.69224
fetal adrenal gland 28	0.8937775	0.69716
fetal muscle 169	0.8868775	0.69954
fetal muscle 170	0.8890365	0.70056
fetal renal cortex 204	0.8816542	0.70058
melanocyte 339	0.9061463	0.70274
fetal testes 254	0.8663763	0.70282
fetal lung 137	0.8929333	0.70514
fetal renal pelvis 219	0.8319297	0.70522
fetal muscle 173	0.8781718	0.70534
fetal kidney 96	0.8782076	0.70764
fetal kidney 85	0.8936701	0.70790
fetal kidney 107	0.8687047	0.70886
fetal kidney 102	0.8566501	0.70934
fetal muscle 143	0.8911111	0.70988
fetal renal pelvis 223	0.8566876	0.70996
fetal renal cortex 213	0.8819687	0.71044
ES derived trophoblast 287	0.8805604	0.71322
fetal kidney 89	0.8644517	0.71370
fetal muscle 191	0.8737479	0.71374
fetal kidney 100	0.8651090	0.71626
fetal muscle 193	0.8643565	0.72016
fetal thymus 263	0.8673399	0.72128
fetal muscle 185	0.8587485	0.72656
fetal renal pelvis 229	0.8596207	0.72722
fetal kidney 84	0.8509718	0.72742
fetal renal pelvis 217	0.8500515	0.72768
fetal kidney 90	0.8511277	0.72932
fetal muscle 184	0.8751902	0.73250
fetal kidney 104	0.8482468	0.73316
fetal renal pelvis 227	0.8515729	0.73382
fetal kidney 98	0.8481977	0.73514
fetal renal pelvis 232	0.8418081	0.73544
fetal renal cortex 210	0.8401834	0.73574
fetal renal pelvis 231	0.8613680	0.73576
fetal muscle 165	0.8743246	0.73602
fetal kidney 86	0.8129861	0.73876
fetal renal cortex 208	0.8447061	0.73884
fetal testes 255	0.8532011	0.74214
fetal muscle 192	0.8621422	0.74476
fetal kidney 101	0.8494829	0.74666
fetal muscle 162	0.8608219	0.74800
fetal renal cortex 202	0.8465635	0.75202
fetal kidney 82	0.8193174	0.75292
fetal kidney 97	0.8384119	0.75526
fetal kidney 99	0.8424863	0.75584
fetal renal cortex 207	0.8373049	0.76000
fetal renal pelvis 216	0.8329560	0.76080
fetal lung 109	0.8739538	0.76134
ES derived trophoblast 288	0.8600584	0.76162
fetal renal pelvis 228	0.8220107	0.76190
fetal renal cortex 205	0.8325461	0.76480
fetal renal cortex 209	0.8283412	0.76826
fetal kidney 106	0.8341056	0.76910
fetal kidney 105	0.8379237	0.76922
fetal renal pelvis 218	0.7810606	0.77194
fetal kidney 103	0.8254320	0.77494
fetal renal pelvis 225	0.8197940	0.77568
fetal ovary 195	0.7764805	0.77810
fetal kidney 83	0.7932472	0.77988
fetal kidney 88	0.8272355	0.78416
fetal renal pelvis 220	0.8175985	0.78600
fetal muscle 171	0.8377848	0.79206
fetal kidney 93	0.8033178	0.79418
fetal renal cortex 211	0.7980079	0.79560
fetal renal pelvis 233	0.8054541	0.80502
ES derived NP 292	0.7864482	0.80734
fetal adrenal gland 25	0.7869664	0.80828
fetal renal cortex 206	0.8101385	0.80988
placenta 197	0.7901457	0.81640
fetal kidney 87	0.8022421	0.82300
fetal kidney 81	0.8146138	0.82810
fetal renal pelvis 221	0.7936445	0.83272
fetal kidney 95	0.7829898	0.83304
fetal renal pelvis 224	0.7957909	0.83594
fetal renal cortex 214	0.7879721	0.83620
ES cells 294	0.7422804	0.84112
fetal renal cortex 215	0.7860929	0.84534
fetal renal cortex 212	0.7830273	0.85000
placenta 199	0.7752133	0.85614
placenta 196	0.7589909	0.85710
placenta 200	0.7904265	0.85844
fetal adrenal gland 23	0.7760972	0.86058
fetal adrenal gland 27	0.7540510	0.86262
ES derived mesoderm 285	0.8037098	0.86728
iPS 302	0.7546141	0.87344
ES derived mesoderm 286	0.8026712	0.88040
fetal adrenal gland 24	0.7069816	0.88250
ES derived NP 291	0.6999682	0.88842
ES cells 296	0.6909263	0.89166
iPS 303	0.7380610	0.89692
fetal spinal cord 236	0.7448264	0.89780
ES cells 295	0.6848768	0.90498
placenta 198	0.7388148	0.90660
fetal spinal cord 238	0.7481399	0.91352
fetal spinal cord 237	0.7509532	0.91622
ES cells 293	0.6430229	0.94312
fetal skin 234	0.6839300	0.94596
fetal spinal cord 235	0.6971949	0.94860
fetal brain 36	0.7305088	0.95384
fetal spinal cord 239	0.6803525	0.96230
iPS 304	0.6612791	0.96726
fetal brain 41	0.6678959	0.98800
fetal brain 39	0.6653146	0.99170
fetal brain 33	0.5972082	0.99282
fetal brain 37	0.6393540	0.99394
fetal brain 34	0.6572832	0.99440
fetal brain 35	0.6657509	0.99496
fetal brain 29	0.6796821	0.99546
fetal brain 31	0.6734209	0.99604
fetal brain 40	0.6274998	0.99644
fetal brain 30	0.6654396	0.99760
fetal brain 38	0.5676152	0.99850
fetal brain 32	0.5554801	0.99904

Blood pressure and cardiovascular risc

DHS sample	fold enrichment	p value
hematopoietic stem cell 310	1.4576630	0.00372
fetal fibroblast 273	1.3890579	0.00734
fetal fibroblast 277	1.4000068	0.00818
fetal lung 141	1.4025188	0.00826
hematopoietic stem cell 314	1.4458694	0.00860
fetal fibroblast 275	1.3661442	0.00908
fetal fibroblast 269	1.3644516	0.00958
fetal fibroblast 271	1.3725322	0.01002
fetal fibroblast 279	1.3770443	0.01028
fetal kidney 85	1.4173303	0.01068
fetal fibroblast 270	1.3733206	0.01134
fetal fibroblast 278	1.3709973	0.01250
fetal muscle 176	1.3966185	0.01304
fetal fibroblast 280	1.3407022	0.01558
fetal lung 140	1.3697103	0.01574
fetal fibroblast 276	1.3308182	0.01606
fetal lung 111	1.3674553	0.01732
fetal fibroblast 274	1.3373383	0.01778
keratinocyte 336	1.2987742	0.01988
fetal lung 298	1.3634320	0.02194
fetal muscle 181	1.3866574	0.02212
fetal fibroblast 272	1.3251689	0.02252
fetal muscle 167	1.3647304	0.02284
Mobilized CD56 324	1.4860005	0.02382
fetal muscle 149	1.3982114	0.02420
Mobilized CD4 323	1.5387670	0.02466
fetal muscle 143	1.3308134	0.02632
hematopoietic stem cell 318	1.4041338	0.02696
ES derived mesoderm 285	1.3332614	0.02712
hematopoietic stem cell 305	1.3790760	0.02822
fetal lung 119	1.3131794	0.02938
hematopoietic stem cell 308	1.3560325	0.03034
fetal fibroblast 281	1.3006190	0.03066
fetal fibroblast 268	1.2910699	0.03148
fibroblast 331	1.2995394	0.03164
fetal lung 299	1.3319825	0.03234
melanocyte 338	1.2683532	0.03248
fetal lung 133	1.3082286	0.03372
keratinocyte 335	1.2664309	0.03410
hematopoietic stem cell 316	1.3749076	0.03550
fetal muscle 151	1.3643229	0.03816
keratinocyte 333	1.2510633	0.03904
fetal thymus 258	1.4293222	0.03916
hematopoietic stem cell 309	1.3342757	0.03936
fetal muscle 169	1.3240367	0.03972
fetal fibroblast 282	1.2715528	0.04040
fetal muscle 180	1.3421610	0.04060
fetal lung 118	1.2752293	0.04098
fibroblast 332	1.2761564	0.04124
fetal lung 301	1.3004231	0.04130
fibroblast 329	1.2826412	0.04174
CD3 9	1.4446304	0.04224
CD19 7	1.4617614	0.04252
fetal lung 116	1.2764477	0.04290
CD56 18	1.4622130	0.04292
CD8 21	1.4623584	0.04298
hematopoietic stem cell 315	1.3411691	0.04304
fetal muscle 179	1.3166366	0.04320
fetal muscle 178	1.3293033	0.04366
fetal muscle 165	1.2981583	0.04558
CD19 6	1.4471514	0.04562
fibroblast 330	1.2779092	0.04632
fetal muscle 166	1.3054050	0.04750
fetal lung 117	1.2760267	0.04812
CD4 13	1.4350975	0.05074
CD8 19	1.4759635	0.05082
fetal lung 300	1.2739208	0.05116
Mobilized CD4 322	1.4278285	0.05268
fetal lung 114	1.2583362	0.05316
keratinocyte 334	1.2314462	0.05518
fetal large intestine 67	1.3216930	0.05564
fetal large intestine 61	1.3929392	0.05658
fetal fibroblast 267	1.2544548	0.05670
fetal kidney 91	1.2922684	0.05762
Mobilized CD3 321	1.4222569	0.05830
fetal muscle 153	1.3173095	0.05848
hematopoietic stem cell 312	1.2904266	0.05910
fetal muscle 152	1.2748233	0.06270
Mobilized CD3 320	1.4212754	0.06290
small intestine 342	1.3039039	0.06406
CD3 10	1.3690138	0.06432
ES derived mesoderm 286	1.2347001	0.06660
fetal thymus 264	1.3506480	0.06676
fetal muscle 154	1.2982503	0.06874
fetal kidney 92	1.2781325	0.07166
fetal renal cortex 213	1.2669002	0.07206
CD3 12	1.3872846	0.07324
fetal muscle 150	1.2817388	0.07456
CD20 8	1.2793254	0.07462
fetal thymus 265	1.3438442	0.07472
melanocyte 337	1.2026062	0.07496
fetal adrenal gland 28	1.2365852	0.07700
CD19 5	1.3546020	0.07704
Mobilized CD8 325	1.3711304	0.07800
fetal heart 47	1.2843063	0.07814
fetal lung 121	1.2232591	0.08000
CD3 11	1.3200948	0.08226
fetal thymus 256	1.3129596	0.08312
CD4 15	1.4135090	0.08482
fetal muscle 164	1.2549658	0.08552
fetal adrenal gland 26	1.2571208	0.08714
fetal renal cortex 207	1.2591874	0.08722
CD4 14	1.3135822	0.08738
hematopoietic stem cell 311	1.2545741	0.08770
fetal lung 130	1.2282977	0.09092
fetal lung 120	1.2264314	0.09372
fetal muscle 182	1.2342487	0.09388
fetal small intestine 68	1.2482047	0.09548
fetal thymus 259	1.3229424	0.09758
fetal heart 45	1.2305511	0.10180
fetal lung 128	1.1931075	0.10214
fetal thymus 263	1.2421514	0.10240
fetal muscle 175	1.2238149	0.10358
melanocyte 340	1.1964720	0.10374
fetal lung 132	1.2179304	0.10482
hematopoietic stem cell 317	1.2036543	0.10698
hematopoietic stem cell 306	1.2368946	0.10722
CD56 17	1.2704560	0.10734
hematopoietic stem cell 307	1.2339215	0.10752
fetal thymus 266	1.2881899	0.10908
fetal muscle 156	1.2153536	0.11170
fetal small intestine 74	1.2366128	0.11286
fetal muscle 171	1.2114840	0.11294
fetal muscle 159	1.2341198	0.11400
hematopoietic stem cell 319	1.2447005	0.11470
hematopoietic stem cell 313	1.2099313	0.11832
fetal lung 129	1.2008063	0.11862
fetal heart 49	1.2145856	0.12088
fetal thymus 260	1.2713448	0.12182
fetal muscle 158	1.1963251	0.12796
CD14 4	1.2487741	0.12940
fetal thymus 257	1.2764501	0.13210
melanocyte 339	1.1617967	0.13376
fetal muscle 157	1.1781011	0.14260
fetal stomach 248	1.2228846	0.14318
fetal muscle 172	1.1700563	0.14368
fetal small intestine 70	1.1860479	0.14434
CD8 22	1.2559305	0.14482
fetal lung 135	1.1818648	0.14738
fetal muscle 184	1.1730819	0.14958
breast 1	1.1566032	0.15020
fetal muscle 173	1.1871003	0.15188
fetal muscle 155	1.1945680	0.15368
fetal renal cortex 202	1.1838342	0.15580
fetal lung 126	1.1571343	0.15644
fetal large intestine 58	1.1823573	0.15668
fetal lung 110	1.1740447	0.15712
fetal muscle 148	1.1833811	0.15772
CD14 3	1.2325193	0.15814
fetal muscle 168	1.1837894	0.15838
fetal skin 234	1.1864268	0.15854
fetal muscle 194	1.1633743	0.15980
fetal muscle 147	1.1777579	0.16084
fetal stomach 244	1.2056682	0.16090
fetal muscle 186	1.1718303	0.16388
fetal muscle 183	1.1637740	0.16670
fetal adrenal gland 27	1.1833294	0.16672
fetal muscle 185	1.1838343	0.16674
fetal small intestine 78	1.1694477	0.16704
fetal lung 125	1.1629750	0.16826
fetal renal pelvis 226	1.1751843	0.16928
fetal muscle 170	1.1610352	0.16958
fetal testes 255	1.1698194	0.16992
fetal lung 136	1.1593244	0.17158
fetal kidney 95	1.1796343	0.17282
fetal muscle 187	1.1584008	0.17788
fetal lung 112	1.1565977	0.17800
CD8 20	1.2064023	0.17890
fetal renal pelvis 220	1.1749114	0.17900
fetal muscle 160	1.1685452	0.18060
fetal lung 115	1.1445918	0.18360
breast 2	1.1358322	0.18504
fetal lung 138	1.1494573	0.18526
fetal lung 124	1.1479380	0.18634
fetal muscle 161	1.1579522	0.18654
fetal lung 134	1.1495644	0.18730
fetal renal pelvis 221	1.1573494	0.18932
fetal stomach 243	1.1663181	0.18958
fetal renal pelvis 227	1.1617054	0.18992
fetal stomach 246	1.1896945	0.19010
fetal thymus 261	1.1993095	0.19294
fetal adrenal gland 24	1.1766177	0.19476
fetal muscle 189	1.1489788	0.19562
fetal large intestine 64	1.1598588	0.19670
fetal lung 137	1.1348285	0.19998
ES derived MSC 290	1.1497275	0.20134
fetal muscle 188	1.1409756	0.20262
CD4 16	1.2023586	0.20352
fetal kidney 100	1.1506992	0.20454
fetal kidney 103	1.1517756	0.20564
fetal large intestine 53	1.1749981	0.20594
ES derived MSC 289	1.1458490	0.20772
fetal kidney 106	1.1463002	0.20822
fetal stomach 250	1.1705300	0.21054
fetal large intestine 57	1.1412573	0.21176
fetal stomach 251	1.1578095	0.21288
fetal muscle 192	1.1312725	0.21732
fetal stomach 240	1.1635832	0.22218
fetal muscle 146	1.1318597	0.22366
fetal lung 123	1.1261462	0.22600
fetal lung 109	1.1090827	0.22910
fetal muscle 174	1.1326296	0.23052
fetal heart 43	1.1203931	0.23146
fetal lung 113	1.1115404	0.23396
fetal muscle 144	1.1320235	0.23448
fetal small intestine 75	1.1909178	0.23500
fetal kidney 99	1.1308682	0.23560
fetal small intestine 73	1.1371108	0.23642
fetal heart 52	1.1143340	0.23646
fetal muscle 163	1.1260147	0.23656
fetal small intestine 71	1.1375529	0.23698
fetal muscle 191	1.1286180	0.23710
fetal adrenal gland 25	1.1356451	0.23928
fetal large intestine 54	1.1363157	0.24050
fetal large intestine 63	1.1371125	0.24240
fetal muscle 162	1.1161309	0.24388
fetal lung 131	1.0996705	0.24420
fetal lung 142	1.1046654	0.24648
fetal kidney 84	1.1259646	0.24782
fetal renal pelvis 231	1.1160467	0.25010
fetal renal pelvis 228	1.1304813	0.25028
fetal muscle 177	1.1198963	0.25264
fetal lung 108	1.1136520	0.25364
fetal small intestine 69	1.1311058	0.25792
fetal kidney 83	1.1282785	0.26284
fetal large intestine 55	1.1120597	0.26292
fetal stomach 249	1.1291663	0.26316
fetal small intestine 72	1.1138729	0.26906
fetal muscle 190	1.1107453	0.26966
fetal small intestine 80	1.1100589	0.27304
ES derived NP 291	1.1248717	0.27358
fetal large intestine 60	1.1107223	0.27482
fetal muscle 193	1.1029016	0.27694
fetal renal pelvis 217	1.1052424	0.28064
fetal large intestine 66	1.1131353	0.28278
fetal kidney 94	1.1050119	0.28324
fetal adrenal gland 23	1.0941281	0.28504
heart 297	1.1082421	0.28652
fetal stomach 252	1.1082518	0.28848
fetal stomach 241	1.1117050	0.28912
fetal testes 254	1.0981030	0.29302
ES derived NP 292	1.1050871	0.29304
placenta 200	1.0866432	0.29498
ES derived trophoblast 288	1.0860816	0.29796
fetal heart 46	1.0938007	0.29810
fetal heart 48	1.0895109	0.29852
fetal kidney 89	1.0925631	0.30048
fetal renal cortex 205	1.0880716	0.30592
fetal thymus 262	1.1186807	0.30852
iPS 303	1.0812572	0.31416
fetal renal cortex 214	1.0829381	0.31428
fetal heart 51	1.0798382	0.31578
fetal small intestine 79	1.0901912	0.31638
fetal stomach 253	1.0925755	0.32030
fetal brain 36	1.0648527	0.32438
fetal stomach 247	1.0974935	0.32616
fetal kidney 102	1.0787750	0.33094
ES cells 295	1.0821521	0.33302
fetal lung 127	1.0684498	0.33520
fetal kidney 81	1.0668871	0.33628
fetal stomach 245	1.0775380	0.33804
fetal kidney 97	1.0685099	0.33954
fetal renal pelvis 230	1.0704717	0.34742
fetal large intestine 62	1.0731016	0.34746
fetal small intestine 77	1.0713125	0.34926
iPS 302	1.0621232	0.35150
fetal lung 139	1.0580870	0.35288
pancreas 328	1.0639712	0.36194
placenta 197	1.0624641	0.36296
iPS 304	1.0529130	0.36694
ES cells 296	1.0625620	0.36742
fetal ovary 195	1.0634415	0.36802
fetal spinal cord 236	1.0534774	0.36830
fetal muscle 145	1.0544022	0.37130
fetal large intestine 59	1.0596223	0.37148
fetal renal pelvis 232	1.0518647	0.37794
placenta 199	1.0501448	0.37930
fetal renal cortex 203	1.0473799	0.38214
placenta 198	1.0447907	0.38494
fetal spinal cord 235	1.0439364	0.38574
fetal kidney 87	1.0441289	0.38632
fetal small intestine 76	1.0467279	0.39342
fetal renal pelvis 233	1.0419179	0.39504
fetal kidney 105	1.0343119	0.40350
fetal renal cortex 206	1.0338541	0.40646
ES cells 293	1.0337679	0.41272
fetal lung 122	1.0251154	0.42314
fetal large intestine 65	1.0266079	0.42710
fetal brain 32	1.0223196	0.42712
stomach 283	1.0296844	0.42816
fetal renal pelvis 224	1.0218985	0.42968
fetal brain 35	1.0180670	0.43050
fetal heart 44	1.0271022	0.43070
fetal renal pelvis 222	1.0193569	0.43722
fetal renal pelvis 219	1.0213873	0.43874
fetal large intestine 56	1.0211739	0.43938
fetal brain 37	1.0167620	0.44002
fetal kidney 101	1.0175638	0.44170
fetal renal cortex 208	1.0142146	0.44898
fetal renal cortex 211	1.0126089	0.45126
fetal brain 31	1.0082455	0.46320
fetal renal pelvis 216	1.0064927	0.46390
muscle 341	1.0079214	0.46440
placenta 201	1.0075130	0.46860
fetal kidney 98	1.0030369	0.47044
fetal renal cortex 212	1.0020650	0.47394
fetal heart 42	1.0060298	0.47412
ovary 326	0.9999854	0.47662
fetal renal cortex 210	0.9987640	0.47662
fetal kidney 104	0.9984372	0.47930
fetal renal pelvis 225	0.9979284	0.48096
fetal renal cortex 215	0.9956725	0.48980
fetal kidney 107	0.9947982	0.49030
fetal renal pelvis 229	0.9928769	0.49486
fetal stomach 242	0.9877641	0.49740
fetal kidney 88	0.9867364	0.50870
fetal kidney 82	0.9799921	0.51328
fetal heart 50	0.9876540	0.51452
fetal brain 33	0.9857476	0.51872
pancreas 327	0.9783141	0.52038
stomach 284	0.9804675	0.52242
ES derived trophoblast 287	0.9821038	0.52786
fetal renal pelvis 223	0.9732278	0.53008
fetal kidney 96	0.9759233	0.53180
fetal spinal cord 238	0.9750424	0.53942
placenta 196	0.9701423	0.54938
fetal renal cortex 204	0.9699394	0.54974
fetal spinal cord 237	0.9723906	0.55038
fetal renal cortex 209	0.9626441	0.55570
ES cells 294	0.9529107	0.56318
fetal brain 39	0.9689091	0.57146
fetal kidney 90	0.9528235	0.57798
fetal brain 34	0.9643466	0.58624
fetal renal pelvis 218	0.9276123	0.59766
fetal kidney 86	0.9114773	0.63104
fetal kidney 93	0.9225281	0.63264
fetal brain 40	0.9482399	0.63688
fetal brain 29	0.9475891	0.65738
fetal spinal cord 239	0.9206797	0.66358
fetal brain 41	0.9338119	0.66418
fetal brain 30	0.9361150	0.69896
fetal brain 38	0.9134422	0.70022

Rheumatoid arthritis

DHS sample	fold enrichment	p value
Mobilized CD56 324	1.8248256	0.00390
Mobilized CD3 320	1.7769947	0.01070
CD4 14	1.6256096	0.01338
CD14 4	1.5485436	0.01774
CD3 10	1.5974423	0.01914
CD4 13	1.6692476	0.02056
fetal thymus 256	1.5224455	0.02182
fetal brain 34	1.3357954	0.02214
CD19 6	1.5968838	0.02746
CD19 7	1.6167692	0.02778
CD19 5	1.5552429	0.02816
fetal muscle 180	1.4050732	0.03510
Mobilized CD4 322	1.5852533	0.03692
fetal muscle 143	1.3539238	0.03808
Mobilized CD4 323	1.5355658	0.04172
CD3 11	1.4796427	0.04186
CD14 3	1.4619754	0.04430
fetal thymus 264	1.4265301	0.05242
fetal muscle 169	1.3369803	0.05366
fetal muscle 149	1.3615284	0.05698
fetal muscle 175	1.3180900	0.06128
fetal muscle 164	1.3358094	0.06198
fetal brain 35	1.2391562	0.06226
fetal muscle 153	1.3512609	0.06248
fetal muscle 181	1.3122377	0.06432
fetal muscle 150	1.3321920	0.06624
fetal thymus 258	1.4024537	0.06678
fetal muscle 165	1.2908866	0.06994
CD8 20	1.4014408	0.07000
fetal muscle 152	1.2999009	0.07074
CD4 16	1.4726110	0.07138
CD3 12	1.4606960	0.07256
fetal muscle 148	1.3124131	0.07510
fetal muscle 192	1.2914900	0.07526
fetal muscle 176	1.2824277	0.07534
fetal muscle 168	1.3187612	0.07610
fetal muscle 167	1.2911957	0.07710
fetal muscle 159	1.3173789	0.07802
hematopoietic stem cell 310	1.2672003	0.08000
fetal fibroblast 273	1.2443874	0.08062
fetal fibroblast 267	1.2523875	0.08264
fetal thymus 259	1.3820159	0.08442
fetal muscle 155	1.3074856	0.08668
fetal muscle 157	1.2676582	0.08712
CD3 9	1.3952716	0.08920
hematopoietic stem cell 317	1.2544171	0.09000
fetal muscle 187	1.2657451	0.09182
fetal muscle 156	1.2680197	0.09292
fetal fibroblast 282	1.2217396	0.09374
fetal thymus 266	1.3373427	0.09506
fetal muscle 154	1.2982832	0.09704
fetal muscle 172	1.2414153	0.09728
fetal muscle 162	1.2578820	0.09748
fetal muscle 178	1.2763693	0.09830
fetal muscle 183	1.2503835	0.10088
fetal muscle 173	1.2628875	0.10210
fetal fibroblast 276	1.2135002	0.10266
muscle 341	1.3168124	0.10464
fetal muscle 188	1.2530012	0.10464
fetal muscle 147	1.2641288	0.10476
fetal muscle 182	1.2537483	0.10608
fetal muscle 151	1.2789081	0.10672
fetal fibroblast 281	1.2181958	0.10758
CD56 17	1.3058731	0.11128
fetal muscle 166	1.2451489	0.11136
Mobilized CD3 321	1.3867454	0.11270
fetal fibroblast 271	1.2102514	0.11288
hematopoietic stem cell 314	1.2393995	0.11312
fetal fibroblast 268	1.2046278	0.11320
fetal lung 299	1.2432253	0.11390
fetal muscle 158	1.2381744	0.11608
fetal muscle 193	1.2524476	0.11688
fetal muscle 177	1.2629463	0.11770
fetal thymus 265	1.3052154	0.11796
fetal muscle 163	1.2493003	0.11954
fetal brain 30	1.1662134	0.11964
fetal small intestine 70	1.2402358	0.12060
ES derived MSC 290	1.2369510	0.12088
fetal muscle 191	1.2441496	0.12302
fetal brain 41	1.1921085	0.12502
fetal large intestine 57	1.2430000	0.12594
fibroblast 332	1.2035949	0.12612
fetal muscle 145	1.2672085	0.12698
fetal muscle 185	1.2454817	0.12702
fetal brain 29	1.1622336	0.12730
fibroblast 331	1.2029013	0.12774
fetal muscle 171	1.2226979	0.12778
CD56 18	1.3366826	0.13002
CD8 21	1.3421700	0.13044
fetal muscle 184	1.2137869	0.13044
fetal muscle 189	1.2291127	0.13122
fetal fibroblast 277	1.2032440	0.13152
fetal lung 298	1.2202458	0.13188
fetal muscle 146	1.2255772	0.13266
fetal muscle 179	1.2221187	0.13616
fetal muscle 160	1.2352532	0.13874
hematopoietic stem cell 313	1.2218018	0.13902
fetal fibroblast 272	1.1879689	0.13934
hematopoietic stem cell 309	1.2309557	0.13942
hematopoietic stem cell 312	1.2215776	0.13978
fetal lung 300	1.2037732	0.13978
ES derived MSC 289	1.2177448	0.14100
fetal brain 36	1.1780680	0.14256
fetal brain 39	1.1694775	0.14436
fetal thymus 260	1.2703169	0.14480
fetal muscle 190	1.2304106	0.14540
fetal fibroblast 274	1.1847877	0.14606
fibroblast 330	1.1936201	0.14610
fetal kidney 81	1.2039761	0.14654
fetal muscle 161	1.2116057	0.15064
hematopoietic stem cell 306	1.2163176	0.15100
fetal muscle 186	1.2023451	0.15288
fetal lung 133	1.1770828	0.15718
fetal muscle 170	1.1947662	0.15730
fetal lung 301	1.1938984	0.16012
fetal muscle 174	1.2049437	0.16218
CD8 22	1.2780587	0.16502
fetal testes 255	1.1973023	0.16904
fetal thymus 263	1.1986586	0.16960
fetal lung 140	1.1728819	0.17030
fetal lung 117	1.1750772	0.17056
fetal lung 131	1.1626268	0.17070
fetal large intestine 61	1.2551210	0.17274
fetal fibroblast 279	1.1638066	0.17496
hematopoietic stem cell 308	1.1871898	0.17506
fetal fibroblast 275	1.1549345	0.17518
fetal muscle 194	1.1684839	0.17572
hematopoietic stem cell 305	1.1969298	0.17686
fetal fibroblast 280	1.1582225	0.17748
fetal small intestine 78	1.1855012	0.18008
fibroblast 329	1.1655973	0.18016
hematopoietic stem cell 311	1.1889544	0.18026
ES derived NP 291	1.2140531	0.18282
fetal lung 119	1.1583331	0.18324
fetal fibroblast 278	1.1582284	0.18856
fetal large intestine 64	1.1948820	0.18860
fetal brain 31	1.1180744	0.19894
CD8 19	1.2521798	0.19898
fetal testes 254	1.1810742	0.20172
fetal small intestine 68	1.1747344	0.20756
fetal large intestine 58	1.1656008	0.21070
fetal brain 37	1.1308808	0.21090
fetal brain 38	1.1360226	0.21248
fetal thymus 261	1.2002978	0.21674
fetal thymus 257	1.2033085	0.21718
fetal muscle 144	1.1620518	0.21856
fetal fibroblast 269	1.1287298	0.22034
fetal lung 118	1.1291548	0.22118
fetal spinal cord 237	1.1378698	0.22256
fetal small intestine 80	1.1644810	0.22262
fetal lung 120	1.1393438	0.22474
fetal large intestine 55	1.1618970	0.22486
fetal kidney 92	1.1431216	0.23162
hematopoietic stem cell 315	1.1539156	0.23230
fetal large intestine 56	1.1604125	0.23384
hematopoietic stem cell 318	1.1552991	0.23486
hematopoietic stem cell 316	1.1575095	0.23520
hematopoietic stem cell 307	1.1472494	0.23530
placenta 200	1.1354781	0.23600
ES derived NP 292	1.1601924	0.23832
CD4 15	1.2180988	0.24384
fetal small intestine 73	1.1488436	0.25102
CD20 8	1.1399902	0.25544
fetal lung 116	1.1091419	0.25684
fetal lung 111	1.1141616	0.26142
ES derived mesoderm 285	1.1121474	0.26170
fetal lung 115	1.1135767	0.26238
small intestine 342	1.1374443	0.26252
fetal lung 121	1.1070219	0.26740
fetal small intestine 75	1.1660625	0.26936
fetal large intestine 54	1.1321898	0.27374
fetal small intestine 72	1.1307598	0.27406
fetal small intestine 76	1.1377339	0.27586
fetal lung 114	1.0943838	0.28400
fetal large intestine 60	1.1196860	0.28772
fetal large intestine 65	1.1207420	0.29136
fetal lung 141	1.0910416	0.29238
fetal lung 108	1.1042154	0.29308
fetal kidney 91	1.0999553	0.29360
fetal renal pelvis 221	1.1020934	0.29762
fetal brain 33	1.0922645	0.29818
hematopoietic stem cell 319	1.1102837	0.30166
fetal brain 40	1.0761514	0.30178
fetal lung 136	1.0919716	0.30222
fetal lung 123	1.0917034	0.30562
fetal lung 112	1.0910223	0.30636
iPS 302	1.0929491	0.30750
fetal small intestine 74	1.1044640	0.30832
fetal fibroblast 270	1.0836484	0.30888
fetal spinal cord 235	1.0890998	0.31028
fetal adrenal gland 23	1.0951719	0.31138
fetal lung 124	1.0853411	0.31330
fetal lung 142	1.0787985	0.31778
fetal brain 32	1.0741037	0.31940
fetal kidney 96	1.0873312	0.32142
melanocyte 337	1.0676703	0.32230
melanocyte 338	1.0662719	0.32350
ES cells 294	1.0989548	0.32360
ES derived trophoblast 287	1.0949945	0.32408
placenta 201	1.0932081	0.32496
fetal lung 130	1.0780899	0.32638
fetal ovary 195	1.0981994	0.32742
fetal small intestine 69	1.0988586	0.32744
stomach 284	1.0986576	0.32954
fetal small intestine 77	1.0952120	0.33000
stomach 283	1.0975165	0.33228
fetal large intestine 66	1.0923396	0.33400
fetal lung 128	1.0670769	0.33570
fetal thymus 262	1.1077876	0.33636
fetal lung 129	1.0733332	0.33686
ovary 326	1.0993687	0.33886
fetal renal cortex 213	1.0750926	0.34034
fetal renal cortex 202	1.0746955	0.34064
fetal large intestine 59	1.0890817	0.34112
fetal lung 132	1.0685812	0.34480
iPS 303	1.0715355	0.34492
fetal lung 126	1.0651112	0.34498
placenta 197	1.0763083	0.34502
fetal lung 138	1.0675962	0.34714
fetal adrenal gland 28	1.0691516	0.34926
fetal kidney 100	1.0719513	0.35112
fetal lung 113	1.0585815	0.35836
placenta 196	1.0672315	0.35928
placenta 199	1.0659979	0.35974
fetal kidney 107	1.0640961	0.36284
fetal lung 125	1.0580261	0.36678
fetal renal cortex 210	1.0656006	0.36700
fetal kidney 84	1.0611319	0.36904
fetal adrenal gland 27	1.0648846	0.37002
fetal small intestine 79	1.0698610	0.37078
ES derived mesoderm 286	1.0492668	0.37208
pancreas 328	1.0655809	0.37430
fetal adrenal gland 26	1.0609785	0.37502
ES derived trophoblast 288	1.0559112	0.37628
melanocyte 340	1.0482156	0.37836
fetal kidney 94	1.0564085	0.38088
fetal lung 135	1.0501289	0.38222
fetal stomach 245	1.0607450	0.38224
fetal renal cortex 204	1.0517151	0.38296
fetal large intestine 67	1.0589296	0.38350
fetal spinal cord 239	1.0505389	0.38374
fetal renal cortex 209	1.0518875	0.38818
fetal renal pelvis 219	1.0553366	0.38836
fetal renal pelvis 226	1.0479184	0.38996
fetal large intestine 63	1.0546274	0.39254
fetal renal cortex 208	1.0472585	0.39588
fetal spinal cord 236	1.0450345	0.39698
fetal renal cortex 205	1.0458260	0.39702
placenta 198	1.0446422	0.39936
ES cells 295	1.0437926	0.40502
iPS 304	1.0364534	0.40886
fetal spinal cord 238	1.0369689	0.41002
fetal kidney 83	1.0350783	0.41424
fetal lung 110	1.0335387	0.41504
fetal skin 234	1.0386580	0.41544
fetal small intestine 71	1.0377999	0.41632
fetal kidney 85	1.0309817	0.41752
fetal renal pelvis 230	1.0321983	0.41872
fetal kidney 88	1.0313385	0.42174
fetal renal pelvis 223	1.0309313	0.42316
fetal stomach 243	1.0302710	0.42484
ES cells 296	1.0305663	0.42490
fetal lung 127	1.0298649	0.42516
fetal renal cortex 214	1.0301105	0.42562
fetal kidney 102	1.0294583	0.42646
fetal kidney 98	1.0250810	0.42978
fetal renal pelvis 217	1.0274252	0.43006
fetal lung 134	1.0260921	0.43114
fetal kidney 106	1.0227396	0.43290
fetal lung 137	1.0246705	0.43382
fetal kidney 104	1.0215737	0.43890
fetal kidney 90	1.0212158	0.43962
fetal kidney 87	1.0210761	0.43978
heart 297	1.0231698	0.44208
fetal renal cortex 211	1.0169342	0.44400
fetal renal pelvis 229	1.0180230	0.44454
fetal large intestine 62	1.0214326	0.44552
fetal renal cortex 206	1.0196010	0.44652
fetal renal cortex 212	1.0140149	0.45112
fetal renal pelvis 224	1.0139094	0.45370
fetal renal pelvis 227	1.0110374	0.45522
ES cells 293	1.0122706	0.45824
fetal renal pelvis 233	1.0101074	0.45840
fetal stomach 246	1.0079131	0.45968
fetal stomach 240	1.0054176	0.46164
fetal lung 122	1.0092041	0.46408
fetal stomach 242	1.0024421	0.46442
fetal kidney 101	1.0063413	0.46736
fetal kidney 89	1.0048428	0.46954
fetal renal pelvis 225	1.0021508	0.47208
fetal stomach 249	0.9971451	0.47330
fetal kidney 105	1.0029348	0.47414
breast 1	1.0052893	0.47880
pancreas 327	0.9986357	0.47956
fetal kidney 93	0.9970246	0.48146
fetal renal cortex 215	0.9987498	0.48236
fetal renal cortex 207	0.9999932	0.48246
fetal renal pelvis 232	0.9972930	0.48288
fetal stomach 251	0.9957029	0.48376
fetal stomach 253	0.9935098	0.48450
fetal renal cortex 203	0.9922319	0.48844
fetal large intestine 53	0.9911967	0.48894
fetal renal pelvis 222	0.9927064	0.48950
keratinocyte 334	0.9982556	0.49642
fetal kidney 82	0.9854916	0.49712
Mobilized CD8 325	0.9774542	0.49870
fetal kidney 99	0.9902149	0.49950
melanocyte 339	0.9962147	0.49954
fetal adrenal gland 24	0.9834269	0.50244
fetal kidney 97	0.9865090	0.50492
fetal renal pelvis 216	0.9822863	0.50684
keratinocyte 335	0.9908208	0.51624
breast 2	0.9877819	0.51968
fetal renal pelvis 228	0.9711040	0.52182
fetal kidney 103	0.9770400	0.52262
fetal lung 109	0.9839407	0.52476
fetal stomach 241	0.9677862	0.52756
fetal renal pelvis 218	0.9572932	0.53018
fetal lung 139	0.9746180	0.53316
fetal renal pelvis 231	0.9731073	0.53352
fetal kidney 95	0.9682976	0.53566
fetal renal pelvis 220	0.9642665	0.54474
fetal adrenal gland 25	0.9621382	0.54664
fetal kidney 86	0.9474397	0.54680
keratinocyte 336	0.9792092	0.54758
fetal stomach 248	0.9543984	0.54940
fetal stomach 250	0.9526229	0.54966
keratinocyte 333	0.9733147	0.56112
fetal stomach 252	0.9476722	0.56240
fetal stomach 244	0.9431272	0.57404
fetal stomach 247	0.9048318	0.60788
fetal heart 52	0.9353835	0.61992
fetal heart 42	0.9103089	0.65620
fetal heart 51	0.9043829	0.66856
fetal heart 48	0.8810899	0.70710
fetal heart 50	0.8776204	0.71066
fetal heart 43	0.8611952	0.75252
fetal heart 49	0.8443984	0.76794
fetal heart 46	0.8141770	0.80736
fetal heart 47	0.8027020	0.80824
fetal heart 44	0.7722392	0.83186
fetal heart 45	0.7756338	0.86398

Type 2 diabetes

DHS sample	fold enrichment	p value
hematopoietic stem cell 310	1.4406137	0.00536
hematopoietic stem cell 314	1.4668601	0.00632
melanocyte 338	1.3534553	0.00652
melanocyte 339	1.3428857	0.00864
melanocyte 337	1.3189620	0.00976
hematopoietic stem cell 313	1.4005555	0.01258
hematopoietic stem cell 309	1.4307119	0.01284
fibroblast 330	1.3746731	0.01376
hematopoietic stem cell 311	1.4097776	0.01388
melanocyte 340	1.3226492	0.01498
hematopoietic stem cell 317	1.3534559	0.01700
fibroblast 329	1.3509564	0.01850
hematopoietic stem cell 308	1.3701011	0.02254
keratinocyte 333	1.2735051	0.02426
hematopoietic stem cell 306	1.3681458	0.02498
hematopoietic stem cell 318	1.3977449	0.02502
hematopoietic stem cell 312	1.3513413	0.02738
hematopoietic stem cell 315	1.3742476	0.02870
hematopoietic stem cell 316	1.3890943	0.02892
fetal adrenal gland 28	1.3286450	0.02960
keratinocyte 335	1.2680832	0.02994
fetal adrenal gland 27	1.3667768	0.03096
CD14 4	1.4566096	0.03118
hematopoietic stem cell 307	1.3522242	0.03196
CD14 3	1.4715985	0.03296
fetal adrenal gland 23	1.3168234	0.03866
hematopoietic stem cell 319	1.3548350	0.03872
keratinocyte 334	1.2445156	0.03926
fibroblast 332	1.2921954	0.04000
keratinocyte 336	1.2420388	0.04146
fetal large intestine 65	1.3574289	0.04336
fetal large intestine 63	1.3694061	0.04488
fibroblast 331	1.2747741	0.04732
breast 2	1.2518040	0.04912
fetal fibroblast 276	1.2432796	0.05064
fetal large intestine 66	1.3443829	0.05116
fetal fibroblast 275	1.2380615	0.05588
fetal small intestine 69	1.3514610	0.05678
CD3 10	1.3941251	0.05816
hematopoietic stem cell 305	1.2968655	0.05866
fetal adrenal gland 25	1.3218575	0.05874
fetal lung 300	1.2689857	0.05980
fetal small intestine 71	1.3202459	0.06158
fetal fibroblast 272	1.2316725	0.06214
fetal lung 301	1.2669489	0.06558
fetal small intestine 74	1.3027088	0.06732
fetal small intestine 76	1.3194126	0.06822
fetal large intestine 60	1.3023764	0.07104
fetal large intestine 62	1.3180712	0.07162
fetal small intestine 72	1.2958180	0.07198
fetal lung 298	1.2635829	0.07350
fetal lung 299	1.2581617	0.07692
fetal large intestine 67	1.3034316	0.07910
fetal small intestine 73	1.2897256	0.07910
fetal large intestine 54	1.2926730	0.08014
fetal small intestine 78	1.2576759	0.08228
fetal large intestine 58	1.2615750	0.08322
breast 1	1.2101801	0.08344
fetal fibroblast 278	1.2191586	0.08552
fetal large intestine 53	1.3046388	0.08562
CD19 6	1.3617428	0.08822
fetal fibroblast 271	1.2040402	0.09082
fetal fibroblast 277	1.2081291	0.09568
CD3 12	1.3487624	0.09598
Mobilized CD4 323	1.3393705	0.09624
fetal large intestine 56	1.2549948	0.10024
fetal thymus 256	1.2914264	0.10028
fetal brain 36	1.1769994	0.10082
CD19 7	1.3426573	0.10096
fetal renal cortex 203	1.2367630	0.10182
fetal small intestine 77	1.2593555	0.10220
fetal spinal cord 237	1.1979145	0.10308
CD56 18	1.3279058	0.10388
fetal fibroblast 280	1.1968589	0.10636
ES derived NP 292	1.2424370	0.10678
fetal fibroblast 273	1.1881328	0.10802
fetal small intestine 79	1.2601304	0.10868
ES derived MSC 290	1.2220382	0.10896
fetal kidney 97	1.2289892	0.10914
fetal large intestine 59	1.2569536	0.11036
fetal fibroblast 268	1.1827068	0.11158
fetal stomach 242	1.2979920	0.11178
fetal thymus 258	1.2798083	0.11236
fetal kidney 105	1.2150777	0.11242
fetal fibroblast 282	1.1779430	0.11378
CD19 5	1.3016105	0.11582
fetal small intestine 70	1.2133745	0.11868
CD20 8	1.2213591	0.11972
fetal kidney 102	1.2246967	0.12164
fetal adrenal gland 24	1.2452669	0.12342
fetal thymus 261	1.2706372	0.12532
fetal fibroblast 274	1.1762126	0.12576
fetal kidney 86	1.2511843	0.12592
CD4 16	1.2968860	0.12614
fetal lung 132	1.1793792	0.12812
CD56 17	1.2489500	0.13052
fetal renal pelvis 218	1.2509648	0.13098
fetal small intestine 80	1.2218588	0.13246
fetal adrenal gland 26	1.2004962	0.13358
fetal brain 37	1.1479994	0.13358
Mobilized CD3 321	1.2924448	0.13666
fetal kidney 82	1.2175360	0.13716
fetal testes 255	1.1906830	0.13746
ES derived mesoderm 286	1.1463322	0.13880
fetal fibroblast 281	1.1633836	0.14000
fetal kidney 107	1.1932353	0.14112
fetal thymus 259	1.2561466	0.14134
fetal kidney 94	1.2060178	0.14194
fetal fibroblast 279	1.1684633	0.14258
fetal renal cortex 204	1.1833449	0.14326
fetal small intestine 68	1.2040381	0.14406
fetal renal pelvis 219	1.2326483	0.14526
fetal kidney 96	1.1822283	0.14598
fetal renal cortex 208	1.1993445	0.14682
fetal thymus 260	1.2366518	0.14728
Mobilized CD8 325	1.2551999	0.14792
fetal large intestine 55	1.1957027	0.14870
fetal spinal cord 238	1.1582780	0.14976
fetal kidney 104	1.1889752	0.15014
fetal fibroblast 267	1.1608787	0.15098
fetal kidney 101	1.1849541	0.15132
fetal lung 109	1.1453996	0.15162
CD4 14	1.2359964	0.15352
ES derived NP 291	1.1992456	0.15392
fetal brain 31	1.1139682	0.15428
fetal renal cortex 206	1.1794879	0.15484
fetal lung 120	1.1603997	0.15570
fetal stomach 250	1.2099693	0.15610
CD4 15	1.2864815	0.15756
CD8 22	1.2460556	0.15818
fetal renal pelvis 230	1.1925920	0.15996
fetal skin 234	1.1795658	0.16048
fetal brain 39	1.1244893	0.16080
fetal lung 136	1.1549185	0.16246
fetal renal pelvis 217	1.1827285	0.16272
fetal brain 34	1.1181784	0.16562
fetal renal pelvis 224	1.1624339	0.16772
CD8 19	1.2533298	0.16780
fetal renal pelvis 222	1.1796005	0.16840
fetal stomach 247	1.2181456	0.16874
fetal renal pelvis 225	1.1767331	0.17070
fetal fibroblast 269	1.1449543	0.17096
fetal kidney 90	1.1757472	0.17110
fetal ovary 195	1.1967072	0.17122
fetal large intestine 64	1.1821430	0.17132
fetal kidney 93	1.1838751	0.17140
fetal renal pelvis 216	1.1758650	0.17188
fetal lung 122	1.1551306	0.17192
fetal stomach 240	1.1986306	0.17434
fetal thymus 266	1.2078013	0.17478
fetal kidney 98	1.1706546	0.17516
fetal spinal cord 239	1.1397885	0.17638
fetal stomach 251	1.1793244	0.17680
CD3 11	1.2153655	0.17766
fetal thymus 257	1.2144156	0.17890
CD8 21	1.2330305	0.17914
fetal renal cortex 212	1.1567967	0.17936
ES derived MSC 289	1.1628760	0.18032
fetal stomach 249	1.1863885	0.18148
fetal renal pelvis 227	1.1633691	0.18174
fetal kidney 83	1.1752662	0.18688
fetal stomach 246	1.1781338	0.18850
fetal brain 35	1.1042748	0.18860
fetal spinal cord 236	1.1392625	0.18904
fetal stomach 245	1.1729021	0.18914
fetal kidney 85	1.1400898	0.19078
fetal large intestine 57	1.1600504	0.19084
CD4 13	1.2182244	0.19216
fetal renal pelvis 231	1.1471714	0.19280
fetal kidney 92	1.1456603	0.19626
fetal stomach 253	1.1740273	0.19666
fetal testes 254	1.1562187	0.19698
fetal fibroblast 270	1.1326618	0.19724
stomach 284	1.1768693	0.19846
fetal renal cortex 210	1.1565385	0.20036
CD3 9	1.2019259	0.20388
fetal kidney 88	1.1414826	0.20414
fetal kidney 91	1.1381543	0.20448
fetal thymus 262	1.2111666	0.20466
fetal lung 112	1.1262443	0.20680
iPS 302	1.1315008	0.20874
fetal renal pelvis 228	1.1469121	0.21400
fetal renal cortex 209	1.1413088	0.21496
fetal stomach 244	1.1541805	0.21520
fetal kidney 95	1.1372629	0.21662
fetal heart 42	1.1345905	0.21790
fetal lung 140	1.1180697	0.22028
fetal renal pelvis 226	1.1348528	0.22056
fetal thymus 265	1.1704453	0.22074
fetal spinal cord 235	1.1166825	0.22252
fetal brain 41	1.0969376	0.22296
fetal stomach 252	1.1413632	0.22308
fetal lung 127	1.1178617	0.22522
fetal lung 125	1.1183430	0.22546
fetal kidney 89	1.1315082	0.22838
fetal kidney 84	1.1325085	0.22926
fetal lung 135	1.1140100	0.22996
fetal lung 123	1.1111036	0.23010
fetal lung 111	1.1108226	0.23066
fetal heart 51	1.1236873	0.23446
fetal lung 110	1.1129231	0.23566
fetal heart 50	1.1250811	0.23572
fetal renal pelvis 221	1.1237601	0.23580
placenta 201	1.1384885	0.23862
fetal renal cortex 214	1.1211119	0.24054
fetal renal pelvis 223	1.1298078	0.24056
fetal stomach 241	1.1354811	0.24138
fetal large intestine 61	1.1545915	0.24254
fetal stomach 248	1.1317619	0.24296
fetal renal pelvis 220	1.1238670	0.24338
fetal small intestine 75	1.1540395	0.24380
ES cells 295	1.1263474	0.24390
fetal renal cortex 202	1.1152598	0.24438
fetal kidney 100	1.1188403	0.24442
fetal lung 115	1.0993510	0.24442
fetal lung 128	1.0956414	0.24530
fetal kidney 106	1.1151849	0.24594
fetal lung 138	1.1031562	0.24882
fetal lung 130	1.1014058	0.24926
fetal renal cortex 207	1.1143322	0.25288
fetal lung 124	1.0987643	0.25366
Mobilized CD4 322	1.1579986	0.25374
pancreas 327	1.1338762	0.25510
fetal renal cortex 215	1.1089659	0.25556
small intestine 342	1.1257092	0.25830
fetal lung 142	1.0919733	0.25830
placenta 197	1.1164840	0.26310
fetal kidney 103	1.1085729	0.26310
ES derived trophoblast 288	1.1028355	0.26492
fetal renal cortex 211	1.1089778	0.26626
fetal lung 119	1.0893758	0.26976
fetal heart 43	1.0994747	0.27004
fetal lung 139	1.0939414	0.27020
iPS 304	1.0883737	0.27052
ES derived mesoderm 285	1.0853049	0.27068
fetal muscle 172	1.0892481	0.27172
CD8 20	1.1317094	0.27656
fetal lung 134	1.0912756	0.27878
fetal stomach 243	1.1037670	0.27960
fetal heart 52	1.0912922	0.28010
fetal renal pelvis 229	1.0992879	0.28020
fetal kidney 87	1.0972633	0.28048
fetal kidney 99	1.0968458	0.28130
ES cells 293	1.0976081	0.28174
fetal thymus 264	1.1181679	0.28284
fetal renal cortex 213	1.0937528	0.28328
fetal lung 113	1.0814418	0.28358
fetal renal pelvis 232	1.0977918	0.28724
fetal heart 44	1.1073717	0.29014
fetal renal pelvis 233	1.0948057	0.29138
placenta 199	1.0918122	0.29328
fetal lung 126	1.0763831	0.29372
ES derived trophoblast 287	1.0912274	0.29692
ES cells 294	1.0916032	0.29828
placenta 196	1.0951865	0.29838
fetal lung 114	1.0740264	0.29988
fetal brain 38	1.0670404	0.30132
stomach 283	1.1016986	0.30198
fetal lung 131	1.0689004	0.30426
fetal brain 40	1.0598195	0.30794
fetal muscle 179	1.0816319	0.30836
fetal lung 116	1.0683728	0.31186
fetal renal cortex 205	1.0817710	0.31192
fetal lung 108	1.0765312	0.31194
fetal lung 117	1.0694048	0.31338
iPS 303	1.0671778	0.32320
fetal heart 45	1.0763197	0.32720
fetal heart 48	1.0735384	0.32824
fetal heart 46	1.0737053	0.33018
muscle 341	1.0852433	0.33114
fetal lung 133	1.0599726	0.33804
placenta 198	1.0660267	0.34248
fetal lung 129	1.0588469	0.34312
fetal lung 141	1.0556957	0.34598
fetal brain 29	1.0403412	0.34838
pancreas 328	1.0665332	0.35592
ES cells 296	1.0582869	0.35916
fetal lung 118	1.0485419	0.36198
placenta 200	1.0544396	0.36330
fetal heart 49	1.0531382	0.36608
fetal brain 33	1.0445121	0.36970
fetal muscle 183	1.0477884	0.37072
fetal muscle 151	1.0485381	0.38374
fetal muscle 155	1.0454568	0.38418
Mobilized CD3 320	1.0611640	0.39240
fetal brain 30	1.0263698	0.39836
fetal muscle 144	1.0340894	0.40794
fetal lung 137	1.0307906	0.40802
fetal heart 47	1.0349336	0.40926
fetal muscle 147	1.0321166	0.41170
fetal lung 121	1.0235439	0.42878
fetal muscle 190	1.0229831	0.43088
fetal muscle 160	1.0211497	0.44010
fetal muscle 188	1.0174342	0.44494
Mobilized CD56 324	1.0213458	0.44598
fetal muscle 156	1.0169127	0.44846
fetal kidney 81	1.0155943	0.44932
fetal muscle 154	1.0166289	0.44948
fetal muscle 192	1.0151495	0.45100
fetal muscle 153	1.0149509	0.45444
fetal muscle 163	1.0126540	0.45564
fetal muscle 148	1.0134123	0.45716
fetal muscle 191	1.0119609	0.45760
fetal muscle 189	1.0092332	0.46266
fetal muscle 159	1.0089368	0.46620
fetal muscle 181	1.0076652	0.46816
fetal muscle 184	1.0065781	0.47066
fetal muscle 193	1.0063296	0.47188
fetal muscle 185	1.0071763	0.47200
fetal muscle 146	1.0046011	0.47744
fetal muscle 186	1.0031362	0.48020
fetal muscle 182	1.0022142	0.48136
fetal brain 32	1.0017516	0.48180
fetal muscle 150	0.9987015	0.48986
fetal muscle 161	0.9974567	0.49036
fetal muscle 187	0.9984315	0.49360
fetal muscle 149	0.9974090	0.49398
fetal muscle 168	0.9918164	0.50650
fetal muscle 145	0.9879657	0.50746
fetal muscle 177	0.9901053	0.50880
fetal muscle 167	0.9913767	0.50950
ovary 326	0.9776770	0.51200
fetal muscle 178	0.9883360	0.51362
fetal muscle 180	0.9899151	0.51380
fetal muscle 170	0.9864242	0.51856
fetal muscle 157	0.9889804	0.51860
fetal muscle 194	0.9857511	0.52526
fetal thymus 263	0.9767051	0.53890
fetal muscle 152	0.9789946	0.53932
fetal muscle 171	0.9780742	0.54192
fetal muscle 143	0.9748127	0.55012
fetal muscle 158	0.9692325	0.56566
heart 297	0.9584284	0.56642
fetal muscle 176	0.9643455	0.57572
fetal muscle 162	0.9621474	0.58200
fetal muscle 164	0.9493539	0.60512
fetal muscle 169	0.9451412	0.61456
fetal muscle 165	0.9452543	0.62220
fetal muscle 174	0.9344448	0.64196
fetal muscle 175	0.9211375	0.67138
fetal muscle 166	0.9176864	0.67782
fetal muscle 173	0.9152138	0.68360

Serum metabolite levels

DHS sample	fold enrichment	p value
melanocyte 338	1.2560760	0.00782
melanocyte 339	1.2382349	0.01578
melanocyte 340	1.2464403	0.01960
melanocyte 337	1.2102364	0.01986
keratinocyte 335	1.2060148	0.02448
pancreas 328	1.2835251	0.02686
keratinocyte 333	1.1949394	0.02986
breast 1	1.1994733	0.03354
fetal large intestine 67	1.2615906	0.03454
keratinocyte 336	1.1858655	0.03574
breast 2	1.1907500	0.04382
CD14 4	1.2531448	0.04550
fetal small intestine 74	1.2293788	0.04814
fetal large intestine 64	1.2249295	0.04946
keratinocyte 334	1.1715122	0.05198
muscle 341	1.2393095	0.05940
fetal large intestine 57	1.1995503	0.05982
fetal small intestine 78	1.1955049	0.06014
fetal fibroblast 269	1.1701422	0.06262
CD20 8	1.2014818	0.06410
small intestine 342	1.2084133	0.06494
fetal small intestine 70	1.1843687	0.06508
fetal large intestine 58	1.1901564	0.06600
fibroblast 330	1.1754791	0.06892
fetal small intestine 80	1.1982689	0.07004
fetal adrenal gland 28	1.1739674	0.07130
fetal small intestine 69	1.2181198	0.07158
fetal large intestine 56	1.1998407	0.07354
ES derived mesoderm 285	1.1724900	0.07430
fetal fibroblast 279	1.1636265	0.07498
placenta 200	1.1733548	0.07540
CD14 3	1.2220031	0.07826
fetal large intestine 60	1.2016839	0.07898
ES derived trophoblast 288	1.1713839	0.08722
placenta 198	1.1662803	0.08722
placenta 199	1.1788630	0.08792
fetal lung 299	1.1670034	0.08882
fetal thymus 256	1.1944787	0.08998
fetal large intestine 55	1.1773250	0.09100
fibroblast 332	1.1473545	0.09330
fetal large intestine 62	1.1950515	0.09496
fibroblast 329	1.1495195	0.09730
ES cells 296	1.1973309	0.09864
fetal lung 301	1.1542371	0.09966
fibroblast 331	1.1420317	0.10270
fetal lung 298	1.1560529	0.10324
fetal adrenal gland 25	1.1721495	0.10578
fetal fibroblast 280	1.1395849	0.10580
fetal small intestine 77	1.1740219	0.10820
fetal fibroblast 282	1.1335189	0.10934
iPS 304	1.1537172	0.11020
fetal adrenal gland 24	1.1791342	0.11350
fetal fibroblast 273	1.1334666	0.11698
ES derived mesoderm 286	1.1260288	0.11862
pancreas 327	1.1788491	0.11898
ES cells 294	1.1809150	0.12036
placenta 201	1.1666113	0.12194
fetal skin 234	1.1592138	0.12732
fetal fibroblast 281	1.1275519	0.12758
fetal small intestine 68	1.1455890	0.13146
fetal lung 300	1.1317046	0.13190
fetal small intestine 72	1.1530129	0.13220
fetal small intestine 79	1.1597465	0.13562
fetal fibroblast 274	1.1245179	0.13598
ES derived MSC 289	1.1378105	0.13872
fetal small intestine 73	1.1521178	0.13928
fetal fibroblast 270	1.1210136	0.14268
hematopoietic stem cell 310	1.1131919	0.15034
fetal thymus 258	1.1530724	0.15216
iPS 303	1.1307653	0.15320
ES cells 295	1.1525628	0.15596
CD4 13	1.1660221	0.16178
fetal large intestine 65	1.1375806	0.16214
fetal lung 119	1.1126231	0.16266
fetal large intestine 54	1.1352801	0.16362
ES derived MSC 290	1.1239315	0.16434
Mobilized CD3 320	1.1694637	0.16564
placenta 197	1.1311236	0.16574
fetal fibroblast 275	1.1052969	0.16746
ES derived trophoblast 287	1.1322832	0.16952
fetal large intestine 59	1.1352196	0.17070
fetal thymus 259	1.1505966	0.17100
fetal adrenal gland 23	1.1169869	0.17134
placenta 196	1.1266157	0.17382
fetal small intestine 76	1.1313695	0.17810
fetal lung 140	1.1075794	0.18086
fetal small intestine 71	1.1250604	0.18272
iPS 302	1.1157714	0.18426
fetal large intestine 63	1.1234304	0.18798
fetal fibroblast 268	1.0950252	0.18960
Mobilized CD56 324	1.1304261	0.19326
Mobilized CD4 323	1.1431873	0.19450
CD8 19	1.1516958	0.19654
fetal fibroblast 276	1.0901008	0.20248
fetal fibroblast 271	1.0908315	0.20426
CD8 21	1.1394435	0.20582
fetal adrenal gland 26	1.1056300	0.20690
CD56 18	1.1319686	0.21194
CD3 9	1.1270407	0.21332
fetal large intestine 53	1.1188997	0.21420
fetal muscle 180	1.1039072	0.21766
fetal fibroblast 278	1.0879545	0.21798
fetal muscle 164	1.0992281	0.22194
fetal large intestine 66	1.1083144	0.22316
fetal lung 111	1.0864912	0.22526
fetal muscle 169	1.0971433	0.22700
fetal thymus 260	1.1087126	0.22920
hematopoietic stem cell 309	1.0907717	0.23220
hematopoietic stem cell 314	1.0839651	0.23482
fetal renal cortex 207	1.0967372	0.23806
hematopoietic stem cell 308	1.0851182	0.23814
CD3 10	1.1081625	0.23892
CD4 14	1.1046374	0.24148
CD19 7	1.1154778	0.24430
fetal fibroblast 272	1.0741849	0.24980
fetal adrenal gland 27	1.0859671	0.25360
fetal thymus 265	1.0965475	0.25400
fetal fibroblast 267	1.0743574	0.25452
fetal muscle 153	1.0879379	0.25734
Mobilized CD4 322	1.1070695	0.25800
fetal fibroblast 277	1.0731519	0.25826
hematopoietic stem cell 312	1.0770856	0.25836
Mobilized CD8 325	1.1068643	0.26040
CD19 5	1.0974172	0.26454
fetal lung 117	1.0715333	0.26498
hematopoietic stem cell 313	1.0727557	0.26906
fetal muscle 151	1.0789884	0.27596
fetal thymus 266	1.0838166	0.27888
ES cells 293	1.0803715	0.27998
fetal thymus 264	1.0811511	0.28042
fetal lung 129	1.0674820	0.28100
fetal muscle 176	1.0720870	0.28128
fetal kidney 103	1.0785700	0.28160
CD56 17	1.0814307	0.28318
fetal stomach 244	1.0799503	0.28332
fetal spinal cord 236	1.0707019	0.28554
fetal lung 133	1.0608003	0.29238
fetal muscle 149	1.0723096	0.29258
CD3 12	1.0885808	0.29270
fetal renal pelvis 219	1.0841304	0.29386
fetal ovary 195	1.0828495	0.29406
fetal renal cortex 213	1.0660892	0.29746
hematopoietic stem cell 306	1.0622972	0.30120
CD3 11	1.0744305	0.30320
CD19 6	1.0809297	0.30382
CD4 15	1.0921896	0.30556
fetal muscle 193	1.0646489	0.30690
fetal muscle 154	1.0665045	0.30694
hematopoietic stem cell 315	1.0622798	0.30814
hematopoietic stem cell 305	1.0622996	0.30862
fetal thymus 261	1.0715793	0.31086
hematopoietic stem cell 307	1.0587792	0.31114
fetal muscle 171	1.0588552	0.31342
fetal muscle 165	1.0582844	0.31456
Mobilized CD3 321	1.0781447	0.31466
ovary 326	1.0721442	0.31670
fetal muscle 167	1.0573489	0.31690
fetal spinal cord 235	1.0560811	0.31712
fetal lung 132	1.0557540	0.31842
fetal thymus 257	1.0709777	0.31950
fetal renal pelvis 220	1.0619667	0.32002
fetal muscle 179	1.0575918	0.32148
fetal renal cortex 214	1.0573114	0.32748
fetal testes 255	1.0536035	0.32918
fetal muscle 157	1.0513359	0.32998
fetal muscle 166	1.0523847	0.33370
fetal kidney 92	1.0520916	0.33404
heart 297	1.0565356	0.33490
fetal stomach 242	1.0662442	0.33502
fetal large intestine 61	1.0639237	0.33634
fetal muscle 194	1.0475631	0.33794
fetal renal pelvis 231	1.0517454	0.33874
fetal spinal cord 238	1.0488672	0.33884
fetal lung 128	1.0430783	0.33884
hematopoietic stem cell 311	1.0489767	0.33916
fetal renal cortex 205	1.0523288	0.34044
fetal kidney 106	1.0513356	0.34096
hematopoietic stem cell 317	1.0426621	0.34350
fetal lung 114	1.0415457	0.34684
fetal renal pelvis 233	1.0512243	0.34712
fetal muscle 159	1.0501713	0.34874
fetal brain 36	1.0418154	0.34936
fetal muscle 150	1.0473870	0.35194
fetal testes 254	1.0477497	0.35218
fetal muscle 183	1.0453014	0.35400
stomach 284	1.0506492	0.35424
stomach 283	1.0507677	0.35466
fetal muscle 152	1.0445031	0.35540
hematopoietic stem cell 316	1.0457459	0.35726
fetal kidney 84	1.0454640	0.35908
fetal kidney 91	1.0424988	0.36232
fetal muscle 173	1.0426524	0.36302
fetal muscle 181	1.0414438	0.36404
fetal muscle 175	1.0407331	0.36610
fetal heart 47	1.0442413	0.36780
fetal kidney 85	1.0368829	0.36878
fetal muscle 143	1.0377018	0.36996
fetal muscle 182	1.0391911	0.37010
fetal lung 125	1.0385067	0.37024
fetal kidney 95	1.0409407	0.37190
hematopoietic stem cell 319	1.0407484	0.37206
fetal kidney 83	1.0445842	0.37288
fetal muscle 185	1.0405957	0.37398
fetal muscle 168	1.0400988	0.37506
fetal kidney 99	1.0394835	0.37548
fetal renal pelvis 228	1.0417591	0.37660
fetal kidney 89	1.0397937	0.37666
fetal muscle 148	1.0374477	0.37802
fetal lung 118	1.0319372	0.37920
fetal muscle 172	1.0327376	0.37998
fetal muscle 174	1.0371225	0.38112
fetal stomach 248	1.0395853	0.38220
fetal lung 108	1.0346585	0.38454
fetal lung 141	1.0304200	0.38670
fetal thymus 262	1.0436649	0.38692
hematopoietic stem cell 318	1.0348753	0.38712
fetal stomach 247	1.0411314	0.38754
fetal heart 49	1.0336639	0.38754
fetal renal pelvis 221	1.0329415	0.38948
fetal lung 116	1.0286198	0.38998
fetal heart 45	1.0327162	0.39028
ES derived NP 291	1.0379645	0.39050
fetal renal pelvis 226	1.0332193	0.39100
fetal kidney 96	1.0321124	0.39308
fetal thymus 263	1.0331639	0.39420
fetal lung 124	1.0290313	0.39424
fetal renal cortex 215	1.0309503	0.39458
fetal renal pelvis 227	1.0301026	0.39890
fetal kidney 87	1.0310199	0.40022
fetal renal cortex 212	1.0282344	0.40282
fetal stomach 240	1.0320351	0.40332
fetal brain 37	1.0254248	0.40348
fetal kidney 81	1.0266764	0.40590
fetal lung 120	1.0262610	0.40682
fetal lung 121	1.0238216	0.41108
CD8 20	1.0305740	0.41308
ES derived NP 292	1.0270061	0.41344
fetal muscle 184	1.0235083	0.41418
fetal stomach 251	1.0261087	0.41448
fetal lung 134	1.0238559	0.41574
fetal kidney 100	1.0248961	0.41714
fetal muscle 146	1.0234257	0.41714
fetal muscle 155	1.0239793	0.42052
fetal renal pelvis 225	1.0230069	0.42056
fetal stomach 246	1.0244221	0.42188
CD4 16	1.0257758	0.42214
fetal renal pelvis 232	1.0219521	0.42548
fetal renal cortex 202	1.0196638	0.42824
fetal lung 130	1.0186128	0.42838
fetal muscle 177	1.0182296	0.43326
fetal muscle 163	1.0182795	0.43386
fetal stomach 241	1.0194095	0.43454
fetal muscle 191	1.0175237	0.43546
fetal kidney 88	1.0168713	0.43606
fetal muscle 189	1.0168490	0.43790
fetal muscle 187	1.0165112	0.43800
fetal lung 112	1.0157763	0.43940
fetal lung 110	1.0150902	0.44078
CD8 22	1.0166966	0.44354
fetal muscle 192	1.0146683	0.44402
fetal stomach 253	1.0148886	0.44566
fetal muscle 160	1.0139984	0.44710
fetal lung 115	1.0121521	0.45026
fetal kidney 101	1.0108903	0.45112
fetal renal pelvis 217	1.0129521	0.45152
fetal renal cortex 206	1.0120457	0.45332
fetal muscle 156	1.0116350	0.45374
fetal kidney 105	1.0107764	0.45442
fetal heart 43	1.0117120	0.45454
fetal stomach 252	1.0103478	0.45618
fetal stomach 250	1.0110979	0.45634
fetal lung 136	1.0102843	0.45672
fetal kidney 102	1.0087229	0.45964
fetal muscle 145	1.0087256	0.46076
fetal kidney 104	1.0076756	0.46190
fetal lung 131	1.0075251	0.46202
fetal muscle 186	1.0086483	0.46268
fetal stomach 249	1.0076445	0.46352
fetal renal pelvis 229	1.0074105	0.46360
fetal lung 137	1.0079114	0.46466
fetal lung 123	1.0075836	0.46474
fetal muscle 162	1.0075838	0.46670
fetal muscle 178	1.0073409	0.46786
fetal muscle 161	1.0058246	0.46808
fetal kidney 97	1.0056879	0.46898
fetal lung 113	1.0057167	0.47244
fetal muscle 144	1.0060028	0.47402
fetal renal cortex 211	1.0037823	0.47456
fetal kidney 107	1.0033206	0.47506
fetal heart 46	1.0051715	0.47654
fetal lung 109	1.0040780	0.47706
fetal lung 135	1.0042341	0.47748
fetal renal pelvis 224	1.0019992	0.47990
fetal lung 127	1.0025380	0.48266
fetal muscle 147	1.0021471	0.48472
fetal muscle 170	1.0010081	0.48628
fetal kidney 82	0.9974518	0.49130
fetal heart 51	0.9993974	0.49266
fetal kidney 98	0.9973575	0.49332
fetal lung 138	0.9983616	0.49834
fetal lung 126	0.9970684	0.50172
fetal kidney 86	0.9906884	0.50704
fetal stomach 243	0.9920999	0.50806
fetal muscle 190	0.9928083	0.50814
fetal heart 42	0.9931770	0.51294
fetal spinal cord 239	0.9923107	0.51322
fetal renal pelvis 223	0.9889069	0.51552
fetal small intestine 75	0.9762415	0.51610
fetal muscle 158	0.9913072	0.51672
fetal heart 44	0.9890321	0.51986
fetal renal pelvis 230	0.9865070	0.52278
fetal spinal cord 237	0.9900877	0.52370
fetal muscle 188	0.9873136	0.52738
fetal brain 40	0.9901637	0.52768
fetal heart 48	0.9864625	0.53208
fetal kidney 94	0.9804248	0.53612
fetal heart 52	0.9863248	0.53720
fetal renal pelvis 218	0.9753834	0.53908
fetal brain 32	0.9856372	0.54290
fetal brain 41	0.9838689	0.54676
fetal renal cortex 204	0.9779147	0.55828
fetal lung 122	0.9771939	0.55914
fetal brain 34	0.9817216	0.56168
fetal stomach 245	0.9697880	0.56774
fetal brain 38	0.9772047	0.56786
fetal lung 142	0.9755363	0.57716
fetal lung 139	0.9715271	0.58036
fetal renal cortex 210	0.9648540	0.58336
fetal kidney 90	0.9650152	0.58516
fetal brain 33	0.9721292	0.58558
fetal renal pelvis 222	0.9625478	0.58804
fetal brain 39	0.9707201	0.59524
fetal heart 50	0.9640757	0.60150
fetal renal pelvis 216	0.9545447	0.61222
fetal renal cortex 209	0.9525747	0.62098
fetal renal cortex 208	0.9512157	0.62324
fetal kidney 93	0.9488096	0.62610
fetal brain 35	0.9638560	0.63410
fetal renal cortex 203	0.9386536	0.65868
fetal brain 30	0.9401579	0.73406
fetal brain 29	0.9343701	0.74894
fetal brain 31	0.9298703	0.76724

Lipid levels 5

DHS sample	fold enrichment	p value
fetal small intestine 80	1.4793850	0.00766
fetal large intestine 57	1.4329530	0.00878
fetal large intestine 56	1.4771288	0.00958
fetal large intestine 64	1.4543945	0.01006
fetal small intestine 78	1.4130534	0.01022
fetal large intestine 58	1.4111902	0.01190
fetal large intestine 55	1.4221412	0.01364
fetal large intestine 60	1.4566019	0.01436
CD20 8	1.4098318	0.01444
small intestine 342	1.4318666	0.01578
fetal small intestine 74	1.4338720	0.01594
fetal large intestine 67	1.4189705	0.01930
fetal small intestine 70	1.3529235	0.01950
fetal large intestine 65	1.4080087	0.02284
fetal small intestine 76	1.4036148	0.02728
fetal small intestine 72	1.3813395	0.02880
fetal large intestine 54	1.3769405	0.03140
fetal small intestine 73	1.3814313	0.03192
ES derived mesoderm 286	1.2646774	0.03218
stomach 284	1.3899379	0.03224
fetal small intestine 69	1.3970066	0.03380
fetal adrenal gland 28	1.2960939	0.03582
fetal small intestine 68	1.3326662	0.03724
fetal small intestine 77	1.3563291	0.03870
fetal small intestine 79	1.3633247	0.04190
placenta 200	1.2877453	0.04190
melanocyte 338	1.2325973	0.04590
fetal small intestine 71	1.3419518	0.04750
fetal large intestine 62	1.3515968	0.05028
keratinocyte 336	1.2245249	0.05034
melanocyte 337	1.2141703	0.05272
muscle 341	1.3512082	0.05328
placenta 198	1.2790879	0.05504
pancreas 328	1.3370326	0.05578
CD14 4	1.3244034	0.06006
keratinocyte 333	1.2048285	0.06458
fetal large intestine 59	1.3119379	0.06570
fetal adrenal gland 23	1.2599043	0.06704
fetal thymus 265	1.3301181	0.06744
fetal adrenal gland 25	1.2991097	0.06806
keratinocyte 334	1.2039526	0.06896
fetal thymus 256	1.3093154	0.06904
ES derived mesoderm 285	1.2212876	0.07246
fetal adrenal gland 24	1.3028363	0.07454
fetal large intestine 63	1.2924275	0.07598
melanocyte 340	1.2211659	0.07710
fetal thymus 258	1.3123277	0.07760
pancreas 327	1.3004000	0.08320
breast 1	1.1981667	0.08672
ES derived trophoblast 288	1.2299783	0.08798
fetal large intestine 66	1.2721482	0.08918
fibroblast 332	1.2018489	0.09138
fetal brain 29	1.1575272	0.10194
breast 2	1.1840687	0.10214
fetal skin 234	1.2319832	0.10352
fetal adrenal gland 26	1.2334169	0.10406
fetal heart 45	1.2217941	0.10520
fetal thymus 264	1.2642873	0.10596
fetal large intestine 53	1.2735240	0.10808
fetal brain 35	1.1617794	0.10856
CD14 3	1.2705649	0.11026
fetal brain 34	1.1655196	0.11122
fibroblast 331	1.1838129	0.11440
fetal brain 38	1.1840529	0.11550
fetal brain 41	1.1762771	0.11554
keratinocyte 335	1.1608146	0.11612
ES derived MSC 290	1.2080716	0.11722
fibroblast 329	1.1823648	0.11984
ES cells 294	1.2442393	0.12394
fetal brain 30	1.1378623	0.12436
ES derived MSC 289	1.2019589	0.12642
fetal thymus 260	1.2497588	0.12754
placenta 197	1.2181453	0.12998
iPS 304	1.1887127	0.13034
fetal brain 39	1.1545822	0.13492
fetal renal cortex 212	1.1891898	0.13732
iPS 302	1.1889586	0.13984
melanocyte 339	1.1529104	0.14390
Mobilized CD56 324	1.2279650	0.14726
stomach 283	1.2166260	0.14782
fetal brain 31	1.1284497	0.14872
fibroblast 330	1.1611805	0.15024
fetal thymus 259	1.2291864	0.15648
fetal heart 52	1.1624295	0.15686
fetal testes 254	1.1814364	0.16198
fetal renal pelvis 231	1.1619447	0.16864
fetal adrenal gland 27	1.1744492	0.16900
hematopoietic stem cell 310	1.1473295	0.16910
ES cells 296	1.1903346	0.17508
fetal thymus 266	1.1978824	0.17704
fetal heart 46	1.1680675	0.17830
fetal brain 40	1.1237743	0.18048
fetal heart 43	1.1535680	0.18066
iPS 303	1.1547194	0.18248
fetal heart 49	1.1562556	0.18620
Mobilized CD8 325	1.2166436	0.18766
fetal kidney 106	1.1542099	0.18850
fetal lung 300	1.1385076	0.18940
placenta 201	1.1690144	0.19048
fetal lung 301	1.1401448	0.19102
CD56 17	1.1723491	0.19590
placenta 199	1.1469699	0.19862
fetal renal pelvis 225	1.1518848	0.20210
fetal renal cortex 204	1.1354680	0.20418
heart 297	1.1551789	0.20570
fetal ovary 195	1.1792032	0.20664
CD4 13	1.1945470	0.20868
fetal heart 42	1.1375185	0.21106
fetal fibroblast 279	1.1127559	0.21820
hematopoietic stem cell 314	1.1263197	0.21978
fetal lung 298	1.1256932	0.22024
fetal lung 299	1.1237352	0.22044
hematopoietic stem cell 315	1.1371240	0.22324
fetal renal pelvis 227	1.1319665	0.22324
CD8 21	1.1821014	0.22430
placenta 196	1.1382733	0.22526
fetal heart 51	1.1257791	0.22716
CD8 19	1.1911150	0.22756
fetal renal pelvis 233	1.1334987	0.22854
CD56 18	1.1745069	0.23218
fetal brain 36	1.1037071	0.23338
fetal kidney 96	1.1223345	0.23372
fetal kidney 105	1.1203682	0.23698
Mobilized CD3 321	1.1741672	0.23742
fetal kidney 82	1.1350854	0.23780
fetal renal pelvis 220	1.1262403	0.23858
CD4 14	1.1467030	0.23914
hematopoietic stem cell 312	1.1164372	0.24050
fetal spinal cord 236	1.1159966	0.24080
fetal renal cortex 206	1.1198147	0.24086
fetal kidney 90	1.1210850	0.24468
fetal renal pelvis 224	1.1141324	0.24488
ES cells 295	1.1360846	0.24620
fetal fibroblast 269	1.0966564	0.24666
CD8 20	1.1451447	0.24708
fetal renal cortex 205	1.1175297	0.24802
fetal stomach 244	1.1275171	0.24942
fetal kidney 89	1.1188861	0.25062
hematopoietic stem cell 305	1.1184639	0.25062
fetal kidney 88	1.1114967	0.25084
fetal fibroblast 273	1.0959100	0.25144
CD3 9	1.1504544	0.25302
fetal kidney 95	1.1148904	0.25418
CD3 11	1.1377247	0.25446
fetal fibroblast 280	1.0929370	0.25730
fetal brain 32	1.0900758	0.25836
fetal large intestine 61	1.1437325	0.25998
fetal kidney 83	1.1228664	0.26106
fetal testes 255	1.1053411	0.26162
hematopoietic stem cell 309	1.1100125	0.26182
fetal fibroblast 281	1.0911424	0.26196
fetal renal pelvis 221	1.1046382	0.26312
fetal heart 48	1.1047590	0.26464
fetal spinal cord 237	1.0975915	0.26562
fetal heart 44	1.1228164	0.26720
fetal lung 128	1.0836387	0.26874
ES derived trophoblast 287	1.1096343	0.27020
fetal thymus 261	1.1298470	0.27028
fetal heart 47	1.1085186	0.27078
fetal renal cortex 208	1.1075232	0.27082
fetal fibroblast 282	1.0844973	0.27184
fetal kidney 99	1.1033258	0.27324
fetal renal pelvis 216	1.1027032	0.27668
fetal lung 134	1.0929714	0.27718
fetal fibroblast 268	1.0825765	0.28006
hematopoietic stem cell 316	1.1025648	0.28260
fetal lung 109	1.0774134	0.28402
CD4 15	1.1458666	0.28456
fetal renal cortex 214	1.0940883	0.28724
fetal renal pelvis 229	1.0924836	0.28766
fetal renal cortex 207	1.0936114	0.28778
fetal lung 125	1.0851245	0.28968
fetal renal cortex 210	1.0974602	0.28970
ES cells 293	1.1009484	0.29022
fetal renal pelvis 217	1.0946903	0.29048
fetal kidney 101	1.0921316	0.29110
fetal renal cortex 215	1.0874077	0.29110
fetal kidney 86	1.1078407	0.29426
fetal stomach 242	1.1170054	0.29614
CD3 10	1.1109017	0.29694
fetal renal pelvis 232	1.0946488	0.29756
fetal brain 37	1.0727950	0.29804
fetal stomach 253	1.1017593	0.29840
CD8 22	1.1149602	0.29970
ovary 326	1.1081757	0.29970
fetal kidney 81	1.0793248	0.29978
fetal renal cortex 211	1.0926291	0.29994
fetal stomach 247	1.1097501	0.30090
fetal kidney 87	1.0855674	0.30124
fetal fibroblast 274	1.0744197	0.30136
fetal spinal cord 238	1.0792133	0.30292
fetal kidney 91	1.0792735	0.30296
fetal thymus 257	1.1123387	0.30298
fetal renal pelvis 219	1.1019560	0.30316
fetal kidney 104	1.0865991	0.30350
hematopoietic stem cell 317	1.0750461	0.30504
fetal renal pelvis 228	1.0894809	0.30634
fetal kidney 98	1.0839095	0.30766
Mobilized CD4 323	1.1104970	0.30808
hematopoietic stem cell 318	1.0886876	0.30986
fetal lung 129	1.0729699	0.31040
hematopoietic stem cell 306	1.0804102	0.31170
fetal kidney 103	1.0791913	0.31414
fetal heart 50	1.0777530	0.31692
fetal stomach 246	1.0907282	0.31734
fetal stomach 248	1.0877749	0.31814
Mobilized CD4 322	1.1051877	0.31818
fetal spinal cord 235	1.0681799	0.31964
fetal fibroblast 271	1.0658438	0.31996
fetal renal pelvis 223	1.0796626	0.32064
fetal renal pelvis 218	1.0906216	0.32170
fetal stomach 250	1.0896649	0.32212
fetal stomach 252	1.0831479	0.32348
fetal fibroblast 270	1.0637066	0.32544
hematopoietic stem cell 308	1.0721455	0.32656
fetal kidney 102	1.0749717	0.33020
fetal kidney 85	1.0606238	0.33118
fetal lung 127	1.0659572	0.33154
fetal lung 132	1.0636277	0.33286
fetal renal pelvis 222	1.0718789	0.33432
fetal kidney 107	1.0696228	0.33484
fetal thymus 262	1.0962968	0.33594
fetal renal cortex 209	1.0708310	0.33650
fetal renal pelvis 226	1.0650503	0.33682
fetal kidney 97	1.0663496	0.33822
fetal lung 112	1.0598907	0.33902
fetal spinal cord 239	1.0587870	0.34480
fetal kidney 84	1.0632009	0.34774
fetal kidney 93	1.0653870	0.34862
fetal fibroblast 278	1.0540338	0.35026
fetal thymus 263	1.0613165	0.35170
hematopoietic stem cell 307	1.0595306	0.35360
fetal fibroblast 272	1.0524577	0.35440
CD4 16	1.0733917	0.35586
fetal stomach 241	1.0656392	0.35750
hematopoietic stem cell 313	1.0547095	0.35838
fetal muscle 187	1.0526536	0.35976
fetal muscle 166	1.0541816	0.35986
CD3 12	1.0754534	0.36072
fetal fibroblast 277	1.0490895	0.36212
hematopoietic stem cell 311	1.0551812	0.36298
fetal kidney 100	1.0531856	0.36444
fetal muscle 144	1.0546168	0.36506
CD19 5	1.0680150	0.36624
CD19 6	1.0653855	0.36758
fetal stomach 249	1.0603418	0.36758
fetal stomach 240	1.0605002	0.36838
fetal renal cortex 202	1.0496329	0.36920
fetal lung 142	1.0434330	0.37098
fetal fibroblast 275	1.0431690	0.37268
fetal small intestine 75	1.0628343	0.37508
fetal brain 33	1.0443475	0.37518
fetal kidney 92	1.0446760	0.37740
fetal muscle 172	1.0435707	0.37762
fetal muscle 185	1.0501899	0.37778
fetal fibroblast 276	1.0414116	0.37868
fetal muscle 151	1.0490771	0.37952
fetal muscle 173	1.0467024	0.37966
fetal lung 117	1.0410298	0.38058
fetal lung 108	1.0443712	0.38074
CD19 7	1.0580466	0.38134
fetal fibroblast 267	1.0410078	0.38182
fetal lung 113	1.0398394	0.38344
fetal muscle 155	1.0476259	0.38356
fetal muscle 175	1.0419829	0.38650
fetal muscle 194	1.0381050	0.38794
fetal kidney 94	1.0439585	0.38852
fetal muscle 193	1.0416182	0.39230
fetal muscle 184	1.0387516	0.39252
fetal lung 123	1.0369066	0.39456
fetal lung 121	1.0343211	0.39704
fetal lung 131	1.0318281	0.39870
fetal lung 137	1.0333721	0.39898
fetal muscle 182	1.0364239	0.39948
fetal lung 138	1.0343509	0.40136
fetal stomach 245	1.0393555	0.40170
fetal muscle 157	1.0334454	0.40508
fetal muscle 189	1.0329724	0.40550
fetal muscle 191	1.0346636	0.40628
fetal stomach 243	1.0350100	0.40780
fetal lung 130	1.0303228	0.40866
fetal muscle 168	1.0320800	0.41410
fetal lung 120	1.0277841	0.41534
fetal muscle 164	1.0301028	0.41558
fetal lung 114	1.0248639	0.42176
fetal muscle 145	1.0266410	0.42212
fetal renal cortex 203	1.0267484	0.42300
fetal renal cortex 213	1.0214441	0.42878
fetal renal pelvis 230	1.0225558	0.42936
fetal lung 122	1.0229870	0.42970
Mobilized CD3 320	1.0303885	0.43174
hematopoietic stem cell 319	1.0228410	0.43408
fetal muscle 174	1.0201286	0.43696
fetal muscle 153	1.0214037	0.43808
fetal muscle 146	1.0182443	0.44118
fetal muscle 192	1.0149672	0.45352
fetal muscle 180	1.0160304	0.45422
fetal lung 110	1.0133099	0.45508
fetal lung 119	1.0121906	0.45780
fetal lung 140	1.0110733	0.45986
fetal stomach 251	1.0059871	0.46392
ES derived NP 292	1.0086242	0.46508
fetal muscle 154	1.0067447	0.47136
fetal lung 124	1.0050040	0.47366
fetal muscle 178	1.0054747	0.47398
fetal muscle 171	1.0045978	0.47596
fetal lung 139	1.0015792	0.48146
fetal muscle 179	1.0005973	0.48436
fetal muscle 188	0.9989195	0.48868
fetal muscle 167	1.0003959	0.48946
fetal lung 116	1.0012013	0.48950
fetal muscle 147	0.9985323	0.48954
fetal muscle 190	0.9957636	0.49050
fetal lung 141	0.9987732	0.49130
fetal lung 135	0.9972414	0.49304
fetal muscle 183	0.9978434	0.49318
fetal lung 118	0.9980693	0.49652
fetal lung 115	0.9964028	0.49768
fetal muscle 156	0.9955877	0.49878
fetal muscle 165	0.9957399	0.49908
fetal muscle 152	0.9942009	0.50410
fetal lung 133	0.9936036	0.50418
fetal muscle 177	0.9915298	0.50480
fetal lung 126	0.9930758	0.51072
fetal muscle 159	0.9885632	0.51316
fetal lung 111	0.9916868	0.51410
fetal lung 136	0.9867983	0.51928
fetal muscle 158	0.9858732	0.52148
fetal muscle 161	0.9816498	0.52576
fetal muscle 150	0.9836385	0.52668
fetal muscle 148	0.9804856	0.53406
fetal muscle 170	0.9813072	0.53410
fetal muscle 169	0.9787964	0.53532
ES derived NP 291	0.9708817	0.53832
fetal muscle 163	0.9702019	0.55282
fetal muscle 160	0.9714071	0.55718
fetal muscle 176	0.9702219	0.56026
fetal muscle 181	0.9663316	0.56800
fetal muscle 162	0.9677461	0.56978
fetal muscle 186	0.9529143	0.59962
fetal muscle 149	0.9471281	0.60990
fetal muscle 143	0.9303141	0.66860

Blood cell traits

DHS sample	fold enrichment	p value
Mobilized CD56 324	1.8492345	0.00072
hematopoietic stem cell 310	1.5364704	0.00142
fetal thymus 256	1.7272822	0.00208
fetal thymus 258	1.7287428	0.00272
fetal thymus 265	1.6550781	0.00470
hematopoietic stem cell 314	1.4973283	0.00516
fetal kidney 85	1.5038442	0.00632
CD56 18	1.6837474	0.00694
fetal thymus 264	1.5878214	0.00904
fetal thymus 259	1.6256924	0.01048
fetal muscle 176	1.4708942	0.01246
Mobilized CD3 320	1.6592157	0.01384
fetal fibroblast 277	1.4310682	0.01398
CD3 11	1.5342544	0.01436
CD8 21	1.5931649	0.01514
hematopoietic stem cell 315	1.4583582	0.01586
fetal fibroblast 273	1.3927120	0.01604
melanocyte 338	1.4008059	0.01644
fetal thymus 260	1.5436196	0.01716
hematopoietic stem cell 309	1.4378164	0.01748
CD8 19	1.6411603	0.01772
fetal fibroblast 278	1.4081454	0.01774
fetal fibroblast 275	1.3823313	0.01870
fetal fibroblast 274	1.3918556	0.01884
fetal muscle 180	1.4869384	0.01900
Mobilized CD4 323	1.5599823	0.02082
CD4 13	1.5541840	0.02282
fetal muscle 143	1.3995398	0.02326
melanocyte 337	1.3461143	0.02394
fetal fibroblast 276	1.3664002	0.02406
fetal fibroblast 272	1.3574737	0.02662
fetal thymus 266	1.4815702	0.02726
fetal lung 111	1.3464101	0.02932
CD3 9	1.5048042	0.02984
fetal muscle 181	1.4030832	0.03070
fetal large intestine 61	1.4944670	0.03184
CD4 14	1.4418481	0.03318
hematopoietic stem cell 308	1.3590684	0.03320
Mobilized CD4 322	1.5236669	0.03388
fetal skin 234	1.4088426	0.03394
hematopoietic stem cell 312	1.3667604	0.03416
fetal thymus 261	1.4687061	0.03538
hematopoietic stem cell 318	1.3986015	0.03554
CD56 17	1.4095556	0.03656
fetal muscle 149	1.3987084	0.03658
hematopoietic stem cell 316	1.3900067	0.03872
fetal muscle 152	1.3689466	0.03978
fetal fibroblast 281	1.3207697	0.04150
CD3 12	1.4759453	0.04238
hematopoietic stem cell 305	1.3561743	0.04436
CD8 20	1.4134686	0.04462
melanocyte 339	1.3302399	0.04484
fetal fibroblast 282	1.2984526	0.04648
fetal fibroblast 268	1.2988316	0.04702
fetal muscle 179	1.3599580	0.04724
Mobilized CD8 325	1.4683647	0.04764
fetal thymus 257	1.4439467	0.04834
fetal lung 298	1.3403225	0.05028
fetal muscle 151	1.3757578	0.05040
hematopoietic stem cell 317	1.2912155	0.05124
fetal fibroblast 280	1.3082057	0.05308
Mobilized CD3 321	1.4616961	0.05446
fetal fibroblast 271	1.2938786	0.05550
fetal muscle 153	1.3702516	0.05576
fetal muscle 166	1.3366580	0.05670
hematopoietic stem cell 313	1.2962411	0.06140
fetal muscle 154	1.3548026	0.06158
fetal muscle 167	1.3133826	0.06578
fetal muscle 169	1.3209400	0.06640
fetal lung 140	1.2814054	0.06726
fetal lung 299	1.3089346	0.06736
fetal fibroblast 279	1.2840839	0.06744
fetal renal cortex 213	1.3178648	0.06822
fetal fibroblast 267	1.2809943	0.07086
fetal lung 128	1.2492750	0.07256
fetal kidney 91	1.3092569	0.07304
CD8 22	1.3897346	0.07390
fetal muscle 160	1.3185094	0.07468
fetal lung 141	1.2565397	0.07700
fetal muscle 164	1.3185391	0.07734
fetal kidney 106	1.3209227	0.07834
hematopoietic stem cell 307	1.2896632	0.07968
CD14 4	1.3246371	0.08022
fetal fibroblast 269	1.2568914	0.08050
fibroblast 332	1.2552467	0.08296
ES derived MSC 289	1.2849031	0.08504
fetal fibroblast 270	1.2563907	0.08974
hematopoietic stem cell 306	1.2644462	0.09122
CD14 3	1.3205425	0.09326
fetal muscle 150	1.2951140	0.09346
fetal renal pelvis 231	1.2865476	0.09400
CD4 15	1.4089745	0.09478
fetal muscle 182	1.2713714	0.09662
fetal muscle 178	1.2848111	0.09686
fetal kidney 92	1.2783220	0.10054
hematopoietic stem cell 311	1.2565175	0.10056
ES derived mesoderm 286	1.2181995	0.10148
fetal renal pelvis 226	1.2753098	0.10156
fetal muscle 158	1.2590262	0.10436
fetal lung 120	1.2418844	0.10590
fetal muscle 187	1.2539901	0.10714
ES derived mesoderm 285	1.2318216	0.10808
fetal renal pelvis 227	1.2723331	0.11068
CD19 5	1.3087370	0.11104
keratinocyte 333	1.2022840	0.11232
CD3 10	1.2985938	0.11260
fetal muscle 165	1.2390358	0.11568
fetal lung 116	1.2107723	0.11864
fetal renal cortex 207	1.2617224	0.11882
fetal lung 119	1.2164852	0.11886
fetal renal pelvis 220	1.2653586	0.11952
fibroblast 331	1.2191309	0.11968
fetal lung 114	1.2032661	0.12014
fetal stomach 248	1.2818964	0.12106
placenta 200	1.2262549	0.12188
fetal muscle 168	1.2514741	0.12316
fetal muscle 159	1.2602024	0.12534
fetal kidney 95	1.2570931	0.12548
fetal kidney 105	1.2453304	0.12548
fetal muscle 148	1.2512014	0.12652
fetal muscle 171	1.2355456	0.12674
keratinocyte 335	1.1909157	0.12898
melanocyte 340	1.2262551	0.12944
ES derived MSC 290	1.2332576	0.12962
fetal lung 123	1.2215316	0.13186
fetal muscle 170	1.2235410	0.13278
keratinocyte 334	1.1905869	0.13336
fetal renal cortex 204	1.2300323	0.13454
fetal small intestine 75	1.3617945	0.13518
fetal lung 117	1.2061280	0.13582
fetal muscle 157	1.2162773	0.13584
fetal lung 301	1.2170786	0.13634
fetal muscle 175	1.2253202	0.13640
fetal renal cortex 202	1.2324557	0.13910
CD19 6	1.2808771	0.14228
fetal stomach 244	1.2417841	0.14906
keratinocyte 336	1.1762118	0.14948
fetal muscle 161	1.2235457	0.14972
fetal large intestine 67	1.2243883	0.15114
fetal lung 133	1.1855218	0.15258
fetal renal pelvis 217	1.2366880	0.15300
fibroblast 330	1.1965967	0.15334
fetal muscle 185	1.2244837	0.15390
CD19 7	1.2695185	0.15414
fetal renal pelvis 221	1.2167268	0.15418
CD4 16	1.2740403	0.15536
fibroblast 329	1.1899901	0.15658
fetal renal pelvis 229	1.2158175	0.15756
fetal kidney 100	1.2195258	0.15816
fetal lung 131	1.1718813	0.16080
fetal kidney 88	1.2067465	0.16474
fetal stomach 243	1.2149384	0.16612
fetal spinal cord 235	1.1894617	0.16630
ovary 326	1.2498135	0.16642
fetal muscle 172	1.1793983	0.16788
fetal kidney 83	1.2336643	0.16844
fetal muscle 162	1.1946087	0.16848
fetal heart 47	1.2155288	0.16980
fetal muscle 183	1.1933777	0.17028
fetal lung 142	1.1684905	0.17214
fetal muscle 189	1.1994522	0.17288
fetal thymus 262	1.2642299	0.17464
fetal kidney 89	1.2056964	0.17586
fetal muscle 184	1.1811678	0.17742
fetal kidney 107	1.2036961	0.17776
fetal lung 121	1.1633041	0.17818
fetal thymus 263	1.1942557	0.17932
fetal lung 108	1.1887043	0.17972
fetal renal cortex 209	1.1990453	0.18384
fetal lung 134	1.1729992	0.18394
fetal kidney 98	1.2015977	0.18448
fetal lung 129	1.1688204	0.18456
fetal muscle 156	1.1860631	0.18486
fetal lung 110	1.1754953	0.18570
fetal kidney 84	1.1996969	0.18594
fetal muscle 194	1.1672542	0.18606
hematopoietic stem cell 319	1.1869269	0.18854
fetal renal pelvis 228	1.2050739	0.18870
fetal kidney 101	1.1855234	0.19114
fetal muscle 155	1.1883401	0.19234
fetal renal cortex 205	1.1832019	0.19402
fetal muscle 190	1.1941489	0.19478
fetal renal cortex 212	1.1814260	0.19540
fetal testes 255	1.1743823	0.19686
fetal adrenal gland 28	1.1527227	0.19874
fetal lung 132	1.1600166	0.19962
fetal brain 33	1.1584714	0.19998
fetal kidney 99	1.1804389	0.20126
fetal lung 118	1.1472991	0.20126
placenta 198	1.1640134	0.20138
fetal renal cortex 206	1.1755681	0.20264
fetal muscle 146	1.1682201	0.20322
breast 1	1.1462610	0.20322
fetal muscle 173	1.1741719	0.20392
fetal lung 300	1.1580786	0.20708
fetal kidney 94	1.1836282	0.20718
fetal lung 113	1.1431476	0.20866
fetal lung 125	1.1557224	0.20910
fetal renal pelvis 224	1.1683270	0.20938
fetal heart 49	1.1650998	0.21048
fetal renal cortex 215	1.1643390	0.21214
fetal renal cortex 214	1.1680494	0.21298
fetal kidney 103	1.1693443	0.21516
fetal renal cortex 208	1.1729699	0.21628
fetal lung 138	1.1501608	0.21730
fetal muscle 192	1.1583669	0.21778
fetal muscle 145	1.1754168	0.21804
fetal brain 37	1.1440923	0.22066
fetal lung 130	1.1408225	0.22134
fetal muscle 186	1.1571297	0.22136
fetal muscle 163	1.1619541	0.22208
fetal lung 126	1.1340098	0.22526
fetal renal pelvis 225	1.1664300	0.22552
fetal kidney 97	1.1620858	0.22852
fetal lung 135	1.1404903	0.23024
fetal stomach 251	1.1651171	0.23092
fetal muscle 193	1.1547972	0.23110
fetal muscle 147	1.1562607	0.23136
fetal stomach 250	1.1730816	0.23182
fetal renal pelvis 232	1.1629583	0.23308
fetal kidney 96	1.1501685	0.23406
breast 2	1.1278466	0.23456
fetal stomach 246	1.1708702	0.23496
fetal muscle 174	1.1512265	0.23578
fetal lung 115	1.1308917	0.23662
fetal renal cortex 210	1.1552853	0.23860
fetal kidney 102	1.1595724	0.23912
fetal lung 137	1.1296739	0.24096
fetal muscle 144	1.1508866	0.24136
fetal stomach 249	1.1683152	0.24152
fetal lung 109	1.1154382	0.24356
fetal lung 112	1.1252903	0.25246
fetal renal pelvis 222	1.1460885	0.25332
fetal stomach 240	1.1530575	0.25554
fetal brain 32	1.1143918	0.25616
fetal large intestine 57	1.1225861	0.26068
fetal kidney 90	1.1356611	0.26200
fetal small intestine 71	1.1298609	0.26318
fetal kidney 104	1.1331207	0.26398
fetal renal pelvis 230	1.1417587	0.26454
fetal adrenal gland 27	1.1230333	0.26560
fetal kidney 81	1.1215620	0.26680
fetal heart 45	1.1264929	0.26842
fetal lung 136	1.1132678	0.27094
fetal renal cortex 203	1.1270437	0.27198
fetal lung 124	1.1094095	0.27226
fetal muscle 188	1.1218267	0.27296
fetal kidney 87	1.1227901	0.27632
fetal brain 31	1.0933610	0.27664
fetal small intestine 72	1.1188619	0.27688
ES cells 294	1.1271515	0.27960
CD20 8	1.1108232	0.28368
fetal small intestine 78	1.1076783	0.28376
fetal small intestine 69	1.1185399	0.28710
fetal stomach 241	1.1261799	0.28742
fetal muscle 191	1.1136434	0.28754
placenta 199	1.1108925	0.28782
stomach 284	1.1245799	0.28824
fetal lung 127	1.1038632	0.28916
muscle 341	1.1259403	0.29036
fetal stomach 253	1.1244558	0.29262
fetal stomach 252	1.1157012	0.29350
fetal large intestine 63	1.1110745	0.29558
fetal renal pelvis 223	1.1140074	0.29562
placenta 197	1.1096174	0.29868
fetal adrenal gland 24	1.1107662	0.30170
fetal adrenal gland 23	1.0948993	0.30256
fetal heart 48	1.1031458	0.30396
fetal adrenal gland 26	1.1010011	0.30560
fetal muscle 177	1.1058422	0.30600
fetal large intestine 54	1.1000230	0.30744
fetal kidney 93	1.1038956	0.30976
fetal kidney 82	1.1067956	0.31192
fetal stomach 242	1.1194369	0.31198
fetal stomach 245	1.1064861	0.31582
fetal adrenal gland 25	1.0962574	0.31764
fetal renal pelvis 233	1.0964924	0.31948
fetal spinal cord 239	1.0845280	0.32152
fetal testes 254	1.0901652	0.32504
fetal large intestine 53	1.0991084	0.32512
fetal brain 34	1.0763099	0.32560
fetal stomach 247	1.1047832	0.32700
stomach 283	1.0970127	0.32714
fetal small intestine 68	1.0823570	0.33164
fetal spinal cord 236	1.0825659	0.33320
placenta 201	1.0847129	0.33522
iPS 304	1.0753322	0.33652
fetal renal cortex 211	1.0855290	0.33966
fetal lung 139	1.0744740	0.34096
fetal lung 122	1.0742853	0.34396
ES derived trophoblast 287	1.0839904	0.34602
fetal small intestine 80	1.0738421	0.34950
fetal renal pelvis 216	1.0773009	0.34990
fetal small intestine 73	1.0744426	0.35106
fetal renal pelvis 219	1.0839808	0.35206
fetal small intestine 70	1.0678427	0.35464
small intestine 342	1.0724695	0.35506
fetal spinal cord 238	1.0659471	0.35564
fetal large intestine 55	1.0672832	0.35928
fetal large intestine 58	1.0673332	0.36052
fetal brain 40	1.0594128	0.36224
fetal brain 36	1.0610856	0.36338
fetal small intestine 77	1.0665862	0.36674
fetal large intestine 66	1.0643567	0.36908
fetal heart 51	1.0596974	0.37580
fetal heart 52	1.0569097	0.37772
fetal heart 46	1.0591316	0.37780
fetal renal pelvis 218	1.0641417	0.38170
fetal large intestine 64	1.0525306	0.38706
fetal kidney 86	1.0573710	0.38966
fetal heart 44	1.0555750	0.39076
placenta 196	1.0499666	0.39154
fetal ovary 195	1.0528400	0.39528
ES derived trophoblast 288	1.0441017	0.40188
fetal large intestine 59	1.0449038	0.40296
iPS 303	1.0377135	0.40794
ES derived NP 292	1.0411635	0.41080
fetal heart 42	1.0354253	0.41922
fetal heart 50	1.0352569	0.42166
fetal brain 35	1.0217248	0.43682
fetal small intestine 74	1.0251579	0.43884
ES derived NP 291	1.0195614	0.45118
ES cells 296	1.0175135	0.45124
fetal small intestine 76	1.0161739	0.45524
pancreas 328	1.0088310	0.46714
fetal large intestine 56	1.0083730	0.47088
fetal brain 30	1.0031673	0.48108
ES cells 293	0.9947581	0.49180
fetal brain 38	0.9986365	0.49370
heart 297	0.9930463	0.49602
fetal large intestine 60	0.9942492	0.49670
fetal brain 29	0.9956895	0.49722
ES cells 295	0.9891288	0.50216
fetal large intestine 65	0.9882840	0.50584
iPS 302	0.9884807	0.50984
fetal small intestine 79	0.9842454	0.50988
fetal heart 43	0.9823403	0.52082
fetal spinal cord 237	0.9680071	0.54504
pancreas 327	0.9586244	0.54880
fetal large intestine 62	0.9610816	0.55054
fetal brain 39	0.9655694	0.55808
fetal brain 41	0.9582281	0.57258

Type 2 diabetes 2

DHS sample	fold enrichment	p value
fetal fibroblast 269	1.3523322	0.00508
fetal fibroblast 280	1.3644083	0.00560
ES derived MSC 290	1.4135292	0.00564
fetal fibroblast 276	1.3427559	0.00614
fetal fibroblast 275	1.3448893	0.00620
fetal fibroblast 277	1.3611019	0.00622
fetal fibroblast 270	1.3419021	0.00830
fibroblast 330	1.3516384	0.00832
fibroblast 331	1.3405342	0.00832
fetal fibroblast 282	1.3189486	0.00864
fetal fibroblast 278	1.3387189	0.00906
fetal fibroblast 273	1.3210442	0.00914
fetal lung 300	1.3523160	0.00974
fetal fibroblast 279	1.3196925	0.01186
fibroblast 332	1.3210301	0.01212
fetal fibroblast 268	1.3099933	0.01220
fetal lung 298	1.3496479	0.01272
fetal fibroblast 274	1.3095602	0.01414
ES derived MSC 289	1.3596507	0.01448
fibroblast 329	1.3055651	0.01502
fetal fibroblast 272	1.2968503	0.01670
fetal lung 301	1.3177243	0.01836
fetal lung 299	1.3271138	0.01872
fetal heart 47	1.3681139	0.02006
fetal fibroblast 281	1.2764880	0.02184
fetal fibroblast 271	1.2742208	0.02354
fetal heart 49	1.3276531	0.02444
fetal fibroblast 267	1.2780872	0.02634
fetal heart 48	1.3047788	0.03082
fetal heart 51	1.2973711	0.03116
fetal heart 44	1.3727831	0.03378
fetal heart 46	1.3123487	0.03386
fetal heart 43	1.2720836	0.03856
fetal heart 42	1.2791576	0.04508
ES derived mesoderm 286	1.2028662	0.04910
fetal heart 50	1.2693174	0.05268
fetal heart 45	1.2655203	0.05376
fetal small intestine 77	1.2813974	0.05492
melanocyte 337	1.1842575	0.05692
fetal large intestine 67	1.2722853	0.06098
hematopoietic stem cell 310	1.2148337	0.06604
fetal heart 52	1.2205388	0.06792
breast 2	1.1869325	0.07290
stomach 284	1.2723375	0.07574
melanocyte 338	1.1694365	0.07632
fetal small intestine 80	1.2282868	0.07816
fetal small intestine 70	1.2115292	0.07898
melanocyte 339	1.1719007	0.08434
fetal thymus 256	1.2583154	0.08620
keratinocyte 336	1.1604118	0.08658
ES derived trophoblast 288	1.1973426	0.08778
fetal large intestine 58	1.2055034	0.09098
CD14 4	1.2550586	0.09314
melanocyte 340	1.1720954	0.09816
fetal small intestine 78	1.1952799	0.09840
fetal muscle 172	1.1805540	0.09898
keratinocyte 333	1.1435556	0.10186
fetal large intestine 64	1.2082274	0.10252
keratinocyte 334	1.1416029	0.10986
fetal thymus 258	1.2368412	0.11024
fetal large intestine 60	1.2038544	0.11280
CD20 8	1.1910538	0.11474
fetal small intestine 76	1.2174545	0.11580
fetal large intestine 53	1.2217848	0.11740
breast 1	1.1516085	0.11908
keratinocyte 335	1.1373799	0.11960
fetal large intestine 62	1.2099698	0.12090
fetal thymus 264	1.2214266	0.12160
fetal large intestine 57	1.1772986	0.12498
fetal large intestine 55	1.1794589	0.12898
fetal large intestine 66	1.1967333	0.13066
fetal stomach 244	1.1908876	0.13230
fetal small intestine 79	1.1982955	0.13402
fetal small intestine 74	1.1776496	0.13788
fetal large intestine 65	1.1843299	0.13876
fetal thymus 265	1.2043810	0.14190
hematopoietic stem cell 314	1.1584615	0.14224
hematopoietic stem cell 309	1.1671225	0.14316
fetal small intestine 71	1.1782110	0.14530
hematopoietic stem cell 311	1.1598190	0.14598
fetal large intestine 59	1.1819523	0.14786
fetal lung 109	1.1269722	0.15010
hematopoietic stem cell 313	1.1507194	0.15382
hematopoietic stem cell 317	1.1400113	0.15568
fetal small intestine 72	1.1653664	0.15640
fetal large intestine 54	1.1673183	0.15698
small intestine 342	1.1668351	0.15704
pancreas 327	1.1833494	0.15920
CD14 3	1.2004034	0.15950
stomach 283	1.1830461	0.15960
fetal thymus 259	1.2017665	0.16096
fetal thymus 261	1.1910919	0.16344
fetal large intestine 63	1.1617195	0.17514
fetal small intestine 68	1.1421114	0.18194
fetal small intestine 73	1.1495236	0.18610
fetal small intestine 69	1.1527119	0.18906
fetal large intestine 56	1.1407994	0.19088
fetal thymus 266	1.1535363	0.20486
fetal lung 142	1.1031462	0.20870
muscle 341	1.1430220	0.21506
fetal stomach 246	1.1371313	0.21942
fetal renal pelvis 231	1.1076788	0.22444
ES derived mesoderm 285	1.0979598	0.22474
fetal skin 234	1.1098373	0.22518
hematopoietic stem cell 306	1.1111701	0.23060
fetal stomach 251	1.1214084	0.23076
heart 297	1.1243946	0.23270
hematopoietic stem cell 308	1.1063232	0.23316
fetal stomach 247	1.1399848	0.23540
ES derived trophoblast 287	1.1132257	0.23756
hematopoietic stem cell 307	1.1054732	0.24118
fetal lung 141	1.0886721	0.24156
fetal stomach 245	1.1190980	0.24250
fetal thymus 260	1.1233413	0.24934
fetal stomach 250	1.1168207	0.25006
hematopoietic stem cell 315	1.1036302	0.25144
fetal thymus 262	1.1387575	0.25622
fetal stomach 252	1.1065271	0.25816
fetal stomach 249	1.1115276	0.26174
fetal lung 134	1.0880098	0.26220
fetal renal cortex 206	1.0855529	0.27428
fetal testes 255	1.0830593	0.27586
fetal renal cortex 215	1.0807701	0.28202
fetal stomach 248	1.0957012	0.28224
fetal stomach 253	1.0980617	0.28354
fetal lung 133	1.0719980	0.28436
ovary 326	1.1060390	0.28470
fetal lung 111	1.0727563	0.28936
fetal adrenal gland 23	1.0767825	0.29012
fetal kidney 101	1.0785792	0.29286
fetal renal pelvis 229	1.0752800	0.29774
fetal kidney 105	1.0721988	0.30154
fetal lung 113	1.0655665	0.30216
fetal renal cortex 214	1.0727587	0.30264
hematopoietic stem cell 316	1.0776040	0.30762
fetal renal pelvis 221	1.0661957	0.31476
fetal kidney 91	1.0640794	0.31478
fetal renal cortex 212	1.0644354	0.31616
fetal lung 131	1.0565868	0.31634
fetal thymus 257	1.0870098	0.31806
fetal kidney 98	1.0678860	0.31820
fetal lung 129	1.0620527	0.31888
fetal renal pelvis 225	1.0695812	0.31946
fetal kidney 95	1.0666102	0.31954
fetal renal pelvis 224	1.0620903	0.31954
fetal lung 128	1.0562548	0.32042
fetal stomach 243	1.0706207	0.32098
fetal lung 122	1.0636619	0.32318
fetal lung 114	1.0567218	0.32334
pancreas 328	1.0729518	0.32640
fetal lung 126	1.0543785	0.32870
fetal renal pelvis 217	1.0634940	0.33102
fetal renal pelvis 228	1.0649802	0.33242
fetal adrenal gland 25	1.0656953	0.33254
fetal kidney 97	1.0598211	0.33310
fetal kidney 100	1.0581002	0.33946
fetal renal cortex 203	1.0589872	0.33954
fetal kidney 82	1.0621430	0.34188
fetal lung 110	1.0541735	0.34194
fetal testes 254	1.0585224	0.34238
fetal renal cortex 208	1.0586514	0.34328
fetal kidney 104	1.0566078	0.34398
hematopoietic stem cell 305	1.0584457	0.34470
fetal kidney 102	1.0591908	0.34472
hematopoietic stem cell 312	1.0577773	0.34540
fetal kidney 90	1.0558397	0.34588
fetal renal cortex 204	1.0540361	0.34618
fetal kidney 84	1.0561632	0.34720
fetal renal pelvis 220	1.0548506	0.34724
fetal lung 130	1.0503053	0.34730
fetal lung 125	1.0513825	0.34876
CD3 10	1.0707204	0.35018
fetal lung 139	1.0503604	0.35034
fetal kidney 85	1.0462949	0.35264
fetal stomach 242	1.0658847	0.35476
fetal renal pelvis 227	1.0514276	0.35494
fetal lung 138	1.0477890	0.35498
fetal lung 132	1.0483830	0.35540
fetal adrenal gland 27	1.0519560	0.35752
fetal kidney 106	1.0486422	0.35912
fetal kidney 88	1.0472754	0.36048
fetal kidney 96	1.0463094	0.36132
hematopoietic stem cell 319	1.0523112	0.36224
fetal renal pelvis 216	1.0479121	0.36522
fetal kidney 103	1.0465039	0.36612
fetal lung 112	1.0416877	0.36928
fetal renal pelvis 219	1.0528081	0.37222
fetal kidney 87	1.0444384	0.37286
fetal kidney 89	1.0444113	0.37358
fetal lung 137	1.0390226	0.37506
fetal lung 136	1.0405296	0.37578
fetal renal pelvis 232	1.0435595	0.37600
fetal adrenal gland 28	1.0385520	0.37800
fetal stomach 240	1.0468364	0.38246
fetal lung 127	1.0364223	0.38642
hematopoietic stem cell 318	1.0402788	0.38966
fetal kidney 92	1.0343350	0.38972
fetal lung 120	1.0343925	0.39502
fetal kidney 107	1.0331633	0.40010
fetal muscle 153	1.0343179	0.40240
fetal stomach 241	1.0350680	0.40348
fetal muscle 167	1.0307222	0.40578
fetal kidney 94	1.0315456	0.40696
fetal renal pelvis 233	1.0315738	0.40750
fetal renal pelvis 222	1.0297412	0.40924
fetal renal cortex 213	1.0272922	0.41168
fetal renal pelvis 230	1.0285179	0.41422
fetal kidney 86	1.0304915	0.41598
fetal kidney 99	1.0256538	0.41740
CD19 5	1.0329052	0.42036
fetal lung 115	1.0238962	0.42042
fetal muscle 157	1.0250562	0.42224
CD19 6	1.0315395	0.42322
CD56 17	1.0273364	0.42716
fetal muscle 146	1.0221173	0.43048
fetal kidney 93	1.0205006	0.43076
fetal renal cortex 211	1.0186894	0.43590
fetal renal cortex 209	1.0178676	0.43838
fetal renal cortex 205	1.0172699	0.44142
fetal renal cortex 202	1.0162423	0.44170
fetal lung 118	1.0153367	0.44258
fetal renal cortex 210	1.0137736	0.44680
fetal renal pelvis 218	1.0162912	0.44744
fetal renal cortex 207	1.0128877	0.44802
fetal lung 121	1.0137249	0.44968
fetal lung 119	1.0137517	0.45120
fetal kidney 83	1.0131101	0.45238
CD19 7	1.0142123	0.45538
fetal renal pelvis 223	1.0115737	0.45554
fetal muscle 148	1.0119325	0.45646
placenta 201	1.0120771	0.45940
fetal lung 135	1.0098495	0.46012
fetal renal pelvis 226	1.0094976	0.46012
fetal muscle 168	1.0106420	0.46086
fetal kidney 81	1.0067899	0.46672
fetal lung 117	1.0076167	0.46820
fetal muscle 192	1.0061993	0.47036
fetal lung 124	1.0049820	0.47212
fetal muscle 165	1.0046351	0.47500
fetal muscle 193	1.0028141	0.47870
fetal muscle 150	1.0003262	0.48518
fetal muscle 152	1.0014904	0.48640
fetal muscle 160	0.9996128	0.48676
fetal lung 123	0.9997991	0.48776
fetal muscle 169	0.9996732	0.48962
fetal lung 116	0.9989508	0.49406
fetal thymus 263	0.9957888	0.49590
iPS 303	0.9967201	0.49666
fetal muscle 173	0.9968018	0.49684
fetal muscle 154	0.9949825	0.50050
fetal ovary 195	0.9890073	0.50288
fetal muscle 179	0.9932823	0.50776
fetal lung 140	0.9942353	0.50850
fetal muscle 183	0.9917020	0.51256
fetal lung 108	0.9891436	0.51456
fetal muscle 191	0.9897835	0.51542
fetal muscle 155	0.9863149	0.51830
fetal muscle 186	0.9886936	0.51852
fetal muscle 164	0.9885872	0.51866
fetal muscle 176	0.9896343	0.52014
fetal muscle 178	0.9855640	0.52244
fetal muscle 177	0.9848906	0.52358
placenta 200	0.9884216	0.52556
fetal muscle 151	0.9828502	0.52726
fetal muscle 143	0.9863251	0.52820
iPS 302	0.9841094	0.52820
fetal muscle 156	0.9846215	0.52858
fetal muscle 187	0.9840726	0.53018
fetal muscle 175	0.9844032	0.53038
placenta 199	0.9842511	0.53218
fetal muscle 180	0.9807137	0.53610
placenta 196	0.9807613	0.53982
fetal muscle 170	0.9814246	0.54004
fetal muscle 188	0.9798577	0.54248
fetal muscle 147	0.9786216	0.54270
fetal muscle 149	0.9776004	0.54462
placenta 198	0.9799751	0.54484
ES derived NP 291	0.9725672	0.54796
fetal muscle 184	0.9795569	0.54858
fetal muscle 182	0.9775727	0.54878
fetal muscle 159	0.9746418	0.55010
fetal muscle 161	0.9754557	0.55102
fetal muscle 163	0.9727990	0.55594
fetal muscle 162	0.9751371	0.55864
ES derived NP 292	0.9678149	0.56194
fetal muscle 145	0.9668884	0.56194
fetal muscle 181	0.9692183	0.56836
fetal adrenal gland 26	0.9693175	0.56924
Mobilized CD8 325	0.9481197	0.58048
iPS 304	0.9652915	0.58828
fetal muscle 189	0.9626981	0.58952
fetal muscle 166	0.9587331	0.59782
placenta 197	0.9564558	0.59948
fetal spinal cord 237	0.9615734	0.60392
fetal muscle 171	0.9582799	0.60502
Mobilized CD4 323	0.9349192	0.60604
CD4 14	0.9419349	0.60770
Mobilized CD3 320	0.9322569	0.61004
ES cells 294	0.9453117	0.61234
fetal muscle 144	0.9467990	0.61658
fetal muscle 158	0.9537727	0.61756
CD3 11	0.9350429	0.61888
fetal muscle 185	0.9465577	0.62006
fetal adrenal gland 24	0.9374855	0.62736
fetal muscle 174	0.9459830	0.62866
fetal muscle 190	0.9393998	0.63422
fetal muscle 194	0.9460592	0.64612
CD4 13	0.9114388	0.64984
ES cells 295	0.9280858	0.65056
ES cells 296	0.9226793	0.66168
fetal spinal cord 235	0.9323883	0.67504
fetal spinal cord 238	0.9288744	0.68712
CD8 20	0.8997950	0.68744
CD4 16	0.8876721	0.68784
fetal spinal cord 239	0.9234601	0.70020
fetal small intestine 75	0.8665094	0.70020
CD56 18	0.8806275	0.70550
CD3 12	0.8765485	0.70602
CD8 22	0.8780897	0.70942
CD8 19	0.8653755	0.71126
fetal brain 34	0.9357300	0.71656
CD8 21	0.8620386	0.73366
Mobilized CD3 321	0.8550673	0.73700
fetal large intestine 61	0.8709984	0.73914
fetal spinal cord 236	0.8968525	0.75452
ES cells 293	0.8799356	0.76660
fetal brain 35	0.9224065	0.76932
Mobilized CD56 324	0.8526612	0.77430
Mobilized CD4 322	0.8276116	0.78172
fetal brain 41	0.9089685	0.78220
CD4 15	0.8025235	0.78672
fetal brain 38	0.9011444	0.79122
fetal brain 36	0.9009917	0.79860
CD3 9	0.8262268	0.80120
fetal brain 39	0.9034984	0.80520
fetal brain 30	0.9126829	0.82460
fetal brain 32	0.8853803	0.83106
fetal brain 33	0.8684835	0.84288
fetal brain 37	0.8786497	0.85480
fetal brain 29	0.8975551	0.86062
fetal brain 31	0.8920408	0.86846
fetal brain 40	0.8389022	0.93818

Pulmonary function

DHS sample	fold enrichment	p value
fetal lung 111	1.5788195	0.00082
fetal lung 129	1.5503618	0.00164
fetal lung 118	1.4996030	0.00176
fetal lung 119	1.5065641	0.00196
fetal heart 47	1.7450893	0.00198
fetal lung 141	1.4923154	0.00230
fetal lung 114	1.4857926	0.00282
fetal heart 45	1.6263818	0.00294
fetal lung 121	1.4759301	0.00346
fetal lung 117	1.4885266	0.00374
fetal lung 128	1.4261615	0.00408
fetal lung 140	1.4837578	0.00426
fetal lung 133	1.4607719	0.00450
fetal heart 49	1.5907304	0.00516
fetal lung 116	1.4002626	0.00794
fetal lung 131	1.3921143	0.00836
fetal muscle 194	1.4172668	0.00984
fetal lung 120	1.4215583	0.01006
fetal heart 46	1.5363003	0.01036
fetal heart 43	1.4809834	0.01150
fetal lung 134	1.4262799	0.01208
fetal lung 110	1.4360574	0.01246
fetal heart 48	1.4847219	0.01296
fetal heart 42	1.4804111	0.01426
fetal lung 115	1.3729768	0.01486
fetal lung 139	1.4146269	0.01522
fetal heart 44	1.5713105	0.01610
fetal lung 113	1.3618031	0.01632
fetal lung 108	1.4102490	0.01940
fetal lung 138	1.3891340	0.01990
fetal lung 132	1.3739768	0.02000
fetal heart 51	1.4399763	0.02010
fetal lung 130	1.3740753	0.02080
fetal muscle 176	1.3815572	0.02208
fetal lung 136	1.3728271	0.02240
fetal lung 126	1.3359470	0.02304
fetal lung 125	1.3801372	0.02442
fetal muscle 160	1.3874768	0.02846
fetal heart 52	1.3782563	0.02888
fetal lung 142	1.3166350	0.02960
fetal fibroblast 281	1.3089975	0.03052
fetal muscle 152	1.3575949	0.03144
fetal fibroblast 282	1.2876225	0.03380
fetal lung 124	1.3313700	0.03434
heart 297	1.4464904	0.03488
fetal heart 50	1.3819537	0.03544
fetal lung 112	1.3289621	0.03712
fetal muscle 167	1.3439811	0.03744
fetal lung 135	1.3252499	0.03804
fetal muscle 179	1.3481071	0.03910
fetal lung 137	1.3064501	0.03982
fetal lung 123	1.3181365	0.04324
fetal muscle 183	1.3137141	0.04408
fetal lung 109	1.2642489	0.04710
fetal muscle 151	1.3593937	0.04772
fetal muscle 164	1.3256015	0.05146
fetal muscle 169	1.3221055	0.05310
fetal muscle 166	1.3101347	0.05364
muscle 341	1.3954839	0.05618
fetal muscle 187	1.2913906	0.05774
fetal muscle 148	1.3138542	0.05824
fetal fibroblast 268	1.2415886	0.06436
fetal muscle 181	1.2964789	0.06500
fetal muscle 182	1.2878232	0.06554
fetal muscle 156	1.2853648	0.06640
fetal muscle 147	1.2899065	0.06998
fetal muscle 188	1.2728259	0.07052
fetal muscle 143	1.2666318	0.07206
fetal muscle 146	1.2733323	0.07376
fetal muscle 149	1.3043544	0.07396
fetal muscle 172	1.2451135	0.07630
fetal muscle 190	1.2793023	0.07904
fetal muscle 186	1.2578331	0.08304
pancreas 327	1.3355628	0.08470
fetal muscle 157	1.2464123	0.08476
fetal lung 127	1.2540097	0.08702
fetal fibroblast 273	1.2153123	0.08794
fetal muscle 180	1.2731348	0.08832
fetal muscle 150	1.2714812	0.08836
fetal lung 122	1.2518258	0.08914
fetal fibroblast 274	1.2177520	0.08934
fetal muscle 154	1.2841844	0.08982
fetal muscle 189	1.2525264	0.08990
fetal muscle 175	1.2525271	0.09090
fetal fibroblast 267	1.2242115	0.09230
fetal muscle 192	1.2495113	0.09246
fetal muscle 153	1.2708793	0.09656
fetal muscle 171	1.2425258	0.09674
fetal muscle 163	1.2527537	0.09686
fetal muscle 161	1.2484957	0.09826
fetal muscle 155	1.2611937	0.10340
fetal muscle 159	1.2556441	0.10478
fetal muscle 184	1.2221057	0.10496
fetal kidney 81	1.2286908	0.10518
fetal muscle 162	1.2265170	0.10798
fetal fibroblast 275	1.1906618	0.10998
fetal muscle 173	1.2340239	0.11008
fetal muscle 165	1.2197213	0.11284
fetal adrenal gland 28	1.2152748	0.11390
fetal muscle 144	1.2385534	0.11420
fetal fibroblast 277	1.1964571	0.11634
fetal muscle 158	1.2173252	0.11726
fetal muscle 191	1.2258812	0.11736
fetal muscle 177	1.2297301	0.12056
fetal fibroblast 276	1.1819155	0.12286
fetal fibroblast 269	1.1858472	0.12622
pancreas 328	1.2623075	0.12784
fetal muscle 170	1.1954412	0.13694
fetal muscle 193	1.2112740	0.13840
fetal muscle 178	1.2111739	0.13874
fetal muscle 168	1.2115818	0.14134
fetal muscle 185	1.2128822	0.14172
fetal fibroblast 271	1.1687240	0.14482
ES derived MSC 290	1.2009305	0.14764
placenta 196	1.2109945	0.15182
fetal fibroblast 270	1.1718360	0.15206
fetal fibroblast 280	1.1653303	0.15420
fetal muscle 145	1.2051617	0.15690
ovary 326	1.2520379	0.16044
ES derived MSC 289	1.1894731	0.16214
placenta 199	1.1913121	0.16236
fetal large intestine 53	1.2252281	0.16712
fetal fibroblast 278	1.1599424	0.16992
fetal adrenal gland 26	1.1850015	0.17032
fetal spinal cord 235	1.1724454	0.17430
fetal muscle 174	1.1757456	0.17518
fetal lung 299	1.1680883	0.18330
fetal renal cortex 213	1.1731716	0.18690
fetal stomach 243	1.1754154	0.18802
fetal stomach 248	1.1988826	0.18826
fetal kidney 91	1.1643880	0.18838
placenta 200	1.1600270	0.18884
fetal fibroblast 279	1.1427856	0.19010
fetal large intestine 57	1.1698090	0.19876
CD3 10	1.2153035	0.19922
fetal spinal cord 236	1.1595709	0.20138
breast 1	1.1391528	0.20272
placenta 197	1.1665736	0.20442
CD19 7	1.2269537	0.20518
fetal testes 255	1.1522455	0.20826
fetal stomach 244	1.1750902	0.20892
placenta 198	1.1513317	0.21002
fetal small intestine 74	1.1713110	0.21126
fetal fibroblast 272	1.1238230	0.21508
fetal adrenal gland 27	1.1520530	0.21624
fetal large intestine 67	1.1707914	0.21702
fetal thymus 263	1.1473208	0.22100
fetal renal pelvis 226	1.1474210	0.22404
fetal kidney 87	1.1392111	0.23380
fetal kidney 100	1.1378562	0.23548
fetal renal pelvis 220	1.1401332	0.23938
fetal lung 300	1.1251455	0.24074
fetal lung 298	1.1295048	0.24110
fibroblast 332	1.1174809	0.24300
fetal small intestine 70	1.1331481	0.24318
keratinocyte 334	1.1033650	0.24738
CD19 5	1.1753223	0.24940
stomach 283	1.1459417	0.25128
fetal stomach 251	1.1362761	0.25364
breast 2	1.1076779	0.25866
fetal stomach 245	1.1346299	0.25928
fetal renal cortex 205	1.1232972	0.26016
fetal lung 301	1.1154664	0.26040
fetal small intestine 78	1.1172391	0.26732
fetal large intestine 56	1.1256743	0.26744
placenta 201	1.1251269	0.26810
fetal adrenal gland 25	1.1237858	0.26944
fetal kidney 92	1.1147790	0.26950
fetal stomach 246	1.1330008	0.26990
fetal large intestine 60	1.1290569	0.26994
CD19 6	1.1546116	0.27556
fetal renal pelvis 217	1.1155986	0.27674
fetal renal pelvis 232	1.1152251	0.27692
fetal stomach 253	1.1274856	0.27760
fetal large intestine 58	1.1145318	0.27916
fetal large intestine 55	1.1123277	0.28412
fetal spinal cord 238	1.0988142	0.28586
fetal small intestine 68	1.1119224	0.28654
fetal kidney 88	1.1009123	0.28796
fetal kidney 82	1.1108527	0.28868
fetal testes 254	1.1029138	0.29212
fetal kidney 106	1.0998694	0.29250
fetal renal cortex 207	1.1009817	0.29648
fetal stomach 250	1.1105808	0.30000
fetal adrenal gland 24	1.1061952	0.30112
CD20 8	1.1000185	0.30592
fetal large intestine 64	1.0991006	0.30788
fetal renal cortex 203	1.0929700	0.30974
fetal large intestine 62	1.1071184	0.30978
fetal large intestine 66	1.1007707	0.31200
fetal small intestine 73	1.0993354	0.31250
fetal stomach 242	1.1046701	0.32102
fetal adrenal gland 23	1.0789502	0.32112
fetal renal pelvis 221	1.0813357	0.32278
keratinocyte 336	1.0702212	0.32282
ES derived trophoblast 288	1.0797786	0.32376
fetal small intestine 80	1.0902609	0.32384
fetal small intestine 72	1.0892339	0.32560
fetal renal cortex 202	1.0809554	0.32676
fetal kidney 83	1.0888573	0.32972
fetal renal cortex 206	1.0760588	0.33152
fetal stomach 249	1.0887652	0.33430
ES derived trophoblast 287	1.0805924	0.33626
stomach 284	1.0830565	0.33834
keratinocyte 333	1.0611026	0.34056
fetal spinal cord 237	1.0670523	0.34242
fetal small intestine 77	1.0814474	0.34298
fetal kidney 99	1.0683307	0.35022
fetal renal pelvis 219	1.0820279	0.35052
fetal kidney 89	1.0691475	0.35114
keratinocyte 335	1.0554145	0.35716
fetal ovary 195	1.0678647	0.36346
fetal kidney 97	1.0604442	0.36578
fetal renal pelvis 228	1.0618869	0.37130
fetal kidney 104	1.0574616	0.37226
fetal kidney 85	1.0516829	0.37262
fetal renal cortex 211	1.0569691	0.37316
fetal kidney 105	1.0541451	0.37444
fetal renal pelvis 227	1.0557249	0.37578
CD14 3	1.0692167	0.37598
fetal large intestine 59	1.0616635	0.37688
fibroblast 329	1.0512435	0.37690
fetal small intestine 76	1.0609932	0.37874
fetal small intestine 71	1.0550760	0.38092
fetal large intestine 65	1.0555987	0.38394
fibroblast 331	1.0487812	0.38460
fetal kidney 101	1.0506653	0.38472
fetal renal pelvis 223	1.0531505	0.38560
fetal kidney 86	1.0536940	0.38594
fetal renal pelvis 222	1.0510967	0.38844
small intestine 342	1.0511196	0.38940
fetal renal cortex 204	1.0469976	0.38964
fetal renal pelvis 229	1.0442252	0.39490
fetal stomach 247	1.0523530	0.39504
fetal renal pelvis 216	1.0466863	0.39606
fetal renal cortex 214	1.0451653	0.39660
fetal stomach 252	1.0431643	0.40122
fetal stomach 240	1.0461224	0.40392
fetal skin 234	1.0421802	0.40564
fetal renal pelvis 230	1.0403067	0.40590
fetal kidney 107	1.0380416	0.40992
fetal kidney 96	1.0362404	0.41070
fibroblast 330	1.0365003	0.41106
fetal stomach 241	1.0379123	0.41284
fetal kidney 102	1.0354935	0.41646
fetal kidney 103	1.0304602	0.42080
fetal renal pelvis 231	1.0307803	0.42134
fetal small intestine 79	1.0330387	0.42324
fetal renal cortex 210	1.0221997	0.44094
fetal renal pelvis 233	1.0237511	0.44214
ES derived mesoderm 286	1.0175389	0.44396
fetal large intestine 54	1.0198601	0.44470
fetal spinal cord 239	1.0199518	0.44486
fetal kidney 95	1.0167493	0.44724
fetal kidney 84	1.0172305	0.44916
CD4 16	1.0139412	0.44998
fetal kidney 90	1.0182977	0.45006
fetal kidney 94	1.0170559	0.45036
Mobilized CD56 324	1.0146125	0.45850
fetal renal cortex 209	1.0128784	0.45986
Mobilized CD4 322	1.0073062	0.46238
CD4 14	1.0088043	0.46392
melanocyte 338	1.0078346	0.47646
CD56 17	1.0023405	0.47660
CD14 4	1.0043410	0.47718
CD3 12	0.9955696	0.47860
Mobilized CD4 323	0.9955090	0.47936
fetal renal pelvis 224	1.0018720	0.47984
fetal renal cortex 208	0.9984767	0.48644
Mobilized CD3 320	0.9926440	0.48786
melanocyte 339	1.0026120	0.48886
ES derived mesoderm 285	0.9977539	0.49276
hematopoietic stem cell 307	0.9924785	0.49980
CD4 13	0.9798840	0.50218
hematopoietic stem cell 312	0.9922876	0.50656
fetal kidney 98	0.9848307	0.51418
fetal renal pelvis 218	0.9770625	0.51648
fetal small intestine 69	0.9796959	0.51668
fetal renal cortex 215	0.9823983	0.51918
Mobilized CD3 321	0.9634214	0.52096
CD8 22	0.9658600	0.52270
CD3 9	0.9664875	0.52684
fetal thymus 257	0.9690194	0.52720
fetal kidney 93	0.9753876	0.52994
fetal renal pelvis 225	0.9770522	0.53028
fetal large intestine 63	0.9706302	0.53356
melanocyte 337	0.9849938	0.53680
fetal small intestine 75	0.9377259	0.53866
fetal renal cortex 212	0.9750650	0.54210
fetal brain 33	0.9755529	0.54522
melanocyte 340	0.9788854	0.54996
CD3 11	0.9439994	0.56786
CD56 18	0.9377082	0.56810
hematopoietic stem cell 318	0.9549074	0.57136
fetal brain 37	0.9642960	0.58204
fetal brain 32	0.9614017	0.58512
fetal large intestine 61	0.9259249	0.59366
fetal thymus 259	0.9226541	0.60030
fetal thymus 262	0.8957165	0.61576
fetal thymus 265	0.9151041	0.61872
hematopoietic stem cell 319	0.9268656	0.62022
hematopoietic stem cell 311	0.9318325	0.62434
Mobilized CD8 325	0.9014238	0.62856
fetal thymus 258	0.9100442	0.63014
hematopoietic stem cell 317	0.9382362	0.63038
ES derived NP 291	0.9111862	0.63128
CD4 15	0.8728730	0.63138
hematopoietic stem cell 305	0.9286451	0.63222
fetal brain 38	0.9394688	0.63624
hematopoietic stem cell 309	0.9268824	0.63980
ES cells 294	0.9048991	0.64426
hematopoietic stem cell 316	0.9128960	0.65074
CD8 20	0.8856260	0.65906
hematopoietic stem cell 308	0.9157251	0.65946
ES derived NP 292	0.8956241	0.66476
hematopoietic stem cell 315	0.9098858	0.66514
CD8 21	0.8677877	0.66760
hematopoietic stem cell 313	0.9137974	0.66860
ES cells 293	0.8991215	0.67078
CD8 19	0.8482050	0.67766
hematopoietic stem cell 306	0.8966109	0.68938
fetal thymus 261	0.8661817	0.69648
hematopoietic stem cell 314	0.8994173	0.69796
fetal brain 35	0.9260387	0.70274
hematopoietic stem cell 310	0.9009435	0.70502
fetal brain 41	0.9151986	0.70840
fetal thymus 260	0.8570260	0.71250
iPS 303	0.8872147	0.71698
fetal thymus 264	0.8546063	0.72478
iPS 302	0.8767402	0.73210
fetal brain 34	0.9079468	0.73986
fetal thymus 266	0.8329902	0.75058
fetal thymus 256	0.8195433	0.77544
iPS 304	0.8568144	0.78012
fetal brain 39	0.8772733	0.80250
fetal brain 36	0.8540131	0.82244
fetal brain 31	0.8702561	0.84328
fetal brain 40	0.8524986	0.84918
ES cells 296	0.7639389	0.86058
fetal brain 30	0.8644634	0.86980
fetal brain 29	0.8601316	0.87000
ES cells 295	0.7533308	0.87908

Chronic lymphocytic leukemia 2

DHS sample	fold enrichment	p value
CD19 5	1.5043625	0.05362
CD14 3	1.3868046	0.08538
CD3 10	1.4007264	0.08566
CD19 6	1.3984627	0.10188
CD14 4	1.3153903	0.11598
CD19 7	1.3238752	0.14712
Mobilized CD56 324	1.2050314	0.20840
CD56 17	1.1961800	0.21664
CD8 20	1.1383265	0.28736
fetal lung 119	1.0796070	0.32536
CD3 11	1.1038484	0.32578
fetal lung 116	1.0610559	0.35844
fetal lung 128	1.0560607	0.36298
hematopoietic stem cell 310	1.0604173	0.36424
fetal lung 111	1.0603380	0.36590
fetal lung 141	1.0571165	0.36790
CD4 14	1.0465896	0.39990
fetal thymus 264	1.0259707	0.43580
fetal thymus 265	1.0273846	0.43738
fetal lung 114	1.0235160	0.43752
CD56 18	1.0181366	0.43912
fetal lung 120	1.0118296	0.45964
fetal lung 115	1.0112916	0.46080
fetal lung 126	1.0079242	0.47016
fetal lung 133	1.0058921	0.47750
CD8 22	0.9765934	0.48372
CD3 9	0.9854129	0.48490
fetal lung 140	1.0003084	0.48496
fetal lung 131	0.9986394	0.48852
CD4 16	0.9674834	0.49310
fetal lung 113	0.9894313	0.50626
CD3 12	0.9580775	0.50810
fetal lung 137	0.9836583	0.52040
Mobilized CD3 321	0.9495014	0.52076
Mobilized CD3 320	0.9575140	0.52154
fetal lung 117	0.9787702	0.53194
fetal small intestine 75	0.8885738	0.54928
fetal thymus 256	0.9559074	0.54944
hematopoietic stem cell 317	0.9671028	0.55190
Mobilized CD4 322	0.9351222	0.55208
fetal lung 132	0.9664211	0.55616
fetal thymus 266	0.9449856	0.56280
fetal lung 109	0.9675521	0.56398
fetal lung 123	0.9564090	0.56772
hematopoietic stem cell 314	0.9569870	0.57074
fetal lung 138	0.9563361	0.57252
fetal spinal cord 239	0.9503637	0.57678
fetal lung 136	0.9539044	0.57828
CD8 21	0.9153855	0.58234
hematopoietic stem cell 311	0.9299367	0.60438
fetal lung 121	0.9490396	0.60482
fetal lung 118	0.9500082	0.60488
fetal lung 108	0.9342752	0.60714
CD4 13	0.8990015	0.60772
fetal brain 32	0.9441185	0.60854
Mobilized CD4 323	0.8860156	0.61232
fetal lung 110	0.9336189	0.61440
hematopoietic stem cell 313	0.9275259	0.61668
fetal lung 124	0.9355970	0.61796
fetal lung 129	0.9356537	0.61974
fetal lung 139	0.9272330	0.62620
fetal lung 112	0.9290741	0.62830
fetal thymus 260	0.9025471	0.62956
fetal stomach 243	0.9115943	0.63476
fetal lung 122	0.9183265	0.63598
hematopoietic stem cell 306	0.9127306	0.63670
fetal thymus 258	0.8980170	0.64028
fetal lung 135	0.9236430	0.64136
hematopoietic stem cell 312	0.9191299	0.64234
fetal thymus 259	0.8833540	0.64328
fetal lung 300	0.9240147	0.64792
fetal lung 134	0.9189328	0.64872
fetal muscle 194	0.9201698	0.65284
fetal large intestine 58	0.9060368	0.66632
fetal large intestine 67	0.8935528	0.66818
fetal lung 127	0.9055348	0.66918
fetal heart 47	0.8791351	0.67360
fetal brain 37	0.9158934	0.67522
fetal stomach 244	0.8837295	0.67594
hematopoietic stem cell 318	0.8796758	0.68808
fetal small intestine 71	0.8713048	0.69912
Mobilized CD8 325	0.8245119	0.69930
fetal thymus 261	0.8470287	0.69946
fetal lung 142	0.8985757	0.70164
CD8 19	0.8101754	0.70310
hematopoietic stem cell 308	0.8831205	0.70414
fetal large intestine 61	0.8367213	0.70748
fetal small intestine 70	0.8853342	0.70838
hematopoietic stem cell 307	0.8710425	0.71314
hematopoietic stem cell 316	0.8583223	0.71752
fetal brain 33	0.8846273	0.71756
fetal spinal cord 236	0.8731867	0.72018
fetal spinal cord 235	0.8658161	0.72412
fetal lung 130	0.8852865	0.72580
fetal lung 125	0.8762149	0.72752
fetal stomach 246	0.8329144	0.73064
fetal large intestine 62	0.8429746	0.73228
hematopoietic stem cell 309	0.8630725	0.73396
fetal adrenal gland 25	0.8448041	0.74068
CD4 15	0.7348218	0.75098
fetal stomach 241	0.8173804	0.75306
hematopoietic stem cell 305	0.8457070	0.75322
fetal small intestine 72	0.8398082	0.75358
fetal thymus 257	0.7997130	0.75398
fetal lung 301	0.8677276	0.75460
fetal thymus 262	0.7621867	0.76324
fetal small intestine 73	0.8300609	0.76334
ovary 326	0.7981660	0.76500
fetal stomach 248	0.8225593	0.76514
fetal spinal cord 238	0.8522090	0.76588
stomach 284	0.8173649	0.77202
fetal small intestine 69	0.8144856	0.77280
fetal small intestine 78	0.8437652	0.77452
ES derived MSC 289	0.8511676	0.77576
fetal muscle 182	0.8396856	0.77580
small intestine 342	0.8245457	0.78276
fetal small intestine 68	0.8300331	0.78298
hematopoietic stem cell 319	0.8099829	0.78576
fetal stomach 251	0.8070262	0.79138
fetal small intestine 77	0.8104702	0.79208
fetal renal cortex 210	0.7973233	0.79406
hematopoietic stem cell 315	0.8220711	0.79490
fetal brain 34	0.8610962	0.79666
fetal spinal cord 237	0.8399216	0.79860
fetal stomach 252	0.7966940	0.80010
fetal stomach 242	0.7529263	0.80148
CD20 8	0.8206023	0.80182
fetal muscle 149	0.8085435	0.80528
ES derived MSC 290	0.8308446	0.80680
fetal renal pelvis 230	0.7836321	0.80890
fetal stomach 245	0.7813897	0.81456
fetal stomach 247	0.7398518	0.81528
fetal stomach 249	0.7668445	0.81560
fetal muscle 181	0.8040698	0.81598
fetal renal cortex 209	0.7860495	0.81672
fetal renal pelvis 223	0.7763135	0.81684
fetal stomach 253	0.7708055	0.81824
heart 297	0.7838020	0.81828
fetal lung 299	0.8240838	0.81902
fetal large intestine 60	0.7983915	0.82032
fetal renal pelvis 217	0.7838501	0.82194
fetal kidney 86	0.7493126	0.82262
fetal small intestine 74	0.7985023	0.82276
fetal large intestine 53	0.7678359	0.82382
fetal muscle 156	0.8088516	0.82452
fetal adrenal gland 28	0.8152077	0.82548
fetal renal pelvis 221	0.7898089	0.82714
fetal large intestine 65	0.7790624	0.82744
fetal renal pelvis 218	0.7413950	0.82788
fibroblast 331	0.8335791	0.83022
fetal renal pelvis 225	0.7724696	0.83086
fetal large intestine 63	0.7773821	0.83134
fetal muscle 179	0.7916535	0.83354
fetal renal cortex 202	0.7875521	0.83362
fetal large intestine 59	0.7741422	0.83432
fetal muscle 168	0.7913085	0.83440
stomach 283	0.7694389	0.83444
fetal heart 49	0.7805513	0.83494
fetal brain 40	0.8393182	0.83506
fetal large intestine 57	0.7988327	0.83598
fetal small intestine 76	0.7650618	0.83664
fetal heart 52	0.7986045	0.83762
fetal renal cortex 204	0.7845986	0.83784
fetal renal pelvis 219	0.7343299	0.83790
fetal heart 43	0.7946057	0.83836
fetal kidney 98	0.7718514	0.83860
fetal heart 42	0.7852659	0.83874
fetal brain 38	0.8179700	0.83936
fetal stomach 250	0.7498095	0.83946
fetal large intestine 64	0.7890215	0.83948
fetal adrenal gland 24	0.7609296	0.83998
fetal muscle 166	0.7946600	0.84072
fetal muscle 190	0.7753276	0.84234
fetal renal cortex 208	0.7662236	0.84382
fetal adrenal gland 23	0.7915035	0.84668
fetal muscle 151	0.7746772	0.84774
fetal kidney 105	0.7742532	0.84786
fetal adrenal gland 26	0.7814321	0.84842
fetal muscle 188	0.7898195	0.84876
fetal small intestine 80	0.7734419	0.84890
fetal large intestine 66	0.7591137	0.84910
fetal brain 36	0.8201703	0.84966
fetal muscle 161	0.7767448	0.85064
fetal lung 298	0.7947662	0.85108
fetal muscle 150	0.7778675	0.85198
placenta 201	0.7602227	0.85210
fetal renal cortex 203	0.7629447	0.85232
fetal kidney 107	0.7627275	0.85290
fetal heart 48	0.7713291	0.85296
fetal large intestine 54	0.7641220	0.85302
fetal kidney 96	0.7694553	0.85316
fetal renal pelvis 222	0.7536124	0.85378
fetal kidney 90	0.7574889	0.85550
fetal heart 45	0.7650528	0.85618
fetal muscle 163	0.7751810	0.85628
fetal brain 29	0.8403586	0.85778
fetal kidney 93	0.7443847	0.85892
fetal muscle 177	0.7638953	0.86116
fetal muscle 145	0.7535859	0.86370
fetal muscle 169	0.7719650	0.86422
fetal ovary 195	0.7154842	0.86452
fetal stomach 240	0.7212832	0.86474
fetal muscle 183	0.7853403	0.86478
fetal renal cortex 205	0.7533655	0.86736
fetal muscle 170	0.7812005	0.86772
fetal kidney 106	0.7533299	0.86894
fetal renal pelvis 227	0.7533094	0.86914
fetal testes 254	0.7609908	0.86996
fetal muscle 147	0.7609804	0.87004
fetal muscle 185	0.7582131	0.87068
fetal brain 35	0.8204721	0.87168
fetal muscle 180	0.7601994	0.87228
fetal muscle 146	0.7688443	0.87246
fetal kidney 102	0.7344154	0.87328
fetal brain 41	0.8022938	0.87404
fetal muscle 186	0.7692821	0.87470
fetal fibroblast 273	0.7931158	0.87792
melanocyte 338	0.8154995	0.87812
fetal kidney 101	0.7448859	0.87874
fetal large intestine 56	0.7435422	0.87996
melanocyte 337	0.8205252	0.88006
fetal muscle 157	0.7764534	0.88026
fetal muscle 144	0.7479050	0.88086
fetal renal pelvis 232	0.7370561	0.88112
fetal brain 31	0.8228359	0.88146
fetal adrenal gland 27	0.7448494	0.88146
fetal fibroblast 275	0.7963818	0.88202
fetal renal pelvis 216	0.7346279	0.88236
fetal kidney 89	0.7379223	0.88250
fetal muscle 175	0.7637427	0.88266
fetal kidney 104	0.7377060	0.88330
fetal renal cortex 212	0.7473102	0.88430
fetal kidney 99	0.7415200	0.88434
fetal muscle 193	0.7520949	0.88448
fetal kidney 103	0.7382699	0.88652
fetal kidney 85	0.7616558	0.88658
fetal fibroblast 272	0.7892038	0.88706
fetal muscle 167	0.7638921	0.88788
fetal muscle 155	0.7365512	0.88806
fetal muscle 174	0.7515723	0.88822
fetal fibroblast 274	0.7842380	0.88844
fetal muscle 154	0.7382798	0.88892
fetal muscle 178	0.7416754	0.89074
fetal renal cortex 213	0.7462995	0.89230
fetal muscle 184	0.7658055	0.89256
fetal heart 44	0.6894356	0.89316
fetal kidney 82	0.7100476	0.89420
fetal muscle 191	0.7452669	0.89422
fetal fibroblast 278	0.7762997	0.89518
fetal muscle 192	0.7506019	0.89566
fetal fibroblast 271	0.7783826	0.89624
fibroblast 332	0.7827529	0.89664
fetal renal pelvis 229	0.7255816	0.89664
fetal heart 50	0.7318962	0.89712
fetal renal cortex 211	0.7109797	0.89722
fetal heart 51	0.7361017	0.89820
fetal small intestine 79	0.7064974	0.90130
fetal muscle 189	0.7416481	0.90140
fetal kidney 83	0.6904004	0.90182
fetal kidney 97	0.7203539	0.90306
fetal renal cortex 214	0.7226114	0.90356
fetal brain 30	0.8122086	0.90416
fetal brain 39	0.7871492	0.90424
fetal muscle 164	0.7377582	0.90442
fetal kidney 88	0.7217016	0.90472
fetal muscle 165	0.7517207	0.90548
fibroblast 330	0.7695420	0.90618
fetal muscle 171	0.7474345	0.90646
fetal muscle 160	0.7294253	0.90678
fetal fibroblast 282	0.7783303	0.90702
fetal fibroblast 281	0.7728912	0.90752
fetal muscle 148	0.7297708	0.90764
fetal muscle 153	0.7179248	0.90850
fetal muscle 162	0.7382106	0.90966
fetal renal cortex 206	0.7202376	0.91034
fetal muscle 143	0.7496365	0.91052
ES cells 293	0.7102696	0.91136
fetal renal pelvis 224	0.7225403	0.91400
fetal large intestine 55	0.7186033	0.91474
fetal kidney 94	0.6953482	0.91596
fetal fibroblast 277	0.7521024	0.91750
pancreas 328	0.6847602	0.91760
fetal skin 234	0.7074940	0.91788
fetal muscle 159	0.7105977	0.91854
fetal renal pelvis 233	0.7034070	0.91898
fetal fibroblast 276	0.7628543	0.92032
fetal kidney 100	0.7005598	0.92216
fetal muscle 187	0.7271840	0.92288
fetal heart 46	0.6883719	0.92332
placenta 200	0.7371681	0.92410
fetal renal pelvis 226	0.6977450	0.92642
melanocyte 340	0.7516711	0.92686
fetal kidney 92	0.7079757	0.92716
fetal kidney 95	0.6982996	0.92768
fetal muscle 176	0.7157145	0.92868
fetal renal pelvis 231	0.7058422	0.92902
fetal fibroblast 269	0.7557008	0.92946
pancreas 327	0.6567310	0.93066
fetal kidney 91	0.7077668	0.93092
fetal renal pelvis 228	0.6738845	0.93294
ES derived NP 292	0.6785447	0.93472
fetal muscle 173	0.7052824	0.93480
fetal fibroblast 268	0.7419251	0.93500
fetal testes 255	0.7114905	0.93654
fetal kidney 81	0.7178644	0.93664
fetal fibroblast 280	0.7371907	0.93850
fibroblast 329	0.7355794	0.93924
fetal renal cortex 215	0.6921984	0.93948
fetal kidney 84	0.6703220	0.94222
fetal muscle 152	0.6969425	0.94248
placenta 198	0.7049313	0.94414
melanocyte 339	0.7395376	0.94570
fetal muscle 158	0.6939624	0.94734
ES cells 296	0.6341844	0.94852
fetal fibroblast 279	0.7181804	0.94912
fetal thymus 263	0.6779623	0.94928
placenta 197	0.6579857	0.95430
fetal renal cortex 207	0.6607969	0.95488
fetal muscle 172	0.6950320	0.95622
fetal fibroblast 270	0.7103454	0.95650
iPS 303	0.6764805	0.95922
placenta 196	0.6432252	0.95922
fetal fibroblast 267	0.6873259	0.96528
ES cells 294	0.6016390	0.96784
iPS 304	0.6755286	0.96920
iPS 302	0.6487268	0.97158
ES cells 295	0.5960583	0.97282
fetal kidney 87	0.6157893	0.97644
fetal renal pelvis 220	0.6018206	0.97646
breast 2	0.6769363	0.97914
muscle 341	0.5635965	0.97964
ES derived NP 291	0.5822129	0.97968
placenta 199	0.5892613	0.98710
keratinocyte 336	0.6611703	0.98794
breast 1	0.6447106	0.98798
ES derived mesoderm 285	0.6508342	0.98856
keratinocyte 333	0.6441264	0.99178
keratinocyte 334	0.6397821	0.99178
ES derived trophoblast 287	0.5471628	0.99178
keratinocyte 335	0.6311142	0.99270
ES derived trophoblast 288	0.5659638	0.99376
ES derived mesoderm 286	0.6326551	0.99544

Alzheimers disease

DHS sample	fold enrichment	p value
hematopoietic stem cell 310	1.3946830	0.00574
CD3 10	1.5637822	0.00826
CD19 6	1.6118211	0.00832
CD14 4	1.5097663	0.00970
CD14 3	1.5336256	0.01076
CD19 5	1.5421147	0.01308
CD19 7	1.5780923	0.01380
hematopoietic stem cell 312	1.3267966	0.02956
hematopoietic stem cell 314	1.3205147	0.03076
hematopoietic stem cell 317	1.2938674	0.03164
CD8 20	1.3995516	0.03898
hematopoietic stem cell 309	1.3052675	0.04444
CD56 17	1.3605663	0.04564
hematopoietic stem cell 305	1.3103029	0.04644
CD8 22	1.3715972	0.06744
hematopoietic stem cell 308	1.2485851	0.07568
hematopoietic stem cell 318	1.2723270	0.07630
hematopoietic stem cell 316	1.2701760	0.07712
hematopoietic stem cell 315	1.2578213	0.08226
hematopoietic stem cell 313	1.2332315	0.08728
hematopoietic stem cell 307	1.2428510	0.08804
hematopoietic stem cell 306	1.2380507	0.08968
hematopoietic stem cell 311	1.2374263	0.09112
CD4 16	1.3316443	0.09244
Mobilized CD3 321	1.3299446	0.09736
Mobilized CD4 322	1.3132750	0.10486
CD4 14	1.2563897	0.11334
Mobilized CD4 323	1.2939787	0.11540
CD3 9	1.2772499	0.12272
CD3 11	1.2431171	0.13184
fetal small intestine 75	1.3127195	0.13620
CD4 13	1.2601018	0.14464
CD4 15	1.2802623	0.15484
Mobilized CD8 325	1.2369376	0.16218
CD3 12	1.2308139	0.17306
fetal thymus 264	1.2012150	0.17428
CD8 19	1.2148346	0.19496
Mobilized CD56 324	1.1784917	0.19856
fetal thymus 256	1.1729150	0.20102
CD56 18	1.1958128	0.20538
fetal thymus 265	1.1713050	0.21064
fetal thymus 260	1.1717279	0.21090
fetal large intestine 61	1.1702552	0.21700
Mobilized CD3 320	1.1914838	0.21728
fetal thymus 266	1.1455960	0.23836
fetal brain 40	1.0952036	0.24728
CD8 21	1.1519040	0.25354
fetal thymus 257	1.1435237	0.25452
fetal thymus 258	1.1344124	0.25636
fetal thymus 262	1.1443701	0.26970
hematopoietic stem cell 319	1.1067389	0.28040
placenta 201	1.1006058	0.28912
fetal brain 30	1.0566567	0.31804
placenta 200	1.0717162	0.32302
fetal brain 31	1.0563829	0.32314
muscle 341	1.0755818	0.33566
placenta 196	1.0644654	0.35382
fetal brain 29	1.0446992	0.35528
fetal thymus 259	1.0673020	0.36444
fetal brain 36	1.0365018	0.39266
ovary 326	1.0423370	0.40534
fetal large intestine 53	1.0304185	0.42040
fetal thymus 261	1.0322563	0.42216
fetal lung 117	1.0256208	0.42372
placenta 197	1.0288424	0.42604
fetal small intestine 70	1.0219628	0.43950
fetal spinal cord 235	1.0134705	0.44692
fetal stomach 242	1.0096311	0.46146
fetal muscle 180	1.0064127	0.47146
fetal spinal cord 237	1.0047518	0.47466
fetal muscle 190	1.0040536	0.47624
fetal large intestine 60	1.0026848	0.47866
fetal small intestine 77	0.9971990	0.49060
fetal muscle 148	0.9958176	0.49404
fetal lung 133	0.9987274	0.49530
fetal large intestine 57	0.9980217	0.49552
fetal brain 41	0.9971010	0.49650
small intestine 342	0.9916513	0.50530
fetal brain 39	0.9939411	0.50720
fetal muscle 153	0.9857080	0.51542
placenta 199	0.9846239	0.52532
fetal muscle 181	0.9812515	0.52770
placenta 198	0.9837083	0.52830
fetal muscle 168	0.9812261	0.52842
fetal muscle 152	0.9821039	0.52870
fetal small intestine 76	0.9798701	0.52942
fetal small intestine 71	0.9792552	0.52970
fetal stomach 249	0.9737851	0.53038
fetal brain 34	0.9865523	0.53448
fetal small intestine 74	0.9771024	0.53496
fetal small intestine 69	0.9738492	0.53852
fetal large intestine 62	0.9718186	0.54110
fetal adrenal gland 24	0.9708466	0.54242
fetal large intestine 63	0.9727049	0.54278
fetal large intestine 55	0.9751038	0.54402
melanocyte 338	0.9804951	0.54470
fetal muscle 164	0.9747356	0.54580
fetal muscle 151	0.9693870	0.54938
fetal stomach 244	0.9646857	0.55516
fetal muscle 191	0.9680185	0.55634
fetal lung 140	0.9731330	0.55742
fetal lung 128	0.9779363	0.55806
fetal large intestine 58	0.9701625	0.55834
fetal stomach 240	0.9568314	0.56298
fetal brain 35	0.9768029	0.56394
fetal small intestine 73	0.9631637	0.56438
fetal large intestine 66	0.9602517	0.56456
fetal large intestine 59	0.9614107	0.56624
fetal ovary 195	0.9515551	0.57258
fetal large intestine 56	0.9611538	0.57306
fetal muscle 167	0.9622256	0.57320
fetal muscle 155	0.9580026	0.57598
fetal small intestine 79	0.9566894	0.57660
fetal muscle 188	0.9616808	0.57842
fetal muscle 159	0.9561398	0.57992
fetal muscle 176	0.9591225	0.58030
fetal lung 132	0.9631591	0.58200
fetal muscle 150	0.9563608	0.58366
fetal small intestine 80	0.9560052	0.58540
fetal lung 110	0.9595508	0.58748
fetal muscle 177	0.9515429	0.58964
fetal adrenal gland 27	0.9556976	0.59020
fetal renal pelvis 218	0.9379482	0.59146
fetal lung 129	0.9593279	0.59168
fetal small intestine 78	0.9574147	0.59252
fetal muscle 154	0.9490486	0.59254
fetal adrenal gland 26	0.9540694	0.59334
fetal stomach 253	0.9416950	0.59444
fetal muscle 149	0.9498495	0.59548
fetal muscle 163	0.9521225	0.59562
fetal large intestine 67	0.9490803	0.59706
fetal muscle 187	0.9535430	0.59846
fetal muscle 175	0.9515183	0.59936
fetal muscle 147	0.9498450	0.60092
fetal muscle 194	0.9560200	0.60228
fetal lung 136	0.9538036	0.60424
fetal stomach 252	0.9410906	0.60432
fetal stomach 250	0.9375236	0.60476
fetal muscle 145	0.9410988	0.60924
fetal stomach 247	0.9252501	0.61262
fetal muscle 179	0.9456111	0.61506
fetal muscle 192	0.9445728	0.61674
fetal renal pelvis 219	0.9232580	0.61866
fetal large intestine 65	0.9360235	0.62030
fetal muscle 182	0.9429213	0.62228
fetal brain 37	0.9509720	0.62470
fetal muscle 186	0.9420061	0.62530
fetal large intestine 64	0.9369324	0.62902
melanocyte 339	0.9499340	0.62958
fetal lung 126	0.9500842	0.63022
fetal lung 111	0.9453197	0.63042
fetal muscle 189	0.9360130	0.63104
fetal stomach 245	0.9221180	0.63690
CD20 8	0.9329809	0.63696
fetal testes 255	0.9338925	0.63860
fetal lung 114	0.9465298	0.63882
fetal lung 120	0.9413964	0.63968
fetal kidney 91	0.9324184	0.64052
fetal muscle 160	0.9288778	0.64140
fetal lung 138	0.9376644	0.64336
fetal thymus 263	0.9286841	0.64396
fetal lung 137	0.9395843	0.64576
fetal kidney 103	0.9226449	0.64608
fetal muscle 166	0.9301813	0.64844
fetal small intestine 72	0.9254845	0.64926
fetal large intestine 54	0.9239509	0.65132
fetal renal pelvis 220	0.9204561	0.65342
fetal small intestine 68	0.9251977	0.65374
fetal lung 119	0.9372561	0.65544
fetal muscle 173	0.9237975	0.65780
fetal muscle 193	0.9210728	0.65804
ES derived trophoblast 287	0.9215174	0.65828
fetal stomach 251	0.9139827	0.65860
fetal muscle 144	0.9206989	0.65922
fetal stomach 243	0.9142691	0.66378
fetal lung 141	0.9330908	0.66638
fetal spinal cord 236	0.9200914	0.66772
fetal stomach 241	0.9023388	0.66882
fetal lung 124	0.9278379	0.66970
ES cells 295	0.9021748	0.67388
fetal lung 108	0.9208295	0.67484
fetal lung 139	0.9223127	0.67532
pancreas 327	0.8982088	0.67726
fibroblast 331	0.9310048	0.67728
pancreas 328	0.9003612	0.67762
fetal muscle 169	0.9140105	0.67830
ES cells 296	0.8991073	0.67866
melanocyte 340	0.9240353	0.68360
fetal lung 121	0.9272653	0.68438
fetal muscle 143	0.9186009	0.68472
fetal renal pelvis 230	0.8976462	0.68504
fetal lung 135	0.9193046	0.68548
fetal muscle 185	0.9086374	0.68578
fetal lung 115	0.9248162	0.68618
fetal stomach 248	0.8966023	0.68666
fetal lung 122	0.9156322	0.68780
fetal stomach 246	0.8913333	0.68850
fetal spinal cord 238	0.9128496	0.69002
fetal muscle 161	0.9083538	0.69192
melanocyte 337	0.9293771	0.69396
fetal renal cortex 207	0.9036836	0.69404
fibroblast 332	0.9241477	0.69580
fetal muscle 174	0.9045049	0.69592
fetal brain 38	0.9191989	0.69672
ES cells 293	0.8972011	0.69692
fetal lung 130	0.9174001	0.69776
fetal lung 125	0.9138930	0.69790
fibroblast 330	0.9182564	0.69854
fetal kidney 83	0.8879196	0.69860
fetal muscle 165	0.9108167	0.69924
fetal lung 299	0.9140057	0.70206
fetal adrenal gland 25	0.8993094	0.70338
fetal lung 123	0.9102684	0.70546
fetal renal pelvis 228	0.8869311	0.71032
fetal lung 113	0.9153470	0.71036
fetal lung 134	0.9087875	0.71066
fetal lung 127	0.9073762	0.71158
fetal kidney 84	0.8886190	0.71332
fetal kidney 82	0.8838213	0.71374
fetal kidney 86	0.8731374	0.71446
ES derived NP 291	0.8773001	0.71494
fetal muscle 156	0.9004169	0.71616
fetal lung 301	0.9072625	0.71688
fetal muscle 157	0.9021974	0.72228
fetal lung 131	0.9135829	0.72288
fetal lung 118	0.9121642	0.72310
fetal muscle 183	0.8985520	0.72424
fetal testes 254	0.8887743	0.72556
fetal muscle 184	0.9021464	0.72592
ES derived trophoblast 288	0.8950830	0.72778
ES cells 294	0.8705381	0.72910
fetal muscle 158	0.8934571	0.73382
stomach 283	0.8678152	0.73542
fetal adrenal gland 28	0.8976804	0.73576
fetal kidney 106	0.8800701	0.73954
fetal kidney 104	0.8807603	0.74090
ES derived MSC 290	0.8864932	0.74246
fetal muscle 162	0.8881415	0.74372
fetal spinal cord 239	0.8881102	0.74588
fetal renal cortex 213	0.8827315	0.74694
fetal renal pelvis 227	0.8758642	0.74850
fetal muscle 172	0.8977140	0.74984
fetal muscle 146	0.8835795	0.74984
iPS 302	0.8796925	0.75304
fibroblast 329	0.8949965	0.75378
fetal kidney 95	0.8711106	0.75444
fetal muscle 170	0.8843482	0.75714
fetal adrenal gland 23	0.8826503	0.75906
fetal renal cortex 211	0.8628518	0.75910
fetal lung 116	0.8970059	0.75982
fetal fibroblast 282	0.8990384	0.76032
fetal muscle 178	0.8711327	0.76268
fetal kidney 81	0.8826120	0.76324
fetal renal pelvis 232	0.8595872	0.76488
fetal fibroblast 279	0.8909782	0.76580
fetal lung 112	0.8840070	0.76646
fetal kidney 94	0.8572372	0.76882
ES derived mesoderm 285	0.8863752	0.77280
fetal kidney 87	0.8646196	0.77692
fetal renal pelvis 231	0.8664209	0.77796
fetal renal cortex 208	0.8519873	0.78482
fetal renal pelvis 216	0.8520256	0.78820
fetal kidney 107	0.8524336	0.79158
ES derived MSC 289	0.8585076	0.79282
fetal renal cortex 214	0.8551549	0.79378
fetal renal cortex 210	0.8442068	0.79666
fetal kidney 85	0.8652231	0.79902
fetal muscle 171	0.8593899	0.79912
iPS 304	0.8575571	0.80010
fetal fibroblast 267	0.8680848	0.80572
fetal fibroblast 272	0.8735790	0.80586
fetal fibroblast 281	0.8708358	0.80692
fetal lung 298	0.8572590	0.81260
fetal kidney 102	0.8294817	0.81342
fetal renal cortex 202	0.8440792	0.81390
fetal lung 142	0.8710417	0.81596
fetal kidney 100	0.8399289	0.81636
fetal renal pelvis 217	0.8342217	0.81756
fetal fibroblast 276	0.8689815	0.81804
fetal kidney 97	0.8383733	0.81806
fetal kidney 89	0.8341568	0.81822
fetal lung 300	0.8603685	0.81898
fetal kidney 99	0.8356213	0.82224
iPS 303	0.8408239	0.82248
fetal fibroblast 275	0.8653867	0.82446
fetal renal pelvis 223	0.8232837	0.82604
fetal fibroblast 273	0.8648682	0.82628
fetal kidney 98	0.8289524	0.82800
fetal kidney 93	0.8219364	0.83022
fetal renal pelvis 233	0.8238426	0.83204
fetal renal cortex 205	0.8302011	0.83240
fetal renal cortex 203	0.8286906	0.83328
keratinocyte 334	0.8724218	0.83440
fetal fibroblast 274	0.8565601	0.83610
ES derived mesoderm 286	0.8607494	0.84446
fetal kidney 92	0.8273736	0.84450
fetal fibroblast 278	0.8481816	0.84490
keratinocyte 333	0.8711748	0.84540
keratinocyte 335	0.8655630	0.84724
fetal kidney 101	0.8192008	0.84840
fetal renal pelvis 226	0.8217721	0.84992
fetal kidney 88	0.8217581	0.85248
breast 1	0.8542769	0.85256
fetal renal cortex 212	0.8224445	0.85286
fetal fibroblast 271	0.8471269	0.85494
breast 2	0.8514486	0.85666
fetal fibroblast 277	0.8381717	0.85788
stomach 284	0.7877144	0.85954
ES derived NP 292	0.7910364	0.86120
fetal heart 44	0.7865188	0.86194
fetal renal cortex 209	0.8041651	0.86224
keratinocyte 336	0.8574509	0.86260
fetal brain 32	0.8521893	0.86368
fetal lung 109	0.8500375	0.86498
fetal brain 33	0.8385285	0.86540
fetal renal pelvis 222	0.7971352	0.86758
fetal kidney 90	0.7976998	0.87024
fetal fibroblast 280	0.8373081	0.87032
fetal skin 234	0.8073234	0.87374
fetal renal cortex 215	0.8071672	0.87550
fetal kidney 96	0.8036028	0.87850
fetal renal pelvis 224	0.8026753	0.88374
fetal renal pelvis 221	0.8006381	0.88376
fetal renal cortex 204	0.8012506	0.88696
fetal renal pelvis 225	0.7807571	0.88734
fetal renal cortex 206	0.7943067	0.88766
fetal renal pelvis 229	0.7888353	0.89262
fetal fibroblast 268	0.8210798	0.89414
fetal fibroblast 270	0.8128221	0.89980
fetal kidney 105	0.7856868	0.90138
fetal heart 50	0.7787172	0.90944
heart 297	0.7554013	0.91236
fetal heart 52	0.7828728	0.92294
fetal fibroblast 269	0.8030788	0.92312
fetal heart 46	0.7465525	0.93160
fetal heart 51	0.7625810	0.93168
fetal heart 42	0.7464971	0.93832
fetal heart 43	0.7580902	0.94212
fetal heart 47	0.7222338	0.94424
fetal heart 45	0.7214198	0.95514
fetal heart 48	0.7087445	0.96690
fetal heart 49	0.6985948	0.96916

Plasma lipoproteins

DHS sample	fold enrichment	p value
small intestine 342	1.3726521	0.05048
fetal skin 234	1.3361204	0.05070
stomach 284	1.3960454	0.05154
CD14 4	1.3644928	0.05668
melanocyte 338	1.2340902	0.06870
breast 1	1.2469085	0.07052
keratinocyte 333	1.2183452	0.07084
keratinocyte 336	1.2187214	0.07452
CD20 8	1.3018352	0.07462
breast 2	1.2312952	0.08432
pancreas 328	1.3062003	0.08916
fetal lung 119	1.2383714	0.08992
fetal large intestine 67	1.3089476	0.09028
fetal small intestine 78	1.2616255	0.09258
ES derived trophoblast 288	1.2429939	0.09286
fetal large intestine 64	1.2793040	0.09740
keratinocyte 335	1.1921428	0.10234
fetal large intestine 60	1.2853810	0.10290
placenta 200	1.2269141	0.10570
keratinocyte 334	1.1867494	0.10684
fetal small intestine 70	1.2427647	0.10860
fetal small intestine 68	1.2510931	0.11154
fetal small intestine 74	1.2647273	0.11308
fetal large intestine 55	1.2507405	0.11490
fetal lung 132	1.2256781	0.11618
placenta 198	1.2212737	0.11894
fetal adrenal gland 28	1.2060979	0.12350
fetal large intestine 56	1.2450006	0.12620
melanocyte 337	1.1740111	0.12902
fetal small intestine 80	1.2411872	0.12948
fetal large intestine 57	1.2231649	0.13282
fetal small intestine 73	1.2423449	0.13378
ES derived trophoblast 287	1.2205563	0.13430
muscle 341	1.2473046	0.13728
fetal large intestine 62	1.2487990	0.13950
fetal large intestine 54	1.2295162	0.14390
fetal lung 140	1.1902668	0.14710
fetal lung 298	1.1881392	0.15554
fetal small intestine 76	1.2250283	0.15598
placenta 201	1.2033506	0.15994
fetal small intestine 79	1.2233459	0.15996
melanocyte 340	1.1738930	0.15996
fibroblast 329	1.1701116	0.16116
fetal lung 300	1.1758007	0.16210
fetal lung 129	1.1796610	0.16232
CD14 3	1.2341603	0.16354
fetal lung 299	1.1820665	0.16410
fetal lung 301	1.1782625	0.16434
fetal lung 118	1.1655030	0.16606
fetal small intestine 72	1.2048078	0.16608
fetal small intestine 71	1.2069174	0.16776
fetal adrenal gland 24	1.2173554	0.16932
fibroblast 332	1.1574726	0.17036
fetal large intestine 59	1.2079877	0.17130
fetal large intestine 58	1.1860235	0.17296
ES derived MSC 290	1.1778704	0.17426
fetal lung 111	1.1666080	0.17666
fetal small intestine 69	1.2136722	0.17716
fetal kidney 91	1.1747779	0.17822
fetal lung 117	1.1652145	0.17894
ES derived MSC 289	1.1738478	0.17970
fetal large intestine 63	1.1979722	0.18256
fetal lung 133	1.1563016	0.18302
fetal fibroblast 282	1.1446058	0.18664
pancreas 327	1.1960974	0.19468
fetal lung 120	1.1584291	0.19488
fibroblast 330	1.1421900	0.20280
stomach 283	1.1874909	0.20340
fetal fibroblast 281	1.1384748	0.20416
fetal kidney 100	1.1588050	0.21198
fetal lung 141	1.1354453	0.21420
placenta 197	1.1579119	0.21518
fetal large intestine 65	1.1639186	0.21584
fibroblast 331	1.1321463	0.21660
fetal lung 136	1.1345832	0.22468
fetal renal pelvis 220	1.1501496	0.22606
fetal large intestine 53	1.1688889	0.22810
fetal small intestine 77	1.1588219	0.22854
fetal kidney 95	1.1453581	0.23086
placenta 196	1.1430019	0.23144
fetal lung 134	1.1316150	0.23550
fetal renal cortex 212	1.1340216	0.23736
fetal lung 125	1.1267999	0.23908
fetal fibroblast 269	1.1129867	0.24090
fetal kidney 106	1.1352667	0.24108
fetal fibroblast 273	1.1145954	0.24422
fetal lung 113	1.1137117	0.24720
fetal lung 128	1.1079094	0.25132
fetal renal pelvis 231	1.1239515	0.25164
fetal lung 121	1.1124934	0.25362
Mobilized CD56 324	1.1500800	0.25548
fetal renal cortex 207	1.1260542	0.25626
fetal renal cortex 205	1.1256606	0.25686
fetal muscle 182	1.1172737	0.25862
fetal renal pelvis 227	1.1247654	0.25930
fetal kidney 85	1.1069886	0.26644
fetal muscle 194	1.1057551	0.26680
fetal lung 124	1.1074417	0.26862
fetal thymus 258	1.1414277	0.26880
fetal renal pelvis 233	1.1187868	0.27034
fetal fibroblast 280	1.0978300	0.27372
fetal lung 112	1.1035888	0.27614
fetal adrenal gland 25	1.1216176	0.27694
ES derived mesoderm 286	1.0905172	0.27774
fetal adrenal gland 26	1.1146290	0.27864
fetal fibroblast 267	1.0990720	0.28040
fetal kidney 99	1.1107244	0.28066
fetal renal cortex 213	1.1032511	0.28132
fetal brain 34	1.0873817	0.28306
fetal fibroblast 279	1.0943666	0.28436
fetal fibroblast 274	1.0932768	0.28452
fetal renal pelvis 232	1.1107807	0.28526
fetal adrenal gland 27	1.1091944	0.28614
fetal kidney 92	1.1024609	0.28794
melanocyte 339	1.0876754	0.28948
fetal lung 114	1.0895675	0.28956
fetal adrenal gland 23	1.0981383	0.29040
Mobilized CD8 325	1.1260847	0.29462
fetal muscle 169	1.0996190	0.29552
fetal lung 123	1.0921429	0.29672
fetal muscle 151	1.1013925	0.29854
fetal large intestine 66	1.1078005	0.29896
fetal renal cortex 215	1.0942875	0.29982
fetal muscle 185	1.0966195	0.30422
fetal lung 131	1.0812788	0.30476
fetal kidney 89	1.0971736	0.30484
fetal brain 35	1.0731153	0.30628
fetal lung 138	1.0883627	0.30638
fetal lung 108	1.0917391	0.30660
fetal muscle 175	1.0921387	0.30686
fetal lung 137	1.0840402	0.30708
fetal thymus 265	1.1082030	0.30842
fetal lung 130	1.0835313	0.30926
placenta 199	1.0877081	0.30942
fetal lung 127	1.0878570	0.30952
fetal stomach 244	1.1012165	0.30984
fetal thymus 263	1.0909873	0.31028
fetal thymus 256	1.1029962	0.31154
fetal muscle 184	1.0839302	0.31224
fetal lung 116	1.0797536	0.31296
fetal lung 109	1.0761002	0.31298
fetal kidney 84	1.0921931	0.31306
fetal lung 110	1.0846187	0.31428
fetal kidney 96	1.0845000	0.31554
fetal muscle 179	1.0857284	0.31594
fetal renal pelvis 224	1.0841986	0.31684
fetal kidney 87	1.0867168	0.31772
fetal kidney 83	1.0938575	0.31806
fetal kidney 82	1.0903175	0.32114
fetal renal cortex 202	1.0802441	0.32664
fetal muscle 157	1.0768207	0.32674
ES derived mesoderm 285	1.0730582	0.32724
fetal renal pelvis 228	1.0839807	0.32906
fetal lung 135	1.0743373	0.32946
fetal kidney 105	1.0776689	0.33012
fetal renal pelvis 217	1.0782676	0.33406
fetal renal pelvis 221	1.0751249	0.33414
fetal stomach 253	1.0846150	0.33520
fetal muscle 154	1.0788498	0.33652
fetal fibroblast 268	1.0657052	0.34144
fetal renal pelvis 219	1.0842235	0.34164
fetal muscle 153	1.0747211	0.34194
fetal fibroblast 271	1.0657201	0.34402
fetal lung 115	1.0653991	0.34416
ovary 326	1.0855325	0.34610
CD4 14	1.0829542	0.34860
fetal renal pelvis 225	1.0666877	0.35412
fetal muscle 166	1.0637216	0.35504
fetal kidney 104	1.0655752	0.35528
fetal renal pelvis 229	1.0636901	0.35622
CD56 17	1.0764118	0.35648
fetal kidney 103	1.0652416	0.35654
fetal renal cortex 211	1.0634322	0.35920
fetal muscle 189	1.0618937	0.35936
fetal brain 31	1.0478829	0.36064
fetal kidney 88	1.0604124	0.36104
fetal lung 126	1.0561065	0.36124
fetal brain 41	1.0515333	0.36524
fetal kidney 81	1.0562585	0.36570
fetal testes 254	1.0607339	0.36596
fetal muscle 155	1.0626722	0.36736
ES cells 294	1.0625438	0.36810
fetal muscle 164	1.0608487	0.36810
fetal brain 39	1.0495496	0.36818
fetal lung 139	1.0564948	0.36832
fetal muscle 174	1.0590078	0.36930
fetal renal pelvis 226	1.0552920	0.37322
fetal muscle 171	1.0527935	0.37484
fetal testes 255	1.0532562	0.37514
fetal renal cortex 214	1.0540533	0.37604
fetal muscle 149	1.0555534	0.37774
fetal renal cortex 206	1.0526531	0.37868
fetal muscle 167	1.0527987	0.38136
fetal kidney 101	1.0500854	0.38230
fetal ovary 195	1.0551471	0.38272
heart 297	1.0551703	0.38298
fetal thymus 259	1.0619646	0.38490
fetal muscle 176	1.0487809	0.38592
fetal kidney 102	1.0473007	0.38778
fetal kidney 107	1.0454980	0.39128
fetal muscle 193	1.0447537	0.39442
fetal thymus 260	1.0514884	0.39804
fetal stomach 242	1.0431622	0.40132
fetal lung 142	1.0375136	0.40170
CD8 21	1.0513305	0.40280
fetal renal cortex 210	1.0392025	0.40352
fetal muscle 146	1.0405328	0.40394
fetal muscle 168	1.0418137	0.40452
fetal fibroblast 277	1.0374865	0.40790
ES cells 296	1.0405742	0.40810
fetal muscle 150	1.0377378	0.41120
fetal fibroblast 270	1.0343059	0.41216
fetal muscle 188	1.0341021	0.41254
fetal muscle 178	1.0360334	0.41442
fetal renal pelvis 230	1.0312643	0.41530
fetal brain 38	1.0308098	0.41600
fetal kidney 86	1.0327272	0.41608
fetal muscle 148	1.0342073	0.41674
fetal renal cortex 204	1.0311505	0.41680
fetal renal pelvis 223	1.0322191	0.41754
fetal muscle 144	1.0298060	0.42482
CD8 19	1.0291812	0.42550
fetal brain 29	1.0253162	0.42584
fetal spinal cord 237	1.0288423	0.42620
fetal kidney 98	1.0261573	0.42768
fetal stomach 247	1.0249872	0.42768
fetal stomach 240	1.0237019	0.43060
fetal muscle 145	1.0260520	0.43264
fetal fibroblast 275	1.0248847	0.43418
fetal large intestine 61	1.0254928	0.43424
fetal muscle 180	1.0253676	0.43682
fetal muscle 172	1.0222796	0.43778
fetal stomach 250	1.0193279	0.43778
fetal muscle 187	1.0238380	0.43962
fetal muscle 156	1.0216722	0.43988
Mobilized CD3 321	1.0157592	0.44080
fetal fibroblast 278	1.0214040	0.44242
iPS 304	1.0173436	0.44302
fetal muscle 191	1.0198389	0.44492
fetal muscle 181	1.0213054	0.44524
CD3 9	1.0190490	0.44542
fetal muscle 152	1.0200868	0.44642
fetal muscle 165	1.0199625	0.44754
fetal muscle 183	1.0180168	0.44770
CD4 15	0.9996558	0.44904
fetal brain 40	1.0161213	0.45018
fetal brain 36	1.0151934	0.45154
iPS 302	1.0150488	0.45160
Mobilized CD4 322	1.0107555	0.45386
fetal muscle 143	1.0165277	0.45466
CD3 11	1.0164644	0.45524
fetal spinal cord 236	1.0149024	0.45652
fetal fibroblast 276	1.0149992	0.45736
fetal thymus 266	1.0145939	0.45736
fetal muscle 192	1.0131170	0.45868
fetal brain 30	1.0120764	0.46030
CD4 13	1.0087851	0.46266
fetal renal pelvis 218	1.0006254	0.46368
fetal renal pelvis 216	1.0054167	0.46432
fetal muscle 158	1.0119907	0.46444
fetal muscle 170	1.0109371	0.46450
fetal thymus 261	1.0066598	0.47152
fetal kidney 90	1.0033503	0.47158
fetal thymus 262	0.9945473	0.47158
fetal renal cortex 208	1.0029003	0.47178
fetal stomach 243	1.0024008	0.47220
fetal muscle 177	1.0035639	0.47342
CD8 20	1.0027441	0.47578
fetal kidney 94	0.9986320	0.47640
fetal muscle 159	1.0046621	0.47694
CD8 22	0.9913146	0.47722
fetal muscle 186	1.0038013	0.47764
Mobilized CD4 323	0.9983494	0.47824
fetal kidney 97	0.9987219	0.47826
fetal stomach 246	0.9933494	0.48040
fetal fibroblast 272	1.0049397	0.48236
fetal renal pelvis 222	0.9957575	0.48260
CD4 16	0.9804908	0.48446
fetal stomach 248	0.9955693	0.48542
fetal stomach 249	0.9872406	0.48654
CD19 5	0.9926524	0.49030
fetal renal cortex 209	0.9942011	0.49110
fetal muscle 147	0.9976550	0.49148
fetal brain 37	0.9968085	0.49300
fetal lung 122	0.9942705	0.49380
CD56 18	0.9890902	0.49526
fetal muscle 161	0.9915485	0.49806
fetal stomach 252	0.9821147	0.49976
fetal muscle 173	0.9889135	0.50564
fetal stomach 241	0.9763987	0.50994
ES derived NP 291	0.9790834	0.51128
iPS 303	0.9851246	0.51192
fetal muscle 162	0.9878610	0.51502
CD3 10	0.9797394	0.51568
fetal spinal cord 238	0.9884240	0.51580
fetal stomach 251	0.9774624	0.51602
fetal muscle 163	0.9830248	0.51664
fetal thymus 257	0.9708117	0.51942
CD3 12	0.9649399	0.52224
ES cells 295	0.9715430	0.52518
fetal muscle 160	0.9736455	0.53980
ES cells 293	0.9621366	0.54336
fetal thymus 264	0.9649064	0.54652
fetal renal cortex 203	0.9627752	0.55212
CD19 6	0.9492244	0.55538
fetal stomach 245	0.9535172	0.55726
hematopoietic stem cell 308	0.9658049	0.55942
hematopoietic stem cell 318	0.9601777	0.56248
fetal small intestine 75	0.8923241	0.56804
hematopoietic stem cell 306	0.9561335	0.57320
fetal kidney 93	0.9478403	0.57512
hematopoietic stem cell 319	0.9532962	0.57566
fetal brain 32	0.9610944	0.58108
Mobilized CD3 320	0.9342029	0.58512
CD19 7	0.9234976	0.58860
ES derived NP 292	0.9349851	0.59352
fetal heart 43	0.9497710	0.59724
fetal muscle 190	0.9416408	0.59908
hematopoietic stem cell 312	0.9466750	0.60360
fetal heart 46	0.9400475	0.60480
hematopoietic stem cell 307	0.9332071	0.61788
hematopoietic stem cell 316	0.9248157	0.62382
hematopoietic stem cell 305	0.9239046	0.63892
fetal spinal cord 235	0.9309372	0.64194
hematopoietic stem cell 311	0.9227730	0.64370
hematopoietic stem cell 310	0.9341852	0.64520
fetal brain 33	0.9258183	0.65266
fetal spinal cord 239	0.9232874	0.65400
hematopoietic stem cell 314	0.9248301	0.65672
hematopoietic stem cell 313	0.9206427	0.65760
fetal heart 49	0.9135027	0.66230
fetal heart 42	0.9087877	0.66778
fetal heart 51	0.9033920	0.68592
fetal heart 45	0.8979972	0.68796
fetal heart 44	0.8785751	0.68830
fetal heart 52	0.9045978	0.69434
fetal heart 50	0.8923436	0.70002
hematopoietic stem cell 315	0.8899721	0.70356
fetal heart 47	0.8754384	0.71396
fetal heart 48	0.8805039	0.72388
hematopoietic stem cell 309	0.8747347	0.73306
hematopoietic stem cell 317	0.8576807	0.79684

Fibrinogen

DHS sample	fold enrichment	p value
CD14 4	1.6040142	0.00134
CD14 3	1.5317241	0.00704
ES derived MSC 289	1.2112292	0.09652
keratinocyte 335	1.1617848	0.10496
fetal lung 298	1.1893885	0.10668
keratinocyte 336	1.1587612	0.10698
fetal lung 301	1.1739579	0.11990
CD4 14	1.2339253	0.12060
fetal lung 299	1.1631868	0.13940
CD4 13	1.2422472	0.14048
fetal lung 300	1.1511120	0.15038
hematopoietic stem cell 310	1.1412641	0.16430
fetal fibroblast 271	1.1305828	0.17000
keratinocyte 334	1.1189192	0.17526
CD56 17	1.1733460	0.18054
breast 1	1.1205316	0.18124
keratinocyte 333	1.1120571	0.18532
ES derived MSC 290	1.1387572	0.18604
hematopoietic stem cell 312	1.1372725	0.18830
fetal fibroblast 281	1.1146721	0.19528
fetal fibroblast 280	1.1106234	0.21004
placenta 201	1.1399019	0.21110
Mobilized CD4 323	1.1714704	0.21120
stomach 284	1.1440241	0.21712
hematopoietic stem cell 314	1.1196975	0.21726
hematopoietic stem cell 317	1.1084229	0.22272
fetal fibroblast 273	1.0993541	0.22482
fetal small intestine 78	1.1142266	0.23022
fetal fibroblast 279	1.1012649	0.23362
placenta 200	1.1063067	0.23670
hematopoietic stem cell 309	1.1149789	0.23888
fetal small intestine 80	1.1150573	0.24644
fetal fibroblast 272	1.0897343	0.25158
CD4 16	1.1449371	0.25266
fetal fibroblast 269	1.0884926	0.25612
breast 2	1.0868184	0.25678
fetal fibroblast 276	1.0861294	0.25736
fetal fibroblast 282	1.0852779	0.25760
fetal fibroblast 268	1.0855899	0.25794
fetal large intestine 60	1.1097213	0.25932
fetal fibroblast 277	1.0864760	0.26378
hematopoietic stem cell 315	1.1012167	0.26812
fetal fibroblast 275	1.0806237	0.26948
Mobilized CD4 322	1.1273898	0.27338
CD20 8	1.0967562	0.27438
melanocyte 338	1.0752417	0.27648
fetal lung 134	1.0842757	0.28166
hematopoietic stem cell 313	1.0853704	0.28270
fetal fibroblast 270	1.0787269	0.28340
fetal fibroblast 274	1.0759470	0.28350
fibroblast 331	1.0788356	0.28440
muscle 341	1.0981807	0.29066
fetal small intestine 74	1.0885752	0.29258
fetal large intestine 64	1.0790261	0.31080
fetal large intestine 58	1.0734010	0.31606
fetal large intestine 67	1.0786567	0.31670
fibroblast 332	1.0632818	0.31720
fetal small intestine 79	1.0811779	0.31758
fetal large intestine 55	1.0751724	0.31880
CD3 9	1.0897721	0.32258
Mobilized CD56 324	1.0853499	0.32318
fetal small intestine 77	1.0736681	0.32850
hematopoietic stem cell 305	1.0684684	0.32946
fetal lung 141	1.0556589	0.33636
stomach 283	1.0736669	0.33654
fetal small intestine 68	1.0643440	0.33766
fetal lung 110	1.0597579	0.33772
fetal small intestine 70	1.0609708	0.33780
placenta 196	1.0649446	0.33992
hematopoietic stem cell 306	1.0588675	0.34502
fetal small intestine 72	1.0622856	0.34632
fetal lung 133	1.0512769	0.34712
fetal large intestine 54	1.0630482	0.34886
fetal lung 115	1.0512180	0.34894
fetal fibroblast 267	1.0519310	0.34900
fetal large intestine 56	1.0604740	0.35056
CD8 20	1.0703780	0.35126
Mobilized CD3 321	1.0723447	0.35438
fetal fibroblast 278	1.0484681	0.35550
fetal muscle 194	1.0483168	0.35858
small intestine 342	1.0584768	0.35950
fetal lung 118	1.0453614	0.36096
fetal small intestine 76	1.0585542	0.36146
fetal thymus 256	1.0610159	0.36158
hematopoietic stem cell 311	1.0518060	0.36174
pancreas 327	1.0589453	0.36190
fetal lung 121	1.0441880	0.36226
fetal lung 117	1.0462183	0.36306
placenta 198	1.0514168	0.36312
fetal stomach 244	1.0544189	0.36892
placenta 199	1.0490314	0.36998
CD4 15	1.0672380	0.37056
fetal lung 111	1.0429026	0.37068
fibroblast 330	1.0437391	0.37374
CD3 11	1.0566447	0.37568
CD8 21	1.0613428	0.37724
CD8 22	1.0555043	0.37964
fetal lung 138	1.0393841	0.38316
hematopoietic stem cell 316	1.0438173	0.38584
ES derived trophoblast 288	1.0394259	0.38676
fetal muscle 167	1.0405191	0.38692
melanocyte 340	1.0376406	0.38772
fetal large intestine 62	1.0433970	0.38974
fetal lung 125	1.0350102	0.39588
fetal thymus 266	1.0439543	0.39810
fetal lung 131	1.0313303	0.39810
fetal large intestine 59	1.0372471	0.40008
placenta 197	1.0366129	0.40014
fetal lung 132	1.0310679	0.40176
fetal small intestine 75	1.0370903	0.40534
fetal large intestine 57	1.0334480	0.40756
CD56 18	1.0401076	0.40942
fetal lung 129	1.0292165	0.41042
CD3 10	1.0351156	0.41242
CD19 6	1.0374573	0.41304
fetal lung 126	1.0261558	0.41444
fetal lung 116	1.0255724	0.41470
CD8 19	1.0383231	0.41498
fetal lung 136	1.0266870	0.41768
hematopoietic stem cell 307	1.0278137	0.41824
fetal large intestine 53	1.0291889	0.41834
fetal lung 130	1.0235145	0.42252
fetal lung 140	1.0242015	0.42346
Mobilized CD8 325	1.0297030	0.42388
fetal small intestine 69	1.0257958	0.42514
fetal lung 139	1.0231398	0.42578
melanocyte 337	1.0217145	0.42704
fibroblast 329	1.0227758	0.42976
CD19 5	1.0274768	0.43056
CD3 12	1.0208714	0.43780
fetal large intestine 65	1.0201979	0.43782
fetal lung 128	1.0164400	0.44278
fetal small intestine 71	1.0159698	0.44676
fetal muscle 180	1.0172802	0.44722
fetal lung 114	1.0143503	0.44910
hematopoietic stem cell 318	1.0120677	0.45234
fetal thymus 262	1.0134551	0.45326
fetal large intestine 66	1.0115390	0.45538
fetal lung 137	1.0093391	0.46050
fetal lung 135	1.0093142	0.46222
fetal lung 123	1.0085619	0.46280
fetal lung 120	1.0086495	0.46358
fetal small intestine 73	1.0074295	0.46420
fetal stomach 241	1.0071185	0.46558
fetal stomach 243	1.0074791	0.46562
fetal large intestine 63	1.0059458	0.46884
fetal heart 43	1.0054294	0.47482
fetal lung 119	1.0035125	0.47714
fetal stomach 245	0.9998196	0.48112
fetal lung 109	1.0023424	0.48114
fetal thymus 264	1.0006623	0.48414
fetal lung 108	0.9989640	0.48440
fetal thymus 257	0.9959028	0.48838
fetal heart 42	0.9990365	0.48878
fetal heart 47	0.9923920	0.49152
fetal kidney 100	0.9945209	0.49238
fetal muscle 187	0.9979930	0.49346
fetal muscle 151	0.9944409	0.49480
hematopoietic stem cell 319	0.9942521	0.49486
fetal lung 122	0.9945659	0.49628
fetal heart 45	0.9922148	0.49972
fetal muscle 164	0.9935576	0.50028
fetal muscle 143	0.9941199	0.50064
fetal kidney 91	0.9910584	0.50332
fetal lung 124	0.9921035	0.50422
fetal stomach 247	0.9826451	0.50632
fetal renal pelvis 217	0.9853850	0.51044
hematopoietic stem cell 308	0.9877334	0.51496
melanocyte 339	0.9899332	0.52270
fetal muscle 154	0.9836163	0.52354
pancreas 328	0.9813227	0.52370
fetal large intestine 61	0.9775285	0.52404
fetal muscle 172	0.9874573	0.52450
fetal muscle 160	0.9813787	0.52996
fetal stomach 240	0.9742214	0.53042
fetal lung 113	0.9850176	0.53182
fetal kidney 86	0.9698507	0.53436
CD19 7	0.9683517	0.53490
fetal renal cortex 211	0.9741918	0.53540
Mobilized CD3 320	0.9700646	0.53834
fetal thymus 260	0.9740565	0.53902
fetal kidney 87	0.9718871	0.54564
fetal stomach 249	0.9659647	0.54652
heart 297	0.9699734	0.55032
fetal kidney 89	0.9682907	0.55232
ES derived trophoblast 287	0.9731006	0.55438
fetal stomach 242	0.9535822	0.55784
fetal stomach 250	0.9590451	0.55994
fetal kidney 93	0.9633828	0.56200
fetal heart 46	0.9672423	0.56210
fetal muscle 145	0.9625589	0.56698
fetal renal pelvis 216	0.9616518	0.56784
fetal thymus 259	0.9568632	0.56806
fetal thymus 261	0.9585087	0.56928
fetal renal cortex 203	0.9599435	0.57162
fetal muscle 179	0.9652220	0.57336
ovary 326	0.9509966	0.57376
fetal lung 127	0.9658910	0.57542
fetal muscle 181	0.9623924	0.57868
fetal muscle 162	0.9655146	0.57880
fetal thymus 265	0.9528560	0.58314
fetal muscle 174	0.9602996	0.58330
fetal stomach 246	0.9480827	0.58632
fetal muscle 177	0.9549411	0.58824
fetal kidney 82	0.9482912	0.58860
fetal muscle 146	0.9604631	0.59024
fetal renal pelvis 220	0.9500086	0.59296
fetal renal pelvis 218	0.9383194	0.59312
fetal kidney 83	0.9426625	0.59408
fetal renal pelvis 219	0.9382963	0.59540
fetal stomach 252	0.9447138	0.59900
fetal renal pelvis 228	0.9441578	0.60036
fetal stomach 253	0.9383226	0.60466
fetal kidney 97	0.9465339	0.60476
fetal lung 142	0.9597521	0.60608
fetal renal cortex 214	0.9447086	0.60922
fetal muscle 176	0.9552933	0.60930
fetal heart 49	0.9448896	0.61194
fetal muscle 193	0.9455589	0.61410
fetal renal cortex 206	0.9459256	0.61460
fetal muscle 168	0.9459893	0.61482
fetal stomach 248	0.9373179	0.61486
fetal muscle 188	0.9472838	0.62078
fetal muscle 148	0.9453784	0.62136
fetal muscle 163	0.9413860	0.62282
fetal kidney 106	0.9364775	0.62778
fetal kidney 90	0.9351704	0.62898
fetal muscle 149	0.9395390	0.62974
fetal renal pelvis 230	0.9271709	0.63094
fetal muscle 190	0.9339324	0.63116
fetal kidney 81	0.9446082	0.63136
fetal muscle 189	0.9408084	0.63348
fetal renal pelvis 226	0.9363378	0.63426
fetal muscle 147	0.9392266	0.63490
fetal kidney 107	0.9324949	0.63810
fetal lung 112	0.9419655	0.64026
fetal kidney 94	0.9245836	0.64104
fetal kidney 84	0.9241493	0.64440
fetal heart 51	0.9342341	0.64800
fetal renal pelvis 231	0.9306244	0.64900
fetal muscle 150	0.9296748	0.65122
fetal muscle 178	0.9298068	0.65274
fetal muscle 191	0.9315451	0.65394
fetal renal cortex 207	0.9255971	0.65406
fetal muscle 166	0.9330672	0.65452
fetal kidney 96	0.9268863	0.65900
fetal kidney 88	0.9269571	0.65950
fetal stomach 251	0.9176407	0.65966
fetal renal pelvis 222	0.9183835	0.66124
fetal renal pelvis 232	0.9191442	0.66126
fetal renal pelvis 233	0.9207642	0.66182
fetal kidney 101	0.9240828	0.66216
fetal muscle 192	0.9309662	0.66400
fetal muscle 183	0.9299699	0.66416
fetal adrenal gland 25	0.9195846	0.66612
fetal muscle 157	0.9337731	0.66630
fetal renal cortex 205	0.9208543	0.66714
fetal kidney 105	0.9220320	0.66846
fetal kidney 85	0.9290330	0.67138
fetal muscle 182	0.9257588	0.67256
fetal muscle 171	0.9275575	0.67258
fetal heart 48	0.9205977	0.67372
fetal renal pelvis 223	0.9115477	0.67454
fetal heart 50	0.9210030	0.67646
fetal thymus 263	0.9196120	0.67664
fetal muscle 185	0.9176199	0.67934
fetal heart 52	0.9258260	0.67966
fetal kidney 92	0.9170634	0.68052
fetal renal cortex 208	0.9108799	0.68218
fetal muscle 144	0.9143225	0.68250
fetal thymus 258	0.9032589	0.68256
fetal muscle 175	0.9192845	0.68308
fetal renal cortex 213	0.9195975	0.68364
fetal renal cortex 202	0.9170540	0.68364
fetal muscle 153	0.9139754	0.68502
fetal muscle 184	0.9255196	0.68632
fetal muscle 186	0.9191875	0.68902
fetal kidney 102	0.9003396	0.69360
fetal renal pelvis 229	0.9101152	0.69524
fetal testes 254	0.9097263	0.69662
fetal renal cortex 204	0.9128440	0.69846
fetal renal cortex 215	0.9099108	0.69986
fetal muscle 156	0.9127924	0.70302
fetal renal pelvis 225	0.8967630	0.70864
fetal kidney 98	0.8984275	0.70882
fetal kidney 104	0.8980711	0.70970
fetal muscle 161	0.9036389	0.71222
fetal kidney 99	0.8984797	0.71318
fetal kidney 95	0.8970979	0.71372
fetal ovary 195	0.8780728	0.71436
fetal renal cortex 209	0.8937161	0.72184
fetal muscle 165	0.9081326	0.72276
fetal renal pelvis 227	0.8948950	0.72364
fetal adrenal gland 26	0.8969153	0.72646
fetal renal pelvis 224	0.8984520	0.72706
fetal muscle 169	0.8984112	0.72970
fetal renal cortex 210	0.8838237	0.73526
fetal renal cortex 212	0.8933736	0.73796
fetal muscle 173	0.8900794	0.74370
fetal muscle 158	0.8967240	0.74686
fetal muscle 152	0.8935431	0.75360
fetal adrenal gland 23	0.8919129	0.75366
fetal heart 44	0.8642653	0.75720
fetal testes 255	0.8881429	0.75792
fetal adrenal gland 27	0.8735482	0.77530
fetal muscle 159	0.8690091	0.77632
fetal kidney 103	0.8621718	0.77808
fetal muscle 170	0.8796048	0.78218
ES derived NP 291	0.8504081	0.78494
fetal adrenal gland 28	0.8823740	0.79072
fetal muscle 155	0.8562509	0.79866
fetal adrenal gland 24	0.8370860	0.81044
fetal skin 234	0.8504913	0.81530
fetal renal pelvis 221	0.8484237	0.82244
ES derived mesoderm 285	0.8728261	0.82642
ES cells 293	0.8221641	0.85080
fetal spinal cord 236	0.8180353	0.87018
ES cells 294	0.7953311	0.87070
ES derived NP 292	0.7979492	0.87668
iPS 303	0.8181747	0.88124
ES cells 296	0.7694693	0.90240
iPS 302	0.7975753	0.90654
ES cells 295	0.7623277	0.91296
fetal spinal cord 237	0.7951480	0.91768
fetal spinal cord 238	0.7947779	0.92076
ES derived mesoderm 286	0.8250470	0.92490
fetal spinal cord 235	0.7707235	0.94010
fetal spinal cord 239	0.7719473	0.94014
iPS 304	0.7692963	0.94558
fetal brain 38	0.7512844	0.96840
fetal brain 31	0.7867502	0.97118
fetal brain 33	0.7434993	0.97282
fetal brain 41	0.7452224	0.97600
fetal brain 37	0.7428117	0.97694
fetal brain 30	0.7792534	0.97860
fetal brain 29	0.7684604	0.98250
fetal brain 39	0.7351224	0.98346
fetal brain 35	0.7400009	0.98860
fetal brain 32	0.7205732	0.98952
fetal brain 34	0.7107751	0.99410
fetal brain 40	0.6917101	0.99500
fetal brain 36	0.6583194	0.99718

Vitiligo

DHS sample	fold enrichment	p value
Mobilized CD3 320	2.1150241	0.00156
fetal fibroblast 277	1.4890919	0.00796
fetal fibroblast 274	1.4495364	0.01036
fetal fibroblast 273	1.4510679	0.01060
CD4 13	1.8112341	0.01072
CD3 9	1.7633056	0.01170
Mobilized CD56 324	1.6871156	0.01178
fetal fibroblast 269	1.4237083	0.01252
fetal fibroblast 272	1.4270762	0.01260
fetal fibroblast 271	1.4162756	0.01390
hematopoietic stem cell 310	1.4469243	0.01402
melanocyte 339	1.4076312	0.01428
fetal fibroblast 280	1.4038942	0.01734
CD4 14	1.6266161	0.01760
fetal fibroblast 282	1.3856402	0.01764
melanocyte 340	1.4169911	0.01866
Mobilized CD4 323	1.7021445	0.01924
fetal fibroblast 278	1.4007959	0.02098
fetal thymus 256	1.5574209	0.02122
fetal large intestine 61	1.6508483	0.02168
fetal fibroblast 281	1.3900364	0.02206
hematopoietic stem cell 314	1.4505962	0.02270
fetal fibroblast 275	1.3649484	0.02368
fetal fibroblast 270	1.3835165	0.02446
fetal fibroblast 279	1.3790287	0.02510
CD3 11	1.5794397	0.02584
fetal fibroblast 267	1.3817121	0.02702
melanocyte 338	1.3359760	0.02756
CD56 17	1.5275231	0.02780
fetal fibroblast 268	1.3513914	0.02872
melanocyte 337	1.3290650	0.02952
CD8 20	1.5635599	0.03006
CD56 18	1.6068402	0.03306
CD8 21	1.6208707	0.03494
hematopoietic stem cell 305	1.4443698	0.03588
fetal fibroblast 276	1.3316719	0.03718
hematopoietic stem cell 309	1.4045497	0.04256
hematopoietic stem cell 312	1.3725369	0.04896
CD4 16	1.5723055	0.05426
fetal thymus 260	1.4576921	0.05470
fibroblast 331	1.3123899	0.05902
Mobilized CD4 322	1.5300724	0.06070
fetal thymus 265	1.4394141	0.06168
CD3 12	1.5160114	0.06540
CD3 10	1.4531015	0.06542
CD14 4	1.4100584	0.07118
hematopoietic stem cell 315	1.3583403	0.07374
CD19 6	1.4557116	0.07558
CD19 5	1.4387226	0.07596
fibroblast 332	1.2816544	0.07632
fibroblast 329	1.2894875	0.07698
fetal thymus 258	1.4000520	0.07716
CD19 7	1.4623739	0.07768
Mobilized CD3 321	1.4918804	0.08006
ES derived MSC 290	1.3194510	0.08046
CD8 22	1.4543497	0.08066
fetal small intestine 75	1.5843808	0.08146
fibroblast 330	1.2795620	0.08480
keratinocyte 336	1.2278584	0.08548
hematopoietic stem cell 318	1.3361382	0.08836
fetal thymus 266	1.3736420	0.09000
keratinocyte 335	1.2195336	0.09448
Mobilized CD8 325	1.4341960	0.09482
ES derived MSC 289	1.2943374	0.09698
fetal lung 299	1.2821652	0.09872
CD14 3	1.3640532	0.10294
hematopoietic stem cell 316	1.3086777	0.10382
CD8 19	1.4100121	0.11376
CD4 15	1.4452693	0.11890
hematopoietic stem cell 308	1.2560831	0.11980
fetal lung 298	1.2484509	0.12092
fetal lung 300	1.2372402	0.12108
keratinocyte 334	1.1843631	0.12540
fetal muscle 176	1.2367236	0.13052
fetal brain 35	1.1771000	0.13214
fetal brain 30	1.1575171	0.14310
hematopoietic stem cell 313	1.2250260	0.14348
fetal thymus 259	1.3024275	0.14810
keratinocyte 333	1.1686624	0.14864
fetal brain 40	1.1790511	0.15892
fetal large intestine 67	1.2347646	0.16530
fetal brain 34	1.1611103	0.16950
fetal thymus 264	1.2473967	0.17212
fetal thymus 261	1.2465647	0.18204
placenta 200	1.1842613	0.18720
hematopoietic stem cell 306	1.1959202	0.18736
fetal thymus 257	1.2565864	0.18860
hematopoietic stem cell 317	1.1677403	0.19034
fetal lung 301	1.1737039	0.19296
fetal brain 29	1.1281671	0.19534
placenta 199	1.1878579	0.20086
fetal brain 37	1.1507058	0.20298
fetal muscle 180	1.1812789	0.21434
fetal muscle 172	1.1525488	0.21598
hematopoietic stem cell 307	1.1666475	0.21842
fetal muscle 152	1.1602367	0.21954
hematopoietic stem cell 311	1.1662964	0.21996
pancreas 328	1.1892798	0.22106
fetal small intestine 78	1.1594717	0.22352
fetal muscle 169	1.1673397	0.22412
breast 2	1.1372862	0.22574
fetal brain 31	1.1139034	0.22706
placenta 197	1.1716761	0.22908
placenta 201	1.1742947	0.22970
breast 1	1.1322325	0.23000
ES derived trophoblast 288	1.1471161	0.23088
heart 297	1.1786605	0.23210
fetal thymus 262	1.2332010	0.23290
fetal muscle 143	1.1436928	0.23360
pancreas 327	1.1799058	0.23832
fetal adrenal gland 25	1.1646740	0.24018
fetal muscle 166	1.1425983	0.24826
fetal adrenal gland 24	1.1565849	0.25620
CD20 8	1.1413736	0.25942
fetal large intestine 55	1.1385201	0.26124
fetal muscle 154	1.1433756	0.27014
fetal muscle 150	1.1313828	0.27542
placenta 196	1.1354068	0.27722
fetal muscle 155	1.1332300	0.27914
fetal heart 43	1.1171357	0.28272
fetal muscle 179	1.1194281	0.28302
fetal muscle 181	1.1181938	0.28576
ovary 326	1.1427533	0.28640
placenta 198	1.1159987	0.29062
fetal brain 39	1.0861027	0.29706
small intestine 342	1.1173568	0.29898
fetal muscle 149	1.1083036	0.30716
ES derived mesoderm 285	1.0899735	0.30770
fetal adrenal gland 28	1.0934284	0.31148
fetal large intestine 64	1.1025555	0.31506
fetal muscle 164	1.0987963	0.32102
fetal adrenal gland 26	1.0947326	0.32348
fetal brain 38	1.0782424	0.32466
fetal muscle 159	1.0978788	0.32660
fetal heart 46	1.0912300	0.33026
fetal lung 128	1.0768301	0.33054
fetal muscle 182	1.0882008	0.33104
fetal small intestine 77	1.0973612	0.33368
fetal muscle 151	1.0908086	0.33420
fetal stomach 244	1.0953173	0.33614
fetal muscle 184	1.0812818	0.33674
fetal muscle 187	1.0805248	0.33948
fetal large intestine 57	1.0843208	0.34000
fetal lung 140	1.0770955	0.34050
fetal ovary 195	1.0973360	0.34094
fetal muscle 175	1.0820996	0.34170
fetal large intestine 58	1.0813046	0.34256
fetal large intestine 60	1.0884223	0.34442
fetal small intestine 70	1.0762829	0.34558
stomach 284	1.0856172	0.34732
fetal small intestine 80	1.0813043	0.34862
fetal brain 41	1.0648380	0.34910
fetal muscle 153	1.0823535	0.34994
fetal muscle 160	1.0812055	0.35086
fetal muscle 146	1.0736326	0.35144
fetal muscle 167	1.0745349	0.35476
fetal skin 234	1.0773370	0.35740
fetal muscle 183	1.0680516	0.36012
fetal small intestine 68	1.0703953	0.36614
fetal adrenal gland 27	1.0657254	0.36930
fetal heart 47	1.0661074	0.37206
fetal muscle 162	1.0601803	0.37404
fetal small intestine 74	1.0652248	0.37470
fetal small intestine 79	1.0676087	0.37662
fetal lung 134	1.0583523	0.37670
fetal brain 36	1.0527969	0.37812
fetal large intestine 56	1.0633038	0.37976
fetal muscle 178	1.0574814	0.38552
fetal muscle 186	1.0545959	0.38688
fetal heart 49	1.0507633	0.38776
fetal large intestine 62	1.0596644	0.38856
fetal muscle 177	1.0529598	0.39024
fetal heart 44	1.0533672	0.39184
fetal muscle 165	1.0494876	0.39292
fetal heart 45	1.0469571	0.39394
fetal muscle 174	1.0502429	0.39462
fetal muscle 185	1.0512128	0.39638
fetal muscle 156	1.0466976	0.39752
fetal spinal cord 237	1.0430696	0.39810
stomach 283	1.0493224	0.39858
fetal thymus 263	1.0478748	0.39904
fetal lung 116	1.0398929	0.40624
hematopoietic stem cell 319	1.0440724	0.40704
fetal small intestine 69	1.0455331	0.40716
fetal renal pelvis 220	1.0431670	0.40804
fetal heart 42	1.0359634	0.41230
fetal heart 52	1.0352366	0.41244
fetal lung 118	1.0368612	0.41334
fetal muscle 148	1.0381590	0.41820
fetal heart 51	1.0336249	0.41848
fetal heart 48	1.0312103	0.42060
ES derived trophoblast 287	1.0383143	0.42140
fetal lung 119	1.0322914	0.42232
fetal lung 111	1.0302730	0.42614
fetal lung 126	1.0297853	0.42674
muscle 341	1.0292224	0.42960
fetal muscle 191	1.0290455	0.42978
fetal muscle 157	1.0272100	0.43352
fetal large intestine 66	1.0289780	0.43356
fetal muscle 193	1.0264390	0.43502
fetal large intestine 65	1.0256937	0.43668
fetal muscle 158	1.0242578	0.43874
fetal spinal cord 236	1.0192575	0.44516
fetal small intestine 71	1.0180799	0.44926
fetal kidney 81	1.0164102	0.45326
fetal muscle 147	1.0136577	0.45544
fetal muscle 163	1.0121197	0.45646
fetal muscle 194	1.0137479	0.45846
fetal muscle 189	1.0106356	0.46070
fetal muscle 168	1.0113988	0.46376
fetal muscle 173	1.0097337	0.46500
fetal lung 110	1.0070347	0.46762
fetal muscle 145	1.0052526	0.46828
fetal stomach 252	1.0008060	0.46886
fetal adrenal gland 23	1.0059236	0.47180
fetal muscle 190	1.0024635	0.47220
fetal lung 137	1.0042043	0.47514
fetal large intestine 54	1.0024486	0.47544
fetal heart 50	1.0003281	0.47550
fetal stomach 248	0.9973812	0.47550
fetal muscle 161	1.0020026	0.47860
fetal muscle 192	1.0019755	0.47926
fetal lung 133	1.0049462	0.48036
fetal large intestine 53	0.9919883	0.48272
fetal muscle 188	0.9978926	0.48532
fetal muscle 171	0.9970330	0.49030
fetal large intestine 59	0.9908607	0.49156
fetal renal cortex 207	0.9931454	0.49242
fetal muscle 170	0.9950066	0.49442
fetal stomach 251	0.9829042	0.50076
fetal stomach 243	0.9858824	0.50238
ES derived mesoderm 286	0.9927352	0.50308
ES derived NP 292	0.9821841	0.50308
fetal renal pelvis 219	0.9746403	0.50402
fetal small intestine 76	0.9813340	0.50530
fetal large intestine 63	0.9823425	0.50724
fetal stomach 253	0.9770140	0.50768
fetal spinal cord 238	0.9848465	0.50898
fetal kidney 85	0.9867593	0.51030
fetal small intestine 72	0.9825252	0.51056
ES cells 296	0.9767463	0.51086
fetal lung 121	0.9854663	0.51724
fetal lung 108	0.9787808	0.51794
fetal renal pelvis 228	0.9727054	0.51972
fetal lung 125	0.9804497	0.52094
fetal small intestine 73	0.9732431	0.52234
fetal lung 129	0.9810931	0.52258
fetal stomach 242	0.9534333	0.52686
fetal renal cortex 213	0.9746637	0.52766
fetal muscle 144	0.9693579	0.53102
fetal lung 117	0.9777928	0.53182
fetal stomach 250	0.9545028	0.53698
fetal stomach 240	0.9487765	0.54082
fetal lung 138	0.9686687	0.54388
fetal lung 141	0.9740116	0.54448
fetal lung 115	0.9728675	0.54474
fetal renal pelvis 233	0.9584474	0.55216
fetal lung 109	0.9694095	0.55698
fetal lung 120	0.9611482	0.56322
ES derived NP 291	0.9422864	0.56324
iPS 304	0.9532271	0.57000
fetal spinal cord 235	0.9538471	0.57068
fetal lung 132	0.9559658	0.57208
fetal kidney 103	0.9428213	0.57282
fetal kidney 91	0.9504578	0.57412
fetal stomach 245	0.9357798	0.57586
fetal renal cortex 205	0.9452619	0.57654
fetal renal cortex 214	0.9447831	0.57784
fetal lung 127	0.9509977	0.57900
fetal lung 114	0.9575585	0.58016
fetal brain 32	0.9553992	0.58350
fetal testes 254	0.9403263	0.58430
fetal stomach 249	0.9199800	0.58812
fetal testes 255	0.9391866	0.59572
fetal lung 123	0.9398044	0.60130
fetal lung 124	0.9409534	0.60234
fetal lung 113	0.9446186	0.60446
fetal stomach 241	0.9092425	0.60514
fetal kidney 83	0.9119792	0.60732
fetal kidney 87	0.9256536	0.61356
fetal kidney 99	0.9202021	0.61560
iPS 303	0.9235913	0.62552
fetal lung 122	0.9218656	0.62714
fetal renal pelvis 232	0.9086592	0.62824
fetal kidney 95	0.9123443	0.63018
fetal stomach 246	0.8917178	0.63190
ES cells 294	0.8948889	0.63350
fetal kidney 100	0.9123758	0.63600
fetal spinal cord 239	0.9168610	0.63950
fetal stomach 247	0.8686048	0.64342
fetal lung 112	0.9169664	0.64464
fetal renal pelvis 230	0.8924750	0.64482
fetal lung 135	0.9185660	0.64730
fetal lung 136	0.9158332	0.65364
fetal lung 131	0.9235446	0.65662
fetal renal cortex 211	0.8912505	0.65738
fetal kidney 106	0.8993460	0.65878
fetal kidney 82	0.8826352	0.65946
fetal lung 142	0.9150124	0.66456
fetal kidney 86	0.8653724	0.66586
fetal kidney 102	0.8830986	0.67240
iPS 302	0.8961781	0.67330
fetal lung 130	0.9041421	0.67366
fetal kidney 92	0.8905644	0.67586
fetal kidney 89	0.8865696	0.68058
fetal lung 139	0.8918256	0.68762
fetal renal pelvis 217	0.8788951	0.68792
fetal kidney 84	0.8734804	0.69388
fetal renal pelvis 227	0.8752240	0.70112
fetal kidney 94	0.8615231	0.70334
ES cells 295	0.8563739	0.70488
fetal kidney 104	0.8648504	0.70732
fetal renal cortex 202	0.8718495	0.71124
ES cells 293	0.8562104	0.71372
fetal renal pelvis 229	0.8608108	0.72410
fetal renal pelvis 216	0.8568846	0.72466
fetal renal pelvis 218	0.8202307	0.72806
fetal renal pelvis 226	0.8595491	0.73108
fetal brain 33	0.8675293	0.74574
fetal renal pelvis 224	0.8509797	0.75114
fetal renal pelvis 231	0.8530001	0.75220
fetal renal cortex 204	0.8538485	0.75458
fetal renal pelvis 221	0.8460276	0.75656
fetal kidney 88	0.8482374	0.75890
fetal renal cortex 206	0.8434195	0.76050
fetal kidney 107	0.8347683	0.76518
fetal kidney 105	0.8297386	0.78432
fetal kidney 97	0.8118596	0.79804
fetal renal cortex 210	0.7984761	0.80338
fetal renal pelvis 223	0.7921552	0.80382
fetal renal cortex 203	0.8062330	0.80730
fetal renal cortex 212	0.8139690	0.81046
fetal renal cortex 209	0.7998661	0.81206
fetal renal cortex 208	0.7927636	0.81958
fetal kidney 93	0.7851872	0.82044
fetal kidney 98	0.7889898	0.82702
fetal renal pelvis 225	0.7804766	0.82858
fetal renal pelvis 222	0.7820273	0.83162
fetal kidney 96	0.7875951	0.84376
fetal renal cortex 215	0.7850829	0.84906
fetal kidney 90	0.7556798	0.86552
fetal kidney 101	0.7629882	0.86760

Prostate cancer susceptibility

DHS sample	fold enrichment	p value
fetal lung 114	1.411675	0.00164
fetal lung 118	1.408532	0.00164
fetal lung 131	1.406743	0.00178
fetal lung 119	1.412220	0.00258
fetal lung 140	1.414539	0.00286
fetal lung 126	1.388120	0.00312
fetal lung 116	1.381013	0.00312
fetal lung 124	1.417022	0.00344
fetal muscle 194	1.406227	0.00344
fetal lung 134	1.426681	0.00352
fetal lung 110	1.427133	0.00374
fetal lung 125	1.429504	0.00388
fetal lung 141	1.370203	0.00394
fetal lung 120	1.404405	0.00418
fetal lung 117	1.386965	0.00422
ES derived NP 291	1.552204	0.00426
fetal lung 112	1.407427	0.00432
fetal lung 139	1.418195	0.00484
iPS 302	1.453450	0.00488
fetal lung 132	1.397020	0.00498
fetal muscle 154	1.488250	0.00506
fetal lung 108	1.423212	0.00512
fetal lung 130	1.388919	0.00532
fetal muscle 151	1.473422	0.00684
fetal lung 121	1.342250	0.00694
fetal lung 142	1.349825	0.00712
fetal lung 138	1.384739	0.00734
fetal lung 127	1.389755	0.00742
fetal lung 111	1.358911	0.00742
fetal muscle 156	1.415037	0.00758
fetal lung 123	1.376053	0.00786
fetal lung 115	1.353890	0.00786
fetal lung 135	1.377033	0.00814
fetal lung 129	1.359086	0.00826
fetal muscle 157	1.379677	0.00850
fetal muscle 149	1.445033	0.00866
fetal lung 136	1.370787	0.00892
fetal muscle 192	1.399714	0.00904
fetal muscle 160	1.422131	0.00928
fetal muscle 153	1.444129	0.00978
fetal muscle 168	1.431610	0.00986
fetal muscle 181	1.406179	0.00990
fetal lung 113	1.336978	0.01028
iPS 304	1.373141	0.01030
fetal muscle 185	1.437794	0.01096
fetal lung 128	1.303867	0.01120
fetal stomach 248	1.480117	0.01126
fibroblast 330	1.348234	0.01142
fetal lung 133	1.322099	0.01176
fetal muscle 163	1.406834	0.01194
fetal lung 122	1.377167	0.01244
fetal muscle 173	1.402100	0.01258
fetal muscle 166	1.368035	0.01274
fetal muscle 172	1.333702	0.01284
fetal kidney 81	1.371559	0.01302
ES cells 293	1.431640	0.01312
fetal muscle 159	1.417119	0.01312
fetal muscle 155	1.417754	0.01322
fibroblast 329	1.331439	0.01342
fetal muscle 179	1.376832	0.01354
fetal muscle 164	1.385972	0.01356
fetal spinal cord 238	1.382447	0.01440
fetal muscle 193	1.390553	0.01544
fetal muscle 147	1.369441	0.01566
fetal muscle 150	1.382318	0.01648
fetal muscle 187	1.354140	0.01654
fetal thymus 263	1.387781	0.01688
fetal muscle 171	1.347330	0.01702
fetal muscle 191	1.377158	0.01712
fetal muscle 184	1.338172	0.01744
fetal muscle 148	1.361985	0.01762
fetal muscle 165	1.335452	0.01818
fetal muscle 152	1.338088	0.01872
ES cells 295	1.419088	0.01910
fibroblast 331	1.301350	0.01910
fetal muscle 167	1.338271	0.01912
fetal muscle 146	1.346689	0.01938
fetal muscle 180	1.373120	0.01992
fetal spinal cord 235	1.376277	0.02064
fetal muscle 175	1.345126	0.02076
ES cells 294	1.417034	0.02094
fetal muscle 143	1.319124	0.02108
fetal spinal cord 236	1.370277	0.02170
ES derived NP 292	1.395378	0.02192
fetal lung 137	1.296365	0.02196
ES cells 296	1.406477	0.02264
iPS 303	1.332880	0.02266
fetal stomach 243	1.360424	0.02334
fetal muscle 144	1.363266	0.02338
pancreas 327	1.397838	0.02434
fetal muscle 178	1.356133	0.02438
fetal muscle 182	1.331409	0.02466
fetal muscle 183	1.324804	0.02498
fibroblast 332	1.278243	0.02522
fetal lung 109	1.262573	0.02530
fetal muscle 170	1.325241	0.02538
fetal muscle 174	1.338672	0.02782
fetal muscle 145	1.375511	0.02822
fetal stomach 251	1.370343	0.02860
fetal stomach 249	1.399913	0.03000
fetal muscle 158	1.311464	0.03046
ES derived MSC 290	1.310808	0.03160
fetal spinal cord 239	1.309374	0.03284
fetal lung 300	1.279572	0.03318
fetal muscle 169	1.318406	0.03328
ES derived MSC 289	1.312223	0.03352
fetal muscle 188	1.306395	0.03370
fetal renal cortex 213	1.328514	0.03380
fetal muscle 186	1.307238	0.03404
fetal muscle 177	1.339151	0.03416
fetal kidney 107	1.331930	0.03438
Mobilized CD4 322	1.428833	0.03480
fetal lung 301	1.278598	0.03542
pancreas 328	1.348566	0.03580
fetal testes 255	1.300964	0.03584
fetal renal pelvis 226	1.329458	0.03602
ES derived mesoderm 285	1.253745	0.03634
muscle 341	1.358710	0.03662
fetal kidney 87	1.317985	0.03670
placenta 197	1.304428	0.03722
fetal muscle 189	1.298848	0.03766
fetal muscle 176	1.281908	0.03788
fetal kidney 82	1.357713	0.03812
fetal renal cortex 215	1.311137	0.03816
placenta 200	1.259620	0.03818
fetal adrenal gland 25	1.329182	0.03820
fetal renal pelvis 229	1.323701	0.03862
fetal stomach 240	1.379036	0.03890
fetal kidney 104	1.324666	0.03910
fetal adrenal gland 24	1.353673	0.03924
fetal kidney 98	1.325819	0.03978
fetal kidney 99	1.330594	0.04008
fetal kidney 105	1.309249	0.04126
stomach 283	1.333127	0.04174
stomach 284	1.328095	0.04190
fetal kidney 84	1.328294	0.04216
fetal thymus 258	1.356656	0.04240
fetal muscle 161	1.303652	0.04240
fetal stomach 246	1.352124	0.04252
fetal kidney 100	1.322550	0.04252
fetal renal pelvis 228	1.340700	0.04290
placenta 199	1.270516	0.04422
fetal thymus 256	1.341628	0.04468
fetal muscle 162	1.274408	0.04486
fetal renal cortex 206	1.295683	0.04510
Mobilized CD3 320	1.409711	0.04512
fetal kidney 89	1.319068	0.04536
ES derived mesoderm 286	1.219889	0.04552
fetal lung 298	1.265040	0.04562
placenta 198	1.262465	0.04584
fetal renal pelvis 223	1.330454	0.04586
placenta 196	1.291406	0.04762
fetal stomach 253	1.338235	0.04794
fetal testes 254	1.294140	0.04808
fetal brain 38	1.239668	0.04884
fetal renal cortex 210	1.316965	0.04894
fetal renal cortex 212	1.280609	0.04968
fetal heart 44	1.331829	0.04986
fetal ovary 195	1.355877	0.05038
fetal renal pelvis 233	1.301751	0.05052
fetal renal pelvis 225	1.306506	0.05178
fetal small intestine 76	1.313323	0.05186
fetal kidney 90	1.299245	0.05242
fetal thymus 257	1.358273	0.05338
fetal kidney 101	1.283451	0.05450
ES derived trophoblast 288	1.248108	0.05452
fetal renal cortex 208	1.299071	0.05478
fetal muscle 190	1.297012	0.05522
breast 1	1.206880	0.05526
fetal renal cortex 209	1.294706	0.05538
fetal stomach 252	1.301938	0.05586
fetal kidney 94	1.313334	0.05604
fetal heart 46	1.279997	0.05604
fetal kidney 96	1.275581	0.05614
heart 297	1.301424	0.05656
fetal stomach 245	1.306393	0.05690
fetal thymus 266	1.318839	0.05716
fetal small intestine 71	1.290043	0.05758
fetal renal pelvis 216	1.290754	0.05760
fetal kidney 92	1.280455	0.05764
fetal stomach 250	1.322890	0.05776
fetal stomach 241	1.318886	0.05796
fetal stomach 242	1.367136	0.05806
fetal brain 32	1.219558	0.05806
fetal renal cortex 214	1.286349	0.05834
fetal kidney 106	1.283571	0.05864
fetal heart 51	1.260977	0.05936
fetal renal cortex 205	1.279623	0.05962
fetal heart 48	1.260118	0.05962
fetal heart 50	1.265394	0.06044
fetal renal pelvis 217	1.296314	0.06058
CD3 9	1.340080	0.06100
fetal kidney 97	1.279986	0.06106
fetal heart 42	1.258949	0.06168
fetal renal pelvis 230	1.301459	0.06324
fetal thymus 264	1.307417	0.06326
fetal renal pelvis 231	1.261084	0.06400
fetal renal cortex 202	1.268917	0.06410
fetal lung 299	1.236100	0.06430
fetal kidney 93	1.287993	0.06500
fetal adrenal gland 26	1.259506	0.06606
fetal renal cortex 207	1.271860	0.06758
fetal renal pelvis 221	1.262064	0.06852
fetal kidney 86	1.315879	0.06974
keratinocyte 334	1.174900	0.07024
fetal renal pelvis 227	1.264561	0.07068
fetal spinal cord 237	1.232056	0.07122
fetal kidney 85	1.246077	0.07176
fetal renal pelvis 222	1.273264	0.07186
CD3 10	1.298114	0.07196
fetal large intestine 61	1.311268	0.07252
fetal small intestine 75	1.398659	0.07292
hematopoietic stem cell 314	1.223533	0.07428
fetal renal pelvis 224	1.243803	0.07496
fetal large intestine 66	1.273886	0.07510
fetal renal cortex 211	1.278735	0.07516
fetal heart 52	1.223206	0.07624
fetal renal pelvis 218	1.311585	0.07782
fetal thymus 265	1.288816	0.07794
fetal kidney 83	1.286356	0.07824
hematopoietic stem cell 308	1.224317	0.07858
fetal renal pelvis 219	1.302633	0.07978
hematopoietic stem cell 313	1.222129	0.07998
fetal renal pelvis 220	1.254661	0.08016
fetal renal cortex 203	1.251679	0.08206
fetal kidney 102	1.269899	0.08222
breast 2	1.178990	0.08322
fetal stomach 244	1.256067	0.08400
fetal small intestine 78	1.219792	0.08402
fetal thymus 261	1.290432	0.08418
fetal adrenal gland 23	1.222902	0.08524
hematopoietic stem cell 306	1.225549	0.08604
fetal heart 43	1.218977	0.08616
hematopoietic stem cell 318	1.236039	0.08670
fetal large intestine 53	1.275627	0.08702
hematopoietic stem cell 317	1.194568	0.08714
fetal kidney 103	1.250735	0.08792
fetal stomach 247	1.300550	0.08852
hematopoietic stem cell 315	1.230634	0.08862
fetal small intestine 74	1.233486	0.08932
fetal renal cortex 204	1.220472	0.08982
CD20 8	1.222971	0.09014
fetal thymus 262	1.338928	0.09016
fetal thymus 260	1.269065	0.09036
fetal adrenal gland 27	1.228233	0.09070
hematopoietic stem cell 307	1.217230	0.09200
fetal large intestine 57	1.213765	0.09222
ES derived trophoblast 287	1.223602	0.09236
placenta 201	1.237860	0.09428
fetal kidney 95	1.234414	0.09488
fetal large intestine 65	1.236883	0.09636
fetal brain 33	1.193541	0.09642
fetal brain 36	1.183136	0.09672
fetal large intestine 59	1.242612	0.09698
fetal small intestine 70	1.202985	0.09698
CD4 13	1.294378	0.09728
fetal kidney 88	1.219440	0.09852
hematopoietic stem cell 305	1.213274	0.09866
fetal renal pelvis 232	1.238257	0.09986
fetal small intestine 72	1.222983	0.10034
CD3 11	1.254757	0.10402
keratinocyte 336	1.148071	0.10450
fetal small intestine 79	1.234285	0.10544
fetal kidney 91	1.211022	0.10568
CD4 16	1.288638	0.10584
ovary 326	1.266310	0.10612
CD3 12	1.283354	0.10702
Mobilized CD4 323	1.272158	0.10776
hematopoietic stem cell 311	1.198643	0.10804
fetal adrenal gland 28	1.185998	0.11078
fetal large intestine 56	1.211676	0.11098
fetal large intestine 67	1.218457	0.11100
Mobilized CD8 325	1.275275	0.11190
Mobilized CD3 321	1.287900	0.11226
keratinocyte 333	1.140077	0.11272
fetal heart 49	1.202492	0.11312
fetal large intestine 64	1.201082	0.11556
CD14 3	1.244844	0.11698
fetal thymus 259	1.257952	0.11782
fetal small intestine 77	1.214796	0.11782
hematopoietic stem cell 310	1.167937	0.12118
fetal small intestine 68	1.191970	0.12406
fetal large intestine 62	1.219416	0.12664
CD8 22	1.253765	0.12802
fetal large intestine 63	1.207173	0.12936
CD56 18	1.251194	0.12976
fetal small intestine 69	1.211103	0.12986
CD4 14	1.220810	0.13152
keratinocyte 335	1.133079	0.13202
fetal fibroblast 282	1.144173	0.13250
fetal small intestine 80	1.181602	0.13398
hematopoietic stem cell 309	1.176769	0.13638
fetal skin 234	1.185238	0.13756
CD8 20	1.218506	0.13834
CD4 15	1.281757	0.13860
hematopoietic stem cell 319	1.190917	0.14054
fetal fibroblast 272	1.144000	0.14102
CD8 19	1.253883	0.14118
CD19 7	1.231848	0.14252
fetal large intestine 60	1.184428	0.14270
fetal small intestine 73	1.188961	0.14348
hematopoietic stem cell 316	1.180722	0.14440
fetal heart 45	1.173472	0.14512
fetal large intestine 54	1.184332	0.14610
fetal fibroblast 267	1.149938	0.14660
CD8 21	1.225922	0.15180
fetal large intestine 55	1.163893	0.15496
CD19 6	1.214172	0.15794
melanocyte 340	1.135432	0.16496
CD56 17	1.184661	0.16542
fetal brain 41	1.122522	0.17698
fetal large intestine 58	1.137261	0.18876
melanocyte 338	1.107560	0.18894
fetal brain 37	1.113996	0.18988
CD19 5	1.176749	0.19130
fetal fibroblast 271	1.115371	0.19316
Mobilized CD56 324	1.169133	0.19380
hematopoietic stem cell 312	1.133818	0.19544
fetal fibroblast 268	1.110020	0.20048
fetal fibroblast 274	1.110170	0.20260
fetal fibroblast 273	1.108710	0.20680
small intestine 342	1.133435	0.20998
fetal heart 47	1.146126	0.21018
fetal brain 31	1.088891	0.21502
fetal brain 40	1.098735	0.21858
fetal fibroblast 278	1.101330	0.23100
fetal fibroblast 281	1.096142	0.23200
fetal fibroblast 276	1.088351	0.24572
CD14 4	1.125611	0.25178
fetal fibroblast 277	1.085359	0.26626
fetal fibroblast 275	1.072840	0.28306
fetal brain 34	1.065339	0.29462
fetal brain 39	1.059339	0.31390
melanocyte 337	1.056039	0.31662
fetal brain 35	1.053508	0.31892
fetal fibroblast 269	1.055707	0.32812
fetal fibroblast 279	1.057227	0.33090
melanocyte 339	1.044595	0.35380
fetal fibroblast 270	1.047564	0.35570
fetal fibroblast 280	1.043916	0.36422
fetal brain 29	1.017675	0.42662
fetal brain 30	1.006698	0.46582

Lung function

DHS sample	fold enrichment	p value
fibroblast 331	1.3723550	0.02104
fibroblast 332	1.3686851	0.02110
fetal fibroblast 277	1.3794277	0.02288
fetal muscle 166	1.4005820	0.02510
fetal fibroblast 282	1.3436204	0.02700
fetal fibroblast 268	1.3380863	0.03020
fibroblast 330	1.3500260	0.03116
keratinocyte 334	1.3057821	0.03192
keratinocyte 336	1.3023239	0.03360
keratinocyte 335	1.3055827	0.03394
fetal fibroblast 274	1.3343782	0.03542
fetal lung 141	1.3335729	0.03574
fetal lung 111	1.3357319	0.03734
fetal heart 45	1.3718539	0.03788
fetal muscle 169	1.3791682	0.03822
fetal fibroblast 273	1.3183463	0.04116
fetal muscle 176	1.3411178	0.04140
fetal fibroblast 281	1.3141126	0.04384
fetal fibroblast 267	1.3226962	0.04748
fetal fibroblast 278	1.3084614	0.04950
CD56 18	1.4979010	0.05138
fibroblast 329	1.3034372	0.05142
fetal muscle 152	1.3317135	0.05186
fetal muscle 179	1.3312329	0.05218
breast 1	1.2866292	0.05324
keratinocyte 333	1.2588971	0.05470
fetal lung 114	1.2862959	0.05482
fetal muscle 143	1.2978196	0.05914
fetal heart 47	1.3530549	0.05968
fetal fibroblast 270	1.2893025	0.06060
fetal heart 49	1.3154267	0.06296
fetal fibroblast 276	1.2615834	0.07276
fetal fibroblast 272	1.2639651	0.07344
fetal muscle 181	1.2953318	0.07600
fetal fibroblast 271	1.2604271	0.07606
fetal lung 133	1.2660819	0.07812
fetal muscle 160	1.2996649	0.07898
fetal lung 140	1.2721967	0.07900
fetal fibroblast 280	1.2553946	0.07910
breast 2	1.2486482	0.07970
fetal fibroblast 269	1.2477106	0.08114
stomach 284	1.3294019	0.08322
fetal lung 117	1.2581828	0.08344
fetal lung 119	1.2536136	0.08530
muscle 341	1.3283106	0.08620
fetal muscle 164	1.2837544	0.08814
fetal lung 300	1.2603918	0.08848
fetal lung 129	1.2602617	0.09144
fetal heart 44	1.3221349	0.09458
fetal muscle 183	1.2570017	0.09672
fetal muscle 187	1.2451819	0.09826
fetal heart 46	1.2739750	0.09870
fetal muscle 182	1.2590917	0.09870
fetal muscle 153	1.2813936	0.09928
fetal lung 301	1.2543823	0.09930
fetal muscle 151	1.2771873	0.09970
fetal muscle 167	1.2522116	0.10080
fetal lung 128	1.2159228	0.10100
fetal testes 255	1.2623536	0.10114
fetal muscle 168	1.2671748	0.10154
fetal muscle 154	1.2824870	0.10306
pancreas 328	1.2967526	0.10396
fetal thymus 264	1.3045192	0.10568
fetal fibroblast 279	1.2281760	0.10594
ES derived MSC 289	1.2668773	0.10748
fetal muscle 155	1.2708234	0.10780
fetal fibroblast 275	1.2167622	0.10940
melanocyte 338	1.2074295	0.11008
fetal muscle 163	1.2557782	0.11116
fetal muscle 175	1.2445126	0.11324
fetal thymus 265	1.2957255	0.11442
fetal lung 130	1.2263031	0.11728
fetal muscle 158	1.2364686	0.11804
fetal muscle 170	1.2307980	0.11814
fetal muscle 165	1.2299343	0.11840
placenta 200	1.2272843	0.11846
CD3 12	1.3503165	0.11898
fetal muscle 157	1.2210850	0.12022
fetal muscle 180	1.2518100	0.12090
stomach 283	1.2773364	0.12188
ES derived MSC 290	1.2443832	0.12194
Mobilized CD4 323	1.3414188	0.12196
fetal kidney 81	1.2246408	0.12448
fetal lung 124	1.2215803	0.12524
fetal lung 299	1.2301255	0.12538
fetal adrenal gland 26	1.2442586	0.12586
hematopoietic stem cell 314	1.2307900	0.12654
fetal lung 118	1.2000841	0.12692
fetal muscle 150	1.2421097	0.12804
fetal muscle 184	1.2128816	0.12916
fetal lung 116	1.1963698	0.12960
fetal muscle 148	1.2346477	0.12974
heart 297	1.2558584	0.12988
CD3 11	1.2905349	0.13100
pancreas 327	1.2722777	0.13304
fetal stomach 244	1.2598908	0.13536
fetal muscle 178	1.2265123	0.13764
fetal heart 52	1.2060896	0.13884
ES derived trophoblast 287	1.2250523	0.14010
hematopoietic stem cell 310	1.2005508	0.14162
fetal muscle 185	1.2324551	0.14204
fetal muscle 188	1.2130952	0.14274
fetal thymus 256	1.2515797	0.14282
fetal lung 113	1.1895457	0.14342
fetal muscle 186	1.2117866	0.14474
fetal muscle 149	1.2217139	0.14516
fetal lung 121	1.1849292	0.14892
fetal thymus 259	1.2657131	0.14908
fetal muscle 171	1.2045626	0.14962
ES derived trophoblast 288	1.1980993	0.14970
CD3 9	1.2902606	0.14984
fetal lung 120	1.1935984	0.15030
fetal spinal cord 235	1.2004694	0.15044
fetal heart 43	1.1958224	0.15186
fetal adrenal gland 23	1.2014742	0.15204
fetal heart 48	1.2049425	0.15224
fetal muscle 161	1.2103661	0.15256
fetal muscle 156	1.2042041	0.15276
fetal thymus 263	1.2154867	0.15320
fetal muscle 146	1.2035839	0.15326
fetal adrenal gland 25	1.2229262	0.15390
fetal muscle 147	1.2091403	0.15448
fetal lung 298	1.2041674	0.15642
fetal muscle 190	1.2186027	0.15666
fetal heart 51	1.1991955	0.15690
Mobilized CD4 322	1.2891228	0.15814
fetal adrenal gland 27	1.2033483	0.16100
fetal testes 254	1.2088701	0.16166
CD3 10	1.2570129	0.16248
CD8 20	1.2467006	0.16414
ES derived mesoderm 286	1.1607270	0.16482
fetal spinal cord 236	1.1955853	0.16490
fetal large intestine 67	1.2208974	0.16570
fetal muscle 172	1.1763478	0.16668
fetal lung 125	1.1855486	0.16672
hematopoietic stem cell 308	1.1925594	0.16702
CD20 8	1.2055533	0.16872
Mobilized CD56 324	1.2523419	0.16888
CD19 6	1.2639503	0.16898
fetal stomach 248	1.2242755	0.17060
fetal heart 42	1.1900214	0.17074
fetal small intestine 68	1.2048110	0.17100
fetal muscle 189	1.1887342	0.17234
fetal muscle 162	1.1809002	0.17258
hematopoietic stem cell 318	1.2037635	0.17266
fetal small intestine 70	1.1894909	0.17268
CD19 5	1.2450356	0.17296
CD4 14	1.2369746	0.17322
CD8 22	1.2559697	0.17650
fetal stomach 246	1.2268472	0.17802
CD8 21	1.2670650	0.17890
fetal thymus 257	1.2309779	0.17982
fetal adrenal gland 28	1.1665414	0.17998
CD56 17	1.2195913	0.18046
CD4 13	1.2533194	0.18070
CD8 19	1.2776428	0.18158
fetal thymus 258	1.2189809	0.18294
fetal large intestine 53	1.2229089	0.18446
fetal muscle 194	1.1658728	0.18454
fetal lung 109	1.1486788	0.18524
ES derived mesoderm 285	1.1570637	0.18534
fetal small intestine 74	1.1981834	0.18746
hematopoietic stem cell 305	1.1840656	0.18902
fetal thymus 266	1.2054849	0.19134
fetal lung 138	1.1645585	0.19178
iPS 304	1.1686261	0.19240
fetal muscle 173	1.1780615	0.19262
placenta 197	1.1889556	0.19314
Mobilized CD8 325	1.2443768	0.19320
hematopoietic stem cell 316	1.1872882	0.19490
placenta 198	1.1645359	0.19544
fetal muscle 191	1.1731183	0.19568
Mobilized CD3 320	1.2421533	0.19684
hematopoietic stem cell 307	1.1744098	0.19730
fetal lung 131	1.1431038	0.19922
fetal large intestine 59	1.1921440	0.19928
fetal lung 134	1.1582461	0.20018
small intestine 342	1.1843615	0.20144
fetal muscle 145	1.1818096	0.20280
fetal small intestine 77	1.1885354	0.20310
fetal lung 142	1.1438590	0.20334
fetal large intestine 57	1.1698497	0.20422
CD19 7	1.2267920	0.20426
fetal lung 110	1.1578765	0.20556
fetal lung 123	1.1562042	0.20610
fetal muscle 192	1.1558511	0.20744
fetal stomach 241	1.1900165	0.20816
hematopoietic stem cell 317	1.1445940	0.20826
melanocyte 339	1.1369513	0.20932
fetal lung 115	1.1430743	0.20970
fetal small intestine 78	1.1618191	0.21274
fetal large intestine 61	1.2024541	0.21318
fetal large intestine 58	1.1660901	0.21340
fetal renal cortex 213	1.1551576	0.21356
placenta 199	1.1581548	0.21404
fetal renal cortex 207	1.1661714	0.21556
hematopoietic stem cell 309	1.1612542	0.21562
fetal lung 136	1.1464270	0.21840
fetal large intestine 62	1.1771966	0.21902
melanocyte 340	1.1378034	0.22060
CD4 15	1.2355106	0.22124
fetal lung 126	1.1320492	0.22220
fetal ovary 195	1.1848478	0.22274
fetal adrenal gland 24	1.1695206	0.22364
fetal small intestine 73	1.1653823	0.22484
fetal muscle 177	1.1577093	0.22506
hematopoietic stem cell 312	1.1499099	0.22534
fetal small intestine 80	1.1598176	0.22540
hematopoietic stem cell 311	1.1497621	0.22578
melanocyte 337	1.1214934	0.22638
Mobilized CD3 321	1.2123269	0.22722
fetal muscle 144	1.1516010	0.23052
ES derived NP 292	1.1627086	0.23070
fetal large intestine 60	1.1628507	0.23132
fetal muscle 174	1.1469413	0.23214
fetal muscle 193	1.1462284	0.23332
fetal small intestine 71	1.1575553	0.23544
hematopoietic stem cell 306	1.1442249	0.23726
hematopoietic stem cell 313	1.1341811	0.23782
fetal thymus 260	1.1680883	0.23930
fetal brain 36	1.1148096	0.23946
fetal heart 50	1.1378482	0.23972
fetal lung 139	1.1372887	0.24030
fetal small intestine 72	1.1489744	0.24214
fetal lung 137	1.1241162	0.24520
fetal renal pelvis 220	1.1414402	0.24598
fetal stomach 253	1.1586237	0.24612
fetal stomach 250	1.1601553	0.24646
fetal large intestine 56	1.1454883	0.24828
fetal lung 112	1.1246320	0.25032
fetal large intestine 55	1.1399107	0.25128
fetal kidney 85	1.1200440	0.25288
CD14 3	1.1669640	0.25308
fetal lung 108	1.1267893	0.25480
fetal stomach 247	1.1635393	0.25536
placenta 196	1.1354274	0.25598
fetal kidney 92	1.1287927	0.25600
fetal stomach 240	1.1518226	0.25670
placenta 201	1.1408796	0.25854
fetal lung 132	1.1194122	0.25978
CD14 4	1.1493517	0.26164
fetal thymus 261	1.1509406	0.26228
fetal large intestine 54	1.1358483	0.26432
fetal lung 135	1.1163909	0.26442
fetal muscle 159	1.1283562	0.26482
fetal stomach 245	1.1402060	0.26484
fetal stomach 243	1.1301691	0.26730
fetal small intestine 69	1.1338365	0.27020
fetal large intestine 64	1.1234281	0.27546
CD4 16	1.1521835	0.27606
fetal brain 33	1.0997693	0.27618
fetal kidney 91	1.1102036	0.27626
fetal renal pelvis 226	1.1136879	0.27682
hematopoietic stem cell 315	1.1210641	0.27712
fetal stomach 249	1.1332106	0.27948
fetal renal pelvis 221	1.1106656	0.28018
fetal spinal cord 237	1.1042268	0.28296
fetal stomach 251	1.1189453	0.28746
fetal spinal cord 238	1.0987329	0.28780
fetal brain 29	1.0717133	0.28848
fetal thymus 262	1.1430396	0.28878
iPS 302	1.1069055	0.28878
fetal kidney 95	1.1062823	0.29338
fetal stomach 252	1.1091684	0.29972
fetal stomach 242	1.1241908	0.30182
fetal brain 41	1.0787252	0.30404
hematopoietic stem cell 319	1.1012742	0.30772
ES derived NP 291	1.1086908	0.30790
ovary 326	1.1153935	0.30796
fetal brain 30	1.0577313	0.32350
fetal renal pelvis 227	1.0839608	0.32474
fetal large intestine 66	1.0918399	0.32698
fetal kidney 84	1.0849169	0.33028
fetal lung 127	1.0766342	0.33224
fetal renal cortex 202	1.0801264	0.33306
fetal brain 35	1.0564307	0.33658
fetal kidney 100	1.0784736	0.33666
iPS 303	1.0745498	0.33960
ES cells 294	1.0845603	0.33998
fetal small intestine 75	1.0895189	0.34008
fetal spinal cord 239	1.0696524	0.34248
fetal kidney 103	1.0740494	0.34450
fetal small intestine 79	1.0795043	0.34796
fetal brain 38	1.0575395	0.35616
fetal renal pelvis 232	1.0687041	0.35858
fetal kidney 97	1.0659713	0.35908
fetal kidney 94	1.0675508	0.35986
fetal large intestine 63	1.0690152	0.36234
ES cells 293	1.0669037	0.36354
ES cells 296	1.0684135	0.36672
fetal kidney 102	1.0624642	0.36718
fetal kidney 89	1.0591780	0.37072
fetal brain 34	1.0456781	0.37170
fetal brain 32	1.0483276	0.37352
fetal renal pelvis 229	1.0533687	0.37718
fetal kidney 87	1.0522907	0.37760
fetal renal cortex 214	1.0536476	0.37828
fetal large intestine 65	1.0565144	0.38336
fetal kidney 82	1.0515308	0.38438
fetal lung 122	1.0486370	0.38526
fetal renal pelvis 217	1.0492995	0.38592
fetal renal pelvis 230	1.0474178	0.39198
fetal renal pelvis 231	1.0446490	0.39278
fetal renal cortex 212	1.0444118	0.39368
fetal kidney 105	1.0434705	0.39632
fetal renal pelvis 222	1.0428032	0.39794
fetal kidney 86	1.0444068	0.39810
fetal small intestine 76	1.0453645	0.40130
fetal kidney 99	1.0397049	0.40144
fetal kidney 83	1.0411383	0.40328
fetal renal pelvis 219	1.0441967	0.40446
fetal kidney 98	1.0375233	0.40662
fetal renal pelvis 228	1.0380602	0.40870
fetal brain 31	1.0257890	0.41328
fetal kidney 101	1.0349283	0.41340
fetal renal cortex 203	1.0338277	0.41512
fetal renal cortex 215	1.0311873	0.41598
fetal renal pelvis 233	1.0324111	0.41634
fetal renal pelvis 225	1.0306748	0.41802
fetal renal pelvis 223	1.0309418	0.42016
fetal kidney 104	1.0311420	0.42120
fetal kidney 90	1.0296760	0.42230
fetal kidney 107	1.0282107	0.42294
fetal renal cortex 205	1.0269105	0.42760
fetal brain 40	1.0235030	0.42986
fetal renal pelvis 216	1.0192598	0.43970
fetal renal cortex 204	1.0208671	0.44122
fetal renal cortex 209	1.0153302	0.44656
fetal renal cortex 208	1.0170312	0.44710
fetal brain 39	1.0156409	0.44808
fetal kidney 88	1.0129265	0.45012
fetal kidney 106	1.0083975	0.45848
fetal kidney 96	1.0068472	0.46502
ES cells 295	0.9964550	0.48030
fetal renal pelvis 224	0.9980572	0.48334
fetal renal cortex 206	0.9960939	0.48588
fetal renal cortex 210	0.9919103	0.48766
fetal brain 37	0.9953395	0.49550
fetal kidney 93	0.9879259	0.49608
fetal renal cortex 211	0.9724991	0.51980
fetal renal pelvis 218	0.9485760	0.54634
fetal skin 234	0.9469177	0.59058

Age at menopause

DHS sample	fold enrichment	p value
Mobilized CD56 324	1.4395441	0.02808
ES cells 294	1.3049565	0.08616
fetal brain 36	1.2067116	0.10552
CD3 10	1.2634591	0.11266
CD19 5	1.2698684	0.11452
CD19 7	1.2869622	0.11616
CD19 6	1.2809804	0.11666
fetal adrenal gland 28	1.1963832	0.11756
Mobilized CD3 320	1.2955450	0.12280
ES derived NP 291	1.2545874	0.12298
CD3 9	1.2643043	0.12432
ES derived mesoderm 285	1.1730812	0.14368
ES cells 296	1.2235725	0.14534
CD56 18	1.2470039	0.14594
Mobilized CD4 323	1.2379922	0.14892
Mobilized CD4 322	1.2455013	0.15012
keratinocyte 335	1.1423432	0.15066
CD3 11	1.2110486	0.15408
keratinocyte 333	1.1378103	0.15448
fetal large intestine 61	1.2249573	0.15786
CD4 13	1.2282474	0.16346
CD8 19	1.2383367	0.16764
CD8 21	1.2234855	0.16982
fetal brain 40	1.1435528	0.17538
fetal brain 37	1.1431839	0.18826
CD4 14	1.1774040	0.19014
ES derived NP 292	1.1729727	0.19256
iPS 304	1.1398122	0.19822
fetal spinal cord 239	1.1400908	0.20094
fetal adrenal gland 27	1.1428607	0.20350
fetal brain 35	1.1139834	0.20450
ES cells 295	1.1630223	0.20978
Mobilized CD3 321	1.1815620	0.21840
fetal brain 31	1.1007184	0.22214
fetal adrenal gland 24	1.1458847	0.22504
fetal thymus 256	1.1429633	0.23060
CD3 12	1.1625742	0.23366
iPS 302	1.1171782	0.24350
keratinocyte 336	1.0933825	0.24394
iPS 303	1.1143038	0.24534
ES cells 293	1.1265068	0.24596
Mobilized CD8 325	1.1543076	0.24878
CD4 15	1.1706964	0.24880
CD8 20	1.1298828	0.25758
fetal brain 38	1.1006249	0.26096
fetal thymus 259	1.1369918	0.26156
fetal brain 34	1.0862344	0.26902
fetal adrenal gland 25	1.1093133	0.27094
hematopoietic stem cell 314	1.0955407	0.27102
keratinocyte 334	1.0766041	0.28386
ES derived trophoblast 287	1.1006284	0.29062
fetal thymus 258	1.1054399	0.29626
CD8 22	1.1083121	0.30368
fetal ovary 195	1.1003380	0.30614
fetal thymus 260	1.0986697	0.30890
fetal thymus 257	1.0961323	0.31768
ES derived mesoderm 286	1.0647659	0.31844
fetal adrenal gland 26	1.0767295	0.32344
fetal thymus 264	1.0838739	0.32904
fetal spinal cord 238	1.0628066	0.33834
fetal spinal cord 235	1.0658792	0.33974
fetal thymus 265	1.0774160	0.34328
breast 1	1.0551545	0.34364
fetal skin 234	1.0703696	0.34506
fetal adrenal gland 23	1.0602124	0.34656
fetal small intestine 75	1.0830977	0.34794
CD4 16	1.0788238	0.34902
fetal stomach 248	1.0670524	0.35528
fetal small intestine 69	1.0655970	0.36032
ES derived trophoblast 288	1.0549057	0.36368
melanocyte 338	1.0445050	0.36930
breast 2	1.0422971	0.37594
fetal heart 47	1.0516514	0.37666
fetal renal cortex 207	1.0491483	0.38032
hematopoietic stem cell 308	1.0459493	0.38076
fetal spinal cord 236	1.0463010	0.38270
fetal small intestine 80	1.0455144	0.39328
fetal brain 30	1.0305516	0.39938
fetal brain 32	1.0331222	0.40410
fetal thymus 261	1.0406608	0.40846
fetal brain 33	1.0288999	0.41546
fetal stomach 244	1.0343628	0.41584
fetal kidney 103	1.0289876	0.42030
fetal brain 41	1.0235195	0.42184
hematopoietic stem cell 305	1.0287203	0.42428
fetal stomach 247	1.0268363	0.42580
fetal brain 39	1.0139189	0.44496
fetal renal cortex 213	1.0175611	0.44552
stomach 284	1.0193432	0.44690
CD14 3	1.0209653	0.44796
fetal kidney 95	1.0145230	0.44986
small intestine 342	1.0152213	0.45224
fetal spinal cord 237	1.0108783	0.45932
fetal stomach 242	1.0078982	0.46282
fetal kidney 91	1.0097462	0.46344
CD56 17	1.0127048	0.46406
ES derived MSC 290	1.0082150	0.46672
hematopoietic stem cell 319	1.0094610	0.46696
CD14 4	1.0090047	0.46912
fetal thymus 266	1.0078402	0.46998
fetal large intestine 64	1.0082229	0.47010
CD20 8	1.0071459	0.47216
fetal thymus 262	0.9977880	0.47356
hematopoietic stem cell 315	1.0056579	0.47646
fetal kidney 85	1.0017787	0.47646
hematopoietic stem cell 309	1.0040932	0.47936
fetal kidney 84	0.9995462	0.48152
melanocyte 337	1.0033571	0.48210
fetal stomach 253	0.9975859	0.48314
fetal large intestine 60	1.0020478	0.48364
fetal stomach 252	0.9962360	0.48544
fetal kidney 94	0.9968823	0.48558
hematopoietic stem cell 318	0.9994056	0.48666
fetal large intestine 57	0.9971201	0.49370
fetal kidney 83	0.9942272	0.49452
fetal lung 299	0.9977509	0.49624
fetal large intestine 67	0.9944827	0.49686
fetal stomach 246	0.9868127	0.50232
fetal heart 45	0.9894354	0.50358
fetal large intestine 62	0.9856915	0.51332
fetal small intestine 68	0.9861634	0.51692
fetal stomach 240	0.9756136	0.52278
placenta 199	0.9830880	0.52596
fibroblast 330	0.9849713	0.52608
fetal large intestine 63	0.9807590	0.52628
hematopoietic stem cell 310	0.9882740	0.52684
fetal small intestine 74	0.9811515	0.52932
ES derived MSC 289	0.9819546	0.52938
fetal large intestine 54	0.9768968	0.53430
fetal large intestine 56	0.9765274	0.53462
fetal kidney 92	0.9784333	0.53536
fetal small intestine 73	0.9754251	0.53554
hematopoietic stem cell 307	0.9779556	0.53922
fetal lung 298	0.9788657	0.54072
fetal small intestine 78	0.9781243	0.54152
pancreas 328	0.9696740	0.54384
hematopoietic stem cell 306	0.9752951	0.54408
fetal renal cortex 214	0.9728246	0.54522
fetal renal pelvis 228	0.9679374	0.54658
fetal small intestine 72	0.9710223	0.54700
fetal brain 29	0.9801665	0.54702
hematopoietic stem cell 316	0.9707879	0.54982
fetal large intestine 58	0.9712964	0.55414
fetal stomach 241	0.9591215	0.55652
fetal testes 255	0.9698708	0.55828
fibroblast 331	0.9718519	0.56076
fetal renal cortex 202	0.9639106	0.56560
fetal testes 254	0.9645895	0.56578
placenta 198	0.9679948	0.56920
placenta 200	0.9681810	0.56980
fetal lung 301	0.9655010	0.57074
fetal renal pelvis 232	0.9568134	0.57326
melanocyte 340	0.9672588	0.57434
fetal renal pelvis 219	0.9456958	0.57562
fetal renal pelvis 230	0.9526638	0.57616
fetal kidney 98	0.9553461	0.58044
fetal kidney 89	0.9545546	0.58160
fetal renal cortex 204	0.9604802	0.58234
fetal stomach 243	0.9531599	0.58272
fetal small intestine 70	0.9623968	0.58326
fetal kidney 82	0.9478068	0.58376
placenta 197	0.9552511	0.58474
fetal renal cortex 208	0.9516772	0.58754
fetal renal cortex 206	0.9542776	0.58920
fetal kidney 99	0.9511385	0.59328
fetal renal cortex 212	0.9531694	0.59398
fetal renal cortex 209	0.9484955	0.59656
fetal kidney 96	0.9486459	0.60046
fetal stomach 251	0.9400703	0.60154
fetal kidney 104	0.9416318	0.60908
fetal kidney 86	0.9286492	0.61068
fetal heart 49	0.9419755	0.61196
fetal renal pelvis 226	0.9433009	0.61272
fetal large intestine 65	0.9390240	0.61304
fetal kidney 107	0.9391593	0.61540
fetal renal cortex 211	0.9339303	0.61720
hematopoietic stem cell 312	0.9473034	0.61962
fetal small intestine 79	0.9336146	0.61984
fetal lung 112	0.9473499	0.62046
fibroblast 329	0.9479160	0.62172
fetal renal pelvis 217	0.9348520	0.62274
melanocyte 339	0.9511025	0.62368
fetal large intestine 53	0.9238456	0.62440
fetal renal pelvis 224	0.9391431	0.62512
fetal large intestine 59	0.9298352	0.62544
fetal heart 44	0.9263197	0.62636
muscle 341	0.9264631	0.62746
fetal muscle 149	0.9270571	0.63146
fetal small intestine 71	0.9295898	0.63176
hematopoietic stem cell 317	0.9465018	0.63492
fetal renal pelvis 223	0.9266072	0.63570
fetal large intestine 55	0.9331661	0.63666
fetal renal pelvis 221	0.9335710	0.64018
fetal thymus 263	0.9246175	0.64034
fetal renal pelvis 227	0.9281428	0.64042
fetal renal cortex 205	0.9300851	0.64062
fetal kidney 87	0.9295681	0.64098
fetal fibroblast 272	0.9405010	0.64326
fetal renal pelvis 220	0.9239050	0.64550
fetal muscle 153	0.9161525	0.64638
fetal lung 140	0.9376894	0.64912
fetal heart 46	0.9221861	0.65202
fetal renal pelvis 233	0.9224144	0.65326
fetal kidney 105	0.9257182	0.65406
fetal fibroblast 275	0.9367925	0.65422
fetal renal pelvis 225	0.9211459	0.65446
fetal kidney 88	0.9250731	0.65450
stomach 283	0.9085654	0.65666
hematopoietic stem cell 311	0.9273838	0.65750
fetal stomach 250	0.9068268	0.65750
fetal renal pelvis 231	0.9224652	0.66026
placenta 201	0.9167921	0.66288
placenta 196	0.9177907	0.66446
fetal stomach 249	0.8973627	0.66768
fetal small intestine 77	0.9115249	0.66948
fetal kidney 100	0.9142833	0.67076
fetal muscle 165	0.9176768	0.67166
fetal lung 300	0.9250689	0.67264
fetal renal pelvis 218	0.8908512	0.67336
fetal muscle 154	0.9045256	0.67478
fetal small intestine 76	0.9048756	0.67530
fetal renal cortex 203	0.9089429	0.67808
fetal kidney 93	0.9066012	0.67916
fetal large intestine 66	0.9019190	0.68280
fibroblast 332	0.9241195	0.68290
fetal kidney 97	0.9074730	0.68662
fetal renal cortex 215	0.9119988	0.68672
fetal renal pelvis 229	0.9080288	0.68762
fetal kidney 102	0.8980373	0.68814
fetal renal cortex 210	0.9020234	0.69056
pancreas 327	0.8901312	0.69068
fetal fibroblast 271	0.9198388	0.69462
fetal kidney 90	0.9030286	0.69570
fetal muscle 159	0.8947958	0.69774
hematopoietic stem cell 313	0.9092196	0.71016
fetal heart 52	0.9046252	0.71134
fetal fibroblast 277	0.9098630	0.71512
fetal muscle 185	0.8860827	0.71566
fetal renal pelvis 222	0.8891511	0.71762
fetal muscle 168	0.8817368	0.72332
fetal lung 127	0.8992319	0.72602
fetal fibroblast 281	0.9080075	0.72756
fetal muscle 178	0.8780670	0.73074
fetal kidney 101	0.8869836	0.73258
fetal kidney 106	0.8856609	0.73262
fetal muscle 173	0.8800320	0.73434
fetal muscle 175	0.8808093	0.73638
fetal fibroblast 274	0.8970492	0.74374
fetal muscle 158	0.8801193	0.74430
fetal renal pelvis 216	0.8755165	0.74504
fetal muscle 160	0.8703248	0.74674
fetal stomach 245	0.8642798	0.74728
fetal muscle 156	0.8759122	0.74930
fetal fibroblast 276	0.8961625	0.74978
fetal muscle 180	0.8652798	0.75020
fetal muscle 181	0.8715020	0.75024
heart 297	0.8667606	0.75416
fetal fibroblast 278	0.8904849	0.75632
fetal muscle 169	0.8655857	0.75728
fetal lung 130	0.8856998	0.76204
fetal muscle 182	0.8679572	0.76346
fetal muscle 150	0.8578920	0.76634
fetal muscle 155	0.8493588	0.76796
fetal fibroblast 273	0.8883852	0.76920
fetal muscle 148	0.8594780	0.77026
fetal muscle 151	0.8462529	0.77056
fetal heart 42	0.8613295	0.77916
fetal heart 51	0.8655412	0.78100
fetal lung 111	0.8766333	0.78220
ovary 326	0.8209687	0.79046
fetal muscle 171	0.8526497	0.79646
fetal muscle 152	0.8482928	0.79978
fetal lung 119	0.8732505	0.80110
fetal muscle 157	0.8535291	0.80138
fetal muscle 176	0.8459353	0.80392
fetal muscle 174	0.8393970	0.81010
fetal fibroblast 267	0.8630863	0.81154
fetal fibroblast 282	0.8694839	0.81188
fetal lung 128	0.8711939	0.81796
fetal muscle 193	0.8284415	0.81878
fetal lung 117	0.8582812	0.81894
fetal muscle 164	0.8306323	0.82020
fetal fibroblast 268	0.8620987	0.82118
fetal lung 124	0.8548377	0.82130
fetal lung 116	0.8668164	0.82388
fetal lung 133	0.8616284	0.82434
fetal lung 141	0.8602010	0.82510
fetal heart 43	0.8467891	0.82570
fetal muscle 146	0.8282862	0.83670
fetal lung 123	0.8419138	0.83860
fetal lung 118	0.8578383	0.84034
fetal fibroblast 280	0.8506661	0.84174
fetal lung 129	0.8420578	0.84444
fetal lung 142	0.8528276	0.84576
fetal lung 125	0.8343646	0.84748
fetal lung 122	0.8281043	0.84790
fetal fibroblast 279	0.8427064	0.84804
fetal fibroblast 269	0.8478063	0.84854
fetal muscle 147	0.8127740	0.85000
fetal heart 48	0.8223573	0.85012
fetal lung 120	0.8410066	0.85068
fetal lung 114	0.8465129	0.85104
fetal muscle 170	0.8217108	0.85158
fetal muscle 144	0.8090143	0.85186
fetal muscle 189	0.8156464	0.85314
fetal heart 50	0.8185609	0.85486
fetal muscle 166	0.8093596	0.85586
fetal lung 126	0.8443830	0.85696
fetal muscle 190	0.8005259	0.85696
fetal lung 131	0.8511859	0.85760
fetal muscle 143	0.8173141	0.85882
fetal muscle 167	0.8105900	0.85978
fetal muscle 163	0.8012056	0.86298
fetal muscle 179	0.8003812	0.86502
fetal muscle 186	0.8102019	0.86928
fetal muscle 183	0.8043684	0.87192
fetal fibroblast 270	0.8269082	0.87366
fetal lung 115	0.8282499	0.87524
fetal muscle 177	0.7827784	0.87538
fetal muscle 187	0.8056478	0.87566
fetal lung 109	0.8375025	0.87648
fetal muscle 145	0.7719095	0.88100
fetal lung 132	0.8175004	0.88128
fetal muscle 184	0.8032970	0.88304
fetal lung 134	0.8080552	0.88332
fetal lung 136	0.8122404	0.88420
fetal muscle 194	0.8172054	0.88518
fetal lung 139	0.8042976	0.88572
fetal lung 110	0.8094543	0.88870
fetal lung 108	0.7901636	0.89680
fetal muscle 162	0.7886605	0.89792
fetal muscle 161	0.7718895	0.90120
fetal lung 121	0.8117062	0.90528
fetal muscle 192	0.7767656	0.90696
fetal lung 138	0.7918096	0.90894
fetal muscle 188	0.7706422	0.91286
fetal lung 135	0.7867356	0.91588
fetal lung 113	0.8036732	0.91694
fetal muscle 191	0.7439420	0.92856
fetal lung 137	0.7800032	0.93012
fetal muscle 172	0.7723241	0.93144
fetal kidney 81	0.7423478	0.94490

Heart rate

DHS sample	fold enrichment	p value
fetal heart 47	1.6118045	0.00220
fetal heart 45	1.4754002	0.00644
fetal heart 49	1.4290649	0.01276
fetal heart 44	1.4946922	0.01330
fetal heart 46	1.4091264	0.01570
fetal heart 52	1.3171314	0.02702
fetal heart 48	1.3343751	0.03066
fetal heart 51	1.3018928	0.04338
fetal heart 42	1.2952340	0.04744
heart 297	1.3175837	0.05160
fetal heart 43	1.2528742	0.06488
fetal heart 50	1.2630808	0.06874
ovary 326	1.2246810	0.14272
ES derived MSC 289	1.1697185	0.14990
ES derived mesoderm 285	1.1377547	0.17524
ES cells 296	1.1406196	0.22538
ES derived MSC 290	1.1184205	0.22916
ES cells 295	1.1281424	0.24442
iPS 304	1.1036260	0.24538
ES derived mesoderm 286	1.0750916	0.27968
ES cells 294	1.0814940	0.31862
fetal testes 254	1.0716364	0.32778
ES cells 293	1.0688727	0.33448
fetal adrenal gland 26	1.0627892	0.34258
fetal testes 255	1.0592964	0.34598
placenta 201	1.0646118	0.35218
muscle 341	1.0640852	0.35658
fetal stomach 242	1.0581920	0.37258
fetal ovary 195	1.0495096	0.38350
fetal muscle 181	1.0438562	0.38354
fetal stomach 249	1.0441480	0.38568
melanocyte 338	1.0296350	0.40358
iPS 302	1.0345472	0.40394
fetal adrenal gland 27	1.0353438	0.40596
fetal stomach 253	1.0356308	0.40734
fetal large intestine 53	1.0355796	0.41046
fetal adrenal gland 28	1.0249309	0.42562
placenta 198	1.0258257	0.42706
CD14 4	1.0272722	0.43304
fetal lung 299	1.0241258	0.43468
melanocyte 337	1.0159487	0.44810
fetal lung 298	1.0163392	0.45128
fetal stomach 246	1.0135063	0.45302
fetal lung 300	1.0139318	0.45730
fetal large intestine 66	1.0121253	0.45824
fetal adrenal gland 24	1.0124181	0.46006
iPS 303	1.0086813	0.46170
fetal adrenal gland 25	1.0107037	0.46454
fetal small intestine 69	1.0095910	0.46490
ES derived NP 292	1.0049476	0.46722
fetal stomach 247	1.0030102	0.46812
fetal stomach 240	1.0044267	0.46860
fetal muscle 179	1.0073805	0.46886
fetal large intestine 55	1.0082522	0.47006
fetal muscle 150	1.0047420	0.47534
pancreas 327	1.0010554	0.48018
fetal large intestine 54	0.9977363	0.49102
fetal muscle 180	0.9985465	0.49432
fetal stomach 248	0.9943049	0.49520
pancreas 328	0.9911690	0.50276
fetal muscle 182	0.9949600	0.50414
hematopoietic stem cell 314	0.9915370	0.50844
fetal stomach 251	0.9858732	0.51320
fetal lung 301	0.9909516	0.51334
fetal large intestine 59	0.9856946	0.51372
fibroblast 331	0.9929182	0.51500
hematopoietic stem cell 310	0.9901077	0.51776
melanocyte 339	0.9919274	0.51980
placenta 200	0.9874364	0.51980
placenta 197	0.9844632	0.52100
fetal spinal cord 236	0.9805851	0.52898
fetal small intestine 74	0.9813827	0.53150
fetal small intestine 72	0.9785947	0.53400
fetal adrenal gland 23	0.9809823	0.53492
fetal muscle 166	0.9801617	0.53538
fetal stomach 244	0.9754433	0.53798
fetal lung 119	0.9830449	0.53890
fetal stomach 250	0.9692842	0.53958
fetal large intestine 60	0.9729587	0.54446
fetal muscle 175	0.9772773	0.54518
melanocyte 340	0.9825393	0.54616
fetal muscle 184	0.9780059	0.54980
fetal renal pelvis 219	0.9656375	0.55026
placenta 196	0.9715342	0.55030
placenta 199	0.9739967	0.55162
fetal large intestine 65	0.9675676	0.55182
fibroblast 329	0.9771816	0.55322
fetal muscle 167	0.9759992	0.55454
fetal stomach 241	0.9624633	0.55494
fetal muscle 153	0.9721596	0.55682
fetal muscle 176	0.9716155	0.55866
fetal small intestine 71	0.9658164	0.55884
ES derived NP 291	0.9616501	0.55966
fetal muscle 168	0.9699044	0.56226
fetal muscle 161	0.9694800	0.56512
fetal large intestine 67	0.9638002	0.56750
CD14 3	0.9511392	0.57106
fibroblast 332	0.9706714	0.57202
fetal muscle 187	0.9678597	0.57394
hematopoietic stem cell 319	0.9566274	0.57614
fetal muscle 155	0.9609104	0.57956
fetal muscle 151	0.9588888	0.58008
fetal muscle 193	0.9606437	0.58072
fetal muscle 190	0.9598270	0.58132
fetal stomach 245	0.9519631	0.58184
fetal spinal cord 237	0.9608247	0.58508
fetal large intestine 56	0.9527161	0.58962
fetal muscle 174	0.9577130	0.59172
fetal stomach 252	0.9474390	0.59380
fetal small intestine 77	0.9494212	0.59396
hematopoietic stem cell 308	0.9565606	0.59540
fetal stomach 243	0.9510527	0.59580
fetal large intestine 58	0.9542896	0.59734
fetal muscle 171	0.9577232	0.59838
fetal renal cortex 203	0.9526381	0.60006
fetal muscle 154	0.9516869	0.60246
fetal renal cortex 207	0.9547601	0.60298
hematopoietic stem cell 306	0.9496192	0.60390
CD20 8	0.9487976	0.60502
fetal spinal cord 235	0.9509044	0.60610
fetal fibroblast 269	0.9597208	0.60846
fetal renal pelvis 218	0.9329093	0.60996
fetal small intestine 79	0.9394308	0.61010
fetal muscle 183	0.9535174	0.61062
fetal kidney 91	0.9538803	0.61334
fetal muscle 148	0.9468843	0.61690
hematopoietic stem cell 307	0.9443620	0.61716
hematopoietic stem cell 311	0.9448876	0.61732
fetal muscle 177	0.9421233	0.61810
fetal small intestine 76	0.9352844	0.62002
fetal muscle 185	0.9445596	0.62028
fetal large intestine 64	0.9395574	0.62244
ES derived trophoblast 287	0.9399519	0.62276
fetal muscle 147	0.9450582	0.62298
fetal lung 116	0.9552967	0.62520
fetal kidney 99	0.9413303	0.62984
fetal renal pelvis 216	0.9374230	0.63210
fetal muscle 192	0.9444411	0.63438
fetal small intestine 73	0.9315791	0.63524
fetal large intestine 63	0.9279699	0.63550
fetal muscle 149	0.9381362	0.63562
fetal renal pelvis 220	0.9384939	0.63572
fetal kidney 81	0.9420012	0.63672
fetal large intestine 62	0.9255210	0.63908
fetal muscle 143	0.9421544	0.64044
fetal lung 111	0.9442808	0.64210
fetal renal cortex 213	0.9411584	0.64224
CD4 15	0.8866644	0.64288
stomach 283	0.9173090	0.64546
fibroblast 330	0.9406853	0.64920
fetal muscle 152	0.9356738	0.65298
fetal renal pelvis 227	0.9296479	0.65540
fetal fibroblast 270	0.9382453	0.65938
fetal muscle 165	0.9337798	0.66248
fetal kidney 103	0.9262600	0.66340
fetal small intestine 80	0.9215044	0.66396
fetal lung 123	0.9349455	0.66432
hematopoietic stem cell 318	0.9178685	0.66512
fetal renal pelvis 217	0.9222451	0.66528
fetal muscle 178	0.9257403	0.66696
fetal small intestine 78	0.9243658	0.66868
fetal muscle 173	0.9243947	0.67348
fetal thymus 263	0.9205603	0.67836
fetal renal pelvis 230	0.9120490	0.67876
fetal lung 112	0.9298394	0.68082
fetal muscle 164	0.9208163	0.68082
fetal kidney 104	0.9144871	0.68168
fetal muscle 156	0.9213417	0.68400
fetal renal cortex 211	0.9100662	0.68800
fetal muscle 170	0.9231761	0.68868
fetal kidney 86	0.8951346	0.68964
fetal lung 118	0.9332534	0.69012
fetal fibroblast 280	0.9267297	0.69152
fetal muscle 169	0.9147474	0.69568
fetal muscle 159	0.9125812	0.69594
hematopoietic stem cell 312	0.9142380	0.69678
fetal muscle 189	0.9147706	0.69688
Mobilized CD8 325	0.8758229	0.69688
fetal muscle 191	0.9097239	0.69738
fetal muscle 157	0.9202001	0.69790
fetal lung 117	0.9269089	0.69792
fetal kidney 102	0.9042412	0.69868
fetal muscle 163	0.9125029	0.69924
fetal kidney 83	0.8990523	0.70240
fetal lung 110	0.9169970	0.70276
fetal spinal cord 239	0.9075948	0.70820
fetal lung 141	0.9218177	0.70934
fetal renal pelvis 228	0.8987349	0.70940
fetal lung 129	0.9167077	0.71046
fetal lung 140	0.9195015	0.71104
fetal renal cortex 206	0.9078156	0.71188
fetal small intestine 68	0.8991914	0.71282
fetal kidney 97	0.9023898	0.71502
fetal muscle 162	0.9077896	0.71710
fetal kidney 82	0.8912456	0.71822
breast 2	0.9119104	0.71844
fetal renal cortex 214	0.9031316	0.71912
fetal muscle 160	0.9020673	0.72056
fetal muscle 158	0.9064795	0.72120
fetal small intestine 70	0.9025370	0.72224
fetal fibroblast 279	0.9127281	0.72398
hematopoietic stem cell 305	0.8967670	0.72416
hematopoietic stem cell 309	0.8928001	0.73078
fetal skin 234	0.9005043	0.73092
fetal lung 135	0.9065471	0.73470
fetal muscle 144	0.8925638	0.73598
hematopoietic stem cell 316	0.8811642	0.73744
fetal muscle 194	0.9070853	0.74010
fetal renal cortex 202	0.8946720	0.74112
fetal fibroblast 274	0.9051596	0.74208
fetal lung 132	0.9033069	0.74342
CD19 6	0.8486144	0.74386
fetal brain 31	0.9207427	0.74460
fetal kidney 87	0.8909842	0.74464
fetal spinal cord 238	0.8929551	0.74586
fetal large intestine 57	0.8839825	0.74680
fetal kidney 84	0.8861030	0.74968
fetal renal pelvis 223	0.8747624	0.75026
fetal lung 115	0.9026581	0.75386
fetal lung 130	0.8986508	0.75386
fetal kidney 88	0.8894190	0.75392
fetal muscle 188	0.8916203	0.75396
fetal renal cortex 205	0.8862889	0.75396
fetal renal cortex 204	0.8903160	0.75432
fetal lung 126	0.9054515	0.75450
hematopoietic stem cell 317	0.8968156	0.75478
small intestine 342	0.8704936	0.75846
fetal lung 127	0.8930532	0.75920
fetal renal cortex 210	0.8735557	0.76048
hematopoietic stem cell 313	0.8853914	0.76122
Mobilized CD4 323	0.8317658	0.76160
fetal lung 133	0.9013617	0.76304
fetal kidney 100	0.8829061	0.76324
fetal brain 38	0.8954177	0.76360
fetal lung 137	0.8955346	0.76386
fetal muscle 146	0.8830092	0.76546
fetal lung 108	0.8880449	0.76586
fetal lung 124	0.8927500	0.76824
fetal kidney 85	0.8953707	0.76908
fetal lung 114	0.9021592	0.76928
fetal brain 36	0.8976069	0.76992
fetal fibroblast 277	0.8899019	0.77082
fetal lung 138	0.8908058	0.77098
fetal lung 128	0.9012172	0.77280
fetal brain 40	0.9012745	0.77492
breast 1	0.8892356	0.77616
fetal muscle 186	0.8797510	0.77674
fetal kidney 93	0.8604372	0.78062
fetal lung 120	0.8873617	0.78066
fetal kidney 98	0.8679174	0.78144
fetal brain 34	0.8968979	0.78724
fetal kidney 92	0.8771962	0.78778
fetal kidney 106	0.8742797	0.78790
fetal fibroblast 278	0.8817317	0.78866
fetal kidney 94	0.8595481	0.78990
fetal fibroblast 282	0.8880881	0.79028
fetal kidney 107	0.8659163	0.79032
fetal fibroblast 273	0.8824319	0.79242
fetal renal pelvis 222	0.8580271	0.79328
fetal kidney 101	0.8668604	0.79342
fetal renal pelvis 233	0.8636461	0.79342
fetal kidney 89	0.8626428	0.79712
fetal fibroblast 276	0.8842502	0.79752
fetal renal pelvis 232	0.8541598	0.79834
CD56 18	0.8085218	0.79910
fetal thymus 257	0.8207240	0.79948
fetal lung 142	0.8822596	0.80078
fetal kidney 95	0.8625398	0.80186
CD8 19	0.7917832	0.80314
fetal brain 37	0.8836870	0.80320
fetal lung 134	0.8681167	0.80406
fetal lung 131	0.8869203	0.80422
ES derived trophoblast 288	0.8628809	0.80422
stomach 284	0.8344963	0.80486
fetal brain 33	0.8678984	0.80600
fetal fibroblast 275	0.8791021	0.80862
fetal lung 113	0.8803733	0.80912
fetal kidney 96	0.8614791	0.80958
fetal renal cortex 208	0.8463138	0.81476
fetal renal cortex 215	0.8588477	0.81726
fetal lung 121	0.8776496	0.81776
fetal renal cortex 209	0.8433240	0.81858
CD19 7	0.7921561	0.81900
fetal renal pelvis 224	0.8581550	0.82012
fetal brain 32	0.8736022	0.82026
fetal muscle 145	0.8367307	0.82232
fetal lung 139	0.8588577	0.82356
fetal lung 122	0.8539395	0.82518
fetal fibroblast 281	0.8663564	0.82598
fetal lung 125	0.8579562	0.82746
CD56 17	0.8127876	0.82814
CD19 5	0.7937639	0.82884
fetal renal pelvis 225	0.8369124	0.82974
fetal fibroblast 268	0.8667379	0.83064
fetal fibroblast 272	0.8619392	0.83190
keratinocyte 334	0.8725950	0.83202
CD4 16	0.7844325	0.83238
fetal lung 136	0.8628207	0.83308
keratinocyte 336	0.8727972	0.83364
CD3 10	0.7922254	0.84382
fetal kidney 105	0.8407221	0.84424
fetal brain 41	0.8631491	0.84556
fetal kidney 90	0.8283686	0.84626
fetal renal pelvis 231	0.8458249	0.84812
keratinocyte 335	0.8631051	0.84918
fetal renal pelvis 226	0.8348872	0.85152
fetal renal pelvis 221	0.8360394	0.85254
hematopoietic stem cell 315	0.8200031	0.85336
Mobilized CD3 321	0.7571684	0.85494
fetal brain 29	0.8788815	0.85548
fetal muscle 172	0.8463015	0.85596
fetal thymus 258	0.7832773	0.86192
fetal brain 30	0.8769070	0.86258
fetal renal pelvis 229	0.8246972	0.86414
CD8 21	0.7533215	0.86424
fetal brain 35	0.8620913	0.87072
fetal thymus 259	0.7591523	0.87138
fetal thymus 262	0.7317265	0.87152
fetal fibroblast 271	0.8335024	0.88008
CD4 13	0.7453990	0.88034
CD3 12	0.7425490	0.88106
Mobilized CD4 322	0.7372421	0.88304
fetal small intestine 75	0.7182306	0.88314
fetal renal cortex 212	0.8178622	0.88476
fetal fibroblast 267	0.8192038	0.89520
fetal thymus 264	0.7422098	0.90536
CD3 9	0.7206710	0.90644
fetal brain 39	0.8287516	0.90652
keratinocyte 333	0.8282038	0.91022
fetal lung 109	0.8255612	0.91104
CD4 14	0.7388138	0.91270
fetal thymus 261	0.7265535	0.91354
fetal large intestine 61	0.7062921	0.93306
fetal thymus 266	0.7078738	0.93512
CD8 20	0.7017451	0.93598
CD8 22	0.6729982	0.94550
fetal thymus 260	0.6863994	0.94798
Mobilized CD3 320	0.6486875	0.94998
CD3 11	0.6815988	0.95346
fetal thymus 265	0.6774119	0.95472
fetal thymus 256	0.6776321	0.96126
Mobilized CD56 324	0.6338123	0.97310

N−glycosylation of immunoglobulin G

DHS sample	fold enrichment	p value
placenta 199	1.4067158	0.01210
fetal muscle 192	1.3921672	0.01834
placenta 198	1.3534138	0.01986
fetal fibroblast 272	1.3288232	0.02148
fetal muscle 172	1.3409019	0.02262
fetal muscle 160	1.3748394	0.02852
CD4 14	1.4265466	0.02948
placenta 197	1.3642524	0.03068
placenta 196	1.3602748	0.03140
fetal fibroblast 276	1.2914213	0.03292
fetal fibroblast 278	1.2998787	0.03516
fetal muscle 193	1.3624829	0.03594
fetal muscle 145	1.3874309	0.03598
fetal fibroblast 271	1.2917500	0.03660
fibroblast 330	1.2874335	0.03746
hematopoietic stem cell 317	1.2818812	0.03800
fetal large intestine 56	1.3468855	0.03838
CD3 11	1.4058044	0.03844
fetal thymus 258	1.4145056	0.03846
fetal muscle 164	1.3338576	0.03908
hematopoietic stem cell 310	1.2810975	0.03928
fetal fibroblast 277	1.2906048	0.03966
Mobilized CD4 322	1.4701861	0.04004
fetal stomach 244	1.3682117	0.04038
Mobilized CD3 320	1.4901364	0.04086
fetal small intestine 70	1.2997638	0.04098
fetal fibroblast 275	1.2736529	0.04118
fetal fibroblast 268	1.2741748	0.04134
fetal muscle 143	1.3059431	0.04246
CD4 13	1.4540492	0.04324
fetal muscle 177	1.3444839	0.04366
fetal muscle 178	1.3334574	0.04438
fetal stomach 243	1.3320503	0.04484
placenta 200	1.2763748	0.04510
CD3 9	1.4252424	0.04534
fetal muscle 150	1.3310919	0.04558
fetal small intestine 80	1.3158479	0.04692
CD19 5	1.4056643	0.04696
fibroblast 329	1.2661787	0.04780
fetal fibroblast 267	1.2801564	0.04792
fetal muscle 169	1.3111234	0.04946
fetal large intestine 57	1.2966528	0.04954
fetal fibroblast 270	1.2760031	0.04970
fetal muscle 151	1.3460676	0.04994
fetal thymus 256	1.3589817	0.05016
fetal muscle 174	1.3129259	0.05074
fetal muscle 182	1.3034085	0.05108
fetal muscle 155	1.3384644	0.05244
fetal large intestine 60	1.3178422	0.05294
fetal muscle 191	1.3050993	0.05332
fetal fibroblast 282	1.2504127	0.05348
fetal small intestine 76	1.3324256	0.05352
Mobilized CD4 323	1.4027325	0.05434
fetal muscle 176	1.2893992	0.05446
fetal muscle 188	1.2919584	0.05556
fetal lung 130	1.2570768	0.05708
fetal small intestine 78	1.2773693	0.05710
fetal stomach 251	1.3253414	0.05800
fetal lung 134	1.2636831	0.05804
fetal muscle 154	1.3127312	0.05838
fetal large intestine 65	1.3130517	0.05920
CD3 10	1.3633875	0.05950
fetal thymus 266	1.3604057	0.05958
fetal muscle 166	1.2800337	0.05972
CD56 17	1.3314413	0.05974
fetal muscle 149	1.3066404	0.05992
fetal muscle 153	1.3139932	0.06070
fetal large intestine 58	1.2750087	0.06120
fetal muscle 180	1.3022379	0.06164
fetal muscle 157	1.2657291	0.06174
fetal muscle 159	1.3024699	0.06238
fetal lung 129	1.2516732	0.06276
fetal muscle 146	1.2723267	0.06300
fetal fibroblast 274	1.2434259	0.06316
fetal lung 108	1.2720406	0.06324
fetal small intestine 74	1.2914382	0.06364
fetal large intestine 64	1.2833385	0.06430
CD19 6	1.3808643	0.06444
fetal muscle 147	1.2823030	0.06556
fetal muscle 187	1.2682269	0.06570
fetal fibroblast 280	1.2415521	0.06570
fetal muscle 165	1.2595589	0.06574
fetal muscle 189	1.2757173	0.06594
fetal muscle 158	1.2652332	0.06624
fetal stomach 241	1.3322181	0.06752
fetal muscle 181	1.2781886	0.06754
fetal muscle 173	1.2875829	0.06758
fetal lung 121	1.2224522	0.06788
fetal stomach 246	1.3345633	0.06790
fetal muscle 179	1.2735025	0.06886
fetal fibroblast 279	1.2393309	0.06932
fetal fibroblast 273	1.2320657	0.07018
fetal large intestine 66	1.3024944	0.07088
CD19 7	1.3703343	0.07144
CD8 20	1.3318423	0.07206
muscle 341	1.3151083	0.07206
fetal small intestine 71	1.2845528	0.07208
fetal muscle 194	1.2339026	0.07262
ES derived MSC 290	1.2705502	0.07374
fetal stomach 252	1.3072315	0.07378
fetal stomach 248	1.3107624	0.07418
hematopoietic stem cell 314	1.2490828	0.07434
stomach 283	1.3087384	0.07440
fetal kidney 81	1.2569339	0.07554
fetal muscle 168	1.2764138	0.07584
fetal muscle 162	1.2522227	0.07596
fetal thymus 265	1.3276143	0.07666
fetal lung 116	1.2096615	0.07692
ovary 326	1.3406608	0.07714
fetal stomach 245	1.3005790	0.07744
fetal muscle 163	1.2731111	0.07774
fetal muscle 161	1.2644772	0.07858
fetal small intestine 77	1.2797809	0.07886
fetal thymus 264	1.3197369	0.07926
fetal lung 126	1.2083950	0.07946
hematopoietic stem cell 313	1.2432846	0.07950
fetal fibroblast 281	1.2253315	0.07958
placenta 201	1.2794181	0.07970
fetal lung 133	1.2175810	0.07980
fetal muscle 175	1.2541311	0.07998
fetal lung 137	1.2198963	0.08002
fetal muscle 184	1.2435256	0.08060
hematopoietic stem cell 311	1.2492643	0.08074
fetal thymus 263	1.2640291	0.08108
hematopoietic stem cell 305	1.2632507	0.08122
fetal small intestine 69	1.2899526	0.08134
fetal muscle 156	1.2512790	0.08150
fetal lung 299	1.2482141	0.08162
fetal lung 300	1.2348642	0.08266
fetal lung 120	1.2252282	0.08314
fetal large intestine 67	1.2689747	0.08348
fetal stomach 240	1.3137387	0.08394
fetal fibroblast 269	1.2169284	0.08420
fetal thymus 257	1.3391408	0.08454
fetal stomach 242	1.3402305	0.08468
fetal stomach 253	1.3024967	0.08504
fetal stomach 249	1.3036174	0.08604
fetal lung 301	1.2350497	0.08620
fetal muscle 170	1.2338701	0.08676
fetal muscle 144	1.2644834	0.08790
fibroblast 331	1.2102098	0.08802
CD3 12	1.3452069	0.08840
fetal lung 128	1.1888363	0.08870
fetal muscle 171	1.2362844	0.08902
fibroblast 332	1.2062399	0.08928
fetal large intestine 55	1.2453352	0.08948
fetal muscle 186	1.2423972	0.09108
fetal stomach 247	1.3246883	0.09124
fetal lung 117	1.2114892	0.09158
fetal muscle 183	1.2333368	0.09162
fetal lung 141	1.1999769	0.09166
Mobilized CD56 324	1.3104135	0.09170
fetal muscle 167	1.2341815	0.09370
fetal lung 138	1.2166541	0.09390
fetal large intestine 53	1.2886443	0.09424
pancreas 328	1.2716137	0.09440
hematopoietic stem cell 309	1.2336381	0.09494
fetal small intestine 73	1.2526489	0.09502
melanocyte 338	1.1955685	0.09582
fetal lung 298	1.2336206	0.09634
ES derived MSC 289	1.2391447	0.09792
CD4 16	1.3383935	0.09824
fetal large intestine 63	1.2542011	0.09876
CD56 18	1.3235163	0.09930
fetal small intestine 72	1.2408946	0.09948
fetal lung 118	1.1898811	0.09950
fetal muscle 185	1.2430382	0.09994
fetal small intestine 79	1.2559638	0.10028
fetal small intestine 75	1.4045642	0.10226
pancreas 327	1.2683647	0.10370
fetal heart 42	1.2409197	0.10390
fetal lung 127	1.2052728	0.10402
fetal large intestine 62	1.2601540	0.10470
fetal lung 139	1.2173419	0.10520
fetal stomach 250	1.2724902	0.10836
fetal large intestine 59	1.2482833	0.10850
fetal muscle 148	1.2306474	0.10868
fetal lung 140	1.1968058	0.11000
hematopoietic stem cell 318	1.2342717	0.11022
fetal lung 136	1.1992902	0.11068
CD8 21	1.3043412	0.11094
fetal heart 52	1.2142585	0.11096
fetal lung 132	1.1999072	0.11166
fetal lung 125	1.2015482	0.11174
fetal lung 114	1.1751363	0.11200
Mobilized CD8 325	1.2998912	0.11212
fetal small intestine 68	1.2195368	0.11248
fetal lung 112	1.1938559	0.11366
fetal large intestine 61	1.2822237	0.11486
fetal lung 122	1.2056062	0.11512
hematopoietic stem cell 306	1.2148925	0.11540
fetal thymus 259	1.2863900	0.11556
fetal lung 142	1.1753898	0.11828
hematopoietic stem cell 312	1.2033644	0.11832
fetal thymus 260	1.2681508	0.11892
CD20 8	1.2122533	0.12006
fetal muscle 190	1.2310811	0.12086
hematopoietic stem cell 308	1.1987470	0.12146
fetal large intestine 54	1.2246730	0.12164
hematopoietic stem cell 307	1.2058100	0.12652
CD8 19	1.3032187	0.12684
fetal lung 110	1.1892295	0.12780
fetal renal pelvis 217	1.2247668	0.12884
fetal lung 123	1.1865644	0.12886
fetal muscle 152	1.1951390	0.12946
Mobilized CD3 321	1.2941723	0.12948
CD8 22	1.2723533	0.13238
hematopoietic stem cell 315	1.2040666	0.13314
hematopoietic stem cell 316	1.2106832	0.13360
fetal lung 131	1.1574329	0.13366
fetal renal pelvis 228	1.2292449	0.13448
fetal heart 46	1.2201457	0.13474
heart 297	1.2203683	0.13562
fetal heart 47	1.2393388	0.13604
melanocyte 337	1.1601626	0.13666
fetal lung 109	1.1537604	0.13896
stomach 284	1.2210348	0.14036
fetal heart 51	1.1996158	0.14256
fetal thymus 261	1.2444150	0.14354
hematopoietic stem cell 319	1.2034347	0.14468
fetal heart 44	1.2329901	0.14624
fetal heart 48	1.2000189	0.14706
melanocyte 339	1.1644043	0.14776
fetal renal cortex 207	1.1946328	0.15384
fetal testes 255	1.1820383	0.15608
fetal kidney 83	1.2138926	0.15816
fetal heart 45	1.1939374	0.15824
fetal kidney 92	1.1856241	0.15918
CD14 4	1.2058232	0.16008
fetal thymus 262	1.2625834	0.16078
fetal lung 113	1.1428289	0.16420
fetal testes 254	1.1847715	0.16502
CD14 3	1.2092517	0.16550
fetal heart 49	1.1878993	0.16664
fetal lung 115	1.1480110	0.16724
small intestine 342	1.1760488	0.16762
fetal renal cortex 211	1.1965149	0.16936
fetal lung 119	1.1460978	0.17188
ES derived trophoblast 288	1.1648907	0.17200
fetal lung 124	1.1529346	0.17228
melanocyte 340	1.1505109	0.17566
fetal lung 111	1.1360503	0.18468
fetal kidney 87	1.1639998	0.18644
fetal heart 43	1.1558595	0.19200
fetal heart 50	1.1584421	0.19214
fetal kidney 91	1.1509051	0.19410
fetal renal pelvis 232	1.1708271	0.19498
fetal renal pelvis 219	1.1929983	0.19574
fetal kidney 82	1.1735053	0.19858
fetal ovary 195	1.1855206	0.19884
fetal kidney 107	1.1570831	0.20280
fetal kidney 100	1.1540537	0.20424
fetal lung 135	1.1308170	0.20792
fetal renal pelvis 230	1.1648330	0.20892
ES derived mesoderm 286	1.1218791	0.21008
fetal kidney 106	1.1468602	0.21142
fetal renal pelvis 218	1.1825801	0.21144
iPS 303	1.1382167	0.21768
fetal kidney 84	1.1476877	0.22364
fetal renal pelvis 226	1.1388209	0.22454
fetal kidney 102	1.1511257	0.22518
fetal renal cortex 205	1.1401775	0.22738
fetal kidney 95	1.1352506	0.23220
fetal renal pelvis 220	1.1389921	0.23338
fetal renal cortex 203	1.1356738	0.23426
fetal renal pelvis 221	1.1312344	0.23454
keratinocyte 336	1.1002967	0.23690
fetal kidney 94	1.1404857	0.23730
CD4 15	1.1905445	0.23816
fetal kidney 86	1.1568255	0.23836
fetal kidney 99	1.1301170	0.24250
fetal renal pelvis 216	1.1297036	0.24564
fetal renal pelvis 227	1.1254508	0.24616
fetal renal pelvis 231	1.1183710	0.24980
fetal adrenal gland 23	1.1151590	0.25204
fetal kidney 88	1.1175177	0.25408
fetal kidney 103	1.1215568	0.25750
ES derived trophoblast 287	1.1186373	0.26030
ES derived mesoderm 285	1.1026945	0.26088
fetal skin 234	1.1156916	0.26412
fetal kidney 89	1.1176772	0.26474
ES derived NP 292	1.1225450	0.26646
fetal renal cortex 214	1.1061013	0.27804
fetal renal cortex 202	1.1037493	0.28216
fetal renal pelvis 223	1.1121431	0.28222
fetal renal pelvis 233	1.1053582	0.28426
fetal kidney 93	1.1072168	0.28556
keratinocyte 335	1.0789555	0.28656
fetal renal cortex 210	1.1066841	0.28704
fetal renal pelvis 225	1.1050709	0.28840
fetal adrenal gland 26	1.0948915	0.29386
fetal renal pelvis 222	1.1015032	0.29400
keratinocyte 333	1.0721654	0.29526
fetal renal cortex 212	1.0926886	0.29798
fetal kidney 101	1.0943669	0.29882
ES cells 293	1.0989713	0.29898
fetal renal cortex 215	1.0894419	0.30048
fetal renal pelvis 229	1.0907757	0.30264
fetal kidney 90	1.0941422	0.30408
fetal renal cortex 206	1.0835748	0.31284
fetal kidney 96	1.0813820	0.31790
fetal kidney 104	1.0836110	0.32232
fetal adrenal gland 24	1.0826971	0.32718
fetal renal cortex 209	1.0800737	0.32766
fetal spinal cord 235	1.0707438	0.33328
fetal kidney 85	1.0672609	0.33378
fetal adrenal gland 27	1.0749291	0.33426
fetal kidney 105	1.0745267	0.33458
ES derived NP 291	1.0845461	0.33564
fetal adrenal gland 28	1.0647985	0.33830
fetal kidney 97	1.0715572	0.34466
breast 1	1.0571242	0.34752
keratinocyte 334	1.0502259	0.35500
ES cells 296	1.0692312	0.35926
fetal kidney 98	1.0602396	0.36436
fetal renal cortex 208	1.0577758	0.36948
fetal renal pelvis 224	1.0555357	0.37080
breast 2	1.0439792	0.37756
fetal adrenal gland 25	1.0523065	0.38208
fetal spinal cord 237	1.0468266	0.38236
fetal renal cortex 204	1.0471833	0.38350
iPS 302	1.0464699	0.38716
fetal renal cortex 213	1.0459675	0.38784
fetal brain 37	1.0352474	0.40142
ES cells 294	1.0427008	0.40232
iPS 304	1.0203140	0.44190
ES cells 295	1.0199837	0.44508
fetal brain 32	1.0128630	0.45830
fetal brain 41	1.0111482	0.46106
fetal spinal cord 236	1.0068982	0.47320
fetal brain 40	0.9950241	0.50586
fetal brain 29	0.9898725	0.52262
fetal spinal cord 238	0.9847437	0.52428
fetal brain 39	0.9819942	0.53884
fetal spinal cord 239	0.9774718	0.53942
fetal brain 36	0.9768987	0.54388
fetal brain 31	0.9814313	0.54784
fetal brain 33	0.9755715	0.55078
fetal brain 38	0.9654739	0.57700
fetal brain 30	0.9358099	0.68286
fetal brain 34	0.9219551	0.70184
fetal brain 35	0.9188560	0.71864

Mean arterial pressure

DHS sample	fold enrichment	p value
hematopoietic stem cell 310	1.4108654	0.00400
hematopoietic stem cell 314	1.3914572	0.00922
Mobilized CD56 324	1.5167092	0.01334
fetal fibroblast 279	1.3243449	0.01536
melanocyte 338	1.2655074	0.01924
fetal fibroblast 280	1.2983490	0.02106
fetal fibroblast 269	1.2949771	0.02166
fetal fibroblast 270	1.3009353	0.02276
fetal fibroblast 271	1.2872128	0.02286
fetal fibroblast 277	1.2974078	0.02304
hematopoietic stem cell 308	1.3306232	0.02386
fetal fibroblast 273	1.2853425	0.02416
fetal heart 49	1.3499140	0.02518
small intestine 342	1.3714843	0.02590
fetal lung 141	1.2802465	0.02772
hematopoietic stem cell 318	1.3536305	0.02844
fetal heart 47	1.3626390	0.02896
fetal fibroblast 275	1.2640504	0.02902
fetal kidney 85	1.2864045	0.02968
hematopoietic stem cell 305	1.3336600	0.03176
hematopoietic stem cell 316	1.3487654	0.03286
fetal heart 45	1.3166270	0.03404
hematopoietic stem cell 309	1.3157072	0.03538
fetal lung 140	1.2735479	0.03634
hematopoietic stem cell 315	1.3269955	0.03688
keratinocyte 336	1.2310113	0.03756
Mobilized CD3 320	1.4652053	0.03802
fibroblast 331	1.2649773	0.03832
fetal fibroblast 272	1.2506801	0.04106
keratinocyte 335	1.2222674	0.04274
fetal fibroblast 276	1.2369241	0.04378
fetal fibroblast 278	1.2482898	0.04564
fibroblast 329	1.2520879	0.04600
fetal muscle 149	1.2848709	0.04788
fetal large intestine 67	1.3042096	0.04846
fetal heart 52	1.2618644	0.04848
CD20 8	1.2923571	0.04912
fetal lung 111	1.2508718	0.04918
fetal muscle 167	1.2604228	0.04930
fibroblast 332	1.2422709	0.04970
fetal lung 133	1.2422907	0.04986
hematopoietic stem cell 312	1.2783730	0.05008
fetal lung 128	1.2192475	0.05252
fetal muscle 181	1.2620457	0.05284
fetal fibroblast 281	1.2301090	0.05286
fetal muscle 176	1.2442941	0.05412
fetal muscle 143	1.2392937	0.05494
fetal kidney 92	1.2648068	0.05588
fetal muscle 180	1.2668566	0.05772
fetal muscle 166	1.2545000	0.05834
fetal fibroblast 268	1.2202573	0.05848
fetal fibroblast 274	1.2234966	0.05876
fetal muscle 178	1.2586461	0.06028
fetal lung 119	1.2236609	0.06092
fetal fibroblast 282	1.2130377	0.06134
fetal thymus 258	1.3361017	0.06176
fetal heart 46	1.2692575	0.06254
fibroblast 330	1.2329812	0.06276
fetal kidney 91	1.2410233	0.06336
fetal heart 43	1.2461545	0.06382
Mobilized CD4 323	1.3731424	0.06472
fetal muscle 151	1.2666944	0.06494
fetal small intestine 68	1.2593005	0.06580
fetal large intestine 61	1.3462880	0.06622
CD3 10	1.3184473	0.06624
fetal muscle 169	1.2459617	0.06740
hematopoietic stem cell 307	1.2498319	0.06896
fetal lung 118	1.2053230	0.07066
fetal small intestine 74	1.2667591	0.07172
fetal lung 117	1.2173279	0.07224
fetal muscle 165	1.2214035	0.07236
hematopoietic stem cell 313	1.2310987	0.07296
CD8 21	1.3640303	0.07354
fetal lung 121	1.2064001	0.07368
CD8 19	1.3859207	0.07444
hematopoietic stem cell 317	1.2109792	0.07486
CD19 6	1.3222210	0.07674
fetal muscle 153	1.2453358	0.07716
melanocyte 337	1.1759719	0.07770
fetal renal cortex 213	1.2244070	0.07900
fetal renal cortex 207	1.2435655	0.07940
fetal lung 116	1.1961074	0.07994
fetal lung 120	1.2162025	0.08120
fetal lung 132	1.2138245	0.08364
fetal lung 298	1.2217481	0.08474
fetal adrenal gland 26	1.2384870	0.08504
fetal stomach 248	1.2649065	0.08622
CD3 9	1.3173349	0.08740
fetal lung 299	1.2184842	0.08784
fetal small intestine 78	1.2234981	0.08852
CD19 7	1.3126243	0.08854
Mobilized CD3 321	1.3397210	0.08930
fetal heart 51	1.2237758	0.08934
fetal muscle 164	1.2170896	0.08958
ES derived mesoderm 285	1.1823038	0.08974
fetal thymus 265	1.2846802	0.09094
fetal adrenal gland 28	1.2038852	0.09290
fetal small intestine 70	1.2137057	0.09336
CD56 18	1.3212266	0.09388
hematopoietic stem cell 311	1.2198702	0.09412
fetal renal pelvis 220	1.2354147	0.09470
fetal large intestine 58	1.2204386	0.09508
fetal renal pelvis 226	1.2196451	0.09604
fetal muscle 154	1.2244884	0.09660
keratinocyte 333	1.1594702	0.09734
fetal kidney 95	1.2208178	0.09764
fetal muscle 172	1.1862811	0.09830
fetal lung 114	1.1819247	0.09860
Mobilized CD8 325	1.3069118	0.09916
CD4 15	1.3580117	0.09966
fetal fibroblast 267	1.1883379	0.09992
fetal heart 48	1.2139550	0.10090
fetal muscle 175	1.1973977	0.10150
fetal muscle 159	1.2128212	0.10226
CD3 12	1.3036668	0.10256
heart 297	1.2367185	0.10360
fetal thymus 263	1.2081341	0.10466
fetal stomach 244	1.2331422	0.10544
fetal stomach 246	1.2491329	0.10574
fetal heart 42	1.2114265	0.10576
fetal renal pelvis 227	1.2106977	0.10864
CD4 13	1.2951622	0.10874
fetal lung 126	1.1721138	0.10950
hematopoietic stem cell 306	1.2048293	0.10986
fetal muscle 179	1.1953972	0.11066
melanocyte 340	1.1679189	0.11206
Mobilized CD4 322	1.2836871	0.11496
fetal heart 44	1.2406462	0.11538
CD19 5	1.2537278	0.11806
fetal kidney 100	1.1913499	0.12234
fetal thymus 256	1.2342120	0.12286
fetal muscle 150	1.1941789	0.12294
fetal lung 129	1.1703992	0.12334
CD3 11	1.2459149	0.12386
fetal heart 50	1.1931812	0.12482
fetal large intestine 54	1.2071681	0.12636
fetal muscle 152	1.1742434	0.12790
melanocyte 339	1.1474856	0.12982
hematopoietic stem cell 319	1.1977422	0.13122
fetal lung 301	1.1756246	0.13136
fetal kidney 99	1.1874824	0.13262
fetal muscle 171	1.1705524	0.13312
fetal thymus 264	1.2244547	0.13418
fetal kidney 106	1.1789736	0.13676
keratinocyte 334	1.1360624	0.13700
fetal lung 130	1.1587060	0.13868
fetal muscle 156	1.1689452	0.13972
fetal large intestine 64	1.1887827	0.14146
ES derived mesoderm 286	1.1283369	0.14568
CD4 14	1.2174447	0.14596
fetal stomach 243	1.1831644	0.14754
fetal lung 112	1.1577435	0.14756
breast 1	1.1391632	0.14916
fetal muscle 160	1.1670748	0.14938
fetal lung 300	1.1586699	0.15066
fetal skin 234	1.1700772	0.15084
fetal renal cortex 202	1.1654398	0.15330
fetal renal cortex 214	1.1681527	0.15360
fetal muscle 194	1.1461721	0.15414
fetal renal pelvis 221	1.1606879	0.15510
fetal thymus 260	1.2111617	0.15552
fetal renal pelvis 231	1.1563890	0.15604
fetal kidney 84	1.1717470	0.15750
fetal lung 134	1.1551885	0.15782
fetal lung 135	1.1504793	0.15928
fetal muscle 148	1.1589405	0.15954
fetal stomach 240	1.1956472	0.16016
fetal muscle 170	1.1476490	0.16148
fetal kidney 83	1.1849259	0.16214
fetal large intestine 57	1.1616832	0.16362
fetal small intestine 69	1.1853684	0.16418
fetal muscle 158	1.1469245	0.16644
CD56 17	1.1883732	0.16664
fetal renal pelvis 228	1.1692477	0.16676
fetal lung 124	1.1416458	0.16680
fetal large intestine 53	1.1850161	0.16832
CD14 4	1.1852946	0.16838
fetal muscle 155	1.1600168	0.16928
fetal lung 115	1.1346620	0.17054
fetal kidney 103	1.1566586	0.17190
fetal large intestine 55	1.1565781	0.17236
CD8 22	1.2107890	0.17310
fetal stomach 252	1.1703654	0.17358
fetal muscle 174	1.1457211	0.17482
fetal lung 110	1.1424303	0.17544
fetal thymus 266	1.1869503	0.17700
breast 2	1.1256339	0.17800
fetal muscle 173	1.1453022	0.17804
fetal lung 136	1.1354196	0.18014
fetal small intestine 72	1.1594270	0.18104
fetal large intestine 63	1.1623598	0.18326
pancreas 328	1.1665044	0.18508
fetal thymus 259	1.1906326	0.18600
fetal adrenal gland 27	1.1516213	0.18758
stomach 284	1.1674944	0.18786
fetal large intestine 66	1.1602271	0.18894
fetal stomach 251	1.1559637	0.18930
fetal lung 123	1.1309313	0.19066
fetal adrenal gland 24	1.1611603	0.19288
fetal muscle 184	1.1251115	0.19418
fetal muscle 192	1.1266258	0.19752
fetal muscle 185	1.1403324	0.19778
fetal lung 142	1.1136722	0.19818
fetal lung 137	1.1206849	0.19848
fetal large intestine 60	1.1479336	0.20078
fetal adrenal gland 25	1.1494418	0.20250
fetal lung 125	1.1239589	0.20276
fetal thymus 257	1.1750041	0.20438
fetal kidney 89	1.1366795	0.20456
fetal small intestine 75	1.1965950	0.20820
fetal muscle 193	1.1260323	0.20868
fetal stomach 241	1.1519698	0.20876
fetal muscle 183	1.1183201	0.20934
fetal small intestine 73	1.1439951	0.20938
fetal lung 113	1.1105984	0.21120
fetal lung 131	1.1029398	0.21342
fetal muscle 163	1.1246968	0.21348
fetal muscle 168	1.1247505	0.21472
fetal small intestine 79	1.1445749	0.21482
fetal lung 109	1.1006299	0.21784
fetal muscle 147	1.1207838	0.21788
fetal testes 255	1.1163403	0.21858
fetal renal pelvis 217	1.1275350	0.22072
fetal stomach 247	1.1541742	0.22578
fetal small intestine 71	1.1305862	0.22728
CD4 16	1.1638224	0.22748
fetal small intestine 80	1.1253571	0.22800
CD14 3	1.1492432	0.22808
placenta 199	1.1180318	0.23086
placenta 200	1.1132580	0.23158
placenta 198	1.1120706	0.23738
fetal muscle 189	1.1064655	0.23876
fetal small intestine 76	1.1272576	0.23912
fetal kidney 94	1.1193777	0.23914
fetal lung 108	1.1091968	0.23970
fetal muscle 144	1.1101717	0.24106
fetal stomach 250	1.1286225	0.24148
fetal muscle 182	1.1032469	0.24326
stomach 283	1.1297218	0.24348
fetal stomach 249	1.1245725	0.24598
fetal muscle 157	1.0978878	0.24934
fetal muscle 191	1.1042752	0.24986
fetal thymus 261	1.1345692	0.24992
fetal lung 138	1.0975077	0.25092
fetal small intestine 77	1.1169613	0.25098
fetal lung 127	1.0949530	0.25734
fetal kidney 102	1.1078982	0.25758
fetal renal pelvis 233	1.1034591	0.25880
fetal large intestine 62	1.1142573	0.26164
ES derived MSC 290	1.0971364	0.26436
fetal stomach 245	1.1098742	0.26640
fetal muscle 186	1.0916477	0.26724
ES derived MSC 289	1.0967003	0.26758
fetal muscle 187	1.0907650	0.26796
fetal renal cortex 205	1.0940721	0.27460
CD8 20	1.1184200	0.27730
fetal large intestine 59	1.1032377	0.27800
fetal large intestine 56	1.0994610	0.27906
fetal muscle 146	1.0857634	0.28272
fetal renal pelvis 232	1.0913378	0.28644
fetal kidney 87	1.0845078	0.28932
fetal muscle 161	1.0824975	0.28964
fetal stomach 253	1.0981842	0.29060
fetal muscle 145	1.0872292	0.29256
fetal renal pelvis 230	1.0872582	0.29770
fetal muscle 188	1.0748709	0.30082
fetal adrenal gland 23	1.0786843	0.30172
fetal muscle 177	1.0794353	0.30254
fetal large intestine 65	1.0876562	0.30342
fetal kidney 97	1.0759683	0.30532
fetal renal cortex 212	1.0705327	0.31116
ES derived NP 291	1.0834345	0.31536
fetal muscle 190	1.0722133	0.31606
fetal muscle 162	1.0684129	0.31752
fetal renal pelvis 224	1.0631452	0.32594
fetal testes 254	1.0657575	0.32974
fetal renal pelvis 216	1.0646286	0.33280
fetal renal cortex 203	1.0623658	0.33570
fetal ovary 195	1.0660748	0.34286
pancreas 327	1.0663989	0.34828
fetal renal cortex 206	1.0538109	0.35094
fetal renal cortex 215	1.0513620	0.35360
fetal kidney 105	1.0506739	0.35720
fetal kidney 88	1.0490195	0.35798
placenta 197	1.0562170	0.36182
muscle 341	1.0595404	0.36372
fetal renal cortex 211	1.0485077	0.36754
fetal lung 139	1.0411776	0.37588
fetal kidney 101	1.0433785	0.37708
fetal lung 122	1.0414787	0.38006
fetal kidney 104	1.0409594	0.38206
fetal kidney 81	1.0349801	0.39340
fetal renal cortex 210	1.0359390	0.39464
fetal stomach 242	1.0405914	0.39594
fetal thymus 262	1.0392894	0.39952
fetal spinal cord 236	1.0322549	0.40226
fetal renal pelvis 222	1.0323420	0.40416
ES derived NP 292	1.0346396	0.40732
placenta 201	1.0355855	0.40816
fetal kidney 107	1.0248517	0.41932
fetal renal pelvis 219	1.0255074	0.42392
fetal kidney 82	1.0240185	0.42402
fetal renal pelvis 229	1.0200494	0.42914
fetal renal cortex 208	1.0206863	0.43272
fetal kidney 98	1.0139624	0.44608
ES cells 295	1.0125510	0.45152
fetal kidney 96	1.0069098	0.46232
fetal renal pelvis 225	1.0058151	0.46538
fetal renal pelvis 223	1.0027106	0.46734
fetal kidney 90	1.0038145	0.47090
placenta 196	1.0059753	0.47232
fetal renal cortex 209	0.9975005	0.48308
fetal renal cortex 204	0.9988649	0.48618
iPS 302	0.9868536	0.51188
iPS 303	0.9875978	0.51396
fetal renal pelvis 218	0.9658339	0.53688
fetal kidney 86	0.9686654	0.53934
fetal spinal cord 235	0.9785453	0.54440
ES derived trophoblast 288	0.9789340	0.54756
ES cells 293	0.9705985	0.54976
ovary 326	0.9595101	0.55574
ES cells 296	0.9604368	0.56346
fetal brain 37	0.9751122	0.56582
fetal kidney 93	0.9538857	0.58760
iPS 304	0.9585721	0.59456
fetal brain 32	0.9631662	0.59558
ES derived trophoblast 287	0.9392868	0.64380
fetal brain 36	0.9393286	0.67002
fetal brain 35	0.9468052	0.67318
fetal spinal cord 237	0.9258875	0.68444
fetal brain 33	0.9228824	0.69350
fetal spinal cord 238	0.9086922	0.72346
fetal spinal cord 239	0.9062092	0.72852
fetal brain 31	0.9338404	0.73134
fetal brain 39	0.9198724	0.74118
fetal brain 38	0.9049634	0.75690
fetal brain 40	0.9130742	0.76492
ES cells 294	0.8601077	0.77378
fetal brain 41	0.9038329	0.77590
fetal brain 34	0.8925296	0.82322
fetal brain 29	0.9022210	0.83220
fetal brain 30	0.8864930	0.87466

Height 2

DHS sample	fold enrichment	p value
fetal fibroblast 276	1.4990394	0.00304
fetal fibroblast 275	1.4572774	0.00522
fetal fibroblast 274	1.4570033	0.00702
fetal fibroblast 273	1.4276235	0.00844
fetal fibroblast 271	1.4227723	0.01028
fetal fibroblast 272	1.4049124	0.01412
fetal fibroblast 270	1.3946627	0.01674
fetal fibroblast 269	1.3730364	0.01864
fetal fibroblast 277	1.3845703	0.01890
fetal fibroblast 268	1.3545420	0.02468
fetal fibroblast 281	1.3409618	0.02776
fetal fibroblast 278	1.3522716	0.02988
fetal fibroblast 282	1.3215622	0.03018
fibroblast 332	1.3464001	0.03026
fetal fibroblast 280	1.3365696	0.03034
fetal muscle 172	1.3402258	0.03458
fetal muscle 176	1.3852460	0.03650
fetal fibroblast 267	1.3327884	0.03908
fetal fibroblast 279	1.3210511	0.03950
fetal muscle 168	1.3742260	0.04130
fibroblast 329	1.3200022	0.04296
fetal muscle 143	1.3417870	0.04794
fetal thymus 258	1.4351009	0.04814
fibroblast 330	1.3117143	0.04926
keratinocyte 334	1.2605730	0.05084
fibroblast 331	1.3052661	0.05130
breast 2	1.2897702	0.05282
fetal muscle 149	1.3662268	0.05544
breast 1	1.2780697	0.05668
CD4 15	1.4783373	0.05830
fetal lung 298	1.3180683	0.05868
fetal lung 128	1.2621615	0.06014
fetal muscle 181	1.3326382	0.06196
fetal muscle 160	1.3287045	0.06518
fetal lung 300	1.2927228	0.06604
fetal lung 117	1.2722184	0.06844
fetal thymus 260	1.3801645	0.07008
fetal lung 111	1.2714925	0.07010
fetal large intestine 61	1.3720080	0.07138
fetal thymus 256	1.3799149	0.07288
fetal muscle 153	1.3215587	0.07418
keratinocyte 333	1.2231405	0.07538
fetal thymus 263	1.3003034	0.07610
fetal skin 234	1.2962207	0.07618
fetal kidney 85	1.2593375	0.07782
keratinocyte 336	1.2292510	0.07794
fetal muscle 157	1.2726533	0.07856
fetal muscle 187	1.2785234	0.08064
melanocyte 338	1.2264078	0.08310
hematopoietic stem cell 310	1.2607884	0.08356
fetal muscle 169	1.2923609	0.08456
fetal muscle 171	1.2741485	0.08674
CD8 21	1.3793097	0.08978
fetal kidney 91	1.2481031	0.08992
fetal muscle 159	1.2873045	0.08996
keratinocyte 335	1.2186211	0.09118
fetal thymus 257	1.3348227	0.09122
fetal thymus 264	1.3406460	0.09216
fetal muscle 192	1.2689595	0.09232
fetal muscle 164	1.2733838	0.09534
fetal thymus 265	1.3374049	0.09544
fetal muscle 167	1.2688487	0.09732
fetal lung 140	1.2326282	0.09892
fetal muscle 175	1.2576001	0.10256
fetal muscle 152	1.2544103	0.10398
CD8 19	1.3585849	0.10460
fetal lung 133	1.2210700	0.10484
fetal lung 301	1.2340695	0.11422
fetal muscle 165	1.2302727	0.11598
fetal muscle 151	1.2714488	0.11600
CD3 12	1.3220609	0.11664
fetal muscle 154	1.2596273	0.11834
Mobilized CD56 324	1.3063837	0.11870
fetal lung 114	1.2028001	0.11912
fetal lung 121	1.2035694	0.12070
fetal lung 141	1.2067726	0.12154
melanocyte 339	1.1863783	0.12174
fetal thymus 266	1.2853161	0.12306
Mobilized CD8 325	1.3018763	0.12316
Mobilized CD3 320	1.3503648	0.12426
fetal lung 129	1.2072402	0.12900
fetal lung 125	1.2159638	0.13072
fetal muscle 166	1.2276717	0.13222
fetal lung 109	1.1841324	0.13262
melanocyte 337	1.1709330	0.13322
fetal muscle 184	1.2119180	0.13620
fetal muscle 156	1.2136050	0.14032
fetal lung 120	1.1989622	0.14152
fetal heart 47	1.2468518	0.14210
fetal lung 118	1.1815396	0.14296
Mobilized CD4 323	1.2849939	0.14370
fetal muscle 182	1.2105704	0.14456
fetal muscle 148	1.2206538	0.14554
fetal large intestine 67	1.2368915	0.14572
fetal muscle 193	1.2138767	0.14814
fetal muscle 179	1.2137264	0.14820
CD4 13	1.2908624	0.15034
fetal lung 299	1.2057652	0.15184
melanocyte 340	1.1701406	0.15634
fetal muscle 194	1.1815396	0.15702
fetal thymus 259	1.2553848	0.15796
Mobilized CD4 322	1.2719065	0.15952
fetal muscle 173	1.1952895	0.16248
fetal muscle 155	1.2101921	0.16252
CD3 9	1.2633927	0.16326
fetal lung 108	1.1896254	0.16344
fetal lung 134	1.1797385	0.16420
fetal muscle 178	1.2026240	0.16434
hematopoietic stem cell 314	1.1866524	0.16522
Mobilized CD3 321	1.2636897	0.16562
fetal lung 112	1.1707323	0.16972
hematopoietic stem cell 315	1.1903696	0.17014
fetal lung 132	1.1729797	0.17056
fetal lung 138	1.1739051	0.17248
fetal muscle 158	1.1813824	0.17250
fetal kidney 103	1.1888584	0.17418
fetal kidney 94	1.1912662	0.17500
fetal kidney 106	1.1791849	0.17546
fetal muscle 180	1.1942566	0.18152
fetal muscle 147	1.1811271	0.18460
CD56 18	1.2503423	0.18534
fetal small intestine 80	1.1870005	0.18732
fetal renal pelvis 220	1.1812935	0.18804
fetal lung 126	1.1502433	0.18940
hematopoietic stem cell 308	1.1640719	0.18966
fetal kidney 95	1.1742499	0.19030
fetal muscle 183	1.1654847	0.19086
fetal lung 139	1.1627436	0.19204
fetal muscle 174	1.1724225	0.19236
fetal thymus 261	1.2120145	0.19506
fetal muscle 161	1.1666857	0.19686
fetal muscle 186	1.1604621	0.19878
fetal muscle 150	1.1705361	0.20024
fetal lung 116	1.1369924	0.20650
hematopoietic stem cell 309	1.1591256	0.20670
fetal stomach 242	1.2028838	0.21166
fetal lung 127	1.1464455	0.21182
fetal muscle 146	1.1555710	0.21342
fetal lung 130	1.1415980	0.21472
CD3 11	1.1929911	0.21492
fetal muscle 163	1.1565547	0.21532
fetal lung 122	1.1451605	0.21736
fetal muscle 189	1.1491803	0.21784
fetal lung 142	1.1302994	0.21804
fetal kidney 100	1.1484611	0.21996
fetal kidney 81	1.1424473	0.22102
fetal lung 113	1.1321662	0.22322
fetal muscle 191	1.1495120	0.22380
hematopoietic stem cell 305	1.1473275	0.22386
fetal ovary 195	1.1682614	0.22596
fetal stomach 248	1.1641766	0.22758
fetal lung 137	1.1295890	0.22790
hematopoietic stem cell 307	1.1399972	0.22864
fetal small intestine 75	1.1891553	0.22946
ES cells 294	1.1536148	0.23124
fetal kidney 83	1.1548339	0.23146
ES cells 295	1.1564092	0.23206
hematopoietic stem cell 318	1.1467516	0.23336
hematopoietic stem cell 316	1.1420053	0.23482
fetal lung 110	1.1308858	0.23742
fetal kidney 89	1.1387766	0.23800
fetal muscle 170	1.1308530	0.23856
hematopoietic stem cell 313	1.1288388	0.23924
CD20 8	1.1460977	0.23948
ES derived NP 292	1.1484501	0.23994
fetal thymus 262	1.1794183	0.23998
CD8 22	1.1775135	0.24004
fetal small intestine 69	1.1552317	0.24016
fetal muscle 185	1.1408623	0.24018
fetal renal cortex 207	1.1361627	0.24454
CD19 7	1.1727427	0.24474
fetal stomach 252	1.1432042	0.24570
fetal muscle 145	1.1402906	0.24854
ES derived MSC 289	1.1317870	0.24858
hematopoietic stem cell 311	1.1254526	0.25268
fetal small intestine 74	1.1371276	0.25434
hematopoietic stem cell 319	1.1307708	0.25450
fetal stomach 249	1.1458053	0.25456
fetal stomach 244	1.1412099	0.25478
fetal large intestine 53	1.1457422	0.25588
fetal heart 45	1.1298745	0.25654
fetal renal pelvis 217	1.1277556	0.26054
fetal stomach 247	1.1555443	0.26084
ES derived MSC 290	1.1217307	0.26128
fetal stomach 250	1.1431673	0.26136
fetal adrenal gland 26	1.1210966	0.26296
fetal stomach 241	1.1381817	0.26300
fetal lung 123	1.1126244	0.26356
fetal large intestine 58	1.1249908	0.26584
fetal lung 124	1.1095088	0.26596
CD8 20	1.1527399	0.26674
stomach 284	1.1385148	0.26744
fetal stomach 246	1.1361517	0.26882
fetal testes 255	1.1144698	0.26886
fetal small intestine 70	1.1132949	0.27564
fetal small intestine 78	1.1127655	0.27820
hematopoietic stem cell 317	1.1020497	0.27904
fetal kidney 92	1.1056524	0.28210
fetal large intestine 64	1.1178169	0.28248
fetal large intestine 55	1.1134314	0.28400
stomach 283	1.1228402	0.28422
hematopoietic stem cell 306	1.1065947	0.28572
fetal heart 49	1.1088798	0.28890
fetal heart 42	1.1052363	0.29116
fetal kidney 99	1.1038116	0.29156
fetal muscle 144	1.1044634	0.29400
fetal muscle 162	1.0973919	0.29426
fetal muscle 188	1.0974957	0.29510
fetal renal pelvis 231	1.0953366	0.29826
fetal lung 115	1.0869212	0.29874
fetal lung 119	1.0878385	0.30030
fetal kidney 104	1.0985530	0.30086
fetal kidney 105	1.0945890	0.30178
fetal renal pelvis 219	1.1139789	0.30404
fetal lung 135	1.0902652	0.30594
fetal stomach 253	1.1072276	0.30632
CD19 6	1.1212734	0.30648
fetal renal cortex 206	1.0912830	0.30752
fetal small intestine 71	1.0998151	0.30798
fetal lung 136	1.0870043	0.31042
fetal renal pelvis 228	1.0971248	0.31074
fetal renal cortex 214	1.0931510	0.31156
fetal heart 46	1.0957582	0.31424
fetal small intestine 68	1.0934889	0.31762
ES cells 296	1.0964540	0.31814
fetal renal pelvis 232	1.0897942	0.31940
fetal kidney 86	1.0973114	0.32190
fetal lung 131	1.0728297	0.32252
fetal stomach 245	1.0959492	0.32342
ES derived NP 291	1.0943254	0.32362
fetal adrenal gland 28	1.0763637	0.32388
fetal renal cortex 202	1.0816592	0.32480
pancreas 328	1.0934253	0.32776
fetal renal cortex 213	1.0795567	0.32824
fetal kidney 98	1.0847433	0.32890
CD19 5	1.1048646	0.32898
fetal stomach 243	1.0857536	0.32910
fetal kidney 84	1.0828117	0.33004
fetal large intestine 60	1.0872254	0.33340
fetal heart 52	1.0723393	0.33434
fetal renal pelvis 230	1.0849112	0.33462
fetal adrenal gland 27	1.0810974	0.33462
fetal heart 50	1.0793682	0.33554
fetal muscle 177	1.0787958	0.33604
fetal stomach 240	1.0901429	0.33630
fetal renal cortex 205	1.0760258	0.33916
ES derived trophoblast 288	1.0726614	0.34036
fetal testes 254	1.0769411	0.34094
fetal heart 51	1.0746856	0.34236
fetal small intestine 77	1.0802961	0.34566
fetal renal pelvis 233	1.0740332	0.34720
fetal muscle 190	1.0699292	0.35096
fetal heart 44	1.0786495	0.35286
fetal large intestine 57	1.0716536	0.35334
muscle 341	1.0772983	0.35368
fetal kidney 102	1.0679181	0.35714
fetal renal cortex 215	1.0614540	0.36182
fetal heart 48	1.0592535	0.36894
fetal small intestine 73	1.0660389	0.37018
fetal renal pelvis 221	1.0564518	0.37200
fetal renal pelvis 226	1.0573773	0.37438
fetal kidney 107	1.0566466	0.37512
fetal kidney 96	1.0505167	0.38302
heart 297	1.0531882	0.38550
ovary 326	1.0566400	0.38686
fetal renal cortex 208	1.0505434	0.38916
fetal renal pelvis 222	1.0489013	0.39150
fetal small intestine 76	1.0535532	0.39268
fetal stomach 251	1.0497749	0.39320
fetal kidney 87	1.0452330	0.39558
fetal large intestine 62	1.0519706	0.39572
fetal kidney 88	1.0449280	0.39582
CD3 10	1.0552466	0.39686
fetal renal pelvis 223	1.0456597	0.40152
fetal renal pelvis 225	1.0430756	0.40158
fetal large intestine 54	1.0461828	0.40188
fetal kidney 97	1.0404560	0.40284
ES derived trophoblast 287	1.0428838	0.40966
fetal adrenal gland 24	1.0399698	0.41176
CD56 17	1.0428697	0.41346
CD4 14	1.0443875	0.41410
fetal renal pelvis 224	1.0313234	0.41934
CD4 16	1.0354943	0.42534
fetal large intestine 63	1.0334576	0.42728
hematopoietic stem cell 312	1.0315768	0.42740
ES cells 293	1.0278826	0.42812
ES derived mesoderm 285	1.0272998	0.42978
fetal heart 43	1.0225271	0.43178
fetal large intestine 66	1.0292354	0.43368
fetal small intestine 72	1.0281755	0.43636
ES derived mesoderm 286	1.0199838	0.43758
fetal renal cortex 211	1.0232492	0.43916
fetal large intestine 59	1.0223331	0.44272
fetal spinal cord 236	1.0186832	0.44570
iPS 304	1.0182984	0.44612
fetal renal cortex 203	1.0181624	0.44670
fetal adrenal gland 23	1.0178649	0.44982
fetal renal pelvis 227	1.0176333	0.45078
fetal renal cortex 210	1.0174977	0.45110
fetal renal cortex 209	1.0166090	0.45200
fetal renal pelvis 218	1.0166476	0.45202
iPS 303	1.0123178	0.46112
fetal kidney 101	1.0110174	0.46408
fetal renal cortex 204	1.0098938	0.46748
fetal spinal cord 239	1.0085265	0.46872
fetal renal cortex 212	1.0062317	0.47558
fetal renal pelvis 229	1.0000212	0.48348
fetal kidney 90	0.9994281	0.48686
fetal adrenal gland 25	0.9989382	0.48926
fetal large intestine 65	0.9920738	0.49782
fetal kidney 93	0.9924065	0.49784
fetal small intestine 79	0.9890338	0.50016
fetal large intestine 56	0.9928256	0.50048
fetal kidney 82	0.9895704	0.50170
pancreas 327	0.9856681	0.50406
fetal renal pelvis 216	0.9883679	0.50968
small intestine 342	0.9857424	0.51324
placenta 199	0.9830790	0.52034
iPS 302	0.9806642	0.52936
fetal spinal cord 238	0.9717463	0.54714
placenta 200	0.9526535	0.59030
fetal spinal cord 235	0.9542628	0.59068
placenta 197	0.9406171	0.59782
fetal spinal cord 237	0.9465144	0.60756
placenta 196	0.9288474	0.62098
fetal brain 37	0.9387989	0.64526
fetal brain 40	0.9239491	0.70070
fetal brain 31	0.9222083	0.72500
fetal brain 36	0.8994777	0.72992
placenta 201	0.8588361	0.73590
fetal brain 38	0.8970815	0.73616
CD14 4	0.8202091	0.75886
placenta 198	0.8562092	0.76172
CD14 3	0.8091876	0.76454
fetal brain 41	0.8855000	0.76722
fetal brain 32	0.8788575	0.77298
fetal brain 39	0.8694437	0.81394
fetal brain 33	0.8418129	0.81666
fetal brain 34	0.8667065	0.82890
fetal brain 35	0.8619146	0.84030
fetal brain 30	0.8741151	0.85210
fetal brain 29	0.8367939	0.90910

Lipid levels 2

DHS sample	fold enrichment	p value
fetal adrenal gland 28	1.4115965	0.00816
stomach 284	1.4607811	0.01628
fetal adrenal gland 24	1.4382005	0.02058
small intestine 342	1.3917613	0.02358
fetal adrenal gland 23	1.3415079	0.02654
fetal adrenal gland 25	1.3896617	0.02690
CD14 4	1.4099038	0.02766
melanocyte 338	1.2661290	0.02804
placenta 200	1.3045973	0.03318
fetal thymus 256	1.3868375	0.03608
pancreas 328	1.3785626	0.03890
fetal large intestine 64	1.3174870	0.04692
fetal large intestine 56	1.3207955	0.04960
fetal small intestine 78	1.2796743	0.05362
fetal lung 301	1.2751882	0.05658
fetal lung 300	1.2703720	0.05736
melanocyte 337	1.2092372	0.05984
fibroblast 332	1.2470883	0.06010
CD14 3	1.3460000	0.06210
fetal large intestine 57	1.2754708	0.06238
muscle 341	1.3300907	0.06416
placenta 198	1.2502188	0.06570
keratinocyte 336	1.2195158	0.06654
fetal small intestine 76	1.3175359	0.06706
fetal lung 298	1.2668624	0.06902
fetal thymus 265	1.3228566	0.07226
fetal small intestine 74	1.2768660	0.07494
CD20 8	1.2678545	0.07508
fetal small intestine 69	1.3052529	0.07548
fetal fibroblast 269	1.2305752	0.07582
fibroblast 329	1.2351016	0.07680
fetal small intestine 70	1.2415359	0.07884
fetal large intestine 60	1.2768970	0.07902
fibroblast 331	1.2282630	0.08002
fetal small intestine 73	1.2784560	0.08438
fetal thymus 258	1.3026707	0.08480
fetal small intestine 71	1.2791628	0.08538
keratinocyte 333	1.1918682	0.08542
fetal small intestine 79	1.2893004	0.08716
fetal lung 299	1.2404979	0.08816
fetal large intestine 65	1.2697599	0.08872
stomach 283	1.2902893	0.09010
fetal large intestine 58	1.2354620	0.09076
fetal adrenal gland 27	1.2415580	0.09440
fetal large intestine 55	1.2422347	0.09482
breast 1	1.1991687	0.09494
fetal small intestine 72	1.2474790	0.09814
fetal large intestine 59	1.2602722	0.10128
fetal thymus 260	1.2811518	0.10212
fetal adrenal gland 26	1.2340066	0.10448
fibroblast 330	1.2093461	0.10460
fetal large intestine 67	1.2463400	0.10508
fetal large intestine 62	1.2629632	0.10616
fetal small intestine 77	1.2484074	0.10660
pancreas 327	1.2709889	0.10788
ES derived MSC 290	1.2257641	0.10974
fetal small intestine 68	1.2199891	0.11406
fetal large intestine 54	1.2394511	0.11430
fetal fibroblast 273	1.1898927	0.11600
fetal fibroblast 279	1.1892915	0.11748
fetal small intestine 80	1.2210786	0.11780
keratinocyte 334	1.1673528	0.11902
ES derived MSC 289	1.2151267	0.12046
fetal thymus 264	1.2435307	0.12278
keratinocyte 335	1.1642784	0.12410
breast 2	1.1711036	0.12508
melanocyte 340	1.1753821	0.12534
fetal brain 41	1.1751590	0.12678
fetal fibroblast 280	1.1796836	0.12822
fetal thymus 259	1.2566706	0.12956
placenta 197	1.2030025	0.13486
fetal large intestine 63	1.2207553	0.13630
melanocyte 339	1.1534319	0.14124
fetal brain 34	1.1494396	0.14216
fetal large intestine 53	1.2283531	0.14570
fetal fibroblast 268	1.1585254	0.15460
ES derived trophoblast 288	1.1726893	0.15568
CD56 17	1.2021772	0.16302
fetal fibroblast 274	1.1568139	0.16388
fetal fibroblast 282	1.1466563	0.16488
ES derived mesoderm 286	1.1369065	0.16668
CD4 13	1.2282303	0.16694
fetal brain 35	1.1314588	0.16756
Mobilized CD8 325	1.2332324	0.16884
fetal thymus 266	1.1978818	0.17318
fetal fibroblast 270	1.1495676	0.17366
CD56 18	1.2178640	0.17804
placenta 201	1.1729511	0.18068
fetal brain 38	1.1379650	0.18660
Mobilized CD56 324	1.1883708	0.18972
fetal large intestine 66	1.1711180	0.19272
fetal fibroblast 281	1.1353305	0.19304
fetal fibroblast 278	1.1367532	0.19696
fetal brain 39	1.1188845	0.20234
fetal stomach 244	1.1615799	0.20346
fetal fibroblast 277	1.1313034	0.20634
CD8 19	1.2026207	0.21138
fetal stomach 253	1.1676936	0.21158
fetal brain 31	1.0978845	0.21956
fetal brain 29	1.0972739	0.22234
ES cells 296	1.1568895	0.22282
fetal fibroblast 271	1.1165526	0.22630
fetal fibroblast 267	1.1197858	0.22728
fetal skin 234	1.1356724	0.22946
CD8 21	1.1712637	0.23498
placenta 199	1.1199771	0.23618
ES cells 294	1.1422578	0.23642
fetal thymus 261	1.1512146	0.23694
CD8 20	1.1503792	0.23774
fetal fibroblast 276	1.1064893	0.24142
fetal fibroblast 272	1.1084266	0.24148
fetal renal cortex 212	1.1222225	0.24374
iPS 304	1.1106142	0.24916
CD4 14	1.1409714	0.24922
fetal lung 134	1.1089123	0.24930
placenta 196	1.1209776	0.25048
fetal brain 36	1.0974298	0.25232
CD8 22	1.1491447	0.25600
Mobilized CD3 321	1.1472225	0.26706
ES derived trophoblast 287	1.1121222	0.26792
fetal fibroblast 275	1.0939506	0.26814
fetal renal pelvis 233	1.1109369	0.26990
heart 297	1.1145736	0.27008
fetal renal pelvis 220	1.1127854	0.27188
fetal thymus 262	1.1440900	0.27496
fetal brain 30	1.0730260	0.27570
CD3 10	1.1219698	0.27866
fetal renal cortex 207	1.1026245	0.27886
fetal stomach 242	1.1307915	0.27974
fetal kidney 95	1.1019450	0.28148
ovary 326	1.1194197	0.28250
fetal thymus 257	1.1232143	0.28424
fetal brain 40	1.0792534	0.28572
fetal kidney 91	1.0936779	0.28592
Mobilized CD4 322	1.1266308	0.29028
CD3 11	1.1134996	0.29034
hematopoietic stem cell 310	1.0807198	0.29334
fetal renal cortex 205	1.0957027	0.29578
hematopoietic stem cell 309	1.0902905	0.29676
CD3 9	1.1146752	0.30004
fetal renal pelvis 219	1.1080104	0.30396
ES derived mesoderm 285	1.0784574	0.30412
fetal renal cortex 214	1.0878734	0.30460
fetal renal pelvis 231	1.0836259	0.30496
fetal renal pelvis 227	1.0882309	0.30586
Mobilized CD4 323	1.1110857	0.30800
fetal renal pelvis 232	1.0898496	0.30870
fetal renal pelvis 224	1.0833249	0.30880
fetal kidney 106	1.0830089	0.31002
fetal renal pelvis 228	1.0915539	0.31216
fetal renal pelvis 221	1.0812332	0.31292
fetal kidney 103	1.0829795	0.31436
fetal thymus 263	1.0818204	0.31464
fetal lung 132	1.0737439	0.31608
fetal kidney 83	1.0911573	0.31760
fetal kidney 84	1.0840976	0.31960
fetal stomach 247	1.0971535	0.32040
CD19 5	1.0953133	0.32398
fetal large intestine 61	1.0947023	0.32430
fetal spinal cord 237	1.0718330	0.32510
fetal stomach 240	1.0877797	0.32648
fetal kidney 87	1.0790815	0.32656
fetal renal pelvis 225	1.0780376	0.33084
fetal kidney 89	1.0766208	0.33086
CD4 16	1.0955142	0.33108
fetal renal cortex 206	1.0731417	0.33146
CD4 15	1.1067961	0.33386
Mobilized CD3 320	1.0954931	0.33540
iPS 302	1.0678322	0.33660
fetal lung 140	1.0621223	0.33820
hematopoietic stem cell 318	1.0728455	0.33852
fetal kidney 99	1.0677174	0.33942
fetal kidney 82	1.0772418	0.34016
fetal kidney 105	1.0683043	0.34068
fetal lung 120	1.0608247	0.34346
fetal muscle 169	1.0661759	0.34396
fetal kidney 100	1.0663833	0.34610
fetal stomach 248	1.0724632	0.34744
fetal renal cortex 211	1.0689431	0.34954
fetal lung 125	1.0579537	0.35158
hematopoietic stem cell 314	1.0579638	0.35210
fetal stomach 243	1.0645140	0.35406
fetal kidney 96	1.0590000	0.35450
fetal kidney 86	1.0715545	0.35454
fetal muscle 153	1.0632706	0.35502
fetal renal pelvis 229	1.0597457	0.35522
fetal lung 128	1.0516628	0.35566
fetal kidney 104	1.0619111	0.35758
CD3 12	1.0762486	0.35902
hematopoietic stem cell 315	1.0596216	0.35906
CD19 7	1.0731662	0.36006
hematopoietic stem cell 312	1.0552748	0.36234
fetal lung 127	1.0530584	0.36352
fetal stomach 250	1.0631188	0.36394
fetal kidney 92	1.0516559	0.36400
fetal stomach 252	1.0599744	0.36640
fetal stomach 241	1.0619234	0.36718
fetal brain 37	1.0455401	0.36804
iPS 303	1.0527639	0.36808
hematopoietic stem cell 316	1.0543949	0.37020
CD19 6	1.0651972	0.37306
fetal lung 129	1.0484038	0.37326
fetal lung 108	1.0507902	0.37348
hematopoietic stem cell 308	1.0478572	0.37434
fetal renal cortex 210	1.0530307	0.37648
hematopoietic stem cell 307	1.0501256	0.37648
hematopoietic stem cell 305	1.0506804	0.37798
fetal kidney 81	1.0471308	0.37866
fetal renal pelvis 217	1.0476547	0.38090
fetal renal cortex 215	1.0451173	0.38220
ES cells 293	1.0476455	0.38580
fetal renal cortex 202	1.0440624	0.38606
fetal muscle 172	1.0423193	0.38676
fetal muscle 151	1.0461064	0.38864
fetal renal pelvis 218	1.0493437	0.38982
fetal spinal cord 236	1.0425086	0.38984
fetal muscle 175	1.0416493	0.39072
fetal muscle 157	1.0415372	0.39084
fetal stomach 246	1.0447681	0.39472
fetal lung 123	1.0376668	0.39602
fetal lung 118	1.0360148	0.39696
fetal renal pelvis 223	1.0411296	0.39794
fetal lung 112	1.0344685	0.40068
fetal renal cortex 204	1.0360667	0.40114
fetal lung 110	1.0355708	0.40142
ES cells 295	1.0424896	0.40244
fetal lung 136	1.0352159	0.40270
fetal lung 109	1.0317809	0.40322
fetal testes 254	1.0369124	0.40482
fetal lung 117	1.0333078	0.40546
fetal testes 255	1.0343930	0.40780
fetal muscle 191	1.0358174	0.40810
fetal muscle 144	1.0354061	0.40890
fetal ovary 195	1.0371564	0.40976
fetal lung 137	1.0306524	0.40998
fetal lung 119	1.0314053	0.41070
fetal renal cortex 213	1.0316300	0.41098
fetal muscle 194	1.0320036	0.41132
hematopoietic stem cell 313	1.0311224	0.41272
fetal kidney 88	1.0302885	0.41494
fetal muscle 189	1.0307225	0.41608
fetal kidney 102	1.0271560	0.41948
hematopoietic stem cell 319	1.0286009	0.42254
fetal kidney 85	1.0209115	0.42428
fetal lung 121	1.0257894	0.42430
fetal stomach 249	1.0284790	0.42500
fetal brain 32	1.0244828	0.42550
fetal muscle 159	1.0275912	0.42606
fetal small intestine 75	1.0204589	0.42634
fetal kidney 98	1.0241415	0.42764
fetal muscle 185	1.0265709	0.42786
fetal kidney 101	1.0249530	0.42804
fetal muscle 154	1.0260119	0.43020
fetal renal pelvis 226	1.0217835	0.43270
fetal muscle 193	1.0223735	0.43468
fetal muscle 184	1.0193370	0.43802
fetal lung 138	1.0187097	0.44066
fetal lung 116	1.0161105	0.44738
fetal muscle 146	1.0168785	0.44756
fetal kidney 97	1.0143212	0.44756
fetal lung 113	1.0155715	0.44794
hematopoietic stem cell 306	1.0150600	0.45122
fetal stomach 251	1.0137736	0.45156
fetal renal cortex 208	1.0118424	0.45236
fetal kidney 90	1.0118885	0.45424
fetal heart 52	1.0122602	0.45540
fetal lung 130	1.0111803	0.45922
fetal muscle 168	1.0121063	0.46040
fetal muscle 187	1.0105750	0.46108
fetal heart 46	1.0095892	0.46126
fetal heart 45	1.0092138	0.46214
fetal lung 131	1.0091995	0.46248
fetal kidney 107	1.0065898	0.46288
hematopoietic stem cell 317	1.0099849	0.46290
fetal muscle 164	1.0095451	0.46504
fetal renal pelvis 216	1.0051137	0.46760
fetal spinal cord 238	1.0058364	0.46868
fetal renal pelvis 230	1.0022212	0.46902
fetal muscle 182	1.0069897	0.47014
fetal heart 42	1.0048595	0.47132
fetal muscle 150	1.0060764	0.47216
fetal lung 133	1.0059610	0.47332
fetal lung 139	1.0036828	0.47370
fetal spinal cord 235	1.0023039	0.47382
fetal muscle 166	1.0056116	0.47388
fetal muscle 145	1.0001752	0.48020
fetal renal cortex 209	0.9986849	0.48128
fetal muscle 174	0.9984585	0.48790
fetal lung 135	0.9981847	0.48840
fetal spinal cord 239	0.9980253	0.48958
fetal renal pelvis 222	0.9921188	0.49236
fetal muscle 155	0.9930473	0.49306
fetal lung 122	0.9936335	0.49434
fetal lung 115	0.9971653	0.49750
fetal muscle 183	0.9944807	0.49920
fetal kidney 94	0.9853642	0.49980
fetal muscle 188	0.9916133	0.50296
fetal muscle 148	0.9919759	0.50334
hematopoietic stem cell 311	0.9900272	0.50678
fetal stomach 245	0.9850693	0.50710
fetal muscle 158	0.9892873	0.50886
fetal lung 141	0.9916716	0.51132
fetal brain 33	0.9915931	0.51184
fetal lung 124	0.9889386	0.51248
fetal kidney 93	0.9835519	0.51250
fetal muscle 165	0.9882096	0.51366
fetal muscle 170	0.9868005	0.51456
fetal lung 126	0.9897532	0.51560
fetal heart 49	0.9847314	0.51678
fetal muscle 152	0.9866757	0.51720
fetal muscle 147	0.9823562	0.51804
fetal muscle 156	0.9856841	0.51890
fetal muscle 173	0.9830798	0.51918
fetal heart 43	0.9852570	0.51928
fetal lung 114	0.9860304	0.52922
fetal muscle 192	0.9800021	0.53116
fetal muscle 186	0.9754376	0.53780
fetal muscle 167	0.9785928	0.53848
fetal muscle 171	0.9770194	0.53898
fetal lung 111	0.9803170	0.53926
fetal muscle 190	0.9687166	0.54242
fetal lung 142	0.9786848	0.54490
fetal muscle 177	0.9682500	0.54554
ES derived NP 292	0.9674426	0.54662
fetal muscle 179	0.9715704	0.54740
fetal muscle 178	0.9683456	0.55354
fetal muscle 162	0.9697858	0.55418
fetal muscle 160	0.9676212	0.55826
fetal muscle 149	0.9623172	0.56510
fetal muscle 181	0.9619021	0.56554
fetal heart 50	0.9620370	0.56572
fetal muscle 176	0.9622181	0.57412
fetal muscle 163	0.9547166	0.57952
fetal muscle 180	0.9530335	0.58944
fetal heart 48	0.9527631	0.58960
ES derived NP 291	0.9414709	0.59378
fetal renal cortex 203	0.9424919	0.59790
fetal muscle 161	0.9424037	0.60446
fetal heart 51	0.9440696	0.61066
fetal heart 47	0.9341186	0.61806
fetal heart 44	0.9253737	0.62314
fetal muscle 143	0.9367829	0.64014

Lipid levels 1

DHS sample	fold enrichment	p value
fetal adrenal gland 28	1.3313224	0.01370
CD14 4	1.3521633	0.03360
fetal adrenal gland 23	1.2828201	0.03826
fetal adrenal gland 24	1.3179841	0.04972
melanocyte 338	1.2045884	0.05270
fetal thymus 256	1.2865788	0.05774
placenta 200	1.2207368	0.06110
fetal brain 34	1.1970424	0.06730
fetal adrenal gland 25	1.2661981	0.06886
small intestine 342	1.2464862	0.06994
fetal brain 35	1.1790784	0.07422
CD14 3	1.2855885	0.07864
fetal large intestine 56	1.2356980	0.08090
fibroblast 332	1.1868652	0.08240
stomach 284	1.2477920	0.08390
fetal large intestine 60	1.2254189	0.09406
keratinocyte 336	1.1601892	0.09514
CD20 8	1.2040820	0.09818
fibroblast 329	1.1742131	0.09976
melanocyte 337	1.1548672	0.10236
fibroblast 331	1.1709119	0.10376
fetal small intestine 76	1.2230554	0.10440
placenta 198	1.1851200	0.10626
fetal large intestine 64	1.1985558	0.10678
fetal large intestine 67	1.2097370	0.10708
fetal small intestine 78	1.1869413	0.10760
pancreas 328	1.2198942	0.10866
fetal small intestine 70	1.1792135	0.11122
fetal thymus 258	1.2279325	0.11186
fetal thymus 265	1.2242660	0.11710
fetal large intestine 59	1.2110938	0.11808
fetal brain 41	1.1660742	0.12360
fetal small intestine 73	1.1989937	0.12516
Mobilized CD56 324	1.2240890	0.12804
fetal skin 234	1.1903704	0.12924
fetal brain 29	1.1335803	0.13210
fetal large intestine 57	1.1669875	0.13238
muscle 341	1.2015339	0.13434
keratinocyte 333	1.1326434	0.13538
fetal small intestine 74	1.1825189	0.13570
fetal adrenal gland 27	1.1820041	0.13656
fetal fibroblast 269	1.1480361	0.13708
fetal large intestine 58	1.1656705	0.13782
fetal large intestine 62	1.1942525	0.13792
fetal lung 301	1.1568905	0.14100
fetal large intestine 55	1.1689485	0.14112
fetal small intestine 79	1.1924040	0.14244
fetal large intestine 65	1.1828757	0.14344
fetal small intestine 69	1.1906423	0.14350
fetal small intestine 77	1.1805915	0.14422
fetal small intestine 71	1.1826435	0.14490
fetal brain 39	1.1424867	0.14554
fetal brain 31	1.1225045	0.14674
fibroblast 330	1.1451910	0.14764
breast 1	1.1357387	0.14816
fetal brain 30	1.1204048	0.14834
CD4 13	1.2242595	0.14916
fetal brain 36	1.1447118	0.14946
fetal brain 38	1.1477836	0.15402
fetal lung 300	1.1431636	0.15402
fetal small intestine 72	1.1681939	0.15468
fetal small intestine 68	1.1587923	0.15654
fetal lung 298	1.1507982	0.15794
melanocyte 340	1.1369467	0.16016
ES derived mesoderm 286	1.1304325	0.16128
fetal small intestine 80	1.1577393	0.16130
fetal thymus 260	1.1885142	0.16278
CD56 17	1.1730909	0.16284
fetal large intestine 53	1.1845975	0.16720
keratinocyte 334	1.1197480	0.16762
breast 2	1.1261293	0.17210
ES derived MSC 289	1.1391177	0.17988
fetal kidney 91	1.1427040	0.18016
fetal fibroblast 273	1.1230883	0.18048
fetal adrenal gland 26	1.1489722	0.18232
fetal large intestine 54	1.1494119	0.18342
CD8 21	1.1899595	0.18346
placenta 197	1.1428229	0.18714
CD4 14	1.1616932	0.18740
fetal brain 40	1.1164040	0.19006
placenta 201	1.1444521	0.19198
pancreas 327	1.1597421	0.19520
melanocyte 339	1.1101355	0.19552
keratinocyte 335	1.1036696	0.19986
fetal thymus 264	1.1503366	0.20068
CD56 18	1.1718490	0.20130
fetal lung 299	1.1212094	0.20308
ES derived MSC 290	1.1256005	0.20388
Mobilized CD8 325	1.1741650	0.20392
fetal fibroblast 282	1.1085515	0.20418
fetal brain 37	1.1142715	0.20436
fetal large intestine 63	1.1432438	0.20514
fetal thymus 259	1.1596591	0.20744
fetal fibroblast 279	1.1118043	0.21020
ES cells 296	1.1502197	0.21274
CD3 11	1.1422527	0.21390
ES derived trophoblast 288	1.1172616	0.22032
fetal renal cortex 212	1.1202263	0.22146
CD8 19	1.1642938	0.22596
fetal renal cortex 207	1.1215880	0.22644
ES cells 294	1.1411736	0.22658
fetal fibroblast 274	1.1012983	0.22726
fetal muscle 169	1.1161950	0.23132
stomach 283	1.1300123	0.23280
iPS 304	1.1091823	0.23624
CD8 20	1.1284943	0.23682
fetal large intestine 66	1.1207137	0.23910
CD3 10	1.1324611	0.24182
fetal stomach 244	1.1221785	0.24216
fetal renal pelvis 220	1.1162829	0.24300
Mobilized CD3 320	1.1451998	0.24958
CD3 9	1.1322232	0.25036
fetal fibroblast 268	1.0886710	0.25250
fetal fibroblast 280	1.0886566	0.25484
iPS 302	1.1021508	0.25854
fetal thymus 263	1.0958795	0.26970
fetal renal pelvis 231	1.0948796	0.26982
Mobilized CD4 323	1.1219832	0.27028
fetal renal pelvis 233	1.0975274	0.27390
CD8 22	1.1144259	0.27568
placenta 199	1.0865637	0.27664
fetal kidney 95	1.0934030	0.27782
Mobilized CD4 322	1.1182722	0.27836
fetal renal cortex 214	1.0935353	0.27916
fetal renal cortex 205	1.0904280	0.28382
Mobilized CD3 321	1.1162272	0.28452
fetal kidney 103	1.0905285	0.28552
fetal stomach 253	1.1027902	0.28614
fetal thymus 266	1.1003028	0.28782
hematopoietic stem cell 310	1.0735828	0.28860
iPS 303	1.0842479	0.28912
fetal renal pelvis 227	1.0878052	0.28996
fetal muscle 151	1.0897738	0.29034
fetal fibroblast 281	1.0729436	0.29034
fetal renal pelvis 224	1.0835498	0.29208
fetal renal cortex 213	1.0787899	0.29768
fetal renal pelvis 219	1.1014584	0.29832
ES derived mesoderm 285	1.0734041	0.29840
fetal kidney 106	1.0808602	0.29974
fetal muscle 153	1.0860344	0.30026
fetal large intestine 61	1.0987040	0.30058
CD19 7	1.1003859	0.30618
CD19 5	1.0956493	0.30800
fetal kidney 85	1.0690859	0.31106
fetal fibroblast 270	1.0659800	0.31230
fetal renal pelvis 221	1.0753815	0.31258
fetal fibroblast 277	1.0663529	0.31274
fetal lung 140	1.0655409	0.31586
placenta 196	1.0750503	0.31610
fetal fibroblast 271	1.0615205	0.31810
fetal lung 119	1.0633990	0.31844
fetal kidney 83	1.0811251	0.31980
fetal brain 32	1.0619835	0.31992
fetal renal pelvis 228	1.0781225	0.32114
fetal kidney 84	1.0730371	0.32388
fetal kidney 99	1.0686526	0.33022
fetal kidney 87	1.0666415	0.33092
fetal lung 134	1.0617101	0.33204
fetal renal cortex 215	1.0637357	0.33356
fetal renal pelvis 225	1.0686761	0.33392
fetal renal pelvis 232	1.0694731	0.33694
fetal kidney 105	1.0631572	0.33804
fetal fibroblast 278	1.0568703	0.33824
fetal thymus 261	1.0747527	0.33826
fetal muscle 154	1.0659859	0.33920
fetal renal pelvis 229	1.0629798	0.33940
fetal spinal cord 237	1.0589881	0.34008
fetal kidney 92	1.0611993	0.34254
fetal renal cortex 206	1.0594315	0.34386
hematopoietic stem cell 314	1.0529537	0.35018
fetal kidney 82	1.0634899	0.35114
CD19 6	1.0730237	0.35146
fetal kidney 100	1.0577412	0.35158
fetal fibroblast 267	1.0510326	0.35354
CD3 12	1.0693383	0.35552
fetal muscle 167	1.0518126	0.35644
fetal kidney 88	1.0539431	0.35676
hematopoietic stem cell 312	1.0528388	0.35712
fetal lung 120	1.0503169	0.35722
fetal lung 128	1.0440842	0.35830
CD4 16	1.0681899	0.35848
fetal muscle 182	1.0526445	0.35896
fetal fibroblast 276	1.0453038	0.36050
fetal lung 132	1.0482734	0.36248
fetal muscle 164	1.0508769	0.36414
fetal kidney 104	1.0539793	0.36502
fetal muscle 157	1.0482860	0.36528
fetal lung 112	1.0470675	0.36542
fetal brain 33	1.0464128	0.36550
fetal stomach 243	1.0519606	0.36604
hematopoietic stem cell 318	1.0523304	0.36818
fetal kidney 96	1.0488927	0.36892
fetal fibroblast 272	1.0431652	0.36948
ES derived trophoblast 287	1.0535189	0.37004
hematopoietic stem cell 305	1.0499561	0.37098
fetal spinal cord 238	1.0456950	0.37268
fetal spinal cord 235	1.0460227	0.37296
fetal lung 118	1.0409007	0.37366
hematopoietic stem cell 309	1.0470575	0.37384
fetal lung 117	1.0430777	0.37464
hematopoietic stem cell 315	1.0474842	0.37602
fetal muscle 166	1.0441573	0.37616
ES cells 293	1.0496198	0.37668
fetal muscle 187	1.0427603	0.37746
fetal thymus 257	1.0548508	0.37782
fetal muscle 175	1.0433670	0.37796
fetal stomach 242	1.0548615	0.37812
CD4 15	1.0614310	0.38020
fetal thymus 262	1.0572790	0.38112
fetal muscle 178	1.0438956	0.38242
fetal spinal cord 236	1.0434120	0.38410
fetal kidney 89	1.0441411	0.38424
fetal kidney 101	1.0416186	0.38576
fetal muscle 170	1.0397931	0.38678
fetal muscle 185	1.0407865	0.39176
fetal renal pelvis 217	1.0412513	0.39202
fetal testes 255	1.0379369	0.39208
fetal muscle 159	1.0401553	0.39276
fetal renal pelvis 226	1.0394086	0.39454
fetal muscle 150	1.0386008	0.39656
fetal muscle 184	1.0349689	0.39758
fetal fibroblast 275	1.0312391	0.39816
fetal renal cortex 204	1.0351619	0.39934
fetal muscle 171	1.0350357	0.39992
fetal renal cortex 202	1.0357244	0.40080
fetal lung 129	1.0320975	0.40244
fetal kidney 86	1.0379067	0.40536
fetal testes 254	1.0337707	0.40774
ES cells 295	1.0362455	0.40890
fetal lung 125	1.0294901	0.41126
fetal muscle 168	1.0329487	0.41136
fetal renal cortex 211	1.0309753	0.41212
fetal stomach 248	1.0320706	0.41450
fetal lung 127	1.0282510	0.41494
fetal muscle 176	1.0289150	0.41554
fetal stomach 247	1.0298611	0.42210
fetal kidney 81	1.0249206	0.42452
fetal lung 123	1.0237863	0.42590
fetal muscle 191	1.0240162	0.42636
fetal muscle 193	1.0247308	0.42640
fetal muscle 149	1.0240854	0.42936
fetal muscle 194	1.0213185	0.42982
fetal renal pelvis 223	1.0244895	0.43016
hematopoietic stem cell 316	1.0241363	0.43066
ES derived NP 291	1.0260758	0.43232
fetal small intestine 75	1.0212723	0.43314
fetal muscle 174	1.0225831	0.43324
hematopoietic stem cell 308	1.0222097	0.43354
fetal kidney 102	1.0223560	0.43370
fetal muscle 158	1.0210418	0.43374
fetal muscle 180	1.0226775	0.43554
fetal muscle 144	1.0210071	0.43556
fetal muscle 183	1.0194742	0.43638
fetal muscle 165	1.0204460	0.43762
fetal muscle 146	1.0190678	0.43818
fetal stomach 240	1.0186703	0.44036
fetal lung 109	1.0173924	0.44060
fetal muscle 172	1.0184554	0.44078
fetal muscle 156	1.0165119	0.44604
fetal kidney 90	1.0147219	0.44968
fetal lung 136	1.0156234	0.44996
fetal renal cortex 210	1.0144125	0.45278
fetal muscle 152	1.0136718	0.45328
fetal lung 116	1.0130596	0.45448
fetal lung 108	1.0116295	0.45766
heart 297	1.0115936	0.45964
fetal muscle 179	1.0119473	0.45966
fetal renal pelvis 218	1.0088015	0.46122
fetal kidney 98	1.0092940	0.46138
fetal renal cortex 208	1.0096991	0.46312
fetal muscle 189	1.0091061	0.46456
fetal muscle 181	1.0070372	0.46864
fetal stomach 250	1.0053294	0.47032
fetal kidney 97	1.0060284	0.47052
fetal stomach 252	1.0055324	0.47180
ovary 326	1.0047343	0.47338
fetal kidney 94	1.0043938	0.47344
hematopoietic stem cell 307	1.0050681	0.47542
fetal lung 111	1.0056484	0.47720
fetal stomach 241	1.0003120	0.47876
fetal muscle 173	1.0030930	0.47900
fetal lung 137	1.0033029	0.47902
fetal spinal cord 239	1.0032783	0.48224
fetal kidney 107	0.9986017	0.48512
fetal ovary 195	0.9958415	0.48664
fetal lung 121	1.0019615	0.48752
fetal muscle 188	1.0002100	0.48936
fetal muscle 145	0.9978538	0.48942
fetal lung 114	1.0014820	0.49200
fetal muscle 148	0.9980863	0.49266
fetal muscle 155	0.9973446	0.49392
ES derived NP 292	0.9945525	0.49564
hematopoietic stem cell 317	0.9981132	0.49728
fetal stomach 251	0.9929979	0.49916
fetal muscle 160	0.9957879	0.49946
hematopoietic stem cell 306	0.9949599	0.50072
fetal muscle 186	0.9938177	0.50266
fetal muscle 192	0.9939265	0.50400
fetal lung 126	0.9954202	0.50484
fetal renal pelvis 230	0.9883811	0.50612
fetal renal cortex 209	0.9910348	0.50662
fetal lung 113	0.9953725	0.50688
fetal muscle 143	0.9930839	0.50926
fetal stomach 246	0.9851080	0.50926
fetal lung 138	0.9911336	0.51398
hematopoietic stem cell 313	0.9906274	0.51570
fetal renal pelvis 216	0.9832792	0.52274
fetal muscle 147	0.9858483	0.52410
fetal lung 115	0.9821951	0.54036
hematopoietic stem cell 319	0.9757951	0.54092
fetal lung 133	0.9839622	0.54118
fetal lung 139	0.9789050	0.54422
fetal muscle 177	0.9749078	0.54488
fetal muscle 163	0.9760235	0.54830
fetal muscle 162	0.9776089	0.54864
fetal muscle 161	0.9741207	0.55054
fetal lung 110	0.9771082	0.55100
fetal lung 131	0.9799879	0.55316
fetal lung 130	0.9768551	0.55572
fetal kidney 93	0.9683631	0.55602
fetal stomach 249	0.9621302	0.55946
hematopoietic stem cell 311	0.9709022	0.55954
fetal lung 135	0.9745841	0.56126
fetal lung 141	0.9770142	0.56452
fetal renal pelvis 222	0.9639980	0.57074
fetal lung 124	0.9698293	0.57562
fetal muscle 190	0.9582054	0.58488
fetal renal cortex 203	0.9526370	0.59710
fetal heart 46	0.9494074	0.60854
fetal heart 52	0.9532451	0.61480
fetal stomach 245	0.9389143	0.61574
fetal lung 122	0.9483169	0.61828
fetal lung 142	0.9548495	0.62096
fetal heart 45	0.9329626	0.64806
fetal heart 43	0.9353432	0.65526
fetal heart 42	0.9290979	0.66508
fetal heart 49	0.9073622	0.70542
fetal heart 50	0.9104875	0.70728
fetal heart 48	0.8988298	0.72916
fetal heart 44	0.8744757	0.74056
fetal heart 51	0.8962095	0.74064
fetal heart 47	0.8618515	0.78104

Cognitive decline in Alzheimers disease

DHS sample	fold enrichment	p value
fetal lung 141	1.4520482	0.01146
fetal fibroblast 271	1.4295475	0.01272
fetal lung 111	1.4441111	0.01604
fetal fibroblast 273	1.3830567	0.02122
fetal lung 114	1.3833790	0.02186
fetal lung 120	1.3983664	0.02466
fetal lung 140	1.3936427	0.02526
fibroblast 330	1.3873388	0.02608
fetal lung 119	1.3618688	0.02870
fetal fibroblast 274	1.3692922	0.02946
fetal lung 117	1.3704050	0.03030
fetal lung 128	1.3301136	0.03088
fetal fibroblast 276	1.3516696	0.03146
fetal fibroblast 272	1.3517318	0.03216
fibroblast 329	1.3558618	0.03408
fetal lung 130	1.3597170	0.03484
fetal fibroblast 278	1.3551351	0.03538
ES derived trophoblast 287	1.4219610	0.03542
fetal lung 133	1.3391572	0.03656
fetal fibroblast 277	1.3502923	0.03796
fetal lung 300	1.3455298	0.04572
fibroblast 331	1.3339336	0.04614
fetal lung 121	1.3070629	0.04700
fetal fibroblast 282	1.3064535	0.04782
fetal fibroblast 281	1.3157059	0.04874
fetal lung 131	1.2921231	0.04878
fetal fibroblast 275	1.2988891	0.04908
fetal lung 113	1.3082317	0.04910
fetal lung 118	1.2870052	0.05282
fetal lung 298	1.3416381	0.05736
fetal large intestine 67	1.3746021	0.05804
fetal lung 142	1.2832262	0.06060
fetal fibroblast 280	1.2954609	0.06354
fetal lung 116	1.2714278	0.06626
fetal lung 301	1.2989843	0.07180
fetal lung 110	1.2961879	0.07252
fetal lung 125	1.2944575	0.07304
fetal lung 109	1.2563315	0.07340
fetal fibroblast 267	1.2845126	0.07352
ES derived trophoblast 288	1.2983048	0.07452
fibroblast 332	1.2757963	0.07712
fetal lung 126	1.2613215	0.07758
fetal fibroblast 270	1.2668235	0.08098
fetal muscle 172	1.2586295	0.08892
fetal lung 132	1.2652068	0.08898
fetal lung 129	1.2592680	0.09000
fetal muscle 194	1.2507376	0.09118
fetal lung 299	1.2805303	0.09296
fetal fibroblast 268	1.2406434	0.09618
fetal fibroblast 279	1.2504587	0.09764
small intestine 342	1.3001145	0.09776
fetal lung 115	1.2362745	0.09862
fetal lung 134	1.2495718	0.10220
fetal lung 138	1.2513555	0.10296
fetal large intestine 57	1.2689980	0.10770
fetal lung 112	1.2395580	0.11348
fetal lung 124	1.2334150	0.11626
fetal lung 127	1.2308057	0.12242
fetal lung 137	1.2029189	0.13366
fetal lung 139	1.2242249	0.13406
fetal lung 123	1.2183146	0.13544
fetal lung 136	1.2107934	0.14202
melanocyte 337	1.1675397	0.14748
fetal lung 135	1.2050930	0.15008
fetal muscle 184	1.1991091	0.15554
fetal small intestine 72	1.2272685	0.15954
fetal testes 255	1.1919158	0.16060
CD20 8	1.2096160	0.16326
fetal muscle 176	1.2049067	0.16646
fetal small intestine 80	1.2104800	0.16740
ES derived MSC 290	1.1994627	0.16752
fetal fibroblast 269	1.1717080	0.17174
fetal lung 108	1.1920745	0.17248
fetal muscle 157	1.1870503	0.17302
ES derived MSC 289	1.1844672	0.18608
fetal large intestine 64	1.1941312	0.18648
fetal renal cortex 207	1.1932312	0.18724
fetal small intestine 77	1.2075512	0.18782
fetal muscle 179	1.1873249	0.19116
fetal muscle 161	1.1825880	0.19148
fetal small intestine 70	1.1739812	0.19256
fetal large intestine 54	1.1918799	0.19298
fetal muscle 166	1.1847591	0.19488
fetal adrenal gland 28	1.1641261	0.19586
fetal muscle 181	1.1844769	0.19636
fetal muscle 187	1.1679490	0.19690
fetal muscle 143	1.1666581	0.19850
fetal small intestine 71	1.1864376	0.19958
fetal large intestine 66	1.1938151	0.20048
fetal stomach 252	1.1876859	0.20586
fetal muscle 180	1.1749377	0.21018
fetal muscle 188	1.1609794	0.21154
fetal large intestine 58	1.1659365	0.21270
melanocyte 338	1.1256992	0.21468
fetal muscle 162	1.1589992	0.21642
fetal brain 40	1.1305740	0.21764
fetal muscle 158	1.1556118	0.21990
fetal large intestine 60	1.1697061	0.22106
fetal large intestine 56	1.1633303	0.22480
fetal muscle 192	1.1484925	0.22510
fetal muscle 182	1.1499747	0.23082
fetal kidney 92	1.1485528	0.23086
fetal muscle 167	1.1511354	0.23126
fetal small intestine 68	1.1511058	0.23228
pancreas 328	1.1663930	0.23446
fetal muscle 177	1.1557573	0.23454
fetal lung 122	1.1378035	0.23738
melanocyte 339	1.1177002	0.23762
pancreas 327	1.1628996	0.23902
fetal small intestine 73	1.1541946	0.24128
fetal stomach 244	1.1534788	0.24280
fetal small intestine 69	1.1550246	0.24380
fetal small intestine 78	1.1375358	0.24602
fetal kidney 91	1.1302878	0.24748
placenta 201	1.1497705	0.24788
fetal muscle 178	1.1427141	0.24904
fetal small intestine 76	1.1533235	0.25082
fetal muscle 165	1.1285832	0.25508
fetal muscle 186	1.1303191	0.25650
fetal stomach 246	1.1507182	0.25760
fetal large intestine 65	1.1437423	0.25764
fetal muscle 175	1.1285113	0.25856
fetal kidney 85	1.1240787	0.25906
fetal muscle 150	1.1347723	0.25948
fetal renal pelvis 220	1.1353979	0.26086
fetal large intestine 53	1.1513428	0.26186
ES derived mesoderm 285	1.1114764	0.26550
fetal muscle 152	1.1244388	0.26878
fetal muscle 190	1.1309436	0.26958
fetal large intestine 59	1.1345328	0.27136
fetal large intestine 55	1.1242634	0.27352
heart 297	1.1288321	0.27550
fetal large intestine 63	1.1304722	0.27578
fetal muscle 154	1.1277613	0.27780
fetal large intestine 62	1.1326347	0.27798
fetal small intestine 79	1.1303233	0.27960
fetal muscle 183	1.1090449	0.28530
fetal brain 30	1.0788089	0.29008
fetal small intestine 74	1.1155371	0.29438
fetal kidney 103	1.1103844	0.29454
fetal muscle 189	1.1042033	0.29650
fetal stomach 251	1.1131332	0.29748
fetal kidney 106	1.1045011	0.29748
fetal brain 31	1.0751771	0.30178
fetal muscle 170	1.0995266	0.30234
fetal muscle 147	1.1031685	0.30368
ES derived mesoderm 286	1.0830146	0.30550
fetal muscle 164	1.1005764	0.30782
fetal muscle 146	1.0955027	0.31340
fetal renal cortex 213	1.0914731	0.31466
fetal muscle 185	1.0964300	0.31822
keratinocyte 336	1.0772228	0.32124
fetal muscle 144	1.0913761	0.32218
fetal skin 234	1.0921871	0.32346
fetal kidney 100	1.0869420	0.32520
melanocyte 340	1.0746015	0.32700
keratinocyte 333	1.0698042	0.32780
fetal stomach 243	1.0852563	0.33022
fetal muscle 148	1.0872527	0.33032
fetal testes 254	1.0813497	0.33650
fetal kidney 101	1.0816625	0.33660
fetal muscle 156	1.0792266	0.33674
fetal renal cortex 202	1.0781178	0.33980
placenta 200	1.0794988	0.34110
ovary 326	1.0892668	0.34244
fetal kidney 97	1.0752336	0.34662
placenta 196	1.0816726	0.34752
fetal renal pelvis 217	1.0739035	0.35074
fetal renal pelvis 216	1.0739041	0.35204
fetal renal pelvis 228	1.0752239	0.35292
fetal adrenal gland 23	1.0682732	0.35348
fetal adrenal gland 27	1.0697688	0.36188
fetal muscle 151	1.0670976	0.36714
fetal adrenal gland 26	1.0629698	0.37076
fetal muscle 155	1.0633225	0.37270
fetal renal pelvis 226	1.0604437	0.37302
fetal stomach 253	1.0635861	0.37346
fetal muscle 169	1.0585467	0.37560
fetal renal cortex 214	1.0583245	0.37610
fetal renal pelvis 227	1.0569056	0.37630
fetal renal pelvis 221	1.0554250	0.38214
fetal kidney 88	1.0540800	0.38270
fetal kidney 105	1.0528477	0.38602
fetal stomach 250	1.0563035	0.38742
fetal muscle 153	1.0532607	0.38896
fetal brain 36	1.0450528	0.38920
fetal muscle 191	1.0510558	0.39000
fetal renal pelvis 233	1.0521109	0.39132
fetal kidney 81	1.0464587	0.39406
fetal stomach 240	1.0504592	0.39682
fetal kidney 95	1.0448890	0.39782
fetal renal cortex 215	1.0440167	0.39866
fetal renal cortex 203	1.0452302	0.39940
fetal spinal cord 237	1.0416314	0.40358
fetal muscle 149	1.0422691	0.40490
fetal stomach 241	1.0425429	0.40616
fetal brain 29	1.0307152	0.40736
muscle 341	1.0440398	0.40966
fetal kidney 98	1.0380196	0.41224
fetal kidney 82	1.0410686	0.41278
fetal kidney 99	1.0370612	0.41356
fetal muscle 174	1.0366739	0.41420
fetal renal pelvis 230	1.0331654	0.41822
fetal muscle 160	1.0319004	0.42352
fetal stomach 249	1.0324447	0.42426
fetal renal cortex 204	1.0312291	0.42492
fetal stomach 248	1.0310786	0.42510
fetal adrenal gland 25	1.0331853	0.42622
fetal muscle 171	1.0286377	0.42886
fetal kidney 84	1.0283300	0.42910
fetal stomach 247	1.0284011	0.42922
fetal renal cortex 212	1.0264138	0.43196
fetal renal cortex 206	1.0262074	0.43330
fetal renal pelvis 231	1.0244228	0.43376
fetal spinal cord 235	1.0200544	0.43956
fetal thymus 256	1.0223114	0.44162
fetal heart 49	1.0204263	0.44318
fetal muscle 145	1.0171497	0.44814
fetal muscle 173	1.0176475	0.44954
fetal kidney 93	1.0138263	0.45530
keratinocyte 335	1.0158170	0.45546
fetal renal pelvis 224	1.0130306	0.45678
fetal kidney 96	1.0124260	0.45816
fetal brain 38	1.0101314	0.45822
fetal renal pelvis 232	1.0107684	0.46056
fetal muscle 163	1.0094312	0.46238
fetal adrenal gland 24	1.0105665	0.46342
placenta 197	1.0128040	0.46406
fetal renal cortex 205	1.0052828	0.47062
placenta 198	1.0078256	0.47380
fetal renal pelvis 222	1.0034954	0.47422
stomach 284	1.0053335	0.47442
stomach 283	1.0032196	0.47458
breast 2	1.0089937	0.47490
fetal heart 51	1.0031325	0.47522
fetal muscle 193	1.0038380	0.47540
placenta 199	1.0044475	0.47700
fetal stomach 245	0.9981632	0.47784
fetal heart 52	1.0007731	0.47874
fetal brain 39	1.0013978	0.48244
fetal heart 43	0.9962492	0.48752
fetal kidney 107	0.9966475	0.48756
fetal brain 41	0.9980157	0.48926
fetal renal pelvis 223	0.9910900	0.49240
fetal brain 37	0.9952281	0.49590
fetal brain 35	0.9971038	0.49602
keratinocyte 334	0.9995199	0.49636
fetal kidney 102	0.9869382	0.49818
fetal renal cortex 208	0.9893620	0.49952
fetal heart 47	0.9840160	0.50300
fetal muscle 159	0.9872943	0.50416
fetal kidney 89	0.9873820	0.50634
CD14 4	0.9788995	0.51164
hematopoietic stem cell 317	0.9869495	0.51444
fetal kidney 104	0.9798132	0.51666
CD19 5	0.9700671	0.51928
fetal renal pelvis 225	0.9762714	0.52042
fetal renal cortex 209	0.9762909	0.52092
fetal renal cortex 210	0.9761516	0.52408
fetal kidney 87	0.9756424	0.52792
fetal heart 44	0.9612959	0.53122
fetal muscle 168	0.9742960	0.53204
Mobilized CD56 324	0.9593137	0.53652
fetal kidney 83	0.9624778	0.53702
fetal thymus 263	0.9713414	0.53768
CD19 6	0.9490298	0.54394
fetal brain 34	0.9747410	0.54596
fetal renal pelvis 229	0.9626414	0.54976
breast 1	0.9718330	0.55962
fetal spinal cord 239	0.9607744	0.56256
fetal stomach 242	0.9329560	0.56428
ES derived NP 292	0.9469761	0.56582
fetal large intestine 61	0.9415104	0.56676
CD14 3	0.9361480	0.56676
fetal heart 42	0.9519391	0.57242
fetal ovary 195	0.9344810	0.57386
CD3 10	0.9301346	0.57450
fetal heart 48	0.9502556	0.57488
fetal kidney 94	0.9427745	0.58036
fetal spinal cord 236	0.9489410	0.58436
fetal kidney 90	0.9374900	0.59282
fetal thymus 260	0.9217863	0.60112
fetal heart 50	0.9347664	0.60352
fetal renal pelvis 218	0.9143596	0.60404
Mobilized CD4 322	0.9027108	0.60794
fetal heart 46	0.9274535	0.61412
fetal brain 32	0.9409367	0.61714
CD4 13	0.8915905	0.62372
hematopoietic stem cell 306	0.9271735	0.62394
hematopoietic stem cell 319	0.9177399	0.62472
fetal renal cortex 211	0.9172326	0.62532
hematopoietic stem cell 307	0.9229728	0.62722
ES derived NP 291	0.9037724	0.63628
hematopoietic stem cell 313	0.9211131	0.63732
ES cells 296	0.9011286	0.64026
fetal thymus 258	0.8983219	0.64078
CD19 7	0.8723856	0.64252
fetal kidney 86	0.8913479	0.64772
fetal heart 45	0.9108407	0.65486
hematopoietic stem cell 311	0.9090865	0.65570
hematopoietic stem cell 318	0.9022849	0.65788
Mobilized CD3 320	0.8574675	0.65892
hematopoietic stem cell 310	0.9178536	0.66178
fetal thymus 264	0.8852123	0.66454
CD4 14	0.8743665	0.66760
fetal spinal cord 238	0.9025857	0.67160
fetal brain 33	0.9089814	0.67526
ES cells 293	0.8831015	0.68204
fetal renal pelvis 219	0.8673510	0.68462
ES cells 295	0.8724474	0.68840
fetal thymus 257	0.8543356	0.69174
iPS 303	0.8917029	0.69372
iPS 302	0.8872943	0.70100
hematopoietic stem cell 305	0.8812265	0.70270
hematopoietic stem cell 308	0.8865966	0.70716
fetal thymus 265	0.8529553	0.70974
hematopoietic stem cell 315	0.8647036	0.72468
CD3 11	0.8339751	0.72522
hematopoietic stem cell 312	0.8677869	0.73590
fetal thymus 266	0.8369532	0.74188
hematopoietic stem cell 309	0.8489416	0.75264
fetal thymus 259	0.8068372	0.76134
fetal thymus 261	0.8113334	0.76354
fetal small intestine 75	0.7637793	0.76448
CD4 16	0.7758792	0.76726
CD56 18	0.7740455	0.77928
hematopoietic stem cell 314	0.8439444	0.77978
hematopoietic stem cell 316	0.8279901	0.77980
Mobilized CD8 325	0.7648801	0.78648
fetal thymus 262	0.7677434	0.78776
CD8 20	0.7818397	0.78936
Mobilized CD4 323	0.7595314	0.79276
iPS 304	0.8300631	0.80810
CD8 21	0.7310617	0.82220
CD3 12	0.7305637	0.82596
Mobilized CD3 321	0.7194227	0.82968
ES cells 294	0.7768027	0.83346
CD4 15	0.6862046	0.83802
CD8 22	0.6988096	0.85784
CD56 17	0.7267222	0.86314
CD3 9	0.6885776	0.86752
CD8 19	0.6389134	0.89550

Kidney function−related traits

DHS sample	fold enrichment	p value
fetal skin 234	1.5438157	0.00594
fetal kidney 106	1.5469023	0.00664
fetal kidney 103	1.5927456	0.00696
fetal renal pelvis 226	1.5149634	0.00756
fetal kidney 84	1.5558704	0.00764
fetal renal pelvis 231	1.5031450	0.00836
fetal kidney 85	1.4799775	0.00952
fetal lung 141	1.4286699	0.00978
fetal kidney 99	1.5027922	0.00998
fetal renal cortex 202	1.4894256	0.01010
fetal renal pelvis 220	1.5347044	0.01084
stomach 284	1.5734342	0.01106
small intestine 342	1.5443835	0.01138
fetal kidney 107	1.4852735	0.01154
fetal kidney 89	1.4976412	0.01168
fetal renal pelvis 233	1.5034067	0.01242
fetal renal pelvis 227	1.4969872	0.01308
fetal lung 111	1.4149914	0.01322
fetal kidney 92	1.4598678	0.01374
fetal lung 118	1.3722874	0.01378
fetal lung 114	1.3722273	0.01482
fetal renal pelvis 217	1.4868699	0.01502
fetal kidney 105	1.4432817	0.01528
fetal kidney 95	1.4827623	0.01534
fetal large intestine 67	1.4756650	0.01582
fetal renal pelvis 228	1.5073218	0.01666
fetal renal pelvis 221	1.4301835	0.01720
fetal kidney 102	1.4853204	0.01754
fetal renal cortex 208	1.4588267	0.01852
fetal kidney 90	1.4366959	0.01950
fetal kidney 98	1.4523968	0.02006
fetal kidney 100	1.4470362	0.02052
fetal kidney 82	1.4821601	0.02098
fetal renal cortex 207	1.4455932	0.02110
fetal renal cortex 211	1.4615611	0.02142
fetal renal cortex 214	1.4461363	0.02148
fetal kidney 94	1.4604570	0.02192
fetal kidney 96	1.4082816	0.02224
fetal kidney 91	1.4155153	0.02244
fetal lung 133	1.3489548	0.02244
fetal renal pelvis 216	1.4281991	0.02376
fetal kidney 101	1.4106613	0.02398
fetal small intestine 80	1.4182969	0.02464
fetal kidney 97	1.4170081	0.02466
fetal renal pelvis 225	1.4256621	0.02504
fetal renal pelvis 230	1.4558770	0.02606
fetal renal cortex 215	1.3889390	0.02666
fetal renal pelvis 229	1.4018257	0.02746
fetal renal pelvis 232	1.4353056	0.02866
fetal kidney 104	1.4148377	0.02868
fetal lung 140	1.3470925	0.03130
fetal large intestine 57	1.3818212	0.03152
fetal small intestine 70	1.3624630	0.03188
fetal lung 132	1.3455157	0.03188
fetal renal cortex 205	1.3930469	0.03200
fetal lung 129	1.3349209	0.03246
fetal renal cortex 203	1.3921931	0.03256
fetal kidney 93	1.4066910	0.03294
fetal lung 121	1.3136232	0.03336
fetal lung 117	1.3339403	0.03346
fetal large intestine 60	1.4121661	0.03384
fetal renal cortex 204	1.3558340	0.03432
fetal lung 125	1.3381888	0.03566
fetal renal cortex 206	1.3650265	0.03658
fetal kidney 83	1.4429743	0.03662
fetal lung 130	1.3225300	0.03670
fetal renal cortex 212	1.3605506	0.03682
fetal large intestine 58	1.3662528	0.03686
fetal renal pelvis 222	1.3863429	0.03758
fetal renal cortex 213	1.3606083	0.03868
fetal renal pelvis 223	1.3964952	0.03886
fetal kidney 88	1.3559441	0.03908
CD20 8	1.3756092	0.03946
fetal kidney 87	1.3694493	0.04062
fetal stomach 246	1.4365401	0.04078
fetal lung 119	1.3043456	0.04092
fetal thymus 264	1.4270213	0.04098
fetal small intestine 74	1.3873012	0.04162
fetal thymus 258	1.4370185	0.04174
fetal renal cortex 209	1.3661934	0.04202
fetal small intestine 77	1.3865775	0.04256
fetal lung 120	1.3139148	0.04266
fetal large intestine 56	1.3759244	0.04284
fetal renal pelvis 224	1.3380317	0.04538
fetal lung 128	1.2699132	0.04624
fetal renal pelvis 219	1.4325545	0.04892
fetal small intestine 78	1.3253009	0.04948
fetal thymus 265	1.4059268	0.04978
fetal lung 138	1.3033215	0.05024
fetal thymus 259	1.4359429	0.05042
fetal kidney 86	1.4051480	0.05246
fetal lung 139	1.3083340	0.05282
fetal thymus 256	1.3806686	0.05326
ES derived NP 292	1.3710367	0.05332
fetal large intestine 55	1.3372577	0.05460
fetal renal cortex 210	1.3487477	0.05556
fetal lung 134	1.2954252	0.05592
stomach 283	1.3871331	0.05764
fetal thymus 257	1.4190554	0.05846
fetal large intestine 64	1.3326935	0.06030
fetal lung 113	1.2587821	0.06084
fetal renal pelvis 218	1.3975390	0.06210
fetal thymus 266	1.3841079	0.06300
fetal lung 131	1.2435382	0.06334
fetal stomach 248	1.3610777	0.06356
fetal lung 110	1.2812405	0.06562
fetal lung 142	1.2448908	0.06770
ES derived NP 291	1.3558539	0.06772
fetal stomach 240	1.3789458	0.06890
hematopoietic stem cell 310	1.2776513	0.07172
fetal lung 123	1.2674593	0.07326
fetal lung 115	1.2513460	0.07412
fetal small intestine 71	1.3118819	0.07646
fetal lung 137	1.2475182	0.07790
hematopoietic stem cell 318	1.3300878	0.07792
fetal lung 112	1.2569977	0.07812
fetal lung 116	1.2276926	0.07970
pancreas 328	1.3290973	0.08040
fetal lung 109	1.2151490	0.08120
fetal small intestine 73	1.3057651	0.08162
fetal lung 135	1.2540821	0.08224
fetal small intestine 68	1.2859150	0.08348
fetal small intestine 79	1.3228610	0.08394
fetal lung 136	1.2494895	0.08432
hematopoietic stem cell 311	1.2898131	0.08520
CD3 9	1.3933649	0.08580
fetal stomach 252	1.3124529	0.08690
fetal large intestine 54	1.3025478	0.08776
fetal muscle 151	1.2711331	0.08902
fetal stomach 241	1.3322667	0.08934
fetal lung 126	1.2197011	0.08978
fetal stomach 243	1.2816072	0.09128
fetal large intestine 65	1.2930487	0.09164
fetal large intestine 66	1.2976781	0.09168
fetal thymus 262	1.3947048	0.09246
hematopoietic stem cell 306	1.2814868	0.09380
fetal stomach 250	1.3190074	0.09386
fetal stomach 244	1.3042160	0.09394
fetal large intestine 59	1.3030256	0.09410
fetal thymus 261	1.3263196	0.09422
fetal stomach 253	1.3171951	0.09640
fetal large intestine 62	1.3011130	0.09648
Mobilized CD4 323	1.3782814	0.09914
hematopoietic stem cell 313	1.2587186	0.09918
fetal stomach 251	1.2831447	0.09990
fetal lung 122	1.2418730	0.09990
fetal small intestine 69	1.2955212	0.10120
CD8 21	1.3844547	0.10134
fetal thymus 260	1.3142070	0.10178
fetal muscle 194	1.2151375	0.10470
hematopoietic stem cell 308	1.2599770	0.10534
hematopoietic stem cell 307	1.2589705	0.10686
CD3 12	1.3662465	0.10840
CD8 19	1.3913543	0.10972
fetal lung 124	1.2161488	0.10992
fetal muscle 161	1.2317700	0.11054
fetal spinal cord 238	1.2199251	0.11144
hematopoietic stem cell 317	1.2253056	0.11190
fetal lung 127	1.2196423	0.11362
pancreas 327	1.2890880	0.11366
fetal spinal cord 239	1.2193369	0.11456
fetal muscle 155	1.2394164	0.11528
hematopoietic stem cell 315	1.2611881	0.11626
fetal large intestine 63	1.2630841	0.11730
fetal small intestine 72	1.2525178	0.11836
fetal muscle 149	1.2374922	0.11842
CD19 6	1.3373020	0.12016
fetal muscle 167	1.2085820	0.12156
fetal muscle 152	1.2108942	0.12476
CD4 13	1.3324850	0.12616
fetal muscle 179	1.2138887	0.12644
fetal small intestine 76	1.2563631	0.12672
fetal large intestine 61	1.3082807	0.12746
hematopoietic stem cell 319	1.2536312	0.12756
Mobilized CD4 322	1.3403302	0.12758
fetal spinal cord 236	1.2217821	0.12844
hematopoietic stem cell 314	1.2278110	0.12874
fetal large intestine 53	1.2669864	0.12912
fetal muscle 166	1.2086153	0.12948
hematopoietic stem cell 316	1.2530029	0.12984
fetal stomach 247	1.2947225	0.13012
fetal muscle 168	1.2162047	0.13054
CD56 18	1.3319766	0.13140
fetal muscle 143	1.1946068	0.13162
fetal muscle 160	1.2091160	0.13358
fetal thymus 263	1.2128765	0.13432
fetal heart 47	1.2321501	0.13670
fetal stomach 249	1.2597087	0.13694
fetal muscle 181	1.2019759	0.13788
CD8 22	1.3101136	0.13822
CD4 14	1.2803553	0.13882
fetal muscle 180	1.2150785	0.13888
melanocyte 340	1.1821800	0.13914
Mobilized CD56 324	1.2843857	0.13994
ES cells 295	1.2416481	0.14508
ES cells 294	1.2547070	0.14558
hematopoietic stem cell 309	1.2152937	0.14682
CD4 16	1.3107644	0.14754
CD19 7	1.3036245	0.14788
CD4 15	1.3397830	0.14952
fetal lung 108	1.1912693	0.15238
fetal stomach 242	1.2708808	0.15282
CD3 10	1.2691780	0.15318
ES cells 293	1.2183546	0.15490
fetal muscle 176	1.1795864	0.15546
fetal muscle 190	1.2010566	0.15562
fetal small intestine 75	1.3261538	0.15572
ES cells 296	1.2408471	0.15596
fetal muscle 182	1.1850889	0.15602
CD19 5	1.2675883	0.15800
fetal muscle 150	1.1965577	0.15802
fetal muscle 153	1.1962129	0.16126
fetal kidney 81	1.1711590	0.16300
fetal muscle 183	1.1699633	0.16376
CD14 4	1.2316981	0.16566
fetal muscle 156	1.1773333	0.16568
CD3 11	1.2489960	0.16744
hematopoietic stem cell 312	1.1897396	0.16926
fetal adrenal gland 26	1.1961218	0.17262
fetal muscle 157	1.1609228	0.17274
fetal heart 49	1.1821515	0.17628
CD8 20	1.2347639	0.17706
fetal heart 45	1.1796602	0.17890
fetal muscle 175	1.1667552	0.17914
fetal muscle 192	1.1637833	0.17918
fetal muscle 185	1.1756949	0.18440
fetal muscle 187	1.1566829	0.18454
fetal muscle 159	1.1749708	0.18540
hematopoietic stem cell 305	1.1893304	0.18554
fetal brain 32	1.1347917	0.18858
fetal muscle 148	1.1635811	0.18920
fetal muscle 165	1.1531962	0.19094
fetal stomach 245	1.1896150	0.19284
fetal muscle 178	1.1661431	0.19300
Mobilized CD3 321	1.2543235	0.19404
fetal muscle 171	1.1506523	0.19772
fetal muscle 154	1.1682186	0.20002
Mobilized CD3 320	1.2401448	0.20014
melanocyte 339	1.1282144	0.20076
ES derived mesoderm 285	1.1341830	0.20478
fetal brain 38	1.1232967	0.20670
fetal muscle 146	1.1456268	0.20788
CD56 17	1.1934910	0.20860
fetal fibroblast 270	1.1348994	0.20900
fetal muscle 188	1.1407835	0.21256
fetal adrenal gland 27	1.1630824	0.21470
fetal muscle 170	1.1394581	0.21572
fetal muscle 145	1.1526145	0.21830
fetal ovary 195	1.1819641	0.22004
fetal muscle 147	1.1408424	0.22010
fetal muscle 164	1.1408722	0.22018
fetal muscle 158	1.1352550	0.22040
CD14 3	1.1875402	0.22192
iPS 303	1.1385778	0.22836
fetal brain 33	1.1211663	0.22892
fetal adrenal gland 24	1.1657880	0.22994
fetal muscle 186	1.1294402	0.23144
fetal muscle 173	1.1328177	0.23280
fetal muscle 191	1.1327766	0.23356
fetal fibroblast 269	1.1150932	0.23450
fetal muscle 177	1.1364991	0.23618
fetal spinal cord 235	1.1224379	0.23668
fetal heart 44	1.1507564	0.24052
fetal muscle 189	1.1252046	0.24054
fetal heart 51	1.1282130	0.24084
melanocyte 338	1.1048004	0.24176
fetal adrenal gland 25	1.1488179	0.24390
fetal adrenal gland 28	1.1245001	0.24424
iPS 304	1.1206697	0.24656
fetal muscle 144	1.1264983	0.24766
iPS 302	1.1257109	0.25036
melanocyte 337	1.0963511	0.25396
muscle 341	1.1439855	0.25590
Mobilized CD8 325	1.1743634	0.25644
fetal muscle 169	1.1164555	0.26020
fetal heart 42	1.1170870	0.26312
fetal muscle 184	1.1046007	0.26520
fetal muscle 172	1.0994332	0.27048
fetal brain 39	1.0824372	0.27424
fetal testes 254	1.1126998	0.28000
fetal spinal cord 237	1.0950094	0.28192
fibroblast 330	1.1008536	0.28226
fetal heart 46	1.1072370	0.28304
fetal brain 36	1.0836113	0.28552
fibroblast 329	1.0964244	0.28560
fetal muscle 174	1.0984273	0.29066
ES derived mesoderm 286	1.0800743	0.29220
fetal fibroblast 273	1.0813944	0.30088
fetal adrenal gland 23	1.0915587	0.30308
ES derived trophoblast 288	1.0870413	0.30312
placenta 201	1.0979837	0.31094
fibroblast 332	1.0809518	0.31386
fetal muscle 163	1.0853716	0.31474
ES derived MSC 290	1.0858177	0.31900
fetal muscle 162	1.0765740	0.32208
fetal brain 41	1.0614871	0.32648
fetal heart 52	1.0751244	0.32820
fetal heart 43	1.0753559	0.32998
fetal brain 34	1.0571179	0.33136
fetal brain 37	1.0598893	0.33184
fetal heart 50	1.0680644	0.34812
fetal muscle 193	1.0658662	0.35142
fibroblast 331	1.0631163	0.35266
fetal testes 255	1.0618796	0.35480
fetal fibroblast 274	1.0560928	0.35990
placenta 196	1.0584299	0.37540
fetal fibroblast 272	1.0450923	0.38310
heart 297	1.0531165	0.38322
fetal brain 40	1.0390024	0.38346
fetal fibroblast 280	1.0425615	0.39012
fetal heart 48	1.0417554	0.40010
ovary 326	1.0461889	0.40412
fetal brain 35	1.0275430	0.40660
fetal brain 29	1.0257145	0.41040
ES derived MSC 289	1.0361332	0.41176
fetal fibroblast 282	1.0300105	0.41754
fetal fibroblast 271	1.0297558	0.41986
ES derived trophoblast 287	1.0330102	0.42088
breast 2	1.0208988	0.44048
fetal fibroblast 281	1.0209344	0.44172
fetal fibroblast 275	1.0190960	0.44558
fetal fibroblast 268	1.0154620	0.45464
fetal fibroblast 277	1.0125190	0.46180
fetal lung 300	1.0013081	0.49126
fetal fibroblast 278	0.9991635	0.49324
fetal fibroblast 267	0.9984538	0.49452
placenta 197	0.9889735	0.50374
placenta 199	0.9890722	0.50974
breast 1	0.9909710	0.51554
fetal lung 301	0.9872187	0.51860
fetal fibroblast 276	0.9876544	0.51994
fetal lung 299	0.9849145	0.52304
fetal brain 31	0.9873547	0.53090
keratinocyte 335	0.9798640	0.54866
keratinocyte 333	0.9783000	0.55382
keratinocyte 336	0.9759176	0.56108
fetal lung 298	0.9674770	0.56294
placenta 200	0.9530322	0.59348
fetal fibroblast 279	0.9542824	0.60302
fetal brain 30	0.9648380	0.60662
keratinocyte 334	0.9485067	0.63202
placenta 198	0.9118013	0.67290

Kidney function

DHS sample	fold enrichment	p value
fetal large intestine 67	1.4161877	0.00650
fetal small intestine 74	1.3714076	0.01094
fetal small intestine 80	1.3636277	0.01130
CD20 8	1.3241792	0.01742
fetal small intestine 78	1.3034263	0.01750
fetal large intestine 58	1.3108352	0.01798
fetal large intestine 64	1.3177352	0.02128
fetal large intestine 57	1.2982190	0.02274
fetal small intestine 68	1.3012207	0.02296
fetal large intestine 55	1.2945574	0.02720
fetal large intestine 56	1.3045934	0.02794
fetal small intestine 70	1.2487562	0.04020
fetal small intestine 77	1.2755963	0.04742
fetal large intestine 63	1.2675037	0.05240
fetal small intestine 79	1.2636703	0.05762
fetal large intestine 54	1.2442755	0.06376
small intestine 342	1.2460905	0.06378
fetal large intestine 60	1.2437466	0.06946
pancreas 328	1.2396836	0.07834
fetal large intestine 62	1.2266881	0.09196
fetal small intestine 72	1.2039742	0.09212
fetal small intestine 69	1.2147933	0.10138
fetal small intestine 73	1.1939921	0.10278
fetal small intestine 71	1.1958818	0.10474
fetal stomach 244	1.1955421	0.10572
fetal heart 47	1.1994795	0.11496
stomach 283	1.1951573	0.12838
fetal large intestine 65	1.1788018	0.13264
fetal skin 234	1.1626014	0.13520
fetal large intestine 59	1.1767907	0.13722
stomach 284	1.1803049	0.14748
fetal lung 141	1.1318723	0.14776
fetal large intestine 66	1.1591955	0.16048
fetal small intestine 76	1.1604419	0.16234
pancreas 327	1.1692606	0.16434
fetal lung 117	1.1203287	0.16812
fetal heart 46	1.1452502	0.16906
fetal stomach 243	1.1293455	0.19212
fetal kidney 106	1.1205572	0.19924
fetal renal cortex 207	1.1189672	0.20198
ES derived MSC 290	1.1186741	0.20300
fetal kidney 91	1.1095396	0.20476
fetal lung 111	1.1089132	0.20536
hematopoietic stem cell 314	1.1049586	0.21350
fetal lung 125	1.1038784	0.21650
fetal renal pelvis 220	1.1144315	0.22050
fetal lung 129	1.1002326	0.22190
fetal heart 44	1.1261307	0.22390
ES derived MSC 289	1.1071419	0.22652
fetal stomach 248	1.1162955	0.22992
fetal kidney 103	1.1051956	0.23678
fetal stomach 241	1.1155827	0.23816
fetal lung 298	1.0973482	0.24210
fetal heart 45	1.0998688	0.24728
fetal kidney 85	1.0850067	0.25034
fetal stomach 252	1.0979772	0.25796
fetal lung 120	1.0815195	0.26202
fetal adrenal gland 25	1.0958028	0.26380
fetal lung 133	1.0781662	0.26656
fetal kidney 99	1.0871832	0.26756
fetal lung 140	1.0778266	0.27038
fetal lung 301	1.0790807	0.27598
fetal renal cortex 213	1.0790446	0.27814
ES derived mesoderm 285	1.0692246	0.28336
fetal lung 127	1.0728372	0.28592
fetal stomach 251	1.0843521	0.28752
fetal stomach 247	1.0959409	0.28756
CD14 3	1.0965406	0.28926
fetal lung 119	1.0660686	0.29436
fetal lung 121	1.0631018	0.30030
fetal lung 134	1.0683041	0.30084
fetal renal pelvis 231	1.0689983	0.30270
fetal stomach 242	1.0908944	0.30346
fetal adrenal gland 28	1.0663777	0.30362
fetal large intestine 53	1.0815853	0.30418
fetal kidney 92	1.0686829	0.30554
hematopoietic stem cell 316	1.0742279	0.30668
fetal renal pelvis 228	1.0735535	0.30886
fibroblast 330	1.0624375	0.30906
fetal kidney 95	1.0669973	0.31066
fetal heart 49	1.0698423	0.31078
fibroblast 329	1.0606222	0.31150
fetal lung 300	1.0619064	0.31618
fetal renal cortex 202	1.0640669	0.31646
hematopoietic stem cell 315	1.0674538	0.31756
fetal lung 128	1.0521203	0.32108
fetal stomach 246	1.0719925	0.32186
fetal lung 118	1.0531801	0.32320
ES derived NP 291	1.0699877	0.32692
fetal renal pelvis 226	1.0588694	0.32898
fetal stomach 250	1.0679501	0.33094
hematopoietic stem cell 311	1.0575114	0.33308
fetal kidney 84	1.0604854	0.33434
fetal renal cortex 214	1.0564627	0.33834
fetal kidney 83	1.0620049	0.33934
fetal heart 42	1.0551946	0.34038
fetal lung 108	1.0538698	0.34292
hematopoietic stem cell 308	1.0503397	0.34790
fetal lung 123	1.0456298	0.35452
fetal heart 51	1.0497563	0.35470
fetal renal cortex 211	1.0495186	0.35974
fetal kidney 87	1.0466235	0.36196
hematopoietic stem cell 305	1.0467978	0.36756
fetal kidney 100	1.0444787	0.36896
fetal lung 299	1.0441066	0.36900
fetal lung 138	1.0405054	0.37120
fetal heart 48	1.0431896	0.37176
fetal stomach 240	1.0478352	0.37214
fetal adrenal gland 24	1.0484338	0.37500
ES cells 296	1.0478864	0.37678
fetal lung 130	1.0365360	0.37942
fetal renal pelvis 217	1.0404709	0.38012
CD56 18	1.0524696	0.38366
fetal testes 255	1.0375161	0.38608
fetal lung 112	1.0338746	0.38716
ES cells 295	1.0421511	0.38732
heart 297	1.0405841	0.38732
fetal renal pelvis 230	1.0388996	0.38930
fetal renal pelvis 232	1.0365850	0.38996
hematopoietic stem cell 310	1.0317058	0.39408
CD14 4	1.0415281	0.39616
fetal lung 131	1.0290174	0.39682
fetal lung 122	1.0324376	0.39728
fetal lung 110	1.0313282	0.39946
fetal lung 139	1.0308589	0.39990
hematopoietic stem cell 309	1.0330083	0.40022
hematopoietic stem cell 318	1.0326165	0.40062
hematopoietic stem cell 312	1.0309696	0.40068
hematopoietic stem cell 319	1.0345755	0.40076
hematopoietic stem cell 307	1.0315080	0.40076
fetal lung 114	1.0274403	0.40312
fetal stomach 253	1.0328515	0.40636
fibroblast 331	1.0277805	0.40672
fetal adrenal gland 26	1.0314058	0.40786
fetal kidney 98	1.0290351	0.40888
fetal lung 136	1.0260515	0.41068
fetal renal pelvis 221	1.0281792	0.41080
fetal lung 142	1.0232771	0.41530
fetal lung 135	1.0257289	0.41658
fetal kidney 107	1.0237932	0.41940
fetal renal pelvis 216	1.0235041	0.42076
hematopoietic stem cell 306	1.0244833	0.42080
hematopoietic stem cell 313	1.0228226	0.42286
fetal lung 132	1.0226188	0.42354
fetal renal cortex 205	1.0222987	0.42596
fetal stomach 245	1.0238451	0.42710
fetal renal pelvis 233	1.0219898	0.42858
fetal heart 50	1.0224802	0.42868
fibroblast 332	1.0173118	0.43706
fetal renal pelvis 219	1.0193656	0.43844
fetal kidney 104	1.0176853	0.43924
fetal kidney 94	1.0152062	0.44478
muscle 341	1.0154503	0.44816
fetal renal cortex 206	1.0125672	0.45512
fetal kidney 101	1.0107139	0.45648
hematopoietic stem cell 317	1.0108324	0.45684
fetal kidney 89	1.0113862	0.45712
fetal renal pelvis 229	1.0099029	0.45886
fetal kidney 97	1.0091259	0.46026
fetal lung 116	1.0102519	0.46100
CD4 13	1.0112846	0.46122
fetal adrenal gland 27	1.0101667	0.46236
fetal renal cortex 212	1.0091177	0.46388
Mobilized CD4 323	1.0094647	0.46454
fetal heart 43	1.0065541	0.47004
fetal kidney 105	1.0041910	0.47088
fetal kidney 82	1.0040211	0.47106
fetal stomach 249	1.0041307	0.47224
fetal renal cortex 215	1.0030437	0.47758
ES derived NP 292	1.0029565	0.47926
fetal kidney 96	1.0013389	0.47980
fetal renal pelvis 227	1.0006792	0.48292
fetal lung 124	1.0019328	0.48356
fetal kidney 93	0.9975182	0.48996
fetal renal pelvis 225	0.9971700	0.49160
fetal heart 52	0.9989832	0.49276
fetal thymus 257	0.9907119	0.49434
fetal adrenal gland 23	0.9980218	0.49492
fetal renal pelvis 222	0.9949484	0.49710
ES derived mesoderm 286	0.9966497	0.50248
melanocyte 340	0.9955723	0.51276
ES cells 293	0.9862752	0.51518
CD8 19	0.9802444	0.51576
fetal lung 115	0.9908681	0.52108
fetal lung 137	0.9903773	0.52160
ES cells 294	0.9807152	0.52806
fetal lung 109	0.9882592	0.53250
iPS 304	0.9843369	0.53344
fetal kidney 86	0.9771316	0.53424
fetal renal cortex 204	0.9835219	0.53474
fetal renal cortex 203	0.9807696	0.53694
CD19 7	0.9704263	0.53942
fetal renal pelvis 223	0.9781027	0.54070
fetal kidney 102	0.9765825	0.54202
fetal testes 254	0.9793302	0.54440
fetal ovary 195	0.9637657	0.56218
fetal kidney 90	0.9681952	0.56490
fetal lung 113	0.9762543	0.56680
fetal kidney 88	0.9710416	0.56694
fetal muscle 194	0.9752367	0.56926
fetal thymus 259	0.9555329	0.57574
fetal lung 126	0.9733126	0.57962
Mobilized CD56 324	0.9597220	0.58186
fetal large intestine 61	0.9534762	0.58300
fetal renal pelvis 218	0.9448329	0.59860
CD56 17	0.9503426	0.60380
CD8 21	0.9394567	0.60396
Mobilized CD8 325	0.9360211	0.60862
fetal renal cortex 208	0.9509787	0.60906
placenta 196	0.9558010	0.60908
fetal brain 32	0.9602438	0.61918
placenta 201	0.9499893	0.62044
CD19 6	0.9310290	0.62218
CD19 5	0.9320820	0.62906
fetal spinal cord 236	0.9475579	0.63016
fetal renal pelvis 224	0.9486290	0.63306
fetal muscle 155	0.9463020	0.63552
fetal muscle 174	0.9483506	0.63640
iPS 303	0.9459469	0.64232
Mobilized CD3 321	0.9143942	0.64608
CD4 15	0.9003881	0.64674
fetal renal cortex 210	0.9336265	0.64894
Mobilized CD4 322	0.9124613	0.65660
fetal muscle 149	0.9366276	0.65914
fetal fibroblast 269	0.9500509	0.65998
fetal muscle 185	0.9373779	0.66012
fetal brain 36	0.9464619	0.66456
fetal fibroblast 279	0.9471424	0.66552
fetal fibroblast 280	0.9454777	0.67166
CD4 14	0.9169434	0.67324
placenta 200	0.9370744	0.68004
placenta 197	0.9276061	0.68280
fetal renal cortex 209	0.9224169	0.68562
fetal muscle 154	0.9244582	0.68932
CD3 9	0.9018708	0.69070
iPS 302	0.9262006	0.69090
placenta 199	0.9279843	0.69544
fetal muscle 159	0.9220856	0.69600
fetal fibroblast 270	0.9329506	0.69754
fetal muscle 189	0.9244642	0.70144
fetal fibroblast 267	0.9302421	0.70234
fetal muscle 156	0.9245947	0.70452
fetal muscle 191	0.9192371	0.70484
fetal thymus 263	0.9205945	0.70516
CD4 16	0.8836722	0.70620
fetal muscle 148	0.9161434	0.71328
fetal muscle 186	0.9194792	0.71538
fetal muscle 164	0.9184613	0.71554
fetal muscle 178	0.9151629	0.71778
fetal fibroblast 278	0.9250418	0.72038
fetal muscle 182	0.9145015	0.72598
fetal thymus 262	0.8575386	0.72780
fetal thymus 260	0.8855483	0.73138
fetal thymus 264	0.8868231	0.73676
fetal small intestine 75	0.8375736	0.73938
fetal spinal cord 235	0.9070895	0.74402
fetal muscle 181	0.9035705	0.74510
fetal muscle 187	0.9079122	0.74634
fetal muscle 165	0.9107534	0.74824
breast 2	0.9170157	0.74846
fetal muscle 153	0.8989722	0.74956
CD3 12	0.8616572	0.75042
fetal thymus 265	0.8779268	0.75092
melanocyte 339	0.9205156	0.75274
fetal muscle 160	0.9004033	0.75508
fetal muscle 166	0.9046680	0.75514
Mobilized CD3 320	0.8581845	0.75646
fetal muscle 161	0.8995728	0.75700
fetal muscle 150	0.8977439	0.76160
fetal spinal cord 238	0.9026369	0.76190
fetal muscle 190	0.8868431	0.76864
fetal muscle 184	0.9014392	0.76878
fetal thymus 261	0.8635579	0.76958
fetal spinal cord 237	0.9013143	0.77032
fetal muscle 179	0.8937099	0.77054
fetal muscle 151	0.8870435	0.77306
fetal thymus 256	0.8700604	0.77480
fetal fibroblast 277	0.9022672	0.77756
fetal fibroblast 271	0.9052192	0.77950
breast 1	0.9069413	0.78072
fetal muscle 145	0.8749793	0.78092
ovary 326	0.8500768	0.78178
fetal brain 41	0.9046449	0.78346
fetal fibroblast 268	0.9033655	0.78592
fetal muscle 177	0.8783718	0.78928
fetal fibroblast 272	0.9010961	0.78944
fetal muscle 158	0.8911729	0.79228
fetal muscle 168	0.8829243	0.79296
fetal fibroblast 273	0.9011768	0.79298
fetal thymus 266	0.8578750	0.79394
fetal muscle 175	0.8865933	0.79490
fetal fibroblast 276	0.8985879	0.79900
keratinocyte 333	0.9061232	0.79950
fetal muscle 157	0.8890575	0.80020
fetal muscle 169	0.8797874	0.80266
fetal fibroblast 281	0.8970069	0.80278
fetal kidney 81	0.8871784	0.80318
fetal brain 33	0.8890982	0.80382
fetal muscle 147	0.8747720	0.80402
melanocyte 338	0.9052986	0.80664
CD3 10	0.8404768	0.80868
CD3 11	0.8461742	0.81018
fetal muscle 167	0.8780192	0.81044
fetal muscle 171	0.8839621	0.81226
fetal muscle 183	0.8806754	0.81348
fetal muscle 170	0.8798896	0.81412
CD8 22	0.8240047	0.81506
fetal brain 38	0.8856652	0.81586
fetal muscle 192	0.8785485	0.81790
fetal thymus 258	0.8417329	0.81792
placenta 198	0.8763352	0.82096
fetal muscle 188	0.8721170	0.82388
fetal fibroblast 274	0.8840965	0.82506
fetal muscle 173	0.8704204	0.82608
fetal spinal cord 239	0.8721330	0.82764
fetal muscle 180	0.8598689	0.83162
fetal muscle 172	0.8809632	0.83626
fetal fibroblast 275	0.8830342	0.83750
fetal brain 35	0.8937180	0.83880
CD8 20	0.8231589	0.83904
fetal fibroblast 282	0.8796870	0.84760
fetal muscle 152	0.8534069	0.85736
fetal muscle 176	0.8535156	0.85876
fetal muscle 146	0.8471063	0.86166
fetal muscle 144	0.8400731	0.86212
keratinocyte 334	0.8755315	0.86292
fetal muscle 162	0.8477965	0.87098
keratinocyte 336	0.8706711	0.87442
ES derived trophoblast 288	0.8457084	0.87534
fetal muscle 193	0.8366910	0.87746
keratinocyte 335	0.8666092	0.88198
fetal muscle 143	0.8405887	0.88974
fetal muscle 163	0.8305371	0.89024
fetal brain 39	0.8596408	0.89106
fetal brain 34	0.8592933	0.89808
fetal brain 37	0.8460570	0.90794
ES derived trophoblast 287	0.8026360	0.91568
melanocyte 337	0.8549270	0.91592
fetal brain 31	0.8482413	0.93678
fetal brain 40	0.8260180	0.94386
fetal brain 29	0.8309313	0.95504
fetal brain 30	0.8003052	0.98062

Testicular cancer

DHS sample	fold enrichment	p value
Mobilized CD3 320	1.6388319	0.02256
CD19 5	1.4825438	0.04018
CD3 9	1.5088918	0.04042
CD8 22	1.5100786	0.04044
CD8 20	1.4498185	0.04052
CD56 18	1.4939926	0.04450
Mobilized CD56 324	1.4462877	0.04524
iPS 304	1.3057499	0.04538
CD56 17	1.4065509	0.04694
CD19 6	1.4775666	0.04730
CD19 7	1.4940576	0.04968
CD3 11	1.4199636	0.05240
CD4 13	1.4695358	0.05274
CD3 12	1.4814023	0.05446
CD3 10	1.4291041	0.05494
ES cells 294	1.3645539	0.05660
Mobilized CD4 323	1.4608090	0.05734
CD8 21	1.4604375	0.06174
CD4 14	1.3885698	0.06202
CD8 19	1.4848405	0.06530
fetal thymus 260	1.4058359	0.06806
iPS 302	1.2803754	0.07208
ES derived mesoderm 286	1.2243607	0.07420
fetal large intestine 61	1.3935657	0.07714
Mobilized CD4 322	1.4132790	0.08082
hematopoietic stem cell 308	1.2760373	0.08090
Mobilized CD3 321	1.4065289	0.08640
CD4 16	1.3869512	0.09104
ES cells 295	1.3057163	0.09304
CD4 15	1.4521803	0.09358
fetal thymus 261	1.3396542	0.09930
Mobilized CD8 325	1.3641369	0.10004
ES derived MSC 290	1.2570283	0.10250
hematopoietic stem cell 315	1.2596364	0.10558
iPS 303	1.2384201	0.10572
ES derived mesoderm 285	1.2118421	0.10894
ES derived NP 291	1.2844585	0.11264
ES cells 293	1.2480815	0.12108
hematopoietic stem cell 310	1.2039250	0.12404
small intestine 342	1.2412853	0.12476
hematopoietic stem cell 314	1.2150121	0.12480
ES derived MSC 289	1.2340940	0.12564
fetal thymus 264	1.2891184	0.12710
hematopoietic stem cell 312	1.2175949	0.12914
fibroblast 331	1.1915249	0.13114
fibroblast 332	1.1878499	0.13576
fetal thymus 258	1.2775092	0.13644
hematopoietic stem cell 311	1.2109993	0.13734
hematopoietic stem cell 319	1.2328728	0.13918
hematopoietic stem cell 306	1.2136131	0.13946
fetal lung 109	1.1731822	0.14480
fetal thymus 266	1.2603480	0.14856
ES derived trophoblast 287	1.2131931	0.14858
fetal lung 299	1.1961877	0.15204
hematopoietic stem cell 307	1.2023517	0.15318
muscle 341	1.2456135	0.15674
fetal thymus 257	1.2615948	0.15740
fetal stomach 248	1.2281146	0.15756
fetal fibroblast 275	1.1669667	0.16204
fibroblast 330	1.1759395	0.16226
ES cells 296	1.2160967	0.16588
fetal lung 121	1.1697599	0.16740
hematopoietic stem cell 313	1.1773368	0.16986
fibroblast 329	1.1661329	0.17058
hematopoietic stem cell 309	1.1867767	0.17200
fetal lung 128	1.1503147	0.17466
fetal thymus 256	1.2235148	0.17540
fetal lung 117	1.1631558	0.17972
hematopoietic stem cell 316	1.1908303	0.18082
ES derived NP 292	1.1943976	0.18152
fetal stomach 242	1.2355651	0.18196
hematopoietic stem cell 317	1.1524433	0.18784
fetal muscle 149	1.1823465	0.18908
fetal lung 298	1.1664555	0.18940
fetal lung 300	1.1636511	0.19054
fetal muscle 153	1.1836070	0.19260
hematopoietic stem cell 318	1.1753408	0.19382
placenta 200	1.1575179	0.19534
placenta 198	1.1581702	0.20140
breast 2	1.1319278	0.20738
fetal fibroblast 272	1.1378675	0.20968
CD14 3	1.1919010	0.21178
fetal lung 140	1.1427834	0.21180
fetal thymus 265	1.1948356	0.21184
fetal lung 120	1.1441512	0.21526
fetal stomach 249	1.1818356	0.21688
fetal stomach 240	1.1778933	0.21708
fetal muscle 143	1.1409227	0.21846
fetal fibroblast 277	1.1358966	0.22206
fetal small intestine 75	1.2379485	0.22300
breast 1	1.1222204	0.22562
keratinocyte 334	1.1109084	0.22564
fetal fibroblast 274	1.1276225	0.22936
fetal lung 301	1.1371239	0.23090
fetal stomach 253	1.1651769	0.23380
fetal muscle 162	1.1358335	0.23380
fetal thymus 262	1.2005532	0.23658
fetal muscle 151	1.1482581	0.23694
fetal muscle 144	1.1447369	0.23798
fetal lung 127	1.1286411	0.23884
fetal muscle 164	1.1393135	0.23924
fetal lung 133	1.1180263	0.24302
fetal lung 125	1.1263244	0.24518
fetal fibroblast 282	1.1129572	0.24758
fetal lung 112	1.1219649	0.24816
fetal muscle 159	1.1360861	0.24872
fetal muscle 166	1.1299611	0.25062
fetal muscle 190	1.1353812	0.25068
fetal fibroblast 276	1.1118866	0.25078
hematopoietic stem cell 305	1.1282687	0.25220
fetal kidney 98	1.1322305	0.25280
fetal lung 108	1.1255964	0.25286
fetal lung 138	1.1185226	0.25602
fetal fibroblast 278	1.1139132	0.25822
melanocyte 339	1.0978265	0.25970
keratinocyte 333	1.0919831	0.26152
fetal muscle 180	1.1236835	0.26390
fetal lung 142	1.1029961	0.26428
fetal kidney 105	1.1177304	0.26470
fetal fibroblast 273	1.1058211	0.26508
fetal muscle 154	1.1263439	0.26580
fetal muscle 161	1.1214140	0.26722
melanocyte 340	1.0969151	0.26942
fetal muscle 179	1.1162822	0.26962
fetal lung 111	1.1067388	0.26986
fetal muscle 175	1.1148356	0.27318
fetal lung 135	1.1090200	0.27326
melanocyte 338	1.0858224	0.27668
fetal muscle 185	1.1156750	0.27834
fetal large intestine 59	1.1224778	0.27940
fetal muscle 152	1.1076677	0.28026
fetal lung 136	1.1027381	0.28110
keratinocyte 336	1.0867618	0.28148
fetal muscle 156	1.1088628	0.28194
fetal stomach 247	1.1324437	0.28578
fetal lung 115	1.0956916	0.28604
fetal lung 129	1.1000537	0.28634
fetal muscle 170	1.1033972	0.28690
fetal lung 132	1.0971111	0.28840
fetal kidney 96	1.1025861	0.28864
fetal lung 122	1.1018903	0.28990
fetal muscle 155	1.1099495	0.29010
fetal fibroblast 271	1.0922522	0.29274
fetal stomach 246	1.1163982	0.29450
fetal muscle 189	1.1008668	0.29462
fetal fibroblast 268	1.0897712	0.29556
melanocyte 337	1.0777202	0.29688
fetal lung 139	1.0971766	0.29766
fetal stomach 250	1.1130041	0.29918
keratinocyte 335	1.0782261	0.29948
fetal renal cortex 203	1.1014245	0.29994
stomach 283	1.1115777	0.30004
fetal muscle 183	1.0922579	0.30254
fetal muscle 169	1.0962602	0.30314
CD14 4	1.1119921	0.30464
fetal lung 141	1.0817141	0.30626
fetal kidney 107	1.0948908	0.30778
fetal brain 36	1.0751754	0.30866
fetal fibroblast 281	1.0840390	0.30868
fetal thymus 259	1.1191785	0.30926
fetal muscle 171	1.0902829	0.30964
fetal lung 124	1.0869752	0.31022
fetal stomach 251	1.1011573	0.31026
fetal muscle 177	1.0963032	0.31050
stomach 284	1.1016260	0.31120
fetal lung 126	1.0805961	0.31130
fetal small intestine 70	1.0868634	0.31178
fetal muscle 150	1.0934256	0.31340
fetal fibroblast 279	1.0816330	0.31478
fetal muscle 181	1.0889459	0.31542
fetal lung 131	1.0760020	0.31672
fetal kidney 89	1.0872762	0.32108
fetal renal cortex 214	1.0833806	0.32236
fetal small intestine 72	1.0882795	0.32260
fetal lung 137	1.0759723	0.32358
fetal large intestine 53	1.0937522	0.32390
fetal lung 123	1.0791590	0.32414
fetal kidney 106	1.0803777	0.32466
fetal muscle 163	1.0850068	0.32630
fetal kidney 103	1.0786086	0.32642
fetal small intestine 73	1.0874289	0.32648
fetal muscle 194	1.0755298	0.32698
fetal kidney 92	1.0761513	0.32716
fetal large intestine 54	1.0868158	0.32744
fetal muscle 147	1.0838329	0.32786
fetal renal cortex 204	1.0773746	0.32896
fetal lung 130	1.0736689	0.33078
fetal small intestine 76	1.0879189	0.33112
placenta 199	1.0794004	0.33302
fetal renal cortex 209	1.0805274	0.33422
heart 297	1.0864088	0.33678
fetal kidney 102	1.0802329	0.33714
fetal small intestine 79	1.0841057	0.33874
fetal fibroblast 267	1.0691752	0.34172
fetal large intestine 63	1.0799090	0.34198
fetal muscle 167	1.0704393	0.34826
fetal stomach 241	1.0785772	0.34836
fetal stomach 243	1.0745398	0.34846
fetal lung 134	1.0678177	0.34928
fetal kidney 91	1.0602188	0.34998
fetal muscle 193	1.0689851	0.35456
fetal renal cortex 213	1.0596888	0.35538
fetal ovary 195	1.0741171	0.35616
fetal kidney 86	1.0733450	0.35680
fetal large intestine 65	1.0698408	0.35732
placenta 196	1.0684895	0.35738
fetal small intestine 71	1.0677601	0.35880
fetal muscle 186	1.0634304	0.35900
fetal muscle 174	1.0650094	0.35930
fetal muscle 158	1.0616439	0.35958
fetal kidney 85	1.0517647	0.36030
fetal testes 254	1.0631469	0.36164
fetal kidney 94	1.0630268	0.36240
fetal stomach 245	1.0666638	0.36300
fetal muscle 192	1.0594637	0.36374
fetal muscle 176	1.0589007	0.36552
fetal spinal cord 236	1.0535665	0.36786
fetal kidney 90	1.0596060	0.36824
fetal lung 110	1.0546035	0.37192
fetal skin 234	1.0565334	0.37340
fetal muscle 146	1.0560647	0.37382
fetal kidney 81	1.0547428	0.37384
fetal fibroblast 269	1.0504680	0.37390
fetal small intestine 78	1.0554157	0.37466
CD20 8	1.0573081	0.37520
fetal muscle 148	1.0570331	0.37538
fetal small intestine 69	1.0602318	0.37588
fetal muscle 191	1.0563614	0.37710
fetal kidney 83	1.0567234	0.37802
fetal spinal cord 235	1.0457877	0.37958
fetal kidney 84	1.0515914	0.38102
fetal renal pelvis 225	1.0510109	0.38260
fetal lung 118	1.0444585	0.38336
fetal lung 119	1.0455547	0.38532
fetal small intestine 74	1.0515971	0.38646
fetal stomach 252	1.0502988	0.38842
fetal large intestine 67	1.0521935	0.38856
fetal muscle 160	1.0494049	0.38876
fetal renal pelvis 222	1.0474688	0.38926
fetal muscle 178	1.0474719	0.39020
fetal renal cortex 207	1.0417973	0.39048
fetal renal cortex 210	1.0485202	0.39068
fetal kidney 97	1.0449126	0.39070
fetal renal pelvis 223	1.0474839	0.39352
fetal small intestine 68	1.0471990	0.39380
fetal renal cortex 208	1.0456415	0.39568
fetal fibroblast 270	1.0408703	0.39718
fetal heart 46	1.0435648	0.39884
fetal muscle 145	1.0439384	0.39940
fetal lung 116	1.0366107	0.40144
fetal renal pelvis 219	1.0454989	0.40206
fetal lung 114	1.0370915	0.40248
fetal kidney 88	1.0369897	0.40360
fetal renal pelvis 230	1.0373355	0.40450
fetal heart 44	1.0416296	0.40488
fetal renal pelvis 221	1.0345555	0.40606
fetal renal pelvis 226	1.0366868	0.40664
fetal kidney 93	1.0390417	0.40736
fetal renal cortex 202	1.0354088	0.40768
fetal renal pelvis 224	1.0329616	0.40946
fetal large intestine 66	1.0385919	0.41120
fetal renal pelvis 229	1.0356165	0.41350
fetal muscle 188	1.0334492	0.41484
fetal heart 42	1.0315626	0.41500
fetal lung 113	1.0303802	0.41544
fetal large intestine 55	1.0352068	0.41660
fetal muscle 184	1.0316588	0.41758
fetal renal cortex 212	1.0309101	0.41936
fetal muscle 182	1.0325332	0.42124
fetal renal pelvis 216	1.0297930	0.42128
fetal renal pelvis 218	1.0310893	0.42192
fetal large intestine 62	1.0334448	0.42246
fetal testes 255	1.0300822	0.42340
fetal muscle 157	1.0296686	0.42474
fetal renal pelvis 227	1.0256082	0.42584
fetal heart 48	1.0233928	0.42868
fetal small intestine 77	1.0292856	0.42896
fetal large intestine 58	1.0289246	0.42950
fetal fibroblast 280	1.0264608	0.42968
placenta 201	1.0279617	0.43136
ES derived trophoblast 288	1.0270876	0.43146
fetal renal pelvis 220	1.0205581	0.43274
fetal renal cortex 215	1.0215858	0.43278
fetal renal cortex 206	1.0224514	0.43606
placenta 197	1.0266254	0.43762
fetal stomach 244	1.0240114	0.43916
fetal muscle 172	1.0220078	0.43988
fetal heart 50	1.0189145	0.44160
fetal heart 51	1.0179782	0.44192
fetal kidney 104	1.0186763	0.44412
fetal spinal cord 239	1.0155587	0.44430
fetal heart 45	1.0182267	0.44450
fetal renal pelvis 217	1.0172686	0.44506
ovary 326	1.0174919	0.44546
fetal renal pelvis 228	1.0149249	0.44832
fetal muscle 165	1.0173569	0.45070
fetal renal cortex 205	1.0123995	0.45592
fetal kidney 87	1.0102131	0.45618
fetal renal pelvis 231	1.0095136	0.45814
fetal large intestine 56	1.0132321	0.45866
fetal thymus 263	1.0112856	0.46014
fetal small intestine 80	1.0146876	0.46114
fetal large intestine 64	1.0109838	0.46386
fetal large intestine 57	1.0112734	0.46494
fetal kidney 95	1.0023042	0.46580
fetal spinal cord 238	1.0040651	0.46750
fetal heart 47	1.0029424	0.46990
fetal kidney 99	0.9982015	0.47316
fetal muscle 173	1.0029594	0.47414
fetal large intestine 60	1.0048732	0.47744
fetal heart 49	0.9982413	0.47972
fetal muscle 168	1.0025329	0.47982
fetal brain 37	1.0013298	0.48044
fetal renal pelvis 233	0.9959585	0.48262
fetal kidney 101	0.9959679	0.48504
fetal spinal cord 237	0.9935778	0.49244
fetal kidney 100	0.9912132	0.49330
fetal adrenal gland 26	0.9962948	0.49574
fetal muscle 187	0.9912176	0.50284
fetal adrenal gland 25	0.9883290	0.50934
fetal kidney 82	0.9780064	0.51362
pancreas 327	0.9752398	0.51538
fetal renal pelvis 232	0.9777001	0.51634
fetal renal cortex 211	0.9762027	0.51716
fetal heart 43	0.9825761	0.51860
fetal brain 32	0.9725002	0.54962
fetal heart 52	0.9624301	0.56214
fetal adrenal gland 23	0.9613570	0.57046
fetal brain 33	0.9548528	0.58560
fetal adrenal gland 27	0.9460592	0.59554
fetal adrenal gland 24	0.9294623	0.61218
pancreas 328	0.9205320	0.61820
fetal brain 39	0.9286828	0.66754
fetal adrenal gland 28	0.9177666	0.67428
fetal brain 31	0.9371727	0.67576
fetal brain 40	0.9303548	0.67584
fetal brain 38	0.9095352	0.68678
fetal brain 34	0.9197164	0.69242
fetal brain 41	0.9103137	0.69740
fetal brain 30	0.9262378	0.70506
fetal brain 35	0.9166617	0.70570
fetal brain 29	0.8929408	0.78332

Platelet count and volume

DHS sample	fold enrichment	p value
hematopoietic stem cell 310	1.4163227	0.01500
hematopoietic stem cell 314	1.3988187	0.02430
CD8 21	1.5789905	0.03200
hematopoietic stem cell 309	1.3857525	0.04044
fetal thymus 264	1.4319246	0.04268
fetal thymus 266	1.4294095	0.04704
fetal thymus 256	1.4143134	0.04712
Mobilized CD56 324	1.4612948	0.04846
fetal thymus 265	1.4154008	0.05126
fetal thymus 258	1.4161827	0.05420
CD3 12	1.4592183	0.06248
fetal brain 37	1.2766536	0.06686
fetal large intestine 61	1.4209233	0.06860
Mobilized CD4 322	1.4400526	0.07162
fetal thymus 261	1.3772189	0.07294
CD8 19	1.4647066	0.07308
fetal thymus 259	1.3895085	0.07334
hematopoietic stem cell 308	1.2977744	0.07464
CD56 18	1.4238501	0.07478
hematopoietic stem cell 306	1.3030738	0.07626
fetal kidney 85	1.2865441	0.07906
hematopoietic stem cell 317	1.2594580	0.07994
Mobilized CD4 323	1.3984125	0.08454
fetal fibroblast 272	1.2440361	0.08618
fetal thymus 260	1.3414294	0.09228
hematopoietic stem cell 311	1.2741187	0.09232
hematopoietic stem cell 316	1.2997504	0.09518
hematopoietic stem cell 307	1.2713975	0.09608
CD19 5	1.3409742	0.09702
CD3 11	1.3284534	0.09716
hematopoietic stem cell 305	1.2829186	0.10044
hematopoietic stem cell 315	1.2867140	0.10112
CD3 9	1.3591439	0.10188
hematopoietic stem cell 313	1.2503538	0.10278
Mobilized CD8 325	1.3759927	0.10310
fetal kidney 83	1.3111287	0.10460
Mobilized CD3 321	1.3765053	0.10660
fetal kidney 106	1.2694898	0.10792
hematopoietic stem cell 318	1.2734615	0.11080
fetal kidney 91	1.2555214	0.11392
CD4 13	1.3473077	0.11438
CD8 20	1.3018743	0.11514
CD3 10	1.3062977	0.11700
CD8 22	1.3265178	0.11736
fetal brain 31	1.1778621	0.12328
CD56 17	1.2760665	0.12342
melanocyte 338	1.1951867	0.12354
hematopoietic stem cell 312	1.2321471	0.12650
ovary 326	1.3143581	0.12808
CD19 6	1.3072912	0.13140
CD4 14	1.2704918	0.14066
fetal brain 30	1.1584087	0.14220
fetal small intestine 75	1.3444527	0.14230
fetal fibroblast 275	1.1842336	0.14236
fibroblast 331	1.1804708	0.15020
keratinocyte 333	1.1574313	0.15290
melanocyte 337	1.1640733	0.15384
fetal testes 255	1.2078554	0.15428
fetal fibroblast 271	1.1769993	0.15558
fetal fibroblast 273	1.1781998	0.15584
fetal fibroblast 282	1.1722150	0.15616
fetal kidney 89	1.2093221	0.16552
fibroblast 332	1.1668473	0.16658
fetal renal pelvis 221	1.1979030	0.16666
fetal thymus 257	1.2503306	0.16770
CD19 7	1.2638012	0.16908
CD4 15	1.2962794	0.17188
CD4 16	1.2585245	0.17212
fetal brain 39	1.1558344	0.17252
fetal fibroblast 268	1.1628647	0.17426
fetal renal pelvis 231	1.1899524	0.17556
fetal fibroblast 274	1.1608825	0.18068
keratinocyte 336	1.1396833	0.18350
fetal fibroblast 276	1.1543223	0.18456
melanocyte 340	1.1637702	0.18530
fetal renal pelvis 220	1.1932335	0.18850
fetal renal pelvis 227	1.1869690	0.18858
fetal brain 34	1.1430426	0.18924
hematopoietic stem cell 319	1.1894331	0.18998
fetal thymus 262	1.2475191	0.19022
melanocyte 339	1.1493808	0.19060
fetal brain 29	1.1290243	0.19330
Mobilized CD3 320	1.2552049	0.19396
fetal kidney 98	1.1793242	0.19790
fetal kidney 92	1.1672944	0.20348
fetal lung 128	1.1375645	0.20906
fetal fibroblast 281	1.1394564	0.20922
breast 2	1.1347376	0.21022
fetal kidney 102	1.1685902	0.21406
fetal renal cortex 214	1.1599850	0.21692
fetal renal pelvis 229	1.1567859	0.22062
breast 1	1.1288747	0.22062
fetal brain 40	1.1269837	0.22070
fetal kidney 94	1.1662481	0.22214
fetal renal pelvis 233	1.1530464	0.22996
fetal renal cortex 207	1.1484703	0.23170
fetal spinal cord 236	1.1430379	0.23220
fibroblast 329	1.1259230	0.23294
fetal renal cortex 202	1.1451775	0.23490
fetal kidney 87	1.1460413	0.23658
fetal kidney 100	1.1456563	0.23676
fetal renal pelvis 228	1.1573320	0.23742
fetal fibroblast 278	1.1263370	0.23866
fetal renal pelvis 232	1.1496713	0.23894
fetal brain 35	1.1098859	0.23950
CD14 3	1.1728109	0.24156
fetal kidney 105	1.1339846	0.24694
fetal renal cortex 215	1.1308990	0.24756
fetal kidney 99	1.1350926	0.24960
fetal renal pelvis 230	1.1446607	0.24994
fetal fibroblast 277	1.1173409	0.25376
fetal renal pelvis 224	1.1264158	0.25518
fetal kidney 95	1.1279738	0.25764
fetal adrenal gland 28	1.1165004	0.25950
fibroblast 330	1.1112213	0.26048
fetal skin 234	1.1341177	0.26316
fetal kidney 84	1.1290618	0.26322
fetal ovary 195	1.1439935	0.26476
keratinocyte 334	1.0951762	0.26504
fetal renal pelvis 226	1.1233256	0.26564
fetal renal cortex 209	1.1232692	0.26972
fetal kidney 103	1.1223156	0.26990
fetal kidney 96	1.1141709	0.27814
keratinocyte 335	1.0885581	0.27998
ES derived MSC 289	1.1074942	0.28220
fetal spinal cord 239	1.1058533	0.28224
fetal stomach 244	1.1240980	0.28432
fetal kidney 107	1.1113906	0.28444
fetal fibroblast 267	1.1006011	0.28484
fetal renal pelvis 217	1.1142788	0.28766
fetal muscle 148	1.1082797	0.28792
fetal spinal cord 238	1.1020967	0.28932
fetal renal cortex 213	1.0981677	0.29256
ES derived MSC 290	1.1011897	0.29350
fetal renal pelvis 223	1.1055286	0.30442
fetal lung 299	1.0931821	0.30738
fetal spinal cord 235	1.0895912	0.30762
fetal renal cortex 210	1.0987525	0.31016
fetal renal pelvis 225	1.0960600	0.31454
fetal kidney 97	1.0922276	0.31726
fetal muscle 169	1.0859297	0.32132
muscle 341	1.1008316	0.32146
fetal fibroblast 269	1.0761948	0.32156
fetal kidney 88	1.0842782	0.32296
CD14 4	1.1022936	0.32450
fetal kidney 90	1.0877479	0.32606
fetal kidney 82	1.0896881	0.32750
fetal renal cortex 204	1.0799539	0.32884
fetal lung 298	1.0794521	0.33078
fetal lung 300	1.0736567	0.33256
fetal spinal cord 237	1.0755056	0.33422
fetal muscle 193	1.0793472	0.33760
fetal testes 254	1.0795480	0.33768
fetal lung 115	1.0693346	0.33966
fetal renal cortex 205	1.0770003	0.34066
fetal stomach 251	1.0806829	0.34102
fetal brain 41	1.0653687	0.34144
fetal muscle 168	1.0764530	0.34224
fetal renal pelvis 222	1.0784823	0.34334
fetal renal pelvis 216	1.0776008	0.34364
fetal muscle 172	1.0668836	0.34400
fetal kidney 101	1.0766009	0.34450
fetal muscle 181	1.0705670	0.34652
fetal muscle 160	1.0722088	0.34746
fetal renal cortex 212	1.0711782	0.34832
fetal muscle 180	1.0743267	0.34898
ES derived mesoderm 286	1.0587619	0.35252
fetal kidney 104	1.0681603	0.35500
fetal renal pelvis 219	1.0762700	0.35582
fetal renal cortex 206	1.0663894	0.35764
fetal renal cortex 211	1.0691843	0.35782
fetal muscle 155	1.0668770	0.35904
fetal muscle 185	1.0666290	0.36050
fetal renal cortex 208	1.0655122	0.36166
fetal adrenal gland 27	1.0677694	0.36186
fetal adrenal gland 26	1.0666360	0.36262
fetal muscle 187	1.0616063	0.36348
fetal stomach 248	1.0688393	0.36460
fetal lung 301	1.0586594	0.36760
fetal muscle 163	1.0593729	0.36816
ES cells 295	1.0666231	0.37012
fetal kidney 93	1.0625484	0.37086
fetal muscle 174	1.0587029	0.37298
fetal renal cortex 203	1.0555099	0.37562
fetal lung 110	1.0533069	0.37572
fetal muscle 164	1.0541369	0.38004
fetal kidney 86	1.0572304	0.38020
fetal lung 126	1.0462573	0.38522
fetal lung 120	1.0491234	0.38668
fetal lung 135	1.0476096	0.38814
fetal muscle 153	1.0492578	0.39008
fetal lung 118	1.0420508	0.39224
fetal muscle 192	1.0450878	0.39322
fetal lung 116	1.0425339	0.39464
fetal thymus 263	1.0453002	0.39710
ES derived NP 291	1.0491992	0.39808
fetal muscle 173	1.0406899	0.40378
fetal muscle 149	1.0387613	0.40746
fetal lung 125	1.0385553	0.40792
fetal muscle 143	1.0376127	0.40806
fetal lung 142	1.0333356	0.40908
fetal muscle 179	1.0376121	0.41020
fetal brain 36	1.0337404	0.41174
fetal fibroblast 270	1.0337971	0.41578
fetal lung 133	1.0294077	0.41896
fetal stomach 243	1.0363671	0.41900
fetal brain 38	1.0288392	0.42226
fetal muscle 189	1.0295082	0.42280
fetal muscle 170	1.0291303	0.42330
fetal stomach 250	1.0298110	0.42666
fetal brain 33	1.0255771	0.43128
fetal stomach 240	1.0251429	0.43260
fetal muscle 147	1.0230676	0.43544
fetal lung 121	1.0230500	0.43638
ES derived NP 292	1.0240771	0.43726
fetal muscle 182	1.0226479	0.43888
fetal stomach 249	1.0179754	0.43984
fetal fibroblast 280	1.0199681	0.44244
ES cells 296	1.0194348	0.44256
iPS 304	1.0169239	0.44518
iPS 302	1.0176247	0.44536
fetal lung 117	1.0171556	0.44672
fetal lung 113	1.0170996	0.44838
fetal lung 123	1.0157671	0.44850
fetal lung 134	1.0162263	0.44890
fetal muscle 161	1.0135419	0.45196
fetal adrenal gland 25	1.0168420	0.45242
fetal lung 111	1.0144174	0.45510
fetal stomach 253	1.0106056	0.45578
fetal lung 131	1.0129166	0.45630
fetal brain 32	1.0119916	0.45700
fetal muscle 152	1.0126464	0.45914
fetal stomach 246	1.0100886	0.46050
fetal fibroblast 279	1.0132365	0.46058
fetal muscle 190	1.0086394	0.46274
fetal lung 108	1.0099333	0.46288
fetal renal pelvis 218	1.0020720	0.46412
fetal muscle 165	1.0097133	0.46758
fetal muscle 171	1.0097304	0.47028
fetal stomach 252	1.0007926	0.47180
fetal muscle 186	1.0045649	0.47490
fetal muscle 191	1.0024243	0.47640
fetal muscle 194	1.0036593	0.47708
fetal muscle 159	1.0018379	0.47852
fetal muscle 166	1.0018534	0.48280
fetal muscle 184	1.0011380	0.48394
fetal muscle 162	0.9999340	0.48400
fetal stomach 241	0.9926100	0.48438
ES cells 294	0.9882773	0.48562
fetal muscle 167	0.9984913	0.48788
placenta 196	0.9934585	0.48986
fetal muscle 188	0.9951298	0.49322
iPS 303	0.9929120	0.49382
placenta 200	0.9923131	0.50240
placenta 198	0.9896023	0.51018
fetal stomach 245	0.9781908	0.51106
fetal adrenal gland 23	0.9873983	0.51354
fetal muscle 176	0.9868015	0.51416
ES cells 293	0.9750367	0.51490
fetal muscle 150	0.9833476	0.51528
fetal lung 122	0.9807125	0.51812
fetal muscle 144	0.9795472	0.51990
fetal large intestine 53	0.9690279	0.52088
fetal lung 138	0.9819164	0.52104
fetal muscle 158	0.9820457	0.52390
placenta 201	0.9730270	0.52422
fetal muscle 177	0.9756173	0.52432
fetal lung 136	0.9806391	0.52462
fetal muscle 175	0.9761739	0.53194
fetal muscle 156	0.9743299	0.53486
placenta 199	0.9744370	0.53510
fetal stomach 242	0.9512635	0.53674
fetal muscle 146	0.9743830	0.53742
stomach 283	0.9625586	0.54038
fetal muscle 178	0.9681517	0.54736
fetal large intestine 57	0.9706038	0.54816
fetal muscle 154	0.9640988	0.54886
ES derived mesoderm 285	0.9706855	0.55230
fetal muscle 151	0.9627313	0.55254
fetal small intestine 69	0.9554793	0.55430
fetal lung 139	0.9624712	0.55566
fetal muscle 145	0.9582404	0.55694
fetal adrenal gland 24	0.9542685	0.55740
heart 297	0.9554212	0.55752
fetal muscle 183	0.9653931	0.55762
fetal muscle 157	0.9656571	0.56156
fetal lung 119	0.9643713	0.56282
fetal lung 109	0.9660762	0.56514
fetal lung 132	0.9617869	0.56526
fetal large intestine 54	0.9548411	0.56602
fetal small intestine 72	0.9547473	0.56856
fetal small intestine 71	0.9475235	0.57522
pancreas 328	0.9473350	0.57676
fetal heart 48	0.9503934	0.57694
fetal lung 140	0.9581314	0.57736
placenta 197	0.9483203	0.57756
fetal lung 124	0.9507843	0.58524
fetal heart 42	0.9457666	0.58606
fetal lung 127	0.9465019	0.59108
fetal large intestine 59	0.9345624	0.59476
pancreas 327	0.9317396	0.59570
fetal small intestine 68	0.9420923	0.60084
fetal lung 137	0.9443771	0.60540
fetal lung 114	0.9484251	0.60546
fetal stomach 247	0.9144801	0.60616
fetal small intestine 78	0.9408986	0.60708
fetal large intestine 66	0.9256506	0.61176
fetal heart 49	0.9312068	0.61260
fetal small intestine 73	0.9276705	0.61278
fetal heart 47	0.9257246	0.61460
fetal lung 141	0.9401358	0.62184
fetal heart 46	0.9228352	0.62398
fetal large intestine 55	0.9255048	0.62940
fetal lung 129	0.9277155	0.63342
fetal large intestine 64	0.9226887	0.63400
CD20 8	0.9207962	0.64132
fetal large intestine 60	0.9131759	0.64504
fetal lung 112	0.9203986	0.64778
fetal large intestine 65	0.9059791	0.65318
fetal large intestine 67	0.9041609	0.65602
fetal heart 45	0.9082698	0.65678
ES derived trophoblast 288	0.9204691	0.65732
fetal kidney 81	0.9163779	0.65828
fetal large intestine 63	0.9014322	0.66154
fetal small intestine 77	0.8967005	0.66642
fetal heart 44	0.8800770	0.67020
fetal large intestine 58	0.9072277	0.67392
fetal lung 130	0.9070741	0.67590
fetal small intestine 79	0.8862439	0.67780
fetal small intestine 76	0.8861944	0.67828
fetal small intestine 80	0.8926721	0.69020
fetal heart 51	0.8888308	0.69822
fetal heart 52	0.8955775	0.69830
fetal heart 50	0.8813956	0.70566
fetal large intestine 62	0.8658621	0.70940
fetal small intestine 74	0.8709778	0.72466
fetal heart 43	0.8729178	0.73382
stomach 284	0.8488897	0.73790
fetal small intestine 70	0.8731110	0.74732
small intestine 342	0.8577621	0.74976
ES derived trophoblast 287	0.8553055	0.76136
fetal large intestine 56	0.8247868	0.79386

Migraine

DHS sample	fold enrichment	p value
fetal spinal cord 239	1.6134677	0.00826
fetal brain 41	1.4654080	0.01120
fetal spinal cord 236	1.5982665	0.01496
fetal spinal cord 237	1.5151314	0.01974
fetal spinal cord 238	1.5203482	0.02004
fetal brain 39	1.3589981	0.02552
ES derived NP 292	1.6670142	0.02832
fetal spinal cord 235	1.4401412	0.03944
fetal fibroblast 268	1.4134502	0.04084
heart 297	1.5999514	0.04262
ES derived NP 291	1.5968743	0.04858
fetal brain 37	1.3014473	0.06518
fetal fibroblast 267	1.3716920	0.06630
fetal brain 36	1.3200465	0.06656
fetal fibroblast 272	1.3678536	0.06672
fibroblast 329	1.3768130	0.07472
fetal fibroblast 270	1.3433578	0.07482
fetal heart 45	1.4104570	0.07960
fibroblast 331	1.3535367	0.08514
fetal brain 38	1.2784112	0.09112
fetal fibroblast 281	1.3200355	0.09142
fetal fibroblast 278	1.3288561	0.09178
fetal fibroblast 271	1.3166249	0.09242
fetal brain 40	1.2426217	0.09300
fetal brain 29	1.2014018	0.09856
fetal heart 46	1.3894819	0.10242
fibroblast 330	1.3280110	0.10676
fetal heart 51	1.3478106	0.11364
fibroblast 332	1.3090460	0.11514
fetal heart 52	1.3236625	0.11524
fetal heart 42	1.3525290	0.11550
fetal brain 33	1.2656293	0.11698
fetal fibroblast 282	1.2694167	0.11740
fetal fibroblast 273	1.2719053	0.11964
fetal fibroblast 274	1.2793095	0.12010
fetal heart 44	1.3942685	0.13206
fetal heart 49	1.3240560	0.13364
fetal fibroblast 280	1.2596617	0.13390
fetal fibroblast 275	1.2547599	0.13408
fetal fibroblast 277	1.2550911	0.14720
fetal brain 30	1.1588445	0.14812
fetal fibroblast 276	1.2380325	0.14964
fetal kidney 84	1.3087624	0.15416
fetal heart 47	1.3131474	0.15510
hematopoietic stem cell 307	1.3065357	0.15692
hematopoietic stem cell 319	1.3296801	0.15866
hematopoietic stem cell 313	1.2882127	0.15986
fetal fibroblast 279	1.2321170	0.16168
fetal stomach 247	1.3859324	0.16308
fetal brain 35	1.1595293	0.16348
fetal fibroblast 269	1.2205688	0.16654
fetal brain 34	1.1664042	0.16676
hematopoietic stem cell 309	1.2873220	0.17054
fetal heart 50	1.2756510	0.17192
fetal muscle 171	1.2408139	0.17242
pancreas 327	1.3136392	0.17556
fetal stomach 250	1.3180070	0.17600
hematopoietic stem cell 306	1.2783086	0.17846
fetal renal pelvis 219	1.3195670	0.18420
fetal heart 43	1.2373251	0.18866
iPS 302	1.2427083	0.18954
fetal renal pelvis 221	1.2425957	0.18962
fetal heart 48	1.2477526	0.19098
fetal stomach 242	1.3370584	0.19140
fetal stomach 240	1.3056558	0.19268
hematopoietic stem cell 317	1.2252931	0.19684
hematopoietic stem cell 316	1.2660085	0.19844
fetal large intestine 66	1.2641913	0.20344
fetal kidney 93	1.2458329	0.20940
fetal stomach 245	1.2573712	0.21226
fetal stomach 251	1.2482114	0.21278
fetal muscle 157	1.1915484	0.21394
fetal stomach 241	1.2637058	0.21442
fetal renal cortex 208	1.2314814	0.21464
fetal stomach 246	1.2625481	0.21774
fetal stomach 249	1.2582769	0.21880
fetal renal pelvis 230	1.2349031	0.21986
fetal muscle 183	1.1820954	0.22008
fetal large intestine 63	1.2448814	0.22324
fetal renal pelvis 233	1.2169524	0.22454
hematopoietic stem cell 311	1.2155032	0.22640
fetal renal cortex 215	1.2038274	0.22700
fetal renal pelvis 225	1.2171776	0.22858
fetal kidney 100	1.2055449	0.22906
fetal muscle 161	1.1917974	0.22942
fetal renal cortex 203	1.2091255	0.23018
fetal brain 32	1.1452921	0.23454
fetal kidney 94	1.2119558	0.23518
fetal renal pelvis 216	1.2065574	0.23556
iPS 304	1.1737587	0.23610
fetal muscle 159	1.2019515	0.23636
fetal renal cortex 202	1.1933193	0.23952
fetal kidney 88	1.1889345	0.24016
fetal muscle 186	1.1707665	0.24242
hematopoietic stem cell 308	1.1904838	0.24376
ES cells 294	1.2139336	0.24378
ES cells 295	1.2078093	0.24392
fetal renal pelvis 232	1.1981909	0.24686
fetal muscle 143	1.1630299	0.24746
fetal renal pelvis 226	1.1888054	0.24800
fetal stomach 252	1.2067969	0.24848
fetal muscle 190	1.1829682	0.24852
fetal muscle 158	1.1671220	0.24934
fetal muscle 174	1.1737578	0.24944
fetal small intestine 73	1.2044949	0.24958
fetal renal cortex 212	1.1778856	0.25016
fetal renal pelvis 222	1.1911738	0.25022
fetal kidney 103	1.1941752	0.25130
fetal muscle 176	1.1606371	0.25174
fetal brain 31	1.1027568	0.25384
pancreas 328	1.2012436	0.25422
fetal renal pelvis 218	1.2184522	0.25434
fetal muscle 163	1.1694586	0.25472
fetal stomach 244	1.2041854	0.25706
fetal renal cortex 207	1.1836857	0.25758
fetal muscle 144	1.1742782	0.25832
fetal muscle 175	1.1586750	0.25944
fetal renal cortex 204	1.1633754	0.26200
fetal kidney 102	1.1832473	0.26308
fetal muscle 178	1.1642358	0.26320
fetal renal cortex 206	1.1681551	0.26428
fetal renal pelvis 223	1.1846127	0.26474
fetal renal pelvis 220	1.1769437	0.26714
fetal small intestine 68	1.1714484	0.26926
iPS 303	1.1590023	0.27070
fetal large intestine 62	1.1902162	0.27082
fetal kidney 104	1.1722760	0.27114
ES cells 296	1.1780830	0.27222
hematopoietic stem cell 305	1.1737668	0.27222
fetal testes 255	1.1503009	0.27408
fetal muscle 172	1.1324661	0.27700
fetal large intestine 59	1.1795724	0.27812
fetal muscle 147	1.1452319	0.27844
fetal kidney 107	1.1548282	0.27970
fetal stomach 248	1.1775706	0.27972
CD8 21	1.2237586	0.28234
fetal muscle 156	1.1414419	0.28248
fetal stomach 253	1.1769110	0.28352
fetal small intestine 71	1.1686439	0.28394
fetal muscle 191	1.1441662	0.28634
fetal renal pelvis 229	1.1495413	0.28836
fetal renal cortex 211	1.1547454	0.28854
fetal muscle 185	1.1493143	0.28896
ES cells 293	1.1548499	0.29058
fetal renal pelvis 224	1.1393279	0.29192
fetal kidney 105	1.1428985	0.29366
fetal muscle 154	1.1435681	0.29504
fetal kidney 90	1.1442559	0.29840
fetal renal pelvis 217	1.1432626	0.30234
hematopoietic stem cell 312	1.1379835	0.30312
Mobilized CD3 321	1.1903740	0.30316
fetal kidney 99	1.1381701	0.30316
fetal muscle 189	1.1250553	0.30334
fetal kidney 97	1.1317452	0.30598
fetal renal cortex 213	1.1321637	0.30640
fetal kidney 86	1.1480764	0.30722
fetal kidney 98	1.1354612	0.30746
fetal thymus 262	1.1732823	0.30912
stomach 284	1.1463930	0.31402
fetal muscle 150	1.1224945	0.31630
fetal renal cortex 214	1.1253431	0.31690
fetal large intestine 54	1.1383232	0.31768
Mobilized CD4 322	1.1679757	0.31778
fetal lung 300	1.1165906	0.31822
fetal muscle 177	1.1177214	0.31882
fetal large intestine 61	1.1576112	0.32034
hematopoietic stem cell 318	1.1309476	0.32060
CD3 11	1.1586354	0.32158
fetal small intestine 74	1.1321590	0.32212
hematopoietic stem cell 314	1.1189137	0.32274
fetal muscle 152	1.1085040	0.32512
fetal muscle 151	1.1170800	0.32608
fetal kidney 82	1.1232576	0.32684
CD19 5	1.1504646	0.32712
fetal large intestine 65	1.1281891	0.32716
fetal muscle 162	1.1051472	0.32764
fetal testes 254	1.1157545	0.32830
fetal small intestine 72	1.1233891	0.32860
fetal lung 124	1.1003754	0.33086
fetal muscle 193	1.1058397	0.33216
fetal renal cortex 209	1.1141148	0.33328
fetal muscle 166	1.1000821	0.33334
CD8 20	1.1413590	0.33504
fetal muscle 168	1.1061737	0.33676
fetal renal pelvis 227	1.1087489	0.33684
fetal muscle 148	1.1011492	0.33746
hematopoietic stem cell 310	1.1027101	0.33880
stomach 283	1.1209131	0.33944
CD20 8	1.1097187	0.34172
CD4 15	1.1395323	0.34364
fetal renal cortex 210	1.1032971	0.34514
CD3 12	1.1321491	0.34548
CD19 7	1.1248957	0.34560
fetal renal pelvis 231	1.0929245	0.34794
fetal muscle 155	1.0964288	0.34822
fetal lung 113	1.0809486	0.35226
fetal muscle 192	1.0865058	0.35326
fetal stomach 243	1.0968514	0.35330
fetal renal pelvis 228	1.0996568	0.35388
fetal small intestine 69	1.1083330	0.35394
fetal muscle 170	1.0844644	0.35420
fetal kidney 91	1.0897458	0.35572
hematopoietic stem cell 315	1.0941253	0.35872
fetal muscle 188	1.0788124	0.35968
fetal lung 112	1.0791448	0.35986
fetal muscle 165	1.0792022	0.36016
fetal lung 142	1.0754379	0.36022
fetal muscle 145	1.0853735	0.36190
fetal small intestine 70	1.0859576	0.36206
fetal muscle 179	1.0808690	0.36322
fetal lung 109	1.0690619	0.36420
CD8 19	1.1130354	0.36666
fetal muscle 160	1.0822634	0.36800
fetal large intestine 60	1.0876497	0.37010
fetal kidney 83	1.0866585	0.37036
fetal lung 138	1.0734303	0.37066
fetal skin 234	1.0820234	0.37082
fetal lung 132	1.0726027	0.37140
ES derived MSC 290	1.0753197	0.37174
fetal muscle 187	1.0716782	0.37348
fetal muscle 146	1.0734152	0.37352
fetal lung 131	1.0620992	0.37384
fetal small intestine 79	1.0872458	0.37408
fetal lung 123	1.0706958	0.37502
fetal small intestine 76	1.0827119	0.37618
fetal muscle 180	1.0756495	0.37636
ES derived mesoderm 286	1.0610516	0.37788
fetal kidney 95	1.0780024	0.37792
CD19 6	1.0908121	0.38072
fetal muscle 153	1.0732969	0.38094
fetal kidney 101	1.0725724	0.38094
keratinocyte 334	1.0608454	0.38120
fetal kidney 89	1.0724286	0.38142
CD3 10	1.0856454	0.38176
fetal lung 110	1.0644722	0.38302
melanocyte 337	1.0568483	0.38402
fetal lung 139	1.0625186	0.38504
fetal kidney 92	1.0674360	0.38562
fetal ovary 195	1.0723302	0.38618
fetal large intestine 58	1.0692027	0.38882
fetal lung 301	1.0677553	0.38908
fetal lung 130	1.0587065	0.39026
fetal muscle 184	1.0583727	0.39110
fetal small intestine 78	1.0602434	0.39654
fetal large intestine 56	1.0658114	0.39750
fetal small intestine 75	1.0352484	0.40068
CD56 18	1.0680609	0.40512
CD56 17	1.0607798	0.40552
fetal kidney 87	1.0504556	0.40922
ES derived trophoblast 287	1.0532452	0.41088
fetal kidney 96	1.0465599	0.41294
fetal muscle 181	1.0456799	0.41374
fetal lung 115	1.0428545	0.41390
fetal large intestine 57	1.0465860	0.41728
Mobilized CD8 325	1.0473866	0.41788
fetal kidney 81	1.0420498	0.41816
fetal thymus 263	1.0413388	0.42064
muscle 341	1.0425873	0.42176
fetal renal cortex 205	1.0373959	0.42410
fetal large intestine 64	1.0385906	0.42600
fetal lung 122	1.0317342	0.42662
fetal lung 136	1.0336957	0.42750
fetal muscle 164	1.0348083	0.42958
keratinocyte 336	1.0294683	0.43256
CD8 22	1.0181068	0.43424
fetal lung 114	1.0283955	0.43436
fetal muscle 182	1.0300177	0.43558
fetal kidney 106	1.0261615	0.43634
fetal muscle 194	1.0264158	0.43686
ES derived trophoblast 288	1.0295119	0.43886
fetal lung 117	1.0274096	0.44116
ES derived MSC 289	1.0227357	0.44442
fetal lung 135	1.0230869	0.44522
fetal muscle 167	1.0221194	0.44674
fetal lung 129	1.0219919	0.44678
keratinocyte 333	1.0210887	0.44870
ovary 326	1.0114676	0.44996
fetal lung 126	1.0202695	0.45074
CD4 14	1.0138978	0.45602
fetal kidney 85	1.0133033	0.45932
Mobilized CD4 323	1.0020400	0.45970
fetal muscle 169	1.0144053	0.46002
fetal muscle 173	1.0092784	0.46646
fetal lung 128	1.0078150	0.47160
fetal lung 299	1.0093699	0.47296
small intestine 342	1.0026672	0.47316
keratinocyte 335	1.0068718	0.47620
fetal large intestine 55	1.0001686	0.47660
fetal lung 125	0.9994796	0.47888
fetal large intestine 53	0.9918405	0.47902
CD3 9	0.9887553	0.48006
fetal lung 127	0.9948675	0.48416
fetal thymus 261	0.9771626	0.49076
CD4 13	0.9793076	0.49092
CD4 16	0.9458683	0.49986
fetal small intestine 77	0.9764094	0.50038
Mobilized CD3 320	0.9697402	0.50386
fetal thymus 265	0.9722772	0.50458
fetal small intestine 80	0.9804660	0.50704
fetal lung 134	0.9818768	0.50762
fetal thymus 264	0.9652553	0.51374
fetal lung 137	0.9765757	0.51960
fetal lung 119	0.9757185	0.52662
fetal thymus 260	0.9481383	0.53072
fetal lung 120	0.9669684	0.53606
fetal lung 108	0.9552077	0.54142
Mobilized CD56 324	0.9375225	0.54196
breast 2	0.9658086	0.54530
fetal lung 298	0.9570845	0.54668
fetal thymus 266	0.9364463	0.55002
breast 1	0.9607387	0.55512
fetal lung 133	0.9501352	0.56840
fetal thymus 259	0.9052835	0.57232
fetal lung 140	0.9433891	0.57522
fetal lung 118	0.9499801	0.57598
fetal lung 141	0.9440853	0.57608
fetal thymus 256	0.9121945	0.58366
melanocyte 338	0.9477464	0.59302
fetal thymus 258	0.8915983	0.59918
melanocyte 339	0.9420451	0.60454
fetal lung 116	0.9274533	0.61356
fetal lung 121	0.9199006	0.61986
melanocyte 340	0.9289061	0.62540
fetal thymus 257	0.8554743	0.62566
fetal muscle 149	0.8944708	0.62812
ES derived mesoderm 285	0.9157789	0.63782
fetal lung 111	0.8938568	0.64860
CD14 4	0.8174066	0.67508
fetal adrenal gland 27	0.8546542	0.67872
fetal large intestine 67	0.8349007	0.68718
fetal adrenal gland 23	0.8594677	0.68924
placenta 201	0.8138508	0.69062
CD14 3	0.7769017	0.70358
fetal adrenal gland 26	0.8374756	0.70414
fetal adrenal gland 24	0.7661129	0.75050
placenta 199	0.7907257	0.75458
placenta 197	0.7577065	0.76636
fetal adrenal gland 28	0.8042463	0.78470
placenta 200	0.7747380	0.78732
placenta 198	0.7546498	0.80030
fetal adrenal gland 25	0.7300325	0.80688
placenta 196	0.6692517	0.86050

Atherosclerosis

DHS sample	fold enrichment	p value
pancreas 328	1.2976053	0.03226
keratinocyte 335	1.1585894	0.06912
CD14 4	1.2319453	0.07542
fetal fibroblast 269	1.1666203	0.07794
breast 1	1.1652106	0.07862
keratinocyte 333	1.1475253	0.07968
fetal large intestine 67	1.2114834	0.08228
keratinocyte 336	1.1457744	0.08328
ES derived trophoblast 288	1.1778616	0.08566
fetal large intestine 64	1.1931279	0.09276
pancreas 327	1.2133673	0.09454
ES derived trophoblast 287	1.1801872	0.09994
fetal large intestine 56	1.1793683	0.11174
CD20 8	1.1665696	0.11622
breast 2	1.1388742	0.11864
fetal small intestine 70	1.1514614	0.12450
fetal small intestine 78	1.1510053	0.12838
fetal small intestine 74	1.1624959	0.13026
fetal large intestine 57	1.1525989	0.13132
CD14 3	1.1811436	0.13802
fetal large intestine 58	1.1464890	0.14088
fetal lung 301	1.1326343	0.14408
melanocyte 338	1.1123606	0.14814
fetal large intestine 60	1.1553890	0.14854
fetal lung 298	1.1348772	0.14890
muscle 341	1.1610982	0.15002
fetal small intestine 69	1.1596967	0.15208
fetal small intestine 80	1.1464342	0.15234
fetal fibroblast 280	1.1172160	0.15786
fetal fibroblast 279	1.1145930	0.16238
melanocyte 339	1.1069878	0.17058
melanocyte 340	1.1134966	0.17212
keratinocyte 334	1.0974581	0.17442
fetal small intestine 68	1.1294708	0.17774
fetal large intestine 55	1.1272834	0.18152
fetal large intestine 62	1.1429191	0.18168
fetal adrenal gland 28	1.1106227	0.18346
fetal fibroblast 270	1.1065100	0.18518
fetal lung 300	1.1064726	0.19314
fibroblast 332	1.1020182	0.19416
fetal small intestine 77	1.1241841	0.19976
fetal fibroblast 273	1.0962935	0.20176
fibroblast 330	1.0988514	0.20564
fetal fibroblast 281	1.0933243	0.20882
small intestine 342	1.1150802	0.20892
fetal lung 299	1.1041011	0.20986
fetal fibroblast 282	1.0898224	0.21098
fetal adrenal gland 24	1.1204569	0.21282
melanocyte 337	1.0831293	0.21582
fetal adrenal gland 25	1.1080794	0.22774
fibroblast 329	1.0884926	0.23060
stomach 284	1.1106036	0.23166
fetal adrenal gland 23	1.0890708	0.24490
fetal large intestine 65	1.0999923	0.24746
fetal fibroblast 276	1.0766357	0.24788
fetal fibroblast 274	1.0771787	0.25290
fibroblast 331	1.0755239	0.25842
fetal small intestine 79	1.0929382	0.26616
fetal small intestine 76	1.0917423	0.26902
fetal large intestine 54	1.0887286	0.27032
fetal small intestine 72	1.0854837	0.27250
fetal small intestine 71	1.0866126	0.27408
fetal fibroblast 275	1.0658864	0.27454
ES derived MSC 289	1.0760243	0.27538
fetal testes 255	1.0757072	0.27786
fetal small intestine 73	1.0788251	0.29058
hematopoietic stem cell 310	1.0651915	0.29600
fetal fibroblast 271	1.0584501	0.30226
hematopoietic stem cell 314	1.0656063	0.30244
fetal fibroblast 268	1.0577748	0.30336
fetal fibroblast 277	1.0598039	0.30488
fetal large intestine 53	1.0732824	0.31562
placenta 201	1.0679654	0.31702
hematopoietic stem cell 309	1.0630372	0.31822
hematopoietic stem cell 312	1.0605243	0.31910
ES derived mesoderm 285	1.0546909	0.31978
ES derived MSC 290	1.0578914	0.32288
fetal stomach 244	1.0671957	0.32412
fetal adrenal gland 26	1.0597765	0.32548
hematopoietic stem cell 306	1.0572954	0.33248
fetal fibroblast 278	1.0497707	0.33356
fetal large intestine 59	1.0590710	0.33836
fetal lung 119	1.0465496	0.33984
fetal fibroblast 267	1.0483352	0.34038
ES cells 296	1.0618899	0.34176
ovary 326	1.0602079	0.34796
fetal large intestine 63	1.0520265	0.35356
fetal stomach 253	1.0550581	0.35548
iPS 304	1.0452053	0.35702
fetal muscle 164	1.0458770	0.35864
hematopoietic stem cell 313	1.0457275	0.36008
stomach 283	1.0491816	0.36506
ES cells 294	1.0519794	0.36526
fetal thymus 259	1.0537527	0.36596
fetal thymus 258	1.0504149	0.36896
fetal muscle 151	1.0424084	0.37156
hematopoietic stem cell 317	1.0373682	0.37524
fetal muscle 166	1.0378999	0.37646
fetal large intestine 66	1.0428811	0.37862
fetal testes 254	1.0381641	0.38230
fetal thymus 256	1.0432353	0.38444
fetal muscle 169	1.0357119	0.38716
iPS 302	1.0352388	0.39088
fetal adrenal gland 27	1.0347472	0.39680
hematopoietic stem cell 311	1.0320111	0.40222
hematopoietic stem cell 315	1.0317609	0.40240
iPS 303	1.0298671	0.40566
fetal muscle 167	1.0285431	0.40592
fetal muscle 150	1.0289664	0.40702
fetal lung 129	1.0255846	0.41028
fetal fibroblast 272	1.0236763	0.41440
heart 297	1.0280826	0.41734
CD3 10	1.0283996	0.42242
fetal thymus 265	1.0272244	0.42438
fetal muscle 180	1.0232204	0.42622
fetal thymus 266	1.0212288	0.43668
placenta 200	1.0170932	0.44004
fetal lung 140	1.0168113	0.44016
hematopoietic stem cell 308	1.0155287	0.44464
CD19 5	1.0183985	0.44668
fetal thymus 260	1.0145190	0.45124
hematopoietic stem cell 319	1.0142784	0.45268
ES cells 295	1.0129924	0.45314
fetal thymus 261	1.0125658	0.45542
fetal muscle 154	1.0090287	0.46206
fetal ovary 195	1.0076427	0.46612
hematopoietic stem cell 305	1.0084575	0.46716
hematopoietic stem cell 316	1.0080448	0.46822
fetal muscle 179	1.0068134	0.46860
fetal muscle 149	1.0058229	0.47100
hematopoietic stem cell 318	1.0062049	0.47300
fetal stomach 247	1.0044214	0.47406
CD19 7	1.0059947	0.47506
hematopoietic stem cell 307	1.0041274	0.47650
fetal thymus 264	1.0043103	0.47760
fetal muscle 157	1.0043791	0.47790
fetal muscle 171	1.0041557	0.47938
fetal muscle 193	1.0018190	0.48326
fetal muscle 165	1.0019474	0.48446
fetal muscle 153	1.0013481	0.48600
ES derived mesoderm 286	1.0020100	0.48644
fetal stomach 242	0.9978097	0.48816
CD19 6	0.9976686	0.49068
fetal stomach 248	0.9971129	0.49250
fetal muscle 181	0.9976250	0.49414
fetal muscle 159	0.9983901	0.49534
fetal muscle 176	0.9978216	0.49610
placenta 199	0.9975411	0.49792
fetal lung 134	0.9956701	0.50390
placenta 198	0.9966303	0.50430
fetal renal pelvis 220	0.9924622	0.50556
fetal thymus 263	0.9942579	0.50578
fetal muscle 185	0.9924396	0.50908
fetal lung 112	0.9932086	0.51074
Mobilized CD4 323	0.9875537	0.51222
fetal renal cortex 207	0.9903190	0.51234
placenta 196	0.9918862	0.51392
fetal lung 111	0.9939038	0.51472
fetal muscle 168	0.9915681	0.51480
fetal muscle 162	0.9924970	0.51516
CD4 13	0.9874593	0.51520
fetal muscle 184	0.9925389	0.51570
fetal kidney 91	0.9906253	0.51694
fetal stomach 241	0.9884185	0.51758
fetal heart 45	0.9903200	0.52122
fetal thymus 257	0.9810501	0.52620
fetal brain 36	0.9896529	0.52776
fetal heart 43	0.9869743	0.52862
fetal stomach 243	0.9848275	0.52914
fetal muscle 152	0.9882181	0.52956
fetal muscle 143	0.9874968	0.52998
fetal stomach 240	0.9797401	0.53372
fetal muscle 174	0.9828436	0.53798
fetal muscle 146	0.9839261	0.54004
fetal muscle 178	0.9827785	0.54014
CD4 14	0.9793521	0.54134
CD4 15	0.9691118	0.54136
fetal heart 46	0.9813153	0.54210
fetal muscle 182	0.9831616	0.54224
fetal lung 125	0.9838728	0.54274
fetal lung 117	0.9843897	0.54398
fetal muscle 175	0.9807488	0.54630
fetal heart 52	0.9815281	0.54672
fetal brain 38	0.9841125	0.54852
fetal skin 234	0.9801097	0.55328
fetal lung 133	0.9831402	0.55428
fetal stomach 246	0.9715204	0.55518
fetal heart 49	0.9772080	0.55526
fetal stomach 249	0.9702982	0.55550
fetal lung 108	0.9776482	0.55724
fetal stomach 251	0.9722842	0.55794
fetal muscle 155	0.9748897	0.55880
Mobilized CD56 324	0.9703084	0.56224
fetal muscle 194	0.9787501	0.56346
fetal renal pelvis 233	0.9713411	0.56424
fetal lung 130	0.9777391	0.56452
fetal muscle 190	0.9703868	0.56600
fetal heart 47	0.9701257	0.56604
fetal kidney 103	0.9696640	0.56728
fetal renal pelvis 219	0.9653699	0.56738
fetal kidney 92	0.9699939	0.56922
fetal lung 116	0.9785024	0.56960
fetal heart 51	0.9734734	0.57000
fetal spinal cord 235	0.9734991	0.57070
placenta 197	0.9707675	0.57146
fetal stomach 250	0.9642275	0.57336
fetal muscle 144	0.9690027	0.57550
fetal muscle 189	0.9716458	0.57742
fetal lung 121	0.9753626	0.57882
fetal thymus 262	0.9495915	0.57966
fetal muscle 177	0.9661449	0.57968
ES cells 293	0.9641702	0.58112
fetal muscle 173	0.9687217	0.58170
fetal stomach 252	0.9610329	0.58650
Mobilized CD3 320	0.9542965	0.58804
fetal kidney 96	0.9643557	0.58940
fetal muscle 172	0.9711354	0.59032
fetal heart 42	0.9639505	0.59416
fetal muscle 187	0.9670742	0.59422
fetal lung 127	0.9655772	0.59728
CD56 17	0.9565565	0.59772
fetal lung 114	0.9704830	0.59792
fetal muscle 191	0.9623870	0.59844
fetal muscle 183	0.9644591	0.60058
fetal muscle 148	0.9628681	0.60082
fetal muscle 188	0.9628947	0.60182
fetal renal pelvis 221	0.9585815	0.60624
fetal muscle 161	0.9585863	0.60764
fetal muscle 145	0.9550778	0.60906
fetal stomach 245	0.9528787	0.60920
fetal renal cortex 212	0.9566871	0.60970
fetal lung 118	0.9667856	0.61240
fetal renal pelvis 225	0.9532121	0.61280
fetal lung 128	0.9661875	0.61334
ES derived NP 291	0.9494525	0.61440
fetal muscle 147	0.9563996	0.61468
fetal muscle 160	0.9561607	0.61488
fetal lung 113	0.9637537	0.61578
fetal lung 123	0.9601203	0.61618
fetal muscle 156	0.9576048	0.61694
fetal muscle 192	0.9566953	0.61898
fetal kidney 81	0.9580228	0.61970
fetal heart 44	0.9451756	0.62040
fetal muscle 163	0.9538104	0.62184
fetal lung 137	0.9609415	0.62358
fetal spinal cord 238	0.9576586	0.62402
fetal renal cortex 202	0.9495172	0.62530
fetal lung 120	0.9587584	0.62550
fetal kidney 83	0.9418313	0.62678
CD3 9	0.9391605	0.62878
fetal renal pelvis 231	0.9506763	0.62930
fetal renal pelvis 228	0.9438642	0.63296
fetal lung 109	0.9600010	0.63608
fetal heart 48	0.9484983	0.63612
fetal kidney 85	0.9514325	0.63652
fetal lung 122	0.9502969	0.64018
fetal heart 50	0.9467058	0.64044
fetal lung 141	0.9573779	0.64074
fetal kidney 100	0.9438791	0.64180
fetal lung 131	0.9577435	0.64186
fetal muscle 186	0.9493970	0.64230
fetal brain 41	0.9575770	0.64252
fetal kidney 106	0.9435864	0.64292
fetal renal pelvis 226	0.9439493	0.64352
fetal renal cortex 213	0.9479105	0.64522
fetal renal pelvis 227	0.9416525	0.64612
fetal kidney 87	0.9420906	0.64988
fetal lung 132	0.9504687	0.65080
fetal kidney 84	0.9381339	0.65234
fetal brain 34	0.9576035	0.65364
Mobilized CD8 325	0.9211631	0.65616
fetal lung 115	0.9506947	0.65634
fetal kidney 95	0.9386828	0.65810
fetal muscle 170	0.9445188	0.66010
fetal renal cortex 205	0.9369386	0.66152
fetal lung 124	0.9449354	0.66572
fetal renal cortex 214	0.9356806	0.66684
fetal kidney 82	0.9282964	0.66830
fetal lung 110	0.9415545	0.67212
CD8 21	0.9141251	0.67272
fetal renal cortex 211	0.9274005	0.67294
fetal renal cortex 215	0.9347335	0.67362
fetal spinal cord 237	0.9407670	0.67392
Mobilized CD4 322	0.9112090	0.67618
CD3 11	0.9219551	0.67700
fetal kidney 102	0.9249952	0.67750
fetal kidney 99	0.9301315	0.67988
ES derived NP 292	0.9239282	0.68214
fetal lung 139	0.9359996	0.68284
Mobilized CD3 321	0.9026400	0.68874
fetal lung 136	0.9374342	0.68896
fetal lung 135	0.9358164	0.69408
CD8 19	0.8975099	0.69494
fetal renal pelvis 232	0.9195968	0.69512
fetal brain 35	0.9479157	0.69548
fetal renal pelvis 230	0.9168649	0.69596
fetal kidney 94	0.9172272	0.69688
fetal renal pelvis 229	0.9234068	0.69770
fetal kidney 89	0.9214941	0.69952
fetal kidney 105	0.9235899	0.70244
fetal lung 126	0.9376572	0.70252
CD4 16	0.8958324	0.70528
fetal renal pelvis 218	0.9020509	0.70578
fetal renal cortex 209	0.9186421	0.70580
fetal lung 138	0.9295349	0.70728
fetal large intestine 61	0.9007559	0.71294
fetal spinal cord 236	0.9223354	0.71426
fetal renal pelvis 217	0.9128854	0.71436
fetal kidney 86	0.9006294	0.71612
fetal renal cortex 204	0.9214351	0.71696
fetal kidney 104	0.9135939	0.71728
fetal brain 37	0.9343886	0.71874
fetal lung 142	0.9307891	0.71886
fetal muscle 158	0.9234453	0.71946
fetal renal cortex 206	0.9163625	0.72290
CD3 12	0.8870426	0.72482
fetal kidney 90	0.9103331	0.72650
fetal renal pelvis 224	0.9164603	0.72656
fetal kidney 98	0.9081067	0.72838
fetal spinal cord 239	0.9205502	0.72954
fetal brain 39	0.9324770	0.73080
CD56 18	0.8863589	0.73202
fetal kidney 88	0.9119763	0.73720
fetal renal pelvis 222	0.9028818	0.73830
fetal brain 32	0.9287489	0.74308
fetal kidney 107	0.9035847	0.74630
fetal kidney 101	0.9045894	0.74890
fetal kidney 97	0.9009760	0.75146
CD8 20	0.8853054	0.75356
fetal renal pelvis 223	0.8939299	0.75436
fetal small intestine 75	0.8472675	0.75956
CD8 22	0.8700492	0.76068
fetal renal cortex 210	0.8848394	0.77784
fetal brain 40	0.9158231	0.78634
fetal renal pelvis 216	0.8809866	0.78962
fetal renal cortex 203	0.8835309	0.78992
fetal renal cortex 208	0.8733911	0.80450
fetal brain 33	0.8985118	0.80530
fetal kidney 93	0.8687694	0.81104
fetal brain 30	0.9114150	0.83824
fetal brain 29	0.9039574	0.84804
fetal brain 31	0.8969469	0.86684

Cardiovascular−related traits

DHS sample	fold enrichment	p value
melanocyte 338	1.2955936	0.03976
melanocyte 337	1.2443488	0.06670
CD14 4	1.3480721	0.07356
fetal adrenal gland 28	1.2047763	0.13468
stomach 284	1.2650926	0.13784
placenta 200	1.2054535	0.15276
pancreas 328	1.2171835	0.17794
fetal large intestine 64	1.1876365	0.18862
small intestine 342	1.1892202	0.18940
melanocyte 339	1.1502073	0.18996
melanocyte 340	1.1600846	0.19088
CD14 3	1.1931255	0.21356
ES derived trophoblast 288	1.1514767	0.21854
fetal large intestine 67	1.1655593	0.22538
placenta 198	1.1473293	0.22982
keratinocyte 333	1.1129876	0.23286
ES derived trophoblast 287	1.1423482	0.24714
fetal small intestine 78	1.1245796	0.26014
keratinocyte 334	1.0988566	0.26104
fetal small intestine 74	1.1322464	0.26424
CD20 8	1.1257452	0.26510
fetal adrenal gland 24	1.1390695	0.26612
keratinocyte 336	1.0939758	0.27308
fetal small intestine 70	1.1104367	0.27862
Mobilized CD56 324	1.1358592	0.27994
fetal adrenal gland 25	1.1225567	0.28284
fetal large intestine 58	1.1115078	0.28324
fetal large intestine 55	1.1083848	0.29418
fetal thymus 256	1.1197514	0.29440
fetal large intestine 57	1.1028454	0.30020
CD56 17	1.1115481	0.30256
fetal large intestine 56	1.1059778	0.30392
placenta 196	1.1067323	0.30614
fetal large intestine 60	1.1020703	0.30998
fetal thymus 258	1.1120536	0.31056
CD4 14	1.1109299	0.31134
fetal skin 234	1.0989641	0.31322
pancreas 327	1.1127468	0.31338
fetal lung 299	1.0843862	0.31996
fetal small intestine 73	1.0942125	0.32180
keratinocyte 335	1.0700938	0.32606
placenta 201	1.0912201	0.32648
fetal small intestine 72	1.0886480	0.32796
fetal small intestine 68	1.0850662	0.33092
fetal adrenal gland 23	1.0784072	0.33098
breast 1	1.0695133	0.33406
fetal small intestine 80	1.0836785	0.33512
fetal large intestine 54	1.0850513	0.33814
fetal small intestine 69	1.0845428	0.34082
fetal adrenal gland 27	1.0809281	0.34276
fetal small intestine 79	1.0851541	0.34416
fibroblast 329	1.0685467	0.34424
placenta 197	1.0760470	0.34960
ES derived MSC 290	1.0681068	0.35128
fetal thymus 260	1.0781627	0.35720
fetal lung 119	1.0576875	0.35994
CD56 18	1.0834730	0.36156
Mobilized CD3 320	1.0892746	0.36182
CD4 13	1.0803010	0.36288
fetal small intestine 71	1.0675173	0.36376
hematopoietic stem cell 310	1.0584383	0.36380
fetal lung 140	1.0558825	0.36714
fetal large intestine 62	1.0699025	0.36726
fetal thymus 265	1.0728844	0.36904
fibroblast 330	1.0497485	0.38104
muscle 341	1.0568673	0.38426
placenta 199	1.0512435	0.38426
fetal large intestine 63	1.0564718	0.38774
ovary 326	1.0572204	0.38854
fetal lung 129	1.0454175	0.39006
CD8 21	1.0592142	0.39226
fetal lung 298	1.0464893	0.39462
fetal lung 128	1.0400440	0.39624
ES derived MSC 289	1.0434362	0.39658
fetal lung 111	1.0370274	0.40522
fetal large intestine 59	1.0432335	0.40954
fetal lung 301	1.0359198	0.41284
fetal lung 134	1.0330411	0.41630
heart 297	1.0376667	0.41904
fetal lung 125	1.0310011	0.42116
fetal adrenal gland 26	1.0354233	0.42130
CD8 19	1.0345976	0.42198
fetal large intestine 53	1.0329086	0.42226
fetal thymus 259	1.0381425	0.42336
fibroblast 332	1.0277803	0.42806
CD3 9	1.0306080	0.42880
fetal lung 120	1.0261435	0.42914
fetal lung 118	1.0238111	0.43374
stomach 283	1.0289794	0.43436
fetal renal pelvis 220	1.0220032	0.43530
fetal small intestine 77	1.0264330	0.43608
hematopoietic stem cell 314	1.0238114	0.43936
fetal lung 133	1.0196780	0.44492
fetal small intestine 76	1.0207396	0.44602
fetal lung 121	1.0170669	0.44678
CD3 11	1.0195050	0.44890
fetal lung 132	1.0149064	0.45012
fetal fibroblast 269	1.0179406	0.45022
fetal lung 127	1.0139307	0.45226
fetal kidney 91	1.0099292	0.45388
fetal stomach 244	1.0151677	0.45526
fetal lung 116	1.0119759	0.45964
breast 2	1.0135548	0.45984
fetal large intestine 65	1.0140679	0.46022
fetal lung 141	1.0113510	0.46190
fetal thymus 266	1.0094450	0.46732
fetal lung 300	1.0099267	0.46882
fetal renal pelvis 227	1.0017923	0.47210
Mobilized CD4 323	0.9991882	0.47690
fetal renal cortex 207	0.9998866	0.47718
fetal stomach 253	0.9979119	0.47792
fetal renal cortex 205	1.0002522	0.47924
fetal lung 112	1.0025220	0.48026
hematopoietic stem cell 312	1.0006914	0.48394
fetal kidney 106	0.9955219	0.48496
fetal lung 117	0.9993838	0.48586
Mobilized CD4 322	0.9868035	0.48868
fetal kidney 84	0.9897699	0.49362
hematopoietic stem cell 318	0.9885983	0.50190
CD8 20	0.9835929	0.50216
fetal kidney 95	0.9846866	0.50282
fetal kidney 100	0.9854371	0.50380
Mobilized CD3 321	0.9744960	0.50530
fetal muscle 151	0.9882039	0.50584
fibroblast 331	0.9924036	0.50874
fetal large intestine 61	0.9773255	0.51090
fetal renal pelvis 231	0.9840660	0.51094
fetal lung 109	0.9900340	0.51220
fetal kidney 99	0.9812960	0.51368
fetal kidney 85	0.9820978	0.51600
hematopoietic stem cell 308	0.9807933	0.52422
fetal fibroblast 279	0.9826246	0.52916
hematopoietic stem cell 309	0.9751103	0.53176
fetal lung 110	0.9753914	0.53288
fetal heart 43	0.9768758	0.53386
CD4 16	0.9442242	0.53778
fetal kidney 83	0.9597162	0.54060
fetal stomach 242	0.9529431	0.54284
Mobilized CD8 325	0.9500304	0.54380
fetal lung 131	0.9743839	0.54564
fetal renal pelvis 232	0.9605778	0.54632
fetal lung 137	0.9704269	0.54940
fetal renal pelvis 228	0.9544485	0.55284
fetal renal pelvis 233	0.9596636	0.55298
CD8 22	0.9375607	0.55384
fetal thymus 261	0.9573017	0.55482
fetal renal cortex 214	0.9594535	0.55500
fetal large intestine 66	0.9561700	0.55778
fetal kidney 92	0.9594939	0.55902
fetal lung 114	0.9711254	0.55946
fetal lung 115	0.9663752	0.56196
CD3 12	0.9342416	0.56338
fetal fibroblast 280	0.9693298	0.56456
fetal muscle 194	0.9624907	0.56492
fetal fibroblast 282	0.9687193	0.56514
fetal renal pelvis 217	0.9496302	0.56670
fetal lung 123	0.9579884	0.56816
CD3 10	0.9394116	0.56834
fetal thymus 264	0.9512185	0.56928
fetal lung 126	0.9634557	0.57036
fetal lung 108	0.9546251	0.57090
hematopoietic stem cell 315	0.9547506	0.57164
hematopoietic stem cell 306	0.9533793	0.57314
fetal muscle 154	0.9504837	0.57370
fetal lung 138	0.9567619	0.57400
fetal kidney 87	0.9482440	0.57622
fetal thymus 257	0.9355791	0.57758
fetal renal cortex 206	0.9487245	0.58012
fetal renal pelvis 221	0.9476056	0.58056
fetal lung 139	0.9499803	0.58218
fetal lung 130	0.9541767	0.58244
fetal renal cortex 212	0.9481181	0.58314
CD4 15	0.9008389	0.58674
fetal muscle 153	0.9391862	0.58858
fetal fibroblast 281	0.9588448	0.58962
hematopoietic stem cell 311	0.9459959	0.59040
fetal stomach 248	0.9328920	0.59134
fetal thymus 263	0.9437320	0.59154
fetal kidney 89	0.9360827	0.59222
fetal fibroblast 271	0.9577743	0.59284
fetal renal pelvis 219	0.9214517	0.59322
fetal fibroblast 270	0.9554556	0.59482
fetal muscle 169	0.9454795	0.59560
hematopoietic stem cell 305	0.9384225	0.59664
fetal muscle 166	0.9442037	0.59682
hematopoietic stem cell 307	0.9394406	0.59766
fetal muscle 182	0.9447816	0.59778
fetal muscle 179	0.9455933	0.59834
fetal stomach 243	0.9352938	0.59862
fetal heart 45	0.9447902	0.59928
hematopoietic stem cell 316	0.9322213	0.60338
fetal lung 113	0.9461913	0.60402
fetal fibroblast 274	0.9521324	0.60500
fetal muscle 180	0.9370172	0.60612
fetal stomach 240	0.9197294	0.60616
fetal fibroblast 275	0.9514797	0.60656
fetal muscle 167	0.9406374	0.60968
fetal muscle 172	0.9440943	0.61044
fetal renal cortex 202	0.9305366	0.61078
fetal fibroblast 276	0.9509701	0.61100
fetal muscle 181	0.9381071	0.61152
fetal lung 135	0.9376252	0.61206
fetal kidney 105	0.9306171	0.61322
CD19 7	0.9000722	0.61340
fetal fibroblast 273	0.9475274	0.61634
fetal kidney 104	0.9226705	0.61662
fetal renal pelvis 226	0.9284124	0.61884
fetal kidney 82	0.9128335	0.61892
fetal kidney 96	0.9262538	0.61912
fetal muscle 192	0.9290900	0.62136
fetal muscle 164	0.9281854	0.62160
fetal muscle 156	0.9278152	0.62242
fetal renal cortex 213	0.9280300	0.62248
fetal muscle 175	0.9292973	0.62532
hematopoietic stem cell 313	0.9288433	0.62572
fetal renal pelvis 225	0.9167950	0.62588
fetal thymus 262	0.8874154	0.62716
fetal renal pelvis 224	0.9221672	0.62818
fetal fibroblast 272	0.9432133	0.62958
fetal kidney 103	0.9139301	0.63250
fetal renal cortex 215	0.9218718	0.63364
fetal kidney 81	0.9267966	0.63404
fetal muscle 146	0.9246732	0.63418
fetal heart 46	0.9216754	0.63516
fetal small intestine 75	0.8585326	0.63734
CD19 5	0.8924373	0.64126
fetal heart 51	0.9223866	0.64144
fetal stomach 247	0.8891556	0.64162
fetal muscle 176	0.9273127	0.64206
hematopoietic stem cell 319	0.9078568	0.64286
fetal stomach 241	0.8965168	0.64552
fetal lung 124	0.9202264	0.64598
fetal renal pelvis 229	0.9099730	0.64656
fetal brain 31	0.9433198	0.64738
fetal kidney 101	0.9101363	0.64782
fetal fibroblast 267	0.9315147	0.64856
fetal testes 255	0.9165794	0.64878
fetal renal cortex 204	0.9151264	0.65038
fetal renal cortex 211	0.8980987	0.65070
fetal lung 136	0.9185000	0.65092
fetal fibroblast 268	0.9331265	0.65168
fetal muscle 157	0.9220259	0.65176
fetal heart 52	0.9220063	0.65226
fetal muscle 193	0.9082797	0.65296
fetal muscle 191	0.9074458	0.65588
fetal muscle 149	0.9056591	0.65762
fetal kidney 107	0.9025613	0.65838
fetal heart 42	0.9101311	0.65896
fetal renal cortex 210	0.8977481	0.65938
fetal muscle 144	0.9015700	0.65992
fetal stomach 250	0.8879157	0.66046
fetal lung 142	0.9208110	0.66154
ES cells 296	0.8898565	0.66586
fetal muscle 168	0.9010155	0.66706
fetal kidney 102	0.8888833	0.66734
fetal renal pelvis 218	0.8707843	0.66782
fetal muscle 160	0.8991276	0.66960
fetal muscle 189	0.9021129	0.66994
fetal muscle 145	0.8878447	0.67082
fetal stomach 246	0.8831869	0.67094
CD19 6	0.8621675	0.67200
fetal renal pelvis 223	0.8870100	0.67360
fetal muscle 187	0.9078099	0.67366
fetal fibroblast 278	0.9188086	0.67408
fetal muscle 188	0.9035605	0.67418
fetal muscle 174	0.8966247	0.67430
fetal kidney 88	0.8959931	0.67482
fetal muscle 173	0.8962470	0.67634
fetal renal pelvis 230	0.8796466	0.67666
fetal stomach 251	0.8838188	0.67678
fetal kidney 86	0.8656629	0.67834
fetal muscle 171	0.9033403	0.67860
ES cells 294	0.8817241	0.67926
fetal kidney 94	0.8828419	0.67998
fetal muscle 184	0.9074893	0.68012
fetal heart 49	0.9006956	0.68188
fetal kidney 90	0.8868145	0.68376
fetal muscle 178	0.8956160	0.68428
fetal muscle 148	0.8930125	0.68468
fetal muscle 183	0.9004568	0.68480
fetal muscle 159	0.8901591	0.68556
fetal fibroblast 277	0.9150661	0.68654
hematopoietic stem cell 317	0.9061523	0.68746
fetal heart 47	0.8858064	0.69104
fetal testes 254	0.8867376	0.69458
fetal stomach 252	0.8660680	0.69744
fetal muscle 150	0.8847019	0.70068
fetal stomach 249	0.8574523	0.70182
fetal renal pelvis 216	0.8725406	0.70256
fetal muscle 155	0.8750919	0.70466
fetal renal cortex 208	0.8757145	0.70536
fetal kidney 98	0.8709221	0.70816
fetal ovary 195	0.8506215	0.70950
fetal muscle 185	0.8775962	0.71018
ES derived mesoderm 285	0.9011804	0.71314
fetal muscle 158	0.8833592	0.71530
fetal heart 48	0.8825809	0.71588
fetal brain 34	0.9080655	0.72098
fetal muscle 147	0.8692754	0.72426
fetal muscle 152	0.8757985	0.72672
fetal muscle 186	0.8747604	0.72722
fetal muscle 143	0.8856765	0.72884
fetal heart 44	0.8454917	0.73260
fetal stomach 245	0.8456805	0.73470
fetal renal cortex 209	0.8541844	0.73716
fetal muscle 165	0.8734418	0.73866
fetal kidney 97	0.8558715	0.74134
iPS 303	0.8649508	0.74330
fetal lung 122	0.8628606	0.74332
iPS 304	0.8693204	0.74404
fetal renal pelvis 222	0.8444299	0.74818
fetal muscle 163	0.8566755	0.74834
iPS 302	0.8597691	0.74962
fetal muscle 177	0.8454430	0.75084
fetal heart 50	0.8554405	0.75512
fetal renal cortex 203	0.8414643	0.75652
fetal muscle 161	0.8515964	0.75698
fetal muscle 190	0.8408695	0.75860
fetal kidney 93	0.8336991	0.75922
fetal brain 35	0.8919991	0.76182
fetal muscle 162	0.8568973	0.76208
fetal muscle 170	0.8571725	0.76456
fetal spinal cord 237	0.8545456	0.77608
fetal spinal cord 236	0.8491384	0.77684
ES derived mesoderm 286	0.8682552	0.79014
fetal brain 36	0.8619545	0.79550
fetal brain 41	0.8591833	0.79550
fetal brain 38	0.8501278	0.79890
fetal brain 37	0.8568206	0.80540
ES cells 295	0.7964826	0.80576
ES cells 293	0.8036513	0.80882
ES derived NP 291	0.7875437	0.81344
fetal brain 40	0.8541723	0.81544
fetal brain 29	0.8709378	0.81950
fetal brain 39	0.8371618	0.84372
fetal spinal cord 238	0.7988451	0.85356
ES derived NP 292	0.7612184	0.85756
fetal brain 30	0.8565553	0.85920
fetal spinal cord 235	0.8006716	0.86544
fetal brain 32	0.8111605	0.88056
fetal spinal cord 239	0.7735206	0.89164
fetal brain 33	0.7779011	0.90162

Polygenic dyslipidemia

DHS sample	fold enrichment	p value
fetal adrenal gland 28	1.2816246	0.01180
fetal adrenal gland 23	1.2422113	0.03050
small intestine 342	1.2531920	0.03850
fetal adrenal gland 25	1.2330989	0.05212
fetal adrenal gland 24	1.2394854	0.06004
fetal large intestine 60	1.2255120	0.06108
fetal large intestine 56	1.2093863	0.07254
fetal large intestine 67	1.2144120	0.07274
stomach 284	1.2103257	0.08232
CD14 4	1.2039689	0.08740
fetal large intestine 58	1.1756269	0.08906
fetal adrenal gland 26	1.1815636	0.09072
fetal small intestine 74	1.1867393	0.09154
fetal thymus 256	1.1970523	0.09390
fetal large intestine 64	1.1774286	0.09408
fetal small intestine 80	1.1784567	0.09606
CD20 8	1.1748803	0.09642
fetal adrenal gland 27	1.1702162	0.10200
iPS 304	1.1560114	0.10626
fetal small intestine 78	1.1596286	0.10640
fetal small intestine 73	1.1738787	0.11396
fetal small intestine 70	1.1479783	0.11708
fetal large intestine 57	1.1528249	0.11832
ES cells 296	1.1778459	0.12336
fetal large intestine 54	1.1623470	0.12568
fetal small intestine 79	1.1703449	0.12584
fetal small intestine 69	1.1734891	0.12620
fetal large intestine 55	1.1514348	0.12662
fetal large intestine 62	1.1653834	0.13340
CD14 3	1.1736194	0.13726
fetal thymus 265	1.1687341	0.13730
fetal thymus 258	1.1672719	0.13866
ES cells 294	1.1652042	0.14008
ES derived mesoderm 286	1.1150956	0.14028
placenta 200	1.1308887	0.14060
pancreas 328	1.1608098	0.14120
fetal small intestine 76	1.1583753	0.14188
fetal large intestine 59	1.1521269	0.14906
fetal small intestine 72	1.1438496	0.14970
fetal large intestine 65	1.1431732	0.15606
muscle 341	1.1575780	0.15728
fetal thymus 260	1.1564524	0.16102
fetal small intestine 77	1.1367651	0.16672
fetal small intestine 68	1.1280083	0.16700
keratinocyte 336	1.0991862	0.16854
Mobilized CD56 324	1.1399001	0.18306
placenta 198	1.1121206	0.18412
fetal small intestine 71	1.1256637	0.18590
keratinocyte 334	1.0889084	0.19700
keratinocyte 333	1.0867917	0.19930
iPS 302	1.1064063	0.20284
CD56 17	1.1199532	0.20940
fetal large intestine 63	1.1134525	0.21052
fetal thymus 259	1.1242434	0.21488
melanocyte 338	1.0810823	0.21694
ES cells 295	1.1125956	0.22024
fetal thymus 266	1.1130011	0.22344
fetal brain 41	1.0833381	0.22356
fetal brain 34	1.0760350	0.22564
fetal brain 39	1.0783880	0.22984
fibroblast 332	1.0827358	0.23148
pancreas 327	1.1082676	0.23904
fetal thymus 264	1.1025570	0.24078
CD56 18	1.1155507	0.24616
fetal large intestine 66	1.0954960	0.25250
iPS 303	1.0824395	0.25358
fetal large intestine 53	1.0995069	0.25538
ES derived MSC 290	1.0827445	0.25828
keratinocyte 335	1.0671917	0.25842
fibroblast 329	1.0739965	0.26084
fetal brain 35	1.0612301	0.26246
ES derived MSC 289	1.0796374	0.26696
CD4 13	1.1042268	0.26714
fibroblast 331	1.0676599	0.27344
fetal brain 36	1.0599315	0.29130
ES derived mesoderm 285	1.0606119	0.29616
melanocyte 337	1.0541423	0.29716
CD4 14	1.0773634	0.29822
melanocyte 340	1.0602146	0.29946
CD3 9	1.0830075	0.30068
fibroblast 330	1.0576737	0.30900
fetal brain 29	1.0456637	0.31108
fetal brain 38	1.0567340	0.31136
CD8 21	1.0805775	0.31474
stomach 283	1.0678590	0.31836
ES derived trophoblast 288	1.0560958	0.32372
breast 1	1.0499397	0.32446
fetal brain 31	1.0409137	0.32626
fetal brain 40	1.0453631	0.32690
placenta 201	1.0566822	0.33808
fetal skin 234	1.0522322	0.34204
ES cells 293	1.0538751	0.34330
CD8 20	1.0589015	0.34442
fetal brain 30	1.0350119	0.34586
heart 297	1.0525933	0.34714
Mobilized CD3 321	1.0620126	0.35164
fetal renal pelvis 220	1.0481249	0.35432
breast 2	1.0396292	0.35470
CD8 19	1.0590348	0.35890
fetal thymus 261	1.0498826	0.36250
fetal spinal cord 236	1.0427621	0.36318
placenta 197	1.0428147	0.36542
fetal renal pelvis 231	1.0373605	0.37418
fetal brain 37	1.0330223	0.37476
fetal large intestine 61	1.0456349	0.37702
fetal renal cortex 207	1.0373427	0.37892
fetal testes 254	1.0388269	0.37912
CD3 11	1.0434389	0.38016
Mobilized CD4 322	1.0444130	0.38426
Mobilized CD4 323	1.0425235	0.38730
fetal lung 129	1.0308187	0.39054
Mobilized CD8 325	1.0403305	0.39308
fetal lung 134	1.0301708	0.39584
fetal kidney 91	1.0285008	0.39870
fetal lung 140	1.0272146	0.40146
hematopoietic stem cell 312	1.0275289	0.40598
fetal testes 255	1.0258718	0.40998
fetal thymus 262	1.0301451	0.41396
fetal renal pelvis 219	1.0269953	0.41492
fetal lung 301	1.0236436	0.41862
fetal renal cortex 214	1.0239004	0.41888
fetal kidney 106	1.0230646	0.42080
fetal renal cortex 212	1.0210641	0.42460
fetal fibroblast 269	1.0189196	0.42622
fetal thymus 257	1.0231785	0.42990
fetal lung 119	1.0183687	0.43030
CD8 22	1.0215455	0.43170
fetal kidney 95	1.0185295	0.43544
fetal spinal cord 237	1.0171313	0.43666
fetal spinal cord 238	1.0164032	0.43860
ES derived NP 291	1.0185158	0.44072
fetal ovary 195	1.0156255	0.44556
placenta 196	1.0142526	0.44650
fetal kidney 82	1.0134332	0.44808
fetal kidney 87	1.0133404	0.44860
fetal lung 300	1.0143526	0.44914
fetal renal pelvis 217	1.0121088	0.45132
fetal renal pelvis 228	1.0114584	0.45198
fetal renal pelvis 233	1.0120329	0.45222
fetal renal pelvis 225	1.0113899	0.45318
CD4 15	1.0103399	0.45566
fetal lung 132	1.0111250	0.45610
melanocyte 339	1.0090030	0.45912
fetal stomach 242	1.0089367	0.46148
hematopoietic stem cell 310	1.0098842	0.46202
fetal lung 130	1.0092774	0.46268
fetal lung 141	1.0083412	0.46288
fetal lung 125	1.0079273	0.46306
placenta 199	1.0058190	0.47086
fetal renal pelvis 227	1.0051226	0.47112
hematopoietic stem cell 315	1.0064656	0.47126
fetal lung 118	1.0053016	0.47548
fetal lung 117	1.0037274	0.47670
fetal thymus 263	1.0051128	0.47772
fetal kidney 103	1.0019095	0.48078
fetal renal cortex 205	1.0016684	0.48150
fetal spinal cord 235	1.0025532	0.48166
fetal lung 298	1.0039451	0.48246
fetal lung 109	1.0030574	0.48342
fetal renal cortex 211	1.0005360	0.48376
fetal small intestine 75	0.9909344	0.48426
fetal fibroblast 273	1.0027553	0.48524
fetal brain 32	1.0016689	0.48656
fetal kidney 89	0.9997784	0.48688
fetal lung 299	1.0014198	0.49006
CD4 16	0.9937922	0.49052
fetal renal pelvis 232	0.9974790	0.49054
fetal lung 138	0.9997903	0.49246
fetal stomach 244	0.9971921	0.49326
fetal lung 120	0.9992431	0.49390
hematopoietic stem cell 305	0.9987411	0.49584
fetal kidney 88	0.9961353	0.49602
fetal renal pelvis 224	0.9961746	0.50018
fetal renal cortex 215	0.9960807	0.50124
fetal lung 128	0.9980340	0.50220
hematopoietic stem cell 314	0.9967078	0.50492
fetal kidney 96	0.9946059	0.50506
fetal kidney 84	0.9930176	0.50636
fetal renal pelvis 218	0.9894180	0.50772
hematopoietic stem cell 316	0.9940638	0.50812
ovary 326	0.9901745	0.50814
fetal lung 123	0.9944323	0.50944
fetal stomach 253	0.9890468	0.51336
hematopoietic stem cell 308	0.9916052	0.51800
fetal kidney 83	0.9862489	0.52078
fetal fibroblast 279	0.9925213	0.52112
CD3 10	0.9860530	0.52206
fetal renal cortex 206	0.9884483	0.52212
fetal spinal cord 239	0.9886763	0.52490
fetal lung 133	0.9909902	0.52528
fetal lung 136	0.9892053	0.52598
fetal kidney 105	0.9866037	0.52612
ES derived trophoblast 287	0.9891153	0.52614
fetal stomach 247	0.9808668	0.52642
fetal muscle 194	0.9902648	0.52654
fetal renal pelvis 221	0.9872359	0.52808
hematopoietic stem cell 318	0.9854521	0.52892
ES derived NP 292	0.9850962	0.52958
fetal lung 127	0.9883096	0.53008
fetal renal cortex 213	0.9874453	0.53056
fetal renal pelvis 229	0.9850535	0.53090
fetal kidney 100	0.9841533	0.53382
fetal lung 112	0.9861567	0.53864
CD3 12	0.9739947	0.54024
fetal kidney 99	0.9822623	0.54196
fetal kidney 104	0.9804637	0.54406
fetal lung 111	0.9854892	0.54502
fetal fibroblast 280	0.9863431	0.54562
fetal stomach 250	0.9755325	0.54698
fetal muscle 193	0.9801077	0.54700
fetal fibroblast 274	0.9852881	0.54770
hematopoietic stem cell 309	0.9825529	0.54774
hematopoietic stem cell 307	0.9809925	0.54912
fetal lung 108	0.9799498	0.55416
fetal kidney 86	0.9693803	0.55680
fetal kidney 92	0.9771845	0.55796
fetal renal cortex 204	0.9782504	0.55858
fetal fibroblast 270	0.9809626	0.55908
fetal kidney 85	0.9796490	0.56010
fetal kidney 102	0.9726197	0.56036
fetal stomach 248	0.9712552	0.56058
fetal lung 121	0.9803847	0.56284
fetal stomach 252	0.9709341	0.56342
hematopoietic stem cell 306	0.9759050	0.56500
fetal muscle 153	0.9734804	0.56518
fetal fibroblast 282	0.9810525	0.56520
fetal kidney 101	0.9749443	0.56556
fetal muscle 175	0.9750579	0.56644
CD19 5	0.9675296	0.56652
fetal lung 116	0.9800932	0.56804
hematopoietic stem cell 313	0.9763715	0.56974
Mobilized CD3 320	0.9644955	0.57116
fetal renal pelvis 216	0.9674591	0.57714
fetal lung 110	0.9721652	0.57932
fetal renal pelvis 223	0.9644181	0.58194
CD19 6	0.9559076	0.58646
fetal fibroblast 268	0.9733813	0.58970
fetal kidney 97	0.9632850	0.59100
fetal muscle 164	0.9660390	0.59134
fetal lung 113	0.9725386	0.59140
hematopoietic stem cell 317	0.9722427	0.59162
fetal lung 135	0.9691825	0.59368
fetal lung 124	0.9683002	0.59570
fetal kidney 107	0.9617299	0.59724
fetal lung 139	0.9649006	0.60330
fetal heart 45	0.9609688	0.60424
CD19 7	0.9462422	0.60692
fetal heart 46	0.9585009	0.60804
fetal renal cortex 210	0.9551373	0.60990
fetal renal pelvis 226	0.9596502	0.61042
fetal muscle 185	0.9578011	0.61278
fetal brain 33	0.9634717	0.61322
hematopoietic stem cell 311	0.9602573	0.61418
fetal renal cortex 208	0.9545425	0.61680
fetal lung 115	0.9640612	0.61774
fetal stomach 241	0.9472443	0.61780
fetal lung 142	0.9640584	0.62030
fetal lung 126	0.9636049	0.62056
fetal kidney 90	0.9533822	0.62108
fetal kidney 93	0.9503847	0.62176
fetal stomach 240	0.9443946	0.62192
fetal renal pelvis 222	0.9506036	0.62428
fetal lung 137	0.9607668	0.62430
fetal heart 52	0.9576594	0.62458
fetal heart 49	0.9541916	0.62538
fetal muscle 174	0.9529369	0.62910
fetal stomach 246	0.9418909	0.63442
fetal renal pelvis 230	0.9432177	0.63606
fetal kidney 98	0.9469429	0.63644
fetal lung 114	0.9616765	0.63660
fetal muscle 173	0.9510644	0.63676
hematopoietic stem cell 319	0.9489318	0.63690
fetal muscle 191	0.9497679	0.63732
fetal renal cortex 202	0.9496867	0.63800
fetal stomach 249	0.9372176	0.64040
fetal muscle 182	0.9505138	0.64400
fetal lung 122	0.9498431	0.64574
fetal muscle 169	0.9475488	0.64638
fetal muscle 184	0.9510609	0.64682
fetal muscle 171	0.9490246	0.65196
fetal muscle 151	0.9416726	0.65332
fetal lung 131	0.9565638	0.65342
fetal fibroblast 281	0.9550911	0.65490
fetal muscle 155	0.9412497	0.65522
fetal muscle 157	0.9487750	0.65690
fetal stomach 243	0.9408235	0.65812
fetal kidney 81	0.9450139	0.65882
fetal muscle 145	0.9360098	0.65986
fetal heart 43	0.9454868	0.66120
fetal muscle 154	0.9385338	0.66474
fetal heart 44	0.9293228	0.66496
fetal fibroblast 272	0.9519498	0.66678
fetal muscle 189	0.9405916	0.66760
fetal muscle 144	0.9371487	0.67046
fetal muscle 150	0.9364890	0.67200
fetal muscle 187	0.9421432	0.67324
fetal fibroblast 278	0.9465301	0.67776
fetal fibroblast 271	0.9471607	0.68222
fetal muscle 165	0.9378914	0.68510
fetal fibroblast 277	0.9440871	0.68590
fetal stomach 251	0.9243868	0.68646
fetal muscle 149	0.9316617	0.68664
fetal kidney 94	0.9258572	0.68666
fetal muscle 188	0.9331382	0.69038
fetal muscle 148	0.9305119	0.69368
fetal renal cortex 209	0.9257597	0.69506
fetal heart 42	0.9294086	0.69642
fetal muscle 192	0.9294532	0.70224
fetal muscle 159	0.9250055	0.70268
fetal muscle 168	0.9272732	0.70394
fetal fibroblast 276	0.9401830	0.70512
fetal stomach 245	0.9158408	0.70558
fetal muscle 146	0.9280304	0.71002
fetal renal cortex 203	0.9205526	0.71208
fetal muscle 166	0.9255074	0.71558
fetal muscle 160	0.9213531	0.71638
fetal muscle 167	0.9267238	0.72038
fetal muscle 190	0.9146866	0.72168
fetal fibroblast 267	0.9304427	0.72228
fetal muscle 183	0.9211303	0.72772
fetal heart 47	0.9080072	0.72848
fetal heart 50	0.9160080	0.72992
fetal heart 51	0.9181354	0.73012
fetal muscle 156	0.9199270	0.73220
fetal fibroblast 275	0.9325220	0.73278
fetal muscle 179	0.9163817	0.73944
fetal muscle 158	0.9179415	0.74062
fetal muscle 172	0.9226857	0.74146
fetal muscle 180	0.9100377	0.74512
fetal muscle 152	0.9157121	0.74698
fetal heart 48	0.9095057	0.75118
fetal muscle 178	0.9086622	0.75140
fetal muscle 147	0.9060349	0.75496
fetal muscle 163	0.9039878	0.75610
fetal muscle 162	0.9068381	0.76742
fetal muscle 170	0.9046562	0.77422
fetal muscle 177	0.8872658	0.78212
fetal muscle 143	0.9039233	0.78846
fetal muscle 181	0.8957998	0.79134
fetal muscle 186	0.8891959	0.80602
fetal muscle 161	0.8822152	0.81094
fetal muscle 176	0.8881501	0.82422

Primary tooth eruption

DHS sample	fold enrichment	p value
fetal fibroblast 276	1.3949928	0.02154
fetal fibroblast 267	1.3959804	0.02328
fetal kidney 85	1.3921424	0.02362
fetal lung 141	1.3593608	0.02642
fetal fibroblast 275	1.3633321	0.03166
fetal skin 234	1.4112802	0.03574
fetal fibroblast 278	1.3640866	0.03604
fetal fibroblast 281	1.3466025	0.03662
fetal lung 111	1.3551802	0.03686
fetal fibroblast 277	1.3635955	0.03798
fetal fibroblast 268	1.3340139	0.03902
fetal heart 47	1.4356327	0.04182
fetal lung 133	1.3164143	0.04428
fetal lung 114	1.3025119	0.04708
fetal fibroblast 273	1.3149329	0.05276
fetal fibroblast 282	1.2993768	0.05522
fibroblast 331	1.3205031	0.05664
keratinocyte 334	1.2767272	0.05966
fetal fibroblast 272	1.2906739	0.06578
fetal fibroblast 271	1.2931226	0.06620
fibroblast 332	1.2913699	0.07228
fetal heart 45	1.3190468	0.07840
fibroblast 329	1.2842725	0.08242
fetal fibroblast 274	1.2717424	0.08252
ES derived MSC 289	1.2901601	0.09616
fetal lung 300	1.2664229	0.10064
fetal heart 49	1.2882954	0.10126
fetal lung 121	1.2289467	0.10266
fibroblast 330	1.2508496	0.10856
melanocyte 338	1.2151939	0.11854
keratinocyte 333	1.2054449	0.11896
fetal small intestine 70	1.2561089	0.12124
fetal fibroblast 279	1.2248468	0.12284
fetal fibroblast 280	1.2202918	0.12524
fetal renal pelvis 231	1.2432599	0.13178
fetal kidney 91	1.2307990	0.13432
fetal lung 118	1.1893622	0.13896
fetal muscle 172	1.2057924	0.13986
fetal small intestine 80	1.2532384	0.14044
fetal lung 129	1.2048736	0.14302
fetal muscle 167	1.2134208	0.14626
fetal fibroblast 270	1.2006627	0.14940
fetal kidney 103	1.2379543	0.14984
fetal lung 299	1.2235787	0.15096
ES derived MSC 290	1.2256557	0.15212
fetal heart 43	1.2125436	0.15584
fetal fibroblast 269	1.1899307	0.15616
fetal lung 298	1.2199568	0.15684
fetal heart 42	1.2152308	0.16422
fetal lung 140	1.1804423	0.16592
fetal heart 52	1.1989714	0.16782
fetal heart 48	1.2073905	0.17078
fetal kidney 92	1.2000658	0.17410
fetal large intestine 57	1.2088915	0.17432
fetal lung 117	1.1697547	0.18014
fetal lung 119	1.1610168	0.18026
fetal lung 116	1.1616638	0.18174
fetal renal cortex 213	1.1905062	0.18338
fetal large intestine 67	1.2148234	0.18536
keratinocyte 335	1.1586001	0.18550
fetal heart 46	1.1988918	0.18712
fetal muscle 166	1.1768184	0.18876
fetal muscle 158	1.1746862	0.19200
ES derived mesoderm 285	1.1510681	0.19806
ES derived trophoblast 287	1.1897300	0.20076
fetal muscle 155	1.1825006	0.20162
fetal kidney 106	1.1795056	0.20264
fetal kidney 81	1.1630110	0.20268
CD20 8	1.1919379	0.20398
fetal renal cortex 207	1.1843956	0.20552
fetal lung 131	1.1420927	0.20582
keratinocyte 336	1.1451991	0.20612
fetal lung 112	1.1570886	0.20628
fetal large intestine 55	1.1838458	0.20714
fetal muscle 192	1.1636449	0.20880
fetal renal pelvis 226	1.1755963	0.20946
fetal lung 301	1.1688665	0.21036
melanocyte 337	1.1399042	0.21048
fetal heart 51	1.1690326	0.21412
fetal muscle 176	1.1549475	0.21492
fetal small intestine 78	1.1727353	0.21614
fetal kidney 99	1.1706501	0.21822
fetal lung 142	1.1408964	0.21874
fetal large intestine 58	1.1714919	0.22006
fetal kidney 95	1.1660058	0.22482
fetal muscle 181	1.1536653	0.22608
fetal muscle 180	1.1618322	0.22738
fetal heart 44	1.1876525	0.22836
fetal muscle 178	1.1565392	0.22928
fetal muscle 143	1.1397215	0.23584
fetal small intestine 74	1.1663193	0.23762
fetal lung 128	1.1221813	0.23776
fetal lung 124	1.1336945	0.23828
fetal lung 134	1.1357706	0.23870
fetal large intestine 60	1.1597331	0.24632
fetal renal pelvis 220	1.1508267	0.24692
fetal lung 120	1.1251529	0.25260
fetal muscle 165	1.1290808	0.25560
fetal lung 127	1.1262765	0.25694
fetal lung 130	1.1212647	0.25696
fetal lung 132	1.1214013	0.25856
fetal muscle 184	1.1238735	0.25984
fetal muscle 186	1.1268293	0.25990
fetal muscle 169	1.1282805	0.26042
fetal muscle 171	1.1242593	0.26062
fetal large intestine 56	1.1459930	0.26170
fetal lung 137	1.1153272	0.26540
fetal muscle 164	1.1297585	0.26608
fetal renal pelvis 221	1.1289934	0.26856
fetal muscle 185	1.1294113	0.27258
fetal muscle 194	1.1096346	0.27268
fetal kidney 87	1.1248324	0.27468
fetal muscle 161	1.1192547	0.27636
fetal kidney 100	1.1251916	0.27664
fetal muscle 187	1.1133794	0.27726
fetal muscle 151	1.1236456	0.27756
fetal muscle 170	1.1143530	0.27956
fetal large intestine 54	1.1363048	0.28028
fetal small intestine 73	1.1343088	0.28272
fetal large intestine 64	1.1301295	0.28290
fetal kidney 89	1.1232755	0.28334
fetal lung 110	1.1090646	0.28414
fetal small intestine 68	1.1235729	0.28562
fetal kidney 96	1.1102485	0.29044
fetal lung 109	1.0918404	0.29264
fetal lung 126	1.0949308	0.29338
fetal muscle 179	1.1047227	0.29836
fetal renal pelvis 227	1.1097482	0.29924
fetal muscle 163	1.1073791	0.30148
fetal muscle 149	1.1074205	0.30290
fetal muscle 173	1.1031919	0.30360
fetal lung 123	1.0952824	0.30574
fetal muscle 152	1.0979495	0.30656
fetal thymus 263	1.1036173	0.30666
fetal muscle 157	1.0908185	0.30992
fetal adrenal gland 28	1.0926215	0.31138
fetal muscle 182	1.0942277	0.31292
fetal heart 50	1.0999478	0.31552
fetal muscle 159	1.1006509	0.31618
fetal muscle 183	1.0884225	0.31634
fetal lung 113	1.0825999	0.31792
fetal muscle 191	1.0931341	0.32078
fetal muscle 189	1.0892554	0.32152
fetal muscle 175	1.0891256	0.32158
fetal kidney 84	1.0956071	0.32408
fetal muscle 188	1.0858813	0.32556
fetal renal cortex 205	1.0927654	0.32558
fetal renal pelvis 217	1.0957833	0.32892
fetal lung 136	1.0799992	0.33408
fetal lung 135	1.0788073	0.33568
fetal small intestine 76	1.0984656	0.33576
fetal muscle 162	1.0792409	0.33748
fetal renal cortex 215	1.0789307	0.33772
breast 2	1.0774450	0.33772
fetal large intestine 63	1.0936653	0.33786
fetal small intestine 72	1.0909623	0.33886
fetal muscle 168	1.0819502	0.33908
fetal renal pelvis 224	1.0782788	0.34008
fetal lung 139	1.0758334	0.34146
fetal muscle 150	1.0792234	0.34708
fetal muscle 193	1.0764483	0.34810
fetal small intestine 79	1.0862818	0.35168
melanocyte 339	1.0670017	0.35194
breast 1	1.0697252	0.35270
fetal renal pelvis 233	1.0748340	0.35406
fetal muscle 160	1.0718915	0.35568
fetal lung 138	1.0666380	0.35768
fetal kidney 98	1.0709853	0.35870
fetal small intestine 77	1.0797781	0.35912
fetal muscle 154	1.0712092	0.36042
fetal renal pelvis 228	1.0734144	0.36170
fetal lung 115	1.0615843	0.36188
fetal large intestine 62	1.0790925	0.36204
hematopoietic stem cell 314	1.0715935	0.36210
fetal renal cortex 206	1.0666492	0.36386
fetal renal cortex 214	1.0694849	0.36548
fetal muscle 145	1.0656652	0.36916
fetal muscle 156	1.0605557	0.37018
ES derived trophoblast 288	1.0627497	0.37036
fetal lung 125	1.0607191	0.37108
fetal renal cortex 211	1.0640205	0.37248
fetal muscle 147	1.0606087	0.37610
fetal renal pelvis 229	1.0579268	0.37644
fetal lung 108	1.0545015	0.38204
fetal muscle 144	1.0573384	0.38312
fetal kidney 104	1.0523737	0.39340
fetal large intestine 65	1.0563682	0.39430
fetal muscle 146	1.0497078	0.39486
fetal kidney 94	1.0521535	0.39494
fetal renal cortex 202	1.0480649	0.39602
fetal stomach 243	1.0509827	0.39608
hematopoietic stem cell 310	1.0483342	0.39632
fetal small intestine 71	1.0524354	0.39806
fetal adrenal gland 26	1.0482430	0.39868
fetal muscle 153	1.0461807	0.40360
fetal kidney 88	1.0424826	0.40418
fetal renal cortex 212	1.0408745	0.40474
fetal stomach 248	1.0467219	0.40838
fetal large intestine 59	1.0479741	0.40854
fetal kidney 105	1.0359943	0.41352
fetal large intestine 66	1.0422141	0.41432
fetal renal pelvis 232	1.0385795	0.41536
fetal muscle 174	1.0369601	0.41584
fetal kidney 107	1.0345750	0.41816
fetal renal pelvis 230	1.0347983	0.41890
fetal kidney 101	1.0347420	0.42006
fetal small intestine 69	1.0376346	0.42358
fetal testes 255	1.0329293	0.42436
ES derived mesoderm 286	1.0289437	0.42488
fetal thymus 256	1.0295661	0.42998
fetal muscle 177	1.0278421	0.43282
fetal kidney 102	1.0207919	0.43886
fetal renal pelvis 216	1.0214520	0.43928
fetal renal cortex 203	1.0194694	0.44432
fetal large intestine 53	1.0221840	0.44616
fetal renal pelvis 223	1.0157478	0.45126
fetal renal cortex 204	1.0159444	0.45312
fetal kidney 83	1.0156453	0.45444
fetal lung 122	1.0148696	0.45554
fetal thymus 259	1.0122400	0.45866
fetal muscle 190	1.0099742	0.46614
fetal thymus 258	1.0027787	0.47454
fetal stomach 251	1.0011001	0.47940
fetal kidney 97	0.9978958	0.48120
fetal kidney 86	0.9928958	0.48404
fetal kidney 82	0.9932770	0.48424
fetal muscle 148	0.9992060	0.48578
fetal brain 37	1.0006569	0.48670
ovary 326	0.9949239	0.48672
fetal thymus 261	0.9877630	0.49034
fetal brain 35	0.9986271	0.49248
hematopoietic stem cell 308	0.9964078	0.49330
fetal renal cortex 208	0.9829122	0.50340
CD14 4	0.9863344	0.50400
fetal renal cortex 209	0.9817284	0.50866
fetal stomach 252	0.9812359	0.50984
fetal thymus 257	0.9709125	0.51324
melanocyte 340	0.9875127	0.51482
hematopoietic stem cell 305	0.9801987	0.51824
hematopoietic stem cell 317	0.9844316	0.51858
fetal renal cortex 210	0.9731378	0.52160
fetal thymus 264	0.9704592	0.52186
fetal adrenal gland 27	0.9776891	0.52246
fetal kidney 93	0.9737044	0.52250
muscle 341	0.9681093	0.52766
fetal adrenal gland 23	0.9765858	0.52940
fetal stomach 244	0.9694139	0.53254
fetal renal pelvis 225	0.9659945	0.53336
CD14 3	0.9665652	0.53488
fetal renal pelvis 222	0.9631488	0.53752
fetal thymus 260	0.9544210	0.54254
fetal thymus 265	0.9547771	0.54418
fetal testes 254	0.9644377	0.54600
fetal stomach 240	0.9549983	0.54756
fetal ovary 195	0.9504714	0.55218
fetal adrenal gland 25	0.9599173	0.55292
fetal renal pelvis 219	0.9439380	0.55420
hematopoietic stem cell 319	0.9478230	0.56306
fetal kidney 90	0.9472301	0.56350
fetal stomach 253	0.9388146	0.56938
fetal adrenal gland 24	0.9408741	0.57156
heart 297	0.9425920	0.57632
fetal renal pelvis 218	0.9259556	0.57748
fetal stomach 245	0.9372719	0.57780
fetal stomach 250	0.9331916	0.58146
ES cells 295	0.9349000	0.58790
fetal stomach 246	0.9248991	0.59230
hematopoietic stem cell 309	0.9392335	0.59354
fetal stomach 242	0.9091499	0.59500
fetal brain 34	0.9565860	0.59522
fetal stomach 247	0.9082712	0.60078
fetal stomach 249	0.9165437	0.60572
fetal stomach 241	0.9168840	0.60638
iPS 303	0.9365132	0.60802
fetal thymus 266	0.9092598	0.61058
small intestine 342	0.9087442	0.62584
ES cells 294	0.8989317	0.63314
fetal brain 40	0.9385942	0.63740
hematopoietic stem cell 311	0.9106708	0.64048
ES derived NP 292	0.9002186	0.64442
CD19 6	0.8734930	0.64508
stomach 284	0.8888564	0.65176
hematopoietic stem cell 313	0.9068998	0.65456
iPS 304	0.9117276	0.66040
hematopoietic stem cell 318	0.8846126	0.67016
stomach 283	0.8686260	0.67652
hematopoietic stem cell 306	0.8841591	0.68086
ES derived NP 291	0.8727357	0.68666
fetal brain 30	0.9298101	0.68992
fetal brain 36	0.9097970	0.69042
ES cells 296	0.8705317	0.69232
placenta 200	0.8896334	0.69322
hematopoietic stem cell 312	0.8800696	0.70022
hematopoietic stem cell 315	0.8694248	0.70482
fetal spinal cord 236	0.8768270	0.71488
CD19 5	0.8224859	0.71660
fetal brain 41	0.8937658	0.72208
fetal spinal cord 235	0.8784533	0.73054
fetal thymus 262	0.7864187	0.73848
CD19 7	0.7985519	0.74006
iPS 302	0.8575324	0.74102
hematopoietic stem cell 307	0.8471359	0.74200
hematopoietic stem cell 316	0.8360737	0.74482
placenta 198	0.8500576	0.74918
CD3 10	0.7977522	0.75932
fetal spinal cord 237	0.8554898	0.76900
Mobilized CD56 324	0.7944120	0.77288
placenta 201	0.8103241	0.77374
pancreas 328	0.8034866	0.78284
fetal brain 29	0.8871914	0.78486
fetal brain 39	0.8707448	0.78526
fetal brain 31	0.8867588	0.78828
ES cells 293	0.8113313	0.78902
Mobilized CD4 323	0.7533795	0.79270
fetal spinal cord 238	0.8363776	0.79626
CD8 19	0.7283415	0.79712
CD56 18	0.7448904	0.79938
fetal large intestine 61	0.7583885	0.80514
fetal brain 38	0.8437234	0.80652
pancreas 327	0.7700383	0.81206
placenta 196	0.7862753	0.81538
fetal brain 32	0.8491536	0.82140
CD4 13	0.7268788	0.82466
CD4 14	0.7439826	0.82894
placenta 199	0.7796866	0.83728
CD8 21	0.6951375	0.84540
fetal spinal cord 239	0.7994115	0.84630
CD3 9	0.6976717	0.85406
Mobilized CD8 325	0.6752510	0.86724
Mobilized CD3 320	0.6763983	0.86744
placenta 197	0.7366996	0.87042
CD4 16	0.6632539	0.87812
Mobilized CD4 322	0.6542680	0.88024
CD4 15	0.6118125	0.88344
CD56 17	0.6834354	0.88870
fetal small intestine 75	0.6203566	0.88936
CD3 11	0.6636163	0.89482
fetal brain 33	0.7762300	0.89792
Mobilized CD3 321	0.6205274	0.89940
CD3 12	0.6155096	0.90784
CD8 20	0.6179471	0.92486
CD8 22	0.5677076	0.93944

Thyroid−related traits

DHS sample	fold enrichment	p value
fetal brain 37	1.3316771	0.01004
fetal lung 119	1.3505498	0.01016
fetal lung 141	1.3420988	0.01210
fetal muscle 148	1.4258109	0.01390
fetal spinal cord 237	1.3851041	0.01520
fetal muscle 179	1.4128378	0.01536
fetal muscle 155	1.4333783	0.01592
fetal lung 131	1.3119948	0.01594
fetal muscle 157	1.3765890	0.01610
fetal muscle 180	1.4177667	0.01644
fetal lung 129	1.3462283	0.01694
fetal lung 128	1.3153903	0.01722
fetal lung 142	1.3343358	0.01760
fetal muscle 169	1.4054470	0.01792
fetal muscle 165	1.3711553	0.01824
fetal lung 113	1.3263495	0.01952
fetal muscle 167	1.3833951	0.01960
fetal muscle 186	1.3789605	0.01976
fetal muscle 159	1.4133715	0.02026
fetal lung 111	1.3219095	0.02064
fetal brain 36	1.3010841	0.02178
fetal muscle 150	1.3935041	0.02204
fetal muscle 183	1.3550055	0.02266
fetal muscle 194	1.3232334	0.02270
fetal muscle 161	1.3823818	0.02334
fetal muscle 181	1.3783736	0.02362
heart 297	1.4363358	0.02400
fetal lung 110	1.3456813	0.02418
fetal brain 35	1.2517363	0.02420
fetal muscle 153	1.3962183	0.02476
fetal muscle 193	1.3846298	0.02496
fetal muscle 182	1.3656568	0.02514
fetal muscle 192	1.3577136	0.02514
fetal muscle 168	1.3782490	0.02566
fetal muscle 178	1.3762432	0.02590
fetal muscle 151	1.3878918	0.02678
fetal muscle 147	1.3646380	0.02690
fetal muscle 149	1.3844237	0.02708
fetal muscle 164	1.3670462	0.02708
fetal lung 109	1.2847918	0.02722
fetal muscle 187	1.3407432	0.02772
fetal lung 300	1.3290914	0.02894
fetal muscle 176	1.3383728	0.02904
fetal muscle 146	1.3477120	0.02940
fetal muscle 163	1.3561258	0.03044
fetal muscle 174	1.3590160	0.03118
fetal muscle 144	1.3733657	0.03144
fetal lung 133	1.2858523	0.03164
fetal lung 125	1.3212991	0.03194
fetal muscle 190	1.3731510	0.03242
fetal lung 114	1.2712668	0.03348
fetal brain 32	1.2761672	0.03390
fetal thymus 263	1.3569065	0.03392
fetal lung 138	1.3056893	0.03402
fetal lung 121	1.2738531	0.03466
fetal muscle 191	1.3515883	0.03494
fetal muscle 143	1.3109466	0.03554
fetal lung 139	1.3091777	0.03588
fetal muscle 177	1.3582569	0.03604
fetal muscle 156	1.3303498	0.03670
fetal muscle 166	1.3308465	0.03678
fetal muscle 162	1.3233781	0.03684
fetal brain 39	1.2493980	0.03728
fetal lung 116	1.2596283	0.03756
fetal lung 137	1.2843318	0.03826
fetal brain 38	1.2746433	0.03916
fetal muscle 158	1.3160373	0.03972
fetal lung 134	1.3066017	0.03998
fetal brain 30	1.1957179	0.04028
fetal muscle 189	1.3228680	0.04048
fetal brain 34	1.2314467	0.04076
fetal lung 135	1.2974357	0.04080
fetal spinal cord 235	1.3104916	0.04138
fetal muscle 185	1.3460247	0.04162
fetal lung 115	1.2687188	0.04240
fetal lung 301	1.3034490	0.04252
fetal stomach 245	1.3870623	0.04262
fetal lung 120	1.2817991	0.04328
fetal lung 124	1.2814654	0.04474
fetal lung 140	1.2638869	0.04474
fetal muscle 145	1.3493795	0.04578
fetal lung 136	1.2774130	0.04688
fetal brain 41	1.2488524	0.04736
fetal muscle 188	1.2996811	0.04796
fetal lung 123	1.2765669	0.04998
fetal large intestine 59	1.3565506	0.05008
fetal lung 117	1.2563123	0.05014
fetal small intestine 76	1.3610880	0.05016
fibroblast 332	1.2700774	0.05070
fetal large intestine 63	1.3537300	0.05124
fetal muscle 154	1.3203391	0.05148
pancreas 327	1.3580817	0.05378
fetal stomach 251	1.3415982	0.05498
fetal small intestine 79	1.3526262	0.05530
fetal large intestine 67	1.3253588	0.05558
fetal muscle 170	1.2808179	0.05622
fetal muscle 172	1.2570540	0.05642
fetal small intestine 77	1.3275657	0.05664
fetal stomach 243	1.3217866	0.05672
fetal lung 122	1.2816246	0.05696
fetal muscle 173	1.2949124	0.05848
fetal lung 127	1.2682621	0.05874
stomach 283	1.3460981	0.05930
fetal brain 29	1.1800440	0.05948
fetal lung 108	1.2777272	0.05958
ovary 326	1.3677386	0.05966
fibroblast 329	1.2579992	0.05978
fetal muscle 152	1.2759183	0.06068
fetal lung 126	1.2364629	0.06074
fetal lung 299	1.2740693	0.06120
fetal lung 112	1.2505154	0.06222
fetal large intestine 64	1.3011903	0.06240
fetal large intestine 62	1.3320944	0.06374
fetal stomach 250	1.3504559	0.06416
fetal muscle 160	1.2852814	0.06434
fetal muscle 171	1.2701438	0.06450
fetal large intestine 65	1.3167673	0.06596
fetal muscle 175	1.2777959	0.06622
fetal stomach 244	1.3155857	0.06646
fetal large intestine 54	1.3059628	0.06716
fetal lung 130	1.2392537	0.06816
fibroblast 331	1.2466544	0.06824
fibroblast 330	1.2499346	0.06852
fetal small intestine 69	1.3246479	0.06932
fetal brain 33	1.2392236	0.07016
fetal muscle 184	1.2567680	0.07038
fetal kidney 105	1.2752882	0.07040
fetal spinal cord 236	1.2775399	0.07042
ES derived MSC 290	1.2704465	0.07086
fetal lung 298	1.2625267	0.07128
fetal renal cortex 206	1.2726634	0.07302
stomach 284	1.3078807	0.07374
fetal small intestine 80	1.2778878	0.07382
fetal lung 132	1.2363209	0.07392
fetal stomach 248	1.3045588	0.07570
fetal large intestine 66	1.3026014	0.07582
fetal small intestine 73	1.2941372	0.07650
fetal small intestine 72	1.2830535	0.07782
fetal small intestine 68	1.2692521	0.07942
fetal lung 118	1.2035499	0.07982
keratinocyte 334	1.1985875	0.08040
fetal small intestine 74	1.2742132	0.08092
fetal large intestine 58	1.2543070	0.08150
fetal renal cortex 208	1.2748088	0.08468
keratinocyte 335	1.1981423	0.08490
fetal brain 40	1.1819308	0.08550
fetal small intestine 78	1.2451507	0.08612
fetal small intestine 71	1.2789354	0.08708
breast 2	1.2121488	0.08720
fetal stomach 252	1.2841046	0.08748
fetal large intestine 56	1.2678765	0.08836
fetal kidney 101	1.2550403	0.08914
CD20 8	1.2539143	0.09014
fetal fibroblast 271	1.2053360	0.09106
fetal renal pelvis 216	1.2643820	0.09122
fetal stomach 253	1.3011888	0.09272
fetal kidney 81	1.2306537	0.09314
small intestine 342	1.2659643	0.09458
fetal small intestine 70	1.2263270	0.09950
fetal adrenal gland 26	1.2406928	0.09960
fetal kidney 90	1.2487896	0.10180
fetal adrenal gland 23	1.2190529	0.10256
fetal fibroblast 278	1.2049509	0.10298
fetal renal cortex 204	1.2254623	0.10302
fetal kidney 93	1.2580305	0.10338
fetal large intestine 60	1.2465793	0.10604
keratinocyte 333	1.1734490	0.10640
fetal brain 31	1.1414094	0.10736
fetal stomach 246	1.2737767	0.10770
fetal fibroblast 269	1.1866025	0.10776
fetal stomach 247	1.3096339	0.10810
fetal large intestine 57	1.2255932	0.11110
fetal stomach 249	1.2721441	0.11248
pancreas 328	1.2551879	0.11300
fetal fibroblast 280	1.1847621	0.11328
fetal large intestine 53	1.2635418	0.11558
fetal fibroblast 273	1.1837099	0.11576
fetal fibroblast 270	1.1855704	0.11582
fetal fibroblast 274	1.1851883	0.11582
fetal large intestine 55	1.2239723	0.11586
fetal fibroblast 277	1.1911054	0.11610
fetal fibroblast 272	1.1842356	0.11796
fetal stomach 241	1.2573825	0.12056
fetal heart 43	1.2088719	0.12248
fetal kidney 107	1.2200988	0.12276
fetal stomach 240	1.2615613	0.12352
breast 1	1.1782603	0.12468
fetal renal pelvis 224	1.2043483	0.12542
fetal fibroblast 282	1.1702066	0.12572
fetal spinal cord 239	1.1984399	0.12612
fetal fibroblast 279	1.1769251	0.12718
fetal renal cortex 214	1.2138595	0.12726
fetal renal cortex 203	1.2140901	0.12992
fetal kidney 97	1.2135626	0.13022
fetal fibroblast 276	1.1705933	0.13070
fetal renal cortex 212	1.2011046	0.13216
fetal renal pelvis 229	1.2113026	0.13246
fetal renal cortex 205	1.2067227	0.13316
fetal spinal cord 238	1.1926803	0.13338
fetal renal pelvis 222	1.2175757	0.13412
fetal renal cortex 210	1.2216747	0.13430
fetal adrenal gland 24	1.2303244	0.13448
fetal renal pelvis 231	1.1934949	0.13520
fetal testes 254	1.2093986	0.13634
fetal renal pelvis 228	1.2183068	0.13902
fetal renal cortex 211	1.2133421	0.14072
fetal heart 52	1.1841705	0.14154
fetal stomach 242	1.2679928	0.14280
fetal renal pelvis 223	1.2154601	0.14380
fetal heart 42	1.2028008	0.14386
fetal renal pelvis 219	1.2435505	0.14780
fetal fibroblast 275	1.1560949	0.14912
fetal kidney 88	1.1877433	0.14964
fetal renal pelvis 227	1.1932106	0.14966
fetal renal pelvis 232	1.2032593	0.15020
fetal renal pelvis 225	1.2025196	0.15148
fetal fibroblast 281	1.1551240	0.15244
ES derived MSC 289	1.1826808	0.15346
ES cells 293	1.1958042	0.15386
fetal renal pelvis 233	1.1911718	0.15398
fetal kidney 98	1.1912471	0.15706
fetal kidney 82	1.2080302	0.15712
fetal renal pelvis 218	1.2296400	0.15878
fetal heart 50	1.1892777	0.15882
fetal renal cortex 215	1.1743551	0.16106
fetal adrenal gland 27	1.1850311	0.16160
fetal kidney 86	1.2153642	0.16514
fetal renal cortex 213	1.1708203	0.16596
fetal testes 255	1.1656689	0.16808
fetal kidney 87	1.1730708	0.16902
fetal renal cortex 209	1.1817610	0.17054
fetal heart 51	1.1769237	0.17086
fetal kidney 96	1.1685009	0.17280
keratinocyte 336	1.1316231	0.17536
fetal renal pelvis 226	1.1685437	0.17652
fetal renal pelvis 217	1.1781354	0.17744
fetal kidney 106	1.1675059	0.17824
fetal heart 48	1.1699994	0.18080
fetal kidney 92	1.1573545	0.18134
fetal kidney 89	1.1689377	0.18210
fetal kidney 84	1.1711430	0.18328
ES derived NP 291	1.1810156	0.18532
fetal renal pelvis 220	1.1677683	0.18800
fetal kidney 104	1.1664715	0.18960
iPS 302	1.1502956	0.19182
muscle 341	1.1787616	0.19278
fetal kidney 94	1.1664252	0.19302
fetal fibroblast 268	1.1294419	0.19354
fetal heart 46	1.1633025	0.19850
fetal renal pelvis 230	1.1637308	0.20426
fetal kidney 102	1.1600773	0.20884
fetal kidney 95	1.1464609	0.20898
fetal kidney 100	1.1461374	0.20972
iPS 303	1.1356197	0.21018
ES derived NP 292	1.1547548	0.21218
fetal adrenal gland 25	1.1524185	0.21508
fetal fibroblast 267	1.1231785	0.21718
iPS 304	1.1221415	0.21952
fetal renal pelvis 221	1.1349054	0.22240
hematopoietic stem cell 311	1.1301876	0.22726
fetal heart 47	1.1419365	0.23160
fetal kidney 103	1.1294494	0.23908
fetal skin 234	1.1195304	0.24760
fetal renal cortex 202	1.1154734	0.25252
ES cells 294	1.1204453	0.26590
fetal kidney 83	1.1246844	0.26748
melanocyte 337	1.0847628	0.26748
hematopoietic stem cell 313	1.1017263	0.26910
fetal renal cortex 207	1.1044609	0.27552
hematopoietic stem cell 306	1.1021777	0.27776
fetal ovary 195	1.1233112	0.27812
fetal kidney 91	1.0920621	0.28886
fetal heart 49	1.0936684	0.29370
hematopoietic stem cell 319	1.0968498	0.29766
fetal adrenal gland 28	1.0810101	0.29788
hematopoietic stem cell 305	1.0879671	0.30602
melanocyte 339	1.0682647	0.30902
hematopoietic stem cell 308	1.0787197	0.31610
hematopoietic stem cell 316	1.0776018	0.33106
fetal heart 44	1.0819108	0.33664
fetal kidney 99	1.0693503	0.33828
hematopoietic stem cell 318	1.0691244	0.34566
fetal kidney 85	1.0574959	0.34892
ES derived mesoderm 286	1.0482446	0.35544
hematopoietic stem cell 307	1.0595051	0.35882
ES cells 295	1.0607443	0.36602
ES cells 296	1.0574237	0.37044
fetal thymus 262	1.0667300	0.37286
ES derived trophoblast 287	1.0547889	0.37292
hematopoietic stem cell 309	1.0514676	0.37618
melanocyte 340	1.0384516	0.39418
fetal thymus 259	1.0504623	0.39782
placenta 201	1.0430998	0.40458
hematopoietic stem cell 317	1.0348978	0.40642
fetal heart 45	1.0334523	0.40966
fetal thymus 261	1.0395990	0.41084
fetal thymus 260	1.0416355	0.41170
placenta 197	1.0372178	0.41196
fetal thymus 257	1.0371289	0.41882
hematopoietic stem cell 315	1.0273496	0.43000
fetal thymus 266	1.0244189	0.43780
hematopoietic stem cell 310	1.0223154	0.43916
fetal small intestine 75	1.0166669	0.44018
hematopoietic stem cell 312	1.0205160	0.44438
CD8 22	1.0206089	0.44666
placenta 196	1.0168618	0.45054
CD4 16	1.0106049	0.46032
CD14 3	1.0126464	0.46304
Mobilized CD3 321	0.9998684	0.47848
Mobilized CD3 320	0.9927471	0.49672
melanocyte 338	0.9965445	0.50178
hematopoietic stem cell 314	0.9863603	0.52262
CD4 15	0.9506604	0.54522
CD3 12	0.9584291	0.54614
CD8 20	0.9614852	0.55084
ES derived trophoblast 288	0.9733918	0.55128
CD14 4	0.9631915	0.55334
fetal thymus 258	0.9607777	0.55526
placenta 200	0.9633280	0.57724
placenta 199	0.9592881	0.57956
fetal large intestine 61	0.9375518	0.59372
fetal thymus 265	0.9318514	0.60436
Mobilized CD4 322	0.9231492	0.60486
CD3 9	0.9235937	0.61248
CD4 14	0.9279923	0.61590
Mobilized CD8 325	0.9136899	0.61768
CD3 11	0.9201934	0.62596
CD19 7	0.9084410	0.62918
CD56 18	0.9078200	0.62966
CD8 19	0.8858364	0.65072
CD4 13	0.8955401	0.65578
ES derived mesoderm 285	0.9391127	0.65770
CD8 21	0.8829838	0.66650
CD56 17	0.8971097	0.67156
Mobilized CD4 323	0.8751356	0.68370
fetal thymus 264	0.8802748	0.69534
placenta 198	0.9019738	0.70880
CD19 5	0.8320812	0.75540
Mobilized CD56 324	0.8276737	0.78164
fetal thymus 256	0.8286054	0.78466
CD3 10	0.7985900	0.80838
CD19 6	0.7863137	0.81078

Prostate cancer susceptibility 2

DHS sample	fold enrichment	p value
keratinocyte 333	1.2848831	0.03718
breast 1	1.3053433	0.04028
keratinocyte 335	1.2703214	0.04776
breast 2	1.2761623	0.05990
fetal small intestine 80	1.3202915	0.06598
keratinocyte 334	1.2386665	0.06960
keratinocyte 336	1.2326271	0.07256
stomach 284	1.3179691	0.08326
fetal small intestine 76	1.3015450	0.08972
pancreas 327	1.2994540	0.09628
fetal small intestine 70	1.2440574	0.09662
pancreas 328	1.2810571	0.10250
fetal large intestine 58	1.2526432	0.10286
stomach 283	1.2791507	0.10884
fetal small intestine 77	1.2569252	0.11702
fibroblast 332	1.1913050	0.13164
fetal small intestine 69	1.2395485	0.14084
fetal large intestine 64	1.2163067	0.14346
fibroblast 329	1.1803259	0.14990
fibroblast 330	1.1812984	0.15098
fetal large intestine 57	1.2015300	0.15162
fetal small intestine 78	1.1939230	0.15350
fetal small intestine 68	1.2042462	0.15362
small intestine 342	1.2131547	0.15392
fetal large intestine 56	1.2107776	0.15524
CD20 8	1.2000780	0.15542
fetal stomach 247	1.2568589	0.15686
fibroblast 331	1.1726579	0.16084
fetal stomach 248	1.1967930	0.18176
fetal large intestine 65	1.1892198	0.18586
fetal small intestine 79	1.1916531	0.18912
fetal large intestine 53	1.1900545	0.19584
hematopoietic stem cell 309	1.1578231	0.19630
fetal lung 109	1.1311830	0.20176
fetal large intestine 59	1.1746603	0.20374
fetal large intestine 66	1.1758486	0.20428
fetal large intestine 55	1.1577874	0.20576
fetal large intestine 54	1.1692343	0.20634
hematopoietic stem cell 308	1.1453392	0.20696
fetal large intestine 63	1.1724669	0.20950
fetal small intestine 73	1.1606849	0.21258
fetal lung 122	1.1441997	0.21336
fetal small intestine 74	1.1618380	0.21338
fetal kidney 81	1.1322894	0.22654
fetal stomach 244	1.1504290	0.22906
fetal large intestine 60	1.1468144	0.23424
fetal stomach 241	1.1560960	0.23676
fetal small intestine 71	1.1439806	0.23710
hematopoietic stem cell 312	1.1241568	0.23916
hematopoietic stem cell 315	1.1313244	0.23918
fetal large intestine 62	1.1493595	0.23920
hematopoietic stem cell 316	1.1345177	0.23938
fetal lung 141	1.1133325	0.24510
placenta 201	1.1399773	0.24560
fetal small intestine 72	1.1360927	0.24676
hematopoietic stem cell 317	1.1072006	0.24988
CD14 4	1.1538156	0.25252
fetal renal cortex 203	1.1280167	0.25458
fetal stomach 253	1.1398133	0.25682
fetal large intestine 67	1.1329999	0.25842
fetal renal pelvis 219	1.1446234	0.25926
hematopoietic stem cell 314	1.1097437	0.26166
hematopoietic stem cell 319	1.1232692	0.26194
fetal kidney 106	1.1158403	0.26486
fetal lung 142	1.0995336	0.26642
fetal muscle 179	1.1143682	0.26744
fetal fibroblast 269	1.0981829	0.26872
fetal renal pelvis 218	1.1337939	0.27412
fetal renal pelvis 216	1.1137856	0.27434
fetal lung 138	1.1021356	0.27788
hematopoietic stem cell 313	1.0999309	0.27876
fetal lung 136	1.0993762	0.28022
fetal lung 129	1.0972413	0.28454
fetal stomach 250	1.1176086	0.28726
fetal lung 114	1.0895657	0.28774
fetal renal pelvis 228	1.1115521	0.28786
hematopoietic stem cell 310	1.0916486	0.29152
fetal muscle 171	1.0920451	0.29462
fetal stomach 240	1.1128547	0.29480
fetal muscle 155	1.1005418	0.29508
fetal stomach 252	1.1051753	0.29946
fetal renal pelvis 227	1.0980373	0.30046
hematopoietic stem cell 306	1.0904111	0.30054
fetal lung 127	1.0884333	0.30068
fetal stomach 251	1.1030789	0.30140
hematopoietic stem cell 311	1.0879576	0.30322
fetal kidney 88	1.0912111	0.30380
fetal kidney 102	1.0986036	0.30486
fetal muscle 190	1.0947277	0.30488
fetal muscle 167	1.0895939	0.30518
fetal fibroblast 270	1.0830968	0.30526
fetal lung 137	1.0837743	0.30654
fetal lung 134	1.0864940	0.31004
fetal kidney 84	1.0896679	0.31370
fetal stomach 246	1.0985044	0.31536
fetal muscle 170	1.0824115	0.31752
fetal stomach 243	1.0870310	0.31858
fetal lung 125	1.0795774	0.32058
hematopoietic stem cell 318	1.0822042	0.32274
fetal lung 113	1.0727159	0.32316
ES derived MSC 289	1.0807532	0.32336
fetal kidney 93	1.0873031	0.32510
fetal renal pelvis 230	1.0878022	0.32650
hematopoietic stem cell 307	1.0760761	0.32706
fetal stomach 245	1.0863294	0.32828
fetal muscle 148	1.0775007	0.32874
fetal muscle 166	1.0765264	0.32984
fetal lung 111	1.0720977	0.33132
fetal muscle 172	1.0684692	0.33330
fetal muscle 158	1.0720964	0.33422
fetal lung 131	1.0651917	0.33430
fetal lung 139	1.0707899	0.33564
fetal muscle 163	1.0728479	0.33582
fetal renal cortex 204	1.0705193	0.33832
fetal lung 124	1.0679848	0.34080
fetal renal pelvis 226	1.0723709	0.34088
fetal muscle 189	1.0683000	0.34090
fetal muscle 157	1.0668548	0.34162
fetal kidney 82	1.0770221	0.34212
fetal muscle 149	1.0727762	0.34396
fetal muscle 181	1.0697244	0.34444
CD14 3	1.0898296	0.34572
fetal muscle 161	1.0679185	0.34672
fetal muscle 151	1.0721613	0.34692
fetal lung 128	1.0588938	0.35112
fetal muscle 188	1.0625852	0.35348
fetal muscle 177	1.0635361	0.35674
fetal muscle 169	1.0625870	0.35676
fetal muscle 187	1.0588011	0.35782
fetal kidney 104	1.0639883	0.35930
fetal lung 110	1.0575763	0.36078
fetal lung 300	1.0594982	0.36180
fetal stomach 249	1.0662267	0.36302
ES derived MSC 290	1.0567950	0.36786
fetal lung 301	1.0577005	0.36826
fetal muscle 185	1.0578224	0.36874
fetal skin 234	1.0605776	0.36926
fetal kidney 86	1.0619779	0.37084
fetal kidney 103	1.0547975	0.37292
fetal muscle 186	1.0498478	0.37652
fetal muscle 145	1.0512382	0.37766
fetal muscle 183	1.0482711	0.37912
fetal renal pelvis 223	1.0536647	0.37990
fetal kidney 94	1.0515101	0.38050
fetal lung 130	1.0469175	0.38104
fetal stomach 242	1.0564906	0.38114
fetal renal cortex 213	1.0490647	0.38264
fetal fibroblast 280	1.0458480	0.38286
fetal lung 298	1.0505081	0.38302
fetal kidney 95	1.0503513	0.38308
fetal renal cortex 214	1.0487429	0.38546
fetal muscle 184	1.0454506	0.38656
fetal thymus 263	1.0466431	0.38664
fetal renal cortex 209	1.0481227	0.38770
fetal muscle 144	1.0468566	0.38806
fetal renal cortex 207	1.0467816	0.38916
fetal muscle 180	1.0480166	0.39138
fetal lung 135	1.0435892	0.39186
fetal lung 117	1.0418516	0.39384
fetal kidney 83	1.0461686	0.39526
fetal kidney 97	1.0422990	0.39724
fetal renal pelvis 222	1.0442528	0.39732
fetal muscle 147	1.0400691	0.39762
fetal kidney 90	1.0438605	0.39770
fetal renal cortex 212	1.0399780	0.39798
fetal renal pelvis 220	1.0417154	0.39926
hematopoietic stem cell 305	1.0396302	0.39950
fetal renal pelvis 233	1.0411934	0.40322
fetal renal pelvis 225	1.0390083	0.40364
placenta 200	1.0367778	0.40624
fetal adrenal gland 28	1.0359750	0.40884
fetal renal cortex 210	1.0377083	0.40916
fetal muscle 175	1.0336801	0.40936
fetal renal cortex 211	1.0359512	0.40952
fetal kidney 107	1.0355426	0.40968
fetal muscle 178	1.0340808	0.41274
fetal kidney 98	1.0332654	0.41362
fetal muscle 160	1.0315241	0.41612
fetal fibroblast 273	1.0306501	0.41698
fetal kidney 87	1.0312026	0.41978
fetal renal pelvis 221	1.0291374	0.42180
fetal renal cortex 202	1.0258795	0.42554
fetal kidney 99	1.0263176	0.42626
fetal lung 133	1.0272002	0.42696
fetal muscle 150	1.0265752	0.42844
CD56 17	1.0324780	0.42950
fetal renal cortex 205	1.0256889	0.43266
fetal kidney 100	1.0229462	0.43366
fetal fibroblast 281	1.0235499	0.43442
placenta 196	1.0247704	0.43472
fetal renal pelvis 232	1.0226849	0.43626
fetal small intestine 75	1.0052090	0.43756
fetal muscle 192	1.0209583	0.43772
fetal muscle 146	1.0212645	0.43798
fetal muscle 173	1.0178297	0.44058
fetal lung 115	1.0204436	0.44176
fetal renal cortex 208	1.0188465	0.44272
fetal fibroblast 274	1.0195464	0.44306
fetal muscle 182	1.0193344	0.44336
fetal renal cortex 215	1.0172759	0.44388
fetal fibroblast 282	1.0192938	0.44410
fetal lung 121	1.0186636	0.44492
fetal muscle 154	1.0181845	0.44584
fetal muscle 156	1.0183193	0.44630
fetal fibroblast 277	1.0184406	0.44678
fetal muscle 174	1.0166999	0.44760
fetal heart 47	1.0175425	0.44776
fetal lung 123	1.0168571	0.44922
fetal renal pelvis 224	1.0159985	0.44962
fetal lung 126	1.0168867	0.45062
fetal renal pelvis 217	1.0117408	0.45482
fetal muscle 191	1.0119122	0.45558
fetal heart 42	1.0152472	0.45568
fetal heart 48	1.0137618	0.45624
fetal kidney 89	1.0112772	0.45656
fetal heart 50	1.0135719	0.45694
fetal lung 116	1.0136613	0.45784
fetal muscle 193	1.0093138	0.46000
fetal renal pelvis 229	1.0076254	0.46222
ovary 326	1.0104800	0.46332
fetal muscle 152	1.0098659	0.46356
CD3 10	1.0119382	0.46424
fetal heart 49	1.0091605	0.46612
fetal heart 43	1.0089192	0.46794
fetal lung 132	1.0073299	0.47330
fetal heart 46	1.0041594	0.47350
fetal kidney 96	1.0031603	0.47384
fetal kidney 92	1.0009365	0.47448
fetal kidney 105	1.0015234	0.47770
fetal muscle 159	1.0007299	0.47928
fetal renal cortex 206	0.9992108	0.47984
fetal fibroblast 272	1.0031216	0.48292
fetal renal pelvis 231	0.9997906	0.48316
CD19 5	0.9984958	0.48354
fetal lung 108	0.9973764	0.48624
fetal fibroblast 278	1.0006810	0.48692
placenta 199	1.0011065	0.48876
fetal adrenal gland 23	0.9998285	0.48934
fetal muscle 168	0.9935135	0.49232
fetal heart 52	0.9958145	0.49600
fetal muscle 164	0.9914375	0.50016
fetal heart 45	0.9908036	0.50072
ES derived trophoblast 288	0.9922402	0.50132
muscle 341	0.9870200	0.50392
CD8 20	0.9857109	0.50396
fetal lung 120	0.9933980	0.50398
fetal adrenal gland 25	0.9925678	0.50460
fetal heart 51	0.9891729	0.50654
fetal fibroblast 276	0.9931786	0.50710
fetal heart 44	0.9805584	0.51056
fetal kidney 101	0.9844561	0.51262
fetal fibroblast 268	0.9903592	0.51686
fetal muscle 194	0.9864617	0.51798
fetal thymus 262	0.9619077	0.51936
fetal fibroblast 279	0.9859247	0.52044
fetal lung 119	0.9856833	0.52582
fetal fibroblast 267	0.9840550	0.52714
fetal kidney 85	0.9771899	0.53360
fetal muscle 153	0.9709344	0.53474
fetal thymus 261	0.9646685	0.53680
fetal muscle 165	0.9768976	0.53754
fetal lung 140	0.9762685	0.54380
fetal lung 299	0.9745112	0.55016
CD19 6	0.9552130	0.55166
placenta 197	0.9661503	0.55384
ES derived NP 291	0.9610754	0.55488
fetal adrenal gland 24	0.9608681	0.55860
fetal muscle 143	0.9665703	0.56616
fetal adrenal gland 27	0.9643084	0.56630
fetal fibroblast 271	0.9675442	0.57166
fetal muscle 176	0.9577643	0.57502
CD4 14	0.9404991	0.58654
fetal fibroblast 275	0.9596738	0.59048
CD19 7	0.9302706	0.59104
fetal muscle 162	0.9473912	0.59940
CD4 16	0.9200097	0.59988
fetal lung 112	0.9480255	0.60834
fetal adrenal gland 26	0.9393084	0.62286
fetal large intestine 61	0.9114571	0.62442
fetal thymus 260	0.9161150	0.62794
placenta 198	0.9361781	0.62934
heart 297	0.9187966	0.63762
fetal thymus 265	0.9070564	0.64700
Mobilized CD8 325	0.8865458	0.65398
fetal thymus 259	0.8925696	0.65674
CD8 21	0.8779239	0.66420
Mobilized CD3 321	0.8751475	0.66500
fetal ovary 195	0.8844920	0.67156
CD8 22	0.8733277	0.67180
fetal thymus 264	0.8902400	0.67458
CD3 11	0.8838704	0.67802
fetal testes 254	0.9008472	0.68526
fetal thymus 257	0.8711415	0.68644
fetal lung 118	0.9135744	0.69956
ES derived mesoderm 286	0.9191828	0.70680
fetal testes 255	0.8908294	0.71412
ES cells 295	0.8706083	0.71478
fetal thymus 256	0.8710728	0.71592
CD3 12	0.8433310	0.71620
ES derived trophoblast 287	0.8805292	0.71714
ES derived NP 292	0.8733691	0.71812
fetal thymus 266	0.8593420	0.72394
CD8 19	0.8263066	0.72706
Mobilized CD4 322	0.8260507	0.73916
fetal kidney 91	0.8796397	0.74338
melanocyte 339	0.8902686	0.76048
ES cells 294	0.8495344	0.76532
CD3 9	0.8082443	0.77430
ES cells 293	0.8456764	0.77766
CD4 15	0.7696551	0.77866
ES derived mesoderm 285	0.8775877	0.78292
melanocyte 337	0.8789476	0.79514
ES cells 296	0.8120302	0.81074
Mobilized CD3 320	0.7640763	0.81108
melanocyte 338	0.8624128	0.81670
CD56 18	0.7646115	0.81934
Mobilized CD56 324	0.7821981	0.82376
fetal thymus 258	0.7836908	0.83218
CD4 13	0.7560431	0.83868
Mobilized CD4 323	0.7494596	0.84456
melanocyte 340	0.8241940	0.86638
iPS 302	0.7912794	0.88144
iPS 304	0.7890794	0.90086
iPS 303	0.7518138	0.93090
fetal spinal cord 237	0.7554988	0.93092
fetal brain 39	0.7559858	0.95910
fetal spinal cord 236	0.6944486	0.96428
fetal brain 41	0.7311189	0.96876
fetal spinal cord 235	0.6742249	0.97578
fetal spinal cord 238	0.6669242	0.98038
fetal brain 35	0.7252135	0.98104
fetal brain 29	0.7399661	0.98322
fetal brain 37	0.6741455	0.99122
fetal brain 40	0.6825593	0.99156
fetal brain 34	0.6862438	0.99186
fetal spinal cord 239	0.6258650	0.99190
fetal brain 32	0.6588295	0.99306
fetal brain 33	0.6308206	0.99358
fetal brain 30	0.7023661	0.99380
fetal brain 38	0.6242302	0.99584
fetal brain 36	0.5742373	0.99926
fetal brain 31	0.6123360	0.99964

Stroke and coronary artery disease

DHS sample	fold enrichment	p value
fetal adrenal gland 25	1.6155286	0.01188
fetal adrenal gland 26	1.5038111	0.01936
fetal adrenal gland 28	1.4350394	0.02014
fetal adrenal gland 24	1.5497064	0.02556
fetal adrenal gland 27	1.4600610	0.02638
fetal adrenal gland 23	1.4109158	0.03234
fibroblast 329	1.3545524	0.03524
fetal muscle 154	1.4498454	0.03780
fetal thymus 260	1.5401986	0.03874
hematopoietic stem cell 309	1.3984909	0.03914
hematopoietic stem cell 319	1.4378134	0.03916
fibroblast 330	1.3531445	0.04004
hematopoietic stem cell 318	1.4007528	0.04884
hematopoietic stem cell 310	1.3254367	0.04926
fetal thymus 257	1.5175899	0.04946
fetal testes 255	1.3690801	0.05032
hematopoietic stem cell 315	1.3704819	0.05074
ES cells 294	1.4125514	0.05240
fibroblast 331	1.3091774	0.05578
hematopoietic stem cell 305	1.3714338	0.05608
hematopoietic stem cell 314	1.3343182	0.05628
fetal thymus 258	1.4565630	0.05632
fetal brain 36	1.3141561	0.05846
fetal thymus 261	1.4869100	0.05860
fetal thymus 265	1.4477613	0.06192
ES cells 295	1.4119448	0.06404
ES cells 296	1.3966985	0.06590
fetal thymus 264	1.4324580	0.06694
hematopoietic stem cell 311	1.3270733	0.06788
hematopoietic stem cell 312	1.3173596	0.06826
hematopoietic stem cell 316	1.3548559	0.06834
fetal testes 254	1.3493919	0.06922
fibroblast 332	1.2865432	0.06934
ES derived NP 291	1.3815104	0.07130
fetal thymus 266	1.4193800	0.07412
fetal thymus 259	1.4362373	0.07568
hematopoietic stem cell 307	1.3152261	0.07718
hematopoietic stem cell 317	1.2727934	0.07936
muscle 341	1.3828467	0.08060
hematopoietic stem cell 308	1.2951793	0.08092
hematopoietic stem cell 313	1.2923394	0.08210
iPS 302	1.2946438	0.08420
ES derived MSC 289	1.3055623	0.08628
heart 297	1.3459148	0.08966
fetal small intestine 72	1.3332474	0.09042
hematopoietic stem cell 306	1.2890158	0.09066
ES derived mesoderm 286	1.2265736	0.09316
fetal thymus 262	1.4467699	0.09404
ES derived mesoderm 285	1.2326960	0.09534
fetal muscle 150	1.3060465	0.09560
fetal ovary 195	1.3671587	0.09652
small intestine 342	1.3176214	0.09764
fetal fibroblast 269	1.2376048	0.09796
fetal muscle 178	1.2982757	0.10096
CD19 7	1.4105988	0.10124
fetal muscle 155	1.3038454	0.10356
fetal muscle 169	1.2813368	0.10654
CD8 21	1.4097521	0.10778
fetal muscle 191	1.2745659	0.10966
fetal muscle 149	1.2827512	0.11174
fetal kidney 95	1.2827776	0.11272
CD4 15	1.4493057	0.11354
iPS 304	1.2406873	0.11386
fetal muscle 146	1.2553397	0.11748
ES derived MSC 290	1.2612573	0.11806
iPS 303	1.2425111	0.11888
fetal muscle 151	1.2812188	0.11996
fetal muscle 175	1.2540669	0.12004
fetal muscle 152	1.2477481	0.12264
fetal muscle 164	1.2620339	0.12448
pancreas 327	1.3030249	0.12546
ES derived NP 292	1.2801213	0.12742
fetal kidney 106	1.2540639	0.12762
fetal thymus 263	1.2602562	0.12800
fetal muscle 166	1.2474215	0.12810
fetal fibroblast 275	1.2012778	0.12812
fetal renal cortex 214	1.2545004	0.13004
fetal muscle 157	1.2263438	0.13124
fetal fibroblast 280	1.2074160	0.13176
fetal muscle 143	1.2253771	0.13320
fetal muscle 159	1.2554716	0.13334
fetal renal pelvis 221	1.2444937	0.13386
Mobilized CD4 322	1.3469898	0.13452
fetal fibroblast 270	1.2123530	0.13490
Mobilized CD3 321	1.3573533	0.13538
fetal large intestine 63	1.2816227	0.13602
fetal kidney 83	1.2832003	0.13636
fetal fibroblast 279	1.2067354	0.13720
CD3 12	1.3412550	0.13896
fetal muscle 168	1.2503378	0.14132
Mobilized CD3 320	1.3541652	0.14140
ES cells 293	1.2450999	0.14284
fetal muscle 148	1.2368941	0.14352
fetal renal pelvis 228	1.2574661	0.14414
fetal kidney 99	1.2397567	0.14418
fetal muscle 181	1.2417455	0.14540
fetal small intestine 71	1.2540608	0.14720
fetal renal pelvis 219	1.2911723	0.14838
CD56 18	1.3196774	0.14888
Mobilized CD4 323	1.3131209	0.14958
fetal small intestine 79	1.2640488	0.14992
fetal muscle 189	1.2249700	0.15030
Mobilized CD8 325	1.3149540	0.15194
fetal fibroblast 272	1.1850655	0.15234
fetal muscle 167	1.2138271	0.15328
fetal small intestine 69	1.2582497	0.15386
fetal large intestine 66	1.2539287	0.15432
ovary 326	1.2827631	0.15530
fetal muscle 174	1.2263078	0.15552
CD3 9	1.3117012	0.15584
fetal renal pelvis 232	1.2318429	0.15738
fetal renal cortex 207	1.2269301	0.15794
fetal muscle 182	1.2186560	0.15798
fetal thymus 256	1.2636963	0.16016
pancreas 328	1.2441094	0.16234
fetal muscle 193	1.2205409	0.16252
fetal renal cortex 202	1.2135612	0.16320
fetal large intestine 53	1.2498860	0.16344
fetal lung 133	1.1856461	0.16372
fetal muscle 147	1.2147844	0.16386
fetal fibroblast 276	1.1744041	0.16386
CD8 19	1.3187967	0.16436
fetal muscle 172	1.1791549	0.16594
fetal large intestine 62	1.2486095	0.16598
fetal large intestine 59	1.2380089	0.16772
fetal lung 129	1.1857532	0.16796
fetal muscle 165	1.1982564	0.16824
fetal muscle 156	1.2029529	0.16992
fetal muscle 192	1.2002241	0.17038
fetal renal pelvis 217	1.2209569	0.17076
fetal large intestine 54	1.2247681	0.17268
fetal muscle 179	1.2061423	0.17286
fetal renal pelvis 231	1.1978269	0.17432
fetal renal pelvis 220	1.2182133	0.17434
fetal kidney 103	1.2139559	0.17480
fetal muscle 184	1.1894469	0.17552
fetal kidney 92	1.2018859	0.17572
fetal large intestine 56	1.2124065	0.17780
fetal kidney 87	1.2000320	0.17796
fetal muscle 145	1.2175359	0.17980
fetal muscle 144	1.2072065	0.18010
fetal large intestine 60	1.2187446	0.18012
fetal renal pelvis 227	1.2057785	0.18032
fetal muscle 153	1.2088946	0.18310
fetal fibroblast 277	1.1631653	0.18594
fetal kidney 100	1.1978299	0.18636
fetal small intestine 75	1.2808456	0.18642
stomach 283	1.2198347	0.18714
fetal large intestine 61	1.2486389	0.18728
CD8 22	1.2485733	0.18744
fetal fibroblast 267	1.1625432	0.18788
fetal muscle 160	1.1936744	0.18852
fetal lung 121	1.1603273	0.18868
fetal large intestine 65	1.2149128	0.18900
fetal fibroblast 278	1.1607209	0.18910
fetal muscle 176	1.1822957	0.18974
fetal renal cortex 213	1.1825186	0.19136
fetal lung 139	1.1708306	0.19140
fetal small intestine 76	1.2175256	0.19148
fetal stomach 242	1.2396338	0.19184
fetal muscle 190	1.2013271	0.19264
fetal muscle 171	1.1742300	0.19324
CD19 6	1.2482230	0.19372
fetal stomach 240	1.2180057	0.19540
fetal muscle 177	1.1968797	0.19554
CD4 13	1.2541069	0.19556
fetal heart 45	1.1919042	0.19898
fetal spinal cord 239	1.1620099	0.20022
fetal brain 40	1.1401528	0.20054
fetal kidney 97	1.1796017	0.20270
fetal fibroblast 268	1.1462135	0.20314
fetal kidney 89	1.1822330	0.20372
CD19 5	1.2306761	0.20740
fetal spinal cord 236	1.1643001	0.20834
fetal renal cortex 211	1.1861197	0.20836
fetal heart 52	1.1634939	0.21198
fetal muscle 180	1.1804215	0.21220
fetal renal cortex 205	1.1724139	0.21248
fetal renal pelvis 218	1.2066538	0.21362
fetal small intestine 74	1.1800525	0.21414
fetal kidney 91	1.1599179	0.21512
fetal muscle 183	1.1592272	0.21692
fetal muscle 185	1.1732384	0.21832
fetal fibroblast 282	1.1356909	0.21858
fetal kidney 94	1.1759772	0.21944
fetal lung 125	1.1498421	0.21986
fetal lung 123	1.1468496	0.21990
fetal renal pelvis 233	1.1681831	0.22012
fetal small intestine 80	1.1730377	0.22068
fetal muscle 158	1.1542942	0.22208
fetal muscle 194	1.1413394	0.22450
fetal stomach 250	1.1810374	0.22496
fetal heart 44	1.1913165	0.22586
CD8 20	1.1987033	0.22906
CD20 8	1.1625618	0.22944
CD3 10	1.1955785	0.23328
fetal muscle 173	1.1544806	0.23348
fetal renal pelvis 216	1.1599601	0.23410
fetal stomach 247	1.1882999	0.23482
fetal renal pelvis 225	1.1599076	0.23566
fetal large intestine 55	1.1558611	0.23576
keratinocyte 333	1.1109188	0.23630
fetal small intestine 77	1.1628775	0.23708
fetal lung 118	1.1223473	0.23760
fetal lung 134	1.1352740	0.23824
fetal small intestine 68	1.1536397	0.23846
stomach 284	1.1651681	0.23908
fetal kidney 98	1.1538822	0.23984
fetal kidney 88	1.1437532	0.24010
fetal large intestine 67	1.1604106	0.24040
fetal muscle 187	1.1419305	0.24180
fetal kidney 96	1.1416592	0.24224
fetal kidney 84	1.1504950	0.24352
fetal fibroblast 271	1.1225903	0.24406
melanocyte 338	1.1136523	0.24432
fetal small intestine 73	1.1589601	0.24546
fetal renal pelvis 230	1.1533128	0.24702
fetal lung 120	1.1290177	0.24782
fetal fibroblast 273	1.1196309	0.24824
fetal lung 137	1.1241141	0.24946
fetal large intestine 58	1.1447246	0.24948
fetal small intestine 70	1.1349054	0.25000
fetal muscle 163	1.1428051	0.25016
fetal kidney 81	1.1322510	0.25070
fetal lung 138	1.1258100	0.25136
fetal large intestine 64	1.1462727	0.25172
fetal stomach 246	1.1559164	0.25324
fetal lung 135	1.1259262	0.25340
CD4 16	1.1732030	0.25562
fetal kidney 86	1.1558375	0.25762
fetal muscle 162	1.1284856	0.25880
fetal lung 119	1.1172342	0.25896
fetal small intestine 78	1.1319689	0.26072
fetal renal pelvis 226	1.1335961	0.26140
fetal heart 46	1.1387885	0.26170
fetal renal cortex 203	1.1346636	0.26300
fetal stomach 252	1.1357044	0.26532
fetal renal cortex 215	1.1231866	0.26538
fetal fibroblast 274	1.1082945	0.26802
fetal kidney 105	1.1248489	0.26894
fetal muscle 188	1.1227606	0.26916
fetal spinal cord 238	1.1160073	0.26980
fetal lung 128	1.1023246	0.27034
fetal lung 300	1.1182155	0.27060
fetal lung 130	1.1121437	0.27106
fetal kidney 82	1.1358367	0.27178
fetal lung 299	1.1185824	0.27190
fetal lung 132	1.1128143	0.27210
fetal muscle 161	1.1268511	0.27276
fetal lung 136	1.1126485	0.27332
fetal renal cortex 206	1.1203159	0.27544
fetal spinal cord 237	1.1102137	0.27698
fetal renal cortex 212	1.1159039	0.27732
fetal muscle 170	1.1158002	0.27968
fetal lung 108	1.1123866	0.27974
CD3 11	1.1511402	0.28030
fetal brain 41	1.0971872	0.28220
Mobilized CD56 324	1.1500887	0.28302
fetal lung 124	1.1037310	0.28662
fetal lung 301	1.1078314	0.28740
fetal stomach 248	1.1265405	0.28776
fetal muscle 186	1.1103619	0.28992
fetal renal pelvis 223	1.1177435	0.29026
fetal lung 117	1.0959428	0.29096
fetal kidney 102	1.1146659	0.29154
CD4 14	1.1382349	0.29162
fetal fibroblast 281	1.0943859	0.29262
fetal kidney 107	1.1086283	0.29276
melanocyte 337	1.0886075	0.29306
fetal stomach 249	1.1207799	0.29340
fetal kidney 104	1.1094543	0.29458
keratinocyte 336	1.0845508	0.29528
fetal lung 122	1.1019192	0.29566
fetal stomach 251	1.1135676	0.29586
placenta 199	1.1041570	0.29710
fetal kidney 85	1.0959601	0.29796
fetal lung 141	1.0917608	0.29856
fetal stomach 245	1.1121877	0.30232
fetal lung 127	1.0966313	0.30274
placenta 196	1.1040873	0.30294
fetal stomach 253	1.1152588	0.30350
fetal renal pelvis 222	1.1045443	0.30450
fetal kidney 90	1.1005750	0.30846
fetal lung 112	1.0884620	0.31008
fetal renal pelvis 229	1.0964516	0.31136
fetal kidney 93	1.1008125	0.31472
fetal kidney 101	1.0954802	0.31512
fetal stomach 241	1.1030259	0.31520
fetal lung 298	1.0924029	0.31584
fetal stomach 244	1.0997748	0.31672
fetal skin 234	1.0954201	0.31700
fetal heart 43	1.0903586	0.31818
fetal lung 142	1.0798184	0.31932
fetal brain 38	1.0790240	0.31938
fetal heart 51	1.0933583	0.32040
fetal heart 48	1.0928525	0.32144
fetal renal cortex 208	1.0920475	0.32312
fetal lung 131	1.0753203	0.32422
fetal heart 49	1.0937681	0.32566
fetal heart 47	1.0998901	0.32768
fetal lung 110	1.0804419	0.32906
fetal renal pelvis 224	1.0810009	0.32928
fetal heart 42	1.0868011	0.33188
CD56 17	1.1044653	0.33194
fetal lung 126	1.0732570	0.33226
fetal lung 111	1.0754419	0.33386
fetal stomach 243	1.0826772	0.33754
fetal heart 50	1.0781167	0.34404
fetal lung 115	1.0683569	0.34518
placenta 200	1.0664081	0.35192
placenta 197	1.0707322	0.35410
fetal renal cortex 210	1.0664024	0.36082
melanocyte 340	1.0586076	0.36508
melanocyte 339	1.0547458	0.36752
fetal brain 33	1.0565117	0.36800
fetal lung 140	1.0572416	0.36984
fetal lung 116	1.0548155	0.37290
fetal spinal cord 235	1.0509231	0.37634
fetal renal cortex 209	1.0570243	0.37826
keratinocyte 335	1.0449713	0.38528
CD14 4	1.0643491	0.38692
fetal renal cortex 204	1.0454293	0.39240
placenta 198	1.0421227	0.40348
CD14 3	1.0522708	0.40366
keratinocyte 334	1.0354961	0.40600
placenta 201	1.0372627	0.41252
fetal lung 109	1.0304812	0.41568
fetal large intestine 57	1.0351518	0.41810
fetal lung 113	1.0287534	0.42258
fetal brain 39	1.0262200	0.42318
fetal lung 114	1.0214966	0.44036
fetal brain 32	1.0122340	0.45566
breast 1	1.0112566	0.46426
fetal brain 37	0.9978344	0.47998
breast 2	0.9986255	0.49234
fetal brain 34	0.9868141	0.51690
fetal brain 29	0.9825778	0.53434
ES derived trophoblast 288	0.9513704	0.57846
fetal brain 30	0.9478803	0.63244
fetal brain 31	0.9437641	0.63396
fetal brain 35	0.9320638	0.65688
ES derived trophoblast 287	0.8885856	0.67400

Plasma homocysteine concentrations

DHS sample	fold enrichment	p value
Mobilized CD3 320	1.6178966	0.01364
melanocyte 339	1.2703985	0.05300
fetal small intestine 80	1.3409575	0.05372
fetal large intestine 64	1.3363898	0.05552
fetal large intestine 58	1.2935169	0.07078
fetal large intestine 67	1.3281113	0.07316
melanocyte 338	1.2265394	0.07534
melanocyte 337	1.2208851	0.07940
fetal small intestine 78	1.2728262	0.08162
fetal large intestine 57	1.2669707	0.09048
fetal heart 47	1.3054466	0.09074
fetal small intestine 68	1.2625116	0.10096
CD20 8	1.2529477	0.10590
fetal small intestine 74	1.2564718	0.10960
ES derived mesoderm 285	1.2178922	0.11112
fetal small intestine 70	1.2298083	0.11200
melanocyte 340	1.2033936	0.12044
fetal lung 111	1.2037758	0.13110
fetal heart 45	1.2332341	0.13248
CD56 18	1.2782145	0.13526
fetal large intestine 55	1.2222485	0.13894
small intestine 342	1.2253338	0.13966
fetal large intestine 61	1.2454915	0.15430
fetal large intestine 56	1.2048497	0.16696
Mobilized CD4 323	1.2274680	0.17580
fetal heart 49	1.1926448	0.17678
CD3 9	1.2170440	0.17948
fetal small intestine 69	1.2004170	0.18372
iPS 303	1.1692719	0.19126
fetal large intestine 59	1.1860687	0.19616
iPS 304	1.1546201	0.20484
fetal heart 51	1.1625990	0.20702
fetal heart 52	1.1545474	0.21168
fetal small intestine 72	1.1648286	0.21254
stomach 284	1.1762102	0.21292
CD4 13	1.1875983	0.21618
fetal thymus 256	1.1638840	0.21716
fetal small intestine 79	1.1698424	0.21866
fetal large intestine 54	1.1604091	0.22054
fetal large intestine 62	1.1656641	0.22492
fetal heart 46	1.1530308	0.22604
fetal thymus 258	1.1650465	0.22738
fetal lung 121	1.1324749	0.22776
Mobilized CD56 324	1.1618236	0.23060
fetal large intestine 60	1.1516095	0.23122
fetal heart 48	1.1476689	0.23128
fetal heart 43	1.1418445	0.23132
fetal lung 114	1.1257051	0.23244
ES derived mesoderm 286	1.1162068	0.23426
fetal small intestine 73	1.1507943	0.23642
fetal lung 140	1.1277898	0.23818
CD8 19	1.1817768	0.23834
fetal stomach 244	1.1590981	0.23846
fetal large intestine 63	1.1491978	0.24214
CD8 21	1.1707615	0.24220
ES derived trophoblast 288	1.1304927	0.24342
Mobilized CD8 325	1.1711667	0.24528
fetal lung 133	1.1226908	0.24558
fetal lung 128	1.1123718	0.24734
fetal lung 118	1.1157132	0.24888
fetal lung 141	1.1176790	0.25014
ES cells 296	1.1494385	0.25118
fetal thymus 265	1.1483017	0.25512
fetal stomach 250	1.1520105	0.25568
fetal small intestine 77	1.1365396	0.25936
fetal thymus 260	1.1427546	0.25996
pancreas 328	1.1375576	0.26274
fetal small intestine 76	1.1321467	0.26768
fetal muscle 180	1.1223197	0.26834
fetal lung 110	1.1165320	0.26904
fetal kidney 85	1.1083086	0.26910
fetal large intestine 65	1.1265921	0.26994
fetal thymus 266	1.1314804	0.27000
fetal heart 44	1.1379710	0.27136
fetal small intestine 75	1.1674817	0.27366
fetal heart 42	1.1169802	0.27846
fetal lung 116	1.0965011	0.28144
fetal lung 134	1.1096722	0.28162
ES cells 295	1.1246588	0.28594
fetal small intestine 71	1.1131527	0.29274
fetal stomach 242	1.1329565	0.29376
fetal heart 50	1.1059317	0.29382
ovary 326	1.1243598	0.29552
fetal testes 255	1.1011639	0.29762
stomach 283	1.1123620	0.30168
hematopoietic stem cell 314	1.0881307	0.30528
fetal muscle 143	1.0878599	0.31004
ES cells 294	1.1069250	0.31092
fetal adrenal gland 27	1.0923090	0.31620
fetal lung 135	1.0853544	0.31764
CD3 11	1.0997023	0.31784
fetal lung 125	1.0890196	0.31924
fibroblast 331	1.0781194	0.31980
fetal lung 112	1.0842060	0.32050
fetal muscle 166	1.0883200	0.32064
fetal lung 132	1.0798933	0.32626
hematopoietic stem cell 305	1.0852748	0.32628
fetal lung 119	1.0771498	0.32802
fetal stomach 252	1.0940972	0.32862
fetal testes 254	1.0866357	0.32886
placenta 200	1.0765641	0.32928
fetal stomach 253	1.0969117	0.32974
fetal kidney 106	1.0830890	0.33190
fetal renal cortex 213	1.0784343	0.33334
Mobilized CD3 321	1.0988828	0.33412
fetal muscle 164	1.0793543	0.33490
CD4 15	1.1065154	0.33524
hematopoietic stem cell 310	1.0667463	0.34048
placenta 198	1.0728804	0.34138
CD4 16	1.0917981	0.34328
fetal stomach 240	1.0866862	0.34516
Mobilized CD4 322	1.0897991	0.34620
fetal large intestine 53	1.0830298	0.34646
fetal lung 120	1.0702179	0.34718
fetal stomach 246	1.0826706	0.34732
fetal large intestine 66	1.0778789	0.34898
heart 297	1.0778961	0.35182
fibroblast 332	1.0610622	0.35338
CD3 12	1.0822887	0.35644
fetal lung 139	1.0666775	0.35644
fetal lung 124	1.0651606	0.35644
ES derived MSC 289	1.0649128	0.35764
fetal muscle 179	1.0664808	0.35824
fetal muscle 165	1.0641259	0.35824
fetal thymus 259	1.0786861	0.35842
fetal stomach 245	1.0717012	0.36410
fetal muscle 176	1.0602060	0.36502
fetal thymus 262	1.0749106	0.36920
fibroblast 329	1.0550240	0.37024
fetal muscle 181	1.0582843	0.37156
fetal muscle 183	1.0565066	0.37414
fetal lung 117	1.0532267	0.37696
iPS 302	1.0539807	0.37766
fetal lung 129	1.0533675	0.37892
hematopoietic stem cell 308	1.0513298	0.37922
CD8 22	1.0651994	0.37934
placenta 199	1.0540618	0.37994
fetal stomach 251	1.0604703	0.38076
fetal stomach 249	1.0601966	0.38194
fetal stomach 243	1.0551811	0.38732
placenta 201	1.0515571	0.38768
fetal muscle 152	1.0485197	0.38940
ES cells 293	1.0525270	0.39102
fetal thymus 264	1.0516069	0.39262
fetal muscle 182	1.0469982	0.39394
fetal lung 136	1.0447590	0.39630
muscle 341	1.0514022	0.39688
fetal adrenal gland 26	1.0455833	0.39816
hematopoietic stem cell 318	1.0447754	0.39902
fetal kidney 103	1.0445043	0.39954
fetal fibroblast 271	1.0390041	0.39990
CD8 20	1.0486215	0.40206
CD3 10	1.0481396	0.40288
fetal skin 234	1.0464112	0.40304
fetal muscle 149	1.0405397	0.40608
fetal kidney 84	1.0418999	0.40618
CD14 3	1.0473112	0.40692
fetal lung 130	1.0373068	0.40832
ES derived MSC 290	1.0374934	0.41192
fetal kidney 95	1.0368511	0.41286
keratinocyte 336	1.0315725	0.41630
fibroblast 330	1.0346243	0.41640
fetal thymus 261	1.0390297	0.41718
CD19 7	1.0422963	0.41722
fetal stomach 247	1.0413239	0.41744
fetal stomach 241	1.0384313	0.41874
fetal fibroblast 278	1.0299172	0.42212
fetal stomach 248	1.0341879	0.42302
fetal renal pelvis 228	1.0297474	0.42406
fetal lung 126	1.0294985	0.42590
fetal muscle 178	1.0305097	0.42602
fetal fibroblast 277	1.0272647	0.42622
fetal muscle 171	1.0293023	0.42752
fetal muscle 185	1.0285556	0.42882
fetal lung 123	1.0268173	0.43058
fetal lung 127	1.0263534	0.43308
CD56 17	1.0296825	0.43346
fetal muscle 157	1.0267687	0.43444
fetal muscle 173	1.0250637	0.43570
fetal renal pelvis 221	1.0232574	0.43816
fetal muscle 158	1.0225569	0.43928
fetal renal cortex 214	1.0211526	0.44088
fetal lung 137	1.0217487	0.44152
fetal adrenal gland 25	1.0248071	0.44198
fetal brain 37	1.0185358	0.44316
CD14 4	1.0254056	0.44338
fetal muscle 160	1.0182267	0.44966
fetal lung 131	1.0183351	0.44974
fetal muscle 155	1.0179796	0.44986
fetal renal pelvis 220	1.0178729	0.45026
fetal muscle 147	1.0141483	0.45176
keratinocyte 335	1.0174676	0.45178
fetal lung 108	1.0164044	0.45520
fetal muscle 163	1.0144210	0.45554
fetal renal pelvis 227	1.0102342	0.45762
fetal muscle 189	1.0129229	0.45844
fetal lung 138	1.0137740	0.45854
fetal kidney 83	1.0113313	0.45934
CD19 6	1.0135485	0.46010
fetal thymus 263	1.0128302	0.46134
fetal fibroblast 268	1.0113785	0.46200
fetal muscle 159	1.0107488	0.46220
fetal muscle 167	1.0127167	0.46330
fetal adrenal gland 24	1.0124642	0.46400
pancreas 327	1.0112444	0.46420
hematopoietic stem cell 315	1.0129608	0.46446
CD4 14	1.0139708	0.46472
hematopoietic stem cell 312	1.0114419	0.46498
fetal muscle 150	1.0082911	0.46706
fetal renal cortex 207	1.0054359	0.46782
fetal kidney 99	1.0077289	0.46790
fetal muscle 194	1.0073906	0.46928
fetal muscle 153	1.0080930	0.46980
placenta 197	1.0070051	0.47030
fetal renal pelvis 231	1.0045857	0.47132
ES derived trophoblast 287	1.0131866	0.47140
hematopoietic stem cell 316	1.0066475	0.47332
fetal muscle 154	1.0037755	0.47360
fetal renal pelvis 217	1.0042113	0.47390
fetal muscle 175	1.0055487	0.47396
fetal fibroblast 272	1.0062041	0.47798
fetal lung 109	1.0052271	0.47842
fetal lung 115	1.0042778	0.47900
fetal brain 32	1.0034858	0.48006
fetal muscle 186	1.0023831	0.48014
fetal kidney 87	1.0004576	0.48186
fetal muscle 168	1.0019746	0.48360
fetal muscle 172	1.0012976	0.48556
fetal muscle 190	0.9968520	0.48736
fetal adrenal gland 23	0.9989963	0.48782
fetal kidney 82	0.9924128	0.48930
fetal fibroblast 267	0.9992745	0.48994
fetal renal cortex 206	0.9950898	0.49268
fetal lung 298	0.9987961	0.49300
fetal kidney 104	0.9936504	0.49312
fetal muscle 184	0.9980186	0.49316
fetal muscle 192	0.9963367	0.49378
fetal kidney 91	0.9924794	0.49436
fetal muscle 156	0.9928022	0.49794
hematopoietic stem cell 309	0.9946057	0.50110
fetal lung 142	0.9932993	0.50406
fetal fibroblast 275	0.9918377	0.50686
fetal brain 36	0.9903412	0.50938
placenta 196	0.9843323	0.51070
fetal kidney 92	0.9834697	0.51222
fetal renal pelvis 230	0.9762994	0.51574
fetal renal cortex 211	0.9771507	0.51706
fetal brain 40	0.9869787	0.51970
fetal muscle 151	0.9784869	0.52172
fetal kidney 100	0.9768404	0.52258
fetal fibroblast 281	0.9853711	0.52550
breast 2	0.9882209	0.52604
fetal adrenal gland 28	0.9831850	0.52638
fetal muscle 148	0.9760347	0.53076
fetal thymus 257	0.9680491	0.53078
fetal muscle 162	0.9785828	0.53124
fetal muscle 191	0.9745481	0.53196
fetal fibroblast 280	0.9813813	0.53212
fetal lung 122	0.9747052	0.53548
fetal muscle 169	0.9750010	0.53800
fetal brain 31	0.9813740	0.53842
breast 1	0.9832342	0.53872
fetal fibroblast 276	0.9802813	0.53900
fetal fibroblast 279	0.9780880	0.54050
fetal fibroblast 273	0.9792487	0.54122
fetal muscle 187	0.9742714	0.54188
fetal kidney 102	0.9632090	0.54214
fetal kidney 105	0.9685785	0.54462
fetal muscle 174	0.9702472	0.54480
fetal renal cortex 205	0.9658347	0.54568
fetal lung 299	0.9738410	0.54702
fetal renal pelvis 226	0.9651056	0.54754
fetal spinal cord 236	0.9683887	0.54858
fetal muscle 144	0.9637611	0.54864
fetal renal cortex 202	0.9646159	0.54916
fetal renal pelvis 232	0.9606903	0.54988
fetal brain 38	0.9696561	0.55014
fetal renal pelvis 216	0.9603743	0.55120
fetal muscle 170	0.9672694	0.55396
fetal kidney 89	0.9591486	0.55408
fetal brain 34	0.9741300	0.55620
fetal fibroblast 269	0.9713383	0.55768
fetal renal pelvis 225	0.9541289	0.56172
fetal lung 113	0.9662311	0.56468
CD19 5	0.9512612	0.56806
keratinocyte 333	0.9729794	0.56816
hematopoietic stem cell 319	0.9550340	0.56822
fetal kidney 97	0.9525727	0.56888
fetal brain 33	0.9632731	0.56952
fetal renal pelvis 223	0.9454597	0.57468
fetal renal pelvis 233	0.9495602	0.57564
fetal muscle 161	0.9496993	0.57642
fetal kidney 107	0.9467203	0.57942
fetal muscle 193	0.9496420	0.58120
fetal muscle 177	0.9452113	0.58144
fetal muscle 145	0.9452987	0.58206
fetal renal pelvis 219	0.9324983	0.58564
keratinocyte 334	0.9654442	0.58814
fetal muscle 146	0.9495928	0.58816
fetal renal pelvis 229	0.9436088	0.58872
hematopoietic stem cell 306	0.9513358	0.58884
fetal kidney 88	0.9448137	0.58926
fetal muscle 188	0.9492703	0.58968
fetal fibroblast 270	0.9559860	0.59010
fetal kidney 94	0.9375967	0.59020
fetal brain 35	0.9618450	0.59150
fetal renal cortex 215	0.9438531	0.59272
fetal fibroblast 274	0.9559520	0.59280
fetal kidney 98	0.9350397	0.59656
fetal spinal cord 235	0.9436124	0.59974
hematopoietic stem cell 311	0.9451666	0.60268
fetal spinal cord 239	0.9428066	0.60412
fetal brain 30	0.9590592	0.60436
fetal fibroblast 282	0.9507738	0.60910
hematopoietic stem cell 307	0.9393673	0.61130
fetal kidney 81	0.9404735	0.61210
fetal kidney 96	0.9294777	0.61638
ES derived NP 291	0.9210383	0.61730
fetal kidney 86	0.9042963	0.62514
fetal renal cortex 210	0.9160866	0.62702
fetal ovary 195	0.9081125	0.62716
fetal renal cortex 208	0.9189864	0.62862
fetal renal pelvis 218	0.8923220	0.64130
fetal renal cortex 212	0.9169329	0.64320
fetal spinal cord 237	0.9148583	0.65962
fetal renal cortex 204	0.9122754	0.66010
fetal kidney 101	0.9026161	0.66314
ES derived NP 292	0.8984974	0.66342
fetal renal cortex 209	0.8976575	0.66876
fetal kidney 90	0.8954782	0.67146
fetal renal pelvis 224	0.9002204	0.67194
hematopoietic stem cell 313	0.9088003	0.68350
fetal lung 301	0.9121980	0.68582
fetal renal cortex 203	0.8854958	0.68734
fetal kidney 93	0.8823441	0.68738
fetal lung 300	0.9112542	0.69070
fetal brain 41	0.9052399	0.69264
fetal spinal cord 238	0.8985237	0.69362
hematopoietic stem cell 317	0.9077791	0.70394
fetal renal pelvis 222	0.8710950	0.70664
fetal brain 29	0.8913583	0.76440
fetal brain 39	0.8595884	0.79772

Sudden cardiac arrest

DHS sample	fold enrichment	p value
keratinocyte 333	1.4782797	0.01578
keratinocyte 334	1.4630621	0.02018
breast 1	1.5131436	0.02766
keratinocyte 335	1.3926670	0.04606
keratinocyte 336	1.3788070	0.05012
fetal lung 111	1.4288983	0.05312
fibroblast 330	1.4717268	0.05716
breast 2	1.4072826	0.05832
fibroblast 329	1.4413294	0.06310
fibroblast 331	1.4428379	0.06460
fetal fibroblast 273	1.3706813	0.06852
ES derived mesoderm 285	1.3275239	0.07546
fetal lung 298	1.4379397	0.07798
fetal lung 119	1.3476570	0.08320
fetal fibroblast 281	1.3400019	0.08572
fetal lung 141	1.3381560	0.08634
fetal fibroblast 267	1.3483967	0.08906
fetal fibroblast 274	1.3248377	0.09460
fibroblast 332	1.3827324	0.09560
fetal lung 114	1.3220339	0.09730
fetal lung 140	1.3367567	0.09910
fetal fibroblast 268	1.3160283	0.09930
fetal lung 133	1.3133128	0.10442
fetal fibroblast 277	1.3190975	0.10636
fetal fibroblast 271	1.3014997	0.10918
fetal lung 299	1.3694284	0.11162
fetal fibroblast 282	1.2785448	0.12188
fetal fibroblast 278	1.2891540	0.12786
fetal fibroblast 276	1.2732752	0.12822
fetal lung 116	1.2710880	0.13150
fetal lung 118	1.2658103	0.13552
melanocyte 338	1.2474580	0.13954
fetal lung 128	1.2557948	0.13994
fetal fibroblast 272	1.2589302	0.14814
fetal lung 301	1.3042692	0.14922
fetal fibroblast 275	1.2358207	0.15988
fetal lung 121	1.2373898	0.16428
fetal lung 300	1.2792779	0.16512
fetal fibroblast 280	1.2371734	0.17040
ES derived MSC 289	1.2777730	0.17474
fetal lung 129	1.2342629	0.18072
fetal lung 117	1.2213190	0.18508
ES derived MSC 290	1.2504201	0.19612
fetal kidney 85	1.2224967	0.19730
fetal lung 132	1.2164271	0.19948
fetal lung 115	1.2011413	0.20444
melanocyte 337	1.1783894	0.20544
ES derived mesoderm 286	1.1578967	0.21046
fetal testes 255	1.2042177	0.21488
fetal fibroblast 279	1.1940699	0.21578
Mobilized CD3 320	1.3395821	0.22166
fetal lung 109	1.1700513	0.22594
melanocyte 339	1.1675714	0.22766
fetal fibroblast 269	1.1714526	0.23232
fetal lung 126	1.1749196	0.23380
fetal lung 138	1.1796766	0.23466
fetal adrenal gland 28	1.1798155	0.24178
fetal heart 47	1.1976895	0.24886
fetal adrenal gland 27	1.1954657	0.24922
fetal fibroblast 270	1.1614193	0.24976
fetal lung 131	1.1451639	0.25876
fetal lung 120	1.1564258	0.26012
fetal lung 113	1.1431692	0.26804
fetal lung 135	1.1448299	0.27684
fetal adrenal gland 26	1.1523959	0.28312
fetal adrenal gland 24	1.1709758	0.28330
fetal lung 130	1.1251991	0.29190
fetal heart 43	1.1313618	0.29222
fetal lung 139	1.1314136	0.29316
fetal muscle 172	1.1255736	0.29410
ES derived trophoblast 288	1.1272424	0.29832
fetal muscle 176	1.1268328	0.30522
fetal lung 124	1.1097661	0.31504
fetal lung 123	1.1093025	0.31656
ES derived trophoblast 287	1.1244322	0.31724
fetal skin 234	1.1267554	0.31912
fetal lung 134	1.1079841	0.32206
fetal lung 110	1.1024033	0.32742
fetal muscle 194	1.0959153	0.33886
fetal heart 48	1.0970829	0.34168
fetal lung 136	1.0916505	0.34232
fetal kidney 91	1.0962837	0.34372
fetal adrenal gland 25	1.0936749	0.36112
fetal muscle 143	1.0746201	0.36986
fetal lung 137	1.0662807	0.37450
fetal lung 142	1.0610540	0.37614
fetal heart 45	1.0706351	0.38218
fetal muscle 179	1.0683487	0.38384
fetal adrenal gland 23	1.0488602	0.39766
CD4 13	1.0697877	0.40024
fetal lung 112	1.0436674	0.40298
fetal heart 52	1.0471200	0.40722
muscle 341	1.0469405	0.40906
fetal lung 125	1.0383717	0.41362
fetal lung 108	1.0381949	0.41498
fetal lung 127	1.0342295	0.41676
fetal heart 49	1.0330482	0.42870
fetal muscle 181	1.0327711	0.43164
fetal heart 46	1.0265681	0.43330
melanocyte 340	1.0270118	0.44354
fetal kidney 106	1.0182952	0.44380
CD3 9	1.0179552	0.44920
fetal spinal cord 235	1.0060510	0.45684
fetal renal pelvis 220	1.0100536	0.45952
fetal muscle 149	1.0086329	0.46304
fetal muscle 175	1.0043575	0.46618
Mobilized CD4 322	0.9844183	0.46666
fetal heart 42	0.9995722	0.47136
fetal muscle 166	1.0038719	0.47328
fetal renal cortex 213	0.9992244	0.47854
CD4 14	0.9861667	0.48048
fetal heart 51	0.9893930	0.48452
fetal renal cortex 207	0.9927833	0.48578
fetal lung 122	0.9724837	0.49890
fetal muscle 164	0.9776870	0.50492
fetal muscle 157	0.9772334	0.51110
fetal kidney 92	0.9667649	0.51838
fetal kidney 103	0.9657288	0.52214
fetal muscle 178	0.9617606	0.52258
fetal muscle 151	0.9543765	0.52768
fetal renal pelvis 231	0.9635520	0.52822
Mobilized CD56 324	0.9328108	0.52914
fetal muscle 152	0.9604214	0.53544
fetal muscle 184	0.9558608	0.53866
CD3 11	0.9208087	0.54070
fetal muscle 154	0.9443537	0.54282
ovary 326	0.8947394	0.54318
fetal renal cortex 215	0.9499095	0.54366
fetal renal pelvis 229	0.9457870	0.54420
fetal muscle 168	0.9459018	0.54636
fetal muscle 156	0.9458413	0.55006
stomach 284	0.9187574	0.55228
fetal muscle 167	0.9448721	0.55296
fetal muscle 169	0.9417580	0.55368
CD8 20	0.9045653	0.55686
fetal kidney 96	0.9378479	0.55688
fetal muscle 147	0.9382927	0.55842
fetal muscle 183	0.9412351	0.56166
fetal muscle 163	0.9270963	0.56516
fetal renal cortex 206	0.9272073	0.56956
fetal muscle 155	0.9183735	0.57314
Mobilized CD3 321	0.8742835	0.57560
fetal muscle 182	0.9266747	0.57714
fetal brain 31	0.9605518	0.57902
fetal muscle 165	0.9287171	0.58046
fetal stomach 248	0.9013096	0.58716
fetal renal pelvis 226	0.9130749	0.58988
fetal heart 50	0.9048781	0.59584
fetal kidney 105	0.9097800	0.59702
fetal kidney 99	0.9021525	0.59776
fetal muscle 192	0.9161431	0.59964
fetal muscle 158	0.9127258	0.60258
fetal kidney 95	0.8976882	0.60434
fetal muscle 148	0.9027421	0.60822
fetal kidney 88	0.9009347	0.61182
fetal kidney 89	0.8937310	0.61216
fetal renal cortex 214	0.9002269	0.61318
fetal renal pelvis 224	0.9020055	0.61536
fetal renal pelvis 227	0.8917622	0.61644
CD8 19	0.8288678	0.61956
fetal heart 44	0.8602017	0.62024
fetal kidney 87	0.8906300	0.62296
fetal renal cortex 204	0.8931253	0.62366
fetal muscle 161	0.8906259	0.62518
fetal muscle 171	0.8973220	0.62760
fetal muscle 188	0.8966248	0.62812
fetal muscle 187	0.8967925	0.62878
fetal muscle 160	0.8847910	0.62930
CD3 12	0.8103005	0.63752
fetal testes 254	0.8709860	0.64042
fetal renal cortex 211	0.8598259	0.64576
fetal muscle 180	0.8728301	0.64600
fetal brain 30	0.9318480	0.64792
fetal brain 32	0.9077049	0.65506
fetal muscle 189	0.8714396	0.65742
fetal stomach 244	0.8470668	0.65796
fetal brain 33	0.8904066	0.66134
fetal brain 29	0.9251738	0.66204
fetal muscle 150	0.8542301	0.66210
fetal muscle 146	0.8651248	0.66314
fetal renal pelvis 232	0.8469301	0.66390
fetal kidney 98	0.8539468	0.66462
fetal muscle 170	0.8717519	0.66582
iPS 303	0.8898640	0.66624
fetal renal cortex 202	0.8635864	0.66766
fetal spinal cord 236	0.8598735	0.66808
fetal stomach 252	0.8369626	0.66862
CD4 16	0.8015191	0.66908
fetal renal pelvis 228	0.8430906	0.66942
fetal renal cortex 210	0.8423762	0.67214
fetal muscle 186	0.8622666	0.67318
fetal stomach 243	0.8419193	0.67688
fetal kidney 100	0.8513770	0.67812
fetal renal cortex 205	0.8438256	0.68028
fetal muscle 193	0.8465150	0.68360
fetal muscle 162	0.8556657	0.68500
fetal stomach 251	0.8195806	0.68772
fetal renal pelvis 216	0.8221097	0.69082
fetal renal pelvis 221	0.8403579	0.69128
fetal renal cortex 209	0.8290897	0.69212
fetal renal cortex 212	0.8440228	0.69578
fetal muscle 190	0.8273735	0.69608
fetal muscle 174	0.8394117	0.69674
CD8 21	0.7526555	0.69746
fetal kidney 107	0.8294536	0.69982
fetal large intestine 61	0.7905566	0.70098
fetal kidney 93	0.8067950	0.70378
fetal muscle 173	0.8351994	0.70474
fetal kidney 94	0.8076570	0.70568
hematopoietic stem cell 310	0.8374136	0.70694
fetal muscle 153	0.8172719	0.70754
hematopoietic stem cell 314	0.8303152	0.70774
placenta 199	0.8137642	0.70782
fetal stomach 241	0.7864142	0.70820
fetal renal pelvis 233	0.8183731	0.70922
fetal muscle 144	0.8180906	0.70948
fetal small intestine 75	0.7627810	0.71322
Mobilized CD8 325	0.7468242	0.71700
fetal renal pelvis 222	0.8075351	0.71984
heart 297	0.7787309	0.72104
fetal renal pelvis 219	0.7608173	0.72180
placenta 200	0.8165499	0.72258
CD14 4	0.7545917	0.72342
fetal brain 34	0.8804021	0.72486
fetal renal cortex 208	0.8056163	0.72618
fetal brain 37	0.8712465	0.72966
fetal thymus 263	0.8116287	0.73014
fetal kidney 84	0.7981981	0.73498
fetal renal pelvis 217	0.7884090	0.73540
hematopoietic stem cell 318	0.7854818	0.73730
fetal kidney 101	0.7998592	0.73922
fetal brain 35	0.8772627	0.74152
fetal kidney 90	0.7956352	0.74202
CD56 17	0.7390339	0.74656
iPS 304	0.8501779	0.74930
CD8 22	0.7033988	0.75346
CD20 8	0.7741055	0.75382
fetal kidney 81	0.8142862	0.75446
Mobilized CD4 323	0.7144231	0.75666
placenta 198	0.7783613	0.75892
fetal kidney 83	0.7596389	0.75936
fetal muscle 191	0.7885941	0.76376
fetal stomach 250	0.7365426	0.76702
fetal renal cortex 203	0.7728451	0.76750
fetal kidney 104	0.7714174	0.76888
fetal muscle 177	0.7742916	0.77000
fetal renal pelvis 225	0.7639395	0.77006
placenta 201	0.7330606	0.77040
fetal brain 36	0.8407634	0.77168
hematopoietic stem cell 317	0.7897326	0.77184
fetal kidney 86	0.7298711	0.77442
fetal stomach 253	0.7269164	0.77528
fetal kidney 97	0.7740653	0.77664
fetal kidney 102	0.7541525	0.77694
fetal thymus 264	0.6846350	0.78144
fetal brain 40	0.8562812	0.78216
fetal spinal cord 238	0.7987207	0.78258
CD4 15	0.6382667	0.78414
CD14 3	0.6688733	0.78460
fetal stomach 245	0.7200922	0.78750
fetal stomach 249	0.7190917	0.78960
CD3 10	0.6667364	0.79110
hematopoietic stem cell 316	0.7402078	0.79212
fetal spinal cord 239	0.7931268	0.79534
fetal muscle 159	0.7435512	0.80052
placenta 196	0.7098989	0.80306
fetal renal pelvis 223	0.7276827	0.80550
fetal kidney 82	0.7155593	0.80692
hematopoietic stem cell 308	0.7562408	0.80714
pancreas 327	0.6906345	0.80848
fetal stomach 246	0.6882628	0.81304
fetal muscle 145	0.7189951	0.81424
hematopoietic stem cell 305	0.7228175	0.81802
CD56 18	0.6062215	0.81984
fetal renal pelvis 230	0.7077572	0.82112
hematopoietic stem cell 312	0.7227623	0.82120
fetal small intestine 70	0.7306445	0.82296
fetal thymus 265	0.6356794	0.82512
fetal brain 39	0.8174839	0.82586
pancreas 328	0.6842898	0.82672
iPS 302	0.7782926	0.82674
fetal stomach 247	0.6339715	0.82870
hematopoietic stem cell 313	0.7250234	0.83308
fetal ovary 195	0.6941070	0.83450
fetal small intestine 68	0.6964185	0.83472
fetal renal pelvis 218	0.6561331	0.83626
ES cells 293	0.7518398	0.83940
placenta 197	0.6707629	0.84222
fetal large intestine 55	0.6917705	0.84554
fetal small intestine 76	0.6429428	0.84738
hematopoietic stem cell 306	0.7046410	0.84766
fetal small intestine 72	0.6740112	0.84782
fetal muscle 185	0.6951006	0.84852
hematopoietic stem cell 307	0.7050979	0.84854
hematopoietic stem cell 311	0.7098796	0.84888
hematopoietic stem cell 309	0.6888593	0.84892
hematopoietic stem cell 319	0.6805901	0.85088
CD19 5	0.5885503	0.85150
fetal large intestine 63	0.6446182	0.85198
fetal large intestine 58	0.6859178	0.85724
fetal small intestine 78	0.6934484	0.85800
CD19 6	0.5574882	0.86354
fetal thymus 260	0.5902923	0.86362
stomach 283	0.6242433	0.86586
fetal stomach 240	0.6264394	0.86588
ES cells 294	0.7194049	0.86792
fetal thymus 257	0.5737027	0.87004
fetal thymus 266	0.5793519	0.87462
CD19 7	0.5418422	0.87860
hematopoietic stem cell 315	0.6481696	0.87978
fetal large intestine 67	0.6320237	0.88150
fetal large intestine 66	0.6194608	0.88208
fetal brain 41	0.7663085	0.88274
ES cells 296	0.6816225	0.88540
fetal brain 38	0.7616562	0.88706
fetal thymus 256	0.5538511	0.88854
fetal small intestine 80	0.6377997	0.88996
fetal large intestine 57	0.6474230	0.89056
fetal large intestine 64	0.6178871	0.89578
fetal thymus 261	0.5249785	0.90146
fetal small intestine 77	0.5979805	0.90154
fetal large intestine 60	0.5999338	0.90434
fetal thymus 258	0.5231680	0.90462
fetal large intestine 59	0.5854034	0.90768
fetal large intestine 62	0.5711525	0.90816
ES derived NP 291	0.6335752	0.91138
ES cells 295	0.6573113	0.91212
fetal spinal cord 237	0.6937395	0.91318
fetal large intestine 56	0.5825259	0.91790
fetal thymus 262	0.4656921	0.91818
fetal thymus 259	0.4780196	0.92278
fetal stomach 242	0.5206087	0.92280
fetal small intestine 69	0.5490025	0.92534
fetal small intestine 74	0.5727002	0.92686
fetal large intestine 65	0.5501099	0.92802
fetal large intestine 54	0.5513140	0.93034
fetal large intestine 53	0.5379831	0.93356
fetal small intestine 73	0.5481356	0.93466
fetal small intestine 79	0.5247048	0.93616
small intestine 342	0.5360558	0.94476
fetal small intestine 71	0.5370283	0.94722
ES derived NP 292	0.5301922	0.97612

Lipid levels 3

DHS sample	fold enrichment	p value
pancreas 328	1.2766798	0.05190
placenta 200	1.2148118	0.05788
small intestine 342	1.2430609	0.06348
placenta 198	1.2065536	0.07548
stomach 284	1.2492360	0.07658
CD14 4	1.2447557	0.08362
muscle 341	1.2374577	0.08986
fetal adrenal gland 28	1.1841722	0.09062
fetal large intestine 56	1.2088249	0.09684
fetal large intestine 62	1.2162277	0.10212
fetal large intestine 64	1.1928707	0.11060
pancreas 327	1.2087030	0.11182
fetal small intestine 76	1.2061243	0.11210
fetal large intestine 60	1.1943403	0.11394
fetal adrenal gland 24	1.1990486	0.12052
CD20 8	1.1778765	0.12112
fetal large intestine 65	1.1923005	0.12132
fetal small intestine 79	1.1981582	0.12368
fetal small intestine 78	1.1669844	0.12606
fetal small intestine 74	1.1760511	0.13292
fetal thymus 260	1.1908745	0.14368
fetal small intestine 69	1.1774718	0.14726
fetal small intestine 73	1.1670727	0.14914
fetal thymus 258	1.1860025	0.14992
fetal large intestine 54	1.1647269	0.15206
placenta 201	1.1615304	0.15292
fetal large intestine 55	1.1553986	0.15370
fetal small intestine 72	1.1610370	0.15456
placenta 197	1.1510054	0.16180
fetal thymus 256	1.1673799	0.16206
fetal adrenal gland 25	1.1560390	0.16466
CD14 3	1.1764367	0.16802
fetal small intestine 71	1.1559126	0.16812
fetal thymus 265	1.1670479	0.17174
fetal large intestine 57	1.1408099	0.17230
fetal large intestine 58	1.1390750	0.17252
fetal small intestine 70	1.1321118	0.17334
fetal large intestine 59	1.1492556	0.18128
keratinocyte 333	1.0984117	0.18264
melanocyte 338	1.1030676	0.19048
fibroblast 329	1.1083259	0.19496
fetal small intestine 80	1.1312592	0.19694
fetal large intestine 63	1.1352888	0.20096
fetal small intestine 77	1.1339932	0.20140
melanocyte 337	1.0960853	0.20184
fibroblast 332	1.1007027	0.20708
fibroblast 330	1.1041432	0.20782
fetal large intestine 53	1.1372807	0.21008
ES derived mesoderm 286	1.0979123	0.21234
ES derived trophoblast 288	1.1115076	0.21596
keratinocyte 336	1.0861012	0.22064
fetal small intestine 68	1.1104838	0.23008
keratinocyte 334	1.0795185	0.23206
fetal large intestine 67	1.1118601	0.23408
CD4 13	1.1318976	0.24570
placenta 199	1.0971461	0.24714
Mobilized CD56 324	1.1190933	0.24848
placenta 196	1.0983127	0.25136
fetal adrenal gland 23	1.0914789	0.25340
stomach 283	1.1095779	0.25354
melanocyte 340	1.0821452	0.25990
ES derived MSC 289	1.0893399	0.26286
fetal lung 300	1.0829747	0.26378
breast 1	1.0722619	0.27136
ES derived MSC 290	1.0848635	0.27154
fetal thymus 259	1.1064493	0.27282
breast 2	1.0707238	0.27726
fetal large intestine 66	1.0937617	0.27764
fibroblast 331	1.0729351	0.27868
fetal adrenal gland 26	1.0830441	0.28432
keratinocyte 335	1.0618900	0.29064
ES cells 294	1.0899309	0.29544
fetal lung 129	1.0687812	0.29932
fetal lung 301	1.0696853	0.29972
fetal lung 119	1.0671559	0.29972
fetal thymus 264	1.0836855	0.30278
fetal thymus 262	1.0969543	0.31010
fetal adrenal gland 27	1.0720037	0.31126
fetal lung 132	1.0640977	0.31168
fetal lung 140	1.0629217	0.31458
CD8 21	1.0904521	0.31592
fetal lung 299	1.0656763	0.31606
fetal large intestine 61	1.0837709	0.31816
fetal lung 134	1.0619614	0.32004
CD4 15	1.0973995	0.32058
Mobilized CD8 325	1.0856653	0.32106
fetal stomach 253	1.0743532	0.32142
CD3 9	1.0839254	0.32224
ES derived mesoderm 285	1.0580472	0.32818
fetal stomach 244	1.0671674	0.33304
fetal stomach 242	1.0769475	0.33352
fetal ovary 195	1.0718669	0.33352
CD56 17	1.0673882	0.33582
fetal thymus 266	1.0673385	0.33706
Mobilized CD4 323	1.0702301	0.34402
fetal skin 234	1.0589277	0.34448
CD56 18	1.0717636	0.34484
Mobilized CD3 321	1.0730536	0.34558
fetal lung 118	1.0478039	0.34660
CD8 19	1.0716265	0.35418
fetal thymus 261	1.0615059	0.35530
fetal brain 41	1.0440739	0.35596
CD4 14	1.0599819	0.35642
fetal lung 298	1.0496840	0.35686
ES cells 296	1.0582896	0.35890
heart 297	1.0530934	0.36164
fetal fibroblast 269	1.0414956	0.36582
fetal fibroblast 280	1.0403328	0.37000
fetal renal pelvis 219	1.0532321	0.37208
ovary 326	1.0538273	0.37266
fetal lung 123	1.0402173	0.37608
ES derived trophoblast 287	1.0453043	0.37768
fetal lung 125	1.0397792	0.37788
fetal renal pelvis 231	1.0387179	0.37882
fetal lung 120	1.0391114	0.37972
iPS 304	1.0391491	0.38102
Mobilized CD4 322	1.0526605	0.38292
fetal renal cortex 212	1.0383016	0.38504
fetal lung 111	1.0367150	0.38508
fetal lung 136	1.0373581	0.38656
fetal thymus 257	1.0476255	0.38696
fetal stomach 247	1.0468182	0.38782
fetal kidney 106	1.0357555	0.38950
fetal fibroblast 279	1.0325187	0.39364
fetal stomach 250	1.0384181	0.39736
CD3 11	1.0404171	0.39772
melanocyte 339	1.0278758	0.40130
fetal kidney 83	1.0336374	0.40316
fetal fibroblast 273	1.0283749	0.40386
fetal lung 133	1.0293465	0.40404
fetal stomach 248	1.0355701	0.40406
fetal thymus 263	1.0329302	0.40570
fetal spinal cord 236	1.0291639	0.40748
fetal fibroblast 282	1.0262189	0.41064
fetal fibroblast 274	1.0270313	0.41160
fetal renal pelvis 220	1.0272201	0.41306
fetal lung 112	1.0269727	0.41310
fetal testes 254	1.0287421	0.41360
fetal renal pelvis 233	1.0264706	0.41446
fetal lung 128	1.0241051	0.41566
fetal kidney 89	1.0265893	0.41636
Mobilized CD3 320	1.0350379	0.41706
fetal brain 38	1.0246771	0.41778
fetal lung 109	1.0225458	0.41778
CD8 20	1.0312815	0.41836
fetal renal pelvis 232	1.0251033	0.42228
CD8 22	1.0297773	0.42290
fetal renal pelvis 228	1.0246335	0.42614
fetal lung 138	1.0218842	0.42814
fetal renal pelvis 227	1.0178205	0.43496
fetal fibroblast 268	1.0183487	0.43524
fetal lung 141	1.0178492	0.43542
fetal brain 34	1.0168290	0.43606
fetal renal pelvis 224	1.0184684	0.43654
CD4 16	1.0193848	0.44044
fetal renal cortex 205	1.0165791	0.44096
fetal renal cortex 214	1.0155588	0.44096
fetal spinal cord 237	1.0182337	0.44120
fetal renal pelvis 217	1.0169598	0.44238
fetal lung 117	1.0159561	0.44350
fetal small intestine 75	1.0135964	0.44678
fetal renal cortex 213	1.0118864	0.44918
fetal lung 121	1.0136658	0.44926
fetal kidney 91	1.0112762	0.44934
fetal kidney 96	1.0121836	0.45018
fetal kidney 82	1.0136103	0.45040
fetal kidney 103	1.0106080	0.45098
fetal renal pelvis 218	1.0140227	0.45124
fetal lung 130	1.0140414	0.45136
fetal lung 108	1.0123827	0.45430
fetal stomach 240	1.0115056	0.45888
fetal stomach 241	1.0098780	0.46308
fetal kidney 87	1.0082782	0.46454
fetal kidney 86	1.0078394	0.46534
fetal renal pelvis 221	1.0060608	0.46556
fetal brain 40	1.0065817	0.46632
fetal fibroblast 281	1.0086923	0.46848
fetal lung 127	1.0066579	0.46920
fetal kidney 81	1.0065859	0.46964
ES cells 293	1.0054837	0.47030
CD3 12	1.0059565	0.47044
fetal stomach 246	1.0051627	0.47118
iPS 302	1.0044992	0.47268
fetal kidney 88	1.0051324	0.47290
fetal kidney 95	1.0013625	0.47414
fetal kidney 100	1.0030655	0.47594
fetal muscle 194	1.0048304	0.47618
fetal muscle 184	1.0050148	0.47628
fetal stomach 252	1.0034443	0.47636
fetal lung 113	1.0048472	0.47788
fetal lung 114	1.0049988	0.47828
fetal lung 137	1.0038119	0.47892
ES cells 295	1.0004762	0.48170
fetal brain 37	1.0027469	0.48224
fetal brain 39	1.0025483	0.48258
fetal lung 110	1.0024969	0.48346
hematopoietic stem cell 310	1.0038745	0.48354
fetal muscle 151	1.0004349	0.48670
fetal renal cortex 207	0.9949968	0.49010
fetal muscle 169	0.9990496	0.49340
fetal fibroblast 270	0.9987720	0.49478
fetal kidney 105	0.9947338	0.49502
fetal renal cortex 211	0.9946244	0.49502
fetal brain 35	0.9975999	0.50146
iPS 303	0.9922995	0.50304
fetal kidney 104	0.9909339	0.50438
fetal brain 36	0.9961160	0.50514
hematopoietic stem cell 312	0.9947450	0.50598
fetal renal pelvis 225	0.9903318	0.50710
fetal muscle 193	0.9912662	0.51362
CD3 10	0.9878345	0.51496
fetal stomach 243	0.9890377	0.51666
fetal fibroblast 278	0.9913561	0.51828
fetal renal cortex 202	0.9853983	0.51908
fetal muscle 145	0.9862916	0.51942
fetal kidney 99	0.9844725	0.52132
fetal muscle 189	0.9876863	0.52196
fetal lung 135	0.9888732	0.52204
fetal lung 124	0.9874583	0.52468
fetal renal pelvis 229	0.9832094	0.52544
fetal kidney 92	0.9805503	0.52636
fetal brain 31	0.9907634	0.52706
fetal kidney 101	0.9830487	0.52718
fetal fibroblast 267	0.9878507	0.52984
fetal lung 131	0.9890220	0.53030
fetal muscle 154	0.9835132	0.53046
fetal stomach 249	0.9774335	0.53236
fetal kidney 84	0.9787562	0.53320
fetal muscle 153	0.9821977	0.53332
fetal renal pelvis 226	0.9796456	0.53340
fetal stomach 251	0.9820730	0.53402
fetal muscle 174	0.9830855	0.53494
fetal muscle 155	0.9807279	0.53682
fetal muscle 182	0.9823976	0.53712
fetal muscle 185	0.9812854	0.53764
fetal lung 139	0.9807690	0.54332
fetal renal pelvis 223	0.9737758	0.54378
hematopoietic stem cell 316	0.9778667	0.54382
fetal muscle 150	0.9794716	0.54426
CD19 7	0.9698794	0.54606
fetal testes 255	0.9787627	0.55008
ES derived NP 292	0.9717845	0.55158
fetal renal cortex 210	0.9714857	0.55250
CD19 6	0.9669555	0.55250
fetal renal cortex 206	0.9737414	0.55350
fetal kidney 102	0.9671689	0.55568
fetal fibroblast 277	0.9793353	0.55578
hematopoietic stem cell 315	0.9751460	0.55796
hematopoietic stem cell 318	0.9721379	0.55804
fetal muscle 167	0.9749172	0.55988
fetal renal cortex 215	0.9690527	0.56282
fetal spinal cord 238	0.9720490	0.56314
fetal muscle 157	0.9742765	0.56400
fetal lung 126	0.9766254	0.56650
CD19 5	0.9625079	0.56694
hematopoietic stem cell 305	0.9713109	0.56702
hematopoietic stem cell 314	0.9746030	0.56760
fetal muscle 173	0.9694786	0.57160
fetal muscle 164	0.9691976	0.57258
fetal fibroblast 271	0.9735469	0.57414
fetal muscle 171	0.9700318	0.57556
fetal muscle 175	0.9694444	0.57572
hematopoietic stem cell 308	0.9695353	0.57732
ES derived NP 291	0.9581200	0.58226
fetal muscle 179	0.9644934	0.58312
fetal kidney 90	0.9594924	0.58446
fetal muscle 149	0.9620496	0.58676
fetal kidney 98	0.9568712	0.58712
fetal lung 122	0.9650814	0.58886
fetal heart 46	0.9595070	0.58992
fetal fibroblast 275	0.9689240	0.59012
fetal muscle 187	0.9625932	0.59412
fetal brain 32	0.9653819	0.59440
fetal muscle 159	0.9602083	0.59466
fetal muscle 180	0.9580359	0.59496
fetal renal pelvis 216	0.9547568	0.59554
fetal lung 116	0.9681904	0.59676
fetal lung 115	0.9658613	0.59684
fetal muscle 183	0.9631754	0.59710
fetal muscle 170	0.9613396	0.59760
fetal muscle 191	0.9587073	0.59830
fetal renal cortex 204	0.9583143	0.59848
fetal muscle 144	0.9561115	0.60292
hematopoietic stem cell 307	0.9572925	0.60418
fetal muscle 148	0.9583930	0.60524
fetal brain 29	0.9680392	0.60660
fetal renal cortex 208	0.9479783	0.60974
fetal muscle 186	0.9552613	0.61004
fetal heart 43	0.9554540	0.61192
fetal muscle 156	0.9543933	0.61462
fetal kidney 97	0.9468883	0.61716
fetal muscle 147	0.9516325	0.61734
fetal heart 44	0.9397895	0.61968
fetal renal cortex 203	0.9458328	0.61978
fetal muscle 168	0.9505535	0.62026
fetal renal pelvis 222	0.9424678	0.62158
fetal kidney 107	0.9449905	0.62188
fetal stomach 245	0.9421649	0.62434
fetal fibroblast 272	0.9557938	0.62468
fetal lung 142	0.9576375	0.62536
fetal muscle 181	0.9466589	0.62568
fetal muscle 188	0.9502399	0.62752
hematopoietic stem cell 306	0.9489440	0.62844
fetal renal pelvis 230	0.9371035	0.62844
hematopoietic stem cell 309	0.9490475	0.62908
hematopoietic stem cell 313	0.9498799	0.62952
fetal muscle 166	0.9461242	0.63436
fetal muscle 158	0.9474626	0.63712
fetal heart 45	0.9412304	0.63864
fetal spinal cord 239	0.9433282	0.63988
fetal renal cortex 209	0.9352329	0.64134
fetal muscle 178	0.9379069	0.64698
fetal heart 42	0.9399841	0.64764
fetal muscle 146	0.9430039	0.64962
fetal muscle 192	0.9416916	0.65002
fetal muscle 177	0.9343342	0.65032
fetal muscle 152	0.9405404	0.65138
fetal muscle 160	0.9377518	0.65256
fetal fibroblast 276	0.9461831	0.65550
fetal brain 30	0.9541917	0.65888
fetal muscle 190	0.9284562	0.66680
fetal spinal cord 235	0.9327212	0.66840
fetal muscle 172	0.9410331	0.66868
fetal kidney 85	0.9303919	0.66978
fetal muscle 165	0.9363029	0.66984
fetal kidney 94	0.9179270	0.67146
fetal muscle 176	0.9308483	0.67412
fetal brain 33	0.9293105	0.68792
fetal muscle 162	0.9272604	0.68928
fetal muscle 161	0.9212857	0.69210
hematopoietic stem cell 319	0.9157909	0.69638
fetal muscle 143	0.9255668	0.70056
hematopoietic stem cell 311	0.9225799	0.70288
fetal kidney 93	0.9069077	0.70548
fetal heart 52	0.9221875	0.70898
fetal muscle 163	0.9161898	0.71098
fetal heart 51	0.9134686	0.71294
fetal heart 49	0.9094299	0.71730
hematopoietic stem cell 317	0.9223055	0.72596
fetal heart 50	0.9028956	0.73938
fetal heart 48	0.8942265	0.75686
fetal heart 47	0.8804833	0.75982

Blood metabolites 2

DHS sample	fold enrichment	p value
small intestine 342	1.1467022	0.01666
fetal large intestine 58	1.1221670	0.02714
fetal small intestine 78	1.1178477	0.03114
fetal small intestine 80	1.1246779	0.03284
fetal small intestine 74	1.1215092	0.03964
fetal thymus 258	1.1296700	0.04652
fetal large intestine 60	1.1121304	0.05530
fetal large intestine 64	1.1060506	0.05762
CD20 8	1.1022067	0.05970
fetal thymus 265	1.1188817	0.06260
fetal small intestine 69	1.1125642	0.06580
fetal thymus 256	1.1120460	0.06598
fetal large intestine 57	1.0945322	0.06746
fetal large intestine 55	1.0981333	0.06766
fetal small intestine 72	1.1025952	0.07020
fetal small intestine 70	1.0889535	0.07318
fetal thymus 260	1.1120117	0.07364
fetal thymus 266	1.1105968	0.07594
fetal small intestine 68	1.0923775	0.07938
fetal thymus 264	1.1033920	0.08476
fetal large intestine 54	1.0952378	0.08778
fetal thymus 259	1.1098990	0.09036
fetal large intestine 67	1.0924385	0.09592
fetal large intestine 56	1.0877813	0.10038
fetal small intestine 73	1.0897938	0.10290
Mobilized CD56 324	1.0965710	0.10360
fetal large intestine 63	1.0882726	0.11048
hematopoietic stem cell 310	1.0670751	0.11396
CD3 9	1.0987661	0.11758
fetal small intestine 71	1.0802797	0.12770
pancreas 328	1.0811250	0.13080
hematopoietic stem cell 309	1.0672849	0.14646
fetal small intestine 79	1.0744521	0.15294
Mobilized CD4 323	1.0848639	0.16100
CD8 21	1.0829953	0.16718
fetal large intestine 62	1.0710969	0.16796
hematopoietic stem cell 314	1.0574085	0.16844
CD4 13	1.0811295	0.16998
fetal thymus 261	1.0721714	0.17520
Mobilized CD4 322	1.0788888	0.18150
fetal thymus 257	1.0729072	0.18622
CD4 15	1.0878632	0.19132
hematopoietic stem cell 318	1.0586912	0.19384
hematopoietic stem cell 315	1.0546320	0.20166
hematopoietic stem cell 312	1.0507119	0.20402
fetal small intestine 76	1.0591139	0.20440
fetal small intestine 77	1.0573622	0.20504
fetal thymus 262	1.0734249	0.21510
CD3 11	1.0584677	0.21756
hematopoietic stem cell 305	1.0500752	0.22072
CD8 20	1.0588130	0.22136
Mobilized CD8 325	1.0663087	0.22214
fetal large intestine 65	1.0518304	0.22938
CD8 22	1.0608339	0.23864
CD3 12	1.0602390	0.24160
fetal large intestine 59	1.0493960	0.24240
hematopoietic stem cell 316	1.0460169	0.24758
fetal large intestine 61	1.0544661	0.25028
CD8 19	1.0583837	0.25544
placenta 200	1.0383692	0.26010
CD4 14	1.0473232	0.26140
CD56 18	1.0530345	0.26224
hematopoietic stem cell 308	1.0381971	0.26386
stomach 284	1.0456397	0.26518
placenta 198	1.0362247	0.27570
stomach 283	1.0385845	0.29564
hematopoietic stem cell 311	1.0330902	0.29870
hematopoietic stem cell 306	1.0328022	0.29996
Mobilized CD3 320	1.0439597	0.30590
CD3 10	1.0381517	0.30694
Mobilized CD3 321	1.0421419	0.31392
melanocyte 338	1.0242874	0.31422
CD19 7	1.0398672	0.31618
placenta 199	1.0294631	0.31866
pancreas 327	1.0346911	0.31876
CD56 17	1.0327755	0.32022
fetal large intestine 66	1.0308065	0.32942
CD19 5	1.0341755	0.33152
CD19 6	1.0331486	0.34278
melanocyte 340	1.0198988	0.36048
fetal small intestine 75	1.0338876	0.36076
placenta 197	1.0221785	0.36782
muscle 341	1.0228047	0.37342
hematopoietic stem cell 307	1.0185378	0.37896
CD4 16	1.0244509	0.38240
CD14 4	1.0199108	0.38876
hematopoietic stem cell 313	1.0162774	0.39034
melanocyte 337	1.0110909	0.40958
fetal large intestine 53	1.0148619	0.41552
CD14 3	1.0131266	0.42976
hematopoietic stem cell 319	1.0063994	0.45990
hematopoietic stem cell 317	1.0043299	0.46628
fetal stomach 244	1.0044732	0.46952
fetal adrenal gland 25	1.0031833	0.47610
ES cells 294	1.0007993	0.48728
fetal adrenal gland 28	1.0001009	0.49760
iPS 304	0.9993305	0.49980
fetal stomach 252	0.9983047	0.50152
melanocyte 339	0.9992562	0.50392
fetal testes 254	0.9968334	0.51392
fetal adrenal gland 24	0.9939925	0.52692
placenta 196	0.9921260	0.53996
fetal testes 255	0.9924921	0.54326
placenta 201	0.9888443	0.55784
fetal stomach 242	0.9841250	0.56404
fetal stomach 241	0.9856245	0.56494
fetal adrenal gland 27	0.9872727	0.57382
fetal stomach 247	0.9823810	0.57610
fetal ovary 195	0.9805435	0.58542
ES cells 296	0.9818140	0.58904
fetal heart 45	0.9843857	0.59204
fetal stomach 253	0.9777749	0.60608
heart 297	0.9790112	0.61606
fetal adrenal gland 26	0.9792906	0.61958
iPS 302	0.9784360	0.62666
ovary 326	0.9716376	0.63186
fetal lung 298	0.9781933	0.63760
fibroblast 332	0.9795008	0.64114
fetal adrenal gland 23	0.9766890	0.64210
fetal heart 44	0.9696078	0.65022
fetal stomach 246	0.9686712	0.65298
fetal stomach 240	0.9681354	0.65324
fibroblast 331	0.9776525	0.65422
iPS 303	0.9736273	0.65920
fetal stomach 250	0.9645436	0.67224
ES cells 295	0.9654209	0.67258
fetal lung 299	0.9720632	0.67346
fetal stomach 249	0.9627674	0.67442
fibroblast 329	0.9734638	0.67928
fetal stomach 248	0.9624112	0.68748
fetal heart 47	0.9637643	0.68970
fibroblast 330	0.9710042	0.69030
fetal stomach 251	0.9636711	0.69062
breast 2	0.9703649	0.70684
ES derived MSC 290	0.9649869	0.71092
keratinocyte 336	0.9717998	0.71438
fetal heart 46	0.9616727	0.71540
fetal lung 301	0.9654923	0.71984
fetal stomach 245	0.9567457	0.71988
ES derived MSC 289	0.9626134	0.72348
fetal heart 49	0.9591936	0.73142
keratinocyte 333	0.9686771	0.73772
fetal heart 42	0.9580620	0.73918
breast 1	0.9651421	0.74142
ES derived NP 291	0.9505198	0.74190
keratinocyte 335	0.9672671	0.74206
fetal stomach 243	0.9497719	0.76724
ES cells 293	0.9470797	0.76754
ES derived NP 292	0.9452471	0.76970
ES derived trophoblast 288	0.9540911	0.77622
fetal heart 51	0.9512283	0.77908
fetal lung 300	0.9527218	0.79130
fetal renal pelvis 219	0.9327871	0.79366
fetal heart 52	0.9481236	0.80334
fetal heart 48	0.9444801	0.80514
fetal heart 43	0.9470496	0.80796
ES derived mesoderm 286	0.9479760	0.83778
ES derived mesoderm 285	0.9438872	0.83934
fetal kidney 83	0.9243712	0.84132
fetal heart 50	0.9299003	0.86196
fetal lung 108	0.9308387	0.86498
fetal renal cortex 211	0.9201116	0.86654
ES derived trophoblast 287	0.9269451	0.86732
fetal kidney 106	0.9257748	0.86748
fetal renal pelvis 216	0.9194079	0.87738
keratinocyte 334	0.9418701	0.87948
fetal kidney 86	0.9061984	0.88120
fetal renal pelvis 228	0.9145387	0.88258
fetal kidney 82	0.9098213	0.88844
fetal kidney 99	0.9189129	0.89060
fetal renal pelvis 218	0.9002243	0.89110
fetal muscle 149	0.9154922	0.89768
fetal renal pelvis 217	0.9094715	0.90372
fetal spinal cord 236	0.9167927	0.90380
fetal kidney 84	0.9096614	0.90434
fetal lung 127	0.9196823	0.90846
fetal renal pelvis 220	0.9084084	0.91004
fetal kidney 95	0.9094235	0.91238
fetal lung 134	0.9182082	0.91304
fetal lung 125	0.9176293	0.91360
fetal lung 122	0.9141445	0.91542
fetal lung 129	0.9192637	0.91562
fetal lung 139	0.9162383	0.91598
fetal lung 123	0.9180168	0.91800
fetal renal pelvis 233	0.9039559	0.92184
fetal renal pelvis 230	0.8975279	0.92252
fetal renal pelvis 232	0.8992622	0.92308
fetal kidney 103	0.9020868	0.92420
fetal lung 132	0.9146479	0.92594
fetal kidney 104	0.9011025	0.92700
fetal lung 137	0.9163419	0.92886
fetal renal pelvis 225	0.8993409	0.92890
fetal fibroblast 280	0.9195327	0.93046
fetal renal pelvis 223	0.8949770	0.93090
fetal lung 138	0.9113664	0.93098
fetal lung 115	0.9166984	0.93230
fetal kidney 87	0.9018660	0.93366
fetal kidney 102	0.8931325	0.93494
fetal kidney 107	0.8992786	0.93542
fetal kidney 90	0.8968817	0.93608
fetal fibroblast 270	0.9156955	0.93620
fetal lung 130	0.9116308	0.93684
fetal renal cortex 214	0.8986267	0.93810
fetal renal cortex 207	0.8972269	0.93866
fetal kidney 89	0.8954039	0.93952
fetal fibroblast 269	0.9174254	0.93998
fetal muscle 177	0.8944426	0.94176
fetal muscle 164	0.9004082	0.94180
fetal renal cortex 206	0.8988415	0.94196
fetal kidney 100	0.8960019	0.94270
fetal kidney 94	0.8879106	0.94362
fetal renal cortex 205	0.8955796	0.94452
fetal muscle 151	0.8936591	0.94480
fetal kidney 97	0.8940441	0.94494
fetal lung 121	0.9116154	0.94644
fetal muscle 145	0.8884071	0.94682
fetal muscle 159	0.8922561	0.94772
fetal muscle 148	0.8949822	0.94794
fetal lung 133	0.9097592	0.94800
fetal muscle 150	0.8940890	0.94868
fetal kidney 93	0.8855363	0.94990
fetal lung 109	0.9134877	0.95050
fetal lung 142	0.9095716	0.95074
fetal fibroblast 271	0.9108456	0.95278
fetal lung 140	0.9055984	0.95284
fetal muscle 193	0.8913423	0.95294
fetal lung 126	0.9088239	0.95376
fetal renal pelvis 231	0.8928736	0.95464
fetal renal cortex 213	0.8933949	0.95610
fetal skin 234	0.8889742	0.95612
fetal lung 117	0.9041257	0.95614
fetal lung 116	0.9099158	0.95622
fetal muscle 191	0.8908536	0.95670
fetal lung 112	0.9010101	0.95686
fetal fibroblast 276	0.9086498	0.95688
fetal lung 136	0.8980866	0.95692
fetal lung 131	0.9103012	0.95704
fetal muscle 154	0.8834618	0.95766
fetal fibroblast 279	0.9055716	0.95890
fetal fibroblast 282	0.9087624	0.95896
fetal fibroblast 275	0.9084775	0.95908
fetal lung 124	0.8978196	0.95972
fetal muscle 190	0.8800869	0.96086
fetal fibroblast 281	0.9056515	0.96132
fetal fibroblast 278	0.9016302	0.96276
fetal kidney 88	0.8859822	0.96282
fetal thymus 263	0.8862351	0.96390
fetal kidney 98	0.8792172	0.96434
fetal renal pelvis 227	0.8813297	0.96438
fetal kidney 101	0.8835962	0.96446
fetal fibroblast 272	0.9025777	0.96476
fetal muscle 153	0.8791994	0.96594
fetal renal cortex 212	0.8842471	0.96598
fetal lung 128	0.9050376	0.96674
fetal renal cortex 210	0.8723662	0.96734
fetal muscle 175	0.8861371	0.96738
fetal renal pelvis 226	0.8811159	0.96826
fetal lung 110	0.8905013	0.96832
fetal lung 118	0.8998009	0.96866
fetal muscle 174	0.8825214	0.96894
fetal muscle 156	0.8846810	0.96916
fetal muscle 180	0.8790007	0.96918
fetal fibroblast 267	0.8951774	0.96956
fetal muscle 168	0.8796805	0.96960
fetal lung 119	0.8976471	0.96980
fetal muscle 169	0.8824664	0.97004
fetal renal cortex 202	0.8781752	0.97074
fetal renal pelvis 221	0.8791310	0.97098
fetal fibroblast 274	0.8961437	0.97106
fetal muscle 171	0.8844413	0.97162
fetal muscle 173	0.8791823	0.97178
fetal renal cortex 208	0.8706217	0.97192
fetal muscle 155	0.8748373	0.97228
fetal kidney 105	0.8773436	0.97260
fetal renal cortex 203	0.8721070	0.97296
fetal kidney 92	0.8778588	0.97342
fetal fibroblast 268	0.8976831	0.97360
fetal muscle 192	0.8821546	0.97390
fetal muscle 147	0.8753189	0.97418
fetal lung 111	0.8891322	0.97566
fetal muscle 188	0.8784918	0.97580
fetal muscle 182	0.8803313	0.97610
fetal lung 120	0.8869935	0.97626
fetal renal cortex 209	0.8679229	0.97626
fetal lung 113	0.8923167	0.97644
fetal renal pelvis 224	0.8746883	0.97720
fetal lung 135	0.8830592	0.97724
fetal brain 36	0.8920053	0.97740
fetal kidney 96	0.8724726	0.97754
fetal muscle 194	0.8871275	0.97792
fetal brain 38	0.8879705	0.97796
fetal kidney 91	0.8747175	0.97800
fetal spinal cord 238	0.8795288	0.97810
fetal renal pelvis 229	0.8697336	0.97878
fetal renal cortex 215	0.8721782	0.97910
fetal muscle 167	0.8764468	0.97974
fetal renal pelvis 222	0.8600253	0.98048
fetal spinal cord 237	0.8777642	0.98086
fetal muscle 186	0.8733919	0.98150
fetal lung 141	0.8881629	0.98158
fetal spinal cord 239	0.8744260	0.98218
fetal muscle 163	0.8640858	0.98238
fetal muscle 160	0.8665842	0.98242
fetal fibroblast 277	0.8841277	0.98244
fetal spinal cord 235	0.8740511	0.98348
fetal renal cortex 204	0.8696377	0.98350
fetal brain 37	0.8858773	0.98384
fetal fibroblast 273	0.8855226	0.98392
fetal muscle 162	0.8712529	0.98394
fetal brain 41	0.8850726	0.98468
fetal muscle 146	0.8671853	0.98478
fetal muscle 183	0.8713449	0.98492
fetal muscle 187	0.8705960	0.98542
fetal brain 40	0.8891071	0.98548
fetal muscle 170	0.8696473	0.98574
fetal muscle 152	0.8684366	0.98610
fetal muscle 166	0.8685799	0.98614
fetal muscle 189	0.8618379	0.98680
fetal muscle 185	0.8571092	0.98680
fetal kidney 85	0.8686285	0.98846
fetal muscle 144	0.8528129	0.98868
fetal kidney 81	0.8651891	0.98892
fetal muscle 176	0.8631108	0.99010
fetal muscle 161	0.8525581	0.99050
fetal muscle 184	0.8624456	0.99106
fetal muscle 158	0.8581296	0.99106
fetal lung 114	0.8739265	0.99112
fetal muscle 179	0.8555544	0.99152
fetal muscle 143	0.8627664	0.99168
fetal brain 39	0.8753930	0.99254
fetal muscle 172	0.8642688	0.99322
fetal muscle 157	0.8560389	0.99338
fetal muscle 178	0.8441399	0.99372
fetal muscle 181	0.8432465	0.99478
fetal muscle 165	0.8490768	0.99518
fetal brain 29	0.8811486	0.99608
fetal brain 35	0.8732449	0.99610
fetal brain 33	0.8461005	0.99752
fetal brain 32	0.8474738	0.99806
fetal brain 34	0.8557018	0.99854
fetal brain 30	0.8662303	0.99882
fetal brain 31	0.8444718	0.99966

Fasting glucose and Type 2 diabetes

DHS sample	fold enrichment	p value
fetal heart 42	1.4611431	0.01814
fetal heart 46	1.4704313	0.02234
fetal heart 47	1.5131007	0.02408
fetal heart 52	1.3812325	0.02754
fetal heart 48	1.4212639	0.02792
fetal heart 45	1.4319405	0.03004
fetal heart 44	1.4854520	0.03402
fetal heart 49	1.4095201	0.03504
fetal heart 50	1.3696695	0.04528
fetal heart 43	1.3431562	0.04670
fetal heart 51	1.3445049	0.05408
pancreas 328	1.2913658	0.10190
pancreas 327	1.2741874	0.12090
heart 297	1.1839111	0.20160
muscle 341	1.1986944	0.20236
fetal large intestine 58	1.1673056	0.20318
fetal adrenal gland 28	1.1502228	0.20674
ES derived mesoderm 286	1.1178209	0.21890
ES derived mesoderm 285	1.1282294	0.22542
fetal small intestine 74	1.1521883	0.23548
fetal large intestine 67	1.1521259	0.24316
fetal small intestine 68	1.1392137	0.24666
fetal small intestine 70	1.1276437	0.25152
fetal spinal cord 239	1.1067648	0.26274
ES derived trophoblast 287	1.1278831	0.26278
fetal adrenal gland 25	1.1298433	0.26884
CD20 8	1.1223163	0.27044
fetal testes 255	1.1114574	0.27148
fetal brain 32	1.0886860	0.27654
fetal adrenal gland 23	1.1063535	0.28274
small intestine 342	1.1183151	0.28508
ES derived trophoblast 288	1.1040547	0.28550
fetal testes 254	1.1119298	0.28664
fetal ovary 195	1.1234885	0.28902
fetal small intestine 78	1.1069265	0.28902
fetal large intestine 62	1.1194233	0.29162
fetal large intestine 63	1.1164163	0.29358
breast 2	1.0835565	0.29464
fetal small intestine 69	1.1170176	0.29610
fetal small intestine 72	1.1072623	0.29892
fetal large intestine 66	1.1097831	0.29948
fetal stomach 244	1.1003754	0.31250
stomach 284	1.0999209	0.31748
fetal large intestine 55	1.0887481	0.32356
iPS 304	1.0775588	0.32632
ES cells 294	1.0902942	0.32760
fetal spinal cord 237	1.0732772	0.32852
fetal small intestine 73	1.0916256	0.32858
fetal large intestine 54	1.0895616	0.32954
fetal adrenal gland 24	1.0932127	0.33200
fetal large intestine 60	1.0878964	0.33206
fetal adrenal gland 26	1.0820327	0.33374
fetal spinal cord 236	1.0724088	0.33782
fetal brain 37	1.0593544	0.33948
fetal muscle 193	1.0722109	0.34076
ES cells 293	1.0749472	0.34388
fetal stomach 246	1.0810600	0.34862
fetal large intestine 56	1.0771255	0.34942
fetal large intestine 57	1.0714228	0.35112
fetal stomach 248	1.0783257	0.35116
fetal muscle 168	1.0667356	0.35512
ovary 326	1.0772595	0.36106
fetal spinal cord 235	1.0564112	0.36132
fetal small intestine 79	1.0700687	0.36666
fetal small intestine 80	1.0660083	0.36750
ES derived NP 292	1.0619101	0.36906
fetal small intestine 71	1.0643541	0.36982
stomach 283	1.0672782	0.37102
fetal large intestine 64	1.0625458	0.37316
fetal stomach 242	1.0687532	0.37440
iPS 302	1.0511752	0.37958
fetal muscle 192	1.0441137	0.38976
fetal muscle 163	1.0446286	0.39060
fibroblast 330	1.0436081	0.39446
fetal muscle 165	1.0408826	0.39852
fetal spinal cord 238	1.0397241	0.40034
fetal stomach 252	1.0445009	0.40120
ES derived NP 291	1.0440079	0.40382
fetal stomach 247	1.0480396	0.40444
fetal stomach 251	1.0414026	0.40542
fibroblast 329	1.0371282	0.40594
fetal kidney 83	1.0425538	0.40714
fetal stomach 240	1.0435945	0.40766
fetal stomach 249	1.0413969	0.40796
fetal small intestine 76	1.0407748	0.41172
fetal stomach 245	1.0354366	0.41466
breast 1	1.0293227	0.41956
keratinocyte 333	1.0259521	0.42184
keratinocyte 334	1.0260047	0.42196
fetal large intestine 65	1.0343730	0.42210
fetal stomach 243	1.0317051	0.42310
fetal stomach 241	1.0323875	0.42522
fetal small intestine 77	1.0324192	0.42656
ES derived MSC 290	1.0239380	0.43338
fetal large intestine 59	1.0266113	0.43470
fetal renal pelvis 218	1.0171442	0.44464
fetal renal cortex 207	1.0188358	0.44794
fetal muscle 159	1.0186089	0.44872
fetal renal pelvis 227	1.0174263	0.44918
fetal muscle 147	1.0174014	0.45142
fetal renal cortex 204	1.0145617	0.45362
fetal kidney 84	1.0151247	0.45430
fetal kidney 103	1.0120881	0.45814
fetal muscle 175	1.0110824	0.46302
fetal kidney 82	1.0095181	0.46390
fetal kidney 101	1.0090396	0.46500
fetal muscle 187	1.0087256	0.46764
fetal stomach 253	1.0046891	0.46846
fetal kidney 94	1.0043657	0.47030
fetal muscle 180	1.0082890	0.47154
fetal renal cortex 214	1.0059410	0.47156
fetal brain 29	1.0066320	0.47214
fetal renal cortex 205	1.0037378	0.47514
fetal adrenal gland 27	1.0074704	0.47558
fetal large intestine 53	1.0031134	0.47698
fetal renal cortex 213	1.0026517	0.47714
fetal muscle 155	1.0050162	0.47736
fetal kidney 87	1.0026518	0.47774
fetal muscle 191	1.0038155	0.47860
fetal renal cortex 202	1.0024049	0.47962
fetal kidney 81	1.0031337	0.47970
fetal stomach 250	0.9977801	0.48038
fetal kidney 88	1.0012126	0.48176
iPS 303	1.0011817	0.48242
ES cells 295	0.9975036	0.48278
fetal renal pelvis 217	0.9990500	0.48282
fetal kidney 105	0.9999536	0.48294
fetal thymus 263	1.0022326	0.48398
fetal renal pelvis 216	0.9976196	0.48480
fetal renal cortex 208	0.9979781	0.48556
fetal muscle 173	0.9997596	0.48600
fetal muscle 174	0.9997322	0.48614
keratinocyte 336	1.0013789	0.49008
fetal brain 33	0.9973508	0.49162
fetal muscle 171	0.9995208	0.49236
fetal renal cortex 206	0.9954658	0.49244
ES cells 296	0.9911511	0.49444
fibroblast 332	1.0000221	0.49514
fetal renal pelvis 228	0.9916812	0.49540
fetal renal cortex 203	0.9900826	0.49936
fetal kidney 92	0.9913598	0.49946
fetal muscle 151	0.9923299	0.50022
fetal renal pelvis 231	0.9927947	0.50076
fetal renal pelvis 220	0.9911634	0.50086
fetal renal pelvis 225	0.9892927	0.50130
fetal muscle 177	0.9908425	0.50172
fetal kidney 96	0.9911142	0.50248
fetal renal pelvis 232	0.9878209	0.50310
fetal renal cortex 212	0.9905281	0.50374
fetal fibroblast 268	0.9947562	0.50398
fetal muscle 143	0.9940318	0.50402
fetal muscle 149	0.9900935	0.50436
fetal muscle 188	0.9910056	0.50618
fetal fibroblast 282	0.9927466	0.50632
ES derived MSC 289	0.9868040	0.50688
fetal brain 41	0.9911892	0.50850
fetal renal pelvis 222	0.9844735	0.51064
fetal kidney 99	0.9822889	0.51608
fetal kidney 90	0.9805576	0.51628
fetal muscle 166	0.9859380	0.51926
fetal muscle 164	0.9839443	0.52008
fetal muscle 172	0.9876875	0.52018
fetal kidney 86	0.9756580	0.52028
fetal renal pelvis 230	0.9774933	0.52120
fetal kidney 100	0.9805200	0.52172
fetal muscle 157	0.9852856	0.52416
fetal renal pelvis 224	0.9798893	0.52584
fetal renal cortex 215	0.9783542	0.52626
fibroblast 331	0.9860900	0.52636
fetal skin 234	0.9804634	0.53062
fetal fibroblast 274	0.9828950	0.53180
fetal muscle 145	0.9735472	0.53434
fetal muscle 179	0.9766403	0.53530
fetal brain 39	0.9818185	0.53558
fetal kidney 89	0.9713981	0.53682
fetal muscle 189	0.9757759	0.53874
fetal brain 34	0.9824881	0.53886
fetal kidney 106	0.9728004	0.53932
fetal kidney 107	0.9694489	0.54226
fetal muscle 152	0.9754964	0.54286
fetal muscle 183	0.9759378	0.54328
fetal muscle 148	0.9721803	0.54338
fetal fibroblast 270	0.9757168	0.54914
fetal muscle 144	0.9686890	0.55006
fetal kidney 98	0.9640279	0.55030
fetal brain 36	0.9741687	0.55224
fetal kidney 104	0.9651125	0.55304
fetal muscle 161	0.9668195	0.55568
fetal muscle 162	0.9696784	0.55622
fetal renal pelvis 233	0.9615500	0.55772
fetal renal cortex 209	0.9608924	0.55788
fetal brain 38	0.9705754	0.56144
fetal lung 116	0.9722286	0.56148
fetal fibroblast 280	0.9712206	0.56246
fetal kidney 93	0.9556762	0.56306
fetal muscle 153	0.9616375	0.56468
fetal fibroblast 273	0.9703824	0.56534
keratinocyte 335	0.9723787	0.56810
fetal muscle 182	0.9625471	0.56924
fetal renal cortex 211	0.9520205	0.56948
fetal renal pelvis 223	0.9506761	0.56962
fetal lung 119	0.9637295	0.57022
fetal muscle 185	0.9591279	0.57148
fetal renal pelvis 219	0.9402668	0.57558
fetal fibroblast 281	0.9655108	0.57578
fetal brain 35	0.9722660	0.57600
fetal kidney 95	0.9517031	0.57630
fetal renal pelvis 226	0.9491845	0.57910
fetal lung 125	0.9595443	0.57942
fetal muscle 194	0.9621354	0.58028
fetal muscle 160	0.9551336	0.58234
fetal muscle 146	0.9563410	0.58372
fetal muscle 178	0.9527599	0.58706
fetal kidney 97	0.9474665	0.58738
fetal kidney 102	0.9421898	0.58808
fetal renal cortex 210	0.9414517	0.58894
fetal fibroblast 269	0.9586877	0.59046
fetal renal pelvis 221	0.9469736	0.59076
CD4 15	0.9022324	0.59106
fetal kidney 91	0.9468871	0.59136
fetal muscle 170	0.9513569	0.59670
fetal brain 30	0.9677497	0.59766
fetal lung 138	0.9518132	0.59782
fetal thymus 262	0.9054857	0.59976
fetal lung 142	0.9538483	0.60194
fetal brain 40	0.9598576	0.60224
fetal lung 135	0.9480881	0.60342
fetal lung 124	0.9484962	0.60414
fetal muscle 167	0.9458433	0.60632
fetal lung 118	0.9526433	0.60672
fetal lung 110	0.9451399	0.60874
fetal muscle 154	0.9369865	0.61126
placenta 196	0.9288840	0.61532
fetal lung 133	0.9470148	0.61626
fetal lung 115	0.9466050	0.61738
CD14 3	0.9171153	0.61774
placenta 198	0.9353515	0.61824
fetal lung 109	0.9504848	0.61972
fetal muscle 176	0.9379916	0.62172
fetal muscle 184	0.9412306	0.62218
fetal lung 123	0.9392588	0.62264
fetal renal pelvis 229	0.9306502	0.62386
fetal fibroblast 276	0.9466658	0.62410
fetal fibroblast 275	0.9447475	0.62584
fetal lung 131	0.9454795	0.62706
fetal muscle 190	0.9259283	0.63264
fetal thymus 257	0.9009365	0.63312
fetal fibroblast 271	0.9403377	0.63370
fetal fibroblast 279	0.9387796	0.63442
fetal muscle 181	0.9270595	0.63604
placenta 197	0.9155196	0.63904
fetal muscle 158	0.9337945	0.63918
fetal muscle 150	0.9240102	0.63984
fetal lung 122	0.9280342	0.64118
fetal kidney 85	0.9249813	0.64242
placenta 201	0.9102360	0.64580
fetal thymus 259	0.8928621	0.64896
placenta 199	0.9174375	0.64918
fetal fibroblast 272	0.9337632	0.64954
fetal lung 301	0.9254357	0.65104
fetal lung 140	0.9264531	0.65404
fetal lung 114	0.9302660	0.65688
fetal lung 129	0.9243036	0.65906
fetal lung 121	0.9284612	0.65992
hematopoietic stem cell 316	0.9058878	0.66092
CD14 4	0.8924549	0.66426
fetal lung 136	0.9180435	0.67284
fetal lung 112	0.9150791	0.67290
CD4 13	0.8582409	0.67324
hematopoietic stem cell 306	0.9011399	0.68018
fetal muscle 186	0.9106104	0.68036
fetal thymus 261	0.8726464	0.68104
fetal lung 126	0.9195880	0.68220
fetal brain 31	0.9357258	0.68430
placenta 200	0.9043270	0.68722
fetal lung 300	0.9076564	0.68900
fetal lung 130	0.9074332	0.69524
melanocyte 337	0.9245480	0.69824
Mobilized CD3 321	0.8330534	0.70106
fetal thymus 258	0.8661809	0.70612
fetal fibroblast 267	0.9051718	0.70702
fetal lung 128	0.9094157	0.71568
fetal large intestine 61	0.8451011	0.71598
fetal fibroblast 278	0.9011654	0.71784
fetal lung 108	0.8871929	0.72084
fetal lung 132	0.8945485	0.72210
fetal lung 137	0.8970944	0.72402
fetal lung 134	0.8894697	0.72504
fetal lung 139	0.8886037	0.72580
fetal thymus 265	0.8496192	0.72588
Mobilized CD8 325	0.8284192	0.72614
melanocyte 338	0.9096849	0.72720
fetal muscle 156	0.8850009	0.72982
Mobilized CD4 323	0.8186697	0.73096
fetal lung 299	0.8822050	0.73300
fetal small intestine 75	0.7797737	0.73592
fetal lung 117	0.8889248	0.73906
Mobilized CD3 320	0.8006040	0.74056
fetal lung 120	0.8837547	0.74206
fetal lung 113	0.8867778	0.74560
hematopoietic stem cell 307	0.8671275	0.74702
fetal thymus 260	0.8309907	0.74816
fetal lung 141	0.8832707	0.75092
fetal fibroblast 277	0.8822850	0.75146
fetal lung 127	0.8744039	0.75310
CD4 16	0.7946398	0.75542
melanocyte 340	0.8867227	0.75866
fetal muscle 169	0.8692284	0.76018
Mobilized CD4 322	0.7898809	0.76248
hematopoietic stem cell 309	0.8549969	0.76336
hematopoietic stem cell 311	0.8579196	0.76346
melanocyte 339	0.8920775	0.76448
hematopoietic stem cell 313	0.8633613	0.76474
fetal thymus 266	0.8183835	0.77242
fetal lung 111	0.8655369	0.77344
CD3 9	0.7907439	0.77398
CD8 22	0.7836682	0.77826
fetal thymus 264	0.8125974	0.78366
CD3 11	0.7942673	0.78420
fetal lung 298	0.8478435	0.79532
CD8 19	0.7481405	0.79720
hematopoietic stem cell 319	0.8239947	0.79856
CD8 21	0.7552947	0.80344
hematopoietic stem cell 315	0.8244094	0.80402
CD3 12	0.7517026	0.80948
hematopoietic stem cell 317	0.8477002	0.81022
CD19 6	0.7560873	0.82340
hematopoietic stem cell 308	0.8251655	0.82634
hematopoietic stem cell 305	0.8099784	0.83154
hematopoietic stem cell 310	0.8298383	0.83344
fetal thymus 256	0.7788009	0.83452
CD4 14	0.7469467	0.84266
hematopoietic stem cell 312	0.8109435	0.84318
CD56 17	0.7631461	0.84324
hematopoietic stem cell 318	0.7905485	0.84356
hematopoietic stem cell 314	0.8112600	0.84688
CD19 7	0.7269212	0.84886
CD8 20	0.7218845	0.86334
CD56 18	0.6907207	0.87396
Mobilized CD56 324	0.6978064	0.88566
CD3 10	0.7021479	0.89312
CD19 5	0.6927075	0.89506

Crohns disease 3

DHS sample	fold enrichment	p value
CD56 17	1.3848953	0.08258
CD14 4	1.2985659	0.12784
CD8 20	1.2936580	0.15840
CD14 3	1.2793937	0.15940
CD3 11	1.1975261	0.23556
hematopoietic stem cell 313	1.1203952	0.27704
CD4 14	1.1429271	0.28736
hematopoietic stem cell 310	1.0959837	0.30848
hematopoietic stem cell 317	1.0890375	0.31384
CD8 19	1.1181418	0.33228
CD3 10	1.1035622	0.33348
CD8 21	1.1142635	0.33758
CD8 22	1.1013340	0.33840
hematopoietic stem cell 309	1.0670104	0.36664
CD4 13	1.0736866	0.37874
Mobilized CD56 324	1.0693921	0.38108
CD3 12	1.0684914	0.38402
keratinocyte 334	1.0429497	0.38686
CD19 5	1.0552795	0.39322
Mobilized CD4 323	1.0469266	0.40642
hematopoietic stem cell 312	1.0295395	0.42978
CD3 9	1.0237237	0.43254
hematopoietic stem cell 314	1.0240718	0.43518
keratinocyte 335	1.0218362	0.44142
Mobilized CD4 322	1.0141590	0.44570
Mobilized CD3 320	1.0173801	0.45232
CD56 18	1.0108513	0.45680
CD19 6	1.0012673	0.46058
hematopoietic stem cell 308	1.0084361	0.46178
Mobilized CD3 321	0.9932540	0.46204
melanocyte 340	1.0098415	0.46386
keratinocyte 336	1.0090128	0.47354
hematopoietic stem cell 311	0.9938734	0.48484
CD4 16	0.9698748	0.48806
hematopoietic stem cell 307	0.9805594	0.50302
keratinocyte 333	0.9910555	0.51718
hematopoietic stem cell 318	0.9716917	0.51900
hematopoietic stem cell 316	0.9668237	0.52326
Mobilized CD8 325	0.9455519	0.52454
melanocyte 339	0.9848611	0.52466
breast 2	0.9667762	0.56126
fetal thymus 256	0.9407467	0.56270
hematopoietic stem cell 315	0.9479446	0.56816
breast 1	0.9649871	0.56924
hematopoietic stem cell 306	0.9377708	0.58346
hematopoietic stem cell 319	0.9169370	0.60702
melanocyte 338	0.9504048	0.60908
melanocyte 337	0.9509065	0.61128
hematopoietic stem cell 305	0.8976406	0.64808
fetal thymus 265	0.8780288	0.65102
CD19 7	0.8508879	0.65188
fetal large intestine 61	0.8527052	0.66888
fetal thymus 258	0.8530920	0.68006
fetal thymus 260	0.8574537	0.68184
fetal thymus 264	0.8565156	0.68664
CD4 15	0.7824836	0.68910
fetal thymus 261	0.8390060	0.69566
fetal thymus 266	0.8411306	0.70288
fetal muscle 148	0.8638922	0.71750
ES derived mesoderm 285	0.8895072	0.71864
fetal muscle 149	0.8538149	0.72162
fetal muscle 179	0.8661766	0.72238
fetal lung 133	0.8787447	0.72464
muscle 341	0.8252411	0.72574
ES cells 294	0.8260112	0.72700
ES cells 296	0.8211236	0.72990
fetal stomach 244	0.8245618	0.74014
fetal lung 134	0.8593871	0.74188
iPS 304	0.8477493	0.74870
fetal small intestine 75	0.7245347	0.74886
fetal muscle 153	0.8379391	0.75090
fetal muscle 167	0.8490648	0.75798
fetal muscle 151	0.8268887	0.75848
fetal lung 122	0.8413466	0.76330
fetal lung 121	0.8596442	0.76838
fetal muscle 180	0.8261763	0.77086
pancreas 328	0.8029127	0.77408
fetal kidney 81	0.8399131	0.77424
fetal heart 50	0.8199761	0.77884
fetal lung 132	0.8424379	0.77972
fetal lung 111	0.8465218	0.78042
fetal muscle 164	0.8248199	0.78070
fetal heart 42	0.8191030	0.78340
fetal muscle 145	0.8047703	0.78432
fetal muscle 159	0.8096073	0.78918
fetal lung 126	0.8488314	0.79056
fetal testes 255	0.8239225	0.79080
fetal thymus 262	0.7193527	0.79184
fetal muscle 191	0.8160973	0.79312
fetal muscle 190	0.8028362	0.79576
fetal lung 129	0.8302922	0.80016
fetal lung 140	0.8357338	0.80022
fetal muscle 168	0.8082915	0.80124
ES cells 295	0.7730963	0.80196
iPS 303	0.8072980	0.80296
fetal muscle 193	0.8009357	0.80654
fetal lung 128	0.8466784	0.80804
fetal muscle 169	0.8055226	0.80876
fetal stomach 251	0.7774621	0.80902
fetal heart 46	0.7911217	0.80904
fetal muscle 150	0.7942509	0.81218
fetal lung 125	0.8155255	0.81388
pancreas 327	0.7571331	0.81456
fetal spinal cord 237	0.8171337	0.81572
fetal muscle 174	0.7970141	0.81640
fetal heart 51	0.7983505	0.81710
fetal stomach 252	0.7627784	0.81716
stomach 283	0.7526946	0.81928
fetal stomach 247	0.7168743	0.81956
fetal muscle 152	0.8113695	0.81974
fetal lung 114	0.8332149	0.82174
fetal muscle 187	0.8031741	0.82984
heart 297	0.7605459	0.83054
stomach 284	0.7572823	0.83148
fetal lung 139	0.7996909	0.83192
fetal lung 141	0.8193927	0.83278
fetal muscle 177	0.7773466	0.83306
fetal muscle 157	0.8050040	0.83308
fetal muscle 165	0.8024664	0.83314
fetal lung 120	0.8053281	0.83580
fetal ovary 195	0.7251296	0.83584
fetal thymus 259	0.7130961	0.83590
fetal stomach 250	0.7320121	0.83632
fetal brain 33	0.8127876	0.83702
fetal muscle 176	0.7989954	0.83742
fetal lung 299	0.8040710	0.83770
fetal stomach 242	0.6923600	0.83794
fetal lung 124	0.8042499	0.83848
fetal stomach 246	0.7304525	0.83866
fetal heart 45	0.7774690	0.83988
fetal muscle 181	0.7846419	0.84004
fetal fibroblast 267	0.8192630	0.84036
fetal lung 117	0.8074393	0.84148
fetal lung 108	0.7851373	0.84276
fetal brain 37	0.8210784	0.84320
fetal stomach 241	0.7253025	0.84492
fetal lung 138	0.7945686	0.84600
fetal muscle 143	0.7965726	0.84628
fetal stomach 248	0.7309064	0.84760
fetal lung 115	0.8066567	0.84800
fetal muscle 173	0.7718196	0.84844
fetal muscle 182	0.7854258	0.84886
fetal stomach 240	0.7100824	0.85080
fetal thymus 257	0.7024415	0.85134
fetal muscle 147	0.7725226	0.85188
fetal stomach 249	0.7125036	0.85192
fetal heart 47	0.7473375	0.85314
fetal muscle 178	0.7712987	0.85384
fetal stomach 245	0.7286662	0.85392
ovary 326	0.6995288	0.85418
fetal muscle 154	0.7598087	0.85472
fetal lung 109	0.8160136	0.85614
fetal heart 52	0.7759157	0.85624
fetal thymus 263	0.7653235	0.85752
fetal muscle 166	0.7726364	0.85848
fetal muscle 144	0.7583093	0.85868
fetal spinal cord 235	0.7787214	0.85924
fetal muscle 155	0.7543755	0.85950
fetal lung 136	0.7831645	0.86098
fetal lung 135	0.7831682	0.86224
fetal muscle 186	0.7686077	0.86254
fetal muscle 188	0.7726771	0.86394
fetal lung 130	0.7828848	0.86408
fetal heart 48	0.7560405	0.86590
fetal muscle 184	0.7775037	0.86630
fetal heart 43	0.7681557	0.86634
fetal lung 110	0.7776419	0.86672
iPS 302	0.7527116	0.86690
fetal muscle 160	0.7548818	0.86700
ES derived mesoderm 286	0.8091233	0.86716
ES derived trophoblast 287	0.7702749	0.86734
fetal brain 29	0.8425691	0.86950
ES cells 293	0.7279991	0.86960
fetal muscle 158	0.7698592	0.86968
fetal muscle 175	0.7573869	0.87360
small intestine 342	0.7275727	0.87398
fetal stomach 253	0.6995972	0.87414
fetal lung 131	0.7988126	0.87472
fetal muscle 194	0.7821909	0.87496
fetal lung 137	0.7831869	0.87562
fetal brain 39	0.8123564	0.87568
fetal muscle 146	0.7592968	0.87664
fetal muscle 189	0.7519741	0.87740
fetal adrenal gland 27	0.7425369	0.87758
fetal heart 44	0.7054288	0.87864
fibroblast 331	0.7803928	0.87944
fetal renal pelvis 219	0.6758956	0.88018
fetal lung 113	0.7861881	0.88048
fetal renal cortex 207	0.7459579	0.88098
fetal muscle 163	0.7373843	0.88202
fetal lung 119	0.7845977	0.88404
fetal muscle 170	0.7573627	0.88430
fetal muscle 162	0.7591622	0.88612
fetal testes 254	0.7295670	0.88630
fetal skin 234	0.7386518	0.88722
fetal spinal cord 236	0.7408055	0.88818
fetal muscle 185	0.7296819	0.88864
fibroblast 332	0.7743938	0.88972
fetal muscle 161	0.7416946	0.89066
fetal lung 298	0.7591499	0.89134
fetal muscle 192	0.7466280	0.89136
fetal fibroblast 268	0.7871798	0.89202
fetal lung 300	0.7624518	0.89224
fetal lung 142	0.7772626	0.89248
fetal muscle 183	0.7481224	0.89308
fetal fibroblast 282	0.7899220	0.89450
fetal adrenal gland 24	0.6943626	0.89456
fetal renal pelvis 220	0.7170553	0.89586
ES derived NP 292	0.6973450	0.89598
fetal kidney 94	0.7089990	0.89614
fetal brain 38	0.7799780	0.89636
fetal muscle 171	0.7393366	0.89672
fetal large intestine 53	0.6706613	0.89966
fetal large intestine 66	0.6913018	0.89996
fetal fibroblast 272	0.7770672	0.90028
fetal heart 49	0.7230642	0.90196
fetal stomach 243	0.7058315	0.90296
fetal adrenal gland 26	0.7196393	0.90310
fetal lung 123	0.7456433	0.90316
fetal lung 118	0.7682277	0.90526
fetal lung 116	0.7641389	0.90802
fetal muscle 156	0.7247406	0.90814
fetal kidney 92	0.7233783	0.90820
fetal renal cortex 214	0.7126432	0.90854
fetal lung 127	0.7381571	0.90900
placenta 201	0.6812892	0.90952
fetal kidney 100	0.7124715	0.91050
fetal renal cortex 213	0.7259731	0.91092
fetal small intestine 71	0.6860570	0.91176
fetal spinal cord 238	0.7350256	0.91220
fetal large intestine 59	0.6735200	0.91240
ES derived NP 291	0.6644521	0.91316
fetal renal pelvis 223	0.6862586	0.91360
fetal renal cortex 208	0.7028934	0.91404
fetal renal cortex 211	0.6884071	0.91436
fetal brain 41	0.7725756	0.91450
fetal large intestine 58	0.7056443	0.91456
fetal fibroblast 274	0.7645604	0.91594
fetal brain 36	0.7648714	0.91696
fetal renal cortex 210	0.6875395	0.91836
fetal small intestine 79	0.6644849	0.91874
fetal fibroblast 277	0.7521844	0.91920
fetal kidney 91	0.7199818	0.91950
fetal kidney 82	0.6669196	0.91998
placenta 200	0.7155922	0.92032
fetal kidney 87	0.6998912	0.92078
fetal brain 32	0.7660684	0.92138
fetal kidney 102	0.6756687	0.92212
fetal lung 301	0.7275601	0.92234
fetal adrenal gland 23	0.7107955	0.92272
fetal kidney 88	0.7096964	0.92338
fetal small intestine 70	0.7114675	0.92426
placenta 196	0.6739121	0.92428
fetal large intestine 62	0.6565039	0.92438
fibroblast 330	0.7228170	0.92474
fetal large intestine 57	0.6977714	0.92620
fetal fibroblast 279	0.7444989	0.92656
fetal small intestine 73	0.6603644	0.92720
fetal kidney 96	0.7006079	0.92878
fetal renal pelvis 218	0.6222396	0.92934
fetal renal pelvis 226	0.6933974	0.93026
fetal small intestine 68	0.6774939	0.93062
fetal fibroblast 276	0.7484536	0.93080
fetal large intestine 65	0.6618644	0.93144
fetal large intestine 64	0.6752080	0.93198
fetal kidney 86	0.6280561	0.93248
fetal kidney 84	0.6693897	0.93304
fetal brain 34	0.7687839	0.93316
fetal renal cortex 203	0.6835157	0.93320
fetal kidney 93	0.6643084	0.93352
fetal brain 30	0.7991838	0.93572
fetal small intestine 76	0.6401834	0.93622
fetal renal cortex 202	0.6881378	0.93670
fetal adrenal gland 28	0.7131300	0.93672
fetal renal pelvis 217	0.6615473	0.93686
fetal kidney 104	0.6664365	0.93702
fetal renal cortex 212	0.6911461	0.93714
fetal brain 35	0.7752778	0.93718
fetal renal pelvis 232	0.6602868	0.93720
fetal renal pelvis 228	0.6511677	0.93742
fetal small intestine 72	0.6541262	0.93746
fetal renal cortex 206	0.6849157	0.93754
fetal kidney 89	0.6690343	0.93786
fetal fibroblast 281	0.7374172	0.93810
fetal renal pelvis 230	0.6527618	0.93836
fibroblast 329	0.7108941	0.93886
fetal large intestine 67	0.6550738	0.93890
fetal large intestine 55	0.6693551	0.93920
fetal spinal cord 239	0.7020379	0.93934
fetal small intestine 77	0.6423824	0.93942
fetal muscle 172	0.7207972	0.93960
fetal fibroblast 271	0.7338298	0.93972
fetal kidney 107	0.6705081	0.94060
fetal kidney 83	0.6290032	0.94074
fetal kidney 101	0.6787389	0.94092
fetal renal pelvis 225	0.6614627	0.94156
fetal small intestine 74	0.6521068	0.94156
fetal fibroblast 273	0.7357393	0.94214
fetal fibroblast 278	0.7261438	0.94266
fetal kidney 98	0.6646272	0.94274
CD20 8	0.6564887	0.94428
fetal renal cortex 204	0.6844495	0.94512
fetal small intestine 69	0.6193821	0.94562
fetal large intestine 63	0.6251421	0.94588
fetal renal pelvis 216	0.6584271	0.94674
placenta 199	0.6531795	0.94764
fetal small intestine 78	0.6689095	0.94792
fetal large intestine 54	0.6310812	0.94848
fetal kidney 106	0.6625572	0.94958
fetal adrenal gland 25	0.6292755	0.95044
fetal renal pelvis 229	0.6662965	0.95046
placenta 197	0.6294478	0.95164
fetal renal cortex 209	0.6492055	0.95270
fetal lung 112	0.6899068	0.95284
fetal kidney 97	0.6528246	0.95288
fetal kidney 99	0.6490604	0.95322
fetal kidney 90	0.6480248	0.95344
fetal renal pelvis 233	0.6487661	0.95442
fetal fibroblast 275	0.7185775	0.95460
fetal kidney 103	0.6432141	0.95512
fetal renal pelvis 222	0.6380266	0.95638
fetal kidney 105	0.6532892	0.95724
fetal kidney 85	0.6816776	0.95930
fetal large intestine 56	0.6216908	0.95942
fetal kidney 95	0.6326673	0.96104
fetal renal pelvis 227	0.6343949	0.96142
fetal renal cortex 205	0.6344166	0.96206
ES derived trophoblast 288	0.6603244	0.96728
placenta 198	0.6334249	0.96834
fetal large intestine 60	0.5956735	0.96900
fetal fibroblast 280	0.6838544	0.96938
fetal renal pelvis 224	0.6356654	0.97074
fetal fibroblast 270	0.6684770	0.97266
fetal renal cortex 215	0.6297996	0.97344
fetal renal pelvis 231	0.6181867	0.97496
fetal renal pelvis 221	0.6123581	0.97714
fetal small intestine 80	0.5832641	0.97736
ES derived MSC 290	0.6070675	0.97764
fetal brain 31	0.7273333	0.97804
fetal brain 40	0.6842817	0.98220
fetal fibroblast 269	0.6438488	0.98398
ES derived MSC 289	0.5553340	0.99040

Body mass index 2

DHS sample	fold enrichment	p value
fetal heart 46	1.3445634	0.02042
fetal heart 42	1.3230842	0.02146
fetal heart 49	1.3199108	0.02530
fetal heart 52	1.2816784	0.02666
fetal heart 48	1.2993261	0.02774
fetal heart 45	1.3145035	0.02838
fetal heart 43	1.2791531	0.02932
fetal heart 47	1.3367965	0.03162
fetal heart 44	1.3540642	0.03250
fetal heart 51	1.2801292	0.03416
fetal heart 50	1.2515117	0.05284
pancreas 328	1.2204065	0.10806
pancreas 327	1.1992916	0.13860
fetal small intestine 74	1.1535168	0.17688
fetal small intestine 70	1.1240093	0.19938
heart 297	1.1367081	0.20944
muscle 341	1.1449983	0.20958
fetal large intestine 58	1.1230054	0.21288
keratinocyte 334	1.0900762	0.22522
fetal large intestine 67	1.1209465	0.23036
fetal small intestine 68	1.1120134	0.23408
fetal small intestine 80	1.1140451	0.23902
breast 2	1.0841849	0.25314
CD20 8	1.0990646	0.25968
fetal small intestine 78	1.0950609	0.26004
stomach 284	1.1037228	0.26898
fetal large intestine 60	1.0973034	0.27550
fetal large intestine 55	1.0889822	0.28008
fetal large intestine 64	1.0907801	0.28220
ES derived mesoderm 285	1.0692005	0.28542
keratinocyte 333	1.0651802	0.28612
fetal large intestine 63	1.0918149	0.29032
ES derived NP 292	1.0875934	0.29060
fetal small intestine 72	1.0862925	0.29210
ES derived mesoderm 286	1.0573561	0.30116
ES cells 295	1.0825651	0.30306
fetal stomach 246	1.0861868	0.30460
breast 1	1.0637200	0.30634
iPS 304	1.0653661	0.30802
ES cells 296	1.0722956	0.32250
fetal stomach 244	1.0720712	0.32292
fetal large intestine 62	1.0756959	0.32412
fetal large intestine 54	1.0691552	0.32842
fetal small intestine 69	1.0720497	0.33020
fetal stomach 242	1.0782917	0.33286
fetal large intestine 57	1.0615691	0.33304
fetal small intestine 73	1.0687265	0.33330
fetal large intestine 65	1.0614592	0.34364
fetal small intestine 79	1.0631766	0.34708
fetal stomach 248	1.0616228	0.35002
ES derived NP 291	1.0563688	0.35800
fetal brain 32	1.0418202	0.35842
fetal stomach 251	1.0514846	0.36372
fetal large intestine 56	1.0525991	0.36376
fetal large intestine 66	1.0533302	0.36406
keratinocyte 335	1.0387759	0.36934
fetal stomach 247	1.0530957	0.37542
fetal muscle 168	1.0406834	0.38580
fetal small intestine 77	1.0408426	0.39184
fetal stomach 245	1.0392192	0.39400
fetal small intestine 76	1.0402704	0.39462
iPS 303	1.0318638	0.39966
stomach 283	1.0377406	0.40120
fetal stomach 243	1.0334076	0.40178
iPS 302	1.0293415	0.40708
ES cells 294	1.0299638	0.41100
small intestine 342	1.0318862	0.41260
ES cells 293	1.0263659	0.41890
fetal stomach 241	1.0272322	0.41960
fetal stomach 249	1.0260910	0.42058
keratinocyte 336	1.0223760	0.42218
fetal small intestine 71	1.0248461	0.42434
fetal muscle 193	1.0223449	0.43044
fetal stomach 240	1.0186353	0.43870
fetal stomach 252	1.0174469	0.44124
fibroblast 329	1.0135997	0.44966
fetal stomach 250	1.0093413	0.46046
fetal adrenal gland 28	1.0104187	0.46242
fetal stomach 253	1.0055318	0.46814
fetal ovary 195	1.0035724	0.46888
fetal adrenal gland 23	1.0057553	0.47178
fibroblast 330	1.0053993	0.47456
fetal muscle 192	1.0031330	0.47740
fetal muscle 159	1.0025336	0.47898
ES derived MSC 290	1.0027127	0.48238
fetal testes 254	0.9999033	0.48496
fetal large intestine 53	0.9949409	0.48924
fetal brain 38	0.9989588	0.49106
fetal adrenal gland 24	0.9957005	0.49254
fetal large intestine 59	0.9935798	0.49822
fetal fibroblast 282	0.9952737	0.50422
fetal adrenal gland 25	0.9923228	0.50458
CD14 4	0.9884913	0.51236
ES derived trophoblast 288	0.9832845	0.53132
fetal muscle 188	0.9825024	0.53432
fetal brain 36	0.9870066	0.53518
fetal fibroblast 275	0.9861162	0.53678
fetal muscle 163	0.9807950	0.53766
fetal muscle 173	0.9801297	0.53810
fetal muscle 177	0.9765408	0.54510
fetal muscle 148	0.9772779	0.54780
fetal muscle 180	0.9772009	0.54818
CD14 3	0.9683032	0.54984
fetal brain 33	0.9783539	0.55080
fibroblast 332	0.9801933	0.55442
ovary 326	0.9649163	0.55490
fetal kidney 81	0.9756347	0.55984
fetal fibroblast 276	0.9783985	0.56222
fetal muscle 165	0.9738319	0.56406
fetal fibroblast 268	0.9776980	0.56430
fetal muscle 191	0.9706992	0.56462
fetal muscle 175	0.9717036	0.56498
fetal spinal cord 237	0.9721749	0.56600
melanocyte 338	0.9782567	0.56774
fetal fibroblast 274	0.9737097	0.57110
ES derived trophoblast 287	0.9657858	0.57134
fetal muscle 172	0.9730282	0.57198
fetal muscle 155	0.9651474	0.57560
fetal fibroblast 281	0.9716226	0.57980
fetal muscle 160	0.9647422	0.58212
fetal muscle 187	0.9660942	0.58460
fetal adrenal gland 27	0.9630002	0.58592
fetal muscle 147	0.9613466	0.58958
fetal spinal cord 239	0.9629428	0.59426
fetal fibroblast 280	0.9662053	0.59436
fetal adrenal gland 26	0.9575570	0.59626
fetal muscle 164	0.9581951	0.59818
fetal muscle 171	0.9592813	0.59954
fetal kidney 83	0.9464314	0.60066
fetal muscle 151	0.9528659	0.60150
fetal muscle 176	0.9578208	0.60226
fetal spinal cord 236	0.9564912	0.60450
fetal fibroblast 269	0.9605149	0.61210
fetal fibroblast 270	0.9579236	0.61576
fetal thymus 263	0.9486516	0.61808
fetal spinal cord 238	0.9539215	0.61886
melanocyte 340	0.9593732	0.62126
fetal muscle 174	0.9489526	0.62144
fetal fibroblast 273	0.9575639	0.62312
fetal lung 119	0.9578258	0.62394
fetal lung 125	0.9514259	0.62644
fetal muscle 149	0.9455420	0.62694
ES derived MSC 289	0.9479069	0.62800
fetal lung 299	0.9493322	0.62878
fetal muscle 152	0.9476786	0.63090
fetal muscle 182	0.9457939	0.63594
fetal muscle 167	0.9448200	0.63818
fetal muscle 153	0.9397080	0.63836
fetal renal cortex 202	0.9423828	0.63952
fetal muscle 166	0.9416060	0.64148
fetal muscle 145	0.9344095	0.64374
fibroblast 331	0.9479661	0.64480
fetal lung 300	0.9448795	0.64520
fetal muscle 179	0.9401839	0.64656
fetal testes 255	0.9412670	0.64768
fetal renal pelvis 230	0.9289900	0.64946
fetal lung 298	0.9403593	0.65038
fetal kidney 103	0.9339816	0.65186
fetal spinal cord 235	0.9424233	0.65412
hematopoietic stem cell 319	0.9254469	0.65590
fetal muscle 183	0.9392486	0.65722
fetal muscle 143	0.9410992	0.65756
fetal muscle 157	0.9413482	0.65844
fetal renal pelvis 218	0.9116473	0.66028
fetal lung 301	0.9368766	0.66492
fetal lung 115	0.9413528	0.66750
fetal lung 140	0.9406373	0.66808
placenta 201	0.9211043	0.66848
fetal muscle 146	0.9325841	0.67080
fetal muscle 162	0.9323617	0.67304
fetal fibroblast 279	0.9392053	0.67432
fetal brain 35	0.9508633	0.67496
fetal lung 112	0.9342986	0.67938
CD4 15	0.8600748	0.68096
fetal fibroblast 271	0.9366673	0.68288
fetal fibroblast 272	0.9379757	0.68336
hematopoietic stem cell 311	0.9210317	0.68476
fetal brain 34	0.9465714	0.68478
fetal renal cortex 205	0.9222687	0.68736
placenta 197	0.9171796	0.68742
fetal brain 37	0.9404920	0.68774
hematopoietic stem cell 316	0.9129013	0.68864
melanocyte 339	0.9402038	0.68936
fetal fibroblast 277	0.9328755	0.68998
fetal lung 135	0.9300198	0.69056
fetal kidney 82	0.9077507	0.69516
hematopoietic stem cell 306	0.9155418	0.69582
CD19 6	0.8784736	0.69596
fetal skin 234	0.9210589	0.69622
fetal muscle 181	0.9191966	0.69626
fetal kidney 94	0.9121018	0.69722
fetal muscle 189	0.9169871	0.70718
fetal lung 138	0.9231750	0.70808
fetal lung 116	0.9307718	0.71040
fetal kidney 96	0.9148777	0.71048
fetal muscle 144	0.9092554	0.71070
fetal muscle 185	0.9094775	0.71362
fetal lung 130	0.9233941	0.71418
fetal renal pelvis 219	0.8858423	0.71438
fetal kidney 86	0.8903470	0.71460
fetal brain 39	0.9347252	0.71496
melanocyte 337	0.9330144	0.71810
fetal renal cortex 207	0.9082142	0.71896
fetal muscle 170	0.9154169	0.71906
fetal kidney 101	0.9077846	0.72438
fetal kidney 84	0.9017465	0.72492
fetal fibroblast 278	0.9193038	0.72702
fetal lung 139	0.9126565	0.72718
fetal lung 133	0.9197748	0.72772
fetal kidney 99	0.9036599	0.72790
fetal brain 41	0.9259873	0.73004
fetal muscle 178	0.9031947	0.73058
fetal muscle 190	0.8998306	0.73078
fetal lung 114	0.9222005	0.73092
fetal renal cortex 208	0.8979050	0.73092
fetal thymus 262	0.8400272	0.73160
hematopoietic stem cell 314	0.9078660	0.73238
fetal renal pelvis 217	0.8972785	0.73272
fetal kidney 88	0.9056526	0.73274
hematopoietic stem cell 313	0.9032889	0.73368
fetal muscle 161	0.9034651	0.73392
fetal fibroblast 267	0.9164208	0.73434
fetal renal pelvis 227	0.9004197	0.73662
fetal renal pelvis 220	0.8964615	0.73686
fetal lung 124	0.9134001	0.73706
fetal lung 110	0.9108814	0.73716
fetal kidney 105	0.9048531	0.73768
fetal lung 142	0.9190061	0.73790
fetal lung 123	0.9099653	0.74336
fetal lung 122	0.9042538	0.74490
placenta 196	0.8907110	0.74540
fetal lung 117	0.9130823	0.74626
fetal lung 134	0.9068634	0.74648
fetal renal pelvis 222	0.8908950	0.74650
fetal renal cortex 204	0.9006652	0.74742
fetal lung 109	0.9204035	0.74874
CD19 7	0.8475501	0.74894
fetal renal pelvis 224	0.9001450	0.74898
fetal thymus 257	0.8537626	0.74916
fetal renal cortex 209	0.8916994	0.74920
fetal lung 129	0.9081645	0.75316
fetal muscle 194	0.9077706	0.75496
fetal renal cortex 203	0.8898624	0.75526
hematopoietic stem cell 312	0.8924228	0.75680
fetal muscle 184	0.9018190	0.75830
fetal muscle 158	0.8977505	0.75876
hematopoietic stem cell 317	0.8998713	0.76022
fetal renal pelvis 225	0.8849013	0.76102
fetal kidney 98	0.8875593	0.76106
fetal renal pelvis 216	0.8846552	0.76166
fetal kidney 102	0.8810425	0.76178
fetal thymus 259	0.8504040	0.76262
fetal renal cortex 210	0.8821388	0.76312
fetal muscle 169	0.8897762	0.76330
fetal renal cortex 214	0.8880537	0.76406
hematopoietic stem cell 310	0.8967293	0.76620
fetal renal pelvis 231	0.8922211	0.76864
fetal muscle 150	0.8830497	0.76888
fetal renal cortex 212	0.8915975	0.76984
fetal kidney 97	0.8841937	0.77084
placenta 199	0.8855038	0.77088
hematopoietic stem cell 307	0.8812657	0.77150
fetal kidney 90	0.8817590	0.77244
fetal lung 128	0.9099348	0.77276
CD19 5	0.8377448	0.77402
fetal renal pelvis 223	0.8726654	0.77700
fetal lung 132	0.8972141	0.77726
hematopoietic stem cell 309	0.8793092	0.77840
fetal renal pelvis 232	0.8741330	0.77868
fetal renal cortex 206	0.8845753	0.77946
fetal muscle 154	0.8755867	0.77972
fetal renal pelvis 228	0.8691185	0.78246
fetal lung 108	0.8867535	0.78396
fetal lung 118	0.9028803	0.78480
hematopoietic stem cell 315	0.8711991	0.78494
fetal brain 40	0.9104929	0.78518
fetal large intestine 61	0.8363886	0.78548
fetal kidney 89	0.8745318	0.78644
fetal lung 121	0.8995049	0.78702
fetal kidney 106	0.8791828	0.78808
hematopoietic stem cell 305	0.8708501	0.78844
fetal lung 126	0.9000050	0.78854
fetal renal pelvis 233	0.8751804	0.79020
hematopoietic stem cell 308	0.8777542	0.79040
fetal kidney 93	0.8642053	0.79190
fetal renal pelvis 226	0.8747501	0.79350
fetal kidney 104	0.8701610	0.79448
placenta 198	0.8765967	0.79606
fetal brain 31	0.9181370	0.79616
fetal kidney 107	0.8720831	0.79778
fetal kidney 100	0.8709392	0.79884
fetal renal cortex 213	0.8769617	0.79898
fetal lung 131	0.8991495	0.80042
fetal renal cortex 215	0.8766868	0.80084
fetal renal cortex 211	0.8603047	0.80088
fetal muscle 186	0.8778363	0.80164
fetal lung 127	0.8832742	0.80208
hematopoietic stem cell 318	0.8573640	0.80364
fetal kidney 95	0.8682538	0.80584
Mobilized CD4 323	0.8085112	0.80930
fetal small intestine 75	0.7676429	0.81296
placenta 200	0.8741719	0.81332
Mobilized CD3 321	0.7913185	0.81350
fetal brain 29	0.9129722	0.81410
fetal muscle 156	0.8685909	0.81728
fetal lung 113	0.8849153	0.81826
fetal thymus 258	0.8278104	0.81830
fetal renal pelvis 221	0.8660379	0.81852
fetal kidney 85	0.8745636	0.81994
CD4 13	0.7987923	0.82110
Mobilized CD8 325	0.7976159	0.82344
fetal thymus 261	0.8159011	0.82508
fetal lung 111	0.8750419	0.82654
fetal lung 136	0.8720354	0.82756
fetal lung 141	0.8793488	0.82850
CD3 10	0.8105340	0.82994
fetal kidney 87	0.8554022	0.83310
fetal kidney 91	0.8600499	0.83442
fetal lung 120	0.8704596	0.83536
fetal kidney 92	0.8524526	0.84514
fetal brain 30	0.9017767	0.84940
fetal lung 137	0.8654485	0.85350
CD8 21	0.7672127	0.85776
CD4 16	0.7598628	0.86170
fetal thymus 260	0.7902366	0.86382
CD8 19	0.7473936	0.86418
fetal renal pelvis 229	0.8368591	0.86440
Mobilized CD4 322	0.7496374	0.87282
fetal thymus 266	0.7889246	0.87528
Mobilized CD3 320	0.7488026	0.88064
CD56 17	0.7730302	0.89310
CD4 14	0.7600396	0.89540
fetal thymus 265	0.7693899	0.89832
CD56 18	0.7366107	0.89852
CD3 9	0.7402878	0.89884
fetal thymus 264	0.7658586	0.90180
CD3 12	0.7232028	0.90328
CD3 11	0.7474039	0.91120
CD8 22	0.7132653	0.91694
Mobilized CD56 324	0.7358408	0.92418
fetal thymus 256	0.7472759	0.92692
CD8 20	0.7008052	0.94098

Metabolic traits 1

DHS sample	fold enrichment	p value
CD14 4	1.2604075	0.11774
melanocyte 338	1.1519333	0.15892
small intestine 342	1.1755749	0.19662
keratinocyte 334	1.1118798	0.23556
ES derived trophoblast 288	1.1217828	0.24748
ES derived trophoblast 287	1.1384029	0.24854
melanocyte 337	1.1000874	0.25008
keratinocyte 333	1.1014627	0.25042
placenta 200	1.1082430	0.26876
CD14 3	1.1159644	0.29780
fetal large intestine 58	1.0969681	0.29868
CD20 8	1.0917309	0.31314
pancreas 328	1.0868042	0.33486
fetal small intestine 70	1.0740063	0.33712
fetal large intestine 64	1.0826346	0.33792
stomach 284	1.0867897	0.33898
fetal thymus 256	1.0781573	0.34866
fetal small intestine 74	1.0750972	0.34932
fetal small intestine 78	1.0634318	0.36088
keratinocyte 336	1.0494908	0.36888
fetal thymus 258	1.0656311	0.37286
keratinocyte 335	1.0438911	0.38076
fetal small intestine 68	1.0454976	0.39920
fetal large intestine 57	1.0419455	0.40278
hematopoietic stem cell 310	1.0402711	0.40388
melanocyte 340	1.0373998	0.40632
fetal heart 45	1.0370650	0.41214
ES derived MSC 290	1.0345790	0.41494
placenta 198	1.0281304	0.43208
CD56 17	1.0277616	0.43742
fetal large intestine 67	1.0277767	0.43770
heart 297	1.0258542	0.44008
ES derived MSC 289	1.0196488	0.44494
fetal thymus 260	1.0200515	0.44884
fetal large intestine 56	1.0189373	0.45174
breast 1	1.0170303	0.45214
fetal large intestine 60	1.0159979	0.45430
fetal adrenal gland 28	1.0131917	0.46622
fetal thymus 265	1.0065960	0.47184
pancreas 327	0.9997689	0.48284
melanocyte 339	1.0038094	0.48584
CD56 18	1.0010804	0.48626
breast 2	1.0012097	0.48634
fetal large intestine 55	0.9986708	0.49074
fetal small intestine 79	0.9954269	0.49078
hematopoietic stem cell 312	0.9980568	0.49726
placenta 201	0.9911821	0.50070
CD8 21	0.9862301	0.50848
fetal heart 49	0.9876855	0.51284
fetal lung 300	0.9918217	0.51338
fibroblast 329	0.9912555	0.51694
fetal small intestine 73	0.9841951	0.51758
fetal small intestine 72	0.9852726	0.51762
fetal fibroblast 269	0.9899642	0.51764
fetal large intestine 62	0.9786059	0.52064
fetal heart 46	0.9828077	0.52336
fetal heart 47	0.9795066	0.52512
fibroblast 330	0.9844690	0.52938
hematopoietic stem cell 314	0.9838529	0.52980
ES derived mesoderm 285	0.9821485	0.53328
fetal large intestine 63	0.9733554	0.53472
fetal small intestine 80	0.9767512	0.53498
fibroblast 332	0.9836214	0.53580
fetal adrenal gland 25	0.9768267	0.53832
fetal lung 301	0.9771678	0.54354
fetal large intestine 54	0.9688908	0.54436
Mobilized CD56 324	0.9680538	0.54654
fetal small intestine 69	0.9662916	0.54684
hematopoietic stem cell 315	0.9682024	0.55600
placenta 197	0.9627431	0.55914
fetal lung 298	0.9687996	0.56172
fibroblast 331	0.9717815	0.56242
fetal small intestine 77	0.9575376	0.56572
fetal heart 42	0.9623029	0.56946
fetal thymus 266	0.9505052	0.57442
fetal large intestine 59	0.9498284	0.57760
fetal adrenal gland 23	0.9581533	0.58098
CD4 13	0.9404794	0.58132
fetal large intestine 65	0.9498488	0.58266
muscle 341	0.9450560	0.58486
fetal heart 43	0.9550442	0.58892
fetal small intestine 71	0.9470212	0.58908
fetal thymus 259	0.9368033	0.59402
hematopoietic stem cell 309	0.9486020	0.59736
CD8 19	0.9221627	0.60264
placenta 199	0.9441141	0.60470
fetal adrenal gland 24	0.9378126	0.60584
fetal large intestine 53	0.9237436	0.61154
fetal skin 234	0.9372825	0.61578
fetal small intestine 76	0.9283115	0.61940
placenta 196	0.9280872	0.62680
hematopoietic stem cell 306	0.9340030	0.62838
fetal adrenal gland 26	0.9308905	0.63008
stomach 283	0.9212931	0.63130
fetal heart 52	0.9342374	0.63410
hematopoietic stem cell 316	0.9239317	0.63618
fetal lung 299	0.9350074	0.63628
fetal large intestine 61	0.9137344	0.63658
fetal fibroblast 279	0.9390402	0.63982
CD3 11	0.9184891	0.64208
fetal fibroblast 270	0.9347632	0.64350
Mobilized CD4 323	0.9023638	0.64670
fetal thymus 264	0.9106071	0.64740
fetal fibroblast 280	0.9343405	0.65000
CD3 9	0.9039009	0.65108
fetal adrenal gland 27	0.9196417	0.65742
hematopoietic stem cell 305	0.9162445	0.66182
CD8 20	0.9024398	0.66238
fetal thymus 257	0.8866502	0.66574
Mobilized CD8 325	0.8826038	0.66946
CD4 14	0.9015302	0.67100
CD8 22	0.8834446	0.67170
fetal fibroblast 273	0.9223166	0.67234
hematopoietic stem cell 311	0.9068646	0.68008
fetal thymus 261	0.8863510	0.68166
CD3 10	0.8896035	0.68240
Mobilized CD3 321	0.8675721	0.68618
fetal stomach 244	0.8878764	0.68650
hematopoietic stem cell 317	0.9140364	0.68752
hematopoietic stem cell 308	0.9085258	0.69006
fetal fibroblast 282	0.9179437	0.69086
hematopoietic stem cell 313	0.9065789	0.69096
fetal lung 119	0.9113534	0.69176
hematopoietic stem cell 318	0.8955459	0.69280
Mobilized CD3 320	0.8698646	0.69576
fetal heart 48	0.8956624	0.70110
fetal large intestine 66	0.8826494	0.70206
fetal fibroblast 274	0.9066450	0.70588
CD4 15	0.8374567	0.70830
fetal fibroblast 271	0.9069377	0.70968
ES derived mesoderm 286	0.9069862	0.71964
hematopoietic stem cell 307	0.8860302	0.72108
fetal heart 51	0.8861070	0.72340
fetal lung 140	0.8918562	0.72470
ovary 326	0.8496484	0.72634
fetal lung 118	0.8954344	0.72670
fetal heart 44	0.8606979	0.72794
CD4 16	0.8392820	0.72878
fetal fibroblast 275	0.8980079	0.73080
fetal heart 50	0.8777457	0.73286
CD19 5	0.8523873	0.73440
CD19 7	0.8417529	0.73440
fetal fibroblast 268	0.8936612	0.73520
fetal lung 111	0.8860103	0.74236
fetal thymus 262	0.8088090	0.74474
fetal fibroblast 281	0.8883297	0.74628
fetal fibroblast 278	0.8859672	0.74720
fetal lung 129	0.8788869	0.74836
fetal spinal cord 236	0.8648508	0.74954
fetal stomach 253	0.8379201	0.75420
hematopoietic stem cell 319	0.8579453	0.75430
fetal stomach 242	0.8197594	0.75448
fetal lung 125	0.8687294	0.75610
fetal fibroblast 276	0.8856877	0.75670
fetal lung 121	0.8806577	0.75696
fetal brain 31	0.8972735	0.76118
fetal lung 116	0.8815635	0.76164
fetal stomach 247	0.8160333	0.76256
fetal brain 34	0.8878680	0.76296
fetal stomach 240	0.8268702	0.76362
fetal muscle 180	0.8479834	0.76878
CD19 6	0.8192328	0.76916
fetal fibroblast 277	0.8730676	0.77220
fetal muscle 175	0.8522356	0.77280
fetal renal cortex 207	0.8440962	0.77466
Mobilized CD4 322	0.8130244	0.77652
fetal lung 120	0.8591684	0.77836
CD3 12	0.8106542	0.77838
fetal muscle 153	0.8347525	0.77944
fetal renal pelvis 220	0.8376834	0.77972
fetal fibroblast 272	0.8715796	0.78324
fetal stomach 248	0.8223015	0.78384
fetal lung 132	0.8532263	0.78526
fetal brain 41	0.8618857	0.79128
fetal brain 38	0.8563539	0.79132
fetal kidney 85	0.8513706	0.79186
fetal brain 35	0.8764182	0.79332
fetal renal pelvis 227	0.8254188	0.79440
fetal lung 112	0.8484235	0.79586
fetal lung 109	0.8651526	0.79940
fetal muscle 167	0.8437045	0.80046
fetal spinal cord 238	0.8408773	0.80068
fetal muscle 176	0.8458310	0.80224
fetal kidney 95	0.8221997	0.80324
fetal stomach 243	0.8192688	0.80426
fetal lung 128	0.8650194	0.80474
fetal kidney 89	0.8169116	0.80500
fetal spinal cord 235	0.8366422	0.80514
fetal lung 133	0.8510770	0.80516
fetal kidney 87	0.8231079	0.80676
fetal kidney 84	0.8166393	0.80768
fetal stomach 252	0.8057680	0.80800
fetal kidney 81	0.8339281	0.80856
fetal lung 127	0.8365978	0.80908
fetal muscle 164	0.8253009	0.80978
fetal small intestine 75	0.7360000	0.81078
fetal lung 134	0.8374995	0.81090
fetal stomach 251	0.8043848	0.81110
fetal muscle 194	0.8377973	0.81292
fetal lung 113	0.8452501	0.81346
fetal muscle 166	0.8278553	0.81684
ES cells 294	0.7942758	0.81700
fetal kidney 100	0.8128127	0.81760
fetal renal pelvis 232	0.8076840	0.81812
fetal renal pelvis 221	0.8222043	0.81832
fetal renal pelvis 231	0.8240436	0.81854
fetal lung 130	0.8362834	0.81938
fetal lung 141	0.8448534	0.81998
fetal lung 110	0.8278251	0.82034
fetal lung 123	0.8266818	0.82334
fetal kidney 83	0.7913589	0.82336
fetal renal pelvis 228	0.7986751	0.82342
fetal muscle 168	0.8136868	0.82402
fetal spinal cord 237	0.8271239	0.82502
fetal thymus 263	0.8114001	0.82542
fetal renal cortex 215	0.8191161	0.82552
fetal lung 137	0.8359784	0.82600
fetal renal cortex 205	0.8118195	0.82732
fetal stomach 246	0.7800958	0.82756
fetal stomach 241	0.7841371	0.82810
fetal muscle 182	0.8184311	0.82946
fetal muscle 151	0.8007386	0.83040
fetal muscle 157	0.8281454	0.83444
fetal kidney 99	0.8021659	0.83490
fetal renal pelvis 233	0.8010968	0.83498
fetal kidney 91	0.8095963	0.83588
fetal renal cortex 214	0.8026422	0.83650
fetal muscle 149	0.8023507	0.83652
fetal brain 30	0.8663806	0.83706
fetal stomach 249	0.7697134	0.83754
fetal renal pelvis 219	0.7663340	0.83810
fetal ovary 195	0.7688649	0.83824
fetal muscle 178	0.8042192	0.83854
fetal muscle 193	0.8019264	0.83900
fetal renal cortex 212	0.8070944	0.84014
fetal muscle 173	0.7996868	0.84110
fetal muscle 188	0.8082117	0.84140
fetal lung 117	0.8256402	0.84154
fetal lung 126	0.8297041	0.84200
fetal kidney 106	0.7985287	0.84420
fetal stomach 250	0.7660698	0.84510
fetal renal pelvis 218	0.7566845	0.84566
fetal fibroblast 267	0.8249790	0.84580
iPS 304	0.8132778	0.84602
fetal lung 115	0.8224867	0.84662
ES cells 296	0.7685372	0.85014
fetal kidney 92	0.7956163	0.85032
fetal lung 131	0.8293770	0.85052
fetal muscle 187	0.8061930	0.85088
fetal testes 255	0.8028919	0.85096
fetal muscle 159	0.7854597	0.85146
fetal lung 138	0.8098350	0.85184
fetal renal pelvis 217	0.7827292	0.85224
fetal muscle 172	0.8167492	0.85256
fetal kidney 82	0.7726007	0.85368
fetal muscle 154	0.7853614	0.85448
fetal brain 37	0.8269722	0.85464
fetal muscle 165	0.8032923	0.85640
fetal brain 39	0.8310061	0.85682
fetal lung 108	0.7970774	0.85684
fetal muscle 169	0.7937938	0.85718
fetal lung 135	0.8041728	0.85826
fetal muscle 191	0.7891249	0.85828
fetal muscle 174	0.7889464	0.85874
iPS 302	0.7961282	0.85958
fetal stomach 245	0.7637802	0.86050
fetal muscle 148	0.7871358	0.86224
fetal renal cortex 213	0.7955632	0.86232
fetal renal pelvis 225	0.7789091	0.86238
fetal muscle 189	0.7919253	0.86252
fetal lung 114	0.8236376	0.86360
fetal brain 29	0.8432312	0.86398
fetal lung 139	0.7932906	0.86522
fetal renal pelvis 230	0.7596276	0.86610
fetal kidney 104	0.7736093	0.86618
fetal renal cortex 211	0.7625899	0.86662
fetal renal cortex 202	0.7818218	0.86776
fetal renal pelvis 226	0.7801752	0.86964
fetal kidney 103	0.7697765	0.86966
fetal renal cortex 204	0.7914510	0.86978
fetal renal cortex 206	0.7829395	0.87044
fetal muscle 146	0.7897402	0.87074
fetal lung 124	0.7947124	0.87080
fetal renal cortex 210	0.7691540	0.87110
fetal kidney 107	0.7756365	0.87170
fetal kidney 96	0.7810685	0.87278
fetal lung 136	0.7927150	0.87310
fetal kidney 105	0.7797624	0.87326
fetal renal pelvis 224	0.7843972	0.87348
fetal muscle 155	0.7625913	0.87356
fetal muscle 143	0.7924778	0.87700
fetal muscle 163	0.7707461	0.87742
fetal spinal cord 239	0.7885174	0.87754
fetal kidney 86	0.7295541	0.87788
fetal brain 40	0.8182565	0.87872
fetal kidney 88	0.7748932	0.87898
fetal muscle 192	0.7785581	0.88024
fetal muscle 184	0.7912235	0.88080
fetal muscle 156	0.7793604	0.88100
fetal muscle 144	0.7642135	0.88132
fetal renal pelvis 223	0.7583465	0.88146
fetal muscle 183	0.7832804	0.88266
fetal testes 254	0.7648707	0.88266
fetal kidney 101	0.7646103	0.88600
fetal renal pelvis 229	0.7622863	0.88732
fetal muscle 181	0.7735507	0.88844
fetal muscle 145	0.7418477	0.88846
fetal brain 36	0.8007670	0.88882
fetal muscle 179	0.7741475	0.88894
ES derived NP 291	0.7255658	0.89402
fetal renal pelvis 216	0.7506624	0.89412
fetal lung 142	0.7922952	0.89442
iPS 303	0.7696738	0.89450
fetal kidney 90	0.7513162	0.89524
fetal muscle 150	0.7489672	0.89616
fetal muscle 185	0.7505304	0.89662
fetal kidney 94	0.7353040	0.89712
fetal muscle 162	0.7680479	0.89764
fetal kidney 102	0.7345949	0.89778
ES cells 295	0.7235526	0.89914
fetal muscle 152	0.7696542	0.89918
fetal muscle 171	0.7617875	0.90096
ES derived NP 292	0.7283900	0.90490
fetal kidney 98	0.7371568	0.90552
fetal muscle 147	0.7440474	0.90660
fetal muscle 186	0.7538486	0.90896
fetal renal pelvis 222	0.7285396	0.91016
fetal muscle 170	0.7554427	0.91076
fetal renal cortex 208	0.7312690	0.91120
fetal brain 32	0.7860562	0.91420
fetal muscle 161	0.7375051	0.91630
ES cells 293	0.7196507	0.91638
fetal muscle 160	0.7325089	0.91712
fetal muscle 158	0.7455002	0.91820
fetal renal cortex 203	0.7245345	0.92030
fetal renal cortex 209	0.7231709	0.92218
fetal kidney 97	0.7196888	0.92478
fetal muscle 190	0.7066889	0.92892
fetal lung 122	0.7251745	0.92962
fetal muscle 177	0.7055687	0.93012
fetal kidney 93	0.7024380	0.93106
fetal brain 33	0.7525262	0.93660

Schizophrenia

DHS sample	fold enrichment	p value
fetal heart 47	1.3713777	0.05246
fetal fibroblast 271	1.2452268	0.06894
fetal fibroblast 272	1.2442928	0.06952
fetal fibroblast 276	1.2328196	0.07418
fetal heart 45	1.2964884	0.07778
fetal heart 49	1.2967326	0.07802
fetal fibroblast 275	1.2199227	0.08344
fetal brain 30	1.1652464	0.08544
fetal thymus 264	1.3330228	0.08702
fetal lung 298	1.2531140	0.09040
fetal fibroblast 277	1.2255095	0.09100
fetal brain 37	1.1909602	0.09156
fetal fibroblast 268	1.2098465	0.09614
fetal brain 36	1.1904305	0.10064
fetal fibroblast 273	1.2071304	0.10354
fetal thymus 265	1.3119477	0.10498
fibroblast 331	1.2121592	0.11058
fetal fibroblast 282	1.1913463	0.11228
fetal brain 40	1.1723043	0.11576
fetal thymus 259	1.3155137	0.11608
fetal brain 31	1.1431279	0.12428
fetal fibroblast 278	1.1924256	0.12640
fetal fibroblast 279	1.1917581	0.12852
fetal heart 46	1.2370455	0.12918
fetal fibroblast 274	1.1814573	0.13638
fetal thymus 256	1.2692116	0.13656
fetal brain 35	1.1374269	0.14490
fetal lung 300	1.1902078	0.14952
fetal brain 29	1.1267437	0.15024
fibroblast 332	1.1604777	0.17532
fetal fibroblast 267	1.1543398	0.17672
fetal lung 301	1.1672351	0.18250
fetal brain 34	1.1211455	0.18440
fetal thymus 258	1.2145854	0.18510
fetal fibroblast 281	1.1442790	0.18950
fetal heart 48	1.1703692	0.19290
fetal fibroblast 270	1.1418476	0.19500
fibroblast 329	1.1434299	0.20002
fetal fibroblast 280	1.1388926	0.20050
hematopoietic stem cell 310	1.1469203	0.20354
fetal thymus 266	1.1966284	0.20398
fetal brain 39	1.1155341	0.20412
fetal lung 299	1.1504448	0.20738
fibroblast 330	1.1397896	0.21100
fetal fibroblast 269	1.1278193	0.21536
fetal heart 42	1.1500981	0.22122
fetal thymus 261	1.1830884	0.22534
fetal heart 52	1.1356399	0.22616
fetal thymus 260	1.1578464	0.24894
fetal thymus 262	1.1817809	0.24988
ES derived MSC 289	1.1118850	0.27314
fetal thymus 257	1.1466047	0.27844
fetal heart 43	1.1001964	0.29084
fetal heart 50	1.1056552	0.29178
fetal adrenal gland 28	1.0883477	0.29798
fetal heart 51	1.0869747	0.31688
fetal brain 41	1.0586172	0.33568
fetal adrenal gland 25	1.0796466	0.34070
hematopoietic stem cell 317	1.0636832	0.34992
ES derived MSC 290	1.0606527	0.36156
fetal brain 38	1.0516056	0.36180
hematopoietic stem cell 309	1.0626882	0.36372
fetal lung 141	1.0498780	0.36910
fetal heart 44	1.0608354	0.38170
fetal spinal cord 237	1.0414058	0.39472
hematopoietic stem cell 315	1.0482379	0.39684
hematopoietic stem cell 314	1.0345488	0.41618
CD14 4	1.0378671	0.42156
fetal spinal cord 235	1.0244469	0.42384
hematopoietic stem cell 305	1.0279925	0.42976
fetal spinal cord 238	1.0237614	0.43072
fetal brain 32	1.0150464	0.45036
fetal muscle 172	1.0128892	0.45720
hematopoietic stem cell 312	1.0093155	0.46670
fetal adrenal gland 23	1.0073769	0.46960
hematopoietic stem cell 318	1.0048045	0.46976
fetal spinal cord 239	1.0014435	0.48016
heart 297	0.9999555	0.48716
CD56 18	0.9920384	0.48944
fetal brain 33	0.9956687	0.49822
fetal lung 117	0.9959771	0.49934
fetal adrenal gland 24	0.9882511	0.50278
hematopoietic stem cell 313	0.9904447	0.50314
hematopoietic stem cell 316	0.9868406	0.50386
fetal lung 118	0.9897307	0.51768
fetal lung 140	0.9858977	0.52350
CD14 3	0.9715315	0.52814
fetal muscle 181	0.9668176	0.55628
melanocyte 340	0.9725328	0.55864
fetal spinal cord 236	0.9613027	0.56016
CD56 17	0.9515676	0.56784
fetal muscle 194	0.9609003	0.57840
fetal lung 126	0.9591023	0.59064
hematopoietic stem cell 308	0.9480409	0.59116
CD8 20	0.9303097	0.59260
fetal lung 129	0.9541813	0.59566
fetal adrenal gland 27	0.9447974	0.59632
fetal lung 111	0.9564187	0.59902
fetal lung 132	0.9496267	0.60086
fetal muscle 150	0.9433709	0.60478
fetal lung 109	0.9558555	0.60512
hematopoietic stem cell 306	0.9370952	0.60558
CD19 7	0.9103121	0.60674
melanocyte 337	0.9555658	0.61240
fetal muscle 179	0.9409648	0.61256
Mobilized CD3 320	0.9071778	0.61876
hematopoietic stem cell 307	0.9305505	0.61884
fetal lung 121	0.9463926	0.62372
CD3 10	0.9090749	0.62412
hematopoietic stem cell 311	0.9252872	0.63054
melanocyte 338	0.9490474	0.63146
fetal lung 120	0.9370142	0.63198
fetal muscle 184	0.9344201	0.63290
fetal muscle 167	0.9320592	0.64016
Mobilized CD4 323	0.8866750	0.64018
fetal lung 128	0.9430529	0.64082
CD3 11	0.9023183	0.64112
fetal lung 116	0.9406839	0.64172
fetal lung 113	0.9378863	0.64274
fetal adrenal gland 26	0.9228759	0.64298
fetal muscle 147	0.9223267	0.64600
fetal muscle 151	0.9194643	0.64636
fetal lung 125	0.9250739	0.64868
fetal lung 124	0.9292896	0.64930
fetal renal pelvis 231	0.9162139	0.65034
fetal lung 136	0.9279313	0.65042
CD8 22	0.8769584	0.65058
fetal lung 119	0.9352112	0.65174
fetal muscle 182	0.9223712	0.65206
fetal small intestine 75	0.8349774	0.65290
fetal lung 131	0.9355334	0.65422
fetal muscle 153	0.9061722	0.66540
fetal lung 135	0.9192160	0.66560
CD8 19	0.8592651	0.66754
fetal renal cortex 209	0.8966005	0.66856
fetal lung 110	0.9159291	0.66888
Mobilized CD3 321	0.8589788	0.66994
fetal muscle 169	0.9130696	0.67348
fetal lung 114	0.9280358	0.67410
fetal muscle 183	0.9144217	0.67496
fetal muscle 154	0.8997215	0.68060
fetal lung 115	0.9163621	0.68442
melanocyte 339	0.9230221	0.68704
fetal large intestine 61	0.8639819	0.68976
fetal lung 133	0.9182070	0.69106
fetal muscle 160	0.9001177	0.69152
fetal lung 137	0.9084498	0.69510
fetal muscle 178	0.8935620	0.70176
fetal muscle 144	0.8834564	0.70388
fetal muscle 157	0.9032538	0.70504
Mobilized CD56 324	0.8578675	0.70546
fetal kidney 104	0.8746950	0.70552
fetal kidney 95	0.8756248	0.70732
fetal renal pelvis 230	0.8634065	0.70792
fetal lung 134	0.8982941	0.70796
hematopoietic stem cell 319	0.8708317	0.70948
fetal muscle 164	0.8872942	0.71244
fetal muscle 148	0.8875267	0.71270
fetal renal cortex 210	0.8663470	0.71322
fetal kidney 90	0.8712114	0.71446
fetal lung 138	0.8977320	0.71470
fetal lung 123	0.8942998	0.71590
fetal muscle 158	0.8946227	0.71740
CD3 12	0.8262633	0.71822
fetal kidney 82	0.8510999	0.72002
CD4 16	0.8193038	0.72006
ovary 326	0.8453785	0.72048
CD19 5	0.8395093	0.72126
fetal lung 130	0.8955505	0.72204
fetal muscle 146	0.8877998	0.72216
CD19 6	0.8297071	0.72394
CD3 9	0.8302671	0.72508
fetal renal cortex 215	0.8738035	0.72622
fetal kidney 107	0.8635086	0.72832
fetal kidney 93	0.8553062	0.73172
fetal kidney 96	0.8680263	0.73478
fetal muscle 175	0.8792823	0.73536
fetal muscle 187	0.8832273	0.73684
muscle 341	0.8471795	0.73964
fetal muscle 152	0.8788575	0.74400
Mobilized CD4 322	0.8060650	0.74492
CD8 21	0.8135434	0.74498
fetal renal cortex 208	0.8495318	0.74586
fetal kidney 105	0.8606336	0.74602
fetal kidney 97	0.8518837	0.74632
fetal muscle 155	0.8659624	0.74706
fetal muscle 159	0.8614792	0.74710
fetal renal cortex 206	0.8591996	0.74976
fetal lung 142	0.8874708	0.75036
fetal muscle 173	0.8687460	0.75048
fetal muscle 162	0.8746246	0.75106
fetal lung 122	0.8683874	0.75132
fetal kidney 89	0.8477840	0.75286
fetal renal cortex 204	0.8633845	0.75324
fetal renal cortex 203	0.8480033	0.75532
fetal lung 139	0.8715339	0.75652
fetal muscle 190	0.8513896	0.75784
fetal renal cortex 214	0.8502888	0.75832
fetal lung 127	0.8681878	0.76004
fetal muscle 165	0.8726650	0.76216
fetal kidney 81	0.8700635	0.76242
fetal renal pelvis 221	0.8517457	0.76366
fetal kidney 98	0.8395455	0.76368
CD4 15	0.7580451	0.76428
fetal kidney 99	0.8447736	0.76638
fetal muscle 192	0.8668848	0.76680
fetal muscle 185	0.8522132	0.76686
fetal renal pelvis 216	0.8373862	0.76726
fetal renal pelvis 223	0.8273385	0.76934
Mobilized CD8 325	0.7904693	0.76936
fetal muscle 180	0.8536492	0.76972
CD4 13	0.7956497	0.77064
fetal lung 112	0.8659150	0.77068
fetal muscle 189	0.8554849	0.77090
fetal lung 108	0.8560540	0.77220
fetal muscle 177	0.8461198	0.77328
fetal renal pelvis 227	0.8372489	0.77350
fetal kidney 103	0.8332355	0.77352
fetal kidney 106	0.8389205	0.77646
fetal muscle 170	0.8584832	0.77670
fetal kidney 92	0.8461680	0.77696
fetal muscle 191	0.8510125	0.77818
fetal renal cortex 205	0.8347070	0.77880
fetal renal cortex 212	0.8453950	0.77890
fetal renal pelvis 218	0.7898615	0.77972
fetal renal pelvis 226	0.8417259	0.77976
fetal kidney 88	0.8425408	0.78082
fetal muscle 186	0.8524066	0.78220
placenta 201	0.8212419	0.78312
fetal muscle 145	0.8326622	0.78416
fetal kidney 86	0.7940493	0.78520
CD4 14	0.8018758	0.78564
fetal renal pelvis 222	0.8231617	0.78612
fetal muscle 171	0.8520962	0.78924
fetal renal pelvis 232	0.8181736	0.79064
fetal muscle 163	0.8443227	0.79076
fetal muscle 166	0.8503607	0.79314
fetal muscle 188	0.8490497	0.79382
fetal muscle 176	0.8571577	0.79606
fetal muscle 193	0.8393074	0.79740
fetal testes 254	0.8265536	0.79758
fetal thymus 263	0.8359403	0.79776
fetal renal pelvis 228	0.8068944	0.79804
fetal testes 255	0.8429666	0.79912
fetal renal pelvis 217	0.8145076	0.80176
fetal renal pelvis 224	0.8320754	0.80184
fetal renal pelvis 219	0.7752942	0.80272
ES derived NP 292	0.8097111	0.80410
fetal renal cortex 202	0.8253338	0.80572
fetal kidney 102	0.7979732	0.81088
fetal renal pelvis 225	0.8033002	0.81146
fetal muscle 168	0.8262852	0.81528
fetal renal cortex 211	0.7970092	0.81862
fetal kidney 84	0.8036649	0.81870
fetal kidney 101	0.8132770	0.81890
fetal muscle 143	0.8440458	0.82118
fetal stomach 248	0.7879467	0.82256
fetal kidney 87	0.8131274	0.82408
fetal kidney 94	0.7911864	0.82506
fetal renal cortex 207	0.8080786	0.82540
fetal kidney 100	0.8019520	0.83048
fetal kidney 85	0.8322009	0.83146
fetal kidney 91	0.8157749	0.83292
fetal renal pelvis 220	0.7918331	0.83466
fetal ovary 195	0.7569031	0.83804
ES cells 294	0.7768616	0.84146
fetal muscle 149	0.8055861	0.84236
fetal renal pelvis 233	0.7894056	0.84320
fetal muscle 161	0.8084144	0.84396
fetal muscle 156	0.8130481	0.84494
keratinocyte 333	0.8523323	0.84552
fetal renal pelvis 229	0.7935350	0.84690
ES derived trophoblast 287	0.8010984	0.84834
fetal renal cortex 213	0.8035343	0.85126
ES derived NP 291	0.7602948	0.85474
placenta 199	0.7895799	0.85752
fetal large intestine 67	0.7739299	0.86404
fetal kidney 83	0.7384236	0.86910
fetal muscle 174	0.7855978	0.87254
ES cells 293	0.7550246	0.87608
placenta 196	0.7576186	0.87612
iPS 303	0.7840705	0.87864
fetal stomach 246	0.7301224	0.87924
placenta 200	0.7822054	0.88242
placenta 198	0.7763044	0.88384
ES cells 295	0.7339602	0.88462
iPS 302	0.7724039	0.88670
fetal stomach 251	0.7447502	0.88724
breast 2	0.8067709	0.88896
breast 1	0.8073504	0.88944
CD20 8	0.7652827	0.89018
keratinocyte 334	0.8231826	0.89278
fetal stomach 240	0.7136726	0.89284
fetal stomach 253	0.7110765	0.89878
fetal large intestine 53	0.7044680	0.90102
fetal stomach 250	0.7068150	0.90138
ES cells 296	0.7162408	0.90272
ES derived trophoblast 288	0.7725528	0.90436
fetal large intestine 60	0.7355121	0.90566
fetal stomach 245	0.7072008	0.90644
ES derived mesoderm 286	0.8002620	0.90668
fetal skin 234	0.7519007	0.90738
keratinocyte 336	0.8083264	0.90802
fetal stomach 242	0.6573997	0.90826
fetal large intestine 66	0.7165888	0.90858
fetal stomach 252	0.7141051	0.91008
fetal small intestine 76	0.7127178	0.91168
placenta 197	0.7176423	0.91420
fetal stomach 247	0.6608391	0.91554
fetal small intestine 68	0.7380109	0.91588
pancreas 327	0.6907347	0.91874
fetal large intestine 58	0.7394260	0.92182
iPS 304	0.7549222	0.92296
fetal large intestine 54	0.7062004	0.92452
fetal large intestine 63	0.7016091	0.92474
stomach 284	0.6994571	0.92532
fetal stomach 244	0.6974962	0.92552
fetal stomach 249	0.6734829	0.92558
fetal large intestine 59	0.6951024	0.92664
fetal large intestine 55	0.7212736	0.93026
ES derived mesoderm 285	0.7667460	0.93204
stomach 283	0.6783962	0.93258
fetal small intestine 72	0.6987256	0.93452
fetal small intestine 70	0.7340915	0.93462
fetal large intestine 62	0.6825108	0.93508
fetal large intestine 56	0.7002530	0.93564
fetal large intestine 65	0.6904763	0.93622
keratinocyte 335	0.7751323	0.93920
small intestine 342	0.6852758	0.94090
fetal stomach 243	0.6862102	0.94170
fetal small intestine 74	0.6927657	0.94186
fetal small intestine 78	0.7107574	0.94306
fetal small intestine 71	0.6849825	0.94322
fetal small intestine 80	0.6994941	0.94444
fetal stomach 241	0.6495651	0.94498
fetal small intestine 79	0.6672194	0.94638
fetal small intestine 73	0.6762928	0.94862
fetal small intestine 69	0.6552783	0.94906
fetal large intestine 57	0.6968546	0.95186
pancreas 328	0.6574239	0.95216
fetal large intestine 64	0.6841851	0.95270
fetal small intestine 77	0.6612207	0.95504

Body mass index 3

DHS sample	fold enrichment	p value
fetal lung 301	1.2293720	0.12606
fetal lung 298	1.2059523	0.15996
fetal lung 300	1.1936305	0.16176
melanocyte 337	1.1611232	0.16446
melanocyte 338	1.1553003	0.17470
fetal lung 299	1.1911087	0.17548
keratinocyte 334	1.1466894	0.18626
keratinocyte 333	1.1254456	0.22146
fetal fibroblast 275	1.1337781	0.22976
fetal fibroblast 271	1.1310142	0.23686
melanocyte 340	1.1269614	0.24122
ES derived MSC 289	1.1443879	0.24468
fetal fibroblast 273	1.1245255	0.24508
breast 1	1.1170063	0.25234
fetal fibroblast 272	1.1165676	0.26014
breast 2	1.1117219	0.26126
fetal fibroblast 278	1.1201945	0.26258
fetal fibroblast 276	1.1096664	0.27018
fetal fibroblast 277	1.1052450	0.28694
keratinocyte 335	1.0923227	0.28706
keratinocyte 336	1.0923409	0.28764
melanocyte 339	1.0936743	0.29114
fibroblast 332	1.0965215	0.29864
fetal lung 111	1.0954194	0.29926
ES derived MSC 290	1.1041376	0.30094
fetal fibroblast 274	1.0906823	0.30626
fibroblast 331	1.0913089	0.31082
fetal fibroblast 270	1.0855564	0.31622
fibroblast 330	1.0879857	0.31826
fetal fibroblast 268	1.0746820	0.33298
stomach 284	1.0921277	0.33302
fetal fibroblast 269	1.0723879	0.33726
fetal lung 133	1.0672934	0.34836
fetal lung 126	1.0619195	0.35244
fetal fibroblast 281	1.0603926	0.36338
fetal fibroblast 282	1.0538222	0.37070
fetal fibroblast 279	1.0559012	0.37722
fetal lung 118	1.0463559	0.38462
fetal lung 128	1.0439284	0.39056
Mobilized CD3 320	1.0701328	0.39072
fetal lung 141	1.0414153	0.39604
muscle 341	1.0507789	0.39694
fetal fibroblast 280	1.0357457	0.41166
fetal fibroblast 267	1.0362796	0.41476
Mobilized CD8 325	1.0391123	0.41802
fibroblast 329	1.0344082	0.41828
fetal lung 114	1.0287905	0.42576
fetal stomach 244	1.0274164	0.43214
fetal lung 129	1.0222725	0.44254
fetal lung 131	1.0160558	0.44954
placenta 198	1.0114663	0.46182
stomach 283	1.0095616	0.46194
ES cells 294	1.0081920	0.46206
pancreas 328	1.0091547	0.46332
placenta 200	1.0092931	0.46466
fetal stomach 253	1.0018343	0.46638
fetal lung 112	1.0065602	0.47286
fetal stomach 242	0.9916758	0.47324
fetal lung 119	1.0076072	0.47542
fetal lung 121	1.0051038	0.47662
fetal lung 115	1.0019567	0.48006
fetal lung 108	0.9981061	0.48530
pancreas 327	0.9914551	0.48658
fetal lung 132	0.9980167	0.48934
fetal lung 127	0.9957030	0.49254
fetal renal pelvis 220	0.9920662	0.49354
fetal heart 47	0.9904415	0.49762
fetal lung 137	0.9926356	0.50014
fetal lung 116	0.9950982	0.50144
fetal lung 139	0.9886171	0.50630
fetal lung 134	0.9870207	0.51200
CD8 19	0.9575567	0.51492
fetal kidney 83	0.9726086	0.51590
fetal lung 113	0.9866796	0.51792
fetal lung 140	0.9852195	0.52072
fetal skin 234	0.9810727	0.52102
fetal lung 120	0.9823258	0.52280
fetal lung 142	0.9830790	0.52378
fetal kidney 87	0.9751611	0.52554
fetal large intestine 53	0.9620388	0.52734
ES derived NP 291	0.9675267	0.52904
CD4 13	0.9557782	0.52916
fetal kidney 100	0.9713026	0.53034
ovary 326	0.9514105	0.53826
fetal lung 136	0.9725456	0.54176
fetal muscle 143	0.9726236	0.54272
fetal adrenal gland 28	0.9676166	0.54476
fetal adrenal gland 23	0.9654157	0.54564
fetal stomach 251	0.9555029	0.54786
fetal stomach 248	0.9541713	0.55344
small intestine 342	0.9575763	0.55582
fetal small intestine 77	0.9545073	0.55620
fetal lung 124	0.9640789	0.55928
fetal kidney 91	0.9613434	0.56076
fetal lung 125	0.9619579	0.56210
Mobilized CD56 324	0.9426428	0.56698
fetal adrenal gland 26	0.9508569	0.56842
fetal kidney 92	0.9562800	0.56844
fetal stomach 243	0.9471765	0.56916
placenta 197	0.9481950	0.56940
fetal lung 130	0.9592620	0.57056
fetal muscle 159	0.9523085	0.57104
placenta 196	0.9473048	0.57384
fetal renal pelvis 219	0.9301239	0.57616
CD56 18	0.9246368	0.57770
fetal kidney 84	0.9408939	0.57924
fetal muscle 153	0.9472652	0.58064
ES derived NP 292	0.9404005	0.58136
fetal lung 109	0.9579057	0.58298
fetal heart 49	0.9476260	0.58450
fetal renal cortex 214	0.9416828	0.58778
fetal renal pelvis 221	0.9429685	0.58912
fetal muscle 168	0.9439638	0.58930
fetal stomach 246	0.9250354	0.58930
fetal kidney 96	0.9401764	0.59420
fetal large intestine 67	0.9369073	0.59436
fetal lung 110	0.9433790	0.59626
fetal lung 123	0.9448070	0.59632
fetal stomach 249	0.9152038	0.59886
fetal kidney 81	0.9434116	0.59950
fetal lung 122	0.9380629	0.60014
fetal kidney 89	0.9315140	0.60278
fetal muscle 154	0.9326724	0.60554
fetal lung 138	0.9397428	0.60868
fetal testes 255	0.9362077	0.61060
fetal lung 135	0.9371697	0.61302
heart 297	0.9281392	0.61344
fetal kidney 86	0.9029767	0.61848
ES cells 296	0.9080051	0.62118
placenta 199	0.9233321	0.62194
fetal lung 117	0.9381908	0.62208
fetal heart 45	0.9275044	0.62266
CD8 22	0.8790264	0.62304
fetal renal pelvis 229	0.9227987	0.62316
fetal muscle 175	0.9288548	0.62394
fetal renal pelvis 230	0.9101883	0.62408
CD3 9	0.8920843	0.62446
fetal muscle 193	0.9267667	0.62464
fetal thymus 265	0.9022811	0.62514
fetal large intestine 58	0.9245049	0.62790
fetal muscle 155	0.9201240	0.62864
fetal small intestine 78	0.9233380	0.62902
fetal muscle 194	0.9328832	0.62918
fetal stomach 250	0.8991000	0.63040
fetal kidney 94	0.9128037	0.63052
fetal thymus 262	0.8622210	0.63194
fetal adrenal gland 25	0.9114751	0.63276
fetal renal pelvis 227	0.9146353	0.63440
iPS 304	0.9203961	0.63582
Mobilized CD4 323	0.8759841	0.63718
CD4 16	0.8623436	0.63734
fetal kidney 95	0.9148507	0.63736
CD19 7	0.8696001	0.63760
fetal small intestine 74	0.9119714	0.63778
CD14 4	0.9005492	0.63840
fetal kidney 103	0.9121069	0.63864
CD3 12	0.8676685	0.63872
Mobilized CD3 321	0.8604465	0.63946
hematopoietic stem cell 315	0.9113183	0.64018
fetal renal pelvis 217	0.9069156	0.64042
fetal small intestine 76	0.8999424	0.64136
fetal renal pelvis 232	0.9050877	0.64142
fetal heart 46	0.9157148	0.64270
fetal muscle 172	0.9276203	0.64374
hematopoietic stem cell 318	0.9020365	0.64510
fetal renal pelvis 218	0.8810569	0.64658
ES cells 295	0.8954576	0.64718
fetal kidney 106	0.9087865	0.64786
fetal renal pelvis 231	0.9112916	0.65032
fetal stomach 252	0.8890496	0.65032
fetal large intestine 62	0.8929460	0.65148
fetal small intestine 69	0.8892047	0.65344
CD3 11	0.8820512	0.65352
fetal kidney 88	0.9075576	0.65408
fetal thymus 266	0.8798953	0.65556
fetal large intestine 65	0.8939658	0.65778
fetal kidney 107	0.9008335	0.65910
fetal muscle 181	0.9095957	0.65914
fetal muscle 180	0.9057993	0.65914
fetal renal cortex 211	0.8903094	0.65924
fetal large intestine 61	0.8690859	0.65936
Mobilized CD4 322	0.8520933	0.66018
fetal large intestine 60	0.8944001	0.66082
fetal renal pelvis 224	0.9048360	0.66110
fetal renal cortex 212	0.9040364	0.66168
hematopoietic stem cell 309	0.8998241	0.66276
fetal adrenal gland 27	0.9016015	0.66358
fetal stomach 240	0.8764765	0.66406
CD8 21	0.8530685	0.66416
fetal renal cortex 213	0.9041718	0.66716
fetal stomach 241	0.8756520	0.66716
fetal small intestine 73	0.8881461	0.66722
fetal thymus 263	0.9022446	0.66810
fetal large intestine 59	0.8812392	0.67116
fetal renal pelvis 225	0.8891865	0.67128
fetal renal cortex 204	0.9002095	0.67172
fetal kidney 105	0.8971546	0.67280
fetal renal cortex 207	0.8944751	0.67280
fetal kidney 98	0.8905760	0.67342
fetal kidney 101	0.8939191	0.67546
fetal renal cortex 205	0.8919784	0.67746
fetal muscle 148	0.8966562	0.67770
fetal muscle 179	0.8986495	0.67808
fetal muscle 169	0.8965893	0.67892
fetal heart 43	0.9067147	0.67968
fetal stomach 247	0.8544626	0.68038
fetal kidney 104	0.8815830	0.68072
CD14 3	0.8610732	0.68098
fetal small intestine 70	0.8996027	0.68138
fetal kidney 93	0.8794559	0.68312
hematopoietic stem cell 316	0.8767982	0.68402
fetal renal cortex 202	0.8910317	0.68454
fetal kidney 102	0.8782329	0.68480
fetal muscle 149	0.8872876	0.68492
fetal muscle 150	0.8919736	0.68498
fetal renal cortex 206	0.8872832	0.68650
fetal renal cortex 210	0.8766303	0.68652
fetal renal cortex 208	0.8800921	0.69060
fetal thymus 264	0.8581529	0.69066
fetal small intestine 75	0.7942358	0.69140
fetal renal pelvis 216	0.8779999	0.69208
placenta 201	0.8759384	0.69296
fetal renal pelvis 233	0.8792292	0.69360
fetal muscle 160	0.8870742	0.69400
hematopoietic stem cell 312	0.8880188	0.69474
fetal thymus 257	0.8362895	0.69630
fetal heart 42	0.8889730	0.69650
hematopoietic stem cell 314	0.8892406	0.69654
fetal kidney 82	0.8611639	0.69794
CD8 20	0.8472955	0.69806
fetal muscle 151	0.8755003	0.69970
fetal brain 40	0.9119917	0.70014
fetal small intestine 71	0.8695105	0.70044
fetal small intestine 79	0.8632163	0.70076
fetal adrenal gland 24	0.8617887	0.70260
fetal thymus 260	0.8440657	0.70322
fetal muscle 182	0.8868363	0.70470
hematopoietic stem cell 305	0.8705427	0.70484
fetal renal cortex 215	0.8783030	0.70566
fetal renal pelvis 228	0.8626180	0.70772
CD4 14	0.8451184	0.70782
iPS 303	0.8779194	0.71076
hematopoietic stem cell 308	0.8768537	0.71104
hematopoietic stem cell 310	0.8887635	0.71122
fetal thymus 259	0.8301219	0.71198
fetal muscle 156	0.8792457	0.71352
fetal renal pelvis 222	0.8629796	0.71454
fetal thymus 258	0.8424448	0.71472
fetal kidney 90	0.8651922	0.71506
fetal brain 30	0.9168528	0.71518
fetal kidney 97	0.8671290	0.71544
fetal ovary 195	0.8389754	0.71732
fetal brain 37	0.9000847	0.72022
ES cells 293	0.8547939	0.72226
iPS 302	0.8669197	0.72260
fetal muscle 186	0.8768645	0.72316
CD20 8	0.8667390	0.72448
fetal stomach 245	0.8458615	0.72462
fetal large intestine 54	0.8521865	0.72474
fetal muscle 189	0.8706399	0.72508
fetal large intestine 55	0.8611949	0.72528
fetal small intestine 72	0.8551505	0.72550
fetal testes 254	0.8645697	0.72646
hematopoietic stem cell 317	0.8775731	0.72724
fetal thymus 256	0.8363486	0.72832
fetal muscle 164	0.8694168	0.72944
fetal heart 48	0.8684607	0.73076
fetal kidney 85	0.8810617	0.73126
fetal renal cortex 203	0.8546019	0.73178
CD4 15	0.7543694	0.73320
fetal large intestine 66	0.8423787	0.73474
fetal muscle 178	0.8643838	0.73520
fetal muscle 165	0.8742862	0.73678
fetal muscle 171	0.8715529	0.73680
fetal muscle 167	0.8684707	0.73716
fetal brain 35	0.9008217	0.73866
fetal muscle 146	0.8659991	0.73950
fetal large intestine 64	0.8489132	0.73986
fetal muscle 158	0.8652504	0.74198
fetal renal cortex 209	0.8500226	0.74200
CD56 17	0.8291811	0.74310
fetal thymus 261	0.8131842	0.74346
fetal large intestine 57	0.8537418	0.74698
fetal muscle 185	0.8519270	0.74752
fetal renal pelvis 226	0.8548005	0.74906
fetal spinal cord 238	0.8671443	0.75038
fetal muscle 157	0.8662012	0.75076
fetal small intestine 68	0.8481730	0.75232
fetal heart 51	0.8581119	0.75436
ES derived trophoblast 288	0.8606969	0.75484
fetal muscle 144	0.8440627	0.75668
fetal muscle 184	0.8606682	0.75814
fetal large intestine 63	0.8255857	0.75978
fetal muscle 152	0.8571925	0.76044
fetal muscle 166	0.8537302	0.76114
fetal brain 29	0.8942248	0.76172
fetal renal pelvis 223	0.8282277	0.76246
fetal kidney 99	0.8419493	0.76374
fetal muscle 176	0.8556998	0.76412
hematopoietic stem cell 313	0.8440199	0.76558
fetal spinal cord 236	0.8522579	0.76620
fetal spinal cord 239	0.8558755	0.76746
fetal brain 34	0.8841248	0.76776
fetal brain 39	0.8756203	0.76968
fetal muscle 183	0.8506846	0.76974
CD3 10	0.7836270	0.77050
fetal muscle 191	0.8426348	0.77076
CD19 6	0.7648561	0.77166
fetal heart 50	0.8415538	0.77276
fetal brain 31	0.8892630	0.77368
fetal muscle 173	0.8400582	0.77428
fetal muscle 170	0.8450557	0.77676
fetal muscle 188	0.8426349	0.77812
hematopoietic stem cell 311	0.8306272	0.77828
fetal muscle 147	0.8360221	0.78198
hematopoietic stem cell 306	0.8227887	0.78460
hematopoietic stem cell 319	0.8162855	0.78472
fetal muscle 187	0.8404930	0.78630
hematopoietic stem cell 307	0.8218134	0.78764
fetal large intestine 56	0.8160292	0.78900
CD19 5	0.7626740	0.78912
fetal brain 38	0.8580517	0.79008
fetal heart 44	0.7962368	0.79752
ES derived trophoblast 287	0.8185810	0.79914
fetal muscle 163	0.8212935	0.80272
fetal spinal cord 237	0.8291615	0.81170
fetal heart 52	0.8303112	0.81196
fetal spinal cord 235	0.8246015	0.82356
fetal muscle 145	0.7870843	0.82770
fetal muscle 192	0.8128272	0.82950
fetal brain 36	0.8372932	0.83390
fetal muscle 161	0.7956009	0.83674
fetal brain 41	0.8324135	0.83786
fetal muscle 162	0.8061808	0.83864
fetal muscle 190	0.7773308	0.84522
fetal small intestine 80	0.7731139	0.84888
fetal brain 33	0.8162599	0.85042
fetal muscle 174	0.7863154	0.85072
fetal muscle 177	0.7687352	0.85920
ES derived mesoderm 285	0.7775407	0.89468
ES derived mesoderm 286	0.7886994	0.89642
fetal brain 32	0.7827050	0.91066

Blood metabolites 1

DHS sample	fold enrichment	p value
small intestine 342	1.2147503	0.03510
fetal large intestine 58	1.1870680	0.04222
fetal small intestine 78	1.1584913	0.06670
fetal large intestine 57	1.1559461	0.07674
fetal small intestine 74	1.1516697	0.09660
fetal small intestine 80	1.1461256	0.09840
fetal large intestine 67	1.1506398	0.10212
fetal large intestine 55	1.1404653	0.10276
CD20 8	1.1332934	0.11352
fetal large intestine 60	1.1409569	0.11564
fetal small intestine 68	1.1307069	0.12022
fetal large intestine 64	1.1303661	0.12238
fetal small intestine 70	1.1051860	0.14866
fetal large intestine 54	1.1178426	0.15444
fetal small intestine 69	1.1116852	0.18018
fetal small intestine 72	1.0954783	0.20136
fetal large intestine 56	1.0918083	0.21000
fetal small intestine 73	1.0898415	0.21890
fetal large intestine 62	1.0914856	0.22344
fetal large intestine 63	1.0866043	0.23116
fetal small intestine 79	1.0851162	0.23912
fetal small intestine 71	1.0635455	0.28978
stomach 284	1.0677887	0.29088
fetal small intestine 77	1.0615130	0.29704
fetal small intestine 76	1.0371535	0.37136
pancreas 328	1.0364906	0.37452
fetal large intestine 59	1.0311128	0.38888
melanocyte 338	1.0228532	0.39688
breast 2	1.0152602	0.43018
melanocyte 340	1.0161996	0.43098
fetal large intestine 65	1.0161522	0.43810
fetal large intestine 66	1.0130808	0.44824
keratinocyte 334	1.0110102	0.44952
breast 1	1.0078327	0.46160
melanocyte 337	1.0004459	0.49510
stomach 283	0.9899846	0.52180
keratinocyte 333	0.9942181	0.52456
fetal large intestine 53	0.9834976	0.54144
fetal stomach 252	0.9829713	0.54288
pancreas 327	0.9828433	0.54338
melanocyte 339	0.9867547	0.55192
fetal thymus 260	0.9783836	0.55346
keratinocyte 336	0.9855191	0.56336
fetal thymus 258	0.9674342	0.58960
keratinocyte 335	0.9796074	0.59280
CD3 9	0.9625644	0.59672
fetal stomach 244	0.9584035	0.62470
Mobilized CD3 320	0.9475194	0.62934
fetal heart 45	0.9594010	0.63170
fetal thymus 259	0.9487800	0.63356
fetal adrenal gland 25	0.9556361	0.63948
fetal thymus 256	0.9503540	0.64462
fetal adrenal gland 28	0.9602576	0.64830
CD4 15	0.9298581	0.64834
fetal stomach 246	0.9441771	0.65730
fetal stomach 241	0.9434924	0.65918
fetal stomach 251	0.9466129	0.66428
muscle 341	0.9445562	0.66428
fetal stomach 242	0.9342042	0.66434
iPS 304	0.9515426	0.66506
Mobilized CD8 325	0.9310744	0.66970
fetal thymus 264	0.9414030	0.67010
fetal thymus 257	0.9343999	0.67190
fetal stomach 247	0.9338752	0.67292
fetal stomach 248	0.9394977	0.68016
fetal stomach 240	0.9323159	0.68838
CD8 21	0.9250384	0.69078
placenta 199	0.9435453	0.69184
fetal adrenal gland 27	0.9395813	0.69892
Mobilized CD56 324	0.9307786	0.70026
fetal adrenal gland 24	0.9304956	0.70750
fetal thymus 265	0.9268674	0.70782
fetal thymus 261	0.9233127	0.71048
ES cells 296	0.9233448	0.71160
CD3 11	0.9252624	0.71276
CD4 13	0.9170473	0.71344
placenta 198	0.9398752	0.71628
fetal stomach 253	0.9233789	0.71700
placenta 200	0.9407628	0.71736
fetal thymus 266	0.9210215	0.72002
fetal heart 42	0.9314500	0.72246
fetal stomach 243	0.9292945	0.72342
placenta 197	0.9276945	0.72702
ES cells 294	0.9160100	0.72764
fetal large intestine 61	0.9129144	0.72860
fetal stomach 250	0.9188984	0.72952
CD56 18	0.9058253	0.74052
hematopoietic stem cell 310	0.9382315	0.74218
fetal heart 46	0.9218168	0.74336
fetal stomach 245	0.9171607	0.74382
fetal stomach 249	0.9110124	0.74794
CD8 22	0.8951505	0.75802
fetal thymus 262	0.8812702	0.75868
Mobilized CD3 321	0.8902570	0.75936
fetal heart 44	0.9040594	0.75974
Mobilized CD4 322	0.8935228	0.76060
fetal small intestine 75	0.8653035	0.76198
Mobilized CD4 323	0.8942963	0.76732
fetal heart 51	0.9175212	0.76746
CD14 4	0.9095285	0.76880
fetal ovary 195	0.8968426	0.76896
fetal lung 111	0.9273735	0.76996
hematopoietic stem cell 314	0.9247393	0.77034
CD8 19	0.8818603	0.77200
fetal testes 254	0.9135348	0.77428
fetal testes 255	0.9175335	0.77836
CD19 7	0.8841660	0.77954
iPS 303	0.9133203	0.78486
fetal adrenal gland 23	0.9152879	0.78810
fetal heart 48	0.9046070	0.79980
fetal heart 47	0.8931121	0.80026
CD3 12	0.8723473	0.80626
fibroblast 330	0.9157692	0.80628
fetal fibroblast 270	0.9148746	0.81038
fibroblast 329	0.9151620	0.81166
fetal heart 49	0.8979606	0.81296
CD8 20	0.8827839	0.81506
fetal lung 129	0.9097049	0.81878
CD14 3	0.8826106	0.81886
ES derived MSC 289	0.9015494	0.82052
ES cells 293	0.8853864	0.82084
hematopoietic stem cell 309	0.9013272	0.82248
placenta 196	0.8926298	0.82444
fetal kidney 83	0.8802521	0.82516
CD19 6	0.8626183	0.82754
fetal lung 125	0.9008549	0.82800
CD3 10	0.8704841	0.82858
fetal fibroblast 280	0.9114156	0.82882
heart 297	0.8854147	0.83094
fetal heart 43	0.8966167	0.83122
ES cells 295	0.8747551	0.83326
fetal fibroblast 269	0.9089004	0.83374
hematopoietic stem cell 319	0.8874025	0.83376
fetal lung 134	0.8988967	0.83766
fetal lung 115	0.9046433	0.83840
fetal lung 298	0.8985216	0.83866
fetal adrenal gland 26	0.8891371	0.83934
fetal lung 133	0.9054641	0.84026
fetal lung 119	0.9072656	0.84086
fetal kidney 106	0.8846328	0.84396
iPS 302	0.8883192	0.84478
hematopoietic stem cell 308	0.8949408	0.84560
fibroblast 332	0.9046710	0.84694
fetal heart 52	0.8939179	0.84748
CD4 16	0.8468336	0.84854
ES derived mesoderm 285	0.8989941	0.84948
ES derived MSC 290	0.8921450	0.84950
fetal lung 108	0.8907539	0.85094
fetal renal pelvis 217	0.8768603	0.85126
fibroblast 331	0.8995047	0.85594
fetal renal pelvis 216	0.8757680	0.85666
hematopoietic stem cell 311	0.8853907	0.85728
fetal kidney 85	0.8895870	0.85742
fetal fibroblast 279	0.8979559	0.86062
fetal lung 122	0.8838224	0.86140
fetal renal pelvis 228	0.8691272	0.86278
fetal lung 130	0.8937141	0.86284
CD19 5	0.8494157	0.86324
CD4 14	0.8588617	0.86630
fetal lung 140	0.8934409	0.86736
hematopoietic stem cell 307	0.8784152	0.86946
ES derived NP 291	0.8564342	0.87116
ovary 326	0.8489851	0.87274
fetal renal pelvis 231	0.8759721	0.87404
hematopoietic stem cell 318	0.8695005	0.87482
fetal renal pelvis 219	0.8442616	0.87510
fetal lung 299	0.8805191	0.87842
hematopoietic stem cell 305	0.8702619	0.87968
fetal lung 138	0.8816853	0.88138
fetal heart 50	0.8670949	0.88278
fetal renal pelvis 227	0.8650714	0.88294
hematopoietic stem cell 312	0.8773415	0.88312
fetal fibroblast 271	0.8900957	0.88368
fetal lung 110	0.8790170	0.88416
fetal lung 141	0.8876900	0.88422
hematopoietic stem cell 316	0.8632049	0.88438
fetal lung 139	0.8749379	0.88484
fetal kidney 99	0.8660106	0.88506
CD56 17	0.8534457	0.88558
fetal kidney 103	0.8620913	0.88636
hematopoietic stem cell 315	0.8658711	0.88920
fetal lung 117	0.8817105	0.88932
hematopoietic stem cell 306	0.8694041	0.88996
fetal kidney 95	0.8618773	0.89126
fetal renal pelvis 226	0.8635474	0.89136
fetal lung 126	0.8853497	0.89152
ES derived mesoderm 286	0.8888695	0.89276
fetal kidney 107	0.8591777	0.89304
fetal kidney 104	0.8562718	0.89338
fetal lung 128	0.8885131	0.89352
fetal lung 137	0.8781017	0.89382
fetal lung 121	0.8850842	0.89420
fetal lung 301	0.8764838	0.89424
fetal lung 142	0.8829122	0.89518
fetal lung 127	0.8711349	0.89640
fetal lung 112	0.8724170	0.89714
hematopoietic stem cell 313	0.8704447	0.89716
fetal kidney 84	0.8532495	0.89750
fetal lung 116	0.8845832	0.90056
fetal renal cortex 205	0.8548490	0.90228
fetal lung 300	0.8739576	0.90310
fetal kidney 87	0.8556389	0.90346
fetal fibroblast 276	0.8811388	0.90426
fetal kidney 89	0.8497566	0.90454
fetal renal cortex 211	0.8423429	0.90556
fetal renal pelvis 232	0.8444489	0.90626
fetal lung 123	0.8683400	0.90662
fetal renal pelvis 220	0.8477829	0.90688
fetal lung 118	0.8799368	0.90692
fetal renal pelvis 225	0.8446482	0.90746
fetal fibroblast 281	0.8765720	0.90806
fetal kidney 82	0.8330858	0.91112
fetal fibroblast 272	0.8754979	0.91276
fetal fibroblast 275	0.8768367	0.91280
fetal renal cortex 214	0.8484374	0.91400
fetal renal pelvis 223	0.8366703	0.91432
fetal kidney 98	0.8419839	0.91564
fetal muscle 149	0.8481610	0.91570
fetal lung 131	0.8749124	0.91676
fetal kidney 100	0.8425722	0.91816
fetal lung 124	0.8620474	0.91860
placenta 201	0.8405899	0.92000
fetal kidney 102	0.8301006	0.92130
fetal kidney 86	0.8127508	0.92290
fetal lung 136	0.8570490	0.92318
fetal renal cortex 206	0.8431645	0.92352
fetal renal pelvis 230	0.8245211	0.92660
fetal kidney 93	0.8276840	0.92662
ES derived NP 292	0.8275487	0.92774
fetal renal cortex 207	0.8398687	0.92874
fetal lung 109	0.8693275	0.92880
fetal fibroblast 274	0.8642707	0.92976
fetal renal pelvis 233	0.8337305	0.92984
fetal renal pelvis 218	0.8021561	0.93042
fetal kidney 96	0.8390851	0.93148
fetal muscle 153	0.8325876	0.93166
fetal kidney 97	0.8315266	0.93198
fetal skin 234	0.8355711	0.93206
ES derived trophoblast 288	0.8444367	0.93362
fetal muscle 159	0.8319567	0.93396
fetal fibroblast 282	0.8653017	0.93412
fetal muscle 173	0.8330364	0.93470
fetal lung 132	0.8487892	0.93560
fetal kidney 94	0.8190199	0.93672
fetal muscle 194	0.8500132	0.93964
fetal kidney 91	0.8381991	0.94034
fetal muscle 180	0.8306205	0.94066
fetal lung 114	0.8589009	0.94104
fetal kidney 88	0.8311930	0.94156
ES derived trophoblast 287	0.8183672	0.94328
fetal renal pelvis 229	0.8255084	0.94352
fetal kidney 105	0.8272422	0.94376
fetal muscle 147	0.8267064	0.94380
hematopoietic stem cell 317	0.8501039	0.94462
fetal muscle 154	0.8199595	0.94480
fetal kidney 90	0.8187946	0.94490
fetal thymus 263	0.8257491	0.94508
fetal lung 113	0.8525963	0.94516
fetal lung 135	0.8404908	0.94576
fetal kidney 101	0.8221183	0.94592
fetal fibroblast 278	0.8470752	0.94612
fetal lung 120	0.8436180	0.94662
fetal muscle 193	0.8220546	0.94734
fetal renal cortex 213	0.8295025	0.94846
fetal muscle 150	0.8235949	0.94876
fetal renal cortex 202	0.8233145	0.94890
fetal muscle 168	0.8199569	0.94936
fetal fibroblast 273	0.8496963	0.94964
fetal muscle 145	0.8082005	0.94970
fetal spinal cord 236	0.8255020	0.94996
fetal muscle 177	0.8123028	0.95010
fetal kidney 92	0.8228149	0.95066
fetal muscle 151	0.8145424	0.95136
fetal fibroblast 268	0.8495872	0.95284
fetal muscle 164	0.8224007	0.95528
fetal renal cortex 208	0.8041004	0.95536
fetal muscle 192	0.8206913	0.95590
fetal fibroblast 277	0.8396033	0.95600
fetal renal cortex 215	0.8171724	0.95684
fetal renal pelvis 221	0.8145584	0.95760
fetal renal pelvis 222	0.8014385	0.95808
fetal muscle 190	0.8027274	0.95878
fetal muscle 182	0.8180377	0.95884
fetal renal cortex 212	0.8131697	0.95904
fetal muscle 152	0.8233820	0.95954
fetal muscle 148	0.8111025	0.96070
fetal muscle 158	0.8196644	0.96090
fetal muscle 171	0.8206971	0.96110
fetal fibroblast 267	0.8326609	0.96188
fetal muscle 174	0.8058982	0.96228
fetal renal cortex 204	0.8137863	0.96322
fetal muscle 191	0.8054905	0.96432
fetal renal cortex 203	0.7963911	0.96504
fetal renal pelvis 224	0.8030931	0.96760
fetal muscle 155	0.7929758	0.96800
fetal muscle 156	0.8060517	0.96870
fetal muscle 165	0.8124339	0.96944
fetal brain 36	0.8326522	0.96946
fetal muscle 169	0.8004739	0.97104
fetal renal cortex 210	0.7808964	0.97108
fetal muscle 175	0.8024282	0.97214
fetal muscle 163	0.7911917	0.97218
fetal muscle 189	0.7958145	0.97254
fetal muscle 144	0.7875271	0.97380
fetal muscle 172	0.8129422	0.97522
fetal muscle 185	0.7843250	0.97526
fetal renal cortex 209	0.7808746	0.97546
fetal muscle 178	0.7888369	0.97584
fetal muscle 167	0.8011521	0.97668
fetal muscle 160	0.7863901	0.97676
fetal muscle 162	0.7960965	0.97808
fetal muscle 187	0.7950284	0.97822
fetal muscle 146	0.7927553	0.97836
fetal muscle 170	0.7935781	0.97896
fetal muscle 183	0.7916134	0.97918
fetal muscle 186	0.7885681	0.98008
fetal muscle 176	0.7939961	0.98036
fetal spinal cord 239	0.7903649	0.98052
fetal muscle 181	0.7827432	0.98146
fetal kidney 81	0.7878114	0.98164
fetal muscle 179	0.7813521	0.98204
fetal muscle 161	0.7755775	0.98232
fetal muscle 166	0.7852982	0.98276
fetal muscle 188	0.7796311	0.98378
fetal muscle 143	0.7890396	0.98642
fetal spinal cord 238	0.7809941	0.98686
fetal brain 40	0.8054579	0.98848
fetal muscle 157	0.7741160	0.98980
fetal muscle 184	0.7671637	0.99012
fetal brain 38	0.7817159	0.99150
fetal brain 37	0.7766876	0.99428
fetal spinal cord 237	0.7564443	0.99430
fetal spinal cord 235	0.7501311	0.99510
fetal brain 41	0.7690453	0.99574
fetal brain 29	0.7924893	0.99642
fetal brain 32	0.7547813	0.99782
fetal brain 33	0.7371097	0.99802
fetal brain 39	0.7453783	0.99868
fetal brain 30	0.7674334	0.99892
fetal brain 35	0.7535484	0.99908
fetal brain 31	0.7581222	0.99926
fetal brain 34	0.7360996	0.99934

Metabolic syndrome traits

DHS sample	fold enrichment	p value
fetal thymus 256	1.2307276	0.10542
keratinocyte 333	1.1351847	0.14288
fetal thymus 265	1.1900142	0.16012
fetal thymus 258	1.1887541	0.16782
keratinocyte 334	1.1165954	0.18566
keratinocyte 336	1.1098826	0.19430
fetal thymus 264	1.1563530	0.20038
fetal thymus 260	1.1512102	0.21468
fetal fibroblast 269	1.1065569	0.21536
fetal adrenal gland 28	1.1135890	0.22036
fetal thymus 259	1.1438649	0.23380
muscle 341	1.1328746	0.23850
keratinocyte 335	1.0862578	0.25122
pancreas 328	1.1197434	0.25260
CD14 4	1.1071467	0.27716
fibroblast 329	1.0797825	0.28654
fibroblast 330	1.0795080	0.29204
melanocyte 337	1.0679153	0.29448
melanocyte 338	1.0686196	0.29652
fetal thymus 266	1.0986161	0.29732
fetal heart 45	1.0838743	0.29748
small intestine 342	1.0887573	0.29898
breast 2	1.0711295	0.30050
CD4 13	1.0917670	0.32408
fetal lung 301	1.0625916	0.32934
fetal lung 300	1.0617635	0.32992
fetal fibroblast 282	1.0563213	0.33068
fetal thymus 261	1.0790955	0.33368
fetal thymus 257	1.0813298	0.33540
fetal fibroblast 268	1.0541035	0.33772
breast 1	1.0553505	0.33782
fetal fibroblast 270	1.0561671	0.33812
fetal lung 298	1.0572163	0.34586
fetal adrenal gland 25	1.0629418	0.35198
fetal fibroblast 274	1.0463257	0.36114
pancreas 327	1.0608072	0.36318
fetal adrenal gland 23	1.0507731	0.36588
fetal fibroblast 279	1.0460820	0.36680
ES derived trophoblast 288	1.0494592	0.36844
fetal fibroblast 271	1.0432616	0.37008
fetal fibroblast 280	1.0436027	0.37212
fetal heart 49	1.0488376	0.37358
fetal heart 46	1.0489133	0.37732
fetal adrenal gland 24	1.0500290	0.38634
ES derived MSC 289	1.0410565	0.38850
fetal thymus 262	1.0447783	0.40280
fetal fibroblast 275	1.0301014	0.40440
fetal large intestine 64	1.0366353	0.40484
heart 297	1.0369194	0.40654
CD8 21	1.0440722	0.40662
ES derived MSC 290	1.0313498	0.41116
ES derived mesoderm 286	1.0268621	0.41278
CD3 10	1.0342576	0.41864
fibroblast 331	1.0272411	0.42040
CD56 18	1.0338322	0.42246
fetal heart 52	1.0265143	0.42388
fetal fibroblast 273	1.0232229	0.42438
fetal fibroblast 276	1.0231986	0.42504
fetal heart 42	1.0263077	0.42616
fetal fibroblast 272	1.0212107	0.42848
fetal small intestine 74	1.0249185	0.43166
CD14 3	1.0243409	0.43820
fibroblast 332	1.0197814	0.44174
fetal fibroblast 278	1.0168707	0.44560
fetal large intestine 57	1.0175090	0.44570
fetal small intestine 69	1.0173641	0.44748
fetal heart 43	1.0158612	0.44754
fetal small intestine 70	1.0161683	0.44914
fetal large intestine 58	1.0138778	0.45590
Mobilized CD56 324	1.0150269	0.45758
placenta 200	1.0098566	0.46478
stomach 284	1.0103698	0.46596
CD56 17	1.0086727	0.47154
fetal heart 47	1.0051062	0.47190
Mobilized CD4 323	1.0028830	0.47882
fetal large intestine 61	0.9989622	0.48294
fetal heart 50	1.0009865	0.48632
CD20 8	1.0000016	0.48886
CD8 19	0.9883532	0.49404
hematopoietic stem cell 310	1.0001906	0.49500
Mobilized CD3 320	0.9930696	0.50160
fetal small intestine 78	0.9949194	0.50188
fetal large intestine 67	0.9932958	0.50282
fetal skin 234	0.9944327	0.50402
fetal lung 299	0.9965806	0.50406
CD3 11	0.9913579	0.50420
fetal fibroblast 277	0.9954592	0.50666
fetal heart 48	0.9915240	0.50726
fetal fibroblast 281	0.9945889	0.50784
hematopoietic stem cell 315	0.9915421	0.51036
hematopoietic stem cell 309	0.9899698	0.51268
hematopoietic stem cell 312	0.9901503	0.51588
fetal heart 44	0.9839357	0.51800
fetal small intestine 77	0.9854078	0.51886
ES derived mesoderm 285	0.9893787	0.51950
fetal large intestine 60	0.9826302	0.52200
CD3 9	0.9791598	0.52384
Mobilized CD8 325	0.9697645	0.52834
CD19 6	0.9729924	0.53076
fetal large intestine 56	0.9808168	0.53272
fetal small intestine 76	0.9760500	0.53586
fetal adrenal gland 26	0.9786748	0.53768
fetal large intestine 65	0.9742773	0.53780
hematopoietic stem cell 305	0.9784684	0.53824
CD19 5	0.9728974	0.54048
fetal small intestine 68	0.9741065	0.54896
fetal small intestine 72	0.9709963	0.55044
fetal small intestine 79	0.9663529	0.55344
CD8 20	0.9672616	0.55432
hematopoietic stem cell 314	0.9747585	0.55648
ES derived trophoblast 287	0.9716667	0.55868
hematopoietic stem cell 316	0.9668297	0.56042
CD4 15	0.9426004	0.56052
fetal large intestine 62	0.9612594	0.56434
melanocyte 340	0.9767659	0.56470
CD19 7	0.9535512	0.56516
hematopoietic stem cell 306	0.9667072	0.56566
Mobilized CD3 321	0.9460719	0.57034
fetal heart 51	0.9639979	0.57662
fetal adrenal gland 27	0.9622541	0.57956
melanocyte 339	0.9709106	0.58048
fetal small intestine 73	0.9528801	0.58544
ES cells 294	0.9500387	0.58806
fetal muscle 176	0.9605773	0.59220
hematopoietic stem cell 318	0.9491912	0.59776
fetal small intestine 71	0.9469925	0.59930
fetal large intestine 59	0.9467337	0.59984
ES cells 296	0.9440063	0.60022
fetal brain 30	0.9689876	0.60254
CD8 22	0.9276635	0.60694
fetal kidney 83	0.9385325	0.60808
Mobilized CD4 322	0.9274685	0.60988
hematopoietic stem cell 313	0.9512322	0.61064
fetal small intestine 80	0.9479993	0.61092
fetal fibroblast 267	0.9594047	0.61270
placenta 198	0.9486968	0.61746
fetal large intestine 53	0.9323125	0.61856
fetal large intestine 55	0.9443507	0.61906
fetal stomach 253	0.9308056	0.62006
fetal brain 38	0.9534856	0.62270
hematopoietic stem cell 308	0.9464949	0.62392
fetal large intestine 54	0.9339941	0.62700
CD4 14	0.9321045	0.62802
fetal brain 41	0.9505008	0.62928
fetal brain 32	0.9534427	0.62934
CD3 12	0.9146996	0.62978
fetal renal pelvis 220	0.9346872	0.63068
hematopoietic stem cell 307	0.9373740	0.63734
fetal kidney 81	0.9424953	0.63904
fetal renal cortex 207	0.9337725	0.63916
iPS 304	0.9403145	0.64048
fetal large intestine 63	0.9255333	0.64104
hematopoietic stem cell 317	0.9443972	0.64166
hematopoietic stem cell 311	0.9329780	0.64504
fetal small intestine 75	0.8692452	0.64684
fetal renal pelvis 232	0.9263823	0.64746
fetal muscle 180	0.9329901	0.64886
fetal muscle 157	0.9397908	0.64916
fetal muscle 165	0.9372183	0.65244
fetal spinal cord 238	0.9365456	0.65474
fetal thymus 263	0.9310848	0.65544
fetal brain 39	0.9448970	0.65588
fetal spinal cord 236	0.9309713	0.65800
fetal muscle 175	0.9285643	0.66262
fetal muscle 153	0.9234407	0.66398
fetal stomach 244	0.9174789	0.66448
placenta 197	0.9174778	0.66550
fetal kidney 85	0.9296478	0.66868
iPS 302	0.9247063	0.67070
fetal large intestine 66	0.9103413	0.67120
fetal renal pelvis 233	0.9166971	0.67320
fetal spinal cord 235	0.9254255	0.67488
placenta 201	0.9099285	0.67718
ovary 326	0.8948050	0.67934
CD4 16	0.8802323	0.68262
fetal renal pelvis 225	0.9080260	0.68420
fetal muscle 143	0.9278586	0.68436
fetal brain 37	0.9317520	0.68454
fetal muscle 182	0.9212282	0.68516
fetal renal cortex 205	0.9126155	0.68666
fetal muscle 172	0.9286451	0.68744
fetal renal pelvis 231	0.9148279	0.68792
fetal muscle 167	0.9227184	0.68896
stomach 283	0.8980961	0.69332
fetal kidney 87	0.9105305	0.69364
fetal kidney 100	0.9064436	0.69692
fetal renal pelvis 227	0.9043624	0.69884
fetal stomach 242	0.8742900	0.70080
fetal renal cortex 213	0.9121918	0.70316
fetal kidney 89	0.9029002	0.70338
fetal kidney 82	0.8923281	0.70370
fetal spinal cord 237	0.9161677	0.70580
fetal kidney 103	0.8978521	0.70846
fetal muscle 174	0.9069088	0.70852
fetal muscle 191	0.9058065	0.70916
fetal muscle 151	0.9008498	0.71340
fetal renal pelvis 219	0.8784506	0.71446
fetal muscle 168	0.9015727	0.71476
fetal muscle 193	0.9002851	0.71674
fetal muscle 166	0.9085818	0.71760
fetal muscle 148	0.9007961	0.71812
fetal renal cortex 211	0.8873306	0.72052
fetal renal pelvis 228	0.8864315	0.72150
fetal brain 34	0.9233598	0.72158
hematopoietic stem cell 319	0.8907409	0.72266
fetal muscle 149	0.8991239	0.72288
fetal lung 118	0.9180761	0.72422
fetal kidney 92	0.8992164	0.72450
fetal muscle 163	0.8983368	0.72450
fetal muscle 183	0.9046377	0.72596
fetal brain 31	0.9290880	0.72618
fetal renal cortex 215	0.8965453	0.72822
fetal kidney 99	0.8916794	0.72900
fetal muscle 188	0.9003761	0.73116
ES cells 295	0.8762780	0.73280
ES cells 293	0.8812258	0.73324
fetal renal pelvis 218	0.8615084	0.73506
fetal muscle 184	0.9031761	0.73604
placenta 199	0.8908138	0.73610
fetal brain 35	0.9210211	0.73686
fetal kidney 88	0.8920947	0.73836
fetal muscle 169	0.8950917	0.73838
fetal ovary 195	0.8615509	0.73936
fetal kidney 95	0.8849574	0.73968
fetal kidney 106	0.8856562	0.74214
fetal kidney 86	0.8613040	0.74326
fetal muscle 173	0.8891102	0.74346
fetal brain 36	0.9074288	0.74390
fetal muscle 146	0.8939170	0.74512
iPS 303	0.8933779	0.74600
fetal renal pelvis 221	0.8904311	0.74608
fetal stomach 247	0.8495493	0.74736
fetal muscle 150	0.8848593	0.74798
fetal renal cortex 212	0.8878008	0.74802
fetal muscle 170	0.8959178	0.74838
fetal muscle 187	0.8959598	0.74916
fetal renal cortex 214	0.8834940	0.75128
fetal muscle 164	0.8858960	0.75254
fetal muscle 189	0.8852534	0.75506
ES derived NP 291	0.8651258	0.75650
fetal lung 119	0.9016628	0.75900
fetal kidney 91	0.8859919	0.76142
fetal lung 134	0.8894463	0.76192
fetal brain 40	0.9063327	0.76254
fetal muscle 155	0.8743538	0.76352
fetal brain 29	0.9163044	0.76702
fetal renal pelvis 217	0.8679894	0.76730
fetal kidney 105	0.8777553	0.76784
fetal muscle 162	0.8830686	0.77018
fetal muscle 178	0.8760094	0.77022
fetal muscle 179	0.8798901	0.77200
fetal renal cortex 206	0.8753408	0.77286
fetal stomach 252	0.8534898	0.77416
fetal stomach 240	0.8461863	0.77474
fetal renal pelvis 224	0.8762634	0.77504
placenta 196	0.8608907	0.77576
fetal muscle 192	0.8785590	0.77630
fetal renal pelvis 226	0.8723880	0.77744
fetal muscle 156	0.8742790	0.77930
fetal lung 132	0.8811440	0.78158
fetal spinal cord 239	0.8812394	0.78276
fetal kidney 84	0.8592740	0.78356
ES derived NP 292	0.8577366	0.78360
fetal renal pelvis 229	0.8641207	0.78456
fetal renal pelvis 223	0.8539176	0.78486
fetal muscle 152	0.8758111	0.78708
fetal muscle 171	0.8747429	0.78744
fetal muscle 159	0.8628615	0.78768
fetal lung 116	0.8929915	0.79018
fetal stomach 248	0.8415367	0.79056
fetal muscle 144	0.8601761	0.79208
fetal renal cortex 202	0.8622002	0.79280
fetal kidney 101	0.8611123	0.79432
fetal muscle 185	0.8597256	0.79612
fetal kidney 107	0.8539791	0.79996
fetal lung 140	0.8774621	0.80114
fetal brain 33	0.8803901	0.80174
fetal renal cortex 204	0.8642327	0.80260
fetal lung 114	0.8863420	0.80416
fetal muscle 154	0.8527201	0.80540
fetal kidney 98	0.8474648	0.80564
fetal kidney 96	0.8582092	0.80570
fetal muscle 160	0.8557065	0.80668
fetal lung 117	0.8760472	0.80776
fetal kidney 104	0.8471185	0.80818
fetal muscle 145	0.8412092	0.81034
fetal kidney 90	0.8473838	0.81062
fetal stomach 243	0.8478411	0.81158
fetal lung 128	0.8850055	0.81172
fetal renal pelvis 230	0.8323091	0.81252
fetal muscle 161	0.8523675	0.81508
fetal stomach 251	0.8309652	0.81698
fetal muscle 186	0.8567303	0.81706
fetal renal cortex 208	0.8411549	0.81744
fetal muscle 147	0.8485716	0.81824
fetal lung 121	0.8750556	0.81936
fetal muscle 190	0.8390530	0.81988
fetal kidney 97	0.8439870	0.82032
fetal muscle 194	0.8683102	0.82204
fetal renal cortex 210	0.8331209	0.82254
fetal muscle 158	0.8564652	0.82376
fetal lung 112	0.8588309	0.82692
fetal testes 254	0.8344588	0.82984
fetal stomach 241	0.8144161	0.83100
fetal lung 125	0.8535098	0.83142
fetal kidney 102	0.8259622	0.83160
fetal lung 137	0.8608590	0.83174
fetal lung 109	0.8735107	0.83264
fetal muscle 181	0.8477248	0.83326
fetal kidney 94	0.8265825	0.83348
fetal renal pelvis 216	0.8296947	0.83404
fetal lung 141	0.8696745	0.83514
fetal testes 255	0.8404040	0.83608
fetal renal cortex 203	0.8307710	0.83946
fetal lung 120	0.8522912	0.84228
fetal renal cortex 209	0.8262864	0.84628
fetal lung 127	0.8423951	0.84904
fetal stomach 249	0.7923441	0.84996
fetal lung 123	0.8421902	0.85286
fetal renal pelvis 222	0.8162896	0.85392
fetal stomach 250	0.7931752	0.85734
fetal lung 113	0.8508005	0.85916
fetal lung 108	0.8270835	0.85986
fetal stomach 246	0.7879772	0.86070
fetal kidney 93	0.8088363	0.86090
fetal muscle 177	0.8165022	0.86134
fetal lung 129	0.8429697	0.86182
fetal lung 110	0.8338182	0.86270
fetal lung 111	0.8488651	0.86636
fetal lung 130	0.8396800	0.86732
fetal lung 126	0.8491384	0.86748
fetal stomach 245	0.7942633	0.86826
fetal lung 131	0.8520839	0.87282
fetal lung 115	0.8338464	0.88422
fetal lung 133	0.8330837	0.89028
fetal lung 138	0.8193648	0.89096
fetal lung 136	0.8177038	0.89150
fetal lung 135	0.8130232	0.89556
fetal lung 142	0.8262119	0.89896
fetal lung 122	0.7967509	0.90458
fetal lung 124	0.7998755	0.91934
fetal lung 139	0.7836230	0.92464

Childhood obesity

DHS sample	fold enrichment	p value
hematopoietic stem cell 310	1.3373211	0.04164
hematopoietic stem cell 315	1.3793726	0.05588
hematopoietic stem cell 314	1.3354080	0.05712
hematopoietic stem cell 309	1.3508097	0.06174
fetal brain 36	1.2937670	0.06260
hematopoietic stem cell 312	1.3088398	0.08008
CD14 4	1.3474418	0.08414
hematopoietic stem cell 306	1.3093794	0.08428
hematopoietic stem cell 316	1.3271572	0.09418
CD56 17	1.3176078	0.10238
muscle 341	1.3124015	0.10274
CD14 3	1.3388951	0.10412
hematopoietic stem cell 308	1.2627719	0.10660
hematopoietic stem cell 313	1.2456474	0.11910
hematopoietic stem cell 311	1.2506851	0.12504
hematopoietic stem cell 317	1.2128803	0.13046
fetal brain 37	1.2068928	0.13308
hematopoietic stem cell 307	1.2443881	0.13476
hematopoietic stem cell 318	1.2552171	0.13794
CD8 20	1.2726548	0.14856
fetal heart 45	1.1963606	0.16712
hematopoietic stem cell 319	1.2257687	0.16974
fetal heart 47	1.2087263	0.17046
fetal fibroblast 279	1.1674325	0.17316
fetal fibroblast 269	1.1629260	0.17376
hematopoietic stem cell 305	1.2074691	0.17510
iPS 302	1.1940333	0.17860
fetal renal pelvis 222	1.2015135	0.18260
fetal lung 300	1.1688414	0.18310
fetal heart 43	1.1724329	0.18660
fetal renal cortex 209	1.1956896	0.18694
iPS 304	1.1775474	0.18954
fetal brain 33	1.1627173	0.19420
fetal heart 50	1.1783411	0.19434
fetal heart 51	1.1764441	0.19434
fetal fibroblast 274	1.1548526	0.19506
fetal fibroblast 280	1.1482396	0.19610
fetal fibroblast 281	1.1514802	0.19680
fetal heart 42	1.1757412	0.19700
fetal kidney 82	1.1993415	0.20084
fetal fibroblast 275	1.1423861	0.20474
CD19 7	1.2335271	0.20762
fetal fibroblast 278	1.1451069	0.20798
fetal spinal cord 239	1.1561812	0.20906
fetal fibroblast 272	1.1381613	0.21582
fetal muscle 169	1.1603625	0.21752
CD4 14	1.1961306	0.21790
fetal brain 32	1.1336784	0.21982
fetal brain 41	1.1335142	0.22016
ES cells 294	1.1898299	0.22252
ES derived MSC 290	1.1507813	0.22280
CD56 18	1.2113048	0.22450
fetal fibroblast 267	1.1372713	0.22550
fetal spinal cord 235	1.1459929	0.22564
fetal muscle 172	1.1337715	0.22628
fetal renal pelvis 233	1.1580318	0.22692
fetal fibroblast 270	1.1330611	0.22706
CD4 16	1.2114609	0.22776
ES derived mesoderm 286	1.1231827	0.22792
iPS 303	1.1504570	0.22836
fetal brain 34	1.1194701	0.22940
fetal spinal cord 238	1.1435723	0.23038
fetal renal pelvis 217	1.1591488	0.23042
fetal fibroblast 271	1.1308507	0.23124
fetal renal pelvis 218	1.1880915	0.23192
Mobilized CD4 323	1.1968122	0.23594
fetal heart 48	1.1459711	0.23598
fetal muscle 178	1.1437866	0.23784
ovary 326	1.1701694	0.24178
fetal kidney 88	1.1410273	0.24274
fetal muscle 151	1.1486054	0.24288
ES derived NP 291	1.1693563	0.24336
CD3 11	1.1696616	0.24494
CD19 6	1.1892752	0.24496
ES cells 295	1.1662749	0.24516
fetal spinal cord 237	1.1318484	0.24684
ES derived NP 292	1.1569600	0.24726
fetal renal pelvis 225	1.1432844	0.24908
fetal muscle 179	1.1314310	0.24932
fetal renal cortex 210	1.1433327	0.25088
fetal renal pelvis 232	1.1430588	0.25222
fetal small intestine 80	1.1412667	0.25280
fetal kidney 90	1.1378334	0.25316
ES derived mesoderm 285	1.1156643	0.25552
ES derived MSC 289	1.1269871	0.25622
keratinocyte 336	1.1063046	0.25664
fetal heart 46	1.1336641	0.26164
fetal renal pelvis 228	1.1378379	0.26200
fetal spinal cord 236	1.1272892	0.26212
fetal renal pelvis 227	1.1254160	0.26402
fetal renal cortex 211	1.1352948	0.26466
fetal fibroblast 273	1.1091269	0.26480
fetal brain 40	1.1015719	0.26516
CD19 5	1.1649763	0.26660
breast 1	1.1060416	0.26762
fetal kidney 95	1.1240892	0.26826
CD8 22	1.1601681	0.26906
fetal kidney 104	1.1274962	0.26920
fetal fibroblast 277	1.1090727	0.26958
melanocyte 339	1.1008015	0.26996
fetal kidney 102	1.1289674	0.27154
ES cells 296	1.1417974	0.27184
fetal fibroblast 276	1.1016377	0.27358
fetal renal pelvis 231	1.1173896	0.27372
fetal kidney 87	1.1201574	0.27380
fetal thymus 260	1.1472439	0.27394
ES cells 293	1.1335044	0.27460
fetal renal pelvis 216	1.1222219	0.27528
fetal small intestine 79	1.1356179	0.27578
fetal muscle 192	1.1132131	0.27634
CD3 12	1.1578037	0.27786
CD3 10	1.1477467	0.27826
fetal muscle 182	1.1130355	0.27832
fetal heart 49	1.1164038	0.27862
fetal heart 44	1.1367307	0.28138
fetal fibroblast 282	1.0959192	0.28256
fetal large intestine 55	1.1183542	0.28374
fetal kidney 93	1.1210885	0.28458
fetal renal cortex 208	1.1180267	0.28472
fetal fibroblast 268	1.0965135	0.28522
fetal renal cortex 202	1.1118709	0.28534
fetal kidney 101	1.1130090	0.28588
fetal small intestine 69	1.1279349	0.28668
fetal kidney 96	1.1087070	0.28798
fetal large intestine 66	1.1223141	0.28832
CD8 19	1.1571139	0.28848
fetal kidney 92	1.1072006	0.28922
keratinocyte 333	1.0883792	0.29052
fetal muscle 174	1.1056200	0.29188
fetal large intestine 53	1.1304238	0.29208
fetal muscle 173	1.1068416	0.29208
fetal muscle 149	1.1108937	0.29282
fetal renal pelvis 221	1.1041845	0.29298
fetal kidney 105	1.1051635	0.29408
fetal kidney 86	1.1266888	0.29428
fetal thymus 265	1.1246252	0.29564
fetal brain 39	1.0874777	0.29740
fetal renal pelvis 224	1.1017902	0.29788
Mobilized CD8 325	1.1320165	0.30150
CD3 9	1.1298847	0.30284
melanocyte 337	1.0808545	0.30322
fetal renal cortex 205	1.1003551	0.30470
heart 297	1.1103716	0.30530
breast 2	1.0866781	0.30640
fetal small intestine 76	1.1122461	0.30708
fetal renal pelvis 223	1.1074763	0.30712
fetal thymus 258	1.1175167	0.30788
fetal large intestine 59	1.1086302	0.30936
fetal kidney 89	1.0979991	0.31104
fetal kidney 97	1.0935327	0.31340
fetal muscle 191	1.0957647	0.31464
CD4 13	1.1245126	0.31502
fetal lung 298	1.0909197	0.31552
fetal kidney 107	1.0926050	0.31658
fetal renal cortex 213	1.0881207	0.31900
fetal large intestine 65	1.0995909	0.32080
fetal muscle 181	1.0869236	0.32088
pancreas 327	1.1056639	0.32128
fetal lung 299	1.0884222	0.32136
fetal renal cortex 206	1.0872300	0.32200
fetal muscle 175	1.0872690	0.32210
fetal muscle 176	1.0852879	0.32306
fetal large intestine 62	1.0983270	0.32486
fetal renal cortex 212	1.0838558	0.32522
fetal muscle 168	1.0890131	0.32612
fetal large intestine 56	1.0943988	0.32710
fetal heart 52	1.0810813	0.32874
fetal muscle 167	1.0832945	0.32908
Mobilized CD3 321	1.1113701	0.32916
fetal muscle 161	1.0831018	0.32958
fetal large intestine 60	1.0923598	0.33134
fetal brain 38	1.0730499	0.33216
fetal renal cortex 204	1.0780501	0.33240
fetal renal cortex 214	1.0826883	0.33308
keratinocyte 334	1.0672463	0.33576
fetal kidney 94	1.0834683	0.33628
fetal muscle 163	1.0789219	0.33780
melanocyte 338	1.0644942	0.33806
fetal small intestine 75	1.0988932	0.33818
fetal large intestine 67	1.0882025	0.33864
fetal renal cortex 215	1.0761285	0.33900
fetal renal pelvis 230	1.0831216	0.33930
fetal small intestine 71	1.0827049	0.33984
fetal renal pelvis 226	1.0770020	0.34054
fetal lung 301	1.0745887	0.34176
fetal stomach 253	1.0892645	0.34252
fetal muscle 150	1.0795570	0.34386
fetal muscle 183	1.0726160	0.34402
fetal renal pelvis 220	1.0753170	0.34656
fetal kidney 99	1.0732160	0.34686
fetal large intestine 64	1.0783716	0.34832
fetal kidney 98	1.0740477	0.34852
Mobilized CD4 322	1.0947166	0.35032
melanocyte 340	1.0652335	0.35270
fetal testes 254	1.0722656	0.35380
CD8 21	1.0933725	0.35480
fetal muscle 157	1.0648409	0.35560
fetal kidney 81	1.0651547	0.35686
fetal kidney 83	1.0705235	0.35740
fibroblast 332	1.0612711	0.35768
fetal small intestine 77	1.0756411	0.35802
fetal kidney 106	1.0628806	0.35950
CD20 8	1.0698662	0.35954
Mobilized CD3 320	1.0914871	0.36092
fetal large intestine 63	1.0720993	0.36274
fetal renal pelvis 229	1.0609949	0.36436
fetal large intestine 61	1.0755177	0.36598
fetal brain 35	1.0481260	0.36642
fetal muscle 166	1.0626256	0.36660
fetal muscle 193	1.0633278	0.36704
fetal muscle 155	1.0663329	0.36738
fetal renal pelvis 219	1.0650614	0.37226
fetal renal cortex 207	1.0597309	0.37426
fetal small intestine 73	1.0623514	0.37518
fetal thymus 261	1.0677688	0.37532
fetal muscle 171	1.0555900	0.37534
pancreas 328	1.0644467	0.37576
fetal renal cortex 203	1.0553145	0.37586
fetal muscle 187	1.0545199	0.37768
fetal muscle 147	1.0555751	0.38118
fetal muscle 165	1.0523805	0.38208
fetal muscle 186	1.0529865	0.38296
fetal kidney 85	1.0449298	0.38522
fetal muscle 188	1.0512745	0.38542
fetal small intestine 72	1.0498219	0.39152
placenta 201	1.0487615	0.39290
fibroblast 331	1.0440750	0.39522
fetal kidney 100	1.0427551	0.39540
fetal adrenal gland 24	1.0515809	0.39618
fetal lung 110	1.0427458	0.39642
fetal lung 135	1.0426667	0.39642
fetal adrenal gland 23	1.0428483	0.39650
fetal muscle 180	1.0499386	0.39678
fetal thymus 264	1.0489761	0.39872
fetal lung 123	1.0400039	0.39970
fetal thymus 256	1.0474998	0.40040
fetal muscle 177	1.0451297	0.40068
Mobilized CD56 324	1.0512358	0.40178
fetal muscle 190	1.0410780	0.40544
fetal skin 234	1.0443812	0.40592
fetal muscle 148	1.0425357	0.40718
fetal lung 138	1.0373443	0.40794
fetal muscle 144	1.0405438	0.40804
fetal muscle 162	1.0396360	0.40910
fetal thymus 266	1.0439017	0.40966
fetal muscle 184	1.0368281	0.41296
fetal large intestine 57	1.0382813	0.41310
small intestine 342	1.0396904	0.41394
fetal kidney 103	1.0333436	0.41394
fetal muscle 153	1.0392047	0.41420
fetal muscle 160	1.0360413	0.41638
fetal thymus 259	1.0376971	0.41724
fetal lung 129	1.0326420	0.41946
fetal muscle 154	1.0338268	0.42370
fetal muscle 158	1.0312210	0.42442
fetal muscle 170	1.0296701	0.42698
fetal adrenal gland 26	1.0284146	0.42856
fetal lung 124	1.0262629	0.42884
keratinocyte 335	1.0257722	0.43106
fetal small intestine 70	1.0269026	0.43132
fetal adrenal gland 25	1.0298902	0.43134
fetal ovary 195	1.0224307	0.43312
CD4 15	1.0171403	0.43456
fetal lung 113	1.0222119	0.43464
fetal stomach 242	1.0220452	0.43498
fetal muscle 194	1.0230922	0.43614
fetal small intestine 74	1.0241846	0.43770
fetal brain 31	1.0168465	0.44154
fetal thymus 263	1.0239198	0.44312
fetal muscle 145	1.0191777	0.44370
fibroblast 330	1.0210682	0.44376
fetal stomach 245	1.0162218	0.44434
fetal large intestine 54	1.0158526	0.44472
fetal kidney 84	1.0167618	0.44546
fetal muscle 189	1.0192958	0.44560
fetal muscle 143	1.0198061	0.44808
fetal lung 108	1.0162354	0.44876
fetal lung 115	1.0163234	0.45024
fetal lung 119	1.0190232	0.45080
fetal lung 125	1.0138133	0.45252
fetal lung 131	1.0142048	0.45430
fetal thymus 257	1.0064537	0.45590
fetal muscle 164	1.0148910	0.45712
fetal small intestine 68	1.0140253	0.45746
fetal lung 136	1.0098481	0.45830
fibroblast 329	1.0127340	0.46108
fetal lung 132	1.0099600	0.46520
fetal lung 142	1.0061022	0.46852
fetal muscle 146	1.0084589	0.47096
fetal stomach 248	1.0006812	0.47278
fetal muscle 152	1.0045674	0.47676
fetal large intestine 58	1.0043290	0.47698
fetal adrenal gland 28	1.0040759	0.47956
fetal stomach 249	0.9884294	0.48302
fetal stomach 252	0.9897311	0.48310
fetal small intestine 78	1.0011048	0.48378
fetal lung 127	0.9974024	0.48500
fetal stomach 251	0.9880251	0.49348
fetal stomach 240	0.9823388	0.49376
fetal muscle 185	0.9940877	0.49540
fetal stomach 244	0.9884679	0.49608
fetal lung 126	0.9957007	0.49778
fetal lung 137	0.9913870	0.50424
fetal stomach 247	0.9677710	0.50438
placenta 196	0.9814687	0.51000
fetal lung 116	0.9922605	0.51020
fetal stomach 246	0.9710149	0.51166
placenta 200	0.9868305	0.51574
fetal thymus 262	0.9468158	0.51646
fetal lung 112	0.9800753	0.52150
fetal brain 30	0.9872700	0.52566
fetal lung 109	0.9821538	0.52572
fetal stomach 243	0.9701604	0.52830
fetal lung 128	0.9828847	0.52920
fetal muscle 156	0.9778467	0.53034
fetal muscle 159	0.9747317	0.53272
ES derived trophoblast 288	0.9804036	0.53412
stomach 283	0.9646747	0.53640
fetal lung 117	0.9785061	0.53736
fetal lung 122	0.9680424	0.53808
fetal lung 139	0.9704434	0.53990
fetal lung 118	0.9804749	0.54046
fetal lung 121	0.9747274	0.54570
fetal lung 120	0.9728932	0.54628
fetal adrenal gland 27	0.9616315	0.55726
fetal kidney 91	0.9581614	0.56118
fetal stomach 250	0.9374095	0.56368
placenta 197	0.9508302	0.56630
fetal testes 255	0.9579568	0.56782
fetal stomach 241	0.9342864	0.56938
fetal brain 29	0.9691273	0.57186
placenta 199	0.9526231	0.57332
fetal lung 111	0.9588856	0.57784
placenta 198	0.9533358	0.58174
fetal lung 114	0.9593396	0.58416
fetal lung 141	0.9525315	0.59932
fetal lung 130	0.9344445	0.62394
fetal lung 134	0.9256624	0.63240
fetal lung 133	0.9276236	0.64954
stomach 284	0.8961551	0.65874
ES derived trophoblast 287	0.9097425	0.65994
fetal lung 140	0.9228536	0.66294

Bone mineral density 1

DHS sample	fold enrichment	p value
melanocyte 337	1.2565841	0.05250
melanocyte 338	1.2283104	0.07468
ES derived MSC 290	1.2681220	0.09484
ES derived MSC 289	1.2366225	0.12072
iPS 304	1.2093741	0.13962
melanocyte 340	1.1814819	0.14578
melanocyte 339	1.1642374	0.15658
fetal adrenal gland 28	1.1791581	0.16912
iPS 303	1.1796253	0.18342
ES derived mesoderm 286	1.1456236	0.18396
fetal adrenal gland 26	1.1720136	0.20748
iPS 302	1.1421582	0.23560
fetal large intestine 57	1.1416430	0.24004
fetal adrenal gland 25	1.1400437	0.25870
fetal adrenal gland 27	1.1313383	0.26062
ES cells 296	1.1342154	0.27084
fetal adrenal gland 23	1.1104413	0.27886
Mobilized CD56 324	1.1455163	0.28218
ES cells 295	1.1155998	0.29276
CD56 18	1.1412992	0.29828
fetal fibroblast 269	1.0839989	0.29888
small intestine 342	1.1119407	0.29898
ES cells 293	1.0975265	0.31090
fetal renal pelvis 230	1.0892126	0.32924
fetal small intestine 78	1.0775362	0.33886
ES derived mesoderm 285	1.0635660	0.34048
ES cells 294	1.0808201	0.34362
fetal heart 49	1.0716747	0.34492
fetal adrenal gland 24	1.0823033	0.34660
fetal renal cortex 213	1.0640075	0.35620
fetal muscle 159	1.0656590	0.36318
fetal stomach 250	1.0695940	0.36520
fetal heart 48	1.0581388	0.36946
fetal fibroblast 270	1.0498991	0.37354
fetal kidney 101	1.0552474	0.37418
fetal kidney 93	1.0588265	0.37432
fetal stomach 240	1.0631087	0.37546
fetal heart 47	1.0540061	0.38100
fetal stomach 244	1.0575210	0.38208
fetal large intestine 60	1.0587007	0.38276
fetal small intestine 69	1.0571324	0.38506
heart 297	1.0564186	0.38570
fetal stomach 246	1.0549010	0.38620
fetal heart 42	1.0498081	0.38818
breast 1	1.0420748	0.39582
fetal heart 45	1.0437152	0.39700
ES derived NP 292	1.0408673	0.40564
fetal large intestine 64	1.0432708	0.40582
fetal muscle 160	1.0399074	0.40656
fetal muscle 181	1.0388761	0.40690
fetal muscle 173	1.0355155	0.40856
fetal small intestine 70	1.0389633	0.40938
fetal stomach 251	1.0389601	0.41162
fetal heart 43	1.0332135	0.41258
fetal kidney 100	1.0357324	0.41276
fetal stomach 241	1.0371195	0.41688
fetal renal cortex 203	1.0312503	0.41762
fetal large intestine 67	1.0381517	0.41890
fetal muscle 150	1.0320078	0.41896
fetal muscle 155	1.0312917	0.42032
fetal renal cortex 208	1.0301263	0.42150
fetal heart 46	1.0313169	0.42172
fetal lung 126	1.0282040	0.42184
fetal renal cortex 206	1.0280687	0.42296
fetal renal cortex 205	1.0294597	0.42338
fetal small intestine 74	1.0325087	0.42358
fetal muscle 191	1.0285258	0.42618
fetal lung 128	1.0244739	0.42722
fetal kidney 107	1.0264998	0.42790
fetal large intestine 55	1.0308882	0.42808
stomach 284	1.0313610	0.42868
fetal heart 51	1.0256596	0.42876
fetal lung 301	1.0287917	0.42936
fetal muscle 145	1.0264522	0.42952
fetal renal pelvis 226	1.0234262	0.43238
fetal stomach 252	1.0256650	0.43288
fetal renal cortex 202	1.0240932	0.43406
fetal kidney 98	1.0227781	0.43536
fetal stomach 248	1.0244263	0.43568
fetal muscle 165	1.0214684	0.43596
fetal muscle 174	1.0233078	0.43654
fetal lung 116	1.0211843	0.43682
fetal fibroblast 279	1.0230188	0.43686
fetal thymus 263	1.0219932	0.43790
fetal lung 113	1.0189665	0.43812
fetal lung 114	1.0196545	0.43972
breast 2	1.0220711	0.44014
fibroblast 329	1.0213620	0.44144
fetal stomach 249	1.0178606	0.44166
fetal kidney 85	1.0159719	0.44492
fetal kidney 92	1.0163585	0.44502
fetal renal pelvis 217	1.0170596	0.44572
fetal muscle 154	1.0201453	0.44586
fetal large intestine 58	1.0204947	0.44622
fetal lung 298	1.0201369	0.44764
fibroblast 330	1.0169351	0.44966
fetal small intestine 71	1.0163188	0.45122
fetal lung 300	1.0167703	0.45144
fetal stomach 243	1.0154384	0.45384
fetal muscle 182	1.0132799	0.45422
fetal muscle 192	1.0130167	0.45502
fetal large intestine 65	1.0136697	0.45596
fibroblast 331	1.0127805	0.46112
fetal lung 115	1.0110073	0.46198
fetal muscle 172	1.0100410	0.46228
fetal renal cortex 209	1.0082630	0.46282
hematopoietic stem cell 308	1.0093122	0.46328
fetal muscle 189	1.0094720	0.46378
fetal fibroblast 282	1.0105776	0.46462
fetal lung 137	1.0086397	0.46478
fetal kidney 96	1.0063235	0.46560
fetal muscle 149	1.0093224	0.46582
fetal kidney 88	1.0060683	0.46614
fetal renal cortex 212	1.0052157	0.46690
placenta 199	1.0083416	0.46816
fetal lung 109	1.0049358	0.47308
fetal muscle 151	1.0037480	0.47354
fetal muscle 143	1.0056258	0.47376
fetal renal pelvis 227	0.9994096	0.47416
stomach 283	1.0040667	0.47440
fetal fibroblast 280	1.0068413	0.47468
fetal stomach 253	0.9994519	0.47660
fetal small intestine 72	1.0020633	0.47732
fetal muscle 193	1.0003820	0.47786
fetal kidney 84	0.9980242	0.47800
fetal renal pelvis 216	0.9976779	0.47828
fetal stomach 247	0.9959929	0.47856
fetal muscle 144	1.0002436	0.47866
fetal kidney 104	0.9974454	0.47972
fetal lung 127	1.0010714	0.48074
fetal renal pelvis 223	0.9971047	0.48094
fetal renal cortex 210	0.9974919	0.48172
fetal renal pelvis 232	0.9965753	0.48288
fetal kidney 90	0.9972537	0.48582
fetal lung 140	1.0009461	0.48586
fibroblast 332	1.0013181	0.48592
fetal testes 254	0.9957196	0.48794
fetal muscle 158	0.9987037	0.48818
fetal muscle 187	0.9966122	0.49026
fetal renal pelvis 225	0.9911786	0.49336
fetal stomach 245	0.9898649	0.49382
fetal large intestine 56	0.9925668	0.49392
fetal muscle 185	0.9925472	0.49466
fetal renal pelvis 224	0.9920112	0.49494
fetal stomach 242	0.9803986	0.49528
fetal kidney 81	0.9928465	0.49596
fetal kidney 97	0.9889342	0.49716
fetal muscle 177	0.9893909	0.49736
fetal heart 52	0.9919286	0.49848
fetal renal cortex 207	0.9889116	0.49986
fetal small intestine 80	0.9921291	0.49988
CD20 8	0.9928306	0.50002
fetal fibroblast 281	0.9950945	0.50082
fetal muscle 167	0.9922226	0.50112
fetal renal cortex 211	0.9842361	0.50248
placenta 196	0.9864253	0.50356
fetal brain 39	0.9919620	0.50412
fetal kidney 91	0.9885902	0.50586
fetal muscle 169	0.9876885	0.50750
fetal large intestine 63	0.9839657	0.50886
fetal kidney 82	0.9806730	0.50908
fetal muscle 175	0.9868146	0.50912
fetal renal pelvis 222	0.9822723	0.51034
fetal muscle 148	0.9843635	0.51142
fetal small intestine 77	0.9829572	0.51206
fetal lung 130	0.9869096	0.51238
fetal muscle 163	0.9824583	0.51258
fetal renal cortex 214	0.9805551	0.51342
fetal testes 255	0.9834097	0.51432
fetal renal pelvis 229	0.9807915	0.51452
fetal kidney 89	0.9787572	0.51640
fetal lung 134	0.9830045	0.51922
fetal muscle 180	0.9809535	0.51938
fetal brain 29	0.9895309	0.51980
fetal large intestine 62	0.9746113	0.52070
fetal muscle 168	0.9794484	0.52086
fetal renal cortex 204	0.9813706	0.52090
fetal heart 44	0.9718898	0.52372
fetal renal cortex 215	0.9783501	0.52390
hematopoietic stem cell 314	0.9821700	0.52402
fetal muscle 190	0.9715624	0.52550
fetal large intestine 59	0.9720452	0.52636
fetal small intestine 68	0.9769762	0.52656
fetal fibroblast 275	0.9843274	0.52796
fetal muscle 188	0.9761786	0.52856
fetal muscle 162	0.9785696	0.52888
fetal renal pelvis 220	0.9726799	0.52938
fetal heart 50	0.9735233	0.52944
fetal kidney 102	0.9687720	0.53038
fetal lung 132	0.9783914	0.53088
fetal lung 142	0.9798797	0.53174
fetal kidney 87	0.9724862	0.53348
fetal large intestine 66	0.9672899	0.53426
keratinocyte 335	0.9842325	0.53434
ES derived NP 291	0.9661359	0.53476
fetal large intestine 53	0.9624035	0.53628
fetal kidney 99	0.9707591	0.53686
fetal renal pelvis 221	0.9720603	0.53694
fetal kidney 105	0.9710665	0.53832
fetal kidney 83	0.9636218	0.53874
fetal muscle 157	0.9747972	0.53964
fetal fibroblast 276	0.9796724	0.53988
fetal small intestine 76	0.9655620	0.54072
fetal lung 120	0.9743981	0.54096
fetal kidney 94	0.9643231	0.54166
fetal muscle 184	0.9727520	0.54226
fetal muscle 166	0.9701689	0.54264
fetal ovary 195	0.9506448	0.54274
fetal lung 129	0.9740930	0.54378
fetal lung 136	0.9723041	0.54398
fetal muscle 178	0.9667313	0.54400
hematopoietic stem cell 310	0.9743523	0.54530
fetal lung 124	0.9721028	0.54680
fetal small intestine 79	0.9600005	0.54900
fetal lung 135	0.9697220	0.54914
fetal small intestine 73	0.9616493	0.54990
fetal muscle 164	0.9655583	0.54996
fetal renal pelvis 219	0.9506790	0.55000
fetal spinal cord 236	0.9663949	0.55126
fetal lung 108	0.9669838	0.55208
fetal brain 30	0.9815831	0.55330
fetal muscle 152	0.9662660	0.55442
fetal lung 121	0.9704379	0.55912
fetal brain 35	0.9769047	0.55950
fetal muscle 171	0.9628520	0.55992
fetal lung 118	0.9718841	0.56060
fetal lung 122	0.9611982	0.56062
fetal muscle 194	0.9673095	0.56100
ES derived trophoblast 288	0.9680634	0.56138
keratinocyte 334	0.9747147	0.56238
fetal renal pelvis 218	0.9385494	0.56556
fetal fibroblast 277	0.9658132	0.56690
pancreas 327	0.9423543	0.56746
fetal kidney 103	0.9511288	0.56886
fetal fibroblast 274	0.9666488	0.57050
fetal lung 112	0.9616730	0.57058
hematopoietic stem cell 307	0.9529414	0.57112
hematopoietic stem cell 305	0.9527523	0.57298
fetal fibroblast 273	0.9649961	0.57568
hematopoietic stem cell 319	0.9461206	0.57752
fetal fibroblast 278	0.9617966	0.57836
fetal thymus 256	0.9389096	0.57898
fetal muscle 179	0.9524956	0.57922
fetal lung 123	0.9537344	0.58088
fetal large intestine 54	0.9414333	0.58202
pancreas 328	0.9387375	0.58484
fetal brain 41	0.9609473	0.58524
fetal kidney 106	0.9442970	0.58592
fetal renal pelvis 231	0.9456163	0.58698
fetal thymus 258	0.9294111	0.58838
fetal muscle 147	0.9441205	0.58854
fetal muscle 146	0.9469144	0.59010
fetal lung 117	0.9566657	0.59018
ovary 326	0.9226755	0.59116
fetal renal pelvis 233	0.9403966	0.59132
CD8 22	0.9113177	0.59134
fetal muscle 153	0.9415298	0.59228
fetal fibroblast 272	0.9582796	0.59230
fetal lung 138	0.9485898	0.59392
fetal lung 110	0.9483016	0.59398
fetal small intestine 75	0.8745303	0.59662
fetal lung 131	0.9544552	0.59894
fetal lung 119	0.9555398	0.60058
fetal muscle 170	0.9425051	0.60100
fetal lung 139	0.9439917	0.60172
fetal spinal cord 238	0.9433067	0.60714
placenta 200	0.9436240	0.60744
fetal lung 299	0.9442813	0.60926
fetal kidney 95	0.9320163	0.61046
CD56 17	0.9214393	0.61084
fetal spinal cord 239	0.9407367	0.61198
hematopoietic stem cell 311	0.9331405	0.61348
fetal renal pelvis 228	0.9220583	0.61618
fetal muscle 183	0.9378849	0.61624
fetal spinal cord 235	0.9378032	0.61666
fetal lung 111	0.9445318	0.61690
fetal muscle 161	0.9304303	0.61694
fetal lung 125	0.9362648	0.62100
keratinocyte 336	0.9509940	0.62140
fetal muscle 156	0.9320810	0.62140
placenta 198	0.9322624	0.62506
hematopoietic stem cell 316	0.9206953	0.62604
fetal fibroblast 268	0.9432283	0.62674
fetal muscle 186	0.9270361	0.62842
fetal kidney 86	0.9043907	0.62888
hematopoietic stem cell 315	0.9232629	0.62996
fetal brain 37	0.9458150	0.63432
CD3 9	0.8853683	0.63464
hematopoietic stem cell 306	0.9193044	0.63508
fetal spinal cord 237	0.9335806	0.63560
CD8 19	0.8653408	0.63748
fetal fibroblast 267	0.9347821	0.63760
fetal lung 133	0.9366901	0.63858
placenta 197	0.9135220	0.63942
fetal lung 141	0.9375634	0.63944
Mobilized CD3 321	0.8653146	0.64080
CD8 20	0.8915028	0.64102
fetal large intestine 61	0.8829905	0.64152
CD8 21	0.8708711	0.64532
fetal muscle 176	0.9242182	0.64968
ES derived trophoblast 287	0.9211618	0.65224
Mobilized CD4 323	0.8696094	0.65384
fetal skin 234	0.9169608	0.65548
hematopoietic stem cell 309	0.9115775	0.65694
hematopoietic stem cell 318	0.9026512	0.65810
keratinocyte 333	0.9369333	0.66120
fetal brain 33	0.9226315	0.66516
CD3 12	0.8553628	0.66560
muscle 341	0.8878401	0.66678
fetal thymus 259	0.8705404	0.66870
fetal brain 38	0.9192018	0.68288
fetal thymus 264	0.8712392	0.68328
fetal thymus 257	0.8615152	0.68378
fetal thymus 265	0.8681566	0.68950
fetal thymus 261	0.8634162	0.69050
fetal fibroblast 271	0.9149238	0.69110
CD3 11	0.8574707	0.69170
CD4 16	0.8308955	0.69210
Mobilized CD4 322	0.8352817	0.69376
CD4 15	0.8038129	0.69422
fetal brain 32	0.9141369	0.69540
fetal thymus 266	0.8614210	0.69640
CD19 7	0.8301825	0.70332
hematopoietic stem cell 313	0.8859054	0.71106
CD19 6	0.8270866	0.71150
fetal brain 31	0.9274998	0.71268
fetal thymus 260	0.8409084	0.72218
fetal brain 40	0.9108076	0.72770
placenta 201	0.8519847	0.73336
CD4 13	0.8101701	0.73350
hematopoietic stem cell 312	0.8714858	0.73716
CD19 5	0.8182850	0.73782
fetal brain 34	0.9069287	0.73860
Mobilized CD8 325	0.7877623	0.75264
fetal thymus 262	0.7828545	0.75980
fetal brain 36	0.8866252	0.76888
CD14 3	0.7948108	0.77276
hematopoietic stem cell 317	0.8611569	0.77424
CD4 14	0.7922495	0.78362
CD14 4	0.7931229	0.79980
CD3 10	0.7502168	0.82438
Mobilized CD3 320	0.6839233	0.86396

Five major psychiatric disorders

DHS sample	fold enrichment	p value
fetal heart 45	1.4419042	0.05326
fetal heart 47	1.4171578	0.07720
fetal heart 49	1.3673160	0.09150
fetal heart 46	1.3096508	0.14202
fetal heart 48	1.2691918	0.15580
fetal heart 43	1.2408317	0.16944
fetal heart 42	1.2044752	0.22226
fetal heart 52	1.1594122	0.25502
fetal brain 30	1.0778509	0.29280
fetal heart 51	1.1279203	0.30154
fetal heart 50	1.1119005	0.32666
fetal lung 298	1.0714232	0.37494
fetal lung 300	1.0556938	0.39632
fetal fibroblast 273	1.0374439	0.41692
fibroblast 331	1.0355619	0.43046
fetal fibroblast 270	1.0255705	0.43970
fetal fibroblast 271	1.0238794	0.44642
fetal lung 301	1.0197186	0.45858
fetal fibroblast 274	1.0149860	0.46152
fetal fibroblast 269	1.0146430	0.46708
fetal fibroblast 279	1.0072459	0.47366
fibroblast 332	1.0054031	0.47918
fetal fibroblast 275	1.0052478	0.48052
fibroblast 329	1.0027612	0.48244
fetal fibroblast 277	1.0005532	0.48620
fetal fibroblast 272	1.0001009	0.49110
fetal adrenal gland 28	0.9950627	0.49348
fetal brain 29	0.9948472	0.49492
fetal adrenal gland 23	0.9901780	0.50028
fetal adrenal gland 25	0.9814460	0.51010
fetal adrenal gland 26	0.9779192	0.51398
fetal fibroblast 280	0.9855689	0.51854
fetal fibroblast 276	0.9842996	0.52476
fetal heart 44	0.9566404	0.52528
fetal fibroblast 282	0.9757487	0.53808
fibroblast 330	0.9530799	0.56744
heart 297	0.9228117	0.57954
fetal lung 299	0.9416566	0.58576
fetal fibroblast 281	0.9477380	0.59270
fetal brain 35	0.9417319	0.61482
fetal fibroblast 268	0.9381832	0.61586
fetal brain 40	0.9291528	0.62676
fetal fibroblast 278	0.9215395	0.63360
ES derived MSC 289	0.8999435	0.64086
fetal brain 36	0.9078458	0.66190
ES derived MSC 290	0.8816973	0.66630
fetal brain 37	0.9091932	0.67082
fetal adrenal gland 24	0.8453220	0.68220
fetal brain 31	0.9196794	0.68312
fetal adrenal gland 27	0.8279544	0.72592
fetal spinal cord 235	0.8441078	0.72654
hematopoietic stem cell 310	0.8370324	0.72750
fetal fibroblast 267	0.8658386	0.73700
fetal lung 133	0.8440459	0.74656
fetal brain 39	0.8692432	0.75358
fetal brain 34	0.8738764	0.75642
fetal spinal cord 236	0.8077281	0.75652
ovary 326	0.7597799	0.75764
fetal lung 121	0.8411708	0.76090
fetal lung 118	0.8409046	0.76800
fetal lung 117	0.8010149	0.80090
fetal lung 111	0.8009298	0.80466
fetal muscle 181	0.7857711	0.80492
hematopoietic stem cell 318	0.7241610	0.81012
fetal lung 141	0.8033430	0.81332
fetal lung 126	0.7898315	0.81838
hematopoietic stem cell 314	0.7498607	0.81844
CD14 4	0.6993911	0.82004
fetal small intestine 69	0.6965434	0.82814
fetal lung 124	0.7696861	0.83020
fetal lung 136	0.7646056	0.83336
fetal lung 132	0.7695977	0.83376
hematopoietic stem cell 317	0.7496291	0.83482
fetal lung 140	0.7751216	0.83550
ES derived mesoderm 285	0.7858889	0.83670
fetal large intestine 55	0.7239533	0.83778
fetal lung 109	0.7824594	0.83914
fetal kidney 92	0.7246629	0.83970
fetal large intestine 58	0.7289836	0.84008
fetal brain 41	0.8026087	0.84030
fetal kidney 85	0.7576684	0.84078
fetal kidney 96	0.7187655	0.84136
fetal lung 129	0.7624996	0.84162
fetal renal cortex 206	0.7146858	0.84326
fetal lung 116	0.7780402	0.84776
fetal spinal cord 238	0.7519222	0.84828
fetal kidney 91	0.7295442	0.84916
hematopoietic stem cell 306	0.6930372	0.85094
fetal muscle 154	0.7063838	0.85266
placenta 199	0.7066985	0.85314
fetal kidney 93	0.6765242	0.85352
hematopoietic stem cell 305	0.6866605	0.85398
Mobilized CD4 323	0.6038172	0.85524
hematopoietic stem cell 315	0.6904746	0.85530
fetal spinal cord 237	0.7490529	0.85550
fetal lung 120	0.7487759	0.85586
fetal testes 255	0.7222846	0.85610
fetal spinal cord 239	0.7382262	0.85740
fetal large intestine 57	0.7113136	0.85892
fetal lung 113	0.7584236	0.85914
fetal lung 134	0.7360627	0.85950
fetal thymus 261	0.6205063	0.86078
fetal lung 125	0.7299640	0.86112
fetal renal pelvis 231	0.7017133	0.86256
hematopoietic stem cell 307	0.6778228	0.86440
hematopoietic stem cell 309	0.6870410	0.86528
fetal lung 135	0.7305603	0.86564
fetal thymus 259	0.6017975	0.86650
fetal muscle 157	0.7442346	0.86712
fetal lung 138	0.7302222	0.86724
hematopoietic stem cell 312	0.6925434	0.86790
fetal muscle 172	0.7541985	0.86802
fetal thymus 256	0.6394023	0.86820
fetal lung 119	0.7644212	0.86874
fetal renal cortex 203	0.6725908	0.86886
fetal kidney 89	0.6702393	0.86902
fetal renal pelvis 227	0.6736267	0.86936
fetal renal cortex 215	0.6920057	0.86980
fetal kidney 106	0.6778148	0.86990
fetal large intestine 67	0.6798089	0.86992
fetal renal cortex 204	0.7029985	0.86996
hematopoietic stem cell 313	0.6902304	0.87046
fetal renal pelvis 226	0.6786786	0.87218
fetal large intestine 65	0.6538445	0.87234
fetal lung 115	0.7419400	0.87236
fetal renal pelvis 216	0.6618122	0.87362
fetal muscle 167	0.7302359	0.87464
fetal renal pelvis 230	0.6424175	0.87468
fetal renal cortex 207	0.6787324	0.87646
small intestine 342	0.6604125	0.87858
hematopoietic stem cell 311	0.6669232	0.87908
fetal renal pelvis 217	0.6453756	0.87908
hematopoietic stem cell 308	0.6747082	0.87912
CD14 3	0.6022415	0.88050
Mobilized CD56 324	0.6080779	0.88126
fetal renal cortex 211	0.6401388	0.88256
fetal muscle 178	0.6938725	0.88288
fetal kidney 104	0.6524732	0.88390
fetal kidney 100	0.6593171	0.88426
fetal muscle 194	0.7266254	0.88430
fetal lung 131	0.7433788	0.88478
fetal skin 234	0.6854504	0.88484
fetal kidney 99	0.6577923	0.88580
fetal muscle 176	0.7381309	0.88610
fetal renal pelvis 228	0.6266739	0.88618
fetal kidney 95	0.6569221	0.88654
fetal kidney 103	0.6511943	0.88694
CD56 18	0.5564454	0.88788
CD20 8	0.6692762	0.88808
fetal renal cortex 214	0.6566163	0.88852
fetal renal cortex 213	0.6802613	0.88986
hematopoietic stem cell 319	0.6250155	0.89016
fetal lung 128	0.7400463	0.89060
fetal large intestine 60	0.6521433	0.89064
CD19 7	0.5649121	0.89068
fetal muscle 182	0.7000901	0.89156
fetal renal cortex 205	0.6513724	0.89268
fetal kidney 88	0.6671690	0.89304
fetal small intestine 71	0.6342094	0.89336
fetal testes 254	0.6542361	0.89374
fetal muscle 150	0.6708791	0.89396
fetal kidney 107	0.6480717	0.89398
fetal kidney 87	0.6559009	0.89406
fetal thymus 266	0.5850067	0.89570
pancreas 327	0.6117710	0.89594
fetal lung 110	0.6931134	0.89724
fetal large intestine 56	0.6384015	0.89750
hematopoietic stem cell 316	0.6273630	0.89766
CD8 19	0.5110050	0.89794
fetal renal pelvis 229	0.6450174	0.89800
fetal renal cortex 202	0.6507934	0.89826
fetal thymus 260	0.5816673	0.89882
fetal renal pelvis 221	0.6513217	0.89888
fetal kidney 94	0.6192464	0.89938
fetal renal cortex 212	0.6614884	0.89964
fetal large intestine 64	0.6468027	0.89972
placenta 198	0.6680034	0.89980
fetal kidney 98	0.6286196	0.89986
fetal small intestine 68	0.6516768	0.89994
fetal small intestine 78	0.6663456	0.90020
fetal muscle 183	0.6953887	0.90066
fetal lung 137	0.7034091	0.90096
fetal renal cortex 210	0.6198166	0.90128
fetal kidney 97	0.6425277	0.90142
fetal lung 114	0.7231774	0.90188
fetal small intestine 70	0.6749548	0.90272
fetal muscle 147	0.6713635	0.90306
fetal renal pelvis 225	0.6236892	0.90308
Mobilized CD3 320	0.5436587	0.90336
fetal lung 139	0.6855684	0.90350
fetal lung 123	0.6827254	0.90404
fetal kidney 84	0.6180839	0.90406
placenta 196	0.6233671	0.90534
fetal muscle 179	0.6881889	0.90628
fetal ovary 195	0.5714240	0.90644
fetal brain 32	0.7428407	0.90688
fetal large intestine 53	0.5737759	0.90710
fetal kidney 90	0.6245970	0.90742
fetal kidney 82	0.5878985	0.90886
fetal small intestine 77	0.6135148	0.90890
fetal small intestine 74	0.6246922	0.90914
placenta 200	0.6627907	0.90952
fetal thymus 264	0.5691074	0.90990
fetal lung 108	0.6682794	0.91084
fetal kidney 101	0.6284449	0.91130
fetal muscle 155	0.6454171	0.91178
fetal renal pelvis 222	0.6117233	0.91182
breast 1	0.7103754	0.91240
fetal kidney 83	0.5722203	0.91242
CD4 13	0.5116871	0.91256
fetal muscle 184	0.6867891	0.91370
fetal muscle 173	0.6523495	0.91382
fetal muscle 153	0.6373916	0.91456
fetal lung 122	0.6579355	0.91496
fetal muscle 169	0.6638609	0.91518
fetal large intestine 54	0.5992143	0.91522
melanocyte 340	0.7067309	0.91528
fetal kidney 102	0.5926771	0.91552
stomach 283	0.5829014	0.91554
CD3 10	0.5600291	0.91566
fetal renal cortex 209	0.6135567	0.91578
fetal stomach 240	0.5499148	0.91660
fetal renal cortex 208	0.6051042	0.91692
CD19 5	0.5409185	0.91700
fetal muscle 160	0.6485578	0.91704
fetal small intestine 76	0.5809005	0.91784
fetal stomach 251	0.5872232	0.91812
fetal thymus 265	0.5492818	0.91830
CD19 6	0.5224227	0.91866
fetal small intestine 73	0.5944711	0.91890
fetal muscle 190	0.6224307	0.91892
fetal muscle 146	0.6634507	0.91900
fetal small intestine 72	0.6017401	0.91926
fetal renal pelvis 220	0.6025189	0.91946
CD3 12	0.4945376	0.91960
Mobilized CD3 321	0.4824100	0.91968
fetal renal pelvis 224	0.6365878	0.91990
ES derived trophoblast 288	0.6794864	0.92024
fetal muscle 159	0.6265172	0.92026
fetal lung 127	0.6580132	0.92052
fetal muscle 170	0.6692190	0.92062
fetal renal pelvis 232	0.5901362	0.92064
fetal muscle 152	0.6787281	0.92074
fetal stomach 243	0.6038669	0.92144
CD56 17	0.5596989	0.92188
keratinocyte 334	0.7291819	0.92204
muscle 341	0.5754171	0.92210
fetal renal pelvis 219	0.5279433	0.92216
fetal thymus 262	0.4694046	0.92272
fetal muscle 180	0.6422530	0.92416
fetal large intestine 63	0.5775540	0.92460
fetal lung 130	0.6690766	0.92474
melanocyte 337	0.7227319	0.92486
fetal kidney 105	0.6213453	0.92530
fetal thymus 257	0.5065487	0.92574
fetal muscle 143	0.6868385	0.92632
fetal muscle 189	0.6408642	0.92726
pancreas 328	0.5850957	0.92768
Mobilized CD4 322	0.4797425	0.92772
fetal thymus 258	0.5346592	0.92820
fetal stomach 248	0.5472644	0.92978
fetal renal pelvis 223	0.5745734	0.92994
ES derived trophoblast 287	0.6528888	0.93000
fetal muscle 175	0.6462264	0.93042
placenta 197	0.5808595	0.93064
fetal lung 142	0.6789734	0.93092
fetal muscle 185	0.6084147	0.93264
fetal large intestine 62	0.5528742	0.93336
fetal muscle 162	0.6485295	0.93340
CD3 11	0.5149005	0.93362
fetal kidney 86	0.5237031	0.93386
fetal renal pelvis 233	0.5848072	0.93402
fetal muscle 165	0.6575280	0.93410
fetal brain 38	0.7052348	0.93414
fetal large intestine 66	0.5550681	0.93414
fetal thymus 263	0.6102616	0.93430
fetal small intestine 80	0.6023521	0.93434
CD4 14	0.5197963	0.93508
CD4 15	0.3992404	0.93516
fetal large intestine 59	0.5562176	0.93614
fetal kidney 81	0.6452678	0.93626
fetal muscle 158	0.6389143	0.93644
fetal muscle 187	0.6515317	0.93660
CD3 9	0.4798837	0.93684
placenta 201	0.5597568	0.93712
fetal stomach 246	0.5235123	0.93818
fetal muscle 151	0.6100586	0.93844
fetal muscle 161	0.6240069	0.93860
fetal muscle 144	0.6050502	0.93958
fetal muscle 171	0.6378895	0.93968
Mobilized CD8 325	0.4656870	0.93974
ES cells 293	0.5853132	0.94014
fetal stomach 250	0.5222547	0.94040
fetal small intestine 79	0.5413773	0.94114
fetal muscle 186	0.6250725	0.94152
fetal stomach 245	0.5297536	0.94248
keratinocyte 333	0.7027232	0.94290
breast 2	0.6709791	0.94296
fetal large intestine 61	0.4861647	0.94312
melanocyte 338	0.6967256	0.94330
ES cells 294	0.5702747	0.94440
fetal muscle 156	0.6171208	0.94560
fetal muscle 188	0.6207334	0.94616
fetal stomach 242	0.4554637	0.94620
iPS 303	0.6170642	0.94700
fetal muscle 149	0.5972311	0.94788
keratinocyte 335	0.6858832	0.94968
fetal muscle 148	0.5958957	0.95094
fetal stomach 252	0.5257913	0.95096
CD8 22	0.4405333	0.95136
fetal muscle 166	0.6226584	0.95152
fetal lung 112	0.6161918	0.95180
ES derived mesoderm 286	0.6763521	0.95206
fetal small intestine 75	0.3928903	0.95236
fetal muscle 174	0.5897734	0.95270
fetal stomach 244	0.5176358	0.95308
CD8 20	0.4682599	0.95328
fetal muscle 163	0.5902541	0.95402
ES cells 295	0.5431627	0.95412
fetal stomach 253	0.4889059	0.95502
fetal renal pelvis 218	0.4633947	0.95526
fetal muscle 191	0.5804532	0.95592
fetal stomach 241	0.4848084	0.95638
fetal muscle 177	0.5726628	0.95706
keratinocyte 336	0.6705735	0.95844
CD8 21	0.4076036	0.95852
iPS 302	0.5847577	0.95940
fetal muscle 145	0.5324775	0.96030
iPS 304	0.5998777	0.96100
fetal stomach 247	0.4225498	0.96272
stomach 284	0.5098177	0.96280
fetal stomach 249	0.4723952	0.96286
fetal muscle 193	0.5673962	0.96376
ES cells 296	0.5170104	0.96416
fetal brain 33	0.6297376	0.96434
fetal muscle 164	0.5677736	0.96730
CD4 16	0.3813478	0.96834
melanocyte 339	0.6334499	0.96994
ES derived NP 291	0.4835876	0.97154
fetal muscle 168	0.5175301	0.97832
fetal muscle 192	0.5479246	0.97918
ES derived NP 292	0.4492876	0.98350

Bone mineral density 4

DHS sample	fold enrichment	p value
keratinocyte 334	1.1786639	0.13608
fetal muscle 181	1.1978998	0.16956
fetal fibroblast 282	1.1587313	0.18796
fetal lung 298	1.1687961	0.20462
fetal fibroblast 281	1.1470056	0.21496
fetal fibroblast 271	1.1425395	0.22228
ES derived MSC 289	1.1640803	0.22296
keratinocyte 333	1.1224864	0.22426
keratinocyte 336	1.1176230	0.23812
keratinocyte 335	1.1171245	0.23998
fetal muscle 172	1.1294817	0.24188
fetal adrenal gland 26	1.1417159	0.24738
fetal lung 300	1.1308586	0.25248
fetal lung 301	1.1319884	0.25542
fetal fibroblast 274	1.1202957	0.25566
fetal fibroblast 278	1.1239434	0.25626
fetal muscle 187	1.1265632	0.25670
fetal fibroblast 275	1.1059766	0.27258
fetal fibroblast 273	1.1083243	0.27358
fetal fibroblast 268	1.1058028	0.27502
fetal lung 299	1.1198552	0.27728
fetal fibroblast 272	1.1051546	0.28000
fetal fibroblast 269	1.0990775	0.28962
breast 1	1.0966102	0.29148
breast 2	1.0957594	0.29356
fetal fibroblast 277	1.0935707	0.30570
fetal fibroblast 276	1.0880556	0.30964
ES derived MSC 290	1.0986625	0.31050
fetal muscle 155	1.0984740	0.31462
fetal fibroblast 280	1.0779866	0.32944
fetal muscle 159	1.0895058	0.33244
fetal muscle 158	1.0795777	0.33354
fetal muscle 149	1.0858788	0.33430
fetal muscle 165	1.0781618	0.33668
fetal muscle 166	1.0779147	0.34368
fetal muscle 150	1.0785417	0.34712
fetal fibroblast 279	1.0668372	0.35262
fetal lung 119	1.0598232	0.36056
fetal muscle 167	1.0681684	0.36224
fetal adrenal gland 25	1.0707740	0.36288
fetal muscle 160	1.0686335	0.36372
fetal muscle 169	1.0642179	0.36820
fetal adrenal gland 27	1.0636213	0.37082
fetal fibroblast 267	1.0547025	0.37698
fetal muscle 178	1.0570616	0.38182
fetal muscle 175	1.0519600	0.38688
fetal adrenal gland 28	1.0476354	0.39016
small intestine 342	1.0545919	0.39320
fetal muscle 146	1.0463182	0.39510
fetal muscle 148	1.0471345	0.39848
fetal muscle 174	1.0417167	0.40406
fetal muscle 151	1.0427844	0.40844
fetal muscle 153	1.0423157	0.40980
fetal muscle 179	1.0365631	0.41540
fetal muscle 143	1.0349142	0.41668
fetal muscle 186	1.0342149	0.41738
fetal kidney 81	1.0285354	0.42660
fetal stomach 243	1.0283589	0.42716
fetal muscle 189	1.0261105	0.42976
fetal muscle 191	1.0264875	0.43244
fetal muscle 147	1.0262422	0.43314
fetal fibroblast 270	1.0242835	0.43702
fetal muscle 152	1.0235394	0.44178
fetal muscle 171	1.0212478	0.44306
fetal muscle 164	1.0203095	0.44592
fetal muscle 192	1.0185982	0.44696
Mobilized CD56 324	1.0240997	0.44954
fetal muscle 180	1.0184482	0.44988
fetal muscle 176	1.0172669	0.45042
fetal muscle 163	1.0163687	0.45176
fetal muscle 170	1.0133731	0.45880
fetal muscle 173	1.0086197	0.46538
fetal muscle 157	1.0089683	0.46662
fetal adrenal gland 24	1.0070912	0.46856
fetal muscle 184	1.0074111	0.46964
fibroblast 329	1.0100012	0.47050
ES derived trophoblast 288	1.0067496	0.47322
fetal lung 111	1.0084475	0.47418
fetal muscle 168	1.0067797	0.47556
fetal muscle 161	1.0031513	0.47600
fetal muscle 182	1.0033338	0.47890
fetal stomach 250	0.9947371	0.47988
fetal muscle 193	0.9995810	0.48534
melanocyte 337	1.0009094	0.49568
fibroblast 332	0.9979399	0.49852
fetal kidney 92	0.9891208	0.50266
fetal muscle 183	0.9898742	0.50270
fibroblast 330	0.9927258	0.50424
melanocyte 339	0.9957348	0.50438
fetal muscle 162	0.9881411	0.50480
fetal muscle 156	0.9885433	0.50534
fetal muscle 145	0.9807204	0.50728
fetal lung 133	0.9920756	0.51018
fetal lung 132	0.9882605	0.51028
fetal lung 123	0.9844858	0.51560
fetal muscle 154	0.9777519	0.52070
fetal heart 47	0.9694498	0.52120
fetal adrenal gland 23	0.9777254	0.52606
fetal thymus 263	0.9787728	0.52666
fetal muscle 185	0.9753011	0.52802
placenta 199	0.9744699	0.53268
fetal muscle 188	0.9746517	0.53322
fibroblast 331	0.9804638	0.53422
placenta 200	0.9769501	0.53444
heart 297	0.9626044	0.54070
fetal stomach 246	0.9491603	0.54300
fetal lung 116	0.9784933	0.54502
fetal large intestine 53	0.9506217	0.54520
fetal lung 129	0.9754591	0.54610
iPS 303	0.9673472	0.54788
fetal lung 118	0.9767503	0.54914
fetal muscle 144	0.9617900	0.55042
fetal kidney 96	0.9628940	0.55180
fetal kidney 91	0.9589207	0.56372
fetal lung 113	0.9649607	0.56452
ES cells 293	0.9502047	0.56640
iPS 304	0.9600339	0.56646
melanocyte 338	0.9690967	0.56992
placenta 198	0.9577995	0.57070
fetal stomach 252	0.9397855	0.57074
fetal skin 234	0.9592669	0.57126
fetal lung 139	0.9538270	0.57336
stomach 284	0.9387833	0.57408
fetal lung 114	0.9644855	0.57448
fetal lung 136	0.9564529	0.57634
fetal large intestine 67	0.9470584	0.57738
fetal small intestine 80	0.9518474	0.57758
fetal muscle 194	0.9570933	0.58062
fetal renal pelvis 230	0.9337711	0.58694
fetal kidney 101	0.9417527	0.58818
ES derived mesoderm 286	0.9570934	0.59080
pancreas 328	0.9352078	0.59104
fetal stomach 253	0.9199791	0.59252
fetal lung 140	0.9535642	0.59592
stomach 283	0.9193314	0.59724
fetal lung 110	0.9438706	0.59870
fetal small intestine 72	0.9298934	0.59946
fetal small intestine 74	0.9335717	0.60024
fetal small intestine 68	0.9363356	0.60108
fetal stomach 240	0.9068575	0.60728
fetal stomach 244	0.9197190	0.61296
fetal lung 126	0.9455107	0.61328
fetal renal pelvis 226	0.9294425	0.61424
fetal lung 131	0.9453737	0.61612
placenta 197	0.9227483	0.61788
fetal muscle 190	0.9190147	0.61870
iPS 302	0.9320505	0.61914
fetal stomach 251	0.9058086	0.62730
fetal kidney 85	0.9361831	0.62752
fetal stomach 247	0.8765954	0.63020
fetal lung 108	0.9245367	0.63076
fetal large intestine 66	0.9029397	0.63216
fetal stomach 241	0.8899579	0.63418
fetal lung 138	0.9271665	0.63558
fetal lung 121	0.9350589	0.63588
fetal kidney 87	0.9184776	0.63666
ES cells 295	0.9041031	0.63756
fetal stomach 242	0.8659002	0.63838
fetal kidney 83	0.8945056	0.64072
ES derived trophoblast 287	0.9131346	0.64438
fetal lung 142	0.9268680	0.64482
fetal large intestine 63	0.8967618	0.64898
fetal renal pelvis 220	0.9060821	0.64934
fetal small intestine 70	0.9155366	0.65000
fetal lung 124	0.9232771	0.65078
fetal lung 115	0.9259197	0.65092
ES cells 294	0.8995124	0.65216
fetal large intestine 59	0.8896871	0.65302
fetal lung 135	0.9157001	0.65364
fetal stomach 245	0.8850157	0.65530
fetal large intestine 57	0.9066147	0.65634
fetal testes 255	0.9089197	0.65728
fetal small intestine 78	0.9076854	0.65848
placenta 196	0.8964589	0.65892
fetal renal cortex 207	0.9057330	0.66170
fetal large intestine 64	0.8969444	0.66252
fetal muscle 177	0.8929124	0.66302
fetal lung 130	0.9174141	0.66440
fetal renal pelvis 219	0.8691217	0.66496
fetal large intestine 55	0.8995305	0.66576
fetal kidney 99	0.8977193	0.67082
fetal lung 128	0.9240048	0.67132
fetal stomach 249	0.8647278	0.67228
fetal lung 109	0.9179938	0.67322
fetal renal cortex 202	0.9008840	0.67322
fetal renal pelvis 221	0.9011857	0.67412
fetal renal cortex 213	0.9070226	0.67472
fetal small intestine 77	0.8820360	0.67644
fetal renal pelvis 229	0.8926628	0.67918
fetal renal pelvis 233	0.8894866	0.67920
fetal heart 48	0.8842493	0.67962
fetal renal cortex 206	0.8949011	0.67972
fetal kidney 100	0.8893769	0.68038
pancreas 327	0.8654287	0.68194
fetal large intestine 58	0.8922184	0.68328
CD19 7	0.8364488	0.68506
fetal renal cortex 210	0.8784187	0.68546
fetal renal cortex 212	0.8937657	0.68604
fetal heart 46	0.8771631	0.68686
fetal testes 254	0.8839018	0.68692
fetal heart 43	0.8868019	0.68726
fetal kidney 98	0.8819034	0.68788
fetal lung 137	0.9017824	0.69058
fetal renal cortex 208	0.8791782	0.69242
fetal large intestine 56	0.8771124	0.69298
fetal heart 45	0.8748366	0.69360
fetal renal cortex 214	0.8861613	0.69376
fetal thymus 261	0.8459553	0.69530
fetal heart 49	0.8736298	0.69584
fetal lung 134	0.8910953	0.69722
fetal kidney 104	0.8753155	0.69762
fetal renal pelvis 232	0.8703025	0.70124
fetal kidney 95	0.8788864	0.70302
melanocyte 340	0.9009165	0.70330
fetal renal cortex 205	0.8773017	0.70370
fetal lung 112	0.8935447	0.70524
ES cells 296	0.8566186	0.70790
fetal kidney 84	0.8679354	0.70930
CD20 8	0.8756113	0.70938
CD19 6	0.8161514	0.70974
fetal lung 127	0.8870133	0.71004
hematopoietic stem cell 305	0.8730534	0.71020
fetal renal pelvis 217	0.8651082	0.71054
fetal kidney 107	0.8728697	0.71122
fetal lung 120	0.8923707	0.71196
fetal kidney 88	0.8744619	0.71210
fetal renal cortex 203	0.8645828	0.71778
fetal kidney 93	0.8544576	0.71858
fetal lung 125	0.8831966	0.71952
fetal stomach 248	0.8467511	0.71970
fetal kidney 89	0.8610279	0.72088
fetal kidney 102	0.8564860	0.72188
fetal renal pelvis 225	0.8561410	0.72408
fetal brain 30	0.9156344	0.72426
fetal renal pelvis 231	0.8736749	0.72510
fetal renal pelvis 228	0.8490512	0.72510
fetal renal pelvis 222	0.8532115	0.72640
fetal large intestine 65	0.8438976	0.72932
fetal renal pelvis 227	0.8634236	0.73064
CD14 4	0.8266054	0.73110
hematopoietic stem cell 306	0.8570749	0.73336
fetal renal cortex 215	0.8673945	0.73396
fetal renal pelvis 224	0.8675962	0.73402
CD3 10	0.8125653	0.73434
fetal lung 122	0.8609761	0.73654
hematopoietic stem cell 315	0.8504466	0.73754
placenta 201	0.8387549	0.73778
fetal large intestine 60	0.8463250	0.73958
CD14 3	0.8062608	0.74116
fetal thymus 264	0.8181299	0.74126
fetal small intestine 69	0.8288330	0.74130
fetal kidney 94	0.8471646	0.74392
fetal kidney 90	0.8423060	0.74648
fetal heart 44	0.8107089	0.74736
CD56 17	0.8122152	0.74880
fetal renal cortex 209	0.8421178	0.75172
fetal kidney 105	0.8535369	0.75212
fetal kidney 86	0.8061650	0.75214
CD19 5	0.7958123	0.75222
CD56 18	0.7718687	0.75576
fetal lung 117	0.8736858	0.75588
fetal lung 141	0.8763419	0.75720
hematopoietic stem cell 318	0.8341035	0.75726
fetal kidney 106	0.8491260	0.75734
fetal thymus 257	0.7820587	0.75804
hematopoietic stem cell 319	0.8336382	0.75908
fetal renal cortex 211	0.8241442	0.76008
fetal kidney 103	0.8432710	0.76220
fetal small intestine 71	0.8225624	0.76418
fetal spinal cord 236	0.8498244	0.76540
fetal thymus 262	0.7501195	0.76614
fetal heart 52	0.8444285	0.76742
fetal renal pelvis 216	0.8286952	0.76894
fetal small intestine 76	0.8057775	0.76956
fetal large intestine 62	0.8059262	0.77140
fetal thymus 266	0.7965516	0.77164
fetal spinal cord 235	0.8571884	0.77250
fetal large intestine 54	0.8145057	0.77556
fetal thymus 259	0.7795152	0.77660
fetal thymus 265	0.7831220	0.77772
fetal renal pelvis 218	0.7832628	0.77798
fetal renal cortex 204	0.8393700	0.78120
ES derived mesoderm 285	0.8635527	0.78132
fetal brain 41	0.8627612	0.78154
ES derived NP 292	0.8138339	0.78284
fetal renal pelvis 223	0.8039348	0.78634
fetal small intestine 79	0.7937530	0.78756
fetal small intestine 73	0.8080715	0.78790
CD8 22	0.7349661	0.78806
hematopoietic stem cell 307	0.8242567	0.78868
fetal kidney 97	0.8220466	0.78890
hematopoietic stem cell 309	0.8224336	0.78906
fetal brain 39	0.8658936	0.79006
CD3 11	0.7666521	0.79098
fetal heart 51	0.8163153	0.79424
fetal heart 42	0.8103889	0.79676
fetal brain 35	0.8728058	0.79780
hematopoietic stem cell 308	0.8303331	0.80014
fetal heart 50	0.8037202	0.80134
hematopoietic stem cell 312	0.8225930	0.80182
ovary 326	0.7639748	0.80196
hematopoietic stem cell 311	0.8174987	0.80232
fetal brain 33	0.8338674	0.80354
muscle 341	0.7839763	0.80542
fetal thymus 256	0.7683159	0.80860
Mobilized CD3 320	0.7286201	0.80996
hematopoietic stem cell 314	0.8219167	0.81346
CD8 20	0.7345082	0.81674
fetal brain 32	0.8385852	0.81678
Mobilized CD8 325	0.7166278	0.81772
hematopoietic stem cell 310	0.8260339	0.81820
CD3 9	0.7226538	0.81840
hematopoietic stem cell 313	0.8115781	0.81888
ES derived NP 291	0.7812275	0.82238
fetal small intestine 75	0.6675323	0.82330
fetal brain 40	0.8551781	0.82392
fetal large intestine 61	0.7429757	0.82624
fetal ovary 195	0.7598501	0.82682
hematopoietic stem cell 317	0.8169489	0.82778
fetal thymus 260	0.7344385	0.83766
hematopoietic stem cell 316	0.7659720	0.84074
CD8 19	0.6636090	0.84482
CD3 12	0.6800622	0.84496
fetal kidney 82	0.7473030	0.85406
fetal brain 29	0.8484445	0.85480
CD8 21	0.6735707	0.85502
fetal spinal cord 238	0.7937400	0.86156
CD4 13	0.6627231	0.86236
fetal brain 36	0.8186248	0.86324
CD4 15	0.6055871	0.86332
CD4 14	0.7061118	0.86496
Mobilized CD4 323	0.6745514	0.86658
CD4 16	0.6296703	0.88584
fetal brain 38	0.7846279	0.88618
fetal spinal cord 239	0.7571196	0.89734
fetal brain 34	0.8044305	0.90158
Mobilized CD3 321	0.6010525	0.90458
Mobilized CD4 322	0.6031002	0.90788
fetal spinal cord 237	0.7580703	0.90814
fetal thymus 258	0.6491490	0.91636
fetal brain 31	0.8063105	0.91828
fetal brain 37	0.7644328	0.93012

Bone mineral density 3

DHS sample	fold enrichment	p value
fetal renal pelvis 230	1.3431078	0.09376
fetal renal cortex 208	1.3195115	0.09610
fetal stomach 240	1.3577332	0.10848
fetal kidney 101	1.2888459	0.10892
ES derived MSC 289	1.2881824	0.10930
fetal stomach 241	1.3404207	0.10932
ES derived MSC 290	1.2827946	0.10954
fetal renal cortex 209	1.2703696	0.13122
fetal kidney 98	1.2647473	0.13188
fetal renal cortex 202	1.2513490	0.13706
fetal kidney 93	1.2758064	0.13946
fetal kidney 96	1.2369744	0.14156
fetal kidney 107	1.2453615	0.14510
fetal stomach 249	1.2811155	0.15256
fetal renal cortex 212	1.2213205	0.15570
fetal kidney 90	1.2349570	0.15800
ES derived NP 292	1.2534353	0.16028
fetal stomach 250	1.2709420	0.16200
iPS 304	1.1997850	0.16428
fetal renal pelvis 222	1.2347356	0.16530
fetal renal pelvis 225	1.2306044	0.16672
iPS 303	1.2075394	0.16830
fetal kidney 104	1.2224181	0.16980
fetal kidney 84	1.2230826	0.17066
fetal kidney 102	1.2297753	0.17184
fetal kidney 92	1.1930127	0.18408
fetal stomach 248	1.2348170	0.18436
fetal renal pelvis 217	1.2105630	0.18626
fetal renal cortex 203	1.2004768	0.19236
fetal stomach 242	1.2647212	0.19250
fetal renal cortex 215	1.1850248	0.19302
fetal stomach 247	1.2563233	0.19520
fetal renal cortex 206	1.1845685	0.19880
fetal stomach 252	1.2081996	0.20048
fetal renal cortex 210	1.2005371	0.20146
fetal renal pelvis 223	1.2033890	0.20272
fetal renal cortex 214	1.1863763	0.20442
fetal kidney 83	1.2096002	0.20640
fetal renal pelvis 219	1.2243716	0.20876
fetal renal pelvis 233	1.1815605	0.20990
iPS 302	1.1649390	0.21572
fetal renal pelvis 218	1.2138075	0.21800
fetal renal cortex 213	1.1662633	0.21906
breast 1	1.1380674	0.22000
fetal kidney 86	1.2040251	0.22010
fetal kidney 89	1.1751780	0.22014
fetal renal pelvis 229	1.1675558	0.22380
ES cells 293	1.1734677	0.22554
fetal renal cortex 205	1.1669596	0.22824
fetal renal pelvis 216	1.1613150	0.23580
ES cells 295	1.1716292	0.23582
fetal kidney 94	1.1663201	0.23920
fetal renal cortex 211	1.1646192	0.24000
fetal renal pelvis 224	1.1458763	0.24156
fetal renal pelvis 232	1.1601958	0.24468
fetal stomach 246	1.1742453	0.24796
fetal renal cortex 207	1.1478031	0.24896
fetal stomach 244	1.1633002	0.25148
stomach 283	1.1657281	0.25308
fetal kidney 97	1.1427151	0.25636
fetal kidney 88	1.1377090	0.25636
fetal renal pelvis 228	1.1505560	0.25852
fetal kidney 95	1.1423834	0.26124
melanocyte 337	1.1001676	0.26138
fetal renal pelvis 227	1.1341224	0.26824
breast 2	1.1087535	0.27004
fetal kidney 103	1.1334179	0.27058
fetal muscle 145	1.1388665	0.27154
fetal renal pelvis 220	1.1350933	0.27248
fetal renal cortex 204	1.1232865	0.27464
fetal adrenal gland 26	1.1271069	0.27966
fetal kidney 91	1.1187254	0.27996
fetal stomach 251	1.1390117	0.28144
ES cells 296	1.1363084	0.28212
fetal kidney 82	1.1331292	0.28344
fetal kidney 100	1.1245307	0.28364
fetal stomach 245	1.1374360	0.28678
ES derived NP 291	1.1330979	0.28698
fetal renal pelvis 226	1.1168808	0.29224
melanocyte 340	1.0936660	0.29258
fetal lung 298	1.1130073	0.29654
fetal kidney 87	1.1111829	0.29858
fetal lung 126	1.0966415	0.30176
keratinocyte 334	1.0839413	0.30578
fetal fibroblast 269	1.0896664	0.31258
fetal lung 112	1.0930169	0.31466
fetal muscle 191	1.1000064	0.31564
fetal renal pelvis 221	1.1007012	0.31586
fetal kidney 105	1.0970303	0.31928
fetal adrenal gland 25	1.1036903	0.32198
fetal kidney 99	1.0958664	0.32510
keratinocyte 333	1.0736301	0.32550
fetal muscle 172	1.0831907	0.32590
ES derived mesoderm 286	1.0745148	0.32636
fetal lung 123	1.0863823	0.33062
fetal adrenal gland 27	1.0942294	0.33112
fetal adrenal gland 24	1.1032137	0.33156
fetal lung 113	1.0810137	0.33200
fetal stomach 243	1.0948502	0.33268
stomach 284	1.0996321	0.33282
fetal muscle 174	1.0879671	0.33322
keratinocyte 336	1.0712043	0.33338
fetal lung 124	1.0839622	0.33418
fetal lung 301	1.0832540	0.33966
pancreas 328	1.0933815	0.34110
fetal lung 115	1.0731772	0.34640
fetal lung 136	1.0715997	0.35250
fetal renal pelvis 231	1.0722261	0.35298
ES cells 294	1.0726348	0.35834
fetal stomach 253	1.0836848	0.36004
fetal testes 254	1.0736541	0.36212
fetal lung 135	1.0644022	0.36704
fetal lung 122	1.0645465	0.36848
fetal kidney 85	1.0570320	0.36908
fetal lung 118	1.0583871	0.37020
small intestine 342	1.0701025	0.37118
fetal brain 39	1.0462514	0.37830
fetal fibroblast 270	1.0496758	0.38614
fetal kidney 81	1.0516139	0.38868
fetal lung 130	1.0500237	0.38868
keratinocyte 335	1.0452945	0.39068
fetal adrenal gland 23	1.0483514	0.39514
pancreas 327	1.0565837	0.39592
fetal large intestine 53	1.0547478	0.39730
fetal muscle 177	1.0476448	0.39952
fetal ovary 195	1.0487062	0.39958
fetal brain 41	1.0377186	0.40130
fetal muscle 144	1.0445289	0.40312
fetal spinal cord 237	1.0405609	0.40450
fetal lung 142	1.0372625	0.41062
fetal lung 300	1.0406021	0.41238
fetal kidney 106	1.0371071	0.41532
melanocyte 339	1.0353375	0.41552
fetal muscle 192	1.0344821	0.41622
fetal muscle 163	1.0325172	0.41958
fetal lung 116	1.0320539	0.42292
fetal muscle 190	1.0317487	0.42602
fetal lung 131	1.0284865	0.42636
fetal lung 119	1.0304466	0.42832
fetal brain 29	1.0214713	0.42948
fetal brain 35	1.0209446	0.43408
fetal lung 127	1.0250261	0.43554
fetal lung 109	1.0235728	0.43588
ES derived mesoderm 285	1.0258699	0.43590
fetal lung 139	1.0196923	0.44250
fetal small intestine 76	1.0177015	0.44812
fetal large intestine 59	1.0183905	0.44948
fetal lung 129	1.0197738	0.45062
fetal lung 121	1.0177287	0.45178
fetal large intestine 57	1.0211112	0.45230
fetal muscle 193	1.0129959	0.45418
fetal small intestine 70	1.0158628	0.46126
fetal lung 114	1.0092375	0.46708
fetal muscle 194	1.0075977	0.46868
fetal lung 299	1.0084184	0.46948
fetal fibroblast 279	1.0076369	0.47084
fetal lung 120	1.0084647	0.47162
fetal lung 140	1.0087445	0.47164
fetal large intestine 64	1.0052290	0.47544
placenta 199	1.0054373	0.47676
fetal muscle 161	1.0000630	0.47728
fetal lung 134	1.0006025	0.48102
fetal muscle 150	0.9967625	0.48288
fibroblast 329	1.0024496	0.48500
fetal muscle 162	0.9982153	0.48554
fetal small intestine 71	0.9955414	0.48592
fetal muscle 160	0.9968226	0.48598
fetal large intestine 63	0.9936566	0.48648
fetal muscle 173	0.9951798	0.48734
fetal large intestine 65	0.9920502	0.48756
fetal thymus 263	0.9974678	0.48808
fetal spinal cord 235	0.9982042	0.48904
fetal large intestine 66	0.9869552	0.49458
fetal muscle 159	0.9890735	0.49496
fetal muscle 187	0.9945401	0.49556
fetal muscle 188	0.9935191	0.49592
fetal muscle 165	0.9938736	0.49658
fetal muscle 185	0.9885862	0.49686
fibroblast 332	0.9956918	0.49748
fetal muscle 175	0.9879611	0.50294
fetal muscle 147	0.9848936	0.50392
fetal adrenal gland 28	0.9908363	0.50544
fetal lung 132	0.9879441	0.50710
CD19 6	0.9701430	0.50776
fetal small intestine 77	0.9806839	0.50990
fetal testes 255	0.9843825	0.51010
Mobilized CD56 324	0.9775913	0.51198
fetal lung 133	0.9873985	0.51208
fetal muscle 182	0.9837133	0.51232
fetal lung 138	0.9839171	0.51308
fibroblast 330	0.9872845	0.51324
fetal lung 108	0.9822519	0.51334
hematopoietic stem cell 319	0.9763396	0.51492
fetal lung 117	0.9852282	0.51690
fetal muscle 171	0.9808173	0.51908
fetal muscle 181	0.9815096	0.52024
fetal lung 137	0.9801668	0.52056
fetal muscle 158	0.9790494	0.52346
fetal muscle 184	0.9788679	0.52512
fetal muscle 154	0.9703750	0.52742
fetal brain 30	0.9864544	0.52836
fetal brain 34	0.9817016	0.53026
fetal lung 125	0.9695700	0.53354
fetal muscle 189	0.9702711	0.53356
fetal spinal cord 236	0.9740647	0.53430
fetal fibroblast 281	0.9779963	0.53458
placenta 196	0.9691395	0.53746
fetal muscle 170	0.9698966	0.53938
CD19 7	0.9411206	0.54118
fetal muscle 148	0.9658045	0.54166
fetal small intestine 78	0.9685942	0.54326
fetal skin 234	0.9707526	0.54348
fetal fibroblast 276	0.9727086	0.54360
fibroblast 331	0.9719209	0.54616
fetal small intestine 69	0.9525225	0.54644
fetal large intestine 55	0.9621671	0.54670
fetal fibroblast 282	0.9717270	0.54762
fetal large intestine 67	0.9603062	0.54780
fetal large intestine 60	0.9579164	0.54858
fetal small intestine 72	0.9559023	0.54918
fetal fibroblast 277	0.9664019	0.55176
fetal muscle 167	0.9633741	0.55248
placenta 198	0.9647819	0.55492
melanocyte 338	0.9772846	0.55526
fetal fibroblast 275	0.9649200	0.56050
ES derived trophoblast 287	0.9560139	0.56094
fetal muscle 168	0.9527497	0.56332
fetal large intestine 62	0.9390295	0.56556
fetal spinal cord 238	0.9583915	0.57010
fetal muscle 186	0.9472160	0.57854
fetal small intestine 74	0.9378970	0.58408
fetal muscle 146	0.9435096	0.58532
fetal muscle 143	0.9458789	0.58568
CD19 5	0.9154552	0.58770
fetal muscle 166	0.9431509	0.58782
fetal thymus 257	0.9151766	0.58826
ES derived trophoblast 288	0.9443031	0.58918
fetal fibroblast 280	0.9456496	0.59314
fetal brain 38	0.9498358	0.59760
fetal muscle 183	0.9400084	0.59810
fetal muscle 169	0.9345323	0.60080
placenta 197	0.9313915	0.60328
fetal lung 141	0.9405048	0.60814
fetal spinal cord 239	0.9367256	0.60936
ovary 326	0.8988264	0.61042
fetal large intestine 58	0.9272224	0.61098
fetal large intestine 56	0.9205061	0.61194
fetal brain 33	0.9409322	0.61238
fetal thymus 262	0.8704860	0.61298
fetal lung 110	0.9269379	0.61456
fetal fibroblast 278	0.9351269	0.61514
fetal fibroblast 273	0.9350684	0.61986
fetal muscle 156	0.9215475	0.62198
fetal muscle 151	0.9133340	0.62400
fetal fibroblast 274	0.9314206	0.62546
fetal muscle 178	0.9162358	0.62624
fetal small intestine 79	0.9009965	0.62722
fetal brain 31	0.9479133	0.62850
fetal small intestine 75	0.8409844	0.63076
fetal lung 111	0.9269727	0.63084
fetal fibroblast 272	0.9305531	0.63180
fetal fibroblast 267	0.9257035	0.63520
fetal lung 128	0.9274763	0.64092
fetal muscle 149	0.9075126	0.64248
fetal muscle 179	0.9129620	0.64436
fetal fibroblast 268	0.9263793	0.64456
fetal brain 36	0.9287163	0.64902
fetal muscle 157	0.9144218	0.65242
fetal small intestine 80	0.8970139	0.65658
placenta 201	0.8882407	0.65880
fetal small intestine 73	0.8814385	0.66586
CD20 8	0.8924238	0.66634
fetal fibroblast 271	0.9092577	0.67348
fetal muscle 153	0.8850023	0.67380
fetal brain 32	0.9156346	0.67588
hematopoietic stem cell 306	0.8848695	0.67782
CD3 10	0.8457859	0.67808
hematopoietic stem cell 307	0.8864209	0.67832
fetal large intestine 54	0.8708350	0.68274
placenta 200	0.8973770	0.68400
fetal thymus 261	0.8413644	0.68600
fetal muscle 164	0.8831492	0.68794
fetal brain 37	0.9155163	0.68898
CD3 11	0.8423567	0.69044
fetal muscle 155	0.8705898	0.69220
CD56 18	0.8232362	0.69442
CD8 22	0.8149985	0.70046
fetal muscle 152	0.8798197	0.70174
hematopoietic stem cell 305	0.8691681	0.70184
heart 297	0.8549834	0.70944
fetal muscle 180	0.8616077	0.71408
fetal thymus 259	0.8171713	0.71410
hematopoietic stem cell 318	0.8498288	0.71818
hematopoietic stem cell 308	0.8605617	0.72978
Mobilized CD3 320	0.7844729	0.73846
hematopoietic stem cell 316	0.8340659	0.74036
fetal large intestine 61	0.8023472	0.74288
fetal small intestine 68	0.8377277	0.74912
hematopoietic stem cell 311	0.8336442	0.75838
fetal thymus 260	0.7921478	0.76026
Mobilized CD4 322	0.7557883	0.76476
muscle 341	0.8036489	0.76858
CD4 16	0.7513470	0.76902
CD8 19	0.7347735	0.77198
fetal muscle 176	0.8446246	0.77382
CD8 21	0.7447278	0.77820
CD4 15	0.7002169	0.77964
fetal thymus 265	0.7689082	0.78532
CD3 12	0.7330886	0.78928
hematopoietic stem cell 309	0.8090724	0.79366
fetal heart 44	0.7700452	0.79732
Mobilized CD8 325	0.7350592	0.79938
Mobilized CD3 321	0.7129967	0.80694
fetal heart 50	0.7990535	0.80826
CD56 17	0.7528507	0.81078
hematopoietic stem cell 313	0.8006823	0.81508
fetal thymus 264	0.7464003	0.81908
fetal brain 40	0.8538506	0.81928
hematopoietic stem cell 317	0.8107613	0.81956
Mobilized CD4 323	0.7083711	0.82050
hematopoietic stem cell 314	0.7989369	0.82124
CD4 14	0.7340728	0.82256
CD8 20	0.7219533	0.82670
hematopoietic stem cell 315	0.7722774	0.82810
CD3 9	0.6970373	0.83332
hematopoietic stem cell 312	0.7789266	0.84154
fetal heart 42	0.7750961	0.84394
fetal heart 43	0.7829596	0.85224
fetal heart 51	0.7658591	0.85798
fetal thymus 266	0.6992836	0.85882
fetal heart 46	0.7500376	0.86004
fetal heart 48	0.7572563	0.86344
fetal heart 49	0.7499563	0.86796
hematopoietic stem cell 310	0.7678043	0.86824
fetal thymus 256	0.6949364	0.87194
fetal heart 45	0.7443928	0.87590
fetal thymus 258	0.6810672	0.88072
fetal heart 47	0.7085090	0.89002
fetal heart 52	0.7486927	0.89196
CD4 13	0.6225969	0.89306
CD14 3	0.6217650	0.90922
CD14 4	0.6051289	0.92928

Refractive error and myopia

DHS sample	fold enrichment	p value
ES cells 295	1.2506582	0.10510
iPS 302	1.1585782	0.16950
iPS 304	1.1153017	0.22390
ES cells 296	1.0972653	0.29078
ES cells 294	1.0875449	0.29982
iPS 303	1.0764785	0.30906
fetal heart 45	1.0858197	0.31934
ES cells 293	1.0737038	0.32248
ES derived NP 291	1.0747424	0.33664
fetal spinal cord 235	1.0567668	0.35350
fetal heart 47	1.0334891	0.41356
ES derived NP 292	1.0272862	0.41968
ES derived mesoderm 286	1.0127662	0.45060
fetal heart 49	1.0131227	0.45188
ES derived mesoderm 285	1.0034186	0.48338
fetal brain 33	0.9739413	0.55386
hematopoietic stem cell 319	0.9619914	0.55608
fetal brain 32	0.9750880	0.56002
fetal spinal cord 239	0.9720144	0.56198
fetal spinal cord 238	0.9690801	0.56760
fetal heart 44	0.9328544	0.58274
fetal spinal cord 236	0.9585010	0.58998
fetal ovary 195	0.9292977	0.59970
fetal brain 37	0.9531821	0.62590
fetal brain 36	0.9502097	0.63008
fetal brain 38	0.9482712	0.63918
hematopoietic stem cell 318	0.9149134	0.65542
fetal heart 43	0.9195805	0.65734
fetal muscle 168	0.9175323	0.66168
fetal muscle 178	0.9191662	0.66184
fetal heart 48	0.9110147	0.66226
fetal renal pelvis 230	0.9063423	0.66774
fetal muscle 166	0.9148207	0.67538
hematopoietic stem cell 314	0.9156858	0.67616
fetal lung 118	0.9257500	0.67648
fetal muscle 143	0.9154689	0.68276
fetal heart 52	0.9117149	0.68482
fetal thymus 263	0.9081176	0.68674
fetal muscle 154	0.8989645	0.69174
fetal heart 51	0.8954305	0.69482
fetal muscle 155	0.8960103	0.69862
fetal heart 42	0.8870977	0.70696
fetal muscle 150	0.8927283	0.70920
fetal brain 31	0.9381052	0.71056
fetal brain 29	0.9361374	0.71860
fetal renal cortex 202	0.8923254	0.72108
fetal muscle 182	0.8930932	0.72372
fetal brain 40	0.9248574	0.72622
fetal lung 141	0.8978978	0.72802
fetal lung 111	0.8931735	0.72922
fetal muscle 160	0.8857581	0.72982
fetal heart 46	0.8674244	0.73024
fetal adrenal gland 26	0.8847726	0.73104
fetal heart 50	0.8750941	0.73158
fetal brain 34	0.9202755	0.73528
fetal lung 119	0.8962212	0.73546
fetal muscle 167	0.8850339	0.73724
fetal kidney 92	0.8810063	0.73812
fetal stomach 250	0.8572755	0.74072
fetal kidney 103	0.8701326	0.74486
Mobilized CD56 324	0.8293170	0.74736
fetal muscle 187	0.8855577	0.74794
fetal muscle 181	0.8751330	0.75108
fetal kidney 99	0.8663359	0.75526
fetal muscle 169	0.8703174	0.75566
fetal muscle 165	0.8795471	0.75598
fetal thymus 262	0.7928113	0.75788
fetal muscle 173	0.8775705	0.75790
fetal muscle 193	0.8734305	0.75854
fetal brain 30	0.9237559	0.75908
fetal lung 115	0.8873005	0.75924
hematopoietic stem cell 308	0.8767533	0.75932
fetal muscle 176	0.8751935	0.76184
fetal lung 112	0.8832454	0.76396
fetal lung 123	0.8779332	0.76426
melanocyte 337	0.9012335	0.76514
muscle 341	0.8369720	0.76606
fetal muscle 186	0.8714564	0.76972
fetal muscle 163	0.8651288	0.77096
fetal lung 135	0.8725518	0.77432
fetal muscle 164	0.8581216	0.77586
fetal muscle 171	0.8693305	0.77636
fetal muscle 183	0.8706760	0.77660
fetal lung 116	0.8818526	0.77786
hematopoietic stem cell 311	0.8603254	0.77926
fetal muscle 175	0.8641854	0.78000
fetal lung 124	0.8706743	0.78134
fetal lung 131	0.8833144	0.78194
fetal muscle 153	0.8544832	0.78200
hematopoietic stem cell 317	0.8686657	0.78276
fetal muscle 145	0.8488798	0.78288
fetal brain 41	0.8965820	0.78302
fetal lung 121	0.8749571	0.78344
fetal muscle 161	0.8610400	0.78428
fetal muscle 174	0.8591992	0.78570
fetal renal cortex 208	0.8511443	0.78606
hematopoietic stem cell 307	0.8549125	0.78632
small intestine 342	0.8358097	0.78672
fetal muscle 148	0.8530935	0.78676
fetal lung 109	0.8816120	0.78748
fetal spinal cord 237	0.8791939	0.78788
fetal muscle 147	0.8561218	0.78800
hematopoietic stem cell 313	0.8582883	0.78822
fetal renal cortex 215	0.8616150	0.78910
fetal kidney 95	0.8473968	0.78944
fetal muscle 159	0.8495434	0.79236
fetal stomach 240	0.8207222	0.79246
fetal muscle 151	0.8382999	0.79296
stomach 284	0.8220611	0.79300
fetal adrenal gland 27	0.8477604	0.79304
fetal adrenal gland 24	0.8299775	0.79348
fetal muscle 156	0.8538195	0.79396
fetal muscle 180	0.8455616	0.79558
fetal stomach 241	0.8204398	0.79638
fetal muscle 170	0.8594020	0.79828
CD4 15	0.7269444	0.80054
pancreas 328	0.8227618	0.80306
fetal lung 130	0.8596912	0.80406
fetal muscle 188	0.8538144	0.80408
fetal muscle 189	0.8504484	0.80472
fetal thymus 259	0.7827444	0.80532
hematopoietic stem cell 306	0.8409978	0.80902
fetal lung 128	0.8674740	0.81012
fetal muscle 157	0.8522963	0.81056
fetal renal pelvis 233	0.8376472	0.81114
fetal thymus 266	0.7937251	0.81208
fetal kidney 86	0.8104494	0.81286
fetal lung 140	0.8496942	0.81392
fetal muscle 179	0.8397853	0.81394
fetal muscle 144	0.8379825	0.81416
fetal renal pelvis 219	0.7964864	0.81622
fetal muscle 194	0.8524078	0.82080
fetal kidney 94	0.8215935	0.82142
fetal brain 35	0.8924705	0.82174
fetal renal pelvis 218	0.7911601	0.82270
fetal thymus 261	0.7765325	0.82270
fetal muscle 149	0.8266014	0.82274
fetal brain 39	0.8842516	0.82372
melanocyte 338	0.8719370	0.82410
fetal lung 133	0.8501838	0.82500
fetal stomach 246	0.8004076	0.82704
fetal lung 114	0.8526999	0.82794
fetal lung 113	0.8511326	0.82860
fetal renal cortex 210	0.8214275	0.82902
fetal stomach 242	0.7792150	0.83146
fetal renal pelvis 225	0.8225743	0.83176
fetal kidney 96	0.8337500	0.83306
fetal lung 126	0.8503111	0.83336
fetal muscle 158	0.8359494	0.83502
fetal muscle 191	0.8280887	0.83566
fetal renal pelvis 227	0.8227742	0.83636
ES derived trophoblast 288	0.8352727	0.83676
fetal kidney 98	0.8198140	0.84152
fetal testes 254	0.8217198	0.84160
fetal lung 120	0.8338943	0.84192
fetal thymus 260	0.7625255	0.84274
fetal muscle 152	0.8274018	0.84368
fetal renal pelvis 222	0.8121840	0.84380
fetal muscle 190	0.8107319	0.84602
hematopoietic stem cell 316	0.8025538	0.84610
hematopoietic stem cell 309	0.8099316	0.84670
fetal lung 134	0.8279513	0.84716
CD20 8	0.8049727	0.84832
fetal renal pelvis 224	0.8296552	0.84840
fetal large intestine 60	0.7962336	0.84996
fetal renal pelvis 229	0.8162415	0.85080
fetal small intestine 80	0.7997093	0.85080
fetal small intestine 71	0.8032224	0.85150
fetal lung 142	0.8441642	0.85164
fetal lung 132	0.8326451	0.85186
fetal kidney 105	0.8207122	0.85312
fetal renal pelvis 221	0.8190527	0.85392
hematopoietic stem cell 305	0.8060522	0.85682
fetal stomach 248	0.7830959	0.85708
fetal lung 137	0.8307666	0.85766
fetal kidney 93	0.8015676	0.85824
fetal stomach 249	0.7768750	0.85850
pancreas 327	0.7718818	0.85976
fetal muscle 192	0.8191832	0.86048
CD8 19	0.6919858	0.86082
fetal lung 138	0.8212774	0.86122
fetal renal cortex 205	0.8060101	0.86124
fetal renal pelvis 232	0.7973246	0.86232
fetal muscle 172	0.8305262	0.86252
fetal renal cortex 209	0.8023314	0.86288
fetal stomach 247	0.7499042	0.86376
fetal muscle 185	0.8024626	0.86500
Mobilized CD8 325	0.7272182	0.86524
fetal small intestine 69	0.7744285	0.86544
fetal muscle 184	0.8172823	0.86790
fetal small intestine 72	0.7833729	0.86854
fetal kidney 102	0.7920733	0.86866
fetal kidney 85	0.8141466	0.87112
fetal fibroblast 271	0.8233670	0.87154
fetal lung 139	0.8093804	0.87204
fetal lung 129	0.8151372	0.87232
fetal muscle 162	0.8110171	0.87272
fetal kidney 81	0.8173354	0.87450
fetal lung 122	0.8054263	0.87476
fetal muscle 146	0.8022035	0.87500
Mobilized CD4 322	0.6884090	0.87506
fetal large intestine 67	0.7700897	0.87512
fetal lung 136	0.8106926	0.87514
CD4 13	0.6900386	0.87598
fetal thymus 258	0.7338860	0.87634
fetal thymus 265	0.7344433	0.87642
fetal thymus 257	0.7336216	0.87646
stomach 283	0.7565249	0.87712
ES derived trophoblast 287	0.7911595	0.87730
fetal kidney 84	0.7868755	0.87796
fetal lung 127	0.8136739	0.87926
heart 297	0.7603853	0.87974
CD3 12	0.6955004	0.87998
fetal large intestine 64	0.7735236	0.88320
fetal kidney 83	0.7637786	0.88320
CD56 18	0.6976864	0.88366
Mobilized CD4 323	0.6916703	0.88366
fetal fibroblast 269	0.8212109	0.88444
fetal kidney 89	0.7878715	0.88468
fetal renal cortex 212	0.8020673	0.88492
fetal kidney 82	0.7710435	0.88498
fetal large intestine 53	0.7501828	0.88598
fetal large intestine 58	0.7765504	0.88636
Mobilized CD3 321	0.6793264	0.88640
fetal testes 255	0.7953589	0.88752
fetal lung 125	0.7980084	0.88822
fetal lung 117	0.8094157	0.88870
fetal adrenal gland 25	0.7689970	0.88908
fibroblast 329	0.7999929	0.88936
fetal large intestine 62	0.7515039	0.88956
fibroblast 330	0.7965248	0.88994
fetal renal cortex 211	0.7779372	0.89020
fetal renal pelvis 217	0.7750532	0.89026
fetal renal pelvis 226	0.7807765	0.89056
fetal renal cortex 214	0.7817889	0.89090
fetal large intestine 63	0.7557310	0.89158
hematopoietic stem cell 315	0.7692762	0.89232
fetal small intestine 74	0.7646270	0.89238
fetal kidney 104	0.7829571	0.89302
ovary 326	0.7013034	0.89492
placenta 200	0.7792616	0.89590
fetal kidney 101	0.7844891	0.89652
fetal kidney 88	0.7914471	0.89656
fetal kidney 90	0.7803054	0.89668
fetal kidney 107	0.7861525	0.89694
hematopoietic stem cell 312	0.7808110	0.89732
fetal stomach 244	0.7590707	0.89814
fetal thymus 256	0.7132113	0.89822
fetal small intestine 70	0.7756811	0.89978
fetal small intestine 78	0.7702912	0.90066
fetal fibroblast 272	0.8024509	0.90146
fetal fibroblast 270	0.7963323	0.90218
fetal renal cortex 204	0.7892320	0.90240
fetal lung 110	0.7852980	0.90286
fetal thymus 264	0.7130438	0.90292
placenta 197	0.7474141	0.90424
fetal renal pelvis 223	0.7568175	0.90660
fetal stomach 245	0.7452326	0.90724
fetal fibroblast 279	0.7942571	0.90790
breast 2	0.7968184	0.90872
fetal renal pelvis 228	0.7528854	0.90914
fetal stomach 243	0.7586659	0.90946
fetal stomach 252	0.7499196	0.91134
fetal stomach 253	0.7297670	0.91172
CD14 3	0.6671074	0.91228
melanocyte 340	0.8030623	0.91268
CD8 21	0.6475805	0.91490
CD14 4	0.6791604	0.91502
fetal kidney 97	0.7688307	0.91564
placenta 199	0.7463031	0.91580
fetal large intestine 59	0.7338948	0.91588
fibroblast 332	0.7752152	0.91720
fetal fibroblast 277	0.7811118	0.91782
fetal muscle 177	0.7576641	0.91784
fetal fibroblast 275	0.7965163	0.91788
fetal large intestine 56	0.7365064	0.91812
fetal renal cortex 207	0.7499448	0.91954
CD4 16	0.6457712	0.92022
fetal kidney 106	0.7648295	0.92036
fetal renal cortex 203	0.7602774	0.92216
fetal fibroblast 274	0.7836378	0.92236
fetal large intestine 66	0.7283803	0.92298
fetal large intestine 55	0.7385573	0.92348
fetal renal pelvis 220	0.7399059	0.92492
fetal adrenal gland 23	0.7678205	0.92552
fetal small intestine 76	0.7115695	0.92700
placenta 198	0.7410401	0.92744
fetal small intestine 75	0.6216235	0.92848
CD19 7	0.6324281	0.92884
CD19 6	0.6339621	0.92944
fetal lung 108	0.7590260	0.92954
fetal large intestine 54	0.7172737	0.93034
CD4 14	0.6622819	0.93082
fetal small intestine 77	0.7116442	0.93098
fetal fibroblast 273	0.7776858	0.93140
fetal fibroblast 280	0.7749232	0.93176
fetal fibroblast 276	0.7807466	0.93206
CD56 17	0.6770721	0.93242
fetal small intestine 79	0.7058993	0.93286
fetal small intestine 68	0.7252824	0.93490
hematopoietic stem cell 310	0.7530881	0.93504
fibroblast 331	0.7514087	0.93670
fetal renal pelvis 231	0.7529309	0.93912
fetal stomach 251	0.7204378	0.94058
fetal kidney 100	0.7361832	0.94136
fetal renal cortex 213	0.7406458	0.94206
fetal small intestine 73	0.7093948	0.94240
fetal renal cortex 206	0.7449275	0.94300
CD19 5	0.6202978	0.94390
fetal large intestine 65	0.6916852	0.94622
fetal kidney 87	0.7325079	0.94632
fetal kidney 91	0.7426208	0.94640
fetal large intestine 61	0.6441753	0.94698
fetal fibroblast 278	0.7490125	0.94816
melanocyte 339	0.7762426	0.95116
fetal lung 299	0.7081058	0.95304
fetal renal pelvis 216	0.7176037	0.95322
breast 1	0.7474651	0.95432
fetal adrenal gland 28	0.7406589	0.95438
placenta 196	0.6838275	0.95476
CD3 10	0.6109921	0.95494
CD8 22	0.5999470	0.95656
fetal large intestine 57	0.6943421	0.95674
CD3 11	0.6152157	0.95766
placenta 201	0.6651819	0.95966
CD8 20	0.6041882	0.96030
fetal lung 298	0.6896059	0.96200
fetal fibroblast 282	0.7417292	0.96226
fetal lung 301	0.6936393	0.96332
fetal lung 300	0.6990854	0.96430
Mobilized CD3 320	0.5490282	0.96746
fetal skin 234	0.6847758	0.97068
CD3 9	0.5559619	0.97096
fetal fibroblast 281	0.7144617	0.97366
fetal fibroblast 268	0.7147574	0.97530
ES derived MSC 290	0.6507463	0.98060
fetal fibroblast 267	0.6861392	0.98186
ES derived MSC 289	0.6439057	0.98238
keratinocyte 336	0.7046761	0.98418
keratinocyte 334	0.6980865	0.98722
keratinocyte 335	0.6838519	0.98884
keratinocyte 333	0.6927930	0.98906

phospho− and sphingolipid concentrations

DHS sample	fold enrichment	p value
pancreas 328	1.2050674	0.11360
fetal adrenal gland 25	1.1699663	0.14526
CD56 18	1.2058548	0.15456
pancreas 327	1.1511355	0.19072
ES cells 294	1.1324289	0.21750
melanocyte 340	1.0916809	0.23204
fetal small intestine 69	1.1100979	0.24940
fetal thymus 258	1.1105829	0.25654
fetal adrenal gland 26	1.0896859	0.26602
ES cells 296	1.1021811	0.26856
placenta 198	1.0836513	0.27376
fetal adrenal gland 23	1.0768640	0.28632
fetal thymus 260	1.0942849	0.29150
melanocyte 339	1.0643392	0.29158
fetal adrenal gland 24	1.0869128	0.29240
fetal large intestine 60	1.0797530	0.29422
ES derived mesoderm 286	1.0591729	0.29976
fetal small intestine 72	1.0778874	0.30290
fetal small intestine 76	1.0817165	0.30292
fetal large intestine 62	1.0813212	0.30460
fetal large intestine 63	1.0798897	0.30624
CD4 13	1.0904403	0.31718
fetal large intestine 61	1.0838983	0.31842
placenta 200	1.0607129	0.32202
fetal small intestine 71	1.0684697	0.32432
fetal adrenal gland 28	1.0548370	0.33184
stomach 284	1.0662464	0.33396
fetal stomach 244	1.0663053	0.33532
small intestine 342	1.0629269	0.33534
fetal ovary 195	1.0689635	0.34230
muscle 341	1.0631736	0.34442
fetal large intestine 57	1.0528054	0.34684
fetal small intestine 78	1.0517240	0.35090
fetal large intestine 59	1.0568849	0.35198
fetal small intestine 74	1.0539924	0.35298
fetal large intestine 54	1.0553926	0.35572
fetal small intestine 70	1.0479085	0.35714
iPS 304	1.0455278	0.35724
fetal stomach 248	1.0574631	0.35790
CD4 15	1.0721257	0.35878
fetal small intestine 73	1.0509588	0.36260
fetal stomach 242	1.0613699	0.36440
fetal large intestine 56	1.0487579	0.36440
Mobilized CD4 323	1.0603839	0.36628
fetal thymus 256	1.0517103	0.36988
CD56 17	1.0527519	0.37078
fetal thymus 265	1.0493941	0.37950
fetal large intestine 64	1.0407524	0.38200
fetal large intestine 58	1.0391025	0.38498
ES derived trophoblast 288	1.0363747	0.38618
Mobilized CD3 320	1.0526497	0.38650
CD8 19	1.0542626	0.38652
CD8 21	1.0504797	0.38856
fetal testes 255	1.0348489	0.39240
fetal adrenal gland 27	1.0371765	0.39286
fetal thymus 257	1.0445896	0.39346
fetal thymus 259	1.0414206	0.40038
placenta 197	1.0331283	0.40414
CD20 8	1.0322555	0.40690
melanocyte 338	1.0253590	0.40818
melanocyte 337	1.0243406	0.40900
CD3 11	1.0345109	0.40934
fetal large intestine 65	1.0316765	0.40956
CD3 9	1.0332497	0.41684
placenta 199	1.0261283	0.41808
ES derived mesoderm 285	1.0223898	0.41858
fetal testes 254	1.0254101	0.41892
fetal stomach 247	1.0257870	0.42924
CD14 4	1.0241705	0.43210
fetal small intestine 79	1.0220020	0.43258
fetal renal pelvis 219	1.0224364	0.43386
Mobilized CD4 322	1.0211617	0.44102
fetal thymus 262	1.0194986	0.44228
Mobilized CD3 321	1.0187421	0.44322
CD8 22	1.0180674	0.44404
CD4 14	1.0192708	0.44606
fetal stomach 241	1.0153745	0.44782
heart 297	1.0137597	0.45330
fetal stomach 253	1.0115543	0.45488
fetal kidney 83	1.0109454	0.45532
CD14 3	1.0133646	0.45548
fetal large intestine 66	1.0107487	0.45634
placenta 201	1.0110923	0.45834
fetal stomach 246	1.0064935	0.46634
CD4 16	1.0051512	0.46688
CD8 20	1.0066407	0.47144
hematopoietic stem cell 315	1.0058988	0.47308
fetal muscle 151	1.0047069	0.47630
CD3 12	0.9992329	0.48150
fetal thymus 261	0.9975690	0.48886
fetal thymus 266	0.9993624	0.49062
hematopoietic stem cell 314	0.9997135	0.49148
fetal stomach 240	0.9950636	0.49172
fetal small intestine 80	1.0002048	0.49196
ES cells 293	0.9956893	0.49432
fetal small intestine 68	0.9988238	0.49496
fetal thymus 264	0.9952919	0.49898
Mobilized CD56 324	0.9948242	0.49980
fetal large intestine 55	0.9935672	0.50708
ES cells 295	0.9881099	0.51156
Mobilized CD8 325	0.9830545	0.51370
fetal stomach 243	0.9892351	0.51476
fetal large intestine 53	0.9842045	0.51864
fetal muscle 150	0.9892052	0.51880
iPS 303	0.9899866	0.52008
fetal heart 45	0.9879699	0.52022
fetal small intestine 77	0.9860592	0.52062
fetal renal pelvis 228	0.9838528	0.52158
fetal muscle 148	0.9834236	0.53240
fetal small intestine 75	0.9584941	0.53368
fetal muscle 164	0.9828647	0.53692
fetal large intestine 67	0.9799510	0.53950
breast 2	0.9839796	0.54324
breast 1	0.9842116	0.54572
fetal stomach 252	0.9721451	0.54934
stomach 283	0.9724743	0.55150
fetal stomach 245	0.9708065	0.55176
hematopoietic stem cell 305	0.9769421	0.55206
fetal muscle 169	0.9771415	0.55362
fetal muscle 173	0.9738908	0.55806
hematopoietic stem cell 316	0.9708652	0.56014
hematopoietic stem cell 309	0.9745184	0.56140
CD19 7	0.9585225	0.56320
fetal muscle 193	0.9702189	0.56454
fetal renal pelvis 218	0.9585732	0.56620
fetal stomach 251	0.9657043	0.56646
CD3 10	0.9605515	0.56790
fetal stomach 249	0.9605670	0.56882
hematopoietic stem cell 312	0.9717087	0.57022
fetal stomach 250	0.9595942	0.57092
fetal muscle 153	0.9668764	0.57346
ovary 326	0.9570524	0.57582
hematopoietic stem cell 318	0.9622990	0.58150
placenta 196	0.9615446	0.58318
fetal renal pelvis 220	0.9609740	0.58328
fetal renal pelvis 217	0.9592139	0.58430
fetal muscle 189	0.9629280	0.58806
fetal heart 49	0.9624672	0.58808
hematopoietic stem cell 308	0.9637958	0.59158
hematopoietic stem cell 311	0.9603317	0.59328
fetal muscle 175	0.9616515	0.59706
fetal muscle 178	0.9587236	0.59776
fetal muscle 167	0.9629286	0.59902
fibroblast 330	0.9627306	0.60474
fetal thymus 263	0.9552028	0.60484
CD19 6	0.9386209	0.60752
fetal muscle 160	0.9567708	0.60780
ES derived NP 292	0.9444827	0.61492
fetal muscle 180	0.9528916	0.61528
fetal kidney 86	0.9352146	0.61630
fetal renal pelvis 233	0.9482612	0.61722
fetal heart 46	0.9493900	0.62154
fetal renal pelvis 232	0.9419397	0.62354
fetal renal pelvis 226	0.9473708	0.62394
hematopoietic stem cell 306	0.9483709	0.62456
fetal muscle 190	0.9450314	0.62496
fetal kidney 87	0.9464560	0.62598
fetal muscle 185	0.9474401	0.62612
fetal heart 44	0.9367316	0.62780
fetal muscle 168	0.9482486	0.62830
fetal muscle 156	0.9484236	0.63236
fetal muscle 171	0.9495719	0.63282
fetal muscle 149	0.9442240	0.63646
fetal muscle 147	0.9445240	0.63700
fetal muscle 177	0.9397649	0.63752
fibroblast 329	0.9518458	0.63962
fetal muscle 145	0.9349963	0.64174
fetal muscle 163	0.9420507	0.64350
hematopoietic stem cell 319	0.9344883	0.64458
fetal renal cortex 211	0.9326801	0.64468
iPS 302	0.9431250	0.64552
fetal heart 51	0.9420614	0.64752
fetal muscle 187	0.9456033	0.64798
hematopoietic stem cell 307	0.9391054	0.65040
fetal muscle 183	0.9433744	0.65214
fetal muscle 191	0.9382279	0.65240
fetal muscle 152	0.9427336	0.65302
hematopoietic stem cell 310	0.9476068	0.65362
fibroblast 332	0.9481722	0.65432
fetal muscle 181	0.9415646	0.65516
ES derived trophoblast 287	0.9396668	0.65532
fetal muscle 144	0.9341143	0.65714
fetal kidney 82	0.9240141	0.65730
fetal renal pelvis 230	0.9259312	0.65944
fetal kidney 104	0.9308435	0.65946
keratinocyte 335	0.9535405	0.65952
fetal kidney 103	0.9283770	0.66010
fetal muscle 174	0.9353118	0.66044
fetal muscle 154	0.9296674	0.66600
fetal muscle 184	0.9404519	0.66648
fetal heart 50	0.9316752	0.67176
keratinocyte 336	0.9500655	0.67270
keratinocyte 333	0.9500968	0.67276
fetal muscle 166	0.9329991	0.67686
fetal renal cortex 205	0.9254507	0.67782
fetal muscle 155	0.9261602	0.67944
fetal heart 42	0.9281452	0.68100
fetal muscle 158	0.9329287	0.68110
fetal heart 47	0.9207333	0.68130
fetal muscle 159	0.9236613	0.68156
fetal renal pelvis 216	0.9205723	0.68184
fibroblast 331	0.9392753	0.68216
fetal renal cortex 214	0.9228865	0.68354
fetal heart 43	0.9308100	0.68426
CD19 5	0.9058314	0.68444
fetal kidney 102	0.9144858	0.68468
fetal renal cortex 210	0.9162455	0.68792
fetal muscle 161	0.9244904	0.68826
fetal kidney 81	0.9302294	0.68852
fetal muscle 165	0.9310478	0.68878
fetal skin 234	0.9235622	0.69368
fetal muscle 170	0.9274700	0.69380
fetal lung 127	0.9259074	0.69640
fetal muscle 146	0.9245889	0.69726
fetal renal pelvis 227	0.9148314	0.69868
hematopoietic stem cell 313	0.9230922	0.69870
fetal lung 301	0.9297588	0.69876
fetal muscle 192	0.9250921	0.69924
fetal lung 112	0.9301732	0.69930
fetal muscle 182	0.9252047	0.69942
fetal kidney 106	0.9164175	0.70200
fetal kidney 99	0.9146038	0.70248
fetal renal cortex 207	0.9122498	0.71024
fetal kidney 94	0.9038639	0.71222
fetal lung 299	0.9225946	0.71294
fetal muscle 179	0.9197036	0.71344
fetal kidney 91	0.9121590	0.71724
fetal kidney 96	0.9094689	0.72324
fetal kidney 84	0.8996276	0.72432
fetal renal pelvis 223	0.8978419	0.72474
fetal kidney 89	0.9025780	0.72630
fetal kidney 95	0.9031952	0.72812
fetal heart 52	0.9160692	0.72920
ES derived NP 291	0.8871199	0.72986
fetal lung 300	0.9178038	0.73176
fetal muscle 188	0.9118644	0.73480
keratinocyte 334	0.9302074	0.73580
fetal brain 37	0.9269397	0.73686
fetal kidney 100	0.8992655	0.73876
fetal renal pelvis 225	0.8924485	0.74126
fetal lung 129	0.9119353	0.74428
fetal muscle 186	0.9067164	0.74476
fetal heart 48	0.9017706	0.74640
fetal muscle 157	0.9131919	0.74666
ES derived MSC 290	0.9028060	0.74722
fetal lung 298	0.9083812	0.74992
fetal renal cortex 202	0.8968537	0.75034
fetal lung 134	0.9048787	0.75162
fetal kidney 88	0.8980187	0.75242
fetal muscle 162	0.9031104	0.75526
fetal kidney 107	0.8911576	0.75618
ES derived MSC 289	0.8994509	0.75650
fetal lung 136	0.9035914	0.75822
fetal renal cortex 209	0.8865800	0.75974
fetal lung 108	0.8979143	0.76012
fetal kidney 98	0.8839794	0.76416
fetal renal pelvis 231	0.8937070	0.76558
fetal spinal cord 237	0.9019094	0.76576
fetal renal cortex 212	0.8917872	0.76712
fetal renal pelvis 222	0.8827245	0.76766
fetal kidney 105	0.8895226	0.76864
fetal kidney 92	0.8887799	0.76930
fetal kidney 97	0.8844926	0.76980
fetal renal cortex 208	0.8813311	0.76994
fetal renal cortex 206	0.8888317	0.77086
fetal renal pelvis 221	0.8893259	0.77092
fetal renal cortex 203	0.8823856	0.77194
fetal muscle 143	0.9028937	0.77356
fetal renal pelvis 229	0.8849259	0.77358
fetal lung 132	0.8993810	0.77468
fetal lung 125	0.8928247	0.77926
fetal kidney 93	0.8724898	0.78006
fetal muscle 176	0.8974178	0.78322
fetal kidney 90	0.8737248	0.78850
fetal brain 35	0.9183855	0.79304
fetal lung 123	0.8862665	0.79762
fetal renal pelvis 224	0.8783379	0.80052
fetal lung 115	0.8925079	0.80132
fetal lung 131	0.8981943	0.80582
fetal lung 109	0.8970840	0.80956
fetal lung 135	0.8796206	0.81318
fetal brain 41	0.8993428	0.81336
fetal lung 116	0.8936341	0.81340
fetal lung 128	0.8950899	0.81480
fetal spinal cord 239	0.8761091	0.81560
fetal kidney 101	0.8653608	0.81616
fetal brain 36	0.8929873	0.81884
fetal renal cortex 204	0.8708090	0.81942
fetal lung 122	0.8688353	0.82014
fetal spinal cord 236	0.8650124	0.82124
hematopoietic stem cell 317	0.8790710	0.82196
fetal lung 110	0.8721115	0.82710
fetal renal cortex 215	0.8647202	0.82758
fetal lung 133	0.8813033	0.82872
fetal renal cortex 213	0.8644075	0.82932
fetal lung 121	0.8816557	0.83158
fetal kidney 85	0.8704707	0.83310
fetal lung 140	0.8791817	0.83332
fetal lung 142	0.8797446	0.83676
fetal lung 139	0.8601926	0.84052
fetal lung 118	0.8826483	0.84232
fetal lung 130	0.8688457	0.84324
fetal brain 39	0.8914167	0.84326
fetal lung 117	0.8722886	0.84362
fetal muscle 194	0.8683121	0.85070
fetal muscle 172	0.8715064	0.85116
fetal brain 34	0.8909433	0.85308
fetal lung 126	0.8702014	0.85802
fetal lung 138	0.8569926	0.85816
fetal brain 31	0.8976449	0.86108
fetal spinal cord 238	0.8530155	0.86178
fetal fibroblast 279	0.8709645	0.86222
fetal lung 137	0.8586764	0.86566
fetal brain 38	0.8659191	0.87030
fetal fibroblast 281	0.8697296	0.87194
fetal brain 30	0.8972968	0.87284
fetal lung 120	0.8528916	0.87462
fetal lung 124	0.8463982	0.87912
fetal lung 111	0.8543638	0.88206
fetal fibroblast 269	0.8639743	0.88418
fetal fibroblast 278	0.8552032	0.88608
fetal brain 29	0.8851674	0.88932
fetal spinal cord 235	0.8343748	0.89230
fetal fibroblast 280	0.8565708	0.89340
fetal fibroblast 276	0.8552847	0.89946
fetal fibroblast 273	0.8523935	0.89982
fetal lung 141	0.8453575	0.90484
fetal lung 113	0.8363968	0.91136
fetal lung 114	0.8445994	0.91228
fetal lung 119	0.8377079	0.91252
fetal fibroblast 275	0.8444328	0.91312
fetal fibroblast 274	0.8382739	0.92104
fetal fibroblast 272	0.8369978	0.92198
fetal fibroblast 277	0.8303311	0.92324
fetal fibroblast 267	0.8290419	0.92410
fetal brain 40	0.8467602	0.92492
fetal fibroblast 271	0.8340338	0.92646
fetal fibroblast 282	0.8383071	0.92766
fetal fibroblast 270	0.8214626	0.93902
fetal brain 32	0.8324059	0.93948
fetal fibroblast 268	0.8199758	0.94472
fetal brain 33	0.7980471	0.95410

C−reactive protein levels

DHS sample	fold enrichment	p value
fetal large intestine 58	1.1668543	0.14562
fetal large intestine 60	1.1719702	0.16038
fetal small intestine 74	1.1652034	0.16566
fetal large intestine 64	1.1432931	0.19230
CD14 4	1.1632554	0.19360
CD4 13	1.1824243	0.19562
small intestine 342	1.1414079	0.19730
CD20 8	1.1356099	0.20038
fetal small intestine 70	1.1277026	0.20394
fetal small intestine 78	1.1254976	0.20700
fetal large intestine 62	1.1401407	0.21770
fetal large intestine 67	1.1341712	0.22068
fetal small intestine 80	1.1285092	0.22192
CD4 14	1.1365778	0.23642
fetal small intestine 68	1.0944391	0.27306
fetal large intestine 55	1.0966621	0.27338
fetal large intestine 56	1.1011841	0.27380
fetal large intestine 54	1.0984529	0.27724
stomach 284	1.1025635	0.28252
fetal small intestine 72	1.0910775	0.29086
fetal small intestine 79	1.0931356	0.29592
fetal large intestine 57	1.0803111	0.30078
fetal small intestine 73	1.0863158	0.30342
CD3 9	1.0908036	0.32096
CD8 21	1.0919472	0.32180
fetal small intestine 77	1.0730190	0.33092
fetal large intestine 63	1.0666962	0.34178
fetal large intestine 65	1.0648006	0.34594
fetal small intestine 76	1.0578121	0.36244
CD14 3	1.0587650	0.37562
fetal small intestine 69	1.0513920	0.37562
fetal large intestine 59	1.0474795	0.38416
Mobilized CD4 322	1.0481996	0.39472
keratinocyte 335	1.0253889	0.41428
keratinocyte 334	1.0238604	0.41940
CD56 18	1.0343969	0.42276
CD3 11	1.0288624	0.42826
Mobilized CD4 323	1.0224174	0.44198
fetal small intestine 71	1.0182721	0.44204
Mobilized CD56 324	1.0153694	0.45740
fetal large intestine 66	1.0109163	0.46130
CD56 17	1.0138753	0.46228
CD4 16	1.0030805	0.47046
Mobilized CD8 325	1.0036500	0.47100
fetal lung 301	1.0077594	0.47178
Mobilized CD3 321	1.0025271	0.47332
breast 1	1.0067394	0.47338
CD8 19	1.0020917	0.47440
Mobilized CD3 320	1.0019591	0.47972
fetal lung 300	1.0028815	0.48666
fetal thymus 256	0.9994157	0.49036
CD8 20	0.9972131	0.49384
keratinocyte 333	0.9995691	0.49664
fetal adrenal gland 28	0.9977011	0.49730
fetal large intestine 53	0.9905240	0.50056
keratinocyte 336	0.9952492	0.50900
CD3 12	0.9836239	0.50966
breast 2	0.9863159	0.53360
ES derived MSC 290	0.9757039	0.55108
fetal fibroblast 269	0.9747774	0.56694
fetal fibroblast 281	0.9722554	0.57056
stomach 283	0.9573958	0.57368
fetal adrenal gland 23	0.9638593	0.57792
fetal thymus 258	0.9545909	0.58018
hematopoietic stem cell 310	0.9686550	0.58190
fetal adrenal gland 25	0.9581322	0.58462
fetal stomach 244	0.9521687	0.58684
pancreas 328	0.9525020	0.59202
CD8 22	0.9324057	0.60698
fetal fibroblast 270	0.9576607	0.60816
CD4 15	0.9111467	0.62084
fetal thymus 264	0.9346438	0.62778
fetal fibroblast 279	0.9456970	0.64354
fetal adrenal gland 24	0.9272826	0.64618
fetal thymus 259	0.9172619	0.64932
hematopoietic stem cell 314	0.9389942	0.65238
ES derived MSC 289	0.9342484	0.65312
pancreas 327	0.9175246	0.65828
fetal adrenal gland 27	0.9271929	0.65922
muscle 341	0.9172035	0.66366
fetal stomach 246	0.9079590	0.66464
fetal kidney 103	0.9156457	0.66562
fetal stomach 240	0.9090425	0.66576
fetal lung 299	0.9322073	0.66602
iPS 304	0.9270459	0.67038
fibroblast 331	0.9364282	0.67180
fetal lung 298	0.9297411	0.67480
fetal kidney 91	0.9158426	0.67874
fetal thymus 260	0.9040778	0.68044
fetal thymus 265	0.9065377	0.68052
fetal thymus 266	0.9062879	0.68060
fetal fibroblast 267	0.9281563	0.68248
fetal fibroblast 274	0.9291138	0.68662
fetal kidney 85	0.9155202	0.69004
fetal stomach 242	0.8814645	0.69160
fetal fibroblast 278	0.9227969	0.69266
ES derived trophoblast 288	0.9195587	0.69366
fetal renal cortex 207	0.9032374	0.70060
hematopoietic stem cell 309	0.9071204	0.71094
fetal heart 45	0.9038077	0.71538
fetal stomach 247	0.8736196	0.71610
fetal fibroblast 273	0.9155792	0.71880
ES cells 294	0.8866883	0.71940
hematopoietic stem cell 313	0.9070520	0.72006
fetal renal pelvis 226	0.8926955	0.72126
fetal stomach 241	0.8765477	0.72470
fetal stomach 252	0.8799792	0.72588
fetal kidney 95	0.8884404	0.72914
fetal adrenal gland 26	0.8944825	0.72938
fetal large intestine 61	0.8693950	0.73068
fibroblast 330	0.9088766	0.73246
fetal heart 49	0.8969958	0.73282
fetal kidney 83	0.8704272	0.73662
fetal stomach 245	0.8730949	0.73774
fetal thymus 257	0.8629090	0.73812
hematopoietic stem cell 312	0.8991802	0.73888
hematopoietic stem cell 315	0.8919453	0.73894
ES derived mesoderm 285	0.9064924	0.73908
fetal fibroblast 277	0.9046694	0.73934
fetal stomach 253	0.8686208	0.74028
fetal fibroblast 280	0.9081389	0.74130
hematopoietic stem cell 305	0.8894268	0.74176
hematopoietic stem cell 316	0.8818997	0.74502
fetal kidney 87	0.8815241	0.74504
fetal kidney 84	0.8746002	0.74698
fetal kidney 106	0.8765993	0.74742
iPS 303	0.8868570	0.75172
hematopoietic stem cell 308	0.8916271	0.75408
hematopoietic stem cell 319	0.8772771	0.75478
hematopoietic stem cell 311	0.8869412	0.75518
hematopoietic stem cell 306	0.8851780	0.75608
hematopoietic stem cell 317	0.8981588	0.75624
fetal thymus 261	0.8610235	0.75894
fetal fibroblast 268	0.8993620	0.75934
fetal skin 234	0.8790624	0.76182
fetal renal pelvis 220	0.8680331	0.76274
fibroblast 332	0.9009160	0.76354
fetal stomach 243	0.8670694	0.76924
hematopoietic stem cell 307	0.8777678	0.76934
fetal renal pelvis 228	0.8554581	0.77432
fetal stomach 250	0.8443878	0.77780
fetal fibroblast 282	0.8899259	0.78492
fetal renal pelvis 231	0.8612525	0.78592
fetal kidney 89	0.8530675	0.78662
fibroblast 329	0.8866048	0.78898
fetal spinal cord 236	0.8667906	0.78916
fetal renal pelvis 219	0.8287058	0.79262
melanocyte 338	0.8919394	0.79424
ES derived NP 291	0.8389236	0.79824
fetal fibroblast 272	0.8799753	0.79890
fetal stomach 251	0.8426115	0.79960
fetal ovary 195	0.8259376	0.79996
fetal kidney 107	0.8461561	0.80236
fetal renal cortex 211	0.8365211	0.80290
fetal renal pelvis 217	0.8409702	0.80356
CD3 10	0.8299637	0.80508
fetal fibroblast 276	0.8796427	0.80586
fetal stomach 249	0.8212783	0.81090
fetal renal cortex 213	0.8517647	0.81110
fetal fibroblast 271	0.8743461	0.81148
fetal renal pelvis 230	0.8262817	0.81224
placenta 201	0.8391606	0.81454
ES derived mesoderm 286	0.8804259	0.81512
fetal renal pelvis 218	0.8064992	0.81820
fetal fibroblast 275	0.8724967	0.81928
fetal kidney 99	0.8387528	0.82134
fetal kidney 82	0.8218595	0.82212
hematopoietic stem cell 318	0.8397253	0.82246
fetal thymus 262	0.7847318	0.82362
fetal kidney 100	0.8335313	0.82424
fetal renal cortex 205	0.8390285	0.82446
fetal spinal cord 235	0.8544534	0.82782
ES derived trophoblast 287	0.8420959	0.82838
iPS 302	0.8469127	0.82900
fetal renal cortex 214	0.8303448	0.83464
CD19 7	0.7918455	0.83486
fetal kidney 96	0.8310800	0.83560
fetal renal pelvis 216	0.8211835	0.83586
fetal kidney 92	0.8334219	0.83676
fetal heart 47	0.8290710	0.83792
fetal kidney 86	0.7957216	0.83856
heart 297	0.8267647	0.84028
fetal kidney 94	0.8123604	0.84176
CD19 6	0.7902541	0.84198
fetal small intestine 75	0.7482056	0.84244
fetal renal pelvis 232	0.8141716	0.84346
fetal kidney 97	0.8194309	0.84626
ovary 326	0.7930265	0.84648
placenta 200	0.8464488	0.84728
fetal renal pelvis 223	0.8082071	0.84892
fetal stomach 248	0.8029311	0.84998
CD19 5	0.7915181	0.85072
fetal renal cortex 212	0.8206975	0.85610
fetal muscle 172	0.8419515	0.85696
ES cells 296	0.8017778	0.85722
fetal kidney 102	0.7982753	0.85778
fetal kidney 101	0.8108042	0.86204
fetal renal cortex 215	0.8191777	0.86206
fetal renal cortex 202	0.8126824	0.86248
fetal heart 46	0.8209379	0.86326
fetal renal pelvis 233	0.8082773	0.86604
fetal kidney 104	0.8011207	0.86614
ES derived NP 292	0.7969143	0.86692
fetal testes 254	0.8065723	0.87126
fetal kidney 98	0.7962118	0.87198
fetal renal pelvis 225	0.7931218	0.87542
fetal renal pelvis 227	0.7980667	0.87554
fetal renal cortex 206	0.8044906	0.87652
fetal heart 44	0.7840557	0.87704
fetal heart 42	0.8135401	0.87742
fetal kidney 93	0.7894992	0.87750
fetal heart 43	0.8221142	0.87956
fetal testes 255	0.8078645	0.88476
fetal kidney 105	0.7965695	0.88640
fetal renal cortex 210	0.7799609	0.88812
fetal lung 112	0.8134816	0.88918
fetal renal cortex 204	0.8013812	0.88924
ES cells 293	0.7856860	0.89066
ES cells 295	0.7704818	0.89436
fetal renal pelvis 221	0.7909740	0.89454
fetal kidney 90	0.7801551	0.89488
fetal kidney 88	0.7898227	0.89580
fetal renal pelvis 222	0.7745321	0.89842
placenta 196	0.7846173	0.89934
placenta 197	0.7808608	0.90166
fetal renal pelvis 224	0.7857111	0.90372
fetal heart 50	0.7917636	0.90442
fetal heart 51	0.7936103	0.90450
fetal lung 125	0.7964689	0.90458
fetal renal cortex 209	0.7692483	0.90844
fetal heart 48	0.7912436	0.90860
fetal renal pelvis 229	0.7747517	0.91034
fetal muscle 175	0.7795834	0.91254
placenta 199	0.7788723	0.91360
fetal lung 123	0.7899131	0.91396
fetal spinal cord 238	0.7906800	0.91578
placenta 198	0.7845269	0.91850
fetal lung 116	0.8026213	0.91912
fetal spinal cord 239	0.7859167	0.92006
fetal lung 138	0.7838114	0.92254
fetal renal cortex 208	0.7516682	0.92268
fetal brain 37	0.7970203	0.92416
melanocyte 337	0.8166301	0.92486
fetal lung 128	0.8034269	0.92606
fetal heart 52	0.7806968	0.92880
fetal lung 134	0.7717439	0.93270
fetal renal cortex 203	0.7370886	0.93606
fetal lung 109	0.7926809	0.93654
fetal muscle 193	0.7405087	0.94076
fetal spinal cord 237	0.7601759	0.94120
fetal lung 108	0.7547711	0.94136
fetal thymus 263	0.7427687	0.94226
fetal muscle 173	0.7408065	0.94252
fetal muscle 159	0.7323397	0.94340
fetal lung 127	0.7536147	0.94558
fetal muscle 154	0.7223853	0.94678
fetal muscle 168	0.7314957	0.94704
fetal muscle 185	0.7257888	0.94754
fetal muscle 149	0.7314765	0.94772
melanocyte 339	0.7858780	0.94880
fetal lung 139	0.7463529	0.95016
fetal muscle 160	0.7307638	0.95034
fetal muscle 165	0.7482566	0.95036
fetal muscle 153	0.7219817	0.95036
fetal lung 110	0.7475277	0.95052
fetal muscle 145	0.7049318	0.95242
fetal lung 142	0.7654089	0.95258
fetal muscle 144	0.7159283	0.95384
fetal lung 131	0.7717345	0.95396
fetal muscle 190	0.7097845	0.95418
fetal lung 115	0.7574689	0.95500
fetal muscle 166	0.7325288	0.95520
fetal lung 135	0.7404958	0.95544
fetal muscle 150	0.7179113	0.95546
fetal muscle 179	0.7280706	0.95548
fetal muscle 164	0.7302590	0.95558
fetal lung 136	0.7443581	0.95632
fetal muscle 143	0.7465469	0.95806
fetal lung 129	0.7449329	0.95894
melanocyte 340	0.7635134	0.95900
fetal lung 118	0.7608682	0.95908
fetal muscle 146	0.7286409	0.95942
fetal brain 40	0.7686524	0.95958
fetal kidney 81	0.7295906	0.96036
fetal muscle 151	0.7084646	0.96044
fetal muscle 174	0.7140240	0.96048
fetal muscle 192	0.7281919	0.96096
fetal lung 124	0.7376587	0.96132
fetal muscle 191	0.7104370	0.96250
fetal lung 132	0.7368471	0.96290
fetal brain 31	0.7882297	0.96292
fetal lung 121	0.7484885	0.96292
fetal muscle 194	0.7392614	0.96318
fetal muscle 157	0.7332973	0.96472
fetal brain 38	0.7453343	0.96552
fetal lung 126	0.7452444	0.96586
fetal lung 122	0.7162707	0.96590
fetal lung 130	0.7328603	0.96622
fetal muscle 178	0.6995328	0.96694
fetal muscle 169	0.7092563	0.96702
fetal muscle 182	0.7113633	0.96710
fetal lung 113	0.7450358	0.96774
fetal lung 137	0.7337549	0.96826
fetal lung 141	0.7428826	0.96836
fetal muscle 189	0.7081407	0.96882
fetal muscle 183	0.7114052	0.97006
fetal muscle 187	0.7122877	0.97036
fetal muscle 167	0.7108865	0.97062
fetal muscle 161	0.6906110	0.97090
fetal muscle 171	0.7135675	0.97130
fetal muscle 188	0.7070526	0.97130
fetal muscle 152	0.7065134	0.97198
fetal muscle 148	0.6966261	0.97214
fetal lung 120	0.7210216	0.97234
fetal muscle 186	0.7015575	0.97336
fetal muscle 180	0.6802733	0.97354
fetal muscle 158	0.7032377	0.97370
fetal lung 133	0.7261053	0.97480
fetal muscle 162	0.7023876	0.97496
fetal brain 41	0.7303375	0.97626
fetal muscle 156	0.6933218	0.97654
fetal muscle 163	0.6804891	0.97704
fetal muscle 147	0.6834315	0.97706
fetal muscle 177	0.6667454	0.97756
fetal brain 34	0.7433053	0.97840
fetal muscle 170	0.6929565	0.97842
fetal muscle 181	0.6773548	0.97994
fetal lung 114	0.7208917	0.98028
fetal muscle 155	0.6576130	0.98056
fetal lung 111	0.7050645	0.98062
fetal muscle 184	0.6951308	0.98090
fetal lung 117	0.7075277	0.98146
fetal brain 39	0.7240435	0.98172
fetal brain 32	0.7293052	0.98218
fetal lung 140	0.7001917	0.98364
fetal lung 119	0.7016736	0.98444
fetal brain 30	0.7420164	0.98872
fetal brain 36	0.7043997	0.98888
fetal brain 29	0.7322528	0.98920
fetal brain 33	0.6824284	0.98982
fetal brain 35	0.7169020	0.99092
fetal muscle 176	0.6574041	0.99144

Cytokine responses to smallpox vaccine

DHS sample	fold enrichment	p value
fetal muscle 181	1.1581440	0.15926
fetal thymus 256	1.1493066	0.22134
fetal muscle 151	1.1100434	0.25106
fetal heart 47	1.1091726	0.25314
fetal muscle 179	1.0831527	0.29210
fetal thymus 258	1.1059516	0.29486
fetal muscle 153	1.0874242	0.29714
fetal muscle 160	1.0802732	0.30120
fetal muscle 180	1.0787265	0.30800
fetal muscle 182	1.0683681	0.32104
fetal thymus 264	1.0848176	0.32636
fetal muscle 169	1.0632046	0.33476
fetal muscle 149	1.0663525	0.33532
fetal muscle 167	1.0546459	0.35072
fetal muscle 176	1.0521027	0.35388
fetal muscle 148	1.0517804	0.36226
fetal muscle 168	1.0435150	0.38218
CD56 18	1.0604768	0.38340
heart 297	1.0465946	0.38712
fetal muscle 164	1.0376370	0.39570
fetal fibroblast 280	1.0314367	0.39934
fetal muscle 166	1.0323223	0.40548
Mobilized CD56 324	1.0411063	0.40790
fetal muscle 150	1.0311649	0.41488
fetal muscle 152	1.0274554	0.41922
CD8 21	1.0348791	0.41948
fetal fibroblast 270	1.0199278	0.43314
CD3 9	1.0275845	0.43334
fetal muscle 143	1.0199457	0.43482
fetal heart 49	1.0209267	0.43526
fetal fibroblast 279	1.0197748	0.43614
fetal muscle 193	1.0195070	0.44106
fetal fibroblast 269	1.0166059	0.44232
fetal heart 45	1.0185528	0.44526
fetal muscle 178	1.0169060	0.44540
fetal thymus 260	1.0169659	0.45148
fetal heart 42	1.0134678	0.45370
Mobilized CD4 323	1.0135990	0.45488
fetal heart 52	1.0129339	0.45504
fetal fibroblast 273	1.0118203	0.45554
CD3 11	1.0134184	0.45952
CD4 13	1.0107945	0.45956
fetal fibroblast 282	1.0105548	0.45968
fetal muscle 157	1.0101011	0.46046
fetal muscle 165	1.0097634	0.46322
fetal lung 298	1.0114086	0.46348
Mobilized CD3 320	1.0123619	0.46374
fetal muscle 155	1.0070894	0.46876
fetal heart 43	1.0060683	0.47110
fetal muscle 175	1.0067537	0.47214
fetal lung 300	1.0061962	0.47260
fetal muscle 158	1.0058369	0.47296
fetal muscle 187	1.0044259	0.47554
hematopoietic stem cell 314	1.0058508	0.47580
fetal muscle 183	1.0031080	0.48082
melanocyte 340	1.0038152	0.48218
fetal thymus 265	1.0006538	0.48236
fetal heart 46	1.0013696	0.48348
fetal muscle 171	1.0010426	0.48478
fibroblast 330	1.0022203	0.48546
hematopoietic stem cell 309	0.9985842	0.49034
fetal muscle 154	0.9975361	0.49150
fetal muscle 185	0.9968513	0.49290
melanocyte 337	1.0003256	0.49338
muscle 341	0.9903338	0.50104
Mobilized CD4 322	0.9836466	0.50210
fetal thymus 259	0.9870583	0.50606
fetal thymus 266	0.9828465	0.51684
fetal muscle 172	0.9886058	0.52242
fetal fibroblast 276	0.9908525	0.52274
CD8 19	0.9642901	0.53354
fetal large intestine 61	0.9708381	0.53404
fetal heart 48	0.9814267	0.53426
hematopoietic stem cell 318	0.9759631	0.53576
fetal fibroblast 271	0.9859708	0.53624
CD3 12	0.9637923	0.53690
CD3 10	0.9692218	0.53758
fetal muscle 173	0.9794188	0.53824
fetal lung 301	0.9815288	0.54056
CD4 14	0.9695831	0.54080
fetal fibroblast 272	0.9824237	0.54540
fetal muscle 159	0.9737422	0.54544
CD56 17	0.9677392	0.54736
hematopoietic stem cell 310	0.9764065	0.55348
fetal muscle 184	0.9747983	0.55700
fibroblast 329	0.9752408	0.55866
fetal heart 51	0.9711578	0.56056
melanocyte 338	0.9778141	0.56806
fetal fibroblast 275	0.9750547	0.57052
fetal muscle 186	0.9676112	0.57316
hematopoietic stem cell 305	0.9610006	0.57354
fetal muscle 146	0.9649945	0.57580
fetal fibroblast 277	0.9695802	0.57700
fetal muscle 192	0.9649579	0.57900
hematopoietic stem cell 312	0.9613988	0.57922
fetal brain 37	0.9740812	0.58248
fetal muscle 174	0.9613574	0.58256
fetal muscle 189	0.9619747	0.58370
fetal fibroblast 281	0.9695248	0.58640
fibroblast 331	0.9643549	0.58642
fetal lung 299	0.9586359	0.59574
fetal muscle 191	0.9530012	0.60248
hematopoietic stem cell 316	0.9434488	0.60624
fetal thymus 263	0.9505365	0.60696
fetal muscle 147	0.9510007	0.60864
fetal brain 35	0.9695357	0.60944
fetal fibroblast 267	0.9581615	0.61114
Mobilized CD3 321	0.9117410	0.61444
fetal muscle 156	0.9489051	0.61620
fetal muscle 190	0.9403521	0.62132
melanocyte 339	0.9586749	0.62386
fetal kidney 81	0.9497757	0.62434
fibroblast 332	0.9502020	0.62466
CD8 20	0.9231255	0.62482
fetal brain 30	0.9707645	0.62494
Mobilized CD8 325	0.9090513	0.62612
hematopoietic stem cell 315	0.9345790	0.63508
fetal fibroblast 274	0.9511015	0.63542
fetal adrenal gland 28	0.9472558	0.63782
hematopoietic stem cell 308	0.9332861	0.64454
fetal thymus 257	0.9061275	0.64474
fetal fibroblast 278	0.9459804	0.64592
fetal thymus 261	0.9098736	0.65006
CD19 7	0.8978283	0.65210
fetal fibroblast 268	0.9455711	0.65474
ES derived MSC 289	0.9342221	0.65750
CD19 5	0.9018903	0.65932
breast 2	0.9393342	0.66272
CD19 6	0.8893141	0.66878
CD8 22	0.8831543	0.66934
fetal lung 120	0.9322031	0.67178
fetal muscle 145	0.9154371	0.67272
fetal muscle 162	0.9284396	0.67596
fetal muscle 170	0.9279744	0.67774
fetal lung 140	0.9324348	0.67902
fetal thymus 262	0.8673815	0.67942
CD14 4	0.9011713	0.68084
hematopoietic stem cell 306	0.9125505	0.68398
fetal muscle 188	0.9216530	0.68590
fetal muscle 163	0.9167348	0.68794
fetal heart 44	0.8984618	0.68828
hematopoietic stem cell 311	0.9082143	0.69286
CD4 16	0.8627971	0.69322
fetal lung 119	0.9299955	0.69430
fetal lung 111	0.9252760	0.69936
fetal testes 255	0.9159173	0.70160
fetal heart 50	0.9097534	0.70212
keratinocyte 334	0.9313390	0.70408
ES derived MSC 290	0.9116634	0.70832
ovary 326	0.8751129	0.70844
keratinocyte 335	0.9276734	0.71030
fetal muscle 194	0.9178884	0.71438
CD14 3	0.8755863	0.71540
keratinocyte 333	0.9258078	0.72188
hematopoietic stem cell 307	0.8923761	0.72310
fetal lung 141	0.9171820	0.72374
fetal muscle 177	0.8938854	0.72438
fetal adrenal gland 25	0.8865394	0.73046
hematopoietic stem cell 317	0.9023443	0.73608
fetal brain 41	0.9234940	0.73810
breast 1	0.9087708	0.73990
CD4 15	0.8066068	0.74622
fetal adrenal gland 24	0.8672080	0.75452
fetal lung 128	0.9083352	0.75512
fetal lung 116	0.9070243	0.75524
fetal small intestine 75	0.7925011	0.76010
fetal lung 121	0.9024540	0.76106
hematopoietic stem cell 313	0.8747229	0.77128
fetal lung 132	0.8879033	0.77416
fetal lung 114	0.8976961	0.77538
fetal brain 29	0.9281852	0.77688
fetal muscle 161	0.8740127	0.78268
keratinocyte 336	0.8999968	0.78390
fetal lung 133	0.8905535	0.78590
pancreas 328	0.8474132	0.78898
fetal brain 39	0.9084887	0.79552
fetal muscle 144	0.8591381	0.79586
fetal lung 118	0.8897400	0.79692
fetal lung 130	0.8770295	0.79754
fetal brain 34	0.9113339	0.79782
fetal spinal cord 235	0.8744764	0.79798
hematopoietic stem cell 319	0.8421135	0.80036
ES cells 295	0.8372590	0.80238
fetal lung 110	0.8683916	0.80294
ES derived NP 291	0.8291751	0.80744
placenta 200	0.8664062	0.80856
fetal lung 109	0.8860400	0.80874
fetal stomach 243	0.8420542	0.81008
fetal adrenal gland 27	0.8511508	0.81224
fetal stomach 248	0.8226143	0.81544
fetal small intestine 78	0.8512324	0.81828
fetal lung 138	0.8657799	0.81872
fetal lung 125	0.8610797	0.81882
fetal lung 117	0.8702357	0.81886
fetal lung 131	0.8794920	0.82084
fetal lung 137	0.8680334	0.82308
fetal lung 123	0.8600950	0.82430
fetal kidney 85	0.8624140	0.82570
fetal lung 115	0.8686358	0.82604
fetal brain 32	0.8876598	0.82664
fetal adrenal gland 23	0.8525946	0.82938
fetal large intestine 58	0.8408247	0.82984
fetal lung 134	0.8554696	0.83018
fetal adrenal gland 26	0.8407363	0.83360
fetal lung 139	0.8486539	0.83452
ES derived NP 292	0.8180911	0.83476
fetal kidney 103	0.8299731	0.83696
fetal large intestine 60	0.8190757	0.83760
fetal lung 135	0.8523856	0.83766
fetal lung 129	0.8570586	0.83806
fetal stomach 244	0.8129880	0.83830
fetal lung 112	0.8545934	0.83920
fetal small intestine 70	0.8426306	0.83952
fetal kidney 106	0.8323216	0.84650
fetal lung 126	0.8579291	0.84724
iPS 302	0.8365985	0.84740
fetal renal cortex 207	0.8261353	0.84848
fetal lung 142	0.8592935	0.84856
fetal renal cortex 214	0.8243180	0.85346
fetal stomach 246	0.7865315	0.85442
fetal stomach 253	0.7881997	0.85498
fetal testes 254	0.8218656	0.85568
fetal large intestine 55	0.8172726	0.85664
fetal kidney 92	0.8271634	0.85748
fetal stomach 251	0.7973106	0.86102
fetal renal cortex 213	0.8264873	0.86126
fetal stomach 252	0.7931449	0.86284
fetal large intestine 67	0.8004208	0.86304
fetal brain 36	0.8663736	0.86394
placenta 198	0.8305990	0.86422
ES derived mesoderm 285	0.8582611	0.86424
fetal large intestine 57	0.8171034	0.86728
fetal small intestine 74	0.8034464	0.86738
ES cells 296	0.7904686	0.86744
fetal large intestine 65	0.7944383	0.86838
fetal brain 33	0.8542647	0.86872
fetal kidney 95	0.8105644	0.86878
iPS 304	0.8332537	0.86948
placenta 199	0.8174999	0.87106
fetal small intestine 68	0.8092029	0.87132
fetal lung 108	0.8248073	0.87138
fetal large intestine 56	0.7988804	0.87140
fetal stomach 250	0.7713792	0.87152
fetal lung 113	0.8419006	0.87296
fetal lung 127	0.8228553	0.87734
fetal stomach 242	0.7371387	0.87830
fetal lung 122	0.8183560	0.87850
fetal stomach 249	0.7642025	0.88080
stomach 283	0.7739122	0.88108
fetal large intestine 62	0.7753643	0.88296
stomach 284	0.7824891	0.88446
fetal kidney 83	0.7730648	0.88502
placenta 197	0.7916350	0.88614
fetal lung 124	0.8269599	0.88646
fetal renal pelvis 219	0.7482120	0.88812
fetal skin 234	0.8093740	0.88962
fetal renal pelvis 233	0.7927118	0.89048
fetal kidney 100	0.7968152	0.89100
fetal renal pelvis 228	0.7766753	0.89100
pancreas 327	0.7637473	0.89180
fetal lung 136	0.8205428	0.89194
placenta 201	0.7778077	0.89198
fetal renal pelvis 232	0.7814030	0.89244
small intestine 342	0.7827831	0.89312
CD20 8	0.7917797	0.89366
fetal kidney 89	0.7898164	0.89398
fetal brain 40	0.8641454	0.89480
fetal small intestine 80	0.7851591	0.89588
fetal kidney 84	0.7839710	0.89646
fetal large intestine 59	0.7609223	0.89862
fetal kidney 91	0.7981991	0.90240
fetal ovary 195	0.7339639	0.90526
fetal small intestine 73	0.7649273	0.90588
fetal large intestine 54	0.7635658	0.90634
fetal small intestine 69	0.7509515	0.90648
fetal renal pelvis 221	0.7903181	0.90650
fetal brain 31	0.8754602	0.90668
fetal small intestine 79	0.7537736	0.90736
fetal large intestine 64	0.7726648	0.90800
fetal spinal cord 237	0.8149582	0.90944
fetal stomach 245	0.7465224	0.90978
fetal kidney 99	0.7800329	0.91076
fetal renal pelvis 231	0.7918389	0.91260
fetal spinal cord 239	0.8030334	0.91296
fetal large intestine 53	0.7329766	0.91342
iPS 303	0.7917401	0.91530
fetal renal cortex 202	0.7797201	0.91534
fetal renal cortex 206	0.7827453	0.91812
fetal renal pelvis 216	0.7661163	0.91834
fetal renal cortex 215	0.7837705	0.91912
placenta 196	0.7636949	0.91952
fetal small intestine 76	0.7399192	0.91966
fetal spinal cord 238	0.7975324	0.91972
ES derived trophoblast 288	0.7983238	0.92182
fetal renal pelvis 227	0.7681290	0.92194
fetal small intestine 72	0.7487355	0.92292
fetal stomach 241	0.7185948	0.92532
fetal spinal cord 236	0.7795729	0.92556
fetal large intestine 66	0.7362382	0.92608
fetal renal pelvis 226	0.7668037	0.92612
ES derived mesoderm 286	0.8233839	0.92684
ES cells 294	0.7344727	0.92696
fetal small intestine 77	0.7433234	0.92778
fetal renal pelvis 229	0.7655214	0.92786
fetal stomach 240	0.7093891	0.92954
fetal renal pelvis 220	0.7531302	0.93078
fetal renal pelvis 217	0.7487494	0.93246
ES cells 293	0.7418552	0.93284
ES derived trophoblast 287	0.7710655	0.93516
fetal kidney 87	0.7577163	0.93856
fetal kidney 107	0.7509849	0.93914
fetal stomach 247	0.6718360	0.94026
fetal renal pelvis 230	0.7228401	0.94134
fetal large intestine 63	0.7158930	0.94214
fetal kidney 97	0.7499542	0.94266
fetal kidney 82	0.7207409	0.94276
fetal renal cortex 211	0.7338773	0.94314
fetal kidney 94	0.7297857	0.94334
fetal small intestine 71	0.7206038	0.94412
fetal brain 38	0.8089390	0.94444
fetal kidney 102	0.7253264	0.94488
fetal renal cortex 204	0.7606578	0.94568
fetal renal cortex 203	0.7416409	0.94686
fetal renal pelvis 223	0.7206829	0.94848
fetal kidney 86	0.6850962	0.95090
fetal kidney 88	0.7453846	0.95112
fetal renal pelvis 224	0.7511609	0.95232
fetal renal pelvis 222	0.7212717	0.95346
fetal kidney 105	0.7412447	0.95352
fetal kidney 101	0.7343425	0.95432
fetal kidney 93	0.7109821	0.95604
fetal kidney 96	0.7408939	0.95638
fetal renal cortex 205	0.7224476	0.95948
fetal kidney 104	0.7158955	0.96078
fetal kidney 98	0.7131642	0.96236
fetal renal pelvis 225	0.6991298	0.96664
fetal renal cortex 212	0.7228114	0.96702
fetal renal cortex 209	0.7034354	0.96770
fetal kidney 90	0.7035547	0.96872
fetal renal cortex 208	0.6833806	0.97396
fetal renal cortex 210	0.6700943	0.97822
fetal renal pelvis 218	0.5987447	0.98402

Body mass index 1

DHS sample	fold enrichment	p value
ES derived NP 291	1.1752527	0.16658
iPS 304	1.1282672	0.18066
ES derived NP 292	1.1548563	0.18260
fetal brain 30	1.0825100	0.18762
ES cells 296	1.1431012	0.20878
ES cells 294	1.1281141	0.22922
fetal brain 40	1.0765726	0.24032
ES derived mesoderm 285	1.0814311	0.26270
fetal brain 34	1.0651611	0.26846
fetal brain 35	1.0613337	0.27334
fetal brain 31	1.0540621	0.28742
iPS 303	1.0801672	0.28866
fetal lung 299	1.0819037	0.29352
fetal brain 41	1.0576347	0.30526
fetal brain 29	1.0437534	0.31936
melanocyte 340	1.0580698	0.32126
fetal brain 36	1.0527227	0.32488
melanocyte 337	1.0511865	0.33026
melanocyte 338	1.0486396	0.33566
fetal adrenal gland 26	1.0615732	0.33648
fetal stomach 244	1.0693448	0.34028
ES cells 295	1.0582962	0.35708
fetal adrenal gland 25	1.0571083	0.35796
fetal lung 298	1.0523714	0.35948
Mobilized CD3 320	1.0735171	0.36076
fetal lung 301	1.0461682	0.36968
fetal adrenal gland 27	1.0453583	0.37858
fetal brain 39	1.0289942	0.38842
melanocyte 339	1.0317796	0.39146
iPS 302	1.0341172	0.39808
ES cells 293	1.0354523	0.39858
fetal adrenal gland 28	1.0304544	0.40490
fetal adrenal gland 24	1.0347237	0.40844
fetal brain 32	1.0223178	0.41692
fetal heart 47	1.0217752	0.43428
fetal lung 300	1.0199960	0.43630
fetal spinal cord 235	1.0123749	0.44966
fetal kidney 91	1.0117963	0.45260
fetal kidney 85	1.0035328	0.46928
fetal stomach 242	1.0011717	0.47122
fetal spinal cord 236	1.0043147	0.47480
fetal renal cortex 213	1.0014262	0.47904
fetal stomach 248	1.0028756	0.47966
CD19 6	0.9992518	0.47966
fetal ovary 195	0.9985388	0.47988
fetal kidney 83	0.9969740	0.48582
CD19 7	0.9911368	0.49252
fetal spinal cord 238	0.9960027	0.49906
ES derived MSC 289	0.9950958	0.50066
fetal spinal cord 239	0.9949903	0.50320
ES derived mesoderm 286	0.9957383	0.50506
fetal brain 37	0.9945344	0.51010
fetal brain 38	0.9940265	0.51022
fetal kidney 92	0.9862547	0.51460
fetal fibroblast 273	0.9912257	0.51702
fetal small intestine 75	0.9579303	0.51904
fetal heart 45	0.9862216	0.52124
fetal thymus 258	0.9792175	0.52848
fetal spinal cord 237	0.9855633	0.53120
CD4 13	0.9712051	0.53432
fetal stomach 253	0.9742504	0.53442
pancreas 328	0.9761994	0.53512
fetal adrenal gland 23	0.9811519	0.53828
ES derived MSC 290	0.9784557	0.54030
fetal thymus 265	0.9734899	0.54220
fetal renal pelvis 221	0.9764542	0.54548
fetal large intestine 61	0.9658818	0.54830
fetal heart 49	0.9755133	0.55034
fetal kidney 89	0.9705388	0.55372
keratinocyte 334	0.9808004	0.55522
fetal stomach 243	0.9680475	0.55706
pancreas 327	0.9631805	0.56142
fetal renal cortex 205	0.9682335	0.56406
fetal renal pelvis 219	0.9565346	0.56422
fetal renal cortex 202	0.9673854	0.56520
fetal large intestine 53	0.9586697	0.56528
fetal renal pelvis 232	0.9636533	0.56558
fetal kidney 96	0.9674495	0.56864
fetal thymus 259	0.9550656	0.57318
fetal kidney 95	0.9607376	0.57420
fetal large intestine 67	0.9615807	0.57820
fetal skin 234	0.9654131	0.57960
stomach 284	0.9561377	0.58334
fetal kidney 100	0.9569470	0.58720
fetal thymus 260	0.9487084	0.58922
fetal renal pelvis 220	0.9524585	0.59074
fetal renal cortex 214	0.9565509	0.59134
fetal brain 33	0.9650240	0.59338
fetal stomach 241	0.9462288	0.59412
hematopoietic stem cell 305	0.9572206	0.59438
CD19 5	0.9399599	0.59650
fetal fibroblast 274	0.9648503	0.59668
fetal fibroblast 278	0.9618577	0.60206
fetal kidney 103	0.9473254	0.60206
hematopoietic stem cell 315	0.9526399	0.60420
fetal stomach 250	0.9407411	0.60506
fetal fibroblast 271	0.9630099	0.60548
CD4 15	0.9116031	0.60854
Mobilized CD4 322	0.9255098	0.60924
fetal kidney 98	0.9472227	0.60970
fetal stomach 247	0.9306403	0.60994
fetal thymus 256	0.9407561	0.61136
fetal renal pelvis 228	0.9412770	0.61480
stomach 283	0.9384576	0.61518
fetal thymus 266	0.9357625	0.61548
fetal muscle 176	0.9552611	0.61890
fetal muscle 193	0.9484659	0.62450
fetal kidney 102	0.9360484	0.62474
fetal small intestine 77	0.9368653	0.62684
fetal stomach 249	0.9291353	0.62690
fetal fibroblast 275	0.9557081	0.62846
fetal heart 42	0.9418995	0.63156
fetal kidney 105	0.9428023	0.63304
fetal kidney 84	0.9354406	0.63532
fetal muscle 143	0.9502927	0.63612
fetal renal pelvis 230	0.9303268	0.63614
keratinocyte 336	0.9549400	0.63908
fibroblast 332	0.9483693	0.64114
fetal kidney 99	0.9349957	0.64140
fetal kidney 87	0.9371976	0.64644
CD8 19	0.8980170	0.64652
Mobilized CD3 321	0.9019540	0.64682
fetal renal cortex 207	0.9318625	0.64716
fibroblast 330	0.9436279	0.64760
fetal lung 140	0.9458670	0.64766
fetal renal pelvis 225	0.9289447	0.65020
fetal renal cortex 215	0.9358897	0.65044
fetal testes 254	0.9317404	0.65068
fetal thymus 262	0.8994653	0.65262
fetal fibroblast 277	0.9438593	0.65382
keratinocyte 333	0.9521814	0.65416
fetal kidney 94	0.9234448	0.65624
fetal fibroblast 281	0.9448490	0.65632
fetal renal pelvis 229	0.9287902	0.65862
CD3 10	0.9114667	0.65862
fibroblast 331	0.9402213	0.66000
fetal stomach 240	0.9124205	0.66058
fibroblast 329	0.9386157	0.66142
fetal stomach 251	0.9184162	0.66652
fetal fibroblast 267	0.9389126	0.66708
fetal kidney 86	0.9073987	0.66822
fetal heart 52	0.9338318	0.66864
fetal renal cortex 208	0.9231213	0.66880
fetal heart 43	0.9320433	0.66912
fetal renal cortex 212	0.9274882	0.67142
fetal lung 112	0.9345689	0.67244
fetal muscle 168	0.9276717	0.67344
fetal stomach 246	0.9067745	0.67694
fetal fibroblast 272	0.9376243	0.67764
fetal lung 118	0.9397323	0.67796
fetal renal pelvis 222	0.9163098	0.68034
fetal kidney 107	0.9209150	0.68046
fetal fibroblast 269	0.9388329	0.68062
fetal stomach 252	0.9102136	0.68086
fetal renal pelvis 227	0.9183315	0.68088
keratinocyte 335	0.9414516	0.68178
hematopoietic stem cell 316	0.9158381	0.68186
fetal thymus 261	0.9005196	0.68374
hematopoietic stem cell 309	0.9214279	0.68384
fetal muscle 153	0.9180864	0.68452
fetal fibroblast 276	0.9363257	0.68568
breast 1	0.9330455	0.68770
fetal kidney 88	0.9203513	0.68884
fetal lung 119	0.9340538	0.69060
CD3 9	0.8861080	0.69210
fetal renal pelvis 233	0.9122588	0.69228
fetal thymus 257	0.8901162	0.69288
Mobilized CD8 325	0.8781748	0.69296
fetal lung 128	0.9355886	0.69356
fetal testes 255	0.9202775	0.69406
fetal heart 46	0.9144947	0.69462
fetal fibroblast 268	0.9323935	0.69576
fetal heart 50	0.9156270	0.69616
hematopoietic stem cell 318	0.9095972	0.69624
fetal renal cortex 211	0.9054341	0.69648
fetal kidney 106	0.9104738	0.69906
CD3 12	0.8756293	0.70022
fetal stomach 245	0.8978796	0.70198
fetal heart 44	0.8979841	0.70238
CD20 8	0.9084206	0.70376
hematopoietic stem cell 319	0.9035242	0.70456
fetal renal pelvis 231	0.9128748	0.70482
fetal heart 51	0.9134394	0.70584
fetal lung 132	0.9206902	0.70606
fetal small intestine 69	0.8925999	0.70702
fetal renal cortex 209	0.9075572	0.70756
Mobilized CD4 323	0.8712536	0.70928
fetal renal cortex 206	0.9097946	0.71124
fetal renal cortex 204	0.9122319	0.71246
fetal fibroblast 270	0.9231061	0.71300
fetal renal pelvis 224	0.9106199	0.71644
fetal thymus 263	0.9096605	0.71708
fetal kidney 93	0.8970530	0.71824
fetal renal cortex 210	0.8987106	0.71908
fetal renal pelvis 217	0.8952791	0.72246
fetal heart 48	0.9037499	0.72562
fetal kidney 97	0.9018294	0.72664
fetal kidney 104	0.8964173	0.72842
fetal muscle 180	0.9029619	0.72934
hematopoietic stem cell 312	0.9032142	0.72944
fetal renal pelvis 226	0.8984235	0.73058
fetal muscle 159	0.9008724	0.73124
CD8 22	0.8578145	0.73276
fetal thymus 264	0.8787197	0.73378
fetal fibroblast 282	0.9191997	0.73510
heart 297	0.8867354	0.73702
fetal kidney 101	0.8959542	0.73806
CD4 14	0.8690327	0.74182
breast 2	0.9111832	0.74410
CD3 11	0.8660529	0.74544
fetal large intestine 57	0.8905391	0.74784
fetal small intestine 74	0.8824120	0.74804
CD56 18	0.8489035	0.74840
fetal lung 108	0.8966367	0.74888
CD4 16	0.8398454	0.74994
placenta 201	0.8752586	0.75240
muscle 341	0.8677991	0.75250
CD8 21	0.8425844	0.75376
fetal small intestine 71	0.8763907	0.75432
hematopoietic stem cell 314	0.8912039	0.75898
fetal kidney 81	0.8994309	0.75952
fetal lung 141	0.9062096	0.76038
fetal kidney 90	0.8815178	0.76054
fetal lung 133	0.9055117	0.76164
fetal lung 117	0.9021075	0.76246
fetal muscle 160	0.8904247	0.76412
fetal muscle 175	0.8933133	0.76442
fetal small intestine 73	0.8703463	0.76630
fetal muscle 158	0.8950731	0.76698
fetal muscle 151	0.8820333	0.76772
fetal renal cortex 203	0.8789990	0.76906
fetal lung 126	0.9034112	0.76912
fetal fibroblast 279	0.8997407	0.77040
fetal muscle 150	0.8827696	0.77252
fetal kidney 82	0.8622084	0.77396
fetal muscle 167	0.8905709	0.77618
placenta 198	0.8791674	0.77676
hematopoietic stem cell 310	0.8908213	0.77806
fetal lung 120	0.8942026	0.77840
fetal fibroblast 280	0.8978063	0.77864
fetal small intestine 78	0.8771695	0.78028
fetal muscle 149	0.8786006	0.78054
fetal large intestine 56	0.8663555	0.78066
fetal small intestine 79	0.8554358	0.78070
fetal muscle 173	0.8821181	0.78128
fetal muscle 164	0.8805863	0.78188
fetal renal pelvis 218	0.8421964	0.78222
fetal renal pelvis 223	0.8622147	0.78308
fetal small intestine 80	0.8668044	0.78358
fetal lung 137	0.8916016	0.78362
ovary 326	0.8364594	0.78754
hematopoietic stem cell 308	0.8748138	0.78890
fetal lung 121	0.8955564	0.78946
fetal muscle 189	0.8802590	0.79002
fetal muscle 181	0.8797936	0.79056
fetal muscle 174	0.8773511	0.79104
fetal muscle 148	0.8769015	0.79138
fetal lung 116	0.8971308	0.79198
hematopoietic stem cell 311	0.8692834	0.79568
CD14 3	0.8321546	0.79602
fetal muscle 154	0.8671920	0.79642
fetal muscle 192	0.8794170	0.79788
fetal muscle 188	0.8793792	0.79956
fetal large intestine 66	0.8465473	0.80052
placenta 197	0.8512618	0.80098
fetal lung 111	0.8861616	0.80244
placenta 196	0.8512516	0.80296
fetal lung 114	0.8915797	0.80328
fetal muscle 184	0.8807010	0.80664
fetal muscle 172	0.8861341	0.80692
fetal lung 115	0.8842805	0.80702
fetal lung 123	0.8749418	0.80894
fetal large intestine 58	0.8595263	0.81024
fetal muscle 186	0.8716517	0.81058
fetal muscle 194	0.8798094	0.81134
fetal small intestine 72	0.8470306	0.81340
fetal muscle 169	0.8659302	0.81360
fetal lung 129	0.8757849	0.81668
placenta 200	0.8623907	0.81704
hematopoietic stem cell 307	0.8535791	0.82124
fetal muscle 182	0.8663024	0.82176
fetal lung 134	0.8663553	0.82214
fetal muscle 171	0.8670867	0.82398
fetal muscle 191	0.8588001	0.82494
fetal muscle 155	0.8509872	0.82682
hematopoietic stem cell 306	0.8506322	0.82806
fetal large intestine 64	0.8426704	0.82822
fetal muscle 144	0.8503097	0.82900
fetal small intestine 76	0.8274481	0.82976
small intestine 342	0.8371578	0.82992
fetal renal pelvis 216	0.8429159	0.83018
fetal large intestine 62	0.8245608	0.83110
fetal muscle 178	0.8548510	0.83122
fetal small intestine 70	0.8539715	0.83406
fetal large intestine 59	0.8252686	0.83602
placenta 199	0.8405635	0.83604
Mobilized CD56 324	0.8105434	0.83610
fetal muscle 177	0.8441207	0.83930
fetal large intestine 63	0.8247574	0.83962
fetal lung 127	0.8576240	0.83980
fetal muscle 156	0.8556303	0.83990
fetal large intestine 65	0.8274888	0.84028
fetal lung 139	0.8550024	0.84118
fetal muscle 187	0.8609760	0.84308
fetal muscle 162	0.8582416	0.84376
hematopoietic stem cell 313	0.8464117	0.84516
fetal lung 136	0.8578103	0.84558
fetal muscle 179	0.8499810	0.84624
fetal muscle 146	0.8543762	0.84656
CD14 4	0.8087723	0.84678
ES derived trophoblast 288	0.8529082	0.84986
fetal muscle 185	0.8380396	0.85108
fetal large intestine 55	0.8284665	0.85464
fetal lung 130	0.8553313	0.85568
fetal muscle 170	0.8513099	0.85568
fetal muscle 157	0.8559242	0.85594
fetal lung 124	0.8515292	0.85816
fetal large intestine 54	0.8134312	0.85944
hematopoietic stem cell 317	0.8501840	0.85990
fetal large intestine 60	0.8190369	0.86030
fetal lung 131	0.8678903	0.86074
CD8 20	0.7847326	0.86224
fetal small intestine 68	0.8241195	0.86276
fetal muscle 145	0.8201663	0.86776
fetal lung 138	0.8446081	0.86816
fetal muscle 163	0.8326800	0.86960
fetal muscle 147	0.8338908	0.87046
fetal muscle 165	0.8412112	0.87710
CD56 17	0.7854168	0.87792
fetal lung 109	0.8569574	0.88056
fetal muscle 183	0.8377447	0.88230
fetal lung 113	0.8437563	0.88694
fetal lung 122	0.8218302	0.88876
fetal lung 110	0.8235054	0.89420
fetal lung 135	0.8242338	0.89722
fetal lung 125	0.8180003	0.90086
fetal muscle 152	0.8214661	0.90114
ES derived trophoblast 287	0.8000476	0.90664
fetal muscle 166	0.8146408	0.90764
fetal lung 142	0.8292486	0.91082
fetal muscle 190	0.7910894	0.91538
fetal muscle 161	0.7987097	0.91616

Age at menarche

DHS sample	fold enrichment	p value
fetal brain 31	1.0781738	0.21854
fetal adrenal gland 24	1.1172599	0.25000
fetal adrenal gland 26	1.0787295	0.29964
fetal brain 30	1.0454627	0.31244
fetal adrenal gland 28	1.0629024	0.32092
fetal fibroblast 271	1.0555940	0.32610
fetal fibroblast 272	1.0509124	0.34068
fetal fibroblast 268	1.0480097	0.34436
fetal lung 299	1.0563115	0.34644
fetal fibroblast 273	1.0473057	0.34680
melanocyte 338	1.0434912	0.35334
fetal adrenal gland 27	1.0502334	0.36536
fetal adrenal gland 25	1.0522595	0.36644
muscle 341	1.0545671	0.36852
fetal fibroblast 274	1.0403195	0.36960
melanocyte 339	1.0347617	0.38268
fetal fibroblast 281	1.0361312	0.38314
ES cells 294	1.0322242	0.40880
fetal fibroblast 277	1.0274108	0.40994
fetal muscle 149	1.0310534	0.41046
fetal lung 300	1.0291228	0.41296
pancreas 327	1.0281040	0.42106
fetal fibroblast 282	1.0219507	0.42260
ES derived NP 291	1.0276345	0.42342
fetal muscle 150	1.0250541	0.42644
fetal muscle 179	1.0234466	0.42646
fetal muscle 151	1.0247763	0.42662
fetal muscle 180	1.0240183	0.42714
fibroblast 329	1.0218411	0.42990
fetal fibroblast 278	1.0207421	0.43038
fetal adrenal gland 23	1.0190473	0.43378
fetal fibroblast 275	1.0170573	0.43634
fetal fibroblast 276	1.0171452	0.43738
melanocyte 337	1.0166823	0.43760
fetal fibroblast 280	1.0180777	0.43872
placenta 197	1.0182137	0.44300
fetal muscle 154	1.0172471	0.44546
fibroblast 330	1.0127056	0.45538
melanocyte 340	1.0110143	0.45696
fetal lung 298	1.0111897	0.46252
fetal fibroblast 267	1.0082702	0.46574
fetal muscle 176	1.0064427	0.47046
fetal brain 36	1.0047877	0.47188
fetal muscle 153	1.0059604	0.47356
ES derived NP 292	1.0025112	0.47476
placenta 199	1.0049076	0.47514
fetal lung 301	1.0060382	0.47518
breast 2	1.0036023	0.47892
placenta 198	1.0036039	0.48002
fetal muscle 175	1.0018489	0.48142
placenta 200	1.0017747	0.48346
fetal muscle 160	1.0012051	0.48568
pancreas 328	0.9981111	0.48638
ovary 326	0.9950369	0.49022
iPS 303	0.9978944	0.49312
fetal lung 117	0.9978236	0.49520
fetal brain 29	0.9984005	0.49606
fibroblast 331	0.9980219	0.49682
ES cells 296	0.9914843	0.49908
fetal muscle 167	0.9952298	0.50078
fetal muscle 155	0.9944301	0.50152
keratinocyte 334	0.9978328	0.50168
fibroblast 332	0.9947534	0.50692
fetal lung 108	0.9919662	0.50696
ES cells 295	0.9882919	0.50792
fetal testes 254	0.9891558	0.51064
fetal fibroblast 279	0.9943692	0.51180
fetal muscle 169	0.9898506	0.51472
fetal lung 120	0.9886970	0.52066
fetal muscle 164	0.9871166	0.52230
fetal muscle 168	0.9858199	0.52568
fetal muscle 143	0.9868311	0.52666
small intestine 342	0.9823274	0.52670
fetal spinal cord 235	0.9844145	0.52756
fetal muscle 158	0.9837164	0.53320
fetal muscle 148	0.9784288	0.54286
fetal muscle 181	0.9737956	0.55318
fetal muscle 171	0.9756583	0.55462
keratinocyte 335	0.9800763	0.56008
fetal lung 118	0.9769300	0.56078
breast 1	0.9756826	0.56532
fetal fibroblast 269	0.9756816	0.56906
fetal muscle 159	0.9674232	0.56906
fetal brain 35	0.9787122	0.56938
fetal muscle 186	0.9696161	0.57226
fetal spinal cord 239	0.9685124	0.57372
keratinocyte 333	0.9759636	0.57392
fetal lung 140	0.9710064	0.57588
fetal muscle 178	0.9642664	0.58158
keratinocyte 336	0.9729062	0.58362
fetal brain 34	0.9732575	0.58448
fetal muscle 172	0.9675885	0.58832
placenta 196	0.9571466	0.58886
fetal muscle 165	0.9645980	0.59168
fetal muscle 189	0.9604134	0.59188
Mobilized CD8 325	0.9323614	0.59268
fetal brain 37	0.9691519	0.59568
fetal lung 111	0.9634660	0.59568
fetal stomach 242	0.9330426	0.59854
ES cells 293	0.9504223	0.60072
CD20 8	0.9531602	0.60278
fetal brain 40	0.9683327	0.60410
fetal testes 255	0.9543450	0.60440
fetal thymus 264	0.9411480	0.60594
iPS 304	0.9578446	0.60606
fetal muscle 156	0.9557249	0.60658
fetal muscle 174	0.9534225	0.60704
fetal muscle 166	0.9546837	0.60748
ES derived trophoblast 288	0.9578500	0.61030
fetal kidney 91	0.9511170	0.61034
fetal small intestine 74	0.9468880	0.61296
fetal muscle 157	0.9534515	0.62214
fetal thymus 259	0.9245377	0.62420
fetal thymus 258	0.9299741	0.62566
fetal stomach 253	0.9283623	0.62854
iPS 302	0.9462870	0.62868
fetal muscle 182	0.9480520	0.62990
fetal muscle 173	0.9458072	0.62992
fetal muscle 185	0.9418309	0.63136
Mobilized CD56 324	0.9219220	0.63204
fetal large intestine 64	0.9398527	0.63298
fetal muscle 183	0.9482113	0.63328
fetal lung 137	0.9489402	0.63636
ES derived trophoblast 287	0.9442785	0.63652
fetal fibroblast 270	0.9513055	0.63904
fetal small intestine 69	0.9285824	0.63930
fetal stomach 243	0.9345046	0.63950
fetal lung 132	0.9457802	0.64234
fetal thymus 257	0.9153922	0.64252
fetal kidney 83	0.9228959	0.64304
fetal brain 41	0.9543265	0.64324
ES derived MSC 290	0.9385836	0.64668
fetal renal cortex 207	0.9321430	0.64692
fetal thymus 263	0.9379450	0.64892
fetal muscle 152	0.9407197	0.65050
fetal stomach 244	0.9249701	0.65056
fetal spinal cord 238	0.9387933	0.65078
Mobilized CD4 323	0.9044407	0.65178
placenta 201	0.9258772	0.65218
fetal muscle 193	0.9346613	0.65362
fetal large intestine 62	0.9209553	0.65416
fetal small intestine 80	0.9320175	0.65566
CD3 12	0.8939237	0.65790
CD8 19	0.8862426	0.65960
fetal brain 39	0.9497496	0.66408
fetal ovary 195	0.9051307	0.66432
fetal small intestine 77	0.9193880	0.66474
Mobilized CD3 321	0.8851413	0.66548
fetal large intestine 54	0.9206704	0.66626
fetal spinal cord 236	0.9287758	0.66672
fetal muscle 162	0.9340175	0.66706
fetal lung 134	0.9339082	0.67078
CD8 21	0.8873075	0.67274
fetal renal pelvis 219	0.8989890	0.67480
fetal lung 141	0.9387629	0.67652
fetal muscle 145	0.9188537	0.67708
fetal lung 119	0.9382859	0.67804
fetal lung 116	0.9395653	0.67818
fetal lung 133	0.9372968	0.67884
ES derived MSC 289	0.9264223	0.67972
fetal muscle 188	0.9279745	0.68162
fetal spinal cord 237	0.9308396	0.68166
fetal muscle 191	0.9248982	0.68168
CD4 15	0.8574647	0.68202
hematopoietic stem cell 319	0.9117367	0.68286
fetal renal pelvis 230	0.9090011	0.68334
fetal muscle 184	0.9302067	0.68366
stomach 284	0.9098328	0.68380
fetal lung 125	0.9274253	0.68450
fetal muscle 177	0.9189683	0.68534
fetal stomach 249	0.8995708	0.68696
fetal muscle 192	0.9263142	0.68774
fetal stomach 241	0.8996150	0.68930
fetal large intestine 58	0.9189282	0.69050
fetal large intestine 53	0.8977595	0.69222
fetal muscle 147	0.9204505	0.69248
fetal renal pelvis 233	0.9121700	0.69296
fetal small intestine 78	0.9204440	0.69414
fetal muscle 163	0.9168059	0.69748
fetal thymus 256	0.8956934	0.69862
fetal stomach 250	0.8963937	0.69882
fetal muscle 144	0.9113146	0.70156
fetal large intestine 67	0.9056969	0.70250
fetal lung 128	0.9328788	0.70350
fetal brain 38	0.9302715	0.70372
fetal kidney 81	0.9223468	0.70458
fetal thymus 265	0.8865134	0.70774
fetal lung 123	0.9196340	0.70848
fetal kidney 89	0.9028143	0.70912
fetal lung 126	0.9263984	0.70956
fetal skin 234	0.9127863	0.70992
fetal muscle 161	0.9108324	0.71112
fetal kidney 103	0.9029269	0.71112
fetal muscle 187	0.9189917	0.71176
fetal stomach 240	0.8853585	0.71306
fetal stomach 248	0.8902167	0.71374
fetal kidney 92	0.9072927	0.71382
fetal stomach 247	0.8741675	0.71516
Mobilized CD4 322	0.8599226	0.71526
ES derived mesoderm 286	0.9306282	0.71798
fetal kidney 85	0.9146189	0.71818
fetal thymus 260	0.8805188	0.71956
fetal brain 32	0.9265233	0.72004
fetal renal pelvis 220	0.8975228	0.72284
fetal renal cortex 205	0.9014266	0.72440
fetal lung 136	0.9131002	0.72922
fetal muscle 194	0.9157621	0.72984
Mobilized CD3 320	0.8509543	0.73090
fetal thymus 262	0.8447940	0.73180
fetal renal cortex 214	0.8949282	0.73252
stomach 283	0.8807463	0.73272
fetal renal cortex 210	0.8906625	0.73282
fetal large intestine 60	0.8912159	0.73350
fetal lung 129	0.9121978	0.73362
fetal renal pelvis 232	0.8885141	0.73400
fetal muscle 170	0.9072528	0.73414
fetal small intestine 76	0.8836999	0.73434
fetal small intestine 73	0.8869795	0.73608
fetal renal cortex 213	0.9003702	0.73676
fetal lung 135	0.9077457	0.73896
fetal small intestine 68	0.8937096	0.74190
fetal large intestine 65	0.8845087	0.74240
CD4 13	0.8458365	0.74272
fetal thymus 266	0.8675701	0.74292
fetal large intestine 63	0.8818429	0.74374
fetal lung 124	0.9073303	0.74422
fetal kidney 100	0.8895049	0.74438
fetal large intestine 59	0.8799099	0.74490
fetal large intestine 56	0.8851073	0.74582
fetal large intestine 57	0.8947932	0.74632
fetal renal pelvis 229	0.8907111	0.75076
fetal small intestine 71	0.8816877	0.75148
fetal stomach 252	0.8739060	0.75308
fetal kidney 107	0.8879094	0.75350
ES derived mesoderm 285	0.9134241	0.75418
fetal lung 121	0.9094791	0.75500
fetal lung 114	0.9109569	0.75684
fetal renal cortex 208	0.8820735	0.75736
CD3 9	0.8377744	0.76062
fetal renal cortex 211	0.8739646	0.76212
fetal brain 33	0.9039932	0.76286
fetal muscle 146	0.8928393	0.76596
CD56 18	0.8354014	0.76596
fetal lung 127	0.8923260	0.76760
fetal renal cortex 215	0.8849460	0.77112
fetal kidney 104	0.8747962	0.77134
fetal stomach 246	0.8560724	0.77270
fetal kidney 106	0.8771008	0.77272
hematopoietic stem cell 318	0.8673749	0.77468
fetal lung 112	0.8933748	0.77508
CD19 7	0.8280025	0.77620
fetal large intestine 66	0.8606758	0.77748
fetal small intestine 70	0.8853731	0.77978
fetal muscle 190	0.8730799	0.78050
fetal small intestine 79	0.8567725	0.78188
fetal kidney 90	0.8700138	0.78294
fetal renal pelvis 226	0.8760049	0.78354
fetal lung 130	0.8916795	0.78414
CD3 11	0.8355291	0.78424
fetal small intestine 72	0.8659944	0.78476
fetal kidney 102	0.8589253	0.78550
fetal renal cortex 202	0.8747886	0.78624
fetal renal pelvis 218	0.8371186	0.78698
fetal renal pelvis 225	0.8648122	0.78752
fetal thymus 261	0.8352620	0.78780
fetal kidney 97	0.8691720	0.78832
fetal lung 115	0.8917589	0.79026
fetal lung 131	0.8993170	0.79094
fetal renal pelvis 224	0.8768766	0.79110
fetal renal cortex 212	0.8755921	0.79176
hematopoietic stem cell 314	0.8734020	0.79292
fetal lung 138	0.8848328	0.79308
CD19 6	0.8181180	0.79364
fetal kidney 95	0.8639185	0.79400
fetal kidney 98	0.8629729	0.79424
hematopoietic stem cell 305	0.8636568	0.79448
fetal stomach 251	0.8556388	0.79722
fetal kidney 94	0.8555016	0.79770
fetal kidney 96	0.8698656	0.79788
hematopoietic stem cell 315	0.8610533	0.79878
fetal renal pelvis 222	0.8586434	0.79962
fetal kidney 99	0.8627301	0.80040
fetal kidney 86	0.8347692	0.80192
fetal lung 139	0.8755719	0.80280
fetal stomach 245	0.8440956	0.80448
fetal renal pelvis 217	0.8523832	0.80826
CD4 16	0.7923142	0.80832
fetal large intestine 55	0.8615469	0.80948
fetal lung 110	0.8734677	0.81064
CD8 22	0.7961769	0.81272
fetal renal pelvis 228	0.8439074	0.81524
heart 297	0.8404951	0.82146
fetal large intestine 61	0.8054062	0.82292
fetal kidney 82	0.8345855	0.82324
fetal kidney 105	0.8552703	0.82414
fetal lung 122	0.8620351	0.82462
fetal small intestine 75	0.7462844	0.82528
CD8 20	0.8076392	0.82550
fetal kidney 84	0.8430366	0.82628
fetal renal pelvis 231	0.8563767	0.82654
fetal lung 109	0.8844836	0.82676
fetal lung 142	0.8785898	0.82846
CD3 10	0.8067592	0.82908
hematopoietic stem cell 308	0.8521353	0.83100
fetal renal pelvis 223	0.8334409	0.83356
fetal renal pelvis 227	0.8411721	0.83878
hematopoietic stem cell 316	0.8269536	0.83910
fetal kidney 87	0.8441139	0.83956
fetal renal pelvis 216	0.8360576	0.84186
CD4 14	0.7936500	0.84712
fetal renal pelvis 221	0.8439386	0.84732
CD19 5	0.7882073	0.84826
fetal renal cortex 203	0.8342461	0.84972
fetal renal cortex 206	0.8406882	0.85022
fetal renal cortex 204	0.8473069	0.85086
fetal kidney 101	0.8381701	0.85206
fetal kidney 88	0.8411431	0.85440
fetal heart 42	0.8386609	0.85646
fetal lung 113	0.8621014	0.85868
fetal heart 46	0.8288325	0.86484
fetal heart 45	0.8296078	0.87034
fetal renal cortex 209	0.8193691	0.87040
hematopoietic stem cell 310	0.8382443	0.87314
hematopoietic stem cell 306	0.8190125	0.87340
fetal heart 44	0.7942374	0.87550
fetal kidney 93	0.8062832	0.87764
fetal heart 49	0.8183896	0.88200
hematopoietic stem cell 311	0.8147752	0.88238
fetal heart 47	0.8053307	0.88302
fetal heart 48	0.8188176	0.88750
fetal heart 51	0.8207960	0.88896
fetal heart 43	0.8254407	0.88906
hematopoietic stem cell 312	0.8146495	0.89010
fetal heart 50	0.8016214	0.90472
hematopoietic stem cell 307	0.7937132	0.90580
CD56 17	0.7487071	0.91314
CD14 3	0.7278249	0.92324
hematopoietic stem cell 309	0.7768040	0.92572
fetal heart 52	0.7991293	0.92688
hematopoietic stem cell 313	0.7760572	0.93800
CD14 4	0.7085627	0.94548
hematopoietic stem cell 317	0.7610589	0.96170

Myopia

DHS sample	fold enrichment	p value
CD3 10	1.1158415	0.28790
CD19 5	1.1059306	0.30828
CD19 7	1.1067734	0.31460
fetal thymus 258	1.0911269	0.31876
Mobilized CD3 320	1.0820048	0.35624
CD19 6	1.0731579	0.36150
fetal thymus 265	1.0623209	0.36844
fetal thymus 256	1.0550838	0.38174
CD4 13	1.0592471	0.38218
hematopoietic stem cell 314	1.0439587	0.38328
fetal thymus 266	1.0500606	0.38636
CD3 9	1.0546255	0.38766
CD8 20	1.0459947	0.40046
hematopoietic stem cell 317	1.0307068	0.40912
fetal brain 30	1.0200545	0.41050
CD4 14	1.0302574	0.42684
Mobilized CD4 322	1.0295032	0.42774
fetal thymus 257	1.0285353	0.42796
hematopoietic stem cell 312	1.0205906	0.43754
fetal thymus 264	1.0238143	0.43844
CD3 11	1.0253483	0.43870
hematopoietic stem cell 310	1.0191475	0.44254
CD56 17	1.0204619	0.44800
fetal thymus 261	1.0111598	0.45914
fetal thymus 259	1.0106569	0.46280
fetal brain 40	1.0059316	0.46756
fetal brain 29	1.0030520	0.47950
hematopoietic stem cell 313	1.0006746	0.48710
fetal thymus 260	0.9905588	0.50110
CD8 22	0.9812586	0.50632
hematopoietic stem cell 315	0.9894835	0.51290
hematopoietic stem cell 305	0.9824150	0.52222
Mobilized CD4 323	0.9731681	0.52244
CD14 4	0.9810152	0.52248
Mobilized CD56 324	0.9782029	0.52462
hematopoietic stem cell 309	0.9838346	0.52590
CD8 21	0.9668969	0.53344
fetal thymus 262	0.9482632	0.54844
hematopoietic stem cell 311	0.9717208	0.55360
fetal brain 31	0.9836046	0.55632
fetal brain 41	0.9776660	0.56082
fetal brain 37	0.9745529	0.57014
hematopoietic stem cell 318	0.9599545	0.57076
fetal brain 38	0.9734058	0.57216
CD3 12	0.9385126	0.57544
hematopoietic stem cell 306	0.9579496	0.58296
fetal brain 39	0.9704943	0.58852
Mobilized CD3 321	0.9272387	0.59266
CD4 16	0.9264507	0.59338
hematopoietic stem cell 307	0.9496103	0.59906
hematopoietic stem cell 308	0.9515119	0.60476
melanocyte 339	0.9632217	0.61070
fetal large intestine 61	0.9224333	0.62184
hematopoietic stem cell 316	0.9342333	0.62728
CD14 3	0.9183981	0.63294
hematopoietic stem cell 319	0.9277872	0.63812
fetal small intestine 75	0.8725814	0.64150
melanocyte 337	0.9529485	0.64866
placenta 197	0.9240174	0.65436
CD56 18	0.8957344	0.65534
fetal brain 34	0.9508420	0.66130
melanocyte 340	0.9427265	0.66246
CD4 15	0.8355017	0.69970
CD8 19	0.8537583	0.70326
fetal brain 35	0.9375647	0.71568
iPS 304	0.9119013	0.72150
placenta 199	0.8975066	0.72560
fetal brain 36	0.9178700	0.72964
Mobilized CD8 325	0.8426094	0.73728
fetal fibroblast 270	0.9107933	0.74184
placenta 200	0.8986297	0.74202
fetal fibroblast 269	0.9127110	0.74442
iPS 302	0.8930478	0.74594
melanocyte 338	0.9157234	0.75008
ES cells 293	0.8699927	0.76344
small intestine 342	0.8664628	0.76532
fetal muscle 179	0.8883606	0.77006
placenta 198	0.8797948	0.77270
fetal fibroblast 275	0.9024747	0.77702
fetal fibroblast 279	0.8963633	0.77794
fetal fibroblast 277	0.8929883	0.78012
placenta 201	0.8511233	0.78386
fetal spinal cord 239	0.8792168	0.78406
fetal fibroblast 271	0.8947221	0.78586
fetal fibroblast 280	0.8934589	0.78612
placenta 196	0.8554136	0.78756
fetal muscle 183	0.8837993	0.78816
fetal brain 32	0.9001277	0.78864
stomach 284	0.8449921	0.78904
fetal muscle 158	0.8790902	0.79052
fetal muscle 180	0.8707822	0.79154
ES cells 294	0.8484171	0.79442
ES cells 295	0.8431753	0.79946
fetal fibroblast 267	0.8801117	0.81016
muscle 341	0.8272837	0.81030
fetal muscle 160	0.8611455	0.81184
fetal spinal cord 237	0.8710881	0.81432
fetal thymus 263	0.8564225	0.81690
fetal fibroblast 276	0.8827560	0.81876
fetal muscle 157	0.8701566	0.81990
fetal muscle 189	0.8616146	0.81994
keratinocyte 336	0.8851561	0.82012
keratinocyte 334	0.8889341	0.82046
fetal spinal cord 235	0.8614766	0.82094
fetal small intestine 70	0.8525450	0.82636
fetal muscle 170	0.8603111	0.82854
fetal muscle 152	0.8596728	0.82972
fetal lung 129	0.8660630	0.83036
ES derived mesoderm 286	0.8802035	0.83268
fetal lung 128	0.8776503	0.83290
fetal kidney 89	0.8330291	0.83472
fetal fibroblast 278	0.8683834	0.83498
fetal muscle 154	0.8367185	0.83658
fetal fibroblast 282	0.8763879	0.83692
fetal renal pelvis 223	0.8209918	0.83714
ES derived mesoderm 285	0.8727525	0.83718
fetal lung 125	0.8555844	0.83830
fetal spinal cord 236	0.8427550	0.83832
fetal fibroblast 272	0.8699959	0.83850
fetal renal cortex 204	0.8450660	0.83888
fetal muscle 149	0.8409188	0.83964
fetal muscle 161	0.8465006	0.84078
fetal kidney 81	0.8555390	0.84086
fetal brain 33	0.8639126	0.84164
fetal muscle 187	0.8560206	0.84232
fetal muscle 171	0.8529045	0.84268
fetal fibroblast 268	0.8684841	0.84488
fetal muscle 144	0.8374084	0.84508
fetal muscle 186	0.8493436	0.84540
fetal muscle 166	0.8505910	0.84568
fetal fibroblast 273	0.8665202	0.84622
fetal lung 138	0.8527044	0.84624
ES cells 296	0.8129512	0.84768
fetal muscle 169	0.8436967	0.84828
fetal renal pelvis 222	0.8193475	0.84966
fetal large intestine 58	0.8260674	0.85076
fetal renal pelvis 226	0.8259573	0.85202
fetal muscle 153	0.8275171	0.85270
fetal renal cortex 203	0.8205576	0.85390
fetal fibroblast 281	0.8633720	0.85452
fetal muscle 159	0.8253192	0.85700
ES derived NP 292	0.8050702	0.85800
ES derived NP 291	0.8005920	0.85868
fetal muscle 175	0.8409338	0.86006
fetal muscle 194	0.8498719	0.86048
fetal muscle 173	0.8341112	0.86058
fetal muscle 184	0.8448652	0.86472
fetal small intestine 72	0.8037521	0.86510
keratinocyte 333	0.8667884	0.86670
fetal muscle 165	0.8423104	0.86670
fetal muscle 156	0.8346628	0.86734
iPS 303	0.8301811	0.86894
fetal stomach 240	0.7745237	0.87004
fetal kidney 100	0.8147297	0.87010
fetal renal pelvis 216	0.8072203	0.87012
fetal lung 141	0.8516552	0.87180
fetal renal cortex 215	0.8206088	0.87198
fetal lung 121	0.8505948	0.87204
fetal lung 139	0.8312348	0.87264
fetal lung 119	0.8510594	0.87288
fetal muscle 167	0.8364205	0.87370
fetal lung 133	0.8493366	0.87388
fetal muscle 174	0.8232643	0.87392
fetal kidney 91	0.8216021	0.87400
fetal fibroblast 274	0.8494909	0.87456
fetal ovary 195	0.7738104	0.87462
fetal muscle 193	0.8202754	0.87490
fetal small intestine 68	0.8070059	0.87498
keratinocyte 335	0.8566951	0.87534
fetal renal pelvis 229	0.8106867	0.87542
fetal muscle 182	0.8305652	0.87564
fetal muscle 185	0.8142493	0.87580
fetal renal pelvis 233	0.8063719	0.87732
fetal lung 135	0.8318843	0.87798
fetal renal pelvis 225	0.7987466	0.87884
fetal muscle 192	0.8319007	0.87900
breast 2	0.8416121	0.87948
fetal lung 120	0.8363807	0.88030
CD20 8	0.8030477	0.88160
fetal lung 109	0.8504022	0.88174
fetal muscle 190	0.8102662	0.88174
fetal lung 130	0.8342712	0.88322
fetal renal cortex 208	0.7934213	0.88356
fetal renal cortex 209	0.7988616	0.88364
fetal kidney 90	0.7962643	0.88436
fetal renal cortex 205	0.8011471	0.88462
fetal lung 132	0.8314027	0.88510
fetal kidney 107	0.8019494	0.88510
fetal renal cortex 210	0.7894986	0.88516
fetal muscle 151	0.8055127	0.88592
fetal lung 126	0.8418788	0.88670
fetal muscle 181	0.8192469	0.88726
fetal kidney 88	0.8095236	0.88774
fetal renal cortex 212	0.8097942	0.88802
fetal lung 115	0.8366392	0.88874
fetal muscle 145	0.7947084	0.88874
fetal kidney 93	0.7840785	0.88914
fetal muscle 150	0.8073356	0.88920
fetal muscle 155	0.8069124	0.88928
fetal large intestine 67	0.7865220	0.88934
breast 1	0.8327919	0.89196
fetal renal pelvis 219	0.7430958	0.89196
fetal kidney 101	0.7996484	0.89218
fetal renal pelvis 221	0.8025047	0.89276
fetal renal cortex 202	0.8008010	0.89298
fetal small intestine 73	0.7764394	0.89352
fetal renal pelvis 231	0.8084485	0.89390
fetal muscle 191	0.8079424	0.89404
fetal kidney 84	0.7872379	0.89424
fetal kidney 104	0.7884171	0.89436
fetal large intestine 63	0.7707874	0.89502
fetal muscle 188	0.8190454	0.89584
fetal lung 136	0.8217714	0.89662
fetal lung 131	0.8412340	0.89668
fetal lung 110	0.8170477	0.89790
fetal muscle 164	0.8079096	0.89804
fetal muscle 147	0.8081890	0.89884
fetal lung 116	0.8376554	0.89956
fetal renal pelvis 230	0.7686577	0.90064
fetal muscle 172	0.8279223	0.90114
fetal renal cortex 211	0.7753980	0.90136
fetal kidney 95	0.7858379	0.90140
fetal muscle 176	0.8232448	0.90284
fetal kidney 97	0.7889711	0.90300
fetal lung 124	0.8187681	0.90380
fetal muscle 178	0.8006642	0.90482
stomach 283	0.7511593	0.90626
fetal muscle 143	0.8211517	0.90690
fetal kidney 98	0.7807215	0.90690
fetal muscle 162	0.8084654	0.90830
fetal renal pelvis 232	0.7707038	0.90868
fetal lung 142	0.8254803	0.90872
fetal renal pelvis 218	0.7297682	0.90878
fetal large intestine 64	0.7728810	0.90932
fetal spinal cord 238	0.8062622	0.91064
heart 297	0.7645873	0.91116
fetal muscle 163	0.7967483	0.91138
fetal lung 111	0.8196852	0.91170
fetal small intestine 74	0.7668355	0.91256
fetal kidney 105	0.7870201	0.91262
fetal muscle 148	0.7920552	0.91292
fetal lung 114	0.8295496	0.91488
fetal muscle 177	0.7863333	0.91530
fetal small intestine 76	0.7451975	0.91536
fetal lung 123	0.8021383	0.91682
fetal kidney 87	0.7825620	0.91780
fetal muscle 146	0.7973996	0.91782
fetal renal pelvis 217	0.7655073	0.91784
fetal renal pelvis 227	0.7735278	0.91864
fetal lung 137	0.8099817	0.91876
fetal small intestine 79	0.7422176	0.91938
fetal testes 255	0.7961154	0.92004
fetal large intestine 59	0.7432833	0.92014
fetal kidney 86	0.7266894	0.92014
fetal lung 117	0.8118116	0.92070
fetal large intestine 54	0.7484985	0.92112
fetal renal cortex 206	0.7804259	0.92146
fetal lung 298	0.7856072	0.92156
fetal lung 113	0.8142518	0.92276
fetal kidney 82	0.7459094	0.92350
fetal heart 47	0.7643482	0.92432
fetal kidney 96	0.7766691	0.92498
fetal lung 122	0.7818419	0.92668
fetal stomach 247	0.6940379	0.92684
fetal stomach 248	0.7342457	0.92802
fetal large intestine 66	0.7381801	0.92814
fetal stomach 253	0.7247010	0.92956
fetal kidney 94	0.7438484	0.93040
fetal large intestine 56	0.7450425	0.93056
fetal testes 254	0.7628650	0.93072
fetal large intestine 60	0.7474869	0.93080
fetal small intestine 71	0.7396712	0.93126
fetal heart 49	0.7683098	0.93142
fetal kidney 102	0.7456130	0.93156
fetal renal pelvis 224	0.7734536	0.93170
fetal stomach 252	0.7349900	0.93236
fetal kidney 103	0.7506126	0.93266
fetal lung 108	0.7802596	0.93270
fibroblast 331	0.7881354	0.93288
fetal stomach 246	0.7160165	0.93332
fetal small intestine 78	0.7638600	0.93348
fetal renal cortex 214	0.7641907	0.93372
fetal heart 46	0.7613513	0.93402
fetal lung 140	0.8000396	0.93548
ovary 326	0.6983567	0.93562
fetal stomach 241	0.7110649	0.93628
ES derived trophoblast 287	0.7638755	0.93670
fetal lung 127	0.7785767	0.93714
fetal renal pelvis 228	0.7332994	0.93744
fetal lung 118	0.8069445	0.93802
fetal lung 112	0.7862125	0.93872
fetal kidney 99	0.7520864	0.93882
fetal kidney 92	0.7633295	0.93888
fetal lung 300	0.7715531	0.93894
fetal renal cortex 213	0.7647459	0.93942
fetal heart 45	0.7589385	0.94118
fetal heart 52	0.7796842	0.94142
fetal large intestine 57	0.7506705	0.94182
fetal heart 43	0.7750737	0.94292
pancreas 327	0.7068006	0.94364
fetal stomach 249	0.7002332	0.94364
fetal large intestine 65	0.7253596	0.94380
fetal kidney 85	0.7771248	0.94502
fetal stomach 250	0.6960188	0.94530
fetal small intestine 80	0.7413479	0.94534
fetal heart 44	0.7077525	0.94602
fetal large intestine 62	0.7082818	0.94726
fetal heart 51	0.7560823	0.94760
fetal stomach 243	0.7318093	0.94768
fetal heart 50	0.7480745	0.94844
fetal kidney 106	0.7458272	0.94866
fibroblast 329	0.7720950	0.94870
fetal stomach 251	0.7170227	0.94918
ES derived trophoblast 288	0.7607744	0.94934
fetal lung 134	0.7661504	0.95078
fetal muscle 168	0.7535011	0.95100
fetal kidney 83	0.7002485	0.95112
fetal stomach 244	0.7101580	0.95140
fetal heart 42	0.7492586	0.95196
fetal lung 299	0.7486680	0.95238
fetal stomach 242	0.6509393	0.95262
fetal large intestine 55	0.7305967	0.95334
fetal stomach 245	0.7030902	0.95448
pancreas 328	0.7063867	0.95464
fibroblast 330	0.7607773	0.95546
fetal renal cortex 207	0.7259596	0.95744
fetal heart 48	0.7426249	0.95746
fetal lung 301	0.7391531	0.95954
fibroblast 332	0.7510659	0.96254
fetal small intestine 69	0.6815614	0.96262
ES derived MSC 290	0.7293419	0.96480
fetal small intestine 77	0.6881441	0.96530
ES derived MSC 289	0.7165954	0.97212
fetal renal pelvis 220	0.6915340	0.97440
fetal adrenal gland 23	0.7121568	0.97680
fetal adrenal gland 27	0.6755240	0.98224
fetal adrenal gland 24	0.6398158	0.98292
fetal adrenal gland 26	0.6812933	0.98302
fetal large intestine 53	0.6113956	0.98544
fetal skin 234	0.6759603	0.98722
fetal adrenal gland 28	0.6951702	0.98798
fetal adrenal gland 25	0.6319510	0.98970
