# Supplementary material for: Extensive Association of Common Disease Variants with Regulatory Sequence
Source: PLoS One. 2016 Nov 22;11(11):e0165893. doi: 10.1371/journal.pone.0165893 (PMC5119736; doi:10.1371/journal.pone.0165893)
Supplement: S4 Table — (PDF) [file pone.0165893.s008.pdf]

# Crohns disease 1

| DHS sample | fold enrichment | p value |
|---|---|---|
| Th2 | 1.8723392 | 0.00002 |
| GM12865 | 1.5574424 | 0.00002 |
| GM12864 | 1.5513738 | 0.00004 |
| GM06990 | 1.6066787 | 0.00018 |
| SAEC | 1.3161175 | 0.00022 |
| RPTEC | 1.2929015 | 0.00086 |
| HEEpiC | 1.2817663 | 0.00110 |
| HRCEpiC | 1.2951248 | 0.00132 |
| MonocytesCD14RO01746 | 1.4452453 | 0.00180 |
| PrEC | 1.2714171 | 0.00184 |
| CD20 | 1.4926316 | 0.00236 |
| HL60 | 1.4154033 | 0.00370 |
| GM18507 | 1.3656566 | 0.00406 |
| HCFaa | 1.2878981 | 0.00468 |
| HRE | 1.2433487 | 0.00670 |
| Jurkat | 1.2914860 | 0.00786 |
| GM12878 | 1.3337864 | 0.01044 |
| HIPEpiC | 1.2343222 | 0.01084 |
| SKMC | 1.2308123 | 0.01586 |
| HNPCEpiC | 1.2096596 | 0.01632 |
| NB4 | 1.2697117 | 0.02338 |
| HAsp | 1.2130925 | 0.02418 |
| HMVECLBl | 1.2274418 | 0.02704 |
| AG09309 | 1.1842522 | 0.03730 |
| HVMF | 1.2082686 | 0.04280 |
| CD34Mobilized | 1.2191692 | 0.04540 |
| Th1 | 1.2153345 | 0.04574 |
| HBMEC | 1.1654572 | 0.04744 |
| HPdLF | 1.1849859 | 0.05266 |
| HAEpiC | 1.1751718 | 0.05332 |
| WI38 | 1.1880548 | 0.05794 |
| AG04449 | 1.1732987 | 0.06012 |
| AoSMC | 1.1535288 | 0.06458 |
| HMF | 1.1667934 | 0.06474 |
| RWPE1 | 1.1784975 | 0.06526 |
| NHLF | 1.1541975 | 0.06576 |
| HCPEpiC | 1.1531380 | 0.06592 |
| Th0 | 1.1979712 | 0.06668 |
| HRGEC | 1.1703869 | 0.06950 |
| BJ | 1.1659082 | 0.06956 |
| GM19238 | 1.2066157 | 0.07404 |
| AG04450 | 1.1698785 | 0.07466 |
| NHA | 1.1465108 | 0.07818 |
| GM19240 | 1.1815015 | 0.08050 |
| PANC1 | 1.1609899 | 0.10470 |
| HPDE6E6E7 | 1.1557041 | 0.10724 |
| NHEK | 1.1404163 | 0.10730 |
| HCT116 | 1.1594825 | 0.10954 |
| AG10803 | 1.1384986 | 0.11094 |
| NHDFAd | 1.1242723 | 0.11336 |
| HRPEpiC | 1.1041947 | 0.12200 |
| HMVECdBlAd | 1.1310067 | 0.13104 |
| HAc | 1.1090851 | 0.13190 |
| CLL | 1.1655136 | 0.13830 |
| HGF | 1.1251002 | 0.13998 |
| HTR8svn | 1.1329621 | 0.14480 |
| AG09319 | 1.1212055 | 0.14580 |
| HFFMyc | 1.1087980 | 0.14956 |
| HPF | 1.1158053 | 0.15168 |
| HFF | 1.1061256 | 0.16180 |
| HPAF | 1.1038399 | 0.16364 |
| HCM | 1.0996991 | 0.16492 |
| A549 | 1.1214028 | 0.17960 |
| HeLaS3 | 1.1171781 | 0.18154 |
| AoAF | 1.0982944 | 0.18860 |
| HeLaS3IFNa4h | 1.1208531 | 0.19792 |
| HPAEC | 1.0989561 | 0.20008 |
| GM19239 | 1.1232149 | 0.20250 |
| CMK | 1.1248506 | 0.20360 |
| HSMM | 1.0770563 | 0.21534 |
| K562 | 1.1048303 | 0.22504 |
| HCF | 1.0799857 | 0.23058 |
| HMVECdBlNeo | 1.0813198 | 0.24598 |
| HMVECdLyNeo | 1.0772781 | 0.25542 |
| SKNMC | 1.0676474 | 0.26100 |
| HMVECdLyAd | 1.0711952 | 0.27932 |
| HMVECLLy | 1.0694220 | 0.27998 |
| HMVECdAd | 1.0702696 | 0.28198 |
| NHDFneo | 1.0589854 | 0.29012 |
| HAh | 1.0489526 | 0.29282 |
| HMVECdNeo | 1.0580138 | 0.31334 |
| UrotheliaUT189 | 1.0586404 | 0.31634 |
| Urothelia | 1.0508516 | 0.33808 |
| HConF | 1.0333286 | 0.37982 |
| IshikawaEstradiol | 1.0342034 | 0.38374 |
| GM12891 | 1.0416459 | 0.38656 |
| HepG2 | 1.0344902 | 0.39414 |
| PanIsletD | 1.0267928 | 0.39878 |
| H7hESC | 1.0109518 | 0.43698 |
| Huh7 | 1.0187524 | 0.43744 |
| Stellate | 1.0102106 | 0.46366 |
| HSMMemb | 1.0052056 | 0.47864 |
| IshikawaTamoxifen | 1.0042255 | 0.47968 |
| GM12892 | 1.0046345 | 0.48174 |
| FibroP | 1.0031647 | 0.48816 |
| HSMMtube | 1.0001016 | 0.49810 |
| HUVEC | 0.9977591 | 0.49930 |
| Gliobla | 0.9879105 | 0.52712 |
| Myometr | 0.9880879 | 0.53210 |
| HMEC | 0.9871906 | 0.53858 |
| Melano | 0.9874821 | 0.55408 |
| T47D | 0.9644476 | 0.58644 |
| MCF7 | 0.9615914 | 0.61260 |
| MCF7Hypoxia | 0.9487654 | 0.63510 |
| WERIRb1 | 0.9547521 | 0.65608 |
| pHTE | 0.9402317 | 0.68382 |
| ProgFib | 0.9385414 | 0.68560 |
| Huh7.5 | 0.9301434 | 0.68598 |
| NT2D1 | 0.9493805 | 0.69364 |
| Fibrobl | 0.9358403 | 0.70250 |
| Medullo | 0.9254489 | 0.72042 |
| PanIslets | 0.9222353 | 0.72314 |
| LNCaP | 0.9219277 | 0.73848 |
| 8988T | 0.8912026 | 0.74710 |
| LNCaPAndrogen | 0.8890209 | 0.79016 |
| Osteobl | 0.8977966 | 0.79604 |
| H9ES | 0.8781070 | 0.83442 |
| Hepatocytes | 0.8479229 | 0.84316 |
| BE2C | 0.8708785 | 0.84978 |
| Caco2 | 0.7905930 | 0.85254 |
| H1hESC | 0.8310512 | 0.88788 |
| Chorion | 0.7996103 | 0.89796 |
| iPS | 0.8003965 | 0.89994 |
| SKNSHRA | 0.6580504 | 0.99378 |

# Type 1 diabetes

| DHS sample | fold enrichment | p value |
|---|---|---|
| Th2 | 1.7359869 | 0.00002 |
| Jurkat | 1.3886281 | 0.00016 |
| RPTEC | 1.2765270 | 0.00094 |
| HRCEpiC | 1.2877834 | 0.00120 |
| SAEC | 1.2664436 | 0.00150 |
| HRE | 1.2555096 | 0.00284 |
| GM12865 | 1.3437479 | 0.00316 |
| GM12864 | 1.3251246 | 0.00522 |
| NHA | 1.2453078 | 0.00522 |
| HAsp | 1.2540817 | 0.00584 |
| SKMC | 1.2389446 | 0.00646 |
| HPF | 1.2604812 | 0.00654 |
| HEEpiC | 1.2183406 | 0.00742 |
| HMF | 1.2339157 | 0.00744 |
| HCFaa | 1.2272205 | 0.00842 |
| HIPEpiC | 1.2182290 | 0.00846 |
| HBMEC | 1.2205391 | 0.00856 |
| HNPCEpiC | 1.2056542 | 0.00912 |
| NHLF | 1.2184487 | 0.00998 |
| MonocytesCD14RO01746 | 1.3222114 | 0.01002 |
| HPAF | 1.2246775 | 0.01006 |
| GM06990 | 1.3336424 | 0.01044 |
| HAEpiC | 1.2261391 | 0.01198 |
| AG04450 | 1.2346454 | 0.01228 |
| NB4 | 1.2679619 | 0.01444 |
| WI38 | 1.2292578 | 0.01446 |
| PrEC | 1.1914465 | 0.01466 |
| HRGEC | 1.2213529 | 0.01692 |
| CD20 | 1.3202543 | 0.01728 |
| NHDFneo | 1.2113075 | 0.01936 |
| HCPEpiC | 1.1883529 | 0.02026 |
| HCF | 1.2054789 | 0.02042 |
| HVMF | 1.2132891 | 0.02238 |
| BJ | 1.2008829 | 0.02250 |
| AoAF | 1.2040091 | 0.02260 |
| HGF | 1.2111653 | 0.02314 |
| Th1 | 1.2196406 | 0.02430 |
| AoSMC | 1.1736989 | 0.02440 |
| HFF | 1.1838673 | 0.02652 |
| AG10803 | 1.2012109 | 0.02678 |
| HMVECLBl | 1.2013503 | 0.02740 |
| HMVECdBlAd | 1.2039301 | 0.02744 |
| Th0 | 1.2213606 | 0.02786 |
| GM12878 | 1.2495411 | 0.02870 |
| HCM | 1.1737966 | 0.02904 |
| NHDFAd | 1.1754104 | 0.02940 |
| HAc | 1.1705976 | 0.03006 |
| HPdLF | 1.1941458 | 0.03026 |
| AG09319 | 1.1938767 | 0.03260 |
| HFFMyc | 1.1681770 | 0.03594 |
| AG09309 | 1.1611884 | 0.03858 |
| HPDE6E6E7 | 1.2039356 | 0.03940 |
| HMVECdLyAd | 1.1978092 | 0.03974 |
| HMVECdAd | 1.2020938 | 0.03982 |
| GM18507 | 1.2144226 | 0.04452 |
| HAh | 1.1398763 | 0.04682 |
| HPAEC | 1.1773222 | 0.04842 |
| HMVECdBlNeo | 1.1722949 | 0.05158 |
| CD34Mobilized | 1.1866008 | 0.05266 |
| HConF | 1.1611591 | 0.05464 |
| AG04449 | 1.1556225 | 0.05636 |
| HMVECdNeo | 1.1757000 | 0.05842 |
| HMVECdLyNeo | 1.1668477 | 0.06196 |
| HMVECLLy | 1.1548386 | 0.08032 |
| CLL | 1.1640679 | 0.11906 |
| HL60 | 1.1558815 | 0.11968 |
| HRPEpiC | 1.1016921 | 0.12210 |
| Stellate | 1.1289334 | 0.13756 |
| CMK | 1.1399332 | 0.15982 |
| HTR8svn | 1.1097256 | 0.16710 |
| PANC1 | 1.1087514 | 0.17414 |
| HCT116 | 1.1019541 | 0.19318 |
| RWPE1 | 1.0855321 | 0.20562 |
| IshikawaEstradiol | 1.0891991 | 0.20772 |
| Urothelia | 1.0906553 | 0.21180 |
| GM19238 | 1.0990415 | 0.21682 |
| Huh7 | 1.0968120 | 0.21830 |
| NHEK | 1.0810226 | 0.21976 |
| GM19240 | 1.0851253 | 0.22728 |
| A549 | 1.0855179 | 0.24344 |
| HSMM | 1.0551634 | 0.26470 |
| HUVEC | 1.0660899 | 0.27332 |
| UrotheliaUT189 | 1.0683169 | 0.27612 |
| IshikawaTamoxifen | 1.0568942 | 0.30190 |
| FibroP | 1.0428983 | 0.31078 |
| GM19239 | 1.0621958 | 0.31624 |
| Myometr | 1.0474600 | 0.31850 |
| MCF7 | 1.0403317 | 0.34858 |
| HSMMemb | 1.0393530 | 0.35758 |
| K562 | 1.0386953 | 0.37306 |
| PanIsletD | 1.0295944 | 0.37930 |
| SKNMC | 1.0143463 | 0.43850 |
| MCF7Hypoxia | 1.0152947 | 0.44460 |
| HepG2 | 1.0141478 | 0.44744 |
| Gliobla | 1.0132412 | 0.44892 |
| Melano | 1.0067134 | 0.46854 |
| Huh7.5 | 1.0031196 | 0.48130 |
| HSMMtube | 0.9984080 | 0.50142 |
| NT2D1 | 0.9924201 | 0.52566 |
| GM12892 | 0.9834020 | 0.54236 |
| HMEC | 0.9863151 | 0.54956 |
| GM12891 | 0.9743323 | 0.56556 |
| T47D | 0.9750615 | 0.56750 |
| HeLaS3 | 0.9679595 | 0.59500 |
| Fibrobl | 0.9716288 | 0.60412 |
| ProgFib | 0.9620199 | 0.62546 |
| pHTE | 0.9605406 | 0.63784 |
| HeLaS3IFNa4h | 0.9490623 | 0.64218 |
| PanIslets | 0.9554409 | 0.64416 |
| H9ES | 0.9362927 | 0.70078 |
| LNCaPAndrogen | 0.9291605 | 0.70788 |
| Osteobl | 0.9299193 | 0.73732 |
| SKNSHRA | 0.8994965 | 0.75442 |
| WERIRb1 | 0.9285877 | 0.76528 |
| H7hESC | 0.9451935 | 0.77854 |
| LNCaP | 0.9114199 | 0.78884 |
| 8988T | 0.8628472 | 0.82516 |
| BE2C | 0.8873572 | 0.83402 |
| Caco2 | 0.8184181 | 0.84686 |
| Chorion | 0.8462201 | 0.86030 |
| Hepatocytes | 0.8503269 | 0.86040 |
| iPS | 0.8348004 | 0.87638 |
| H1hESC | 0.8508196 | 0.87862 |
| Medullo | 0.8048017 | 0.95234 |

# Celiac disease

| DHS sample | fold enrichment | p value |
|---|---|---|
| Th2 | 2.0885836 | 0.00002 |
| CD20 | 1.7410799 | 0.00002 |
| GM12865 | 1.6399225 | 0.00002 |
| GM12864 | 1.6389793 | 0.00002 |
| GM06990 | 1.6840644 | 0.00006 |
| Jurkat | 1.4864791 | 0.00010 |
| MonocytesCD14RO01746 | 1.6461172 | 0.00012 |
| GM12878 | 1.4546144 | 0.00060 |
| GM18507 | 1.4238609 | 0.00074 |
| CD34Mobilized | 1.3886191 | 0.00108 |
| HBMEC | 1.2933084 | 0.00182 |
| Th1 | 1.3721329 | 0.00236 |
| Th0 | 1.3609568 | 0.00272 |
| HAEpiC | 1.2982280 | 0.00364 |
| HIPEpiC | 1.2686446 | 0.00400 |
| SKMC | 1.2597842 | 0.00706 |
| CMK | 1.3854781 | 0.00722 |
| NB4 | 1.3209430 | 0.00824 |
| AG04449 | 1.2590504 | 0.00852 |
| HCFaa | 1.2466900 | 0.01236 |
| HAsp | 1.2370796 | 0.01284 |
| HRE | 1.2198535 | 0.01396 |
| WI38 | 1.2560766 | 0.01506 |
| HL60 | 1.3224797 | 0.01596 |
| GM19238 | 1.2998019 | 0.01648 |
| AG04450 | 1.2421259 | 0.01800 |
| AG09309 | 1.2119457 | 0.01880 |
| NHA | 1.2164980 | 0.01932 |
| HCPEpiC | 1.2090737 | 0.02006 |
| HPdLF | 1.2314272 | 0.02126 |
| BJ | 1.2249719 | 0.02272 |
| HPF | 1.2224741 | 0.02484 |
| NHLF | 1.1976884 | 0.02672 |
| GM19239 | 1.2772458 | 0.02836 |
| HMVECLBl | 1.2178804 | 0.02910 |
| HNPCEpiC | 1.1825929 | 0.02926 |
| SAEC | 1.1825473 | 0.02978 |
| HRCEpiC | 1.1855076 | 0.03224 |
| HVMF | 1.2151940 | 0.03762 |
| CLL | 1.2607957 | 0.04422 |
| HEEpiC | 1.1595975 | 0.04594 |
| GM19240 | 1.2079182 | 0.04866 |
| HSMM | 1.1540619 | 0.04978 |
| RPTEC | 1.1496248 | 0.05552 |
| HPAF | 1.1594192 | 0.06442 |
| AoSMC | 1.1385775 | 0.07040 |
| HGF | 1.1670272 | 0.07058 |
| HRGEC | 1.1655450 | 0.07158 |
| HMVECdBlAd | 1.1615611 | 0.07752 |
| HFF | 1.1489435 | 0.07764 |
| NHDFneo | 1.1597312 | 0.07814 |
| HMF | 1.1448801 | 0.08528 |
| PrEC | 1.1279326 | 0.08676 |
| AG10803 | 1.1521026 | 0.08812 |
| HMVECdBlNeo | 1.1527095 | 0.08832 |
| AG09319 | 1.1530489 | 0.08954 |
| HCM | 1.1299077 | 0.09576 |
| HCF | 1.1412288 | 0.10150 |
| HMVECdLyNeo | 1.1475543 | 0.10326 |
| HFFMyc | 1.1280241 | 0.10496 |
| HUVEC | 1.1399235 | 0.10686 |
| AoAF | 1.1361152 | 0.10878 |
| GM12892 | 1.1877288 | 0.11480 |
| HTR8svn | 1.1380437 | 0.12802 |
| GM12891 | 1.1720434 | 0.13170 |
| PANC1 | 1.1430692 | 0.13678 |
| HSMMtube | 1.1058858 | 0.14000 |
| SKNMC | 1.1232308 | 0.15080 |
| Stellate | 1.1314248 | 0.15372 |
| HMVECdAd | 1.1154030 | 0.16348 |
| HAc | 1.0916740 | 0.17034 |
| Huh7 | 1.1224551 | 0.17748 |
| HRPEpiC | 1.0827359 | 0.18356 |
| FibroP | 1.0834374 | 0.18502 |
| NHDFAd | 1.0870431 | 0.19870 |
| HConF | 1.0900445 | 0.20082 |
| Urothelia | 1.1056249 | 0.20208 |
| PanIsletD | 1.0829728 | 0.20678 |
| HMVECdLyAd | 1.0898516 | 0.22018 |
| HPAEC | 1.0873893 | 0.22282 |
| K562 | 1.0986195 | 0.22624 |
| NHEK | 1.0775916 | 0.23620 |
| HAh | 1.0638455 | 0.23626 |
| HSMMemb | 1.0823823 | 0.24112 |
| HMVECdNeo | 1.0722672 | 0.26044 |
| IshikawaEstradiol | 1.0722417 | 0.26078 |
| HMVECLLy | 1.0613431 | 0.29070 |
| HepG2 | 1.0681959 | 0.29404 |
| Myometr | 1.0515253 | 0.30594 |
| ProgFib | 1.0577121 | 0.31044 |
| HPDE6E6E7 | 1.0537433 | 0.31896 |
| IshikawaTamoxifen | 1.0515924 | 0.32504 |
| RWPE1 | 1.0375362 | 0.36176 |
| HCT116 | 1.0314682 | 0.38402 |
| UrotheliaUT189 | 1.0350880 | 0.38442 |
| Huh7.5 | 1.0240940 | 0.42200 |
| pHTE | 1.0206189 | 0.42468 |
| Gliobla | 1.0165862 | 0.44180 |
| HeLaS3IFNa4h | 1.0157748 | 0.44220 |
| MCF7 | 1.0112772 | 0.44816 |
| Melano | 1.0080756 | 0.46338 |
| HMEC | 1.0029936 | 0.48142 |
| Fibrobl | 1.0034087 | 0.48244 |
| 8988T | 0.9952141 | 0.50122 |
| SKNSHRA | 0.9913960 | 0.50126 |
| BE2C | 0.9880965 | 0.52898 |
| A549 | 0.9790482 | 0.55172 |
| T47D | 0.9651357 | 0.58354 |
| H1hESC | 0.9662562 | 0.58628 |
| Osteobl | 0.9663282 | 0.59860 |
| H7hESC | 0.9774753 | 0.61354 |
| Caco2 | 0.9259216 | 0.61818 |
| PanIslets | 0.9584244 | 0.61934 |
| MCF7Hypoxia | 0.9525184 | 0.62026 |
| NT2D1 | 0.9694446 | 0.62190 |
| Chorion | 0.9438120 | 0.62440 |
| HeLaS3 | 0.9545579 | 0.63628 |
| Hepatocytes | 0.9382926 | 0.65062 |
| iPS | 0.9331332 | 0.65092 |
| LNCaP | 0.9504462 | 0.66078 |
| H9ES | 0.9477251 | 0.66254 |
| WERIRb1 | 0.9533698 | 0.66590 |
| LNCaPAndrogen | 0.9244619 | 0.70824 |
| Medullo | 0.8296076 | 0.90722 |

# Lipid levels 4

| DHS sample | fold enrichment | p value |
|---|---|---|
| HCFaa | 1.2448994 | 0.00002 |
| RPTEC | 1.2436949 | 0.00002 |
| HGF | 1.2308769 | 0.00004 |
| HCPEpiC | 1.2065278 | 0.00006 |
| HRCEpiC | 1.2127774 | 0.00008 |
| HIPEpiC | 1.1960413 | 0.00012 |
| HPAF | 1.2114465 | 0.00014 |
| HAEpiC | 1.2075890 | 0.00016 |
| HCF | 1.2066381 | 0.00022 |
| HRGEC | 1.1995209 | 0.00032 |
| HCM | 1.1870994 | 0.00032 |
| HAsp | 1.1906183 | 0.00034 |
| NB4 | 1.2301466 | 0.00044 |
| HMVECLBl | 1.1940462 | 0.00048 |
| AoSMC | 1.1717000 | 0.00050 |
| HPAEC | 1.2048951 | 0.00052 |
| HNPCEpiC | 1.1698580 | 0.00058 |
| HRE | 1.1669437 | 0.00064 |
| MonocytesCD14RO01746 | 1.2507835 | 0.00070 |
| HBMEC | 1.1734176 | 0.00078 |
| HEEpiC | 1.1593749 | 0.00078 |
| HVMF | 1.2012047 | 0.00080 |
| SKMC | 1.1708607 | 0.00096 |
| SAEC | 1.1513678 | 0.00112 |
| HMF | 1.1692220 | 0.00126 |
| AG04449 | 1.1761359 | 0.00142 |
| HMVECdBlNeo | 1.1783399 | 0.00160 |
| K562 | 1.2225396 | 0.00162 |
| HMVECdAd | 1.1919353 | 0.00190 |
| HL60 | 1.2294506 | 0.00196 |
| HConF | 1.1769790 | 0.00196 |
| AoAF | 1.1670390 | 0.00196 |
| HPF | 1.1663648 | 0.00226 |
| NHDFAd | 1.1502055 | 0.00270 |
| HAc | 1.1474237 | 0.00288 |
| AG04450 | 1.1654701 | 0.00322 |
| BJ | 1.1591883 | 0.00326 |
| HMVECdLyNeo | 1.1679085 | 0.00330 |
| GM12864 | 1.1940229 | 0.00334 |
| PANC1 | 1.1846101 | 0.00350 |
| HMVECdBlAd | 1.1650470 | 0.00360 |
| HPdLF | 1.1610839 | 0.00368 |
| GM12865 | 1.1771981 | 0.00446 |
| AG09319 | 1.1606972 | 0.00450 |
| HCT116 | 1.1803923 | 0.00476 |
| AG09309 | 1.1391188 | 0.00488 |
| HepG2 | 1.1804944 | 0.00508 |
| NHA | 1.1440856 | 0.00510 |
| WI38 | 1.1572714 | 0.00540 |
| PrEC | 1.1244872 | 0.00552 |
| Jurkat | 1.1572492 | 0.00564 |
| HMVECdNeo | 1.1577109 | 0.00670 |
| SKNMC | 1.1582680 | 0.00680 |
| HFF | 1.1335658 | 0.00696 |
| NHLF | 1.1335158 | 0.00726 |
| HeLaS3 | 1.1674405 | 0.00786 |
| HFFMyc | 1.1279836 | 0.00824 |
| AG10803 | 1.1429965 | 0.00832 |
| HMVECdLyAd | 1.1528451 | 0.00838 |
| HPDE6E6E7 | 1.1566570 | 0.00846 |
| GM12878 | 1.1758000 | 0.00866 |
| HTR8svn | 1.1505581 | 0.01018 |
| Th2 | 1.1783062 | 0.01158 |
| HRPEpiC | 1.1130781 | 0.01184 |
| NHEK | 1.1357784 | 0.01198 |
| HMVECLLy | 1.1423135 | 0.01200 |
| GM06990 | 1.1759870 | 0.01354 |
| NHDFneo | 1.1296193 | 0.01442 |
| RWPE1 | 1.1297849 | 0.01654 |
| A549 | 1.1509590 | 0.01672 |
| HAh | 1.1047804 | 0.01678 |
| IshikawaEstradiol | 1.1372273 | 0.01688 |
| Stellate | 1.1335404 | 0.02624 |
| CD20 | 1.1615124 | 0.02854 |
| HeLaS3IFNa4h | 1.1437135 | 0.03070 |
| CMK | 1.1407113 | 0.04202 |
| CD34Mobilized | 1.1111728 | 0.04552 |
| GM18507 | 1.1183045 | 0.04654 |
| HUVEC | 1.1076100 | 0.04660 |
| HSMMemb | 1.1022805 | 0.04992 |
| IshikawaTamoxifen | 1.1057706 | 0.05118 |
| HSMM | 1.0779338 | 0.06376 |
| PanIsletD | 1.0860168 | 0.06472 |
| FibroP | 1.0727407 | 0.06936 |
| H7hESC | 1.0623840 | 0.07010 |
| Huh7 | 1.1030873 | 0.08050 |
| CLL | 1.1072062 | 0.08578 |
| Myometr | 1.0777473 | 0.09734 |
| Melano | 1.0608107 | 0.10076 |
| NT2D1 | 1.0592800 | 0.12278 |
| BE2C | 1.0739730 | 0.13172 |
| Urothelia | 1.0699988 | 0.14180 |
| T47D | 1.0789053 | 0.15346 |
| HSMMtube | 1.0520747 | 0.16792 |
| MCF7 | 1.0596759 | 0.17522 |
| GM19238 | 1.0657022 | 0.18124 |
| Huh7.5 | 1.0676716 | 0.18202 |
| GM19240 | 1.0592734 | 0.18468 |
| GM19239 | 1.0658119 | 0.19654 |
| Gliobla | 1.0585514 | 0.20708 |
| Th1 | 1.0524559 | 0.20830 |
| Medullo | 1.0545726 | 0.21028 |
| Th0 | 1.0470653 | 0.24230 |
| LNCaPAndrogen | 1.0454091 | 0.25716 |
| ProgFib | 1.0410945 | 0.26194 |
| WERIRb1 | 1.0334156 | 0.27934 |
| GM12891 | 1.0454572 | 0.28394 |
| Fibrobl | 1.0257019 | 0.33982 |
| GM12892 | 1.0306631 | 0.34436 |
| Caco2 | 1.0377338 | 0.34836 |
| UrotheliaUT189 | 1.0236435 | 0.35810 |
| PanIslets | 1.0209390 | 0.37466 |
| MCF7Hypoxia | 1.0216753 | 0.38114 |
| HMEC | 1.0124068 | 0.41444 |
| pHTE | 1.0091161 | 0.43890 |
| H9ES | 1.0044480 | 0.46476 |
| SKNSHRA | 1.0002354 | 0.49160 |
| Hepatocytes | 0.9946343 | 0.51912 |
| LNCaP | 0.9950559 | 0.52550 |
| 8988T | 0.9859521 | 0.56204 |
| Osteobl | 0.9855759 | 0.58522 |
| Chorion | 0.9431930 | 0.74718 |
| H1hESC | 0.9497554 | 0.74874 |
| iPS | 0.9334040 | 0.78716 |

# Height 3

| DHS sample | fold enrichment | p value |
|---|---|---|
| HPdLF | 1.2038098 | 0.00002 |
| HCFaa | 1.2034178 | 0.00002 |
| AG09319 | 1.2030211 | 0.00002 |
| NHDFneo | 1.1960445 | 0.00002 |
| HCPEpiC | 1.1881112 | 0.00002 |
| HAEpiC | 1.1865597 | 0.00002 |
| HPF | 1.1792183 | 0.00002 |
| HIPEpiC | 1.1764390 | 0.00002 |
| HFFMyc | 1.1741713 | 0.00002 |
| HBMEC | 1.1630462 | 0.00002 |
| HNPCEpiC | 1.1531859 | 0.00002 |
| HGF | 1.2155993 | 0.00004 |
| HAsp | 1.2028841 | 0.00004 |
| AG04450 | 1.1917643 | 0.00004 |
| AoAF | 1.1917021 | 0.00004 |
| HVMF | 1.1832023 | 0.00006 |
| HConF | 1.1802677 | 0.00006 |
| NHDFAd | 1.1686937 | 0.00006 |
| HMF | 1.1673131 | 0.00006 |
| AoSMC | 1.1572199 | 0.00006 |
| AG04449 | 1.1699742 | 0.00008 |
| SKMC | 1.1616999 | 0.00010 |
| HPAF | 1.1565538 | 0.00010 |
| AG09309 | 1.1671062 | 0.00012 |
| NHLF | 1.1559727 | 0.00012 |
| HRPEpiC | 1.1341483 | 0.00014 |
| WI38 | 1.1691180 | 0.00016 |
| AG10803 | 1.1683074 | 0.00018 |
| BJ | 1.1661909 | 0.00018 |
| HCM | 1.1518737 | 0.00018 |
| SKNMC | 1.1739919 | 0.00020 |
| HCF | 1.1589999 | 0.00020 |
| NHA | 1.1506002 | 0.00022 |
| GM12865 | 1.1803257 | 0.00024 |
| HRGEC | 1.1521688 | 0.00028 |
| HFF | 1.1445957 | 0.00036 |
| HAc | 1.1328056 | 0.00054 |
| HAh | 1.1198298 | 0.00054 |
| GM12864 | 1.1672493 | 0.00112 |
| HRCEpiC | 1.1210114 | 0.00130 |
| HPAEC | 1.1420888 | 0.00154 |
| RPTEC | 1.1110349 | 0.00192 |
| HMVECLBl | 1.1250343 | 0.00330 |
| HTR8svn | 1.1329504 | 0.00392 |
| PANC1 | 1.1394421 | 0.00394 |
| HEEpiC | 1.0947395 | 0.00616 |
| Jurkat | 1.1181694 | 0.00632 |
| GM06990 | 1.1550862 | 0.00646 |
| SAEC | 1.0917972 | 0.00728 |
| HRE | 1.0945128 | 0.00748 |
| HMVECdLyNeo | 1.1147532 | 0.00776 |
| HL60 | 1.1414732 | 0.00896 |
| HMVECdBlNeo | 1.1105184 | 0.00936 |
| GM12878 | 1.1332713 | 0.00946 |
| HCT116 | 1.1199751 | 0.01024 |
| HUVEC | 1.1128697 | 0.01160 |
| RWPE1 | 1.1073162 | 0.01198 |
| CD20 | 1.1457411 | 0.01472 |
| HMVECdBlAd | 1.1003426 | 0.01498 |
| HMVECdNeo | 1.1032618 | 0.01704 |
| A549 | 1.1160527 | 0.01722 |
| NB4 | 1.1123641 | 0.01828 |
| HeLaS3 | 1.1096132 | 0.01830 |
| HSMM | 1.0795627 | 0.02002 |
| HPDE6E6E7 | 1.1029157 | 0.02034 |
| IshikawaEstradiol | 1.0948421 | 0.02660 |
| Th2 | 1.1157821 | 0.02866 |
| HMVECLLy | 1.0910378 | 0.03076 |
| CMK | 1.1148712 | 0.03406 |
| PanIsletD | 1.0739812 | 0.04192 |
| HMVECdAd | 1.0871146 | 0.04262 |
| CD34Mobilized | 1.0874756 | 0.04362 |
| HSMMemb | 1.0833057 | 0.04366 |
| Stellate | 1.0893872 | 0.04572 |
| MonocytesCD14RO01746 | 1.1013275 | 0.04646 |
| K562 | 1.0960655 | 0.04724 |
| HMVECdLyAd | 1.0822451 | 0.04828 |
| PrEC | 1.0593319 | 0.05642 |
| IshikawaTamoxifen | 1.0749869 | 0.06858 |
| Myometr | 1.0666729 | 0.07318 |
| HeLaS3IFNa4h | 1.0824848 | 0.07454 |
| FibroP | 1.0528496 | 0.08150 |
| NHEK | 1.0617837 | 0.08846 |
| CLL | 1.0786547 | 0.10136 |
| GM18507 | 1.0684972 | 0.10384 |
| BE2C | 1.0592173 | 0.11826 |
| Gliobla | 1.0650175 | 0.12388 |
| NT2D1 | 1.0389469 | 0.15516 |
| MCF7 | 1.0482879 | 0.16426 |
| Melano | 1.0338898 | 0.17698 |
| T47D | 1.0534782 | 0.18198 |
| GM19239 | 1.0497141 | 0.20178 |
| HSMMtube | 1.0330867 | 0.21156 |
| SKNSHRA | 1.0487794 | 0.21610 |
| ProgFib | 1.0374603 | 0.22612 |
| HepG2 | 1.0399026 | 0.22968 |
| Urothelia | 1.0298005 | 0.27590 |
| GM19238 | 1.0265288 | 0.31662 |
| Huh7.5 | 1.0253865 | 0.32368 |
| WERIRb1 | 1.0183744 | 0.33456 |
| MCF7Hypoxia | 1.0240740 | 0.33526 |
| GM19240 | 1.0189604 | 0.35084 |
| Huh7 | 1.0117377 | 0.41372 |
| Th0 | 1.0111293 | 0.41544 |
| H9ES | 1.0026642 | 0.47574 |
| UrotheliaUT189 | 0.9935828 | 0.54760 |
| H7hESC | 0.9958923 | 0.55016 |
| GM12891 | 0.9846100 | 0.59326 |
| Medullo | 0.9837871 | 0.61814 |
| LNCaPAndrogen | 0.9825774 | 0.62008 |
| GM12892 | 0.9749817 | 0.65188 |
| pHTE | 0.9800747 | 0.65736 |
| 8988T | 0.9692038 | 0.67944 |
| Hepatocytes | 0.9696658 | 0.68512 |
| HMEC | 0.9735597 | 0.71858 |
| Th1 | 0.9693547 | 0.72386 |
| H1hESC | 0.9639532 | 0.73476 |
| Fibrobl | 0.9665647 | 0.75258 |
| LNCaP | 0.9665985 | 0.75530 |
| Chorion | 0.9477570 | 0.78878 |
| PanIslets | 0.9535088 | 0.81214 |
| Osteobl | 0.9488947 | 0.84800 |
| iPS | 0.9268030 | 0.87424 |
| Caco2 | 0.8922697 | 0.90404 |

# Waist−hip ratio

| DHS sample | fold enrichment | p value |
|---|---|---|
| HRCEpiC | 1.5678935 | 0.00032 |
| RPTEC | 1.4791609 | 0.00082 |
| SKMC | 1.4586966 | 0.00276 |
| NHDFAd | 1.4471189 | 0.00340 |
| HRE | 1.3623773 | 0.01170 |
| HAEpiC | 1.3833484 | 0.01620 |
| NB4 | 1.4667839 | 0.02312 |
| NHDFneo | 1.3412465 | 0.02830 |
| AG04449 | 1.3135367 | 0.03744 |
| HCPEpiC | 1.2953750 | 0.03746 |
| AG09309 | 1.2848220 | 0.04346 |
| HAsp | 1.2683769 | 0.04808 |
| MCF7Hypoxia | 1.3913981 | 0.05490 |
| BJ | 1.2587911 | 0.07166 |
| HFF | 1.2476943 | 0.07748 |
| AG10803 | 1.2592430 | 0.07760 |
| HMF | 1.2429910 | 0.07780 |
| AoSMC | 1.2300222 | 0.07898 |
| Melano | 1.2163799 | 0.08112 |
| HCM | 1.2323430 | 0.08508 |
| AoAF | 1.2392016 | 0.08820 |
| HRPEpiC | 1.2004124 | 0.08820 |
| HSMM | 1.2172100 | 0.09102 |
| WERIRb1 | 1.2209580 | 0.10682 |
| HVMF | 1.2353416 | 0.10710 |
| Huh7 | 1.2975582 | 0.10930 |
| SKNMC | 1.2111553 | 0.11376 |
| AG09319 | 1.2127515 | 0.12092 |
| FibroP | 1.1840594 | 0.12594 |
| BE2C | 1.2400426 | 0.12818 |
| HConF | 1.2071558 | 0.12840 |
| SAEC | 1.1627222 | 0.14066 |
| PrEC | 1.1665590 | 0.14224 |
| HPdLF | 1.1852237 | 0.14444 |
| MCF7 | 1.2109692 | 0.15020 |
| PANC1 | 1.2068402 | 0.15832 |
| HPAF | 1.1704624 | 0.15834 |
| HAc | 1.1510855 | 0.16108 |
| HAh | 1.1378925 | 0.16758 |
| K562 | 1.2273779 | 0.17336 |
| HCFaa | 1.1542002 | 0.17362 |
| HFFMyc | 1.1596428 | 0.17760 |
| IshikawaEstradiol | 1.1856956 | 0.17788 |
| HNPCEpiC | 1.1363803 | 0.18060 |
| HGF | 1.1607996 | 0.18856 |
| HCF | 1.1606088 | 0.18914 |
| MonocytesCD14RO01746 | 1.2043579 | 0.20832 |
| IshikawaTamoxifen | 1.1658643 | 0.20894 |
| HL60 | 1.1982727 | 0.21426 |
| NHA | 1.1255255 | 0.21808 |
| HeLaS3 | 1.1615191 | 0.22612 |
| Huh7.5 | 1.1798560 | 0.22668 |
| H7hESC | 1.0890170 | 0.22700 |
| NHLF | 1.1151640 | 0.23172 |
| HRGEC | 1.1289664 | 0.23664 |
| HPF | 1.1254952 | 0.23706 |
| HSMMtube | 1.1161641 | 0.24430 |
| NHEK | 1.1263685 | 0.25042 |
| HIPEpiC | 1.1063010 | 0.25344 |
| CD20 | 1.1731271 | 0.25374 |
| GM12865 | 1.1249818 | 0.27472 |
| HEEpiC | 1.0821783 | 0.28922 |
| NT2D1 | 1.0828932 | 0.29190 |
| GM18507 | 1.1234633 | 0.29552 |
| HBMEC | 1.0690582 | 0.32344 |
| CD34Mobilized | 1.0891507 | 0.33200 |
| CMK | 1.1039264 | 0.33234 |
| GM12864 | 1.0914206 | 0.33430 |
| GM12878 | 1.0932076 | 0.33698 |
| SKNSHRA | 1.0854954 | 0.34836 |
| GM06990 | 1.0926392 | 0.34872 |
| HeLaS3IFNa4h | 1.0767714 | 0.35726 |
| T47D | 1.0722167 | 0.36438 |
| AG04450 | 1.0594166 | 0.37114 |
| ProgFib | 1.0581915 | 0.37482 |
| HPAEC | 1.0571823 | 0.37700 |
| Gliobla | 1.0597732 | 0.38356 |
| Urothelia | 1.0499435 | 0.39360 |
| HepG2 | 1.0517339 | 0.39722 |
| GM19239 | 1.0469492 | 0.41152 |
| GM19238 | 1.0449789 | 0.41240 |
| HMVECLBl | 1.0330979 | 0.42400 |
| PanIsletD | 1.0305503 | 0.42448 |
| GM19240 | 1.0334607 | 0.43008 |
| GM12891 | 1.0344334 | 0.43308 |
| HMEC | 1.0225248 | 0.43960 |
| UrotheliaUT189 | 1.0154658 | 0.46172 |
| LNCaP | 1.0095649 | 0.46494 |
| HMVECdBlNeo | 1.0122035 | 0.46650 |
| HMVECLLy | 1.0103158 | 0.46822 |
| HMVECdBlAd | 1.0100174 | 0.47360 |
| HUVEC | 1.0028467 | 0.47874 |
| 8988T | 0.9938347 | 0.48308 |
| CLL | 0.9966164 | 0.48762 |
| Th0 | 1.0009278 | 0.48816 |
| pHTE | 0.9966988 | 0.49596 |
| Th1 | 0.9964244 | 0.49872 |
| HSMMemb | 0.9930544 | 0.50246 |
| Fibrobl | 0.9932351 | 0.50424 |
| Medullo | 0.9925164 | 0.50432 |
| Osteobl | 0.9925106 | 0.50538 |
| HMVECdNeo | 0.9906976 | 0.50696 |
| Th2 | 0.9834026 | 0.51306 |
| Hepatocytes | 0.9743558 | 0.51652 |
| GM12892 | 0.9733974 | 0.52320 |
| HMVECdLyAd | 0.9794423 | 0.52638 |
| HCT116 | 0.9689675 | 0.53342 |
| HMVECdLyNeo | 0.9745852 | 0.54186 |
| LNCaPAndrogen | 0.9606636 | 0.54956 |
| H9ES | 0.9546621 | 0.56188 |
| iPS | 0.9382665 | 0.57366 |
| RWPE1 | 0.9558329 | 0.57466 |
| Caco2 | 0.9044574 | 0.58878 |
| HPDE6E6E7 | 0.9441455 | 0.59274 |
| A549 | 0.9370667 | 0.59328 |
| Jurkat | 0.9395268 | 0.60270 |
| H1hESC | 0.9196691 | 0.61396 |
| HTR8svn | 0.9210992 | 0.63388 |
| PanIslets | 0.9117903 | 0.64018 |
| Myometr | 0.9159039 | 0.65764 |
| Chorion | 0.8704035 | 0.66646 |
| HMVECdAd | 0.8993485 | 0.67922 |
| WI38 | 0.8996282 | 0.70564 |
| Stellate | 0.8671220 | 0.71626 |

# Crohns disease 2

| DHS sample | fold enrichment | p value |
|---|---|---|
| Th2 | 1.859972 | 0.00002 |
| GM06990 | 1.555823 | 0.00002 |
| CD20 | 1.534260 | 0.00002 |
| MonocytesCD14RO01746 | 1.520789 | 0.00002 |
| GM12864 | 1.481283 | 0.00002 |
| GM12865 | 1.462237 | 0.00002 |
| Jurkat | 1.343135 | 0.00002 |
| AG04450 | 1.321676 | 0.00002 |
| HRCEpiC | 1.313375 | 0.00002 |
| HCFaa | 1.284095 | 0.00002 |
| HAc | 1.268601 | 0.00002 |
| RPTEC | 1.265542 | 0.00002 |
| NHLF | 1.260369 | 0.00002 |
| GM12878 | 1.384828 | 0.00004 |
| GM18507 | 1.380525 | 0.00004 |
| Th0 | 1.309725 | 0.00004 |
| SKMC | 1.278430 | 0.00004 |
| HIPEpiC | 1.262179 | 0.00004 |
| HEEpiC | 1.259877 | 0.00004 |
| HL60 | 1.405856 | 0.00006 |
| HRE | 1.280022 | 0.00006 |
| HAsp | 1.263274 | 0.00006 |
| AoSMC | 1.249840 | 0.00006 |
| NB4 | 1.339304 | 0.00008 |
| CD34Mobilized | 1.322341 | 0.00008 |
| SAEC | 1.267549 | 0.00008 |
| HBMEC | 1.258360 | 0.00008 |
| NHA | 1.256461 | 0.00008 |
| HNPCEpiC | 1.249866 | 0.00008 |
| PrEC | 1.244207 | 0.00008 |
| HMF | 1.277819 | 0.00010 |
| K562 | 1.340969 | 0.00014 |
| HPF | 1.279459 | 0.00014 |
| BJ | 1.262914 | 0.00016 |
| Th1 | 1.281529 | 0.00018 |
| HCPEpiC | 1.248958 | 0.00018 |
| HAEpiC | 1.249989 | 0.00022 |
| AG09309 | 1.240001 | 0.00022 |
| AoAF | 1.259130 | 0.00028 |
| HAh | 1.209564 | 0.00034 |
| RWPE1 | 1.259006 | 0.00036 |
| AG04449 | 1.241461 | 0.00036 |
| HRPEpiC | 1.221288 | 0.00036 |
| HPAF | 1.236506 | 0.00040 |
| HCM | 1.224587 | 0.00040 |
| WI38 | 1.266045 | 0.00044 |
| HCT116 | 1.300138 | 0.00048 |
| HFF | 1.227630 | 0.00048 |
| HCF | 1.240462 | 0.00052 |
| HPdLF | 1.251851 | 0.00054 |
| HFFMyc | 1.225005 | 0.00056 |
| HMVECLBl | 1.250008 | 0.00068 |
| HPDE6E6E7 | 1.265417 | 0.00070 |
| GM19238 | 1.299166 | 0.00076 |
| NHDFAd | 1.217555 | 0.00082 |
| CLL | 1.320692 | 0.00090 |
| CMK | 1.316547 | 0.00092 |
| PANC1 | 1.283264 | 0.00100 |
| HVMF | 1.243395 | 0.00102 |
| AG10803 | 1.238302 | 0.00108 |
| HGF | 1.240031 | 0.00116 |
| HRGEC | 1.228329 | 0.00120 |
| HTR8svn | 1.242725 | 0.00132 |
| AG09319 | 1.229643 | 0.00146 |
| HConF | 1.223509 | 0.00146 |
| A549 | 1.269014 | 0.00150 |
| GM19240 | 1.244906 | 0.00150 |
| HMVECdBlAd | 1.227664 | 0.00156 |
| HeLaS3 | 1.251949 | 0.00184 |
| GM19239 | 1.274623 | 0.00222 |
| HPAEC | 1.211536 | 0.00298 |
| HMVECdAd | 1.223528 | 0.00346 |
| NHDFneo | 1.197221 | 0.00370 |
| HSMM | 1.171846 | 0.00374 |
| HMVECdLyNeo | 1.205187 | 0.00462 |
| HMVECdLyAd | 1.214676 | 0.00466 |
| Urothelia | 1.211404 | 0.00476 |
| PanIsletD | 1.181047 | 0.00480 |
| HMVECdBlNeo | 1.201073 | 0.00496 |
| NHEK | 1.191225 | 0.00512 |
| HeLaS3IFNa4h | 1.240729 | 0.00532 |
| HMVECdNeo | 1.205489 | 0.00542 |
| Stellate | 1.222039 | 0.00554 |
| HepG2 | 1.213447 | 0.00664 |
| HUVEC | 1.201721 | 0.00690 |
| IshikawaEstradiol | 1.190714 | 0.00796 |
| HSMMemb | 1.191343 | 0.00816 |
| Huh7 | 1.213687 | 0.01046 |
| HMVECLLy | 1.185704 | 0.01088 |
| IshikawaTamoxifen | 1.187141 | 0.01100 |
| Myometr | 1.160565 | 0.01474 |
| UrotheliaUT189 | 1.171439 | 0.01902 |
| Gliobla | 1.179363 | 0.02468 |
| Melano | 1.115543 | 0.02492 |
| ProgFib | 1.157655 | 0.02528 |
| FibroP | 1.118583 | 0.02640 |
| GM12892 | 1.190076 | 0.02684 |
| GM12891 | 1.192898 | 0.02728 |
| Huh7.5 | 1.178255 | 0.02862 |
| HSMMtube | 1.124095 | 0.03324 |
| BE2C | 1.150618 | 0.03404 |
| H7hESC | 1.090603 | 0.03854 |
| H9ES | 1.138321 | 0.04896 |
| T47D | 1.152849 | 0.04994 |
| MCF7 | 1.130878 | 0.05436 |
| PanIslets | 1.129823 | 0.06030 |
| SKNMC | 1.112879 | 0.06458 |
| MCF7Hypoxia | 1.138603 | 0.06942 |
| HMEC | 1.100088 | 0.08842 |
| 8988T | 1.131259 | 0.09900 |
| pHTE | 1.086759 | 0.13236 |
| Hepatocytes | 1.107883 | 0.13468 |
| WERIRb1 | 1.071177 | 0.15394 |
| Caco2 | 1.122650 | 0.16788 |
| LNCaPAndrogen | 1.082857 | 0.17484 |
| H1hESC | 1.081523 | 0.17708 |
| Fibrobl | 1.066503 | 0.19102 |
| NT2D1 | 1.054452 | 0.19260 |
| LNCaP | 1.060839 | 0.21202 |
| iPS | 1.077875 | 0.21814 |
| SKNSHRA | 1.068346 | 0.24442 |
| Medullo | 1.041887 | 0.30502 |
| Osteobl | 1.037946 | 0.31356 |
| Chorion | 1.043205 | 0.32796 |

# Ulcerative colitis

| DHS sample | fold enrichment | p value |
|---|---|---|
| Th2 | 1.4929833 | 0.00002 |
| Th1 | 1.3326575 | 0.00016 |
| Th0 | 1.3216203 | 0.00036 |
| GM12865 | 1.3086964 | 0.00100 |
| HAEpiC | 1.2547699 | 0.00118 |
| MonocytesCD14RO01746 | 1.3414129 | 0.00138 |
| SAEC | 1.2118356 | 0.00170 |
| GM12864 | 1.3068433 | 0.00186 |
| GM19240 | 1.2671866 | 0.00220 |
| CD20 | 1.3643497 | 0.00236 |
| GM06990 | 1.3434420 | 0.00236 |
| PrEC | 1.1986805 | 0.00246 |
| NB4 | 1.2755275 | 0.00288 |
| HSMM | 1.1896896 | 0.00374 |
| GM18507 | 1.2710450 | 0.00448 |
| CD34Mobilized | 1.2512123 | 0.00520 |
| AoSMC | 1.1838135 | 0.00596 |
| HVMF | 1.2323829 | 0.00604 |
| GM19238 | 1.2646184 | 0.00676 |
| HSMMtube | 1.1893997 | 0.00798 |
| SKMC | 1.1928921 | 0.00812 |
| 8988T | 1.2919750 | 0.00920 |
| HCFaa | 1.1841776 | 0.01124 |
| RWPE1 | 1.1992892 | 0.01128 |
| GM12878 | 1.2381908 | 0.01244 |
| K562 | 1.2432619 | 0.01284 |
| NHEK | 1.1940458 | 0.01288 |
| HL60 | 1.2516787 | 0.01306 |
| HEEpiC | 1.1558567 | 0.01538 |
| HMF | 1.1742362 | 0.01668 |
| HRCEpiC | 1.1591322 | 0.01764 |
| HepG2 | 1.2164596 | 0.01790 |
| Melano | 1.1431743 | 0.01792 |
| HCPEpiC | 1.1598215 | 0.02018 |
| Stellate | 1.2050153 | 0.02036 |
| HPDE6E6E7 | 1.1901005 | 0.02062 |
| pHTE | 1.1846397 | 0.02178 |
| AG09309 | 1.1570561 | 0.02264 |
| HeLaS3 | 1.1998758 | 0.02304 |
| HRE | 1.1461047 | 0.02334 |
| HMEC | 1.1742873 | 0.02366 |
| GM19239 | 1.2221304 | 0.02538 |
| HBMEC | 1.1512613 | 0.02616 |
| Huh7 | 1.2054690 | 0.02730 |
| PanIsletD | 1.1579608 | 0.02738 |
| HTR8svn | 1.1793668 | 0.02790 |
| HPF | 1.1639824 | 0.02794 |
| GM12892 | 1.2249703 | 0.02824 |
| HPAF | 1.1518484 | 0.02956 |
| WI38 | 1.1630382 | 0.03016 |
| HIPEpiC | 1.1426401 | 0.03074 |
| CMK | 1.2212159 | 0.03186 |
| AG04450 | 1.1624561 | 0.03234 |
| Gliobla | 1.2005288 | 0.03338 |
| HNPCEpiC | 1.1353116 | 0.03378 |
| HSMMemb | 1.1668835 | 0.03516 |
| HFFMyc | 1.1408502 | 0.03554 |
| GM12891 | 1.2119483 | 0.03730 |
| MCF7 | 1.1696815 | 0.03820 |
| FibroP | 1.1249810 | 0.03836 |
| HCM | 1.1344639 | 0.03920 |
| HCF | 1.1490018 | 0.03944 |
| Fibrobl | 1.1545433 | 0.04090 |
| ProgFib | 1.1663714 | 0.04100 |
| Osteobl | 1.1580632 | 0.04190 |
| AG04449 | 1.1417235 | 0.04518 |
| NHLF | 1.1300947 | 0.04660 |
| PanIslets | 1.1644406 | 0.04726 |
| HFF | 1.1345142 | 0.04730 |
| NHA | 1.1305241 | 0.04798 |
| HeLaS3IFNa4h | 1.1837654 | 0.04868 |
| AoAF | 1.1338146 | 0.05484 |
| Caco2 | 1.2540203 | 0.05602 |
| CLL | 1.1807280 | 0.05646 |
| UrotheliaUT189 | 1.1545018 | 0.05816 |
| AG09319 | 1.1351756 | 0.06564 |
| Huh7.5 | 1.1614385 | 0.06724 |
| MCF7Hypoxia | 1.1612559 | 0.06868 |
| LNCaP | 1.1366835 | 0.07064 |
| RPTEC | 1.1043743 | 0.07154 |
| Chorion | 1.1785701 | 0.07352 |
| HMVECLBl | 1.1225958 | 0.07554 |
| Jurkat | 1.1282987 | 0.07666 |
| HGF | 1.1256307 | 0.07726 |
| Myometr | 1.1227709 | 0.07824 |
| HConF | 1.1196165 | 0.07884 |
| BJ | 1.1136965 | 0.08784 |
| PANC1 | 1.1262106 | 0.09266 |
| Hepatocytes | 1.1529194 | 0.09458 |
| iPS | 1.1579496 | 0.09860 |
| HAh | 1.0880075 | 0.10322 |
| HUVEC | 1.1160405 | 0.10400 |
| Urothelia | 1.1197285 | 0.10826 |
| H1hESC | 1.1328985 | 0.11428 |
| NHDFneo | 1.1014806 | 0.11526 |
| T47D | 1.1319170 | 0.11574 |
| HPdLF | 1.1012057 | 0.12144 |
| HAsp | 1.0931907 | 0.12462 |
| AG10803 | 1.0946906 | 0.13542 |
| IshikawaEstradiol | 1.1004091 | 0.13596 |
| HAc | 1.0828415 | 0.13728 |
| IshikawaTamoxifen | 1.1029381 | 0.13814 |
| NHDFAd | 1.0763442 | 0.16116 |
| H9ES | 1.0915750 | 0.17654 |
| HMVECdLyAd | 1.0823094 | 0.18470 |
| WERIRb1 | 1.0748774 | 0.18624 |
| HMVECdAd | 1.0824377 | 0.18886 |
| HCT116 | 1.0817746 | 0.19764 |
| LNCaPAndrogen | 1.0858479 | 0.20210 |
| HMVECLLy | 1.0750417 | 0.20228 |
| HMVECdBlAd | 1.0711638 | 0.20300 |
| HMVECdNeo | 1.0706876 | 0.21872 |
| NT2D1 | 1.0534814 | 0.23464 |
| HMVECdBlNeo | 1.0587717 | 0.24762 |
| SKNSHRA | 1.0757597 | 0.26336 |
| HMVECdLyNeo | 1.0545989 | 0.26864 |
| HRPEpiC | 1.0434529 | 0.27220 |
| HPAEC | 1.0515477 | 0.27370 |
| A549 | 1.0583622 | 0.27942 |
| HRGEC | 1.0440084 | 0.30044 |
| H7hESC | 1.0252260 | 0.33734 |
| Medullo | 1.0332943 | 0.36662 |
| BE2C | 1.0122880 | 0.44412 |
| SKNMC | 0.9355956 | 0.76790 |

## Celiac disease and rheumatoid arthritis

| DHS sample | fold enrichment | p value |
|---|---|---|
| Th2 | 2.3549959 | 0.00002 |
| Jurkat | 1.8422814 | 0.00002 |
| GM12864 | 1.7798677 | 0.00036 |
| K562 | 1.7791432 | 0.00068 |
| CD20 | 1.8365135 | 0.00126 |
| GM12865 | 1.6520452 | 0.00154 |
| Th0 | 1.5937325 | 0.00234 |
| GM06990 | 1.7447390 | 0.00284 |
| GM12878 | 1.5950039 | 0.00536 |
| CMK | 1.6622527 | 0.00544 |
| CD34Mobilized | 1.5258816 | 0.00552 |
| GM18507 | 1.5348857 | 0.00718 |
| SKNMC | 1.4481746 | 0.00786 |
| HL60 | 1.5320686 | 0.01468 |
| CLL | 1.5631108 | 0.01480 |
| HMVECdAd | 1.4271812 | 0.01792 |
| MonocytesCD14RO01746 | 1.5303608 | 0.01824 |
| HMVECdLyAd | 1.4108643 | 0.02028 |
| Th1 | 1.4359290 | 0.02080 |
| AoSMC | 1.3079542 | 0.02298 |
| HMVECdLyNeo | 1.3869910 | 0.02370 |
| HMVECdNeo | 1.3859440 | 0.02418 |
| GM19239 | 1.4704627 | 0.02494 |
| GM19240 | 1.3954461 | 0.02502 |
| GM19238 | 1.4494392 | 0.02586 |
| MCF7 | 1.3799551 | 0.02640 |
| SAEC | 1.2944789 | 0.02828 |
| HMF | 1.3225460 | 0.02856 |
| HIPEpiC | 1.3144480 | 0.02912 |
| HPAEC | 1.3481530 | 0.03354 |
| NB4 | 1.3944225 | 0.03366 |
| HMVECLLy | 1.3564394 | 0.03372 |
| AG10803 | 1.3291317 | 0.03642 |
| HRE | 1.2800738 | 0.03660 |
| HEEpiC | 1.2725413 | 0.03706 |
| AG04449 | 1.3130477 | 0.03876 |
| Urothelia | 1.3620863 | 0.04106 |
| HMVECLBl | 1.3210023 | 0.04246 |
| HRCEpiC | 1.2792334 | 0.04316 |
| HCM | 1.2785592 | 0.04636 |
| AG04450 | 1.3038488 | 0.04640 |
| HCFaa | 1.2913102 | 0.04654 |
| RPTEC | 1.2525518 | 0.04838 |
| BJ | 1.2916773 | 0.05000 |
| HMVECdBlAd | 1.3127935 | 0.05088 |
| HCPEpiC | 1.2711531 | 0.05096 |
| NT2D1 | 1.2556101 | 0.05130 |
| HCF | 1.2950955 | 0.05178 |
| UrotheliaUT189 | 1.3477000 | 0.05246 |
| IshikawaTamoxifen | 1.3116816 | 0.05598 |
| Huh7 | 1.3633747 | 0.05618 |
| HPDE6E6E7 | 1.2966984 | 0.05832 |
| A549 | 1.3406220 | 0.05854 |
| PANC1 | 1.3264787 | 0.05942 |
| HRGEC | 1.2951693 | 0.05950 |
| HConF | 1.2660252 | 0.06088 |
| HAEpiC | 1.2699479 | 0.06146 |
| HPAF | 1.2611055 | 0.06158 |
| HMVECdBlNeo | 1.2872568 | 0.06358 |
| HTR8svn | 1.2961903 | 0.06432 |
| MCF7Hypoxia | 1.3558988 | 0.06568 |
| IshikawaEstradiol | 1.2799891 | 0.06776 |
| HGF | 1.2675372 | 0.07122 |
| AG09319 | 1.2622951 | 0.07220 |
| WI38 | 1.2677417 | 0.07528 |
| HNPCEpiC | 1.2243918 | 0.07536 |
| NHA | 1.2390883 | 0.07722 |
| AG09309 | 1.2382377 | 0.07778 |
| HFF | 1.2359320 | 0.07810 |
| HUVEC | 1.2643909 | 0.08126 |
| NHLF | 1.2295925 | 0.08154 |
| NHDFneo | 1.2425775 | 0.08314 |
| SKMC | 1.2240440 | 0.08582 |
| HAsp | 1.2297269 | 0.08724 |
| AoAF | 1.2348646 | 0.08856 |
| HVMF | 1.2496719 | 0.09942 |
| RWPE1 | 1.2245584 | 0.10802 |
| Huh7.5 | 1.2776133 | 0.11028 |
| PrEC | 1.1892227 | 0.11068 |
| Stellate | 1.2438171 | 0.11598 |
| HPdLF | 1.2136149 | 0.11908 |
| PanIsletD | 1.1914491 | 0.12414 |
| GM12892 | 1.2859349 | 0.12780 |
| HAh | 1.1680718 | 0.12838 |
| HPF | 1.1962099 | 0.13000 |
| Gliobla | 1.2503165 | 0.13098 |
| HSMM | 1.1631264 | 0.13594 |
| HBMEC | 1.1749734 | 0.13600 |
| H7hESC | 1.1269183 | 0.14238 |
| GM12891 | 1.2659594 | 0.14270 |
| HeLaS3 | 1.2103923 | 0.14284 |
| NHEK | 1.1885050 | 0.14498 |
| LNCaPAndrogen | 1.2064643 | 0.16460 |
| HepG2 | 1.2115788 | 0.16478 |
| FibroP | 1.1475641 | 0.16518 |
| NHDFAd | 1.1527009 | 0.17026 |
| Medullo | 1.1995875 | 0.17130 |
| T47D | 1.2052265 | 0.17888 |
| Melano | 1.1291043 | 0.18476 |
| HeLaS3IFNa4h | 1.1937108 | 0.18594 |
| H9ES | 1.1627462 | 0.19690 |
| HSMMemb | 1.1586454 | 0.19922 |
| HCT116 | 1.1626843 | 0.20028 |
| BE2C | 1.1558337 | 0.20628 |
| HFFMyc | 1.1286784 | 0.21678 |
| HAc | 1.1075054 | 0.23734 |
| Myometr | 1.1182679 | 0.24296 |
| HSMMtube | 1.1080234 | 0.24860 |
| HRPEpiC | 1.0919410 | 0.26394 |
| HMEC | 1.1058440 | 0.27558 |
| ProgFib | 1.0837505 | 0.32278 |
| PanIslets | 1.0901141 | 0.32974 |
| WERIRb1 | 1.0673239 | 0.33592 |
| Fibrobl | 1.0773230 | 0.34498 |
| pHTE | 1.0732947 | 0.34594 |
| Osteobl | 1.0696803 | 0.36094 |
| 8988T | 1.0724523 | 0.37310 |
| LNCaP | 1.0385357 | 0.40308 |
| H1hESC | 1.0271654 | 0.43430 |
| Chorion | 1.0197671 | 0.44830 |
| Caco2 | 1.0140694 | 0.45052 |
| Hepatocytes | 1.0196287 | 0.45110 |
| SKNSHRA | 1.0024294 | 0.47030 |
| iPS | 0.9731587 | 0.52892 |

# Biliary cirrhosis

| DHS sample | fold enrichment | p value |
|---|---|---|
| GM06990 | 2.0072038 | 0.00002 |
| GM12865 | 1.9994829 | 0.00002 |
| GM12864 | 1.9313258 | 0.00002 |
| GM12878 | 1.7774663 | 0.00002 |
| MonocytesCD14RO01746 | 1.7268939 | 0.00004 |
| CD20 | 1.8187128 | 0.00016 |
| GM18507 | 1.6489942 | 0.00020 |
| CD34Mobilized | 1.5456904 | 0.00038 |
| Th2 | 1.6161621 | 0.00058 |
| RPTEC | 1.3857832 | 0.00060 |
| GM19240 | 1.4963257 | 0.00084 |
| GM19238 | 1.5356445 | 0.00142 |
| HRCEpiC | 1.3840039 | 0.00168 |
| GM19239 | 1.5506133 | 0.00212 |
| HTR8svn | 1.4363613 | 0.00308 |
| HMVECLBl | 1.3704137 | 0.00522 |
| HRE | 1.3249429 | 0.00524 |
| CLL | 1.4927447 | 0.00602 |
| AoSMC | 1.2977895 | 0.00882 |
| HFFMyc | 1.3126385 | 0.00928 |
| HL60 | 1.4172609 | 0.01084 |
| AG04450 | 1.3326566 | 0.01180 |
| HCT116 | 1.3713483 | 0.01260 |
| Th0 | 1.3593335 | 0.01280 |
| HMVECdLyNeo | 1.3385265 | 0.01286 |
| HIPEpiC | 1.2885078 | 0.01288 |
| Urothelia | 1.3485863 | 0.01318 |
| HPAF | 1.2950917 | 0.01368 |
| NT2D1 | 1.2803639 | 0.01370 |
| WI38 | 1.3346372 | 0.01372 |
| HCM | 1.2822946 | 0.01488 |
| NB4 | 1.3546780 | 0.01490 |
| HPdLF | 1.3191387 | 0.01580 |
| AG09319 | 1.3223757 | 0.01592 |
| HRGEC | 1.3121128 | 0.01612 |
| HMVECdLyAd | 1.3298363 | 0.01628 |
| HMVECdBlAd | 1.3075099 | 0.01666 |
| HPF | 1.2996234 | 0.01744 |
| HCFaa | 1.2866979 | 0.01880 |
| HMVECLLy | 1.3152564 | 0.01942 |
| GM12892 | 1.3858898 | 0.01974 |
| Jurkat | 1.3149392 | 0.02156 |
| HConF | 1.2879423 | 0.02178 |
| SKMC | 1.2751153 | 0.02208 |
| HCF | 1.2854370 | 0.02220 |
| GM12891 | 1.3879051 | 0.02324 |
| BJ | 1.2922893 | 0.02340 |
| HUVEC | 1.2992827 | 0.02382 |
| AG09309 | 1.2631433 | 0.02390 |
| HNPCEpiC | 1.2435723 | 0.02474 |
| HMVECdAd | 1.3099550 | 0.02482 |
| HFF | 1.2652409 | 0.02524 |
| AoAF | 1.2732338 | 0.02528 |
| NHDFneo | 1.2818088 | 0.02534 |
| IshikawaEstradiol | 1.3047538 | 0.02566 |
| MCF7 | 1.3009204 | 0.02620 |
| HVMF | 1.2982656 | 0.02686 |
| HGF | 1.2884681 | 0.02734 |
| Stellate | 1.3023336 | 0.03160 |
| NHA | 1.2431401 | 0.03162 |
| HAEpiC | 1.2593292 | 0.03186 |
| Th1 | 1.2905333 | 0.03208 |
| HCPEpiC | 1.2390993 | 0.03244 |
| HMVECdBlNeo | 1.2694974 | 0.03372 |
| HSMM | 1.2278464 | 0.03460 |
| HEEpiC | 1.2148977 | 0.03572 |
| SAEC | 1.2113355 | 0.03630 |
| HAh | 1.2096698 | 0.03632 |
| HMVECdNeo | 1.2721712 | 0.03776 |
| AG10803 | 1.2577458 | 0.03888 |
| HeLaS3 | 1.2888903 | 0.03922 |
| HPAEC | 1.2528693 | 0.04064 |
| NHLF | 1.2235142 | 0.04222 |
| AG04449 | 1.2460228 | 0.04302 |
| PanIsletD | 1.2270366 | 0.04462 |
| PrEC | 1.2015129 | 0.04484 |
| CMK | 1.3209069 | 0.04492 |
| H9ES | 1.2708125 | 0.04674 |
| A549 | 1.2831515 | 0.04724 |
| K562 | 1.2967513 | 0.04784 |
| NHEK | 1.2352616 | 0.04932 |
| Myometr | 1.2339422 | 0.04944 |
| NHDFAd | 1.2168828 | 0.05050 |
| HBMEC | 1.2075927 | 0.05160 |
| Gliobla | 1.2738755 | 0.05182 |
| HPDE6E6E7 | 1.2452828 | 0.05320 |
| HMF | 1.2179670 | 0.05480 |
| HAsp | 1.2130125 | 0.05640 |
| IshikawaTamoxifen | 1.2387376 | 0.06404 |
| Huh7.5 | 1.2674939 | 0.06444 |
| ProgFib | 1.2333881 | 0.06544 |
| Huh7 | 1.2555738 | 0.07242 |
| HAc | 1.1777723 | 0.07598 |
| HSMMtube | 1.1836370 | 0.07648 |
| Melano | 1.1567044 | 0.08082 |
| MCF7Hypoxia | 1.2365183 | 0.08760 |
| BE2C | 1.2180619 | 0.08816 |
| HepG2 | 1.2223762 | 0.08886 |
| PANC1 | 1.2188667 | 0.08888 |
| HSMMemb | 1.1980094 | 0.09240 |
| RWPE1 | 1.1921520 | 0.09274 |
| Caco2 | 1.3242312 | 0.09528 |
| UrotheliaUT189 | 1.2012327 | 0.10016 |
| FibroP | 1.1478187 | 0.10068 |
| HRPEpiC | 1.1557448 | 0.10304 |
| HeLaS3IFNa4h | 1.2151882 | 0.10974 |
| H1hESC | 1.2067646 | 0.11500 |
| H7hESC | 1.1130942 | 0.13184 |
| SKNMC | 1.1578416 | 0.13680 |
| HMEC | 1.1473820 | 0.14412 |
| T47D | 1.1785029 | 0.15646 |
| Chorion | 1.1896213 | 0.15866 |
| pHTE | 1.1469606 | 0.15946 |
| LNCaP | 1.1387931 | 0.17020 |
| PanIslets | 1.1418468 | 0.17828 |
| LNCaPAndrogen | 1.1448268 | 0.18866 |
| Hepatocytes | 1.1522220 | 0.19890 |
| Fibrobl | 1.1213089 | 0.20160 |
| SKNSHRA | 1.1564410 | 0.20618 |
| iPS | 1.1523280 | 0.21074 |
| 8988T | 1.1526762 | 0.21116 |
| Osteobl | 1.1179188 | 0.21518 |
| WERIRb1 | 1.0802965 | 0.27910 |
| Medullo | 0.9948834 | 0.50946 |

# Type 1 diabetes − autoantibody positive

| DHS sample | fold enrichment | p value |
|---|---|---|
| Th2 | 1.9525961 | 0.00002 |
| GM12864 | 1.5198736 | 0.00130 |
| GM12865 | 1.5006754 | 0.00160 |
| GM06990 | 1.5521202 | 0.00334 |
| Jurkat | 1.4262439 | 0.00370 |
| CD20 | 1.5374483 | 0.00718 |
| GM12878 | 1.3976152 | 0.01716 |
| Th0 | 1.3500778 | 0.01772 |
| GM18507 | 1.3678940 | 0.02016 |
| MonocytesCD14RO01746 | 1.3976635 | 0.02064 |
| CLL | 1.3833750 | 0.02712 |
| Th1 | 1.2864620 | 0.03678 |
| NB4 | 1.2905182 | 0.03752 |
| CD34Mobilized | 1.2781598 | 0.04784 |
| GM19240 | 1.2621711 | 0.05526 |
| HL60 | 1.3101660 | 0.05670 |
| AG04450 | 1.2204014 | 0.07326 |
| GM19238 | 1.2543201 | 0.08250 |
| HIPEpiC | 1.1772051 | 0.09046 |
| HEEpiC | 1.1599840 | 0.09982 |
| HCM | 1.1660747 | 0.10032 |
| GM19239 | 1.2428873 | 0.10434 |
| HAEpiC | 1.1790070 | 0.10968 |
| Stellate | 1.2064647 | 0.11084 |
| NHA | 1.1659059 | 0.12044 |
| K562 | 1.2105043 | 0.12280 |
| HPF | 1.1698708 | 0.12534 |
| SAEC | 1.1404145 | 0.12648 |
| WI38 | 1.1695492 | 0.13218 |
| HFFMyc | 1.1440122 | 0.13290 |
| HBMEC | 1.1399421 | 0.14440 |
| SKMC | 1.1405440 | 0.14818 |
| HFF | 1.1404279 | 0.15056 |
| HNPCEpiC | 1.1310726 | 0.15346 |
| HPAF | 1.1313883 | 0.16704 |
| NHLF | 1.1323214 | 0.16770 |
| HCF | 1.1340918 | 0.17296 |
| HSMM | 1.1164876 | 0.17682 |
| AoSMC | 1.1133492 | 0.17964 |
| HGF | 1.1362251 | 0.18262 |
| CMK | 1.1750073 | 0.18744 |
| MCF7 | 1.1402637 | 0.18850 |
| Huh7 | 1.1577095 | 0.18888 |
| HPdLF | 1.1303461 | 0.18984 |
| HPDE6E6E7 | 1.1378120 | 0.19058 |
| NHDFneo | 1.1215399 | 0.20164 |
| Urothelia | 1.1334104 | 0.20380 |
| GM12891 | 1.1595487 | 0.21018 |
| HCPEpiC | 1.1054243 | 0.21038 |
| BJ | 1.1142329 | 0.21262 |
| RWPE1 | 1.1090749 | 0.22308 |
| MCF7Hypoxia | 1.1360141 | 0.22442 |
| HCFaa | 1.1010261 | 0.22698 |
| HMF | 1.0992061 | 0.22770 |
| PrEC | 1.0875708 | 0.23516 |
| AG09309 | 1.0903551 | 0.24162 |
| HConF | 1.0971827 | 0.24314 |
| HVMF | 1.1050571 | 0.24336 |
| HAc | 1.0879353 | 0.24466 |
| HRCEpiC | 1.0880107 | 0.25120 |
| HAh | 1.0767384 | 0.26030 |
| PanIsletD | 1.0837306 | 0.26988 |
| AG10803 | 1.0880536 | 0.26994 |
| Huh7.5 | 1.1074317 | 0.27024 |
| AG04449 | 1.0859341 | 0.27050 |
| AoAF | 1.0856286 | 0.27278 |
| HTR8svn | 1.0918198 | 0.27482 |
| HSMMemb | 1.0910542 | 0.27878 |
| GM12892 | 1.1121988 | 0.28054 |
| AG09319 | 1.0823300 | 0.28876 |
| HMVECdLyAd | 1.0845218 | 0.28892 |
| HMVECdNeo | 1.0818274 | 0.29292 |
| HMVECLBl | 1.0777681 | 0.29418 |
| HMVECdAd | 1.0789008 | 0.30178 |
| HMVECLLy | 1.0743996 | 0.30934 |
| HUVEC | 1.0717290 | 0.31464 |
| HCT116 | 1.0711571 | 0.31476 |
| HPAEC | 1.0703649 | 0.31494 |
| HepG2 | 1.0770700 | 0.32048 |
| HRE | 1.0591742 | 0.32434 |
| HAsp | 1.0599859 | 0.32724 |
| HMVECdBlNeo | 1.0618862 | 0.33248 |
| Myometr | 1.0563245 | 0.34278 |
| HRGEC | 1.0512315 | 0.35718 |
| HSMMtube | 1.0461187 | 0.36012 |
| IshikawaEstradiol | 1.0482418 | 0.36684 |
| SKNMC | 1.0445900 | 0.36976 |
| PANC1 | 1.0462124 | 0.37262 |
| HMVECdBlAd | 1.0414036 | 0.38372 |
| NT2D1 | 1.0336283 | 0.38418 |
| NHEK | 1.0358697 | 0.39468 |
| RPTEC | 1.0305793 | 0.39854 |
| H9ES | 1.0292577 | 0.41636 |
| 8988T | 1.0344772 | 0.41984 |
| NHDFAd | 1.0229025 | 0.42740 |
| HMVECdLyNeo | 1.0252793 | 0.42956 |
| Gliobla | 1.0255445 | 0.43108 |
| Fibrobl | 1.0187244 | 0.45064 |
| Medullo | 1.0185283 | 0.45104 |
| PanIslets | 1.0161491 | 0.45396 |
| pHTE | 1.0121637 | 0.46076 |
| FibroP | 1.0119529 | 0.46090 |
| IshikawaTamoxifen | 1.0104906 | 0.46196 |
| HMEC | 1.0102997 | 0.46540 |
| Hepatocytes | 0.9975398 | 0.48774 |
| UrotheliaUT189 | 0.9945027 | 0.50346 |
| SKNSHRA | 0.9820052 | 0.51258 |
| ProgFib | 0.9868192 | 0.52022 |
| BE2C | 0.9819736 | 0.53166 |
| HeLaS3IFNa4h | 0.9732909 | 0.54118 |
| LNCaPAndrogen | 0.9710771 | 0.54714 |
| T47D | 0.9692754 | 0.55102 |
| Caco2 | 0.9509454 | 0.55358 |
| Osteobl | 0.9694495 | 0.57540 |
| iPS | 0.9520484 | 0.57720 |
| Melano | 0.9753167 | 0.58206 |
| Chorion | 0.9478298 | 0.58388 |
| H1hESC | 0.9447750 | 0.60036 |
| LNCaP | 0.9503614 | 0.61314 |
| HeLaS3 | 0.9426344 | 0.62096 |
| A549 | 0.9409526 | 0.62724 |
| H7hESC | 0.9649259 | 0.62804 |
| HRPEpiC | 0.9501013 | 0.65100 |
| WERIRb1 | 0.9278549 | 0.69610 |

# Multiple sclerosis

| DHS sample | fold enrichment | p value |
|---|---|---|
| Th2 | 1.949430 | 0.00002 |
| GM06990 | 1.791461 | 0.00002 |
| CD20 | 1.712251 | 0.00002 |
| GM12865 | 1.668143 | 0.00002 |
| GM12864 | 1.653555 | 0.00002 |
| MonocytesCD14RO01746 | 1.630810 | 0.00002 |
| GM12878 | 1.573774 | 0.00002 |
| CD34Mobilized | 1.419081 | 0.00002 |
| NB4 | 1.415170 | 0.00002 |
| GM18507 | 1.449134 | 0.00004 |
| Jurkat | 1.383747 | 0.00004 |
| HL60 | 1.454853 | 0.00008 |
| SAEC | 1.265699 | 0.00014 |
| HMVECLBl | 1.295141 | 0.00020 |
| CLL | 1.436310 | 0.00022 |
| HIPEpiC | 1.284762 | 0.00022 |
| HAEpiC | 1.298402 | 0.00028 |
| Th1 | 1.307691 | 0.00038 |
| RPTEC | 1.237539 | 0.00038 |
| HMVECdBlAd | 1.284464 | 0.00042 |
| HEEpiC | 1.247843 | 0.00046 |
| AoSMC | 1.243551 | 0.00050 |
| Th0 | 1.323066 | 0.00054 |
| HPAF | 1.267764 | 0.00054 |
| HCFaa | 1.263397 | 0.00056 |
| K562 | 1.355092 | 0.00058 |
| HCM | 1.247476 | 0.00058 |
| HRCEpiC | 1.244580 | 0.00058 |
| HMVECdAd | 1.303556 | 0.00062 |
| HPF | 1.288035 | 0.00068 |
| HTR8svn | 1.294634 | 0.00070 |
| HPAEC | 1.281563 | 0.00076 |
| HRE | 1.229885 | 0.00080 |
| AG04450 | 1.281794 | 0.00090 |
| HGF | 1.276628 | 0.00090 |
| HPdLF | 1.262679 | 0.00090 |
| HCF | 1.265756 | 0.00096 |
| HFFMyc | 1.238479 | 0.00098 |
| PrEC | 1.218204 | 0.00110 |
| HRGEC | 1.255212 | 0.00124 |
| HNPCEpiC | 1.221389 | 0.00124 |
| AoAF | 1.255937 | 0.00132 |
| HCPEpiC | 1.234210 | 0.00136 |
| AG09319 | 1.259019 | 0.00140 |
| HMF | 1.239638 | 0.00150 |
| HAsp | 1.236737 | 0.00152 |
| NHA | 1.229945 | 0.00178 |
| HMVECLLy | 1.268015 | 0.00184 |
| HMVECdLyAd | 1.276038 | 0.00200 |
| GM19239 | 1.331684 | 0.00210 |
| GM19238 | 1.310440 | 0.00214 |
| HMVECdBlNeo | 1.253703 | 0.00218 |
| AG09309 | 1.221585 | 0.00220 |
| HMVECdLyNeo | 1.261406 | 0.00230 |
| WI38 | 1.253361 | 0.00232 |
| HBMEC | 1.216916 | 0.00236 |
| HPDE6E6E7 | 1.271835 | 0.00242 |
| GM19240 | 1.268125 | 0.00266 |
| HMVECdNeo | 1.261582 | 0.00280 |
| CMK | 1.335645 | 0.00282 |
| HConF | 1.228823 | 0.00376 |
| Urothelia | 1.255040 | 0.00380 |
| NHLF | 1.210199 | 0.00388 |
| HVMF | 1.244452 | 0.00410 |
| AG04449 | 1.215401 | 0.00478 |
| HCT116 | 1.252470 | 0.00562 |
| HAc | 1.186747 | 0.00570 |
| SKMC | 1.200599 | 0.00572 |
| HFF | 1.200229 | 0.00592 |
| BJ | 1.211296 | 0.00632 |
| NHDFneo | 1.205704 | 0.00692 |
| SKNMC | 1.222026 | 0.00750 |
| NHDFAd | 1.183476 | 0.00824 |
| HUVEC | 1.223880 | 0.00938 |
| NHEK | 1.206321 | 0.00952 |
| Huh7 | 1.247891 | 0.01054 |
| RWPE1 | 1.202004 | 0.01170 |
| AG10803 | 1.185420 | 0.01548 |
| HAh | 1.150241 | 0.01566 |
| A549 | 1.223181 | 0.01662 |
| MCF7 | 1.194461 | 0.02236 |
| PANC1 | 1.197566 | 0.02270 |
| Stellate | 1.201789 | 0.02302 |
| HeLaS3IFNa4h | 1.218050 | 0.02764 |
| UrotheliaUT189 | 1.186342 | 0.02780 |
| HSMM | 1.138031 | 0.02810 |
| IshikawaEstradiol | 1.169178 | 0.03128 |
| HRPEpiC | 1.131203 | 0.03340 |
| Myometr | 1.155401 | 0.03416 |
| GM12891 | 1.213271 | 0.03658 |
| Huh7.5 | 1.190016 | 0.04152 |
| GM12892 | 1.203994 | 0.04166 |
| PanIsletD | 1.139799 | 0.04198 |
| FibroP | 1.119938 | 0.04558 |
| HepG2 | 1.173199 | 0.04686 |
| IshikawaTamoxifen | 1.149058 | 0.05554 |
| HeLaS3 | 1.155484 | 0.05862 |
| MCF7Hypoxia | 1.170724 | 0.06314 |
| ProgFib | 1.136278 | 0.07298 |
| HSMMemb | 1.130786 | 0.07442 |
| HSMMtube | 1.100078 | 0.09666 |
| BE2C | 1.117606 | 0.11840 |
| HMEC | 1.102480 | 0.11846 |
| pHTE | 1.102108 | 0.13280 |
| Melano | 1.074217 | 0.13812 |
| H7hESC | 1.055957 | 0.16910 |
| NT2D1 | 1.063184 | 0.18862 |
| T47D | 1.094968 | 0.19362 |
| Gliobla | 1.089005 | 0.19760 |
| H9ES | 1.079046 | 0.20106 |
| Caco2 | 1.111971 | 0.22910 |
| Hepatocytes | 1.076317 | 0.24438 |
| Fibrobl | 1.058555 | 0.25726 |
| LNCaPAndrogen | 1.063161 | 0.26504 |
| PanIslets | 1.051198 | 0.29572 |
| SKNSHRA | 1.060933 | 0.29614 |
| LNCaP | 1.043031 | 0.30904 |
| Osteobl | 1.042471 | 0.32130 |
| 8988T | 1.042073 | 0.35338 |
| Chorion | 1.037440 | 0.37116 |
| WERIRb1 | 1.021720 | 0.39470 |
| H1hESC | 1.013932 | 0.43680 |
| Medullo | 1.006996 | 0.46652 |
| iPS | 1.007342 | 0.46656 |

# Liver enzymes in plasma

| DHS sample | fold enrichment | p value |
|---|---|---|
| RPTEC | 1.3579542 | 0.00002 |
| HRCEpiC | 1.3190878 | 0.00002 |
| HRE | 1.2442359 | 0.00054 |
| HMVECdAd | 1.2854978 | 0.00158 |
| HIPEpiC | 1.2253587 | 0.00170 |
| HMVECdBlAd | 1.2608269 | 0.00178 |
| HEEpiC | 1.2110716 | 0.00198 |
| HMVECdBlNeo | 1.2613939 | 0.00224 |
| HCPEpiC | 1.2239709 | 0.00256 |
| HRGEC | 1.2385423 | 0.00296 |
| HMVECLLy | 1.2535239 | 0.00302 |
| PrEC | 1.1961119 | 0.00322 |
| SAEC | 1.2002249 | 0.00326 |
| HMVECdLyNeo | 1.2438234 | 0.00402 |
| HAc | 1.1961059 | 0.00424 |
| HPAEC | 1.2387390 | 0.00446 |
| HNPCEpiC | 1.1968737 | 0.00448 |
| HCFaa | 1.2135406 | 0.00460 |
| HMVECdLyAd | 1.2510619 | 0.00474 |
| WI38 | 1.2358387 | 0.00482 |
| AG04450 | 1.2313725 | 0.00530 |
| HAh | 1.1760916 | 0.00574 |
| HMVECdNeo | 1.2380345 | 0.00580 |
| NHLF | 1.1964956 | 0.00612 |
| PANC1 | 1.2647177 | 0.00614 |
| HCT116 | 1.2557481 | 0.00692 |
| HCF | 1.2162216 | 0.00700 |
| HAsp | 1.1944288 | 0.00884 |
| HBMEC | 1.1790783 | 0.01018 |
| HRPEpiC | 1.1708047 | 0.01052 |
| HPAF | 1.1878001 | 0.01180 |
| HAEpiC | 1.1910940 | 0.01200 |
| SKMC | 1.1823167 | 0.01270 |
| NHA | 1.1735762 | 0.01326 |
| AoSMC | 1.1634182 | 0.01376 |
| HPdLF | 1.1938344 | 0.01424 |
| HPDE6E6E7 | 1.2137908 | 0.01490 |
| IshikawaEstradiol | 1.1980273 | 0.01514 |
| AG04449 | 1.1769579 | 0.01652 |
| HCM | 1.1696775 | 0.01690 |
| BJ | 1.1812007 | 0.01692 |
| AG09309 | 1.1639986 | 0.01836 |
| HMVECLBl | 1.1801114 | 0.01980 |
| HGF | 1.1829564 | 0.02016 |
| A549 | 1.2176432 | 0.02020 |
| HMF | 1.1634150 | 0.02206 |
| AG09319 | 1.1702377 | 0.02744 |
| SKNMC | 1.1736366 | 0.02748 |
| NHDFAd | 1.1468386 | 0.03002 |
| HFF | 1.1499754 | 0.03076 |
| HFFMyc | 1.1447496 | 0.03126 |
| BE2C | 1.1788271 | 0.03146 |
| NHEK | 1.1627383 | 0.03226 |
| AG10803 | 1.1601534 | 0.03270 |
| HConF | 1.1540569 | 0.03614 |
| IshikawaTamoxifen | 1.1702370 | 0.03674 |
| HeLaS3 | 1.1783943 | 0.03852 |
| H7hESC | 1.1039232 | 0.04594 |
| Huh7 | 1.1806516 | 0.04706 |
| HeLaS3IFNa4h | 1.1869097 | 0.04760 |
| HTR8svn | 1.1566310 | 0.04898 |
| HUVEC | 1.1533966 | 0.05112 |
| RWPE1 | 1.1487463 | 0.05176 |
| HSMMemb | 1.1517029 | 0.05236 |
| Myometr | 1.1390917 | 0.05642 |
| HVMF | 1.1457434 | 0.05654 |
| AoAF | 1.1338157 | 0.05726 |
| HepG2 | 1.1608077 | 0.05934 |
| GM12865 | 1.1503936 | 0.06032 |
| HPF | 1.1304104 | 0.06570 |
| GM12878 | 1.1542519 | 0.06982 |
| GM18507 | 1.1438720 | 0.07892 |
| HSMM | 1.1023069 | 0.08212 |
| GM06990 | 1.1568825 | 0.08440 |
| Stellate | 1.1315170 | 0.09350 |
| Huh7.5 | 1.1347968 | 0.10790 |
| PanIsletD | 1.0988502 | 0.11102 |
| NHDFneo | 1.1044824 | 0.11294 |
| GM12864 | 1.1180178 | 0.11812 |
| FibroP | 1.0828481 | 0.12344 |
| Jurkat | 1.0995922 | 0.13014 |
| MCF7 | 1.1059361 | 0.13672 |
| Gliobla | 1.1086256 | 0.15156 |
| CMK | 1.1158339 | 0.15726 |
| Melano | 1.0681402 | 0.16102 |
| ProgFib | 1.0920474 | 0.16520 |
| Urothelia | 1.0856248 | 0.17824 |
| K562 | 1.0940521 | 0.18796 |
| LNCaPAndrogen | 1.0906398 | 0.18972 |
| HSMMtube | 1.0669778 | 0.19592 |
| UrotheliaUT189 | 1.0804712 | 0.19644 |
| WERIRb1 | 1.0692743 | 0.19870 |
| PanIslets | 1.0798597 | 0.20486 |
| Th2 | 1.0859534 | 0.21892 |
| GM19239 | 1.0841377 | 0.22066 |
| HMEC | 1.0665362 | 0.22068 |
| SKNSHRA | 1.0892389 | 0.22396 |
| GM19240 | 1.0709366 | 0.22484 |
| MCF7Hypoxia | 1.0760673 | 0.24162 |
| HL60 | 1.0750027 | 0.24264 |
| GM19238 | 1.0723108 | 0.24580 |
| T47D | 1.0749483 | 0.24802 |
| H9ES | 1.0626907 | 0.25340 |
| pHTE | 1.0594960 | 0.25462 |
| CD34Mobilized | 1.0597017 | 0.26044 |
| LNCaP | 1.0513499 | 0.27960 |
| Hepatocytes | 1.0592824 | 0.29976 |
| Th0 | 1.0475833 | 0.31078 |
| NB4 | 1.0465949 | 0.31378 |
| Medullo | 1.0429809 | 0.32598 |
| Fibrobl | 1.0393524 | 0.32784 |
| GM12891 | 1.0462243 | 0.34074 |
| Osteobl | 1.0335227 | 0.35588 |
| CD20 | 1.0280734 | 0.39864 |
| MonocytesCD14RO01746 | 1.0223220 | 0.41372 |
| H1hESC | 1.0186510 | 0.42104 |
| GM12892 | 1.0203927 | 0.42254 |
| NT2D1 | 1.0129602 | 0.42504 |
| Chorion | 1.0189907 | 0.42636 |
| CLL | 1.0163438 | 0.43646 |
| Th1 | 1.0077124 | 0.46670 |
| Caco2 | 0.9924080 | 0.50482 |
| 8988T | 0.9779354 | 0.56172 |
| iPS | 0.9756973 | 0.57348 |

# Inflammatory bowel disease

| DHS sample | fold enrichment | p value |
|---|---|---|
| Th2 | 1.5605672 | 0.00002 |
| MonocytesCD14RO01746 | 1.4522236 | 0.00002 |
| GM06990 | 1.3978133 | 0.00002 |
| CD20 | 1.3748379 | 0.00002 |
| GM12864 | 1.3704559 | 0.00002 |
| GM12865 | 1.3697360 | 0.00002 |
| HL60 | 1.3530555 | 0.00002 |
| NB4 | 1.3195411 | 0.00002 |
| GM12878 | 1.3022969 | 0.00002 |
| GM18507 | 1.2858764 | 0.00002 |
| HAEpiC | 1.2664704 | 0.00002 |
| HCFaa | 1.2545686 | 0.00002 |
| K562 | 1.2530956 | 0.00002 |
| PANC1 | 1.2513551 | 0.00002 |
| HVMF | 1.2476648 | 0.00002 |
| AG04450 | 1.2458335 | 0.00002 |
| Jurkat | 1.2419438 | 0.00002 |
| CD34Mobilized | 1.2403363 | 0.00002 |
| WI38 | 1.2381576 | 0.00002 |
| HBMEC | 1.2313508 | 0.00002 |
| SAEC | 1.2256857 | 0.00002 |
| Th0 | 1.2252290 | 0.00002 |
| Th1 | 1.2232685 | 0.00002 |
| AG04449 | 1.2217069 | 0.00002 |
| HIPEpiC | 1.2208245 | 0.00002 |
| HPdLF | 1.2204170 | 0.00002 |
| HCPEpiC | 1.2188435 | 0.00002 |
| BJ | 1.2187719 | 0.00002 |
| HRCEpiC | 1.2180408 | 0.00002 |
| HPF | 1.2153037 | 0.00002 |
| HMF | 1.2118561 | 0.00002 |
| SKMC | 1.2112358 | 0.00002 |
| AG09309 | 1.2110332 | 0.00002 |
| HNPCEpiC | 1.2105244 | 0.00002 |
| HEEpiC | 1.2093627 | 0.00002 |
| HGF | 1.2071753 | 0.00002 |
| AoAF | 1.2043194 | 0.00002 |
| AG09319 | 1.2039572 | 0.00002 |
| AoSMC | 1.2031043 | 0.00002 |
| HRE | 1.2027713 | 0.00002 |
| HPAF | 1.2027458 | 0.00002 |
| NHLF | 1.2010558 | 0.00002 |
| PrEC | 1.2009768 | 0.00002 |
| RWPE1 | 1.2006691 | 0.00002 |
| NHA | 1.1989755 | 0.00002 |
| HAc | 1.1673876 | 0.00002 |
| HAsp | 1.2193941 | 0.00004 |
| HCF | 1.1960134 | 0.00004 |
| AG10803 | 1.1919012 | 0.00004 |
| HCM | 1.1863460 | 0.00004 |
| RPTEC | 1.1793144 | 0.00004 |
| NHDFAd | 1.1700476 | 0.00004 |
| HFF | 1.1895647 | 0.00006 |
| NHDFneo | 1.1800161 | 0.00006 |
| HFFMyc | 1.1726752 | 0.00006 |
| CMK | 1.2367787 | 0.00008 |
| HPDE6E6E7 | 1.2098762 | 0.00008 |
| HMVECLBl | 1.1901862 | 0.00010 |
| HRGEC | 1.1768436 | 0.00010 |
| HAh | 1.1387978 | 0.00010 |
| HeLaS3 | 1.1953042 | 0.00012 |
| HConF | 1.1675559 | 0.00014 |
| HTR8svn | 1.1951967 | 0.00016 |
| HPAEC | 1.1730737 | 0.00018 |
| HMVECdBlAd | 1.1602649 | 0.00022 |
| GM19238 | 1.2075242 | 0.00026 |
| HCT116 | 1.1933244 | 0.00028 |
| GM19240 | 1.1787862 | 0.00034 |
| HMVECdBlNeo | 1.1583518 | 0.00038 |
| Stellate | 1.1724433 | 0.00076 |
| HMVECdAd | 1.1601504 | 0.00102 |
| A549 | 1.1795339 | 0.00106 |
| NHEK | 1.1485408 | 0.00106 |
| HepG2 | 1.1717660 | 0.00112 |
| HRPEpiC | 1.1208340 | 0.00114 |
| GM19239 | 1.1888795 | 0.00142 |
| HeLaS3IFNa4h | 1.1761386 | 0.00166 |
| HMVECdLyNeo | 1.1422425 | 0.00174 |
| HSMM | 1.1156369 | 0.00182 |
| PanIsletD | 1.1248465 | 0.00222 |
| CLL | 1.1784243 | 0.00236 |
| HUVEC | 1.1409438 | 0.00290 |
| HMVECLLy | 1.1361267 | 0.00324 |
| HMVECdLyAd | 1.1387206 | 0.00364 |
| Urothelia | 1.1381188 | 0.00376 |
| SKNMC | 1.1240448 | 0.00458 |
| HMVECdNeo | 1.1294185 | 0.00500 |
| Myometr | 1.1150719 | 0.00668 |
| IshikawaEstradiol | 1.1222452 | 0.00730 |
| HSMMemb | 1.1210802 | 0.00814 |
| Gliobla | 1.1349407 | 0.00886 |
| Huh7 | 1.1298044 | 0.01204 |
| FibroP | 1.0866902 | 0.01286 |
| IshikawaTamoxifen | 1.1128873 | 0.01464 |
| MCF7 | 1.1128472 | 0.01546 |
| UrotheliaUT189 | 1.1100177 | 0.01766 |
| MCF7Hypoxia | 1.1237500 | 0.02090 |
| ProgFib | 1.1035006 | 0.02186 |
| GM12892 | 1.1213983 | 0.02768 |
| GM12891 | 1.1236198 | 0.02784 |
| HSMMtube | 1.0779292 | 0.03182 |
| Melano | 1.0668797 | 0.03496 |
| Huh7.5 | 1.1066468 | 0.03522 |
| T47D | 1.1048839 | 0.04282 |
| HMEC | 1.0714917 | 0.06572 |
| Caco2 | 1.1047547 | 0.10170 |
| 8988T | 1.0812410 | 0.10960 |
| pHTE | 1.0607135 | 0.11168 |
| PanIslets | 1.0526875 | 0.15970 |
| Fibrobl | 1.0463638 | 0.17040 |
| BE2C | 1.0387307 | 0.22226 |
| H7hESC | 1.0244549 | 0.22470 |
| Osteobl | 1.0356890 | 0.23776 |
| H9ES | 1.0359170 | 0.24572 |
| Medullo | 1.0261186 | 0.31042 |
| WERIRb1 | 1.0187832 | 0.33218 |
| LNCaPAndrogen | 1.0239337 | 0.33268 |
| Hepatocytes | 1.0205466 | 0.36796 |
| NT2D1 | 1.0129568 | 0.37144 |
| Chorion | 1.0189438 | 0.38092 |
| LNCaP | 1.0108670 | 0.40650 |
| iPS | 1.0081351 | 0.44616 |
| H1hESC | 1.0059554 | 0.45408 |
| SKNSHRA | 0.9773738 | 0.63464 |

# Systemic lupus erythematosus 2

| DHS sample | fold enrichment | p value |
|---|---|---|
| Th2 | 1.9655765 | 0.00002 |
| GM12865 | 1.8754164 | 0.00002 |
| GM12864 | 1.9349725 | 0.00004 |
| AG04449 | 1.5925916 | 0.00004 |
| HCFaa | 1.6353442 | 0.00006 |
| Jurkat | 1.6046585 | 0.00026 |
| GM06990 | 1.8519802 | 0.00030 |
| GM12878 | 1.7389125 | 0.00030 |
| AG09309 | 1.4972166 | 0.00042 |
| AG09319 | 1.5522848 | 0.00048 |
| AoSMC | 1.4384047 | 0.00058 |
| BJ | 1.5051443 | 0.00066 |
| HPAF | 1.4913356 | 0.00072 |
| HIPEpiC | 1.4689899 | 0.00080 |
| HCM | 1.4707813 | 0.00090 |
| HPdLF | 1.5115916 | 0.00106 |
| AoAF | 1.4958611 | 0.00112 |
| HMVECLBl | 1.5213871 | 0.00116 |
| SAEC | 1.4168984 | 0.00122 |
| HNPCEpiC | 1.4094108 | 0.00136 |
| HCF | 1.5048566 | 0.00148 |
| CD20 | 1.7606212 | 0.00160 |
| AG10803 | 1.4652156 | 0.00204 |
| HAsp | 1.4464241 | 0.00218 |
| NHLF | 1.4319182 | 0.00218 |
| NHA | 1.4338031 | 0.00234 |
| NHDFAd | 1.4117223 | 0.00312 |
| SKMC | 1.4032320 | 0.00346 |
| HVMF | 1.5047273 | 0.00352 |
| GM18507 | 1.5313222 | 0.00366 |
| HCPEpiC | 1.4049354 | 0.00406 |
| HRGEC | 1.4369952 | 0.00410 |
| HFF | 1.3964690 | 0.00424 |
| HEEpiC | 1.3558708 | 0.00458 |
| HBMEC | 1.3909558 | 0.00494 |
| HAEpiC | 1.4078861 | 0.00548 |
| HMVECdBlAd | 1.4360437 | 0.00610 |
| HMVECdBlNeo | 1.4250044 | 0.00638 |
| HFFMyc | 1.3533278 | 0.00710 |
| HMF | 1.3788723 | 0.00718 |
| PrEC | 1.3403738 | 0.00732 |
| WI38 | 1.4204529 | 0.00770 |
| NHDFneo | 1.3782300 | 0.00828 |
| HGF | 1.3974958 | 0.00850 |
| HMVECdAd | 1.4439841 | 0.00870 |
| AG04450 | 1.4059073 | 0.00894 |
| RPTEC | 1.3223082 | 0.00968 |
| PANC1 | 1.4653788 | 0.01226 |
| HPAEC | 1.3882675 | 0.01316 |
| HMVECdLyAd | 1.4078193 | 0.01332 |
| MonocytesCD14RO01746 | 1.4752367 | 0.01352 |
| HRCEpiC | 1.3237201 | 0.01352 |
| HMVECLLy | 1.3761348 | 0.01708 |
| HMVECdLyNeo | 1.3711664 | 0.01956 |
| HRE | 1.2952399 | 0.02268 |
| HConF | 1.3071088 | 0.02510 |
| Th1 | 1.3722821 | 0.02562 |
| HPF | 1.3151088 | 0.02650 |
| NB4 | 1.3613772 | 0.03218 |
| HRPEpiC | 1.2476195 | 0.03252 |
| GM19238 | 1.3857762 | 0.03652 |
| HAc | 1.2504856 | 0.03708 |
| HMVECdNeo | 1.3085191 | 0.03886 |
| GM19240 | 1.3185968 | 0.04598 |
| HSMM | 1.2235063 | 0.04776 |
| Th0 | 1.3196040 | 0.05170 |
| HAh | 1.2140075 | 0.05186 |
| CD34Mobilized | 1.2806529 | 0.05984 |
| HUVEC | 1.2690027 | 0.06120 |
| NHEK | 1.2505538 | 0.06362 |
| FibroP | 1.1751159 | 0.09922 |
| Myometr | 1.1989157 | 0.10210 |
| HSMMtube | 1.1784192 | 0.11244 |
| Stellate | 1.2249229 | 0.11770 |
| HL60 | 1.2376831 | 0.11906 |
| K562 | 1.2354565 | 0.12138 |
| CLL | 1.2667334 | 0.12546 |
| GM19239 | 1.2460382 | 0.12924 |
| HPDE6E6E7 | 1.1913032 | 0.13674 |
| PanIsletD | 1.1632887 | 0.13996 |
| HTR8svn | 1.1500749 | 0.20008 |
| Melano | 1.1064286 | 0.20100 |
| SKNMC | 1.1144177 | 0.23882 |
| CMK | 1.1436264 | 0.24136 |
| ProgFib | 1.1216355 | 0.24298 |
| GM12892 | 1.1453475 | 0.25880 |
| Urothelia | 1.1059214 | 0.27338 |
| HSMMemb | 1.0959093 | 0.28250 |
| IshikawaEstradiol | 1.0857535 | 0.29732 |
| Gliobla | 1.0870682 | 0.31994 |
| HCT116 | 1.0686962 | 0.33916 |
| NT2D1 | 1.0507426 | 0.34538 |
| IshikawaTamoxifen | 1.0619144 | 0.34880 |
| A549 | 1.0630134 | 0.35464 |
| GM12891 | 1.0693134 | 0.36912 |
| BE2C | 1.0479009 | 0.38296 |
| RWPE1 | 1.0392131 | 0.39386 |
| SKNSHRA | 1.0138315 | 0.45304 |
| HepG2 | 1.0071576 | 0.47166 |
| UrotheliaUT189 | 1.0037697 | 0.47756 |
| HMEC | 1.0049370 | 0.47824 |
| pHTE | 0.9853802 | 0.52100 |
| Fibrobl | 0.9795041 | 0.53792 |
| MCF7 | 0.9703963 | 0.53860 |
| H7hESC | 0.9852005 | 0.54526 |
| HeLaS3 | 0.9689750 | 0.55204 |
| Huh7 | 0.9561419 | 0.57590 |
| PanIslets | 0.9535000 | 0.58666 |
| HeLaS3IFNa4h | 0.9381637 | 0.60040 |
| LNCaP | 0.9414150 | 0.62036 |
| LNCaPAndrogen | 0.9299221 | 0.62626 |
| Osteobl | 0.9314356 | 0.63322 |
| H9ES | 0.9283443 | 0.64270 |
| Huh7.5 | 0.9009336 | 0.66796 |
| Hepatocytes | 0.8874160 | 0.67522 |
| Chorion | 0.8770300 | 0.67666 |
| T47D | 0.8894823 | 0.68188 |
| MCF7Hypoxia | 0.8761010 | 0.69960 |
| 8988T | 0.8570253 | 0.71214 |
| Caco2 | 0.7925875 | 0.74006 |
| H1hESC | 0.8389182 | 0.77582 |
| WERIRb1 | 0.8745424 | 0.78298 |
| Medullo | 0.8163570 | 0.82632 |
| iPS | 0.7655681 | 0.82974 |

# Central corneal thickness and keratoconus

| DHS sample | fold enrichment | p value |
|---|---|---|
| HAEpiC | 1.784874 | 0.00002 |
| AoAF | 1.753381 | 0.00002 |
| HIPEpiC | 1.739519 | 0.00002 |
| HCF | 1.734954 | 0.00002 |
| HConF | 1.709480 | 0.00002 |
| HCM | 1.706053 | 0.00002 |
| HCPEpiC | 1.701671 | 0.00002 |
| HPF | 1.685083 | 0.00002 |
| AoSMC | 1.645369 | 0.00002 |
| NHA | 1.638861 | 0.00002 |
| HNPCEpiC | 1.615502 | 0.00002 |
| HSMM | 1.543210 | 0.00002 |
| HVMF | 1.794620 | 0.00004 |
| WI38 | 1.735138 | 0.00004 |
| AG04450 | 1.731276 | 0.00004 |
| AG09319 | 1.695139 | 0.00004 |
| HPAF | 1.658377 | 0.00004 |
| HCFaa | 1.656877 | 0.00004 |
| HMF | 1.646394 | 0.00004 |
| SKMC | 1.572849 | 0.00006 |
| AG09309 | 1.570820 | 0.00006 |
| HGF | 1.723266 | 0.00008 |
| NHLF | 1.578744 | 0.00008 |
| HAc | 1.516467 | 0.00008 |
| HAsp | 1.533918 | 0.00012 |
| HBMEC | 1.519210 | 0.00012 |
| HMVECdBlNeo | 1.623398 | 0.00014 |
| HMVECLBl | 1.609192 | 0.00018 |
| BJ | 1.593309 | 0.00020 |
| HFFMyc | 1.538459 | 0.00020 |
| HMVECdLyNeo | 1.633639 | 0.00022 |
| NHDFAd | 1.525297 | 0.00022 |
| HFF | 1.513174 | 0.00022 |
| NHDFneo | 1.576282 | 0.00024 |
| HMVECLLy | 1.603851 | 0.00026 |
| HUVEC | 1.598260 | 0.00028 |
| HMVECdBlAd | 1.578228 | 0.00028 |
| HRPEpiC | 1.407594 | 0.00030 |
| HPdLF | 1.558046 | 0.00034 |
| AG04449 | 1.515528 | 0.00038 |
| HMVECdNeo | 1.582195 | 0.00040 |
| RWPE1 | 1.541124 | 0.00040 |
| HMVECdLyAd | 1.603551 | 0.00044 |
| HAh | 1.407104 | 0.00044 |
| HeLaS3IFNa4h | 1.652483 | 0.00052 |
| HMVECdAd | 1.594871 | 0.00052 |
| AG10803 | 1.554498 | 0.00060 |
| PanIsletD | 1.468829 | 0.00064 |
| HSMMtube | 1.457765 | 0.00072 |
| HRGEC | 1.527070 | 0.00074 |
| Stellate | 1.608508 | 0.00078 |
| HeLaS3 | 1.535567 | 0.00108 |
| A549 | 1.594683 | 0.00118 |
| HPAEC | 1.530046 | 0.00124 |
| HSMMemb | 1.528857 | 0.00158 |
| RPTEC | 1.380735 | 0.00182 |
| Myometr | 1.451583 | 0.00194 |
| T47D | 1.586294 | 0.00224 |
| HRE | 1.364401 | 0.00228 |
| HRCEpiC | 1.386748 | 0.00232 |
| FibroP | 1.365305 | 0.00282 |
| NHEK | 1.421909 | 0.00306 |
| GM12865 | 1.511890 | 0.00316 |
| IshikawaTamoxifen | 1.465460 | 0.00344 |
| Gliobla | 1.509218 | 0.00382 |
| PrEC | 1.332127 | 0.00496 |
| SAEC | 1.317183 | 0.00566 |
| ProgFib | 1.431983 | 0.00606 |
| GM06990 | 1.585847 | 0.00630 |
| MonocytesCD14RO01746 | 1.559485 | 0.00662 |
| CD20 | 1.586022 | 0.00718 |
| IshikawaEstradiol | 1.392796 | 0.00802 |
| HTR8svn | 1.418029 | 0.00836 |
| Huh7 | 1.470655 | 0.00952 |
| Th2 | 1.537467 | 0.01000 |
| HPDE6E6E7 | 1.380347 | 0.01222 |
| GM12878 | 1.440980 | 0.01274 |
| PANC1 | 1.402893 | 0.01300 |
| GM18507 | 1.432876 | 0.01344 |
| SKNMC | 1.323616 | 0.01368 |
| HEEpiC | 1.273281 | 0.01458 |
| LNCaPAndrogen | 1.423801 | 0.01490 |
| HCT116 | 1.383782 | 0.01590 |
| Huh7.5 | 1.430502 | 0.01702 |
| Melano | 1.250090 | 0.02032 |
| CD34Mobilized | 1.365173 | 0.02054 |
| GM19238 | 1.407730 | 0.02150 |
| NB4 | 1.393230 | 0.02250 |
| LNCaP | 1.315612 | 0.02348 |
| GM19239 | 1.412471 | 0.02350 |
| GM12864 | 1.398624 | 0.02434 |
| MCF7 | 1.337943 | 0.02468 |
| CLL | 1.432943 | 0.02528 |
| SKNSHRA | 1.406118 | 0.03246 |
| HL60 | 1.387551 | 0.03526 |
| Chorion | 1.414817 | 0.03930 |
| HMEC | 1.268510 | 0.04232 |
| Th0 | 1.330707 | 0.04590 |
| MCF7Hypoxia | 1.365675 | 0.04662 |
| HepG2 | 1.327389 | 0.04694 |
| BE2C | 1.270851 | 0.04930 |
| K562 | 1.321473 | 0.05884 |
| GM12891 | 1.345717 | 0.06194 |
| GM19240 | 1.272810 | 0.06240 |
| PanIslets | 1.282315 | 0.06272 |
| Urothelia | 1.257897 | 0.06480 |
| Fibrobl | 1.255283 | 0.06900 |
| pHTE | 1.245272 | 0.07002 |
| GM12892 | 1.330424 | 0.07274 |
| CMK | 1.272696 | 0.08390 |
| H1hESC | 1.247808 | 0.08642 |
| 8988T | 1.297430 | 0.09450 |
| Th1 | 1.245889 | 0.09836 |
| Hepatocytes | 1.266464 | 0.10002 |
| UrotheliaUT189 | 1.208111 | 0.11482 |
| Caco2 | 1.324839 | 0.13666 |
| Osteobl | 1.169889 | 0.16902 |
| H9ES | 1.143804 | 0.17550 |
| iPS | 1.192454 | 0.19330 |
| Jurkat | 1.134228 | 0.20500 |
| NT2D1 | 1.082941 | 0.24110 |
| Medullo | 1.080551 | 0.32474 |
| H7hESC | 1.036413 | 0.33468 |
| WERIRb1 | 0.995821 | 0.50498 |

## Macular degeneration

| DHS sample | fold enrichment | p value |
|---|---|---|
| HRE | 1.5204596 | 0.00002 |
| HRCEpiC | 1.5669927 | 0.00004 |
| RPTEC | 1.5433901 | 0.00004 |
| HRPEpiC | 1.5009514 | 0.00004 |
| HVMF | 1.5650472 | 0.00006 |
| HCM | 1.5347469 | 0.00006 |
| HAEpiC | 1.5453819 | 0.00008 |
| NHA | 1.5021148 | 0.00008 |
| HPdLF | 1.5397898 | 0.00010 |
| SKMC | 1.4951590 | 0.00014 |
| HNPCEpiC | 1.4634263 | 0.00018 |
| HCFaa | 1.4784614 | 0.00020 |
| HCPEpiC | 1.4769721 | 0.00020 |
| HIPEpiC | 1.4590425 | 0.00028 |
| AG09319 | 1.5363406 | 0.00032 |
| HAc | 1.4438847 | 0.00032 |
| SAEC | 1.4197456 | 0.00036 |
| HPF | 1.4975110 | 0.00038 |
| HCF | 1.5132016 | 0.00040 |
| HAh | 1.4054228 | 0.00040 |
| NHLF | 1.4528839 | 0.00050 |
| HPAF | 1.4663961 | 0.00058 |
| HMVECdBlAd | 1.4999007 | 0.00068 |
| AoAF | 1.4594212 | 0.00072 |
| WI38 | 1.4704714 | 0.00076 |
| HMF | 1.4479290 | 0.00078 |
| HRGEC | 1.4777495 | 0.00080 |
| HPAEC | 1.4875605 | 0.00082 |
| AG10803 | 1.4831559 | 0.00084 |
| HGF | 1.4860756 | 0.00090 |
| HAsp | 1.4618370 | 0.00090 |
| AG09309 | 1.4279135 | 0.00094 |
| HMVECLBl | 1.4513045 | 0.00120 |
| BJ | 1.4651763 | 0.00122 |
| HEEpiC | 1.3898264 | 0.00122 |
| AoSMC | 1.3829429 | 0.00122 |
| NHDFAd | 1.4094150 | 0.00142 |
| AG04449 | 1.4348334 | 0.00144 |
| HMVECdBlNeo | 1.4632190 | 0.00154 |
| HMVECdLyNeo | 1.4609116 | 0.00202 |
| HConF | 1.4379319 | 0.00208 |
| HBMEC | 1.3822114 | 0.00210 |
| AG04450 | 1.4447954 | 0.00224 |
| NHDFneo | 1.4268721 | 0.00228 |
| HFFMyc | 1.3808098 | 0.00264 |
| GM12865 | 1.4917568 | 0.00290 |
| PrEC | 1.3430883 | 0.00324 |
| HMVECdLyAd | 1.4140045 | 0.00622 |
| HFF | 1.3543108 | 0.00674 |
| HMVECdAd | 1.4153565 | 0.00698 |
| HMVECdNeo | 1.4008151 | 0.00808 |
| HMVECLLy | 1.3807957 | 0.00986 |
| GM12864 | 1.4065589 | 0.01198 |
| HSMM | 1.2698415 | 0.01998 |
| SKNMC | 1.3112944 | 0.02006 |
| GM12878 | 1.3751016 | 0.02420 |
| HeLaS3 | 1.3328852 | 0.03294 |
| GM06990 | 1.3550552 | 0.03944 |
| NHEK | 1.2657326 | 0.04102 |
| HCT116 | 1.3065386 | 0.04182 |
| HUVEC | 1.2880026 | 0.04208 |
| HSMMtube | 1.2304384 | 0.04514 |
| FibroP | 1.2115187 | 0.04528 |
| K562 | 1.3069415 | 0.05064 |
| IshikawaTamoxifen | 1.2716292 | 0.05534 |
| HTR8svn | 1.2548484 | 0.06050 |
| MCF7Hypoxia | 1.2963779 | 0.06390 |
| Jurkat | 1.2271758 | 0.06878 |
| BE2C | 1.2455402 | 0.06938 |
| Melano | 1.1750241 | 0.06978 |
| HSMMemb | 1.2379205 | 0.07102 |
| RWPE1 | 1.2307521 | 0.07182 |
| Stellate | 1.2515845 | 0.07598 |
| GM18507 | 1.2636542 | 0.07636 |
| WERIRb1 | 1.2106671 | 0.07762 |
| Urothelia | 1.2307953 | 0.07812 |
| PANC1 | 1.2317172 | 0.08176 |
| HPDE6E6E7 | 1.2249646 | 0.08914 |
| PanIsletD | 1.1947172 | 0.08948 |
| HL60 | 1.2596939 | 0.09090 |
| IshikawaEstradiol | 1.2165935 | 0.09146 |
| CMK | 1.2657839 | 0.09380 |
| HeLaS3IFNa4h | 1.2518374 | 0.09814 |
| H7hESC | 1.1317780 | 0.10808 |
| HepG2 | 1.2141419 | 0.11218 |
| UrotheliaUT189 | 1.1917905 | 0.12416 |
| GM19240 | 1.1922482 | 0.12534 |
| MCF7 | 1.1871825 | 0.12718 |
| Myometr | 1.1719684 | 0.12900 |
| A549 | 1.1893472 | 0.14740 |
| CLL | 1.1967621 | 0.15854 |
| MonocytesCD14RO01746 | 1.1865841 | 0.16412 |
| Th2 | 1.1817300 | 0.17204 |
| GM19238 | 1.1632323 | 0.18900 |
| CD34Mobilized | 1.1396280 | 0.19872 |
| NB4 | 1.1363538 | 0.21252 |
| Th0 | 1.1349736 | 0.21408 |
| Fibrobl | 1.1121468 | 0.23168 |
| CD20 | 1.1482590 | 0.23636 |
| HMEC | 1.1038729 | 0.24204 |
| ProgFib | 1.1133040 | 0.24322 |
| GM12892 | 1.1149991 | 0.27480 |
| Th1 | 1.0882153 | 0.29658 |
| GM12891 | 1.1008295 | 0.30196 |
| Huh7.5 | 1.0945806 | 0.30196 |
| GM19239 | 1.0959522 | 0.30506 |
| NT2D1 | 1.0593730 | 0.31656 |
| Gliobla | 1.0791606 | 0.32472 |
| Huh7 | 1.0777001 | 0.32724 |
| PanIslets | 1.0584433 | 0.35832 |
| pHTE | 1.0491681 | 0.37290 |
| Osteobl | 1.0456772 | 0.38598 |
| H9ES | 1.0263717 | 0.42620 |
| Caco2 | 1.0146811 | 0.45618 |
| Chorion | 1.0008060 | 0.48308 |
| 8988T | 0.9981931 | 0.48696 |
| Hepatocytes | 0.9962801 | 0.49444 |
| SKNSHRA | 0.9776875 | 0.52212 |
| iPS | 0.9797585 | 0.52676 |
| H1hESC | 0.9561682 | 0.58114 |
| LNCaP | 0.9338710 | 0.65516 |
| LNCaPAndrogen | 0.9230358 | 0.65582 |
| T47D | 0.9152573 | 0.65876 |
| Medullo | 0.9121512 | 0.69276 |

# Breast cancer risk

| DHS sample | fold enrichment | p value |
|---|---|---|
| PrEC | 1.3247018 | 0.00002 |
| Th2 | 1.5230855 | 0.00018 |
| SAEC | 1.3005721 | 0.00020 |
| HEEpiC | 1.2800591 | 0.00032 |
| HGF | 1.3443506 | 0.00054 |
| MonocytesCD14RO01746 | 1.4538710 | 0.00092 |
| HL60 | 1.4371357 | 0.00146 |
| HCT116 | 1.3486826 | 0.00156 |
| NHDFAd | 1.2633980 | 0.00262 |
| HPdLF | 1.2817963 | 0.00270 |
| SKMC | 1.2533838 | 0.00270 |
| AG09319 | 1.2855364 | 0.00274 |
| HRCEpiC | 1.2335123 | 0.00390 |
| RPTEC | 1.2145916 | 0.00452 |
| A549 | 1.3149589 | 0.00532 |
| HMF | 1.2421758 | 0.00546 |
| NHEK | 1.2540169 | 0.00548 |
| CD20 | 1.4059228 | 0.00568 |
| NHDFneo | 1.2502145 | 0.00576 |
| HRE | 1.2098907 | 0.00676 |
| HConF | 1.2453837 | 0.00682 |
| HFFMyc | 1.2303285 | 0.00694 |
| HMVECLBl | 1.2635387 | 0.00736 |
| HVMF | 1.2690455 | 0.00782 |
| HRPEpiC | 1.1860233 | 0.00814 |
| GM12865 | 1.2801859 | 0.00828 |
| CD34Mobilized | 1.2836853 | 0.00896 |
| GM06990 | 1.3519836 | 0.00900 |
| AoAF | 1.2382798 | 0.00970 |
| NB4 | 1.2888337 | 0.01054 |
| AoSMC | 1.2111603 | 0.01054 |
| HCM | 1.2152753 | 0.01066 |
| GM12864 | 1.2948096 | 0.01086 |
| HCF | 1.2342781 | 0.01104 |
| AG10803 | 1.2288356 | 0.01108 |
| HCFaa | 1.2150440 | 0.01164 |
| CMK | 1.3153918 | 0.01252 |
| HSMM | 1.1901028 | 0.01260 |
| HPF | 1.2222448 | 0.01264 |
| PANC1 | 1.2521198 | 0.01302 |
| BJ | 1.2215165 | 0.01322 |
| Huh7 | 1.2888285 | 0.01356 |
| GM12878 | 1.2907855 | 0.01472 |
| HAEpiC | 1.2129611 | 0.01566 |
| Myometr | 1.2091529 | 0.01590 |
| HAh | 1.1672320 | 0.01758 |
| RWPE1 | 1.2175451 | 0.01812 |
| HUVEC | 1.2240160 | 0.01998 |
| HMVECdLyAd | 1.2355327 | 0.02026 |
| HFF | 1.1910438 | 0.02124 |
| MCF7 | 1.2247268 | 0.02140 |
| HMVECdBlAd | 1.2187427 | 0.02166 |
| Jurkat | 1.2207302 | 0.02228 |
| HPDE6E6E7 | 1.2281193 | 0.02238 |
| Huh7.5 | 1.2586962 | 0.02294 |
| NHA | 1.1763120 | 0.02564 |
| IshikawaEstradiol | 1.2075848 | 0.02628 |
| HAsp | 1.1719061 | 0.02702 |
| AG09309 | 1.1753292 | 0.02840 |
| AG04450 | 1.1922917 | 0.03086 |
| HTR8svn | 1.2140225 | 0.03108 |
| HBMEC | 1.1604378 | 0.03122 |
| HIPEpiC | 1.1723388 | 0.03154 |
| WI38 | 1.1972093 | 0.03222 |
| HCPEpiC | 1.1716858 | 0.03238 |
| K562 | 1.2492878 | 0.03290 |
| HPAF | 1.1746305 | 0.03318 |
| NHLF | 1.1599759 | 0.03386 |
| HMVECdBlNeo | 1.1994590 | 0.03416 |
| T47D | 1.2422614 | 0.03616 |
| HMVECLLy | 1.2001451 | 0.03750 |
| IshikawaTamoxifen | 1.1944476 | 0.04044 |
| GM19239 | 1.2401382 | 0.04152 |
| Melano | 1.1360577 | 0.04174 |
| GM18507 | 1.2224283 | 0.04254 |
| HepG2 | 1.2160837 | 0.04256 |
| HeLaS3 | 1.1941162 | 0.04472 |
| HMVECdNeo | 1.1872458 | 0.04656 |
| HMVECdAd | 1.1938836 | 0.04666 |
| HSMMemb | 1.1819823 | 0.05078 |
| HMVECdLyNeo | 1.1822500 | 0.05110 |
| AG04449 | 1.1560936 | 0.05226 |
| GM19240 | 1.1911319 | 0.05540 |
| SKNMC | 1.1504187 | 0.05594 |
| FibroP | 1.1330268 | 0.06124 |
| HNPCEpiC | 1.1284816 | 0.06330 |
| Urothelia | 1.1734687 | 0.06498 |
| Th0 | 1.1907465 | 0.06800 |
| HPAEC | 1.1597969 | 0.06968 |
| Gliobla | 1.1845620 | 0.07338 |
| MCF7Hypoxia | 1.1930570 | 0.07488 |
| HRGEC | 1.1463191 | 0.07510 |
| GM19238 | 1.1905674 | 0.07584 |
| HAc | 1.1178810 | 0.07902 |
| PanIsletD | 1.1289988 | 0.08766 |
| CLL | 1.1939844 | 0.08770 |
| Stellate | 1.1579975 | 0.09250 |
| 8988T | 1.1946400 | 0.09266 |
| HeLaS3IFNa4h | 1.1654553 | 0.09758 |
| HSMMtube | 1.1126470 | 0.10680 |
| Caco2 | 1.2345640 | 0.10712 |
| ProgFib | 1.1349381 | 0.10788 |
| BE2C | 1.1315411 | 0.10798 |
| LNCaPAndrogen | 1.1438121 | 0.12284 |
| LNCaP | 1.1140196 | 0.12616 |
| pHTE | 1.1150508 | 0.14682 |
| HMEC | 1.1045193 | 0.15162 |
| GM12891 | 1.1483652 | 0.15224 |
| SKNSHRA | 1.1302714 | 0.15316 |
| Th1 | 1.1238430 | 0.15772 |
| Hepatocytes | 1.1345934 | 0.16160 |
| H9ES | 1.0967385 | 0.17758 |
| UrotheliaUT189 | 1.0979404 | 0.19792 |
| NT2D1 | 1.0677735 | 0.20286 |
| PanIslets | 1.0953865 | 0.20726 |
| GM12892 | 1.1114601 | 0.21844 |
| Chorion | 1.1069837 | 0.22354 |
| H1hESC | 1.0738040 | 0.25814 |
| H7hESC | 1.0354275 | 0.27892 |
| Fibrobl | 1.0560106 | 0.30188 |
| iPS | 1.0537326 | 0.35092 |
| Osteobl | 1.0224667 | 0.41582 |
| Medullo | 0.9795571 | 0.55852 |
| WERIRb1 | 0.9761829 | 0.59692 |

# Lipid levels 6

| DHS sample | fold enrichment | p value |
|---|---|---|
| HCFaa | 1.2095105 | 0.00002 |
| RPTEC | 1.1902102 | 0.00002 |
| HGF | 1.1953139 | 0.00004 |
| HRGEC | 1.1832295 | 0.00004 |
| HAsp | 1.1812214 | 0.00004 |
| HCPEpiC | 1.1721591 | 0.00004 |
| HRCEpiC | 1.1710850 | 0.00004 |
| HCF | 1.1735628 | 0.00008 |
| HPAF | 1.1732228 | 0.00010 |
| HIPEpiC | 1.1522344 | 0.00010 |
| HPAEC | 1.1781098 | 0.00012 |
| HPdLF | 1.1698725 | 0.00014 |
| HCM | 1.1494282 | 0.00016 |
| AoAF | 1.1605645 | 0.00020 |
| HConF | 1.1614510 | 0.00028 |
| NB4 | 1.1786991 | 0.00030 |
| HMVECLBl | 1.1580952 | 0.00034 |
| MonocytesCD14RO01746 | 1.2077613 | 0.00036 |
| HVMF | 1.1672120 | 0.00036 |
| AoSMC | 1.1347726 | 0.00036 |
| HAEpiC | 1.1570554 | 0.00038 |
| HNPCEpiC | 1.1320905 | 0.00038 |
| HMF | 1.1450523 | 0.00044 |
| HBMEC | 1.1348497 | 0.00060 |
| SKMC | 1.1366761 | 0.00062 |
| HMVECdBlNeo | 1.1533961 | 0.00064 |
| AG04449 | 1.1404184 | 0.00082 |
| SAEC | 1.1219092 | 0.00092 |
| HMVECdLyNeo | 1.1472937 | 0.00102 |
| NHDFAd | 1.1301287 | 0.00104 |
| PANC1 | 1.1656191 | 0.00106 |
| AG09319 | 1.1499048 | 0.00114 |
| HPF | 1.1410583 | 0.00114 |
| HMVECdBlAd | 1.1397552 | 0.00144 |
| AG10803 | 1.1370029 | 0.00148 |
| HMVECdAd | 1.1505957 | 0.00154 |
| BJ | 1.1365961 | 0.00160 |
| HL60 | 1.1762789 | 0.00178 |
| HRE | 1.1141916 | 0.00194 |
| AG09309 | 1.1227640 | 0.00200 |
| AG04450 | 1.1359536 | 0.00234 |
| NHDFneo | 1.1234754 | 0.00290 |
| HEEpiC | 1.1073343 | 0.00296 |
| K562 | 1.1544562 | 0.00334 |
| HAc | 1.1087971 | 0.00336 |
| WI38 | 1.1261447 | 0.00396 |
| NHA | 1.1131180 | 0.00458 |
| HFF | 1.1109623 | 0.00472 |
| Th2 | 1.1560284 | 0.00540 |
| HMVECdNeo | 1.1237551 | 0.00614 |
| NHLF | 1.1032891 | 0.00652 |
| GM12865 | 1.1299274 | 0.00712 |
| GM12864 | 1.1335516 | 0.00742 |
| HMVECdLyAd | 1.1206393 | 0.00796 |
| HCT116 | 1.1256165 | 0.00914 |
| HFFMyc | 1.0967835 | 0.01076 |
| HAh | 1.0858315 | 0.01130 |
| HMVECLLy | 1.1098398 | 0.01242 |
| PrEC | 1.0792545 | 0.01930 |
| HRPEpiC | 1.0798595 | 0.01984 |
| GM06990 | 1.1216049 | 0.02520 |
| HepG2 | 1.1016781 | 0.02888 |
| SKNMC | 1.0921135 | 0.02922 |
| A549 | 1.1027124 | 0.03006 |
| HUVEC | 1.0920695 | 0.03184 |
| NHEK | 1.0866964 | 0.03184 |
| Jurkat | 1.0872962 | 0.03272 |
| CD20 | 1.1154806 | 0.03960 |
| GM12878 | 1.0956889 | 0.04476 |
| HTR8svn | 1.0841383 | 0.04662 |
| CMK | 1.1032264 | 0.04924 |
| CD34Mobilized | 1.0813623 | 0.05334 |
| IshikawaEstradiol | 1.0760700 | 0.05930 |
| HPDE6E6E7 | 1.0776925 | 0.06200 |
| HeLaS3 | 1.0804312 | 0.06704 |
| GM18507 | 1.0797791 | 0.07056 |
| Huh7 | 1.0789779 | 0.07676 |
| Stellate | 1.0751906 | 0.07870 |
| RWPE1 | 1.0632612 | 0.09018 |
| HSMM | 1.0476853 | 0.11048 |
| IshikawaTamoxifen | 1.0597931 | 0.11778 |
| HSMMemb | 1.0546106 | 0.13062 |
| CLL | 1.0634315 | 0.14816 |
| Myometr | 1.0475280 | 0.15182 |
| PanIsletD | 1.0410929 | 0.17038 |
| Huh7.5 | 1.0484057 | 0.19672 |
| HeLaS3IFNa4h | 1.0453476 | 0.21344 |
| Urothelia | 1.0370501 | 0.22990 |
| FibroP | 1.0226528 | 0.27410 |
| GM19239 | 1.0315242 | 0.29316 |
| Gliobla | 1.0294128 | 0.29656 |
| NT2D1 | 1.0205662 | 0.29660 |
| BE2C | 1.0239735 | 0.32050 |
| ProgFib | 1.0222731 | 0.32712 |
| HSMMtube | 1.0153729 | 0.35302 |
| Melano | 1.0123042 | 0.36776 |
| GM19238 | 1.0174970 | 0.37332 |
| H7hESC | 1.0096147 | 0.37944 |
| GM19240 | 1.0122576 | 0.40064 |
| T47D | 1.0102516 | 0.42588 |
| Th1 | 1.0086627 | 0.43260 |
| Th0 | 1.0083252 | 0.43466 |
| LNCaPAndrogen | 1.0067861 | 0.44422 |
| MCF7 | 1.0044216 | 0.45776 |
| SKNSHRA | 1.0030499 | 0.47448 |
| Caco2 | 0.9964609 | 0.50848 |
| UrotheliaUT189 | 0.9986179 | 0.51004 |
| GM12891 | 0.9920851 | 0.54600 |
| Hepatocytes | 0.9911236 | 0.55290 |
| Fibrobl | 0.9910572 | 0.57164 |
| WERIRb1 | 0.9899589 | 0.58962 |
| GM12892 | 0.9840737 | 0.59896 |
| LNCaP | 0.9827782 | 0.63496 |
| HMEC | 0.9830962 | 0.64238 |
| pHTE | 0.9815567 | 0.64736 |
| PanIslets | 0.9774752 | 0.66634 |
| 8988T | 0.9652875 | 0.70098 |
| Medullo | 0.9712796 | 0.70946 |
| MCF7Hypoxia | 0.9654533 | 0.71876 |
| H9ES | 0.9662251 | 0.73992 |
| Osteobl | 0.9675785 | 0.74492 |
| H1hESC | 0.9483466 | 0.81788 |
| Chorion | 0.9277994 | 0.87068 |
| iPS | 0.9037805 | 0.93630 |

# Rheumatoid arthritis 2

| DHS sample | fold enrichment | p value |
|---|---|---|
| Th2 | 1.9284290 | 0.00002 |
| GM06990 | 1.8007391 | 0.00002 |
| GM12864 | 1.6962085 | 0.00002 |
| CD20 | 1.6683008 | 0.00002 |
| GM12865 | 1.6505086 | 0.00002 |
| GM12878 | 1.5810287 | 0.00002 |
| MonocytesCD14RO01746 | 1.5344062 | 0.00002 |
| Jurkat | 1.5026690 | 0.00002 |
| GM18507 | 1.4722417 | 0.00002 |
| Th0 | 1.3928562 | 0.00002 |
| AoAF | 1.3243886 | 0.00002 |
| HCFaa | 1.3131251 | 0.00002 |
| AoSMC | 1.3091648 | 0.00002 |
| HPAF | 1.2995928 | 0.00002 |
| HIPEpiC | 1.2969348 | 0.00002 |
| HCM | 1.2889742 | 0.00002 |
| HBMEC | 1.2861020 | 0.00002 |
| NHLF | 1.2838592 | 0.00002 |
| SAEC | 1.2823224 | 0.00002 |
| HNPCEpiC | 1.2803567 | 0.00002 |
| PrEC | 1.2424797 | 0.00002 |
| NB4 | 1.3798462 | 0.00004 |
| Th1 | 1.3695686 | 0.00004 |
| AG04449 | 1.3224251 | 0.00004 |
| AG09309 | 1.3093746 | 0.00004 |
| HCF | 1.3027852 | 0.00004 |
| AG10803 | 1.3010379 | 0.00004 |
| BJ | 1.2973667 | 0.00004 |
| HAsp | 1.2893704 | 0.00004 |
| HGF | 1.2841910 | 0.00004 |
| NHA | 1.2810224 | 0.00004 |
| HCPEpiC | 1.2687337 | 0.00004 |
| HRCEpiC | 1.2654727 | 0.00004 |
| HEEpiC | 1.2596095 | 0.00004 |
| AG09319 | 1.3226513 | 0.00006 |
| HMF | 1.2778276 | 0.00006 |
| RPTEC | 1.2464907 | 0.00006 |
| CD34Mobilized | 1.3382933 | 0.00008 |
| GM19240 | 1.3215998 | 0.00008 |
| HPdLF | 1.2958230 | 0.00008 |
| HVMF | 1.2949987 | 0.00008 |
| HAEpiC | 1.2758813 | 0.00008 |
| SKMC | 1.2719553 | 0.00008 |
| HRE | 1.2455887 | 0.00010 |
| AG04450 | 1.2912132 | 0.00012 |
| CLL | 1.4108753 | 0.00014 |
| PANC1 | 1.3147416 | 0.00014 |
| HFFMyc | 1.2489675 | 0.00014 |
| HL60 | 1.3663453 | 0.00018 |
| GM19238 | 1.3527144 | 0.00018 |
| NHDFneo | 1.2752207 | 0.00018 |
| HFF | 1.2590969 | 0.00020 |
| HMVECLBl | 1.2665830 | 0.00026 |
| HRGEC | 1.2567681 | 0.00028 |
| NHDFAd | 1.2311224 | 0.00028 |
| HConF | 1.2548500 | 0.00030 |
| WI38 | 1.2717031 | 0.00042 |
| HAh | 1.2022112 | 0.00048 |
| HPAEC | 1.2642737 | 0.00072 |
| HMVECdBlAd | 1.2507366 | 0.00080 |
| HPF | 1.2340633 | 0.00106 |
| HMVECdLyNeo | 1.2494682 | 0.00110 |
| HAc | 1.1954753 | 0.00114 |
| HMVECdLyAd | 1.2493015 | 0.00132 |
| K562 | 1.2890693 | 0.00140 |
| HTR8svn | 1.2542239 | 0.00152 |
| HMVECdAd | 1.2522902 | 0.00152 |
| GM19239 | 1.3094519 | 0.00168 |
| HMVECdBlNeo | 1.2336340 | 0.00172 |
| HPDE6E6E7 | 1.2477098 | 0.00200 |
| HMVECLLy | 1.2278717 | 0.00262 |
| HCT116 | 1.2408584 | 0.00270 |
| HRPEpiC | 1.1655004 | 0.00302 |
| IshikawaEstradiol | 1.2182522 | 0.00314 |
| NHEK | 1.2099217 | 0.00342 |
| PanIsletD | 1.1958930 | 0.00392 |
| Stellate | 1.2324987 | 0.00434 |
| Urothelia | 1.2238174 | 0.00440 |
| HSMM | 1.1679692 | 0.00470 |
| HUVEC | 1.2091198 | 0.00556 |
| HMVECdNeo | 1.2047839 | 0.00638 |
| RWPE1 | 1.1924984 | 0.00770 |
| FibroP | 1.1541128 | 0.00794 |
| MCF7 | 1.2005509 | 0.00834 |
| Myometr | 1.1770634 | 0.00924 |
| CMK | 1.2427039 | 0.00962 |
| IshikawaTamoxifen | 1.1884510 | 0.01080 |
| GM12892 | 1.2329608 | 0.01372 |
| HSMMemb | 1.1750309 | 0.01714 |
| MCF7Hypoxia | 1.1985668 | 0.02058 |
| Huh7 | 1.1924199 | 0.02210 |
| GM12891 | 1.2124194 | 0.02292 |
| SKNMC | 1.1457733 | 0.02326 |
| HeLaS3 | 1.1714059 | 0.02520 |
| Gliobla | 1.1833781 | 0.02568 |
| H7hESC | 1.0971512 | 0.02648 |
| UrotheliaUT189 | 1.1652332 | 0.02682 |
| Melano | 1.1162159 | 0.02796 |
| ProgFib | 1.1502412 | 0.03564 |
| A549 | 1.1628995 | 0.03602 |
| HSMMtube | 1.1191049 | 0.04062 |
| HepG2 | 1.1550544 | 0.04698 |
| NT2D1 | 1.1038893 | 0.04968 |
| HeLaS3IFNa4h | 1.1510245 | 0.05890 |
| H9ES | 1.1189393 | 0.07776 |
| BE2C | 1.1162805 | 0.08424 |
| T47D | 1.1305130 | 0.08922 |
| Huh7.5 | 1.1285964 | 0.09194 |
| HMEC | 1.0957422 | 0.10074 |
| PanIslets | 1.1075855 | 0.10836 |
| Fibrobl | 1.0984973 | 0.10960 |
| LNCaPAndrogen | 1.1121270 | 0.11170 |
| pHTE | 1.0851470 | 0.14402 |
| Osteobl | 1.0664607 | 0.20808 |
| Medullo | 1.0602702 | 0.24096 |
| 8988T | 1.0569927 | 0.29016 |
| Hepatocytes | 1.0485021 | 0.31044 |
| LNCaP | 1.0292655 | 0.34608 |
| WERIRb1 | 1.0233908 | 0.36484 |
| H1hESC | 1.0102919 | 0.44560 |
| SKNSHRA | 1.0101085 | 0.44824 |
| Chorion | 1.0040039 | 0.47904 |
| iPS | 1.0025896 | 0.48648 |
| Caco2 | 0.9712124 | 0.57250 |

## Colorectal cancer

| DHS sample | fold enrichment | p value |
|---|---|---|
| PrEC | 1.7753049 | 0.00002 |
| HEEpiC | 1.7186095 | 0.00002 |
| SAEC | 1.7069229 | 0.00002 |
| HVMF | 1.8638713 | 0.00006 |
| WI38 | 1.7964275 | 0.00006 |
| HAEpiC | 1.7432684 | 0.00010 |
| SKMC | 1.6395229 | 0.00016 |
| AG04450 | 1.7589508 | 0.00020 |
| HPDE6E6E7 | 1.8473502 | 0.00022 |
| RWPE1 | 1.7638469 | 0.00034 |
| HCT116 | 1.7823203 | 0.00036 |
| HUVEC | 1.7139360 | 0.00044 |
| HSMM | 1.5833790 | 0.00048 |
| NHDFneo | 1.6618620 | 0.00056 |
| HPF | 1.6702400 | 0.00058 |
| HMVECLBl | 1.7223271 | 0.00070 |
| HGF | 1.6921968 | 0.00072 |
| HFFMyc | 1.6023270 | 0.00074 |
| NHDFAd | 1.6192664 | 0.00076 |
| NHEK | 1.6531073 | 0.00078 |
| HFF | 1.6126583 | 0.00080 |
| HMVECdAd | 1.7509260 | 0.00094 |
| HConF | 1.6376802 | 0.00100 |
| HMVECdBlNeo | 1.6841775 | 0.00102 |
| HCF | 1.6250542 | 0.00104 |
| HRPEpiC | 1.4884815 | 0.00110 |
| HRE | 1.5282214 | 0.00118 |
| MonocytesCD14RO01746 | 1.9154289 | 0.00124 |
| AG04449 | 1.6071497 | 0.00126 |
| BJ | 1.6068793 | 0.00136 |
| HMF | 1.5704190 | 0.00136 |
| AG10803 | 1.6266165 | 0.00140 |
| HMVECLLy | 1.6942792 | 0.00142 |
| AoAF | 1.6016950 | 0.00146 |
| AG09309 | 1.5509320 | 0.00168 |
| HSMMtube | 1.5353994 | 0.00190 |
| A549 | 1.7104203 | 0.00196 |
| HMVECdLyAd | 1.6661433 | 0.00204 |
| NHLF | 1.5289200 | 0.00204 |
| HMVECdLyNeo | 1.6376186 | 0.00220 |
| HMVECdNeo | 1.6529965 | 0.00224 |
| AG09319 | 1.6082914 | 0.00226 |
| HMEC | 1.6063568 | 0.00228 |
| HSMMemb | 1.6704126 | 0.00230 |
| HPdLF | 1.6017111 | 0.00230 |
| Stellate | 1.7552947 | 0.00232 |
| H7hESC | 1.3670252 | 0.00246 |
| HL60 | 1.8392585 | 0.00252 |
| CD34Mobilized | 1.6773970 | 0.00262 |
| NHA | 1.5067871 | 0.00268 |
| IshikawaTamoxifen | 1.6451404 | 0.00290 |
| HAh | 1.4344307 | 0.00292 |
| HPAF | 1.5077166 | 0.00304 |
| HAc | 1.4777308 | 0.00320 |
| HPAEC | 1.5972685 | 0.00324 |
| HTR8svn | 1.6451438 | 0.00328 |
| HeLaS3IFNa4h | 1.7329146 | 0.00330 |
| Myometr | 1.5162507 | 0.00332 |
| HeLaS3 | 1.6557799 | 0.00364 |
| HRGEC | 1.5648184 | 0.00370 |
| HBMEC | 1.4681353 | 0.00384 |
| FibroP | 1.4530729 | 0.00400 |
| HCM | 1.4835541 | 0.00406 |
| Gliobla | 1.6837964 | 0.00420 |
| HMVECdBlAd | 1.5694032 | 0.00422 |
| SKNSHRA | 1.7497559 | 0.00542 |
| ProgFib | 1.5813155 | 0.00594 |
| HRCEpiC | 1.4487386 | 0.00598 |
| PanIsletD | 1.4878127 | 0.00654 |
| Urothelia | 1.5855424 | 0.00672 |
| IshikawaEstradiol | 1.5441415 | 0.00688 |
| HIPEpiC | 1.4538554 | 0.00742 |
| HNPCEpiC | 1.4119328 | 0.00760 |
| AoSMC | 1.4465689 | 0.00796 |
| Fibrobl | 1.5768417 | 0.00822 |
| NT2D1 | 1.4396114 | 0.00824 |
| pHTE | 1.5527958 | 0.00942 |
| Jurkat | 1.5339019 | 0.00962 |
| UrotheliaUT189 | 1.5636214 | 0.00990 |
| Huh7 | 1.6122883 | 0.01020 |
| Melano | 1.3670531 | 0.01038 |
| Th0 | 1.6156453 | 0.01068 |
| HCPEpiC | 1.4137392 | 0.01122 |
| Th1 | 1.5994773 | 0.01130 |
| HAsp | 1.4084181 | 0.01274 |
| GM12878 | 1.5789310 | 0.01510 |
| GM12892 | 1.6685913 | 0.01584 |
| GM19239 | 1.5857944 | 0.01666 |
| HepG2 | 1.5504408 | 0.01862 |
| Osteobl | 1.5087449 | 0.02030 |
| GM12865 | 1.5131592 | 0.02100 |
| Medullo | 1.5365405 | 0.02126 |
| LNCaPAndrogen | 1.5292510 | 0.02160 |
| CD20 | 1.6498280 | 0.02164 |
| GM19238 | 1.5542329 | 0.02174 |
| CMK | 1.5620636 | 0.02316 |
| GM12864 | 1.5369447 | 0.02360 |
| Chorion | 1.6225732 | 0.02386 |
| GM06990 | 1.6052146 | 0.02492 |
| HCFaa | 1.3698991 | 0.02762 |
| RPTEC | 1.3100481 | 0.02780 |
| Huh7.5 | 1.5100380 | 0.02796 |
| Th2 | 1.5804648 | 0.03020 |
| GM18507 | 1.4805770 | 0.03076 |
| BE2C | 1.4963215 | 0.03128 |
| PanIslets | 1.4506796 | 0.03284 |
| H9ES | 1.3909587 | 0.03570 |
| GM12891 | 1.5542146 | 0.03642 |
| GM19240 | 1.4251705 | 0.03724 |
| LNCaP | 1.3649108 | 0.04094 |
| PANC1 | 1.4028938 | 0.04504 |
| NB4 | 1.4051768 | 0.05438 |
| Hepatocytes | 1.4341497 | 0.06112 |
| iPS | 1.4738273 | 0.06526 |
| H1hESC | 1.3635764 | 0.06630 |
| Caco2 | 1.6212764 | 0.06998 |
| K562 | 1.4016520 | 0.07082 |
| 8988T | 1.4402328 | 0.07626 |
| MCF7Hypoxia | 1.3980073 | 0.08954 |
| WERIRb1 | 1.2533873 | 0.09102 |
| CLL | 1.3721026 | 0.09678 |
| MCF7 | 1.3067996 | 0.09986 |
| T47D | 1.2745426 | 0.13506 |
| SKNMC | 0.9984477 | 0.48886 |

# Anthropometric traits

| DHS sample | fold enrichment | p value |
|---|---|---|
| HCFaa | 1.2517298 | 0.00004 |
| HAsp | 1.2278764 | 0.00014 |
| HAEpiC | 1.2222989 | 0.00016 |
| AG04449 | 1.2093938 | 0.00020 |
| HCPEpiC | 1.1971840 | 0.00020 |
| HGF | 1.2302308 | 0.00026 |
| HPdLF | 1.2216716 | 0.00030 |
| AoAF | 1.2097424 | 0.00030 |
| HNPCEpiC | 1.1804123 | 0.00038 |
| AG09309 | 1.1940475 | 0.00042 |
| HRGEC | 1.2082971 | 0.00044 |
| HPAF | 1.1915946 | 0.00048 |
| HBMEC | 1.1781879 | 0.00048 |
| AG04450 | 1.2147742 | 0.00050 |
| HIPEpiC | 1.1859072 | 0.00056 |
| HVMF | 1.2167844 | 0.00060 |
| NHDFneo | 1.2093550 | 0.00062 |
| AG09319 | 1.2077450 | 0.00064 |
| SKMC | 1.1833622 | 0.00070 |
| NHLF | 1.1729647 | 0.00078 |
| SKNMC | 1.1983010 | 0.00080 |
| AoSMC | 1.1734752 | 0.00080 |
| HFFMyc | 1.1827781 | 0.00088 |
| HConF | 1.1937715 | 0.00110 |
| BJ | 1.1877613 | 0.00140 |
| WI38 | 1.1965212 | 0.00152 |
| NHDFAd | 1.1645326 | 0.00178 |
| HPF | 1.1854517 | 0.00194 |
| NHA | 1.1600241 | 0.00234 |
| HMF | 1.1697763 | 0.00246 |
| HCM | 1.1572759 | 0.00290 |
| HCF | 1.1732489 | 0.00292 |
| HMVECLBl | 1.1763379 | 0.00308 |
| HFF | 1.1606353 | 0.00322 |
| RPTEC | 1.1418261 | 0.00322 |
| Jurkat | 1.1814752 | 0.00334 |
| PANC1 | 1.1975479 | 0.00350 |
| HL60 | 1.2121318 | 0.00580 |
| AG10803 | 1.1593866 | 0.00632 |
| HAh | 1.1222249 | 0.00716 |
| NB4 | 1.1826033 | 0.00726 |
| HRPEpiC | 1.1192623 | 0.00860 |
| HAc | 1.1250279 | 0.00942 |
| HPAEC | 1.1545072 | 0.00948 |
| HMVECdLyNeo | 1.1548466 | 0.01038 |
| HMVECdBlNeo | 1.1501497 | 0.01068 |
| GM12865 | 1.1646244 | 0.01074 |
| HRCEpiC | 1.1257907 | 0.01186 |
| Th2 | 1.1887351 | 0.01410 |
| MonocytesCD14RO01746 | 1.1837043 | 0.01510 |
| HRE | 1.1132329 | 0.01690 |
| HMVECdBlAd | 1.1302232 | 0.02120 |
| HEEpiC | 1.1016847 | 0.02374 |
| SAEC | 1.1002665 | 0.02410 |
| GM12864 | 1.1477436 | 0.02624 |
| HMVECdNeo | 1.1308690 | 0.02682 |
| HMVECdLyAd | 1.1267977 | 0.03084 |
| HMVECdAd | 1.1295980 | 0.03126 |
| HUVEC | 1.1252726 | 0.03194 |
| CD20 | 1.1702651 | 0.03228 |
| CD34Mobilized | 1.1299715 | 0.03338 |
| HMVECLLy | 1.1222995 | 0.03506 |
| PrEC | 1.0863029 | 0.04670 |
| K562 | 1.1273999 | 0.05422 |
| GM06990 | 1.1377796 | 0.05660 |
| HCT116 | 1.1125549 | 0.05790 |
| HeLaS3 | 1.1078451 | 0.06540 |
| GM12878 | 1.1161795 | 0.06732 |
| Stellate | 1.1077738 | 0.06884 |
| HPDE6E6E7 | 1.1004225 | 0.07022 |
| HTR8svn | 1.0816372 | 0.11466 |
| BE2C | 1.0806836 | 0.11734 |
| HSMM | 1.0595586 | 0.12660 |
| A549 | 1.0830405 | 0.12836 |
| Myometr | 1.0686969 | 0.13002 |
| RWPE1 | 1.0680682 | 0.13858 |
| HSMMemb | 1.0690641 | 0.15070 |
| IshikawaEstradiol | 1.0635931 | 0.15838 |
| NHEK | 1.0606223 | 0.16278 |
| HeLaS3IFNa4h | 1.0717542 | 0.17858 |
| CMK | 1.0743837 | 0.18530 |
| IshikawaTamoxifen | 1.0501911 | 0.21976 |
| PanIsletD | 1.0445602 | 0.22294 |
| HepG2 | 1.0526806 | 0.23554 |
| GM18507 | 1.0525795 | 0.23878 |
| NT2D1 | 1.0333705 | 0.25932 |
| WERIRb1 | 1.0280045 | 0.30534 |
| Huh7 | 1.0372835 | 0.30678 |
| MCF7Hypoxia | 1.0379492 | 0.30792 |
| SKNSHRA | 1.0378708 | 0.31912 |
| Gliobla | 1.0313249 | 0.33562 |
| CLL | 1.0322670 | 0.34728 |
| GM19239 | 1.0281814 | 0.36034 |
| ProgFib | 1.0202758 | 0.37862 |
| Urothelia | 1.0179666 | 0.39426 |
| MCF7 | 1.0124781 | 0.41756 |
| H7hESC | 1.0078907 | 0.41860 |
| T47D | 1.0082176 | 0.45244 |
| GM19238 | 1.0016942 | 0.48550 |
| HSMMtube | 0.9938713 | 0.54038 |
| Huh7.5 | 0.9879645 | 0.55502 |
| FibroP | 0.9909157 | 0.56746 |
| Th0 | 0.9779720 | 0.61786 |
| GM19240 | 0.9766914 | 0.62648 |
| GM12891 | 0.9685606 | 0.63770 |
| UrotheliaUT189 | 0.9726852 | 0.65122 |
| Melano | 0.9742877 | 0.69782 |
| H9ES | 0.9607381 | 0.71802 |
| LNCaPAndrogen | 0.9397820 | 0.78028 |
| Th1 | 0.9425432 | 0.79122 |
| Hepatocytes | 0.9305632 | 0.79484 |
| Medullo | 0.9369932 | 0.80880 |
| GM12892 | 0.9230827 | 0.81402 |
| H1hESC | 0.9223161 | 0.85220 |
| pHTE | 0.9308973 | 0.85380 |
| Caco2 | 0.8802715 | 0.85906 |
| HMEC | 0.9277163 | 0.87838 |
| PanIslets | 0.9129865 | 0.89168 |
| Chorion | 0.8891758 | 0.90144 |
| LNCaP | 0.9165082 | 0.90828 |
| Fibrobl | 0.9103236 | 0.91430 |
| Osteobl | 0.8984559 | 0.93186 |
| 8988T | 0.8715851 | 0.93448 |
| iPS | 0.8508465 | 0.96032 |

## Human red blood cell

| DHS sample | fold enrichment | p value |
|---|---|---|
| CMK | 1.4687405 | 0.00006 |
| Jurkat | 1.3018863 | 0.00020 |
| Th2 | 1.4023732 | 0.00034 |
| NB4 | 1.3334019 | 0.00036 |
| CD34Mobilized | 1.2918115 | 0.00110 |
| K562 | 1.3347132 | 0.00116 |
| HL60 | 1.3382040 | 0.00144 |
| GM12864 | 1.2938844 | 0.00168 |
| MonocytesCD14RO01746 | 1.2995650 | 0.00338 |
| GM12865 | 1.2450640 | 0.00562 |
| GM06990 | 1.2897629 | 0.00596 |
| GM12878 | 1.2585093 | 0.00650 |
| CD20 | 1.3001616 | 0.00720 |
| GM18507 | 1.2117482 | 0.01598 |
| CLL | 1.2323177 | 0.02038 |
| HPDE6E6E7 | 1.1834141 | 0.02200 |
| GM19240 | 1.1825504 | 0.02280 |
| Th1 | 1.1785263 | 0.02394 |
| UrotheliaUT189 | 1.1854961 | 0.02614 |
| GM19238 | 1.1959654 | 0.02732 |
| GM12892 | 1.2179025 | 0.02776 |
| Urothelia | 1.1748206 | 0.03142 |
| Th0 | 1.1700540 | 0.03452 |
| Huh7 | 1.1850376 | 0.03710 |
| MCF7 | 1.1540943 | 0.04960 |
| MCF7Hypoxia | 1.1758578 | 0.05136 |
| Huh7.5 | 1.1733492 | 0.05270 |
| GM19239 | 1.1703922 | 0.06096 |
| HTR8svn | 1.1373819 | 0.06474 |
| HIPEpiC | 1.1047349 | 0.06910 |
| HMVECdAd | 1.1297325 | 0.07264 |
| HMF | 1.1081135 | 0.07566 |
| PanIsletD | 1.1105930 | 0.07656 |
| HCM | 1.1019371 | 0.07704 |
| HeLaS3IFNa4h | 1.1477510 | 0.07928 |
| GM12891 | 1.1595022 | 0.08128 |
| HMVECLBl | 1.1124837 | 0.08438 |
| HCPEpiC | 1.0981892 | 0.08744 |
| HAEpiC | 1.1045544 | 0.08880 |
| Stellate | 1.1236353 | 0.09372 |
| WI38 | 1.1090217 | 0.09464 |
| HepG2 | 1.1280257 | 0.10010 |
| HVMF | 1.1068883 | 0.10056 |
| AG10803 | 1.1013176 | 0.10688 |
| HMVECdNeo | 1.1080339 | 0.10726 |
| NHEK | 1.1043350 | 0.10784 |
| HMVECLLy | 1.1066381 | 0.10990 |
| HMVECdLyAd | 1.1066484 | 0.11080 |
| Myometr | 1.1009236 | 0.11134 |
| HeLaS3 | 1.1145112 | 0.11286 |
| HConF | 1.0977398 | 0.11294 |
| NT2D1 | 1.0873962 | 0.11516 |
| PrEC | 1.0815447 | 0.11734 |
| HGF | 1.0964440 | 0.12242 |
| HEEpiC | 1.0783968 | 0.12570 |
| ProgFib | 1.1030488 | 0.13102 |
| HPF | 1.0909388 | 0.13224 |
| BJ | 1.0879637 | 0.13288 |
| NHDFAd | 1.0813900 | 0.13550 |
| RPTEC | 1.0748685 | 0.13720 |
| HMVECdLyNeo | 1.0925234 | 0.13800 |
| AoSMC | 1.0747160 | 0.13850 |
| IshikawaEstradiol | 1.0930725 | 0.14080 |
| HSMM | 1.0740730 | 0.14350 |
| HPAF | 1.0760376 | 0.15242 |
| FibroP | 1.0687867 | 0.15288 |
| HNPCEpiC | 1.0692956 | 0.15520 |
| HCFaa | 1.0740300 | 0.15526 |
| LNCaP | 1.0849968 | 0.16470 |
| HFF | 1.0723800 | 0.16766 |
| PanIslets | 1.0897976 | 0.17316 |
| HCF | 1.0736811 | 0.17354 |
| HSMMemb | 1.0808837 | 0.17580 |
| Caco2 | 1.1384151 | 0.17606 |
| LNCaPAndrogen | 1.0944939 | 0.17628 |
| AG09309 | 1.0667526 | 0.17786 |
| Medullo | 1.0887936 | 0.17832 |
| HMEC | 1.0753213 | 0.18242 |
| HPdLF | 1.0752043 | 0.18306 |
| SAEC | 1.0611786 | 0.18454 |
| pHTE | 1.0784194 | 0.18742 |
| H9ES | 1.0839827 | 0.18754 |
| Hepatocytes | 1.0991542 | 0.18766 |
| AG04450 | 1.0723844 | 0.18896 |
| HFFMyc | 1.0635057 | 0.19358 |
| AG09319 | 1.0718863 | 0.19370 |
| HSMMtube | 1.0640399 | 0.19386 |
| RWPE1 | 1.0717647 | 0.19708 |
| HCT116 | 1.0775427 | 0.19736 |
| AG04449 | 1.0644999 | 0.20224 |
| T47D | 1.0849497 | 0.21158 |
| Gliobla | 1.0796510 | 0.21256 |
| HMVECdBlNeo | 1.0656905 | 0.21358 |
| SKNSHRA | 1.0917236 | 0.21490 |
| IshikawaTamoxifen | 1.0694078 | 0.21774 |
| SKMC | 1.0573912 | 0.22122 |
| iPS | 1.0869776 | 0.22528 |
| NHDFneo | 1.0597367 | 0.22562 |
| Fibrobl | 1.0647860 | 0.22680 |
| HRGEC | 1.0592950 | 0.23030 |
| HRE | 1.0512895 | 0.23336 |
| HUVEC | 1.0628704 | 0.23368 |
| HRCEpiC | 1.0511323 | 0.23634 |
| Melano | 1.0466816 | 0.23728 |
| HMVECdBlAd | 1.0567079 | 0.23966 |
| 8988T | 1.0819870 | 0.24304 |
| AoAF | 1.0526865 | 0.25038 |
| H1hESC | 1.0685922 | 0.25134 |
| HPAEC | 1.0541258 | 0.25612 |
| BE2C | 1.0582441 | 0.26478 |
| Osteobl | 1.0545100 | 0.27272 |
| HAc | 1.0422921 | 0.27498 |
| PANC1 | 1.0494765 | 0.29494 |
| A549 | 1.0451085 | 0.31474 |
| HAsp | 1.0268042 | 0.35778 |
| HBMEC | 1.0243105 | 0.36552 |
| HAh | 1.0217648 | 0.37338 |
| Chorion | 1.0355440 | 0.37354 |
| WERIRb1 | 1.0228239 | 0.38298 |
| NHA | 1.0213097 | 0.38636 |
| NHLF | 1.0181842 | 0.40056 |
| HRPEpiC | 1.0147569 | 0.41422 |
| SKNMC | 1.0079670 | 0.45850 |
| H7hESC | 0.9956367 | 0.52390 |

# Chronic lymphocytic leukemia 1

| DHS sample | fold enrichment | p value |
|---|---|---|
| GM12865 | 1.7674472 | 0.00006 |
| Th2 | 1.8206136 | 0.00016 |
| GM12864 | 1.7547635 | 0.00016 |
| GM06990 | 1.7210479 | 0.00116 |
| GM12878 | 1.5360937 | 0.00760 |
| GM18507 | 1.4548853 | 0.01414 |
| Th1 | 1.3608967 | 0.02688 |
| CD20 | 1.4789989 | 0.02990 |
| Th0 | 1.3650511 | 0.03012 |
| GM19240 | 1.3445531 | 0.03506 |
| Jurkat | 1.3125973 | 0.03758 |
| CLL | 1.4114017 | 0.04008 |
| GM19238 | 1.3513904 | 0.04858 |
| MonocytesCD14RO01746 | 1.3371672 | 0.05784 |
| GM12892 | 1.3278126 | 0.08236 |
| SAEC | 1.1785937 | 0.09346 |
| HAsp | 1.1941753 | 0.10136 |
| HL60 | 1.2559750 | 0.12056 |
| HPdLF | 1.1897510 | 0.12480 |
| NB4 | 1.2117521 | 0.13394 |
| NHDFAd | 1.1632432 | 0.13460 |
| AG09309 | 1.1658269 | 0.13520 |
| GM19239 | 1.2391993 | 0.13938 |
| SKMC | 1.1556169 | 0.14890 |
| RPTEC | 1.1389238 | 0.15444 |
| HCFaa | 1.1523156 | 0.16140 |
| NHDFneo | 1.1526125 | 0.17556 |
| CD34Mobilized | 1.1603491 | 0.18190 |
| HNPCEpiC | 1.1223267 | 0.18802 |
| HRCEpiC | 1.1260548 | 0.19114 |
| HGF | 1.1431516 | 0.19870 |
| HCPEpiC | 1.1225687 | 0.20076 |
| GM12891 | 1.1926667 | 0.20128 |
| AG10803 | 1.1329660 | 0.21104 |
| AoSMC | 1.1070491 | 0.22616 |
| HBMEC | 1.1055487 | 0.22776 |
| AG09319 | 1.1206087 | 0.23028 |
| Medullo | 1.1400249 | 0.23492 |
| HSMM | 1.1006665 | 0.24002 |
| HMF | 1.1052464 | 0.24160 |
| HPAF | 1.1056058 | 0.24288 |
| HIPEpiC | 1.1006383 | 0.24522 |
| Melano | 1.0893940 | 0.25062 |
| HMVECLBl | 1.1106708 | 0.25232 |
| HEEpiC | 1.0807708 | 0.27300 |
| WI38 | 1.1011223 | 0.27320 |
| HSMMtube | 1.0893189 | 0.27504 |
| HFF | 1.0872160 | 0.28380 |
| AoAF | 1.0863827 | 0.28908 |
| HCT116 | 1.0960204 | 0.29018 |
| HRGEC | 1.0854525 | 0.29852 |
| PrEC | 1.0686914 | 0.30654 |
| BJ | 1.0780964 | 0.30954 |
| HVMF | 1.0792796 | 0.31462 |
| HCM | 1.0710324 | 0.31790 |
| HFFMyc | 1.0702365 | 0.31910 |
| HPF | 1.0742595 | 0.32066 |
| HPDE6E6E7 | 1.0793054 | 0.32158 |
| HCF | 1.0721395 | 0.32510 |
| AG04450 | 1.0711423 | 0.32856 |
| AG04449 | 1.0676222 | 0.33312 |
| pHTE | 1.0704353 | 0.33826 |
| SKNMC | 1.0608177 | 0.34446 |
| Myometr | 1.0566059 | 0.35254 |
| FibroP | 1.0484730 | 0.36078 |
| HAc | 1.0456740 | 0.36610 |
| HRPEpiC | 1.0407945 | 0.37760 |
| HMVECdBlAd | 1.0505937 | 0.37792 |
| BE2C | 1.0489980 | 0.37974 |
| RWPE1 | 1.0458595 | 0.38360 |
| HConF | 1.0432804 | 0.38880 |
| ProgFib | 1.0393813 | 0.40166 |
| PanIslets | 1.0404239 | 0.40396 |
| PanIsletD | 1.0342586 | 0.40532 |
| IshikawaEstradiol | 1.0346905 | 0.40936 |
| HPAEC | 1.0364767 | 0.41072 |
| HAh | 1.0231795 | 0.43004 |
| Fibrobl | 1.0289748 | 0.43024 |
| HAEpiC | 1.0247991 | 0.43238 |
| Urothelia | 1.0265608 | 0.43360 |
| HSMMemb | 1.0229825 | 0.44066 |
| NHLF | 1.0183571 | 0.44772 |
| NHA | 1.0171408 | 0.44924 |
| HeLaS3 | 1.0131390 | 0.45662 |
| IshikawaTamoxifen | 1.0104096 | 0.46112 |
| Stellate | 1.0111220 | 0.46502 |
| HMEC | 1.0026698 | 0.48174 |
| HRE | 1.0042412 | 0.48384 |
| 8988T | 0.9905685 | 0.49362 |
| NHEK | 0.9967017 | 0.49522 |
| PANC1 | 0.9930679 | 0.49978 |
| Hepatocytes | 0.9763398 | 0.52332 |
| HepG2 | 0.9808888 | 0.52426 |
| Osteobl | 0.9839053 | 0.52902 |
| SKNSHRA | 0.9619490 | 0.54158 |
| LNCaPAndrogen | 0.9618207 | 0.55518 |
| T47D | 0.9546267 | 0.56090 |
| HMVECdBlNeo | 0.9681553 | 0.56718 |
| HUVEC | 0.9560760 | 0.58432 |
| iPS | 0.9285097 | 0.60052 |
| HTR8svn | 0.9436143 | 0.61646 |
| H9ES | 0.9357952 | 0.61686 |
| LNCaP | 0.9391546 | 0.61842 |
| MCF7 | 0.9309373 | 0.63064 |
| Chorion | 0.9086764 | 0.63092 |
| WERIRb1 | 0.9358892 | 0.64586 |
| Gliobla | 0.8998605 | 0.67676 |
| HMVECdLyNeo | 0.9161757 | 0.68124 |
| UrotheliaUT189 | 0.9087874 | 0.68442 |
| HMVECLLy | 0.9124574 | 0.68586 |
| K562 | 0.8950379 | 0.68618 |
| HMVECdAd | 0.9087868 | 0.68658 |
| HMVECdNeo | 0.9074741 | 0.69222 |
| HMVECdLyAd | 0.9013625 | 0.70036 |
| CMK | 0.8683750 | 0.70710 |
| H1hESC | 0.8629807 | 0.73228 |
| MCF7Hypoxia | 0.8576319 | 0.74026 |
| HeLaS3IFNa4h | 0.8609523 | 0.74186 |
| Huh7.5 | 0.8554847 | 0.74226 |
| Caco2 | 0.7911867 | 0.75408 |
| H7hESC | 0.9163593 | 0.76882 |
| A549 | 0.8385604 | 0.79628 |
| NT2D1 | 0.8800273 | 0.80214 |
| Huh7 | 0.8148190 | 0.81104 |

# Erythrocyte phenotypes

| DHS sample | fold enrichment | p value |
|---|---|---|
| CMK | 1.722972 | 0.00008 |
| Th2 | 1.675617 | 0.00008 |
| NB4 | 1.549735 | 0.00014 |
| HL60 | 1.595314 | 0.00036 |
| GM12864 | 1.522364 | 0.00040 |
| CD34Mobilized | 1.482296 | 0.00056 |
| MonocytesCD14RO01746 | 1.569433 | 0.00066 |
| GM06990 | 1.560170 | 0.00108 |
| K562 | 1.460461 | 0.00164 |
| GM12865 | 1.427172 | 0.00176 |
| CD20 | 1.549188 | 0.00180 |
| Jurkat | 1.364040 | 0.00406 |
| GM12878 | 1.426252 | 0.00462 |
| GM18507 | 1.401045 | 0.00530 |
| HPDE6E6E7 | 1.361750 | 0.00548 |
| CLL | 1.421230 | 0.00730 |
| HTR8svn | 1.289823 | 0.01860 |
| HCM | 1.237357 | 0.01954 |
| HPAF | 1.234884 | 0.02232 |
| Urothelia | 1.285224 | 0.02316 |
| GM19238 | 1.305452 | 0.02472 |
| HMVECLBl | 1.250931 | 0.02490 |
| Huh7 | 1.304784 | 0.02544 |
| HCF | 1.241708 | 0.02622 |
| GM19239 | 1.328470 | 0.02646 |
| HEEpiC | 1.202651 | 0.02672 |
| RPTEC | 1.201299 | 0.02756 |
| Myometr | 1.242179 | 0.02786 |
| HMVECdAd | 1.266964 | 0.02802 |
| HAEpiC | 1.238199 | 0.02848 |
| GM19240 | 1.265073 | 0.02896 |
| NHEK | 1.245440 | 0.02922 |
| HMVECLLy | 1.245357 | 0.03120 |
| HMVECdNeo | 1.244076 | 0.03546 |
| Th1 | 1.247838 | 0.03684 |
| PanIsletD | 1.211359 | 0.03748 |
| Stellate | 1.256508 | 0.03958 |
| SAEC | 1.178613 | 0.04004 |
| IshikawaEstradiol | 1.229848 | 0.04284 |
| HPAEC | 1.222997 | 0.04324 |
| HVMF | 1.229224 | 0.04508 |
| AG04449 | 1.210742 | 0.04520 |
| Th0 | 1.244552 | 0.04544 |
| HeLaS3IFNa4h | 1.279201 | 0.04606 |
| GM12892 | 1.290700 | 0.04612 |
| Huh7.5 | 1.267331 | 0.04658 |
| HIPEpiC | 1.188082 | 0.04840 |
| PrEC | 1.171266 | 0.04920 |
| HepG2 | 1.250843 | 0.04926 |
| HMVECdLyNeo | 1.217017 | 0.05152 |
| PANC1 | 1.234516 | 0.05198 |
| HCPEpiC | 1.180585 | 0.05466 |
| AoSMC | 1.172517 | 0.05678 |
| HConF | 1.195337 | 0.05804 |
| AG10803 | 1.202130 | 0.05976 |
| UrotheliaUT189 | 1.218401 | 0.06022 |
| HRGEC | 1.188468 | 0.06244 |
| GM12891 | 1.262634 | 0.06426 |
| LNCaPAndrogen | 1.238864 | 0.06472 |
| HRCEpiC | 1.168489 | 0.06492 |
| WI38 | 1.202255 | 0.06632 |
| HMVECdLyAd | 1.204893 | 0.06668 |
| HMVECdBlAd | 1.189726 | 0.06686 |
| ProgFib | 1.207576 | 0.06694 |
| HPF | 1.188072 | 0.06736 |
| HMVECdBlNeo | 1.187672 | 0.07116 |
| AG09309 | 1.165993 | 0.07380 |
| HFF | 1.169837 | 0.07686 |
| HSMMemb | 1.189008 | 0.07938 |
| HeLaS3 | 1.205137 | 0.08078 |
| IshikawaTamoxifen | 1.190411 | 0.08276 |
| HMF | 1.165133 | 0.08284 |
| FibroP | 1.144569 | 0.08312 |
| HPdLF | 1.174243 | 0.08512 |
| SKNSHRA | 1.241330 | 0.08754 |
| HGF | 1.177957 | 0.08754 |
| H9ES | 1.194542 | 0.08824 |
| BE2C | 1.183996 | 0.08942 |
| HUVEC | 1.177039 | 0.09068 |
| HCT116 | 1.190517 | 0.09304 |
| MCF7 | 1.187017 | 0.09364 |
| HRE | 1.141750 | 0.09442 |
| BJ | 1.155331 | 0.10986 |
| HNPCEpiC | 1.131084 | 0.11082 |
| Gliobla | 1.186903 | 0.11290 |
| RWPE1 | 1.154034 | 0.11498 |
| HCFaa | 1.140179 | 0.11700 |
| NHDFAd | 1.133404 | 0.12076 |
| PanIslets | 1.164324 | 0.12196 |
| HRPEpiC | 1.120075 | 0.12582 |
| MCF7Hypoxia | 1.184147 | 0.12728 |
| AoAF | 1.138508 | 0.12772 |
| pHTE | 1.149251 | 0.13062 |
| Hepatocytes | 1.180348 | 0.13920 |
| HSMM | 1.114723 | 0.14186 |
| AG04450 | 1.137207 | 0.14438 |
| Caco2 | 1.232084 | 0.15052 |
| SKMC | 1.117036 | 0.15244 |
| HBMEC | 1.113550 | 0.15326 |
| HAc | 1.111972 | 0.15528 |
| HFFMyc | 1.117677 | 0.15628 |
| H1hESC | 1.151037 | 0.16350 |
| NHA | 1.111780 | 0.16554 |
| Melano | 1.096106 | 0.17242 |
| T47D | 1.149020 | 0.17662 |
| Fibrobl | 1.118324 | 0.17926 |
| AG09319 | 1.118239 | 0.18052 |
| LNCaP | 1.120993 | 0.18384 |
| HAh | 1.092276 | 0.18556 |
| Medullo | 1.123782 | 0.19344 |
| H7hESC | 1.071759 | 0.19630 |
| WERIRb1 | 1.099492 | 0.19688 |
| HSMMtube | 1.094327 | 0.20190 |
| HMEC | 1.101559 | 0.20620 |
| NHLF | 1.086040 | 0.22056 |
| A549 | 1.111228 | 0.22110 |
| HAsp | 1.085930 | 0.23064 |
| SKNMC | 1.088718 | 0.23184 |
| NT2D1 | 1.077563 | 0.23206 |
| Osteobl | 1.092496 | 0.24078 |
| iPS | 1.120545 | 0.24080 |
| NHDFneo | 1.084563 | 0.24540 |
| Chorion | 1.112249 | 0.25718 |
| 8988T | 1.105215 | 0.26636 |

# Megakaryopoiesis and platelet formation

| DHS sample | fold enrichment | p value |
|---|---|---|
| AoSMC | 1.222779 | 0.00008 |
| HCF | 1.240394 | 0.00038 |
| MonocytesCD14RO01746 | 1.300835 | 0.00042 |
| HGF | 1.232748 | 0.00058 |
| AoAF | 1.225222 | 0.00062 |
| NHLF | 1.199367 | 0.00062 |
| HVMF | 1.230759 | 0.00072 |
| HCFaa | 1.205222 | 0.00078 |
| HMVECdBlNeo | 1.216823 | 0.00080 |
| HRPEpiC | 1.192403 | 0.00080 |
| RPTEC | 1.180070 | 0.00094 |
| Th2 | 1.287154 | 0.00096 |
| HMVECLBl | 1.213758 | 0.00098 |
| HMVECLLy | 1.229373 | 0.00106 |
| NHA | 1.196752 | 0.00106 |
| HIPEpiC | 1.188611 | 0.00106 |
| HPdLF | 1.214410 | 0.00110 |
| HRCEpiC | 1.191334 | 0.00110 |
| HPAF | 1.199388 | 0.00112 |
| HAEpiC | 1.208105 | 0.00114 |
| HMVECdLyAd | 1.229592 | 0.00130 |
| HCM | 1.187896 | 0.00130 |
| HMVECdAd | 1.233288 | 0.00138 |
| HAsp | 1.202652 | 0.00138 |
| WI38 | 1.214046 | 0.00144 |
| HAc | 1.184594 | 0.00144 |
| CD34Mobilized | 1.225907 | 0.00146 |
| SAEC | 1.173268 | 0.00176 |
| HMVECdBlAd | 1.198343 | 0.00190 |
| HRGEC | 1.201459 | 0.00206 |
| AG04449 | 1.189592 | 0.00206 |
| HBMEC | 1.176627 | 0.00218 |
| HPAEC | 1.201558 | 0.00220 |
| HFF | 1.180294 | 0.00222 |
| NHDFneo | 1.194185 | 0.00226 |
| SKNMC | 1.208619 | 0.00234 |
| HConF | 1.197214 | 0.00240 |
| HMVECdNeo | 1.211309 | 0.00242 |
| GM06990 | 1.265439 | 0.00258 |
| HTR8svn | 1.211980 | 0.00262 |
| SKMC | 1.176451 | 0.00276 |
| AG09309 | 1.170757 | 0.00278 |
| HMVECdLyNeo | 1.203452 | 0.00286 |
| HEEpiC | 1.163172 | 0.00314 |
| HAh | 1.154594 | 0.00332 |
| HNPCEpiC | 1.162846 | 0.00334 |
| GM12865 | 1.215776 | 0.00338 |
| GM12864 | 1.219730 | 0.00358 |
| NHDFAd | 1.165042 | 0.00380 |
| AG04450 | 1.189848 | 0.00384 |
| HFFMyc | 1.164210 | 0.00418 |
| HCPEpiC | 1.163234 | 0.00418 |
| HRE | 1.158826 | 0.00420 |
| HPF | 1.178764 | 0.00570 |
| AG10803 | 1.170700 | 0.00650 |
| HMF | 1.161757 | 0.00654 |
| K562 | 1.209073 | 0.00700 |
| GM12878 | 1.208850 | 0.00718 |
| HUVEC | 1.184881 | 0.00720 |
| AG09319 | 1.172872 | 0.00732 |
| BJ | 1.167079 | 0.00778 |
| BE2C | 1.192107 | 0.00780 |
| GM18507 | 1.197229 | 0.00812 |
| CD20 | 1.234768 | 0.00894 |
| NB4 | 1.184252 | 0.00910 |
| HPDE6E6E7 | 1.178064 | 0.00970 |
| Myometr | 1.168076 | 0.00976 |
| PanIsletD | 1.148492 | 0.01144 |
| Stellate | 1.177078 | 0.01302 |
| A549 | 1.183747 | 0.01438 |
| HeLaS3 | 1.175819 | 0.01492 |
| HSMM | 1.126379 | 0.01712 |
| NHEK | 1.145962 | 0.01916 |
| HSMMemb | 1.145859 | 0.02156 |
| PrEC | 1.117753 | 0.02224 |
| HL60 | 1.170944 | 0.02640 |
| RWPE1 | 1.138815 | 0.02754 |
| HCT116 | 1.151075 | 0.03056 |
| Melano | 1.099683 | 0.03552 |
| CMK | 1.167943 | 0.03682 |
| FibroP | 1.100822 | 0.03716 |
| GM19238 | 1.147804 | 0.03982 |
| IshikawaTamoxifen | 1.129539 | 0.04318 |
| MCF7 | 1.129995 | 0.04556 |
| CLL | 1.154602 | 0.04654 |
| GM19239 | 1.149273 | 0.04716 |
| HeLaS3IFNa4h | 1.146139 | 0.04894 |
| IshikawaEstradiol | 1.123237 | 0.04950 |
| Th1 | 1.117289 | 0.05350 |
| ProgFib | 1.122523 | 0.05430 |
| GM19240 | 1.118397 | 0.05634 |
| HepG2 | 1.120999 | 0.06706 |
| PANC1 | 1.117504 | 0.07082 |
| T47D | 1.131174 | 0.07336 |
| HSMMtube | 1.091214 | 0.07336 |
| Th0 | 1.109317 | 0.07340 |
| SKNSHRA | 1.141270 | 0.07556 |
| Urothelia | 1.105007 | 0.08240 |
| WERIRb1 | 1.092536 | 0.08266 |
| LNCaPAndrogen | 1.115605 | 0.08736 |
| Jurkat | 1.094119 | 0.09806 |
| GM12891 | 1.117110 | 0.10016 |
| Hepatocytes | 1.119315 | 0.10148 |
| GM12892 | 1.113951 | 0.10286 |
| Gliobla | 1.106121 | 0.10530 |
| MCF7Hypoxia | 1.106839 | 0.10770 |
| 8988T | 1.117425 | 0.11680 |
| Medullo | 1.090697 | 0.12192 |
| LNCaP | 1.085395 | 0.12320 |
| H1hESC | 1.098986 | 0.12398 |
| HMEC | 1.079337 | 0.12524 |
| Fibrobl | 1.078009 | 0.13148 |
| H9ES | 1.083579 | 0.14126 |
| pHTE | 1.077254 | 0.14246 |
| Huh7 | 1.085526 | 0.15446 |
| PanIslets | 1.078910 | 0.15518 |
| NT2D1 | 1.059181 | 0.16122 |
| Huh7.5 | 1.082082 | 0.17166 |
| Chorion | 1.087573 | 0.18298 |
| UrotheliaUT189 | 1.066844 | 0.18756 |
| H7hESC | 1.041505 | 0.19758 |
| Osteobl | 1.058235 | 0.21004 |
| iPS | 1.057610 | 0.26926 |
| Caco2 | 1.056576 | 0.31266 |

# Serum urate concentrations

| DHS sample | fold enrichment | p value |
|---|---|---|
| RPTEC | 1.336450 | 0.00008 |
| HRCEpiC | 1.280872 | 0.00098 |
| MCF7 | 1.346317 | 0.00194 |
| HPF | 1.312320 | 0.00204 |
| HPdLF | 1.286772 | 0.00398 |
| HGF | 1.299608 | 0.00404 |
| AoSMC | 1.229824 | 0.00452 |
| MCF7Hypoxia | 1.346743 | 0.00590 |
| WI38 | 1.261728 | 0.00830 |
| Stellate | 1.296879 | 0.00834 |
| HConF | 1.245064 | 0.01122 |
| A549 | 1.286218 | 0.01314 |
| AG04450 | 1.238542 | 0.01366 |
| Myometr | 1.224181 | 0.01448 |
| PANC1 | 1.263253 | 0.01476 |
| AG04449 | 1.220217 | 0.01486 |
| AG09319 | 1.236293 | 0.01514 |
| NB4 | 1.258721 | 0.01586 |
| AoAF | 1.206729 | 0.02038 |
| HCFaa | 1.195068 | 0.02132 |
| HMF | 1.202728 | 0.02192 |
| BJ | 1.206313 | 0.02248 |
| HSMMemb | 1.223456 | 0.02292 |
| SKMC | 1.193922 | 0.02438 |
| AG10803 | 1.203587 | 0.02454 |
| PanIsletD | 1.191614 | 0.02604 |
| HCF | 1.208618 | 0.02610 |
| HEEpiC | 1.172438 | 0.02610 |
| AG09309 | 1.184991 | 0.02644 |
| HCPEpiC | 1.183602 | 0.02708 |
| HRE | 1.173212 | 0.02758 |
| Huh7 | 1.238325 | 0.02876 |
| IshikawaEstradiol | 1.214482 | 0.02936 |
| HIPEpiC | 1.172579 | 0.02990 |
| HBMEC | 1.175762 | 0.02996 |
| SAEC | 1.165287 | 0.03094 |
| HTR8svn | 1.211465 | 0.03110 |
| HAEpiC | 1.189415 | 0.03136 |
| HSMM | 1.164909 | 0.03320 |
| HFF | 1.179224 | 0.03354 |
| HCM | 1.173307 | 0.03358 |
| NHDFneo | 1.187661 | 0.03502 |
| HPAF | 1.175174 | 0.03780 |
| NHDFAd | 1.164334 | 0.04082 |
| IshikawaTamoxifen | 1.200049 | 0.04220 |
| HNPCEpiC | 1.151529 | 0.04236 |
| HPDE6E6E7 | 1.196804 | 0.04320 |
| HAc | 1.156053 | 0.04778 |
| HMVECdLyAd | 1.189555 | 0.05054 |
| HMVECdAd | 1.191845 | 0.05096 |
| HCT116 | 1.200250 | 0.05452 |
| HepG2 | 1.196577 | 0.05462 |
| SKNSHRA | 1.244944 | 0.05472 |
| K562 | 1.204445 | 0.05528 |
| HAh | 1.136459 | 0.05556 |
| HMVECdNeo | 1.181472 | 0.05602 |
| HRGEC | 1.166426 | 0.05658 |
| Huh7.5 | 1.205620 | 0.05660 |
| T47D | 1.206642 | 0.05946 |
| HAsp | 1.153372 | 0.06120 |
| HeLaS3 | 1.188602 | 0.06334 |
| HVMF | 1.168100 | 0.06482 |
| HMVECLLy | 1.167783 | 0.06656 |
| CMK | 1.211404 | 0.06906 |
| BE2C | 1.180259 | 0.06970 |
| LNCaPAndrogen | 1.185573 | 0.07316 |
| CD34Mobilized | 1.165663 | 0.07626 |
| PrEC | 1.123710 | 0.08050 |
| HeLaS3IFNa4h | 1.187615 | 0.08076 |
| NHEK | 1.145322 | 0.08534 |
| HL60 | 1.183867 | 0.08990 |
| Gliobla | 1.168578 | 0.09170 |
| NHLF | 1.123719 | 0.09364 |
| FibroP | 1.112483 | 0.09384 |
| HMVECdLyNeo | 1.146284 | 0.09436 |
| Th2 | 1.183637 | 0.09464 |
| NHA | 1.125248 | 0.09574 |
| H9ES | 1.151485 | 0.09582 |
| HPAEC | 1.142394 | 0.09784 |
| CD20 | 1.188886 | 0.10120 |
| NT2D1 | 1.114211 | 0.10148 |
| Hepatocytes | 1.174183 | 0.10458 |
| ProgFib | 1.137594 | 0.11216 |
| WERIRb1 | 1.121908 | 0.11244 |
| HFFMyc | 1.115118 | 0.11404 |
| HRPEpiC | 1.105212 | 0.11930 |
| HSMMtube | 1.106421 | 0.12706 |
| HUVEC | 1.128207 | 0.12832 |
| HMVECLBl | 1.119483 | 0.12904 |
| RWPE1 | 1.119608 | 0.12930 |
| Jurkat | 1.117599 | 0.13428 |
| Urothelia | 1.124259 | 0.13738 |
| Medullo | 1.128041 | 0.14150 |
| HMVECdBlAd | 1.111195 | 0.14830 |
| HMVECdBlNeo | 1.111651 | 0.14894 |
| SKNMC | 1.110431 | 0.15600 |
| MonocytesCD14RO01746 | 1.131875 | 0.16356 |
| GM19239 | 1.122888 | 0.17402 |
| CLL | 1.121734 | 0.18590 |
| Melano | 1.069445 | 0.20002 |
| GM19238 | 1.097108 | 0.21150 |
| Th0 | 1.090459 | 0.21796 |
| 8988T | 1.110345 | 0.21804 |
| H7hESC | 1.055350 | 0.22118 |
| GM12878 | 1.093253 | 0.22526 |
| GM18507 | 1.091482 | 0.22540 |
| UrotheliaUT189 | 1.085634 | 0.22672 |
| pHTE | 1.081022 | 0.22968 |
| Caco2 | 1.126232 | 0.23546 |
| PanIslets | 1.076329 | 0.25556 |
| GM12865 | 1.069269 | 0.27588 |
| HMEC | 1.056863 | 0.28896 |
| LNCaP | 1.057071 | 0.29504 |
| GM19240 | 1.058581 | 0.29780 |
| GM12891 | 1.059638 | 0.32794 |
| GM12892 | 1.054736 | 0.34006 |
| H1hESC | 1.041250 | 0.36090 |
| Th1 | 1.037528 | 0.36830 |
| Fibrobl | 1.034982 | 0.37462 |
| GM06990 | 1.039888 | 0.37982 |
| Osteobl | 1.020453 | 0.42254 |
| GM12864 | 1.019195 | 0.42928 |
| iPS | 1.020923 | 0.43256 |
| Chorion | 1.005486 | 0.47676 |

# Height 1

| DHS sample | fold enrichment | p value |
|---|---|---|
| HRGEC | 1.4980273 | 0.00010 |
| HCFaa | 1.4669950 | 0.00014 |
| HPAF | 1.3941383 | 0.00056 |
| PANC1 | 1.5123528 | 0.00070 |
| HPAEC | 1.4380213 | 0.00070 |
| HCM | 1.3530875 | 0.00100 |
| HAsp | 1.3668778 | 0.00112 |
| HNPCEpiC | 1.3264669 | 0.00122 |
| SKMC | 1.3494507 | 0.00142 |
| HCF | 1.3785429 | 0.00156 |
| HMVECdNeo | 1.4206862 | 0.00176 |
| HMVECdLyNeo | 1.4098924 | 0.00190 |
| HIPEpiC | 1.3170504 | 0.00222 |
| SKNMC | 1.3816010 | 0.00244 |
| HMVECLLy | 1.3894106 | 0.00266 |
| HMVECdBlNeo | 1.3814984 | 0.00294 |
| HMVECdLyAd | 1.4007261 | 0.00320 |
| HMVECdBlAd | 1.3579862 | 0.00386 |
| HBMEC | 1.3056231 | 0.00422 |
| HCPEpiC | 1.2923194 | 0.00446 |
| HMF | 1.3046098 | 0.00492 |
| HRPEpiC | 1.2696166 | 0.00556 |
| WI38 | 1.3304635 | 0.00580 |
| HVMF | 1.3440022 | 0.00588 |
| HMVECdAd | 1.3722441 | 0.00592 |
| HMVECLBl | 1.3280594 | 0.00608 |
| HConF | 1.3096798 | 0.00616 |
| NHLF | 1.2799101 | 0.00616 |
| HAEpiC | 1.3070722 | 0.00620 |
| HAc | 1.2656883 | 0.00692 |
| HAh | 1.2523308 | 0.00712 |
| HRE | 1.2476598 | 0.00938 |
| HFFMyc | 1.2684588 | 0.00950 |
| AG04450 | 1.3008423 | 0.00958 |
| HPdLF | 1.2964585 | 0.01026 |
| Jurkat | 1.3238602 | 0.01048 |
| AoSMC | 1.2491035 | 0.01162 |
| SAEC | 1.2412413 | 0.01184 |
| AoAF | 1.2787265 | 0.01202 |
| HEEpiC | 1.2346778 | 0.01268 |
| GM12865 | 1.3311947 | 0.01276 |
| NHA | 1.2498412 | 0.01326 |
| RPTEC | 1.2296350 | 0.01336 |
| HUVEC | 1.3014363 | 0.01414 |
| AG04449 | 1.2635154 | 0.01420 |
| HPF | 1.2712014 | 0.01486 |
| HRCEpiC | 1.2353894 | 0.01518 |
| AG10803 | 1.2725131 | 0.01630 |
| HGF | 1.2742071 | 0.01658 |
| BJ | 1.2422469 | 0.02528 |
| Th2 | 1.3384656 | 0.02824 |
| AG09309 | 1.2132533 | 0.03036 |
| AG09319 | 1.2381773 | 0.03120 |
| HFF | 1.2143366 | 0.03334 |
| PrEC | 1.1923934 | 0.03334 |
| GM12864 | 1.2758187 | 0.03818 |
| HPDE6E6E7 | 1.2282630 | 0.04782 |
| GM06990 | 1.2815002 | 0.05150 |
| HCT116 | 1.2291556 | 0.05432 |
| NHDFAd | 1.1795343 | 0.05786 |
| NHDFneo | 1.1789480 | 0.07474 |
| CD34Mobilized | 1.2027728 | 0.07562 |
| NHEK | 1.1760919 | 0.08406 |
| NB4 | 1.1866497 | 0.10756 |
| GM12878 | 1.1779418 | 0.12386 |
| RWPE1 | 1.1386490 | 0.13818 |
| CD20 | 1.1830908 | 0.15552 |
| PanIsletD | 1.1158722 | 0.16506 |
| MonocytesCD14RO01746 | 1.1612503 | 0.16962 |
| Stellate | 1.1268930 | 0.18934 |
| HTR8svn | 1.1134494 | 0.19742 |
| HL60 | 1.1280592 | 0.21438 |
| HeLaS3 | 1.1063575 | 0.21774 |
| K562 | 1.1032262 | 0.24196 |
| CMK | 1.1105214 | 0.24670 |
| H7hESC | 1.0507625 | 0.27516 |
| A549 | 1.0792602 | 0.28444 |
| HSMM | 1.0556137 | 0.29584 |
| NT2D1 | 1.0563148 | 0.29852 |
| MCF7 | 1.0629598 | 0.30666 |
| WERIRb1 | 1.0562592 | 0.31186 |
| GM18507 | 1.0601364 | 0.33724 |
| T47D | 1.0564572 | 0.35154 |
| Myometr | 1.0313852 | 0.39240 |
| HepG2 | 1.0304865 | 0.41050 |
| FibroP | 1.0211791 | 0.41936 |
| CLL | 1.0230536 | 0.43474 |
| IshikawaEstradiol | 1.0150217 | 0.44238 |
| MCF7Hypoxia | 1.0110517 | 0.46162 |
| SKNSHRA | 0.9940620 | 0.49354 |
| Urothelia | 0.9993208 | 0.49494 |
| HeLaS3IFNa4h | 0.9950108 | 0.50084 |
| BE2C | 0.9931038 | 0.50714 |
| HSMMemb | 0.9933310 | 0.51258 |
| IshikawaTamoxifen | 0.9833602 | 0.53682 |
| GM19239 | 0.9725074 | 0.55384 |
| ProgFib | 0.9773192 | 0.55686 |
| Gliobla | 0.9696415 | 0.56668 |
| Huh7 | 0.9610715 | 0.59272 |
| HSMMtube | 0.9713754 | 0.59532 |
| H9ES | 0.9608383 | 0.60114 |
| Th0 | 0.9573547 | 0.60984 |
| UrotheliaUT189 | 0.9568423 | 0.61126 |
| Melano | 0.9634069 | 0.63750 |
| GM19240 | 0.9468728 | 0.64094 |
| GM19238 | 0.9390329 | 0.64470 |
| HMEC | 0.9384755 | 0.67940 |
| Huh7.5 | 0.9237337 | 0.68310 |
| Th1 | 0.9157605 | 0.71656 |
| H1hESC | 0.9047531 | 0.72572 |
| pHTE | 0.9150721 | 0.72830 |
| Caco2 | 0.8475708 | 0.73128 |
| GM12891 | 0.8811386 | 0.74632 |
| Chorion | 0.8303416 | 0.82984 |
| PanIslets | 0.8619170 | 0.83256 |
| LNCaP | 0.8720485 | 0.84082 |
| GM12892 | 0.8277027 | 0.84214 |
| Medullo | 0.8534398 | 0.84258 |
| Fibrobl | 0.8580829 | 0.84696 |
| iPS | 0.8086177 | 0.85542 |
| 8988T | 0.8016770 | 0.87356 |
| LNCaPAndrogen | 0.8265875 | 0.87528 |
| Osteobl | 0.8188997 | 0.90276 |
| Hepatocytes | 0.7796806 | 0.90736 |

# Skeletal frame size

| DHS sample | fold enrichment | p value |
|---|---|---|
| PANC1 | 1.9096689 | 0.00010 |
| HCFaa | 1.6402361 | 0.00014 |
| HAsp | 1.5975611 | 0.00036 |
| HRGEC | 1.6374724 | 0.00072 |
| HIPEpiC | 1.5348436 | 0.00072 |
| HNPCEpiC | 1.5303458 | 0.00078 |
| Jurkat | 1.6776355 | 0.00098 |
| SKMC | 1.5405199 | 0.00100 |
| HRPEpiC | 1.4639326 | 0.00110 |
| HCPEpiC | 1.5191282 | 0.00138 |
| HFFMyc | 1.5237231 | 0.00148 |
| AG04450 | 1.6066218 | 0.00158 |
| HCM | 1.5106940 | 0.00160 |
| HPF | 1.5795989 | 0.00162 |
| GM12865 | 1.6954877 | 0.00174 |
| NHDFneo | 1.5646731 | 0.00186 |
| HAEpiC | 1.5439601 | 0.00186 |
| HPAF | 1.5319605 | 0.00210 |
| HVMF | 1.6021076 | 0.00220 |
| AoSMC | 1.4724995 | 0.00236 |
| AG04449 | 1.5349549 | 0.00248 |
| HRE | 1.4666983 | 0.00254 |
| NHLF | 1.4775702 | 0.00296 |
| SAEC | 1.4437055 | 0.00298 |
| NHDFAd | 1.4770025 | 0.00302 |
| HBMEC | 1.4746198 | 0.00316 |
| HCF | 1.5174542 | 0.00332 |
| HPAEC | 1.5836390 | 0.00354 |
| SKNMC | 1.5190892 | 0.00366 |
| HMF | 1.5049899 | 0.00372 |
| HAh | 1.4085366 | 0.00410 |
| HAc | 1.4460270 | 0.00414 |
| AG10803 | 1.5118772 | 0.00424 |
| HConF | 1.4980467 | 0.00450 |
| Th2 | 1.7302986 | 0.00452 |
| HMVECdLyNeo | 1.5626647 | 0.00456 |
| GM12864 | 1.6379981 | 0.00466 |
| AG09319 | 1.5353142 | 0.00468 |
| BJ | 1.5009856 | 0.00480 |
| AG09309 | 1.4562953 | 0.00506 |
| HEEpiC | 1.4129223 | 0.00536 |
| NHA | 1.4567838 | 0.00554 |
| WI38 | 1.5211694 | 0.00568 |
| HMVECdBlNeo | 1.5384523 | 0.00574 |
| RPTEC | 1.4084609 | 0.00594 |
| HMVECLBl | 1.5360774 | 0.00602 |
| HRCEpiC | 1.4283136 | 0.00606 |
| HPdLF | 1.4855549 | 0.00660 |
| HCT116 | 1.5927960 | 0.00680 |
| HFF | 1.4532611 | 0.00680 |
| AoAF | 1.4535805 | 0.00840 |
| CD34Mobilized | 1.5377596 | 0.00904 |
| HMVECdNeo | 1.5118630 | 0.00924 |
| NB4 | 1.5463751 | 0.00994 |
| HGF | 1.4641121 | 0.01028 |
| GM06990 | 1.6368773 | 0.01060 |
| CD20 | 1.6925160 | 0.01116 |
| HMVECdBlAd | 1.4570462 | 0.01312 |
| HMVECdLyAd | 1.4657890 | 0.01590 |
| HMVECLLy | 1.4652589 | 0.01622 |
| HMVECdAd | 1.4767623 | 0.01726 |
| PrEC | 1.3422669 | 0.01786 |
| MonocytesCD14RO01746 | 1.5179343 | 0.02314 |
| HUVEC | 1.4207174 | 0.02428 |
| GM12878 | 1.4940556 | 0.02714 |
| RWPE1 | 1.3654346 | 0.03628 |
| NHEK | 1.3574081 | 0.03686 |
| HPDE6E6E7 | 1.3795081 | 0.03778 |
| BE2C | 1.3598305 | 0.04336 |
| GM18507 | 1.4153354 | 0.04584 |
| HL60 | 1.4182932 | 0.05084 |
| NT2D1 | 1.2638254 | 0.05670 |
| HSMMemb | 1.3095437 | 0.07146 |
| HTR8svn | 1.3179282 | 0.07280 |
| Stellate | 1.3269332 | 0.08068 |
| H7hESC | 1.1713353 | 0.08258 |
| K562 | 1.3413531 | 0.08558 |
| PanIsletD | 1.2531169 | 0.08722 |
| CMK | 1.3653702 | 0.08848 |
| WERIRb1 | 1.2324589 | 0.09454 |
| HSMM | 1.2127060 | 0.10126 |
| HepG2 | 1.2929644 | 0.10258 |
| IshikawaEstradiol | 1.2546780 | 0.10506 |
| UrotheliaUT189 | 1.2675067 | 0.10884 |
| Urothelia | 1.2659294 | 0.11172 |
| CLL | 1.3263703 | 0.11178 |
| Gliobla | 1.2844921 | 0.11520 |
| T47D | 1.2823512 | 0.12204 |
| FibroP | 1.1839682 | 0.13104 |
| A549 | 1.2466694 | 0.13854 |
| HeLaS3 | 1.2263118 | 0.14514 |
| ProgFib | 1.2030614 | 0.16714 |
| Myometr | 1.1813276 | 0.16822 |
| IshikawaTamoxifen | 1.1950069 | 0.16976 |
| MCF7 | 1.1766211 | 0.17740 |
| HSMMtube | 1.1515094 | 0.18912 |
| GM19238 | 1.1983287 | 0.20074 |
| SKNSHRA | 1.2065304 | 0.20432 |
| GM19240 | 1.1723057 | 0.21156 |
| Th0 | 1.1725194 | 0.22090 |
| Huh7 | 1.1593697 | 0.23904 |
| Th1 | 1.1516816 | 0.24030 |
| H9ES | 1.1376247 | 0.24538 |
| GM19239 | 1.1613732 | 0.25422 |
| Melano | 1.0976392 | 0.25894 |
| HeLaS3IFNa4h | 1.1344248 | 0.27176 |
| MCF7Hypoxia | 1.1296400 | 0.27788 |
| Huh7.5 | 1.1092779 | 0.31046 |
| HMEC | 1.0867585 | 0.31318 |
| GM12891 | 1.1143492 | 0.32006 |
| H1hESC | 1.0918742 | 0.32288 |
| Caco2 | 1.1042026 | 0.34918 |
| LNCaP | 1.0618812 | 0.35688 |
| pHTE | 1.0639425 | 0.36762 |
| GM12892 | 1.0469758 | 0.41528 |
| Fibrobl | 1.0189502 | 0.45306 |
| PanIslets | 1.0148287 | 0.46042 |
| LNCaPAndrogen | 0.9764485 | 0.52182 |
| Chorion | 0.9634395 | 0.53128 |
| Osteobl | 0.9643117 | 0.55972 |
| Medullo | 0.9594746 | 0.56024 |
| iPS | 0.9460825 | 0.56206 |
| Hepatocytes | 0.9276404 | 0.59144 |
| 8988T | 0.9057955 | 0.62242 |

# Hematological parameters

| DHS sample | fold enrichment | p value |
|---|---|---|
| RPTEC | 1.3647444 | 0.00016 |
| Th2 | 1.6326418 | 0.00018 |
| HCM | 1.3762693 | 0.00024 |
| GM06990 | 1.6002014 | 0.00056 |
| HRE | 1.3455672 | 0.00060 |
| HEEpiC | 1.3357606 | 0.00070 |
| SAEC | 1.3333368 | 0.00090 |
| HRCEpiC | 1.3371988 | 0.00094 |
| HCF | 1.3802968 | 0.00096 |
| GM12864 | 1.4583502 | 0.00112 |
| GM12865 | 1.4378644 | 0.00146 |
| HPAF | 1.3506392 | 0.00156 |
| HFF | 1.3323949 | 0.00158 |
| AoSMC | 1.3100522 | 0.00176 |
| AG09309 | 1.3236246 | 0.00188 |
| HAEpiC | 1.3544099 | 0.00194 |
| HFFMyc | 1.3276971 | 0.00206 |
| HNPCEpiC | 1.3071347 | 0.00232 |
| HPDE6E6E7 | 1.3798782 | 0.00308 |
| HVMF | 1.3673259 | 0.00314 |
| HIPEpiC | 1.3082096 | 0.00352 |
| BJ | 1.3373895 | 0.00360 |
| WI38 | 1.3472931 | 0.00372 |
| AoAF | 1.3290123 | 0.00372 |
| PrEC | 1.2759314 | 0.00378 |
| HAsp | 1.3278423 | 0.00414 |
| HCFaa | 1.3097142 | 0.00418 |
| HCPEpiC | 1.2932707 | 0.00464 |
| HMF | 1.3081015 | 0.00480 |
| HRPEpiC | 1.2922880 | 0.00484 |
| HBMEC | 1.2873054 | 0.00514 |
| HPF | 1.3260247 | 0.00540 |
| NHLF | 1.2991802 | 0.00562 |
| AG04449 | 1.3083538 | 0.00596 |
| AG10803 | 1.3189678 | 0.00628 |
| HConF | 1.3131249 | 0.00628 |
| Jurkat | 1.3224413 | 0.00662 |
| AG09319 | 1.3201023 | 0.00698 |
| AG04450 | 1.3193636 | 0.00712 |
| HPdLF | 1.3139635 | 0.00714 |
| NB4 | 1.3563538 | 0.00718 |
| HRGEC | 1.2939469 | 0.00850 |
| HMVECLBl | 1.3020534 | 0.00858 |
| HTR8svn | 1.3235332 | 0.01040 |
| K562 | 1.3665045 | 0.01050 |
| NHDFneo | 1.2854734 | 0.01056 |
| CD20 | 1.4431996 | 0.01086 |
| NHDFAd | 1.2542720 | 0.01116 |
| GM12878 | 1.3658066 | 0.01184 |
| A549 | 1.3475233 | 0.01286 |
| CMK | 1.3780906 | 0.01414 |
| MonocytesCD14RO01746 | 1.3544031 | 0.01572 |
| NHEK | 1.2763676 | 0.01610 |
| NHA | 1.2575531 | 0.01718 |
| HMVECdLyAd | 1.2882280 | 0.01764 |
| SKMC | 1.2467877 | 0.01794 |
| HMVECdBlNeo | 1.2667537 | 0.01816 |
| HGF | 1.2716684 | 0.01822 |
| HMVECLLy | 1.2818398 | 0.01844 |
| GM18507 | 1.3191032 | 0.01872 |
| SKNMC | 1.2700605 | 0.01978 |
| HCT116 | 1.3006439 | 0.02154 |
| PANC1 | 1.2885762 | 0.02174 |
| CD34Mobilized | 1.2808934 | 0.02216 |
| HPAEC | 1.2462432 | 0.02584 |
| HMVECdAd | 1.2672078 | 0.02794 |
| Urothelia | 1.2584208 | 0.02822 |
| HAc | 1.2099287 | 0.02860 |
| HL60 | 1.3132229 | 0.02934 |
| HAh | 1.1911373 | 0.02942 |
| HMVECdNeo | 1.2488026 | 0.03196 |
| NT2D1 | 1.2064170 | 0.03308 |
| HMVECdBlAd | 1.2263093 | 0.03590 |
| RWPE1 | 1.2313440 | 0.03660 |
| CLL | 1.3111086 | 0.03716 |
| HMVECdLyNeo | 1.2313584 | 0.04024 |
| UrotheliaUT189 | 1.2211163 | 0.05412 |
| Th1 | 1.2204919 | 0.05488 |
| H7hESC | 1.1349576 | 0.06056 |
| PanIsletD | 1.1846609 | 0.06126 |
| Myometr | 1.1928959 | 0.06242 |
| Stellate | 1.2141710 | 0.07244 |
| HeLaS3 | 1.2093979 | 0.07842 |
| FibroP | 1.1395425 | 0.08646 |
| HSMMemb | 1.1767038 | 0.09186 |
| Th0 | 1.1799641 | 0.10200 |
| HepG2 | 1.1843841 | 0.10548 |
| GM19240 | 1.1696181 | 0.11044 |
| GM19238 | 1.1806265 | 0.12240 |
| HUVEC | 1.1588101 | 0.12388 |
| WERIRb1 | 1.1309159 | 0.13920 |
| HeLaS3IFNa4h | 1.1638565 | 0.15116 |
| Melano | 1.0993477 | 0.15858 |
| IshikawaEstradiol | 1.1240087 | 0.17274 |
| H9ES | 1.1227137 | 0.19072 |
| HSMM | 1.0922977 | 0.19732 |
| Gliobla | 1.1247159 | 0.19970 |
| GM19239 | 1.1328685 | 0.20574 |
| ProgFib | 1.1094333 | 0.20986 |
| BE2C | 1.1124342 | 0.20992 |
| GM12891 | 1.1171367 | 0.24282 |
| HMEC | 1.0811451 | 0.25986 |
| GM12892 | 1.1036216 | 0.26536 |
| Huh7 | 1.0905307 | 0.26652 |
| IshikawaTamoxifen | 1.0727682 | 0.29072 |
| SKNSHRA | 1.0925807 | 0.29160 |
| HSMMtube | 1.0472631 | 0.33630 |
| T47D | 1.0606424 | 0.34358 |
| Fibrobl | 1.0497674 | 0.34810 |
| LNCaPAndrogen | 1.0513814 | 0.35804 |
| MCF7Hypoxia | 1.0499159 | 0.36048 |
| MCF7 | 1.0422875 | 0.37052 |
| H1hESC | 1.0423921 | 0.38020 |
| pHTE | 1.0377099 | 0.38310 |
| Huh7.5 | 1.0277183 | 0.42006 |
| Hepatocytes | 1.0084422 | 0.46710 |
| LNCaP | 0.9950963 | 0.50276 |
| 8988T | 0.9920840 | 0.50446 |
| PanIslets | 0.9958496 | 0.50670 |
| Osteobl | 0.9923192 | 0.52166 |
| Caco2 | 0.9720527 | 0.53422 |
| Medullo | 0.9724412 | 0.57328 |
| iPS | 0.9518993 | 0.60018 |
| Chorion | 0.9236338 | 0.65438 |

# Coronary artery disease

| DHS sample | fold enrichment | p value |
|---|---|---|
| HFF | 1.408275 | 0.00020 |
| NHDFneo | 1.427050 | 0.00036 |
| HAh | 1.347306 | 0.00036 |
| AG09319 | 1.437889 | 0.00040 |
| AG10803 | 1.418258 | 0.00042 |
| HPAF | 1.375239 | 0.00044 |
| NHDFAd | 1.375521 | 0.00054 |
| HGF | 1.407218 | 0.00076 |
| WI38 | 1.403673 | 0.00076 |
| HCF | 1.378403 | 0.00080 |
| HMF | 1.373194 | 0.00080 |
| HAEpiC | 1.371393 | 0.00082 |
| HFFMyc | 1.351167 | 0.00084 |
| HCM | 1.349818 | 0.00086 |
| HCFaa | 1.360684 | 0.00092 |
| HVMF | 1.410187 | 0.00096 |
| HNPCEpiC | 1.318101 | 0.00096 |
| AG04449 | 1.367579 | 0.00110 |
| HCPEpiC | 1.333496 | 0.00110 |
| AoSMC | 1.314625 | 0.00124 |
| NHA | 1.344620 | 0.00128 |
| AG04450 | 1.383215 | 0.00132 |
| AoAF | 1.370586 | 0.00134 |
| BJ | 1.357876 | 0.00146 |
| Stellate | 1.420357 | 0.00170 |
| HAsp | 1.343588 | 0.00176 |
| HConF | 1.357739 | 0.00184 |
| NHLF | 1.326128 | 0.00184 |
| HPdLF | 1.343027 | 0.00258 |
| HAc | 1.297092 | 0.00292 |
| HBMEC | 1.291228 | 0.00310 |
| HMVECLLy | 1.359817 | 0.00326 |
| FibroP | 1.281709 | 0.00360 |
| SKMC | 1.298898 | 0.00380 |
| AG09309 | 1.291856 | 0.00380 |
| HSMMemb | 1.355379 | 0.00382 |
| HPF | 1.321609 | 0.00396 |
| HRPEpiC | 1.277506 | 0.00430 |
| HIPEpiC | 1.277490 | 0.00450 |
| PanIsletD | 1.302708 | 0.00460 |
| HRCEpiC | 1.279939 | 0.00474 |
| HMVECdLyAd | 1.351112 | 0.00492 |
| Myometr | 1.300236 | 0.00650 |
| HMVECdLyNeo | 1.321264 | 0.00736 |
| HMVECdBlAd | 1.307783 | 0.00748 |
| HMVECdAd | 1.333620 | 0.00832 |
| HRE | 1.251173 | 0.00852 |
| HMVECdBlNeo | 1.302346 | 0.00864 |
| HSMM | 1.238287 | 0.01100 |
| ProgFib | 1.308792 | 0.01218 |
| HRGEC | 1.271282 | 0.01380 |
| HMVECdNeo | 1.295650 | 0.01394 |
| HMVECLBl | 1.269921 | 0.01410 |
| RPTEC | 1.218629 | 0.01540 |
| HSMMtube | 1.235967 | 0.01914 |
| PrEC | 1.212837 | 0.01994 |
| Melano | 1.201252 | 0.02506 |
| NT2D1 | 1.213264 | 0.02528 |
| RWPE1 | 1.241473 | 0.02600 |
| HTR8svn | 1.260286 | 0.02642 |
| HUVEC | 1.250707 | 0.02814 |
| H7hESC | 1.157664 | 0.02948 |
| IshikawaEstradiol | 1.239736 | 0.03168 |
| SAEC | 1.177665 | 0.03818 |
| HPAEC | 1.220531 | 0.04134 |
| Gliobla | 1.260735 | 0.04612 |
| SKNSHRA | 1.296039 | 0.04642 |
| HEEpiC | 1.167018 | 0.04704 |
| NHEK | 1.206621 | 0.04804 |
| HPDE6E6E7 | 1.213446 | 0.05048 |
| IshikawaTamoxifen | 1.219609 | 0.05058 |
| WERIRb1 | 1.191988 | 0.05798 |
| HeLaS3IFNa4h | 1.244364 | 0.05924 |
| LNCaPAndrogen | 1.238328 | 0.06116 |
| Fibrobl | 1.205839 | 0.06550 |
| CD34Mobilized | 1.194001 | 0.07666 |
| HeLaS3 | 1.189691 | 0.08306 |
| PanIslets | 1.197758 | 0.08308 |
| Medullo | 1.203426 | 0.08314 |
| K562 | 1.214010 | 0.08710 |
| Hepatocytes | 1.233742 | 0.08936 |
| H9ES | 1.188123 | 0.09088 |
| Osteobl | 1.186838 | 0.09102 |
| CLL | 1.219372 | 0.09246 |
| pHTE | 1.174496 | 0.09984 |
| HepG2 | 1.184246 | 0.10242 |
| LNCaP | 1.156722 | 0.11358 |
| GM12891 | 1.202045 | 0.12044 |
| Huh7 | 1.175506 | 0.12144 |
| A549 | 1.160758 | 0.12584 |
| GM12892 | 1.194052 | 0.12770 |
| T47D | 1.182401 | 0.13144 |
| MCF7 | 1.150137 | 0.13952 |
| GM19240 | 1.148767 | 0.14100 |
| HMEC | 1.134361 | 0.14256 |
| NB4 | 1.150001 | 0.14542 |
| GM18507 | 1.152727 | 0.14644 |
| GM19238 | 1.162040 | 0.14802 |
| Huh7.5 | 1.159927 | 0.14890 |
| CD20 | 1.179174 | 0.14980 |
| HCT116 | 1.134921 | 0.15418 |
| GM19239 | 1.163060 | 0.15438 |
| GM06990 | 1.158666 | 0.16238 |
| H1hESC | 1.151903 | 0.16472 |
| MCF7Hypoxia | 1.155299 | 0.16986 |
| Th2 | 1.149394 | 0.17454 |
| Jurkat | 1.114948 | 0.18348 |
| Caco2 | 1.198170 | 0.18590 |
| iPS | 1.158113 | 0.18866 |
| Chorion | 1.154171 | 0.19020 |
| BE2C | 1.119324 | 0.19678 |
| Th1 | 1.107764 | 0.22098 |
| GM12878 | 1.109151 | 0.22682 |
| CMK | 1.114918 | 0.22948 |
| 8988T | 1.126958 | 0.22958 |
| PANC1 | 1.098149 | 0.23088 |
| Urothelia | 1.091969 | 0.24400 |
| Th0 | 1.098599 | 0.24730 |
| GM12865 | 1.090940 | 0.25090 |
| GM12864 | 1.083220 | 0.27710 |
| MonocytesCD14RO01746 | 1.082355 | 0.29456 |
| UrotheliaUT189 | 1.071233 | 0.29908 |
| SKNMC | 1.057041 | 0.31980 |
| HL60 | 1.069607 | 0.32122 |

# Systemic lupus erythematosus 1

| DHS sample | fold enrichment | p value |
|---|---|---|
| GM12865 | 1.6203194 | 0.00018 |
| Th2 | 1.7127766 | 0.00020 |
| GM12864 | 1.6331612 | 0.00024 |
| GM06990 | 1.6802766 | 0.00052 |
| CD20 | 1.6309603 | 0.00148 |
| GM12878 | 1.4232987 | 0.00962 |
| HCFaa | 1.2548734 | 0.02210 |
| GM18507 | 1.3443373 | 0.02328 |
| Jurkat | 1.2761168 | 0.02478 |
| MonocytesCD14RO01746 | 1.2777666 | 0.06290 |
| AG09309 | 1.1856862 | 0.06544 |
| AG09319 | 1.2135167 | 0.06874 |
| BJ | 1.1983253 | 0.07296 |
| AG04449 | 1.1857600 | 0.07760 |
| Th1 | 1.1967986 | 0.09798 |
| HAsp | 1.1614305 | 0.10526 |
| AG10803 | 1.1695786 | 0.10752 |
| GM19238 | 1.2087492 | 0.11476 |
| Th0 | 1.1863666 | 0.12074 |
| GM19240 | 1.1662513 | 0.14098 |
| NB4 | 1.1677398 | 0.14426 |
| HGF | 1.1340122 | 0.16740 |
| CD34Mobilized | 1.1422405 | 0.16840 |
| CLL | 1.1709666 | 0.17264 |
| HVMF | 1.1324527 | 0.17578 |
| GM19239 | 1.1653979 | 0.18026 |
| HL60 | 1.1613235 | 0.18370 |
| SKMC | 1.1083709 | 0.19028 |
| AoSMC | 1.1022274 | 0.19056 |
| K562 | 1.1441315 | 0.19614 |
| HPdLF | 1.1176495 | 0.19666 |
| NHDFAd | 1.1009099 | 0.20290 |
| NHLF | 1.1021079 | 0.20416 |
| WI38 | 1.1078275 | 0.21526 |
| HMF | 1.0975651 | 0.21676 |
| HPAF | 1.0942528 | 0.22896 |
| AoAF | 1.0962529 | 0.23198 |
| HCM | 1.0884528 | 0.23666 |
| HIPEpiC | 1.0830548 | 0.23800 |
| NHA | 1.0872813 | 0.23988 |
| HMVECLBl | 1.0941177 | 0.24358 |
| HNPCEpiC | 1.0774396 | 0.24850 |
| NHDFneo | 1.0899029 | 0.24916 |
| HBMEC | 1.0814747 | 0.25026 |
| HCF | 1.0900397 | 0.25150 |
| AG04450 | 1.0875411 | 0.26222 |
| PANC1 | 1.0847795 | 0.27988 |
| SAEC | 1.0628964 | 0.28424 |
| HRCEpiC | 1.0658482 | 0.28996 |
| HFF | 1.0662068 | 0.29844 |
| GM12892 | 1.0913537 | 0.30842 |
| HAEpiC | 1.0661921 | 0.30870 |
| HRE | 1.0556225 | 0.31242 |
| HCPEpiC | 1.0526040 | 0.33170 |
| RPTEC | 1.0427303 | 0.35062 |
| HEEpiC | 1.0433346 | 0.35118 |
| HMVECdBlAd | 1.0460998 | 0.36308 |
| GM12891 | 1.0513605 | 0.38368 |
| HPAEC | 1.0346945 | 0.39990 |
| HPF | 1.0291110 | 0.40660 |
| HConF | 1.0278552 | 0.40814 |
| HMVECdLyNeo | 1.0260044 | 0.42286 |
| HMVECdAd | 1.0156657 | 0.44826 |
| HFFMyc | 1.0079441 | 0.47316 |
| PrEC | 1.0069415 | 0.47360 |
| HMVECdLyAd | 1.0042316 | 0.47794 |
| NHEK | 1.0032174 | 0.48088 |
| CMK | 1.0002104 | 0.48474 |
| Stellate | 1.0003650 | 0.48792 |
| FibroP | 1.0013440 | 0.49560 |
| HMVECdBlNeo | 0.9979687 | 0.49754 |
| HSMM | 0.9936856 | 0.51572 |
| HAc | 0.9929699 | 0.51660 |
| HPDE6E6E7 | 0.9866238 | 0.52498 |
| PanIsletD | 0.9890909 | 0.52538 |
| Myometr | 0.9839821 | 0.53394 |
| HRGEC | 0.9829196 | 0.54152 |
| HAh | 0.9826240 | 0.55738 |
| HSMMtube | 0.9797601 | 0.55816 |
| HMVECLLy | 0.9597076 | 0.60016 |
| NT2D1 | 0.9625338 | 0.60510 |
| Melano | 0.9678643 | 0.61320 |
| PanIslets | 0.9464725 | 0.62900 |
| ProgFib | 0.9437182 | 0.63484 |
| HMVECdNeo | 0.9420059 | 0.64800 |
| Urothelia | 0.9383135 | 0.65036 |
| HSMMemb | 0.9399629 | 0.65156 |
| HUVEC | 0.9322919 | 0.67460 |
| Fibrobl | 0.9316891 | 0.68026 |
| H9ES | 0.9111193 | 0.70658 |
| H7hESC | 0.9352319 | 0.73394 |
| Gliobla | 0.8887392 | 0.74154 |
| T47D | 0.8771064 | 0.74710 |
| pHTE | 0.8988994 | 0.75582 |
| HMEC | 0.8992220 | 0.76036 |
| Chorion | 0.8590870 | 0.76134 |
| UrotheliaUT189 | 0.8863189 | 0.76564 |
| Osteobl | 0.8927441 | 0.76572 |
| HCT116 | 0.8862292 | 0.76578 |
| MCF7Hypoxia | 0.8664289 | 0.77902 |
| BE2C | 0.8766339 | 0.78624 |
| SKNMC | 0.8909460 | 0.78682 |
| HTR8svn | 0.8795994 | 0.78892 |
| Huh7 | 0.8616636 | 0.79300 |
| LNCaPAndrogen | 0.8612038 | 0.79558 |
| SKNSHRA | 0.8361564 | 0.79670 |
| 8988T | 0.8361788 | 0.80124 |
| Hepatocytes | 0.8424629 | 0.80126 |
| HRPEpiC | 0.9026312 | 0.80574 |
| WERIRb1 | 0.8770766 | 0.80878 |
| HepG2 | 0.8481025 | 0.82458 |
| LNCaP | 0.8675543 | 0.82466 |
| IshikawaEstradiol | 0.8575817 | 0.83078 |
| H1hESC | 0.8327561 | 0.83702 |
| A549 | 0.8423310 | 0.83998 |
| Caco2 | 0.7513312 | 0.84004 |
| IshikawaTamoxifen | 0.8447904 | 0.84790 |
| iPS | 0.7963119 | 0.85698 |
| Medullo | 0.8312863 | 0.85788 |
| RWPE1 | 0.8502605 | 0.85804 |
| Huh7.5 | 0.8143671 | 0.86488 |
| MCF7 | 0.8354384 | 0.87016 |
| HeLaS3IFNa4h | 0.7855116 | 0.90048 |
| HeLaS3 | 0.7632530 | 0.94200 |

# Metabolic traits 2

| DHS sample | fold enrichment | p value |
|---|---|---|
| RPTEC | 1.3400697 | 0.00024 |
| HRCEpiC | 1.3341318 | 0.00064 |
| HNPCEpiC | 1.2401679 | 0.00610 |
| HCFaa | 1.2633265 | 0.00646 |
| HL60 | 1.3751300 | 0.00776 |
| SKMC | 1.2566664 | 0.00784 |
| HRE | 1.2316170 | 0.01054 |
| HIPEpiC | 1.2236414 | 0.01140 |
| HCPEpiC | 1.2336436 | 0.01152 |
| SAEC | 1.2096166 | 0.01496 |
| HGF | 1.2633162 | 0.01660 |
| NHA | 1.2215483 | 0.01676 |
| HAh | 1.1968096 | 0.01892 |
| NHLF | 1.2112541 | 0.01910 |
| HCM | 1.2139678 | 0.01916 |
| HPdLF | 1.2443518 | 0.02050 |
| PrEC | 1.1933342 | 0.02302 |
| WI38 | 1.2291097 | 0.02684 |
| HAsp | 1.2072883 | 0.02694 |
| HBMEC | 1.1928367 | 0.02734 |
| HVMF | 1.2260915 | 0.02782 |
| HEEpiC | 1.1867249 | 0.02868 |
| HMF | 1.2048662 | 0.03052 |
| HPAF | 1.2022953 | 0.03178 |
| BJ | 1.2075948 | 0.03510 |
| Jurkat | 1.2103057 | 0.03562 |
| HAc | 1.1794834 | 0.03764 |
| AG04450 | 1.2116838 | 0.03794 |
| HMVECLBl | 1.1987089 | 0.03908 |
| MonocytesCD14RO01746 | 1.2708277 | 0.03940 |
| AG09319 | 1.2091933 | 0.03970 |
| AoSMC | 1.1706829 | 0.04204 |
| AG09309 | 1.1755004 | 0.04610 |
| HAEpiC | 1.1855150 | 0.04890 |
| IshikawaEstradiol | 1.2024676 | 0.04968 |
| AG04449 | 1.1795907 | 0.05298 |
| AG10803 | 1.1866496 | 0.05552 |
| HRGEC | 1.1781895 | 0.05560 |
| HCF | 1.1804375 | 0.05834 |
| HPF | 1.1791162 | 0.05950 |
| NHDFAd | 1.1642958 | 0.06038 |
| HSMM | 1.1485891 | 0.06580 |
| HRPEpiC | 1.1480507 | 0.06800 |
| AoAF | 1.1705572 | 0.06984 |
| HPAEC | 1.1735505 | 0.07044 |
| HSMMemb | 1.1757627 | 0.07246 |
| NB4 | 1.1944965 | 0.07288 |
| NHDFneo | 1.1672880 | 0.07398 |
| HFF | 1.1577568 | 0.07488 |
| HMVECdBlAd | 1.1604289 | 0.08022 |
| HMVECdAd | 1.1769364 | 0.08048 |
| HConF | 1.1612913 | 0.08072 |
| GM12865 | 1.1808021 | 0.08142 |
| FibroP | 1.1343521 | 0.08252 |
| PanIsletD | 1.1467088 | 0.09124 |
| Myometr | 1.1587451 | 0.09150 |
| NHEK | 1.1485484 | 0.10480 |
| HFFMyc | 1.1322098 | 0.10492 |
| HMVECdBlNeo | 1.1444745 | 0.10608 |
| HMVECdLyNeo | 1.1487480 | 0.10850 |
| H7hESC | 1.0990669 | 0.11868 |
| HCT116 | 1.1558881 | 0.12246 |
| UrotheliaUT189 | 1.1492792 | 0.12426 |
| IshikawaTamoxifen | 1.1423637 | 0.12878 |
| PANC1 | 1.1410821 | 0.13424 |
| HMVECLLy | 1.1313228 | 0.13774 |
| HSMMtube | 1.1129229 | 0.14420 |
| Urothelia | 1.1329462 | 0.14636 |
| GM12864 | 1.1394089 | 0.14714 |
| Stellate | 1.1382226 | 0.14784 |
| HPDE6E6E7 | 1.1279530 | 0.15122 |
| HMVECdNeo | 1.1223762 | 0.15654 |
| HeLaS3 | 1.1326053 | 0.16118 |
| Th2 | 1.1465227 | 0.16158 |
| HeLaS3IFNa4h | 1.1453697 | 0.16158 |
| GM12878 | 1.1356547 | 0.16200 |
| GM06990 | 1.1492715 | 0.16246 |
| RWPE1 | 1.1178469 | 0.16428 |
| A549 | 1.1335536 | 0.16462 |
| GM19240 | 1.1179698 | 0.17460 |
| HMVECdLyAd | 1.1127864 | 0.17572 |
| K562 | 1.1280354 | 0.18030 |
| Melano | 1.0845133 | 0.18678 |
| CD34Mobilized | 1.1112009 | 0.18780 |
| GM18507 | 1.1194578 | 0.18784 |
| HepG2 | 1.1092449 | 0.20454 |
| GM19239 | 1.1065809 | 0.22976 |
| BE2C | 1.0909436 | 0.23188 |
| HTR8svn | 1.0887605 | 0.23580 |
| PanIslets | 1.0916128 | 0.24328 |
| GM19238 | 1.0862025 | 0.26672 |
| CLL | 1.0889598 | 0.26742 |
| MCF7 | 1.0764199 | 0.26758 |
| Gliobla | 1.0842625 | 0.26796 |
| CMK | 1.0880627 | 0.27610 |
| WERIRb1 | 1.0608729 | 0.28514 |
| Huh7.5 | 1.0773547 | 0.28710 |
| NT2D1 | 1.0545623 | 0.28744 |
| Th0 | 1.0708226 | 0.29158 |
| CD20 | 1.0825598 | 0.29628 |
| GM12891 | 1.0786149 | 0.29938 |
| Huh7 | 1.0701349 | 0.30208 |
| HUVEC | 1.0610023 | 0.30576 |
| pHTE | 1.0564574 | 0.32056 |
| Medullo | 1.0589290 | 0.32152 |
| Fibrobl | 1.0542439 | 0.32198 |
| Caco2 | 1.0769847 | 0.33490 |
| ProgFib | 1.0503730 | 0.34050 |
| GM12892 | 1.0600089 | 0.34122 |
| HMEC | 1.0470573 | 0.34166 |
| Th1 | 1.0513258 | 0.34430 |
| SKNMC | 1.0398378 | 0.35868 |
| Osteobl | 1.0372302 | 0.37976 |
| Hepatocytes | 1.0414543 | 0.38574 |
| SKNSHRA | 1.0330778 | 0.40474 |
| H9ES | 1.0238860 | 0.41794 |
| MCF7Hypoxia | 1.0126011 | 0.45578 |
| Chorion | 1.0030234 | 0.48086 |
| LNCaPAndrogen | 1.0006553 | 0.48688 |
| H1hESC | 0.9977808 | 0.49434 |
| LNCaP | 0.9826251 | 0.55044 |
| 8988T | 0.9704051 | 0.56058 |
| iPS | 0.9719660 | 0.56392 |
| T47D | 0.9694411 | 0.57218 |

# Bone mineral density 2

| DHS sample | fold enrichment | p value |
|---|---|---|
| HAsp | 1.3106595 | 0.00026 |
| SKMC | 1.2531213 | 0.00120 |
| AoSMC | 1.2368408 | 0.00140 |
| AoAF | 1.2620345 | 0.00202 |
| HPdLF | 1.2700559 | 0.00214 |
| HAEpiC | 1.2522850 | 0.00214 |
| HBMEC | 1.2309015 | 0.00240 |
| AG09309 | 1.2354165 | 0.00266 |
| NHLF | 1.2230984 | 0.00334 |
| AG04449 | 1.2348210 | 0.00354 |
| HAh | 1.1970036 | 0.00360 |
| HAc | 1.2055583 | 0.00370 |
| NHA | 1.2232995 | 0.00378 |
| NHDFAd | 1.2198836 | 0.00380 |
| HSMM | 1.2007933 | 0.00420 |
| BJ | 1.2329651 | 0.00444 |
| HVMF | 1.2621556 | 0.00474 |
| AG10803 | 1.2348682 | 0.00496 |
| HCFaa | 1.2186993 | 0.00556 |
| HMF | 1.2114038 | 0.00648 |
| HRE | 1.1942173 | 0.00712 |
| RPTEC | 1.1807291 | 0.00816 |
| HIPEpiC | 1.1974440 | 0.00904 |
| PrEC | 1.1727682 | 0.01108 |
| HGF | 1.2162524 | 0.01158 |
| HCPEpiC | 1.1903682 | 0.01182 |
| AG09319 | 1.2112335 | 0.01274 |
| HRCEpiC | 1.1785256 | 0.01334 |
| HPF | 1.2015849 | 0.01342 |
| HEEpiC | 1.1652656 | 0.01406 |
| HNPCEpiC | 1.1664220 | 0.01540 |
| HFF | 1.1811832 | 0.01620 |
| NHDFneo | 1.1881265 | 0.01656 |
| AG04450 | 1.1982579 | 0.01730 |
| HPAF | 1.1777873 | 0.01872 |
| HFFMyc | 1.1713453 | 0.01908 |
| HMVECLBl | 1.1929791 | 0.01918 |
| WI38 | 1.1942382 | 0.02214 |
| SAEC | 1.1475115 | 0.02294 |
| HCM | 1.1628939 | 0.02376 |
| HUVEC | 1.1908750 | 0.02442 |
| HCF | 1.1705249 | 0.02848 |
| HTR8svn | 1.1842190 | 0.03094 |
| FibroP | 1.1378622 | 0.03312 |
| HMVECdBlAd | 1.1713440 | 0.03350 |
| HConF | 1.1625450 | 0.03392 |
| Myometr | 1.1620649 | 0.03492 |
| PanIsletD | 1.1564534 | 0.03530 |
| HMVECdLyNeo | 1.1716650 | 0.03664 |
| Huh7 | 1.1998216 | 0.03838 |
| PANC1 | 1.1810089 | 0.04086 |
| HeLaS3IFNa4h | 1.2018124 | 0.04126 |
| HRGEC | 1.1533160 | 0.04754 |
| A549 | 1.1790613 | 0.04880 |
| HMVECdBlNeo | 1.1491116 | 0.05436 |
| HRPEpiC | 1.1115833 | 0.06276 |
| IshikawaTamoxifen | 1.1520219 | 0.06326 |
| ProgFib | 1.1477084 | 0.06612 |
| Huh7.5 | 1.1700953 | 0.06922 |
| HMVECdLyAd | 1.1466195 | 0.06936 |
| HPAEC | 1.1377237 | 0.06980 |
| HeLaS3 | 1.1481605 | 0.07602 |
| HPDE6E6E7 | 1.1415610 | 0.07636 |
| HMVECLLy | 1.1359436 | 0.07936 |
| IshikawaEstradiol | 1.1346522 | 0.07962 |
| Melano | 1.0996577 | 0.08032 |
| HSMMtube | 1.1104104 | 0.08340 |
| HMVECdAd | 1.1378156 | 0.08430 |
| HSMMemb | 1.1338537 | 0.08498 |
| HepG2 | 1.1417433 | 0.09338 |
| HCT116 | 1.1279149 | 0.10030 |
| RWPE1 | 1.1130698 | 0.11232 |
| Gliobla | 1.1208513 | 0.13624 |
| HMVECdNeo | 1.1054593 | 0.13700 |
| Stellate | 1.1059970 | 0.15356 |
| NHEK | 1.0921491 | 0.15392 |
| T47D | 1.1074027 | 0.17324 |
| SKNMC | 1.0816955 | 0.17606 |
| H7hESC | 1.0508409 | 0.19110 |
| MCF7 | 1.0815168 | 0.19356 |
| H9ES | 1.0826721 | 0.19908 |
| SKNSHRA | 1.0908675 | 0.22028 |
| NT2D1 | 1.0561838 | 0.22108 |
| LNCaPAndrogen | 1.0802971 | 0.22806 |
| Hepatocytes | 1.0868070 | 0.23090 |
| LNCaP | 1.0647089 | 0.23740 |
| GM12878 | 1.0746902 | 0.25022 |
| HMEC | 1.0530908 | 0.27686 |
| GM19238 | 1.0607842 | 0.28904 |
| MCF7Hypoxia | 1.0597790 | 0.29004 |
| GM18507 | 1.0589180 | 0.29050 |
| H1hESC | 1.0566301 | 0.29336 |
| K562 | 1.0577515 | 0.30162 |
| Urothelia | 1.0497663 | 0.30180 |
| pHTE | 1.0463920 | 0.31020 |
| GM19239 | 1.0562210 | 0.31276 |
| Th2 | 1.0569187 | 0.31546 |
| CD34Mobilized | 1.0413469 | 0.33852 |
| GM12864 | 1.0356152 | 0.36538 |
| GM12865 | 1.0333256 | 0.36682 |
| Chorion | 1.0391851 | 0.37072 |
| GM19240 | 1.0295521 | 0.38024 |
| UrotheliaUT189 | 1.0264096 | 0.38994 |
| 8988T | 1.0305386 | 0.39674 |
| Caco2 | 1.0244611 | 0.42514 |
| NB4 | 1.0180500 | 0.42630 |
| PanIslets | 1.0134288 | 0.44040 |
| GM06990 | 1.0103003 | 0.45776 |
| Th0 | 1.0080147 | 0.46700 |
| CD20 | 1.0077118 | 0.46766 |
| HL60 | 1.0065803 | 0.47118 |
| Fibrobl | 0.9976166 | 0.50678 |
| GM12891 | 0.9928047 | 0.51452 |
| iPS | 0.9903077 | 0.52416 |
| WERIRb1 | 0.9906849 | 0.53990 |
| Osteobl | 0.9881491 | 0.54568 |
| CMK | 0.9775351 | 0.56708 |
| Medullo | 0.9789771 | 0.57968 |
| CLL | 0.9708574 | 0.58722 |
| Th1 | 0.9755884 | 0.59218 |
| GM12892 | 0.9674230 | 0.60102 |
| MonocytesCD14RO01746 | 0.9650357 | 0.61044 |
| BE2C | 0.9658608 | 0.63114 |
| Jurkat | 0.9319414 | 0.76938 |

## Self−reported allergy

| DHS sample | fold enrichment | p value |
|---|---|---|
| Th2 | 1.9487597 | 0.00026 |
| HRGEC | 1.5591457 | 0.00116 |
| HCFaa | 1.4445701 | 0.00312 |
| SAEC | 1.3309233 | 0.01242 |
| HMVECLBl | 1.4226170 | 0.01264 |
| HAsp | 1.3630225 | 0.01326 |
| HMVECdBlAd | 1.4111173 | 0.01436 |
| HIPEpiC | 1.3453885 | 0.01500 |
| HNPCEpiC | 1.2963655 | 0.02332 |
| HCPEpiC | 1.3158927 | 0.02470 |
| HBMEC | 1.3001106 | 0.02476 |
| CD20 | 1.5456081 | 0.02508 |
| GM12865 | 1.4141612 | 0.02648 |
| SKMC | 1.3080036 | 0.02672 |
| PANC1 | 1.3871496 | 0.03290 |
| HPAEC | 1.3500097 | 0.03358 |
| WI38 | 1.3373801 | 0.03688 |
| GM12864 | 1.4044138 | 0.03716 |
| HAEpiC | 1.3122845 | 0.03774 |
| MonocytesCD14RO01746 | 1.4316957 | 0.03882 |
| HMF | 1.2975474 | 0.03966 |
| HPAF | 1.2973112 | 0.04132 |
| PrEC | 1.2488194 | 0.04416 |
| AG04450 | 1.3176005 | 0.04500 |
| HMVECdBlNeo | 1.3187217 | 0.04550 |
| HEEpiC | 1.2353578 | 0.05576 |
| HRE | 1.2335575 | 0.05594 |
| HCM | 1.2579519 | 0.05638 |
| HPdLF | 1.2737724 | 0.05920 |
| AoAF | 1.2697113 | 0.06412 |
| GM06990 | 1.3876098 | 0.06506 |
| HPF | 1.2680478 | 0.06986 |
| HRCEpiC | 1.2267995 | 0.07060 |
| AG04449 | 1.2419959 | 0.07446 |
| HCF | 1.2579936 | 0.07504 |
| HMVECdLyNeo | 1.2629960 | 0.08494 |
| AG09309 | 1.2160177 | 0.08784 |
| HGF | 1.2409218 | 0.09608 |
| HMVECdAd | 1.2525035 | 0.10148 |
| RPTEC | 1.1830624 | 0.10328 |
| HUVEC | 1.2407195 | 0.10768 |
| HVMF | 1.2261264 | 0.11044 |
| HAc | 1.1746290 | 0.11610 |
| HConF | 1.2008775 | 0.12758 |
| HRPEpiC | 1.1526112 | 0.13810 |
| HMVECLLy | 1.2027477 | 0.13866 |
| GM12878 | 1.2440769 | 0.13880 |
| AoSMC | 1.1665116 | 0.14242 |
| AG09319 | 1.1901397 | 0.14536 |
| Th1 | 1.2150263 | 0.14546 |
| HMVECdNeo | 1.1984443 | 0.14792 |
| BJ | 1.1721106 | 0.15128 |
| NHDFAd | 1.1580031 | 0.16112 |
| NHLF | 1.1517540 | 0.16588 |
| NHA | 1.1526524 | 0.16858 |
| NHDFneo | 1.1606114 | 0.16924 |
| HPDE6E6E7 | 1.1868752 | 0.17102 |
| HMVECdLyAd | 1.1778277 | 0.17104 |
| CD34Mobilized | 1.1677270 | 0.19776 |
| HTR8svn | 1.1667295 | 0.20194 |
| RWPE1 | 1.1496922 | 0.20244 |
| GM18507 | 1.1822193 | 0.20382 |
| AG10803 | 1.1427855 | 0.20502 |
| HFFMyc | 1.1331552 | 0.20782 |
| Jurkat | 1.1444499 | 0.20898 |
| HFF | 1.1298421 | 0.21458 |
| NB4 | 1.1467723 | 0.23354 |
| Stellate | 1.1299721 | 0.25714 |
| NHEK | 1.1088439 | 0.26502 |
| Th0 | 1.1296576 | 0.26544 |
| HeLaS3 | 1.1200953 | 0.26568 |
| MCF7 | 1.1090512 | 0.26614 |
| HepG2 | 1.1171954 | 0.28080 |
| A549 | 1.1144901 | 0.28402 |
| HL60 | 1.1229005 | 0.28614 |
| HeLaS3IFNa4h | 1.1042964 | 0.30794 |
| HAh | 1.0669540 | 0.31422 |
| HCT116 | 1.0882850 | 0.31944 |
| CLL | 1.1046743 | 0.32270 |
| Urothelia | 1.0632084 | 0.36232 |
| GM19238 | 1.0714308 | 0.36528 |
| Myometr | 1.0506450 | 0.37536 |
| K562 | 1.0606904 | 0.37896 |
| HSMMemb | 1.0497622 | 0.39016 |
| T47D | 1.0434285 | 0.40612 |
| PanIsletD | 1.0214479 | 0.44010 |
| GM19240 | 1.0181563 | 0.45134 |
| Huh7 | 1.0153278 | 0.45344 |
| HSMM | 1.0111566 | 0.46852 |
| ProgFib | 1.0082287 | 0.47042 |
| SKNMC | 1.0064066 | 0.47110 |
| MCF7Hypoxia | 0.9955421 | 0.48244 |
| IshikawaEstradiol | 1.0004685 | 0.48286 |
| FibroP | 0.9975633 | 0.50310 |
| GM19239 | 0.9833160 | 0.50736 |
| UrotheliaUT189 | 0.9838886 | 0.52296 |
| CMK | 0.9709276 | 0.52822 |
| IshikawaTamoxifen | 0.9765676 | 0.53256 |
| HSMMtube | 0.9576441 | 0.60084 |
| Caco2 | 0.8964732 | 0.60758 |
| Gliobla | 0.9189032 | 0.63150 |
| GM12891 | 0.8892655 | 0.65694 |
| Melano | 0.9328512 | 0.68258 |
| pHTE | 0.9040270 | 0.68900 |
| Huh7.5 | 0.8756272 | 0.69776 |
| HMEC | 0.9043305 | 0.70008 |
| LNCaP | 0.8875343 | 0.72816 |
| GM12892 | 0.8388045 | 0.73506 |
| LNCaPAndrogen | 0.8561454 | 0.73804 |
| Fibrobl | 0.8778056 | 0.74150 |
| BE2C | 0.8490474 | 0.79480 |
| Osteobl | 0.8188647 | 0.82798 |
| PanIslets | 0.8085830 | 0.83148 |
| 8988T | 0.7572470 | 0.83554 |
| H9ES | 0.8073209 | 0.83970 |
| NT2D1 | 0.8513586 | 0.84452 |
| Chorion | 0.7469838 | 0.84986 |
| Hepatocytes | 0.7546456 | 0.85432 |
| H1hESC | 0.7495999 | 0.88864 |
| WERIRb1 | 0.7955917 | 0.90704 |
| iPS | 0.6795365 | 0.91044 |
| H7hESC | 0.8522000 | 0.91340 |
| Medullo | 0.6604974 | 0.95428 |
| SKNSHRA | 0.6404191 | 0.95528 |

## Blood pressure and cardiovascular risc

| DHS sample | fold enrichment | p value |
|---|---|---|
| HCFaa | 1.4720443 | 0.00032 |
| HAsp | 1.4485361 | 0.00040 |
| HRGEC | 1.4822736 | 0.00058 |
| HPdLF | 1.4656946 | 0.00066 |
| HGF | 1.4481774 | 0.00168 |
| HBMEC | 1.3647728 | 0.00200 |
| HNPCEpiC | 1.3526771 | 0.00220 |
| AoAF | 1.4135125 | 0.00234 |
| RPTEC | 1.3406314 | 0.00262 |
| AoSMC | 1.3413880 | 0.00340 |
| PANC1 | 1.4693309 | 0.00440 |
| AG09309 | 1.3408992 | 0.00458 |
| AG09319 | 1.3963485 | 0.00460 |
| HIPEpiC | 1.3344027 | 0.00484 |
| AG04450 | 1.3881905 | 0.00538 |
| NHDFAd | 1.3273083 | 0.00616 |
| SKMC | 1.3308194 | 0.00718 |
| SAEC | 1.2918908 | 0.00724 |
| HAc | 1.3016667 | 0.00826 |
| BJ | 1.3269783 | 0.01088 |
| HEEpiC | 1.2751286 | 0.01160 |
| HPAF | 1.3067283 | 0.01208 |
| NHDFneo | 1.3214570 | 0.01240 |
| HMVECdLyAd | 1.3553362 | 0.01290 |
| HCPEpiC | 1.2827506 | 0.01370 |
| HPF | 1.3143774 | 0.01544 |
| HPAEC | 1.3261224 | 0.01594 |
| AG04449 | 1.2996369 | 0.01602 |
| NHLF | 1.2755240 | 0.01652 |
| HMVECLBl | 1.3124965 | 0.01754 |
| HMVECdNeo | 1.3238835 | 0.01838 |
| HMVECdBlAd | 1.3087759 | 0.01900 |
| PrEC | 1.2468993 | 0.02136 |
| HMVECdAd | 1.3287040 | 0.02182 |
| HCM | 1.2678736 | 0.02214 |
| HRE | 1.2498124 | 0.02358 |
| HRCEpiC | 1.2503642 | 0.02366 |
| NHA | 1.2613499 | 0.02420 |
| HMF | 1.2734040 | 0.02432 |
| HFF | 1.2651262 | 0.02566 |
| HMVECdLyNeo | 1.2967226 | 0.02572 |
| HRPEpiC | 1.2170420 | 0.02796 |
| HConF | 1.2729625 | 0.02838 |
| HCF | 1.2755067 | 0.02860 |
| HeLaS3 | 1.3143308 | 0.03022 |
| AG10803 | 1.2749667 | 0.03110 |
| HFFMyc | 1.2447505 | 0.03402 |
| HMVECdBlNeo | 1.2748043 | 0.03486 |
| SKNMC | 1.2577562 | 0.03528 |
| HMVECLLy | 1.2789220 | 0.03578 |
| WI38 | 1.2561731 | 0.04240 |
| RWPE1 | 1.2544269 | 0.04644 |
| CD20 | 1.3576481 | 0.04948 |
| GM06990 | 1.3340145 | 0.04982 |
| HAh | 1.1851802 | 0.05396 |
| HTR8svn | 1.2608401 | 0.05512 |
| Th2 | 1.3121638 | 0.05526 |
| HAEpiC | 1.2124387 | 0.06544 |
| HUVEC | 1.2299309 | 0.06638 |
| HPDE6E6E7 | 1.2255244 | 0.07680 |
| GM12864 | 1.2484357 | 0.07786 |
| GM12865 | 1.2363522 | 0.07852 |
| CMK | 1.2668220 | 0.08626 |
| HVMF | 1.2059771 | 0.08758 |
| CD34Mobilized | 1.2143235 | 0.09244 |
| HCT116 | 1.2134991 | 0.09526 |
| A549 | 1.2102302 | 0.11106 |
| NB4 | 1.2094391 | 0.11234 |
| IshikawaTamoxifen | 1.1811987 | 0.12404 |
| H7hESC | 1.1112557 | 0.12706 |
| IshikawaEstradiol | 1.1703611 | 0.13074 |
| K562 | 1.2016611 | 0.13090 |
| HeLaS3IFNa4h | 1.1986490 | 0.13430 |
| Myometr | 1.1530872 | 0.13772 |
| BE2C | 1.1667606 | 0.15492 |
| Stellate | 1.1659875 | 0.15516 |
| Jurkat | 1.1410585 | 0.17278 |
| HSMMemb | 1.1390479 | 0.18126 |
| GM12878 | 1.1563963 | 0.18736 |
| CLL | 1.1724022 | 0.18742 |
| HL60 | 1.1606798 | 0.19516 |
| NHEK | 1.1215719 | 0.19594 |
| MonocytesCD14RO01746 | 1.1548825 | 0.20492 |
| WERIRb1 | 1.0997807 | 0.22844 |
| GM18507 | 1.1219727 | 0.24058 |
| Huh7 | 1.1184242 | 0.24766 |
| HSMM | 1.0825878 | 0.24842 |
| T47D | 1.1018429 | 0.28770 |
| ProgFib | 1.0574709 | 0.34556 |
| NT2D1 | 1.0441297 | 0.35104 |
| Gliobla | 1.0583689 | 0.35762 |
| FibroP | 1.0410064 | 0.36552 |
| MCF7 | 1.0442685 | 0.37322 |
| PanIsletD | 1.0399733 | 0.37894 |
| Urothelia | 1.0435298 | 0.38224 |
| Huh7.5 | 1.0356251 | 0.40892 |
| UrotheliaUT189 | 1.0166296 | 0.44748 |
| GM19239 | 1.0017768 | 0.48114 |
| GM19238 | 1.0036293 | 0.48208 |
| H9ES | 0.9912818 | 0.50954 |
| Melano | 0.9966309 | 0.51068 |
| HepG2 | 0.9914007 | 0.51088 |
| MCF7Hypoxia | 0.9853226 | 0.51786 |
| SKNSHRA | 0.9538710 | 0.56742 |
| LNCaPAndrogen | 0.9476267 | 0.60526 |
| GM19240 | 0.9481633 | 0.61798 |
| LNCaP | 0.9467661 | 0.62490 |
| GM12891 | 0.9243008 | 0.63632 |
| Th0 | 0.9382248 | 0.63642 |
| Th1 | 0.9301866 | 0.65824 |
| Medullo | 0.9247024 | 0.66056 |
| HMEC | 0.9380230 | 0.66068 |
| HSMMtube | 0.9426669 | 0.66808 |
| H1hESC | 0.9123687 | 0.67772 |
| pHTE | 0.9257330 | 0.68006 |
| GM12892 | 0.8980566 | 0.68756 |
| Caco2 | 0.8180338 | 0.73484 |
| Osteobl | 0.8975935 | 0.73934 |
| 8988T | 0.8544266 | 0.75104 |
| Hepatocytes | 0.8624409 | 0.75216 |
| Fibrobl | 0.8934120 | 0.75650 |
| PanIslets | 0.8613870 | 0.79576 |
| Chorion | 0.7669942 | 0.87524 |
| iPS | 0.7651877 | 0.87832 |

# Rheumatoid arthritis

| DHS sample | fold enrichment | p value |
|---|---|---|
| Th2 | 1.8341128 | 0.00034 |
| GM06990 | 1.7074243 | 0.00190 |
| GM12864 | 1.5845477 | 0.00252 |
| GM12865 | 1.5073695 | 0.00616 |
| GM18507 | 1.5181181 | 0.00672 |
| GM12878 | 1.5320427 | 0.00680 |
| AoSMC | 1.3208528 | 0.00978 |
| CD20 | 1.5495428 | 0.01376 |
| MonocytesCD14RO01746 | 1.4787193 | 0.01732 |
| Jurkat | 1.3582916 | 0.02054 |
| Th1 | 1.3787713 | 0.02486 |
| NB4 | 1.3793077 | 0.02770 |
| Th0 | 1.3728523 | 0.03164 |
| AG04449 | 1.2843903 | 0.03204 |
| GM19240 | 1.3419286 | 0.03494 |
| HIPEpiC | 1.2516568 | 0.03648 |
| AoAF | 1.2820895 | 0.03696 |
| HCM | 1.2493743 | 0.04182 |
| HCFaa | 1.2476263 | 0.04500 |
| GM19238 | 1.3594356 | 0.04574 |
| HPAF | 1.2479620 | 0.04592 |
| HBMEC | 1.2376184 | 0.04622 |
| HAEpiC | 1.2594991 | 0.04720 |
| HMF | 1.2459429 | 0.05016 |
| HAsp | 1.2415917 | 0.05044 |
| HRGEC | 1.2626826 | 0.05128 |
| RPTEC | 1.2181244 | 0.05168 |
| NHDFneo | 1.2542964 | 0.05288 |
| HCF | 1.2554354 | 0.05424 |
| HFFMyc | 1.2316128 | 0.05716 |
| HCPEpiC | 1.2237291 | 0.05854 |
| GM19239 | 1.3397640 | 0.06410 |
| AG09309 | 1.2183371 | 0.06480 |
| SKMC | 1.2185868 | 0.06482 |
| HL60 | 1.3323447 | 0.06728 |
| CLL | 1.3384331 | 0.06920 |
| K562 | 1.3187278 | 0.07186 |
| HGF | 1.2330029 | 0.07472 |
| HepG2 | 1.2931745 | 0.07524 |
| NHDFAd | 1.2075317 | 0.07642 |
| HFF | 1.2116041 | 0.07664 |
| HPdLF | 1.2268642 | 0.07672 |
| Huh7 | 1.2995661 | 0.07690 |
| HConF | 1.2230479 | 0.07966 |
| AG09319 | 1.2212135 | 0.08464 |
| AG04450 | 1.2224250 | 0.09138 |
| HAh | 1.1676866 | 0.09610 |
| AG10803 | 1.2037176 | 0.09704 |
| HPAEC | 1.2164970 | 0.09862 |
| HRCEpiC | 1.1859472 | 0.09864 |
| BJ | 1.1981988 | 0.09878 |
| FibroP | 1.1743229 | 0.10198 |
| HRE | 1.1748026 | 0.10268 |
| WI38 | 1.2052603 | 0.10364 |
| Melano | 1.1640228 | 0.10536 |
| HMVECdBlAd | 1.1981326 | 0.11074 |
| HNPCEpiC | 1.1649683 | 0.11128 |
| NHA | 1.1748689 | 0.11634 |
| HVMF | 1.2016302 | 0.11790 |
| NHLF | 1.1654479 | 0.12398 |
| PanIsletD | 1.1759954 | 0.13056 |
| CD34Mobilized | 1.2020636 | 0.13276 |
| HMVECLBl | 1.1777635 | 0.13362 |
| HMVECdLyNeo | 1.1871497 | 0.13444 |
| MCF7 | 1.1940812 | 0.13650 |
| Stellate | 1.2040916 | 0.13930 |
| HMVECdAd | 1.1944271 | 0.14022 |
| HMVECdLyAd | 1.1814575 | 0.15020 |
| GM12892 | 1.2345818 | 0.15036 |
| SAEC | 1.1342790 | 0.15672 |
| HAc | 1.1365627 | 0.16008 |
| HPF | 1.1535738 | 0.16312 |
| HTR8svn | 1.1733581 | 0.16658 |
| HMVECdBlNeo | 1.1509822 | 0.17696 |
| HSMMemb | 1.1599485 | 0.18242 |
| HUVEC | 1.1532531 | 0.18288 |
| PANC1 | 1.1540692 | 0.18352 |
| Urothelia | 1.1609133 | 0.18446 |
| Myometr | 1.1392970 | 0.18524 |
| HMVECLLy | 1.1511109 | 0.18872 |
| ProgFib | 1.1544274 | 0.19222 |
| GM12891 | 1.1932217 | 0.19394 |
| HMVECdNeo | 1.1431106 | 0.20230 |
| Huh7.5 | 1.1675726 | 0.20522 |
| H7hESC | 1.0873126 | 0.21064 |
| MCF7Hypoxia | 1.1574138 | 0.22060 |
| HSMM | 1.1034619 | 0.22932 |
| HEEpiC | 1.0983427 | 0.22950 |
| NT2D1 | 1.0995878 | 0.23042 |
| H9ES | 1.1297710 | 0.23284 |
| Medullo | 1.1344978 | 0.24002 |
| PanIslets | 1.1250983 | 0.25318 |
| SKNMC | 1.0919030 | 0.26840 |
| Fibrobl | 1.0963980 | 0.28846 |
| HSMMtube | 1.0715980 | 0.31174 |
| UrotheliaUT189 | 1.0834310 | 0.31736 |
| BE2C | 1.0728895 | 0.33156 |
| WERIRb1 | 1.0667111 | 0.33188 |
| LNCaPAndrogen | 1.0817398 | 0.33472 |
| Osteobl | 1.0737516 | 0.33710 |
| HeLaS3IFNa4h | 1.0779529 | 0.34060 |
| NHEK | 1.0585763 | 0.35388 |
| Hepatocytes | 1.0732789 | 0.35404 |
| pHTE | 1.0603689 | 0.35716 |
| IshikawaEstradiol | 1.0508194 | 0.37308 |
| RWPE1 | 1.0481095 | 0.37816 |
| Gliobla | 1.0546600 | 0.37884 |
| SKNSHRA | 1.0509056 | 0.38770 |
| HPDE6E6E7 | 1.0447146 | 0.38976 |
| CMK | 1.0467397 | 0.39686 |
| PrEC | 1.0301540 | 0.40520 |
| Caco2 | 1.0440628 | 0.41416 |
| HCT116 | 1.0275629 | 0.42478 |
| iPS | 1.0248117 | 0.44224 |
| IshikawaTamoxifen | 1.0204544 | 0.44268 |
| LNCaP | 1.0155238 | 0.44894 |
| HMEC | 1.0130469 | 0.45832 |
| H1hESC | 1.0020866 | 0.47490 |
| T47D | 1.0001413 | 0.47990 |
| 8988T | 0.9971968 | 0.48278 |
| HRPEpiC | 0.9966471 | 0.50538 |
| A549 | 0.9869517 | 0.51364 |
| Chorion | 0.9610141 | 0.54936 |
| HeLaS3 | 0.9361174 | 0.62312 |

# Type 2 diabetes

| DHS sample | fold enrichment | p value |
|---|---|---|
| HCT116 | 1.592576 | 0.00040 |
| CD34Mobilized | 1.396690 | 0.00854 |
| HAEpiC | 1.334224 | 0.01150 |
| HIPEpiC | 1.310822 | 0.01154 |
| HCPEpiC | 1.289020 | 0.01504 |
| HEEpiC | 1.251479 | 0.01594 |
| HAsp | 1.276745 | 0.01648 |
| HFFMyc | 1.285153 | 0.01656 |
| MonocytesCD14RO01746 | 1.442063 | 0.01676 |
| NHDFneo | 1.314494 | 0.01748 |
| HFF | 1.288325 | 0.01928 |
| HNPCEpiC | 1.253446 | 0.02066 |
| HVMF | 1.308729 | 0.02676 |
| SAEC | 1.225568 | 0.02842 |
| HConF | 1.276134 | 0.02996 |
| NB4 | 1.330343 | 0.03280 |
| AG04450 | 1.278579 | 0.03296 |
| HPdLF | 1.277874 | 0.03512 |
| PANC1 | 1.296638 | 0.03588 |
| SKMC | 1.238022 | 0.03724 |
| HRPEpiC | 1.204611 | 0.03734 |
| A549 | 1.303457 | 0.03746 |
| CD20 | 1.377774 | 0.04064 |
| HeLaS3IFNa4h | 1.318211 | 0.04140 |
| HeLaS3 | 1.286315 | 0.04220 |
| GM12865 | 1.301507 | 0.04274 |
| WI38 | 1.258271 | 0.04386 |
| HCFaa | 1.232157 | 0.04452 |
| HCM | 1.223641 | 0.04870 |
| NHEK | 1.230064 | 0.05096 |
| GM12864 | 1.304607 | 0.05192 |
| HBMEC | 1.200073 | 0.05674 |
| AG04449 | 1.223968 | 0.05728 |
| Myometr | 1.214904 | 0.05984 |
| HAh | 1.173701 | 0.05998 |
| BE2C | 1.246199 | 0.06008 |
| AG09309 | 1.204222 | 0.06156 |
| AG09319 | 1.228763 | 0.06422 |
| HPDE6E6E7 | 1.240855 | 0.06472 |
| Huh7 | 1.267675 | 0.06488 |
| 8988T | 1.320403 | 0.06590 |
| HSMM | 1.179521 | 0.06598 |
| NHLF | 1.193625 | 0.06618 |
| HAc | 1.184616 | 0.06654 |
| HPF | 1.213391 | 0.06738 |
| PanIsletD | 1.198118 | 0.06866 |
| HPAF | 1.202684 | 0.06938 |
| GM06990 | 1.309632 | 0.06952 |
| HRGEC | 1.209279 | 0.07794 |
| HGF | 1.213685 | 0.07916 |
| Gliobla | 1.242673 | 0.08110 |
| HSMMemb | 1.208347 | 0.08300 |
| Huh7.5 | 1.250502 | 0.08306 |
| AG10803 | 1.200995 | 0.08330 |
| PrEC | 1.164631 | 0.08576 |
| IshikawaEstradiol | 1.202972 | 0.08966 |
| HMVECLBl | 1.202029 | 0.09208 |
| HRCEpiC | 1.168050 | 0.09438 |
| RWPE1 | 1.185140 | 0.09454 |
| HCF | 1.194183 | 0.09548 |
| Hepatocytes | 1.251941 | 0.09676 |
| NHA | 1.169587 | 0.09730 |
| HL60 | 1.253367 | 0.09798 |
| BJ | 1.183080 | 0.09828 |
| HMVECLLy | 1.202037 | 0.09950 |
| HSMMtube | 1.161625 | 0.10232 |
| HMEC | 1.176323 | 0.10718 |
| HTR8svn | 1.201152 | 0.10732 |
| HMF | 1.171526 | 0.10744 |
| RPTEC | 1.147369 | 0.10826 |
| HMVECdBlNeo | 1.183188 | 0.11508 |
| Stellate | 1.196805 | 0.11668 |
| K562 | 1.213517 | 0.12548 |
| IshikawaTamoxifen | 1.175540 | 0.12624 |
| ProgFib | 1.174572 | 0.12628 |
| HMVECdLyNeo | 1.173936 | 0.12688 |
| MCF7 | 1.163288 | 0.13202 |
| HMVECdBlAd | 1.168501 | 0.13316 |
| NHDFAd | 1.147986 | 0.13474 |
| H1hESC | 1.181167 | 0.13704 |
| WERIRb1 | 1.143419 | 0.13752 |
| HMVECdNeo | 1.167359 | 0.13760 |
| HPAEC | 1.165752 | 0.14020 |
| AoSMC | 1.136834 | 0.14228 |
| HUVEC | 1.156233 | 0.14298 |
| HRE | 1.126808 | 0.14750 |
| AoAF | 1.152339 | 0.14752 |
| HMVECdAd | 1.163288 | 0.15458 |
| SKNSHRA | 1.180500 | 0.15598 |
| H9ES | 1.150288 | 0.15598 |
| Jurkat | 1.153589 | 0.15746 |
| pHTE | 1.155603 | 0.15824 |
| Melano | 1.112421 | 0.15970 |
| HepG2 | 1.167988 | 0.16276 |
| FibroP | 1.116641 | 0.16696 |
| NT2D1 | 1.111326 | 0.16736 |
| HMVECdLyAd | 1.150621 | 0.16808 |
| SKNMC | 1.131502 | 0.16844 |
| GM12878 | 1.172374 | 0.16932 |
| Fibrobl | 1.139811 | 0.18574 |
| GM19239 | 1.160147 | 0.19440 |
| T47D | 1.137820 | 0.20950 |
| Th2 | 1.156923 | 0.21716 |
| GM19238 | 1.138425 | 0.22010 |
| GM19240 | 1.120813 | 0.22572 |
| Osteobl | 1.122164 | 0.22720 |
| GM12892 | 1.147134 | 0.23110 |
| CLL | 1.140297 | 0.23112 |
| CMK | 1.130928 | 0.23200 |
| PanIslets | 1.118556 | 0.23340 |
| iPS | 1.138060 | 0.24532 |
| GM18507 | 1.112041 | 0.25802 |
| Urothelia | 1.098369 | 0.25982 |
| UrotheliaUT189 | 1.094131 | 0.27270 |
| GM12891 | 1.111949 | 0.27822 |
| Chorion | 1.112592 | 0.27856 |
| Caco2 | 1.136795 | 0.28346 |
| LNCaPAndrogen | 1.091625 | 0.28942 |
| H7hESC | 1.043126 | 0.30536 |
| LNCaP | 1.065467 | 0.31050 |
| Th1 | 1.054814 | 0.37106 |
| MCF7Hypoxia | 1.040981 | 0.39482 |
| Th0 | 1.034418 | 0.41536 |
| Medullo | 1.001191 | 0.48790 |

## Serum metabolite levels

| DHS sample | fold enrichment | p value |
|---|---|---|
| RPTEC | 1.2811880 | 0.00048 |
| HRCEpiC | 1.2516468 | 0.00298 |
| HEEpiC | 1.1999645 | 0.00874 |
| HRE | 1.2067082 | 0.00982 |
| PANC1 | 1.2560330 | 0.01054 |
| PrEC | 1.1804825 | 0.01414 |
| SAEC | 1.1800830 | 0.01586 |
| AG09319 | 1.2078905 | 0.02264 |
| HIPEpiC | 1.1747493 | 0.02326 |
| HPdLF | 1.1982361 | 0.02356 |
| HCFaa | 1.1839180 | 0.02370 |
| HConF | 1.1877232 | 0.02772 |
| HPDE6E6E7 | 1.1984309 | 0.03156 |
| HGF | 1.1938778 | 0.03194 |
| SKMC | 1.1764496 | 0.03222 |
| HAsp | 1.1785571 | 0.03550 |
| HPAF | 1.1660674 | 0.03848 |
| AoSMC | 1.1507378 | 0.03856 |
| HMF | 1.1660257 | 0.03956 |
| HAEpiC | 1.1661820 | 0.04024 |
| HCPEpiC | 1.1541581 | 0.04244 |
| NB4 | 1.1898040 | 0.04386 |
| HRGEC | 1.1682526 | 0.04572 |
| HBMEC | 1.1470377 | 0.05076 |
| AoAF | 1.1573586 | 0.05188 |
| HFFMyc | 1.1467221 | 0.05260 |
| NHDFAd | 1.1422183 | 0.05890 |
| HAh | 1.1310308 | 0.05928 |
| HCM | 1.1381895 | 0.06090 |
| HPAEC | 1.1549525 | 0.06116 |
| HRPEpiC | 1.1325855 | 0.06256 |
| HNPCEpiC | 1.1294161 | 0.06776 |
| NHEK | 1.1506629 | 0.06910 |
| MonocytesCD14RO01746 | 1.1849018 | 0.07066 |
| WI38 | 1.1473231 | 0.07126 |
| BJ | 1.1467160 | 0.07212 |
| HPF | 1.1442924 | 0.07268 |
| AG04450 | 1.1444260 | 0.07392 |
| HL60 | 1.1811851 | 0.07446 |
| AG04449 | 1.1363981 | 0.07504 |
| HMVECLBl | 1.1430886 | 0.07578 |
| HFF | 1.1309574 | 0.07860 |
| HMVECdAd | 1.1520965 | 0.08022 |
| NHA | 1.1317733 | 0.08138 |
| NHLF | 1.1265607 | 0.08266 |
| HCF | 1.1332823 | 0.08524 |
| AG09309 | 1.1180299 | 0.09804 |
| RWPE1 | 1.1303109 | 0.10668 |
| HMVECdBlNeo | 1.1247144 | 0.10762 |
| HMVECdBlAd | 1.1228445 | 0.10858 |
| HAc | 1.1070560 | 0.11838 |
| HSMMemb | 1.1214175 | 0.11968 |
| HMVECdLyNeo | 1.1175949 | 0.12662 |
| AG10803 | 1.1134717 | 0.12662 |
| IshikawaEstradiol | 1.1190402 | 0.13072 |
| K562 | 1.1329051 | 0.13128 |
| Jurkat | 1.1096667 | 0.13490 |
| HVMF | 1.1134879 | 0.13728 |
| NHDFneo | 1.1062921 | 0.14134 |
| HeLaS3 | 1.1225660 | 0.14280 |
| GM12864 | 1.1183862 | 0.14752 |
| GM12865 | 1.1103151 | 0.15660 |
| HMVECdNeo | 1.1038629 | 0.16240 |
| HTR8svn | 1.1033411 | 0.16242 |
| Myometr | 1.0979966 | 0.16274 |
| Stellate | 1.1092869 | 0.16520 |
| HMVECdLyAd | 1.1017705 | 0.16596 |
| HepG2 | 1.1088619 | 0.16752 |
| A549 | 1.1103100 | 0.17406 |
| GM12878 | 1.1107238 | 0.17472 |
| HeLaS3IFNa4h | 1.1159139 | 0.17684 |
| HCT116 | 1.0982935 | 0.18362 |
| HMVECLLy | 1.0915535 | 0.18894 |
| CD20 | 1.1146092 | 0.19686 |
| Huh7 | 1.0935641 | 0.21246 |
| HSMM | 1.0660452 | 0.22018 |
| IshikawaTamoxifen | 1.0824619 | 0.22130 |
| CD34Mobilized | 1.0806668 | 0.22460 |
| HUVEC | 1.0733003 | 0.24326 |
| PanIsletD | 1.0600529 | 0.25786 |
| CMK | 1.0813114 | 0.26024 |
| Melano | 1.0501584 | 0.26790 |
| H7hESC | 1.0441599 | 0.27402 |
| CLL | 1.0672594 | 0.29314 |
| ProgFib | 1.0523363 | 0.31076 |
| FibroP | 1.0409242 | 0.31100 |
| GM06990 | 1.0602765 | 0.31562 |
| BE2C | 1.0454963 | 0.33962 |
| NT2D1 | 1.0345618 | 0.34274 |
| GM18507 | 1.0438715 | 0.34512 |
| GM19238 | 1.0396949 | 0.36354 |
| Huh7.5 | 1.0276937 | 0.40362 |
| Th2 | 1.0281372 | 0.40446 |
| H9ES | 1.0245227 | 0.40742 |
| Medullo | 1.0259907 | 0.40900 |
| HSMMtube | 1.0187987 | 0.41504 |
| PanIslets | 1.0223928 | 0.41556 |
| Urothelia | 1.0216813 | 0.41838 |
| Th0 | 1.0166111 | 0.43802 |
| GM19240 | 1.0127655 | 0.44722 |
| GM19239 | 1.0119489 | 0.45214 |
| Hepatocytes | 1.0093637 | 0.46452 |
| UrotheliaUT189 | 1.0077497 | 0.46772 |
| Gliobla | 0.9986879 | 0.49620 |
| WERIRb1 | 0.9990883 | 0.49716 |
| LNCaPAndrogen | 0.9939225 | 0.51250 |
| GM12891 | 0.9918882 | 0.51964 |
| HMEC | 0.9931283 | 0.52380 |
| SKNSHRA | 0.9867042 | 0.52406 |
| 8988T | 0.9856457 | 0.52708 |
| MCF7 | 0.9824987 | 0.55502 |
| Caco2 | 0.9687132 | 0.55722 |
| Fibrobl | 0.9842199 | 0.56084 |
| GM12892 | 0.9752582 | 0.57452 |
| Osteobl | 0.9793646 | 0.57572 |
| pHTE | 0.9733363 | 0.59890 |
| Th1 | 0.9715931 | 0.60988 |
| H1hESC | 0.9530935 | 0.63672 |
| Chorion | 0.9401790 | 0.65896 |
| SKNMC | 0.9555354 | 0.66608 |
| T47D | 0.9423114 | 0.67314 |
| MCF7Hypoxia | 0.9405202 | 0.67876 |
| iPS | 0.9191925 | 0.72000 |
| LNCaP | 0.9212070 | 0.77022 |

# Lipid levels 5

| DHS sample | fold enrichment | p value |
|---|---|---|
| RPTEC | 1.3647259 | 0.00064 |
| HRCEpiC | 1.3460831 | 0.00180 |
| HL60 | 1.4901300 | 0.00380 |
| HRE | 1.2774664 | 0.00928 |
| HCM | 1.2735021 | 0.01264 |
| HAEpiC | 1.2826882 | 0.01608 |
| K562 | 1.3691359 | 0.01656 |
| Jurkat | 1.2932140 | 0.01728 |
| H7hESC | 1.2002492 | 0.01860 |
| SKNMC | 1.3038813 | 0.01910 |
| IshikawaEstradiol | 1.3031382 | 0.02004 |
| HPAF | 1.2654386 | 0.02062 |
| HEEpiC | 1.2253761 | 0.02176 |
| SAEC | 1.2218196 | 0.02278 |
| HIPEpiC | 1.2301875 | 0.02878 |
| HCF | 1.2541326 | 0.03076 |
| HRGEC | 1.2621029 | 0.03084 |
| PrEC | 1.2103511 | 0.03316 |
| HCPEpiC | 1.2229906 | 0.03684 |
| AoSMC | 1.2064162 | 0.03858 |
| IshikawaTamoxifen | 1.2614220 | 0.04118 |
| HCFaa | 1.2202641 | 0.04166 |
| HepG2 | 1.2823361 | 0.04248 |
| HPAEC | 1.2439661 | 0.04708 |
| Huh7 | 1.2837555 | 0.04794 |
| HRPEpiC | 1.1805001 | 0.05026 |
| HMVECdBlAd | 1.2300331 | 0.05126 |
| HNPCEpiC | 1.1827430 | 0.05626 |
| HeLaS3 | 1.2457282 | 0.05782 |
| HAh | 1.1722675 | 0.05876 |
| MonocytesCD14RO01746 | 1.2735778 | 0.06142 |
| HMVECdLyNeo | 1.2258460 | 0.06270 |
| HMVECLBl | 1.2108910 | 0.06320 |
| HMVECdAd | 1.2358115 | 0.06574 |
| HVMF | 1.2174193 | 0.06754 |
| HCT116 | 1.2353056 | 0.06772 |
| SKMC | 1.1850873 | 0.06806 |
| HMVECdBlNeo | 1.2117876 | 0.06838 |
| HeLaS3IFNa4h | 1.2578430 | 0.06894 |
| WI38 | 1.2044441 | 0.07114 |
| NHEK | 1.2006001 | 0.07138 |
| NB4 | 1.2305761 | 0.07322 |
| HGF | 1.1989017 | 0.07500 |
| HMVECdNeo | 1.2145707 | 0.07526 |
| PanIsletD | 1.1879027 | 0.07534 |
| PANC1 | 1.2191316 | 0.07676 |
| Stellate | 1.2268786 | 0.07826 |
| WERIRb1 | 1.1877380 | 0.07876 |
| HPF | 1.1860764 | 0.08272 |
| AoAF | 1.1797229 | 0.08480 |
| AG04450 | 1.1865057 | 0.08540 |
| Huh7.5 | 1.2357531 | 0.08638 |
| HPDE6E6E7 | 1.2068912 | 0.08790 |
| HMVECLLy | 1.1995960 | 0.09022 |
| GM12878 | 1.2236312 | 0.09132 |
| HSMMemb | 1.1911988 | 0.09164 |
| NHA | 1.1623810 | 0.09218 |
| BE2C | 1.2084291 | 0.09290 |
| GM12864 | 1.2081355 | 0.09400 |
| GM12865 | 1.1986353 | 0.09616 |
| Melano | 1.1419873 | 0.09732 |
| GM19240 | 1.1940633 | 0.10054 |
| HMVECdLyAd | 1.1948252 | 0.10100 |
| NHLF | 1.1484660 | 0.10560 |
| FibroP | 1.1409218 | 0.10764 |
| HConF | 1.1601014 | 0.11220 |
| Myometr | 1.1674627 | 0.11350 |
| HBMEC | 1.1414436 | 0.11792 |
| HPdLF | 1.1565930 | 0.12138 |
| Medullo | 1.1800874 | 0.12340 |
| NT2D1 | 1.1332589 | 0.12554 |
| AG04449 | 1.1435134 | 0.12918 |
| HMF | 1.1414059 | 0.13408 |
| HUVEC | 1.1618610 | 0.13882 |
| GM18507 | 1.1739574 | 0.14266 |
| CD34Mobilized | 1.1591083 | 0.14518 |
| PanIslets | 1.1658154 | 0.14692 |
| Th0 | 1.1633370 | 0.14718 |
| HFFMyc | 1.1248349 | 0.14792 |
| RWPE1 | 1.1448475 | 0.14856 |
| CMK | 1.1912482 | 0.14932 |
| HSMM | 1.1150389 | 0.15408 |
| HSMMtube | 1.1236446 | 0.15424 |
| HTR8svn | 1.1481706 | 0.15698 |
| A549 | 1.1583342 | 0.16044 |
| HAsp | 1.1214476 | 0.16294 |
| UrotheliaUT189 | 1.1407547 | 0.17252 |
| AG10803 | 1.1202799 | 0.18120 |
| Urothelia | 1.1302045 | 0.18894 |
| HFF | 1.1076570 | 0.19110 |
| AG09309 | 1.1022852 | 0.19598 |
| HAc | 1.0978027 | 0.19626 |
| pHTE | 1.1216535 | 0.19780 |
| Fibrobl | 1.1186509 | 0.20026 |
| GM19239 | 1.1464139 | 0.20338 |
| Th2 | 1.1413526 | 0.20732 |
| ProgFib | 1.1181566 | 0.21288 |
| Hepatocytes | 1.1439577 | 0.21516 |
| GM06990 | 1.1366758 | 0.21650 |
| GM19238 | 1.1301098 | 0.21746 |
| HMEC | 1.1044069 | 0.21790 |
| Osteobl | 1.1129544 | 0.21926 |
| Gliobla | 1.1259443 | 0.21970 |
| BJ | 1.0989584 | 0.22044 |
| Th1 | 1.1135952 | 0.22394 |
| LNCaPAndrogen | 1.1237280 | 0.22590 |
| MCF7 | 1.1039945 | 0.23796 |
| AG09319 | 1.0897609 | 0.24644 |
| CD20 | 1.1158063 | 0.26750 |
| GM12891 | 1.1119155 | 0.26766 |
| GM12892 | 1.1028541 | 0.28298 |
| H9ES | 1.0787979 | 0.29606 |
| NHDFAd | 1.0623153 | 0.29856 |
| CLL | 1.0891619 | 0.30458 |
| NHDFneo | 1.0608986 | 0.31512 |
| LNCaP | 1.0614445 | 0.32282 |
| Caco2 | 1.0936304 | 0.33204 |
| 8988T | 1.0669536 | 0.35056 |
| Chorion | 1.0577475 | 0.36948 |
| SKNSHRA | 1.0465707 | 0.38686 |
| iPS | 1.0356142 | 0.41706 |
| MCF7Hypoxia | 1.0232474 | 0.43018 |
| H1hESC | 1.0021278 | 0.47908 |
| T47D | 0.9450812 | 0.61026 |

## Blood cell traits

| DHS sample | fold enrichment | p value |
|---|---|---|
| Th2 | 1.6347033 | 0.00070 |
| Jurkat | 1.4663083 | 0.00140 |
| RPTEC | 1.4142033 | 0.00178 |
| SKNMC | 1.4793133 | 0.00312 |
| HCM | 1.4012108 | 0.00324 |
| HRCEpiC | 1.3665842 | 0.00726 |
| HRGEC | 1.4114995 | 0.00786 |
| GM12864 | 1.4393332 | 0.00954 |
| GM06990 | 1.4931719 | 0.01026 |
| HAEpiC | 1.3703102 | 0.01066 |
| AoSMC | 1.3153914 | 0.01090 |
| NB4 | 1.4106308 | 0.01094 |
| BJ | 1.3870704 | 0.01138 |
| GM12865 | 1.4174599 | 0.01168 |
| HRE | 1.3301089 | 0.01254 |
| HCFaa | 1.3432174 | 0.01256 |
| CMK | 1.4814303 | 0.01334 |
| HCF | 1.3635968 | 0.01546 |
| HIPEpiC | 1.3060994 | 0.01632 |
| HVMF | 1.3612323 | 0.01852 |
| AG09309 | 1.3105365 | 0.01898 |
| PANC1 | 1.3892384 | 0.01918 |
| HNPCEpiC | 1.2956666 | 0.02036 |
| HAsp | 1.3331499 | 0.02086 |
| HCPEpiC | 1.3060999 | 0.02096 |
| HPAF | 1.3124510 | 0.02280 |
| K562 | 1.3808797 | 0.02322 |
| AG10803 | 1.3299768 | 0.02386 |
| HMF | 1.3085276 | 0.02496 |
| HL60 | 1.3800092 | 0.02902 |
| SKMC | 1.2950595 | 0.02968 |
| NHDFneo | 1.3279306 | 0.03028 |
| HConF | 1.3196656 | 0.03030 |
| HFFMyc | 1.2876044 | 0.03084 |
| HPdLF | 1.3158509 | 0.03122 |
| AoAF | 1.3098888 | 0.03226 |
| HBMEC | 1.2845649 | 0.03236 |
| CD34Mobilized | 1.3131810 | 0.03398 |
| HFF | 1.2803045 | 0.03548 |
| HMVECdBlNeo | 1.2988932 | 0.03656 |
| HPAEC | 1.2994360 | 0.03728 |
| NHA | 1.2775287 | 0.03728 |
| HMVECLBl | 1.2889407 | 0.03956 |
| MonocytesCD14RO01746 | 1.3494081 | 0.03994 |
| NHDFAd | 1.2733410 | 0.04094 |
| HRPEpiC | 1.2531164 | 0.04688 |
| SAEC | 1.2370728 | 0.04726 |
| NHLF | 1.2574243 | 0.04770 |
| AG04449 | 1.2645306 | 0.04788 |
| AG09319 | 1.2809863 | 0.05278 |
| HMVECdBlAd | 1.2674591 | 0.05562 |
| HMVECdLyNeo | 1.2710990 | 0.06122 |
| HEEpiC | 1.2176420 | 0.06144 |
| HGF | 1.2683057 | 0.06346 |
| GM12878 | 1.2868750 | 0.06388 |
| HAc | 1.2318170 | 0.06482 |
| HPF | 1.2504512 | 0.06720 |
| AG04450 | 1.2488107 | 0.07068 |
| HAh | 1.2000149 | 0.08130 |
| HMVECdAd | 1.2459109 | 0.08482 |
| WI38 | 1.2254874 | 0.08654 |
| HMVECdNeo | 1.2428219 | 0.08784 |
| PrEC | 1.1877474 | 0.08872 |
| HMVECLLy | 1.2343768 | 0.09350 |
| HCT116 | 1.2390210 | 0.09452 |
| H7hESC | 1.1509257 | 0.10978 |
| HMVECdLyAd | 1.2160480 | 0.11532 |
| GM18507 | 1.2052639 | 0.12534 |
| HTR8svn | 1.1798611 | 0.13848 |
| CD20 | 1.2197435 | 0.15168 |
| HPDE6E6E7 | 1.1699332 | 0.15880 |
| WERIRb1 | 1.1618429 | 0.16170 |
| HeLaS3 | 1.1559508 | 0.19244 |
| RWPE1 | 1.1365242 | 0.19646 |
| Myometr | 1.1356671 | 0.19754 |
| BE2C | 1.1403769 | 0.21634 |
| HUVEC | 1.1339276 | 0.22282 |
| NHEK | 1.1201002 | 0.22794 |
| CLL | 1.1298221 | 0.25082 |
| HSMM | 1.0960455 | 0.25398 |
| GM19240 | 1.1011484 | 0.27020 |
| NT2D1 | 1.0791776 | 0.27934 |
| Urothelia | 1.0917521 | 0.28780 |
| Th1 | 1.0859648 | 0.29998 |
| Stellate | 1.0865126 | 0.30910 |
| PanIsletD | 1.0745893 | 0.31202 |
| FibroP | 1.0619209 | 0.32230 |
| Th0 | 1.0733828 | 0.33200 |
| HSMMemb | 1.0654227 | 0.34710 |
| GM19238 | 1.0638267 | 0.35916 |
| MCF7 | 1.0532481 | 0.36704 |
| SKNSHRA | 1.0538018 | 0.38686 |
| UrotheliaUT189 | 1.0423557 | 0.39462 |
| IshikawaEstradiol | 1.0433552 | 0.39488 |
| IshikawaTamoxifen | 1.0432872 | 0.39728 |
| HepG2 | 1.0405207 | 0.40372 |
| H9ES | 1.0376996 | 0.40750 |
| GM19239 | 1.0333691 | 0.42200 |
| HSMMtube | 1.0266421 | 0.42506 |
| HeLaS3IFNa4h | 1.0273787 | 0.43238 |
| Melano | 1.0116858 | 0.46746 |
| A549 | 1.0066351 | 0.47182 |
| GM12892 | 1.0078048 | 0.47884 |
| ProgFib | 0.9785247 | 0.54176 |
| pHTE | 0.9801123 | 0.54548 |
| T47D | 0.9668380 | 0.54950 |
| HMEC | 0.9779482 | 0.55114 |
| GM12891 | 0.9695035 | 0.55254 |
| Gliobla | 0.9674041 | 0.55716 |
| Huh7 | 0.9657708 | 0.56094 |
| H1hESC | 0.9475912 | 0.58748 |
| MCF7Hypoxia | 0.9340873 | 0.62710 |
| LNCaP | 0.9355891 | 0.64508 |
| Fibrobl | 0.9336996 | 0.66334 |
| 8988T | 0.8944756 | 0.67892 |
| Medullo | 0.9139290 | 0.68896 |
| LNCaPAndrogen | 0.9004566 | 0.69962 |
| iPS | 0.8849547 | 0.70486 |
| Hepatocytes | 0.8796591 | 0.71428 |
| PanIslets | 0.8996350 | 0.71792 |
| Osteobl | 0.9075945 | 0.72096 |
| Chorion | 0.8608206 | 0.73976 |
| Huh7.5 | 0.8600510 | 0.76336 |
| Caco2 | 0.7943239 | 0.78846 |

## Type 2 diabetes 2

| DHS sample | fold enrichment | p value |
|---|---|---|
| HAsp | 1.3636591 | 0.00072 |
| HCPEpiC | 1.3327214 | 0.00148 |
| HIPEpiC | 1.3333166 | 0.00162 |
| HNPCEpiC | 1.3022115 | 0.00192 |
| SKMC | 1.3193928 | 0.00266 |
| HRCEpiC | 1.2946532 | 0.00280 |
| HAEpiC | 1.3416976 | 0.00286 |
| WI38 | 1.3440536 | 0.00368 |
| HRPEpiC | 1.2538407 | 0.00398 |
| HBMEC | 1.2781829 | 0.00508 |
| AG04449 | 1.3073911 | 0.00510 |
| NHLF | 1.2879500 | 0.00516 |
| HFFMyc | 1.2884584 | 0.00518 |
| HRE | 1.2611551 | 0.00544 |
| RPTEC | 1.2532383 | 0.00602 |
| HCFaa | 1.2907105 | 0.00650 |
| NHDFAd | 1.2861688 | 0.00666 |
| HVMF | 1.3274969 | 0.00790 |
| NHDFneo | 1.3029104 | 0.00820 |
| HAc | 1.2552499 | 0.00832 |
| HCT116 | 1.3366515 | 0.00876 |
| AG04450 | 1.3002619 | 0.00894 |
| HFF | 1.2795676 | 0.00924 |
| NHA | 1.2692966 | 0.00934 |
| HPdLF | 1.2936461 | 0.01014 |
| HGF | 1.2844270 | 0.01384 |
| BJ | 1.2686724 | 0.01510 |
| AG09309 | 1.2432506 | 0.01612 |
| HEEpiC | 1.2114197 | 0.01614 |
| AG10803 | 1.2641299 | 0.01850 |
| HPF | 1.2551861 | 0.01864 |
| SAEC | 1.2010647 | 0.02076 |
| HConF | 1.2445397 | 0.02226 |
| HMF | 1.2344191 | 0.02254 |
| HPAF | 1.2328738 | 0.02424 |
| HMVECLBl | 1.2452894 | 0.02562 |
| HAh | 1.1815318 | 0.02930 |
| HCM | 1.2115824 | 0.03092 |
| AG09319 | 1.2393921 | 0.03108 |
| AoSMC | 1.1952240 | 0.03550 |
| AoAF | 1.2195778 | 0.03666 |
| HSMM | 1.1762343 | 0.04320 |
| HTR8svn | 1.2311746 | 0.04720 |
| PANC1 | 1.2241755 | 0.05108 |
| HCF | 1.1981852 | 0.05454 |
| PrEC | 1.1553874 | 0.05760 |
| Gliobla | 1.2260870 | 0.06682 |
| HRGEC | 1.1859182 | 0.06692 |
| Stellate | 1.2125928 | 0.06952 |
| MonocytesCD14RO01746 | 1.2451824 | 0.07088 |
| HSMMemb | 1.1864886 | 0.07732 |
| HSMMtube | 1.1515944 | 0.08290 |
| HMVECdBlNeo | 1.1741055 | 0.08402 |
| PanIsletD | 1.1468801 | 0.09308 |
| HeLaS3 | 1.1691723 | 0.10768 |
| HeLaS3IFNa4h | 1.1849869 | 0.11096 |
| HepG2 | 1.1702996 | 0.12002 |
| IshikawaTamoxifen | 1.1499094 | 0.12122 |
| Myometr | 1.1368812 | 0.12146 |
| CD34Mobilized | 1.1537749 | 0.12560 |
| IshikawaEstradiol | 1.1435565 | 0.12606 |
| NHEK | 1.1355508 | 0.12884 |
| A549 | 1.1461166 | 0.14494 |
| HMVECdBlAd | 1.1235481 | 0.15986 |
| ProgFib | 1.1266656 | 0.16198 |
| NB4 | 1.1420612 | 0.16312 |
| HMVECLLy | 1.1260116 | 0.16340 |
| Jurkat | 1.1211446 | 0.16654 |
| SKNMC | 1.1098099 | 0.17010 |
| HPAEC | 1.1207980 | 0.17094 |
| RWPE1 | 1.1093233 | 0.17980 |
| H7hESC | 1.0662536 | 0.19914 |
| T47D | 1.1256438 | 0.20452 |
| FibroP | 1.0804448 | 0.21248 |
| Huh7 | 1.1091278 | 0.22564 |
| HMVECdLyNeo | 1.0955689 | 0.22682 |
| HMVECdLyAd | 1.0923621 | 0.23460 |
| HMVECdAd | 1.0953083 | 0.23520 |
| Fibrobl | 1.0929339 | 0.23740 |
| Huh7.5 | 1.1005339 | 0.24684 |
| HMVECdNeo | 1.0843252 | 0.25228 |
| HMEC | 1.0758151 | 0.26038 |
| CD20 | 1.1073732 | 0.26172 |
| WERIRb1 | 1.0712126 | 0.26550 |
| HL60 | 1.0959210 | 0.27238 |
| HUVEC | 1.0720900 | 0.27824 |
| 8988T | 1.0879102 | 0.30038 |
| GM12865 | 1.0641101 | 0.31068 |
| Osteobl | 1.0632228 | 0.31958 |
| Melano | 1.0405491 | 0.33740 |
| HPDE6E6E7 | 1.0514668 | 0.34128 |
| pHTE | 1.0430553 | 0.36386 |
| UrotheliaUT189 | 1.0413475 | 0.37218 |
| GM12864 | 1.0432462 | 0.37520 |
| K562 | 1.0427586 | 0.38228 |
| Th1 | 1.0388316 | 0.38846 |
| SKNSHRA | 1.0326222 | 0.40024 |
| PanIslets | 1.0313011 | 0.40234 |
| MCF7 | 1.0229950 | 0.41924 |
| Urothelia | 1.0193587 | 0.43512 |
| LNCaPAndrogen | 1.0163912 | 0.44496 |
| GM12878 | 1.0149529 | 0.45022 |
| H1hESC | 1.0109892 | 0.45736 |
| LNCaP | 1.0096475 | 0.45790 |
| BE2C | 1.0021621 | 0.47668 |
| Caco2 | 0.9955828 | 0.48576 |
| Th2 | 0.9976188 | 0.49564 |
| NT2D1 | 0.9985356 | 0.49636 |
| H9ES | 0.9935337 | 0.51026 |
| Th0 | 0.9937075 | 0.51226 |
| GM19240 | 0.9913317 | 0.51832 |
| GM06990 | 0.9859207 | 0.52160 |
| Hepatocytes | 0.9845445 | 0.52438 |
| GM12891 | 0.9842575 | 0.52442 |
| Chorion | 0.9784349 | 0.53422 |
| iPS | 0.9743506 | 0.54852 |
| GM19239 | 0.9736191 | 0.55742 |
| CLL | 0.9635572 | 0.57506 |
| Medullo | 0.9648919 | 0.58740 |
| GM18507 | 0.9625503 | 0.59418 |
| GM12892 | 0.9530947 | 0.59894 |
| MCF7Hypoxia | 0.9323938 | 0.66228 |
| GM19238 | 0.9262822 | 0.67646 |
| CMK | 0.9150578 | 0.69038 |

# Pulmonary function

| DHS sample | fold enrichment | p value |
|---|---|---|
| NHLF | 1.2348740 | 0.04542 |
| HAh | 1.1992030 | 0.05538 |
| HFFMyc | 1.2240599 | 0.06072 |
| NHA | 1.2105857 | 0.06416 |
| HAsp | 1.2097911 | 0.06614 |
| HIPEpiC | 1.2149504 | 0.06676 |
| AG09319 | 1.2261561 | 0.07728 |
| NHDFneo | 1.2145887 | 0.07952 |
| HCFaa | 1.2077036 | 0.08070 |
| HNPCEpiC | 1.1879418 | 0.08090 |
| BJ | 1.2005801 | 0.09464 |
| HCPEpiC | 1.1760958 | 0.10856 |
| HVMF | 1.2015248 | 0.11182 |
| HRPEpiC | 1.1450437 | 0.11622 |
| HBMEC | 1.1569702 | 0.12390 |
| HMVECLBl | 1.1808413 | 0.13212 |
| AoSMC | 1.1589606 | 0.13534 |
| HRGEC | 1.1721070 | 0.14214 |
| HGF | 1.1614439 | 0.15554 |
| HPF | 1.1547318 | 0.15602 |
| SKMC | 1.1313888 | 0.17396 |
| HAEpiC | 1.1403505 | 0.17446 |
| HConF | 1.1415846 | 0.17686 |
| AoAF | 1.1425435 | 0.17930 |
| HRE | 1.1170030 | 0.18748 |
| AG04449 | 1.1333130 | 0.18768 |
| AG10803 | 1.1361577 | 0.18970 |
| HAc | 1.1094190 | 0.19980 |
| HTR8svn | 1.1362197 | 0.21290 |
| HRCEpiC | 1.1079435 | 0.21922 |
| HPAEC | 1.1252939 | 0.22818 |
| HFF | 1.1062899 | 0.23376 |
| NHDFAd | 1.1013201 | 0.23586 |
| WI38 | 1.1104792 | 0.24612 |
| HEEpiC | 1.0892022 | 0.24908 |
| AG04450 | 1.1068200 | 0.24918 |
| SAEC | 1.0882124 | 0.25148 |
| AG09309 | 1.0954773 | 0.25184 |
| RWPE1 | 1.1017498 | 0.25854 |
| PrEC | 1.0842708 | 0.26086 |
| HMVECdBlAd | 1.1026934 | 0.26152 |
| HPdLF | 1.0918053 | 0.27672 |
| Stellate | 1.1010338 | 0.28290 |
| HPDE6E6E7 | 1.0801792 | 0.31310 |
| HUVEC | 1.0749787 | 0.31770 |
| HCM | 1.0572105 | 0.34432 |
| Myometr | 1.0570028 | 0.34810 |
| HSMM | 1.0511015 | 0.34914 |
| PanIsletD | 1.0534605 | 0.35192 |
| HCT116 | 1.0600751 | 0.35242 |
| A549 | 1.0583552 | 0.36174 |
| HCF | 1.0526574 | 0.36378 |
| HPAF | 1.0495155 | 0.36488 |
| HMVECdBlNeo | 1.0538803 | 0.36554 |
| GM12864 | 1.0596773 | 0.36702 |
| HMVECdLyNeo | 1.0492065 | 0.37990 |
| Urothelia | 1.0487920 | 0.38190 |
| HMF | 1.0400916 | 0.38610 |
| RPTEC | 1.0372055 | 0.38636 |
| HSMMemb | 1.0380677 | 0.40732 |
| CD20 | 1.0361823 | 0.41924 |
| HMVECdNeo | 1.0278944 | 0.42686 |
| HMVECdAd | 1.0270937 | 0.42770 |
| HMVECdLyAd | 1.0235484 | 0.43366 |
| IshikawaTamoxifen | 1.0152799 | 0.45228 |
| HMVECLLy | 1.0078480 | 0.47356 |
| GM06990 | 1.0021070 | 0.47752 |
| IshikawaEstradiol | 1.0039137 | 0.47904 |
| FibroP | 1.0056824 | 0.48138 |
| PANC1 | 0.9993108 | 0.48530 |
| HSMMtube | 0.9967841 | 0.50446 |
| MonocytesCD14RO01746 | 0.9882808 | 0.50816 |
| SKNSHRA | 0.9859513 | 0.51150 |
| HeLaS3IFNa4h | 0.9854480 | 0.51542 |
| GM12865 | 0.9862404 | 0.51884 |
| Gliobla | 0.9822806 | 0.52272 |
| HeLaS3 | 0.9797077 | 0.53324 |
| NHEK | 0.9809966 | 0.54060 |
| UrotheliaUT189 | 0.9773210 | 0.54102 |
| GM12878 | 0.9631432 | 0.55914 |
| ProgFib | 0.9554291 | 0.59542 |
| HepG2 | 0.9374533 | 0.61718 |
| CLL | 0.9223384 | 0.62854 |
| GM18507 | 0.9266683 | 0.63982 |
| Melano | 0.9494404 | 0.65254 |
| SKNMC | 0.9370441 | 0.66374 |
| CD34Mobilized | 0.9204396 | 0.66652 |
| 8988T | 0.8967459 | 0.66730 |
| Caco2 | 0.8446223 | 0.68460 |
| Huh7.5 | 0.8940344 | 0.69296 |
| GM19238 | 0.8867555 | 0.70802 |
| Huh7 | 0.8853634 | 0.71474 |
| MCF7 | 0.8996415 | 0.71638 |
| Th0 | 0.8871882 | 0.71974 |
| WERIRb1 | 0.9124261 | 0.72290 |
| GM19240 | 0.8908258 | 0.72450 |
| Chorion | 0.8586761 | 0.73270 |
| T47D | 0.8679661 | 0.74132 |
| pHTE | 0.8858596 | 0.74696 |
| Fibrobl | 0.8803622 | 0.75514 |
| GM12891 | 0.8412073 | 0.76412 |
| H1hESC | 0.8647893 | 0.76424 |
| GM19239 | 0.8466593 | 0.77042 |
| Th2 | 0.8375014 | 0.77764 |
| Th1 | 0.8507363 | 0.78530 |
| GM12892 | 0.8219081 | 0.79022 |
| MCF7Hypoxia | 0.8299512 | 0.79072 |
| K562 | 0.8367060 | 0.79332 |
| Osteobl | 0.8549658 | 0.79354 |
| HMEC | 0.8698116 | 0.79556 |
| LNCaP | 0.8699189 | 0.79808 |
| Hepatocytes | 0.8234914 | 0.79816 |
| NT2D1 | 0.8831262 | 0.81146 |
| LNCaPAndrogen | 0.8315964 | 0.81174 |
| Jurkat | 0.8510801 | 0.81448 |
| PanIslets | 0.8367878 | 0.81828 |
| H9ES | 0.8457249 | 0.81880 |
| BE2C | 0.8531043 | 0.82192 |
| CMK | 0.8132027 | 0.82476 |
| iPS | 0.7863478 | 0.83700 |
| H7hESC | 0.8855887 | 0.86932 |
| NB4 | 0.7851629 | 0.88430 |
| HL60 | 0.7424293 | 0.90406 |
| Medullo | 0.7101786 | 0.94260 |

## Chronic lymphocytic leukemia 2

| DHS sample | fold enrichment | p value |
|---|---|---|
| GM06990 | 1.7299562 | 0.00110 |
| GM12864 | 1.6168871 | 0.00126 |
| GM12865 | 1.5590347 | 0.00178 |
| CD20 | 1.5366869 | 0.02074 |
| Th2 | 1.4473788 | 0.02326 |
| GM18507 | 1.3857411 | 0.02928 |
| GM12878 | 1.3998294 | 0.02944 |
| MonocytesCD14RO01746 | 1.4077149 | 0.03572 |
| GM19238 | 1.3002020 | 0.07732 |
| CLL | 1.3270081 | 0.07896 |
| NB4 | 1.2532981 | 0.08876 |
| GM19240 | 1.2252810 | 0.11866 |
| HL60 | 1.2010608 | 0.17356 |
| GM19239 | 1.1584156 | 0.22604 |
| GM12892 | 1.1698755 | 0.23136 |
| Th1 | 1.1396220 | 0.23632 |
| Th0 | 1.1131808 | 0.28080 |
| GM12891 | 1.0973570 | 0.32650 |
| CD34Mobilized | 1.0334591 | 0.41404 |
| T47D | 1.0249489 | 0.43332 |
| AG04450 | 1.0076830 | 0.47764 |
| AoSMC | 1.0027828 | 0.49102 |
| HAsp | 0.9936395 | 0.50762 |
| Caco2 | 0.9411193 | 0.54128 |
| AoAF | 0.9798420 | 0.54530 |
| HPF | 0.9771030 | 0.54892 |
| HCM | 0.9690715 | 0.58558 |
| HPAF | 0.9557409 | 0.60684 |
| HCF | 0.9447666 | 0.62886 |
| HNPCEpiC | 0.9462439 | 0.64422 |
| Jurkat | 0.9348291 | 0.64576 |
| HMVECdBlAd | 0.9322679 | 0.64970 |
| 8988T | 0.8887679 | 0.65810 |
| Urothelia | 0.9173991 | 0.66352 |
| BE2C | 0.9139000 | 0.66494 |
| pHTE | 0.9135698 | 0.67490 |
| Osteobl | 0.9039484 | 0.68646 |
| NHLF | 0.9211966 | 0.69354 |
| HRGEC | 0.9144214 | 0.69370 |
| NHDFneo | 0.9143625 | 0.69456 |
| HAc | 0.9262046 | 0.69670 |
| AG09319 | 0.9046184 | 0.71800 |
| MCF7Hypoxia | 0.8756364 | 0.71954 |
| WI38 | 0.9017283 | 0.72174 |
| HMVECLBl | 0.9029271 | 0.72214 |
| MCF7 | 0.8971213 | 0.72226 |
| HPdLF | 0.8990091 | 0.73090 |
| SKNMC | 0.8805386 | 0.73096 |
| HAh | 0.9169693 | 0.73408 |
| SKMC | 0.9030826 | 0.73408 |
| HTR8svn | 0.8836782 | 0.73890 |
| NHA | 0.8978958 | 0.74260 |
| Chorion | 0.8271969 | 0.74554 |
| RPTEC | 0.9083425 | 0.75118 |
| HMVECdBlNeo | 0.8853588 | 0.75192 |
| HBMEC | 0.9018124 | 0.75342 |
| SAEC | 0.9054997 | 0.75772 |
| HPAEC | 0.8774330 | 0.75900 |
| Fibrobl | 0.8677155 | 0.76454 |
| HCFaa | 0.8912235 | 0.76526 |
| Melano | 0.9011819 | 0.77292 |
| HRCEpiC | 0.8934336 | 0.77502 |
| HIPEpiC | 0.8904720 | 0.77520 |
| LNCaP | 0.8642208 | 0.77764 |
| HMVECdLyAd | 0.8597324 | 0.77818 |
| PanIslets | 0.8449892 | 0.77836 |
| HCPEpiC | 0.8897950 | 0.77900 |
| HMVECLLy | 0.8556436 | 0.79004 |
| iPS | 0.7942828 | 0.79138 |
| HPDE6E6E7 | 0.8539936 | 0.79666 |
| HepG2 | 0.8383084 | 0.79964 |
| AG09309 | 0.8772900 | 0.80654 |
| PANC1 | 0.8445809 | 0.80758 |
| HGF | 0.8577817 | 0.81332 |
| HMVECdLyNeo | 0.8430637 | 0.81924 |
| HMVECdNeo | 0.8391345 | 0.82440 |
| HConF | 0.8574401 | 0.82488 |
| HMEC | 0.8412374 | 0.82720 |
| HeLaS3 | 0.8239651 | 0.82834 |
| Huh7.5 | 0.8044217 | 0.82886 |
| BJ | 0.8538630 | 0.82974 |
| AG04449 | 0.8555823 | 0.82990 |
| Hepatocytes | 0.7769047 | 0.83134 |
| HCT116 | 0.8344764 | 0.83364 |
| ProgFib | 0.8231772 | 0.84066 |
| PanIsletD | 0.8509915 | 0.84110 |
| HFF | 0.8552734 | 0.84112 |
| Medullo | 0.7938471 | 0.84132 |
| HVMF | 0.8277054 | 0.84550 |
| H1hESC | 0.7916776 | 0.84644 |
| NHDFAd | 0.8452853 | 0.85196 |
| HSMMemb | 0.8181911 | 0.85246 |
| RWPE1 | 0.8264383 | 0.85410 |
| LNCaPAndrogen | 0.7885916 | 0.85754 |
| Stellate | 0.8017713 | 0.85866 |
| AG10803 | 0.8310015 | 0.85906 |
| FibroP | 0.8534506 | 0.86052 |
| UrotheliaUT189 | 0.7957343 | 0.86092 |
| IshikawaTamoxifen | 0.8076936 | 0.86486 |
| HMVECdAd | 0.7999692 | 0.86816 |
| SKNSHRA | 0.7499209 | 0.87068 |
| WERIRb1 | 0.8162034 | 0.87474 |
| Gliobla | 0.7671793 | 0.87474 |
| HEEpiC | 0.8459912 | 0.87582 |
| HFFMyc | 0.8329254 | 0.87732 |
| HUVEC | 0.8085699 | 0.87768 |
| H9ES | 0.7862924 | 0.87946 |
| HRE | 0.8337189 | 0.88200 |
| HAEpiC | 0.8085967 | 0.88332 |
| HSMMtube | 0.8274006 | 0.88426 |
| HMF | 0.8254943 | 0.88542 |
| HeLaS3IFNa4h | 0.7440362 | 0.90320 |
| Huh7 | 0.7494132 | 0.90342 |
| Myometr | 0.7862808 | 0.91722 |
| IshikawaEstradiol | 0.7697309 | 0.91766 |
| NT2D1 | 0.8017501 | 0.92782 |
| HRPEpiC | 0.8048874 | 0.92996 |
| HSMM | 0.7955270 | 0.93666 |
| CMK | 0.6613904 | 0.94440 |
| PrEC | 0.7598589 | 0.96404 |
| H7hESC | 0.8134218 | 0.96458 |
| K562 | 0.6256588 | 0.97214 |
| A549 | 0.6690472 | 0.97436 |
| NHEK | 0.6929104 | 0.97780 |

## Alzheimers disease

| DHS sample | fold enrichment | p value |
|---|---|---|
| MonocytesCD14RO01746 | 1.5741885 | 0.00118 |
| GM12864 | 1.4872225 | 0.00178 |
| GM06990 | 1.5377587 | 0.00250 |
| GM12865 | 1.4436989 | 0.00310 |
| HL60 | 1.4250033 | 0.01200 |
| CD20 | 1.4435787 | 0.01526 |
| GM12878 | 1.3687561 | 0.01550 |
| GM18507 | 1.2895888 | 0.04036 |
| NB4 | 1.2480158 | 0.06206 |
| CD34Mobilized | 1.1936082 | 0.09634 |
| Th2 | 1.2244172 | 0.10030 |
| K562 | 1.1744658 | 0.14922 |
| GM19238 | 1.1716424 | 0.14928 |
| HeLaS3 | 1.1234256 | 0.20958 |
| CMK | 1.1319857 | 0.23016 |
| GM19239 | 1.1145485 | 0.25374 |
| CLL | 1.1095854 | 0.26556 |
| GM19240 | 1.0826497 | 0.28786 |
| HeLaS3IFNa4h | 1.0633954 | 0.34248 |
| GM12891 | 1.0606035 | 0.36154 |
| GM12892 | 1.0290835 | 0.42606 |
| T47D | 1.0227521 | 0.43620 |
| PANC1 | 1.0176630 | 0.43862 |
| HCT116 | 1.0172473 | 0.44152 |
| Jurkat | 1.0160849 | 0.44480 |
| Th0 | 1.0155573 | 0.45802 |
| Gliobla | 1.0114578 | 0.46068 |
| Th1 | 1.0061911 | 0.48426 |
| HRGEC | 1.0009559 | 0.49226 |
| HepG2 | 0.9957668 | 0.50342 |
| HPDE6E6E7 | 0.9966291 | 0.50344 |
| Urothelia | 0.9897195 | 0.52186 |
| AoSMC | 0.9933979 | 0.52322 |
| HMVECdBlNeo | 0.9854113 | 0.53950 |
| IshikawaEstradiol | 0.9804583 | 0.54598 |
| HUVEC | 0.9764100 | 0.55824 |
| LNCaPAndrogen | 0.9661212 | 0.57470 |
| SAEC | 0.9767439 | 0.58112 |
| MCF7Hypoxia | 0.9576567 | 0.58704 |
| MCF7 | 0.9604908 | 0.58902 |
| HMF | 0.9701442 | 0.58930 |
| RPTEC | 0.9722701 | 0.59404 |
| AG09319 | 0.9659678 | 0.59464 |
| HGF | 0.9635883 | 0.59688 |
| IshikawaTamoxifen | 0.9590109 | 0.59798 |
| A549 | 0.9551455 | 0.59938 |
| Huh7.5 | 0.9537253 | 0.60126 |
| NHDFAd | 0.9655076 | 0.61018 |
| HPF | 0.9603711 | 0.61034 |
| HConF | 0.9569731 | 0.61716 |
| HMVECLBl | 0.9587932 | 0.61782 |
| HPAEC | 0.9564879 | 0.61924 |
| HEEpiC | 0.9612328 | 0.63428 |
| HMVECdBlAd | 0.9525215 | 0.63470 |
| NHDFneo | 0.9540553 | 0.63598 |
| SKNMC | 0.9452529 | 0.63638 |
| HVMF | 0.9484731 | 0.63886 |
| RWPE1 | 0.9484651 | 0.63954 |
| HCPEpiC | 0.9545435 | 0.64574 |
| HCFaa | 0.9488983 | 0.65342 |
| HMVECLLy | 0.9423663 | 0.65718 |
| Medullo | 0.9346629 | 0.66076 |
| HTR8svn | 0.9380552 | 0.66208 |
| HRCEpiC | 0.9482572 | 0.66368 |
| 8988T | 0.9172328 | 0.66404 |
| HMVECdLyAd | 0.9295804 | 0.68424 |
| NT2D1 | 0.9394375 | 0.68498 |
| HMVECdAd | 0.9271150 | 0.68992 |
| Myometr | 0.9319920 | 0.69036 |
| HCM | 0.9374798 | 0.69192 |
| H9ES | 0.9183105 | 0.69932 |
| HMVECdLyNeo | 0.9246511 | 0.70060 |
| AoAF | 0.9287041 | 0.70164 |
| HSMM | 0.9372731 | 0.70684 |
| Hepatocytes | 0.8979390 | 0.70790 |
| PanIsletD | 0.9295360 | 0.71148 |
| SKMC | 0.9297053 | 0.71390 |
| BJ | 0.9234166 | 0.71918 |
| HAEpiC | 0.9242816 | 0.72014 |
| HCF | 0.9176675 | 0.72550 |
| UrotheliaUT189 | 0.9094451 | 0.72582 |
| HAsp | 0.9219410 | 0.72750 |
| HMVECdNeo | 0.9128062 | 0.72864 |
| HPdLF | 0.9168099 | 0.72986 |
| Stellate | 0.9048675 | 0.73208 |
| HPAF | 0.9193953 | 0.73504 |
| LNCaP | 0.9029141 | 0.74480 |
| ProgFib | 0.9028887 | 0.74644 |
| HSMMtube | 0.9166038 | 0.75122 |
| HFF | 0.9145690 | 0.75710 |
| Huh7 | 0.8830687 | 0.75906 |
| NHEK | 0.9049486 | 0.75944 |
| FibroP | 0.9217062 | 0.76298 |
| WERIRb1 | 0.9017899 | 0.76538 |
| HMEC | 0.9004129 | 0.76736 |
| HFFMyc | 0.9109826 | 0.76864 |
| AG04450 | 0.8996808 | 0.76998 |
| WI38 | 0.8995038 | 0.77076 |
| Caco2 | 0.8213428 | 0.77452 |
| H1hESC | 0.8689722 | 0.77956 |
| PrEC | 0.9114687 | 0.78644 |
| HBMEC | 0.9064453 | 0.78714 |
| Osteobl | 0.8791599 | 0.80064 |
| HNPCEpiC | 0.9063669 | 0.80070 |
| pHTE | 0.8780302 | 0.80440 |
| AG09309 | 0.8980409 | 0.80616 |
| HRPEpiC | 0.9013277 | 0.80804 |
| HRE | 0.8988425 | 0.80928 |
| iPS | 0.8327108 | 0.81412 |
| PanIslets | 0.8639467 | 0.81572 |
| HIPEpiC | 0.8964186 | 0.81626 |
| NHLF | 0.8860084 | 0.82956 |
| SKNSHRA | 0.8192076 | 0.83230 |
| AG10803 | 0.8690424 | 0.84136 |
| NHA | 0.8773012 | 0.84370 |
| Chorion | 0.8101912 | 0.84614 |
| Melano | 0.8911340 | 0.84616 |
| AG04449 | 0.8676224 | 0.85078 |
| Fibrobl | 0.8495438 | 0.85942 |
| H7hESC | 0.8963552 | 0.85952 |
| HSMMemb | 0.8504605 | 0.86094 |
| HAc | 0.8687073 | 0.87120 |
| HAh | 0.8744660 | 0.87492 |
| BE2C | 0.8224920 | 0.87950 |

## Plasma lipoproteins

| DHS sample | fold enrichment | p value |
|---|---|---|
| HRCEpiC | 1.4207784 | 0.00128 |
| RPTEC | 1.3567232 | 0.00382 |
| HRE | 1.3329167 | 0.00684 |
| HL60 | 1.4540500 | 0.01392 |
| SAEC | 1.2559792 | 0.02720 |
| HEEpiC | 1.2387831 | 0.03422 |
| HTR8svn | 1.2885474 | 0.03574 |
| PrEC | 1.2254498 | 0.04092 |
| HepG2 | 1.2973321 | 0.05026 |
| HeLaS3 | 1.3012976 | 0.05032 |
| HCFaa | 1.2331265 | 0.05474 |
| HCT116 | 1.2752720 | 0.06604 |
| HPF | 1.2292196 | 0.06962 |
| NHA | 1.2093426 | 0.06974 |
| HIPEpiC | 1.1975177 | 0.07224 |
| HNPCEpiC | 1.1898334 | 0.07382 |
| HCM | 1.1962335 | 0.07408 |
| HRGEC | 1.2114874 | 0.07754 |
| HPAF | 1.1957083 | 0.08196 |
| HCPEpiC | 1.1872059 | 0.08344 |
| HAh | 1.1697694 | 0.08788 |
| HGF | 1.2126138 | 0.09038 |
| HeLaS3IFNa4h | 1.2651849 | 0.09142 |
| HMVECLBl | 1.1980412 | 0.09240 |
| WI38 | 1.1987969 | 0.09742 |
| HVMF | 1.2011194 | 0.10198 |
| HMF | 1.1792237 | 0.10280 |
| NHLF | 1.1755186 | 0.10302 |
| MonocytesCD14RO01746 | 1.2381313 | 0.10692 |
| A549 | 1.2163593 | 0.11212 |
| IshikawaEstradiol | 1.1983964 | 0.11472 |
| HPDE6E6E7 | 1.2008404 | 0.11486 |
| PANC1 | 1.1994691 | 0.11620 |
| NHEK | 1.1773735 | 0.11946 |
| HCF | 1.1735760 | 0.11992 |
| HAc | 1.1585760 | 0.12148 |
| SKMC | 1.1618120 | 0.12494 |
| AG04450 | 1.1739598 | 0.12712 |
| HMVECdBlAd | 1.1681871 | 0.12902 |
| BJ | 1.1673898 | 0.13136 |
| HConF | 1.1652627 | 0.13168 |
| HRPEpiC | 1.1485267 | 0.13714 |
| HPdLF | 1.1642647 | 0.14034 |
| HBMEC | 1.1472570 | 0.14106 |
| HPAEC | 1.1663358 | 0.14216 |
| HAEpiC | 1.1542930 | 0.14246 |
| HMVECdLyNeo | 1.1600283 | 0.15326 |
| UrotheliaUT189 | 1.1685044 | 0.15506 |
| HMVECdBlNeo | 1.1502034 | 0.15626 |
| HAsp | 1.1464838 | 0.16138 |
| AG09309 | 1.1357599 | 0.16202 |
| AG04449 | 1.1381599 | 0.16890 |
| AoSMC | 1.1262223 | 0.16950 |
| HMVECdAd | 1.1494650 | 0.17646 |
| Urothelia | 1.1514979 | 0.17868 |
| AG09319 | 1.1406735 | 0.18116 |
| GM12865 | 1.1594855 | 0.18296 |
| AoAF | 1.1334626 | 0.18744 |
| HFFMyc | 1.1198738 | 0.19008 |
| Jurkat | 1.1230710 | 0.20854 |
| H7hESC | 1.0881931 | 0.21010 |
| RWPE1 | 1.1239702 | 0.21296 |
| IshikawaTamoxifen | 1.1310859 | 0.21476 |
| GM12864 | 1.1391695 | 0.21876 |
| FibroP | 1.0947953 | 0.22274 |
| Stellate | 1.1256540 | 0.22392 |
| HMVECdNeo | 1.1171862 | 0.22652 |
| K562 | 1.1382406 | 0.22948 |
| HSMMemb | 1.1141402 | 0.23126 |
| PanIsletD | 1.0944575 | 0.24958 |
| HFF | 1.0927238 | 0.25300 |
| HMVECdLyAd | 1.1039618 | 0.25398 |
| NB4 | 1.1112873 | 0.25534 |
| HMVECLLy | 1.0998746 | 0.25660 |
| Th2 | 1.1186217 | 0.26340 |
| GM12878 | 1.1125663 | 0.26868 |
| WERIRb1 | 1.0860275 | 0.28224 |
| AG10803 | 1.0838706 | 0.28932 |
| Gliobla | 1.0913980 | 0.30276 |
| CMK | 1.0966146 | 0.30424 |
| GM06990 | 1.0902572 | 0.32228 |
| Melano | 1.0543449 | 0.32516 |
| MCF7 | 1.0630930 | 0.34076 |
| BE2C | 1.0663928 | 0.34286 |
| NHDFneo | 1.0547226 | 0.35212 |
| GM19240 | 1.0584610 | 0.35910 |
| GM18507 | 1.0607784 | 0.36124 |
| Myometr | 1.0490379 | 0.36234 |
| NHDFAd | 1.0477908 | 0.36594 |
| HSMM | 1.0387988 | 0.37868 |
| SKNMC | 1.0440352 | 0.38676 |
| Huh7 | 1.0442067 | 0.39558 |
| HUVEC | 1.0373898 | 0.39804 |
| NT2D1 | 1.0293148 | 0.40290 |
| Huh7.5 | 1.0382801 | 0.40840 |
| MCF7Hypoxia | 1.0331130 | 0.41420 |
| CD20 | 1.0321237 | 0.42574 |
| HSMMtube | 1.0249815 | 0.42626 |
| HMEC | 1.0195926 | 0.44184 |
| GM19238 | 1.0219010 | 0.44360 |
| Caco2 | 1.0188823 | 0.44872 |
| ProgFib | 1.0049546 | 0.47796 |
| Medullo | 1.0060644 | 0.48128 |
| GM19239 | 0.9987049 | 0.48778 |
| Th1 | 1.0035983 | 0.49376 |
| GM12891 | 0.9946446 | 0.49924 |
| Fibrobl | 0.9953479 | 0.50980 |
| Th0 | 0.9929257 | 0.51446 |
| CD34Mobilized | 0.9827047 | 0.53548 |
| Hepatocytes | 0.9732623 | 0.53758 |
| Osteobl | 0.9827785 | 0.54094 |
| CLL | 0.9689621 | 0.55408 |
| GM12892 | 0.9631964 | 0.56390 |
| PanIslets | 0.9571808 | 0.59330 |
| 8988T | 0.9314760 | 0.61712 |
| LNCaPAndrogen | 0.9335613 | 0.62838 |
| pHTE | 0.9396846 | 0.63878 |
| H9ES | 0.9288432 | 0.64196 |
| SKNSHRA | 0.8908716 | 0.68510 |
| iPS | 0.8887016 | 0.69094 |
| LNCaP | 0.8985032 | 0.72762 |
| Chorion | 0.8660623 | 0.73152 |
| H1hESC | 0.8614516 | 0.76378 |
| T47D | 0.7946486 | 0.85718 |

## Fibrinogen

| DHS sample | fold enrichment | p value |
|---|---|---|
| MonocytesCD14RO01746 | 1.3888473 | 0.01138 |
| HVMF | 1.2768337 | 0.01832 |
| HCFaa | 1.2433755 | 0.02014 |
| Th2 | 1.3197244 | 0.02550 |
| AG09309 | 1.1935375 | 0.04100 |
| HAsp | 1.1964981 | 0.05196 |
| HIPEpiC | 1.1811094 | 0.05386 |
| HPAF | 1.1835600 | 0.05404 |
| BJ | 1.1961658 | 0.05418 |
| WI38 | 1.2035630 | 0.05452 |
| HPAEC | 1.2047279 | 0.05494 |
| HMF | 1.1809109 | 0.06014 |
| HAEpiC | 1.1887247 | 0.06100 |
| AoAF | 1.1872960 | 0.06180 |
| HCF | 1.1767491 | 0.06930 |
| HPdLF | 1.1809713 | 0.07226 |
| HBMEC | 1.1633219 | 0.07250 |
| HNPCEpiC | 1.1556249 | 0.07258 |
| HPF | 1.1840986 | 0.07364 |
| AG04449 | 1.1727285 | 0.07462 |
| HGF | 1.1810589 | 0.07958 |
| HCM | 1.1519739 | 0.08252 |
| AG10803 | 1.1694741 | 0.08658 |
| HCPEpiC | 1.1500732 | 0.09116 |
| AG09319 | 1.1628221 | 0.09454 |
| AG04450 | 1.1643546 | 0.09812 |
| SKMC | 1.1489241 | 0.09988 |
| NHDFneo | 1.1547031 | 0.10654 |
| HConF | 1.1505217 | 0.11218 |
| SAEC | 1.1230761 | 0.11532 |
| HRCEpiC | 1.1342062 | 0.11802 |
| AoSMC | 1.1235357 | 0.12008 |
| HMVECdAd | 1.1600749 | 0.12162 |
| NHLF | 1.1286388 | 0.12748 |
| HRGEC | 1.1408335 | 0.13014 |
| HUVEC | 1.1450672 | 0.14318 |
| RPTEC | 1.1128539 | 0.14436 |
| HMVECdNeo | 1.1404980 | 0.14570 |
| HMVECLBl | 1.1295658 | 0.14714 |
| NB4 | 1.1506260 | 0.14814 |
| HMVECdLyNeo | 1.1356256 | 0.15018 |
| HAc | 1.1118439 | 0.15366 |
| HSMM | 1.1084285 | 0.15646 |
| NHDFAd | 1.1117691 | 0.15794 |
| HFF | 1.1132092 | 0.16196 |
| HMVECdBlAd | 1.1226831 | 0.16458 |
| HepG2 | 1.1409192 | 0.16898 |
| HMVECdBlNeo | 1.1160622 | 0.17868 |
| HMVECLLy | 1.1173512 | 0.18756 |
| HFFMyc | 1.1002011 | 0.18862 |
| HEEpiC | 1.0883685 | 0.20138 |
| NHA | 1.0955682 | 0.20286 |
| PrEC | 1.0833115 | 0.20910 |
| Th0 | 1.1114193 | 0.21260 |
| HMVECdLyAd | 1.1060618 | 0.21550 |
| Th1 | 1.1064698 | 0.21584 |
| HTR8svn | 1.1000739 | 0.22554 |
| NHEK | 1.0891462 | 0.23582 |
| K562 | 1.1066310 | 0.23622 |
| HL60 | 1.1137085 | 0.23664 |
| CD34Mobilized | 1.0945371 | 0.24392 |
| UrotheliaUT189 | 1.0822823 | 0.26674 |
| IshikawaTamoxifen | 1.0770091 | 0.28096 |
| GM12878 | 1.0869590 | 0.28110 |
| CMK | 1.0926594 | 0.28230 |
| CD20 | 1.0972741 | 0.28624 |
| HRPEpiC | 1.0554364 | 0.29822 |
| IshikawaEstradiol | 1.0687961 | 0.30022 |
| MCF7 | 1.0660265 | 0.30878 |
| Stellate | 1.0669676 | 0.30970 |
| HRE | 1.0513315 | 0.31374 |
| Huh7.5 | 1.0723400 | 0.31584 |
| Myometr | 1.0568867 | 0.31678 |
| ProgFib | 1.0616453 | 0.31802 |
| HSMMtube | 1.0503973 | 0.32392 |
| RWPE1 | 1.0542995 | 0.33000 |
| HPDE6E6E7 | 1.0582876 | 0.33146 |
| GM12864 | 1.0613158 | 0.33182 |
| HAh | 1.0418983 | 0.34266 |
| GM19239 | 1.0604740 | 0.34566 |
| PANC1 | 1.0466017 | 0.35692 |
| Huh7 | 1.0506495 | 0.35760 |
| HMEC | 1.0424921 | 0.36132 |
| T47D | 1.0513505 | 0.36180 |
| GM12865 | 1.0452084 | 0.36432 |
| GM06990 | 1.0537564 | 0.36476 |
| MCF7Hypoxia | 1.0457416 | 0.37062 |
| GM18507 | 1.0394830 | 0.38518 |
| Urothelia | 1.0351876 | 0.39102 |
| HCT116 | 1.0344554 | 0.39286 |
| PanIsletD | 1.0297135 | 0.39462 |
| GM19240 | 1.0311495 | 0.40560 |
| Jurkat | 1.0263523 | 0.41042 |
| GM12892 | 1.0341311 | 0.41374 |
| GM19238 | 1.0285415 | 0.41606 |
| HeLaS3 | 1.0267454 | 0.41672 |
| Chorion | 1.0238621 | 0.43314 |
| Hepatocytes | 1.0163001 | 0.45000 |
| HSMMemb | 1.0147380 | 0.45190 |
| LNCaPAndrogen | 1.0104529 | 0.46006 |
| GM12891 | 1.0099024 | 0.46520 |
| 8988T | 1.0075493 | 0.47026 |
| A549 | 1.0041830 | 0.47798 |
| LNCaP | 1.0009646 | 0.48632 |
| pHTE | 1.0005875 | 0.49588 |
| Gliobla | 0.9937326 | 0.50314 |
| SKNMC | 0.9934743 | 0.50848 |
| CLL | 0.9874709 | 0.51948 |
| H1hESC | 0.9830485 | 0.52936 |
| Osteobl | 0.9848026 | 0.54142 |
| FibroP | 0.9834840 | 0.56416 |
| Melano | 0.9832412 | 0.56558 |
| NT2D1 | 0.9738720 | 0.58718 |
| PanIslets | 0.9628506 | 0.59706 |
| Medullo | 0.9605829 | 0.60796 |
| Fibrobl | 0.9633442 | 0.60998 |
| Caco2 | 0.9197753 | 0.62618 |
| HeLaS3IFNa4h | 0.9429515 | 0.62754 |
| iPS | 0.9298522 | 0.64966 |
| SKNSHRA | 0.8949957 | 0.71180 |
| H9ES | 0.9134950 | 0.72468 |
| BE2C | 0.8789872 | 0.79602 |
| H7hESC | 0.9273373 | 0.79730 |
| WERIRb1 | 0.8477393 | 0.89870 |

# Vitiligo

| DHS sample | fold enrichment | p value |
|---|---|---|
| Th2 | 1.6703401 | 0.00282 |
| HNPCEpiC | 1.3672928 | 0.00714 |
| NHA | 1.3849488 | 0.00834 |
| HBMEC | 1.3608550 | 0.01118 |
| AG04449 | 1.3841387 | 0.01178 |
| SKMC | 1.3676898 | 0.01230 |
| HPdLF | 1.3775948 | 0.01434 |
| HAc | 1.3116761 | 0.01542 |
| NHLF | 1.3295569 | 0.01568 |
| HMF | 1.3515853 | 0.01804 |
| HAsp | 1.3306772 | 0.02032 |
| HAh | 1.2753518 | 0.02176 |
| GM12865 | 1.4028761 | 0.02226 |
| SAEC | 1.2906127 | 0.02236 |
| HIPEpiC | 1.3060952 | 0.02270 |
| HEEpiC | 1.2834267 | 0.02850 |
| AG04450 | 1.3365178 | 0.02884 |
| AG10803 | 1.3208126 | 0.02952 |
| GM12864 | 1.3916878 | 0.03056 |
| HFFMyc | 1.2847669 | 0.03150 |
| HAEpiC | 1.3070243 | 0.03296 |
| HFF | 1.2895488 | 0.03446 |
| CD20 | 1.4625618 | 0.03610 |
| NHDFneo | 1.2996687 | 0.03686 |
| AoAF | 1.3093343 | 0.03740 |
| BJ | 1.2897363 | 0.03976 |
| HMVECLBl | 1.3032104 | 0.04388 |
| HGF | 1.3076292 | 0.04554 |
| NHDFAd | 1.2530190 | 0.04920 |
| HPF | 1.2831023 | 0.05286 |
| HRGEC | 1.2800996 | 0.05358 |
| HRE | 1.2293360 | 0.05556 |
| RPTEC | 1.2203945 | 0.06170 |
| AG09319 | 1.2720471 | 0.06176 |
| Jurkat | 1.2735974 | 0.06542 |
| HCFaa | 1.2425051 | 0.06938 |
| GM06990 | 1.3456337 | 0.07340 |
| PrEC | 1.2026254 | 0.07342 |
| HCPEpiC | 1.2224022 | 0.07756 |
| HConF | 1.2421287 | 0.07896 |
| CLL | 1.3160337 | 0.08984 |
| HCM | 1.2034205 | 0.09426 |
| Th0 | 1.2590472 | 0.09722 |
| HCT116 | 1.2571555 | 0.10054 |
| MonocytesCD14RO01746 | 1.2906918 | 0.10114 |
| AoSMC | 1.1917864 | 0.10334 |
| HCF | 1.2126909 | 0.11082 |
| AG09309 | 1.1890233 | 0.11220 |
| GM12878 | 1.2510395 | 0.11312 |
| HMVECdBlAd | 1.2100024 | 0.11562 |
| HRCEpiC | 1.1741873 | 0.12528 |
| CD34Mobilized | 1.2218856 | 0.12576 |
| HRPEpiC | 1.1649006 | 0.13208 |
| HMVECdAd | 1.2108436 | 0.13254 |
| HPAF | 1.1778777 | 0.13884 |
| WI38 | 1.1861153 | 0.14462 |
| HMVECdNeo | 1.1908721 | 0.14752 |
| NB4 | 1.2035903 | 0.14814 |
| HVMF | 1.1846681 | 0.14930 |
| HPDE6E6E7 | 1.2003027 | 0.15060 |
| HMVECdBlNeo | 1.1756002 | 0.16178 |
| HL60 | 1.2147334 | 0.16834 |
| HPAEC | 1.1687670 | 0.17218 |
| Th1 | 1.1803037 | 0.17270 |
| GM18507 | 1.1808494 | 0.18220 |
| FibroP | 1.1181230 | 0.20060 |
| PANC1 | 1.1569794 | 0.20438 |
| HMVECLLy | 1.1479272 | 0.20636 |
| HMVECdLyAd | 1.1425745 | 0.21776 |
| NHEK | 1.1289689 | 0.22204 |
| RWPE1 | 1.1264992 | 0.22942 |
| HMVECdLyNeo | 1.1270792 | 0.23956 |
| PanIsletD | 1.1058427 | 0.24966 |
| HSMM | 1.0965426 | 0.25224 |
| Urothelia | 1.1133861 | 0.26882 |
| Huh7 | 1.0990399 | 0.30614 |
| CMK | 1.0993450 | 0.32182 |
| GM19240 | 1.0704172 | 0.34764 |
| Melano | 1.0493726 | 0.35516 |
| Stellate | 1.0656068 | 0.36194 |
| GM19238 | 1.0596530 | 0.38046 |
| UrotheliaUT189 | 1.0513059 | 0.38440 |
| SKNMC | 1.0429463 | 0.38914 |
| HSMMemb | 1.0473971 | 0.39046 |
| A549 | 1.0475983 | 0.39174 |
| IshikawaEstradiol | 1.0451303 | 0.39262 |
| HUVEC | 1.0452349 | 0.39400 |
| IshikawaTamoxifen | 1.0389307 | 0.40746 |
| K562 | 1.0406033 | 0.41068 |
| Myometr | 1.0327727 | 0.41226 |
| HeLaS3 | 1.0211027 | 0.44374 |
| HeLaS3IFNa4h | 1.0175328 | 0.45212 |
| GM19239 | 1.0164253 | 0.45240 |
| Huh7.5 | 1.0113919 | 0.46334 |
| HSMMtube | 1.0099398 | 0.46762 |
| HTR8svn | 1.0107217 | 0.46816 |
| ProgFib | 1.0051845 | 0.47566 |
| BE2C | 1.0040710 | 0.47904 |
| Gliobla | 0.9827158 | 0.51442 |
| MCF7 | 0.9726014 | 0.54130 |
| HMEC | 0.9747008 | 0.54740 |
| PanIslets | 0.9632793 | 0.56734 |
| SKNSHRA | 0.9447170 | 0.56940 |
| GM12892 | 0.9517008 | 0.57410 |
| Fibrobl | 0.9606468 | 0.58178 |
| pHTE | 0.9547690 | 0.58558 |
| WERIRb1 | 0.9561468 | 0.59026 |
| LNCaPAndrogen | 0.9435794 | 0.59230 |
| LNCaP | 0.9474999 | 0.60276 |
| GM12891 | 0.9307141 | 0.60352 |
| 8988T | 0.9230734 | 0.61018 |
| Hepatocytes | 0.9025184 | 0.64134 |
| NT2D1 | 0.9423895 | 0.64532 |
| Chorion | 0.8943648 | 0.65222 |
| MCF7Hypoxia | 0.9081825 | 0.65262 |
| H7hESC | 0.9484883 | 0.67652 |
| T47D | 0.8907924 | 0.67732 |
| Caco2 | 0.8453931 | 0.68044 |
| H9ES | 0.8973062 | 0.69508 |
| Osteobl | 0.8912672 | 0.71768 |
| Medullo | 0.8749821 | 0.73434 |
| HepG2 | 0.8651464 | 0.74320 |
| H1hESC | 0.8519215 | 0.74836 |
| iPS | 0.7757517 | 0.82752 |

## Prostate cancer susceptibility

| DHS sample | fold enrichment | p value |
|---|---|---|
| HCT116 | 1.347990 | 0.00962 |
| HPDE6E6E7 | 1.315525 | 0.00976 |
| Medullo | 1.353337 | 0.01052 |
| LNCaPAndrogen | 1.363721 | 0.01110 |
| HTR8svn | 1.309584 | 0.01312 |
| HUVEC | 1.286592 | 0.01470 |
| RWPE1 | 1.278800 | 0.01542 |
| T47D | 1.363389 | 0.01576 |
| CMK | 1.359948 | 0.01944 |
| HepG2 | 1.311218 | 0.02000 |
| HCF | 1.248065 | 0.02036 |
| WERIRb1 | 1.254907 | 0.02090 |
| Urothelia | 1.294911 | 0.02118 |
| UrotheliaUT189 | 1.299323 | 0.02158 |
| AG04450 | 1.250120 | 0.02422 |
| GM19239 | 1.335449 | 0.02578 |
| WI38 | 1.243475 | 0.02638 |
| HSMMemb | 1.263206 | 0.02684 |
| Myometr | 1.239355 | 0.02758 |
| SKMC | 1.215228 | 0.02878 |
| NHDFneo | 1.225711 | 0.02890 |
| HeLaS3 | 1.277135 | 0.03024 |
| H9ES | 1.274165 | 0.03100 |
| HFF | 1.215058 | 0.03134 |
| NT2D1 | 1.203469 | 0.03190 |
| HPF | 1.225524 | 0.03230 |
| MCF7 | 1.278576 | 0.03264 |
| MCF7Hypoxia | 1.331553 | 0.03328 |
| Melano | 1.183942 | 0.03360 |
| LNCaP | 1.247903 | 0.03488 |
| FibroP | 1.186133 | 0.03570 |
| HFFMyc | 1.196342 | 0.03582 |
| HPAEC | 1.224834 | 0.03616 |
| HConF | 1.217379 | 0.03644 |
| HMVECdLyNeo | 1.231885 | 0.03760 |
| AoAF | 1.212316 | 0.03818 |
| HCFaa | 1.190551 | 0.04368 |
| A549 | 1.253387 | 0.04444 |
| Stellate | 1.246216 | 0.04456 |
| HGF | 1.213052 | 0.04474 |
| AG09319 | 1.210958 | 0.04624 |
| PanIslets | 1.245416 | 0.04740 |
| HCM | 1.181461 | 0.04896 |
| HEEpiC | 1.163627 | 0.04988 |
| HMF | 1.185939 | 0.05080 |
| H1hESC | 1.263697 | 0.05292 |
| ProgFib | 1.224873 | 0.05304 |
| HMEC | 1.204878 | 0.05542 |
| HMVECdNeo | 1.208728 | 0.05612 |
| GM19238 | 1.252548 | 0.05622 |
| GM18507 | 1.237401 | 0.05754 |
| Hepatocytes | 1.274921 | 0.05902 |
| Th0 | 1.228177 | 0.05958 |
| HSMM | 1.161583 | 0.05970 |
| AoSMC | 1.161314 | 0.05998 |
| iPS | 1.290082 | 0.06044 |
| PrEC | 1.155218 | 0.06050 |
| HMVECdBlNeo | 1.197463 | 0.06072 |
| HeLaS3IFNa4h | 1.249708 | 0.06166 |
| CLL | 1.260671 | 0.06188 |
| HMVECdLyAd | 1.205033 | 0.06188 |
| SAEC | 1.151721 | 0.06440 |
| GM19240 | 1.214595 | 0.06570 |
| HPAF | 1.169601 | 0.06634 |
| NHDFAd | 1.162783 | 0.06696 |
| pHTE | 1.205707 | 0.06740 |
| SKNSHRA | 1.270536 | 0.06798 |
| PANC1 | 1.205300 | 0.07060 |
| AG10803 | 1.180797 | 0.07088 |
| HSMMtube | 1.165457 | 0.07188 |
| HPdLF | 1.175305 | 0.07316 |
| PanIsletD | 1.166493 | 0.07336 |
| GM12864 | 1.218500 | 0.07378 |
| HMVECLLy | 1.188815 | 0.07536 |
| Chorion | 1.270474 | 0.07602 |
| Caco2 | 1.336037 | 0.07648 |
| CD34Mobilized | 1.200666 | 0.07702 |
| GM12891 | 1.248929 | 0.07912 |
| HRGEC | 1.169276 | 0.07942 |
| NHEK | 1.173140 | 0.08108 |
| HMVECdAd | 1.186738 | 0.08196 |
| Huh7 | 1.209106 | 0.08340 |
| Fibrobl | 1.184648 | 0.08350 |
| RPTEC | 1.140000 | 0.08358 |
| 8988T | 1.251852 | 0.08464 |
| HVMF | 1.173088 | 0.08492 |
| CD20 | 1.244170 | 0.08510 |
| GM06990 | 1.233089 | 0.08560 |
| H7hESC | 1.118517 | 0.08580 |
| HRCEpiC | 1.145854 | 0.08864 |
| HMVECdBlAd | 1.168042 | 0.08884 |
| HMVECLBl | 1.163447 | 0.08992 |
| Osteobl | 1.187234 | 0.09010 |
| Huh7.5 | 1.213537 | 0.09130 |
| GM12878 | 1.203836 | 0.09372 |
| BJ | 1.157787 | 0.09696 |
| BE2C | 1.180121 | 0.10016 |
| GM12865 | 1.176401 | 0.10662 |
| GM12892 | 1.213588 | 0.10944 |
| Th1 | 1.170112 | 0.11302 |
| IshikawaTamoxifen | 1.161640 | 0.11310 |
| Jurkat | 1.153700 | 0.11794 |
| AG09309 | 1.128299 | 0.11938 |
| HRE | 1.121622 | 0.12336 |
| HNPCEpiC | 1.110442 | 0.13382 |
| HCPEpiC | 1.110333 | 0.15030 |
| SKNMC | 1.130705 | 0.15066 |
| AG04449 | 1.119043 | 0.15256 |
| Gliobla | 1.155195 | 0.15452 |
| HAEpiC | 1.116890 | 0.15580 |
| MonocytesCD14RO01746 | 1.152476 | 0.17210 |
| NB4 | 1.130292 | 0.18002 |
| Th2 | 1.147380 | 0.18138 |
| K562 | 1.133956 | 0.19032 |
| HAc | 1.081848 | 0.20742 |
| HRPEpiC | 1.079598 | 0.21476 |
| NHLF | 1.082310 | 0.21860 |
| HIPEpiC | 1.079872 | 0.22374 |
| HL60 | 1.105667 | 0.24818 |
| IshikawaEstradiol | 1.084227 | 0.24922 |
| HAh | 1.064731 | 0.25114 |
| HBMEC | 1.068434 | 0.25372 |
| NHA | 1.057008 | 0.29540 |
| HAsp | 1.024488 | 0.40516 |

# Lung function

| DHS sample | fold enrichment | p value |
|---|---|---|
| BJ | 1.4616745 | 0.00198 |
| HCFaa | 1.4154253 | 0.00278 |
| AG09309 | 1.3910721 | 0.00440 |
| HGF | 1.4529570 | 0.00464 |
| HNPCEpiC | 1.3489092 | 0.00664 |
| NHLF | 1.3596351 | 0.00712 |
| HCPEpiC | 1.3606022 | 0.00762 |
| HFFMyc | 1.3617001 | 0.00770 |
| HIPEpiC | 1.3482984 | 0.00776 |
| AG04449 | 1.3801964 | 0.00924 |
| HBMEC | 1.3328578 | 0.01034 |
| HPAEC | 1.3918594 | 0.01120 |
| HAh | 1.3046862 | 0.01166 |
| NHA | 1.3397654 | 0.01170 |
| HAsp | 1.3457037 | 0.01182 |
| AG09319 | 1.3874714 | 0.01198 |
| AoAF | 1.3655699 | 0.01210 |
| HRGEC | 1.3645921 | 0.01254 |
| SAEC | 1.2959786 | 0.01318 |
| HMVECLBl | 1.3654426 | 0.01346 |
| AoSMC | 1.3163383 | 0.01390 |
| HEEpiC | 1.2935483 | 0.01504 |
| HAc | 1.2926268 | 0.02144 |
| PrEC | 1.2688500 | 0.02310 |
| HRPEpiC | 1.2744820 | 0.02452 |
| AG10803 | 1.3238988 | 0.02544 |
| NHDFAd | 1.2897211 | 0.02700 |
| HRCEpiC | 1.2762979 | 0.02758 |
| HPdLF | 1.3199380 | 0.02890 |
| HPAF | 1.2835004 | 0.03136 |
| HPDE6E6E7 | 1.3421321 | 0.03182 |
| HConF | 1.2987085 | 0.03384 |
| NHDFneo | 1.2940856 | 0.03400 |
| HTR8svn | 1.3298758 | 0.03422 |
| HFF | 1.2807585 | 0.03462 |
| HPF | 1.2885934 | 0.04116 |
| HCM | 1.2508914 | 0.04484 |
| IshikawaEstradiol | 1.2991799 | 0.04526 |
| RPTEC | 1.2301248 | 0.04546 |
| SKMC | 1.2467750 | 0.04986 |
| HVMF | 1.2801796 | 0.05016 |
| HAEpiC | 1.2596777 | 0.05064 |
| HMVECdBlNeo | 1.2736047 | 0.05472 |
| AG04450 | 1.2661450 | 0.05582 |
| WI38 | 1.2681190 | 0.05632 |
| PANC1 | 1.3026109 | 0.05666 |
| HMF | 1.2370640 | 0.05872 |
| HCF | 1.2547184 | 0.05930 |
| HRE | 1.2084990 | 0.06692 |
| HMVECdBlAd | 1.2403144 | 0.07204 |
| IshikawaTamoxifen | 1.2521743 | 0.07528 |
| HMVECdLyNeo | 1.2448257 | 0.07766 |
| HMVECdNeo | 1.2367341 | 0.08694 |
| HUVEC | 1.2352094 | 0.08928 |
| HMVECdLyAd | 1.2368555 | 0.08990 |
| HSMMemb | 1.2305045 | 0.09148 |
| HMVECdAd | 1.2401921 | 0.09184 |
| NB4 | 1.2409395 | 0.09600 |
| RWPE1 | 1.2121468 | 0.10226 |
| CLL | 1.2681014 | 0.10424 |
| HMVECLLy | 1.2165379 | 0.10588 |
| A549 | 1.2406948 | 0.10730 |
| HSMM | 1.1739629 | 0.10766 |
| HeLaS3 | 1.2261409 | 0.11054 |
| CD20 | 1.2679315 | 0.12114 |
| CD34Mobilized | 1.2025206 | 0.12812 |
| HeLaS3IFNa4h | 1.2291712 | 0.12948 |
| Stellate | 1.2017732 | 0.13286 |
| NHEK | 1.1669662 | 0.15184 |
| Huh7 | 1.1989062 | 0.15218 |
| HepG2 | 1.1919277 | 0.15460 |
| CMK | 1.2181016 | 0.15854 |
| GM12864 | 1.1818445 | 0.15998 |
| HCT116 | 1.1761551 | 0.16416 |
| K562 | 1.1840847 | 0.17218 |
| FibroP | 1.1237196 | 0.18436 |
| Gliobla | 1.1722210 | 0.18932 |
| GM18507 | 1.1648248 | 0.19058 |
| Th2 | 1.1789812 | 0.19450 |
| Myometr | 1.1359291 | 0.19716 |
| MonocytesCD14RO01746 | 1.1599090 | 0.21762 |
| T47D | 1.1485228 | 0.22078 |
| HL60 | 1.1528197 | 0.22514 |
| NT2D1 | 1.0980098 | 0.22868 |
| GM06990 | 1.1452707 | 0.23608 |
| PanIsletD | 1.1074460 | 0.23666 |
| ProgFib | 1.1215351 | 0.23850 |
| GM12865 | 1.1202474 | 0.24270 |
| Jurkat | 1.1063151 | 0.24974 |
| H7hESC | 1.0661595 | 0.26890 |
| Th0 | 1.1058785 | 0.27368 |
| HSMMtube | 1.0879191 | 0.27422 |
| GM12878 | 1.1098588 | 0.27732 |
| SKNSHRA | 1.1197454 | 0.28048 |
| Huh7.5 | 1.1042159 | 0.29442 |
| 8988T | 1.1097303 | 0.30330 |
| MCF7Hypoxia | 1.0904105 | 0.30806 |
| Melano | 1.0614587 | 0.31114 |
| Urothelia | 1.0817009 | 0.31540 |
| BE2C | 1.0794716 | 0.31646 |
| SKNMC | 1.0535505 | 0.35576 |
| MCF7 | 1.0551287 | 0.35856 |
| GM19238 | 1.0646753 | 0.35962 |
| Th1 | 1.0521836 | 0.38142 |
| pHTE | 1.0440126 | 0.38860 |
| GM19240 | 1.0424149 | 0.39644 |
| GM12891 | 1.0434946 | 0.40714 |
| Fibrobl | 1.0342039 | 0.41088 |
| UrotheliaUT189 | 1.0355430 | 0.41122 |
| GM19239 | 1.0281170 | 0.43202 |
| GM12892 | 1.0221492 | 0.44598 |
| Osteobl | 1.0182860 | 0.45198 |
| H9ES | 1.0019800 | 0.47792 |
| WERIRb1 | 1.0037219 | 0.47934 |
| LNCaPAndrogen | 1.0032903 | 0.48108 |
| PanIslets | 1.0020971 | 0.48474 |
| H1hESC | 0.9828057 | 0.51614 |
| HMEC | 0.9854238 | 0.52788 |
| Caco2 | 0.9552515 | 0.53880 |
| iPS | 0.9579571 | 0.55952 |
| LNCaP | 0.9616845 | 0.58036 |
| Hepatocytes | 0.9353698 | 0.60300 |
| Chorion | 0.9308234 | 0.60988 |
| Medullo | 0.8566011 | 0.78114 |

# Age at menopause

| DHS sample | fold enrichment | p value |
|---|---|---|
| SKNMC | 1.4245916 | 0.00212 |
| K562 | 1.3178255 | 0.02744 |
| PANC1 | 1.2976393 | 0.03026 |
| HL60 | 1.2666486 | 0.06156 |
| HEEpiC | 1.1688586 | 0.06936 |
| HPDE6E6E7 | 1.1855733 | 0.10394 |
| GM12878 | 1.1943421 | 0.11318 |
| CMK | 1.1944434 | 0.13096 |
| A549 | 1.1693299 | 0.14252 |
| SAEC | 1.1132998 | 0.15594 |
| RWPE1 | 1.1357010 | 0.16054 |
| HeLaS3 | 1.1462761 | 0.17294 |
| GM12864 | 1.1374883 | 0.18584 |
| GM12865 | 1.1291999 | 0.19182 |
| Huh7 | 1.1335418 | 0.19748 |
| HRGEC | 1.1076790 | 0.19938 |
| GM06990 | 1.1468524 | 0.20058 |
| CD20 | 1.1524718 | 0.20306 |
| Th2 | 1.1316984 | 0.21304 |
| HIPEpiC | 1.0900140 | 0.21654 |
| HAEpiC | 1.0980907 | 0.21770 |
| HeLaS3IFNa4h | 1.1272681 | 0.22440 |
| CLL | 1.1317756 | 0.22444 |
| RPTEC | 1.0780755 | 0.24028 |
| H7hESC | 1.0661819 | 0.24208 |
| WERIRb1 | 1.0926773 | 0.24294 |
| HepG2 | 1.1043724 | 0.24584 |
| HTR8svn | 1.0928884 | 0.25258 |
| HRCEpiC | 1.0745905 | 0.25826 |
| IshikawaTamoxifen | 1.0911071 | 0.25974 |
| AoSMC | 1.0719306 | 0.26344 |
| AG04450 | 1.0803874 | 0.27474 |
| Gliobla | 1.0926564 | 0.27652 |
| NB4 | 1.0852003 | 0.27982 |
| Huh7.5 | 1.0875509 | 0.28620 |
| PrEC | 1.0600890 | 0.28750 |
| HMVECdLyAd | 1.0752936 | 0.28970 |
| GM18507 | 1.0822513 | 0.28974 |
| HPAEC | 1.0719210 | 0.29182 |
| HVMF | 1.0713053 | 0.29546 |
| HCT116 | 1.0747392 | 0.30050 |
| HRE | 1.0565829 | 0.30554 |
| HCFaa | 1.0599489 | 0.30832 |
| HMVECdNeo | 1.0544655 | 0.34070 |
| Stellate | 1.0603353 | 0.34076 |
| WI38 | 1.0553998 | 0.34118 |
| HConF | 1.0515103 | 0.34258 |
| IshikawaEstradiol | 1.0524902 | 0.34672 |
| NHEK | 1.0483332 | 0.35158 |
| HMVECdBlNeo | 1.0465155 | 0.35804 |
| LNCaPAndrogen | 1.0526073 | 0.36136 |
| GM19239 | 1.0546337 | 0.36378 |
| GM19238 | 1.0509422 | 0.36932 |
| HNPCEpiC | 1.0367777 | 0.37046 |
| AG04449 | 1.0409952 | 0.37386 |
| Urothelia | 1.0429124 | 0.37934 |
| HUVEC | 1.0347184 | 0.38912 |
| HGF | 1.0327743 | 0.40068 |
| HCPEpiC | 1.0277774 | 0.40400 |
| CD34Mobilized | 1.0267056 | 0.41920 |
| HMVECLLy | 1.0254328 | 0.42014 |
| Myometr | 1.0227677 | 0.42288 |
| HMVECdBlAd | 1.0221633 | 0.42632 |
| H9ES | 1.0232491 | 0.42778 |
| HMVECdAd | 1.0226574 | 0.42892 |
| Jurkat | 1.0206079 | 0.43302 |
| BJ | 1.0209425 | 0.43420 |
| AG09319 | 1.0206202 | 0.43494 |
| MCF7Hypoxia | 1.0201835 | 0.44066 |
| HFF | 1.0145972 | 0.44954 |
| NT2D1 | 1.0097389 | 0.45670 |
| HPAF | 1.0112677 | 0.45998 |
| HMVECdLyNeo | 1.0103326 | 0.46248 |
| HAh | 1.0075771 | 0.46968 |
| MonocytesCD14RO01746 | 1.0086291 | 0.47168 |
| HRPEpiC | 1.0057586 | 0.47176 |
| HFFMyc | 1.0018508 | 0.49382 |
| HMF | 0.9993636 | 0.49412 |
| HPdLF | 0.9994964 | 0.49746 |
| Hepatocytes | 0.9952651 | 0.49832 |
| UrotheliaUT189 | 0.9982042 | 0.49834 |
| GM19240 | 0.9952866 | 0.50828 |
| MCF7 | 0.9920764 | 0.51056 |
| HSMMemb | 0.9950228 | 0.51062 |
| HCF | 0.9929377 | 0.51358 |
| HBMEC | 0.9940792 | 0.51554 |
| HAsp | 0.9917091 | 0.51972 |
| Medullo | 0.9896793 | 0.52210 |
| SKMC | 0.9903877 | 0.52480 |
| HAc | 0.9856952 | 0.54258 |
| HPF | 0.9825606 | 0.54322 |
| ProgFib | 0.9793611 | 0.55002 |
| HMVECLBl | 0.9802351 | 0.55764 |
| AG09309 | 0.9810241 | 0.55968 |
| T47D | 0.9646784 | 0.56838 |
| GM12891 | 0.9620672 | 0.57654 |
| PanIsletD | 0.9754005 | 0.57764 |
| AoAF | 0.9725290 | 0.57834 |
| HCM | 0.9744534 | 0.58174 |
| SKNSHRA | 0.9515787 | 0.58584 |
| BE2C | 0.9630449 | 0.58624 |
| Th0 | 0.9561688 | 0.61740 |
| AG10803 | 0.9557947 | 0.62318 |
| NHLF | 0.9591183 | 0.62724 |
| 8988T | 0.9334762 | 0.62978 |
| H1hESC | 0.9397592 | 0.63092 |
| FibroP | 0.9600068 | 0.64584 |
| LNCaP | 0.9440137 | 0.64658 |
| NHDFAd | 0.9499466 | 0.65618 |
| NHA | 0.9475190 | 0.66316 |
| PanIslets | 0.9351330 | 0.66512 |
| pHTE | 0.9216156 | 0.71052 |
| GM12892 | 0.8968861 | 0.71582 |
| HSMM | 0.9294177 | 0.73132 |
| NHDFneo | 0.9171912 | 0.73134 |
| Chorion | 0.8745298 | 0.74706 |
| Osteobl | 0.9038897 | 0.75516 |
| HMEC | 0.8928707 | 0.79142 |
| Th1 | 0.8856723 | 0.79170 |
| Caco2 | 0.8064328 | 0.79394 |
| iPS | 0.8387850 | 0.81164 |
| Fibrobl | 0.8756430 | 0.82420 |
| Melano | 0.9025647 | 0.82732 |
| HSMMtube | 0.8826263 | 0.83694 |

# Heart rate

| DHS sample | fold enrichment | p value |
|---|---|---|
| HMVECdLyAd | 1.2479432 | 0.04010 |
| HRGEC | 1.2149967 | 0.05424 |
| HMVECLLy | 1.1930685 | 0.07832 |
| HMVECdLyNeo | 1.1931939 | 0.08230 |
| HAEpiC | 1.1748944 | 0.08522 |
| HMVECLBl | 1.1774754 | 0.09544 |
| HMVECdBlNeo | 1.1772981 | 0.09914 |
| HVMF | 1.1772280 | 0.09944 |
| HPAEC | 1.1813375 | 0.10056 |
| HIPEpiC | 1.1511919 | 0.10306 |
| PANC1 | 1.1866975 | 0.10598 |
| NT2D1 | 1.1409415 | 0.11010 |
| HCPEpiC | 1.1402228 | 0.11654 |
| HMVECdBlAd | 1.1579250 | 0.11898 |
| HMVECdAd | 1.1703352 | 0.11990 |
| HMVECdNeo | 1.1620241 | 0.12254 |
| HCFaa | 1.1345673 | 0.13650 |
| HBMEC | 1.1201429 | 0.14728 |
| HAsp | 1.1133904 | 0.17250 |
| HPAF | 1.0950834 | 0.21736 |
| AoSMC | 1.0824703 | 0.23880 |
| HCF | 1.0817715 | 0.25840 |
| SKNMC | 1.0771350 | 0.26950 |
| HCM | 1.0688338 | 0.28258 |
| WI38 | 1.0766285 | 0.28548 |
| AoAF | 1.0689553 | 0.29540 |
| K562 | 1.0743966 | 0.31778 |
| AG04450 | 1.0581679 | 0.33060 |
| HRPEpiC | 1.0445985 | 0.33456 |
| NHA | 1.0498927 | 0.33900 |
| HUVEC | 1.0536344 | 0.33974 |
| HNPCEpiC | 1.0437409 | 0.34210 |
| NHLF | 1.0434183 | 0.35638 |
| HPF | 1.0448366 | 0.35878 |
| HPdLF | 1.0358355 | 0.39160 |
| Huh7 | 1.0383754 | 0.39546 |
| HGF | 1.0327592 | 0.39896 |
| HeLaS3 | 1.0338196 | 0.40156 |
| A549 | 1.0261596 | 0.41794 |
| Huh7.5 | 1.0193175 | 0.44044 |
| AG09309 | 1.0154728 | 0.44758 |
| BJ | 1.0090860 | 0.46526 |
| HMF | 1.0055808 | 0.47562 |
| AG09319 | 0.9971055 | 0.50226 |
| HeLaS3IFNa4h | 0.9865648 | 0.51770 |
| HepG2 | 0.9859758 | 0.52358 |
| SKMC | 0.9904535 | 0.52576 |
| HFFMyc | 0.9911307 | 0.52620 |
| H7hESC | 0.9910808 | 0.53280 |
| T47D | 0.9776787 | 0.53896 |
| HAh | 0.9855213 | 0.55144 |
| HL60 | 0.9681550 | 0.56138 |
| CMK | 0.9658265 | 0.56146 |
| HAc | 0.9789617 | 0.56432 |
| HFF | 0.9776125 | 0.56918 |
| Myometr | 0.9700564 | 0.58814 |
| HSMM | 0.9703691 | 0.60504 |
| HPDE6E6E7 | 0.9596562 | 0.60576 |
| HTR8svn | 0.9564732 | 0.61020 |
| HEEpiC | 0.9627793 | 0.63206 |
| Stellate | 0.9411897 | 0.64552 |
| MonocytesCD14RO01746 | 0.9212755 | 0.66378 |
| HConF | 0.9439433 | 0.66744 |
| SAEC | 0.9491249 | 0.67546 |
| NHDFneo | 0.9389066 | 0.67712 |
| AG04449 | 0.9387986 | 0.68428 |
| MCF7 | 0.9274131 | 0.68800 |
| HSMMtube | 0.9343073 | 0.70620 |
| Caco2 | 0.8522881 | 0.71288 |
| NB4 | 0.9089250 | 0.71364 |
| CD34Mobilized | 0.9126552 | 0.72196 |
| IshikawaTamoxifen | 0.9102020 | 0.73708 |
| AG10803 | 0.9138840 | 0.74454 |
| HRE | 0.9258423 | 0.74564 |
| HSMMemb | 0.9023171 | 0.75366 |
| H9ES | 0.8926593 | 0.76392 |
| 8988T | 0.8596868 | 0.76586 |
| IshikawaEstradiol | 0.9025652 | 0.76716 |
| HRCEpiC | 0.9137360 | 0.77130 |
| H1hESC | 0.8745968 | 0.77478 |
| PanIsletD | 0.9060004 | 0.77786 |
| MCF7Hypoxia | 0.8657736 | 0.78444 |
| LNCaPAndrogen | 0.8676545 | 0.79148 |
| NHDFAd | 0.8995848 | 0.80194 |
| GM12878 | 0.8625157 | 0.80598 |
| LNCaP | 0.8801462 | 0.80778 |
| Gliobla | 0.8566055 | 0.81014 |
| RPTEC | 0.9031716 | 0.81216 |
| GM12891 | 0.8343979 | 0.81218 |
| ProgFib | 0.8693797 | 0.81846 |
| Hepatocytes | 0.8262480 | 0.83250 |
| SKNSHRA | 0.8240147 | 0.84054 |
| Chorion | 0.8053267 | 0.84616 |
| PrEC | 0.8860366 | 0.84896 |
| GM19239 | 0.8191117 | 0.85802 |
| CD20 | 0.8009596 | 0.86350 |
| pHTE | 0.8379009 | 0.87150 |
| iPS | 0.7813728 | 0.87194 |
| GM19238 | 0.8142548 | 0.87276 |
| PanIslets | 0.8202675 | 0.87690 |
| NHEK | 0.8506949 | 0.87704 |
| GM12892 | 0.7853981 | 0.87928 |
| GM18507 | 0.8179310 | 0.88236 |
| Medullo | 0.8135928 | 0.88238 |
| HCT116 | 0.8320193 | 0.88318 |
| RWPE1 | 0.8388860 | 0.89030 |
| BE2C | 0.8231398 | 0.89470 |
| UrotheliaUT189 | 0.7976701 | 0.91628 |
| Melano | 0.8536571 | 0.91740 |
| Urothelia | 0.7973077 | 0.92082 |
| Osteobl | 0.7879469 | 0.92378 |
| FibroP | 0.8383863 | 0.92518 |
| GM12865 | 0.7950495 | 0.92838 |
| Fibrobl | 0.7853646 | 0.93254 |
| GM19240 | 0.7798727 | 0.93382 |
| CLL | 0.7321712 | 0.94020 |
| HMEC | 0.7952853 | 0.94296 |
| Th2 | 0.7326733 | 0.94326 |
| Th0 | 0.7449104 | 0.95218 |
| GM06990 | 0.7029269 | 0.96344 |
| GM12864 | 0.7325582 | 0.96594 |
| Th1 | 0.6993447 | 0.97676 |
| WERIRb1 | 0.7572666 | 0.97730 |
| Jurkat | 0.7103368 | 0.98596 |

# N−glycosylation of immunoglobulin G

| DHS sample | fold enrichment | p value |
|---|---|---|
| Th2 | 1.537846 | 0.00226 |
| AG04450 | 1.316818 | 0.01308 |
| SKMC | 1.284128 | 0.01356 |
| HCM | 1.277838 | 0.01434 |
| HPF | 1.302174 | 0.01686 |
| HCF | 1.297427 | 0.01762 |
| CD20 | 1.435120 | 0.01898 |
| HMVECLLy | 1.314274 | 0.02042 |
| GM06990 | 1.395655 | 0.02076 |
| AoSMC | 1.237821 | 0.02138 |
| HMVECdLyNeo | 1.292030 | 0.02472 |
| GM12864 | 1.324601 | 0.02626 |
| HFFMyc | 1.238307 | 0.02698 |
| GM12865 | 1.304505 | 0.02850 |
| AoAF | 1.261042 | 0.03062 |
| HMVECdLyAd | 1.288946 | 0.03078 |
| HPAEC | 1.266417 | 0.03176 |
| Jurkat | 1.271277 | 0.03178 |
| HMF | 1.247276 | 0.03326 |
| Th0 | 1.278595 | 0.03428 |
| NHDFneo | 1.248860 | 0.03532 |
| HNPCEpiC | 1.220765 | 0.03544 |
| HMVECdAd | 1.282576 | 0.03558 |
| GM12878 | 1.313578 | 0.03620 |
| K562 | 1.300040 | 0.03916 |
| HVMF | 1.263224 | 0.04204 |
| HAEpiC | 1.233680 | 0.04204 |
| HPAF | 1.222411 | 0.04362 |
| GM18507 | 1.279689 | 0.04732 |
| HMVECLBl | 1.233235 | 0.04822 |
| HIPEpiC | 1.205041 | 0.04822 |
| HMVECdNeo | 1.253391 | 0.05000 |
| NB4 | 1.251940 | 0.05070 |
| HConF | 1.221178 | 0.05200 |
| HCPEpiC | 1.203049 | 0.05710 |
| HGF | 1.226914 | 0.05786 |
| SKNMC | 1.229167 | 0.05864 |
| BJ | 1.214439 | 0.06182 |
| HRGEC | 1.215407 | 0.06238 |
| Th1 | 1.226572 | 0.06274 |
| Urothelia | 1.231817 | 0.06554 |
| HUVEC | 1.229086 | 0.06688 |
| HAh | 1.175423 | 0.07100 |
| CMK | 1.267772 | 0.07458 |
| WI38 | 1.202052 | 0.07556 |
| HRCEpiC | 1.180845 | 0.07662 |
| HMVECdBlAd | 1.203800 | 0.07730 |
| HeLaS3 | 1.227281 | 0.07826 |
| RPTEC | 1.168818 | 0.07894 |
| HL60 | 1.246185 | 0.08114 |
| HMVECdBlNeo | 1.199154 | 0.08214 |
| AG10803 | 1.193220 | 0.08338 |
| CD34Mobilized | 1.209532 | 0.08368 |
| PANC1 | 1.226076 | 0.08606 |
| HFF | 1.176990 | 0.08718 |
| BE2C | 1.224021 | 0.08796 |
| GM19238 | 1.230456 | 0.08806 |
| HBMEC | 1.172862 | 0.08832 |
| HAc | 1.167622 | 0.08892 |
| UrotheliaUT189 | 1.210744 | 0.08918 |
| MonocytesCD14RO01746 | 1.235250 | 0.09156 |
| Stellate | 1.203815 | 0.10112 |
| AG04449 | 1.174121 | 0.10272 |
| NHDFAd | 1.156943 | 0.10466 |
| GM19239 | 1.226124 | 0.10578 |
| HRE | 1.152750 | 0.10580 |
| AG09319 | 1.170422 | 0.11304 |
| NHLF | 1.157146 | 0.11568 |
| HPdLF | 1.169274 | 0.11660 |
| HCFaa | 1.159983 | 0.11688 |
| AG09309 | 1.150667 | 0.11834 |
| Huh7 | 1.199137 | 0.11938 |
| Myometr | 1.167967 | 0.12016 |
| HSMMemb | 1.167013 | 0.12678 |
| GM19240 | 1.168830 | 0.13108 |
| HSMM | 1.130901 | 0.13896 |
| IshikawaTamoxifen | 1.162818 | 0.14212 |
| HeLaS3IFNa4h | 1.180845 | 0.14372 |
| HPDE6E6E7 | 1.153625 | 0.14930 |
| SAEC | 1.120227 | 0.15052 |
| HEEpiC | 1.119998 | 0.15350 |
| CLL | 1.179503 | 0.16230 |
| SKNSHRA | 1.194899 | 0.16466 |
| PanIsletD | 1.124889 | 0.17120 |
| NHA | 1.122834 | 0.17616 |
| NHEK | 1.128014 | 0.18076 |
| FibroP | 1.101883 | 0.18468 |
| HCT116 | 1.139091 | 0.18506 |
| IshikawaEstradiol | 1.128426 | 0.18886 |
| A549 | 1.140846 | 0.19412 |
| Hepatocytes | 1.155011 | 0.20462 |
| HAsp | 1.111972 | 0.20542 |
| WERIRb1 | 1.112804 | 0.20886 |
| GM12892 | 1.141875 | 0.21680 |
| HepG2 | 1.120076 | 0.22160 |
| HRPEpiC | 1.089489 | 0.22284 |
| Huh7.5 | 1.124376 | 0.22638 |
| T47D | 1.128371 | 0.23012 |
| LNCaPAndrogen | 1.120484 | 0.23302 |
| Melano | 1.082181 | 0.23466 |
| PrEC | 1.079430 | 0.24356 |
| ProgFib | 1.102653 | 0.24750 |
| MCF7Hypoxia | 1.114045 | 0.24848 |
| HTR8svn | 1.098519 | 0.25260 |
| pHTE | 1.097393 | 0.25352 |
| PanIslets | 1.099048 | 0.26180 |
| Chorion | 1.121488 | 0.26200 |
| GM12891 | 1.112411 | 0.27036 |
| H9ES | 1.087825 | 0.28132 |
| Gliobla | 1.090687 | 0.29094 |
| H7hESC | 1.044604 | 0.32512 |
| HSMMtube | 1.055026 | 0.32976 |
| H1hESC | 1.070600 | 0.33194 |
| MCF7 | 1.060974 | 0.33680 |
| RWPE1 | 1.056275 | 0.34208 |
| Caco2 | 1.086387 | 0.34326 |
| HMEC | 1.055709 | 0.34332 |
| NT2D1 | 1.042637 | 0.35820 |
| LNCaP | 1.042045 | 0.38040 |
| Medullo | 1.046165 | 0.38296 |
| Osteobl | 1.039522 | 0.39454 |
| 8988T | 1.043133 | 0.39758 |
| iPS | 1.034812 | 0.41908 |
| Fibrobl | 1.022347 | 0.43662 |

# Mean arterial pressure

| DHS sample | fold enrichment | p value |
|---|---|---|
| HRGEC | 1.3726387 | 0.00276 |
| AoSMC | 1.3082872 | 0.00350 |
| HAsp | 1.3140746 | 0.00362 |
| HPdLF | 1.3448541 | 0.00474 |
| PANC1 | 1.3968562 | 0.00532 |
| RPTEC | 1.2688122 | 0.00646 |
| HCFaa | 1.3033758 | 0.00712 |
| AoAF | 1.3163487 | 0.00772 |
| HGF | 1.3180882 | 0.00954 |
| AG09319 | 1.3196758 | 0.00984 |
| HMVECdLyAd | 1.3361279 | 0.01088 |
| HBMEC | 1.2498991 | 0.01464 |
| HNPCEpiC | 1.2305820 | 0.01624 |
| HMVECdNeo | 1.2963670 | 0.01818 |
| HRCEpiC | 1.2320699 | 0.02082 |
| SAEC | 1.2094938 | 0.02406 |
| HRPEpiC | 1.2032589 | 0.02424 |
| HMVECLLy | 1.2793537 | 0.02486 |
| AG04450 | 1.2593463 | 0.02606 |
| HAc | 1.2124413 | 0.02644 |
| AG09309 | 1.2366176 | 0.02700 |
| HMVECdAd | 1.2811777 | 0.02728 |
| Th2 | 1.3478565 | 0.02988 |
| HEEpiC | 1.2031782 | 0.03020 |
| HMVECLBl | 1.2529699 | 0.03084 |
| AG04449 | 1.2389247 | 0.03162 |
| NHDFAd | 1.2229410 | 0.03308 |
| IshikawaEstradiol | 1.2583535 | 0.03366 |
| PrEC | 1.2001950 | 0.03514 |
| BJ | 1.2330153 | 0.03518 |
| HMVECdLyNeo | 1.2581077 | 0.03584 |
| NHA | 1.2114062 | 0.03702 |
| NHLF | 1.2085430 | 0.03784 |
| HRE | 1.1941960 | 0.03956 |
| HCM | 1.2052846 | 0.04162 |
| GM06990 | 1.3160152 | 0.04182 |
| HPAEC | 1.2392189 | 0.04194 |
| K562 | 1.2923796 | 0.04242 |
| WI38 | 1.2344456 | 0.04402 |
| HFFMyc | 1.2075817 | 0.04512 |
| HCPEpiC | 1.1996592 | 0.04532 |
| HMVECdBlNeo | 1.2348041 | 0.04534 |
| HIPEpiC | 1.1967850 | 0.04636 |
| NHDFneo | 1.2183658 | 0.04964 |
| HPAF | 1.1984319 | 0.05194 |
| HPF | 1.2097782 | 0.05232 |
| SKMC | 1.1933423 | 0.05310 |
| HFF | 1.1969876 | 0.05586 |
| HeLaS3 | 1.2262769 | 0.06052 |
| RWPE1 | 1.2076912 | 0.06060 |
| HCF | 1.1967910 | 0.06158 |
| HMVECdBlAd | 1.2081650 | 0.06262 |
| HAEpiC | 1.1908162 | 0.06286 |
| CD20 | 1.2815168 | 0.06432 |
| GM12864 | 1.2380658 | 0.06606 |
| HConF | 1.1897097 | 0.06834 |
| IshikawaTamoxifen | 1.2132715 | 0.06912 |
| HCT116 | 1.2181154 | 0.06914 |
| AG10803 | 1.1918849 | 0.07292 |
| GM12865 | 1.2109219 | 0.07360 |
| HMF | 1.1767945 | 0.07524 |
| HAh | 1.1470463 | 0.07996 |
| HTR8svn | 1.2044288 | 0.08028 |
| CMK | 1.2189977 | 0.10320 |
| SKNMC | 1.1565834 | 0.10372 |
| HVMF | 1.1675127 | 0.11196 |
| HUVEC | 1.1683583 | 0.11260 |
| A549 | 1.1800673 | 0.11884 |
| CD34Mobilized | 1.1686617 | 0.11968 |
| HPDE6E6E7 | 1.1615886 | 0.12602 |
| Jurkat | 1.1530836 | 0.12700 |
| HSMM | 1.1194682 | 0.14258 |
| Myometr | 1.1318556 | 0.14582 |
| NB4 | 1.1599578 | 0.14986 |
| GM12878 | 1.1609866 | 0.15386 |
| Huh7 | 1.1625751 | 0.15522 |
| Stellate | 1.1358101 | 0.18094 |
| MonocytesCD14RO01746 | 1.1523512 | 0.18098 |
| HL60 | 1.1487466 | 0.18254 |
| CLL | 1.1431620 | 0.20356 |
| HeLaS3IFNa4h | 1.1234399 | 0.21094 |
| T47D | 1.1296898 | 0.21256 |
| HSMMemb | 1.0923709 | 0.24982 |
| ProgFib | 1.0880736 | 0.25986 |
| NHEK | 1.0783420 | 0.26418 |
| BE2C | 1.0810262 | 0.27102 |
| Huh7.5 | 1.0891476 | 0.28794 |
| PanIsletD | 1.0646547 | 0.29146 |
| GM18507 | 1.0766644 | 0.30170 |
| Urothelia | 1.0489346 | 0.35782 |
| FibroP | 1.0394695 | 0.35846 |
| Gliobla | 1.0490148 | 0.36746 |
| MCF7 | 1.0344275 | 0.39056 |
| UrotheliaUT189 | 1.0302949 | 0.40676 |
| GM19239 | 1.0234033 | 0.42894 |
| GM19238 | 1.0144880 | 0.45384 |
| GM12891 | 1.0037281 | 0.47752 |
| Melano | 1.0038630 | 0.48252 |
| H7hESC | 0.9980442 | 0.50314 |
| LNCaP | 0.9938432 | 0.50816 |
| LNCaPAndrogen | 0.9899297 | 0.51182 |
| MCF7Hypoxia | 0.9812883 | 0.52396 |
| GM19240 | 0.9822309 | 0.54268 |
| WERIRb1 | 0.9827945 | 0.55080 |
| HSMMtube | 0.9826089 | 0.55344 |
| HepG2 | 0.9700984 | 0.56424 |
| SKNSHRA | 0.9580219 | 0.57338 |
| Caco2 | 0.9362092 | 0.57478 |
| H1hESC | 0.9447507 | 0.62888 |
| GM12892 | 0.9344315 | 0.63178 |
| H9ES | 0.9490535 | 0.63428 |
| Hepatocytes | 0.9333627 | 0.63832 |
| HMEC | 0.9497703 | 0.64942 |
| NT2D1 | 0.9534208 | 0.65592 |
| pHTE | 0.9368341 | 0.67376 |
| Th0 | 0.9219382 | 0.68580 |
| Th1 | 0.9190538 | 0.69928 |
| Fibrobl | 0.9186238 | 0.71958 |
| PanIslets | 0.8817873 | 0.78470 |
| Chorion | 0.8545926 | 0.78540 |
| Osteobl | 0.8851074 | 0.78824 |
| 8988T | 0.8524062 | 0.78952 |
| Medullo | 0.8768435 | 0.79034 |
| iPS | 0.7740715 | 0.90108 |

# Height 2

| DHS sample | fold enrichment | p value |
|---|---|---|
| HMVECLLy | 1.4556528 | 0.00480 |
| HCFaa | 1.3910531 | 0.00504 |
| HMVECdLyAd | 1.4637289 | 0.00536 |
| HMVECdNeo | 1.4427698 | 0.00590 |
| Jurkat | 1.4263560 | 0.00822 |
| HMVECdLyNeo | 1.4242818 | 0.00874 |
| HAsp | 1.3354646 | 0.01122 |
| HRGEC | 1.3762548 | 0.01266 |
| HMVECdBlNeo | 1.3830255 | 0.01462 |
| SKMC | 1.3284760 | 0.01514 |
| HAEpiC | 1.3473367 | 0.01622 |
| HPAEC | 1.3757352 | 0.01654 |
| HMVECLBl | 1.3563424 | 0.01824 |
| HPAF | 1.3210255 | 0.01866 |
| HFFMyc | 1.3098794 | 0.01886 |
| HCM | 1.3066088 | 0.01898 |
| HMVECdBlAd | 1.3601493 | 0.01944 |
| HMVECdAd | 1.3813515 | 0.01982 |
| AoSMC | 1.2955934 | 0.01988 |
| HCPEpiC | 1.3005678 | 0.02028 |
| HIPEpiC | 1.3051981 | 0.02118 |
| AoAF | 1.3317085 | 0.02184 |
| HCF | 1.3259077 | 0.02210 |
| HUVEC | 1.3383436 | 0.02222 |
| HGF | 1.3316316 | 0.02416 |
| HNPCEpiC | 1.2772528 | 0.02422 |
| SAEC | 1.2597380 | 0.02644 |
| HPF | 1.3085785 | 0.02946 |
| RPTEC | 1.2479923 | 0.03008 |
| NHLF | 1.2712670 | 0.03254 |
| HAh | 1.2431349 | 0.03416 |
| HMF | 1.2771553 | 0.03580 |
| HRCEpiC | 1.2520272 | 0.03884 |
| AG04449 | 1.2803365 | 0.03912 |
| HPdLF | 1.2948557 | 0.03988 |
| HCT116 | 1.3027969 | 0.04684 |
| AG04450 | 1.2778009 | 0.04778 |
| NHA | 1.2541101 | 0.04816 |
| RWPE1 | 1.2691379 | 0.05026 |
| HRPEpiC | 1.2131405 | 0.05044 |
| AG09309 | 1.2465880 | 0.05186 |
| HEEpiC | 1.2146289 | 0.05536 |
| SKNMC | 1.2477213 | 0.05670 |
| NHDFneo | 1.2623274 | 0.05680 |
| HConF | 1.2467786 | 0.05810 |
| HVMF | 1.2791771 | 0.05956 |
| PANC1 | 1.2839755 | 0.06256 |
| HBMEC | 1.2131282 | 0.06774 |
| HFF | 1.2235691 | 0.07234 |
| HAc | 1.2034907 | 0.07614 |
| NHDFAd | 1.2177137 | 0.07680 |
| AG09319 | 1.2385454 | 0.07956 |
| PrEC | 1.1897192 | 0.08104 |
| WI38 | 1.2334868 | 0.08558 |
| BJ | 1.2102303 | 0.09590 |
| HPDE6E6E7 | 1.2202950 | 0.10026 |
| HSMM | 1.1675097 | 0.10946 |
| AG10803 | 1.1976729 | 0.11706 |
| NHEK | 1.1883673 | 0.11816 |
| A549 | 1.2088415 | 0.13272 |
| K562 | 1.2194596 | 0.13494 |
| Th2 | 1.2429923 | 0.13614 |
| GM12865 | 1.1884385 | 0.15390 |
| Huh7 | 1.1728138 | 0.18106 |
| Gliobla | 1.1680464 | 0.18490 |
| IshikawaEstradiol | 1.1458245 | 0.18588 |
| ProgFib | 1.1422636 | 0.20186 |
| HRE | 1.1123342 | 0.20382 |
| GM12864 | 1.1497825 | 0.21708 |
| CD34Mobilized | 1.1329244 | 0.22126 |
| CMK | 1.1455092 | 0.23210 |
| CD20 | 1.1450357 | 0.24650 |
| NT2D1 | 1.0868502 | 0.24734 |
| Stellate | 1.1235857 | 0.25178 |
| GM12878 | 1.1265791 | 0.25416 |
| PanIsletD | 1.0980642 | 0.25456 |
| Myometr | 1.0994576 | 0.25592 |
| HSMMemb | 1.1083719 | 0.26128 |
| NB4 | 1.1065063 | 0.27998 |
| WERIRb1 | 1.0810848 | 0.28284 |
| HL60 | 1.1151941 | 0.28316 |
| IshikawaTamoxifen | 1.0907945 | 0.29018 |
| FibroP | 1.0720163 | 0.30238 |
| MCF7 | 1.0671397 | 0.33250 |
| Huh7.5 | 1.0763564 | 0.34144 |
| Hepatocytes | 1.0770219 | 0.34566 |
| BE2C | 1.0649249 | 0.34674 |
| HTR8svn | 1.0655011 | 0.35102 |
| Urothelia | 1.0542961 | 0.37052 |
| HeLaS3 | 1.0522614 | 0.37448 |
| T47D | 1.0573408 | 0.37596 |
| GM18507 | 1.0529155 | 0.38280 |
| HSMMtube | 1.0418530 | 0.38436 |
| HeLaS3IFNa4h | 1.0519979 | 0.38502 |
| LNCaPAndrogen | 1.0340689 | 0.41754 |
| GM06990 | 1.0369956 | 0.42144 |
| Melano | 1.0240072 | 0.42388 |
| GM19239 | 1.0293769 | 0.42936 |
| MCF7Hypoxia | 1.0124334 | 0.45064 |
| Medullo | 1.0087081 | 0.47278 |
| CLL | 1.0087230 | 0.47344 |
| HepG2 | 1.0042288 | 0.48080 |
| pHTE | 1.0016635 | 0.48778 |
| GM19240 | 0.9814072 | 0.53596 |
| Caco2 | 0.9250799 | 0.55816 |
| Chorion | 0.9570040 | 0.55852 |
| LNCaP | 0.9643327 | 0.57240 |
| GM19238 | 0.9581796 | 0.57324 |
| UrotheliaUT189 | 0.9591555 | 0.58280 |
| H1hESC | 0.9539407 | 0.58284 |
| H9ES | 0.9618314 | 0.58412 |
| Th0 | 0.9517708 | 0.59048 |
| SKNSHRA | 0.9424326 | 0.59220 |
| HMEC | 0.9584932 | 0.59450 |
| PanIslets | 0.9484952 | 0.59728 |
| GM12891 | 0.9329350 | 0.60852 |
| 8988T | 0.9002565 | 0.65398 |
| MonocytesCD14RO01746 | 0.8977728 | 0.67414 |
| Fibrobl | 0.9064617 | 0.68986 |
| H7hESC | 0.9427753 | 0.71202 |
| Osteobl | 0.8892278 | 0.71768 |
| Th1 | 0.8733288 | 0.74036 |
| GM12892 | 0.8424794 | 0.75192 |
| iPS | 0.8276557 | 0.76340 |

# Lipid levels 2

| DHS sample | fold enrichment | p value |
|---|---|---|
| HL60 | 1.5027653 | 0.00324 |
| RPTEC | 1.3152387 | 0.00414 |
| HRCEpiC | 1.3165277 | 0.00576 |
| HRE | 1.2710591 | 0.01442 |
| HIPEpiC | 1.2724488 | 0.01710 |
| Jurkat | 1.2936882 | 0.02196 |
| WI38 | 1.2856562 | 0.02600 |
| HCPEpiC | 1.2486111 | 0.02706 |
| HNPCEpiC | 1.2310174 | 0.03078 |
| HCFaa | 1.2514138 | 0.03082 |
| HGF | 1.2696699 | 0.03280 |
| HAh | 1.2089248 | 0.03938 |
| HCM | 1.2206255 | 0.04198 |
| MonocytesCD14RO01746 | 1.3021049 | 0.04522 |
| HAEpiC | 1.2361340 | 0.04658 |
| H7hESC | 1.1627256 | 0.04836 |
| AG04450 | 1.2441407 | 0.04874 |
| HPAF | 1.2151969 | 0.04982 |
| HepG2 | 1.2766125 | 0.05032 |
| AG04449 | 1.2271533 | 0.05040 |
| PrEC | 1.1903203 | 0.05214 |
| HeLaS3 | 1.2703437 | 0.05304 |
| HBMEC | 1.2097220 | 0.05310 |
| NHA | 1.2186175 | 0.05410 |
| SAEC | 1.1850067 | 0.05606 |
| NHEK | 1.2234747 | 0.05688 |
| HPdLF | 1.2260024 | 0.05948 |
| HEEpiC | 1.1833012 | 0.06414 |
| HVMF | 1.2243631 | 0.06624 |
| HeLaS3IFNa4h | 1.2783170 | 0.06742 |
| IshikawaEstradiol | 1.2188114 | 0.07144 |
| Stellate | 1.2308500 | 0.07408 |
| K562 | 1.2486940 | 0.07786 |
| NHLF | 1.1866015 | 0.08104 |
| BJ | 1.1969613 | 0.08230 |
| HCT116 | 1.2178250 | 0.08274 |
| HPF | 1.1965305 | 0.08382 |
| GM12865 | 1.2115601 | 0.08420 |
| PANC1 | 1.2188334 | 0.09212 |
| HConF | 1.1845014 | 0.09366 |
| HAc | 1.1622570 | 0.09696 |
| NB4 | 1.1960660 | 0.10286 |
| HTR8svn | 1.1919639 | 0.10402 |
| AoSMC | 1.1511504 | 0.10614 |
| HPDE6E6E7 | 1.1897458 | 0.10810 |
| HCF | 1.1711732 | 0.10884 |
| HMVECdAd | 1.1855847 | 0.10990 |
| GM12864 | 1.1953620 | 0.11036 |
| SKMC | 1.1651162 | 0.11094 |
| NT2D1 | 1.1454215 | 0.11138 |
| FibroP | 1.1354904 | 0.11730 |
| HAsp | 1.1627751 | 0.11930 |
| AG10803 | 1.1599786 | 0.12408 |
| HMVECLLy | 1.1660030 | 0.12510 |
| Huh7.5 | 1.2030776 | 0.12522 |
| PanIsletD | 1.1496240 | 0.12758 |
| HMF | 1.1502930 | 0.12768 |
| HMVECLBl | 1.1571703 | 0.12810 |
| HSMMemb | 1.1632191 | 0.13548 |
| HSMM | 1.1347827 | 0.13594 |
| HFF | 1.1445469 | 0.13602 |
| GM12878 | 1.1860199 | 0.13664 |
| RWPE1 | 1.1584202 | 0.14070 |
| HMVECdLyAd | 1.1561725 | 0.14322 |
| HMVECdLyNeo | 1.1526400 | 0.14590 |
| HRPEpiC | 1.1264909 | 0.14662 |
| IshikawaTamoxifen | 1.1568015 | 0.14802 |
| HMVECdNeo | 1.1502649 | 0.14928 |
| Huh7 | 1.1767997 | 0.14974 |
| AG09319 | 1.1501027 | 0.14980 |
| AG09309 | 1.1321361 | 0.15084 |
| GM06990 | 1.1877760 | 0.15176 |
| Myometr | 1.1430349 | 0.15214 |
| HRGEC | 1.1377145 | 0.15690 |
| Fibrobl | 1.1447806 | 0.15896 |
| CMK | 1.1804279 | 0.16536 |
| Th2 | 1.1707259 | 0.16616 |
| Medullo | 1.1536184 | 0.16620 |
| HSMMtube | 1.1175245 | 0.17602 |
| HPAEC | 1.1286930 | 0.17972 |
| AoAF | 1.1275355 | 0.18102 |
| HMVECdBlAd | 1.1240298 | 0.18318 |
| GM19240 | 1.1339826 | 0.18618 |
| WERIRb1 | 1.1175645 | 0.18716 |
| UrotheliaUT189 | 1.1399010 | 0.19018 |
| Melano | 1.0954580 | 0.19658 |
| Th0 | 1.1318964 | 0.19978 |
| Th1 | 1.1293969 | 0.20036 |
| Urothelia | 1.1277464 | 0.20614 |
| A549 | 1.1311937 | 0.21176 |
| HMEC | 1.1039139 | 0.22624 |
| ProgFib | 1.1110657 | 0.23248 |
| Gliobla | 1.1214028 | 0.23438 |
| HMVECdBlNeo | 1.0965190 | 0.24104 |
| BE2C | 1.1043362 | 0.24722 |
| NHDFAd | 1.0860223 | 0.25050 |
| GM19238 | 1.1091293 | 0.25392 |
| NHDFneo | 1.0875184 | 0.26450 |
| pHTE | 1.0905626 | 0.26758 |
| GM19239 | 1.1060770 | 0.26836 |
| CD34Mobilized | 1.0876598 | 0.27220 |
| GM18507 | 1.0960520 | 0.27256 |
| Osteobl | 1.0883955 | 0.27490 |
| SKNMC | 1.0768727 | 0.28256 |
| HFFMyc | 1.0729397 | 0.28318 |
| PanIslets | 1.0879338 | 0.28688 |
| CD20 | 1.1014595 | 0.29000 |
| CLL | 1.0976520 | 0.29090 |
| GM12892 | 1.0990285 | 0.29202 |
| MCF7 | 1.0790489 | 0.29284 |
| GM12891 | 1.0965743 | 0.29568 |
| Caco2 | 1.1111168 | 0.30994 |
| SKNSHRA | 1.0568229 | 0.37286 |
| LNCaPAndrogen | 1.0486708 | 0.37392 |
| HUVEC | 1.0404791 | 0.38260 |
| Hepatocytes | 1.0478190 | 0.38526 |
| Chorion | 1.0404895 | 0.40146 |
| LNCaP | 1.0292003 | 0.40922 |
| MCF7Hypoxia | 1.0313995 | 0.41772 |
| iPS | 1.0180368 | 0.44836 |
| 8988T | 1.0084801 | 0.46320 |
| H9ES | 0.9977343 | 0.49078 |
| H1hESC | 0.9756670 | 0.53722 |
| T47D | 0.9288270 | 0.64390 |

# Lipid levels 1

| DHS sample | fold enrichment | p value |
|---|---|---|
| RPTEC | 1.2629096 | 0.00478 |
| HL60 | 1.4129253 | 0.00542 |
| HRCEpiC | 1.2337702 | 0.01252 |
| HRE | 1.2177906 | 0.01880 |
| Jurkat | 1.2315208 | 0.03016 |
| HIPEpiC | 1.1815174 | 0.03710 |
| MonocytesCD14RO01746 | 1.2839892 | 0.03802 |
| HCFaa | 1.1869976 | 0.04610 |
| HNPCEpiC | 1.1608533 | 0.05460 |
| HAh | 1.1572872 | 0.05574 |
| SAEC | 1.1549282 | 0.05850 |
| HCPEpiC | 1.1569116 | 0.06394 |
| WERIRb1 | 1.1925033 | 0.06484 |
| HepG2 | 1.2123676 | 0.06576 |
| PrEC | 1.1508479 | 0.06668 |
| HEEpiC | 1.1500026 | 0.07070 |
| NHA | 1.1603945 | 0.07172 |
| HPAF | 1.1570097 | 0.07238 |
| HPF | 1.1643338 | 0.07720 |
| WI38 | 1.1727661 | 0.07864 |
| NHEK | 1.1735989 | 0.07882 |
| H7hESC | 1.1298481 | 0.07970 |
| HeLaS3IFNa4h | 1.2143761 | 0.08150 |
| Medullo | 1.1934211 | 0.08244 |
| IshikawaEstradiol | 1.1790947 | 0.08338 |
| HeLaS3 | 1.1917895 | 0.08392 |
| PANC1 | 1.1888390 | 0.08626 |
| SKMC | 1.1501744 | 0.08766 |
| HBMEC | 1.1382908 | 0.09200 |
| HCM | 1.1363787 | 0.09446 |
| HGF | 1.1549063 | 0.09548 |
| Stellate | 1.1759104 | 0.10128 |
| HCT116 | 1.1744939 | 0.10442 |
| GM12865 | 1.1729604 | 0.10520 |
| GM12864 | 1.1733275 | 0.10626 |
| AG04449 | 1.1400087 | 0.11056 |
| AG04450 | 1.1467826 | 0.11300 |
| NHLF | 1.1291950 | 0.11382 |
| K562 | 1.1793135 | 0.11384 |
| HConF | 1.1388243 | 0.11692 |
| HTR8svn | 1.1581250 | 0.11706 |
| IshikawaTamoxifen | 1.1570728 | 0.11732 |
| NT2D1 | 1.1298239 | 0.12166 |
| AoSMC | 1.1146271 | 0.12252 |
| HMVECLBl | 1.1370485 | 0.12418 |
| HPDE6E6E7 | 1.1483342 | 0.12494 |
| HPdLF | 1.1360290 | 0.12592 |
| FibroP | 1.1119165 | 0.12938 |
| HAEpiC | 1.1261501 | 0.13014 |
| HSMMemb | 1.1439718 | 0.13102 |
| HMVECdAd | 1.1402301 | 0.14180 |
| HVMF | 1.1281520 | 0.14424 |
| HRGEC | 1.1215315 | 0.14722 |
| GM12878 | 1.1522860 | 0.14980 |
| NB4 | 1.1454420 | 0.15250 |
| HAsp | 1.1170573 | 0.15318 |
| HMF | 1.1095861 | 0.15836 |
| PanIsletD | 1.1098400 | 0.16788 |
| HRPEpiC | 1.0968597 | 0.16940 |
| HCF | 1.1052928 | 0.17466 |
| HSMM | 1.0899430 | 0.18168 |
| Myometr | 1.1070749 | 0.18402 |
| Melano | 1.0861465 | 0.18598 |
| BJ | 1.1020154 | 0.18926 |
| HAc | 1.0910722 | 0.18980 |
| HPAEC | 1.1040510 | 0.19218 |
| Th2 | 1.1294334 | 0.19478 |
| Fibrobl | 1.1076580 | 0.19486 |
| AG09309 | 1.0900597 | 0.19974 |
| BE2C | 1.1159702 | 0.20402 |
| Huh7.5 | 1.1237723 | 0.20418 |
| HMVECdLyNeo | 1.1022109 | 0.20422 |
| Huh7 | 1.1191992 | 0.20750 |
| RWPE1 | 1.0995198 | 0.21056 |
| HMVECdBlAd | 1.0937504 | 0.21208 |
| HFF | 1.0862922 | 0.21258 |
| Th0 | 1.1013747 | 0.22026 |
| Th1 | 1.0979568 | 0.22076 |
| GM19240 | 1.1008284 | 0.22274 |
| AG10803 | 1.0866622 | 0.22590 |
| AoAF | 1.0857184 | 0.22636 |
| CMK | 1.1094351 | 0.24262 |
| CD20 | 1.1134729 | 0.24658 |
| CLL | 1.1085652 | 0.24704 |
| AG09319 | 1.0793850 | 0.24788 |
| A549 | 1.0933901 | 0.25356 |
| GM06990 | 1.1047279 | 0.25664 |
| GM18507 | 1.0900884 | 0.25678 |
| HMVECdNeo | 1.0801547 | 0.26064 |
| GM19238 | 1.0881294 | 0.26908 |
| HMEC | 1.0726968 | 0.27330 |
| Osteobl | 1.0784787 | 0.27338 |
| GM12892 | 1.0937527 | 0.27590 |
| HMVECdBlNeo | 1.0693444 | 0.27986 |
| MCF7 | 1.0745814 | 0.28012 |
| Urothelia | 1.0744704 | 0.28416 |
| HSMMtube | 1.0582324 | 0.29344 |
| NHDFAd | 1.0564372 | 0.29506 |
| SKNMC | 1.0669537 | 0.29728 |
| HMVECdLyAd | 1.0633220 | 0.30522 |
| Gliobla | 1.0706713 | 0.30700 |
| NHDFneo | 1.0572519 | 0.30768 |
| ProgFib | 1.0653127 | 0.30914 |
| HFFMyc | 1.0532488 | 0.31030 |
| UrotheliaUT189 | 1.0655435 | 0.31154 |
| PanIslets | 1.0663199 | 0.31386 |
| GM19239 | 1.0704671 | 0.32096 |
| HMVECLLy | 1.0569479 | 0.32366 |
| CD34Mobilized | 1.0553706 | 0.33350 |
| pHTE | 1.0444052 | 0.36282 |
| LNCaPAndrogen | 1.0432527 | 0.37496 |
| GM12891 | 1.0489250 | 0.37732 |
| HUVEC | 1.0330404 | 0.39212 |
| 8988T | 1.0390766 | 0.39800 |
| SKNSHRA | 1.0338877 | 0.40926 |
| Caco2 | 1.0277116 | 0.42992 |
| H9ES | 1.0201395 | 0.43222 |
| MCF7Hypoxia | 1.0209737 | 0.43702 |
| iPS | 1.0196225 | 0.44514 |
| Hepatocytes | 1.0157597 | 0.44986 |
| LNCaP | 1.0083580 | 0.46348 |
| Chorion | 1.0040018 | 0.47972 |
| H1hESC | 0.9699155 | 0.56224 |
| T47D | 0.9575000 | 0.59330 |

# Cognitive decline in Alzheimers disease

| DHS sample | fold enrichment | p value |
|---|---|---|
| HAEpiC | 1.4066693 | 0.00566 |
| HPdLF | 1.4204355 | 0.00638 |
| HCFaa | 1.4048564 | 0.00642 |
| HVMF | 1.4604976 | 0.00688 |
| HPF | 1.4033988 | 0.00844 |
| HMF | 1.3821231 | 0.00900 |
| WI38 | 1.4284176 | 0.00962 |
| SKMC | 1.3280483 | 0.01810 |
| SKNMC | 1.3490234 | 0.02076 |
| HGF | 1.3446234 | 0.02448 |
| HCF | 1.3214299 | 0.02634 |
| HCM | 1.2860683 | 0.02948 |
| HIPEpiC | 1.2886568 | 0.03010 |
| NHDFneo | 1.3231177 | 0.03098 |
| A549 | 1.3831296 | 0.03138 |
| MCF7 | 1.3357115 | 0.03360 |
| HAsp | 1.2890281 | 0.03374 |
| AG04450 | 1.3182940 | 0.03544 |
| HCPEpiC | 1.2709025 | 0.03610 |
| AG04449 | 1.2929049 | 0.03738 |
| RPTEC | 1.2541110 | 0.03802 |
| HRCEpiC | 1.2625562 | 0.03872 |
| AG09319 | 1.3121308 | 0.03894 |
| AoSMC | 1.2461370 | 0.04584 |
| MCF7Hypoxia | 1.3642587 | 0.05098 |
| HBMEC | 1.2382284 | 0.05146 |
| HFF | 1.2635824 | 0.05186 |
| HNPCEpiC | 1.2313998 | 0.05380 |
| HFFMyc | 1.2448725 | 0.05966 |
| NHDFAd | 1.2328266 | 0.06574 |
| HConF | 1.2480572 | 0.07036 |
| HPAF | 1.2267267 | 0.07248 |
| BE2C | 1.2589785 | 0.07718 |
| AG10803 | 1.2460568 | 0.07856 |
| AoAF | 1.2307342 | 0.08278 |
| PANC1 | 1.2757518 | 0.08652 |
| AG09309 | 1.2053680 | 0.09324 |
| BJ | 1.2251407 | 0.09516 |
| HAc | 1.1765328 | 0.11446 |
| HRPEpiC | 1.1517306 | 0.12728 |
| HRE | 1.1431363 | 0.15646 |
| HTR8svn | 1.1778140 | 0.16774 |
| FibroP | 1.1376875 | 0.17204 |
| T47D | 1.2084311 | 0.17370 |
| HAh | 1.1287237 | 0.17662 |
| WERIRb1 | 1.1523047 | 0.17840 |
| HepG2 | 1.1883372 | 0.18034 |
| NHA | 1.1337049 | 0.19310 |
| HeLaS3 | 1.1541271 | 0.20530 |
| HMVECdBlAd | 1.1423662 | 0.20640 |
| HMVECLBl | 1.1247731 | 0.23126 |
| PanIsletD | 1.1123521 | 0.23508 |
| Huh7 | 1.1384417 | 0.24472 |
| NHLF | 1.1040371 | 0.24618 |
| Gliobla | 1.1319522 | 0.24820 |
| RWPE1 | 1.1131884 | 0.25124 |
| SKNSHRA | 1.1379941 | 0.25188 |
| HMVECdLyAd | 1.1091223 | 0.27046 |
| HMVECdBlNeo | 1.1079223 | 0.27192 |
| Melano | 1.0782014 | 0.28410 |
| HSMM | 1.0821920 | 0.28454 |
| Huh7.5 | 1.1145499 | 0.28866 |
| HRGEC | 1.0910375 | 0.29238 |
| Stellate | 1.0975886 | 0.29624 |
| Myometr | 1.0801944 | 0.30020 |
| HMVECdLyNeo | 1.0893740 | 0.30188 |
| SAEC | 1.0690350 | 0.30238 |
| HPDE6E6E7 | 1.0920309 | 0.30700 |
| HMVECLLy | 1.0845523 | 0.31482 |
| H7hESC | 1.0527495 | 0.31948 |
| HMVECdAd | 1.0788139 | 0.33086 |
| Th2 | 1.0771493 | 0.35320 |
| HPAEC | 1.0636552 | 0.35450 |
| HEEpiC | 1.0476717 | 0.36214 |
| HSMMtube | 1.0444988 | 0.38286 |
| HMVECdNeo | 1.0497974 | 0.38462 |
| IshikawaEstradiol | 1.0470868 | 0.38664 |
| ProgFib | 1.0460678 | 0.38730 |
| PrEC | 1.0377133 | 0.39208 |
| HCT116 | 1.0371831 | 0.40562 |
| HeLaS3IFNa4h | 1.0404285 | 0.40866 |
| HUVEC | 1.0144005 | 0.45558 |
| CD20 | 1.0111177 | 0.46268 |
| Caco2 | 0.9907131 | 0.46962 |
| NHEK | 1.0055240 | 0.47640 |
| CD34Mobilized | 0.9976503 | 0.49440 |
| Th0 | 0.9977847 | 0.49534 |
| HSMMemb | 0.9947521 | 0.50288 |
| Medullo | 0.9891839 | 0.51190 |
| Th1 | 0.9878562 | 0.51966 |
| Hepatocytes | 0.9680278 | 0.53190 |
| K562 | 0.9588088 | 0.56432 |
| CLL | 0.9444653 | 0.57958 |
| MonocytesCD14RO01746 | 0.9454658 | 0.58084 |
| GM19238 | 0.9439948 | 0.59332 |
| LNCaPAndrogen | 0.9409156 | 0.59552 |
| Fibrobl | 0.9494957 | 0.59738 |
| CMK | 0.9371783 | 0.59848 |
| IshikawaTamoxifen | 0.9490756 | 0.60240 |
| Chorion | 0.9187157 | 0.60752 |
| UrotheliaUT189 | 0.9405410 | 0.61182 |
| GM12878 | 0.9372786 | 0.61204 |
| PanIslets | 0.9355384 | 0.61388 |
| NT2D1 | 0.9502030 | 0.62178 |
| GM19239 | 0.9216313 | 0.62576 |
| GM12865 | 0.9280596 | 0.63374 |
| LNCaP | 0.9319077 | 0.63690 |
| Jurkat | 0.9286985 | 0.65316 |
| GM19240 | 0.9185442 | 0.66080 |
| 8988T | 0.8807793 | 0.66786 |
| GM18507 | 0.9026018 | 0.67502 |
| Urothelia | 0.9057338 | 0.68924 |
| H9ES | 0.9004368 | 0.69674 |
| GM12891 | 0.8705878 | 0.69886 |
| H1hESC | 0.8826581 | 0.70220 |
| HMEC | 0.8959241 | 0.72354 |
| Osteobl | 0.8736268 | 0.73256 |
| pHTE | 0.8709903 | 0.75176 |
| iPS | 0.8220804 | 0.75758 |
| HL60 | 0.8407134 | 0.76504 |
| GM12892 | 0.8066351 | 0.78352 |
| NB4 | 0.8379065 | 0.79032 |
| GM12864 | 0.8328519 | 0.79074 |
| GM06990 | 0.7851883 | 0.81880 |

# Kidney function−related traits

| DHS sample | fold enrichment | p value |
|---|---|---|
| HRCEpiC | 1.3477683 | 0.00766 |
| RPTEC | 1.3159545 | 0.00906 |
| MCF7 | 1.3917618 | 0.01902 |
| MCF7Hypoxia | 1.3859906 | 0.04146 |
| Th2 | 1.3490730 | 0.05638 |
| HRE | 1.2029023 | 0.06444 |
| Huh7 | 1.2799803 | 0.07256 |
| CD20 | 1.3332205 | 0.07752 |
| IshikawaEstradiol | 1.2448265 | 0.07758 |
| HPF | 1.2215782 | 0.08046 |
| CD34Mobilized | 1.2489871 | 0.08656 |
| IshikawaTamoxifen | 1.2305050 | 0.09552 |
| MonocytesCD14RO01746 | 1.2714799 | 0.10448 |
| HepG2 | 1.1944125 | 0.15170 |
| T47D | 1.1977109 | 0.15400 |
| SAEC | 1.1316919 | 0.15784 |
| Caco2 | 1.2595349 | 0.16262 |
| AoSMC | 1.1354464 | 0.16486 |
| HNPCEpiC | 1.1232386 | 0.16568 |
| HPAEC | 1.1454141 | 0.18908 |
| Huh7.5 | 1.1650455 | 0.19000 |
| SKMC | 1.1227231 | 0.19358 |
| SKNSHRA | 1.1752689 | 0.19382 |
| HPdLF | 1.1358970 | 0.19456 |
| HPDE6E6E7 | 1.1474689 | 0.19760 |
| LNCaPAndrogen | 1.1560517 | 0.20272 |
| HAsp | 1.1116855 | 0.21028 |
| AoAF | 1.1219179 | 0.21080 |
| HSMMemb | 1.1380296 | 0.21168 |
| Jurkat | 1.1261165 | 0.21582 |
| HBMEC | 1.1030121 | 0.22032 |
| HAh | 1.0953617 | 0.22070 |
| FibroP | 1.1014929 | 0.22078 |
| AG09309 | 1.1057159 | 0.22542 |
| Myometr | 1.1178561 | 0.22744 |
| Hepatocytes | 1.1456448 | 0.23176 |
| HCFaa | 1.1051523 | 0.23284 |
| HEEpiC | 1.0940545 | 0.23476 |
| Stellate | 1.1290918 | 0.23874 |
| NB4 | 1.1244041 | 0.24540 |
| HL60 | 1.1403111 | 0.24774 |
| Th0 | 1.1237881 | 0.24896 |
| AG04450 | 1.1046872 | 0.25378 |
| HSMM | 1.0823473 | 0.26754 |
| BJ | 1.0908580 | 0.26762 |
| WI38 | 1.1000891 | 0.26946 |
| HAc | 1.0782951 | 0.27358 |
| UrotheliaUT189 | 1.0999436 | 0.27576 |
| CLL | 1.1197447 | 0.28170 |
| HUVEC | 1.0947871 | 0.28418 |
| NHA | 1.0767976 | 0.28422 |
| Urothelia | 1.0922886 | 0.29484 |
| NHLF | 1.0713339 | 0.29692 |
| HVMF | 1.0834530 | 0.29954 |
| GM19239 | 1.0981229 | 0.30488 |
| HMVECdBlAd | 1.0797097 | 0.30622 |
| NT2D1 | 1.0618111 | 0.30928 |
| AG09319 | 1.0764518 | 0.30944 |
| Gliobla | 1.0878632 | 0.31434 |
| 8988T | 1.0968395 | 0.31642 |
| HeLaS3IFNa4h | 1.0867707 | 0.31678 |
| HCPEpiC | 1.0657371 | 0.31702 |
| HRGEC | 1.0724767 | 0.32166 |
| H9ES | 1.0721552 | 0.32392 |
| H1hESC | 1.0770208 | 0.32446 |
| PanIsletD | 1.0637379 | 0.33002 |
| LNCaP | 1.0642970 | 0.33256 |
| WERIRb1 | 1.0574509 | 0.33738 |
| NHDFAd | 1.0589089 | 0.33780 |
| PrEC | 1.0531723 | 0.33848 |
| NHDFneo | 1.0613085 | 0.33976 |
| RWPE1 | 1.0622213 | 0.34078 |
| CMK | 1.0757144 | 0.34488 |
| GM19238 | 1.0730966 | 0.34550 |
| HCM | 1.0529028 | 0.34856 |
| GM12878 | 1.0701193 | 0.34888 |
| HRPEpiC | 1.0418331 | 0.35978 |
| Th1 | 1.0614898 | 0.36206 |
| H7hESC | 1.0336563 | 0.36570 |
| NHEK | 1.0480481 | 0.37274 |
| ProgFib | 1.0492358 | 0.37766 |
| AG04449 | 1.0431609 | 0.38086 |
| GM12891 | 1.0582345 | 0.38112 |
| HMVECdLyNeo | 1.0484907 | 0.38124 |
| A549 | 1.0497429 | 0.38160 |
| HFF | 1.0415462 | 0.38254 |
| HMVECdBlNeo | 1.0430788 | 0.38908 |
| GM12865 | 1.0437976 | 0.39174 |
| HeLaS3 | 1.0428495 | 0.39268 |
| HConF | 1.0385837 | 0.39422 |
| HCF | 1.0385722 | 0.39540 |
| HSMMtube | 1.0348583 | 0.39740 |
| Melano | 1.0306341 | 0.40212 |
| HMVECLBl | 1.0331992 | 0.41336 |
| AG10803 | 1.0306271 | 0.41472 |
| GM19240 | 1.0319132 | 0.41928 |
| HGF | 1.0294989 | 0.42400 |
| HPAF | 1.0263854 | 0.42494 |
| HIPEpiC | 1.0211874 | 0.43488 |
| pHTE | 1.0208858 | 0.44256 |
| iPS | 1.0190685 | 0.45220 |
| PANC1 | 1.0133226 | 0.45366 |
| HMF | 1.0102699 | 0.46758 |
| HMVECdAd | 1.0086750 | 0.47072 |
| PanIslets | 1.0061431 | 0.47664 |
| HMVECdNeo | 1.0052016 | 0.47706 |
| HTR8svn | 1.0030282 | 0.48452 |
| Chorion | 1.0000895 | 0.48512 |
| GM18507 | 0.9981784 | 0.48858 |
| HFFMyc | 1.0025062 | 0.49006 |
| HMEC | 0.9989831 | 0.49282 |
| Medullo | 0.9993162 | 0.49454 |
| GM06990 | 0.9940312 | 0.49520 |
| HCT116 | 0.9792518 | 0.52998 |
| GM12892 | 0.9763181 | 0.53192 |
| HMVECdLyAd | 0.9810067 | 0.53518 |
| HMVECLLy | 0.9743668 | 0.55208 |
| GM12864 | 0.9705080 | 0.55302 |
| HAEpiC | 0.9761008 | 0.55958 |
| K562 | 0.9554868 | 0.57112 |
| Osteobl | 0.9530717 | 0.60226 |
| SKNMC | 0.9548784 | 0.60950 |
| BE2C | 0.9473877 | 0.61230 |
| Fibrobl | 0.9497511 | 0.61412 |

# Kidney function

| DHS sample | fold enrichment | p value |
|---|---|---|
| MCF7Hypoxia | 1.2080794 | 0.08634 |
| MCF7 | 1.1712572 | 0.09512 |
| RPTEC | 1.0859498 | 0.16892 |
| HRCEpiC | 1.0622696 | 0.25408 |
| BE2C | 1.0832573 | 0.25660 |
| Huh7 | 1.0770315 | 0.27382 |
| A549 | 1.0709935 | 0.28434 |
| HPDE6E6E7 | 1.0631529 | 0.29296 |
| Huh7.5 | 1.0643777 | 0.31260 |
| K562 | 1.0503519 | 0.35150 |
| HepG2 | 1.0463828 | 0.35656 |
| CMK | 1.0441287 | 0.37376 |
| WI38 | 1.0241011 | 0.40988 |
| Gliobla | 1.0268222 | 0.41346 |
| HAEpiC | 1.0157214 | 0.43744 |
| Stellate | 1.0148875 | 0.44686 |
| LNCaPAndrogen | 1.0052779 | 0.47364 |
| HPF | 1.0056235 | 0.47506 |
| CD34Mobilized | 1.0045462 | 0.47684 |
| Hepatocytes | 1.0030672 | 0.47860 |
| SKNSHRA | 0.9999570 | 0.47990 |
| HVMF | 1.0030663 | 0.48062 |
| HRE | 1.0001100 | 0.49772 |
| PANC1 | 0.9970644 | 0.49794 |
| HSMMemb | 0.9964191 | 0.50940 |
| Caco2 | 0.9777197 | 0.51916 |
| IshikawaEstradiol | 0.9915077 | 0.52368 |
| LNCaP | 0.9847730 | 0.54100 |
| AG04450 | 0.9828665 | 0.55998 |
| IshikawaTamoxifen | 0.9772399 | 0.56942 |
| T47D | 0.9678684 | 0.57714 |
| NB4 | 0.9717326 | 0.57848 |
| HL60 | 0.9646813 | 0.58808 |
| HeLaS3IFNa4h | 0.9661865 | 0.58858 |
| H7hESC | 0.9809051 | 0.59234 |
| HeLaS3 | 0.9668411 | 0.59580 |
| Myometr | 0.9701641 | 0.60470 |
| HCPEpiC | 0.9726233 | 0.61076 |
| HRPEpiC | 0.9721489 | 0.61420 |
| HEEpiC | 0.9722736 | 0.61470 |
| HCFaa | 0.9685595 | 0.61672 |
| CLL | 0.9514447 | 0.61962 |
| MonocytesCD14RO01746 | 0.9507682 | 0.62804 |
| AoSMC | 0.9687137 | 0.63350 |
| GM12891 | 0.9367622 | 0.64978 |
| SAEC | 0.9637199 | 0.65340 |
| GM19239 | 0.9384086 | 0.65518 |
| AG04449 | 0.9559697 | 0.66020 |
| HGF | 0.9508738 | 0.66802 |
| GM19240 | 0.9445268 | 0.67096 |
| HBMEC | 0.9558821 | 0.67308 |
| Medullo | 0.9389516 | 0.67468 |
| Osteobl | 0.9431455 | 0.67510 |
| PrEC | 0.9563250 | 0.67662 |
| PanIsletD | 0.9512899 | 0.68224 |
| GM18507 | 0.9340005 | 0.69076 |
| NHEK | 0.9425037 | 0.69504 |
| Chorion | 0.9131245 | 0.69556 |
| 8988T | 0.9112640 | 0.69894 |
| H9ES | 0.9340618 | 0.70040 |
| H1hESC | 0.9209852 | 0.70108 |
| GM19238 | 0.9238426 | 0.70418 |
| pHTE | 0.9318124 | 0.71356 |
| RWPE1 | 0.9360179 | 0.71580 |
| PanIslets | 0.9245205 | 0.71892 |
| HNPCEpiC | 0.9459067 | 0.71902 |
| Th2 | 0.9127020 | 0.72456 |
| Th0 | 0.9227831 | 0.72722 |
| HSMMtube | 0.9387563 | 0.73020 |
| HIPEpiC | 0.9393798 | 0.73258 |
| iPS | 0.8935882 | 0.73458 |
| HConF | 0.9315745 | 0.73824 |
| CD20 | 0.8950196 | 0.73988 |
| HPAF | 0.9336134 | 0.74598 |
| ProgFib | 0.9143952 | 0.75394 |
| HMEC | 0.9216720 | 0.75700 |
| HPdLF | 0.9199658 | 0.75948 |
| HMF | 0.9276859 | 0.75988 |
| GM12892 | 0.8893330 | 0.76184 |
| AG09319 | 0.9206276 | 0.76220 |
| FibroP | 0.9351847 | 0.76236 |
| Melano | 0.9363523 | 0.76748 |
| NHDFneo | 0.9187047 | 0.76884 |
| HSMM | 0.9327231 | 0.76960 |
| SKMC | 0.9251560 | 0.77228 |
| Th1 | 0.9050283 | 0.77602 |
| NT2D1 | 0.9252227 | 0.77994 |
| HCT116 | 0.9058464 | 0.78532 |
| Jurkat | 0.9059043 | 0.78948 |
| Urothelia | 0.9010972 | 0.79030 |
| HCM | 0.9230809 | 0.79036 |
| HAsp | 0.9134115 | 0.79858 |
| HCF | 0.9121020 | 0.79994 |
| Fibrobl | 0.8977987 | 0.80226 |
| HTR8svn | 0.8973922 | 0.80594 |
| GM12878 | 0.8807916 | 0.81490 |
| HUVEC | 0.8934069 | 0.82606 |
| WERIRb1 | 0.8962614 | 0.82632 |
| HFFMyc | 0.9050649 | 0.83568 |
| BJ | 0.8931263 | 0.83960 |
| HAc | 0.9054557 | 0.84508 |
| AoAF | 0.8884497 | 0.85316 |
| UrotheliaUT189 | 0.8648329 | 0.85438 |
| NHA | 0.8934962 | 0.85516 |
| HAh | 0.9061897 | 0.85618 |
| NHLF | 0.8942440 | 0.85812 |
| HFF | 0.8911465 | 0.86210 |
| AG09309 | 0.8905765 | 0.86236 |
| AG10803 | 0.8803993 | 0.86468 |
| HRGEC | 0.8792227 | 0.87188 |
| GM12864 | 0.8454125 | 0.88826 |
| HMVECdBlNeo | 0.8626648 | 0.89700 |
| HMVECdLyNeo | 0.8520134 | 0.90856 |
| HPAEC | 0.8515049 | 0.90992 |
| GM12865 | 0.8379007 | 0.91322 |
| HMVECdAd | 0.8363613 | 0.92110 |
| HMVECdLyAd | 0.8385736 | 0.92304 |
| HMVECLBl | 0.8466417 | 0.92682 |
| HMVECdBlAd | 0.8447716 | 0.92826 |
| SKNMC | 0.8226099 | 0.93998 |
| NHDFAd | 0.8413960 | 0.94520 |
| GM06990 | 0.7682279 | 0.94882 |
| HMVECdNeo | 0.8175213 | 0.95132 |
| HMVECLLy | 0.7981022 | 0.96904 |

# Testicular cancer

| DHS sample | fold enrichment | p value |
|---|---|---|
| Th2 | 1.5255983 | 0.00776 |
| GM06990 | 1.5242566 | 0.00886 |
| Jurkat | 1.3516348 | 0.01462 |
| GM12864 | 1.3977567 | 0.01768 |
| GM12865 | 1.3334160 | 0.02790 |
| CD20 | 1.4373973 | 0.02988 |
| GM12878 | 1.3372078 | 0.03940 |
| WI38 | 1.2834276 | 0.04056 |
| HCFaa | 1.2363535 | 0.04524 |
| Medullo | 1.3097579 | 0.04682 |
| GM18507 | 1.3042482 | 0.05066 |
| SAEC | 1.1998371 | 0.05284 |
| NT2D1 | 1.2134820 | 0.05302 |
| Myometr | 1.2441816 | 0.05424 |
| GM19239 | 1.3172467 | 0.05842 |
| K562 | 1.3124167 | 0.06186 |
| HFFMyc | 1.2124428 | 0.06328 |
| T47D | 1.3039841 | 0.06490 |
| NHDFAd | 1.2093207 | 0.06512 |
| HMF | 1.2141681 | 0.06974 |
| GM19238 | 1.2778451 | 0.07080 |
| AG09319 | 1.2254284 | 0.07376 |
| HConF | 1.2115069 | 0.07630 |
| PANC1 | 1.2374504 | 0.07852 |
| HCT116 | 1.2361478 | 0.08168 |
| 8988T | 1.3007547 | 0.08522 |
| WERIRb1 | 1.1995481 | 0.08908 |
| HPDE6E6E7 | 1.2254274 | 0.09090 |
| AG09309 | 1.1846238 | 0.09198 |
| NB4 | 1.2404153 | 0.09204 |
| MCF7 | 1.2283511 | 0.09668 |
| HGF | 1.1968621 | 0.10036 |
| AG10803 | 1.1980323 | 0.10138 |
| Gliobla | 1.2391675 | 0.10144 |
| GM12892 | 1.2738533 | 0.10200 |
| CLL | 1.2601664 | 0.10566 |
| GM12891 | 1.2632245 | 0.10632 |
| HSMMemb | 1.2073757 | 0.10842 |
| HPAF | 1.1705652 | 0.11276 |
| Th0 | 1.2123039 | 0.11764 |
| AG04450 | 1.1864458 | 0.11780 |
| HEEpiC | 1.1521318 | 0.11986 |
| HPdLF | 1.1833145 | 0.12066 |
| GM19240 | 1.1959987 | 0.12328 |
| PrEC | 1.1451514 | 0.12482 |
| NHDFneo | 1.1741150 | 0.12510 |
| CD34Mobilized | 1.1902476 | 0.12816 |
| H9ES | 1.1900817 | 0.12962 |
| HFF | 1.1603621 | 0.13044 |
| HAsp | 1.1561225 | 0.13346 |
| IshikawaEstradiol | 1.1783217 | 0.13426 |
| Hepatocytes | 1.2265363 | 0.13696 |
| Caco2 | 1.3092064 | 0.13960 |
| NHEK | 1.1589603 | 0.14316 |
| AoSMC | 1.1374091 | 0.14592 |
| BJ | 1.1626007 | 0.14624 |
| SKMC | 1.1476739 | 0.14764 |
| Th1 | 1.1822193 | 0.15074 |
| HVMF | 1.1640647 | 0.15342 |
| Stellate | 1.1805019 | 0.15374 |
| AoAF | 1.1535440 | 0.15416 |
| MCF7Hypoxia | 1.2004146 | 0.15446 |
| HMVECdBlNeo | 1.1572868 | 0.15532 |
| HMVECLBl | 1.1565245 | 0.15562 |
| AG04449 | 1.1478619 | 0.15810 |
| Chorion | 1.2208616 | 0.15838 |
| HSMM | 1.1295385 | 0.16020 |
| H7hESC | 1.1032797 | 0.16140 |
| CMK | 1.2053248 | 0.16158 |
| HCM | 1.1321281 | 0.16354 |
| HRGEC | 1.1455102 | 0.16420 |
| H1hESC | 1.1802366 | 0.16520 |
| HCF | 1.1419103 | 0.16956 |
| IshikawaTamoxifen | 1.1537094 | 0.17406 |
| LNCaP | 1.1462402 | 0.17944 |
| Huh7.5 | 1.1704368 | 0.18088 |
| HIPEpiC | 1.1221278 | 0.18126 |
| RWPE1 | 1.1353759 | 0.18938 |
| iPS | 1.1870670 | 0.19042 |
| HPF | 1.1315354 | 0.19374 |
| HUVEC | 1.1398269 | 0.19386 |
| SKNMC | 1.1325585 | 0.19910 |
| HepG2 | 1.1518513 | 0.20122 |
| Melano | 1.1024723 | 0.20130 |
| HL60 | 1.1586824 | 0.20960 |
| BE2C | 1.1363470 | 0.21750 |
| HeLaS3 | 1.1330126 | 0.21954 |
| FibroP | 1.1000570 | 0.22000 |
| MonocytesCD14RO01746 | 1.1508769 | 0.22362 |
| HeLaS3IFNa4h | 1.1365825 | 0.22592 |
| pHTE | 1.1234305 | 0.22688 |
| SKNSHRA | 1.1554583 | 0.23016 |
| LNCaPAndrogen | 1.1320615 | 0.23222 |
| Huh7 | 1.1309617 | 0.23548 |
| HSMMtube | 1.0951627 | 0.24924 |
| HTR8svn | 1.1105036 | 0.25040 |
| PanIsletD | 1.0959715 | 0.25196 |
| Urothelia | 1.1106499 | 0.25418 |
| HNPCEpiC | 1.0828821 | 0.25578 |
| HMEC | 1.1000898 | 0.25814 |
| ProgFib | 1.1054706 | 0.26062 |
| Fibrobl | 1.1031928 | 0.26586 |
| HPAEC | 1.0898657 | 0.27724 |
| HCPEpiC | 1.0764043 | 0.28318 |
| HAh | 1.0714526 | 0.28484 |
| HMVECdBlAd | 1.0851908 | 0.28574 |
| UrotheliaUT189 | 1.0936497 | 0.28850 |
| PanIslets | 1.0926072 | 0.29378 |
| HBMEC | 1.0714229 | 0.29708 |
| HAEpiC | 1.0779797 | 0.29750 |
| A549 | 1.0835813 | 0.30580 |
| Osteobl | 1.0807647 | 0.31352 |
| HAc | 1.0603920 | 0.31932 |
| NHA | 1.0596073 | 0.33084 |
| HRPEpiC | 1.0451449 | 0.35330 |
| NHLF | 1.0444259 | 0.36904 |
| HRE | 1.0351793 | 0.38610 |
| RPTEC | 1.0315060 | 0.39712 |
| HMVECdLyNeo | 1.0203709 | 0.44362 |
| HMVECdAd | 1.0172364 | 0.45140 |
| HMVECdLyAd | 0.9862486 | 0.52344 |
| HMVECdNeo | 0.9874855 | 0.52420 |
| HRCEpiC | 0.9845848 | 0.54436 |
| HMVECLLy | 0.9747192 | 0.55456 |

# Platelet count and volume

| DHS sample | fold enrichment | p value |
|---|---|---|
| K562 | 1.4974424 | 0.00796 |
| Th2 | 1.5105614 | 0.01174 |
| HAsp | 1.2972262 | 0.02122 |
| HIPEpiC | 1.2700800 | 0.02130 |
| HVMF | 1.3252715 | 0.02234 |
| HNPCEpiC | 1.2523548 | 0.02730 |
| HAEpiC | 1.2870931 | 0.02900 |
| HCFaa | 1.2713167 | 0.03212 |
| HCM | 1.2464369 | 0.03768 |
| HMF | 1.2586872 | 0.03886 |
| RPTEC | 1.2211340 | 0.04020 |
| HCF | 1.2677805 | 0.04072 |
| BJ | 1.2752601 | 0.04190 |
| HFF | 1.2507749 | 0.04362 |
| AoAF | 1.2619000 | 0.04528 |
| NB4 | 1.3200850 | 0.04538 |
| GM06990 | 1.3747336 | 0.04548 |
| Jurkat | 1.2804287 | 0.04584 |
| HCPEpiC | 1.2274705 | 0.04626 |
| CD34Mobilized | 1.2854606 | 0.05100 |
| SKNMC | 1.2669256 | 0.05164 |
| NHDFAd | 1.2317253 | 0.05276 |
| HMVECLBl | 1.2479745 | 0.05304 |
| HPdLF | 1.2524521 | 0.05336 |
| AG09309 | 1.2265673 | 0.05342 |
| NHDFneo | 1.2528460 | 0.05382 |
| AoSMC | 1.2063644 | 0.06042 |
| HBMEC | 1.2060033 | 0.06354 |
| HRCEpiC | 1.1977699 | 0.06546 |
| HPAF | 1.2145527 | 0.06810 |
| MonocytesCD14RO01746 | 1.2893079 | 0.08112 |
| SKMC | 1.1925796 | 0.08308 |
| NHLF | 1.1929845 | 0.08420 |
| AG10803 | 1.2104841 | 0.08994 |
| HTR8svn | 1.2276708 | 0.09510 |
| HRPEpiC | 1.1679476 | 0.09690 |
| AG09319 | 1.2058194 | 0.09770 |
| NHA | 1.1800351 | 0.10064 |
| HGF | 1.2075827 | 0.10186 |
| HAc | 1.1699311 | 0.10254 |
| GM12865 | 1.2214457 | 0.10444 |
| HMVECdBlNeo | 1.1886331 | 0.11088 |
| HRE | 1.1597222 | 0.11106 |
| HFFMyc | 1.1691961 | 0.11274 |
| HRGEC | 1.1824979 | 0.11734 |
| HL60 | 1.2422156 | 0.12110 |
| GM12864 | 1.2140349 | 0.12364 |
| WI38 | 1.1715622 | 0.12896 |
| HMVECdBlAd | 1.1680943 | 0.13592 |
| HMVECLLy | 1.1790146 | 0.13736 |
| CD20 | 1.2409487 | 0.13842 |
| HMVECdLyNeo | 1.1696101 | 0.14214 |
| AG04449 | 1.1581970 | 0.14592 |
| HMVECdLyAd | 1.1705453 | 0.15780 |
| HPF | 1.1489525 | 0.16356 |
| GM12878 | 1.1826476 | 0.16490 |
| HPAEC | 1.1485390 | 0.17392 |
| HAh | 1.1149010 | 0.17952 |
| HConF | 1.1338672 | 0.18950 |
| AG04450 | 1.1349201 | 0.19310 |
| A549 | 1.1579186 | 0.19434 |
| CMK | 1.1773167 | 0.19536 |
| PANC1 | 1.1420299 | 0.20126 |
| HMVECdAd | 1.1380102 | 0.20372 |
| IshikawaEstradiol | 1.1318926 | 0.20586 |
| CLL | 1.1677220 | 0.20636 |
| HMVECdNeo | 1.1320883 | 0.20926 |
| SAEC | 1.1003551 | 0.21364 |
| HCT116 | 1.1280420 | 0.22214 |
| HEEpiC | 1.0966729 | 0.22278 |
| NT2D1 | 1.1054354 | 0.22844 |
| HUVEC | 1.1181258 | 0.23168 |
| PanIsletD | 1.0999641 | 0.24220 |
| GM18507 | 1.1157450 | 0.25852 |
| Myometr | 1.0864909 | 0.27776 |
| IshikawaTamoxifen | 1.0837014 | 0.30288 |
| Gliobla | 1.0908011 | 0.30672 |
| NHEK | 1.0723364 | 0.30790 |
| HeLaS3 | 1.0841696 | 0.31034 |
| Urothelia | 1.0800307 | 0.31252 |
| WERIRb1 | 1.0604545 | 0.33760 |
| HPDE6E6E7 | 1.0654819 | 0.33914 |
| HSMM | 1.0518424 | 0.34070 |
| RWPE1 | 1.0539550 | 0.35496 |
| Th0 | 1.0567843 | 0.37290 |
| HeLaS3IFNa4h | 1.0493980 | 0.38446 |
| HSMMemb | 1.0432307 | 0.38948 |
| BE2C | 1.0389536 | 0.40120 |
| GM19238 | 1.0351650 | 0.41566 |
| FibroP | 1.0255016 | 0.42182 |
| SKNSHRA | 1.0297948 | 0.42340 |
| LNCaPAndrogen | 1.0261987 | 0.42834 |
| PrEC | 1.0200239 | 0.43234 |
| GM19240 | 1.0193277 | 0.44880 |
| Th1 | 1.0215748 | 0.44954 |
| H9ES | 1.0096025 | 0.46342 |
| GM19239 | 1.0107012 | 0.46402 |
| H7hESC | 1.0020657 | 0.48442 |
| T47D | 0.9995984 | 0.48656 |
| Stellate | 0.9902633 | 0.51206 |
| GM12891 | 0.9829467 | 0.51844 |
| Melano | 0.9879351 | 0.54004 |
| 8988T | 0.9675682 | 0.54310 |
| MCF7 | 0.9678766 | 0.56258 |
| UrotheliaUT189 | 0.9656450 | 0.56926 |
| ProgFib | 0.9655259 | 0.57542 |
| LNCaP | 0.9601735 | 0.58380 |
| H1hESC | 0.9464409 | 0.59422 |
| MCF7Hypoxia | 0.9476579 | 0.59844 |
| Medullo | 0.9457122 | 0.61826 |
| Huh7 | 0.9229743 | 0.65094 |
| Hepatocytes | 0.9073408 | 0.65842 |
| GM12892 | 0.9103991 | 0.66192 |
| HMEC | 0.9279194 | 0.67722 |
| HSMMtube | 0.9268121 | 0.69898 |
| Fibrobl | 0.9025204 | 0.72518 |
| HepG2 | 0.8859294 | 0.73294 |
| Huh7.5 | 0.8722049 | 0.74524 |
| pHTE | 0.8819706 | 0.76092 |
| Osteobl | 0.8725199 | 0.77818 |
| Caco2 | 0.7792185 | 0.77934 |
| Chorion | 0.8206520 | 0.78992 |
| iPS | 0.7985029 | 0.82572 |
| PanIslets | 0.7889342 | 0.88664 |

# Migraine

| DHS sample | fold enrichment | p value |
|---|---|---|
| BJ | 1.4047393 | 0.03600 |
| AG10803 | 1.4281437 | 0.03720 |
| NHDFneo | 1.4071141 | 0.03904 |
| AG09319 | 1.3792104 | 0.05870 |
| AoAF | 1.3525298 | 0.06180 |
| NHA | 1.3088001 | 0.06702 |
| NHLF | 1.2973864 | 0.06816 |
| WERIRb1 | 1.3036878 | 0.07228 |
| HCM | 1.3127270 | 0.07900 |
| HFF | 1.3160005 | 0.07904 |
| HRPEpiC | 1.2405937 | 0.07924 |
| HNPCEpiC | 1.2642769 | 0.08422 |
| HConF | 1.3181877 | 0.08556 |
| HFFMyc | 1.3095569 | 0.08624 |
| AG09309 | 1.2915007 | 0.08726 |
| HMF | 1.2849090 | 0.09620 |
| HAh | 1.2292178 | 0.10432 |
| HPdLF | 1.2833121 | 0.10688 |
| NHDFAd | 1.2554324 | 0.11510 |
| AoSMC | 1.2533300 | 0.12016 |
| AG04449 | 1.2573962 | 0.12434 |
| HCFaa | 1.2372815 | 0.12826 |
| SKMC | 1.2281766 | 0.13050 |
| H7hESC | 1.1524863 | 0.13112 |
| HGF | 1.2614411 | 0.13364 |
| Fibrobl | 1.2801603 | 0.13702 |
| HCF | 1.2552177 | 0.13804 |
| HIPEpiC | 1.2234626 | 0.14688 |
| Huh7.5 | 1.3102589 | 0.15440 |
| HSMM | 1.2060038 | 0.15638 |
| HPAF | 1.2211019 | 0.15770 |
| CD34Mobilized | 1.2363252 | 0.18900 |
| Huh7 | 1.2595750 | 0.18916 |
| HBMEC | 1.1713380 | 0.18956 |
| pHTE | 1.2191930 | 0.19258 |
| Osteobl | 1.2206321 | 0.19830 |
| ProgFib | 1.2192994 | 0.19934 |
| AG04450 | 1.1944262 | 0.20578 |
| FibroP | 1.1670074 | 0.20796 |
| HAsp | 1.1510059 | 0.21526 |
| Melano | 1.1467886 | 0.21676 |
| HSMMtube | 1.1621134 | 0.22488 |
| NT2D1 | 1.1343086 | 0.22522 |
| HepG2 | 1.2144870 | 0.22624 |
| SAEC | 1.1318856 | 0.24050 |
| Stellate | 1.1892520 | 0.24560 |
| HCPEpiC | 1.1435871 | 0.24636 |
| HMEC | 1.1493642 | 0.25618 |
| Gliobla | 1.1760624 | 0.25742 |
| LNCaP | 1.1452073 | 0.25870 |
| HRCEpiC | 1.1251521 | 0.26566 |
| H1hESC | 1.1587403 | 0.27012 |
| A549 | 1.1606970 | 0.27094 |
| Jurkat | 1.1341741 | 0.27538 |
| Th1 | 1.1668280 | 0.27666 |
| HAEpiC | 1.1257141 | 0.27912 |
| iPS | 1.1727469 | 0.28698 |
| CMK | 1.1569537 | 0.29082 |
| PanIslets | 1.1368601 | 0.29726 |
| CD20 | 1.1704301 | 0.29774 |
| HRE | 1.1008993 | 0.29792 |
| UrotheliaUT189 | 1.1267945 | 0.30406 |
| Th0 | 1.1398518 | 0.30804 |
| MCF7 | 1.1084806 | 0.30908 |
| HL60 | 1.1404626 | 0.31340 |
| CLL | 1.1451508 | 0.31670 |
| HMVECLBl | 1.1059353 | 0.31952 |
| RPTEC | 1.0855145 | 0.32000 |
| PanIsletD | 1.0981958 | 0.32674 |
| H9ES | 1.0980782 | 0.32974 |
| NHEK | 1.0942491 | 0.33490 |
| HVMF | 1.0918075 | 0.33834 |
| SKNSHRA | 1.0984283 | 0.34426 |
| HSMMemb | 1.0942762 | 0.34654 |
| Chorion | 1.1129011 | 0.34722 |
| MCF7Hypoxia | 1.0890289 | 0.35284 |
| HeLaS3IFNa4h | 1.0956056 | 0.35342 |
| WI38 | 1.0795117 | 0.35920 |
| HCT116 | 1.0772520 | 0.36728 |
| PrEC | 1.0606437 | 0.36894 |
| HeLaS3 | 1.0720607 | 0.37306 |
| HAc | 1.0492935 | 0.38964 |
| K562 | 1.0693480 | 0.39244 |
| 8988T | 1.0647095 | 0.39694 |
| HEEpiC | 1.0448234 | 0.40056 |
| Hepatocytes | 1.0531042 | 0.40906 |
| HRGEC | 1.0438416 | 0.41766 |
| T47D | 1.0401506 | 0.42226 |
| Myometr | 1.0336661 | 0.42788 |
| HPF | 1.0340546 | 0.42926 |
| Th2 | 1.0393711 | 0.43300 |
| Urothelia | 1.0279198 | 0.43884 |
| NB4 | 1.0290218 | 0.44052 |
| GM12892 | 1.0259418 | 0.44800 |
| RWPE1 | 1.0187418 | 0.45082 |
| GM12891 | 1.0032721 | 0.46938 |
| HMVECdLyNeo | 1.0106710 | 0.46976 |
| HMVECdBlNeo | 1.0093124 | 0.47274 |
| MonocytesCD14RO01746 | 1.0020332 | 0.47344 |
| HPDE6E6E7 | 1.0055089 | 0.47372 |
| HMVECdBlAd | 1.0093787 | 0.47530 |
| IshikawaEstradiol | 1.0006307 | 0.47680 |
| Caco2 | 0.9771840 | 0.47884 |
| LNCaPAndrogen | 0.9949793 | 0.48796 |
| GM18507 | 0.9927478 | 0.48884 |
| HTR8svn | 0.9929313 | 0.49244 |
| HMVECdNeo | 0.9850144 | 0.50694 |
| HMVECLLy | 0.9862139 | 0.50744 |
| IshikawaTamoxifen | 0.9813647 | 0.50776 |
| GM19239 | 0.9608220 | 0.51932 |
| GM19240 | 0.9688012 | 0.52560 |
| HPAEC | 0.9742707 | 0.53168 |
| HUVEC | 0.9621354 | 0.54132 |
| HMVECdAd | 0.9590362 | 0.54622 |
| PANC1 | 0.9497222 | 0.55040 |
| BE2C | 0.9329024 | 0.58238 |
| GM19238 | 0.9187968 | 0.58266 |
| SKNMC | 0.9543461 | 0.58284 |
| HMVECdLyAd | 0.9344579 | 0.58708 |
| GM12878 | 0.8909440 | 0.61458 |
| Medullo | 0.8936327 | 0.63854 |
| GM12864 | 0.8193367 | 0.71974 |
| GM06990 | 0.7902516 | 0.72734 |
| GM12865 | 0.7912718 | 0.77574 |

# Atherosclerosis

| DHS sample | fold enrichment | p value |
|---|---|---|
| RPTEC | 1.2149987 | 0.00848 |
| HRCEpiC | 1.2223384 | 0.00976 |
| HCFaa | 1.2308655 | 0.01100 |
| HBMEC | 1.1722081 | 0.03444 |
| HRE | 1.1655855 | 0.03502 |
| HAEpiC | 1.1832580 | 0.03868 |
| HAsp | 1.1730164 | 0.03948 |
| AoAF | 1.1709087 | 0.05344 |
| HGF | 1.1803346 | 0.05352 |
| HPAF | 1.1614880 | 0.05512 |
| HPdLF | 1.1706086 | 0.05946 |
| HCM | 1.1448194 | 0.06758 |
| AoSMC | 1.1399470 | 0.07020 |
| HIPEpiC | 1.1356318 | 0.07386 |
| HEEpiC | 1.1285733 | 0.07772 |
| NHA | 1.1406465 | 0.07786 |
| SAEC | 1.1237946 | 0.07904 |
| HRGEC | 1.1473597 | 0.08460 |
| AG09319 | 1.1514112 | 0.08732 |
| HCF | 1.1443165 | 0.08894 |
| SKMC | 1.1294817 | 0.09120 |
| NHLF | 1.1271141 | 0.09404 |
| HNPCEpiC | 1.1136334 | 0.10252 |
| HPAEC | 1.1395417 | 0.10660 |
| HCPEpiC | 1.1159060 | 0.11060 |
| HMF | 1.1203602 | 0.11812 |
| HConF | 1.1256892 | 0.11898 |
| HRPEpiC | 1.1024426 | 0.12352 |
| HPDE6E6E7 | 1.1333678 | 0.12596 |
| PrEC | 1.0971454 | 0.13732 |
| HAc | 1.0973592 | 0.14794 |
| AG04449 | 1.1082434 | 0.15198 |
| WI38 | 1.1093116 | 0.15746 |
| BJ | 1.1064468 | 0.15868 |
| MonocytesCD14RO01746 | 1.1354902 | 0.16202 |
| HL60 | 1.1267419 | 0.17416 |
| HepG2 | 1.1159091 | 0.17734 |
| HAh | 1.0749041 | 0.19938 |
| Myometr | 1.0924477 | 0.20004 |
| HMVECdBlAd | 1.0916848 | 0.20136 |
| HPF | 1.0871326 | 0.20180 |
| HMVECLBl | 1.0882825 | 0.20372 |
| AG04450 | 1.0889123 | 0.20896 |
| K562 | 1.1044456 | 0.20944 |
| NHDFAd | 1.0794559 | 0.20950 |
| HTR8svn | 1.0942400 | 0.20990 |
| A549 | 1.1028133 | 0.21024 |
| HMVECdBlNeo | 1.0875857 | 0.21238 |
| GM12864 | 1.0995075 | 0.21360 |
| PANC1 | 1.0939119 | 0.21494 |
| AG09309 | 1.0779341 | 0.21510 |
| Stellate | 1.0960360 | 0.21786 |
| Huh7 | 1.0938454 | 0.23034 |
| HFFMyc | 1.0737372 | 0.23078 |
| NHEK | 1.0778905 | 0.23482 |
| GM12865 | 1.0807122 | 0.25068 |
| HVMF | 1.0730888 | 0.25226 |
| NHDFneo | 1.0694460 | 0.25378 |
| HFF | 1.0668706 | 0.25546 |
| RWPE1 | 1.0715622 | 0.25854 |
| NB4 | 1.0740716 | 0.27226 |
| IshikawaEstradiol | 1.0649936 | 0.27908 |
| H7hESC | 1.0454109 | 0.27986 |
| AG10803 | 1.0603239 | 0.28598 |
| HMVECdAd | 1.0651197 | 0.28648 |
| Jurkat | 1.0615295 | 0.28726 |
| HeLaS3 | 1.0585087 | 0.30992 |
| Melano | 1.0434576 | 0.31282 |
| HMVECdLyNeo | 1.0511688 | 0.31806 |
| GM12878 | 1.0552328 | 0.32968 |
| PanIsletD | 1.0453006 | 0.33044 |
| CD34Mobilized | 1.0487751 | 0.33802 |
| FibroP | 1.0397580 | 0.33820 |
| HSMM | 1.0377626 | 0.34676 |
| HMVECdNeo | 1.0423519 | 0.35314 |
| HeLaS3IFNa4h | 1.0420587 | 0.36776 |
| Huh7.5 | 1.0401008 | 0.37468 |
| HMVECLLy | 1.0334008 | 0.38112 |
| HUVEC | 1.0288381 | 0.39746 |
| IshikawaTamoxifen | 1.0277813 | 0.40112 |
| HSMMemb | 1.0230015 | 0.41956 |
| HMVECdLyAd | 1.0214837 | 0.41960 |
| CD20 | 1.0223927 | 0.43240 |
| ProgFib | 1.0180327 | 0.43436 |
| NT2D1 | 1.0146260 | 0.43474 |
| GM18507 | 1.0187330 | 0.43622 |
| Hepatocytes | 1.0120774 | 0.45768 |
| PanIslets | 1.0096730 | 0.46176 |
| GM06990 | 1.0084671 | 0.46796 |
| Urothelia | 1.0017296 | 0.48720 |
| HCT116 | 1.0013348 | 0.48780 |
| CMK | 0.9931568 | 0.50928 |
| UrotheliaUT189 | 0.9949913 | 0.51208 |
| GM19238 | 0.9902488 | 0.52346 |
| LNCaPAndrogen | 0.9886539 | 0.52812 |
| HSMMtube | 0.9904644 | 0.53770 |
| CLL | 0.9835452 | 0.54076 |
| HMEC | 0.9833824 | 0.55336 |
| GM19239 | 0.9781966 | 0.55626 |
| H9ES | 0.9800069 | 0.55632 |
| Th0 | 0.9810291 | 0.55868 |
| GM19240 | 0.9791820 | 0.56432 |
| Medullo | 0.9790029 | 0.56528 |
| MCF7 | 0.9762160 | 0.57214 |
| pHTE | 0.9775091 | 0.57456 |
| Fibrobl | 0.9768277 | 0.58144 |
| Osteobl | 0.9740833 | 0.58806 |
| Caco2 | 0.9425675 | 0.61348 |
| 8988T | 0.9503868 | 0.62162 |
| BE2C | 0.9621725 | 0.62196 |
| Gliobla | 0.9518773 | 0.63596 |
| Th1 | 0.9557046 | 0.64584 |
| GM12891 | 0.9419416 | 0.65312 |
| WERIRb1 | 0.9532098 | 0.66728 |
| Th2 | 0.9365400 | 0.67370 |
| GM12892 | 0.9323710 | 0.67860 |
| SKNSHRA | 0.9275811 | 0.68394 |
| LNCaP | 0.9428525 | 0.68932 |
| H1hESC | 0.9227762 | 0.71514 |
| T47D | 0.9171858 | 0.71840 |
| MCF7Hypoxia | 0.9156971 | 0.72756 |
| iPS | 0.9032578 | 0.74294 |
| Chorion | 0.9012729 | 0.74322 |
| SKNMC | 0.8997082 | 0.82910 |

## Cardiovascular−related traits

| DHS sample | fold enrichment | p value |
|---|---|---|
| RPTEC | 1.3244948 | 0.00872 |
| HRCEpiC | 1.3274087 | 0.00948 |
| HCM | 1.1899959 | 0.08674 |
| HRE | 1.1737152 | 0.10344 |
| HL60 | 1.2270684 | 0.13640 |
| HCFaa | 1.1522307 | 0.13878 |
| MonocytesCD14RO01746 | 1.2144845 | 0.14504 |
| Jurkat | 1.1626260 | 0.15278 |
| HPdLF | 1.1541006 | 0.15462 |
| HPAF | 1.1459089 | 0.15744 |
| HGF | 1.1576051 | 0.16168 |
| HepG2 | 1.1767785 | 0.16962 |
| GM12865 | 1.1712659 | 0.17280 |
| HPAEC | 1.1467847 | 0.17768 |
| HMVECdLyAd | 1.1496035 | 0.18422 |
| HRGEC | 1.1297203 | 0.18630 |
| AoSMC | 1.1130674 | 0.19804 |
| HMVECdAd | 1.1361428 | 0.21042 |
| HEEpiC | 1.1078069 | 0.21092 |
| HCF | 1.1210122 | 0.21100 |
| HMVECdNeo | 1.1314332 | 0.21206 |
| HMVECLLy | 1.1298674 | 0.21284 |
| SAEC | 1.1007007 | 0.21926 |
| CMK | 1.1579866 | 0.22224 |
| GM12878 | 1.1453414 | 0.22324 |
| NB4 | 1.1352228 | 0.22432 |
| K562 | 1.1401952 | 0.23304 |
| HMVECdBlAd | 1.1026365 | 0.24090 |
| HMVECLBl | 1.1027190 | 0.24306 |
| HIPEpiC | 1.0891132 | 0.24980 |
| HMVECdLyNeo | 1.1018084 | 0.25538 |
| GM12864 | 1.1186607 | 0.25940 |
| HPF | 1.0942565 | 0.26056 |
| HNPCEpiC | 1.0831879 | 0.26068 |
| PrEC | 1.0826041 | 0.26578 |
| HVMF | 1.0944782 | 0.27350 |
| HAEpiC | 1.0883923 | 0.27462 |
| HMVECdBlNeo | 1.0884352 | 0.27784 |
| IshikawaEstradiol | 1.0932723 | 0.28700 |
| WI38 | 1.0844000 | 0.29074 |
| AG09319 | 1.0807283 | 0.29866 |
| Th2 | 1.0970814 | 0.30950 |
| HCPEpiC | 1.0661505 | 0.31234 |
| BJ | 1.0695147 | 0.31978 |
| FibroP | 1.0616340 | 0.32050 |
| HeLaS3 | 1.0789441 | 0.32298 |
| BE2C | 1.0798827 | 0.32376 |
| AG04450 | 1.0632412 | 0.33592 |
| AoAF | 1.0599068 | 0.33892 |
| NHEK | 1.0561979 | 0.35494 |
| HAc | 1.0473985 | 0.35994 |
| AG10803 | 1.0507354 | 0.36538 |
| HMF | 1.0467290 | 0.36936 |
| GM06990 | 1.0618961 | 0.37256 |
| IshikawaTamoxifen | 1.0515389 | 0.37782 |
| HAh | 1.0376402 | 0.38356 |
| HeLaS3IFNa4h | 1.0512134 | 0.38378 |
| HConF | 1.0430963 | 0.38414 |
| AG09309 | 1.0406197 | 0.38508 |
| HTR8svn | 1.0462861 | 0.38816 |
| Stellate | 1.0462501 | 0.39354 |
| PanIsletD | 1.0357317 | 0.39946 |
| A549 | 1.0385609 | 0.40906 |
| Th0 | 1.0405916 | 0.40936 |
| Melano | 1.0229502 | 0.42592 |
| GM18507 | 1.0308872 | 0.42600 |
| GM19240 | 1.0290300 | 0.42842 |
| HPDE6E6E7 | 1.0281000 | 0.43002 |
| NHLF | 1.0213889 | 0.43358 |
| NHA | 1.0203376 | 0.43990 |
| CD34Mobilized | 1.0211786 | 0.44230 |
| HSMM | 1.0119334 | 0.46144 |
| HAsp | 1.0078685 | 0.47056 |
| UrotheliaUT189 | 1.0071554 | 0.47376 |
| NHDFneo | 1.0057209 | 0.47800 |
| Th1 | 1.0088656 | 0.47922 |
| SKMC | 1.0054369 | 0.48030 |
| WERIRb1 | 1.0051180 | 0.48082 |
| HRPEpiC | 1.0042451 | 0.48094 |
| Urothelia | 1.0031298 | 0.48676 |
| HFF | 1.0021551 | 0.48972 |
| GM19238 | 0.9977406 | 0.49436 |
| HSMMtube | 0.9983589 | 0.49804 |
| AG04449 | 0.9968218 | 0.50488 |
| HCT116 | 0.9921834 | 0.50546 |
| HSMMemb | 0.9944613 | 0.51150 |
| Myometr | 0.9897994 | 0.51648 |
| HBMEC | 0.9912702 | 0.52186 |
| RWPE1 | 0.9768299 | 0.54514 |
| GM12891 | 0.9688670 | 0.54686 |
| Caco2 | 0.9483301 | 0.54800 |
| CLL | 0.9683641 | 0.54952 |
| GM19239 | 0.9619728 | 0.55660 |
| Huh7.5 | 0.9595337 | 0.56660 |
| MCF7 | 0.9670145 | 0.56672 |
| CD20 | 0.9468096 | 0.58244 |
| HUVEC | 0.9569546 | 0.59042 |
| PanIslets | 0.9554623 | 0.59200 |
| Fibrobl | 0.9567177 | 0.60310 |
| HFFMyc | 0.9632932 | 0.60312 |
| GM12892 | 0.9395522 | 0.60384 |
| PANC1 | 0.9509913 | 0.60576 |
| NHDFAd | 0.9579439 | 0.61178 |
| HMEC | 0.9524688 | 0.61300 |
| Huh7 | 0.9373776 | 0.61828 |
| MCF7Hypoxia | 0.9370861 | 0.62172 |
| Hepatocytes | 0.9255058 | 0.62420 |
| ProgFib | 0.9286792 | 0.65246 |
| pHTE | 0.9302841 | 0.65372 |
| Osteobl | 0.9308079 | 0.65694 |
| LNCaPAndrogen | 0.9088892 | 0.67618 |
| NT2D1 | 0.9339735 | 0.67680 |
| LNCaP | 0.9171167 | 0.68158 |
| SKNMC | 0.9224970 | 0.68328 |
| Medullo | 0.9041663 | 0.69842 |
| 8988T | 0.8613768 | 0.72598 |
| Gliobla | 0.8763500 | 0.72750 |
| H7hESC | 0.9259022 | 0.74632 |
| iPS | 0.8488546 | 0.74914 |
| T47D | 0.8376592 | 0.77688 |
| H9ES | 0.8500203 | 0.79438 |
| Chorion | 0.8070102 | 0.80558 |
| H1hESC | 0.8264790 | 0.80830 |
| SKNSHRA | 0.8039457 | 0.81052 |

# Polygenic dyslipidemia

| DHS sample | fold enrichment | p value |
|---|---|---|
| RPTEC | 1.2010228 | 0.00976 |
| Jurkat | 1.2179074 | 0.01954 |
| HRCEpiC | 1.1801438 | 0.02412 |
| HL60 | 1.2178726 | 0.04750 |
| HepG2 | 1.1763774 | 0.06734 |
| HCFaa | 1.1344658 | 0.06766 |
| MonocytesCD14RO01746 | 1.1626970 | 0.10272 |
| HRE | 1.1085130 | 0.10854 |
| HEEpiC | 1.1078351 | 0.11128 |
| NB4 | 1.1377347 | 0.11310 |
| HCPEpiC | 1.1020756 | 0.12296 |
| H7hESC | 1.0837074 | 0.12644 |
| GM12864 | 1.1342940 | 0.12688 |
| HPAF | 1.1059327 | 0.12732 |
| NT2D1 | 1.0967734 | 0.14248 |
| HTR8svn | 1.1164465 | 0.14286 |
| HeLaS3 | 1.1232460 | 0.14790 |
| HeLaS3IFNa4h | 1.1322438 | 0.14826 |
| SAEC | 1.0908064 | 0.14890 |
| GM12865 | 1.1173831 | 0.15042 |
| HIPEpiC | 1.0907123 | 0.15176 |
| HGF | 1.1058992 | 0.15546 |
| PrEC | 1.0813438 | 0.17204 |
| HNPCEpiC | 1.0791691 | 0.17754 |
| HAEpiC | 1.0905390 | 0.17780 |
| HCM | 1.0797275 | 0.18754 |
| HPDE6E6E7 | 1.0980845 | 0.18918 |
| IshikawaEstradiol | 1.0921359 | 0.19114 |
| PANC1 | 1.0973215 | 0.19374 |
| HMVECLBl | 1.0840256 | 0.19582 |
| HMVECdAd | 1.0934199 | 0.19656 |
| HCF | 1.0830939 | 0.19814 |
| Huh7.5 | 1.1039231 | 0.20250 |
| A549 | 1.0986094 | 0.20290 |
| GM12878 | 1.0945663 | 0.21310 |
| HPF | 1.0780686 | 0.21900 |
| Stellate | 1.0866939 | 0.22014 |
| WI38 | 1.0791688 | 0.22198 |
| RWPE1 | 1.0799432 | 0.22298 |
| Huh7 | 1.0906965 | 0.22924 |
| HVMF | 1.0746890 | 0.23844 |
| HMF | 1.0662044 | 0.24018 |
| AG04449 | 1.0678390 | 0.24182 |
| AG04450 | 1.0709177 | 0.24362 |
| NHEK | 1.0705936 | 0.24450 |
| Medullo | 1.0788431 | 0.24628 |
| HAh | 1.0565009 | 0.25218 |
| K562 | 1.0780150 | 0.25312 |
| FibroP | 1.0560672 | 0.25320 |
| IshikawaTamoxifen | 1.0681963 | 0.26350 |
| HPAEC | 1.0633670 | 0.26568 |
| HBMEC | 1.0555402 | 0.26594 |
| Melano | 1.0502781 | 0.26642 |
| Th1 | 1.0656680 | 0.26856 |
| AoSMC | 1.0531469 | 0.26936 |
| HCT116 | 1.0675327 | 0.27280 |
| SKNMC | 1.0620961 | 0.27334 |
| PanIsletD | 1.0552067 | 0.27990 |
| SKMC | 1.0522178 | 0.28508 |
| HConF | 1.0557434 | 0.28622 |
| HRGEC | 1.0533579 | 0.28780 |
| NHA | 1.0496303 | 0.29302 |
| GM19240 | 1.0555903 | 0.30074 |
| WERIRb1 | 1.0504383 | 0.30656 |
| HMVECdLyNeo | 1.0502043 | 0.30906 |
| Th2 | 1.0598221 | 0.31350 |
| HPdLF | 1.0478253 | 0.31748 |
| GM06990 | 1.0610345 | 0.31858 |
| HSMMemb | 1.0487915 | 0.32008 |
| NHLF | 1.0397186 | 0.33086 |
| HMVECdNeo | 1.0452254 | 0.33118 |
| Th0 | 1.0477755 | 0.33296 |
| HMVECdBlAd | 1.0418484 | 0.33352 |
| AoAF | 1.0360272 | 0.35490 |
| CMK | 1.0451586 | 0.35986 |
| Caco2 | 1.0555044 | 0.36182 |
| HMVECLLy | 1.0367155 | 0.36252 |
| Fibrobl | 1.0352643 | 0.36478 |
| Osteobl | 1.0339721 | 0.37402 |
| GM18507 | 1.0335614 | 0.38060 |
| BE2C | 1.0324269 | 0.38530 |
| HMVECdLyAd | 1.0304419 | 0.38536 |
| HAc | 1.0250980 | 0.38846 |
| HMVECdBlNeo | 1.0269730 | 0.39172 |
| GM19238 | 1.0302595 | 0.39368 |
| HSMM | 1.0231014 | 0.39660 |
| HFFMyc | 1.0231476 | 0.39832 |
| Hepatocytes | 1.0302389 | 0.40124 |
| AG10803 | 1.0238042 | 0.40206 |
| MCF7 | 1.0244596 | 0.40780 |
| CD34Mobilized | 1.0244297 | 0.40844 |
| HSMMtube | 1.0206458 | 0.41082 |
| Gliobla | 1.0259859 | 0.41206 |
| Myometr | 1.0186262 | 0.42034 |
| BJ | 1.0187161 | 0.42134 |
| Urothelia | 1.0172473 | 0.43564 |
| AG09309 | 1.0137671 | 0.43882 |
| HAsp | 1.0095643 | 0.45368 |
| PanIslets | 1.0119750 | 0.45576 |
| NHDFAd | 1.0094781 | 0.45738 |
| UrotheliaUT189 | 1.0089557 | 0.46328 |
| ProgFib | 1.0071038 | 0.46952 |
| HFF | 1.0070854 | 0.47050 |
| HMEC | 1.0064589 | 0.47214 |
| AG09319 | 1.0039185 | 0.48350 |
| GM19239 | 1.0016422 | 0.48556 |
| GM12892 | 0.9953546 | 0.50900 |
| LNCaPAndrogen | 0.9935644 | 0.51318 |
| HRPEpiC | 0.9963773 | 0.51420 |
| CD20 | 0.9908896 | 0.51768 |
| pHTE | 0.9939802 | 0.51934 |
| GM12891 | 0.9848927 | 0.54016 |
| CLL | 0.9817857 | 0.55104 |
| 8988T | 0.9773605 | 0.55506 |
| iPS | 0.9669844 | 0.58772 |
| NHDFneo | 0.9767412 | 0.59084 |
| H9ES | 0.9707323 | 0.59186 |
| HUVEC | 0.9716148 | 0.59814 |
| SKNSHRA | 0.9553651 | 0.60362 |
| MCF7Hypoxia | 0.9600776 | 0.61742 |
| LNCaP | 0.9482753 | 0.67804 |
| T47D | 0.9331330 | 0.68756 |
| Chorion | 0.9260457 | 0.69490 |
| H1hESC | 0.9279639 | 0.70804 |

## Primary tooth eruption

| DHS sample | fold enrichment | p value |
|---|---|---|
| HAsp | 1.3975579 | 0.00978 |
| NHDFneo | 1.3658598 | 0.02236 |
| HPdLF | 1.3489234 | 0.03416 |
| AG09309 | 1.2900266 | 0.04374 |
| NHDFAd | 1.2799482 | 0.04576 |
| SKMC | 1.2511545 | 0.06462 |
| BJ | 1.2739558 | 0.06616 |
| HFFMyc | 1.2479335 | 0.07610 |
| HNPCEpiC | 1.2173413 | 0.07790 |
| HCFaa | 1.2464560 | 0.07922 |
| HRPEpiC | 1.2008949 | 0.07922 |
| HRGEC | 1.2578742 | 0.08228 |
| HIPEpiC | 1.2249526 | 0.08410 |
| HPF | 1.2436930 | 0.08620 |
| AG09319 | 1.2556577 | 0.08896 |
| AoAF | 1.2380695 | 0.09092 |
| HAc | 1.1942874 | 0.10044 |
| HFF | 1.2098789 | 0.11002 |
| HCT116 | 1.2546776 | 0.11036 |
| HGF | 1.2246568 | 0.11104 |
| HAEpiC | 1.2154127 | 0.11130 |
| NHA | 1.1909742 | 0.11964 |
| HVMF | 1.2211704 | 0.12432 |
| NHLF | 1.1825400 | 0.12624 |
| AG04450 | 1.2032211 | 0.13480 |
| AG04449 | 1.1931433 | 0.13704 |
| HAh | 1.1556932 | 0.14174 |
| AoSMC | 1.1731509 | 0.14314 |
| HBMEC | 1.1569865 | 0.16260 |
| HMF | 1.1628127 | 0.16510 |
| AG10803 | 1.1638944 | 0.18192 |
| WI38 | 1.1615671 | 0.19672 |
| HCPEpiC | 1.1348481 | 0.20344 |
| HMVECLBl | 1.1560887 | 0.20372 |
| HConF | 1.1313932 | 0.22836 |
| SKNMC | 1.1139446 | 0.24984 |
| SAEC | 1.1019327 | 0.25288 |
| HRCEpiC | 1.1062465 | 0.25346 |
| HCF | 1.1159325 | 0.25416 |
| HMVECdBlAd | 1.1199901 | 0.25582 |
| HCM | 1.0904258 | 0.28908 |
| HRE | 1.0797055 | 0.29854 |
| HPAF | 1.0829799 | 0.30934 |
| HPAEC | 1.0865469 | 0.31940 |
| HMVECdBlNeo | 1.0861589 | 0.31962 |
| HMVECdLyNeo | 1.0825889 | 0.32968 |
| RPTEC | 1.0526531 | 0.36434 |
| HUVEC | 1.0588302 | 0.37286 |
| HEEpiC | 1.0402775 | 0.39072 |
| HSMM | 1.0346951 | 0.40766 |
| PrEC | 1.0285834 | 0.42268 |
| NHEK | 1.0239102 | 0.44014 |
| PANC1 | 1.0226454 | 0.44386 |
| HSMMemb | 1.0204347 | 0.45056 |
| GM12865 | 1.0154895 | 0.45876 |
| HMVECdAd | 1.0083516 | 0.47622 |
| IshikawaEstradiol | 1.0034535 | 0.47974 |
| Myometr | 1.0026791 | 0.48394 |
| Jurkat | 0.9917536 | 0.50846 |
| MCF7Hypoxia | 0.9784919 | 0.51496 |
| Stellate | 0.9842938 | 0.51870 |
| WERIRb1 | 0.9848601 | 0.52584 |
| NB4 | 0.9796281 | 0.52900 |
| MCF7 | 0.9723147 | 0.53042 |
| Huh7 | 0.9688588 | 0.54134 |
| HMVECLLy | 0.9756788 | 0.54336 |
| MonocytesCD14RO01746 | 0.9661989 | 0.54488 |
| HMVECdNeo | 0.9717758 | 0.54552 |
| FibroP | 0.9792303 | 0.55226 |
| HL60 | 0.9497396 | 0.57094 |
| HPDE6E6E7 | 0.9603126 | 0.57196 |
| IshikawaTamoxifen | 0.9535715 | 0.58442 |
| CD34Mobilized | 0.9468843 | 0.59022 |
| ProgFib | 0.9491770 | 0.59126 |
| HepG2 | 0.9352453 | 0.61044 |
| A549 | 0.9235280 | 0.62490 |
| K562 | 0.9100684 | 0.64548 |
| GM06990 | 0.9041821 | 0.64580 |
| HMVECdLyAd | 0.9216956 | 0.64644 |
| GM12864 | 0.9060523 | 0.65542 |
| HTR8svn | 0.9146030 | 0.66274 |
| Melano | 0.9346244 | 0.66756 |
| T47D | 0.8889944 | 0.67362 |
| PanIsletD | 0.9048537 | 0.70460 |
| HSMMtube | 0.9035883 | 0.71432 |
| CD20 | 0.8479177 | 0.71790 |
| UrotheliaUT189 | 0.8688627 | 0.72724 |
| GM12878 | 0.8506912 | 0.73880 |
| Urothelia | 0.8634323 | 0.74480 |
| RWPE1 | 0.8705807 | 0.75538 |
| Th2 | 0.8317818 | 0.75694 |
| Huh7.5 | 0.8327656 | 0.76578 |
| BE2C | 0.8454729 | 0.78596 |
| HeLaS3 | 0.8347289 | 0.78766 |
| GM19239 | 0.8062082 | 0.79040 |
| GM18507 | 0.8172291 | 0.79386 |
| GM19238 | 0.8082496 | 0.79708 |
| SKNSHRA | 0.7988156 | 0.79750 |
| GM19240 | 0.8274296 | 0.80108 |
| HeLaS3IFNa4h | 0.8089142 | 0.80222 |
| Gliobla | 0.8083982 | 0.80684 |
| CMK | 0.7908784 | 0.81164 |
| Caco2 | 0.7184460 | 0.82104 |
| CLL | 0.7764895 | 0.82142 |
| H9ES | 0.8153457 | 0.82772 |
| pHTE | 0.7987759 | 0.84954 |
| H7hESC | 0.8881137 | 0.85078 |
| Fibrobl | 0.7993543 | 0.85330 |
| HMEC | 0.8023894 | 0.86880 |
| PanIslets | 0.7637150 | 0.87198 |
| GM12891 | 0.7245301 | 0.87382 |
| Medullo | 0.7541104 | 0.87666 |
| Hepatocytes | 0.7222460 | 0.87896 |
| Chorion | 0.6948824 | 0.89170 |
| LNCaPAndrogen | 0.7321273 | 0.89384 |
| NT2D1 | 0.8163474 | 0.90074 |
| LNCaP | 0.7657263 | 0.90482 |
| Th0 | 0.7214311 | 0.90684 |
| GM12892 | 0.6828755 | 0.90776 |
| Osteobl | 0.7353062 | 0.91372 |
| H1hESC | 0.7217395 | 0.91434 |
| Th1 | 0.7183766 | 0.91486 |
| iPS | 0.6560855 | 0.92608 |
| 8988T | 0.6315391 | 0.93920 |

# Thyroid−related traits

| DHS sample | fold enrichment | p value |
|---|---|---|
| RPTEC | 1.2617844 | 0.01318 |
| FibroP | 1.2761033 | 0.01344 |
| HRCEpiC | 1.2707083 | 0.01576 |
| SKNSHRA | 1.4351833 | 0.01862 |
| AoSMC | 1.2712956 | 0.01960 |
| BE2C | 1.3229873 | 0.02030 |
| NHDFneo | 1.2929852 | 0.02036 |
| SAEC | 1.2338640 | 0.02918 |
| PanIsletD | 1.2610685 | 0.02938 |
| HSMM | 1.2255379 | 0.03452 |
| HConF | 1.2538539 | 0.03904 |
| Myometr | 1.2504805 | 0.03918 |
| AoAF | 1.2617630 | 0.03946 |
| HRE | 1.2144574 | 0.04094 |
| Stellate | 1.3021073 | 0.04252 |
| HCF | 1.2519213 | 0.04526 |
| PrEC | 1.2063583 | 0.04772 |
| HGF | 1.2515392 | 0.04866 |
| HAh | 1.1907103 | 0.04882 |
| NHDFAd | 1.2183405 | 0.04900 |
| AG09319 | 1.2410422 | 0.05470 |
| RWPE1 | 1.2390324 | 0.05694 |
| Melano | 1.1845949 | 0.05920 |
| AG09309 | 1.2136492 | 0.05928 |
| Gliobla | 1.2768013 | 0.06262 |
| HCPEpiC | 1.2018951 | 0.06344 |
| HNPCEpiC | 1.1826202 | 0.06530 |
| ProgFib | 1.2387205 | 0.06794 |
| HSMMtube | 1.1997388 | 0.06802 |
| HMEC | 1.2197892 | 0.06962 |
| SKMC | 1.1961641 | 0.07228 |
| HCFaa | 1.2026851 | 0.07248 |
| HRPEpiC | 1.1614373 | 0.07248 |
| AG10803 | 1.2188221 | 0.07270 |
| HCM | 1.1958137 | 0.07396 |
| HEEpiC | 1.1732370 | 0.07404 |
| H7hESC | 1.1308453 | 0.07724 |
| PanIslets | 1.2457756 | 0.07840 |
| HPDE6E6E7 | 1.2237914 | 0.07912 |
| HPF | 1.1989846 | 0.08076 |
| NHEK | 1.2042537 | 0.08356 |
| pHTE | 1.2202003 | 0.08494 |
| LNCaPAndrogen | 1.2447661 | 0.08688 |
| IshikawaEstradiol | 1.2033068 | 0.08896 |
| WERIRb1 | 1.1769430 | 0.09070 |
| AG04449 | 1.1900910 | 0.09084 |
| IshikawaTamoxifen | 1.2111710 | 0.09156 |
| HFF | 1.1811579 | 0.09372 |
| LNCaP | 1.1943281 | 0.09456 |
| Hepatocytes | 1.2623629 | 0.09700 |
| HSMMemb | 1.2000876 | 0.10396 |
| HPAF | 1.1676681 | 0.10952 |
| Fibrobl | 1.1970134 | 0.11082 |
| HMF | 1.1678382 | 0.11110 |
| HPdLF | 1.1789925 | 0.11198 |
| 8988T | 1.2480877 | 0.12016 |
| HepG2 | 1.2052635 | 0.12024 |
| HBMEC | 1.1459951 | 0.12118 |
| AG04450 | 1.1741834 | 0.12306 |
| HAc | 1.1398987 | 0.12724 |
| HMVECdBlAd | 1.1692483 | 0.13308 |
| BJ | 1.1566519 | 0.13686 |
| NHA | 1.1433074 | 0.13744 |
| T47D | 1.2050163 | 0.14040 |
| NHLF | 1.1378747 | 0.14404 |
| HMVECdLyAd | 1.1646783 | 0.14678 |
| Urothelia | 1.1643515 | 0.15232 |
| HUVEC | 1.1553306 | 0.15320 |
| NT2D1 | 1.1213936 | 0.15336 |
| Osteobl | 1.1681660 | 0.15392 |
| A549 | 1.1705991 | 0.15764 |
| H9ES | 1.1572771 | 0.15848 |
| Medullo | 1.1726403 | 0.15904 |
| HFFMyc | 1.1333422 | 0.16128 |
| HeLaS3IFNa4h | 1.1772443 | 0.16568 |
| Huh7 | 1.1696638 | 0.16954 |
| UrotheliaUT189 | 1.1519916 | 0.17290 |
| WI38 | 1.1401735 | 0.17688 |
| HMVECdBlNeo | 1.1388944 | 0.18128 |
| HMVECdLyNeo | 1.1394486 | 0.18396 |
| HVMF | 1.1369255 | 0.18750 |
| HAEpiC | 1.1202871 | 0.19446 |
| H1hESC | 1.1450323 | 0.19864 |
| iPS | 1.1733759 | 0.20050 |
| HeLaS3 | 1.1319372 | 0.20438 |
| HAsp | 1.1024381 | 0.20842 |
| HIPEpiC | 1.1025760 | 0.21432 |
| CMK | 1.1433430 | 0.21792 |
| Huh7.5 | 1.1363996 | 0.22400 |
| MCF7 | 1.1118083 | 0.22876 |
| HTR8svn | 1.1170912 | 0.22944 |
| GM19239 | 1.1393360 | 0.23076 |
| GM12891 | 1.1479098 | 0.23108 |
| MCF7Hypoxia | 1.1375212 | 0.23138 |
| Chorion | 1.1461605 | 0.23386 |
| HMVECdNeo | 1.1089561 | 0.23656 |
| HRGEC | 1.0986735 | 0.24810 |
| HMVECdAd | 1.1053828 | 0.25344 |
| GM12892 | 1.1348711 | 0.25352 |
| GM19240 | 1.1091296 | 0.25760 |
| Th0 | 1.1110499 | 0.26096 |
| HMVECLLy | 1.0940588 | 0.26914 |
| GM19238 | 1.1078809 | 0.27320 |
| K562 | 1.1000670 | 0.28550 |
| HMVECLBl | 1.0799005 | 0.29202 |
| GM12878 | 1.0916952 | 0.29562 |
| HL60 | 1.0962151 | 0.29680 |
| HCT116 | 1.0713004 | 0.32016 |
| Th1 | 1.0663377 | 0.34610 |
| CD34Mobilized | 1.0567249 | 0.35190 |
| HPAEC | 1.0553859 | 0.35370 |
| PANC1 | 1.0547085 | 0.35502 |
| SKNMC | 1.0452850 | 0.35714 |
| GM18507 | 1.0569031 | 0.36564 |
| CLL | 1.0554027 | 0.37842 |
| NB4 | 1.0216361 | 0.44002 |
| Caco2 | 0.9773726 | 0.50654 |
| MonocytesCD14RO01746 | 0.9675823 | 0.55480 |
| GM12865 | 0.9350221 | 0.64774 |
| Jurkat | 0.9292092 | 0.67156 |
| GM12864 | 0.8658013 | 0.78006 |
| Th2 | 0.8501069 | 0.78176 |
| CD20 | 0.8352437 | 0.78464 |
| GM06990 | 0.8090434 | 0.83828 |

# Prostate cancer susceptibility 2

| DHS sample | fold enrichment | p value |
|---|---|---|
| PrEC | 1.3050990 | 0.01042 |
| SAEC | 1.2536299 | 0.02706 |
| HEEpiC | 1.2322128 | 0.03436 |
| HSMM | 1.2158174 | 0.04792 |
| SKMC | 1.1986429 | 0.07832 |
| HCT116 | 1.2194026 | 0.08858 |
| HL60 | 1.2523679 | 0.09756 |
| HVMF | 1.1990364 | 0.10514 |
| NHEK | 1.1835821 | 0.11308 |
| A549 | 1.2100878 | 0.11476 |
| HSMMtube | 1.1583205 | 0.12744 |
| HPDE6E6E7 | 1.1839938 | 0.13214 |
| WI38 | 1.1733820 | 0.13830 |
| HCFaa | 1.1512370 | 0.14732 |
| HPF | 1.1531139 | 0.14912 |
| HRCEpiC | 1.1373866 | 0.14922 |
| LNCaP | 1.1590852 | 0.15596 |
| AG10803 | 1.1531745 | 0.16112 |
| RPTEC | 1.1195793 | 0.16934 |
| HAEpiC | 1.1378754 | 0.17008 |
| HRE | 1.1227586 | 0.17212 |
| HAsp | 1.1320686 | 0.17434 |
| Caco2 | 1.2430075 | 0.19302 |
| AG04449 | 1.1258366 | 0.19816 |
| BJ | 1.1277093 | 0.20064 |
| NHLF | 1.1146247 | 0.20232 |
| HAc | 1.1068650 | 0.20668 |
| CD34Mobilized | 1.1299410 | 0.20770 |
| HMF | 1.1129809 | 0.20990 |
| Myometr | 1.1171416 | 0.20992 |
| NHA | 1.1111666 | 0.21536 |
| Huh7 | 1.1346958 | 0.21702 |
| HFFMyc | 1.1075856 | 0.22072 |
| HBMEC | 1.1008643 | 0.22538 |
| AG04450 | 1.1144802 | 0.22728 |
| HIPEpiC | 1.0944603 | 0.23814 |
| Stellate | 1.1182172 | 0.24140 |
| HCM | 1.0893119 | 0.25606 |
| MonocytesCD14RO01746 | 1.1253692 | 0.25958 |
| HFF | 1.0895402 | 0.26092 |
| HPAF | 1.0893563 | 0.26220 |
| HPdLF | 1.0956626 | 0.27118 |
| HConF | 1.0856497 | 0.27652 |
| HSMMemb | 1.0921481 | 0.27868 |
| HRGEC | 1.0838001 | 0.28546 |
| HMEC | 1.0839039 | 0.28918 |
| HNPCEpiC | 1.0679185 | 0.29332 |
| 8988T | 1.1063356 | 0.29900 |
| HCPEpiC | 1.0691404 | 0.30020 |
| AG09319 | 1.0783938 | 0.30052 |
| NHDFneo | 1.0756418 | 0.30144 |
| HCF | 1.0756407 | 0.30248 |
| AG09309 | 1.0693877 | 0.30722 |
| HepG2 | 1.0845498 | 0.30916 |
| HGF | 1.0766084 | 0.31016 |
| NHDFAd | 1.0653125 | 0.31918 |
| Chorion | 1.0910118 | 0.32230 |
| Huh7.5 | 1.0745980 | 0.32698 |
| ProgFib | 1.0693468 | 0.33048 |
| NB4 | 1.0663295 | 0.34550 |
| HMVECdBlAd | 1.0524116 | 0.35874 |
| CD20 | 1.0662384 | 0.36060 |
| HRPEpiC | 1.0428206 | 0.36388 |
| pHTE | 1.0511850 | 0.36800 |
| HAh | 1.0407544 | 0.37034 |
| AoSMC | 1.0445977 | 0.37184 |
| IshikawaEstradiol | 1.0442230 | 0.37964 |
| RWPE1 | 1.0427477 | 0.37980 |
| HUVEC | 1.0434726 | 0.38060 |
| HMVECLBl | 1.0446628 | 0.38070 |
| SKNSHRA | 1.0491781 | 0.38078 |
| Urothelia | 1.0408708 | 0.39606 |
| GM12891 | 1.0418807 | 0.40390 |
| Hepatocytes | 1.0395639 | 0.40528 |
| PanIsletD | 1.0317216 | 0.40626 |
| AoAF | 1.0302162 | 0.41230 |
| HeLaS3IFNa4h | 1.0200995 | 0.43946 |
| HMVECLLy | 1.0198093 | 0.44376 |
| HMVECdAd | 1.0183638 | 0.45094 |
| GM06990 | 1.0167485 | 0.45372 |
| GM12864 | 1.0127226 | 0.46052 |
| GM12892 | 1.0090619 | 0.47122 |
| CLL | 1.0087963 | 0.47130 |
| IshikawaTamoxifen | 1.0071715 | 0.47284 |
| HMVECdLyNeo | 1.0070451 | 0.47474 |
| UrotheliaUT189 | 1.0067026 | 0.47696 |
| HeLaS3 | 1.0048149 | 0.47776 |
| H1hESC | 0.9969763 | 0.48568 |
| GM19239 | 0.9980969 | 0.48808 |
| CMK | 0.9937199 | 0.49154 |
| Fibrobl | 0.9941887 | 0.50354 |
| HMVECdBlNeo | 0.9958630 | 0.50446 |
| iPS | 0.9878369 | 0.50814 |
| HMVECdLyAd | 0.9897004 | 0.51644 |
| Melano | 0.9912505 | 0.52748 |
| HPAEC | 0.9871246 | 0.52800 |
| Gliobla | 0.9810226 | 0.53006 |
| Th1 | 0.9809662 | 0.54162 |
| GM19240 | 0.9795097 | 0.54326 |
| BE2C | 0.9747877 | 0.54472 |
| Th0 | 0.9767499 | 0.54522 |
| K562 | 0.9663930 | 0.55752 |
| Osteobl | 0.9721978 | 0.55974 |
| GM12878 | 0.9660395 | 0.56590 |
| GM18507 | 0.9648443 | 0.57200 |
| LNCaPAndrogen | 0.9605001 | 0.57648 |
| PanIslets | 0.9613082 | 0.57928 |
| Th2 | 0.9539094 | 0.58412 |
| PANC1 | 0.9557926 | 0.58804 |
| HMVECdNeo | 0.9542834 | 0.60602 |
| FibroP | 0.9629714 | 0.61318 |
| Jurkat | 0.9479676 | 0.62390 |
| GM19238 | 0.9343645 | 0.63132 |
| MCF7 | 0.9198822 | 0.66898 |
| SKNMC | 0.9293286 | 0.67132 |
| HTR8svn | 0.9160010 | 0.69402 |
| GM12865 | 0.9125588 | 0.70264 |
| H9ES | 0.8976343 | 0.72606 |
| NT2D1 | 0.9105611 | 0.74684 |
| H7hESC | 0.9210449 | 0.77784 |
| MCF7Hypoxia | 0.8198570 | 0.81054 |
| T47D | 0.8144187 | 0.83238 |
| Medullo | 0.8204928 | 0.84208 |
| WERIRb1 | 0.6840838 | 0.98876 |

# Stroke and coronary artery disease

| DHS sample | fold enrichment | p value |
|---|---|---|
| HIPEpiC | 1.2987934 | 0.02566 |
| HAEpiC | 1.3216100 | 0.02708 |
| HConF | 1.3115020 | 0.03060 |
| HGF | 1.3475013 | 0.03094 |
| HVMF | 1.3266122 | 0.04206 |
| NHDFneo | 1.2953615 | 0.04266 |
| CD34Mobilized | 1.3078096 | 0.05006 |
| HCM | 1.2396434 | 0.05566 |
| AG09319 | 1.2866329 | 0.05730 |
| RWPE1 | 1.2665837 | 0.06036 |
| NHDFAd | 1.2413825 | 0.06244 |
| HFFMyc | 1.2308001 | 0.06790 |
| AG10803 | 1.2580396 | 0.06934 |
| HFF | 1.2350423 | 0.07216 |
| Huh7 | 1.3019965 | 0.08228 |
| SKNSHRA | 1.3059940 | 0.08866 |
| HMF | 1.2056216 | 0.09372 |
| HCFaa | 1.2079091 | 0.09884 |
| HSMM | 1.1854540 | 0.09892 |
| HSMMemb | 1.2305311 | 0.09972 |
| HCF | 1.2151867 | 0.10022 |
| HNPCEpiC | 1.1843008 | 0.10040 |
| Myometr | 1.2113955 | 0.10056 |
| HPdLF | 1.2243281 | 0.10152 |
| HPAF | 1.1984802 | 0.10532 |
| PANC1 | 1.2295266 | 0.11066 |
| HPDE6E6E7 | 1.2200362 | 0.11106 |
| SKMC | 1.1842938 | 0.11368 |
| HRCEpiC | 1.1867766 | 0.11470 |
| AoAF | 1.1984020 | 0.11494 |
| BE2C | 1.2264010 | 0.11814 |
| BJ | 1.1981154 | 0.11840 |
| HCPEpiC | 1.1749457 | 0.11852 |
| Hepatocytes | 1.2712887 | 0.11872 |
| HRPEpiC | 1.1633599 | 0.12386 |
| NB4 | 1.2237896 | 0.13082 |
| CLL | 1.2581055 | 0.13196 |
| FibroP | 1.1541688 | 0.13616 |
| Stellate | 1.2159623 | 0.13784 |
| GM19239 | 1.2361215 | 0.14442 |
| Huh7.5 | 1.2253573 | 0.14458 |
| SAEC | 1.1405196 | 0.15006 |
| NHEK | 1.1649286 | 0.15246 |
| PanIsletD | 1.1591869 | 0.15254 |
| K562 | 1.2100752 | 0.15588 |
| HBMEC | 1.1475869 | 0.15636 |
| LNCaPAndrogen | 1.2020722 | 0.15752 |
| GM12891 | 1.2329250 | 0.16278 |
| GM12892 | 1.2308147 | 0.16512 |
| HeLaS3IFNa4h | 1.1987464 | 0.16826 |
| AG04449 | 1.1571635 | 0.16928 |
| CMK | 1.2031565 | 0.17374 |
| HRE | 1.1342559 | 0.17562 |
| AG09309 | 1.1445189 | 0.17702 |
| RPTEC | 1.1359177 | 0.17784 |
| ProgFib | 1.1664348 | 0.17792 |
| Gliobla | 1.1809322 | 0.18382 |
| IshikawaTamoxifen | 1.1637718 | 0.18724 |
| HPF | 1.1451458 | 0.18890 |
| HSMMtube | 1.1339820 | 0.18980 |
| HMVECdAd | 1.1601534 | 0.19260 |
| HEEpiC | 1.1163148 | 0.19538 |
| HL60 | 1.1838950 | 0.19716 |
| T47D | 1.1760089 | 0.20026 |
| A549 | 1.1607166 | 0.20040 |
| AoSMC | 1.1221371 | 0.20736 |
| SKNMC | 1.1354219 | 0.21272 |
| AG04450 | 1.1329746 | 0.21442 |
| HepG2 | 1.1536099 | 0.21458 |
| LNCaP | 1.1295159 | 0.21546 |
| HUVEC | 1.1324369 | 0.21692 |
| MCF7Hypoxia | 1.1652123 | 0.21942 |
| HMVECLBl | 1.1327012 | 0.21970 |
| pHTE | 1.1369516 | 0.22006 |
| GM18507 | 1.1589961 | 0.22028 |
| HAsp | 1.1143432 | 0.22396 |
| H9ES | 1.1332635 | 0.22642 |
| GM12878 | 1.1557264 | 0.22978 |
| WI38 | 1.1260068 | 0.23034 |
| CD20 | 1.1740097 | 0.23058 |
| IshikawaEstradiol | 1.1272953 | 0.23108 |
| MCF7 | 1.1268465 | 0.23286 |
| HMVECdLyAd | 1.1315531 | 0.23300 |
| HeLaS3 | 1.1323603 | 0.23812 |
| HTR8svn | 1.1233582 | 0.24298 |
| GM06990 | 1.1598080 | 0.24328 |
| HMVECdBlNeo | 1.1197732 | 0.24362 |
| GM19238 | 1.1428471 | 0.24774 |
| HMVECdBlAd | 1.1149126 | 0.25106 |
| HAh | 1.0881481 | 0.25110 |
| HMVECLLy | 1.1159967 | 0.25224 |
| GM12865 | 1.1248492 | 0.25728 |
| HAc | 1.0891379 | 0.26120 |
| H1hESC | 1.1128279 | 0.27350 |
| PanIslets | 1.1119163 | 0.27642 |
| Jurkat | 1.0996995 | 0.28178 |
| NHA | 1.0801231 | 0.29336 |
| Medullo | 1.1030830 | 0.29750 |
| HMVECdNeo | 1.0911733 | 0.29866 |
| HMVECdLyNeo | 1.0888073 | 0.30578 |
| Urothelia | 1.0881382 | 0.30590 |
| GM19240 | 1.0916310 | 0.30592 |
| HRGEC | 1.0841855 | 0.30652 |
| 8988T | 1.1069309 | 0.31356 |
| MonocytesCD14RO01746 | 1.0995645 | 0.31702 |
| Th2 | 1.1032768 | 0.31748 |
| Caco2 | 1.1246265 | 0.32036 |
| HCT116 | 1.0771141 | 0.32156 |
| PrEC | 1.0489380 | 0.35304 |
| Osteobl | 1.0673164 | 0.35590 |
| Chorion | 1.0724386 | 0.35896 |
| NHLF | 1.0509600 | 0.36116 |
| HMEC | 1.0492827 | 0.37342 |
| GM12864 | 1.0587447 | 0.37372 |
| Melano | 1.0395076 | 0.38016 |
| UrotheliaUT189 | 1.0485267 | 0.38792 |
| iPS | 1.0555350 | 0.39248 |
| HPAEC | 1.0362946 | 0.41006 |
| NT2D1 | 1.0229552 | 0.42400 |
| Fibrobl | 1.0297164 | 0.42422 |
| WERIRb1 | 1.0180571 | 0.44178 |
| Th0 | 1.0167723 | 0.45872 |
| Th1 | 1.0043069 | 0.48414 |
| H7hESC | 0.9972432 | 0.49946 |

# Plasma homocysteine concentrations

| DHS sample | fold enrichment | p value |
|---|---|---|
| RPTEC | 1.2133113 | 0.04892 |
| HRGEC | 1.2346530 | 0.05272 |
| HPAEC | 1.2241763 | 0.06458 |
| HMVECLBl | 1.2021661 | 0.08446 |
| HMVECLLy | 1.2094284 | 0.08952 |
| HMVECdLyAd | 1.2025064 | 0.09826 |
| SKNMC | 1.2068971 | 0.10856 |
| HMVECdBlNeo | 1.1658146 | 0.12896 |
| HMVECdLyNeo | 1.1697023 | 0.12972 |
| HMVECdNeo | 1.1643201 | 0.14548 |
| PANC1 | 1.1724684 | 0.15020 |
| HMVECdAd | 1.1603226 | 0.15352 |
| H7hESC | 1.1124860 | 0.16478 |
| HMVECdBlAd | 1.1239773 | 0.19504 |
| HCT116 | 1.1426309 | 0.19958 |
| HRPEpiC | 1.1082103 | 0.21260 |
| HRCEpiC | 1.1065256 | 0.21774 |
| AG09319 | 1.1140487 | 0.22048 |
| HVMF | 1.1105491 | 0.23604 |
| K562 | 1.1264008 | 0.24032 |
| NT2D1 | 1.0882221 | 0.24998 |
| HPdLF | 1.0835816 | 0.28030 |
| HConF | 1.0819741 | 0.28848 |
| HCM | 1.0736645 | 0.29054 |
| HCFaa | 1.0699558 | 0.30140 |
| CMK | 1.0950690 | 0.30346 |
| HPAF | 1.0618507 | 0.32552 |
| HNPCEpiC | 1.0583964 | 0.32570 |
| HGF | 1.0643030 | 0.33274 |
| HCF | 1.0627773 | 0.33344 |
| HFFMyc | 1.0580620 | 0.33816 |
| Jurkat | 1.0397319 | 0.39272 |
| AG10803 | 1.0372151 | 0.39468 |
| HEEpiC | 1.0302738 | 0.40910 |
| HAsp | 1.0283359 | 0.41296 |
| BJ | 1.0287638 | 0.41576 |
| GM12864 | 1.0304545 | 0.42156 |
| HPF | 1.0268138 | 0.42406 |
| GM12865 | 1.0257482 | 0.42852 |
| MonocytesCD14RO01746 | 1.0289257 | 0.43160 |
| HIPEpiC | 1.0220313 | 0.43404 |
| NB4 | 1.0253699 | 0.43422 |
| A549 | 1.0236325 | 0.43676 |
| CD20 | 1.0249176 | 0.43736 |
| Th2 | 1.0258003 | 0.43928 |
| HPDE6E6E7 | 1.0234280 | 0.43950 |
| HAEpiC | 1.0220864 | 0.44230 |
| HeLaS3 | 1.0215540 | 0.44386 |
| NHDFneo | 1.0170443 | 0.44824 |
| HMF | 1.0161253 | 0.45006 |
| HCPEpiC | 1.0168818 | 0.45078 |
| HepG2 | 1.0186377 | 0.45146 |
| GM06990 | 1.0178117 | 0.45288 |
| HRE | 1.0118450 | 0.46296 |
| IshikawaEstradiol | 1.0111133 | 0.46438 |
| HBMEC | 1.0107365 | 0.46538 |
| HUVEC | 1.0114455 | 0.46578 |
| AG04450 | 1.0056699 | 0.48262 |
| IshikawaTamoxifen | 1.0001934 | 0.49130 |
| SAEC | 1.0028049 | 0.49226 |
| BE2C | 0.9869012 | 0.51842 |
| HAc | 0.9922823 | 0.51976 |
| GM18507 | 0.9871106 | 0.52430 |
| AG04449 | 0.9893863 | 0.52522 |
| GM12878 | 0.9814780 | 0.53176 |
| MCF7Hypoxia | 0.9790843 | 0.53438 |
| SKNSHRA | 0.9636000 | 0.54290 |
| AG09309 | 0.9825784 | 0.54808 |
| Caco2 | 0.9522778 | 0.55568 |
| CD34Mobilized | 0.9736909 | 0.55830 |
| HFF | 0.9790804 | 0.56104 |
| HeLaS3IFNa4h | 0.9611517 | 0.56956 |
| NHA | 0.9735872 | 0.56976 |
| AoSMC | 0.9780540 | 0.57024 |
| UrotheliaUT189 | 0.9666845 | 0.57162 |
| NHDFAd | 0.9746787 | 0.57414 |
| WERIRb1 | 0.9666479 | 0.57580 |
| Huh7 | 0.9598612 | 0.57714 |
| Urothelia | 0.9648174 | 0.58034 |
| GM12892 | 0.9494753 | 0.59244 |
| Huh7.5 | 0.9482010 | 0.59820 |
| HAh | 0.9653194 | 0.60402 |
| RWPE1 | 0.9565459 | 0.60564 |
| PrEC | 0.9650607 | 0.61228 |
| HL60 | 0.9368859 | 0.62070 |
| T47D | 0.9336174 | 0.62134 |
| AoAF | 0.9525276 | 0.62432 |
| LNCaP | 0.9422327 | 0.62674 |
| SKMC | 0.9536822 | 0.62700 |
| GM12891 | 0.9305564 | 0.62702 |
| WI38 | 0.9493627 | 0.63006 |
| NHLF | 0.9516495 | 0.63240 |
| NHEK | 0.9481903 | 0.63240 |
| MCF7 | 0.9381014 | 0.63476 |
| HTR8svn | 0.9404943 | 0.63804 |
| GM19238 | 0.9294007 | 0.64956 |
| CLL | 0.9219732 | 0.64990 |
| H9ES | 0.9190500 | 0.66696 |
| Melano | 0.9454109 | 0.66900 |
| H1hESC | 0.9044845 | 0.67924 |
| GM19239 | 0.8981935 | 0.69416 |
| LNCaPAndrogen | 0.9014348 | 0.69918 |
| Chorion | 0.8789893 | 0.70430 |
| GM19240 | 0.9104697 | 0.70702 |
| PanIslets | 0.9007816 | 0.70838 |
| Myometr | 0.9092380 | 0.71524 |
| Medullo | 0.8961382 | 0.71968 |
| iPS | 0.8663110 | 0.72796 |
| ProgFib | 0.8902166 | 0.73804 |
| Hepatocytes | 0.8597060 | 0.74358 |
| Stellate | 0.8806062 | 0.75328 |
| HSMMemb | 0.8873331 | 0.76220 |
| FibroP | 0.9032662 | 0.77850 |
| HMEC | 0.8736060 | 0.79074 |
| PanIsletD | 0.8796951 | 0.79476 |
| Gliobla | 0.8485738 | 0.79582 |
| Th0 | 0.8537733 | 0.81018 |
| 8988T | 0.8056197 | 0.81758 |
| Osteobl | 0.8459918 | 0.83182 |
| Fibrobl | 0.8460746 | 0.83776 |
| HSMM | 0.8678761 | 0.84080 |
| pHTE | 0.8339827 | 0.84464 |
| Th1 | 0.8283638 | 0.85358 |
| HSMMtube | 0.8305923 | 0.88542 |

## Sudden cardiac arrest

| DHS sample | fold enrichment | p value |
|---|---|---|
| RPTEC | 1.4210286 | 0.01450 |
| HAsp | 1.4565803 | 0.01486 |
| HPdLF | 1.5138083 | 0.02436 |
| HRCEpiC | 1.4002981 | 0.02640 |
| WI38 | 1.5113236 | 0.03096 |
| HCFaa | 1.4375300 | 0.03396 |
| HGF | 1.4728190 | 0.03582 |
| HCT116 | 1.4779057 | 0.03876 |
| HPF | 1.4182144 | 0.04690 |
| SKMC | 1.3688169 | 0.04728 |
| HFFMyc | 1.4027222 | 0.04762 |
| NHDFneo | 1.4036870 | 0.05140 |
| HBMEC | 1.3511728 | 0.05142 |
| BJ | 1.4092981 | 0.05198 |
| NHDFAd | 1.3644786 | 0.05394 |
| HRE | 1.3091827 | 0.05672 |
| HEEpiC | 1.3049566 | 0.05836 |
| HMF | 1.3634657 | 0.06014 |
| HCF | 1.3931731 | 0.06148 |
| HCPEpiC | 1.3526414 | 0.06234 |
| HAc | 1.3012763 | 0.06474 |
| NHA | 1.3240861 | 0.06530 |
| NHLF | 1.3039147 | 0.07280 |
| SAEC | 1.2774263 | 0.07596 |
| AG04450 | 1.3647719 | 0.07630 |
| HRPEpiC | 1.2505438 | 0.08028 |
| HPAF | 1.3217333 | 0.08520 |
| AoAF | 1.3455089 | 0.08580 |
| HNPCEpiC | 1.2727056 | 0.08580 |
| HAh | 1.2491032 | 0.09094 |
| AG10803 | 1.3278140 | 0.09652 |
| HCM | 1.2954811 | 0.10266 |
| PrEC | 1.2433616 | 0.10944 |
| AG09319 | 1.3121111 | 0.11206 |
| HMVECdBlNeo | 1.3241705 | 0.11368 |
| HRGEC | 1.3001381 | 0.11978 |
| AG04449 | 1.2787401 | 0.12528 |
| AG09309 | 1.2619936 | 0.12742 |
| HIPEpiC | 1.2561725 | 0.12764 |
| HFF | 1.2557312 | 0.13846 |
| HVMF | 1.2878240 | 0.14224 |
| HMVECLBl | 1.2699704 | 0.14462 |
| HMVECdNeo | 1.2750724 | 0.15138 |
| HMVECdAd | 1.2800607 | 0.15600 |
| HAEpiC | 1.2440622 | 0.16058 |
| HConF | 1.2249407 | 0.16524 |
| HMVECdBlAd | 1.2411370 | 0.17932 |
| HMVECdLyNeo | 1.2341726 | 0.18646 |
| AoSMC | 1.2073123 | 0.18716 |
| PANC1 | 1.2241122 | 0.18834 |
| HMVECdLyAd | 1.2298094 | 0.19604 |
| HSMM | 1.1681826 | 0.20482 |
| HPAEC | 1.1859328 | 0.22306 |
| IshikawaEstradiol | 1.1642647 | 0.24312 |
| HMVECLLy | 1.1741761 | 0.24906 |
| IshikawaTamoxifen | 1.1596824 | 0.25500 |
| HUVEC | 1.1251842 | 0.29864 |
| Th2 | 1.1555683 | 0.30732 |
| HPDE6E6E7 | 1.1166673 | 0.31544 |
| NHEK | 1.0957042 | 0.33380 |
| WERIRb1 | 1.0426135 | 0.39962 |
| HTR8svn | 1.0318228 | 0.43338 |
| SKNMC | 1.0207724 | 0.44544 |
| Myometr | 1.0189892 | 0.45536 |
| RWPE1 | 1.0101892 | 0.47052 |
| HeLaS3 | 1.0006698 | 0.47702 |
| HSMMtube | 0.9859383 | 0.51470 |
| Stellate | 0.9737020 | 0.51480 |
| FibroP | 0.9781962 | 0.53176 |
| K562 | 0.9480834 | 0.53624 |
| HSMMemb | 0.9408345 | 0.56672 |
| A549 | 0.9336255 | 0.57660 |
| PanIsletD | 0.9430402 | 0.58518 |
| GM12865 | 0.9205494 | 0.59792 |
| H7hESC | 0.9585582 | 0.61668 |
| BE2C | 0.9082972 | 0.61954 |
| GM06990 | 0.8639739 | 0.63220 |
| GM12864 | 0.8828032 | 0.63668 |
| Melano | 0.9265071 | 0.64440 |
| HepG2 | 0.8668083 | 0.65786 |
| HeLaS3IFNa4h | 0.8319645 | 0.70596 |
| Jurkat | 0.8385960 | 0.72674 |
| CD20 | 0.7538644 | 0.74236 |
| CD34Mobilized | 0.8107591 | 0.74558 |
| SKNSHRA | 0.8050235 | 0.74986 |
| ProgFib | 0.8069170 | 0.76038 |
| HMEC | 0.8230097 | 0.76890 |
| GM12878 | 0.7725500 | 0.77220 |
| MonocytesCD14RO01746 | 0.7345932 | 0.77936 |
| GM18507 | 0.7510790 | 0.79846 |
| Urothelia | 0.7715084 | 0.81450 |
| NB4 | 0.7198529 | 0.83798 |
| pHTE | 0.7351270 | 0.84662 |
| CLL | 0.6741440 | 0.84910 |
| UrotheliaUT189 | 0.7322195 | 0.85000 |
| Gliobla | 0.7152158 | 0.85054 |
| T47D | 0.6925143 | 0.85810 |
| GM19238 | 0.6717115 | 0.85932 |
| Medullo | 0.6981329 | 0.86294 |
| Caco2 | 0.5197603 | 0.87544 |
| GM19239 | 0.6512517 | 0.88216 |
| H9ES | 0.7304708 | 0.88810 |
| MCF7 | 0.6918651 | 0.90972 |
| LNCaPAndrogen | 0.6192662 | 0.91154 |
| Th0 | 0.5985566 | 0.91202 |
| Huh7.5 | 0.6140199 | 0.91464 |
| GM12891 | 0.5521603 | 0.91860 |
| PanIslets | 0.6275890 | 0.92242 |
| Huh7 | 0.6018349 | 0.92838 |
| Fibrobl | 0.6146706 | 0.93582 |
| Osteobl | 0.5937794 | 0.93936 |
| H1hESC | 0.6303007 | 0.94366 |
| Th1 | 0.5399050 | 0.94564 |
| GM19240 | 0.5796061 | 0.94694 |
| HL60 | 0.5276263 | 0.95020 |
| LNCaP | 0.6520116 | 0.95086 |
| 8988T | 0.4803505 | 0.95726 |
| GM12892 | 0.4419925 | 0.95886 |
| CMK | 0.5297148 | 0.96016 |
| MCF7Hypoxia | 0.4883735 | 0.97274 |
| Hepatocytes | 0.4816637 | 0.97298 |
| Chorion | 0.4420909 | 0.97478 |
| NT2D1 | 0.6527024 | 0.98464 |
| iPS | 0.3757253 | 0.98480 |

# Lipid levels 3

| DHS sample | fold enrichment | p value |
|---|---|---|
| HL60 | 1.3087516 | 0.01662 |
| HRCEpiC | 1.1355111 | 0.08696 |
| RPTEC | 1.1185018 | 0.11034 |
| HepG2 | 1.1587937 | 0.11816 |
| HeLaS3 | 1.1516362 | 0.11906 |
| HRE | 1.1055657 | 0.13774 |
| HCFaa | 1.1132780 | 0.14348 |
| HEEpiC | 1.0960444 | 0.15156 |
| SAEC | 1.0910876 | 0.15944 |
| MonocytesCD14RO01746 | 1.1413218 | 0.16564 |
| HCPEpiC | 1.0985087 | 0.17024 |
| HPAF | 1.0922470 | 0.18616 |
| HIPEpiC | 1.0890173 | 0.18932 |
| PrEC | 1.0810147 | 0.19020 |
| HeLaS3IFNa4h | 1.1180528 | 0.19810 |
| IshikawaEstradiol | 1.0970777 | 0.20008 |
| WI38 | 1.0903385 | 0.21732 |
| Jurkat | 1.0884253 | 0.21898 |
| HGF | 1.0882976 | 0.22286 |
| AG04450 | 1.0855280 | 0.22696 |
| GM12865 | 1.0881149 | 0.24060 |
| Stellate | 1.0872908 | 0.24506 |
| NHEK | 1.0740039 | 0.24706 |
| Huh7 | 1.0899239 | 0.25062 |
| NB4 | 1.0841855 | 0.25434 |
| HPDE6E6E7 | 1.0777265 | 0.25488 |
| HAh | 1.0624335 | 0.25548 |
| K562 | 1.0870865 | 0.25670 |
| Huh7.5 | 1.0895109 | 0.25702 |
| HPF | 1.0711480 | 0.26132 |
| HMVECdAd | 1.0750307 | 0.26198 |
| HAEpiC | 1.0670969 | 0.26502 |
| HCM | 1.0586887 | 0.27480 |
| HTR8svn | 1.0712247 | 0.27686 |
| PANC1 | 1.0711869 | 0.28120 |
| HCF | 1.0621688 | 0.28180 |
| GM12864 | 1.0708231 | 0.29036 |
| HNPCEpiC | 1.0528107 | 0.29194 |
| BJ | 1.0598898 | 0.29602 |
| HRGEC | 1.0554323 | 0.30038 |
| Th2 | 1.0730625 | 0.30126 |
| HCT116 | 1.0616514 | 0.30318 |
| HConF | 1.0535387 | 0.31238 |
| HVMF | 1.0555600 | 0.31266 |
| HBMEC | 1.0487523 | 0.31284 |
| NT2D1 | 1.0454195 | 0.32024 |
| HSMMemb | 1.0524189 | 0.32758 |
| GM12878 | 1.0579124 | 0.32826 |
| AoSMC | 1.0428671 | 0.33140 |
| NHA | 1.0445644 | 0.33490 |
| IshikawaTamoxifen | 1.0473127 | 0.34048 |
| NHLF | 1.0426279 | 0.34156 |
| RWPE1 | 1.0458377 | 0.34204 |
| PanIsletD | 1.0420513 | 0.34414 |
| HRPEpiC | 1.0374665 | 0.34712 |
| AG04449 | 1.0425446 | 0.34804 |
| HMVECLBl | 1.0400407 | 0.35412 |
| GM06990 | 1.0509038 | 0.35994 |
| HMF | 1.0330722 | 0.37094 |
| SKMC | 1.0332287 | 0.37170 |
| HMVECdLyNeo | 1.0342312 | 0.37454 |
| CMK | 1.0404518 | 0.38168 |
| HMVECdBlAd | 1.0292848 | 0.39014 |
| Myometr | 1.0266100 | 0.39874 |
| HAc | 1.0250425 | 0.39952 |
| HMVECLLy | 1.0284953 | 0.40020 |
| HPdLF | 1.0276736 | 0.40110 |
| HMVECdNeo | 1.0251127 | 0.40984 |
| MCF7 | 1.0248662 | 0.41126 |
| A549 | 1.0255155 | 0.41218 |
| Gliobla | 1.0250823 | 0.41596 |
| GM19240 | 1.0249337 | 0.41596 |
| H7hESC | 1.0159371 | 0.41940 |
| HMVECdBlNeo | 1.0217017 | 0.42122 |
| AoAF | 1.0209086 | 0.42188 |
| HPAEC | 1.0175846 | 0.43528 |
| AG09309 | 1.0139662 | 0.44370 |
| Urothelia | 1.0140433 | 0.45122 |
| FibroP | 1.0088964 | 0.46044 |
| Caco2 | 1.0095096 | 0.46200 |
| WERIRb1 | 1.0077871 | 0.46412 |
| Hepatocytes | 1.0067075 | 0.46974 |
| HMVECdLyAd | 1.0063428 | 0.47382 |
| AG10803 | 1.0049605 | 0.47760 |
| HFF | 1.0043374 | 0.48010 |
| HAsp | 1.0003514 | 0.48988 |
| AG09319 | 1.0001349 | 0.49438 |
| HSMM | 1.0001733 | 0.49562 |
| CD20 | 0.9955037 | 0.49970 |
| UrotheliaUT189 | 0.9965511 | 0.50664 |
| GM19239 | 0.9925745 | 0.51274 |
| PanIslets | 0.9937053 | 0.51598 |
| HFFMyc | 0.9942545 | 0.51986 |
| GM19238 | 0.9887230 | 0.52658 |
| CLL | 0.9848102 | 0.53458 |
| 8988T | 0.9787160 | 0.53842 |
| HSMMtube | 0.9888468 | 0.54180 |
| Melano | 0.9907004 | 0.54212 |
| GM18507 | 0.9825857 | 0.54988 |
| Th0 | 0.9825761 | 0.55450 |
| CD34Mobilized | 0.9805843 | 0.55594 |
| NHDFAd | 0.9813816 | 0.56658 |
| GM12891 | 0.9685865 | 0.57294 |
| ProgFib | 0.9728101 | 0.57970 |
| NHDFneo | 0.9762840 | 0.58086 |
| BE2C | 0.9642464 | 0.58720 |
| Fibrobl | 0.9728305 | 0.59124 |
| Th1 | 0.9692895 | 0.60024 |
| Medullo | 0.9659018 | 0.60178 |
| HMEC | 0.9687604 | 0.60350 |
| MCF7Hypoxia | 0.9582155 | 0.60494 |
| GM12892 | 0.9541091 | 0.61216 |
| Osteobl | 0.9625702 | 0.62068 |
| LNCaPAndrogen | 0.9523324 | 0.62990 |
| pHTE | 0.9579899 | 0.63442 |
| HUVEC | 0.9554787 | 0.64462 |
| H9ES | 0.9448378 | 0.65030 |
| SKNSHRA | 0.9233856 | 0.66982 |
| Chorion | 0.9018430 | 0.72132 |
| iPS | 0.8968022 | 0.73476 |
| H1hESC | 0.8830460 | 0.78904 |
| LNCaP | 0.8990287 | 0.79588 |
| T47D | 0.8660624 | 0.80822 |
| SKNMC | 0.8914075 | 0.81192 |

# Blood metabolites 2

| DHS sample | fold enrichment | p value |
|---|---|---|
| RPTEC | 1.0736780 | 0.03568 |
| Jurkat | 1.0648253 | 0.09864 |
| HL60 | 1.0678569 | 0.13972 |
| Th2 | 1.0578980 | 0.17768 |
| HRCEpiC | 1.0387896 | 0.19014 |
| HPAEC | 1.0406317 | 0.20500 |
| Huh7 | 1.0415737 | 0.24082 |
| PANC1 | 1.0365186 | 0.25074 |
| HepG2 | 1.0374285 | 0.25650 |
| K562 | 1.0370891 | 0.26274 |
| HMVECdAd | 1.0330550 | 0.26520 |
| NB4 | 1.0347273 | 0.26574 |
| GM12878 | 1.0361277 | 0.27148 |
| GM12865 | 1.0334835 | 0.27210 |
| CMK | 1.0367249 | 0.28410 |
| IshikawaEstradiol | 1.0271364 | 0.30150 |
| A549 | 1.0299408 | 0.30156 |
| HeLaS3 | 1.0288386 | 0.30380 |
| HMVECdLyNeo | 1.0251343 | 0.30736 |
| HeLaS3IFNa4h | 1.0291636 | 0.31680 |
| CLL | 1.0295312 | 0.31936 |
| HPDE6E6E7 | 1.0246873 | 0.32130 |
| IshikawaTamoxifen | 1.0242544 | 0.32698 |
| GM12864 | 1.0252344 | 0.32732 |
| HMVECdLyAd | 1.0225791 | 0.33160 |
| HCFaa | 1.0178214 | 0.34630 |
| HMVECdNeo | 1.0193470 | 0.35156 |
| HMVECLLy | 1.0188396 | 0.35598 |
| GM18507 | 1.0203963 | 0.35868 |
| HEEpiC | 1.0148218 | 0.36044 |
| HMVECLBl | 1.0169659 | 0.36146 |
| Huh7.5 | 1.0204629 | 0.36322 |
| HMVECdBlNeo | 1.0164443 | 0.37192 |
| MonocytesCD14RO01746 | 1.0175018 | 0.38722 |
| SAEC | 1.0115458 | 0.38772 |
| GM06990 | 1.0158302 | 0.40110 |
| HMVECdBlAd | 1.0106328 | 0.41022 |
| HRGEC | 1.0102658 | 0.41394 |
| HUVEC | 1.0104262 | 0.41620 |
| HGF | 1.0082689 | 0.43284 |
| Hepatocytes | 1.0091730 | 0.44130 |
| HRPEpiC | 1.0034723 | 0.46772 |
| HPAF | 1.0032869 | 0.46866 |
| LNCaPAndrogen | 1.0034967 | 0.47288 |
| Myometr | 1.0028044 | 0.47528 |
| AoSMC | 1.0012380 | 0.48788 |
| HIPEpiC | 0.9993447 | 0.50458 |
| HCPEpiC | 0.9991588 | 0.50964 |
| GM19239 | 0.9966703 | 0.51558 |
| CD34Mobilized | 0.9974467 | 0.51652 |
| HAEpiC | 0.9978825 | 0.51670 |
| CD20 | 0.9942759 | 0.52816 |
| NHEK | 0.9942534 | 0.54370 |
| HRE | 0.9893988 | 0.59750 |
| HVMF | 0.9845995 | 0.61646 |
| AoAF | 0.9855671 | 0.61850 |
| GM19240 | 0.9812932 | 0.63550 |
| Th0 | 0.9799580 | 0.64330 |
| GM19238 | 0.9770450 | 0.64684 |
| T47D | 0.9735490 | 0.65568 |
| Stellate | 0.9759117 | 0.66244 |
| GM12891 | 0.9717764 | 0.66276 |
| RWPE1 | 0.9777974 | 0.66520 |
| PanIsletD | 0.9794451 | 0.67042 |
| HCT116 | 0.9741553 | 0.67486 |
| HMF | 0.9782777 | 0.68124 |
| HCF | 0.9772852 | 0.68154 |
| HConF | 0.9759524 | 0.68822 |
| HCM | 0.9770609 | 0.69638 |
| AG04450 | 0.9735086 | 0.69866 |
| HPF | 0.9743271 | 0.70132 |
| MCF7 | 0.9702025 | 0.70692 |
| AG09319 | 0.9713886 | 0.71346 |
| HNPCEpiC | 0.9761636 | 0.71532 |
| HAsp | 0.9721048 | 0.72252 |
| HPdLF | 0.9704740 | 0.72430 |
| PrEC | 0.9755173 | 0.72524 |
| NHLF | 0.9727701 | 0.72696 |
| BE2C | 0.9647297 | 0.73260 |
| HBMEC | 0.9719615 | 0.73898 |
| MCF7Hypoxia | 0.9593179 | 0.74036 |
| NHA | 0.9690582 | 0.75062 |
| WI38 | 0.9655329 | 0.75548 |
| SKNSHRA | 0.9501275 | 0.75756 |
| BJ | 0.9658284 | 0.76076 |
| HSMMemb | 0.9630513 | 0.76238 |
| Gliobla | 0.9558690 | 0.76462 |
| GM12892 | 0.9525350 | 0.76472 |
| ProgFib | 0.9596853 | 0.77138 |
| AG04449 | 0.9646080 | 0.77360 |
| NHDFAd | 0.9656168 | 0.77898 |
| NHDFneo | 0.9622576 | 0.77980 |
| PanIslets | 0.9560614 | 0.78314 |
| Melano | 0.9670798 | 0.79692 |
| HFF | 0.9613500 | 0.79870 |
| H9ES | 0.9523374 | 0.79922 |
| HFFMyc | 0.9622294 | 0.80372 |
| LNCaP | 0.9540481 | 0.80832 |
| Th1 | 0.9542783 | 0.80852 |
| 8988T | 0.9366712 | 0.81536 |
| HTR8svn | 0.9522513 | 0.81588 |
| Urothelia | 0.9507188 | 0.81744 |
| SKMC | 0.9553078 | 0.83736 |
| Medullo | 0.9443845 | 0.83912 |
| SKNMC | 0.9465138 | 0.85490 |
| Chorion | 0.9252776 | 0.86204 |
| AG09309 | 0.9516390 | 0.86216 |
| pHTE | 0.9418452 | 0.86716 |
| Caco2 | 0.9047652 | 0.86956 |
| HSMM | 0.9518349 | 0.87228 |
| HAc | 0.9508050 | 0.87434 |
| AG10803 | 0.9424218 | 0.87964 |
| WERIRb1 | 0.9413738 | 0.88930 |
| H1hESC | 0.9243608 | 0.89322 |
| NT2D1 | 0.9449947 | 0.89708 |
| HAh | 0.9449766 | 0.91124 |
| UrotheliaUT189 | 0.9251363 | 0.91264 |
| HSMMtube | 0.9382963 | 0.91718 |
| HMEC | 0.9279447 | 0.92442 |
| Osteobl | 0.9235683 | 0.92798 |
| FibroP | 0.9383832 | 0.93434 |
| iPS | 0.8938599 | 0.94044 |
| H7hESC | 0.9439336 | 0.94220 |
| Fibrobl | 0.9146823 | 0.95346 |

# Fasting glucose and Type 2 diabetes

| DHS sample | fold enrichment | p value |
|---|---|---|
| RPTEC | 1.1780617 | 0.08546 |
| HRCEpiC | 1.1731021 | 0.10644 |
| BE2C | 1.1656505 | 0.17260 |
| NT2D1 | 1.1106128 | 0.19638 |
| HCFaa | 1.1158183 | 0.20832 |
| HCF | 1.1212669 | 0.21390 |
| Huh7 | 1.1500722 | 0.21584 |
| HMVECLLy | 1.1266829 | 0.22230 |
| HMVECdLyAd | 1.1173288 | 0.23992 |
| HAh | 1.0842431 | 0.24648 |
| HRPEpiC | 1.0855947 | 0.24754 |
| HMVECdNeo | 1.1076978 | 0.25634 |
| HeLaS3IFNa4h | 1.1203857 | 0.25832 |
| HeLaS3 | 1.1043337 | 0.26916 |
| SKMC | 1.0832867 | 0.27566 |
| HRGEC | 1.0918713 | 0.27770 |
| HRE | 1.0735888 | 0.28326 |
| H7hESC | 1.0567071 | 0.28518 |
| AoSMC | 1.0759450 | 0.28990 |
| HMVECdAd | 1.0813698 | 0.31150 |
| SAEC | 1.0613778 | 0.31238 |
| HCPEpiC | 1.0683760 | 0.31276 |
| K562 | 1.0919947 | 0.31352 |
| HPAF | 1.0670569 | 0.31856 |
| HPAEC | 1.0678124 | 0.33210 |
| HCM | 1.0594825 | 0.33728 |
| HMVECdLyNeo | 1.0648607 | 0.34312 |
| SKNMC | 1.0590132 | 0.34350 |
| HAEpiC | 1.0578594 | 0.34660 |
| HMVECdBlAd | 1.0608355 | 0.34780 |
| Huh7.5 | 1.0698568 | 0.35084 |
| A549 | 1.0642764 | 0.35100 |
| HMVECdBlNeo | 1.0615119 | 0.35204 |
| AG10803 | 1.0521523 | 0.35792 |
| HSMM | 1.0454849 | 0.36128 |
| Gliobla | 1.0608590 | 0.36168 |
| AoAF | 1.0524875 | 0.36266 |
| HAsp | 1.0455319 | 0.36854 |
| HCT116 | 1.0487802 | 0.37580 |
| HMF | 1.0429542 | 0.37828 |
| HSMMtube | 1.0409109 | 0.37912 |
| NHDFAd | 1.0398658 | 0.38156 |
| IshikawaEstradiol | 1.0448722 | 0.38326 |
| SKNSHRA | 1.0439560 | 0.39662 |
| HEEpiC | 1.0296245 | 0.40454 |
| HUVEC | 1.0347579 | 0.40758 |
| HIPEpiC | 1.0321727 | 0.40794 |
| AG04449 | 1.0333810 | 0.40936 |
| Myometr | 1.0283146 | 0.41704 |
| PrEC | 1.0231708 | 0.42232 |
| IshikawaTamoxifen | 1.0264537 | 0.42568 |
| HMVECLBl | 1.0276918 | 0.42570 |
| HFF | 1.0245728 | 0.42800 |
| NHLF | 1.0180086 | 0.44168 |
| HNPCEpiC | 1.0152308 | 0.44882 |
| PANC1 | 1.0083850 | 0.46430 |
| BJ | 1.0075684 | 0.47326 |
| AG09309 | 1.0087243 | 0.47434 |
| HAc | 1.0061618 | 0.47560 |
| ProgFib | 1.0026736 | 0.48344 |
| PanIsletD | 1.0007003 | 0.49186 |
| HPdLF | 0.9989694 | 0.49784 |
| LNCaPAndrogen | 0.9893463 | 0.50704 |
| Jurkat | 0.9886222 | 0.51644 |
| HepG2 | 0.9854393 | 0.51976 |
| HPDE6E6E7 | 0.9868033 | 0.52202 |
| T47D | 0.9723710 | 0.53826 |
| HConF | 0.9785980 | 0.55108 |
| Hepatocytes | 0.9641156 | 0.55254 |
| HGF | 0.9748747 | 0.56084 |
| PanIslets | 0.9696759 | 0.56130 |
| Stellate | 0.9660250 | 0.56568 |
| 8988T | 0.9455828 | 0.57544 |
| AG04450 | 0.9676167 | 0.57764 |
| NHA | 0.9610212 | 0.60274 |
| HBMEC | 0.9618102 | 0.60304 |
| FibroP | 0.9650860 | 0.60656 |
| NHDFneo | 0.9586427 | 0.60774 |
| AG09319 | 0.9554381 | 0.61116 |
| HSMMemb | 0.9486176 | 0.61486 |
| HVMF | 0.9433946 | 0.62446 |
| H9ES | 0.9404135 | 0.62682 |
| WERIRb1 | 0.9504531 | 0.62770 |
| WI38 | 0.9428390 | 0.63070 |
| HTR8svn | 0.9409727 | 0.63258 |
| NHEK | 0.9429208 | 0.63828 |
| HFFMyc | 0.9474723 | 0.64844 |
| UrotheliaUT189 | 0.9275992 | 0.65028 |
| HPF | 0.9356225 | 0.65482 |
| GM19239 | 0.9120384 | 0.65716 |
| pHTE | 0.9265863 | 0.66386 |
| Medullo | 0.9186585 | 0.66672 |
| HMEC | 0.9303428 | 0.66806 |
| LNCaP | 0.9181883 | 0.68078 |
| Melano | 0.9426555 | 0.68314 |
| MCF7 | 0.9075926 | 0.68976 |
| H1hESC | 0.8916141 | 0.70722 |
| Fibrobl | 0.9038732 | 0.71402 |
| GM12891 | 0.8658700 | 0.72400 |
| Caco2 | 0.8177565 | 0.72524 |
| Osteobl | 0.8947603 | 0.72702 |
| Urothelia | 0.8937157 | 0.72710 |
| Chorion | 0.8596183 | 0.73018 |
| RWPE1 | 0.8974782 | 0.73860 |
| NB4 | 0.8809145 | 0.73972 |
| iPS | 0.8518985 | 0.74628 |
| CMK | 0.8494963 | 0.75836 |
| GM18507 | 0.8652833 | 0.75964 |
| Th0 | 0.8603490 | 0.77016 |
| MonocytesCD14RO01746 | 0.8309443 | 0.78944 |
| Th1 | 0.8512786 | 0.79522 |
| GM12892 | 0.8207993 | 0.79820 |
| GM19240 | 0.8516201 | 0.80314 |
| CD34Mobilized | 0.8430445 | 0.81988 |
| GM19238 | 0.8203280 | 0.82092 |
| HL60 | 0.8083652 | 0.82424 |
| MCF7Hypoxia | 0.8052202 | 0.82662 |
| Th2 | 0.7834957 | 0.84660 |
| CD20 | 0.7649964 | 0.85228 |
| GM12878 | 0.7882639 | 0.86696 |
| GM12865 | 0.8035973 | 0.87294 |
| GM12864 | 0.7804475 | 0.88112 |
| CLL | 0.7531910 | 0.88790 |
| GM06990 | 0.6772372 | 0.94912 |

# Crohns disease 3

| DHS sample | fold enrichment | p value |
|---|---|---|
| Th2 | 1.4975209 | 0.02018 |
| MonocytesCD14RO01746 | 1.3048740 | 0.09588 |
| Th0 | 1.2615471 | 0.10380 |
| Th1 | 1.2472335 | 0.10496 |
| GM12865 | 1.2323563 | 0.11904 |
| GM12864 | 1.1849982 | 0.17994 |
| GM06990 | 1.2089195 | 0.18106 |
| GM19240 | 1.1664922 | 0.19604 |
| CD20 | 1.1933056 | 0.20932 |
| GM12878 | 1.1716195 | 0.21086 |
| Jurkat | 1.1172820 | 0.24724 |
| GM19238 | 1.1391743 | 0.25568 |
| NB4 | 1.1244859 | 0.26010 |
| CD34Mobilized | 1.1065737 | 0.28246 |
| GM18507 | 1.1122058 | 0.28794 |
| HL60 | 1.0994617 | 0.31758 |
| CLL | 1.0668369 | 0.37296 |
| RWPE1 | 1.0397335 | 0.39748 |
| GM19239 | 1.0449885 | 0.40210 |
| AoSMC | 1.0348221 | 0.40896 |
| CMK | 1.0379037 | 0.40922 |
| GM12891 | 1.0320628 | 0.42938 |
| HMEC | 1.0247134 | 0.43502 |
| NHEK | 1.0185962 | 0.44148 |
| UrotheliaUT189 | 1.0225613 | 0.44228 |
| HPDE6E6E7 | 1.0130605 | 0.45672 |
| GM12892 | 1.0147246 | 0.45874 |
| HMVECLBl | 1.0101004 | 0.47216 |
| PanIslets | 1.0025677 | 0.48262 |
| Hepatocytes | 0.9942218 | 0.48966 |
| Osteobl | 0.9989427 | 0.49298 |
| PANC1 | 0.9940740 | 0.49336 |
| HMVECdBlAd | 0.9918912 | 0.50822 |
| PrEC | 0.9948362 | 0.51210 |
| HepG2 | 0.9848459 | 0.51456 |
| pHTE | 0.9881680 | 0.51462 |
| HEEpiC | 0.9918741 | 0.51752 |
| RPTEC | 0.9932046 | 0.51772 |
| HPAEC | 0.9859706 | 0.52178 |
| FibroP | 0.9836929 | 0.54098 |
| 8988T | 0.9600016 | 0.54108 |
| HMVECdAd | 0.9708431 | 0.54790 |
| SAEC | 0.9805399 | 0.55110 |
| Urothelia | 0.9660342 | 0.55320 |
| AG09309 | 0.9785093 | 0.55382 |
| HCFaa | 0.9755998 | 0.55566 |
| HMVECdLyAd | 0.9592180 | 0.57322 |
| HMVECdBlNeo | 0.9535387 | 0.59208 |
| Melano | 0.9625927 | 0.60246 |
| NHDFAd | 0.9552580 | 0.61390 |
| HRCEpiC | 0.9529640 | 0.61664 |
| BJ | 0.9451707 | 0.62782 |
| NT2D1 | 0.9457044 | 0.63138 |
| AoAF | 0.9410473 | 0.63230 |
| K562 | 0.9128106 | 0.63732 |
| Gliobla | 0.9067021 | 0.65542 |
| HMVECdNeo | 0.9188043 | 0.65580 |
| iPS | 0.8901714 | 0.65932 |
| H7hESC | 0.9496677 | 0.65944 |
| HMVECdLyNeo | 0.9186237 | 0.66100 |
| Myometr | 0.9223623 | 0.66164 |
| HRE | 0.9388848 | 0.66234 |
| HMF | 0.9301485 | 0.66336 |
| HCT116 | 0.9058660 | 0.66750 |
| HMVECLLy | 0.9136458 | 0.66808 |
| H1hESC | 0.8951074 | 0.66958 |
| H9ES | 0.8996971 | 0.67374 |
| HRGEC | 0.9177860 | 0.67546 |
| HUVEC | 0.9077223 | 0.67926 |
| Chorion | 0.8680369 | 0.68490 |
| HIPEpiC | 0.9212958 | 0.69584 |
| Fibrobl | 0.9047096 | 0.69774 |
| HFF | 0.9165722 | 0.69794 |
| HCM | 0.9167309 | 0.70284 |
| A549 | 0.8811384 | 0.70536 |
| HFFMyc | 0.9147349 | 0.70658 |
| WERIRb1 | 0.9086379 | 0.70938 |
| IshikawaEstradiol | 0.8892971 | 0.71368 |
| SKNMC | 0.9001448 | 0.71490 |
| HSMMtube | 0.9072764 | 0.72184 |
| SKMC | 0.9074215 | 0.72294 |
| IshikawaTamoxifen | 0.8798994 | 0.72534 |
| HSMM | 0.9038986 | 0.73510 |
| Huh7.5 | 0.8496732 | 0.74472 |
| PanIsletD | 0.8902934 | 0.74564 |
| HGF | 0.8808666 | 0.74726 |
| HTR8svn | 0.8711171 | 0.74922 |
| T47D | 0.8463435 | 0.75174 |
| Huh7 | 0.8465849 | 0.75630 |
| ProgFib | 0.8631362 | 0.75886 |
| HeLaS3IFNa4h | 0.8370172 | 0.75902 |
| HCF | 0.8778511 | 0.76422 |
| Stellate | 0.8508709 | 0.76580 |
| LNCaP | 0.8585987 | 0.77478 |
| HNPCEpiC | 0.8898643 | 0.77936 |
| AG10803 | 0.8687263 | 0.78074 |
| HPAF | 0.8760917 | 0.78100 |
| AG09319 | 0.8591163 | 0.78592 |
| HSMMemb | 0.8533204 | 0.78662 |
| NHDFneo | 0.8623932 | 0.79016 |
| AG04450 | 0.8585015 | 0.79226 |
| HeLaS3 | 0.8340252 | 0.79302 |
| AG04449 | 0.8651103 | 0.79660 |
| HPdLF | 0.8478663 | 0.81772 |
| HPF | 0.8455642 | 0.82288 |
| WI38 | 0.8369328 | 0.82624 |
| HRPEpiC | 0.8682772 | 0.83034 |
| HConF | 0.8326299 | 0.83854 |
| Medullo | 0.7923731 | 0.84172 |
| HAsp | 0.8497929 | 0.84264 |
| NHA | 0.8437386 | 0.84560 |
| LNCaPAndrogen | 0.7791318 | 0.84816 |
| HVMF | 0.8071760 | 0.86214 |
| NHLF | 0.8348613 | 0.86268 |
| HAh | 0.8415542 | 0.87292 |
| HAc | 0.8334302 | 0.87326 |
| HBMEC | 0.8240993 | 0.88238 |
| HCPEpiC | 0.8171407 | 0.88458 |
| Caco2 | 0.6502232 | 0.88820 |
| MCF7Hypoxia | 0.7236736 | 0.89750 |
| HAEpiC | 0.7722982 | 0.91814 |
| SKNSHRA | 0.6733813 | 0.92476 |
| MCF7 | 0.7175963 | 0.94284 |
| BE2C | 0.7014870 | 0.95148 |

# Body mass index 2

| DHS sample | fold enrichment | p value |
|---|---|---|
| Huh7 | 1.1595463 | 0.14628 |
| HRCEpiC | 1.1061608 | 0.15308 |
| RPTEC | 1.0900257 | 0.17920 |
| SAEC | 1.0856606 | 0.19028 |
| HCFaa | 1.0825969 | 0.22890 |
| AoAF | 1.0783560 | 0.24416 |
| HCF | 1.0771652 | 0.25106 |
| AoSMC | 1.0704935 | 0.25296 |
| Huh7.5 | 1.0950191 | 0.26732 |
| HeLaS3 | 1.0797801 | 0.27154 |
| SKNMC | 1.0709442 | 0.27420 |
| HCPEpiC | 1.0571449 | 0.29146 |
| HEEpiC | 1.0533295 | 0.29316 |
| HAEpiC | 1.0601867 | 0.29614 |
| SKMC | 1.0548885 | 0.29962 |
| A549 | 1.0499467 | 0.34986 |
| HepG2 | 1.0537931 | 0.35006 |
| HCM | 1.0395226 | 0.35346 |
| HPAF | 1.0389066 | 0.35562 |
| HSMM | 1.0345867 | 0.36396 |
| HMVECLBl | 1.0414690 | 0.36470 |
| PANC1 | 1.0380574 | 0.37380 |
| NHLF | 1.0323850 | 0.37436 |
| HRPEpiC | 1.0283303 | 0.37794 |
| HRGEC | 1.0351951 | 0.37880 |
| HAsp | 1.0304667 | 0.38052 |
| HeLaS3IFNa4h | 1.0400905 | 0.38396 |
| PrEC | 1.0282784 | 0.38400 |
| HPdLF | 1.0279807 | 0.40112 |
| HCT116 | 1.0287383 | 0.40560 |
| HIPEpiC | 1.0209574 | 0.41918 |
| NHDFAd | 1.0205382 | 0.42014 |
| AG09309 | 1.0186058 | 0.42712 |
| HSMMtube | 1.0172558 | 0.43222 |
| HUVEC | 1.0153405 | 0.44278 |
| HNPCEpiC | 1.0116660 | 0.44674 |
| HMVECLLy | 1.0146901 | 0.44678 |
| BJ | 1.0109924 | 0.45672 |
| AG04449 | 1.0079181 | 0.46782 |
| HMVECdAd | 1.0073014 | 0.47240 |
| Gliobla | 1.0030150 | 0.47712 |
| HPDE6E6E7 | 1.0019899 | 0.48522 |
| Myometr | 1.0014277 | 0.48784 |
| NHA | 1.0007752 | 0.49288 |
| Hepatocytes | 0.9966693 | 0.49306 |
| HMVECdLyAd | 0.9988212 | 0.49714 |
| IshikawaEstradiol | 0.9970746 | 0.49858 |
| HRE | 0.9990653 | 0.50146 |
| HMVECdNeo | 0.9949886 | 0.50558 |
| K562 | 0.9926121 | 0.50812 |
| HMVECdLyNeo | 0.9936713 | 0.51340 |
| 8988T | 0.9852442 | 0.51528 |
| SKNSHRA | 0.9850815 | 0.51740 |
| HFF | 0.9924700 | 0.52310 |
| HAh | 0.9938355 | 0.52642 |
| HPAEC | 0.9851406 | 0.54236 |
| HMF | 0.9864716 | 0.54380 |
| HMVECdBlAd | 0.9825254 | 0.55068 |
| NT2D1 | 0.9849246 | 0.55372 |
| Caco2 | 0.9523804 | 0.55468 |
| BE2C | 0.9798216 | 0.55540 |
| AG04450 | 0.9800494 | 0.55952 |
| HGF | 0.9792263 | 0.56470 |
| PanIslets | 0.9670195 | 0.58048 |
| AG10803 | 0.9720805 | 0.58854 |
| HMVECdBlNeo | 0.9701817 | 0.58886 |
| IshikawaTamoxifen | 0.9644074 | 0.59788 |
| HFFMyc | 0.9724617 | 0.59914 |
| ProgFib | 0.9631965 | 0.60142 |
| AG09319 | 0.9661876 | 0.60428 |
| HAc | 0.9720388 | 0.60504 |
| HVMF | 0.9644235 | 0.60524 |
| HBMEC | 0.9707068 | 0.60612 |
| LNCaPAndrogen | 0.9540375 | 0.60678 |
| RWPE1 | 0.9601492 | 0.61780 |
| MCF7 | 0.9540210 | 0.61996 |
| NHDFneo | 0.9600599 | 0.63158 |
| NHEK | 0.9568332 | 0.63250 |
| PanIsletD | 0.9586575 | 0.63796 |
| HConF | 0.9567301 | 0.64188 |
| GM19239 | 0.9355139 | 0.64316 |
| HTR8svn | 0.9444864 | 0.65538 |
| Stellate | 0.9414019 | 0.65654 |
| WI38 | 0.9477534 | 0.65806 |
| T47D | 0.9198461 | 0.68530 |
| H7hESC | 0.9623842 | 0.68790 |
| pHTE | 0.9327227 | 0.68986 |
| HPF | 0.9323617 | 0.71516 |
| UrotheliaUT189 | 0.9188366 | 0.71868 |
| MonocytesCD14RO01746 | 0.9008380 | 0.71984 |
| HSMMemb | 0.9237507 | 0.71986 |
| H9ES | 0.9171807 | 0.72832 |
| HMEC | 0.9230481 | 0.73164 |
| CD34Mobilized | 0.9100692 | 0.74130 |
| NB4 | 0.9011904 | 0.74738 |
| MCF7Hypoxia | 0.8856257 | 0.75082 |
| Chorion | 0.8699644 | 0.76264 |
| GM12891 | 0.8679580 | 0.77258 |
| Melano | 0.9257224 | 0.77376 |
| CMK | 0.8742305 | 0.77754 |
| WERIRb1 | 0.9120199 | 0.77822 |
| Medullo | 0.8850965 | 0.77822 |
| H1hESC | 0.8784424 | 0.79170 |
| Jurkat | 0.8931854 | 0.79590 |
| LNCaP | 0.8932229 | 0.79986 |
| FibroP | 0.9138287 | 0.80282 |
| Urothelia | 0.8834128 | 0.80542 |
| Fibrobl | 0.8840046 | 0.80632 |
| iPS | 0.8447640 | 0.80664 |
| CD20 | 0.8474343 | 0.80776 |
| HL60 | 0.8466965 | 0.82288 |
| Osteobl | 0.8655559 | 0.83414 |
| GM18507 | 0.8546737 | 0.83588 |
| GM12892 | 0.8251335 | 0.84068 |
| GM19240 | 0.8453807 | 0.86564 |
| CLL | 0.8116376 | 0.86998 |
| GM19238 | 0.8212020 | 0.87628 |
| Th0 | 0.8302246 | 0.87648 |
| Th1 | 0.8027615 | 0.91520 |
| GM12878 | 0.7914256 | 0.91880 |
| GM12865 | 0.8029968 | 0.92854 |
| GM12864 | 0.7862821 | 0.93366 |
| GM06990 | 0.7395187 | 0.94560 |
| Th2 | 0.6932345 | 0.97916 |

# Metabolic traits 1

| DHS sample | fold enrichment | p value |
|---|---|---|
| RPTEC | 1.2488492 | 0.02648 |
| HRCEpiC | 1.2525554 | 0.03118 |
| HRGEC | 1.2410961 | 0.05320 |
| HPAEC | 1.2032686 | 0.09102 |
| HCFaa | 1.1848200 | 0.09370 |
| HL60 | 1.2345892 | 0.10724 |
| HIPEpiC | 1.1633055 | 0.10940 |
| HNPCEpiC | 1.1535062 | 0.11990 |
| HAEpiC | 1.1656093 | 0.12864 |
| MonocytesCD14RO01746 | 1.2061794 | 0.12876 |
| HMVECdBlAd | 1.1624922 | 0.14112 |
| HMVECLBl | 1.1519163 | 0.15208 |
| SAEC | 1.1326866 | 0.15328 |
| HEEpiC | 1.1329792 | 0.15590 |
| HPdLF | 1.1451400 | 0.16686 |
| HGF | 1.1474314 | 0.16848 |
| HAsp | 1.1348785 | 0.17582 |
| HCPEpiC | 1.1248426 | 0.17910 |
| K562 | 1.1612962 | 0.18822 |
| HCM | 1.1221503 | 0.18850 |
| HMVECdBlNeo | 1.1304077 | 0.19506 |
| PrEC | 1.1053021 | 0.19852 |
| HCF | 1.1231293 | 0.20934 |
| Jurkat | 1.1146564 | 0.21912 |
| NHA | 1.1074508 | 0.21928 |
| HPAF | 1.1089652 | 0.22324 |
| SKMC | 1.0991520 | 0.23362 |
| AoSMC | 1.0941614 | 0.23434 |
| HVMF | 1.1099690 | 0.23450 |
| NHLF | 1.0927886 | 0.24768 |
| HRE | 1.0841014 | 0.25918 |
| WI38 | 1.0950249 | 0.26382 |
| HMVECdLyNeo | 1.0947842 | 0.27322 |
| AG04449 | 1.0838369 | 0.27542 |
| HMVECdAd | 1.0958097 | 0.27572 |
| HBMEC | 1.0767725 | 0.28064 |
| HMF | 1.0811683 | 0.28164 |
| HeLaS3IFNa4h | 1.1051974 | 0.28348 |
| NB4 | 1.0900694 | 0.29038 |
| HAc | 1.0728490 | 0.29064 |
| AG04450 | 1.0799334 | 0.29708 |
| HeLaS3 | 1.0876601 | 0.30270 |
| HAh | 1.0639789 | 0.30404 |
| HMVECdLyAd | 1.0789151 | 0.30802 |
| AG09309 | 1.0628987 | 0.32018 |
| HepG2 | 1.0774317 | 0.32210 |
| BJ | 1.0669458 | 0.32266 |
| CMK | 1.0840328 | 0.32648 |
| HMVECdNeo | 1.0680079 | 0.33062 |
| HMVECLLy | 1.0564110 | 0.35600 |
| AoAF | 1.0523181 | 0.35818 |
| HPDE6E6E7 | 1.0464989 | 0.38254 |
| GM12865 | 1.0426322 | 0.39058 |
| GM12878 | 1.0451392 | 0.39132 |
| HRPEpiC | 1.0319562 | 0.39834 |
| AG09319 | 1.0369865 | 0.39866 |
| AG10803 | 1.0330058 | 0.40456 |
| A549 | 1.0356635 | 0.40974 |
| HPF | 1.0305228 | 0.41148 |
| IshikawaEstradiol | 1.0286570 | 0.42386 |
| HConF | 1.0248758 | 0.42514 |
| BE2C | 1.0244760 | 0.43896 |
| NHEK | 1.0166909 | 0.45136 |
| UrotheliaUT189 | 1.0120105 | 0.46810 |
| GM12864 | 1.0076889 | 0.47844 |
| Urothelia | 0.9986841 | 0.49976 |
| H7hESC | 0.9953938 | 0.51260 |
| HUVEC | 0.9870736 | 0.52454 |
| IshikawaTamoxifen | 0.9869578 | 0.52760 |
| HSMM | 0.9909064 | 0.52826 |
| CD34Mobilized | 0.9838717 | 0.53726 |
| HFF | 0.9851634 | 0.53802 |
| FibroP | 0.9890990 | 0.54248 |
| GM18507 | 0.9786242 | 0.54794 |
| Myometr | 0.9789199 | 0.54908 |
| GM19240 | 0.9794966 | 0.55188 |
| NHDFAd | 0.9808103 | 0.55554 |
| HTR8svn | 0.9755698 | 0.55846 |
| NHDFneo | 0.9718183 | 0.57154 |
| RWPE1 | 0.9644963 | 0.58234 |
| PanIsletD | 0.9690714 | 0.58542 |
| NT2D1 | 0.9684833 | 0.58738 |
| Stellate | 0.9601542 | 0.59288 |
| HFFMyc | 0.9674222 | 0.59932 |
| Th2 | 0.9511431 | 0.60300 |
| Huh7 | 0.9464204 | 0.61360 |
| PANC1 | 0.9493942 | 0.61616 |
| Melano | 0.9593978 | 0.63344 |
| GM06990 | 0.9304322 | 0.63382 |
| Th0 | 0.9442756 | 0.64140 |
| HSMMemb | 0.9415719 | 0.64358 |
| MCF7 | 0.9350424 | 0.65132 |
| Gliobla | 0.9245003 | 0.65742 |
| HSMMtube | 0.9385879 | 0.67120 |
| CD20 | 0.9001480 | 0.68434 |
| GM19239 | 0.9091872 | 0.68536 |
| Huh7.5 | 0.9094299 | 0.69066 |
| HCT116 | 0.9102817 | 0.69220 |
| GM19238 | 0.9081049 | 0.69400 |
| MCF7Hypoxia | 0.8993389 | 0.70792 |
| CLL | 0.8873168 | 0.72100 |
| PanIslets | 0.8913562 | 0.73846 |
| LNCaPAndrogen | 0.8818900 | 0.74456 |
| ProgFib | 0.8896962 | 0.74816 |
| Hepatocytes | 0.8672271 | 0.75270 |
| Fibrobl | 0.8943926 | 0.76088 |
| Th1 | 0.8949410 | 0.76224 |
| GM12891 | 0.8609277 | 0.76718 |
| pHTE | 0.8800059 | 0.77800 |
| Osteobl | 0.8814054 | 0.78306 |
| Caco2 | 0.7974031 | 0.79668 |
| 8988T | 0.8214349 | 0.80368 |
| HMEC | 0.8683561 | 0.80544 |
| Medullo | 0.8504585 | 0.82276 |
| GM12892 | 0.8213762 | 0.83176 |
| SKNSHRA | 0.7967004 | 0.84400 |
| T47D | 0.8060782 | 0.84408 |
| WERIRb1 | 0.8461859 | 0.85112 |
| LNCaP | 0.8108938 | 0.88932 |
| Chorion | 0.7516875 | 0.89306 |
| H9ES | 0.7875211 | 0.89672 |
| SKNMC | 0.7981950 | 0.89960 |
| iPS | 0.7192012 | 0.92620 |
| H1hESC | 0.7372736 | 0.92900 |

# Schizophrenia

| DHS sample | fold enrichment | p value |
|---|---|---|
| HPdLF | 1.2950373 | 0.03034 |
| HAsp | 1.2229473 | 0.05344 |
| NHDFneo | 1.2074698 | 0.08472 |
| HGF | 1.2191042 | 0.08708 |
| AG10803 | 1.1939684 | 0.10394 |
| NHA | 1.1726852 | 0.10486 |
| BJ | 1.1865419 | 0.10976 |
| AG04450 | 1.1943812 | 0.11182 |
| NHDFAd | 1.1691162 | 0.11530 |
| HIPEpiC | 1.1642969 | 0.11632 |
| WI38 | 1.1911723 | 0.11654 |
| HCFaa | 1.1705840 | 0.12012 |
| NHLF | 1.1583473 | 0.12174 |
| AG04449 | 1.1724106 | 0.12210 |
| AG09319 | 1.1843538 | 0.12354 |
| HPF | 1.1648434 | 0.14126 |
| SKMC | 1.1339936 | 0.16586 |
| HFF | 1.1403586 | 0.16826 |
| AG09309 | 1.1349357 | 0.17336 |
| HVMF | 1.1425009 | 0.19428 |
| HBMEC | 1.1132681 | 0.19538 |
| HNPCEpiC | 1.1077334 | 0.19758 |
| HAc | 1.1086682 | 0.20222 |
| HAh | 1.0926641 | 0.22464 |
| HFFMyc | 1.1069141 | 0.22798 |
| Medullo | 1.1311256 | 0.23916 |
| HConF | 1.0991054 | 0.25652 |
| NB4 | 1.1131140 | 0.26072 |
| AoAF | 1.0928767 | 0.26948 |
| HMF | 1.0874090 | 0.27242 |
| HCPEpiC | 1.0822825 | 0.27412 |
| AoSMC | 1.0822890 | 0.27688 |
| HMVECLBl | 1.0859441 | 0.29106 |
| MonocytesCD14RO01746 | 1.1029427 | 0.29864 |
| HSMM | 1.0585940 | 0.33084 |
| SKNSHRA | 1.0786710 | 0.33844 |
| HAEpiC | 1.0514127 | 0.36328 |
| GM12864 | 1.0546659 | 0.37580 |
| SKNMC | 1.0401579 | 0.37874 |
| HRPEpiC | 1.0344617 | 0.37996 |
| HPAF | 1.0347269 | 0.40174 |
| CD34Mobilized | 1.0292822 | 0.42300 |
| Stellate | 1.0311013 | 0.42382 |
| HCM | 1.0144618 | 0.45600 |
| HRE | 1.0112460 | 0.45822 |
| RPTEC | 1.0046738 | 0.48114 |
| FibroP | 1.0059231 | 0.48184 |
| HSMMtube | 1.0041348 | 0.48260 |
| Fibrobl | 1.0027675 | 0.48794 |
| GM06990 | 0.9977260 | 0.49158 |
| CLL | 0.9958952 | 0.49226 |
| HCF | 0.9976727 | 0.49752 |
| Caco2 | 0.9804374 | 0.50040 |
| HRGEC | 0.9948234 | 0.50794 |
| HL60 | 0.9876076 | 0.50874 |
| HRCEpiC | 0.9934421 | 0.51478 |
| UrotheliaUT189 | 0.9890870 | 0.51638 |
| HMVECdBlNeo | 0.9907363 | 0.51700 |
| ProgFib | 0.9869068 | 0.51732 |
| GM12878 | 0.9816686 | 0.52318 |
| HTR8svn | 0.9825603 | 0.53262 |
| BE2C | 0.9741744 | 0.54482 |
| GM12865 | 0.9668564 | 0.56440 |
| HUVEC | 0.9672610 | 0.56466 |
| Osteobl | 0.9647085 | 0.57088 |
| CMK | 0.9464581 | 0.58348 |
| Chorion | 0.9366806 | 0.59188 |
| Th0 | 0.9513666 | 0.59222 |
| Melano | 0.9672059 | 0.59654 |
| HPAEC | 0.9585409 | 0.59700 |
| GM19240 | 0.9504359 | 0.60040 |
| LNCaP | 0.9503053 | 0.60042 |
| HSMMemb | 0.9526392 | 0.60292 |
| HMVECdBlAd | 0.9561186 | 0.60488 |
| PanIsletD | 0.9517590 | 0.61784 |
| HMVECdLyNeo | 0.9435351 | 0.62944 |
| Myometr | 0.9426467 | 0.63184 |
| 8988T | 0.9075911 | 0.63478 |
| LNCaPAndrogen | 0.9216360 | 0.64008 |
| GM12892 | 0.9153089 | 0.64234 |
| iPS | 0.9106809 | 0.64318 |
| CD20 | 0.9081176 | 0.64544 |
| HMVECdAd | 0.9338649 | 0.64738 |
| H1hESC | 0.9162422 | 0.64920 |
| GM12891 | 0.9062866 | 0.65278 |
| GM18507 | 0.9191702 | 0.65384 |
| GM19238 | 0.9167209 | 0.65404 |
| Th1 | 0.9203084 | 0.66136 |
| pHTE | 0.9215339 | 0.67108 |
| Gliobla | 0.9014157 | 0.67898 |
| PanIslets | 0.9057690 | 0.68578 |
| K562 | 0.8981614 | 0.68614 |
| Th2 | 0.8921942 | 0.68762 |
| WERIRb1 | 0.9274167 | 0.69342 |
| RWPE1 | 0.9123760 | 0.69484 |
| Urothelia | 0.9071385 | 0.69492 |
| HMEC | 0.9136086 | 0.69672 |
| T47D | 0.8856485 | 0.70160 |
| H9ES | 0.8941878 | 0.71044 |
| GM19239 | 0.8735445 | 0.71474 |
| A549 | 0.8865449 | 0.71722 |
| HMVECLLy | 0.9040496 | 0.71842 |
| NT2D1 | 0.9203447 | 0.72924 |
| HMVECdNeo | 0.8892934 | 0.74592 |
| Huh7.5 | 0.8576224 | 0.75188 |
| Hepatocytes | 0.8377439 | 0.76268 |
| PANC1 | 0.8742377 | 0.76766 |
| HMVECdLyAd | 0.8772932 | 0.76900 |
| MCF7Hypoxia | 0.8485014 | 0.77634 |
| MCF7 | 0.8563169 | 0.80226 |
| Jurkat | 0.8583417 | 0.81086 |
| HCT116 | 0.8415081 | 0.81310 |
| IshikawaEstradiol | 0.8486392 | 0.82284 |
| HepG2 | 0.8184733 | 0.82736 |
| Huh7 | 0.8135191 | 0.83142 |
| H7hESC | 0.9014634 | 0.84104 |
| SAEC | 0.8628650 | 0.86646 |
| NHEK | 0.8227333 | 0.87124 |
| IshikawaTamoxifen | 0.8050639 | 0.87740 |
| HEEpiC | 0.8479882 | 0.88670 |
| HeLaS3IFNa4h | 0.7462258 | 0.90742 |
| HeLaS3 | 0.7657213 | 0.91092 |
| PrEC | 0.8253258 | 0.91396 |
| HPDE6E6E7 | 0.7534391 | 0.93210 |

# Body mass index 3

| DHS sample | fold enrichment | p value |
|---|---|---|
| RPTEC | 1.2603254 | 0.03410 |
| HIPEpiC | 1.2438759 | 0.05594 |
| AG09319 | 1.2498225 | 0.07764 |
| AG04449 | 1.2256319 | 0.08418 |
| HAsp | 1.2148522 | 0.08998 |
| HPdLF | 1.2301388 | 0.09182 |
| HCPEpiC | 1.1993198 | 0.09738 |
| BJ | 1.2070250 | 0.10832 |
| HCFaa | 1.1959847 | 0.11368 |
| HNPCEpiC | 1.1727202 | 0.11972 |
| PANC1 | 1.2204312 | 0.12308 |
| AG09309 | 1.1796041 | 0.12404 |
| HRCEpiC | 1.1721115 | 0.12684 |
| AG10803 | 1.1916549 | 0.12938 |
| HRPEpiC | 1.1623518 | 0.13090 |
| SKMC | 1.1785825 | 0.13288 |
| HGF | 1.1905346 | 0.13594 |
| HAEpiC | 1.1826826 | 0.14034 |
| AG04450 | 1.1832978 | 0.14550 |
| HVMF | 1.1782001 | 0.15486 |
| HFF | 1.1571590 | 0.16114 |
| HBMEC | 1.1509496 | 0.16222 |
| AoSMC | 1.1456403 | 0.16456 |
| HPF | 1.1629563 | 0.16572 |
| HConF | 1.1583204 | 0.17176 |
| WI38 | 1.1603361 | 0.18140 |
| NHDFneo | 1.1384232 | 0.20474 |
| HMF | 1.1303402 | 0.20500 |
| HCF | 1.1342716 | 0.21382 |
| HPAF | 1.1242118 | 0.22096 |
| HRE | 1.1093094 | 0.22858 |
| HAc | 1.1059922 | 0.23394 |
| SAEC | 1.1011149 | 0.23794 |
| NHA | 1.1069813 | 0.24582 |
| HCM | 1.1059661 | 0.24720 |
| HEEpiC | 1.0946989 | 0.25656 |
| NHDFAd | 1.0987984 | 0.26178 |
| NHLF | 1.0972464 | 0.26340 |
| HeLaS3 | 1.1166898 | 0.26466 |
| PrEC | 1.0901135 | 0.26512 |
| HCT116 | 1.1131713 | 0.27484 |
| HMVECLBl | 1.0989344 | 0.28602 |
| HAh | 1.0676992 | 0.31380 |
| AoAF | 1.0682139 | 0.34060 |
| HeLaS3IFNa4h | 1.0544849 | 0.37888 |
| A549 | 1.0475473 | 0.38812 |
| HMVECdBlNeo | 1.0365693 | 0.41236 |
| HFFMyc | 1.0273404 | 0.42750 |
| HRGEC | 1.0241308 | 0.43830 |
| Jurkat | 1.0160970 | 0.45348 |
| HMVECdBlAd | 1.0177003 | 0.45418 |
| RWPE1 | 1.0032625 | 0.47638 |
| HMVECdAd | 1.0017266 | 0.48740 |
| HSMM | 0.9930340 | 0.51470 |
| WERIRb1 | 0.9864823 | 0.52544 |
| HMVECdLyNeo | 0.9798228 | 0.53428 |
| HTR8svn | 0.9805836 | 0.53560 |
| GM12864 | 0.9739178 | 0.53806 |
| HPAEC | 0.9785674 | 0.54070 |
| SKNSHRA | 0.9549566 | 0.56212 |
| HMVECdNeo | 0.9625837 | 0.56938 |
| Gliobla | 0.9547231 | 0.57180 |
| GM12865 | 0.9483724 | 0.59240 |
| PanIsletD | 0.9598524 | 0.59316 |
| Stellate | 0.9449148 | 0.60204 |
| SKNMC | 0.9495356 | 0.60804 |
| HepG2 | 0.9407521 | 0.60850 |
| ProgFib | 0.9432070 | 0.61106 |
| HMVECdLyAd | 0.9371892 | 0.62304 |
| Caco2 | 0.8896574 | 0.62496 |
| IshikawaEstradiol | 0.9366717 | 0.62662 |
| HPDE6E6E7 | 0.9350187 | 0.62928 |
| HMVECLLy | 0.9349929 | 0.63018 |
| Hepatocytes | 0.9141507 | 0.63782 |
| HSMMemb | 0.9292910 | 0.65156 |
| 8988T | 0.8975479 | 0.65756 |
| LNCaP | 0.9225224 | 0.66174 |
| GM18507 | 0.9096128 | 0.66326 |
| GM06990 | 0.8951823 | 0.66408 |
| NHEK | 0.9229071 | 0.66608 |
| IshikawaTamoxifen | 0.9162449 | 0.66758 |
| H9ES | 0.9077405 | 0.67176 |
| CD20 | 0.8783702 | 0.67740 |
| HUVEC | 0.9071824 | 0.68638 |
| H1hESC | 0.8821035 | 0.70462 |
| CD34Mobilized | 0.8943432 | 0.70494 |
| MonocytesCD14RO01746 | 0.8750173 | 0.70704 |
| Th2 | 0.8737830 | 0.71156 |
| K562 | 0.8772366 | 0.71292 |
| NB4 | 0.8801752 | 0.72360 |
| Myometr | 0.8966280 | 0.72684 |
| GM19239 | 0.8597758 | 0.72704 |
| PanIslets | 0.8824402 | 0.72822 |
| HMEC | 0.8952260 | 0.72872 |
| GM12878 | 0.8627834 | 0.73326 |
| HSMMtube | 0.9006296 | 0.73876 |
| Chorion | 0.8416773 | 0.74356 |
| Osteobl | 0.8790207 | 0.74688 |
| FibroP | 0.9062266 | 0.74696 |
| pHTE | 0.8728619 | 0.75726 |
| HL60 | 0.8459477 | 0.75828 |
| LNCaPAndrogen | 0.8528675 | 0.75864 |
| Fibrobl | 0.8758392 | 0.76010 |
| Huh7 | 0.8493295 | 0.76172 |
| H7hESC | 0.9123488 | 0.76720 |
| CLL | 0.8293332 | 0.76732 |
| GM19240 | 0.8609590 | 0.76850 |
| GM12891 | 0.8262609 | 0.77022 |
| GM19238 | 0.8329859 | 0.78394 |
| Th0 | 0.8460389 | 0.78630 |
| GM12892 | 0.8171306 | 0.78708 |
| NT2D1 | 0.8809816 | 0.79028 |
| iPS | 0.8082809 | 0.79246 |
| CMK | 0.8123919 | 0.79570 |
| BE2C | 0.8369657 | 0.81474 |
| UrotheliaUT189 | 0.8360719 | 0.81538 |
| Huh7.5 | 0.8103973 | 0.81756 |
| MCF7Hypoxia | 0.8048610 | 0.81770 |
| Melano | 0.8711725 | 0.82814 |
| MCF7 | 0.8210531 | 0.83522 |
| Urothelia | 0.8169463 | 0.84240 |
| Medullo | 0.8035769 | 0.84844 |
| T47D | 0.7683467 | 0.86382 |
| Th1 | 0.7715864 | 0.89026 |

# Blood metabolites 1

| DHS sample | fold enrichment | p value |
|---|---|---|
| RPTEC | 1.0821592 | 0.12682 |
| SAEC | 1.0453749 | 0.26096 |
| HCFaa | 1.0293086 | 0.35132 |
| HRCEpiC | 1.0270122 | 0.36036 |
| HEEpiC | 1.0163281 | 0.41036 |
| HPDE6E6E7 | 1.0090529 | 0.45518 |
| PrEC | 0.9994527 | 0.49734 |
| K562 | 0.9942697 | 0.51888 |
| HRGEC | 0.9937383 | 0.52714 |
| PANC1 | 0.9910487 | 0.52778 |
| HIPEpiC | 0.9936786 | 0.53270 |
| A549 | 0.9896705 | 0.53274 |
| Jurkat | 0.9906347 | 0.53906 |
| HAsp | 0.9891848 | 0.54838 |
| HPAEC | 0.9828224 | 0.57676 |
| IshikawaEstradiol | 0.9801487 | 0.58316 |
| HGF | 0.9785009 | 0.59534 |
| NHEK | 0.9731276 | 0.62104 |
| HPAF | 0.9731572 | 0.63156 |
| RWPE1 | 0.9697487 | 0.63528 |
| IshikawaTamoxifen | 0.9663365 | 0.64102 |
| HRE | 0.9720115 | 0.64456 |
| HCPEpiC | 0.9716412 | 0.64718 |
| HeLaS3 | 0.9608134 | 0.64924 |
| HRPEpiC | 0.9707169 | 0.65370 |
| HepG2 | 0.9599049 | 0.65478 |
| AoSMC | 0.9698964 | 0.66784 |
| AoAF | 0.9612403 | 0.68046 |
| HCF | 0.9583645 | 0.69144 |
| HMVECdBlAd | 0.9577219 | 0.69280 |
| HMVECdBlNeo | 0.9563278 | 0.69552 |
| NHLF | 0.9558176 | 0.71538 |
| HMVECdLyAd | 0.9465409 | 0.72412 |
| HMVECdNeo | 0.9455223 | 0.73104 |
| AG09309 | 0.9536454 | 0.73110 |
| HMVECdAd | 0.9439942 | 0.73160 |
| HMVECLBl | 0.9475551 | 0.73510 |
| HAEpiC | 0.9477482 | 0.73744 |
| NHDFAd | 0.9508468 | 0.73924 |
| Th2 | 0.9297159 | 0.74550 |
| HNPCEpiC | 0.9517560 | 0.74750 |
| HMVECdLyNeo | 0.9411378 | 0.74940 |
| HConF | 0.9427452 | 0.75300 |
| HeLaS3IFNa4h | 0.9250290 | 0.75518 |
| MCF7 | 0.9338444 | 0.75684 |
| HCM | 0.9460910 | 0.76440 |
| SKNMC | 0.9325484 | 0.77048 |
| AG09319 | 0.9349495 | 0.77226 |
| Huh7 | 0.9236514 | 0.77456 |
| HCT116 | 0.9243528 | 0.77760 |
| HMVECLLy | 0.9324113 | 0.77824 |
| CMK | 0.9110452 | 0.78154 |
| HPdLF | 0.9338692 | 0.78658 |
| HBMEC | 0.9365052 | 0.79680 |
| NHA | 0.9336472 | 0.80340 |
| HMF | 0.9314580 | 0.80742 |
| HUVEC | 0.9212365 | 0.81148 |
| Myometr | 0.9246328 | 0.81714 |
| AG04449 | 0.9256004 | 0.82288 |
| HL60 | 0.9006426 | 0.82764 |
| GM18507 | 0.9027839 | 0.84084 |
| NB4 | 0.9034267 | 0.84656 |
| AG10803 | 0.9142999 | 0.84808 |
| HVMF | 0.9113480 | 0.84848 |
| MCF7Hypoxia | 0.8888247 | 0.85146 |
| CLL | 0.8836804 | 0.85430 |
| GM12878 | 0.8923976 | 0.85542 |
| NHDFneo | 0.9122098 | 0.85676 |
| BE2C | 0.8921714 | 0.86444 |
| Hepatocytes | 0.8751102 | 0.86912 |
| SKMC | 0.9131811 | 0.87100 |
| Huh7.5 | 0.8823771 | 0.87196 |
| HFF | 0.9119825 | 0.87360 |
| LNCaPAndrogen | 0.8848097 | 0.87470 |
| GM19239 | 0.8757015 | 0.87822 |
| HAh | 0.9180776 | 0.87848 |
| MonocytesCD14RO01746 | 0.8772022 | 0.88014 |
| Stellate | 0.8879502 | 0.88308 |
| WI38 | 0.8995345 | 0.88338 |
| Urothelia | 0.8877799 | 0.88708 |
| BJ | 0.8994062 | 0.89172 |
| GM12865 | 0.8828154 | 0.89282 |
| HAc | 0.9077738 | 0.89414 |
| CD34Mobilized | 0.8864351 | 0.89442 |
| HSMMemb | 0.8895508 | 0.89682 |
| UrotheliaUT189 | 0.8791234 | 0.90028 |
| HFFMyc | 0.9037062 | 0.90060 |
| AG04450 | 0.8913684 | 0.90302 |
| HPF | 0.8916811 | 0.90326 |
| SKNSHRA | 0.8451870 | 0.90370 |
| HSMM | 0.9052429 | 0.90616 |
| CD20 | 0.8432842 | 0.90812 |
| HTR8svn | 0.8781791 | 0.91410 |
| Melano | 0.9032976 | 0.92342 |
| GM12864 | 0.8618828 | 0.92374 |
| Th0 | 0.8656295 | 0.92802 |
| H7hESC | 0.9093584 | 0.92862 |
| PanIslets | 0.8593034 | 0.93218 |
| PanIsletD | 0.8849632 | 0.93248 |
| T47D | 0.8330129 | 0.93732 |
| H9ES | 0.8515893 | 0.94072 |
| ProgFib | 0.8583429 | 0.94076 |
| Gliobla | 0.8436997 | 0.94110 |
| GM19240 | 0.8566293 | 0.94374 |
| NT2D1 | 0.8777362 | 0.94856 |
| GM19238 | 0.8360966 | 0.94898 |
| 8988T | 0.8101040 | 0.94922 |
| GM06990 | 0.8150359 | 0.95340 |
| GM12891 | 0.8159740 | 0.95372 |
| pHTE | 0.8532837 | 0.95418 |
| WERIRb1 | 0.8529238 | 0.96112 |
| LNCaP | 0.8451077 | 0.96206 |
| Medullo | 0.8321715 | 0.96318 |
| Th1 | 0.8361409 | 0.96714 |
| HSMMtube | 0.8595382 | 0.96806 |
| H1hESC | 0.8102397 | 0.96902 |
| Caco2 | 0.7326575 | 0.97118 |
| HMEC | 0.8415538 | 0.97188 |
| FibroP | 0.8636438 | 0.97520 |
| Chorion | 0.7769368 | 0.97544 |
| GM12892 | 0.7811741 | 0.97770 |
| iPS | 0.7670183 | 0.97814 |
| Fibrobl | 0.8173271 | 0.98334 |
| Osteobl | 0.8030721 | 0.98762 |

## Metabolic syndrome traits

| DHS sample | fold enrichment | p value |
|---|---|---|
| HCFaa | 1.2106938 | 0.03772 |
| RPTEC | 1.1866513 | 0.04174 |
| Jurkat | 1.2046547 | 0.05202 |
| HGF | 1.1834753 | 0.07728 |
| SKMC | 1.1645649 | 0.08244 |
| HRCEpiC | 1.1552444 | 0.08660 |
| HAEpiC | 1.1553576 | 0.10372 |
| HNPCEpiC | 1.1245423 | 0.12826 |
| HCPEpiC | 1.1269610 | 0.13272 |
| HAsp | 1.1340045 | 0.13600 |
| HIPEpiC | 1.1153112 | 0.14768 |
| HVMF | 1.1324379 | 0.14934 |
| HPdLF | 1.1288875 | 0.15006 |
| HCF | 1.1224486 | 0.16500 |
| HPAF | 1.1151862 | 0.16520 |
| HL60 | 1.1544793 | 0.16746 |
| NHLF | 1.1124117 | 0.16874 |
| HCM | 1.1097185 | 0.16900 |
| AG04449 | 1.1120532 | 0.17522 |
| AG04450 | 1.1058483 | 0.19618 |
| NHA | 1.1012460 | 0.19644 |
| HPAEC | 1.1077380 | 0.19724 |
| WI38 | 1.1043316 | 0.19784 |
| HEEpiC | 1.0916772 | 0.20094 |
| HeLaS3 | 1.1272764 | 0.20182 |
| SAEC | 1.0875838 | 0.20758 |
| AoSMC | 1.0853839 | 0.21396 |
| K562 | 1.1182430 | 0.21754 |
| PrEC | 1.0794031 | 0.22522 |
| PANC1 | 1.1050305 | 0.22650 |
| HMVECLBl | 1.0955627 | 0.22672 |
| HRGEC | 1.0929730 | 0.22748 |
| NB4 | 1.1051285 | 0.22750 |
| HBMEC | 1.0816824 | 0.23052 |
| BE2C | 1.1048615 | 0.23102 |
| AG09309 | 1.0823342 | 0.23298 |
| HMVECdAd | 1.0995304 | 0.23388 |
| HAc | 1.0772616 | 0.24162 |
| AG09319 | 1.0853680 | 0.24724 |
| HAh | 1.0711096 | 0.25372 |
| AoAF | 1.0771235 | 0.26252 |
| MonocytesCD14RO01746 | 1.0960956 | 0.27334 |
| HepG2 | 1.0880085 | 0.27396 |
| BJ | 1.0722113 | 0.27410 |
| HRE | 1.0652234 | 0.27732 |
| HPDE6E6E7 | 1.0717906 | 0.29700 |
| HConF | 1.0645016 | 0.29764 |
| HMVECdNeo | 1.0667985 | 0.30744 |
| HMVECdBlNeo | 1.0651203 | 0.30816 |
| HMVECdLyNeo | 1.0651141 | 0.30936 |
| HMVECdBlAd | 1.0569762 | 0.32368 |
| HeLaS3IFNa4h | 1.0705798 | 0.32540 |
| HPF | 1.0502779 | 0.33862 |
| AG10803 | 1.0460574 | 0.34792 |
| HSMMemb | 1.0486425 | 0.35084 |
| Myometr | 1.0430704 | 0.36126 |
| HMVECdLyAd | 1.0464135 | 0.36250 |
| HMVECLLy | 1.0458850 | 0.36276 |
| HSMM | 1.0354698 | 0.36898 |
| HFF | 1.0358005 | 0.37706 |
| HMF | 1.0317940 | 0.38842 |
| NHEK | 1.0244718 | 0.41788 |
| CMK | 1.0285076 | 0.42234 |
| NHDFneo | 1.0203132 | 0.43016 |
| Stellate | 1.0125831 | 0.45874 |
| RWPE1 | 1.0020457 | 0.48468 |
| HRPEpiC | 1.0028844 | 0.48712 |
| A549 | 0.9988151 | 0.49222 |
| HUVEC | 0.9988395 | 0.49388 |
| NT2D1 | 0.9974030 | 0.50216 |
| Huh7 | 0.9957667 | 0.50288 |
| HTR8svn | 0.9963400 | 0.50814 |
| IshikawaEstradiol | 0.9939170 | 0.50828 |
| GM12878 | 0.9936120 | 0.50864 |
| PanIsletD | 0.9957409 | 0.51042 |
| Th2 | 0.9886192 | 0.52110 |
| HFFMyc | 0.9931772 | 0.52206 |
| GM12865 | 0.9843463 | 0.53458 |
| WERIRb1 | 0.9876785 | 0.53472 |
| GM12864 | 0.9791756 | 0.55070 |
| NHDFAd | 0.9846928 | 0.55350 |
| Caco2 | 0.9611183 | 0.55362 |
| HSMMtube | 0.9821229 | 0.55754 |
| CD20 | 0.9692605 | 0.55924 |
| Urothelia | 0.9684591 | 0.58416 |
| IshikawaTamoxifen | 0.9632059 | 0.59618 |
| Gliobla | 0.9546170 | 0.60554 |
| SKNSHRA | 0.9430017 | 0.60858 |
| H7hESC | 0.9741385 | 0.60892 |
| UrotheliaUT189 | 0.9556404 | 0.61654 |
| FibroP | 0.9675644 | 0.62690 |
| CD34Mobilized | 0.9512218 | 0.62960 |
| ProgFib | 0.9514432 | 0.63030 |
| HCT116 | 0.9437821 | 0.64274 |
| Fibrobl | 0.9509223 | 0.64776 |
| GM19240 | 0.9420956 | 0.66252 |
| Th0 | 0.9414167 | 0.66372 |
| CLL | 0.9229279 | 0.67422 |
| Huh7.5 | 0.9249497 | 0.67732 |
| GM19239 | 0.9215388 | 0.67970 |
| 8988T | 0.9094162 | 0.68434 |
| GM19238 | 0.9224895 | 0.68824 |
| PanIslets | 0.9274399 | 0.69466 |
| pHTE | 0.9304402 | 0.69930 |
| GM06990 | 0.9075377 | 0.70416 |
| Osteobl | 0.9295048 | 0.70616 |
| Th1 | 0.9241213 | 0.71430 |
| Hepatocytes | 0.9009709 | 0.71528 |
| Melano | 0.9424110 | 0.71928 |
| GM12891 | 0.8965107 | 0.72776 |
| LNCaPAndrogen | 0.9004610 | 0.73784 |
| GM18507 | 0.9027551 | 0.74512 |
| T47D | 0.8880933 | 0.74598 |
| Medullo | 0.9008005 | 0.75320 |
| SKNMC | 0.9033946 | 0.76142 |
| GM12892 | 0.8795731 | 0.76706 |
| HMEC | 0.9050954 | 0.77190 |
| Chorion | 0.8573226 | 0.78556 |
| MCF7 | 0.8840656 | 0.79692 |
| LNCaP | 0.8829289 | 0.80854 |
| H9ES | 0.8483568 | 0.85818 |
| MCF7Hypoxia | 0.8289872 | 0.86608 |
| iPS | 0.8046045 | 0.87290 |
| H1hESC | 0.8159359 | 0.88560 |

## Childhood obesity

| DHS sample | fold enrichment | p value |
|---|---|---|
| Caco2 | 1.4297222 | 0.06818 |
| MonocytesCD14RO01746 | 1.2841734 | 0.08880 |
| CD34Mobilized | 1.2342422 | 0.09946 |
| Jurkat | 1.1930329 | 0.12590 |
| Urothelia | 1.2030769 | 0.12992 |
| HCFaa | 1.1595682 | 0.13438 |
| GM12892 | 1.2386455 | 0.13486 |
| HBMEC | 1.1548664 | 0.13872 |
| CMK | 1.2412268 | 0.14640 |
| HL60 | 1.2169400 | 0.14778 |
| LNCaP | 1.1719817 | 0.15014 |
| pHTE | 1.1728689 | 0.15572 |
| NB4 | 1.1829361 | 0.15916 |
| HSMM | 1.1345347 | 0.16052 |
| GM12891 | 1.2086690 | 0.16964 |
| FibroP | 1.1250348 | 0.17174 |
| HMEC | 1.1523553 | 0.17254 |
| Gliobla | 1.1874781 | 0.17426 |
| HSMMtube | 1.1355187 | 0.17446 |
| 8988T | 1.2105948 | 0.17630 |
| CD20 | 1.2100815 | 0.18116 |
| HAEpiC | 1.1336983 | 0.18550 |
| MCF7Hypoxia | 1.1708725 | 0.20346 |
| HRE | 1.1141040 | 0.20380 |
| RWPE1 | 1.1386695 | 0.20466 |
| PanIsletD | 1.1190323 | 0.20942 |
| PanIslets | 1.1453666 | 0.21114 |
| UrotheliaUT189 | 1.1447140 | 0.21118 |
| GM19240 | 1.1419826 | 0.21264 |
| HeLaS3 | 1.1508168 | 0.21272 |
| Th1 | 1.1383283 | 0.21440 |
| HCM | 1.1086354 | 0.21820 |
| H1hESC | 1.1508620 | 0.21822 |
| CLL | 1.1650581 | 0.21936 |
| GM19239 | 1.1584897 | 0.21982 |
| MCF7 | 1.1335998 | 0.22004 |
| HCPEpiC | 1.1078947 | 0.22446 |
| iPS | 1.1635175 | 0.22602 |
| Stellate | 1.1297303 | 0.23260 |
| HVMF | 1.1158413 | 0.23568 |
| HFF | 1.1051425 | 0.23706 |
| Fibrobl | 1.1155498 | 0.23796 |
| HAh | 1.0927189 | 0.23870 |
| HeLaS3IFNa4h | 1.1466032 | 0.23952 |
| NHEK | 1.1125737 | 0.24152 |
| HPDE6E6E7 | 1.1228269 | 0.24158 |
| HTR8svn | 1.1223194 | 0.24226 |
| GM06990 | 1.1476742 | 0.24296 |
| NT2D1 | 1.0908774 | 0.24582 |
| GM12864 | 1.1240371 | 0.24902 |
| H7hESC | 1.0742166 | 0.24934 |
| HIPEpiC | 1.0913992 | 0.25260 |
| GM12878 | 1.1300555 | 0.25286 |
| HMF | 1.0938362 | 0.25830 |
| GM19238 | 1.1269344 | 0.25910 |
| HSMMemb | 1.1056315 | 0.26080 |
| T47D | 1.1267616 | 0.26494 |
| Chorion | 1.1298052 | 0.26702 |
| ProgFib | 1.1034603 | 0.26988 |
| Th0 | 1.1097363 | 0.27002 |
| Medullo | 1.1100555 | 0.27148 |
| Myometr | 1.0939737 | 0.27332 |
| AG10803 | 1.0906348 | 0.27444 |
| Osteobl | 1.0961994 | 0.28326 |
| HRCEpiC | 1.0767181 | 0.28654 |
| AG09309 | 1.0791014 | 0.28856 |
| NHLF | 1.0788622 | 0.28974 |
| HNPCEpiC | 1.0732509 | 0.29244 |
| RPTEC | 1.0686617 | 0.29246 |
| HMVECLBl | 1.0856009 | 0.29430 |
| Melano | 1.0696284 | 0.29672 |
| AG04449 | 1.0733576 | 0.31244 |
| NHA | 1.0701289 | 0.31370 |
| LNCaPAndrogen | 1.0858318 | 0.31416 |
| AoSMC | 1.0636212 | 0.31720 |
| HAsp | 1.0658358 | 0.31948 |
| HPAF | 1.0667149 | 0.32128 |
| HUVEC | 1.0758421 | 0.32220 |
| Th2 | 1.0895597 | 0.32374 |
| Hepatocytes | 1.0855195 | 0.32928 |
| GM18507 | 1.0752153 | 0.33640 |
| HFFMyc | 1.0606680 | 0.33774 |
| HPdLF | 1.0613210 | 0.34562 |
| AoAF | 1.0523400 | 0.36426 |
| AG04450 | 1.0554466 | 0.36482 |
| H9ES | 1.0527568 | 0.36668 |
| BJ | 1.0505857 | 0.36980 |
| HRGEC | 1.0472361 | 0.37378 |
| GM12865 | 1.0462257 | 0.38744 |
| SKNSHRA | 1.0467691 | 0.39554 |
| HMVECdLyNeo | 1.0405478 | 0.39850 |
| HCF | 1.0347547 | 0.40428 |
| HMVECdBlAd | 1.0354638 | 0.40696 |
| SKMC | 1.0290177 | 0.41608 |
| K562 | 1.0366805 | 0.41680 |
| Huh7.5 | 1.0332126 | 0.41960 |
| NHDFneo | 1.0274751 | 0.42494 |
| HMVECdBlNeo | 1.0280651 | 0.42534 |
| HMVECdAd | 1.0261302 | 0.43062 |
| HAc | 1.0209879 | 0.43254 |
| HPF | 1.0213670 | 0.43562 |
| HEEpiC | 1.0190884 | 0.44100 |
| WI38 | 1.0192749 | 0.44494 |
| AG09319 | 1.0157055 | 0.45518 |
| A549 | 1.0129164 | 0.45530 |
| BE2C | 1.0042957 | 0.47952 |
| PrEC | 1.0037263 | 0.47982 |
| HGF | 1.0045115 | 0.48348 |
| NHDFAd | 1.0034600 | 0.48818 |
| IshikawaTamoxifen | 0.9955798 | 0.49676 |
| HMVECLLy | 0.9928177 | 0.50576 |
| HPAEC | 0.9931526 | 0.50962 |
| SAEC | 0.9942638 | 0.51370 |
| HepG2 | 0.9808411 | 0.52762 |
| HMVECdLyAd | 0.9832559 | 0.52808 |
| HRPEpiC | 0.9858630 | 0.53732 |
| HMVECdNeo | 0.9700637 | 0.55824 |
| IshikawaEstradiol | 0.9659474 | 0.56792 |
| HCT116 | 0.9592514 | 0.57286 |
| PANC1 | 0.9594401 | 0.57766 |
| WERIRb1 | 0.9590392 | 0.59848 |
| Huh7 | 0.9313908 | 0.61784 |
| HConF | 0.9414333 | 0.63940 |
| SKNMC | 0.8977037 | 0.73034 |

# Bone mineral density 1

| DHS sample | fold enrichment | p value |
|---|---|---|
| HAsp | 1.2224269 | 0.05708 |
| RPTEC | 1.2046516 | 0.06288 |
| HVMF | 1.2429549 | 0.07624 |
| HMVECdBlAd | 1.2393570 | 0.07950 |
| AoSMC | 1.1947971 | 0.08938 |
| HBMEC | 1.1829675 | 0.08950 |
| HMVECLBl | 1.2196968 | 0.09280 |
| HPdLF | 1.1905644 | 0.11244 |
| HMVECdBlNeo | 1.2024234 | 0.11368 |
| HCFaa | 1.1764910 | 0.11378 |
| AoAF | 1.1889801 | 0.11450 |
| HMVECdLyNeo | 1.2015286 | 0.12254 |
| HCPEpiC | 1.1634824 | 0.12340 |
| HRCEpiC | 1.1566260 | 0.13218 |
| HSMM | 1.1535524 | 0.13284 |
| HMVECdLyAd | 1.1926526 | 0.13822 |
| HMVECLLy | 1.1863642 | 0.14150 |
| HMVECdAd | 1.1935863 | 0.14192 |
| HUVEC | 1.1831943 | 0.14520 |
| HRGEC | 1.1712090 | 0.14616 |
| HAEpiC | 1.1631074 | 0.15256 |
| H7hESC | 1.1060224 | 0.15554 |
| IshikawaTamoxifen | 1.1738147 | 0.15826 |
| Huh7 | 1.1985546 | 0.15882 |
| HAc | 1.1331126 | 0.16080 |
| HIPEpiC | 1.1389233 | 0.16534 |
| IshikawaEstradiol | 1.1605174 | 0.16890 |
| HNPCEpiC | 1.1236292 | 0.17536 |
| NHA | 1.1304855 | 0.17828 |
| NHLF | 1.1256125 | 0.18200 |
| HeLaS3IFNa4h | 1.1807859 | 0.19128 |
| HPAEC | 1.1497439 | 0.19322 |
| HMVECdNeo | 1.1449189 | 0.20092 |
| HSMMtube | 1.1216351 | 0.20292 |
| HRE | 1.1111649 | 0.20348 |
| PrEC | 1.1065376 | 0.21108 |
| HMF | 1.1187482 | 0.21280 |
| SAEC | 1.1044397 | 0.21490 |
| HAh | 1.0974908 | 0.22258 |
| HEEpiC | 1.1011682 | 0.22278 |
| HPAF | 1.1109475 | 0.22822 |
| AG04449 | 1.1057302 | 0.23802 |
| HCM | 1.1021502 | 0.24104 |
| Huh7.5 | 1.1375531 | 0.24376 |
| Melano | 1.0866999 | 0.24626 |
| FibroP | 1.0916389 | 0.24834 |
| UrotheliaUT189 | 1.1162488 | 0.24980 |
| AG09309 | 1.0929894 | 0.25756 |
| HGF | 1.0974721 | 0.26782 |
| T47D | 1.1211083 | 0.26880 |
| HPF | 1.0931895 | 0.26916 |
| PanIsletD | 1.0917988 | 0.26994 |
| HTR8svn | 1.1064073 | 0.27558 |
| AG09319 | 1.0907432 | 0.27962 |
| HeLaS3 | 1.1016583 | 0.28596 |
| HCT116 | 1.1022097 | 0.28690 |
| SKMC | 1.0766656 | 0.28702 |
| Myometr | 1.0802467 | 0.29848 |
| HRPEpiC | 1.0657791 | 0.29900 |
| HCF | 1.0789264 | 0.30530 |
| NHDFAd | 1.0702032 | 0.30558 |
| HepG2 | 1.0922003 | 0.30906 |
| Urothelia | 1.0780566 | 0.32344 |
| A549 | 1.0818477 | 0.32444 |
| WI38 | 1.0630610 | 0.34408 |
| RWPE1 | 1.0624996 | 0.34494 |
| AG04450 | 1.0559232 | 0.35758 |
| MCF7Hypoxia | 1.0645865 | 0.36156 |
| HFFMyc | 1.0501626 | 0.36502 |
| BJ | 1.0488806 | 0.36724 |
| Stellate | 1.0576808 | 0.37002 |
| MCF7 | 1.0489276 | 0.37532 |
| AG10803 | 1.0456467 | 0.37622 |
| HSMMemb | 1.0489108 | 0.38152 |
| K562 | 1.0481793 | 0.39706 |
| WERIRb1 | 1.0333740 | 0.39910 |
| PANC1 | 1.0367839 | 0.40126 |
| GM19238 | 1.0395590 | 0.41020 |
| ProgFib | 1.0341869 | 0.41150 |
| HPDE6E6E7 | 1.0370299 | 0.41210 |
| HFF | 1.0322160 | 0.41216 |
| H9ES | 1.0279813 | 0.41808 |
| Gliobla | 1.0317555 | 0.42106 |
| Fibrobl | 1.0306519 | 0.42128 |
| pHTE | 1.0292676 | 0.42304 |
| Th2 | 1.0294820 | 0.43190 |
| HMEC | 1.0218772 | 0.43604 |
| GM12878 | 1.0174431 | 0.44976 |
| PanIslets | 1.0181401 | 0.45030 |
| Medullo | 1.0127008 | 0.46344 |
| GM18507 | 1.0109705 | 0.46488 |
| Hepatocytes | 1.0007398 | 0.47926 |
| Caco2 | 0.9930844 | 0.48150 |
| HConF | 1.0026103 | 0.48392 |
| Th1 | 1.0030779 | 0.48926 |
| H1hESC | 0.9961903 | 0.49082 |
| NHDFneo | 1.0008831 | 0.49094 |
| HL60 | 0.9937114 | 0.49378 |
| GM19240 | 0.9911333 | 0.50944 |
| CD34Mobilized | 0.9854188 | 0.51686 |
| SKNSHRA | 0.9764041 | 0.52018 |
| Th0 | 0.9851578 | 0.52176 |
| LNCaP | 0.9853119 | 0.52358 |
| GM19239 | 0.9769759 | 0.52544 |
| NB4 | 0.9777789 | 0.52996 |
| Osteobl | 0.9823586 | 0.53336 |
| NT2D1 | 0.9821903 | 0.53902 |
| iPS | 0.9671214 | 0.54492 |
| LNCaPAndrogen | 0.9645858 | 0.55902 |
| GM12864 | 0.9568686 | 0.57706 |
| GM12865 | 0.9575307 | 0.57756 |
| 8988T | 0.9427194 | 0.58636 |
| GM12892 | 0.9438275 | 0.58886 |
| Chorion | 0.9381558 | 0.59486 |
| NHEK | 0.9501430 | 0.61176 |
| SKNMC | 0.9556481 | 0.61192 |
| GM06990 | 0.9217848 | 0.62278 |
| GM12891 | 0.9149200 | 0.63512 |
| CMK | 0.9129766 | 0.63620 |
| Jurkat | 0.9189976 | 0.68314 |
| BE2C | 0.9045685 | 0.69278 |
| MonocytesCD14RO01746 | 0.8854825 | 0.69330 |
| CD20 | 0.8614710 | 0.71314 |
| CLL | 0.8457170 | 0.74930 |

# Five major psychiatric disorders

| DHS sample | fold enrichment | p value |
|---|---|---|
| HIPEpiC | 1.2030101 | 0.13974 |
| AG09309 | 1.1467376 | 0.22136 |
| HAsp | 1.1274295 | 0.23082 |
| NHA | 1.1278032 | 0.23844 |
| HPdLF | 1.1384056 | 0.24756 |
| NHLF | 1.1170548 | 0.25452 |
| HCFaa | 1.1070085 | 0.28334 |
| AoAF | 1.1119624 | 0.29234 |
| AG10803 | 1.0964394 | 0.31682 |
| BJ | 1.0751067 | 0.34644 |
| HGF | 1.0745414 | 0.35740 |
| HNPCEpiC | 1.0593930 | 0.35744 |
| AG04449 | 1.0641152 | 0.36724 |
| AG09319 | 1.0562790 | 0.38872 |
| AG04450 | 1.0492471 | 0.40158 |
| AoSMC | 1.0332878 | 0.43150 |
| HMF | 1.0209511 | 0.44988 |
| NHDFAd | 1.0229867 | 0.45050 |
| HFF | 1.0150032 | 0.46302 |
| SKMC | 1.0085949 | 0.47444 |
| HPAF | 1.0017877 | 0.48966 |
| WI38 | 1.0002352 | 0.49418 |
| HAEpiC | 0.9950622 | 0.50430 |
| HFFMyc | 0.9848284 | 0.52842 |
| NHDFneo | 0.9816564 | 0.52986 |
| HCM | 0.9810786 | 0.53202 |
| HBMEC | 0.9723894 | 0.55794 |
| HCPEpiC | 0.9657726 | 0.56832 |
| HAh | 0.9651353 | 0.57948 |
| HRE | 0.9527280 | 0.59778 |
| HPF | 0.9435474 | 0.60016 |
| HCF | 0.9343830 | 0.61182 |
| HAc | 0.9430895 | 0.62598 |
| RPTEC | 0.9267052 | 0.65844 |
| HConF | 0.9080330 | 0.66292 |
| HRCEpiC | 0.9165162 | 0.66418 |
| Stellate | 0.8798486 | 0.66548 |
| HVMF | 0.8810094 | 0.70772 |
| HRGEC | 0.8800720 | 0.71326 |
| HRPEpiC | 0.8977750 | 0.73848 |
| HMVECLBl | 0.8559615 | 0.74402 |
| Medullo | 0.8220627 | 0.75162 |
| ProgFib | 0.8143310 | 0.77412 |
| MonocytesCD14RO01746 | 0.7719534 | 0.77750 |
| FibroP | 0.8516946 | 0.78220 |
| Myometr | 0.8080582 | 0.80414 |
| HSMM | 0.8283489 | 0.81482 |
| PanIsletD | 0.8071426 | 0.81850 |
| PANC1 | 0.7861817 | 0.82168 |
| HMVECLLy | 0.7804599 | 0.82620 |
| SKNMC | 0.8227528 | 0.82782 |
| HMVECdAd | 0.7668693 | 0.83248 |
| BE2C | 0.7810627 | 0.83284 |
| SAEC | 0.8169783 | 0.84956 |
| HMVECdLyAd | 0.7549731 | 0.84968 |
| HMVECdBlNeo | 0.7686277 | 0.85130 |
| SKNSHRA | 0.6990922 | 0.85188 |
| HMVECdLyNeo | 0.7620302 | 0.85198 |
| CD20 | 0.6792015 | 0.85320 |
| CD34Mobilized | 0.7222346 | 0.86370 |
| GM12865 | 0.7339295 | 0.86464 |
| HeLaS3 | 0.7309811 | 0.86540 |
| GM12864 | 0.7074286 | 0.87542 |
| Gliobla | 0.6900564 | 0.87572 |
| GM12878 | 0.6938930 | 0.87654 |
| HPAEC | 0.7353998 | 0.88270 |
| HMVECdBlAd | 0.7365932 | 0.88660 |
| H7hESC | 0.8341756 | 0.89200 |
| HMVECdNeo | 0.7088163 | 0.89496 |
| HCT116 | 0.6906559 | 0.89796 |
| HeLaS3IFNa4h | 0.6563835 | 0.90142 |
| HTR8svn | 0.6934490 | 0.90286 |
| HEEpiC | 0.7703808 | 0.90704 |
| HSMMemb | 0.6923081 | 0.90730 |
| UrotheliaUT189 | 0.6913768 | 0.90784 |
| Fibrobl | 0.6976695 | 0.90796 |
| WERIRb1 | 0.7425111 | 0.90824 |
| GM19240 | 0.6711448 | 0.91108 |
| GM18507 | 0.6480523 | 0.91134 |
| GM06990 | 0.6310272 | 0.91218 |
| Osteobl | 0.6816212 | 0.91308 |
| GM19238 | 0.6334768 | 0.91374 |
| HL60 | 0.6189262 | 0.91456 |
| HSMMtube | 0.7290407 | 0.91556 |
| CLL | 0.6094432 | 0.91820 |
| Melano | 0.7563959 | 0.92212 |
| PrEC | 0.7572028 | 0.92284 |
| Th0 | 0.6373719 | 0.92296 |
| Th2 | 0.5974236 | 0.92580 |
| NB4 | 0.6389040 | 0.92616 |
| HPDE6E6E7 | 0.6601717 | 0.92726 |
| HUVEC | 0.6703210 | 0.92826 |
| PanIslets | 0.6471041 | 0.92842 |
| pHTE | 0.6642392 | 0.93080 |
| LNCaPAndrogen | 0.6151718 | 0.93270 |
| RWPE1 | 0.6737664 | 0.93378 |
| Urothelia | 0.6519621 | 0.93432 |
| HepG2 | 0.6129676 | 0.93656 |
| NHEK | 0.6677287 | 0.93892 |
| GM19239 | 0.5808305 | 0.94176 |
| Caco2 | 0.4844080 | 0.94576 |
| GM12892 | 0.5456504 | 0.94792 |
| Hepatocytes | 0.5526307 | 0.95152 |
| 8988T | 0.5274478 | 0.95258 |
| Th1 | 0.5888696 | 0.95292 |
| Jurkat | 0.6396700 | 0.95326 |
| H9ES | 0.6185644 | 0.95436 |
| LNCaP | 0.6534618 | 0.95456 |
| HMEC | 0.6464675 | 0.95584 |
| CMK | 0.5410361 | 0.95914 |
| T47D | 0.5591860 | 0.96132 |
| GM12891 | 0.5114203 | 0.96188 |
| MCF7 | 0.6102857 | 0.96380 |
| NT2D1 | 0.6942042 | 0.96474 |
| A549 | 0.5724997 | 0.96604 |
| iPS | 0.4916291 | 0.96648 |
| MCF7Hypoxia | 0.5222006 | 0.96838 |
| Huh7 | 0.5302689 | 0.96870 |
| Chorion | 0.4875692 | 0.97044 |
| Huh7.5 | 0.5214405 | 0.97094 |
| K562 | 0.5111691 | 0.97096 |
| H1hESC | 0.5418200 | 0.97578 |
| IshikawaEstradiol | 0.5530141 | 0.98080 |
| IshikawaTamoxifen | 0.5014382 | 0.98820 |

## Bone mineral density 4

| DHS sample | fold enrichment | p value |
|---|---|---|
| HAsp | 1.2285904 | 0.08114 |
| SKMC | 1.2187959 | 0.08482 |
| HBMEC | 1.1932207 | 0.10608 |
| HFFMyc | 1.1605172 | 0.15034 |
| PrEC | 1.1448245 | 0.15106 |
| HCPEpiC | 1.1594628 | 0.15504 |
| HEEpiC | 1.1421774 | 0.15518 |
| HCFaa | 1.1641121 | 0.16060 |
| NHDFAd | 1.1499973 | 0.16426 |
| HNPCEpiC | 1.1408944 | 0.16450 |
| SAEC | 1.1359371 | 0.16524 |
| BJ | 1.1642269 | 0.16990 |
| HIPEpiC | 1.1453595 | 0.17610 |
| HPdLF | 1.1582870 | 0.18252 |
| NHDFneo | 1.1406025 | 0.20018 |
| AG09319 | 1.1416484 | 0.20512 |
| HeLaS3 | 1.1482349 | 0.20992 |
| AG04449 | 1.1316201 | 0.21214 |
| HGF | 1.1307321 | 0.22242 |
| AG04450 | 1.1194532 | 0.24270 |
| AoSMC | 1.0993503 | 0.24992 |
| HMVECLBl | 1.1076198 | 0.26264 |
| HPF | 1.1014026 | 0.26750 |
| HeLaS3IFNa4h | 1.1216353 | 0.26816 |
| Huh7 | 1.1192376 | 0.27864 |
| AG09309 | 1.0884398 | 0.28054 |
| NHA | 1.0831798 | 0.29278 |
| NHLF | 1.0834746 | 0.29280 |
| HVMF | 1.0934394 | 0.29818 |
| WI38 | 1.0917185 | 0.29866 |
| HAc | 1.0725697 | 0.30518 |
| HCM | 1.0724219 | 0.31628 |
| HAEpiC | 1.0762490 | 0.32344 |
| HPDE6E6E7 | 1.0782675 | 0.32662 |
| HMF | 1.0678357 | 0.33224 |
| HPAF | 1.0595452 | 0.34706 |
| AG10803 | 1.0637187 | 0.34840 |
| HSMM | 1.0536476 | 0.35070 |
| SKNMC | 1.0604905 | 0.35076 |
| AoAF | 1.0595042 | 0.35390 |
| HRE | 1.0513719 | 0.35770 |
| HFF | 1.0526774 | 0.36346 |
| HCF | 1.0528449 | 0.36820 |
| HMVECdBlAd | 1.0493442 | 0.37992 |
| HConF | 1.0446576 | 0.38520 |
| HRGEC | 1.0463463 | 0.38670 |
| RWPE1 | 1.0414132 | 0.39312 |
| RPTEC | 1.0344822 | 0.40044 |
| PANC1 | 1.0360096 | 0.40404 |
| HUVEC | 1.0331603 | 0.41158 |
| GM12864 | 1.0376578 | 0.41364 |
| GM12865 | 1.0320129 | 0.41870 |
| HTR8svn | 1.0296131 | 0.42834 |
| HCT116 | 1.0221154 | 0.43966 |
| HRPEpiC | 1.0161247 | 0.44858 |
| HMVECdBlNeo | 1.0175779 | 0.45444 |
| IshikawaTamoxifen | 1.0051922 | 0.47496 |
| HMVECdLyNeo | 1.0070776 | 0.47846 |
| A549 | 1.0010991 | 0.48000 |
| Huh7.5 | 0.9931140 | 0.49318 |
| HRCEpiC | 1.0012611 | 0.49358 |
| Myometr | 0.9899786 | 0.51490 |
| K562 | 0.9799789 | 0.52066 |
| Caco2 | 0.9524922 | 0.52468 |
| Melano | 0.9890846 | 0.52998 |
| HAh | 0.9864475 | 0.53444 |
| HMVECdLyAd | 0.9764572 | 0.54134 |
| IshikawaEstradiol | 0.9736895 | 0.54644 |
| GM06990 | 0.9505730 | 0.56350 |
| GM19238 | 0.9525293 | 0.56462 |
| HMVECLLy | 0.9667132 | 0.56574 |
| HPAEC | 0.9569338 | 0.59282 |
| GM12878 | 0.9400004 | 0.59374 |
| LNCaP | 0.9464686 | 0.59450 |
| HSMMtube | 0.9605428 | 0.59500 |
| PanIsletD | 0.9569367 | 0.59954 |
| HSMMemb | 0.9494294 | 0.60290 |
| ProgFib | 0.9382149 | 0.61546 |
| NHEK | 0.9438758 | 0.62542 |
| GM18507 | 0.9233187 | 0.63734 |
| pHTE | 0.9296933 | 0.63786 |
| FibroP | 0.9453001 | 0.65122 |
| H7hESC | 0.9538660 | 0.65358 |
| CMK | 0.8838515 | 0.67138 |
| HMVECdNeo | 0.9185439 | 0.67290 |
| CD20 | 0.8762692 | 0.67570 |
| HMVECdAd | 0.9131621 | 0.67814 |
| T47D | 0.8825811 | 0.68210 |
| GM12891 | 0.8756872 | 0.68250 |
| Chorion | 0.8696388 | 0.68312 |
| NB4 | 0.8893545 | 0.69196 |
| Th2 | 0.8727156 | 0.69480 |
| Stellate | 0.8871861 | 0.70656 |
| SKNSHRA | 0.8532355 | 0.71688 |
| HMEC | 0.8945747 | 0.72472 |
| HL60 | 0.8611158 | 0.72580 |
| GM19239 | 0.8451097 | 0.74032 |
| H1hESC | 0.8538203 | 0.74060 |
| HepG2 | 0.8616412 | 0.74298 |
| MCF7Hypoxia | 0.8503307 | 0.74352 |
| CLL | 0.8388537 | 0.74668 |
| GM12892 | 0.8317795 | 0.74778 |
| Hepatocytes | 0.8348530 | 0.75002 |
| BE2C | 0.8669230 | 0.75400 |
| CD34Mobilized | 0.8652227 | 0.75518 |
| MonocytesCD14RO01746 | 0.8279924 | 0.76604 |
| Th1 | 0.8488303 | 0.76720 |
| GM19240 | 0.8429998 | 0.78542 |
| Fibrobl | 0.8464865 | 0.79240 |
| LNCaPAndrogen | 0.8261622 | 0.79394 |
| 8988T | 0.7853180 | 0.79782 |
| iPS | 0.7841963 | 0.79882 |
| MCF7 | 0.8423688 | 0.80004 |
| Th0 | 0.8210296 | 0.80558 |
| Jurkat | 0.8359817 | 0.81716 |
| Urothelia | 0.8332936 | 0.81760 |
| PanIslets | 0.8201730 | 0.81948 |
| UrotheliaUT189 | 0.8123557 | 0.83594 |
| Osteobl | 0.8093661 | 0.83722 |
| Medullo | 0.7985323 | 0.83896 |
| H9ES | 0.8071280 | 0.85456 |
| WERIRb1 | 0.7971563 | 0.88928 |
| Gliobla | 0.7234411 | 0.91982 |
| NT2D1 | 0.7842518 | 0.94684 |

# Bone mineral density 3

| DHS sample | fold enrichment | p value |
|---|---|---|
| HMVECLBl | 1.2459201 | 0.09412 |
| HSMM | 1.1844743 | 0.11086 |
| Myometr | 1.2165981 | 0.11306 |
| HBMEC | 1.1824926 | 0.12042 |
| HUVEC | 1.2177885 | 0.12464 |
| H7hESC | 1.1285570 | 0.13006 |
| Melano | 1.1550752 | 0.14342 |
| Hepatocytes | 1.2609312 | 0.14978 |
| Chorion | 1.2770530 | 0.15090 |
| Caco2 | 1.3482038 | 0.15130 |
| HMVECdBlAd | 1.1888624 | 0.15168 |
| H1hESC | 1.2179651 | 0.16000 |
| pHTE | 1.1945308 | 0.16204 |
| MCF7 | 1.1763332 | 0.16422 |
| HSMMtube | 1.1555892 | 0.17058 |
| PanIslets | 1.2017164 | 0.17334 |
| HAsp | 1.1506137 | 0.17446 |
| Huh7.5 | 1.2149532 | 0.17580 |
| iPS | 1.2502599 | 0.17606 |
| WI38 | 1.1754005 | 0.17736 |
| Osteobl | 1.1889487 | 0.18276 |
| ProgFib | 1.1757520 | 0.18664 |
| FibroP | 1.1369713 | 0.18776 |
| PanIsletD | 1.1496256 | 0.19060 |
| HMVECdLyNeo | 1.1631396 | 0.19274 |
| HMEC | 1.1560809 | 0.19736 |
| HCPEpiC | 1.1326656 | 0.20214 |
| Fibrobl | 1.1608989 | 0.21118 |
| HIPEpiC | 1.1258092 | 0.21476 |
| LNCaP | 1.1413719 | 0.21502 |
| MCF7Hypoxia | 1.1651421 | 0.22356 |
| HVMF | 1.1421795 | 0.22432 |
| HMVECdBlNeo | 1.1372985 | 0.22892 |
| LNCaPAndrogen | 1.1398563 | 0.25268 |
| HCFaa | 1.1090655 | 0.25330 |
| Huh7 | 1.1397256 | 0.25460 |
| HAc | 1.0974010 | 0.25638 |
| 8988T | 1.1667065 | 0.25826 |
| Th1 | 1.1402499 | 0.25960 |
| H9ES | 1.1174243 | 0.26438 |
| HMVECLLy | 1.1158407 | 0.26544 |
| HAEpiC | 1.1068744 | 0.26590 |
| HEEpiC | 1.0888222 | 0.26712 |
| HMVECdAd | 1.1209524 | 0.26852 |
| HRGEC | 1.1091500 | 0.26956 |
| HMVECdLyAd | 1.1148996 | 0.27294 |
| NHA | 1.0955297 | 0.27716 |
| RPTEC | 1.0884299 | 0.27776 |
| HNPCEpiC | 1.0878812 | 0.27826 |
| HeLaS3IFNa4h | 1.1211987 | 0.28098 |
| GM19240 | 1.1184169 | 0.28446 |
| T47D | 1.1194695 | 0.28634 |
| GM12891 | 1.1365459 | 0.29100 |
| AoSMC | 1.0874532 | 0.29330 |
| HCT116 | 1.0945149 | 0.29972 |
| SKMC | 1.0808295 | 0.30048 |
| HSMMemb | 1.0975782 | 0.30204 |
| HPdLF | 1.0904986 | 0.30362 |
| GM19238 | 1.1109486 | 0.30786 |
| HRCEpiC | 1.0754727 | 0.30964 |
| Th0 | 1.1089823 | 0.31046 |
| HPF | 1.0815607 | 0.31564 |
| NHLF | 1.0728621 | 0.31844 |
| HGF | 1.0804202 | 0.32036 |
| AoAF | 1.0778265 | 0.32186 |
| UrotheliaUT189 | 1.0895768 | 0.32388 |
| AG04450 | 1.0810590 | 0.32432 |
| HRE | 1.0653478 | 0.32484 |
| HMVECdNeo | 1.0807631 | 0.32870 |
| AG04449 | 1.0746116 | 0.32906 |
| GM12892 | 1.1061377 | 0.33226 |
| HTR8svn | 1.0771792 | 0.33838 |
| GM19239 | 1.0894481 | 0.34194 |
| IshikawaTamoxifen | 1.0684716 | 0.34970 |
| PrEC | 1.0528755 | 0.35382 |
| HFFMyc | 1.0596763 | 0.35416 |
| SKNSHRA | 1.0584530 | 0.37758 |
| SAEC | 1.0403474 | 0.38430 |
| HPDE6E6E7 | 1.0500556 | 0.38462 |
| HeLaS3 | 1.0503385 | 0.38940 |
| Urothelia | 1.0493541 | 0.39200 |
| HPAEC | 1.0472848 | 0.39288 |
| HMF | 1.0396323 | 0.40068 |
| GM12878 | 1.0461762 | 0.40118 |
| Stellate | 1.0436261 | 0.40594 |
| NHDFAd | 1.0353660 | 0.40908 |
| AG09319 | 1.0372460 | 0.41298 |
| HPAF | 1.0276564 | 0.42710 |
| HAh | 1.0236063 | 0.42888 |
| HepG2 | 1.0226577 | 0.44374 |
| GM18507 | 1.0152522 | 0.45906 |
| RWPE1 | 1.0106440 | 0.46352 |
| NHDFneo | 1.0105899 | 0.46680 |
| IshikawaEstradiol | 1.0053125 | 0.47850 |
| GM12864 | 1.0008471 | 0.48250 |
| AG09309 | 1.0032916 | 0.48892 |
| HCM | 1.0003519 | 0.49414 |
| AG10803 | 0.9867728 | 0.51810 |
| Medullo | 0.9791542 | 0.52864 |
| NT2D1 | 0.9813369 | 0.53664 |
| BJ | 0.9759540 | 0.54478 |
| HFF | 0.9717383 | 0.55690 |
| GM06990 | 0.9398803 | 0.58070 |
| Th2 | 0.9364198 | 0.59030 |
| HConF | 0.9558341 | 0.59444 |
| NHEK | 0.9524052 | 0.59452 |
| K562 | 0.9274897 | 0.60820 |
| CD20 | 0.9137566 | 0.61246 |
| HCF | 0.9439492 | 0.61456 |
| GM12865 | 0.9229693 | 0.63636 |
| Gliobla | 0.9057191 | 0.65006 |
| CLL | 0.8897278 | 0.65080 |
| HRPEpiC | 0.9363589 | 0.67062 |
| PANC1 | 0.9070016 | 0.67586 |
| WERIRb1 | 0.9181238 | 0.68634 |
| CD34Mobilized | 0.8924210 | 0.69248 |
| Jurkat | 0.8511315 | 0.78358 |
| CMK | 0.7974845 | 0.79148 |
| SKNMC | 0.8645443 | 0.79238 |
| NB4 | 0.8053546 | 0.82126 |
| A549 | 0.7987331 | 0.83264 |
| HL60 | 0.7569121 | 0.83982 |
| MonocytesCD14RO01746 | 0.7415734 | 0.85288 |
| BE2C | 0.7839244 | 0.87342 |

## Refractive error and myopia

| DHS sample | fold enrichment | p value |
|---|---|---|
| SKNMC | 1.0254408 | 0.41634 |
| Jurkat | 0.9910200 | 0.50486 |
| T47D | 0.9574667 | 0.57586 |
| Th2 | 0.9463958 | 0.58806 |
| CMK | 0.9473623 | 0.58906 |
| HRGEC | 0.9573650 | 0.60448 |
| H9ES | 0.9504666 | 0.61716 |
| SKNSHRA | 0.9316137 | 0.62214 |
| PANC1 | 0.9330387 | 0.64846 |
| GM19239 | 0.9088567 | 0.67040 |
| HUVEC | 0.9247424 | 0.68152 |
| Huh7.5 | 0.8953173 | 0.70720 |
| GM12864 | 0.8933110 | 0.71300 |
| BE2C | 0.9065222 | 0.71910 |
| HRPEpiC | 0.9326438 | 0.71944 |
| HPdLF | 0.9082628 | 0.72302 |
| HPAEC | 0.9037400 | 0.72526 |
| H1hESC | 0.8921497 | 0.73072 |
| HFFMyc | 0.9122924 | 0.73890 |
| Hepatocytes | 0.8692959 | 0.73986 |
| NT2D1 | 0.9215535 | 0.74608 |
| HGF | 0.8984610 | 0.74728 |
| A549 | 0.8864081 | 0.74950 |
| RPTEC | 0.9174733 | 0.75378 |
| Huh7 | 0.8734013 | 0.75498 |
| HMVECdLyNeo | 0.8860991 | 0.75700 |
| HCFaa | 0.8982949 | 0.76170 |
| CD20 | 0.8386666 | 0.76384 |
| HRCEpiC | 0.9072962 | 0.76428 |
| HCPEpiC | 0.9008185 | 0.76662 |
| HMVECdBlNeo | 0.8825146 | 0.76684 |
| HPDE6E6E7 | 0.8801612 | 0.77180 |
| CD34Mobilized | 0.8754396 | 0.77610 |
| K562 | 0.8509860 | 0.78100 |
| GM06990 | 0.8353092 | 0.78486 |
| WERIRb1 | 0.8966504 | 0.78638 |
| GM12891 | 0.8262748 | 0.79158 |
| HAsp | 0.8926756 | 0.79452 |
| HeLaS3 | 0.8634410 | 0.79770 |
| HMVECdBlAd | 0.8678741 | 0.79900 |
| HSMMemb | 0.8655186 | 0.80032 |
| IshikawaEstradiol | 0.8748594 | 0.80236 |
| GM12878 | 0.8406047 | 0.80312 |
| HNPCEpiC | 0.8941070 | 0.80476 |
| NHDFneo | 0.8686987 | 0.81344 |
| HIPEpiC | 0.8784948 | 0.81614 |
| GM19238 | 0.8290163 | 0.81742 |
| Gliobla | 0.8325443 | 0.82658 |
| HSMM | 0.8837359 | 0.82832 |
| HeLaS3IFNa4h | 0.8288524 | 0.83164 |
| GM18507 | 0.8307033 | 0.83208 |
| Medullo | 0.8261178 | 0.83492 |
| HTR8svn | 0.8401774 | 0.84334 |
| HMVECdLyAd | 0.8387346 | 0.84344 |
| GM12865 | 0.8332143 | 0.84490 |
| BJ | 0.8512626 | 0.84758 |
| HMVECdAd | 0.8293437 | 0.84892 |
| HAc | 0.8748875 | 0.85142 |
| AG09309 | 0.8570658 | 0.85196 |
| GM12892 | 0.7753405 | 0.85364 |
| GM19240 | 0.8233324 | 0.85378 |
| HMVECdNeo | 0.8344115 | 0.85426 |
| Myometr | 0.8519342 | 0.85608 |
| HSMMtube | 0.8628335 | 0.85880 |
| HMVECLLy | 0.8270225 | 0.86138 |
| NB4 | 0.8084581 | 0.86142 |
| UrotheliaUT189 | 0.8232251 | 0.86264 |
| ProgFib | 0.8271128 | 0.86690 |
| LNCaPAndrogen | 0.7988234 | 0.86734 |
| PanIslets | 0.8094156 | 0.86836 |
| SAEC | 0.8632588 | 0.87070 |
| HFF | 0.8432736 | 0.87256 |
| iPS | 0.7548797 | 0.87394 |
| HAh | 0.8676418 | 0.87584 |
| AoAF | 0.8254315 | 0.87810 |
| HPAF | 0.8362168 | 0.87870 |
| Chorion | 0.7564325 | 0.88188 |
| HRE | 0.8546140 | 0.88448 |
| H7hESC | 0.8972374 | 0.88582 |
| 8988T | 0.7462797 | 0.88790 |
| AG09319 | 0.8167178 | 0.88806 |
| HCT116 | 0.8094333 | 0.88934 |
| Urothelia | 0.8090700 | 0.88998 |
| IshikawaTamoxifen | 0.8133023 | 0.89088 |
| LNCaP | 0.8224247 | 0.89288 |
| HConF | 0.8221556 | 0.89646 |
| HepG2 | 0.7743792 | 0.89932 |
| HCM | 0.8236451 | 0.89956 |
| RWPE1 | 0.8180409 | 0.90040 |
| HMVECLBl | 0.8009154 | 0.90278 |
| Th1 | 0.7666263 | 0.90322 |
| CLL | 0.7329828 | 0.91162 |
| HBMEC | 0.8290467 | 0.91204 |
| Stellate | 0.7705313 | 0.91378 |
| AG04449 | 0.8060149 | 0.91410 |
| AoSMC | 0.8151531 | 0.91424 |
| HEEpiC | 0.8352188 | 0.91484 |
| Caco2 | 0.6410217 | 0.91500 |
| SKMC | 0.8171491 | 0.91532 |
| NHLF | 0.8229443 | 0.91658 |
| HPF | 0.7997689 | 0.91662 |
| PanIsletD | 0.8127818 | 0.91796 |
| WI38 | 0.7787354 | 0.92274 |
| HCF | 0.7865150 | 0.92830 |
| AG04450 | 0.7771561 | 0.92918 |
| pHTE | 0.7706593 | 0.93004 |
| HVMF | 0.7698558 | 0.93154 |
| NHA | 0.8061265 | 0.93214 |
| MonocytesCD14RO01746 | 0.7025312 | 0.93246 |
| AG10803 | 0.7804742 | 0.93538 |
| HMEC | 0.7814825 | 0.93852 |
| Osteobl | 0.7365052 | 0.94106 |
| NHDFAd | 0.7894889 | 0.94226 |
| PrEC | 0.8092068 | 0.94542 |
| Melano | 0.8209230 | 0.94658 |
| NHEK | 0.7721346 | 0.94704 |
| Th0 | 0.7113848 | 0.94980 |
| FibroP | 0.7941146 | 0.95344 |
| HMF | 0.7683494 | 0.95604 |
| Fibrobl | 0.7251252 | 0.95940 |
| HL60 | 0.6614278 | 0.96400 |
| MCF7Hypoxia | 0.6571233 | 0.97074 |
| HAEpiC | 0.6888181 | 0.98620 |
| MCF7 | 0.6405987 | 0.99496 |

## phospho− and sphingolipid concentrations

| DHS sample | fold enrichment | p value |
|---|---|---|
| WERIRb1 | 1.0421519 | 0.34034 |
| HepG2 | 1.0494648 | 0.35528 |
| HTR8svn | 1.0433432 | 0.36076 |
| Huh7.5 | 1.0185015 | 0.44026 |
| RPTEC | 1.0123895 | 0.44608 |
| Hepatocytes | 1.0118324 | 0.45958 |
| CMK | 1.0063356 | 0.47116 |
| HPAEC | 1.0020850 | 0.48926 |
| Huh7 | 0.9913452 | 0.51466 |
| PANC1 | 0.9913192 | 0.51666 |
| HPDE6E6E7 | 0.9874822 | 0.53204 |
| LNCaPAndrogen | 0.9845924 | 0.53276 |
| 8988T | 0.9812044 | 0.53306 |
| HL60 | 0.9787364 | 0.54640 |
| HMVECdBlNeo | 0.9845549 | 0.54782 |
| HeLaS3IFNa4h | 0.9767900 | 0.54966 |
| HRGEC | 0.9838939 | 0.55352 |
| Myometr | 0.9798829 | 0.56078 |
| Th2 | 0.9739853 | 0.56326 |
| A549 | 0.9713880 | 0.56856 |
| CD34Mobilized | 0.9705945 | 0.58290 |
| IshikawaTamoxifen | 0.9712489 | 0.58430 |
| HRCEpiC | 0.9776208 | 0.58776 |
| Medullo | 0.9691189 | 0.58938 |
| K562 | 0.9636556 | 0.59326 |
| NB4 | 0.9656913 | 0.59466 |
| UrotheliaUT189 | 0.9650879 | 0.60144 |
| HEEpiC | 0.9754456 | 0.60218 |
| Stellate | 0.9617278 | 0.60512 |
| HMVECdBlAd | 0.9670843 | 0.60706 |
| RWPE1 | 0.9637173 | 0.60984 |
| CLL | 0.9512734 | 0.61354 |
| HMVECdLyAd | 0.9603998 | 0.61770 |
| IshikawaEstradiol | 0.9601300 | 0.61976 |
| Urothelia | 0.9529670 | 0.63462 |
| HMVECLBl | 0.9584660 | 0.63844 |
| H9ES | 0.9494290 | 0.63992 |
| Gliobla | 0.9440803 | 0.64184 |
| GM19239 | 0.9390744 | 0.64270 |
| HAEpiC | 0.9579875 | 0.64382 |
| HeLaS3 | 0.9459443 | 0.64824 |
| HMVECdNeo | 0.9483201 | 0.65486 |
| SKNSHRA | 0.9291696 | 0.65968 |
| GM12878 | 0.9371659 | 0.66426 |
| Jurkat | 0.9480325 | 0.66450 |
| NHEK | 0.9463103 | 0.66986 |
| MonocytesCD14RO01746 | 0.9315326 | 0.67056 |
| HMVECdAd | 0.9410436 | 0.67160 |
| HGF | 0.9458464 | 0.67194 |
| Chorion | 0.9224493 | 0.67250 |
| HMVECLLy | 0.9428098 | 0.67444 |
| Caco2 | 0.9027575 | 0.67462 |
| GM06990 | 0.9253964 | 0.67752 |
| HPAF | 0.9508206 | 0.67776 |
| HMVECdLyNeo | 0.9424758 | 0.67954 |
| GM18507 | 0.9321288 | 0.68298 |
| GM12865 | 0.9361647 | 0.68496 |
| GM12864 | 0.9330586 | 0.68526 |
| GM12891 | 0.9182119 | 0.68588 |
| AoSMC | 0.9515353 | 0.68690 |
| Th0 | 0.9318020 | 0.68914 |
| HSMMemb | 0.9369921 | 0.68944 |
| HCM | 0.9481123 | 0.69166 |
| PanIslets | 0.9301665 | 0.69328 |
| T47D | 0.9209159 | 0.69714 |
| HCFaa | 0.9435847 | 0.70718 |
| HConF | 0.9374268 | 0.71054 |
| HRE | 0.9453410 | 0.71744 |
| AG04450 | 0.9321749 | 0.72006 |
| SAEC | 0.9434124 | 0.72692 |
| PrEC | 0.9415535 | 0.73204 |
| AoAF | 0.9293014 | 0.73536 |
| GM19238 | 0.9061645 | 0.73542 |
| HCT116 | 0.9157358 | 0.73652 |
| HCF | 0.9249644 | 0.74522 |
| MCF7Hypoxia | 0.9001883 | 0.74576 |
| HUVEC | 0.9148933 | 0.74790 |
| H1hESC | 0.9012879 | 0.74854 |
| CD20 | 0.8854685 | 0.75490 |
| GM19240 | 0.9078246 | 0.75536 |
| NHDFAd | 0.9259308 | 0.76014 |
| MCF7 | 0.9100485 | 0.76060 |
| iPS | 0.8817629 | 0.76092 |
| GM12892 | 0.8847938 | 0.76290 |
| HMEC | 0.9130361 | 0.76866 |
| Fibrobl | 0.9070746 | 0.77450 |
| ProgFib | 0.9005159 | 0.77944 |
| Th1 | 0.8973211 | 0.78114 |
| HPdLF | 0.9115829 | 0.78352 |
| pHTE | 0.9016778 | 0.78398 |
| PanIsletD | 0.9125593 | 0.78422 |
| AG09309 | 0.9175413 | 0.78908 |
| FibroP | 0.9202148 | 0.79128 |
| LNCaP | 0.8987620 | 0.79434 |
| HMF | 0.9113967 | 0.79534 |
| HIPEpiC | 0.9165011 | 0.79586 |
| Melano | 0.9200657 | 0.79910 |
| HFF | 0.9068928 | 0.80532 |
| HVMF | 0.8985894 | 0.80568 |
| HCPEpiC | 0.9122384 | 0.80700 |
| HPF | 0.8996985 | 0.81132 |
| NHDFneo | 0.8995927 | 0.81542 |
| HNPCEpiC | 0.9155325 | 0.81780 |
| HSMM | 0.9072130 | 0.82160 |
| Osteobl | 0.8819026 | 0.82336 |
| HAsp | 0.9062920 | 0.82774 |
| AG04449 | 0.8975318 | 0.83034 |
| WI38 | 0.8869323 | 0.83536 |
| HSMMtube | 0.8954289 | 0.83544 |
| BE2C | 0.8758293 | 0.83900 |
| SKMC | 0.8993807 | 0.84072 |
| AG09319 | 0.8835523 | 0.84352 |
| H7hESC | 0.9237321 | 0.84568 |
| HFFMyc | 0.8902297 | 0.85096 |
| AG10803 | 0.8796655 | 0.85690 |
| HAh | 0.9018533 | 0.86138 |
| BJ | 0.8741652 | 0.87444 |
| HBMEC | 0.8899568 | 0.87506 |
| NHLF | 0.8861644 | 0.87830 |
| HRPEpiC | 0.8937313 | 0.88358 |
| NT2D1 | 0.8788437 | 0.89312 |
| NHA | 0.8714805 | 0.90236 |
| HAc | 0.8694303 | 0.91412 |
| SKNMC | 0.8378884 | 0.93050 |

## C−reactive protein levels

| DHS sample | fold enrichment | p value |
|---|---|---|
| RPTEC | 1.1281460 | 0.11386 |
| HRCEpiC | 1.0967442 | 0.18998 |
| PANC1 | 1.0602640 | 0.32302 |
| Jurkat | 1.0489685 | 0.35140 |
| HPAEC | 1.0474897 | 0.35350 |
| Th2 | 1.0483491 | 0.37696 |
| HRGEC | 1.0332708 | 0.38860 |
| H7hESC | 1.0243966 | 0.39206 |
| HAsp | 1.0266343 | 0.40812 |
| HepG2 | 1.0299379 | 0.41414 |
| SAEC | 1.0167594 | 0.43308 |
| UrotheliaUT189 | 1.0188530 | 0.44178 |
| GM12865 | 1.0140331 | 0.45270 |
| Huh7 | 1.0135614 | 0.45700 |
| HCFaa | 1.0108007 | 0.46104 |
| NT2D1 | 1.0090580 | 0.46226 |
| HMVECdBlAd | 1.0075657 | 0.47256 |
| HPDE6E6E7 | 1.0031625 | 0.48534 |
| Urothelia | 1.0024967 | 0.48898 |
| HEEpiC | 1.0017400 | 0.49082 |
| GM12864 | 1.0000159 | 0.49286 |
| A549 | 0.9961414 | 0.49722 |
| HMVECdBlNeo | 1.0005312 | 0.49738 |
| GM12878 | 0.9923502 | 0.51106 |
| SKMC | 0.9918908 | 0.52234 |
| HMVECLBl | 0.9918626 | 0.52408 |
| IshikawaEstradiol | 0.9894731 | 0.52476 |
| HAEpiC | 0.9907457 | 0.52858 |
| MonocytesCD14RO01746 | 0.9816415 | 0.53740 |
| AG04449 | 0.9869586 | 0.53978 |
| HAh | 0.9853685 | 0.55062 |
| HRE | 0.9851358 | 0.55158 |
| RWPE1 | 0.9795612 | 0.55456 |
| HIPEpiC | 0.9846216 | 0.55698 |
| Myometr | 0.9779279 | 0.56202 |
| HMVECLLy | 0.9748578 | 0.56898 |
| HMVECdLyAd | 0.9744663 | 0.57046 |
| Th0 | 0.9758226 | 0.57152 |
| WI38 | 0.9769194 | 0.57324 |
| HSMMemb | 0.9741506 | 0.57516 |
| Huh7.5 | 0.9649042 | 0.58188 |
| NHEK | 0.9697493 | 0.58366 |
| HPdLF | 0.9719502 | 0.58606 |
| HMVECdNeo | 0.9653144 | 0.59694 |
| GM06990 | 0.9517874 | 0.60756 |
| HMVECdAd | 0.9587653 | 0.61228 |
| NHLF | 0.9660992 | 0.61256 |
| AG10803 | 0.9622473 | 0.61528 |
| HTR8svn | 0.9595576 | 0.61584 |
| HNPCEpiC | 0.9679422 | 0.61694 |
| HVMF | 0.9600892 | 0.61990 |
| NB4 | 0.9540777 | 0.62078 |
| K562 | 0.9504503 | 0.62320 |
| HCT116 | 0.9508658 | 0.62388 |
| HMVECdLyNeo | 0.9570618 | 0.62466 |
| AG09309 | 0.9608347 | 0.63702 |
| PrEC | 0.9621560 | 0.64256 |
| HMF | 0.9549033 | 0.64746 |
| GM19240 | 0.9476402 | 0.64890 |
| Hepatocytes | 0.9327327 | 0.64948 |
| BJ | 0.9521121 | 0.65048 |
| PanIslets | 0.9416016 | 0.65462 |
| PanIsletD | 0.9510868 | 0.65840 |
| IshikawaTamoxifen | 0.9413815 | 0.65956 |
| HGF | 0.9450531 | 0.66232 |
| GM18507 | 0.9354716 | 0.66388 |
| HBMEC | 0.9501761 | 0.67048 |
| HAc | 0.9494614 | 0.67128 |
| HUVEC | 0.9358606 | 0.67430 |
| AG04450 | 0.9419523 | 0.67546 |
| CD34Mobilized | 0.9356944 | 0.67616 |
| LNCaPAndrogen | 0.9275981 | 0.68000 |
| GM19239 | 0.9156631 | 0.69560 |
| AoSMC | 0.9456901 | 0.70068 |
| HCM | 0.9394576 | 0.70494 |
| NHA | 0.9350897 | 0.71206 |
| Medullo | 0.9211506 | 0.71326 |
| Gliobla | 0.9098192 | 0.71546 |
| HeLaS3IFNa4h | 0.9057969 | 0.71660 |
| AoAF | 0.9284834 | 0.72062 |
| HSMM | 0.9361561 | 0.72080 |
| Th1 | 0.9174819 | 0.72934 |
| Stellate | 0.9098990 | 0.73250 |
| GM19238 | 0.9019401 | 0.73448 |
| HCF | 0.9226607 | 0.73494 |
| Melano | 0.9356411 | 0.73500 |
| HFF | 0.9204323 | 0.74992 |
| FibroP | 0.9281981 | 0.75716 |
| ProgFib | 0.9002762 | 0.76404 |
| HPAF | 0.9155225 | 0.76694 |
| CD20 | 0.8707061 | 0.76736 |
| CLL | 0.8805603 | 0.76802 |
| HPF | 0.9046979 | 0.77220 |
| HL60 | 0.8812712 | 0.77412 |
| HeLaS3 | 0.8836405 | 0.78168 |
| GM12891 | 0.8697268 | 0.78408 |
| CMK | 0.8662576 | 0.79096 |
| HFFMyc | 0.9051487 | 0.79482 |
| HRPEpiC | 0.9110992 | 0.79740 |
| T47D | 0.8621213 | 0.79972 |
| MCF7Hypoxia | 0.8669266 | 0.79980 |
| MCF7 | 0.8813530 | 0.80028 |
| pHTE | 0.8829795 | 0.80476 |
| GM12892 | 0.8579702 | 0.80496 |
| HCPEpiC | 0.9038119 | 0.80540 |
| HConF | 0.8882812 | 0.81298 |
| NHDFneo | 0.8894734 | 0.81428 |
| Caco2 | 0.8120256 | 0.81732 |
| SKNMC | 0.8762043 | 0.81884 |
| 8988T | 0.8333343 | 0.83006 |
| LNCaP | 0.8717755 | 0.83134 |
| H9ES | 0.8584883 | 0.83412 |
| NHDFAd | 0.8919235 | 0.83470 |
| HSMMtube | 0.8869329 | 0.83526 |
| Fibrobl | 0.8730125 | 0.83694 |
| Osteobl | 0.8674761 | 0.84224 |
| WERIRb1 | 0.8705426 | 0.84680 |
| H1hESC | 0.8374480 | 0.84934 |
| AG09319 | 0.8617054 | 0.86146 |
| HMEC | 0.8596293 | 0.86238 |
| Chorion | 0.8090243 | 0.86870 |
| BE2C | 0.8168175 | 0.90218 |
| iPS | 0.7644133 | 0.91982 |
| SKNSHRA | 0.7402804 | 0.92834 |

# Cytokine responses to smallpox vaccine

| DHS sample | fold enrichment | p value |
|---|---|---|
| HGF | 1.1561205 | 0.12236 |
| HPdLF | 1.1500608 | 0.12832 |
| HCFaa | 1.1224190 | 0.15580 |
| GM12865 | 1.1449440 | 0.16458 |
| HAsp | 1.1058479 | 0.16812 |
| HIPEpiC | 1.1072485 | 0.17758 |
| AG09319 | 1.1213446 | 0.18362 |
| AG04449 | 1.1102962 | 0.19054 |
| WI38 | 1.1187334 | 0.19398 |
| HVMF | 1.1133420 | 0.20202 |
| SKMC | 1.0937888 | 0.20776 |
| HConF | 1.1046029 | 0.20920 |
| HMF | 1.0963951 | 0.21726 |
| GM12864 | 1.1182893 | 0.22528 |
| RPTEC | 1.0773197 | 0.23748 |
| HRCEpiC | 1.0765573 | 0.25024 |
| AoAF | 1.0819428 | 0.25750 |
| HCF | 1.0835585 | 0.25770 |
| AG04450 | 1.0821386 | 0.26976 |
| NHDFAd | 1.0697594 | 0.27828 |
| HMVECdLyNeo | 1.0808908 | 0.28392 |
| BJ | 1.0629667 | 0.30404 |
| HCPEpiC | 1.0575673 | 0.30564 |
| HMVECdBlAd | 1.0697132 | 0.30590 |
| HMVECdBlNeo | 1.0705481 | 0.30630 |
| HCM | 1.0596990 | 0.30884 |
| HRGEC | 1.0632750 | 0.31136 |
| NHDFneo | 1.0598521 | 0.31568 |
| AG09309 | 1.0564560 | 0.31584 |
| HPF | 1.0594359 | 0.31820 |
| SAEC | 1.0507687 | 0.31852 |
| HPAF | 1.0555493 | 0.32196 |
| AoSMC | 1.0537761 | 0.32638 |
| HMVECdAd | 1.0608854 | 0.33590 |
| SKNMC | 1.0460023 | 0.34010 |
| HMVECLBl | 1.0515916 | 0.35054 |
| HPAEC | 1.0492478 | 0.35934 |
| HNPCEpiC | 1.0350197 | 0.36678 |
| Th2 | 1.0555515 | 0.36806 |
| AG10803 | 1.0415108 | 0.37254 |
| HMVECdLyAd | 1.0391899 | 0.38810 |
| HMVECLLy | 1.0366641 | 0.39312 |
| HMVECdNeo | 1.0217707 | 0.42810 |
| HBMEC | 1.0174726 | 0.43258 |
| GM06990 | 1.0227884 | 0.43850 |
| HAEpiC | 1.0177423 | 0.43954 |
| HFF | 1.0133424 | 0.45304 |
| HAc | 1.0036821 | 0.48270 |
| GM12878 | 1.0008121 | 0.48484 |
| HFFMyc | 1.0021232 | 0.48968 |
| NHLF | 0.9999718 | 0.49674 |
| HEEpiC | 0.9977716 | 0.50428 |
| NHA | 0.9962866 | 0.50764 |
| HRE | 0.9947557 | 0.51048 |
| PANC1 | 0.9904283 | 0.51746 |
| A549 | 0.9808740 | 0.53718 |
| MonocytesCD14RO01746 | 0.9756136 | 0.54324 |
| HRPEpiC | 0.9873886 | 0.54628 |
| PrEC | 0.9721170 | 0.59398 |
| HPDE6E6E7 | 0.9620092 | 0.59428 |
| GM18507 | 0.9546923 | 0.59862 |
| HAh | 0.9724854 | 0.60252 |
| WERIRb1 | 0.9661223 | 0.60456 |
| CD20 | 0.9426810 | 0.60530 |
| CD34Mobilized | 0.9549512 | 0.60816 |
| Stellate | 0.9511419 | 0.61252 |
| HL60 | 0.9411878 | 0.62162 |
| HSMM | 0.9588162 | 0.63556 |
| CMK | 0.9148300 | 0.67586 |
| HCT116 | 0.9244312 | 0.68372 |
| HUVEC | 0.9258713 | 0.69126 |
| K562 | 0.9075405 | 0.70308 |
| NHEK | 0.9228959 | 0.70920 |
| Jurkat | 0.9177349 | 0.71832 |
| CLL | 0.8781800 | 0.74326 |
| HTR8svn | 0.8975221 | 0.75412 |
| GM19240 | 0.8885283 | 0.75924 |
| NB4 | 0.8878440 | 0.76014 |
| BE2C | 0.9009468 | 0.76436 |
| H7hESC | 0.9387869 | 0.76592 |
| GM19238 | 0.8701333 | 0.76994 |
| RWPE1 | 0.8941433 | 0.77348 |
| HSMMemb | 0.8834310 | 0.78378 |
| FibroP | 0.9031853 | 0.79584 |
| IshikawaEstradiol | 0.8852248 | 0.79850 |
| PanIsletD | 0.8910270 | 0.79862 |
| HSMMtube | 0.8954017 | 0.80056 |
| GM19239 | 0.8466533 | 0.80348 |
| IshikawaTamoxifen | 0.8660977 | 0.82354 |
| Th0 | 0.8360701 | 0.84394 |
| SKNSHRA | 0.8130712 | 0.86426 |
| Th1 | 0.8224135 | 0.87176 |
| Urothelia | 0.8293693 | 0.87628 |
| GM12891 | 0.7831283 | 0.88000 |
| GM12892 | 0.7820135 | 0.88084 |
| Gliobla | 0.8046920 | 0.88262 |
| Myometr | 0.8373967 | 0.88960 |
| Medullo | 0.8100580 | 0.89288 |
| ProgFib | 0.8174085 | 0.89548 |
| UrotheliaUT189 | 0.8104120 | 0.90396 |
| HeLaS3 | 0.8080334 | 0.90776 |
| NT2D1 | 0.8557044 | 0.91178 |
| MCF7Hypoxia | 0.7758118 | 0.91396 |
| Osteobl | 0.7986104 | 0.91468 |
| Fibrobl | 0.7987713 | 0.92410 |
| H9ES | 0.7885709 | 0.92946 |
| Caco2 | 0.6770727 | 0.92970 |
| LNCaPAndrogen | 0.7575050 | 0.93164 |
| Melano | 0.8361371 | 0.93566 |
| HepG2 | 0.7607060 | 0.93584 |
| LNCaP | 0.8013448 | 0.93796 |
| HMEC | 0.7952722 | 0.93912 |
| MCF7 | 0.7895411 | 0.94376 |
| PanIslets | 0.7555108 | 0.94720 |
| HeLaS3IFNa4h | 0.7238805 | 0.95734 |
| Huh7 | 0.7253370 | 0.95774 |
| Hepatocytes | 0.6978781 | 0.96204 |
| T47D | 0.7125658 | 0.96318 |
| pHTE | 0.7409085 | 0.96882 |
| H1hESC | 0.7185194 | 0.97082 |
| 8988T | 0.6551799 | 0.97358 |
| iPS | 0.6561981 | 0.97370 |
| Huh7.5 | 0.6745996 | 0.97904 |
| Chorion | 0.6301722 | 0.98276 |

# Body mass index 1

| DHS sample | fold enrichment | p value |
|---|---|---|
| PANC1 | 1.0722715 | 0.29750 |
| HAsp | 1.0291244 | 0.39218 |
| HCPEpiC | 1.0246604 | 0.40820 |
| HAh | 1.0218906 | 0.41238 |
| SKNMC | 1.0212130 | 0.41956 |
| RPTEC | 1.0151975 | 0.43582 |
| HPdLF | 1.0156785 | 0.44426 |
| AG04450 | 1.0137141 | 0.45238 |
| SKNSHRA | 1.0041628 | 0.47242 |
| HAc | 1.0050614 | 0.47624 |
| HRPEpiC | 0.9998823 | 0.49588 |
| HIPEpiC | 1.0003953 | 0.49714 |
| HRCEpiC | 0.9989755 | 0.49910 |
| WI38 | 0.9975607 | 0.50244 |
| AG09319 | 0.9959599 | 0.50632 |
| AG04449 | 0.9959638 | 0.50912 |
| HBMEC | 0.9954185 | 0.51226 |
| HAEpiC | 0.9911121 | 0.52690 |
| HGF | 0.9883316 | 0.53164 |
| HNPCEpiC | 0.9870748 | 0.54352 |
| A549 | 0.9780890 | 0.54758 |
| WERIRb1 | 0.9830737 | 0.55212 |
| CD20 | 0.9639408 | 0.56586 |
| BE2C | 0.9710619 | 0.57712 |
| HeLaS3 | 0.9686871 | 0.57874 |
| IshikawaTamoxifen | 0.9662303 | 0.58926 |
| HCFaa | 0.9704355 | 0.59860 |
| H7hESC | 0.9787629 | 0.59882 |
| HRE | 0.9717344 | 0.60100 |
| AG09309 | 0.9684648 | 0.60418 |
| NHLF | 0.9673785 | 0.61102 |
| IshikawaEstradiol | 0.9605806 | 0.61134 |
| HFF | 0.9606230 | 0.62682 |
| HRGEC | 0.9562945 | 0.63172 |
| HEEpiC | 0.9600010 | 0.64622 |
| HPAF | 0.9520770 | 0.65186 |
| Jurkat | 0.9472274 | 0.65242 |
| HConF | 0.9498485 | 0.65314 |
| NB4 | 0.9369563 | 0.65954 |
| BJ | 0.9421306 | 0.67648 |
| H9ES | 0.9340870 | 0.67712 |
| HVMF | 0.9356161 | 0.67814 |
| HL60 | 0.9184638 | 0.68208 |
| SKMC | 0.9447673 | 0.68380 |
| AoSMC | 0.9447815 | 0.68854 |
| CLL | 0.9108529 | 0.69404 |
| NHA | 0.9418455 | 0.69464 |
| SAEC | 0.9460777 | 0.69494 |
| HepG2 | 0.9175681 | 0.69608 |
| Medullo | 0.9236536 | 0.69628 |
| K562 | 0.9135673 | 0.69674 |
| HCT116 | 0.9223416 | 0.69766 |
| Huh7 | 0.9155041 | 0.70022 |
| GM06990 | 0.9051268 | 0.70648 |
| Hepatocytes | 0.8996940 | 0.71898 |
| HMVECdLyNeo | 0.9212964 | 0.71972 |
| HMF | 0.9261579 | 0.72900 |
| Myometr | 0.9202684 | 0.73028 |
| HMVECLBl | 0.9188427 | 0.73540 |
| HSMMemb | 0.9131036 | 0.73700 |
| GM12878 | 0.8973521 | 0.73824 |
| HMVECdAd | 0.9098249 | 0.73862 |
| NHDFneo | 0.9191122 | 0.74426 |
| GM12864 | 0.8973114 | 0.74784 |
| HeLaS3IFNa4h | 0.8943164 | 0.74876 |
| HFFMyc | 0.9194799 | 0.75410 |
| GM19239 | 0.8750846 | 0.77060 |
| Stellate | 0.8893596 | 0.77136 |
| AG10803 | 0.9067757 | 0.77140 |
| HCF | 0.9042776 | 0.77550 |
| HTR8svn | 0.8939326 | 0.77684 |
| T47D | 0.8780849 | 0.77694 |
| GM18507 | 0.8810108 | 0.77702 |
| Huh7.5 | 0.8768678 | 0.77886 |
| NHDFAd | 0.9113039 | 0.78024 |
| MCF7Hypoxia | 0.8743495 | 0.78700 |
| GM12891 | 0.8582219 | 0.78944 |
| HMVECdBlNeo | 0.8942212 | 0.79012 |
| HMVECdLyAd | 0.8857485 | 0.79360 |
| CD34Mobilized | 0.8828052 | 0.79370 |
| HPF | 0.8964085 | 0.79804 |
| HPAEC | 0.8897586 | 0.79840 |
| PrEC | 0.9112414 | 0.80098 |
| HUVEC | 0.8849040 | 0.80234 |
| HSMM | 0.9052333 | 0.80240 |
| AoAF | 0.8930654 | 0.80544 |
| GM19238 | 0.8615193 | 0.80758 |
| HPDE6E6E7 | 0.8778760 | 0.80944 |
| GM12865 | 0.8758548 | 0.80958 |
| RWPE1 | 0.8875373 | 0.81102 |
| CMK | 0.8558498 | 0.81306 |
| HMVECdNeo | 0.8789831 | 0.81644 |
| Caco2 | 0.7990380 | 0.82146 |
| 8988T | 0.8341279 | 0.82262 |
| ProgFib | 0.8712605 | 0.82580 |
| Gliobla | 0.8543900 | 0.82658 |
| GM12892 | 0.8361521 | 0.82678 |
| HCM | 0.8915166 | 0.82748 |
| NHEK | 0.8762810 | 0.83364 |
| HMVECLLy | 0.8705482 | 0.83382 |
| LNCaPAndrogen | 0.8501427 | 0.83442 |
| HMVECdBlAd | 0.8748429 | 0.83624 |
| MonocytesCD14RO01746 | 0.8349355 | 0.84076 |
| PanIslets | 0.8542562 | 0.84346 |
| H1hESC | 0.8502455 | 0.84372 |
| NT2D1 | 0.8975384 | 0.84548 |
| pHTE | 0.8601367 | 0.85192 |
| GM19240 | 0.8497841 | 0.85400 |
| MCF7 | 0.8580606 | 0.85900 |
| Chorion | 0.8024533 | 0.86954 |
| iPS | 0.8014480 | 0.87268 |
| PanIsletD | 0.8611365 | 0.87562 |
| Urothelia | 0.8429612 | 0.87618 |
| LNCaP | 0.8438995 | 0.88796 |
| Th2 | 0.7907764 | 0.90334 |
| Melano | 0.8658553 | 0.90976 |
| Osteobl | 0.8086295 | 0.91654 |
| HMEC | 0.8249256 | 0.91964 |
| HSMMtube | 0.8355181 | 0.92256 |
| Th0 | 0.7877340 | 0.92490 |
| UrotheliaUT189 | 0.7961073 | 0.93380 |
| Fibrobl | 0.7989537 | 0.93450 |
| FibroP | 0.8231191 | 0.95140 |
| Th1 | 0.7474559 | 0.96072 |

# Age at menarche

| DHS sample | fold enrichment | p value |
|---|---|---|
| HPdLF | 1.0824966 | 0.25072 |
| AG04449 | 1.0720531 | 0.26638 |
| HEEpiC | 1.0595008 | 0.28146 |
| HGF | 1.0626270 | 0.30616 |
| HConF | 1.0593554 | 0.30686 |
| AG09319 | 1.0562271 | 0.32214 |
| SAEC | 1.0454876 | 0.32598 |
| HCT116 | 1.0597565 | 0.32734 |
| SKNSHRA | 1.0675391 | 0.32780 |
| PANC1 | 1.0498120 | 0.34446 |
| AG10803 | 1.0458193 | 0.34916 |
| HRGEC | 1.0357950 | 0.38272 |
| HeLaS3 | 1.0297818 | 0.40554 |
| HTR8svn | 1.0273863 | 0.41218 |
| RWPE1 | 1.0221913 | 0.42050 |
| HMVECLBl | 1.0228715 | 0.42368 |
| PrEC | 1.0167536 | 0.43008 |
| BJ | 1.0191323 | 0.43080 |
| HPAEC | 1.0150517 | 0.44924 |
| HeLaS3IFNa4h | 1.0093977 | 0.46228 |
| HVMF | 1.0095737 | 0.46344 |
| Gliobla | 1.0070005 | 0.46676 |
| SKNMC | 1.0065182 | 0.46846 |
| HMF | 1.0042607 | 0.47986 |
| WI38 | 1.0035425 | 0.48416 |
| HIPEpiC | 1.0027271 | 0.48904 |
| HFF | 1.0003826 | 0.49496 |
| BE2C | 0.9943875 | 0.50002 |
| HCFaa | 0.9967841 | 0.50550 |
| AG04450 | 0.9950520 | 0.51000 |
| AoSMC | 0.9928298 | 0.52340 |
| NHLF | 0.9899773 | 0.52920 |
| NHEK | 0.9876286 | 0.53138 |
| HUVEC | 0.9852944 | 0.53526 |
| NHDFneo | 0.9873855 | 0.53726 |
| HFFMyc | 0.9859861 | 0.54450 |
| HPDE6E6E7 | 0.9794250 | 0.54958 |
| HepG2 | 0.9755017 | 0.55406 |
| AG09309 | 0.9811005 | 0.56498 |
| HAEpiC | 0.9770942 | 0.57056 |
| SKMC | 0.9767260 | 0.57812 |
| HAsp | 0.9768130 | 0.57864 |
| NHA | 0.9763055 | 0.58046 |
| HMVECdBlAd | 0.9712178 | 0.58360 |
| NHDFAd | 0.9738338 | 0.59068 |
| Stellate | 0.9626154 | 0.59356 |
| HMVECdBlNeo | 0.9660161 | 0.59702 |
| AoAF | 0.9661881 | 0.60700 |
| HMVECdLyNeo | 0.9599071 | 0.61326 |
| HSMMemb | 0.9554672 | 0.62310 |
| A549 | 0.9486970 | 0.63082 |
| HMVECdAd | 0.9511434 | 0.63152 |
| IshikawaEstradiol | 0.9544477 | 0.63256 |
| HSMM | 0.9608977 | 0.63856 |
| HMVECdNeo | 0.9493343 | 0.63926 |
| HPAF | 0.9509565 | 0.65566 |
| HPF | 0.9489508 | 0.65580 |
| HAh | 0.9588218 | 0.65726 |
| 8988T | 0.9244462 | 0.65748 |
| IshikawaTamoxifen | 0.9410251 | 0.66124 |
| LNCaPAndrogen | 0.9320011 | 0.66266 |
| Hepatocytes | 0.9222699 | 0.66854 |
| HNPCEpiC | 0.9523765 | 0.67466 |
| T47D | 0.9243423 | 0.67670 |
| K562 | 0.9213193 | 0.68016 |
| HCPEpiC | 0.9471574 | 0.68092 |
| WERIRb1 | 0.9449751 | 0.68572 |
| HRPEpiC | 0.9529945 | 0.68594 |
| HBMEC | 0.9479652 | 0.68622 |
| HMVECdLyAd | 0.9279888 | 0.69772 |
| Medullo | 0.9231067 | 0.69962 |
| HCF | 0.9325879 | 0.70398 |
| HAc | 0.9431230 | 0.70596 |
| Myometr | 0.9264715 | 0.71508 |
| CMK | 0.8982363 | 0.71722 |
| NT2D1 | 0.9396915 | 0.72298 |
| ProgFib | 0.9129081 | 0.73198 |
| MCF7Hypoxia | 0.8981480 | 0.73296 |
| H9ES | 0.9105480 | 0.73660 |
| Huh7 | 0.8978612 | 0.74308 |
| PanIsletD | 0.9194499 | 0.74372 |
| Huh7.5 | 0.8941213 | 0.74620 |
| RPTEC | 0.9293766 | 0.75666 |
| Jurkat | 0.9093901 | 0.75726 |
| GM12878 | 0.8905195 | 0.75728 |
| PanIslets | 0.8988964 | 0.75744 |
| GM12864 | 0.8946939 | 0.75908 |
| NB4 | 0.8974291 | 0.75954 |
| Th2 | 0.8757892 | 0.76502 |
| HMVECLLy | 0.8969371 | 0.77696 |
| H7hESC | 0.9378753 | 0.78264 |
| CD20 | 0.8552802 | 0.78264 |
| HRE | 0.9169945 | 0.78862 |
| HRCEpiC | 0.9131710 | 0.78900 |
| HL60 | 0.8648323 | 0.79082 |
| MCF7 | 0.8895091 | 0.79244 |
| GM12865 | 0.8856098 | 0.79492 |
| HCM | 0.9045610 | 0.79646 |
| CLL | 0.8422953 | 0.82280 |
| pHTE | 0.8747491 | 0.82498 |
| Melano | 0.9053740 | 0.82828 |
| FibroP | 0.9011585 | 0.82840 |
| GM19239 | 0.8365789 | 0.83896 |
| CD34Mobilized | 0.8589448 | 0.84318 |
| MonocytesCD14RO01746 | 0.8293298 | 0.84718 |
| Caco2 | 0.7662861 | 0.85334 |
| HSMMtube | 0.8806329 | 0.85604 |
| HMEC | 0.8650195 | 0.86100 |
| Chorion | 0.8091546 | 0.86298 |
| H1hESC | 0.8354756 | 0.86798 |
| GM12891 | 0.8060098 | 0.86954 |
| GM06990 | 0.8163413 | 0.87080 |
| iPS | 0.7974497 | 0.87856 |
| LNCaP | 0.8526972 | 0.87992 |
| Fibrobl | 0.8420026 | 0.88522 |
| GM12892 | 0.7947730 | 0.88524 |
| Th0 | 0.8178552 | 0.89118 |
| Urothelia | 0.8335021 | 0.89386 |
| GM19240 | 0.8183588 | 0.90142 |
| GM18507 | 0.8045066 | 0.90470 |
| Osteobl | 0.8138389 | 0.91550 |
| GM19238 | 0.7881243 | 0.91592 |
| UrotheliaUT189 | 0.8137124 | 0.91870 |
| Th1 | 0.7811256 | 0.93894 |

# Myopia

| DHS sample | fold enrichment | p value |
|---|---|---|
| GM12864 | 1.0661548 | 0.33322 |
| CD20 | 1.0577780 | 0.37060 |
| GM06990 | 1.0515094 | 0.37546 |
| Th2 | 1.0303166 | 0.42358 |
| GM18507 | 1.0057990 | 0.47218 |
| Th0 | 1.0038359 | 0.48308 |
| GM12878 | 0.9900855 | 0.51398 |
| GM12865 | 0.9806500 | 0.54178 |
| GM19238 | 0.9736151 | 0.54678 |
| CLL | 0.9695221 | 0.55034 |
| GM19239 | 0.9613269 | 0.56840 |
| Th1 | 0.9624400 | 0.58636 |
| CD34Mobilized | 0.9606710 | 0.59176 |
| GM19240 | 0.9505696 | 0.61402 |
| MonocytesCD14RO01746 | 0.9423769 | 0.61524 |
| GM12892 | 0.9232192 | 0.64482 |
| Melano | 0.9588744 | 0.64512 |
| SAEC | 0.9569050 | 0.65540 |
| CMK | 0.9184769 | 0.66366 |
| NHDFneo | 0.9423686 | 0.67244 |
| Caco2 | 0.8731330 | 0.67408 |
| NB4 | 0.9251575 | 0.67472 |
| H1hESC | 0.9205049 | 0.67666 |
| AG09309 | 0.9419666 | 0.68352 |
| HAh | 0.9487440 | 0.69180 |
| Jurkat | 0.9206173 | 0.70966 |
| PrEC | 0.9370836 | 0.71646 |
| GM12891 | 0.8786641 | 0.73152 |
| FibroP | 0.9293155 | 0.73176 |
| Fibrobl | 0.9041923 | 0.73802 |
| HRPEpiC | 0.9370630 | 0.73912 |
| PanIslets | 0.8967080 | 0.74066 |
| Chorion | 0.8688873 | 0.74580 |
| H9ES | 0.9012356 | 0.74814 |
| H7hESC | 0.9428790 | 0.75096 |
| T47D | 0.8792030 | 0.75176 |
| HEEpiC | 0.9276278 | 0.75188 |
| iPS | 0.8610909 | 0.75788 |
| HSMMtube | 0.9121332 | 0.76164 |
| Gliobla | 0.8700568 | 0.77114 |
| Osteobl | 0.8867427 | 0.77202 |
| 8988T | 0.8511797 | 0.77278 |
| Myometr | 0.8974803 | 0.77354 |
| ProgFib | 0.8853648 | 0.77562 |
| Hepatocytes | 0.8551937 | 0.78242 |
| pHTE | 0.8838891 | 0.78664 |
| RPTEC | 0.9147517 | 0.78976 |
| A549 | 0.8694585 | 0.79492 |
| Huh7 | 0.8608958 | 0.79534 |
| HAsp | 0.9068188 | 0.79828 |
| AG10803 | 0.8904398 | 0.80082 |
| Medullo | 0.8646220 | 0.80148 |
| IshikawaEstradiol | 0.8804438 | 0.80276 |
| HL60 | 0.8452134 | 0.80362 |
| HRCEpiC | 0.9037050 | 0.80380 |
| HSMMemb | 0.8712107 | 0.80682 |
| HAc | 0.9062677 | 0.81226 |
| HMVECdBlAd | 0.8768590 | 0.81226 |
| PANC1 | 0.8679075 | 0.81356 |
| HMEC | 0.8797799 | 0.81468 |
| NHDFAd | 0.8947330 | 0.81728 |
| AG09319 | 0.8806227 | 0.81738 |
| HFF | 0.8872746 | 0.82298 |
| NHA | 0.8937016 | 0.82318 |
| HMVECLBl | 0.8722973 | 0.82584 |
| HRE | 0.8995879 | 0.82706 |
| HPdLF | 0.8762388 | 0.82882 |
| HSMM | 0.8905850 | 0.82946 |
| HeLaS3 | 0.8539822 | 0.83294 |
| SKNMC | 0.8869469 | 0.83558 |
| AoSMC | 0.8851628 | 0.83650 |
| Stellate | 0.8448978 | 0.83670 |
| NHLF | 0.8908065 | 0.83728 |
| IshikawaTamoxifen | 0.8582292 | 0.83818 |
| BJ | 0.8767838 | 0.84092 |
| HRGEC | 0.8664968 | 0.84698 |
| HFFMyc | 0.8753641 | 0.84728 |
| LNCaPAndrogen | 0.8247431 | 0.85298 |
| HCT116 | 0.8385075 | 0.85834 |
| NT2D1 | 0.8819206 | 0.85860 |
| HMVECdLyAd | 0.8407882 | 0.86484 |
| AG04449 | 0.8621762 | 0.86622 |
| WI38 | 0.8463891 | 0.86840 |
| K562 | 0.8073380 | 0.86858 |
| PanIsletD | 0.8562068 | 0.87126 |
| LNCaP | 0.8466278 | 0.87874 |
| Huh7.5 | 0.8003380 | 0.87984 |
| HConF | 0.8498798 | 0.88560 |
| AG04450 | 0.8397414 | 0.88900 |
| NHEK | 0.8374740 | 0.89172 |
| HBMEC | 0.8658229 | 0.89284 |
| SKMC | 0.8585659 | 0.89302 |
| HPAEC | 0.8274018 | 0.89378 |
| BE2C | 0.8354078 | 0.89436 |
| HPDE6E6E7 | 0.8189004 | 0.89680 |
| HMVECdNeo | 0.8184868 | 0.89772 |
| HMVECLLy | 0.8204699 | 0.89802 |
| HMVECdLyNeo | 0.8177730 | 0.90154 |
| HMVECdBlNeo | 0.8197754 | 0.90204 |
| HIPEpiC | 0.8456282 | 0.90850 |
| HeLaS3IFNa4h | 0.7734511 | 0.91504 |
| Urothelia | 0.8032991 | 0.91572 |
| HNPCEpiC | 0.8527540 | 0.91642 |
| HCFaa | 0.8376334 | 0.91678 |
| RWPE1 | 0.8146504 | 0.91796 |
| HUVEC | 0.8037373 | 0.92078 |
| HTR8svn | 0.7824047 | 0.92722 |
| MCF7Hypoxia | 0.7464817 | 0.93336 |
| SKNSHRA | 0.7621256 | 0.93372 |
| HPF | 0.8090645 | 0.94090 |
| HCM | 0.8169516 | 0.94190 |
| HCPEpiC | 0.8201360 | 0.94428 |
| UrotheliaUT189 | 0.7659577 | 0.94538 |
| AoAF | 0.7965227 | 0.94602 |
| HMVECdAd | 0.7642942 | 0.94788 |
| WERIRb1 | 0.8103083 | 0.94794 |
| HGF | 0.7837115 | 0.95234 |
| HMF | 0.7991308 | 0.95424 |
| HCF | 0.7813938 | 0.96074 |
| HPAF | 0.7910955 | 0.96176 |
| HVMF | 0.7609419 | 0.96592 |
| HAEpiC | 0.7734948 | 0.96806 |
| HepG2 | 0.6961466 | 0.97396 |
| MCF7 | 0.7328884 | 0.97624 |
